# Reactivity–Stereoselectivity Mapping for the Assembly of *Mycobacterium marinum* Lipooligosaccharides

**DOI:** 10.1002/anie.202010280

**Published:** 2020-11-03

**Authors:** Thomas Hansen, Tim P. Ofman, Joey G. C. Vlaming, Ivan A. Gagarinov, Jessey van Beek, Tessa A. Goté, Jacoba M. Tichem, Gijs Ruijgrok, Herman S. Overkleeft, Dmitri V. Filippov, Gijsbert A. van der Marel, Jeroen D. C. Codée

**Affiliations:** ^1^ Leiden University Leiden Institute of Chemistry Einsteinweg 55 2333 CC Leiden The Netherlands

**Keywords:** density functional calculations, glycosyl cation, glycosylation, reactivity, stereoselectivity

## Abstract

The assembly of complex bacterial glycans presenting rare structural motifs and *cis*‐glycosidic linkages is significantly obstructed by the lack of knowledge of the reactivity of the constituting building blocks and the stereoselectivity of the reactions in which they partake. We here report a strategy to map the reactivity of carbohydrate building blocks and apply it to understand the reactivity of the bacterial sugar, caryophyllose, a rare C12‐monosaccharide, containing a characteristic tetrasubstituted stereocenter. We mapped reactivity–stereoselectivity relationships for caryophyllose donor and acceptor glycosides by a systematic series of glycosylations in combination with the detection and characterization of different reactive intermediates using experimental and computational techniques. The insights garnered from these studies enabled the rational design of building blocks with the required properties to assemble mycobacterial lipooligosaccharide fragments of *M. marinum*.

## Graphical abstract

A joint experimental and computational approach was used to assess reactivity–stereoselectivity relationships of the bacterial sugar caryophyllose, a rare C12‐monosaccharide, containing a characteristic tetrasubstituted stereocenter. The fundamental insights gained guided the rational design of building blocks for the effective and stereoselective assembly of mycobacterial lipooligosaccharide fragments.

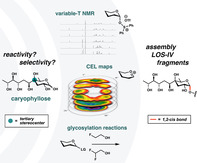

## Introduction

**Figure 1:**
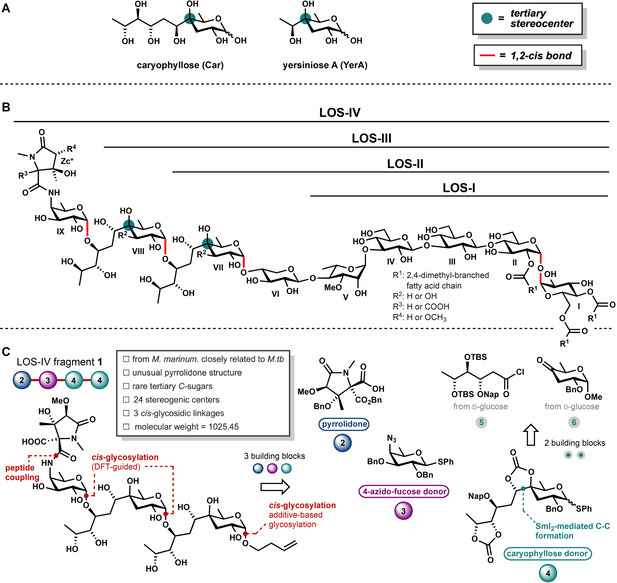
Lipooligosaccharides from *M. marinum* and the target fragment with a retrosynthetic analysis. A) Tertiary *C*‐sugar caryophyllose (Car) found in mycobacterial lipooligosaccharides and the related yersiniose A (YerA). B) LOS‐IV from *M. marinum*. with numbering introduced by Rombouts et al.[^11^, ^12^, ^13^] C) Retrosynthetic analysis for LOS‐IV fragment **1**.

The bacterial glycan repertoire is equally vast and diverse.[^1^, ^2^, ^3^, ^4^, ^5^] As opposed to the mammalian carbohydrate biosynthesis machinery that employs a limited set of 9 monosaccharides^[6]^ to build oligosaccharides and glycoconjugates, the bacterial biomachinery can introduce a wide variety of substitution patterns.[^1^, ^2^, ^3^, ^4^, ^5^] Bacterial monosaccharides can feature diversely substituted amino groups, deoxy centers, carbonyl groups, and tetrasubstituted tertiary carbon atoms at various positions on the carbohydrate ring. Tertiary‐*C* sugars can be found in various natural products, having attractive biological properties.[^7^, ^8^, ^9^, ^10^] Often these tertiary C‐atoms are substituted with a small alkyl group, commonly a methyl substituent, but more complex architectures in which functionalized alkyl chains are attached can be found as well. For example, the tertiary *C*‐sugar caryophyllose (Car, see Figure 1 A) is found in mycobacterial lipooligosaccharides (LOSs).[^11^, ^12^, ^13^] This unique structure bears a hydroxylated C6‐chain at the tetrasubstituted tertiary C4‐atom.

The mycobacterial LOSs are major constituents of the thick and waxy cell wall of mycobacteria.[^11^, ^12^, ^13^, ^14^, ^15^, ^16^] Being at the host–pathogen interface, they play an important role in the interaction with the immune system. Because it is exceedingly laborious to purify these lipophilic compounds from the bacterial cell wall, it has proven difficult to establish the precise role of these glycolipids in shaping an immune response. *Mycobacterium marinum* is a waterborne pathogen that is most closely related to *Mycobacterium tuberculosis*, and causes tuberculosis‐like infections. As such it is often used as a surrogate to study host‐pathogen interactions involved in *M.tb* infections. *M. marinum* produces four LOS structures (LOS‐I–IV; Figure 1 B), which all share an acylated trehalose core functionalized with species‐specific glycans. The LOS‐II, LOS‐III, and LOS‐IV structures of *M. marinum* contain several unusual carbohydrate monosaccharides, including the tertiary *C*‐sugar caryophyllose as well as an *N*‐acylated 4‐amino‐4‐deoxy‐d‐fucose (NAcFuc).[^11^, ^12^, ^13^, ^17^, ^18^, ^19^] The complex carbohydrates of the higher LOS‐structures thus seem to play an important role in immune evasion although the exact mode of action of these remains ill‐understood.

The compelling bioactivity, intriguing structural features, and the fact that well‐defined pure LOS structures cannot be obtained from natural sources in sufficient amounts for biological studies motivated us to develop synthetic chemistry to attain these complex structures. Although great progress has been made in oligosaccharide synthesis, the assembly of bacterial glycans presenting rare structural modifications and challenging *cis*‐glycosidic linkages still presents a major obstacle as the reactivity of the required building blocks is not well understood.[^20^, ^21^, ^22^, ^23^, ^24^, ^25^, ^26^, ^27^, ^28^, ^29^, ^30^, ^31^, ^32^, ^33^, ^34^, ^35^, ^36^, ^37^] We here report an approach to map the reactivity–stereoselectivity relationships for the tertiary *C*‐sugar caryophyllose and its truncated counterpart yersiniose A (YerA; Figure 1 A). This has allowed us to effectively construct the Car‐Car‐NAcFuc LOS‐IV fragment **1** (Figure 1 C), and related shorter fragments, equipped with an alkene spacer for future conjugation purposes. The approach taken to understand the reactivity and stereoselectivity of these rare and challenging bacterial monosaccharides hinges on the detection and characterization of different reactive intermediates using experimental and computational techniques. These combined studies have enabled the rational design of building blocks with the desired reactivity and selectivity to assemble the spacer equipped LOS‐IV fragment **1** with complete stereoselectivity. Our study discloses the intrinsic reactivity of tertiary‐*C* Car donors, which can act as a prototype for related tertiary‐*C* sugars that can fuel biological research.

## Results and Discussion

The Car‐Car‐NAcFuc carbohydrate **1** was assembled from the three key monomeric building blocks, pyrrolidone **2**, 4‐amino‐4‐deoxy‐d‐fucose **3**, and caryophyllose **4** (Figure 1 C). The design of the latter building block was based on reactivity studies, as outlined below. Pyrrolidone **2** can be synthesized based on the work of the Lowary group from d‐serine and the 4‐azido‐fucose donor **3** can be made from d‐glucose by deoxygenation of C6 and an inversion of the C4 position, following established procedures.^[38]^ Car donor **4** can be synthesized from building blocks **5** and **6** by a SmI_2_‐mediated C−C bond formation, as originally described by Prandi and co‐workers.[^39^, ^40^]

**Scheme 1:**
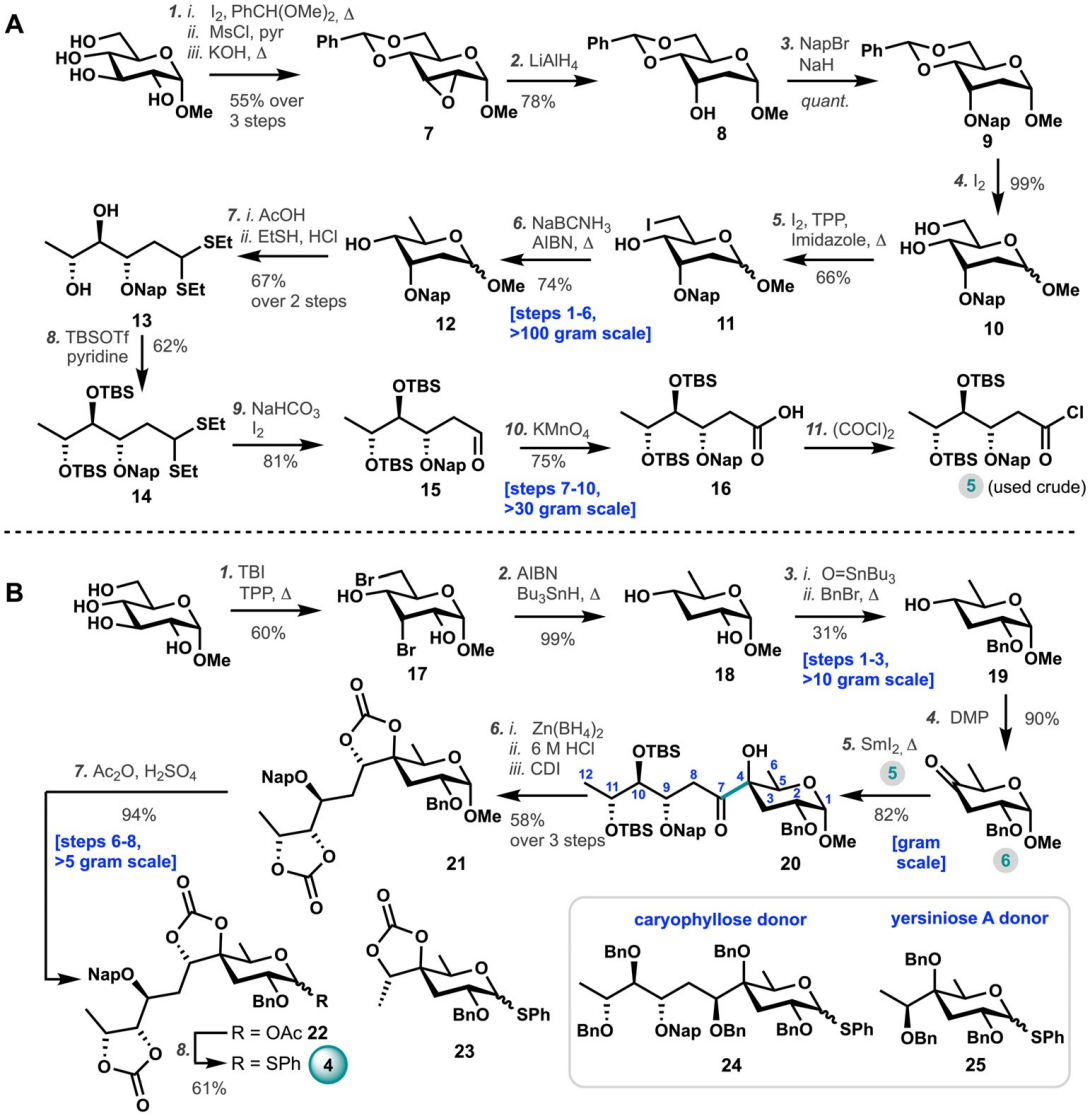
Synthesis of **4**, **5**, **6**, **23**, **24**, and **25**. A) Synthesis of building block **5**. *Reagents and conditions*: (1) *i*. benzaldehyde dimethyl acetal, I_2_, CH_3_CN; *ii*. MsCl, pyridine; *iii*. KOH, THF/MeOH (55 % over three steps); (2) LiAlH_4_, Et_2_O (78 %); (3) NapBr, NaH, DMF (*quant*.); (4) I_2_, MeOH (99 %); (5) imidazole, triphenylphosphine, I_2_, toluene, 75 °C (66 %); (6) NaBCNH_3_, AIBN, *t*‐BuOH, 80 °C (74 %); (7) *i*. aq. 25 % AcOH, reflux; *ii*. EtSH, aq. 37 % HCl (67 % over 2 steps); (8) TBSOTf, pyridine, DCM (62 %); (9) I_2_, NaHCO_3_, acetone, H_2_O (81 %); (10) KMnO_4_ aq., NaH_2_PO_4_ aq., *t*‐BuOH (75 %); (11) (COCl)_2_, pyridine. B) Synthesis of donor **4** and **23**. *Reagents and conditions*: (1) tribromoimidazole, triphenylphosphine, toluene, reflux (60 %); (2) AIBN, Bu_3_SnH, toluene, reflux (99 %); (3) *i*. tributyltin oxide, toluene, reflux; *ii*. benzyl bromide, reflux (31 %); (4) DMP, DCM (91 %); (5) SmI_2_, **5**, THF, 50 °C, 15 min (82 %); (6) *i*. Zn(BH_4_)_2_, THF; *ii*. 6 M HCl, MeOH; *iii*. CDI, DCM (58 % over three steps); (7) Ac_2_O, H_2_SO_4_, 1 min (94 %); (8) thiophenol, BF_3_⋅OEt_2_, DCM (61 %). Pyr=pyridine, NapBr=2‐(bromomethyl)naphthalene, TBI=2,4,5‐tribromoimidazole, TBSOTf=*t*‐butyldimethylsilyl trifluoromethanesulfonate, TPP=triphenylphosphine, AIBN=azobisisobutyronitrile, DMP=Dess–Martin periodinane, CDI=carbonyldiimidazole.

Our first goal was the generation of sufficient amounts of the Car donor glycosides, required to map the reactivity of these building blocks and build the target fragment **1**. To this end acid chloride **5** and 2,6‐dideoxy‐4‐keto‐glucose **6** were assembled. The synthesis of **5** is depicted in Scheme 1 A and started from methyl‐α‐d‐glucopyranose. Epoxide **7** was readily prepared in three steps (>150 gram scale). Regioselective opening of the epoxide with LiAlH_4_ afforded digitoxose‐configured **8** in good yield (78 %, >120 gram scale). Installation of the temporary 2‐methylnaphthyl protecting group resulted in fully protected **9**. The 4,6‐*O*‐benzylidene protecting group was removed using a catalytic amount of I_2_ to yield diol **10** (99 %), and the primary alcohol was converted into iodide **11** using triphenylphosphine, iodine, and imidazole. Radical reduction using NaBCNH_3_ and AIBN yielded the partially protected d‐digitoxose **12** (74 %, >100 gram scale).^[41]^ Hydrolysis of d‐digitoxose **12** with 25 % v:v aq. AcOH, followed by the treatment with ethanethiol and concentrated HCl afforded the linear diethyl dithioacetal **13** (67 % over two steps, 50 gram scale). Subsequently, both hydroxyl functions of the dithioacetal were protected with a TBS group using TBSOTf and pyridine to yield the fully protected **14** (62 %).

Treating dithioacetal **14** with I_2_ and NaHCO_3_ in acetone/water delivered the corresponding aldehyde **15** in 81 % yield (30 gram scale). Oxidation with potassium permanganate in *t*‐BuOH/water of aldehyde **15** furnished the protected acid **16** (75 %), which could be converted to building block **5** with pyridine and oxalyl chloride.

As depicted in Scheme 1 B building block **6** was also synthesized from methyl‐α‐d‐glucopyranoside, starting with the regioselective bromination of the C3‐ and C6‐position using tribromoimidazole in good yield (60 %, >30 gram scale). Removal of the bromides using tributyltin hydride and AIBN afforded the required dideoxy glucoside **18** in excellent yield (99 %, 15 gram scale). The reaction of **18** with tributyltin oxide, followed by benzyl bromide provided the benzylated glucoside **19** in 31 % yield. Oxidation of the C4‐alcohol in **19** with Dess–Martin periodinane then afforded key building block **6** (90 %).

To build the tertiary *C*‐sugar having the required Car‐configuration, a SmI_2_‐promoted C−C bond coupling was employed using acyl chloride **5** and ketone **6** (Scheme 1 B). The best yield for this cross‐coupling was obtained by premixing both coupling partners and quickly adding them, by canula, to a warm (50 °C) solution of SmI_2_ in THF under completely inert atmosphere. This procedure reliably delivered ketone **20** with the required stereochemistry at C4 in 82 % yield (gram scale). A chelation controlled reduction of ketone **20** with Zn(BH_4_)_2_ in THF then afforded the free alcohol. After removal of the silyl protection groups and protection of the two vicinal diols using carbonyldiimidazole caryophyllose **21** was obtained in 58 % (over three steps, 15 gram scale). Proof for the stereochemistry of the C7 position was obtained by NOESY NMR experiments (See SI). The anomeric methoxy group of caryophyllose **21** was then converted to an acetyl group using H_2_SO_4_ in acetic anhydride. The anomeric acetate **22** was formed in excellent yield (94 %, >5 gram scale) and subsequently transformed into the key caryophyllose thioglycoside **4** under the aegis of thiophenol and BF_3_⋅OEt_2_. Following a highly similar route, the per‐*O*‐Bn caryophyllose thioglycoside **24** was constructed (See SI). We also assembled yersiniose A (YerA) donors **23** and **25**, to be used as model donors to map the reactivity–selectivity of these type of donors (See SI).

**Figure 2:**
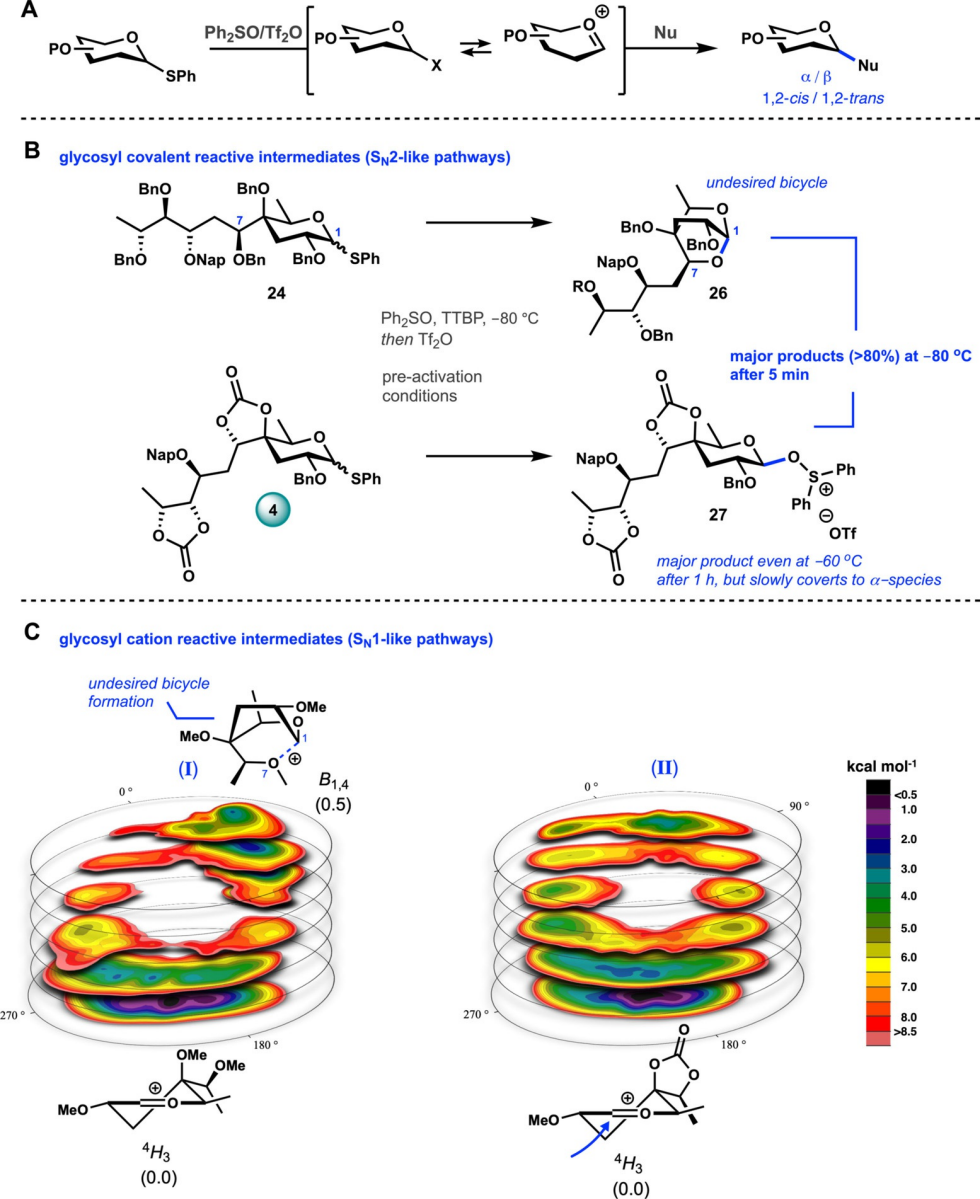
Mapping the relevant reactive intermediates by a combined experimental and computational approach. A) The reaction mechanism continuum operational during glycosylation reactions. Glycosylation reactions are best considered as taking place at a continuum between two formal extremes of the mechanisms, including the S_N_1 and S_N_2 mechanism. B) Upon activation with Ph_2_SO/Tf_2_O of donor **24**, the undesired fused bicycle **26** was formed. This side reaction makes these per‐*O*‐benzylated caryophyllose donors unsuitable for efficient glycosylation reactions. C) Conformational energy landscape (CEL) maps of selected pyranosyl oxocarbenium ions in which the found local minima are indicated with their respective energy. All energies are as computed at PCM(CH_2_Cl_2_)‐B3LYP/6–311G(d,p) at *T*=213.15 K and expressed as solution‐phase Gibbs free energy.

With all donors in hand, we set out to study the glycosylation properties of the building blocks under pre‐activation conditions (Figure 2 A). To do so we first investigated the possible reactive intermediates that can play a role during the glycosylation of these donors. Covalent species, such as anomeric triflates are formed, which can undergo a S_N_2‐like substitution or serve as a reservoir for more reactive oxocarbenium ion type species that partake in substitution reactions with more S_N_1‐character. We started our investigation with the detection of the formation of reactive covalent species by the use of variable‐T NMR.^[42]^ We first tested the per‐*O*‐benzyl donor **24**. To this end a mixture of **24** and Ph_2_SO (1.3 equiv.) in CD_2_Cl_2_ was treated with Tf_2_O (1.3 equiv.) at −80 °C (Figure 2 B).^[43]^ Directly after the addition, NMR data (^1^H, HSQC, COSY) were recorded, to reveal the generation of a single new species. The signals of the anomeric H and C atoms appeared at *δ* 4.7 ppm and *δ* 90.4 ppm for ^1^H and ^13^C respectively, which is significantly upfield from signals corresponding to an anomeric triflate or oxosulfonium triflate species (generally found at ^1^H: *δ*≈5–6.5 ppm and ^13^C: *δ*≈105–110 ppm).[^42^, ^44^, ^45^, ^46^, ^47^] Warming the sample did not lead to any degradation of the initially formed product, and therefore it could be isolated. NMR analysis (^1^H, ^13^C, HSQC, COSY, NOESY and HMBC) identified the formed species to be bicyclic compound **26**. Similarly, upon activation of the structurally simpler yersiniose donor **25**, a corresponding bicycle was formed. These bicycles are formed by nucleophilic attack, of the C7 benzyl ether oxygen atom, on the activated C1 position.

Cyclization reactions on activated glycosyl donors have been reported before (for example from a C6‐OBn to form a 1,6‐anhydrosugar),^[48]^ but the rate with which the caryophyllose/yersiniose cyclization takes place is striking. Apparently, the architecture in these systems is intrinsically geared for this intramolecular nucleophilic cyclization. To prevent this cyclization, the C7‐OH was tethered to the C4‐OH, by the use of a carbonate protection group. Activation of the thus obtained donor **4**, using the conditions described above, resulted in the formation of several species, amongst which the anomeric β‐oxosulfonium triflate **27** species (^1^H: *δ* 5.8 ppm; ^13^C: *δ* 107.6 ppm) as the dominant reactive intermediate (±80 % based on ^1^H‐NMR). To support that this is indeed the oxosulfonium triflate, more Ph_2_SO (+1.7 equiv.) was added after the activation, which led to the increase of the oxosulfonium signals and the disappearance of the signals corresponding to the anomeric triflate. Upon slow warming of the mixture, this species gradually converted into the anomeric α‐triflate and α‐oxosulfonium triflate species (See SI for all variable‐T NMR results).

To study the reactive intermediates on the other side of the reaction mechanism continuum, we studied the caryophyllose and yersiniose oxocarbenium ions by the use of DFT computations. We recently developed a DFT protocol to compute the relative energy of all possible glycopyranosyl oxocarbenium ion conformers, filling the complete conformational space these ions can occupy generating conformational energy landscape (CEL) maps.[^49^, ^50^, ^51^] Based on these CEL maps, a prediction can be made on the stereochemical outcome of reactions involving these ions. Figure 2 C shows the CEL maps of the two YerA oxocarbenium ions (these were selected as the substituted C6‐chain of caryophyllose would demand a significant increase in computing cost). The lowest energy structures are shown next to the CEL maps with their corresponding energy (with the lowest energy depicted in black/purple). The CEL map of oxocarbenium ion **I** (Figure 2 C, left) shows that this species preferentially takes up a ^4^ *H*_3_ conformation. A second local minimum was found on the other side of the CEL map, revealing the *B*_1,4_ conformer to be only slightly higher in energy (Δ*G* ${}_{\mathrm{CH}{}_{2}\mathrm{Cl}{}_{2}}$ =0.5 kcal mol^−1^). This latter conformer explains the rapid formation of the bicycles found upon activation of donors **24** and **25** as the C7 ether is perfectly positioned to attack the C1 position in this cation. The CEL map of oxocarbenium ion **II** (Figure 2 C, right) reveals a single minimal energy conformer. This ^4^ *H*_3_ conformer is preferentially attacked from the diastereotopic face that leads to a chair‐like transition state, and thus based on this analysis this cation is predicted to serve as a 1,2‐*cis*‐selective glycosylating species.

We next probed the donors **4** and **23** for their stereoselectivity in glycosylation reactions (Table 1). To this end, we performed a matrix of glycosylation reactions with a set of model alcohol nucleophiles of gradually decreasing nucleophilicity.[^52^, ^53^] The trends observed relate to changes from an S_N_2‐type substitution reaction of the covalent intermediate for the most nucleophilic alcohols (EtOH and MFE), to reactions involving more oxocarbenium character (for the poorest nucleophiles; TFE, HFIP and TES‐*d*). The outcome of the glycosylation reactions for both the caryophyllose and yersiniose donor show clear trends with changing nucleophilicity of the used acceptors. The caryophyllose donor **4** and yersiniose donor **23** behave very similarly and with decreasing nucleophilicity the 1,2*‐cis*‐selectivity increases for both systems. Even with strong nucleophiles, somewhat more of the 1,2‐*cis*‐product is formed, which may be explained by the direct displacement of the β‐oxosulfonium triflate **27** species. The increasing 1,2‐*cis*‐selectivity can be accounted for by an increase of S_N_1 character in the glycosylation reaction, as the weaker nucleophiles require a more electrophilic glycosylating agent. The CEL maps revealed the ^4^ *H*_3_ oxocarbenium ion conformers to be most stable and a stereoselective addition to these ions can explain the formation of the α‐products.

**Table 1:** Experimentally found stereoselectivities for model glycosylation reactions with ethanol, 2‐fluoroethanol, 2,2‐difluoroethanol, 2,2,2‐trifluoroethanol, 1,1,1,3,3,3‐hexafluoro‐2‐propanol, triethylsilane‐*d*, 3‐butene‐1‐ol, **28**, and **29**. The stereoselectivity of the reaction is expressed as 1,2‐*cis*:1,2‐*trans* and based on the ^1^H‐NMR spectroscopy. The isolated yield of the glycosylation is given in parentheses. Experimental conditions: pre‐activation based glycosylation conditions; Ph_2_SO (1.3 equiv.), TTBP (2.5 equiv.), DCM (0.05 M), *then* Tf_2_O (1.3 equiv.), *then* nucleophile (2 equiv.), −80 °C to −60 °C.

	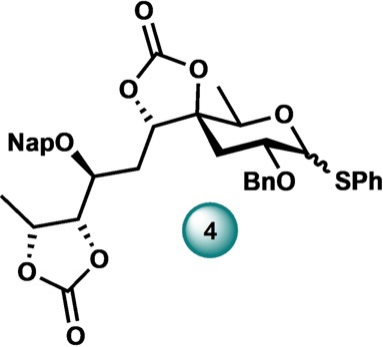	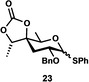
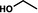	67:33 (94 %)	50:50 (60 %)
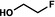	83:17 (100 %)	66:34 (76 %)
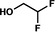	87:13 (63 %)	80:20 (100 %)
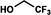	>98:2 (76 %)	>98:2 (77 %)
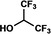	>98:2 (16 %)	>98:2 (28 %)
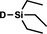	donor hydrolysis	>98:2 (54 %)
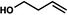	63:37 (97 %)	59:41 (86 %)
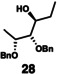	77:23 (50 %)	61:39 (63 %)
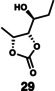	>98:2 (54 %)	>98:2 (74 %)

To evaluate nucleophiles relevant for the assembly of LOS IV‐fragment **1**, three acceptors (3‐butene‐1‐ol, **28**, and **29**) were probed. Acceptors **28** and **29** represent truncated versions of the caryophyllose acceptor, and 3‐butene‐1‐ol will be used to serve as a conjugation‐ready linker moiety. Acceptor **28** is protected with benzyl groups, known to be electronically neutral, while **29** is protected with an electron‐withdrawing carbonate group. The difference in reactivity between these two acceptors is mirrored in the stereoselectivity of the glycosylation reactions with donors **4** and **23**, with the more nucleophilic dibenzylated alcohol **28** providing an α/β‐mixture, while the less nucleophilic alcohol **29**, exclusively formed the 1,2‐*cis*‐product. These results indicate the need for an electron‐withdrawing protecting group on the caryophyllose building block, when employed as an acceptor. The cyclic carbonate spanning hydroxyl groups at C10 and C11 in the synthesized caryophyllose building blocks thus serves this purpose.

**Figure 3:**
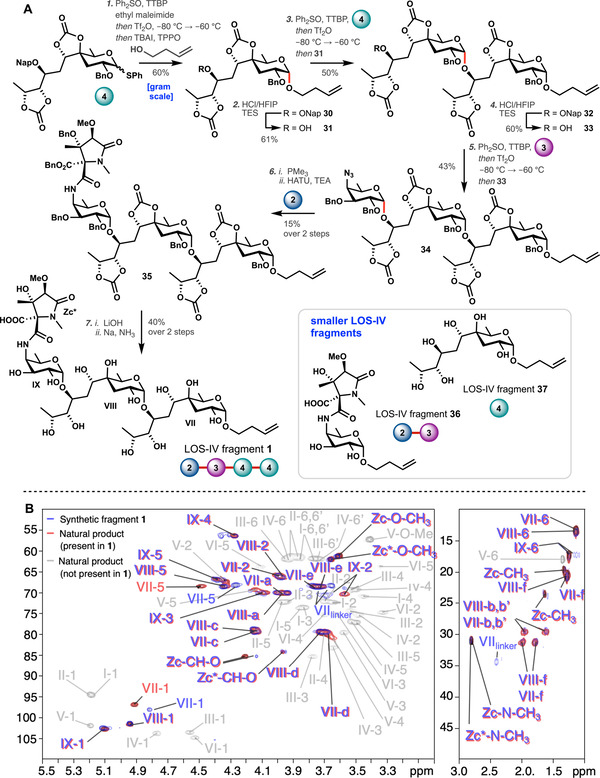
A) Assembly of LOS‐IV fragment **1**. *Reagents and conditions*: (1) Ph_2_SO, TTBP, *N*‐ethyl maleimide, *then* Tf_2_O, *then* TBAI, TPPO, *then* 3‐buten‐1‐ol, −80 °C to 40 °C (60 %); (2) HCl/HFIP, TES, DCM (61 %); (3) Ph_2_SO, TTBP, DCM, *then* Tf_2_O, *then* **31**, −80 °C to −60 °C (50 %); (4) HCl/HFIP, TES, DCM (60 %); (5) Ph_2_SO, TTBP, DCM, *then* Tf_2_O, *then* **33**, −80 °C to −60 °C (43 %); (6) *i*. trimethylphosphine, THF *ii*. **2**, TEA, HATU, CH_3_CN (15 % over 2 steps); (7) *i*. LiOH, H_2_O, THF *ii*. Na, NH_3_, *t*‐BuOH, THF (40 % over 2 steps). Tf_2_O=trifluoromethanesulfonic anhydride, TTBP=2,4,6‐tri‐*t*‐butylpyrimidine, TBAI=*t*‐butylammonium iodide, TPPO=triphenylphosphine oxide, HFIP=hexafluorisopropanol, TES=triethylsilane, HATU=1‐[bis(dimethylamino)methylene]‐1*H*‐1,2,3‐triazolo[4,5‐*b*]pyridinium 3‐oxide hexafluorophosphate, TEA=triethylamine. B) ^1^H‐^13^C HSQC NMR overlay of the acidic OS‐IV fraction isolated by Rombouts et al.^[12]^ (red=residues of the natural product present in the synthesized fragment **1**, and grey=residues absent of the natural product in the synthesized fragment **1**, and blue=synthesized compound **1**). I to IX correspond to the nine monosaccharides of the OS‐IV. In the overlay most signals overlap. Only signals close to the linker on VII are slightly off, because this area is different from the natural compound, which is linked to a xylose.

After having established the glycosylation properties of the donors, we undertook the construction of the target Car‐Car‐NAcFuc LOS‐IV fragment **1** from building blocks **2**, **3**, and **4** (Figure 3 A). Because of the high reactivity of 3‐butene‐1‐ol, we sought to modify the reactivity of the reactive intermediates formed upon activation of the donor glycoside. To this end we turned to the use of an additive‐mediated glycosylation strategy. Various strategies have recently been developed to use exogenous nucleophiles to generate reactive intermediates of which the reactivity can be tuned to match the reactivity of the nucleophile that is to be glycosylated. Based on the work of Mukaiyama and co‐workers,[^54^, ^55^, ^56^, ^57^] and others,^[58]^ we have introduced triphenylphosphine oxide (TPPO)^[59]^ to modulate the reactivity of anomeric iodides, in which the anomeric α‐iodide serves as a reservoir for the more reactive β‐iodide (or β‐phosphoniumiodide), which is the actual glycosylating species (See SI for the complete reactivity–selectivity mapping study with additives). Thus, caryophyllose **4** was pre‐activated in the usual manner, after which a mixture of tetrabutylammonium iodide and TPPO was added, and subsequently 3‐butene‐1‐ol was added. This led to the generation of spacer‐equipped caryophyllose **30** in 60 % yield and excellent stereoselectivity (>98:2; *cis*:*trans*). Subsequent HCl‐mediated deprotection of the 2‐methylnaphthyl protection group yielded caryophyllose acceptor **31** (61 %).^[60]^ Coupling of this acceptor with donor **4** using pre‐activation conditions afforded disaccharide **32** in 50 % yield and with complete 1,2‐*cis*‐selectivity, in line with the results obtained with the model acceptor. Deprotection of the 2‐methylnaphthyl protection group of Car‐Car **32** required more acid compared to the deprotection of **30**, because of the presence of more Lewis basic entities in the substrate, but furnished acceptor **33** in a similar yield (60 %). Coupling of acceptor **33** to 4‐azidofucose donor **3** under pre‐activation conditions provided **32** in 43 % yield with the exclusive formation of the 1,2‐*cis*‐product (See SI for the complete reactivity–selectivity mapping study performed with this donor). A Staudinger reduction was used to generate the free amine. Surprisingly, this transformation proceeded very sluggishly (reduction of the 4‐azido fucose monosaccharide proceeded readily with TPP in 79 % yield, see SI) even with the more reactive trimethyl phosphine. The crude product was directly coupled to the pyrrolidone **2**, to yield the completely protected Car‐Car‐NAcFuc LOS‐IV fragment **35**. Deprotection was done by saponification of the carbonate protection groups and the benzyl ester on the pyrrolidone, followed by debenzylation under Birch condition, to successfully yield the target structure **1** in 40 % yield over the two deprotection steps.

The structure and purity of compound **1**, were confirmed by NMR and HRMS analysis. It was observed that **1** exists as a mixture of atropisomers^[61]^, in line with the behavior of related pyrrolidone‐4‐aminofucose monosaccharides, prepared by Lowary and co‐workers.^[38]^ Figure 3 B compares the ^1^H‐^13^C HSQC NMR spectra of the synthetic LOS‐IV fragment **1** with the natural product, isolated by Rombouts et al.^[12]^ The blue signals originate from the synthesized compound, the red signals are from the natural product, and all residues from the natural product that are absent in the synthetic fragment are grey. From the overlay it is apparent that the spectra closely match, indicating that the assembled fragment resembles the natural product very well.

## Conclusion

In conclusion, we have reported a systematic evaluation of the reactivity of tertiary‐*C* sugar building blocks, caryophyllose and yersiniose. An approach, consisting of a systematic series of glycosylation reactions in combination with the detection and characterization of different reactive intermediates using variable‐T NMR and conformational energy landscape computations, were used to assess reactivity–stereoselectivity relationships. We found for these 4‐*C*‐branched sugars that ether functionalities in the appended side‐chain readily attack the activated anomeric center of the caryophyllose and yersiniose donors, leading to unproductive glycosylation reactions. This behavior has been explained using the conformational preference of oxocarbenium ion intermediates that can form. Prevention of this nucleophilic attack is a prerequisite to generate effective donor glycosides and could be achieved by tethering of the C4 side‐chain. We found that tethered Car and YerA donors can efficiently form the desired 1,2‐*cis* linkages, as long as weak nucleophiles are employed in the glycosylation. In order to achieve 1,2‐*cis*‐selectivity, the reactivity of the Car‐acceptors was tuned using electron‐withdrawing protecting groups. The rationally designed building blocks enabled the first effective and stereoselective assembly of a Car‐Car‐NAcFuc LOS‐IV fragment, and related shorter fragments. The approach taken here can be used to uncover the reactivity of rare bacterial saccharides. The insight gathered will be a solid base to inform future syntheses of bacterial oligosaccharides and glycoconjugates to fuel immunological‐ and biological research.

## Conflict of interest

The authors declare no conflict of interest.

## Supporting information

As a service to our authors and readers, this journal provides supporting information supplied by the authors. Such materials are peer reviewed and may be re‐organized for online delivery, but are not copy‐edited or typeset. Technical support issues arising from supporting information (other than missing files) should be addressed to the authors.

Supplementary
