# Supplementary material for: Reactivity–Stereoselectivity Mapping for the Assembly of Mycobacterium marinum Lipooligosaccharides
Source: Angew Chem Int Ed Engl. 2020 Nov 3;60(2):937–45. doi: 10.1002/anie.202010280 (PMC7821131; doi:10.1002/anie.202010280)

Angewandte Chemie
Eine Zeitschrift der Gesellschaft Deutscher Chemiker

Supporting Information

Reactivity–Stereoselectivity Mapping for the Assembly of *Mycobacterium marinum* Lipooligosaccharides

*Thomas Hansen[+], Tim P. Ofman[+], Joey G. C. Vlaming[+], Ivan A. Gagarinov, Jessey van Beek, Tessa A. Goté, Jacoba M. Tichem, Gijs Ruijgrok, Herman S. Overkleeft, Dmitri V. Filippov, Gijsbert A. van der Marel, and Jeroen D. C. Codée**

anie_202010280_sm_miscellaneous_information.pdf

Author Contributions

T.H., G.A.M., J.D.C.C. designed the experiments. T.H., T.P.O., J.G.C.V., I.A.G., J.B., T.A.G., J.M.T., G.R., carried out the experimental work. T.H. performed all computational work. T.H., T.P.O., J.G.C.V., I.A.G., J.B., T.A.G., J.M.T., G.R., H.S.O., D.V.F., G.A.M., J.D.C.C. were involved in scientific discussions and critically reviewed the manuscript.

Supplementary information

Reactivity-selectivity mapping of 4-azidofucose donor 3 S3
Additives controlled model glycosylation reactions S4
DFT calculations S5
General procedure I: CEL calculations of pyranosyl oxocarbenium ions S5
Variable-temperature NMR S6
General procedure II: pre-activation Tf_2O/Ph_2SO based variable-temperature NMR S6
Results of compound **25** S7
Results of compound **24** S7
Results of compound **3** S8
Results of compound **23** S9
Results of compound **4** S9
Organic synthesis S11
General experimental procedures S11
General procedure III: pre-activation Tf_2O/Ph_2SO based glycosylation S11
General procedure IV: DMF assisted pre-activation Tf_2O/Ph_2SO based glycosylation S12
General procedure V: TBAI assisted pre-activation Tf_2O/Ph_2SO based glycosylation S12
General procedure VI: TMSOTf activation based glycosylation of imidates S12
Preparation of the building blocks S12
Synthesis of compound **4** S12
Synthesis of compound **3** S23
Synthesis of compound **2** S26
Synthesis of compound **23** S32
Synthesis of compound **24** and **S36** S37
Synthesis of compound **25** S44
Preparation of the target compounds S47
Synthesis of compound **1** S47
Synthesis of compound **36** and **S44** S53
Synthesis of compound **37** S56

Preparation of model acceptors S57
Synthesis of acceptor **28** S57
Synthesis of acceptor **29** S59
Model glycosylation reactions S60
Results of compound **4** S60
Results of compound **23** S65
Results of compound **24** S69
Results of compound **S36** S69
Results of compound **25** S72
Results of compound **3** S72
Structural proofs S76
NMR spectra of new and selected compounds S84

Reactivity-selectivity mapping of 4-azidofucose donor 3

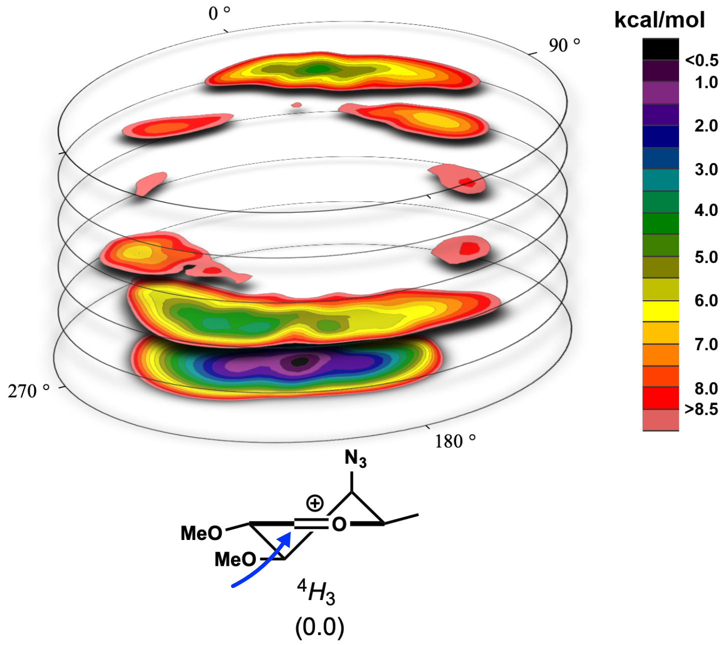

Figure S1. Conformational energy landscape (CEL) maps of 4-azidofucose pyranosyl oxocarbenium ions in which the found local minima are indicated with their respective energy. All energies are as computed at PCM(CH_2Cl_2)-B3LYP/6-311G(d,p) at T = 213.15 K and expressed as solution-phase Gibbs free energy.

Table S1. Experimentally found stereoselectivities for model glycosylation reactions with ethanol, 2-fluoroethanol, 2,2-difluoroethanol, 2,2,2-trifluoroethanol, 1,1,1,3,3,3-hexafluoro-2-propanol, triethylsilane-*d*, 1-buten-4-ol, **28** and **29**. The stereoselectivity of the reaction is expressed as α:β and based on the ^{1}H-NMR spectroscopy. Results of the glycosylation study. Experimental conditions: pre-activation based glycosylation conditions; Ph_2SO (1.3 eq.), TTBP (2.5 eq.), DCM (0.05 M), *then* Tf_2O (1.3 eq.), *then* nucleophile (2 eq.), –80 °C to –60 °C.

	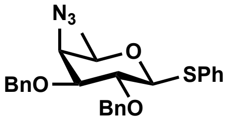 3
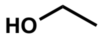	36:64 (87%)
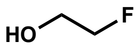	48:52 (100%)
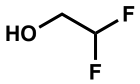	77:23 (91%)
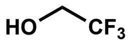	>98:2 (70%)
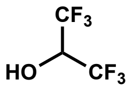	>98:2 (69%)
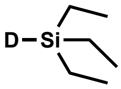	>98:2 (82%)

	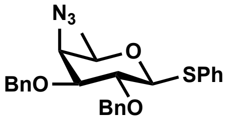 3
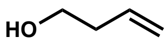	39:61 (85%)
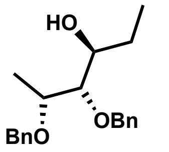	58:42 (76%)
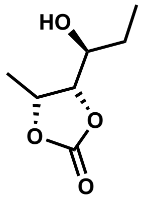	>98:2 (66%)

>90:10	>80:20	>60:40	>50:50	<50:50	<40:60	<20:80	<10:90	(1,2-*cis*:1,2-*trans*)

Additives controlled model glycosylation reactions

Table S2. Experimentally found stereoselectivities for model glycosylation reactions with additives including DMF (16 eq) and TBAI (8 eq). The stereoselectivity of the reaction is expressed as α:β and based on the ^{1}H-NMR spectroscopy. Experimental conditions: pre-activation based glycosylation conditions; Ph_2SO (1.3 eq.), TTBP (2.5 eq.), DCM (0.05 M), *then* Tf_2O (1.3 eq.), *then* nucleophile (2 eq.), –80 °C to –60 °C.

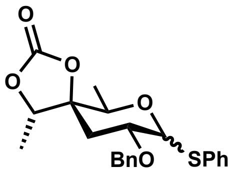

23

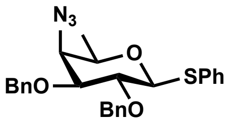

3

Entry	donor	$HOCH_2CF_3$	$HOCH_2CHF_2$	$HOCH_2CH_2F$	$HOCH_2CH_3$	$HOCH_2CH_2CH=CH_2$
1	**23**	>98:2 (77%)	80:20 (100%)	66:34 (76%)	50:50 (60%)	60:40 (86%)
2	+DMF	>98:2 (36%)	88:12 (100%)	81:19 (85%)	63:37 (72%)	67:33 (55%)
3	+TBAI	donor hydrolysis	>98:2 (16%)	>98:2 (65%)	>98:2 (62%)	>98:2 (61%)
4	**3**	>98:2 (70%)	77:23 (91%)	48:52 (100%)	36:64 (87%)	39:61 (85%)
5	+DMF	>98:2 (73%)	>98:2 (85%)	81:19 (100%)	62:38 (93%)	63:37 (61%)
6	+TBAI	donor hydrolysis	>98:2 (81%)	>98:2 (79%)	>98:2 (75%)	>98:2 (95%)

>90:10	>80:20	>60:40	>50:50	<50:50	<40:60	<20:80	<10:90	(1,2-*cis*:1,2-*trans*)

Model glycosylation reaction with imidate donor

Table S3. Experimentally found stereoselectivities for model glycosylation reactions. The stereoselectivity of the reaction is expressed as α:β and based on the ^{1}H-NMR spectroscopy. Experimental conditions: acceptor (2.0 eq.), DCM (0.05 M), *then* TMSOTf (0.5 M solution in DCM) (2 eq.), –80 °C to –10 °C.

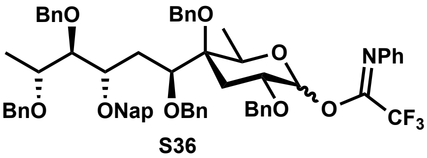

S36

Entry	donor	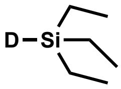	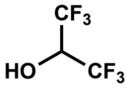	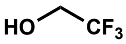	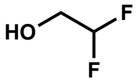	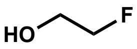	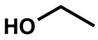
1	**25**	by-product **26**	by-product **26**	>98:2 (68%)	63:37 (86%)	33:67 (70%)	25:75 (100%)

DFT calculations

General procedure I: conformational energy landscape calculation of pyranosyl oxocarbenium ions

To keep the calculation time manageable, large protection groups (OBn) were substituted with electronically comparable smaller groups (OMe). The initial structure for the conformational energy landscape (CEL) was optimized by starting from a ‘conformer distribution search’ option included in the Spartan 10 program by utilizing DFT as the level of theory and the hybrid functional B3LYP in gas phase with 6-31G(d) as the basis set. All generated gas-phase geometries were re-optimized with Gaussian 09 rev D.01 by using B3LYP/6-311G(d,p), after which a vibrational analysis was computed to obtain the thermodynamic properties. The gas-phase structures were than solvated by using the PCM implicit solvation model, with CH_2Cl_2 as solvent. Solvent effects were explicitly used in the solving of the SCF equations and during the optimization of the geometry. The geometry with the lowest solvated energy was selected as the starting point for the CEL map. A complete survey of the possible conformational space was done by scanning three dihedral angles ranging from –60° to 60°, including the C1-C2-C3-C4 (D1), C3-C4-C5-O (D3) and C5-O-C1-C2 (D5). The resolution of this survey is determined by the step size which was set to 15° per puckering parameter, giving a total of 729 pre-fixed conformations per six-membered oxocarbenium ion spanning the entire conformational landscape. All other internal coordinates were unconstrained. With the exception of a C2-substituent being present on the oxocarbenium ring of interest, then the C2-H2 bond length was fixed based on the optimized structure to counteract rearrangements occurring for higher energy conformers. The 729 structures were computed with Gaussian 09 rev D.01 again with a two-step procedure. First, the structures were optimized in the gas-phase with B3LYP/6-311G(d,p), after which a vibrational analysis was computed to obtain the thermodynamic properties. The gas-phase structures were than solvated by using the PCM implicit solvation model, with CH_2Cl_2 as solvent. Solvent effects were explicitly used in the solving of the SCF equations and during the optimization of the geometry. The final denoted free Gibbs energy was calculated using Equation (S1) in which ΔE_{gas} is the gas-phase energy (electronic energy), $\Delta G^{T}_{gas,QH}$ (T = reaction temperature and p = 1 atm.) is the sum of corrections from the electronic energy to free Gibbs energy in the quasi-harmonic oscillator approximation also including zero-point energy (ZPE), and ΔG_{solv} is their corresponding free solvation Gibbs energy. The $\Delta G^{T}_{gas,QH}$ were computed using the quasi-harmonic approximation in the gas phase according to the work of Truhlar.

$$\Delta G^{T}_{CH_2Cl_2} = \Delta E_{gas} + \Delta G^{T}_{gas,QH} + \Delta G_{solv} \qquad \text{(Eq. S1)}$$

$$= \Delta G^{T}_{gas} + \Delta G_{solv}$$

The quasi-harmonic approximation is the same as the harmonic oscillator approximation except that vibrational frequencies lower than 100 cm^{-1} were raised to 100 cm^{-1} as a way to correct for the breakdown of the harmonic oscillator model for the free energies of low-frequency vibrational modes. All found minima were checked for imaginary frequencies. To visualize the energy levels of the conformers on the Cremer-Pople sphere, slices were generated dissecting the sphere that combine closely associated conformers (Figure S1). The OriginPro software was employed to produce the energy heat maps, contoured at 0.5 kcal/mol. For ease of visualization, the Cremer-Pople globe is turned 180° with respect to its common representation and both poles (the $^{4}C_{1}$ and $^{1}C_{4}$ structures) are omitted as these conformations are very high in energy. Visualization of conformations of interest was done with CYLview.

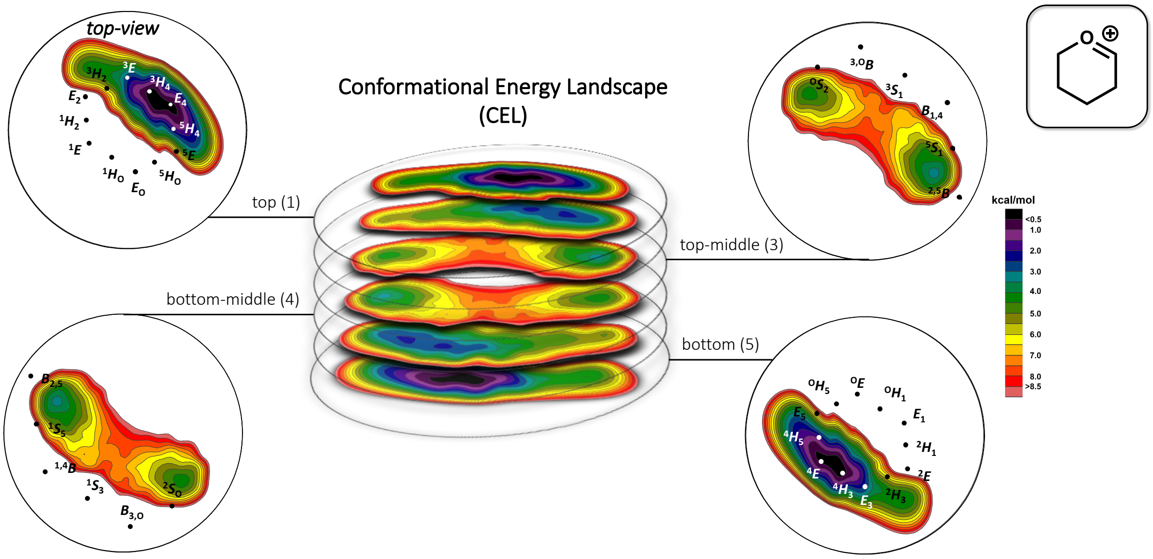

Figure S2.“Deconvolution” of the CEL map showing a top view of the most important slices that have been combined to generate the full CEL map.

Variable-temperature NMR

General procedure II: pre-activation Tf_2O/Ph_2SO based variable-temperature NMR

A mixture of the donor (30 µmol, 1 eq.), Ph_2SO (8.0 mg, 39 µmol, 1.3 eq.) and TTBP (19 mg, 75 µmol, 2.5 eq.) were co-evaporated with toluene (3x). Under a nitrogen atmosphere, CD_2Cl_2 was added after which the mixture was transferred to a nitrogen flushed NMR tube that was then closed with an NMR septum. The NMR magnet was cooled to –80 °C, locked and shimmed prior to activation. The sample was cooled in an ethanol bath of –80 °C, upon which Tf_2O (6.6 µL, 39 µmol, 1.3 eq.) was added, the tube was shaken three times, wiped clean and rapidly inserted back in the NMR magnet. The sample was then re-shimmed and spectra were recorded with 10 °C intervals, securing the temperature to be stable. At –60 °C full characterization of the reactive species was performed by taking ^{13}C, HH-COSY, HSQC, and ^{19}F NMR. ^{1}H spectra were recorded with increasing temperature until degradation was observed.

Results of compound **25**

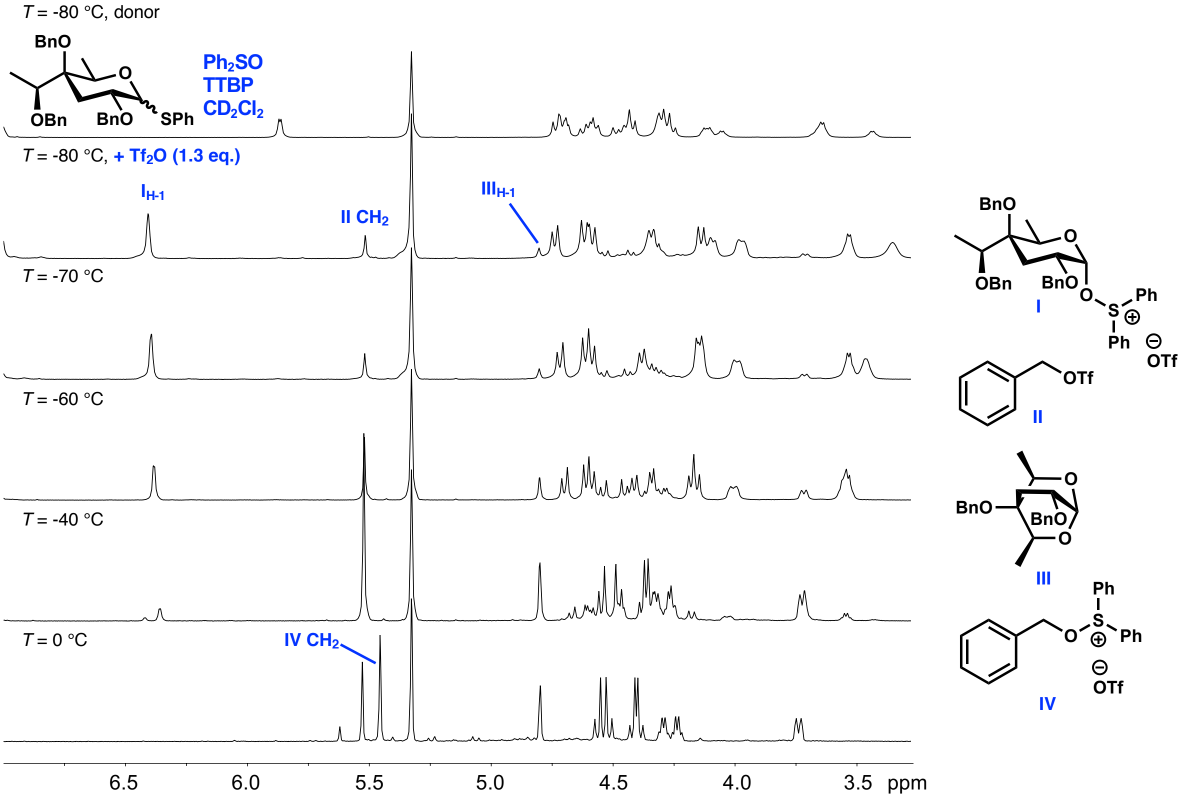

Figure S3. Variable-T NMR of donor **25** under pre-activation conditions.

Results of compound **24**

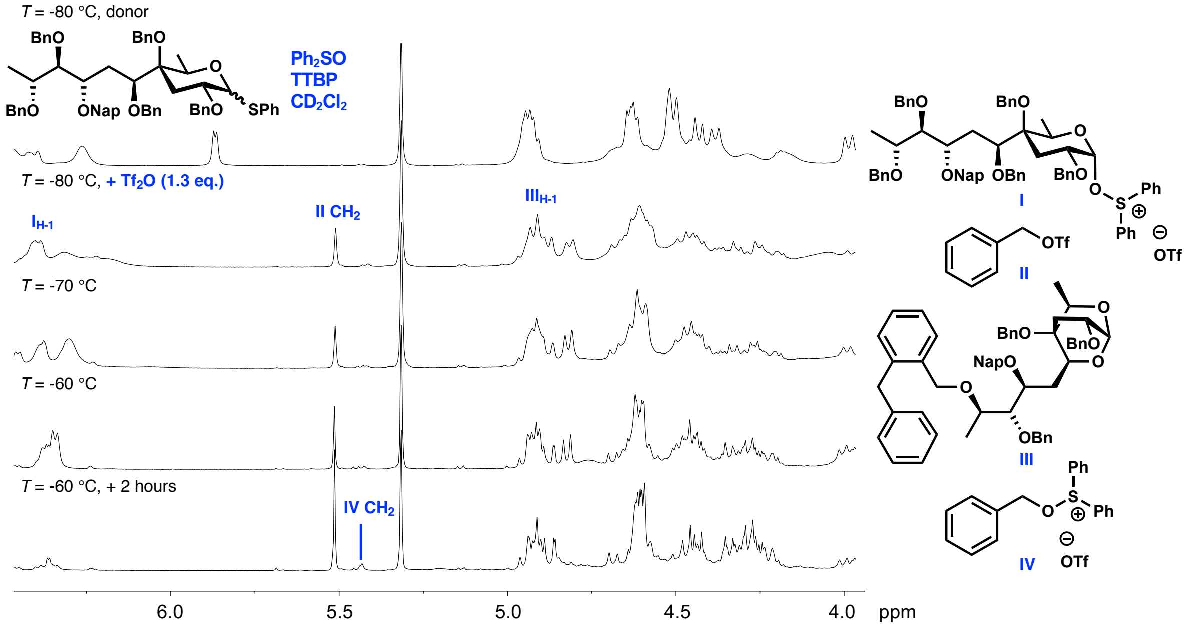

Figure S4. Variable-T NMR of donor **24** under pre-activation conditions.

Results of compound **3**

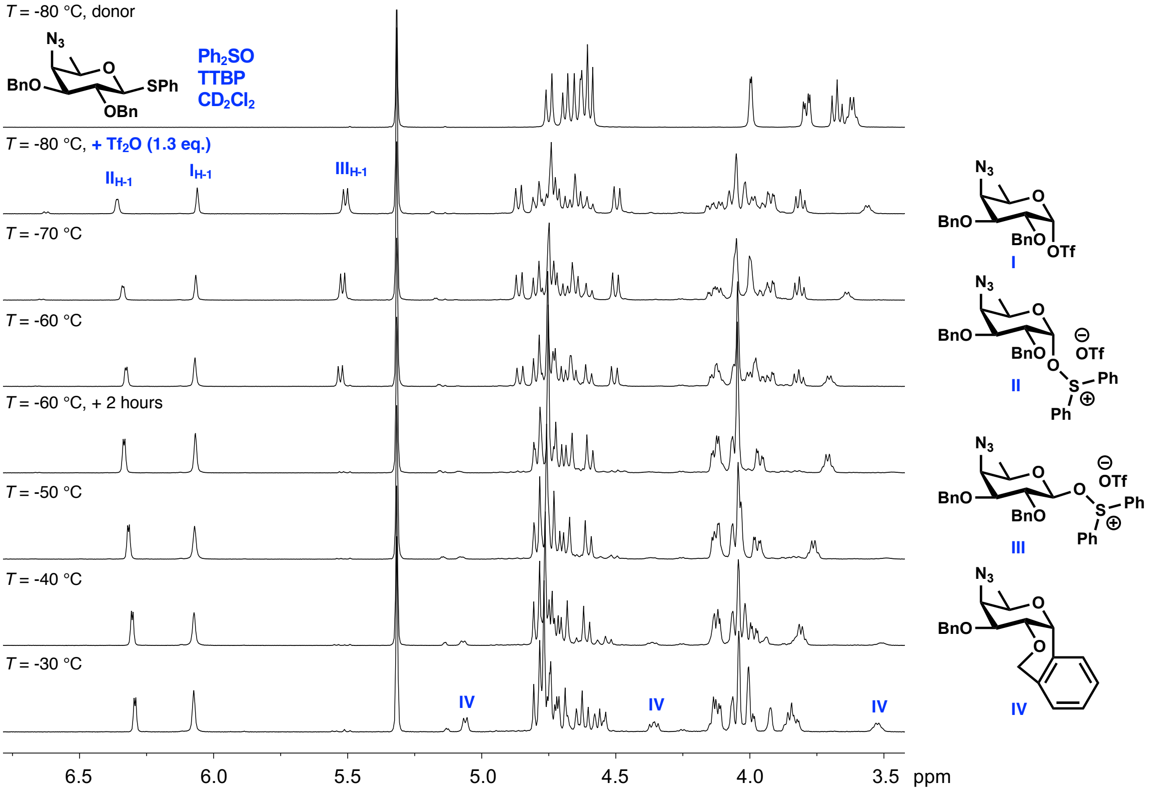

Figure S5. Variable-T NMR of donor **3** under pre-activation conditions.

Results of compound **3** (+DMF)

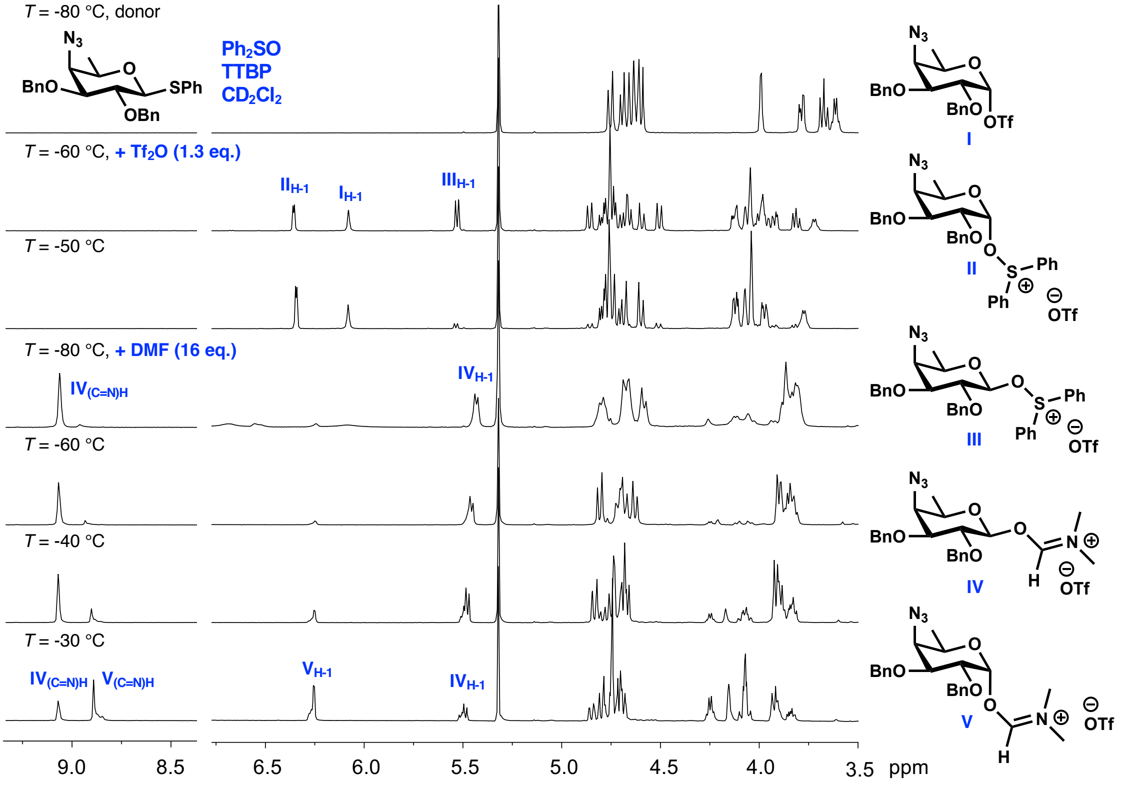

Figure S6. Variable-T NMR of donor **3** under pre-activation conditions with DMF as additive.

Results of compound **23**

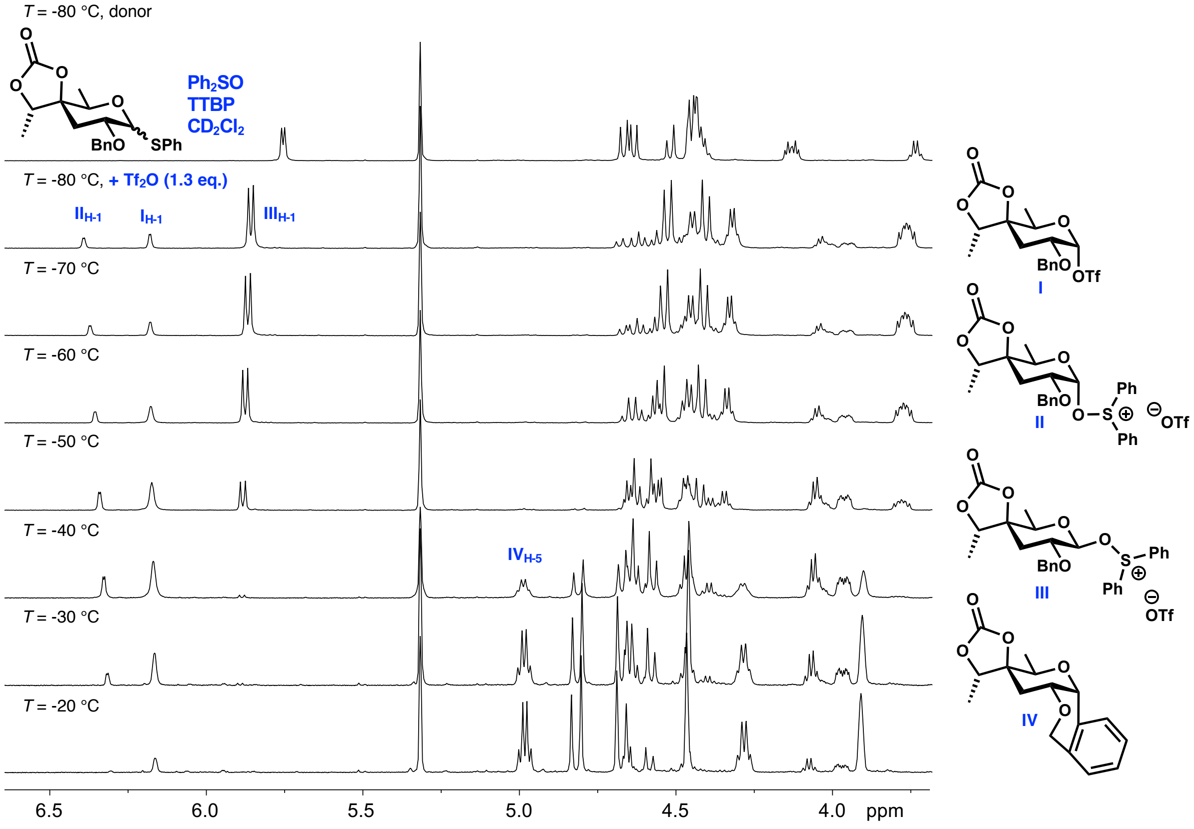

Figure S7. Variable-T NMR of donor **23** under pre-activation conditions.

Results of compound **23** (+extra Ph_2SO)

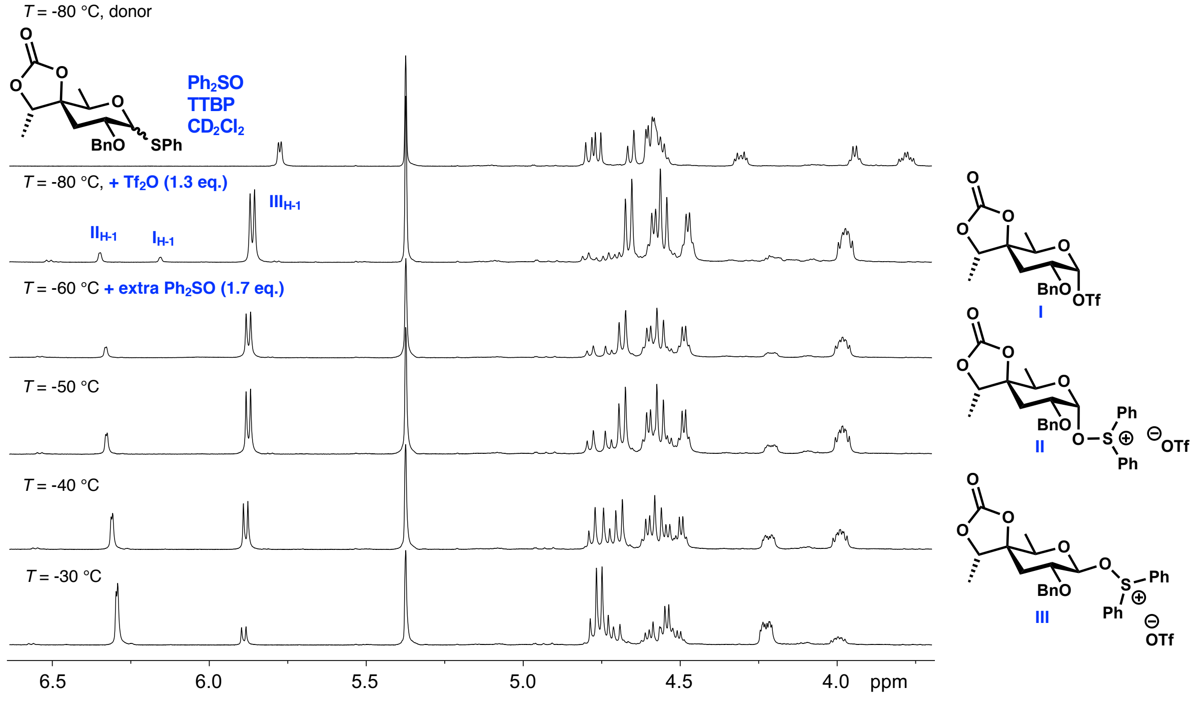

Figure S8. Variable-T NMR of donor **23** under pre-activation conditions with extra Ph_2SO.

Results of compound **4**

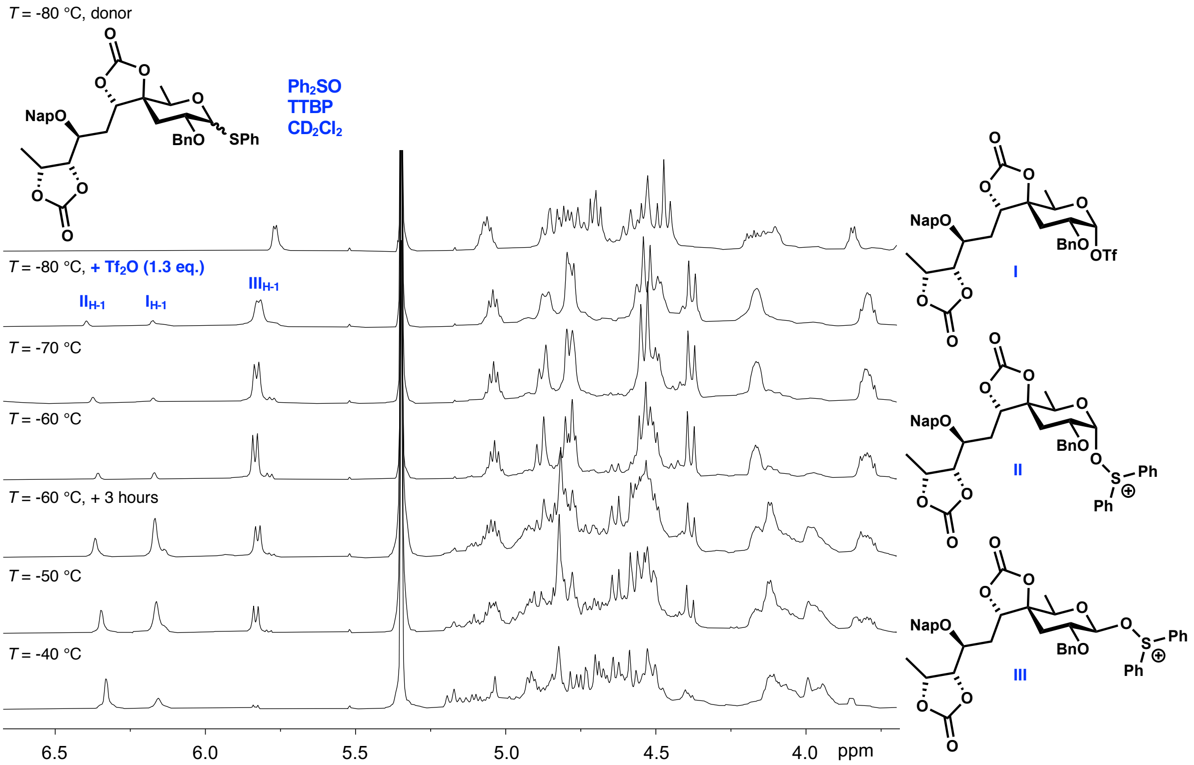

Figure S9. Variable-T NMR of donor **4** under pre-activation conditions.

Organic synthesis

General experimental procedures

All chemicals (Merck, Sigma-Aldrich, Alfa Aesar, Honeywell, Boom and Merck KGaA) were of commercial grade and were used as received unless stated otherwise. Dichloromethane, tetrahydrofuran and toluene were stored over activated 4 Å molecular sieves (beads, 8-12 mesh, Sigma-Aldrich). Before use traces of water present in the donor, diphenyl sulfoxide (Ph_2SO) and tri-*tert*-butylpyrimidine (TTBP) were removed by co-evaporation with dry toluene. The acceptors used in the model glycosylation reactions (ethanol, 2-fluoroethanol, 2,2-difluoroethanol and 2,2,2,-trifluoroethanol, 1,1,1,3,3,3-hexafluoro-2-propanol, triethylsilane-*d* and 3-buten-1-ol) were stored in stock solutions (DCM, 0.5 M) over activated 3 Å molecular rods (rods, size 1/16 in., Sigma Aldrich). Trifluoromethanesulfonic anhydride (Tf_2O) was distilled over P_2O_5 and stored at –20 °C under a nitrogen atmosphere. Deuterated chloroform was stored over activated 3 Å molecular rods (rods, size 1/16 in., Sigma Aldrich) and potassium carbonate. Flash column chromatography was performed on silica gel 60 Å (0.04 – 0.063 mm, Screening Devices B.V.). Size exclusion chromatography was performed on SephadexTM (LH-20, GE Healthcare Life Sciences) by isocratic elution with DCM:MeOH (1:1, v:v). TLC-analysis was performed on TLC Silica gel 60 (Kieselgel 60 F254, Merck) with UV detection (254 nm) and by spraying with 20% H_2SO_4 in ethanol followed by charring at ±260 °C or by spraying with a solution of $(NH_4)_6Mo_7O_{24}\cdot H_2O$ (25 g/L) and $(NH_4)_4Ce(SO_4)_4\cdot 2H_2O$ (10 g/L) in 10% sulfuric acid in water followed by charring at ± 260 °C. TLC-MS analysis was performed on a Camag TLC-MS Interface coupled with an API165 (SCIEX) mass spectrometer (eluted with *tert*-butylmethylether/EtOAc/MeOH, 5/4/1, v/v/v +0.1% formic acid, flow rate 0.12 mL/min). High-resolution mass spectra (HRMS) were recorded on a Waters Synapt G2-Si (TOF) equipped with an electrospray ion source in positive mode (source voltage 3.5 kV) and an internal lock mass LeuEnk ($M+H^+$ = 556.2771). Amberlite resin (Sigma Aldrich Amberlite IR120 H^+ form, Amberlite IRA-67 free base) was pre-washed with MeOH. ^1H and ^{13}C NMR spectra were recorded on a Bruker AV-400 NMR instrument (400 and 101 MHz respectively), a Bruker AV-500 NMR instrument (500 and 126 MHz respectively), a Bruker AV-600 NMR instrument (600 and 151 MHz respectively) or a Bruker AV-850 NMR instrument (850 and 214 MHz respectively. All samples were measured in $CDCl_3$, unless stated otherwise. Chemical shifts (δ) are given in ppm relative to tetramethylsilane as internal standard or the residual signal of the deuterated solvent. Coupling constants (*J*) are given in Hz. All given ^{13}C APT spectra are proton decoupled. NMR peak assignment was accomplished using COSY, HSQC. If necessary, additional NOESY, HMBC, and HMBC-gated experiments were used to further elucidate the structure. Stereochemical product ratios were based on integration of ^1H NMR (crude and purified). IR spectra were recorded on a Shimadzu FTIR-8300 IR spectrometer and are reported in cm^{-1}. Specific rotations were measured on an Anton Paar Polarimeter MCP 100 in $CHCl_3$ (10 mg/mL) at 589 nm, unless stated otherwise.

General procedure III: pre-activation Tf_2O/Ph_2SO based glycosylation

To a solution of the donor (50 µmol, 1 eq.) in DCM (1 mL, 0.05 M), Ph_2SO (13 mg, 65 µmol, 1.3 eq.) and TTBP (31 mg, 125 µmol, 2.5 eq.) were added. The solution was stirred over activated 3 Å molecular sieves (rods, size 1/16 in., Sigma Aldrich) for 30 min. The solution was cooled to –80 °C upon which Tf_2O (11 µL, 65 µmol, 1.3 eq.) was added slowly (5 seconds). Subsequently, the solution was allowed to attain to –60 °C to secure full activation of the donor followed by cooling back to –80 °C after which the acceptor was added (0.2 mL, 0.5 M solution, 2.0 eq.). The reaction was stirred for 16 h at –60 °C (for ethanol, 2-fluoroethanol, 2,2-difluoroethanol and 2,2,2-trifluoroethanol) or for 40 h at –60 °C (for 1,1,1,3,3,3-hexafluoro-2-propanol and triethylsilane-*d*). The reaction was quenched with sat. aq. $NaHCO_3$ followed by the dilution with EtOAc. The aqueous layer was extracted three times with EtOAc. The combined organic layers were washed with H_2O and brine, dried over $MgSO_4$, filtered off and concentrated under reduced pressure. Purification was performed by flash column chromatography to afford the corresponding coupled glycoside.

General procedure IV: DMF assisted pre-activation Tf_2O/Ph_2SO based glycosylation
To a solution of the donor (50 µmol, 1 eq.) in DCM (1 mL, 0.05 M), Ph_2SO (13 mg, 65 µmol, 1.3 eq.) and TTBP (31 mg, 125 µmol, 2.5 eq.) were added. The solution was stirred over activated 3 Å molecular sieves (rods, size 1/16 in., Sigma Aldrich) for 30 min. The solution was cooled to –80 °C upon which Tf_2O (11 µL, 65 µmol, 1.3 eq.) was added slowly. Subsequently, the solution was allowed to attain to –60 °C to secure full activation of the donor followed by cooling back to –80 °C after which DMF (61 µL, 0.8 mmol, 16 eq.) was added. The solution was stirred for 15 min at –80 °C followed by the addition of the acceptor (0.2 mL, 0.5 M solution, 2.0 eq.). The reaction was stirred overnight at 0 °C upon which the reaction was quenched with sat. aq. $NaHCO_3$ followed by the dilution with EtOAc. The aqueous layer was extracted three times with EtOAc. The combined organic layers were washed with H_2O and brine, dried over $MgSO_4$, filtered off and concentrated under reduced pressure. Purification was performed by flash column chromatography to afford the corresponding coupled glycoside.

General procedure V: TBAI assisted pre-activation Tf_2O/Ph_2SO based glycosylation
To a solution of the donor (50 µmol, 1 eq.) in DCM (1 mL, 0.05 M), Ph_2SO (13 mg, 65 µmol, 1.3 eq.), TTBP (31 mg, 125 µmol, 2.5 eq.) and ethyl maleimide (12.5 mg, 100 µmol, 2.0 eq) were added. The solution was stirred over activated 3 Å molecular sieves (rods, size 1/16 in., Sigma Aldrich) for 30 min. The solution was cooled to –80 °C upon which Tf_2O (11 µL, 65 µmol, 1.3 eq.) was added slowly. Subsequently, the solution was allowed to attain to –60 °C to secure full activation of the donor followed by cooling back to –80 °C after which TBAI (148 mg, 0.4 mmol, 8 eq.) was added. The solution was stirred for 15 min at –80 °C followed by the addition of the acceptor (0.2 mL, 0.5 M solution, 2.0 eq.). The reaction was stirred overnight at 0 °C upon which the reaction was quenched with sat. aq. $NaHCO_3$ and sat. aq. thiosulfate sol. followed by the dilution with EtOAc. The aqueous layer was extracted three times with EtOAc. The combined organic layers were washed with H_2O and brine, dried over $MgSO_4$, filtered off and concentrated under reduced pressure. Purification was performed by flash column chromatography to afford the corresponding coupled glycoside.

General procedure VI: TMSOTf activation based glycosylation of imidates
A solution of the donor (22.5 µmol, 1.0 eq.) and acceptor (45 µmol, 2.0 eq.) in DCM (450 µL, 0.05 M) was stirred over activated 3 Å molecular sieves (rods, size 1/16 in., Sigma Aldrich) for 30 min. The solution was cooled to –80 °C upon which TMSOTf (9.0 µL of a 0.5 M solution, 0.2 eq.) was added slowly. Subsequently, the solution was allowed to attain to –10 °C and stirred for 16 h. The reaction was quenched with sat. aq. $NaHCO_3$ followed by the dilution with EtOAc. The aqueous layer was extracted three times with EtOAc. The combined organic layers were washed with H_2O and brine, dried over $MgSO_4$, filtered off and concentrated under reduced pressure. Purification was performed by flash column chromatography to afford the corresponding coupled glycoside.

Preparation of the building blocks

Synthesis of compound 4

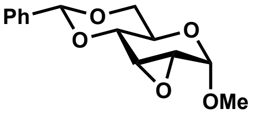

Methyl 2,3-anhydro-4,6-*O*-benzylidene-α-D-allopyranoside (7). Methyl α-D-glucopyranoside (167 g, 860 mmol) was dissolved in dry acetonitrile (1.7 L, 0.5 M), $PhCH(OMe)_2$ (142 mL, 950 mmol, 1.1 eq.) and iodine (21.8 g, 86 mmol, 0.1 eq.) were added. The mixture was stirred for 3 h at 50 °C. The solution was concentrated *in vacuo* and co-evaporated with toluene. The crude solid was recrystallized from EtOAc/pentane to give a white solid. The solid was dissolved in pyridine (1.7 L, 0.5 M), the solution was cooled on ice followed by the dropwise addition of MsCl (200 mL, 2.6 mol, 3.0 eq.), the solution was stirred for 15 h at room temperature. The solution was quenched by diluting with ice water (15 L). The resulting suspension was filtered, followed by washing with water. Co-evaporation with toluene yielded the crude product as a light brown solid. The crude product was divided into two equal portions. The

brown solid was dissolved in a 2:3 mixture of THF/MeOH (3.4 L, 0.125 M), KOH (72.4 g, 1290 mmol, 3.0 eq.) was added, and the solution was refluxed at 80 °C for 15 h, resulting in a thick brown suspension. After cooling to room temperature, both suspensions were combined and diluted with cold water (60 L). Filtration followed by washing with water yielded the crude product. Recrystallization (EtOAc/pentane) yielded the title compound as a white solid (124.7 g, 471.8 mmol, 55% over 3 steps). TLC: R_f 0.4 (pentane:EtOAc, 4:6, v:v); $[\alpha]_D^{20}$ 217.6° (c 0.125, $CHCl_3$); IR (neat, cm^{-1}): 1074, 1144, 1391, 2988; ^1H NMR (400 MHz, $CDCl_3$, HH-COSY, HSQC): δ 7.53 – 7.48 (m, 2H, CH_{arom}), 7.41 – 7.34 (m, 3H, CH_{arom}), 5.58 (s, 1H, CHPh), 4.90 (d, *J* = 2.7 Hz, 1H, H-1), 4.25 (ddd, *J* = 10.1, 5.0, 0.8 Hz, 1H, H-6), 4.09 (ddd, *J* = 10.3, 9.1, 5.0 Hz, 1H, H-5), 3.96 (dd, *J* = 9.1, 1.2 Hz, 1H, H-4), 3.69 (t, *J* = 10.3 Hz, 1H, H-6), 3.53 (d, *J* = 4.4 Hz, 1H, H-3), 3.50 (dd, *J* = 4.3, 2.8 Hz, 1H, H-2), 3.48 (s, 3H, CH_3 OMe); ^{13}C NMR (101 MHz, $CDCl_3$, HSQC): δ 137.2 ($C_{q\text{-}arom}$), 129.4, 128.5, 126.5 (CH_{arom}), 102.9 (CHPh), 95.5 (C-1), 78.0 (C-4), 69.1 (C-6), 60.2 (C-5), 56.1 (CH_3 OMe), 53.3 (C-2), 50.9 (C-3); HRMS: $[M+Na]^+$ calcd for $C_{14}H_{16}O_5Na$ 287.0895, found 287.0897.

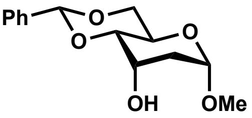

Methyl 4,6-*O*-benzylidene-2-deoxy-D-altropyranoside (8). Compound **7** (124.7 g, 471.8 mmol) was divided into two equal portions of 236 mmol. Compound **7** was dissolved in Et_2O (3.9 L, 0.06 M), and $LiAlH_4$ (119 mL, 476 mmol, 2 eq., 4 M solution in THF) was then added drop-wise. After 2 h of refluxing at 45 °C the mixture was led to cool to room temperature and quenched with 20 mL of water. The excess water was removed by drying over $MgSO_4$, after which the mixture was filtered, and concentrated *in vacuo* to yield a white crystalline solid. The crude products were combined and recrystallized (Et_2O) to afford the title compound (98.5 grams, 369.9 mmol, 78%) as a white solid. TLC: R_f 0.5 (pentane:EtOAc, 4:6, v:v); $[\alpha]_D^{20}$ 84.2° (c 0.5, $CHCl_3$); IR (neat, cm^{-1}): 1045, 1099, 1381, 2932, 3510; ^1H NMR (400 MHz, $CDCl_3$, HH-COSY, HSQC): δ 7.53 – 7.49 (m, 2H, CH_{arom}), 7.40 – 7.33 (m, 3H, CH_{arom}), 5.63 (s, 1H, CHPh), 4.80 (d, *J* = 3.9 Hz, 1H, H-1), 4.33 (dd, *J* = 10.1, 5.1 Hz, 1H, H-6), 4.28 – 4.22 (m, 1H, H-5), 4.19 (dq, *J* = 6.3, 3.1 Hz, 1H, H-3), 3.78 (t, *J* = 10.2 Hz, 1H. H-6), 3.62 (dd, *J* = 9.6, 2.8 Hz, 1H, H-4), 3.42 (s, 3H, CH_3 OMe), 3.03 (d, *J* = 6.7 Hz, 1H, 3-OH), 2.20 (ddd, *J* = 14.9, 3.2, 1.0 Hz, 1H, H-2), 2.01 (dt, *J* = 14.9, 3.7 Hz, 1H, H-2); ^{13}C NMR (101 MHz, $CDCl_3$, HSQC): δ 137.4 ($C_{q\text{-}arom}$), 129.2, 128.4, 126.4 (CH_{arom}), 102.2 (CHPh), 98.8 (C-1), 79.8 (C-4), 69.5 (C-6), 65.2 (C-3), 58.3 (C-5), 55.6 (CH_3 OMe), 35.6 (C-2); HRMS: $[M+Na]^+$ calcd for $C_{14}H_{18}O_5Na$ 289.1052, found 289.1068.

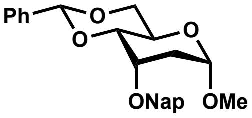

Methyl 4,6-*O*-benzylidene-2-deoxy-3-*O*-(2-methylnaphtalene)-α-D-altropyranoside (9). Compound **8** (98.5 g, 369.9 mmol) was dissolved in DMF (925 mL, 0.4 M) under N_2 atmosphere and cooled on ice. NaH (17.8 g, 443.9 mmol, 1.2 eq., 60% dispersion in mineral oil) was added portion-wise. Subsequently, 2-(bromomethyl)naphthalene (98.1 g, 443.9 mmol, 1.2 eq.) was added portion-wise over a time span of 30 min. The solution was stirred for 1 h after which the solution was concentrated to 1/5th of its original volume. The solution was then quenched with H_2O followed by further dilution with Et_2O and H_2O. The aqueous layer was extracted 5 times with Et_2O after which the combined organic layers were washed with H_2O, sat. aq. $NaHCO_3$ and brine, respectively. The organic layer was dried over $MgSO_4$, filtered, and concentrated *in vacuo* to yield the crude product as a colorless oil. Flash column chromatography (90:10 → 70:30; pentane:EtOAc) yielded the title compound (150.4 g, 369.9 mmol, *quant.*) as a yellow oil. TLC: R_f 0.7 (pentane:EtOAc, 1:1, v:v); $[\alpha]_D^{20}$ 44.3° (c 1.0, $CHCl_3$); IR (neat, cm^{-1}): 474, 699, 748, 1007, 1044, 1099, 1128; ^1H NMR (400 MHz, $CDCl_3$, HH-COSY, HSQC): δ 7.94 – 7.62 (m, 4H, CH_{arom}), 7.60 – 7.33 (m, 8H, CH_{arom}), 5.57 (s, 1H, CHPh), 4.97 (s, 2H, CH_2 Nap), 4.74 (d, *J* = 4.6 Hz, 1H, H-1), 4.49 (td, *J* = 10.1, 5.3 Hz, 1H, H-5), 4.34 (dd, *J* = 10.3, 5.3 Hz, 1H, H-6), 4.01 (q, *J* = 3.0 Hz, 1H, H-3), 3.73 (t, *J* = 10.4 Hz, 1H, H-6), 3.70 (dd, *J* = 9.5, 2.9 Hz, 1H, H-4), 3.44 (s, 3H, CH_3 OMe), 2.24 (dd, *J* = 14.7, 2.5 Hz, 1H, H-2), 1.92 (ddd, *J* = 15.0, 4.6, 3.8 Hz, 1H, H-2); ^{13}C NMR (101 MHz, $CDCl_3$, HSQC):

δ 138.0, 136.7, 133.4, 133.0 ($C_{q\text{-}arom}$), 129.2, 128.5, 128.0, 128.0, 127.8, 126.5, 126.4, 126.0, 126.0, 125.7 (CH_{arom}), 102.4 (CHPh), 98.1 (C-1), 80.5 (C-4), 72.3 (CH_2 Nap), 70.3 (C-3), 69.8 (C-6), 58.4 (C-5), 55.8 (CH_3 OMe), 34.6 (C-2); HRMS: $[M+Na]^+$ calcd for $C_{25}H_{26}O_5Na$ 429.1678, found 429.1680.

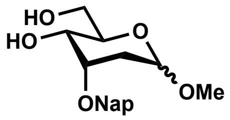

Methyl 2-deoxy-3-*O*-(2-methylnaphtalene)-D-altropyranoside (10). Iodine (9.4 g, 37 mmol, 0.1 eq.) was added to a stirred solution of **9** (150.4 g, 369.9 mmol) in MeOH (1.8 L, 0.2 M). The solution was stirred at room temperature for 18 h after which the reaction was quenched with sat. aq. $Na_2S_2O_3$ and diluted with EtOAc and H_2O. The aqueous layer was extensively extracted with EtOAc, followed by drying the combined organic layers over $MgSO_4$. The organic layer was then filtered, and concentrated *in vacuo* to yield the crude product as a yellow oil. Flash column chromatography (50:50 → 20:80; pentane:EtOAc) yielded the title compound (117.1 g, 368 mmol, 99%, α:β; 50:50) as a colorless oil. TLC: R_f 0.3 (pentane:EtOAc, 1:4, v:v); IR (neat, cm^{-1}): 750, 817, 1041, 2924, 3354; Data of the major stereoisomer (α-anomer): ^1H NMR (400 MHz, $CDCl_3$, HH-COSY, HSQC): δ 7.89 – 7.75 (m, 4H, CH_{arom}), 7.54 – 7.42 (m, 3H, CH_{arom}), 4.95 (d, J = 11.5 Hz, 1H, C*H*H Nap), 4.75 (d, J = 3.5 Hz, 2H, H-1), 4.56 (d, J = 11.5 Hz, 1H, CH*H* Nap), 4.05 – 3.57 (m, 5H, H-3, H-4, H-5, H-6, H-6), 3.40 (s, 3H, CH_3 OMe), 2.72 (bs, 1H, OH), 2.52 (bs, 1H, OH), 2.37 (ddd, J = 15.2, 3.0, 0.9 Hz, 1H, H-2), 1.75 (ddd, J = 15.2, 4.6, 3.4 Hz, 1H, H-2); ^{13}C NMR (101 MHz, $CDCl_3$, HSQC): δ 135.4, 133.2, 133.0 ($C_{q\text{-}arom}$), 128.3, 127.9, 127.7, 126.8, 126.2, 126.0, 126.0 (CH_{arom}), 97.4 (C-1), 72.8 (C-3), 70.6 (CH_2 Nap), 68.2 (C-4), 67.6 (C-5), 63.1 (C-6), 55.3 (CH_3 OMe), 31.2 (C-2); Diagnostic signals of the minor stereoisomer (β-anomer): ^1H NMR (400 MHz, $CDCl_3$, HH-COSY, HSQC): δ 4.89 (d, J = 11.7 Hz, 1H, C*H*H Nap), 4.77 (d, J = 2.0 Hz, 1H, H-1), 4.66 (d, J = 11.7 Hz, 1H, CH*H* Nap), 3.50 (s, 3H, CH_3 OMe), 2.31 (ddd, J = 14.2, 3.7, 2.1 Hz, 1H, H-2), 1.62 (ddd, J = 14.1, 9.4, 2.6 Hz, 1H, H-2); ^{13}C NMR (101 MHz, $CDCl_3$ HSQC): δ 99.1 (C-1), 71.7 (CH_2 Nap), 56.7 (CH_3 OMe), 34.0 (C-2); HRMS: $[M+Na]^+$ calcd for $C_{18}H_{22}O_5Na$ 341.1365, found 341.1364.

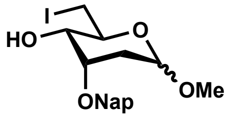

Methyl 2,6-dideoxy-6-*C*-iodo-3-*O*-(2-methylnaphtalene)-D-altropyranoside (11). To a stirred solution of **10** (114.6 g, 360 mmol) in toluene (2.5 L, 0.12 M), imidazole (71.4 g, 1.1 mol, 3.0 eq.) and triphenylphosphine (141.6 g, 540 mmol, 1.5 eq.) were added. The solution was heated to 75 °C upon which an iodine (127.9 g, 504 mmol, 1.4 eq.) solution in toluene (500 mL) was added dropwise over a time span of 15 min. After stirring for 30 min at 75 °C the solution was allowed to cool down to room temperature and quenched with sat. aq. $NaHCO_3$ and sat. aq. $Na_2S_2O_3$ and further diluted with EtOAc. The organic layer was washed with H_2O, sat. aq. $NaHCO_3$ and brine, respectively. Subsequently, the organic layer was dried over $MgSO_4$, filtered, and concentrated *in vacuo* to yield the crude product. Flash column chromatography (90:10 → 60:40; pentane:EtOAc) yielded the title compound (101.7 g, 237.4 mmol, 66%, α:β; 67:33) as a colorless oil. TLC: R_f 0.6 (pentane:EtOAc, 4:1, v:v); IR (neat, cm^{-1}): 750, 815, 1018, 1080, 2926, 3402; Data of the major stereoisomer (α-anomer): ^1H NMR (400 MHz, $CDCl_3$, HH-COSY, HSQC): δ 8.07 – 7.68 (m, 4H, CH_{arom}), 7.63 – 7.38 (m, 3H, CH_{arom}), 4.94 (d, J = 11.4 Hz, 1H, C*H*H Nap), 4.78 (d, J = 4.4 Hz, 1H, H-1), 4.54 (d, J = 11.4 Hz, 1H, CH*H* Nap), 3.88 (q, J = 3.2 Hz, 1H, H-3), 3.79 (ddd, J = 9.7, 7.7, 2.4 Hz, 1H, H-5), 3.64 (dd, J = 10.7, 2.5 Hz, 1H, H-6), 3.49 (s, 3H, CH_3 OMe), 3.40 (td, J = 10.3, 3.7 Hz, 1H, H-4), 3.34 (dd, J = 10.6, 7.6 Hz, 1H, H-6), 2.69 (d, J = 10.8 Hz, 1H, 4-OH), 2.38 (ddd, J = 15.2, 2.9, 1.0 Hz, 1H, H-2), 1.77 (ddd, J = 15.2, 4.5, 3.4 Hz, 1H, H-2); ^{13}C NMR (101 MHz, $CDCl_3$, HSQC): δ 135.3, 133.2, 133.1 ($C_{q\text{-}arom}$), 128.4, 127.9, 127.8, 126.9, 126.3, 126.1, 126.0 (CH_{arom}), 97.7 (C-1), 72.8 (C-3), 71.1 (C-4), 70.7 (CH_2 Nap), 67.9 (C-5), 55.7(CH_3 OMe), 31.4 (C-2), 8.9 (C-6); Diagnostic signals of the minor stereoisomer (β-anomer): ^1H NMR (400 MHz, $CDCl_3$, HH-COSY, HSQC): δ 4.87 (d, J = 11.6 Hz, 1H, C*H*H Nap), 4.75 (dd, J = 9.6, 2.0 Hz, 1H, H-1), 4.60 (d, J =

11.6 Hz, 1H, CH*H* Nap), 3.93 (q, *J* = 3.3 Hz, 1H, H-3), 3.56 (s, 3H, CH_3 OMe), 3.27 (dd, *J* = 10.2, 8.1 Hz, 1H, H-6), 2.47 (d, *J* = 10.7 Hz, 1H, 4-OH), 2.32 (ddd, *J* = 14.2, 3.4, 2.1 Hz, 1H, H-2), 1.64 (ddd, *J* = 14.2, 9.6, 2.6 Hz, 1H, H-2); ^{13}C NMR (101 MHz, $CDCl_3$, HSQC): δ 99.1 (C-1), 75.3 (C-3), 74.4 (C-4), 71.8 (CH_2 Nap), 71.3 (C-5), 56.7 (CH_3 OMe), 34.2 (C-2), 7.7 (C-6); HRMS: $[M+Na]^+$ calcd for $C_{18}H_{21}IO_4Na$ 451.0382, found 451.0385.

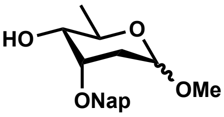

Methyl 2,6-dideoxy-3-*O*-(2-methylnaphtalene)-D-altropyranoside (12). Compound **11** (101.7 g, 237.4 mmol) was dissolved in dry *t*-BuOH (3.4 L, 0.07 M) and stirred under N_2 atmosphere. Subsequently, $NaBH_3CN$ (22.4 g, 356.1 mmol, 1.5 eq) and AIBN (46.8 g, 284.9 mmol, 1.2 eq.) were added. The solution was refluxed at 85 °C for 17 h. After cooling to room temperature, the solution was concentrated to a tenth of its original volume and diluted with EtOAc an H_2O, the aqueous layer was extracted twice, followed by washing the combined organic layers with H_2O, sat. aq. $NaHCO_3$ and brine, respectively. Subsequently, the organic layer was dried over $MgSO_4$, filtered, and concentrated *in vacuo* to yield the crude product as a yellow oil. Flash column chromatography (80:20 → 60:40; pentane:EtOAc) yielded the title compound (52.9 g, 175 mmol, 74%, α:β; 67:33) as a colorless oil. TLC: R_f 0.3 (pentane:EtOAc, 4:1, v:v); IR (neat, cm^{-1}): 748, 817, 1055, 1128, 2927; Data of the major stereoisomer (α-anomer): ^{1}H NMR (400 MHz, $CDCl_3$, HH-COSY, HSQC): δ 7.94 – 7.75 (m, 4H, CH_{arom}), 7.57 – 7.42 (m, 3H, CH_{arom}), 4.95 (d, *J* = 11.6 Hz, 1H, C*H*H Nap), 4.70 (d, *J* = 4.8 Hz, 1H, H-1), 4.57 (d, *J* = 11.6 Hz, 1H, CH*H* Nap), 3.99 (dq, *J* = 9.4, 6.4 Hz, 1H, H-5), 3.88 (q, *J* = 3.4 Hz, 1H, H-3), 3.40 (s, 3H, CH_3 OMe), 3.28 (dd, *J* = 9.4, 3.6 Hz, 1H, H-4), 2.61 – 2.49 (bs, 1H, 4-OH), 2.36 (ddd, *J* = 15.0, 3.1, 1.2 Hz, 1H, H-2), 1.78 (ddd, *J* = 15.1, 4.5, 3.5 Hz, 1H, H-2), 1.29 (d, *J* = 6.4 Hz, 3H, H-6); ^{13}C NMR (101 MHz, $CDCl_3$ HSQC): δ 135.6, 133.3, 133.1 (C_{q-arom}), 128.4, 128.0, 127.9, 127.8, 126.9, 126.3, 126.1 (CH_{arom}), 97.4 (C-1), 73.0 (C-3), 72.3 (C-4), 70.7(CH_2 Nap), 64.6 (C-5), 55.4 (CH_3 OMe), 31.6 (C-2), 18.0 (C-6); Diagnostic signals of the minor stereoisomer (β-anomer): ^{1}H NMR (400 MHz, $CDCl_3$, HH-COSY, HSQC): δ 4.89 (d, *J* = 11.6 Hz, 1H, C*H*H Nap), 4.63 (d, *J* = 11.6 Hz, 1H, CH*H* Nap), 3.72 (dq, *J* = 9.4, 6.3 Hz, 1H, H-5), 3.50 (s, 3H, CH_3 OMe), 1.64 (ddd, *J* = 14.1, 9.5, 2.7 Hz, 1H, H-2), 1.33 (d, *J* = 6.3 Hz, 3H, H-6); ^{13}C NMR (101 MHz, $CDCl_3$, HSQC): δ 99.0 (C-1), 75.5 (C-3), 72.7 (C-4), 71.7 (CH_2 Nap), 71.0 (C-5), 56.6 (CH_3 OMe), 34.3 (C-2), 18.3 (C-6); HRMS: $[M+Na]^+$ calcd for $C_{18}H_{22}O_4Na$ 325.1416, found 325.1418.

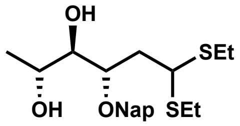

2,6-Dideoxy-1,1-diethyl-thioacetal-3-*O*-(2-methylnaphtalene)-D-altrose (13). Compound **12** (52.9 g, 175 mmol) was dissolved in 25% v:v aqueous acetic acid (3.5 L, 0.05 M) and refluxed at 100 °C for 1 h after which the solution was cooled to 0 °C. Subsequently, solid $NaHCO_3$ (583.8 g, 6.95 mol) was added to quench 50% of the acetic acid. The solution was then extracted with EtOAc (3x) and the combined organic layers were dried over $MgSO_4$, filtered, and concentrated *in vacuo* to yield the crude product as an orange oil. TLC: R_f 0.3 (pentane:EtOAc, 1:1, v:v). The crude product was suspended in ethanethiol (69.4 mL, 962.4 mmol, 5.5 eq.) and cooled on ice, HCl (29.7 mL, 962.4 mmol, 5.5 eq., 37% aqueous solution) was added while stirring vigorously. The solution was stirred for 3 h at 0 °C upon which the reaction was neutralized with sat. aq. $NaHCO_3$ and diluted with EtOAc and H_2O. The aqueous layer was extracted 3x with EtOAc and the combined organic layers were washed with H_2O, sat. aq. $NaHCO_3$ and brine, respectively. Subsequently, the organic layer was dried over $MgSO_4$, filtered, and concentrated *in vacuo* to yield the crude product. Flash column chromatography (90:10 → 50:50; pentane:EtOAc) yielded the title compound (46.1 g, 116.8 mmol, 67%) as a yellow oil. TLC: R_f 0.6 (pentane:EtOAc, 1:1, v:v); $[\alpha]_D^{20}$ –13.3° (c 1.0, $CHCl_3$); IR (neat, cm^{-1}): 750, 815, 1064, 1265, 1373, 1450, 2926, 2968, 3459; ^{1}H NMR (400 MHz, $CDCl_3$, HH-COSY, HSQC): δ 7.87 – 7.77 (m, 4H, CH_{arom}), 7.53 – 7.43 (m, 3H,

CH_{arom}), 4.78 (d, *J* = 11.5 Hz, 1H, C*H*H Nap), 4.74 (d, *J* = 11.5 Hz, 1H, CH*H* Nap), 4.08 – 4.02 (m, 2H, H-1, H-3), 3.87 (p, *J* = 6.2 Hz, 1H, H-5), 3.75 (dd, *J* = 6.3, 4.3 Hz, 1H, H-4), 2.96 (bs, 1H, 4-OH), 2.75 – 2.50 (m, 5H, CH_2CH_3, CH_2CH_3, 5-OH), 2.29 – 2.07 (m, 2H, H-2), 1.29 – 1.21 (m, 6H, H-6, CH_2CH_3), 1.19 (t, *J* = 7.5 Hz, 3H, CH_2CH_3); ^{13}C NMR (101 MHz, $CDCl_3$, HSQC): δ 135.4, 133.2, 133.0 ($C_{q\text{-}arom}$), 128.3, 127.9, 127.7, 126.8, 126.2, 126.0, 125.9 (CH_{arom}), 77.8 (C-1/C-3), 75.1 (C-4), 72.1 (CH_2 Nap), 68.1 (C-5), 47.8 (C-3/C-1), 36.5 (C-2), 24.4 (CH_2CH_3), 23.6 (CH_2CH_3), 19.3 (C-6), 14.5 (CH_2CH_3), 14.3 (CH_2CH_3); HRMS: $[M+Na]^+$ calcd for $C_{21}H_{30}O_3S_2Na$ 417.1534, found 417.1533.

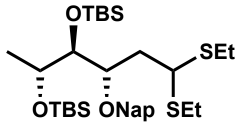

2,6-Dideoxy-1,1-diethyl-thioacetal-3-*O*-(2-methylnaphthalene)-4,5-*O*-di-*tert*-butyldimethylsilyl-D-altrose (14). Pyridine (140 mL, 75 mmol, 15.0 eq.) was added to a solution of compound **13** (46 g, 116.5 mmol) in DCM (1.2 L, 0.1 M), after which the solution was cooled on ice, and TBSOTf (80 mL, 350 mmol, 3.0 eq.) was added dropwise. After stirring for 10 min on ice the reaction was refluxed at 40 °C for 6 h. The reaction mixture was then concentrated to 1/4th of its original volume and quenched with sat. aq. $NaHCO_3$ followed by further dilution with Et_2O and H_2O. The aqueous layer was extracted with Et_2O (3x) followed by washing the combined organic layers with H_2O, sat. aq. $NaHCO_3$ and brine, respectively. Subsequently, the organic layer was dried over $MgSO_4$, filtered, and concentrated *in vacuo* to yield the crude product. Flash column chromatography (98:2 → 95:5; pentane: Et_2O) yielded the title compound (45 g, 72.2 mmol, 62%) as a colorless oil. TLC: R_f 0.5 (pentane:toluene, 9:1, v:v); $[\alpha]_D^{20}$ –31.1° (c 1.0, $CHCl_3$); IR (neat, cm^{-1}): 756, 835, 1105, 1253, 2927; ^1H NMR (400 MHz, $CDCl_3$, HH-COSY, HSQC): δ 7.87 – 7.76 (m, 4H, CH_{arom}), 7.51 – 7.43 (m, 3H, CH_{arom}), 4.87 (dd, *J* = 11.8, 0.8 Hz, 1H, CH*H* Nap), 4.66 (dd, *J* = 11.7, 0.8 Hz, 1H, C*H*H Nap), 4.09 – 3.97 (m, 2H, H-1, H-3), 3.79 (p, *J* = 6.1 Hz, 1H, H-5), 3.70 (dd, *J* = 6.0, 2.4 Hz, 1H, H-4), 2.70 – 2.44 (m, 4H, CH_2CH_3, CH_2CH_3), 2.26 (ddd, *J* = 14.9, 10.4, 3.5 Hz, 1H, H-2), 1.90 (ddd, *J* = 14.9, 11.3, 2.5 Hz, 1H, H-2), 1.25 (t, *J* = 7.4 Hz, 3H, CH_2CH_3), 1.21 (d, *J* = 6.1 Hz, 3H, H-6), 1.13 (t, *J* = 7.4 Hz, 3H, CH_2CH_3), 1.02 – 0.84 (m, 18H, $C(CH_3)_3$, $C(CH_3)_3$), 0.18 – 0.06 (m, 12H, $SiCH_3$, $SiCH_3$, $SiCH_3$, $SiCH_3$); ^{13}C NMR (101 MHz, $CDCl_3$, HSQC): δ 136.5, 133.4, 133.0 ($C_{q\text{-}arom}$), 128.0, 128.0, 127.8, 126.3, 126.1, 126.0, 125.8 (CH_{arom}), 78.8 (C-4), 78.6 (C-1/C-3), 72.4 (CH_2 Nap), 69.8 (C-5), 48.1 (C-3/C-1), 37.4 (C-2), 26.3 ($C(CH_3)_3$), 25.8 ($C(CH_3)_3$), 24.5 (CH_2CH_3), 24.2 (CH_2CH_3), 20.8 (C-6), 18.5 ($C(CH_3)_3$), 18.2 ($C(CH_3)_3$), 14.8 (CH_2CH_3), 14.4 (CH_2CH_3), -2.8 ($SiCH_3$), -3.7 ($SiCH_3$), -3.9 ($SiCH_3$), -4.3 ($SiCH_3$); HRMS: $[M+Na]^+$ calcd for $C_{33}H_{58}O_3S_2Si_2Na$ 645.3264, found 645.3258.

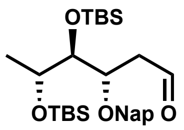

2,6-Dideoxy-3-*O*-(2-methylnaphthalene)-4,5-*O*-di-*tert*-butyldimethylsilyl-D-altrose (15). Compound **14** (45 g, 72.2 mmol) was dissolved in acetone (480 mL, 0.15 M) and H_2O (11 mL, 0.6 mol, 8.5 eq.) and cooled on ice. $NaHCO_3$ (27.3 g, 325 mmol, 4.5 eq.) and iodine (40.3 g, 160 mmol, 2.2 eq.) were added and the mixture was allowed to reach room temperature. After stirring for 6 h the mixture was quenched with sat. aq. $Na_2S_2O_3$ and further diluted with Et_2O and H_2O. The aqueous layer was extracted with Et_2O (3x) followed by washing the combined organic layers with H_2O, sat. aq. $NaHCO_3$ and brine, respectively. Subsequently, the organic layer was dried over $MgSO_4$, filtered, and concentrated *in vacuo* to yield the crude product as a colorless oil. Flash column chromatography (99:1 → 90:10; pentane:Et_2O) yielded the title compound (30.1 g, 58.2 mmol, 81%) as a colorless oil. TLC: R_f 0.2 (pentane,Et_2O, 40:1, v:v); $[\alpha]_D^{20}$ –23.2° (c 1.0, $CHCl_3$); IR (neat, cm^{-1}): 775, 835, 1101, 1253, 1471, 1728, 2927; ^1H NMR (400 MHz, $CDCl_3$, HH-COSY, HSQC): δ 9.79 (dd, *J* = 2.8, 1.6 Hz, 1H, H-1), 7.86 – 7.72 (m, 4H, CH_{arom}), 7.54 – 7.40 (m, 3H, CH_{arom}), 4.76 (d, *J* = 11.7 Hz, 1H, CH*H* Nap), 4.66 (d, *J* = 11.8 Hz, 1H, C*H*H Nap), 4.24 (ddd, *J* = 8.4, 3.2, 2.5 Hz, 1H, H-3), 3.76 (dd, *J* = 6.2, 2.5 Hz, 1H, H-4), 3.69 (p, *J* = 6.1 Hz, 1H, H-5), 2.75 (ddd, *J* = 16.9, 8.4, 2.8 Hz, 1H, H-2), 2.60 (ddd, *J* = 17.0, 3.3, 1.6 Hz, 1H, H-2), 1.17 (d, *J* = 6.0 Hz, 3H, H-6), 0.87 (d, *J* = 22.8 Hz, 18H, $C(CH_3)_3$, $C(CH_3)_3$), 0.10 (d, *J* = 4.1 Hz, 12H, $SiCH_3$, $SiCH_3$, $SiCH_3$,

SiCH3); ^{13}C NMR (101 MHz, $CDCl_3$, HSQC): δ 202.0 (C-1), 135.6, 133.3, 133.1 ($C_{q\text{-arom}}$), 128.2, 128.0, 127.8, 126.7, 126.1, 126.0, 125.9 (CH_{arom}), 78.1 (C-4), 75.2 (C-3), 71.8 (CH_2 Nap), 70.0 (C-5), 44.3 (C-2), 26.2 (C(CH_3)$_3$), 26.0 (C(CH_3)$_3$), 20.7 (C-6), 18.4 (*C*(CH_3)$_3$), 18.0 (*C*(CH_3)$_3$), -3.9 ($SiCH_3$), -4.0 ($SiCH_3$), -4.3 ($SiCH_3$), -4.7 ($SiCH_3$); HRMS: [M+Na]$^+$ calcd for $C_{29}H_{48}O_4Si_2Na$ 539.2989, found 539.2986.

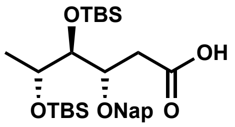

2,6-Dideoxy-3-*O*-(2-methylnaphthalene)-4,5-*O*-di-*tert*-butyldimethylsilyl-D-altronic acid (16). To a stirred solution of **15** (30 g, 58 mmol) in *t*-BuOH (0.5 L, 0.12 M) and aq. NaH_2PO_4 (266 mL, 5% w:w) an aqueous $KMnO_4$ solution (157 mL, 157 mmol, 2.7 eq., 1 M) was added. The reaction mixture was stirred for 3 h after which an excess of solid $Na_2S_2O_3$ was added. After the mixture had turned brown the solution was filtered over Celite© Hyflo Supercel (Merck) and was rinsed with Et_2O and H_2O. The aqueous layer was extracted with Et_2O (3x) followed by washing the combined organic layers with H_2O, sat. aq. $NaHCO_3$ and brine, respectively. Subsequently, the organic layer was dried over $MgSO_4$, filtered, and concentrated *in vacuo* to yield the crude product as a colorless oil. Flash column chromatography (95:5 → 80:20; pentane:Et_2O) yielded the title compound (23.4 g, 43.9 mmol, 75%) as a colorless oil. TLC: R_f 0.3 (pentane:EtOAc, 9:1, v:v); $[\alpha]_D^{20}$ –20.1° (c 1.0, $CHCl_3$); IR (neat, cm^{-1}): 775, 829, 948, 1107, 1253, 1710, 2927; ^1H NMR (400 MHz, $CDCl_3$, HH-COSY, HSQC): δ 7.89 – 7.73 (m, 4H, CH_{arom}), 7.50 – 7.39 (m, 3H, CH_{arom}), 4.76 (d, *J* = 11.6 Hz, 1H, CH*H* Nap), 4.70 (d, *J* = 11.5 Hz, 1H, C*H*H Nap), 4.20 (td, *J* = 6.1, 2.0 Hz, 1H, H-3), 3.75 – 3.67 (m, 2H, H-4, H-5), 2.68 (d, *J* = 6.1 Hz, 2H, H-2), 1.17 (d, *J* = 5.8 Hz, 3H, H-6), 0.91 – 0.85 (m, 18H, C(CH_3)$_3$, C(CH_3)$_3$), 0.11 – 0.04 (m, 12H, $SiCH_3$, $SiCH_3$, $SiCH_3$, $SiCH_3$); ^{13}C NMR (101 MHz, $CDCl_3$, HSQC): δ 178.4 (C-1), 135.8, 133.3, 133.0 ($C_{q\text{-arom}}$), 128.1, 128.0, 127.8, 126.6, 126.1, 126.1, 125.8 (CH_{arom}), 78.1 (C-4/C-5), 77.1 (C-3), 72.4 (CH_2 Nap), 70.0 (C-5/C-4), 35.8 (C-2), 26.2 (C(CH_3)$_3$), 26.0 (C(CH_3)$_3$), 20.6 (C-6), 18.5 (*C*(CH_3)$_3$), 18.1 (*C*(CH_3)$_3$), -3.9 ($SiCH_3$),-4.0 ($SiCH_3$), -4.4 ($SiCH_3$), -4.7 ($SiCH_3$); HRMS: [M+Na]$^+$ calcd for $C_{29}H_{48}O_5Si_2Na$ 555.2938, found 345.1316.

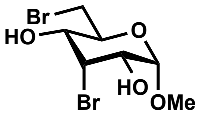

Methyl 3,6-dibromo-3,6-dideoxy-α-D-allopyranoside (17). A mixture of methyl α-D-glucopyranoside (32.5 g, 167 mmol), 2,4,5-tribromoimidazole (102 g, 335 mmol, 2.0 eq.) and triphenylphosphine (87.8 g, 335 mmol, 2.0 eq.) in toluene (2.7 L, 63 mM) was refluxed at 125 °C for 6 h. The mixture was allowed to cool to room temperature and concentrated *in vacuo*, yielding a dark brown syrup. Flash column chromatography (90:10 → 60:40; pentane:EtOAc) yielded the product as a mixture of methyl 3,6-dibromo-3,6-dideoxy-α-D-allopyranoside and triphenylphosphine oxide. The mixture could be separated by flash column chromatography (60:40 → 40:60; pentane:Et_2O) to yield the title compound (32 g, 100 mmol, 60%) as a white solid. TLC: R_f 0.5 (pentane:EtOAc, 4:6, v:v); $[\alpha]_D^{20}$ 63.3° (c 1.0, $CHCl_3$); IR (neat, cm^{-1}): 1161, 1209, 1263, 2909, 3451; ^1H NMR (400 MHz, $CDCl_3$, HH-COSY, HSQC): δ 4.82 (t, *J* = 3.9 Hz, 1H, H-3), 4.79 (d, *J* = 4.3 Hz, 1H, H-1), 3.95 (ddd, *J* = 8.9, 6.2, 2.4 Hz, 1H, H-5), 3.90 (dt, *J* = 12.0, 4.3 Hz, 1H, H-2), 3.77 (dd, *J* = 11.1, 2.4 Hz, 1H, H-6), 3.62 (dd, *J* = 11.1, 6.2 Hz, 1H, H-6), 3.58 (ddd, *J* = 10.8, 9.3, 3.4 Hz, 1H, H-4), 3.48 (s, 3H, CH_3 OMe), 2.74 (d, *J* = 11.9 Hz, 1H, 2-OH), 2.27 (d, *J* = 10.9 Hz, 1H, 4-OH); ^{13}C NMR (101 MHz, $CDCl_3$, HSQC): δ 99.0 (C-1), 68.2 (C-4), 67.9 (C-5), 67.1 (C-2), 62.9 (C-3), 56.2 (CH_3 OMe), 33.0 (C-6); HRMS: [M+Na]$^+$ calcd for $C_7H_{12}Br_2O_4Na$ 342.8980, found 342.8985.

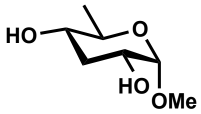

Methyl 3,6-dideoxy-α-D-allopyranoside (18). Compound **17** (16.6 g, 52 mmol) was dissolved in dry toluene (580 mL, 0.09 M) under N_2 atmosphere. Bu_3SnH (37.8 mL, 140 mmol, 2.7 eq.) and AIBN (0.85 g, 5.2 mmol, 0.1 eq.) were added respectively. The solution was refluxed at 120 °C for 17 h, and upon

full conversion, the solution was concentrated *in vacuo*. Flash column chromatography (50:50 → 10:90; pentane:EtOAc) yielded the title compound (8.4 g, 51.5 mmol, 99%) as a colorless oil. TLC: R_f 0.25 (pentane:EtOAc, 4:6, v:v); $[\alpha]_D^{20}$ 39.3° (c 1.0, $CHCl_3$); IR (neat, cm^{-1}): 1150, 2934, 3385; ^1H NMR (400 MHz, $CDCl_3$, HH-COSY, HSQC): δ 4.60 (d, *J* = 3.6 Hz, 1H, H-1), 3.71 (dt, *J* = 11.8, 4.6 Hz, 1H, H-2), 3.51 (dq, *J* = 9.2, 6.3 Hz, 1H, H-5), 3.44 (s, 3H, CH_3 OMe), 3.28 (ddd, *J* = 11.1, 9.1, 4.5 Hz, 1H, H-4), 2.19 (dt, *J* = 11.6, 4.7 Hz, 1H, H-3), 1.65 (q, *J* = 11.4 Hz, 1H H-3), 1.26 (d, *J* = 6.2 Hz, 3H, H-6); ^{13}C NMR (101 MHz, $CDCl_3$, HSQC): δ 98.4 (C-1), 70.8 (C-4), 68.7 (C-5), 67.7 (C-2), 55.2 (CH_3 OMe), 37.0 (C-3), 17.5 (C-6); HRMS: $[M+Na]^+$ calcd for $C_7H_{14}O_4Na$ 185.0790, found 185.0790.

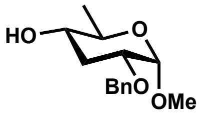

Methyl 2-*O*-benzyl-3,6-dideoxy-α-D-allopyranoside (19). Compound **18** (13.7 g, 84.6 mmol) and tributyltin oxide (86.2 mL, 169 mmol, 2.0 eq.) were dissolved in dry toluene (560 mL, 0.15 M, the solution was refluxed for 20 h under positive N_2 flow in a flask equipped with a Dean-Stark apparatus. The reaction was concentrated in *vacuo* upon which benzyl bromide (60.3 mL, 508 mmol, 6.0 eq.) was added to the residue. The mixture was stirred at 95 °C for 16 h, where after the reaction was cooled to room temperature and purified by flash column chromatography on silica gel. Flash column chromatography (100:0 → 50:50; pentane:EtOAc) yielded the title compound (6.56 g, 26 mmol, 31%) as a colorless oil. TLC: R_f 0.3 (pentane:acetone, 8:2, v:v); $[\alpha]_D^{20}$ 44.5° (c 1.0, $CHCl_3$); IR (neat, cm^{-1}): 1050, 1090, 1454, 2935; ^1H NMR (400 MHz, $CDCl_3$, HH-COSY, HSQC): δ 7.37 – 7.27 (m, 5H, CH_{arom}), 4.63 (d, *J* = 12.4 Hz, 1H, C*H*H Bn), 4.61 (d, *J* = 3.1 Hz, 1H, H-1), 4.57 (d, *J* = 12.4 Hz, 1H, CH*H* Bn), 3.57 – 3.48 (m, 2H, H-2, H-5), 3.42 (s, 3H, CH_3 OMe), 3.23 (ddd, *J* = 11.2, 9.3, 4.6 Hz, 1H, H-4), 2.23 – 2.13 (m, 1H, H-3), 1.81 (q, *J* = 11.6 Hz, 1H, H-3), 1.23 (d, *J* = 6.2 Hz, 3H, H-6); ^{13}C NMR (101 MHz, $CDCl_3$, HSQC): δ 138.2 ($C_{q\text{-}arom}$), 128.6, 128.0, 128.0 (CH_{arom}), 97.2 (C-1), 74.0 (C-2), 71.3 (C-4), 71.2 (CH_2 Bn), 68.7 (C-5), 55.0 (CH_3 OMe), 33.7 (C-3), 17.5 (C-6); HRMS: $[M+Na]^+$ calcd for $C_{14}H_{20}O_4Na$ 275.1259, found 275.1254.

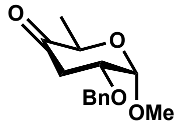

Methyl 2-*O*-benzyl-3,6-dideoxy-α-D-erythropyranosid-4-ulose (6). Compound **19** (6.5 g, 26 mmol) was dissolved in DCM (153 mL, 0.17 M) under N_2 atmosphere. Dess-Martin periodinane (16.5 g, 39 mmol, 1.5 eq.) was added and the mixture was stirred for 2.5 h upon the reaction was quenched with water. The aqueous layer was extracted with DCM (3x) followed by washing the combined organic layers with H_2O, sat. aq. $NaHCO_3$ and brine, respectively. Subsequently, the organic layer was dried over $MgSO_4$, filtered, and concentrated *in vacuo* to yield the crude product as a white oil. Flash column chromatography (95:5 → 90:10; pentane:Et_2O) yielded the title compound (5.8 g, 23.3 mmol, 90%) as a colorless oil. TLC: R_f 0.7 (pentane:acetone, 8:2, v:v); $[\alpha]_D^{20}$ 73.6° (c 1.0, $CHCl_3$); IR (neat, cm^{-1}): 1047, 1077, 1454, 1724, 2938; ^1H NMR (400 MHz, $CDCl_3$, HH-COSY, HSQC): δ 7.39 – 7.28 (m, 5H, CH_{arom}), 4.82 (d, *J* = 3.2 Hz, 1H, H-1), 4.65 (d, *J* = 12.4 Hz, 1H, C*H*H Bn), 4.58 (d, *J* = 12.3 Hz, 1H, CH*H* Bn), 4.13 (q, *J* = 6.7 Hz, 1H, H-5), 3.83 (ddd, *J* = 10.6, 6.4, 3.3 Hz, 1H, H-2), 3.51 (s, 3H, CH_3 OMe), 2.83 – 2.69 (m, 2H, H-3, H-3), 1.26 (d, *J* = 6.7 Hz, 3H, H-6); ^{13}C NMR (101 MHz, $CDCl_3$, HSQC): δ 206.1 (C=O), 137.7 ($C_{q\text{-}arom}$), 128.7, 128.2, 128.0 (CH_{arom}), 97.4 (C-1), 74.4 (C-2), 71.7 (CH_2 Bn), 70.2 (C-5), 56.0 (CH_3 OMe), 41.1 (C-3), 14.6 (C-6); HRMS: $[M+Na]^+$ calcd for $C_{14}H_{18}O_4Na$ 273.1103, found 273.1097.

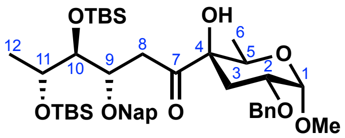

Methyl 2-*O*-benzyl-3,6-dideoxy-4-*C*-([9*S*,10*S*,11*R*]-9-*O*-[2-methylnaphthalene]-10,11-*O*-di-*tert*-butyldimethylsilyl-hexan-7-one)-α-D-galactopyranoside (20). Carboxylic acid **16** (2.66 g, 5.0 mmol) was dissolved in dry THF (50 mL, 0.1 M). This solution was cooled to 0 °C, and while stirring pyridine (604 µL, 7.5 mmol, 1.5 eq.), DMF (77 µL, 1.0 mmol, 0.2 eq.) and oxalyl chloride (557 µL, 6.5 mmol, 1.3 eq.) were added respectively. The solution was stirred for 10 min on ice. The suspension was diluted with pentane and filtered into a flask containing ketone **6** (938.6 mg, 3.75 mmol, 0.75 eq.), resulting in a clear liquid that was concentrated *in vacuo* under N_2 atmosphere to yield the crude acid chloride **5** combined with ketone **6** as a yellow oil. A solution of samarium(II)iodide (175 mL, 17.5 mmol, 3.5 eq. [0.1 M solution in THF, stabilized by samarium chips, Sigma-Aldrich]) was added to a flame dried flask which was under a constant gas flow of nitrogen. The samarium(II)iodide solution was heated to 50 °C followed by the addition of the crude acid chloride **5** and ketone **6** using a cannula. After 10 min the heat source was removed and the solution was quenched with air and diluted with EtOAc, aq. 1.0 M HCl and stirred for 30 min. The aqueous layer was extracted with EtOAc (3x) followed by washing the combined organic layers with sat. aq. NaS_2O_3. Subsequently, the organic layer was dried over $MgSO_4$, filtered, and concentrated *in vacuo* to yield the crude product as a colorless oil. Flash column chromatography (90:10; pentane:Et_2O) afforded the title compound (2.21 g, 2.88 mmol, 82% based on **6**) as a colorless oil. TLC: R_f 0.6 (pentane:acetone, 9:1, v:v); $[\alpha]_D^{20}$ –10.3° (c 1.0, $CHCl_3$); IR (neat, cm^{-1}): 777, 811, 1108, 1255, 1472, 1706, 2856, 2929; ^{1}H NMR (500 MHz, $CDCl_3$, HH-COSY, HSQC): δ 7.84 – 7.18 (m, 12H, CH_{arom}), 4.73 (d, *J* = 11.5 Hz, 1H, C*H*H Bn/Nap), 4.68 (d, *J* = 3.3 Hz, 1H, H-1), 4.56 (d, *J* = 11.5 Hz, 1H, CH*H* Bn/Nap), 4.48 (d, *J* = 12.4 Hz, 1H, C*H*H Bn/Nap), 4.39 – 4.35 (m, 1H, H-9), 4.34 (d, *J* = 12.4 Hz, 1H, CH*H* Bn/Nap), 4.23 (q, *J* = 6.4 Hz, 1H, H-5), 3.89 (s, 1H, 4-OH), 3.83 (ddd, *J* = 11.7, 4.7, 3.3 Hz, 1H, H-2), 3.72 – 3.65 (m, 2H, H-10, H-11), 3.44 (s, 3H, CH_3 OMe), 3.27 (dd, *J* = 16.9, 10.1 Hz, 1H, H-8), 2.43 – 2.35 (m, 2H, H-3, H-8), 1.63 (dd, *J* = 12.4, 4.7 Hz, 1H, H-3), 1.18 (d, *J* = 5.8 Hz, 3H, H-12), 0.96 – 0.88 (m, 21H, H-6, $C(CH_3)_3$, $C(CH_3)_3$), 0.15 – 0.04 (m, 12H, $SiCH_3$, $SiCH_3$, $SiCH_3$, $SiCH_3$); ^{13}C NMR (126 MHz, $CDCl_3$, HSQC): δ 210.2 (C-7), 138.3, 135.8, 133.4, 133.0 ($C_{q\text{-}arom}$), 128.5, 128.1, 128.0, 127.9, 127.8, 126.3, 126.1, 125.9 (CH_{arom}), 98.1 (C-1), 81.1 (C-4), 78.1 (C-10/C-11), 77.2 (C-9), 72.8 (CH_2 Bn/Nap), 71.6 (C-2), 71.0 (CH_2 Bn/Nap), 70.1 (C-11/C-10), 65.2 (C-5), 55.4 (CH_3 OMe), 38.2 (C-8), 32.9 (C-3), 26.2, 26.0 ($C(CH_3)_3$), 20.8 (C-12), 18.5, 18.2 ($C(CH_3)_3$), 14.4 (C-6), -3.8, -4.0, -4.3, -4.6 ($SiCH_3$); HRMS: $[M+Na]^+$ calcd for $C_{43}H_{66}O_8Si_2Na$ 789.4194, found 789.4188.

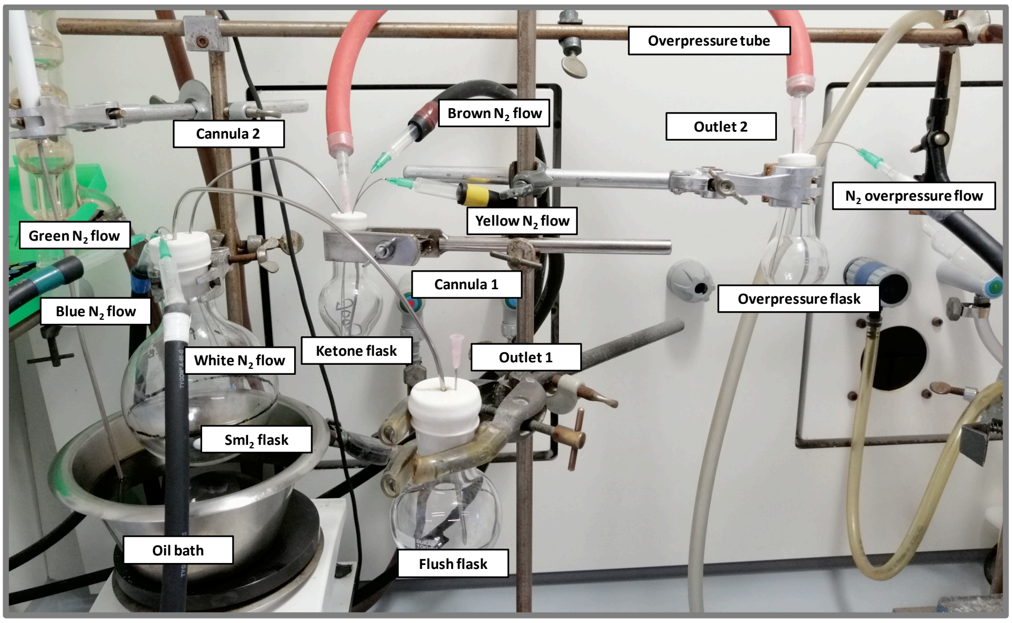

Figure S10. General setup of the SmI_2-promoted C-C bond coupling.

Extra experimental details

Due to the direct oxidation of Sm(II) to the unreactive and more stable Sm(III) in the presence of oxygen, the C-C couplings have to be executed with great care and under completely inert conditions. The experimental setup can be found in Figure S8. The general procedure employed in the C-C couplings was as follows: Both the ketone flask and the SmI_2 flask seen in Figure S8 were flame dried, subsequently, the entire setup was flushed with N_2 (30 mbar overpressure) for 18 h. Then, pyranulose **6** was co-evaporated with toluene under N_2 atmosphere in a flame dried flask. Using an N_2 flushed syringe, the ketone was transferred to the ketone flask in THF. Subsequently, the acid chloride (1.33 eq.) was added to the ketone flask and N_2 was bubbled through the solution of ketone and acid chloride for 30 min through cannula 2 by closing the yellow, brown and overpressure N_2 flow and removing outlet 1. Simultaneously, SmI_2 (3.0 eq.) was added to the SmI_2 flask by transferring the flush flask side of cannula 1 and the green and white N_2 flow to a 0.1 M solution SmI_2 in THF, and closing the blue N_2 flow (Figure S9). Once the necessary amount of SmI_2 was transferred to the SmI_2 flask, cannula 1 was transferred from the 0.1 M solution SmI_2 in THF to the flush flask and the green and white N_2 flow tubes were transferred back to the SmI_2 flask. The SmI_2 flask was then heated to 50 °C using the oil bath. Once the SmI_2 flask reached a temperature of 50 °C, the solution of ketone and acid chloride was added to the SmI_2 flask through cannula 2 over a time span of approximately 10 seconds by removing outlet 2 and installing outlet 1, opening the yellow, brown and overpressure N_2 flows, and closing the green and white N_2 flows. After 15 min, the positive nitrogen flow was removed and a 1 M aq. HCl solution and EtOAc were added, followed by standard workup procedures.

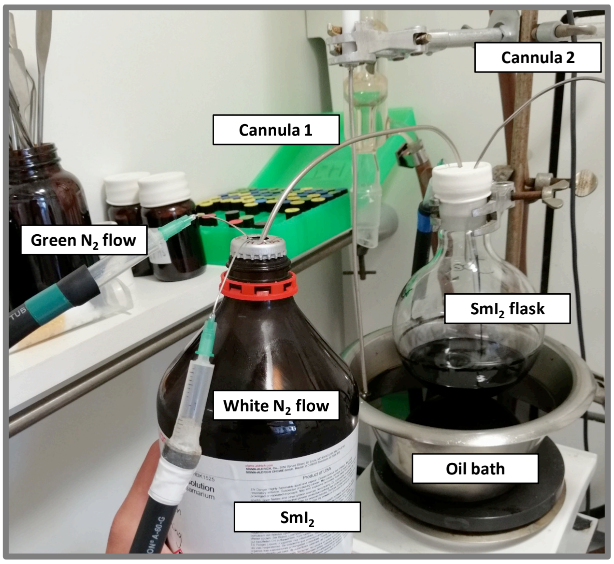

Figure S11. General setup for the addition of SmI_2 to the SmI_2 flask needed for the SmI_2-promoted C-C bond coupling.

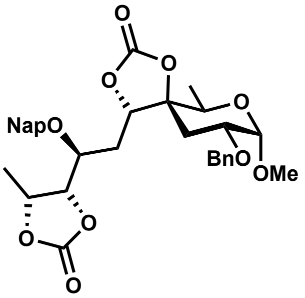

Methyl 2-*O*-benzyl-9-*O*-(2-methylnaphthalene)-10,11-di-*O*-*tert*-butyldimethylsilyl-α-D-caryophylloside (21). A $Zn(BH_4)_2$ solution was prepared by dissolving anhydrous $ZnCl_2$ (12.1 g, 88.5 mmol, 4.2 eq.) in dry THF (177 mL, 0.5 M), at 0 °C $NaBH_4$ (8.4 g, 221.3 mmol, 10.5 eq.) was added and the solution

was stirred for 1 h. **20** (16.2 g, 21.1 mmol, 1.0 eq.) was dissolved in dry THF (422 mL, 0.05 M) after which it was cooled on ice, the $Zn(BH_4)_2$ solution was added. The solution was allowed to warm to room temperature and stirred for 16 h. The reaction was quenched with sat. aq. NH_4Cl and diluted with EtOAc and brine, the aqueous layer was extracted with EtOAc (3x) followed by washing the combined organic layers with H_2O, sat. aq. $NaHCO_3$ and brine, respectively. Subsequently, the organic layer was dried over $MgSO_4$, filtered, and concentrated *in vacuo* to yield the crude products as an inseparable mixture. The mixture (14.9 g, 19.3 mmol, 1.0 eq.) was dissolved in methanol (568 mL, 0.034 M), a 6 M HCl aq. solution (32 mL, 10 eq.) was added and the mixture was stirred for 18 h upon which the reaction was quenched by neutralizing the acid with NaOMe. The reaction mixture was concentrated *in vacuo* to yield the crude products as an inseparable mixture. The mixture (8.4 g, 15.5 mmol) was dissolved in DCM (310 mL, 0.05 M) and CDI (7.6 g, 46.6 mmol, 3 eq.) was added. The resulting mixture was refluxed for 24 h. Upon full conversion, the reaction mixture was concentrated in vacuo. Flash column chromatography (90:10 → 40:60; pentane:EtOAc) afforded the title compound (7.2 g, 12.2 mmol, 58% over 3 steps) as a white foam. TLC: R_f 0.7 (pentane:EtOAc, 4:6, v:v); $[\alpha]_D^{20}$ 149.0° (c 1.0, $CHCl_3$); IR (neat, cm^{-1}): 1056, 1121, 1800; ^1H NMR (500 MHz, $CDCl_3$, HH-COSY, HSQC): δ 7.94 – 7.28 (m, 12H, CH_{arom}), 4.92 (p, *J* = 6.7 Hz, 1H, H-11), 4.79 (s, 2H, CH_2 Bn/Nap), 4.67 – 4.62 (m, 2H, H-1, H-10), 4.57 (d, *J* = 12.1 Hz, 1H, C*H*H Bn/Nap), 4.49 (d, *J* = 12.1 Hz, 1H, CH*H* Bn/Nap), 4.33 (dd, *J* = 11.4, 1.8 Hz, 1H, H-7), 4.03 (ddd, *J* = 9.5, 6.7, 3.1 Hz, 1H, H-9), 3.86 (q, *J* = 6.3 Hz, 1H, H-5), 3.75 (ddd, *J* = 11.7, 4.9, 3.5 Hz, 1H, H-2), 3.42 (s, 3H, CH_3 OMe), 2.12 (ddd, *J* = 14.7, 11.5, 3.1 Hz, 1H, H-8), 2.03 (dd, *J* = 13.4, 11.8 Hz, 1H, H-3), 1.95 (ddd, *J* = 14.9, 8.6, 1.9 Hz, 1H, H-8), 1.83 (dd, *J* = 13.5, 4.9 Hz, 1H, H-3), 1.46 (d, *J* = 6.7 Hz, 3H, H-12), 1.24 (d, *J* = 6.3 Hz, 3H, H-6); ^{13}C NMR (126 MHz, $CDCl_3$, HSQC): δ 153.6, 153.5 (O(C=O)O), 137.8, 134.2, 133.3, 133.3 ($C_{q\text{-}arom}$), 128.9, 128.7, 128.2, 128.1, 128.0, 127.9, 126.8, 126.8, 126.6, 125.4 (CH_{arom}), 96.9 (C-1), 84.8 (C-4), 81.0 (C-7), 79.0 (C-10), 75.8 (C-11), 73.9 (C-9), 73.7, 71.7 (CH_2 Bn/Nap), 71.4 (C-2), 64.7 (C-5), 55.9 (CH_3 OMe), 33.6 (C-3), 29.8 (C-8), 15.3 (C-12), 15.0 (C-6); HRMS: $[M+Na]^+$ calcd for $C_{33}H_{36}O_{10}Na$ 615.2206, found 615.2215.

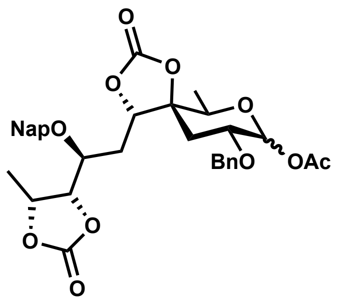

Acetyl 2-*O*-benzyl-4,7,10,11-di-*O*-carbonate-9-*O*-(2-methylnaphthalene)-D-caryophylloside (22). Compound **21** (6.4 g, 10.8 mmol) was dissolved in Ac_2O (216 mL, 0.05 M) and cooled on ice. H_2SO_4 (1.15 mL, 21.6 mmol, 2.0 eq.) was dissolved in Ac_2O (10 mL) and dropwise added to the solution of compound **21**. After stirring the solution for exactly 80 sec, the reaction mixture was poured into a mixture of sat. aq. $NaHCO_3$ and ice, and stirred for another 15 min. The aqueous layer was extracted twice with EtOAc followed by washing the combined organic layers with H_2O, sat. aq. $NaHCO_3$ and brine, respectively. Subsequently, the organic layer was dried over $MgSO_4$, filtered, and concentrated *in vacuo* to yield the crude product. Flash column chromatography (70:30 → 50:50; pentane:EtOAc) yielded the title compound (6.3 g, 10.2 mmol, 94%, α:β; 63:37) as a white foam. TLC: R_f 0.2 (pentane:EtOAc, 7:3, v:v); IR (neat, cm^{-1}): 752, 1054, 1086, 1200, 1229, 1751, 1797; Data of the major stereoisomer (α-anomer): ^1H NMR (400 MHz, $CDCl_3$, HH-COSY, HSQC): δ 8.22 – 6.97 (m, 12H, CH_{arom}), 6.34 (d, *J* = 3.0 Hz, 1H, H-1), 4.95 (p, *J* = 6.9 Hz, 1H, H-11), 4.88 – 4.41 (m, 6H, H-7, H-10, CH_2 Bn/Nap, CH_2 Bn/Nap), 4.11 – 3.85 (m, 3H, H-2, H-5, H-9), 2.20 (s, 3H, CH_3 OAc), 2.16 – 1.96 (m, 4H, H-3, H-3, H-8, H-8), 1.48 (d, *J* = 6.6 Hz, 3H, H-12), 1.29 (d, *J* = 6.2 Hz, 3H, H-6); ^{13}C NMR (101 MHz, $CDCl_3$, HSQC): δ 169.5 (C=O Ac), 153.6, 153.2(O(C=O)O), 137.3, 134.2, 134.0, 133.2 ($C_{q\text{-}arom}$), 128.8, 128.7, 128.7, 128.6, 127.9, 127.9, 127.7, 127.0, 126.7, 126.7, 125.6, 125.6 (CH_{arom}), 88.4 (C-1), 84.2 (C-4), 80.8 (C-7), 78.5 (C-10), 75.8 (C-11), 73.5 (C-9), 73.4, 71.8 (CH_2 Bn/Nap), 70.2 (C-2), 67.3 (C-5), 33.8 (C-3), 29.2 (C-8), 21.2 (CH_3 Ac), 15.1 (C-12), 15.0 (C-6); Diagnostic signals of the minor stereoisomer (β-isomer):

^{1}H NMR (400 MHz, $CDCl_3$, HH-COSY, HSQC): δ 5.65 (d, J = 6.5 Hz, 1H, H-1), 2.13 (s, 3H, CH_3 Ac), 1.35 (d, J = 6.4 Hz, 3H, H-6); ^{13}C NMR (101 MHz, $CDCl_3$, HSQC): δ 169.4 (C=O Ac), 153.7, 153.0 (O(C=O)O), 93.8 (C-1), 83.2 (C-4), 36.5 (C-3), 29.5 (C-8), 21.2 (CH_3 OAc), 15.8 (C-12), 15.2 (C-6); HRMS: $[M+Na]^+$ calcd for $C_{34}H_{36}O_{11}Na$ 643.2155, found 643.2164.

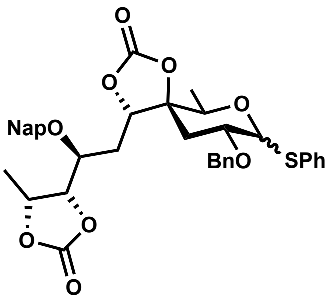

Phenyl 2-*O*-benzyl-4,7,10,11-di-*O*-carbonate-9-*O*-(2-methylnaphthalene)-1-thio-D-caryophylloside (4). Compound **22** (6.3 g, 10.15 mmol) was dissolved in DCM (101.5 mL, 0.1 M) and thiophenol (1.14 mL, 11.16 mmol, 1.1 eq.). Subsequently, the reaction mixture was cooled to –80 °C followed by the dropwise addition of $BF_3{\cdot}OEt_2$ (1.5 mL, 12.18 mmol, 1.2 eq.) and allowed to warm to room temperature overnight. Upon full conversion, sat. aq. $NaHCO_3$ and EtOAc were added. The aqueous layer was extracted twice with EtOAc followed by washing the combined organic layers with H_2O, sat. aq. $NaHCO_3$ and brine, respectively. Subsequently, the organic layer was dried over $MgSO_4$, filtered, and concentrated *in vacuo* to yield the crude product. Flash column chromatography (80:20 → 70:30; pentane:EtOAc) yielded the title compound (4.2 g, 6.2 mmol, 61%, α:β; 49:51) as a white foam. TLC: R_f 0.7 (pentane:EtOAc, 6:4, v:v); IR (neat, cm^{-1}): 746, 1014, 1027, 1801; Data of the major stereoisomer (β-anomer): ^{1}H NMR (500 MHz, $CDCl_3$, HH-COSY, HSQC): δ 8.17 – 6.95 (m, 12H, CH_{arom}), 4.94 – 4.85 (m, 1H, H-11), 4.67 – 4.60 (m, 3H, C*H*H Bn/Nap, CH*H* Bn/Nap, H-10), 4.58 (d, J = 9.0 Hz, 1H, H-1), 4.52 – 4.44 (m, 2H, C*H*H Bn/Nap, CH*H* Bn/Nap), 4.11 – 3.97 (m, 2H, H-7, H-9), 3.70 – 3.55 (m, 2H, H-2, H-5), 2.21 – 2.10 (m, 1H, H-3), 2.10 – 2.02 (m, 1H, H-8), 1.98 – 1.88 (m, 2H, H-3, H-8), 1.44 (d, J = 6.7 Hz, 3H, H-12), 1.33 (d, J = 6.1 Hz, 3H, H-6); ^{13}C NMR (126 MHz, $CDCl_3$, HSQC): δ 153.6, 153.2 (O(C=O)O), 137.6, 137.2, 134.1, 133.3 ($C_{q\text{-}arom}$), 132.5, 131.5, 129.1, 128.6, 128.1, 127.9, 127.5, 127.0, 126.8, 126.7, 125.6, 125.5 (CH_{arom}), 88.5 (C-1), 84.2 (C-4), 78.7 (C-10), 75.7 (C-11), 74.9 (C-2/C-5), 74.0 (C-7/C-9), 73.9 (C-7/C-9), 72.9 (CH_2 Bn/Nap), 72.5 (C-2/C-5), 71.5 (CH_2 Bn/Nap), 39.5 (C-3), 29.4 (C-8), 15.7 (C-6), 15.2 (C-12); Diagnostic signals of the minor stereoisomer (α-isomer): ^{1}H NMR (500 MHz, $CDCl_3$, HH-COSY, HSQC): δ 5.62 (d, J = 4.8 Hz, 1H, H-1), 1.71 (dd, J = 14.1, 10.3 Hz, 1H, H-3), 1.46 (d, J = 6.6 Hz, 3H, H-12), 1.24 (d, J = 6.3 Hz, 3H, H-6); ^{13}C NMR (126 MHz, $CDCl_3$, HSQC): δ 153.6, 153.3 (O(C=O)O), 86.5 (C-1), 84.3 (C-4), 73.8, 73.7 (CH_2 Bn/Nap), 35.7 (C-3), 29.9 (C-8), 15.4 (C-6), 14.9 (C-12); HRMS: $[M+Na]^+$ calcd for $C_{38}H_{38}O_9SNa$ 693.2134, found 693.2145.

Synthesis of compound 3

Scheme S1. Synthesis of 4,6-dideoxy-4-galactosazide donor **3**.

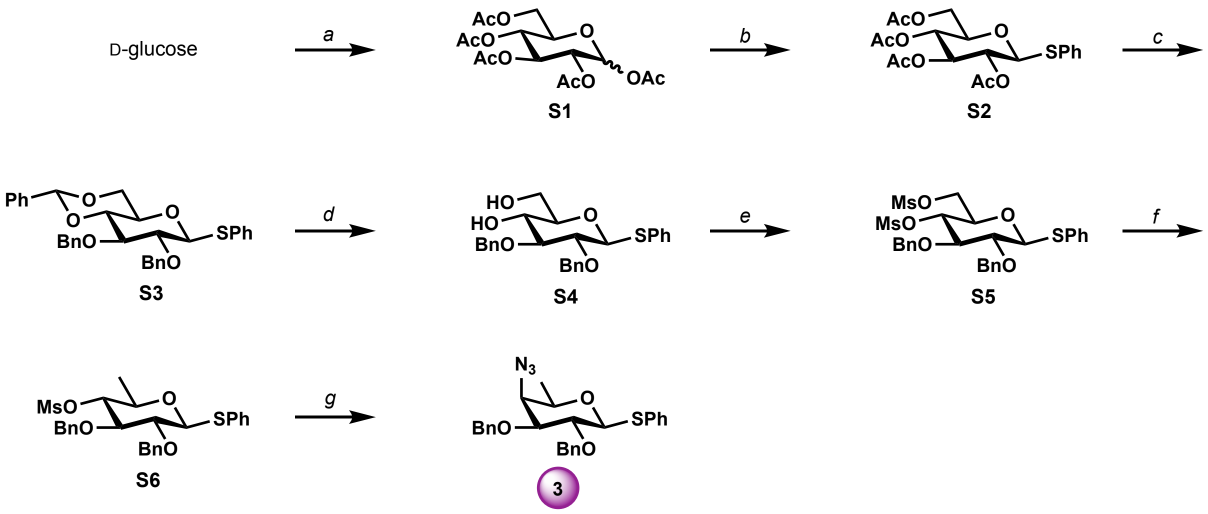

Reagents and conditions: a) Ac_2O, NaOAc, reflux (75%); b) PhSH, BF_3·OEt_2, DCM (72%); c) *i.* NaOMe, MeOH; *ii.* $PhCH(OMe)_2$, *p*TsOH, 50 °C; *iii.* NaH, BnBr, DMF (91% over 3 steps); d) CSA, MeOH (71%); e) MsCl, pyridine (95%); f) $NaBH_4$, DMSO, 85 °C (67%); g) NaN_3, 1,3-dimethyl-3,4,5,6-tetrahydro-2-(*H*)-pyrimidone, 125 °C (62%).

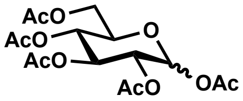

1,2,3,4,6-Penta-*O*-acetyl-D-glucopyranoside (S1). Sodium acetate (8.2 g, 100 mmol, 0.5 eq.) was dissolved in acetic anhydride (190 mL, 2.0 mol, 10 eq.) and heated to 140 °C. D-glucose (36.0 g, 200 mmol) was added portion-wise after which the solution was stirred another 15 min at 140 °C. After the solution had attained room temperature it was poured into a beaker containing ice water. The white precipitate formed on the bottom was collected and dissolved in DCM. The organic layer was washed with water (2x) followed by washing with a sat. aq. brine solution. The organic layer was dried over $MgSO_4$, filtered, and concentrated *in vacuo* to yield a white crystalline solid. Recrystallized from hot EtOH yielded the title compound (58.3 g, 149 mmol, 75%, α:β; 8:92) as a white fluffy solid. TLC: R_f 0.5 (pentane:EtOAc, 9:1, v:v); IR (neat, cm^{-1}): 1036, 1075, 1213, 1367, 1750; Data of the major stereoisomer (β-anomer): ^1H NMR (500 MHz, $CDCl_3$, HH-COSY, HSQC): δ 5.70 (d, J = 8.3 Hz, 1H, H-1), 5.23 (t, J = 9.4 Hz, 1H, H-3), 5.16 – 5.01 (m, 2H, H-2, H-4), 4.27 (dd, J = 12.5, 4.5 Hz, 1H, H-6), 4.09 (dd, J = 12.5, 2.2 Hz, 1H, H-6), 3.82 (ddd, J = 10.1, 4.5, 2.2 Hz, 1H, H-5), 2.23 – 1.76 (m, 15H, $COCH_3$); ^{13}C NMR (101 MHz, $CDCl_3$, HSQC): δ 170.7, 170.2, 169.5, 169.3, 169.0 ($\mathit{C}OCH_3$), 91.8 (C-1), 72.9 (C-3), 72.8 (C-5), 70.3 (C-4/C-2), 67.9 (C-4/C-2), 61.6 (C-6), 20.9, 20.8, 20.7, 20.6, 20.5 ($CO\mathit{C}H_3$); Diagnostic signals of the minor stereoisomer (α-anomer): δ 6.31 (d, J = 3.7 Hz, 1H, H-1), 5.55 – 5.35 (t, J = 9.9 Hz, 1H , 1H, H-3); ^{13}C NMR (101 MHz, $CDCl_3$, HSQC): δ 170.3, 169.7, 168.8 ($\mathit{C}OCH_3$), 89.2 (C-1), 69.9 (C-4), 69.3 (C-2), 68.0 (C-6), 21.0, 20.8 ($CO_2\mathit{C}H_3$); HRMS: $[M+Na]^+$ calcd for $C_{16}H_{22}O_{11}Na$ 413.1060, found 413.1054.

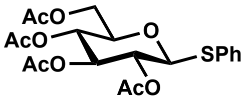

Phenyl 2,3,4,6-tetra-*O*-acetyl-1-thio-β-D-glucopyranoside (S2). Compound **S1** (58.3 g, 149 mmol) was dissolved in DCM (0.5 M, 300 mL) and cooled on ice. While stirring, BF_3·OEt_2 (27.3 mL, 223 mmol, 1.5 eq.) and thiophenol (22.9 mL, 223 mmol, 1.5 eq.) were added and consequently refluxed for 16 h. After cooling to room temperature, the solution was diluted with sat. aq. $NaHCO_3$ and Et_2O. The aqueous

layer was extracted with Et_2O followed by washing the combined organic layers with H_2O, sat. aq. $NaHCO_3$ and brine, respectively. Subsequently, the organic layer was dried over $MgSO_4$, filtered, and concentrated *in vacuo* to yield the crude product as a white crystalline solid. Recrystallization from EtOAc/pentane yielded the title compound (44.9 g, 106 mmol, 72%) as a white fluffy solid. TLC: R_f 0.6 (pentane:EtOAc, 9:1, v:v); $[\alpha]_D^{20}$ –10.4° (c 1.0, $CHCl_3$); IR (neat, cm^{-1}): 1038, 1220, 1367, 1749; ^1H NMR (500 MHz, $CDCl_3$, HH-COSY, HSQC): δ 7.53 – 7.44 (m, 2H, CH_{arom}), 7.32 (m, 3H, CH_{arom}), 5.22 (t, J = 9.4 Hz, 1H, H-3), 5.04 (t, J = 9.8 Hz, 1H, H-4), 4.97 (t, J = 9.7 Hz, 1H, H-2), 4.70 (d, J = 10.1 Hz, 1H, H-1), 4.20 (qd, J = 12.3, 3.8 Hz, 2H, H-6), 3.72 (ddd, J = 10.1, 5.1, 2.5 Hz, 1H, H-5), 2.10 – 1.97 (m, 12H, $COCH_3$); ^{13}C NMR (126 MHz, $CDCl_3$, HSQC): δ 170.7, 170.3, 169.5, 169.4 (*C*OCH_3), 133.2 (CH_{arom}), 131.8 ($C_{q\text{-}arom}$), 129.1, 128.5 (CH_{arom}), 85.9 (C-1), 75.9 (C-5), 74.1 (C-3), 70.1 (C-2), 68.3 (C-4), 62.3 (C-6), 20.9, 20.9, 20.7, 20.7 (CO*C*H_3); HRMS: $[M+Na]^+$ calcd for $C_{20}H_{24}O_9SNa$ 463.1039, found 463.1035.

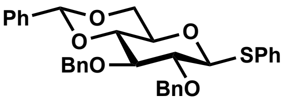

Phenyl 2,3-di-*O*-benzyl-4,6-*O*-benzylidene-1-thio-β-D-glucopyranoside (S3). Compound **S2** (44.9 g, 106 mmol) was dissolved in MeOH (0.2 M, 530 mL) followed by the addition of NaOMe (0.6 g, 10.6 mmol, 0.1 eq.). The solution was stirred for 18 h upon which the reaction was neutralized with amberlite H^+ (Sigma Aldrich Amberlite IR120 H^+ form, pre-washed with MeOH) and filtered over Celite© Hyflo Supercel (Merck). The methanol was removed under reduced pressure to yield the crude product **27** as a colorless oil. TLC: R_f 0.6 (EtOH:EtOAc, 1:2, v:v). The crude product **27** was then dissolved in DMF (56 mL) and CH_3CN (225 mL) followed by the addition of $PhCH(OMe)_2$ (22.3 mL, 148 mmol, 1.4 eq.) and *p*TsOH (1.0 g, 5.3 mmol, 0.05 eq.). After stirring for 5 h at 50 °C the reaction was quenched with solid $NaHCO_3$ (1.0 g). The solution was concentrated under reduced pressure to a fifth of its original volume and consequently diluted with EtOAc and water. The aqueous layer was extracted with Et_2O followed by washing the combined organic layers with H_2O, sat. aq. $NaHCO_3$ and brine, respectively. Subsequently, the organic layer was dried over $MgSO_4$, filtered, and concentrated *in vacuo* to yield the crude product as a brown oil. TLC: R_f 0.9 (EtOH:EtOAc, 1:2, v:v). The crude product was dissolved in DMF (0.3M, 350 mL) and cooled on ice. NaH (21.4 g, 530 mmol, 5.0 eq., 60% in mineral oil) was added followed by the portion-wise addition of BnBr (50.4 mL, 424 mmol, 4.0 eq.). The reaction mixture was allowed to reach room temperature and was stirred vigorously for 18 h. The reaction was quenched with water upon which the solution was diluted with EtOAc and water. The aqueous layer was extracted with EtOAc followed by washing the combined organic layers with H_2O, sat. aq. $NaHCO_3$ and brine, respectively. Subsequently, the organic layer was dried over $MgSO_4$, filtered, and concentrated *in vacuo* to yield the crude product as a yellow solid. Recrystallization from EtOAc/pentane yielded the title compound (52.0 g, 96.3 mmol, 91% over 3 steps) as a white crystalline solid. TLC: R_f 0.6 (pentane:EtOAc, 9:1, v:v); $[\alpha]_D^{20}$ –19.7° (c 1.0, $CHCl_3$); IR (neat, cm^{-1}): 697, 747, 1028, 1092, 2872; ^1H NMR (400 MHz, $CDCl_3$, HH-COSY, HSQC): δ 7.65 – 7.28 (m, 20H, CH_{arom}), 5.60 (s, 1H, CHPh), 4.95 (d, J = 11.1 Hz, 1H, CH*H* Bn), 4.87 (d, J = 10.2 Hz, 1H, C*H*H Bn), 4.82 (d, J = 10.3 Hz, 1H, CH*H* Bn), 4.79 (d, J = 9.9 Hz, 1H, C*H*H Bn), 4.77 (d, J = 8.6 Hz, 1H, H-1), 4.40 (dd, J = 10.5, 5.0 Hz, 1H, H-6), 3.88 – 3.78 (m, 2H, H-6, H-3), 3.72 (t, J = 9.4 Hz, 1H, H-4), 3.52 (dd, J = 9.8, 8.3 Hz, 1H, H-2), 3.48 (dq, J = 9.8, 5.1 Hz, 1H, H-5); ^{13}C NMR (101 MHz, $CDCl_3$, HSQC): δ 138.4, 138.1, 137.3, 133.2 ($C_{q\text{-}arom}$), 132.5, 129.2, 129.1, 128.5, 128.5, 128.4, 128.4, 128.3, 128.0, 128.0, 127.9, 126.1 (CH_{arom}), 101.2 (CHPh), 88.4 (C-1), 83.1 (C-3), 81.6 (C-4), 80.5 (C-2), 76.0, 75.5 (CH_2 Bn), 70.4 (C-5), 68.8 (C-6); HRMS: $[M+Na]^+$ calcd for $C_{33}H_{32}O_5SNa$ 563.1868, found.

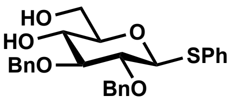

Phenyl 2,3-di-*O*-benzyl-1-thio-β-D-glucopyranoside (S4). Compound **S3** (10.8 g, 20 mmol) was dissolved in MeOH (0.1 M, 200 mL), CSA (2.3 g, 10 mmol, 0.5 eq.) was added and the solution was stirred for 18 h at room temperature. Upon full conversion, solid $NaHCO_3$ was added and the solution was concentrated under reduced pressure to a fifth of its original volume upon which the mixture was

diluted with EtOAc and water. The aqueous layer was extracted with EtOAc followed by washing the combined organic layers with H_2O, sat. aq. $NaHCO_3$ and brine, respectively. Subsequently, the organic layer was dried over $MgSO_4$, filtered, and concentrated *in vacuo* to yield the crude product as a colorless solid. Flash column chromatography (75:25 → 50:50; pentane:EtOAc) yielded the title compound (6.36 g, 14.1 mmol, 71%) as a colorless oil. TLC: R_f 0.3 (pentane:EtOAc, 1:1, v:v); $[\alpha]_D^{20}$ –30.8° (c 1.0, $CHCl_3$); IR (neat, cm^{-1}): 695, 739, 1027, 1058, 1124, 2941, 3410; ^1H NMR (500 MHz, $CDCl_3$, HH-COSY, HSQC): δ 7.55 – 7.28 (m, 15H, CH_{arom}), 4.98 (m, 1H, CH*H* Bn), 4.95 (m, 1H, C*H*H Bn), 4.76 (d, *J* = 10.3 Hz, 1H, CH*H* Bn), 4.73 (d, *J* = 9.4 Hz, 1H, H-1), 4.71 (d, *J* = 11.5 Hz, 1H, C*H*H Bn), 3.88 (ddd, *J* = 11.8, 6.7, 3.5 Hz, 1H, H-6), 3.75 (ddd, *J* = 12.1, 6.8, 5.5 Hz, 1H, H-6), 3.58 (td, *J* = 9.1, 2.5 Hz, 1H, H-5), 3.56 – 3.47 (m, 2H, H-2, H-3), 3.38 – 3.33 (m, 1H, H-5), 2.20 (d, *J* = 2.6 Hz, 1H, 4-OH), 1.98 (t, *J* = 6.7 Hz, 1H, 6-OH); ^{13}C NMR (126 MHz, $CDCl_3$, HSQC): δ 138.4, 137.9, 133.6 ($C_{q\text{-}arom}$), 131.9, 129.2, 128.9, 128.6, 128.4, 128.3, 128.2, 128.1, 127.9 (CH_{arom}), 87.9 (C-1), 86.2 (C-3), 81.1(C-2), 79.2 (C-5), 75.6, 75.6 (CH_2 Bn), 70.6 (C-4), 63.0 (C-6); HRMS: $[M+Na]^+$ calcd for $C_{26}H_{28}O_5SNa$ 475.1555, found 475.1548.

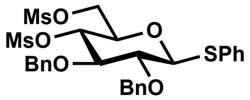

Phenyl 2,3-di-*O*-benzyl-4,6-di-*O*-methylsulfonyl-1-thio-β-D-glucopyranoside (S5). Compound **S4** (5.7 g, 12.7 mmol) was dissolved in pyridine (42 mL, 0.3 M) and cooled on ice. MsCl (3.9 mL, 50.8 mmol, 4.0 eq.) was added dropwise while stirring vigorously. The mixture was stirred for 2 h while attaining room temperature. Upon full conversion, the mixture was slowly poured on ice and EtOAc was added. The aqueous layer was extracted with EtOAc followed by washing the combined organic layers with H_2O, sat. aq. $NaHCO_3$ and brine, respectively. Subsequently, the organic layer was dried over $MgSO_4$, filtered, and concentrated *in vacuo* to yield the crude product as a yellow oil. Flash column chromatography (75:25 → 60:40; pentane:EtOAc) yielded the title compound (7.3 g, 12.0 mmol, 95%) as a colorless oil. TLC: R_f 0.5 (pentane:EtOAc, 2:1, v:v); $[\alpha]_D^{20}$ 13.2° (c 1.0, $CHCl_3$); IR (neat, cm^{-1}): 699, 750, 817, 957, 996, 1175, 1356; ^1H NMR (400 MHz, $CDCl_3$, HH-COSY, HSQC): δ 7.60 – 7.53 (m, 2H, CH_{arom}), 7.41 – 7.27 (m, 13H, CH_{arom}), 4.99 (m, 2H, CH*H* Bn, C*H*H Bn), 4.77 – 4.66 (m, 3H, CH*H* Bn, C*H*H Bn, H-1), 4.57 (dd, *J* = 11.5, 2.4 Hz, 1H, H-6), 4.52 (t, *J* = 9.6 Hz, 1H, H-4), 4.38 (dd, *J* = 11.5, 5.8 Hz, 1H, H-6), 3.79 – 3.72 (m, 2H, H-5, H-3), 3.58 (dd, *J* = 9.7, 8.8 Hz, 1H, H-2), 3.02 (s, 3H, SO_2CH_3), 2.83 (s, 3H, SO_2CH_3); ^{13}C NMR (126 MHz, $CDCl_3$, HSQC): δ 137.4, 137.3, 132.6 ($C_{q\text{-}arom}$), 132.5, 129.4, 128.7, 128.7, 128.4, 128.4, 128.3, 128.2, 127.6 (CH_{arom}), 87.8 (C-1), 83.2 (C-3), 81.1 (c-2), 76.8 (C-4), 75.9 (C-5), 75.7, 75.6 (CH_2 Bn), 68.0 (C-6), 38.7 (SO_2CH_3), 37.8 (SO_2CH_3); HRMS: $[M+Na]^+$ calcd for $C_{28}H_{32}O_9S_3Na$ 631.1106, found 631.1108.

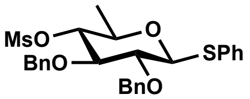

Phenyl 2,3-di-*O*-benzyl-4-*O*-methylsulfonyl-6-deoxy-1-thio-β-D-glucopyranoside (S6). Compound **S5** (6.7 g, 11.0 mmol) was dissolved in DMSO (37 mL, 0.3 M) and subsequently $NaBH_4$ (2.3 g, 60.5 mmol, 5.5 eq.) was added. The reaction mixture was heated to 85 °C and stirred for 2 h. Upon full conversion, the reaction mixture was led to attain room temperature and poured out on ice and sat. aq. NH_4Cl. The mixture was filtered and rinsed with pentane to yield the crude product as a white crystalline solid. Recrystallization from EtOAc/pentane yielded the title compound (3.8 g, 7.4 mmol, 67%) as a white solid. TLC: R_f 0.8 (pentane:EtOAc, 2:1, v:v); $[\alpha]_D^{20}$ 15.9° (c 1.0, $CHCl_3$); IR (neat, cm^{-1}): 698, 751, 813, 958, 1040, 1070, 1094, 1177, 1355, 2930; ^1H NMR (500 MHz, $CDCl_3$, HH-COSY, HSQC): δ 7.58 – 7.52 (m, 2H, CH_{arom}), 7.38 – 7.28 (m, 13H, CH_{arom}), 5.00 (m, 2H, CH*H* Bn, C*H*H Bn), 4.69 (m, 2H, CH*H* Bn, C*H*H Bn), 4.65 (d, *J* = 9.8 Hz, 1H, H-1), 4.30 (t, *J* = 9.5 Hz, 1H, H-4), 3.78 – 3.65 (m, 1H, H-3), 3.59 – 3.52 (m, 2H, H-5, H-2), 2.80 (s, 3H, SO_2CH_3), 1.44 (d, *J* = 6.2 Hz, 3H, H-6); ^{13}C NMR (126 MHz, $CDCl_3$, HSQC): δ 137.7, 137.6, 133.3 ($C_{q\text{-}arom}$), 132.4, 129.2, 128.7, 128.7, 128.4, 128.2, 128.1, 128.1, 127.5 (CH_{arom}), 87.7 (C-1), 83.5 (C-3), 82.5 (C-4), 81.7 (C-2), 75.6, 75.6 (CH_2 Bn), 74.7 (C-5), 38.9 (SO_2CH_3), 18.2 (C-6); HRMS: $[M+Na]^+$ calcd for $C_{27}H_{30}O_6S_2Na$ 537.1381, found 537.1379.

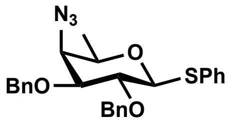

Phenyl 2,3-di-*O*-benzyl-4-azido-4,6-dideoxy-1-thio-β-D-galactopyranoside (3). Compound **S6** (3.81 g, 7.44 mmol) was dissolved in 1,3-dimethyl-3,4,5,6-tetrahydro-2-(*H*)-pyrimidone (15 mL, 0.5 M) and NaN_3 (725 mg, 11.2 mmol, 1.5 eq.) was added. The reaction mixture was heated to 125 °C and stirred for 18 h. Upon full conversion, the reaction was led to attain room temperature and diluted with water and EtOAc. The aqueous layer was extracted with EtOAc followed by washing the combined organic layers with H_2O, sat. aq. $NaHCO_3$ and brine, respectively. Subsequently, the organic layer was dried over $MgSO_4$, filtered, and concentrated *in vacuo* to yield the crude product as a white crystalline solid. Recrystallization from EtOH/pentane yielded the title compound (2.1 g, 4.6 mmol, 62%) as a white solid. TLC: R_f 0.5 (pentane:EtOAc, 9:1, v:v); $[\alpha]_D^{20}$ 15.7° (c 1.0, $CHCl_3$); IR (neat, cm^{-1}): 697, 740, 1077, 1275, 1358, 1454, 2104; ^1H NMR (500 MHz, $CDCl_3$, HH-COSY, HSQC): δ 7.62 – 7.53 (m, 2H, CH_{arom}), 7.45 – 7.27 (m, 13H, CH_{arom}), 4.81 (d, *J* = 10.2 Hz, 1H, CH*H* Bn), 4.76 – 4.73 (m, 3H, C*H*H Bn, CH*H* Bn, C*H*H Bn), 4.67 – 4.44 (m, 1H, H-1), 3.86 – 3.65 (m, 3H, H-2, H-3, H-4), 3.57 (qd, *J* = 6.2, 0.9 Hz, 1H, H-5), 1.34 (d, *J* = 6.3 Hz, 3H, H-6); ^{13}C NMR (126 MHz, $CDCl_3$, HSQC): δ 138.2, 137.7, 133.8 ($C_{q\text{-}arom}$), 132.1, 128.9, 128.7, 128.5, 128.4, 128.1, 128.0, 127.9, 127.6 (CH_{arom}), 87.8 (C-1), 83.2 (C-3), 77.0 (C-2), 75.9 (CH_2 Bn), 73.3 (C-5), 72.9 (CH_2 Bn), 63.8 (C-4), 18.0 (C-6); HRMS: $[M+Na]^+$ calcd for $C_{26}H_{27}O_3N_3SNa$ 484.1671, found 484.1668.

Synthesis of compound 2

Scheme S2. Synthesis of compound **2**.

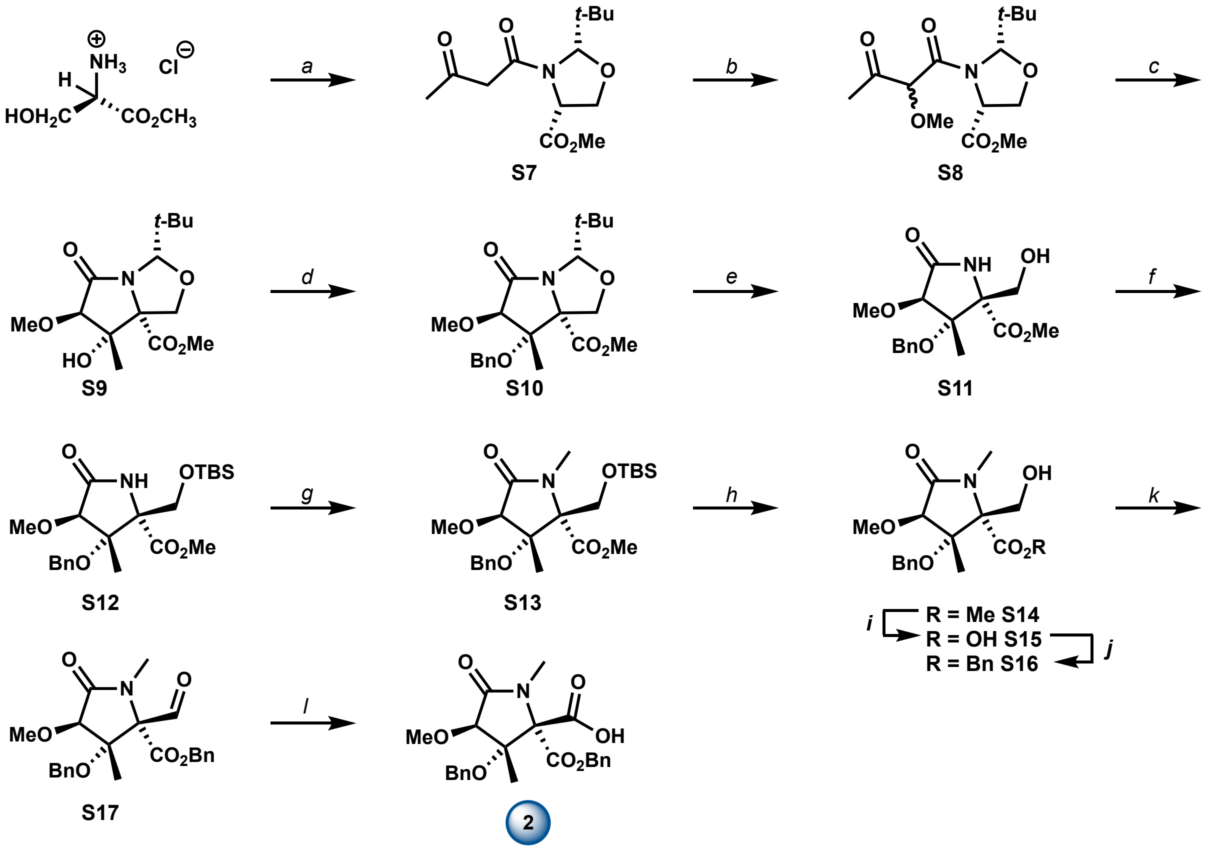

Reagents and conditions: a) *i:* pivaldehyde, Et_3N, pentane; *ii:* acetoacetic acid, EDC·HCl, DMAP, DCM (81%); b) BAIB, $BF_3·OEt_2$, MeOH (64%); c) DBU, toluene (61%); d) BnBr, NaH, TBAI, DMF (95%); e) 1,3-propanedithiol, 37% HCl, TFE (*quant.*); f) TBSCl, imidazole, DCM (93%); g) MeI, NaH, DMF (95%); h) TBAF, THF (76%); i) $LiOH·H_2O$, THF, H_2O (*quant.*); j) *i:* Cs_2CO_3, MeOH, H_2O; *ii:* BnBr, DMF (*quant.*); k) TPAP, NMO, 4 Å MS, DCM (72%); l) $NaClO_2$, 20% aq. NaH_2PO_4, 2-methyl-2-butene, *t*-BuOH (*quant.*).

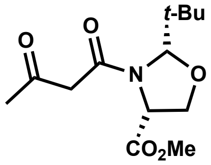

(2*S*,4*R*)-Methyl-2-(*tert*-butyl)-3-(3-oxobutanoyl)oxazolidine-4-carboxylate (S7). D-serine methyl ester hydrochloride (23 g, 150 mmol) was added to a stirred solution of pentane (750 mL, 0.2 M), Et_3N (27 mL, 195 mmol, 1.3 eq.) and *t*-butyl aldehyde (21 mL, 195 mmol, 1.3 eq.) at room temperature. The mixture was refluxed for 18 h using a Dean-Stark apparatus, upon cooling back to room temperature the emulsion was filtered off and the residue thoroughly washed with pentane. The combined filtrate was concentrated to yield crude product as a clear oil. Subsequently, the crude product was dissolved in dry DCM and cooled on ice. Acetoacetic acid (18.4 g, 180 mmol, 1.2 eq.), EDC·HCl (34.5 g, 180 mmol, 1.2 eq.) and DMAP (1.8 g, 15.0 mmol, 0.1 eq.) were added and the mixture was stirred for 18 h at room temperature. Upon full conversion, the solution was diluted with water and EtOAc, the aqueous layer was extracted with EtOAc followed by washing the combined organic layers with H_2O, sat. aq. $NaHCO_3$ and brine, respectively. Subsequently, the organic layer was dried over $MgSO_4$, filtered, and concentrated *in vacuo* to yield the crude product. Flash column chromatography (80:20 → 40:60; pentane:EtOAc) yielded the title compound (32.8 g, 121 mmol, 81% over 2 steps, keto-enol tautomers; 58:42) as a colorless oil. TLC: R_f 0.5 (pentane:EtOAc, 1:1, v:v); IR (neat, cm^{-1}): 1176, 1329, 1634, 1668, 1745, 2957; NMR data for keto form: ^1H NMR (500 MHz, $CDCl_3$, HH-COSY, HSQC): δ 5.30 (s, 1H, C*H*C(CH_3)$_3$), 4.62 (d, *J* = 5.7 Hz, 1H, C*H*CO_2CH_3), 4.56 – 4.43 (m, 1H, OCH_2), 4.10 – 3.95 (m, 1H, OCH_2), 3.78 (s, 3H, CO_2CH_3) 3.74 (s, 2H, CH_2C=ON), 2.30 (s, 3H, CH_3CO), 0.91 (s, 9H, C(CH_3)$_3$); ^{13}C NMR (126 MHz, $CDCl_3$, HSQC): δ 202.8 (CH_3C=O), 170.1 (*C*O_2CH_3), 168.2 (NC=O), 96.8 (*C*HC(CH_3)$_3$), 68.0 (OCH_2), 59.6 (*C*HCO_2CH_3), 52.8 (*C*O_2CH_3), 52.0 (*C*H_2C=ON), 37.5 (*C*(CH_3), 25.9 (*C*H_3C=O), 25.8 (C(*C*H_3); data for enol form: ^1H NMR (500 MHz, $CDCl_3$, HH-COSY, HSQC): 5.09 (s, 1H, C*H*C(CH_3)$_3$), 4.56 – 4.43 (m, 1H, OCH_2), 4.10 – 3.95 (m, 1H, OCH_2), 3.79 (s, 2H, CO_2CH_3), 1.97 (s, 3H, CH_3CO), 0.92 (s, 9H, C(CH_3)$_3$); ^{13}C NMR (126 MHz, $CDCl_3$, HSQC): δ 176.6 (NC=O), 170.5 (*C*O_2CH_3), 89.7 (*C*CH(CH_3)$_3$, 68.0 (OCH_2), 52.9 (*C*O_2CH_3), 30.8 (*C*(CH_3), 26.5 (C(*C*H_3), 22.1 (CH_3C=O); HRMS: $[M+Na]^+$ calcd for $C_{13}H_{21}O_5NNa$ 294.1317, found 294.1317.

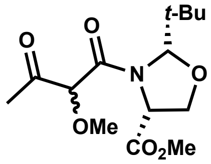

(2*S*,4*R*)-Methyl-2-(*tert*-butyl)-3-(2-methoxy-3-oxobutanoyl)oxazolidine-4-carboxylate (S8). To a vigorously stirred solution of BAIB (50.7 g, 157 mmol, 1.3 eq) in dry methanol (605 mL, 0.2 M) was dropwise added $BF_3·OEt_2$ (19.4 mL, 157 mmol, 1.3 eq.). After the solution became clear, **S7** (32.8 g, 121 mmol) in methanol (85 mL, 1.4 M) was added dropwise. The mixture was stirred for 18 h and subsequently concentrated until a fifth of its original volume. The $BF_3·OEt_2$ was quenched by the addition of a sat. aq. $NaHCO_3$ solution, the aqueous layer was extracted with EtOAc followed by washing the combined organic layers with H_2O, sat. aq. $NaHCO_3$ and brine, respectively. Subsequently, the organic layer was dried over $MgSO_4$, filtered, and concentrated *in vacuo* to yield the crude product. Flash column chromatography (90:10 → 70:30; pentane:EtOAc) yielded the title compound (23.3 g, 77 mmol, 64%, keto-enol tautomers; 95:5, diastereomeric mixture; 62:38) as a colorless oil. TLC: R_f 0.2 (pentane:EtOAc, 8:2, v:v); IR (neat, cm^{-1}): 1100, 1118, 1169, 1672, 1742, 2957; NMR data for major isomer: ^1H NMR (500 MHz, $CDCl_3$, HH-COSY, HSQC): δ 5.27 (s, 1H, C*H*C(CH_3)$_3$), 5.26 (dd, *J* = 6.9, 1.8 Hz, 1H, C*H*CO_2CH_3), 4.69 (s, 1H, C*H*OCH_3), 4.56 (dd, *J* = 8.8, 1.7 Hz, 1H, OCH_2), 3.94 (dd, *J* = 8.9, 6.7 Hz, 1H, OCH_2), 3.78 (s, 3H, CO_2CH_3), 3.47 (s, 3H, $CHOCH_3$), 2.31 (s, 3H, CH_3C=O), 0.90 (s, 9H, C(CH_3)$_3$); ^{13}C NMR (126 MHz, $CDCl_3$, HSQC): δ 207.2 (CH_3*C*=O), 170.2 (*C*O_2CH_3), 168.0 (NC=O), 97.2 ((*C*HC(CH_3), 86.9 (*C*HOCH_3), 67.8 (OCH_2), 58.3 (*C*$H_2CO_2CH_3$), 57.6 (OCH_3), 52.8 (CO_2*C*H_3), 37.3 ((*C*(CH_3)$_3$), 26.9 (*C*H_3C=O), 25.9 ((C(*C*H_3)$_3$); NMR data for minor isomer: ^1H NMR (500 MHz, $CDCl_3$,

HH-COSY, HSQC): δ 5.35 (s, 1H, C*H*C(CH_3)$_3$), 4.75 (dd, *J* = 7.0, 2.3 Hz, 1H, C*H*CO_2CH_3), 4.59 (s, 1H, C*H*OCH_3), 4.42 (dd, *J* = 8.8, 2.4 Hz, 1H, OCH_2), 3.90 (dd, *J* = 8.8, 7.0 Hz, 1H, OCH_2), 3.78 (s, 3H, CO_2CH_3), 3.45 (s, 1H, CHOC*H*$_3$), 2.27 (s, 3H, CH_3C=O), 0.95 (s, 9H, C(CH_3)$_3$); ^{13}C NMR (126 MHz, $CDCl_3$, HSQC): δ 202.7 (CH_3*C*O), 170.1 (*C*O_2CH_3), 168.7 (NC=O), 97.7 (*C*HC(CH_3)$_3$), 88.9 (*C*HOCH_3), 69.9 (OCH_2), 59.4 (*C*HCO_2CH_3), 58.7 (OCH_3), 52.7 (CO_2*C*H_3), 37.2 (*C*(CH_3)$_3$), 27.1 (*C*H_3C=O), 26.1 (C(*C*H_3)$_3$); HRMS: $[M+Na]^+$ calcd for $C_{14}H_{23}O_6NNa$ 324.1423, found 324.1423.

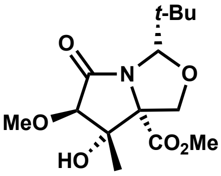

(3*S*,6*R*,7*S*,7a*S*)-Methyl-3-(*tert*-butyl)-7-hydroxy-6-methoxy-7-methyl-5-oxohexahydropyrrolo [1,2-c]oxazole-7a-carboxylate (S9). Compound **S8** (23.3 g, 77.3 mmol) was dissolved in dry toluene (1.55 L, 0.05 M), after which DBU (5.8 mL, 38.7 mmol, 0.5 eq.) was added. After stirring for 18 h at 60 °C the solution was concentrated under reduced pressure to yield the crude product as a brown solid. Flash column chromatography (90:10 → 50:50; pentane:EtOAc) yielded the title compound (14.3 g, 47.4 mmol, 61%) as a colorless oil. TLC: R_f 0.4 (pentane:EtOAc, 4:6, v:v); IR (neat, cm^{-1}): 1095, 1215, 1290, 1320, 1730, 2958; ^{1}H NMR (500 MHz, $CDCl_3$, HH-COSY, HSQC): δ 4.84 (s, 1H, C*H*C(CH_3)$_3$), 4.57 (d, *J* = 9.4 Hz, 1H, OCH_2), 4.54 (s, 1H, C*H*OCH_3), 3.89 (d, *J* = 9.4 Hz, 1H, OCH_2), 3.74 (s, 3H, CO_2CH_3), 3.71 (s, 1H, OH), 3.58 (s, 3H, OCH_3), 1.25 (s, 3H, CCH_3), 0.80 (s, 9H, C(CH_3)$_3$); ^{13}C NMR (126 MHz, $CDCl_3$, HSQC): δ 173.4 (NC=O), 171.3 (*C*O_2CH_3), 95.9 (*C*HC(CH_3)$_3$), 85.7 (CH_3O*C*H), 81.3 (HO*C*CH_3), 76.1 (*C*CO_2CH_3), 69.0 (OCH_2), 59.7 (OCH_3), 52.7 (CO_2*C*H_3), 36.4 (*C*(CH_3)$_3$), 24.9 (C(*C*H_3)$_3$), 18.1 (C*C*H_3); HRMS: $[M+Na]^+$ calcd for $C_{14}H_{23}O_6NNa$ 324.1423, found 324.1425.

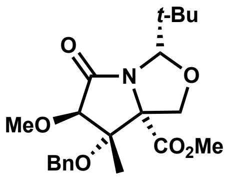

(3*S*,6*R*,7*S*,7a*S*)-Methyl-7-*O*-benzyl-3-(*tert*-butyl)-6-methoxy-7-methyl-5-oxohexahydro pyrrolo [1,2-c]oxazole-7a-carboxylate (S10). To a stirred solution of **S9** (14.3 g, 47.4 mmol) in DMF (66 mL, 0.7 M) and benzyl bromide (200 mL, 1.66 mol, 35 eq.) was added TBAI (21 g, 56.9 mmol, 1.2eq.). The solution was cooled to –15 °C and NaH (2.9 g, 71.1 mmol, 1.5 eq., 60% in mineral oil) was added in two portions. The solution was allowed to attain 0 °C followed by quenching the reaction with a sat. aq. NH_4Cl solution. The aqueous layer was extracted with EtOAc followed by washing the combined organic layers with H_2O, sat. aq. $NaHCO_3$ and brine, respectively. Subsequently, the organic layer was dried over $MgSO_4$, filtered, and concentrated *in vacuo* to yield the crude product. Flash column chromatography (95:5 → 80:20; pentane:EtOAc) yielded the title compound (17.6 g, 45.0 mmol, 95%) as a colorless oil. TLC: R_f 0.6 (pentane:EtOAc, 8:2, v:v); IR (neat, cm^{-1}): 1100, 1136, 1733, 2957; ^{1}H NMR (500 MHz, $CDCl_3$, HH-COSY, HSQC): δ 7.42 – 7.22 (m, 5H, CH_{arom}), 4.93 (s, 1H, C*H*C(CH_3)$_3$), 4.75 (d, *J* = 9.4 Hz, 1H, OCH_2), 4.73 (s, 1H, CH_3OC*H*), 4.55 (d, *J* = 11.1 Hz, 1H, CH*H* Bn), 4.48 (d, *J* = 11.1 Hz, 1H, C*H*H Bn), 4.01 (d, *J* = 9.4 Hz, 1H, OCH_2), 3.68 (s, 3H, CO_2CH_3), 3.65 (s, 3H, OCH_3), 1.37 (s, 3H, CCH_3), 0.89 (s, 9H, C(CH_3)$_3$); ^{13}C NMR (126 MHz, $CDCl_3$, HSQC): δ 173.4 (NC=O), 171.1 (*C*O_2CH_3), 137.9 ($C_{q\text{-}arom}$), 128.5, 127.8, 127.2 (CH_{arom}), 96.0 (*C*HC(CH_3)$_3$), 86.1 (*C*CH_3), 85.6 (CH_3O*C*H), 75.9 (*C*CO_2CH_3), 69.2 (OCH_2), 67.1 (CH_2 Bn), 59.4 (OCH_3), 52.8 (CO_2CH_3), 36.6 (*C*(CH_3), 25.1 (C(*C*H_3)$_3$), 14.0 (C*C*H_3); HRMS: $[M+Na]^+$ calcd for $C_{21}H_{29}O_6NNa$ 414.1893, found 414.1889.

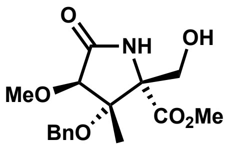

(2*S*,3*S*,4*R*)-Methyl-3-*O*-benzyl-2-(hydroxymethyl)-4-methoxy-3-methyl-5-oxopyrrolidine-2-carboxylate (S11). To a stirred solution of **S10** (17.6 g, 45.0 mmol) in CF_3CH_2OH (100 mL, 0.45 M) was added 1,3-propanedithiol (100 mL, 0.45 M) and 37% HCl aq. (1.4 mL, 17 mmol, 0.4 eq.). The solution was stirred for 2 h at 60 °C. Upon full conversion, the solution was allowed to attain room temperature and concentrated under reduced pressure to yield the crude product as a yellow oil subsequently. Flash column chromatography (40:60 → 10:90; pentane:EtOAc) yielded the title compound (14.3 g, 45.0 mmol, *quant.*) as a colorless oil. TLC: R_f 0.2 (pentane:EtOAc, 2:8, v:v); IR (neat, cm^{-1}): 1101, 1124, 1229, 1712, 3337; ^1H NMR (400 MHz, $CDCl_3$, HH-COSY, HSQC): δ 7.42 – 6.94 (m, 5H, CH_{arom}), 7.03 (s, 1H, NH) 4.58 (d, J = 11.4 Hz, 1H, CH*H* Bn), 4.47 (d, J = 11.4 Hz, 1H, C*H*H Bn), 4.22 (dd, J = 11.1, 6.5 Hz, 1H, CCH_2OH), 3.99 (s, 1H, CH_3OCH), 3.77 (dd, J = 11.1, 6.1 Hz, 1H, CCH_2OH), 3.71 (s, 3H, CO_2CH_3), 3.63 (s, 3H, OCH_3), 3.30 (t, J = 6.4 Hz, 1H, OH), 1.42 (s, 3H, CCH_3); ^{13}C NMR (101 MHz, $CDCl_3$, HSQC): δ 173.6 (NC=O), 171.1 (CO_2CH_3), 138.0 ($C_{q\text{-}arom}$), 128.4, 127.6, 126.9 (CH_{arom}), 84.4 (CCH_3), 82.6 (CH_3OCH), 72.7 (CCO_2CH_3), 65.8 (CH_2 Bn), 64.6 (CCH_2OH), 59.5 (OCH_3), 52.9 (CO_2CH_3), 12.7(CCH_3); HRMS: $[M+Na]^+$ calcd for $C_{16}H_{21}O_6NNa$ 346.1267, found 346.1261.

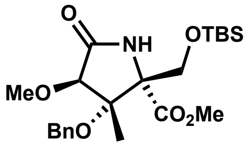

(2*S*,3*S*,4*R*)-Methyl-3-*O*-benzyl-2-(*O*-(*tert*-butyldimethylsilyl)methyl)-4-methoxy-3-methyl-5-oxopyrrolidine-2-carboxylate (S12). Compound **S11** (14.3 g, 45.0 mmol) was dissolved in DCM (900 mL, 0.05 M) followed by the addition of TBSCl (10.2 g, 67.5 mmol, 1.5 eq.) and imidazole (4.6 g, 67.5 mmol, 1.5 eq.). The solution was stirred for 16 h at room temperature, and upon full conversion, the mixture was diluted with water and brine. The aqueous layer was extracted with EtOAc followed by washing the combined organic layers with H_2O, sat. aq. $NaHCO_3$ and brine, respectively. Subsequently, the organic layer was dried over $MgSO_4$, filtered, and concentrated *in vacuo* to yield the crude product. Flash column chromatography (90:10 → 70:30; pentane:EtOAc) yielded the title compound (18.0 g, 42.0 mmol, 93%) as a colorless oil. TLC: R_f 0.5 (pentane:EtOAc, 8:2, v:v); IR (neat, cm^{-1}): 837, 1088, 1252, 1720, 2951; ^1H NMR (400 MHz, $CDCl_3$, HH-COSY, HSQC): δ 7.42 – 7.18 (m, 5H, CH_{arom}), 6.21 (s, 1H, NH), 4.57 (d, J = 11.5 Hz, 1H, C*H*H Bn), 4.48 (d, J = 11.5 Hz, 1H, CH*H* Bn), 4.23 (d, J = 9.1 Hz, 1H, CCH_2OTBS), 3.88 (s, 1H, CH_3OCH), 3.68 (d, J = 9.0 Hz, 1H, CCH_2OTBS), 3.68 (s, 3H, CO_2CH_3), 3.63 (s, 3H, OCH_3), 1.41 (s, 3H, CCH_3), 0.85 (s, 9H, $SiC(CH_3)_3$), 0.05 (d, J = 6.4 Hz, 6H, $SiCH_3$, $SiCH_3$); ^{13}C NMR (101 MHz, $CDCl_3$, HSQC): δ 172.6 (NC=O), 170.7 (CO_2CH_3), 138.1 ($C_{q\text{-}arom}$), 128.4, 127.6, 126.9 (CH_{arom}), 83.9 (CCH_3), 82.3 (CH_3OCH), 73.1 (CCO_2CH_3), 65.6 (CH_2 Bn), 65.6 (CCH_2OTBS), 59.4 (OCH_3), 52.6 (CO_2CH_3), 25.8 ($SiC(CH_3)_3$), 18.2 ($SiC(CH_3)_3$), 12.9 (CCH_3), -5.3 ($SiCH_3$), -5.6 ($SiCH_3$); HRMS: $[M+Na]^+$ calcd for $C_{22}H_{35}O_6NSiNa$ 460.2131, found 460.2127.

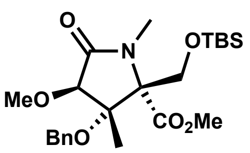

(2*S*,3*S*,4*R*)-Methyl-3-*O*-benzyl-2-(*O*-(*tert*-butyldimethylsilyl)methyl)-4-methoxy-1,3-dimethyl-5-oxopyrrolidine-2-carboxylate (S13). To a stirred solution of **S12** (530 mg, 1.23 mmol) in DMF (25 mL, 0.05 M) was added MeI (0.77 mL, 12.3 mL, 10.0 eq.). The mixture was cooled to 0 °C and NaH (128 mg, 3.2 mmol, 2.6 eq., 60% in mineral oil) was added. The mixture was allowed to attain room temperature, and upon full conversion, quenched with a sat. aq. NH_4Cl solution. The aqueous layer was extracted with EtOAc followed by washing the combined organic layers with H_2O, sat. aq. $NaHCO_3$ and brine, respectively. Subsequently, the organic layer was dried over $MgSO_4$, filtered, and concentrated *in*

vacuo to yield the crude product. Flash column chromatography (90:10 → 70:30; pentane:EtOAc) yielded the title compound (518 mg, 1.16 mmol, 95%) as a colorless oil. TLC: R_f 0.4 (pentane:EtOAc, 9:1, v:v); IR (neat, cm^{-1}): 837, 1098, 1249, 1715, 2952; ^1H NMR (400 MHz, $CDCl_3$, HH-COSY, HSQC): δ 7.39 – 7.18 (m, 5H, CH_{arom}), 4.62 (d, *J* = 11.5 Hz, 1H, C*H*H Bn), 4.51 (d, *J* = 11.5 Hz, 1H, CH*H* Bn), 4.21 (d, *J* = 10.8 Hz, 1H, CCH_2OTBS), 3.99 (s, 1H, CH_3OCH), 3.96 (d, *J* = 10.8 Hz, 1H, CCH_2OTBS), 3.67 (s, 3H, OCH_3), 3.66 (s, 3H, CO_2CH_3), 2.92 (s, 3H, NCH_3), 1.39 (s, 3H, CCH_3), 0.87 (s, 9H, $SiC(CH_3)_3$), 0.08 (S, 3H, $SiCH_3$), 0.08 (S, 3H, $SiCH_3$); ^{13}C NMR (101 MHz, $CDCl_3$, HSQC): δ 172.6 (NC=O), 170.3 (CO_2CH_3), 138.4 ($C_{q\text{-}arom}$), 128.4, 127.6, 126.9 (CH_{arom}), 83.3 (CCH_3), 82.5 (CH_3OCH), 75.2 (CCO_2CH_3), 65.9 (CH_2 Bn), 63.2 (CCH_2OTBS), 59.5 (OCH_3), 52.4 (CO_2CH_3), 28.5 (NCH_3), 25.8 ($SiC(CH_3)_3$), 18.1 ($SiC(CH_3)_3$), 13.4 (CCH_3), -5.6 ($SiCH_3$), -5.8 ($SiCH_3$); HRMS: $[M+Na]^+$ calcd for $C_{23}H_{37}O_6NSiNa$ 474.2288, found 474.2287.

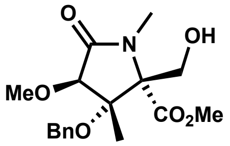

(2*S*,3*S*,4*R*)-Methyl-3-*O*-benzyl-2-(hydroxymethyl)-4-methoxy-1,3-dimethyl-5-oxopyrrolidine-2-carboxylate (S14). To a stirred solution of **S13** (18.7 g, 42 mmol) in THF (500 mL, 0.05 M) TBAF (210 mL, 1.0 M, 5.0 eq.) was added. The mixture was stirred for 3 h, and upon full conversion, quenched with water. The aqueous layer was extracted with EtOAc followed by washing the combined organic layers with H_2O, sat. aq. $NaHCO_3$ and brine, respectively. Subsequently, the organic layer was dried over $MgSO_4$, filtered, and concentrated *in vacuo* to yield the crude product. Flash column chromatography (70:30 → 30:70; pentane:EtOAc) yielded the title compound (10.5 g, 32.0 mmol, 76%) as a colorless oil. TLC: R_f 0.1 (pentane:EtOAc, 7:3, v:v); $[\alpha]_D^{20}$ 55.7° (c 1.0, $CHCl_3$); IR (neat, cm^{-1}): 1099, 1273, 1700, 1736, 3427; ^1H NMR (400 MHz, $CDCl_3$, HH-COSY, HSQC): δ 7.38 – 7.18 (m, 5H, CH_{arom}), 4.64 (d, *J* = 11.6 Hz, 1H, C*H*H Bn), 4.50 (d, *J* = 11.6 Hz, 1H, CH*H* Bn), 4.09 (d, *J* = 12.2 Hz, 1H, CH_2OH), 4.07 (s, 1H, CH_3CH), 3.98 (d, *J* = 12.2 Hz, 1H, CH_2OH), 3.72 (s, 3H, CO_2CH_3), 3.70 (s, 3H, OCH_3), 2.89 (s, 3H, NCH_3), 2.76 (s, 1H, OH), 1.44 (s, 3H, CCH_3); ^{13}C NMR (101 MHz, $CDCl_3$, HSQC): δ 172.6 (NC=O), 167.9 (CO_2CH_3), 137.5 ($C_{q\text{-}arom}$), 128.6, 128.0, 127.1 (CH_{arom}), 84.5 (CCH_3), 82.4 (CH_3OCH), 80.3 (CCO_2CH_3), 67.0 (CH_2 Bn), 62.6 (CH_2OH), 59.5 (OCH_3), 53.4 (CO_2CH_3), 29.1 (NCH_3), 14.4 (CCH_3); HRMS: $[M+Na]^+$ calcd for $C_{17}H_{23}O_6NNa$ 360.1423, found 360.1421.

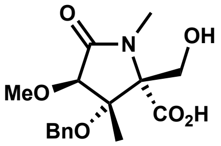

(2*S*,3*S*,4*R*)-3-*O*-Benzyl-2-(hydroxymethyl)-4-methoxy-1,3-dimethyl-5-oxopyrrolidine-2-carboxylic acid (S15). Compound **S14** (1.65 g, 5.0 mmol) was dissolved in THF (50 mL, 0.1 M) and H_2O (50 mL, 0.1 M), $LiOH\cdot H_2O$ (1.05 g, 25.0 mmol, 5.0 eq.) was added and the mixture was stirred for 18 h. Upon full conversion, the pH was adjusted with 1 M aq. HCl until a pH = 1 was obtained. The aqueous layer was extracted with EtOAc followed by washing the combined organic layers with H_2O, sat. aq. $NaHCO_3$ and brine, respectively. Subsequently, the organic layer was dried over $MgSO_4$, filtered, and concentrated *in vacuo* to yield the product (1.58 g, 5.0 mmol, *quant.*) as a white solid. TLC: R_f 0.5 (DCM:MeOH, 8:2, v:v); $[\alpha]_D^{20}$ 29.0° (c 0.5, $CHCl_3$); IR (neat, cm^{-1}): 1099, 1213, 1453, 1691, 2944, 3434; ^1H NMR (400 MHz, $CDCl_3$, HH-COSY, HSQC): δ 7.34 – 7.16 (m, 5H, CH_{arom}), 4.62 (d, *J* = 11.7 Hz, 1H, C*H*H Bn), 4.49 (d, *J* = 11.7 Hz, 1H, CH*H* Bn), 4.14 – 4.05 (m, 2H, CH_2OH, CH_3CH), 3.98 (d, *J* = 12.4 Hz, 1H, CH_2OH), 3.64 (s, 3H, OCH_3), 2.89 (s, 3H, NCH_3), 1.44 (s, 3H, CCH_3); ^{13}C NMR (101 MHz, $CDCl_3$, HSQC): δ 173.7 (CO_2H), 172.7 (NC=O), 138.0 ($C_{q\text{-}arom}$), 128.4, 127.6, 126.8 (CH_{arom}), 83.5 (CCH_3), 81.9 (CH_3OCH), 74.8 (CCO_2H), 66.1 (CH_2 Bn), 62.3 (CH_2OH), 59.6 (OCH_3), 28.0 (NCH_3), 12.9 (CCH_3); HRMS: $[M+Na]^+$ calcd for $C_{16}H_{21}O_6NNa$ 346.1267, found 346.1261.

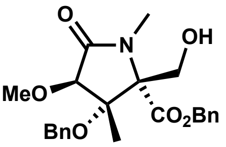

Benzyl-(2*S*,3*S*,4*R*)-Methyl-3-*O*-benzyl-2-(hydroxymethyl)-4-methoxy-1,3-dimethyl-5-oxopyrrolidine-2-carboxylate (S16). To a stirred solution of **S15** (535 mg, 1.7 mmol) in MeOH:H_2O (5:1, 3.4 mL, 0.5 M) was added Cs_2CO_3 (276 mg, 0.85 mmol, 0.5 eq.), after 30 min the mixture was concentrated under reduced pressure, co-evaporated to dryness with toluene (3x) and dissolved in DMF (8.5 mL, 0.2 M). The solution was cooled on ice and consequently BnBr (240 µL, 2.0 mmol, 1.2 eq.) was added. Upon 18 h of stirring the mixture was quenched with a sat. aq. NH_4Cl solution, the aqueous layer was extracted with EtOAc followed by washing the combined organic layers with H_2O, sat. aq. $NaHCO_3$ and brine, respectively. Subsequently, the organic layer was dried over $MgSO_4$, filtered, and concentrated *in vacuo* to yield the crude product. Flash column chromatography (50:50; pentane:EtOAc) yielded the title compound (889 mg, 1.7 mmol, *quant.*) as a colorless oil. TLC: R_f 0.4 (pentane:EtOAc, 1:1, v:v); $[\alpha]_D^{20}$ 69.3° (c 1.0, $CHCl_3$); IR (neat, cm^{-1}): 1098, 1217, 1454, 1700, 3430; ^1H NMR (400 MHz, $CDCl_3$, HH-COSY, HSQC): δ 7.40 – 7.08 (m, 10H, CH_{arom}), 5.16 (d, *J* = 12.1 Hz, 1H, C*H*H Bn), 5.08 (d, *J* = 12.1 Hz, 1H, CH*H* Bn), 4.60 (d, *J* = 11.5 Hz, 1H, C*H*H Bn), 4.43 (d, *J* = 11.5 Hz, 1H, CH*H* Bn), 4.10 (dd, *J* = 12.4, 7.9 Hz, 1H, CH_2OH), 4.04 (s, 1H, CH_3OCH), 3.98 (dd, *J* = 12.4, 6.0 Hz, 1H, CH_2OH), 3.65 (s, 3H, OCH_3), 2.88 (s, 3H, NCH_3), 2.77 (dd, *J* = 7.9, 6.1 Hz, 1H, OH), 1.43 (s, 3H, CCH_3); ^{13}C NMR (101 MHz, $CDCl_3$, HSQC): δ 172.4 (CO_2H), 170.2 (NC=O), 138.1, 134.8 ($C_{q\text{-}arom}$), 128.8, 128.7, 128.5, 127.6, 127.0 (CH_{arom}), 83.5 (CCH_3), 82.1 (CH_3OCH), 74.9 (CCO_2Bn), 67.8, 66.1 (CH_2 Bn), 62.5 (CH_2OH), 59.5 (OCH_3), 27.9 (NCH_3), 12.9 (CCH_3); HRMS: $[M+Na]^+$ calcd for $C_{23}H_{27}O_6NNa$ 436.1736, found 436.1731.

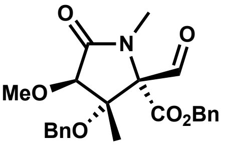

Benzyl-(2*R*,3*S*,4*R*)-Methyl-3-*O*-benzyl-2-formyl-4-methoxy-1,3-dimethyl-5-oxopyrrolidine-2-carboxylate (S17). Compound **S16** (774 mg, 1.9 mmol) was dissolved in dry DCM (38 mL, 0.05 M) and 4 Å molecular sieves were added. After stirring the solution for 30 min under an inert atmosphere, NMO (333 mg, 2.9 mmol, 1.5 eq.) and TPAP (33 mg, 0.1 mmol, 0.05 eq.) were added. Full conversion was achieved in approximately 6 h upon which the solution was concentrated to yield the crude product as a black oil. Flash column chromatography (90:10 → 80:20; pentane:EtOAc) yielded the title compound (551 mg, 1.37 mmol, 72%) as a colorless oil. TLC: R_f 0.8 (pentane:acetone, 8:2, v:v); $[\alpha]_D^{20}$ 56.9° (c 1.0, $CHCl_3$); IR (neat, cm^{-1}): 1101, 1218, 1722, 3033; ^1H NMR (400 MHz, $CDCl_3$, HH-COSY, HSQC): δ 10.07 (s, 1H, CHO), 7.46 – 7.07 (m, 10H, CH_{arom}), 5.23 (d, *J* = 12.0 Hz, 1H, C*H*H Bn), 5.16 (d, *J* = 12.0 Hz, 1H, CH*H* Bn), 4.62 (d, *J* = 11.2 Hz, 1H, C*H*H Bn), 4.52 (d, *J* = 11.2 Hz, 1H, CH*H* Bn), 4.17 (s, 1H, CH_3OCH), 3.65 (s, 3H, OCH_3), 2.83 (s, 3H, NCH_3), 1.33 (s, 3H, CCH_3); ^{13}C NMR (101 MHz, $CDCl_3$, HSQC): δ 194.4 (CHO), 172.6 (CO_2Bn), 167.3 (NC=O), 137.5, 134.4 ($C_{q\text{-}arom}$), 128.9, 128.8, 128.6, 128.6, 128.0, 127.3 (CH_{arom}), 84.5 (CCH_3), 82.3 (CH_3OCH), 80.3 (CCO_2Bn), 68.5, 67.0 (CH_2 Bn), 59.5 (OCH_3), 29.1 (NCH_3), 14.4 (CCH_3); HRMS: $[M+Na]^+$ calcd for $C_{23}H_{25}O_6NNa$ 434.1580, found 434.1576.

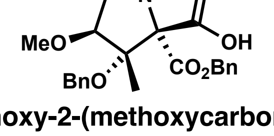

Benzyl-(2*R*,3*S*,4*R*)-3-*O*-benzyl-4-methoxy-2-(methoxycarbonyl)-1,3-dimethyl-5-oxopyrrolidine-2-carboxylic acid (2). A stirred solution of **S17** (551 mg, 1.37 mmol) in *t*-BuOH (15.6 mL, 0.1 M) and 2-methyl-2-butene (9.6 mL) was treated with an aqueous solution of $NaClO_2$ (1.23 g, 13.7 mmol, 10 eq.) in 20% NaH_2PO_4 (9.6 mL). After 2 h the mixture was quenched by adding sat. aq. NaS_2O_3 and sat. aq. NH_4Cl. The aqueous layer was extracted twice with EtOAc followed by washing the combined organic

layers with H_2O, sat. aq. $NaHCO_3$ and brine, respectively. Subsequently, the organic layer was dried over $MgSO_4$, filtered, and concentrated *in vacuo* to yield the crude product. Flash column chromatography (50:50; pentane:EtOAc) yielded the title compound (889 mg, 1.7 mmol, *quant.*) as a colorless oil. TLC: R_f 0.4 (pentane:EtOAc, 1:1, v:v); $[\alpha]_D^{20}$ 64.3° (c 1.0, $CHCl_3$); IR (neat, cm^{-1}): 1050, 1095, 1134, 1274, 1727, 2937; ^1H NMR (400 MHz, $CDCl_3$, HH-COSY, HSQC): δ 7.41 – 7.12 (m, 10H, CH_{arom}), 5.28 (d, J = 11.9 Hz, 1H, C*H*H Ph), 5.22 (d, J = 11.9 Hz, 1H, CH*H* Ph), 4.67 (d, J = 11.6 Hz, 1H, C*H*H Ph), 4.41 (d, J = 11.6 Hz, 1H, CH*H* Ph), 4.00 (s, 1H, CH_3OCH), 3.62 (s, 3H, OCH_3), 2.82 (s, 3H, NCH_3), 1.46 (s, 3H, CCH_3); ^{13}C NMR (101 MHz, $CDCl_3$, HSQC): δ 172.2 (NC=O), 170.4 (CO_2Bn), 164.8 (CO_2H), 137.4, 133.7 ($C_{q\text{-}arom}$), 129.4, 129.0, 128.9, 128.6, 128.0, 127.1 (CH_{arom}), 84.5 (*C*CH_3), 82.6 (CH_3OCH), 78.2 (*C*CO_2Bn), 69.8, 66.9 (CH_2 Bn), 59.6 (OCH_3), 28.9 (NCH_3), 15.3 (C*C*H_3); HRMS: $[M+Na]^+$ calcd for $C_{23}H_{25}O_7NNa$ 450.1529, found 450.1524.

Synthesis of compound 23

Scheme S3. Synthesis of compound **23**.

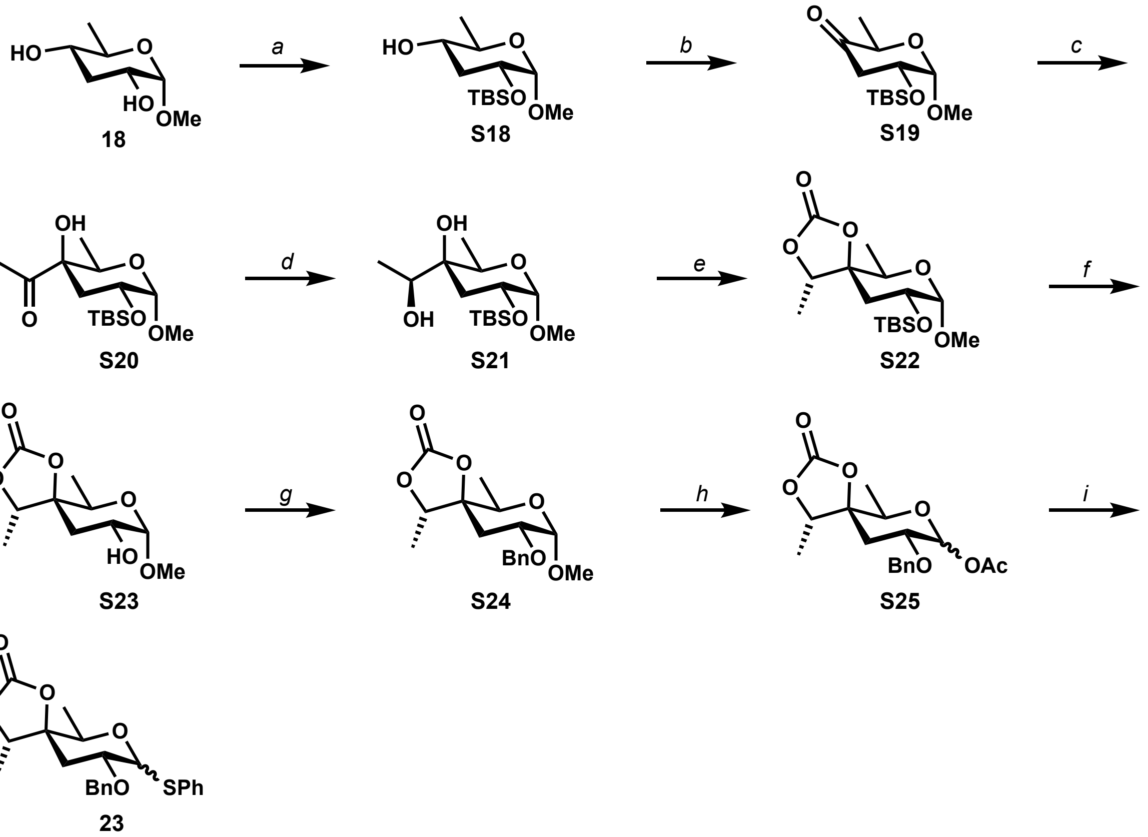

Reagents and conditions: a) imidazole, TBSCl, DMF, –30 °C (69%); b) DMP, DCM (91%); c) AcCl, SmI_2, THF (57%); d) $ZnBH_4$, THF (86%); e) triphosgene, pyridine, DCM (78%); f) HCl aq., MeOH (91%); g) benzyl 2,2,2-trichloroacetimidate, TfOH, dioxane (*quant.*); h) Ac_2O, H_2SO_4 (98%); i) thiophenol, $BF_3{\cdot}OEt_2$, DCM (97%).

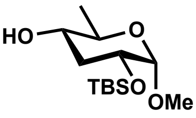

Methyl 3,6-dideoxy-2-*O*-*tert*-butyldimethylsilyl-α-D-allopyranoside (S18). Compound **18** (8.4 g, 51.5 mmol) and imidazole (6.8 g, 103 mmol, 2.0 eq.) were dissolved in DMF (103 mL, 0.5 M), the solution was cooled to –30 °C upon which TBSCl (8.2 g, 54 mmol, 1.05 eq.) was added. The mixture was stirred for 2 h while the mixture was allowed to warm to room temperature. Upon full conversion, the reaction was quenched with water and diluted with Et_2O. The aqueous layer was extracted with Et_2O (3x) followed

by washing the combined organic layers with H_2O, sat. aq. $NaHCO_3$ and brine, respectively. Subsequently, the organic layer was dried over $MgSO_4$, filtered, and concentrated *in vacuo* to yield the crude product as a colorless oil. Flash column chromatography (95:5 → 80:20; pentane:EtOAc) yielded the title compound (9.9 g, 35.7 mmol, 69%) as a colorless oil. TLC: R_f 0.8 (pentane:EtOAc, 7:3, v:v); $[\alpha]_D^{20}$ 36.4° (c 1.0, $CHCl_3$); IR (neat, cm^{-1}): 1260, 2930, 3445; ^1H NMR (400 MHz, $CDCl_3$, HH-COSY, HSQC): δ 4.48 (d, *J* = 3.4 Hz, 1H, H-1), 3.78 (ddd, *J* = 11.7, 4.7, 3.5 Hz, 1H, H-2), 3.53 (dq, *J* = 9.0, 6.2 Hz, 1H, H-5), 3.42 (s, 3H, CH_3 OMe), 3.28 (ddd, *J* = 11.2, 9.2, 4.6 Hz, 1H, H-4), 2.03 (dt, *J* = 11.6, 4.6 Hz, 1H, H-3), 1.90 (s, 1H, 4-OH), 1.82 (q, *J* = 11.5 Hz, 1H, H-3), 1.25 (d, *J* = 6.2 Hz, 3H, H-6), 0.89 (s, 9H, $C(CH_3)_3$), 0.08 (s, 6H, $Si(CH_3)_2$); ^{13}C NMR (101 MHz, $CDCl_3$, HSQC): δ 99.3 (C-1), 71.2 (C-4), 68.9 (C-2), 68.5 (C-5), 55.2 (CH_3 OMe), 36.8 (C-3), 26.0 ($C(\mathit{C}H_3)_3$), 18.4 ($\mathit{C}(CH_3)_3$), 17.5 (C-6), -4.5 ($SiCH_3$), -4.6 ($SiCH_3$); HRMS: $[M+Na]^+$ calcd for $C_{13}H_{28}O_4SiNa$ 299.1655, found 299.1654.

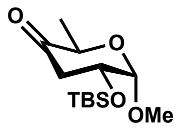

Methyl 3,6-dideoxy-2-*O*-*tert*-butyldimethylsilyl-α-D-erythropyranosid-4-ulose (S19). Compound **S18** (9.8 g, 35.7 mmol) was dissolved in DCM (210 mL, 0.17 M) under N_2 atmosphere. Dess-Martin periodinane (22.7 g, 53.6 mmol, 1.5 eq.) was added and the mixture was stirred for 4.5 h upon the reaction was quenched with water. The aqueous layer was extracted with DCM (3x) followed by washing the combined organic layers with H_2O, sat. aq. $NaHCO_3$ and brine, respectively. Subsequently, the organic layer was dried over $MgSO_4$, filtered, and concentrated *in vacuo* to yield the crude product as a white oil. Flash column chromatography (95:5 → 90:10; pentane:Et_2O) yielded the title compound (8.9 g, 32.5 mmol, 91%) as a colorless oil. TLC: R_f 0.5 (pentane:Et_2O, 9:1, v:v); $[\alpha]_D^{20}$ –8.0° (c 0.5, $CHCl_3$); IR (neat, cm^{-1}): 1119, 1261, 1728, 1794, 2857, 2930; ^1H NMR (400 MHz, $CDCl_3$, HH-COSY, HSQC): δ 4.71 (d, *J* = 3.3 Hz, 1H, H-1), 4.17 – 4.07 (m, 2H, H-5 and H-2), 3.53 (s, 3H, CH_3 OMe), 2.76 (dd, *J* = 15.2, 10.8 Hz, 1H, H-3), 2.61 (dd, *J* = 15.3, 5.6 Hz, 1H, H-3), 1.27 (d, *J* = 6.7 Hz, 3H, CH_3 H-6), 0.89 (s, 9H, $C(CH_3)_3$), 0.09 (s, 3H, $SiCH_3$), 0.08 (s, 3H, $SiCH_3$); ^{13}C NMR (101 MHz, $CDCl_3$, HSQC): δ 206.8 (C-4), 99.4 (C-1), 70.1 (C-5), 69.3 (C-2), 56.1 (CH_3 OMe), 44.0 (C-3), 25.9 ($\mathit{C}(CH_3)_3$), 18.3 (($C(\mathit{C}H_3)_3$), 14.6 (C-6), -4.6 ($SiCH_3$); HRMS: $[M+Na]^+$ calcd for $C_{13}H_{26}O_4SiNa$ 297.1498, found 297.1496.

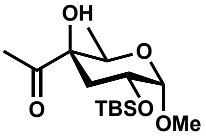

Methyl 4-*C*-acetyl-3,6-dideoxy-2-*O*-*tert*-butyldimethylsilyl-α-D-galactopyranoside (S20). Compound **S19** (221 mg, 0.8 mmol) was co-evaporated with dry toluene once under N_2 atmosphere. Glycoside **S19** was dissolved in THF (1.6 mL, 0.5 M) and cooled to –80 °C. The solution was flushed with N_2 with 30 mbar overpressure for 25 min, after which AcCl (143 µL, 2 mmol, 2.5 eq.) was added and the solution was flushed with N_2 with 30 mbar overpressure for another 5 min. A flame-dried flask was flushed with N_2 by using a Schlenk line for 16 h. After flushing the flask, it was filled with SmI_2 (28 mL, 2.8 mmol, 3.5 eq, [0.1 M solution in THF, stabilized by samarium chips, Sigma-Aldrich]) by using a pre-flushed cannula. The flask with SmI_2 was heated to 40 °C and the solution of ketone **S19** and AcCl in THF was added with a syringe. The reaction was quenched after 10 min with 20 mL 1 M HCl, diluted with 20 mL EtOAc and stirred for 30 min. The mixture was washed with H_2O, sat. aq. $Na_2S_2O_3$ and brine, respectively. The organic phase was dried over $MgSO_4$, filtered, and concentrated *in vacuo*. Flash column chromatography (90:10; pentane:EtOAc) afforded the title compound (145 mg, 455 µmol, 57%) as a colorless oil. TLC: R_f 0.3 (pentane:Et_2O, 8:2, v:v); $[\alpha]_D^{20}$ 42.4° (c 1.0, $CHCl_3$); IR (neat, cm^{-1}): 1115, 1263, 1709, 2930, 3455; ^1H NMR (500 MHz, $CDCl_3$, HH-COSY, HSQC, HMBC): δ 4.63 (d, *J* = 3.4 Hz, 1H, H-1), 4.23 (q, *J* = 6.4 Hz, 1H, H-5), 4.17 (ddd, *J* = 11.5, 4.9, 3.5 Hz, 1H, H-2), 3.95 (s, 1H, 4-OH), 3.50 (s, 3H, CH_3 OMe), 2.32 (t, *J* = 11.9 Hz, 1H, H-3), 2.25 (s, 3H, H-8), 1.60 (ddd, *J* = 12.3, 4.9, 0.9 Hz, 1H, H-3), 0.96 (d, *J* = 6.5 Hz, 3H, H-6), 0.89 (s, 9H, $C(CH_3)_3$), 0.09 (s, 3H, $SiCH_3$), 0.08 (1s, 3H, $SiCH_3$); ^{13}C NMR (126 MHz, $CDCl_3$, HSQC, HMBC): δ 208.5 (C-7), 100.0 (C-1), 81.1 (C-4), 66.0 (C-2), 65.3 (C-

5), 55.9 (CH_3 OMe), 36.4 (C-3), 25.9 (C(*C*H_3)$_3$), 24.5 (C-8), 18.3 (*C*(CH_3)$_3$), 14.1 (C-6), -4.5 ($SiCH_3$), -4.6 ($SiCH_3$); HRMS: $[M+Na]^+$ calcd for $C_{15}H_{30}O_5SiNa$ 341.1759, found 341.1760.

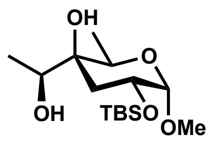

Methyl 2-*O*-*tert*-butyldimethylsilyl-α-D-yersinioside (S21). A solution of $ZnBH_4$ was made by dissolving ZnCl (572 mg, 4.2 mmol, 4.2 eq.) and $NaBH_4$ (397 mg, 10.5 mmol, 10.5 eq.) in THF (8.4 mL, 0.5 M) at 0 °C. This solution was stirred for 1 h at 0 °C. A solution of glycoside **S20** (325 mg, 1.0 mmol) in THF (20 mL, 50 mM) was cooled to 0 °C and the $ZnBH_4$ solution was added. The reaction mixture was stirred for 24 h at room temperature and quenched with sat. NH_4Cl. The aqueous layer was extracted with EtOAc (2x), followed by washing the combined organic layers with brine. The organic phase was dried over $MgSO_4$, filtered, and concentrated *in vacuo*. Purification by flash column chromatography (80:20 → 60:40, pentane:EtOAc) afforded the title compound (275 mg, 860 µmol, 86%) as a colorless oil. TLC: R_f 0.4 (pentane:EtOAc, 6:4, v:v); $[\alpha]_D^{20}$ 40.9° (c 1.0, $CHCl_3$); IR (neat, cm^{-1}): 1263, 2930, 3424; ^1H NMR (400 MHz, $CDCl_3$, HH-COSY, HSQC): δ 4.54 (d, *J* = 3.7 Hz, 1H, H-1), 4.06 (q, *J* = 5.9, 5.1 Hz, 1H, H-5), 4.03 (ddd, *J* = 9.0, 6.3, 4.4 Hz, 1H, H-2), 3.68 (m, *J* = 13.5, 6.6 Hz, 1H, H-7), 3.43 (s, 3H, CH_3 OMe), 2.38 (s, 1H, 4-OH), 2.12 (s, 1H, 7-OH), 1.89 (m, 1H, H-3), 1.59 (ddd, *J* = 12.7, 5.3, 0.9 Hz, 1H, H-3), 1.22 (d, *J* = 6.6 Hz, 3H, H-8), 1.19 (d, *J* = 6.5 Hz, 3H, H-6), 0.90 (s, 9H, C(CH_3)$_3$), 0.09 (s, 6H, $SiCH_3$, $SiCH_3$); ^{13}C NMR (101 MHz, $CDCl_3$, HSQC): δ 99.6 (C-1), 74.9 (C-4), 72.1 (C-7), 67.0 (C-2), 65.7 (C-5), 55.6 (CH_3 OMe), 35.4 (C-3), 26.0 (C(*C*H_3)$_3$), 18.4 (*C*(CH_3)$_3$), 17.2 (C-8), 14.5 (C-6), -4.5 ($SiCH_3$), -4.5 ($SiCH_3$); HRMS: $[M+Na]^+$ calcd for $C_{15}H_{32}O_5SiNa$ 343.1916, found 343.1917.

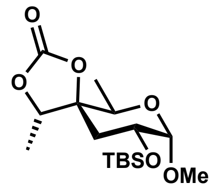

Methyl 2-*O*-*tert*-butyldimethylsilyl-4,7-*O*-carbonate-α-D-yersinioside (S22). Compound **S21** (270 mg, 840 µmol) was dissolved in dry DCM (1 mL, 0.4 M) and pyridine (0.5 mL, 6.3 mmol, 7.5 eq.) and cooled on ice. While stirring, triphosgene (124 mg, 0.42 mmol, 0.5 eq.) dissolved in 1.1 mL dry DCM was added dropwise and the mixture was stirred at 0 °C for 16 h. Upon full conversion, the reaction was quenched with ice-cooled sat. aq. NH_4Cl and diluted with EtOAc. The aqueous layer was extracted twice with EtOAc followed by washing the combined organic layers with brine. Subsequently, the organic layer was dried over $MgSO_4$, filtered, and concentrated *in vacuo* to yield the crude product. Flash column chromatography (95:5 → 80:20; pentane:EtOAc) yielded the title compound (226 mg, 650 µmol, 78%) as a white solid. TLC: R_f 0.7 (pentane:EtOAc, 8:2, v:v); $[\alpha]_D^{20}$ 67.0° (c 1.0, $CHCl_3$); IR (neat, cm^{-1}): 1007, 1054, 1066, 1088, 1812, 2929; ^1H NMR (500 MHz, $CDCl_3$, HH-COSY, HSQC, NOESY) δ 4.55 (d, *J* = 3.4 Hz, 1H, H-1), 4.34 (q, *J* = 6.9 Hz, 1H, H-7), 4.05 (ddd, *J* = 11.6, 5.0, 3.5 Hz, 1H, H-2), 3.90 (q, *J* = 6.3 Hz, 1H, H-5), 3.44 (s, 3H, OCH_3), 2.07 (dd, *J* = 13.5, 11.7 Hz, 1H, H-3), 1.84 (dd, *J* = 13.5, 4.9 Hz, 1H, H-3), 1.44 (d, *J* = 6.9 Hz, 3H, H-8), 1.28 (d, *J* = 6.4 Hz, 3H, H-6), 0.89 (s, 9H, SiC(CH_3)$_3$), 0.08 (s, 6H, $SiCH_3$, $SiCH_3$). ^{13}C NMR (126 MHz, $CDCl_3$) δ 154.2 (O(C=O)O), 99.0 (C-1), 85.0 (C-4), 81.5 (C-7), 66.0 (C-2), 64.6 (C-5), 55.9 (OCH_3), 36.4 (C-3), 25.8 (SiC(*C*H_3)$_3$), 18.2 (Si*C*(CH_3)$_3$),14.7 (C-6), 13.1 (C-8), -4.6 ($SiCH_3$), -4.6 ($SiCH_3$); HRMS: $[M+Na]^+$ calcd for $C_{16}H_{30}O_6SiNa$ 369.1709, found 369.1710.

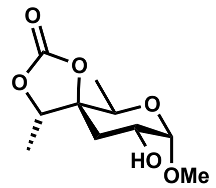

Methyl 4,7-*O*-carbonate-α-D-yersinioside (S23). To a stirred solution of **S22** (230 mg, 660 µmol) in MeOH (19.4 mL, 0.034 M) was added a 6 M aq. HCl solution (1.1 mL, 6.6 mmol, 10 eq.). Upon full conversion, the mixture was neutralized by addition of Amberlite IRA-67 (Sigma Aldrich Amberlite IRA-67 free base, pre-washed with MeOH), filtered, and concentrated under reduced pressure to yield the crude product. Flash column chromatography (50:50 → 0:100; pentane:EtOAc) yielded the title compound (139.3 mg, 0.6 mmol, 91%) as a white solid. TLC: R_f 0.1 (pentane:EtOAc, 1:1, v:v); $[\alpha]_D^{20}$ 125.2° (c 1.0, $CHCl_3$); IR (neat, cm^{-1}): 1008, 1051, 1063, 1201, 1788, 1807, 3460; ^{1}H NMR (500 MHz, $CDCl_3$, HH-COSY, HSQC): δ 4.68 (d, *J* = 3.7 Hz, 1H, H-1), 4.36 (q, *J* = 6.9 Hz, 1H, H-7), 3.97 (dddd, *J* = 11.4, 10.2, 5.1, 3.7 Hz, 1H, H-2), 3.87 (q, *J* = 6.3 Hz, 1H, H-5), 3.46 (s, 3H, CH_3 OCH_3), 2.04 (dd, *J* = 13.4, 5.1 Hz, 1H, H-3), 1.98 (d, *J* = 10.2 Hz, 1H, 2-OH), 1.90 (dd, *J* = 13.4, 11.5 Hz, 1H, H-3), 1.45 (d, *J* = 6.9 Hz, 3H, H-8), 1.29 (d, *J* = 6.4 Hz, 2H, H-6); ^{13}C NMR (126 MHz, $CDCl_3$): δ 154.1 (O(C=O)O), 98.1 (C-1), 84.6 (C-4), 81.6 (C-7), 65.0 (C-2), 64.8 (C-5), 55.8 (CH_3 OCH_3), 36.4 (C-3), 14.8 (C-6), 13.1 (C-8); HRMS: $[M+Na]^+$ calcd for $C_{10}H_{16}O_6Na$ 255.0845, found 255.0845.

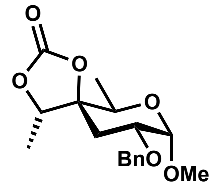

Methyl 2-*O*-benzyl-4,7-*O*-carbonate-α-D-yersinioside (S24). To a stirred solution of **S23** (251 mg, 1.1 mmol) in dioxane (10.8 mL, 0.1 M) was added benzyl 2,2,2-trichloroacetimidate (0.4 mL, 2.2 mmol, 2.0 eq.) followed by the addition of TfOH (19.1 µL, 216 µmol, 0.2 eq.). After stirring for 60 min at room temperature the reaction was quenched by addition of sat. aq. $NaHCO_3$ and diluted with EtOAc. The aqueous layer was extracted twice with EtOAc followed by washing the combined organic layers with H_2O, sat. aq. $NaHCO_3$ and brine, respectively. Subsequently, the organic layer was dried over $MgSO_4$, filtered, and concentrated *in vacuo* to yield the crude product. Flash column chromatography (80:20 → 50:50; pentane:EtOAc) yielded the title compound (354 mg, 1.1 mmol, *quant.*) as a white solid. TLC: R_f 0.5 (pentane:EtOAc, 1:1, v:v); $[\alpha]_D^{20}$ 55.9° (c 1.0, $CHCl_3$); IR (neat, cm^{-1}): 1008, 1052, 1065, 1086, 1206, 1273, 1727, 1792, 1807; ^{1}H NMR (500 MHz, $CDCl_3$, HH-COSY, HSQC): δ 7.38 – 7.27 (m, 5H, CH_{arom}), 4.65 (d, *J* = 3.3 Hz, 1H, H-1), 4.61 (d, *J* = 12.1 Hz, 1H, C*H*H Bn), 4.56 (d, *J* = 12.1 Hz, 1H, CH*H* Bn), 4.33 (q, *J* = 6.9 Hz, 1H, H-7), 3.89 (q, *J* = 6.3 Hz, 1H, H-5), 3.81 (ddd, *J* = 11.7, 5.0, 3.4 Hz, 1H, H-2), 3.41 (s, 3H,CH_3 OCH_3), 2.07 (dd, *J* = 13.4, 11.8 Hz, 1H, H-3), 1.98 (dd, *J* = 13.5, 5.0 Hz, 1H, H-3), 1.44 (d, *J* = 6.9 Hz, 3H, H-8), 1.26 (d, *J* = 6.4 Hz, 3H, H-6); ^{13}C NMR (126 MHz, $CDCl_3$, HSQC): δ 154.1 (O(C=O)O), 137.8 ($C_{q\text{-}arom}$), 128.7, 128.2, 128.0 (CH_{arom}), 96.9 (C-1), 84.8 (C-4), 81.5 (C-7), 71.8 (CH_2 Bn), 71.6 (C-2), 64.8 (C-5), 55.7 (CH_3 OCH_3), 33.6 (C-3), 14.8 (C-6), 13.1 (C-8); HRMS: $[M+Na]^+$ calcd for $C_{17}H_{22}O_6Na$ 345.1314, found 345.1316.

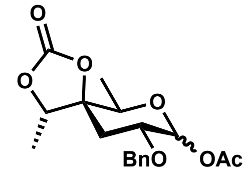

Acetyl 2-*O*-benzyl-4,7-*O*-carbonate-D-yersinioside (S25). Compound **S24** (83 mg, 260 µmol) was dissolved in Ac_2O (4.7 mL, 0.05 M) and cooled on ice. Subsequently, H_2SO_4 (28 µL, 0.5 mmol, 2.0 eq.) was dissolved in 0.5 mL Ac_2O and added dropwise to the mixture. After stirring the solution for exactly 2 min, sat. aq. $NaHCO_3$ and EtOAc were added dropwise and stirred for another 15 min. The aqueous layer was extracted twice with EtOAc followed by washing the combined organic layers with H_2O, sat.

aq. $NaHCO_3$ and brine, respectively. Subsequently, the organic layer was dried over $MgSO_4$, filtered, and concentrated *in vacuo* to yield the crude product. Flash column chromatography (80:20 → 50:50; pentane:EtOAc yielded the title compound (89 mg, 254 µmol, 98%, α:β; 66:34) as a white solid. TLC: R_f 0.5 (pentane:EtOAc, 1:1, v:v); IR (neat, cm^{-1}): 1009, 1064, 1091, 1227, 1751, 1805; Data of the major stereoisomer (α-anomer): ^1H NMR (500 MHz, $CDCl_3$, HH-COSY, HSQC): δ 7.39 – 7.24 (m, 5H, CH_{arom}), 6.33 (d, J = 3.3 Hz, 1H, H-1), 4.63 (d, J = 11.6 Hz, 1H, C*H*H Bn), 4.52 (d, J = 11.6 Hz, 1H, CH*H* Bn), 4.38 (q, J = 6.9 Hz, 1H, H-7), 4.00 (q, J = 6.3 Hz, 1H, H-5), 3.93 (ddd, J = 9.9, 6.9, 3.4 Hz, 1H, H-2), 2.16 (s, 3H, $COCH_3$), 2.10 – 2.06 (m, 2H, H-3, H-3), 1.47 (d, J = 6.9 Hz, 3H, H-8), 1.28 (d, J = 6.3 Hz, 3H, H-6); ^{13}C NMR (126 MHz, $CDCl_3$, HSQC): δ 169.4 (C=O OAc), 153.8 (O(C=O)O), 137.4 ($C_{q\text{-}arom}$), 128.7, 128.7,128.2, 127.9, 127.7 (CH_{arom}), 88.6 (C-1), 81.5 (C-7), 72.0 (CH_2 Bn), 70.4 (C-2), 67.5 (C-5), 33.9 (C-3), 21.1 (CH_3 Ac), 14.9 (C-6), 13.2 (C-8); Diagnostic signals of the minor stereoisomer (β-anomer): ^1H NMR (500 MHz, $CDCl_3$, HH-COSY, HSQC): δ 5.60 (d, J = 7.3 Hz, 1H, H-1), 4.47 (q, J = 6.8 Hz, 1H, H-7), 3.75 (ddd, J = 10.2, 7.2, 5.1 Hz, 1H, H-2), 2.34 (dd, J = 14.3, 5.1 Hz, 1H, H-3), 2.12 (s, 3H, $COCH_3$), 1.85 (dd, J = 14.3, 10.2 Hz, 1H, H-3), 1.45 (d, J = 6.9 Hz, 3H, H-8), 1.35 (d, J = 6.3 Hz, 3H, H-6); ^{13}C NMR (126 MHz, $CDCl_3$, HSQC): δ 169.6 (C=O OAc), 153.7 (O(C=O)O), 137.7 ($C_{q\text{-}arom}$), 94.4 (C-1), 80.8 (C-7), 73.1 (CH_2 Bn), 72.8 (C-2), 72.6 (C-5), 37.6 (C-3), 21.2 (CH_3 Ac), 15.4 (C-6), 13.3 (C-8); HRMS: $[M+Na]^+$ calcd for $C_{18}H_{22}O_7Na$ 373.1263, found 373.1259.

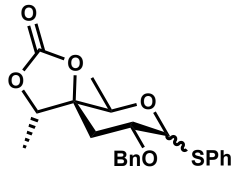

Phenyl 2-*O*-benzyl-4,7-*O*-carbonate-1-thio-D-yersinioside (23). Compound **S25** (347 mg, 990 µmol) was dissolved in DCM (9.9 mL, 0.1 M) and thiophenol (111 µL, 1.1 mmol, 1.1 eq.) was added. Subsequently, the solution was cooled to –80 °C and $BF_3{\cdot}OEt_2$ (147 µL, 1.2 mmol, 1.2 eq.) was added dropwise, the solution was stirred for 16 h while attaining to 0 °C. Upon full conversion, the reaction was quenched with sat. aq. $NaHCO_3$ and diluted with EtOAc. The aqueous layer was extracted twice with EtOAc followed by washing the combined organic layers with H_2O, sat. aq. $NaHCO_3$ and brine, respectively. Subsequently, the organic layer was dried over $MgSO_4$, filtered, and concentrated *in vacuo* to yield the crude product. Flash column chromatography (90:10 → 60:40; pentane:EtOAc) yielded the title compound (383 mg, 956 µmol, 97%, α:β; 56:44) as a colorless oil. TLC: R_f 0.2 (pentane:EtOAc, 9:1, v:v); IR (neat, cm^{-1}): 693, 1009, 1069, 1199, 1793, 1805; Data of the major stereoisomer (α-anomer): ^1H NMR (500 MHz, $CDCl_3$, HH-COSY, HSQC): δ 7.64 – 7.23 (m, 10H, CH_{arom}), 5.68 (d, J = 4.6 Hz, 1H, H-1), 4.71 (d, J = 11.5 Hz, 1H, C*H*H Bn), 4.55 (d, J = 11.5 Hz, 1H, H-3 CH*H* Bn), 4.45 (q, J = 6.3 Hz, 1H, H-5), 4.39 (q, J = 6.6 Hz, 1H, H-7), 4.15 (dt, J = 11.3, 4.9 Hz, 1H, H-2), 2.16 – 1.98 (m, 2H, H-3, H-3), 1.51 (d, J = 6.9 Hz, 3H, H-8), 1.28 (d, J = 6.3 Hz, 3H, H-6); ^{13}C NMR (126 MHz, $CDCl_3$): δ 154.0 (O(C=O)O), 137.7, 133.1 ($C_{q\text{-}arom}$), 132.6, 131.4, 129.2, 129.1, 128.7, 128.7, 128.3, 128.2, 128.1, 127.4 (CH_{arom}), 86.6 (C-1), 84.5 (C-4), 81.6 (C-7), 71.5 (C-2), 71.5 (CH_2 Bn), 66.1 (C-5), 35.8 (C-3), 14.8 (C-6), 13.3 (C-8); Diagnostic signals of the minor stereoisomer (β-anomer): ^1H NMR (500 MHz, $CDCl_3$, HH-COSY, HSQC): δ 4.70 (d, J = 11.3 Hz, 1H, C*H*H Bn), 4.58 (d, J = 9.3 Hz, 1H, H-1), 4.53 (d, J = 11.3 Hz, 1H, CH*H* Bn), 4.35 (q, J = 6.7 Hz, 1H, H-7), 3.72 – 3.65 (m, 2H, H-2, H-5), 2.33 (dd, J = 14.1, 5.0 Hz, 1H, H-3), 1.77 (dd, J = 14.1, 10.6 Hz, 1H, H-3), 1.42 (d, J = 6.9 Hz, 3H, H-8), 1.37 (d, J = 6.2 Hz, 3H, H-6); ^{13}C NMR (126 MHz, $CDCl_3$, HSQC): δ 153.9 (O(C=O)O), 137.3, 133.9 ($C_{q\text{-}arom}$), 88.6 (C-1), 84.2 (C-4), 80.9 (C-7), 75.3 (C-2/C-5), 73.2 (CH_2 Bn), 72.5 (C-2/C-5), 39.9 (C-3), 15.5 (C-6), 13.1 (C-8); HRMS: $[M+Na]^+$ calcd for $C_{22}H_{24}O_5SNa$ 423.1242, found 423.1237.

Synthesis of compound 24 and S36

Scheme S4. Synthesis of compound **24** and **S36**.

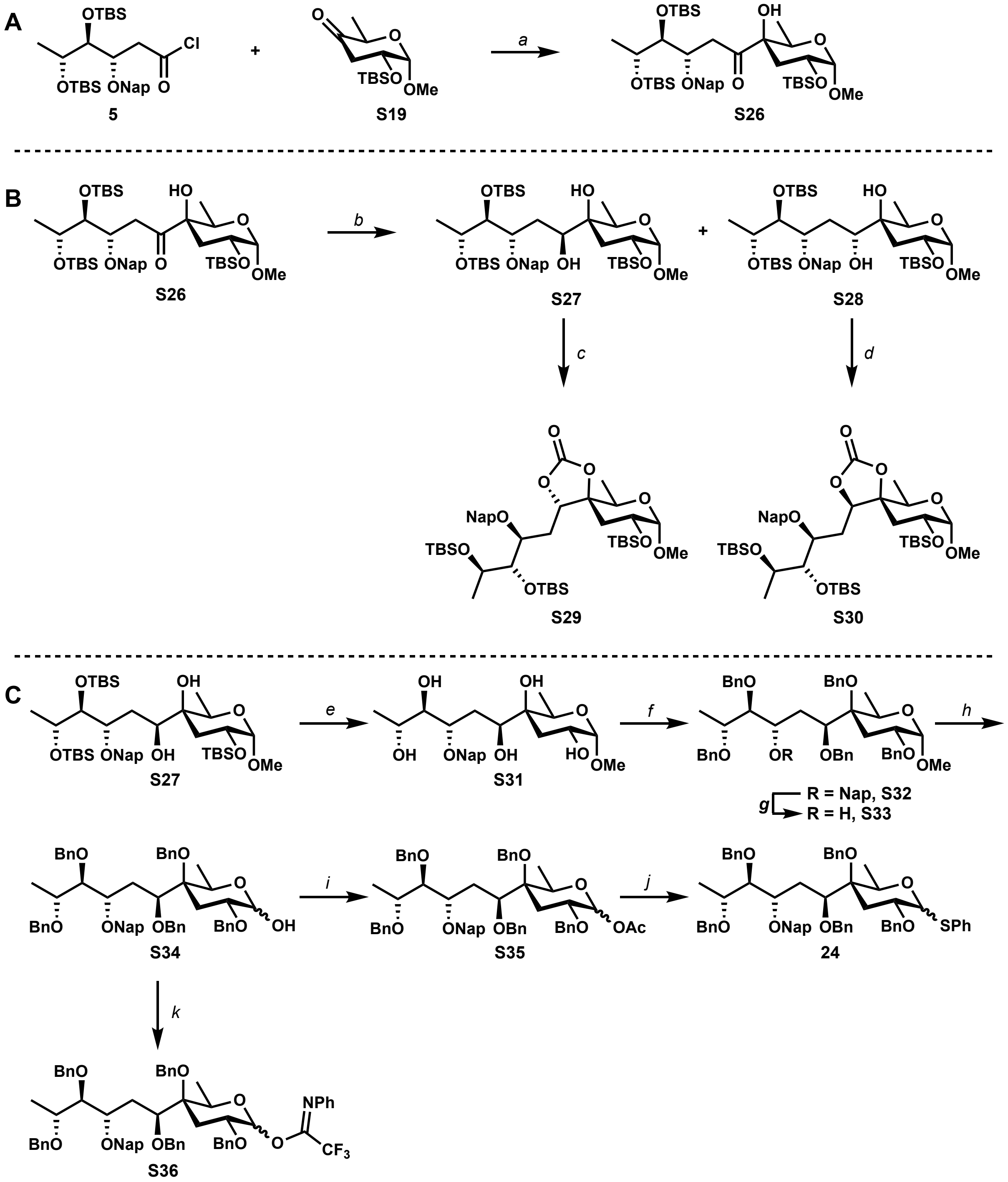

Reagents and conditions: a) SmI_2, THF, 40 °C, 15 min (70%); b) $Zn(BH_4)_2$, THF (82%, and 10% for the C-7 epimer); c) and d) $COCl_2$, Et_3N, THF (79% and 48% for the C-7 epimer); e) 6 M HCl, MeOH (*quant.*); f) BnBr, NaH, DMF (74%); g) DDQ, DCM/MeOH 4:1 (70%); h) $SrCl_2 \cdot H_2O$, aq. HCOOH 80%, dioxane (68%); i) Ac_2O, pyridine (85%); j) thiophenol, $BF_3 \cdot OEt_2$, DCM (61%); k) 2,2,2,-trifluoro-*N*-phenylacetimidoyl chloride, $CsCO_3$, acetone (85%).

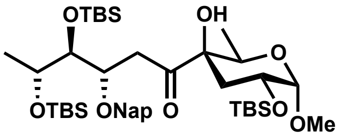

Methyl 2-*O*-*tert*-butyldimethylsilyl-3,6-dideoxy-4-C-((3*S*,4*S*,5*R*)-4,5-*O*-bis((*tert*-butyldimethylsilyl)oxy)-3-*O*-2-methylnaphthalene-hexan-1-one)-α-D-galacto-hexapyranoside (S26). Acid **5** (1.06 g, 2.0 mmol) was dissolved in dry THF (20 mL, 0.1 M). This solution was cooled to 0 °C while stirring, pyridine (242 µL, 3.0 mmol, 1.5 eq.) and oxalyl chloride (220 µL, 2.6 mmol, 1.3 eq.) were added respectively. The solution was stirred for 30 min on ice after which it was warmed to room temperature over a time span of 15 min. The suspension was diluted with pentane and filtered into a flask containing ketone **S19** (457 mg, 1.67 mmol, 0.8 eq.), resulting in a clear liquid which was concentrated *in vacuo* to yield the crude acid chloride **5** combined with ketone **S19** as a colorless oil. While gently stirring, a constant gas flow of nitrogen was applied for 20 min after which the mixture was heated to 40 °C followed by the addition of a solution of samarium(II)iodide (0.1 M) in THF (59 mL, 5.85 mmol, 3.5 eq.). After 10 min the heat source was removed and the solution was quenched with air and diluted with EtOAc, aq. 1.0 M HCl and stirred for 30 min. The aqueous layer was extracted with EtOAc (3x) followed by washing the combined organic layers with sat. aq. NaS_2O_3. Subsequently, the organic layer was dried over $MgSO_4$, filtered, and concentrated *in vacuo* to yield the crude product as a colorless oil. Flash column chromatography (97:3 → 95:5; pentane:Et_2O) yielded the title compound (850 mg, 1.08 mmol, 65% based on **S19**) as a colorless oil. TLC: R_f 0.5 (pentane:Et_2O, 9:1, v:v); $[\alpha]_D^{20}$ 15.7° (c 1.0, $CHCl_3$); IR (neat, cm^{-1}): 775, 835, 1112, 1253, 1471, 1707, 2856, 2929; ^1H NMR (400 MHz, $CDCl_3$, HH-COSY, HSQC): δ 7.83 – 7.65 (m, 4H, CH_{arom}), 7.48 – 7.30 (m, 3H, CH_{arom}), 4.70 (d, *J* = 11.6 Hz, 1H, CH*H* Nap), 4.59 (m, 1H, C*H*H Nap), 4.57 (s, 1H, H-1), 4.33 (dt, *J* = 10.0, 2.5 Hz, 1H, H-9), 4.23 (q, *J* = 6.4 Hz, 1H, H-5), 4.11 (ddd, *J* = 11.5, 4.8, 3.4 Hz, 1H, H-2), 3.94 (s, 1H, 4-OH), 3.77 – 3.67 (m, 2H, H-10, H-11), 3.45 (s, 3H, CH_3 OMe), 3.29 (dd, *J* = 17.1, 10.0 Hz, 1H, H-8), 2.47 – 2.35 (m, 2H, H-8, H-3), 1.50 (dd, *J* = 12.3, 4.6 Hz, 1H, H-3), 1.18 (d, *J* = 5.8 Hz, 3H, H-12), 0.93 (d, *J* = 2.6 Hz, 3H, H-6) 0.92 – 0.78 (m, 27H, $C(CH_3)_3$, $C(CH_3)_3$, $C(CH_3)_3$), 0.14 – -0.07 (m, 18H, $SiCH_3$, $SiCH_3$, $SiCH_3$, $SiCH_3$, $SiCH_3$, $SiCH_3$); ^{13}C NMR (101 MHz, $CDCl_3$ HSQC): δ 210.2 (C-7), 135.7, 133.3, 133.0 ($C_{q\text{-}arom}$), 128.0, 128.0, 127.8, 126.4, 126.1, 126.0, 125.8 (CH_{arom}), 100.1 (C-1), 81.4 (C-4), 78.2 (C-10/C-11), 77.0 (C-9), 72.9 (CH_2 Nap), 70.0 (C-11/C-10), 66.2 (C-2), 65.0 (C-5), 55.7 (CH_3 OMe), 38.3 (C-8), 36.1 (C-3), 26.2 ($C(\mathit{C}H_3)_3$), 26.0 ($C(\mathit{C}H_3)_3$), 25.9 ($C(\mathit{C}H_3)_3$), 20.5 (C-12), 18.5 ($\mathit{C}(CH_3)_3$), 18.2 ($\mathit{C}(CH_3)_3$), 18.2 ($\mathit{C}(CH_3)_3$), 14.3 (C-6), -4.0 ($SiCH_3$), -4.1 ($SiCH_3$), -4.2 ($SiCH_3$), -4.6 ($SiCH_3$), -4.6 ($SiCH_3$), -4.6 ($SiCH_3$); HRMS: $[M+Na]^+$ calcd for $C_{42}H_{74}O_8Si_3Na$ 813.4589, found 813.4598.

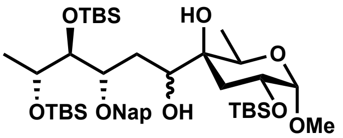

Methyl 2,10,11-tris-*O*-(*tert*-butyldimethylsilyl)-9-*O*-2-methylnaphthalene-α-D-caryophylloside (S27) and **Methyl 7-epi-2,10,11-tris-*O*-(*tert*-butyldimethylsilyl)-9-*O*-2-methylnaphthalene-α-D-caryophylloside (S28).** A $Zn(BH_4)_2$ solution was prepared by dissolving anhydrous $ZnCl_2$ (209 mg, 1.54 mmol) in dry THF (2.95 mL), at 0 °C $NaBH_4$ (148 mg, 3.9 mmol) was added and the solution was stirred for 18 h. **S26** (47.4 mg, 60 µmol) was dissolved in dry THF (2.4 mL, 0.025 M) after which it was cooled on ice, 0.6 mL of the $Zn(BH_4)_2$ solution (0.31 mmol, 5.2 eq.) was added. The solution was led to warm to room temperature and stirred for 18 h. The reaction was quenched with sat. aq. NH_4Cl and diluted with EtOAc and brine, the aqueous layer was extracted with EtOAc (3x) followed by washing the combined organic layers with H_2O, sat. aq. $NaHCO_3$ and brine, respectively. Subsequently, the organic layer was dried over $MgSO_4$, filtered, and concentrated *in vacuo* to yield the crude products as a separable diastereomeric mixture. Flash column chromatography (95:5 → 90:10; pentane:Et_2O) yielded the C-7 epimer **S28** and the caryophyllose **S27** in an 11:89 ratio respectively. Yielding caryophyllose **S27** (39 mg, 49 µmol, 82%) and the C-7 epimer **S28** (5 mg, 6 µmol, 10%) both as colorless oils. TLC: R_f 0.2 and 0.5 for the caryophyllose **S27** and C-7 epimer **S28** respectively (pentane:EtOAc, 9:1, v:v);

Data of the major stereoisomer caryophyllose **S27**: $[\alpha]_D^{20}$ 13.3° (c 1.0, $CHCl_3$); IR (neat, cm^{-1}): 775, 835, 1056, 1104, 1252, 2928, 3483; ^1H NMR (400 MHz, $CDCl_3$, HH-COSY, HSQC): δ 8.01 – 7.65 (m, 4H, CH_{arom}), 7.47 (m, 3H, CH_{arom}), 4.79 (d, *J* = 11.9 Hz, 1H, CH*H* Nap), 4.64 (d, *J* = 11.9 Hz, 1H, C*H*H Nap), 4.52 (d, *J* = 3.6 Hz, 1H, H-1), 4.12 – 3.99 (m, 2H, H-2, H-5), 3.92 – 3.79 (m, 2H, H-9, H-11), 3.75 (dd, *J* = 5.5, 3.6 Hz, 1H, H-10), 3.67 (d, *J* = 10.4 Hz, 1H, H-7), 3.39 (s, 3H, CH_3 OMe), 2.35 (d, *J* = 4.2 Hz, 1H, 4-OH), 2.28 (s, 1H, 7-OH), 2.00 – 1.91 (m, 2H, H-3, H-8), 1.66 – 1.54 (m, 2H, H-3, H-8), 1.17 (d, *J* = 6.1 Hz, 3H, H-12), 1.11 (d, *J* = 6.5 Hz, 3H, H-6), 0.93 – 0.85 (m, 27H, $C(CH_3)_3$, $C(CH_3)_3$, $C(CH_3)_3$), 0.14 – 0.02 (m, 18H, $SiCH_3$, $SiCH_3$, $SiCH_3$, $SiCH_3$, $SiCH_3$, $SiCH_3$); ^{13}C NMR (101 MHz, $CDCl_3$ HSQC): δ 135.9, 133.4, 133.1 ($C_{q\text{-}arom}$), 128.3, 128.0, 127.8, 126.8, 126.3, 126.1, 126.0 (CH_{arom}), 99.7 (C-1), 79.0 (C-10), 77.6 (C-11), 74.7 (C-4), 72.4 (C-7), 72.1 (CH_2 Nap), 69.8 (C-9), 67.1 (C-2), 66.0 (C-5), 55.4 (CH_3 OMe), 34.5 (C-3/C-8), 31.4 (C-8/C-3), 26.3 ($C(\mathit{C}H_3)_3$), 26.1 ($C(\mathit{C}H_3)_3$), 26.0 ($C(\mathit{C}H_3)_3$), 20.3 (C-12), 18.5 ($\mathit{C}(CH_3)_3$), 18.4 ($\mathit{C}(CH_3)_3$), 18.2 ($\mathit{C}(CH_3)_3$), 14.1 (C-6), -4.0 ($SiCH_3$), -4.0 ($SiCH_3$), -4.1 ($SiCH_3$), -4.4 ($SiCH_3$), -4.4 ($SiCH_3$), -4.4 ($SiCH_3$); HRMS: $[M+Na]^+$ calcd for $C_{42}H_{76}O_8Si_3Na$ 815.4746, found 815.4746.
Data of the minor stereoisomer C-7 epimer **S28**: $[\alpha]_D^{20}$ 6.4° (c 1.0, $CHCl_3$); IR (neat, cm^{-1}): 776, 835, 1052, 1104, 1252, 2928, 3502; ^1H NMR (400 MHz, $CDCl_3$, HH-COSY, HSQC): δ 7.87 – 7.74 (m, 4H, CH_{arom}), 7.52 – 7.42 (m, 3H, CH_{arom}), 4.85 (d, *J* = 11.6 Hz, 1H, CH*H* Nap), 4.58 (d, *J* = 11.6 Hz, 1H, H-1), 4.55 (m, 1H, C*H*H Nap), 4.33 (d, *J* = 1.5 Hz, 1H, 7-OH), 4.16 (ddd, *J* = 11.3, 5.2, 3.5 Hz, 1H, H-2), 3.97 (ddd, *J* = 8.9, 3.9, 1.7 Hz, 1H, H-7), 3.76 – 3.69 (m, 2H, H-5, H-10), 3.69 – 3.58 (m, 2H, H-9, H-11), 3.40 (s, 3H, CH_3 OMe), 2.95 (d, *J* = 1.0 Hz, 1H, 4-OH), 1.87 – 1.69 (m, 3H, H-3, H-3, H-8), 1.53 (dd, *J* = 15.1, 3.9 Hz, 1H, H-8), 1.22 (d, *J* = 6.0 Hz, 3H, H-12), 1.08 (d, *J* = 6.5 Hz, 3H, H-6), 0.93 – 0.78 (m, 27H, $C(CH_3)_3$, $C(CH_3)_3$, $C(CH_3)_3$), 0.14 – -0.04 (m, 18H, $SiCH_3$, $SiCH_3$, $SiCH_3$, $SiCH_3$, $SiCH_3$, $SiCH_3$); ^{13}C NMR (101 MHz, $CDCl_3$ HSQC): δ 134.8, 133.3, 133.2 ($C_{q\text{-}arom}$), 128.6, 128.1, 127.8, 127.4, 126.4, 126.3, 126.1 (CH_{arom}), 99.9 (C-1), 80.0 (C-7), 77.9 (C-4), 74.7 (C-10/C-5), 72.2 (C-9/C-11), 72.0 (CH_2 Nap), 69.6 (C-11/C-9), 67.1 (C-2), 66.6 (C-5/C-10), 55.4 (CH_3 OMe), 33.3 (C-3), 30.1 (C-8), 26.3 ($C(\mathit{C}H_3)_3$), 26.1 ($C(\mathit{C}H_3)_3$), 26.0 ($C(\mathit{C}H_3)_3$), 21.6 (C-12), 18.6 ($\mathit{C}(CH_3)_3$), 18.4 ($\mathit{C}(CH_3)_3$), 18.0 ($\mathit{C}(CH_3)_3$), 14.3 ($\mathit{C}(CH_3)_3$), -3.5 ($SiCH_3$), -3.6 ($SiCH_3$), -4.4 ($SiCH_3$), -4.4 ($SiCH_3$), -4.5 ($SiCH_3$), -4.6 ($SiCH_3$); HRMS: $[M+Na]^+$ calcd for $C_{42}H_{76}O_8Si_3Na$ 815.4746, found 815.4722.

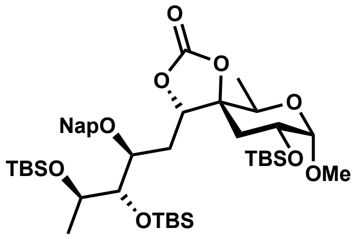

Methyl 2,10,11-tris-*O*-(*tert*-butyldimethylsilyl)-9-*O*-2-methylnaphthalene-4,7-carbonate-α-D-caryophylloside (S29). A phosgene solution was prepared by diluting a 20% phosgene in hexane solution (0.95 mL, 1.75 mmol, 5 eq.) with dry THF (1 mL). The caryophyllose **S27** (280 mg, 0.35 mmol) was dissolved in THF (2.5 mL, 0.1 M) and Et_3N (242 µL, 1.75 mmol, 5.0 eq.) and cooled on ice. The phosgene solution was added dropwise, after which the solution was stirred for 1 h at 0 °C followed by 1 h on room temperature. The reaction was quenched by adding 1 mL of sat. aq. $NaHCO_3$ followed by diluting the mixture with Et_2O and water. The aqueous layer was extracted with Et_2O (3x) followed by washing the combined organic layers with H_2O, sat. aq. $NaHCO_3$ and brine, respectively. Subsequently, the organic layer was dried over $MgSO_4$, filtered, and concentrated *in vacuo* to yield the crude product as a colorless oil. Flash column chromatography (99:1 → 97:3; pentane:EtOAc) yielded the title compound (227 mg, 0.28 mmol, 79%) as a colorless oil. TLC: R_f 0.2 (pentane:EtOAc, 9:1, v:v); $[\alpha]_D^{20}$ 8.4° (c 0.5, $CHCl_3$); IR (neat, cm^{-1}): 776, 835, 1059, 1098, 1253, 1812, 2928; ^1H NMR (400 MHz, $CDCl_3$, HH-COSY, HSQC, NOESY): δ 7.83 (dt, *J* = 11.6, 4.0 Hz, 4H, CH_{arom}), 7.53 – 7.43 (m, 3H, CH_{arom}), 4.74 (d, *J* = 11.8 Hz, 1H, CH*H* Nap), 4.60 (d, *J* = 11.7 Hz, 1H, C*H*H Nap), 4.52 (d, *J* = 3.2 Hz, 1H, H-1), 4.36 (dd, *J* = 9.6, 3.9 Hz, 1H, H-7), 4.02 (ddd, *J* = 11.5, 4.7, 3.3 Hz, 1H, H-2), 3.84 (p, *J* = 6.1 Hz, 1H, H-11), 3.77 – 3.67 (m, 2H, H-9, H-10), 3.56 (q, *J* = 6.4 Hz, 1H, H-5), 3.34 (s, 3H, CH_3 OMe), 2.08 (ddd, *J* = 15.4, 9.6, 6.2 Hz, 1H, H-8), 2.00 (dd, *J* = 13.2, 11.5 Hz, 1H, H-3), 1.94 – 1.86 (m, 2H, H-3, H-8), 1.12 (d, *J* = 6.2 Hz, 3H, H-12), 1.09 (d, *J* = 6.4 Hz, 3H, H-6), 0.93 – 0.82 (m, 27H, $C(CH_3)_3$, $C(CH_3)_3$, $C(CH_3)_3$), 0.12

– 0.03 (m, 18H, $SiCH_3$, $SiCH_3$, $SiCH_3$, $SiCH_3$, $SiCH_3$, $SiCH_3$); ^{13}C NMR (101 MHz, $CDCl_3$ HSQC): δ 153.9 (O(C=O)O), 135.5, 133.4, 133.1 ($C_{q\text{-}arom}$), 128.3, 128.1, 127.8, 127.0, 126.3, 126.2, 126.1 (CH_{arom}), 99.3 (C-1), 85.3 (C-4), 78.7 (C-7/C-10), 78.7 (C-10/C-7), 77.1 (C-9), 71.9 (CH_2 Nap), 70.1 (C-11), 66.5 (C-5), 66.3 (C-2), 55.7 (CH_3 OMe), 32.8 (C-3), 30.8 (C-8), 26.3 (C(CH_3)$_3$), 26.1 (C(CH_3)$_3$), 25.9 (C(CH_3)$_3$), 19.9 (C-12), 18.5 (C(CH_3)$_3$), 18.3 (C(CH_3)$_3$), 18.3 (C(CH_3)$_3$), 13.2 (C-6), -4.0 ($SiCH_3$), -4.0 ($SiCH_3$), -4.1 ($SiCH_3$), -4.4 ($SiCH_3$), -4.5 ($SiCH_3$), -4.6 ($SiCH_3$); HRMS: $[M+Na]^+$ calcd for $C_{43}H_{74}O_9Si_3Na$ 841.4538, found 841.4532.

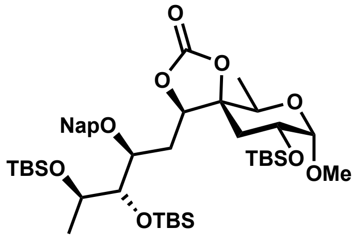

Methyl 7-epi-2,10,11-tris-*O*-(*tert*-butyldimethylsilyl)-9-*O*-2-methylnaphthalene-4,7-carbonate-α-D-caryophylloside (S30). A phosgene solution was prepared by diluting a 20% phosgene in hexane solution (265 µL, 334 µmol, 10 eq.) with dry THF (1.5 mL). The C-7 epimer **S28** (26.5 mg, 33 µmol) was dissolved in THF (0.33 mL, 0.1 M) and Et_3N (90 µL, 660 µmol, 20 eq.) and cooled on ice. The phosgene solution was added dropwise after which the solution was stirred for 1 h at 0 °C followed by 1 h on room temperature. The reaction was quenched by adding 1 mL of sat. aq. $NaHCO_3$ followed by diluting the mixture with Et_2O and water. The aqueous layer was extracted with Et_2O (3x) followed by washing the combined organic layers with H_2O, sat. aq. $NaHCO_3$ and brine, respectively. Subsequently, the organic layer was dried over $MgSO_4$, filtered, and concentrated *in vacuo* to yield the crude product as a colorless oil. Flash column chromatography (99:1 → 97:3; pentane:EtOAc) yielded the title compound (12.9 mg, 16.0 µmol, 48%) as a colorless oil. TLC: R_f 0.3 (pentane:EtOAc, 9:1, v:v); $[\alpha]_D^{20}$ 32.0° (c 1.0, $CHCl_3$); IR (neat, cm^{-1}): 776, 835, 1034, 1066, 1086, 1110, 1471, 1809, 2929; ^1H NMR (400 MHz, $CDCl_3$, HH-COSY, HSQC, NOESY): δ 7.86 – 7.75 (m, 4H, CH_{arom}), 7.52 – 7.37 (m, 3H, CH_{arom}), 4.84 (d, J = 11.9 Hz, 1H, CH*H* Nap), 4.58 (d, J = 11.8 Hz, 1H, C*H*H Nap), 4.54 (d, J = 3.3 Hz, 1H, H-1), 4.44 – 4.38 (m, 1H, H-7), 4.02 (ddd, J = 11.7, 4.9, 3.4 Hz, 1H, H-2), 3.94 – 3.82 (m, 2H, H-9, H-11), 3.70 (m, 2H, H-5, H-10), 3.40 (s, 2H, CH_3 OMe), 2.12 (dd, J = 13.5, 11.9 Hz, 1H, H-3), 1.91 – 1.83 (m, 2H, H-3, H-8), 1.76 (dd, J = 13.5, 4.8 Hz, 1H, H-8), 1.26 (d, J = 6.4 Hz, 3H, H-12), 1.19 (d, J = 5.7 Hz, 3H, H-6), 0.97 – 0.79 (m, 27H, C(CH_3)$_3$, C(CH_3)$_3$, C(CH_3)$_3$), 0.19 – 0.03 (m, 18H, $SiCH_3$, $SiCH_3$, $SiCH_3$, $SiCH_3$, $SiCH_3$, $SiCH_3$); ^{13}C NMR (101 MHz, $CDCl_3$, HSQC): δ 154.2 (C-13), 135.6, 133.4, 133.0 ($C_{q\text{-}arom}$), 128.3, 128.0, 127.8, 126.4, 126.3, 126.1, 125.7 (CH_{arom}), 99.0 (C-1), 85.1 (C-4), 82.4 (C-7), 78.3 (C-5), 77.0 (C-9), 72.6 (CH_2 Nap), 69.7 (C-10), 66.2 (C-2), 64.8 (C-11), 55.6 (CH_3 OMe), 36.3 (C-3), 28.7 (C-8), 26.2 (C(CH_3)$_3$), 26.1 (C(CH_3)$_3$), 26.0 (C(CH_3)$_3$), 20.8 (C-6), 18.5 (C(CH_3)$_3$), 18.4 (C(CH_3)$_3$), 18.2 (C(CH_3)$_3$), 15.0 (C-12), -3.9 ($SiCH_3$), -3.9 ($SiCH_3$), -4.3 ($SiCH_3$), -4.5 ($SiCH_3$), -4.5 ($SiCH_3$), -4.6 ($SiCH_3$); HRMS: $[M+Na]^+$ calcd for $C_{43}H_{74}O_9Si_3Na$ 841.4538, found 841.4538.

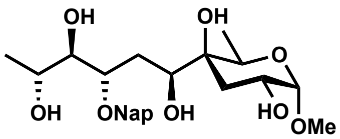

Methyl 9-*O*-2-methylnaphthalene-α-D-caryophylloside (S31). Compound **S30** (400 mg, 0.5 mmol) was dissolved in methanol (15 mL, 0.034 M), a 6 M HCl aq. solution (0.9 mL, 10 eq.) was added and the mixture was stirred for 18 h upon which the reaction was quenched by neutralizing the acid with Amberlite IRA-67 (Sigma Aldrich Amberlite IRA-67 free base, pre-washed with MeOH). The reaction mixture was filtered off and rinsed with excess methanol, concentration of the filtrate yielded the crude product as a colorless oil. Flash column chromatography (50:50 → 0:100; pentane:acetone) yielded the title compound (225 mg, 0.5 mmol, *quant.*) as a colorless oil. TLC: R_f 0.6 (acetone, 9:1, v:v); $[\alpha]_D^{20}$ 31.7° (c 1.0, $CHCl_3$); IR (neat, cm^{-1}): 972, 1052, 1695, 2928, 3352; ^1H NMR (400 MHz, CD_3OD, HH-COSY,

HSQC): δ 7.92 – 7.38 (m, 7H, CH_{arom}), 4.82 (d, *J* = 11.5 Hz, 1H, CH*H* Nap), 4.69 (d, *J* = 11.5 Hz, 1H, *CH*H Nap), 4.55 (d, *J* = 3.6 Hz, 1H, H-1), 4.12 (q, *J* = 6.5 Hz, 1H, H-5), 3.99 – 3.89 (m, 2H, H-2, H-7), 3.79 – 3.67 (m, 3H, H-9, H-10, H-11), 3.38 (s, 3H, CH_3 OMe), 2.08 – 1.92 (m, 2H, H-3, H-8), 1.68 (dd, *J* = 12.5, 5.1 Hz, 1H, H-3), 1.61 (ddd, *J* = 14.1, 10.7, 2.7 Hz, 1H, H-8), 1.23 (d, *J* = 5.7 Hz, 3H, H-12), 1.11 (d, *J* = 6.5 Hz, 3H, H-6); ^{13}C NMR (101 MHz, MeOD, HSQC): δ 137.5, 134.8, 134.4 ($C_{q\text{-}arom}$), 129.0, 128.9, 128.7, 127.7, 127.2, 127.1, 126.9 (CH_{arom}), 100.5 (C-1), 78.7 (C-2/C-7), 77.2 (C-9), 75.7 (C-4), 72.8 (CH_2 Nap), 72.0 (C-11/C-10), 68.7 (C-10/C-11), 67.6 (C-5), 66.4 (C-2/C-7), 55.4 (CH_3 OMe), 32.8 (C-3), 31.2 (C-8), 19.8 (C-12), 13.6 (C-6); HRMS: $[M+Na]^+$ calcd for $C_{24}H_{34}O_8Na$ 473.2151, found 473.2148.

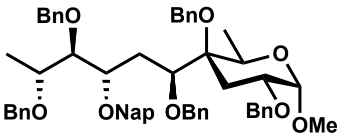

Methyl 2,4,7,10,11-penta-*O*-benzyl-9-*O*-2-methylnaphthalene-α-D-caryophylloside (S32). Compound **S31** (225 mg, 0.5 mmol) was dissolved in DMF (5 ml, 0.1 M) and cooled on ice. NaH (1.0 g, 25.0 mmol, 50.0 eq., 60% dispersion in mineral oil) was added slowly. Consequently, BnBr (3.0 mL, 25.0 mmol, 50.0 eq.) was added and the mixture was stirred for 18 h at 40 °C. Upon full conversion, the reaction mixture was quenched with water and the suspension was diluted with water and Et_2O. The aqueous layer was extracted with Et_2O (3x) followed by washing the combined organic layers with H_2O, sat. aq. $NaHCO_3$ and brine, respectively. Subsequently, the organic layer was dried over $MgSO_4$, filtered, and concentrated *in vacuo* to yield the crude product as a colorless oil. Flash column chromatography (90:10 → 80:20; pentane:EtOAc) yielded the title compound (335 mg, 0.37 mmol, 74%) as a colorless oil. TLC: R_f 0.2 (pentane:EtOAc, 9:1, v:v); $[\alpha]_D^{20}$ –3.0° (c 1.0, $CHCl_3$); IR (neat, cm^{-1}): 1047, 1072, 1095, 1454; ^1H NMR (500 MHz, $CDCl_3$, HH-COSY, HSQC): δ 7.90 – 6.78 (m, 32H, CH_{arom}), 4.88 (d, *J* = 12.3 Hz, 1H, CH*H* Ph), 4.84 (d, *J* = 11.4 Hz, 1H, CH*H* Ph), 4.71 (s, 1H, CH*H* Ph), 4.68 (d, *J* = 3.4 Hz, 1H, H-1), 4.65 (d, *J* = 11.4 Hz, 1H, CH*H* Ph), 4.57 (d, *J* = 12.2 Hz, 1H, CH*H* Ph), 4.53 (m, 2H, CH*H* Ph, CH*H* Ph), 4.50 (m, 1H, CH*H* Ph), 4.46 (m, 1H, CH*H* Ph), 4.43 (m, 1H, CH*H* Ph), 4.18 (d, *J* = 11.5 Hz, 1H, CH*H* Ph), 4.07 – 3.96 (m, 3H, H-5, H-9, CH*H* Ph), 3.81 – 3.72 (m, 3H, H-2, H-10, CH*H* Ph), 3.56 (d, *J* = 9.5 Hz, 1H, H-7), 3.43 (dd, *J* = 7.6, 6.2 Hz, 1H, H-11) 3.36 (s, 3H, CH_3 OMe), 2.24 – 2.09 (m, 3H, H-3, H-8), 1.65 (dd, *J* = 13.9, 9.9 Hz, 1H, H-8), 1.29 (d, *J* = 6.4 Hz, 3H, H-12), 1.26 (d, *J* = 8.5 Hz, 3H, H-6); ^{13}C NMR (126 MHz, $CDCl_3$, HSQC): δ 139.5, 138.9, 138.8, 138.5, 138.4, 136.2, 133.4, 133.1 ($C_{q\text{-}arom}$), 128.7, 128.5, 128.4, 128.4, 128.3, 128.2, 128.2, 128.2, 128.1, 127.9, 127.9, 127.8, 127.7, 127.5, 127.3, 127.1, 127.0, 126.8, 126.6, 126.4, 126.4, 126.2 (CH_{arom}), 97.4 (C-1), 82.0 (C-2), 80.3 (C-4), 79.1 (C-7), 76.8 (C-9), 74.9 (C-11), 74.2, 74.0 (CH_2 Bn), 72.0 (C-10), 71.3, 71.0, 70.7 (CH_2 Bn), 68.0 (C-5), 65.4 (CH_2 Bn), 55.1 (CH_3 OMe), 32.4 (C-8), 27.9 (C-3), 16.9 (C-12), 15.3 (C-6); HRMS: $[M+Na]^+$ calcd for $C_{59}H_{64}O_8Na$ 923.4499, found 923.4507.

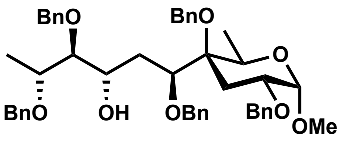

Methyl 2,4,7,10,11-penta-*O*-benzyl-α-D-caryophylloside (S33). Compound **S32** (15.3 mg, 17 µmol) was dissolved in 4:1 DCM:MeOH (340 µL, 0.05 mL) and the solution was cooled on ice. Subsequently DDQ (7.7 mg, 34 µmol, 2.0 eq.) was added. The mixture was stirred for 3 h at room temperature, and upon full conversion, diluted with H_2O and EtOAc. The aqueous layer was extracted (3x) with EtOAc followed by washing the combined organic layer with H_2O, sat. aq. $NaHCO_3$ and brine, respectively. Subsequently, the organic layer was dried over $MgSO_4$, filtered, and concentrated *in vacuo* to yield the crude product as a colorless oil. Flash column chromatography (80:20 → 70:30; pentane:Et_2O) yielded the title compound (108 mg, 102 µmol, 85%) as a colorless oil. TLC: R_f 0.5 (pentane:EtOAc, 9:1, v:v); IR (neat, cm^{-1}): 696, 735, 1047, 1071, 1092, 1453; ^1H NMR (500 MHz, $CDCl_3$, HH-COSY, HSQC): δ 7.39 – 7.15 (m, 25H, CH_{arom}), 4.71 (d, *J* = 3.6 Hz, 1H, H-1), 4.68 (d, *J* = 11.5 Hz, 1H, CH*H* Ph), 4.57 (m,

7H, CH*H* Ph, CH*H* Ph, CH*H* Ph, CH*H* Ph, C*H*H Ph, C*H*H Ph, C*H*H Ph), 4.50 (d, *J* = 11.6 Hz, 1H, C*H*H Ph), 4.37 (d, *J* = 11.6 Hz, 1H, C*H*H Ph), 4.10 (q, *J* = 6.5 Hz, 1H, H-5), 3.94 (dt, *J* = 9.3, 4.7 Hz, 1H, H-9), 3.79 (ddd, *J* = 10.3, 7.0, 3.6 Hz, 1H, H-2), 3.76 – 3.71 (m, 1H, H-7), 3.68 (p, *J* = 6.1 Hz, 1H, H-11), 3.40 (s, 3H, CH_3 OMe), 3.34 (t, *J* = 5.6 Hz, 1H, H-10), 2.64 (s, 1H, 9-OH), 2.21 – 2.16 (m, 2H, H-3), 1.86 (dt, *J* = 7.4, 3.6 Hz, 2H, H-8), 1.28 (d, *J* = 6.2 Hz, 3H, H-6/H-12), 1.26 (d, *J* = 6.0 Hz, 3H, H-6/H-12); ^{13}C NMR (126 MHz, $CDCl_3$, HSQC): δ 139.1, 138.9, 138.5, 138.4, 138.3 ($C_{q\text{-arom}}$), 128.6, 128.5, 128.4, 128.4, 128.1, 128.0, 128.0, 127.9, 127.8, 127.7, 127.5, 127.4, 127.2, 126.8 (CH_{arom}), 97.3 (C-1), 85.1 (C-10), 80.2 (C-4), 78.8 (C-7), 76.6 (C-11), 74.5, 74.3 (CH_2 Bn), 72.1 (C-2), 71.3, 71.0 (CH_2 Bn), 70.1 (C-9), 67.9 (C-5), 65.9 (CH_2 Bn), 55.1 (CH_3 OMe), 34.2 (C-8), 28.0 (C-3), 16.3 (C-12), 15.1 (C-6); HRMS: $[M+Na]^+$ calcd for $C_{48}H_{56}O_8Na$ 783.3873, found 783.3890.

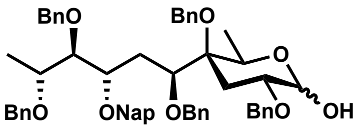

2,4,7,10,11-Penta-*O*-benzyl-9-*O*-2-methylnaphthalene-D-caryophylloside (S34). Compound **S32** (335 mg, 0.37 mmol) was dissolved in formic acid (6.5 mL, 0.05 M, 80% in water) and dioxane (6.5 mL, 0.05 M). $SrCl_2 \cdot 6H_2O$ (88 mg, 0.33 mmol, 1.0 eq) was added and the solution was stirred for 40 h at 60 °C and 250 mbar. The solution was diluted with water and EtOAc, the aqueous layer was extracted with EtOAc (3x) followed by washing the combined organic layers with H_2O, sat. aq. $NaHCO_3$ and brine, respectively. Subsequently, the organic layer was dried over $MgSO_4$, filtered, and concentrated *in vacuo* to yield the crude product as a colorless oil. Flash column chromatography (90:10 → 80:20; pentane:EtOAc) yielded the title compound (199 mg, 0.22 mmol, 68%, α:β; 47:53) as a colorless oil. Starting material was recovered (33.3 mg, 0.037 mmol, 11%) which resulted in a 79% yield based on recovered starting material. TLC: R_f 0.2 (pentane:EtOAc, 8:2, v:v); IR (neat, cm^{-1}): 696, 734, 1028, 1072, 1093; Data of the major stereoisomer (β-anomer): ^{1}H NMR (400 MHz, $CDCl_3$, HH-COSY, HSQC): δ 7.86 – 6.76 (m, 32H), 4.91 – 4.81 (m, 3H, CH*H* Ph, CH*H* Ph, CH*H* Ph), 4.75 – 4.57 (m, 4H, CH*H* Ph, C*H*H Ph, C*H*H Ph, H-1), 4.54 – 4.43 (m, 2H, C*H*H Ph, C*H*H Ph), 4.29 (q, *J* = 6.4 Hz, 1H, H-5), 4.22 (d, *J* = 10.1 Hz, 1H, C*H*H Ph), 4.19 (d, *J* = 10.2 Hz, 1H, C*H*H Ph), 4.06 (dt, *J* = 11.0, 2.5 Hz, 1H, H-9), 3.95 (d, *J* = 12.0 Hz, 1H, CH*H* Ph), 3.89 – 3.71 (m, 1H, H-10), 3.65 – 3.53 (m, 2H, H-2, H-7), 3.46 (ddd, *J* = 10.3, 7.7, 6.1 Hz, 1H, H-11), 3.17 (d, *J* = 5.1 Hz, 1H, 1-OH), 2.35 (dd, *J* = 14.6, 5.4 Hz, 1H, H-3), 2.29 – 2.18 (m, 2H, H-3, H-8), 1.90 (dd, *J* = 14.5, 11.7 Hz, 1H, H-3), 1.70 – 1.58 (m, 1H, H-8), 1.34 – 1.28 (m, 6H, H-6, H-12); ^{13}C NMR (126 MHz, $CDCl_3$, HSQC): δ 139.4, 138.7, 138.6, 138.4, 138.3, 138.0, 136.1, 133.3 ($C_{q\text{-arom}}$), 128.6, 128.5, 128.4, 128.4, 128.4, 128.3, 128.2, 128.2, 128.2, 128.1, 128.1, 128.1, 128.1, 128.0, 128.0, 128.0, 127.8, 127.8, 127.7, 127.7, 127.7, 127.6, 127.5, 127.5, 127.2, 127.1, 127.1, 126.8, 126.8, 126.5, 126.1, 126.1 (CH_{arom}), 99.0 (C-1), 81.9 (C-10), 79.9 (C-4), 78.3 (C-7), 76.5 (C-9), 76.1 (C-2), 74.7 (C-11), 74.2, 74.1, 72.5, 71.0, 70.8 (CH_2 Bn), 68.2 (C-3), 66.5 (CH_2 Bn), 32.6 (C-3), 31.9 (C-8), 16.7 (C-6/C-12), 15.3 (C-6/C-12); Diagnostic signals of the minor stereoisomer (α-isomer): ^{1}H NMR (400 MHz, $CDCl_3$, HH-COSY, HSQC): δ 5.27 (d, *J* = 3.0 Hz, 1H, H-1), 2.85 (s, 1H, 1-OH), 1.90 (dd, *J* = 14.5, 11.7 Hz, 1H, H-3); ^{13}C NMR (126 MHz, $CDCl_3$, HSQC): δ 90.3 (C-1), 81.9 (C-10), 79.8 (C-4), 78.8 (C-7), 76.7 (C-9), 76.0 (C-5), 74.8 (C-11), 73.9, 73.6 (CH_2 Bn), 72.2 (C-2), 70.9, 70.7, 70.5, 65.5 (CH_2 Bn), 32.3 (C-8), 27.3 (C-3), 16.8 (C-12), 15.4 (C-6); HRMS: $[M+Na]^+$ calcd for $C_{58}H_{62}O_8Na$ 909.4342, found 909.4354.

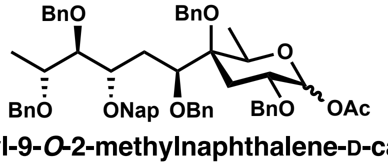

Acetyl 2,4,7,10,11-penta-*O*-benzyl-9-*O*-2-methylnaphthalene-D-caryophylloside (S35). Compound **S34** (44.6 mg, 50 µmol) was dissolved in pyridine (0.5 mL, 0.1 M) and cooled on ice. Ac_2O (15.3 µL, 150 µmol, 3.0 eq.) was added and the reaction was stirred for 18 h and subsequently quenched with water. The mixture was diluted with EtOAc, the aqueous layer was extracted with EtOAc (3x) followed by washing the combined organic layers with H_2O, sat. aq. $NaHCO_3$ and brine, respectively.

Subsequently, the organic layer was dried over $MgSO_4$, filtered, and concentrated *in vacuo* to yield the crude product as a colorless oil. Flash column chromatography (95:5 → 90:10; pentane:EtOAc) yielded the title compound (38.8 mg, 42.3 µmol, 85%, α:β; 20:80) as a colorless oil. TLC: R_f 0.6 (pentane:EtOAc, 8:2, v:v); IR (neat, cm^{-1}): 696, 734, 1053, 1095, 1751; Data of the major stereoisomer (β-anomer): ^{1}H NMR (500 MHz, $CDCl_3$, HH-COSY, HSQC): δ 7.94 – 6.74 (m, 32H,CH_{arom}), 5.55 (d, *J* = 8.1 Hz, 1H, H-1), 4.84 (m, 2H, CH*H* Ph, CH*H* Ph), 4.69 – 4.43 (m, 7H, C*H*H Ph, C*H*H Ph, C*H*H Ph, C*H*H Ph, CH*H* Ph, CH*H* Ph, CH*H* Ph), 4.22 (d, *J* = 11.6 Hz, 1H, C*H*H Ph), 4.05 (dt, *J* = 10.9, 1.8 Hz, 1H, H-9), 4.00 (d, *J* = 11.8 Hz, 1H, CH*H* Ph), 3.95 (q, *J* = 6.6 Hz, 1H, H-5), 3.83 (d, *J* = 11.8 Hz, 1H, C*H*H Ph), 3.77 – 3.70 (m, 2H, H-2, H-10), 3.60 (dd, *J* = 9.7, 1.2 Hz, 1H, H-7), 3.47 (dd, *J* = 7.8, 6.0 Hz, 1H, H-11), 2.40 (dd, *J* = 14.5, 5.4 Hz, 1H, H-3), 2.26 – 2.19 (m, 1H, H-8), 2.11 (s, 3H, $COCH_3$), 1.91 (dd, *J* = 14.5, 11.6 Hz, 1H, H-3), 1.62 (ddd, *J* = 14.9, 9.7, 1.8 Hz, 1H, H-8), 1.33 (d, *J* = 6.5 Hz, 3H, H-6), 1.30 (d, *J* = 6.0 Hz, 3H, H-12); ^{13}C NMR (126 MHz, $CDCl_3$, HSQC): δ 169.7(*C*OCH_3), 139.3, 138.7, 138.5, 138.4, 136.1, 133.4, 133.1 ($C_{q\text{-}arom}$), 128.6, 128.6, 128.5, 128.4, 128.4, 128.3, 128.3, 128.3, 128.2, 128.2, 128.1, 128.0, 127.9, 127.9, 127.8, 127.8, 127.7, 127.6, 127.6, 127.5, 127.4, 127.2, 127.2, 127.1, 127.1, 126.9, 126.8, 126.7, 126.4, 126.4, 126.3, 126.2 (CH_{arom}), 96.3 (C-1), 82.0 (C-10), 79.8 (C-4), 78.3 (C-7), 76.9 (C-5), 76.6 (C-9), 74.7 (C-11), 74.2, 73.7 (CH_2 Bn), 73.6 (C-2), 72.6, 70.9, 70.6, 66.4 (CH_2 Bn), 33.0 (C-3), 32.1 (C-8), 21.4 (CO*C*H_3), 16.8 (C-12), 15.3 (C-6); Diagnostic signals of the minor stereoisomer (α-isomer): ^{1}H NMR (500 MHz, $CDCl_3$, HH-COSY, HSQC): δ 6.31 (d, *J* = 3.4 Hz, 1H, H-1), 4.12 (q, *J* = 7.1 Hz, 1H, H-5), 2.04 (s, 3H, $COCH_3$); ^{13}C NMR (126 MHz, $CDCl_3$, HSQC): δ 170.0 (*C*OCH_3), 89.6 (C-1), 79.8 (C-4), 32.1 (C-3), 28.3 (C-8), 21.2 (CO*C*H_3), 16.9 (C-12), 15.4 (C-6); HRMS: $[M+Na]^+$ calcd for $C_{60}H_{64}O_9Na$ 951.4448, found 951.4462.

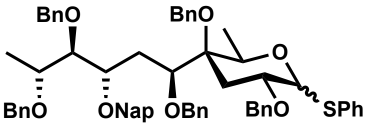

Thiophenol 2,4,7,10,11-penta-*O*-benzyl-9-*O*-2-methylnaphthalene-D-caryophylloside (24). Compound **S35** (38.8 mg, 42.3 µmol) was dissolved in DCM (0.43 mL, 0.1 M), thiophenol (4.8 µL, 47 µmol, 1.1 eq.) was added and subsequently cooled to –80 °C. $BF_3{\cdot}OEt_2$ (6.2 µL, 50.8 µmol, 1.2 eq.) was added and the solution was allowed to attain 0 °C. Upon full conversion, the solution was quenched by adding sat. aq. $NaHCO_3$. The solution was diluted with EtOAc, the aqueous layer was extracted with EtOAc (3x) followed by washing the combined organic layers with H_2O, sat. aq. $NaHCO_3$ and brine, respectively. Subsequently, the organic layer was dried over $MgSO_4$, filtered, and concentrated *in vacuo* to yield the crude product as a colorless oil. Flash column chromatography (95:5 → 80:10; pentane:EtOAc) yielded the title compound (30.1 mg, 32.0 µmol, 61%, α:β; 65:35) as a colorless oil. TLC: R_f 0.7 (pentane:EtOAc, 8:2, v:v); IR (neat, cm^{-1}): 696, 734, 1028, 1072, 1091; Data of the major stereoisomer (α-anomer): ^{1}H NMR (400 MHz, $CDCl_3$, HH-COSY, HSQC): δ 7.83 – 6.65 (m, 37H, CH_{arom}), 5.69 (d, *J* = 5.0 Hz, 1H, H-1), 4.84 – 4.74 (m, 3H, CH*H* Ph, CH*H* Ph, C*H*H Ph), 4.63 – 4.35 (m, 5H, CH*H* Ph, CH*H* Ph, CH*H* Ph, C*H*H Ph, C*H*H Ph), 4.16 – 4.10 (m, 1H, H-2), 4.05 (m, 1H, C*H*H Ph), 4.00 – 3.94 (m, 2H, H-9, CH*H* Ph), 3.77 – 3.65 (m, 3H, H-10, C*H*H Ph, C*H*H Ph), 3.54 (d, *J* = 9.1 Hz, 1H, H-7), 3.43 – 3.33 (m, 1H, H-11), 2.24 – 2.11 (m, 2H, H-3, H-8), 1.99 (dd, *J* = 14.2, 12.2 Hz, 1H, H-3), 1.60 (dd, *J* = 13.7, 9.5 Hz, 1H, H-8), 1.24 (d, *J* = 4.5 Hz, 3H, H-6/H-12), 1.21 (d, *J* = 6.0 Hz, 3H, H-6/H-12); ^{13}C NMR (101 MHz, $CDCl_3$, HSQC): δ 139.3, 138.8, 138.7, 138.4, 138.0, 136.1, 135.3, 133.3, 133.1 ($C_{q\text{-}arom}$), 132.1, 131.3, 128.9, 128.8, 128.6, 128.5, 128.4, 128.3, 128.3, 128.3, 128.2, 128.1, 128.1, 128.0, 127.8, 127.8, 127.7, 127.6, 127.5, 127.4, 127.2, 127.1, 127.1, 126.9, 126.8, 126.7, 126.6, 126.4, 126.4, 126.2 (CH_{arom}), 87.6 (C-1), 82.0 (C-10), 80.0 (C-4), 78.7 (C-7), 76.8 (C-9), 74.9 (C-11), 74.2, 73.9 (CH_2 Bn), 71.9 (C-2), 71.0, 70.9, 70.7 (CH_2 Bn), 69.4 (C-5), 65.4 (CH_2 Bn), 32.4 (C-8), 30.4 (C-3), 16.9 (C-6/C-12), 15.3 (C-6/C-12); Diagnostic signals of the minor stereoisomer (β-isomer): ^{1}H NMR (400 MHz, $CDCl_3$, HH-COSY, HSQC): δ 3.86 (d, *J* = 11.9 Hz, 1H, CH*H* Ph), 3.61 (td, *J* = 10.9, 5.3 Hz, 1H, H-2), 3.50 (d, *J* = 9.2 Hz, 1H, H-7), 2.32 (dd, *J* = 14.4, 5.4 Hz, 1H, H-3), 1.84 (dd, *J* = 14.4, 11.1 Hz, 1H, H-3), 1.52 (dd, *J* = 13.2, 9.7 Hz, 1H, H-8), 1.28 (d, *J* = 6.5 Hz, 3H, H-6/H-12), 1.24 (d, *J* = 6.1 Hz, 3H, H-6/H-12); ^{13}C NMR (101 MHz, $CDCl_3$, HSQC): δ 88.4 (C-1), 82.0 (C-10), 79.7 (C-4), 79.1 (C-7), 78.5 (C-9), 76.5 (C-11), 74.6 (C-2), 74.2, 73.7, 73.0, 72.3 (CH_2 Bn), 70.7 (C-5), 66.4 (CH_2 Bn), 34.3 (C-8),

31.8 (C-3), 16.8 (C-12), 15.7 (C-6); HRMS: $[M+Na]^+$ calcd for $C_{64}H_{66}O_7SNa$ 1001.4427, found 1001.4418.

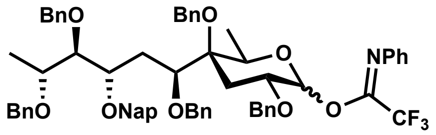

2,2,2-Trifluoro-*N*-phenylacetimido-yl 2,4,7,10,11-penta-*O*-benzyl-9-*O*-2-methylnaphthalene-D-caryophylloside (S36). Compound **S34** (105 mg, 0.12 mmol) was dissolved in acetone (1.2 mL, 0.1 M) and cooled on ice. Subsequently, $CsCO_3$ (40.1 mg, 0.12 mmol, 1.1 eq.) and 2,2,2-trifluoro-*N*-phenylacetimido-yl chloride (37.7 μL, 0.24 mmol, 2.0 eq.) were added and the solution was allowed to attain room temperature. After stirring for 18 h, the solution was diluted with H_2O and EtOAc. The aqueous layer was extracted (3x) with EtOAc followed by washing the combined organic layer with H_2O, sat. aq. $NaHCO_3$ and brine, respectively. Subsequently, the organic layer was dried over $MgSO_4$, filtered, and concentrated *in vacuo* to yield the crude product as a colorless oil. Flash column chromatography (95:5 → 80:20; pentane:Et_2O) yielded the title compound (10.3 mg, 13.5 μmol, 80%, α:β; 28:72) as a colorless oil. TLC: R_f 0.2 (pentane:EtOAc, 8:2, v:v); IR (neat, cm^{-1}): 695, 734, 1027, 1093, 1207, 1453, 1717; Data of the major stereoisomer (β-anomer): ^1H NMR (400 MHz, toluene-d_8, HH-COSY, HSQC, T = 333 K): δ 7.94 – 6.64 (m, 37H, CH_{arom}), 4.89 – 4.74 (m, 4H, CH*H* Ph, CH*H* Ph, C*H*H Ph, C*H*H Ph), 5.64 (d, J = 7.5 Hz, 1H), 4.59 (s, 6H, CH*H* Ph, CH*H* Ph, CH*H* Ph, C*H*H Ph, C*H*H Ph, C*H*H Ph), 4.26 (m, 2H, CH*H* Ph, C*H*H Ph), 4.12 – 3.92 (m, 2H, H-5, H-9), 3.78 (dt, J = 12.1, 6.6 Hz, 1H, H-2), 3.71 (dd, J = 6.9, 2.0 Hz, 1H, H-10), 3.58 (d, J = 9.2 Hz, 1H, H-7), 3.55 – 3.48 (m, 1H, H-11), 2.32 (dd, J = 14.6, 5.5 Hz, 1H, H-3), 2.18 (dd, J = 10.2, 5.0 Hz, 1H, H-8), 1.86 (dd, J = 14.7, 11.4 Hz, 1H, H-3), 1.62 (dd, J = 14.9, 9.4 Hz, 1H, H-8), 1.37 – 1.20 (m, 6H, H-6, H-12); ^{13}C NMR (101 MHz, toluene-d_8, T = 333 K): δ 137.8 (C=N), 129.0 (t, J = 23.7 Hz, CF_3), 128.8, 128.7, 128.6, 128.5, 128.0, 127.8, 127.6, 126.9, 126.7, 126.5, 126.4, 126.3, 125.9, 125.7, 124.6, 124.4, 120.3 (CH_{arom}), 101.1 (C-1), 84.0 (C-10), 80.0 (C-4), 78.4 (C-7), 77.6 (C-11), 76.0, 74.8 (CH_2 Bn), 74.6 (C-2), 74.5, 73.1 (CH_2 Bn), 72.6 C-9), 71.8 (C-5), 71.4, 67.4 (CH_2 Bn), 33.6 (C-3/C-8), 33.5 (C-3/C-8), 16.8 (C-12), 15.7 (C-6); Diagnostic signals of the minor stereoisomer (α-isomer): ^1H NMR (400 MHz, toluene-d_8, HH-COSY, HSQC, T = 333 K): δ 6.53 (d, J = 3.4 Hz, 1H, H-1), 1.94 (dd, J = 14.9, 9.2 Hz, 1H, H-8); ^{13}C NMR (101 MHz, toluene-d_8, T = 333 K): δ 125.43 (t, J = 24.2 Hz, CF_3), 94.9 (C-1).

Synthesis of compound 25

Scheme S5. Synthesis of compound **25**.

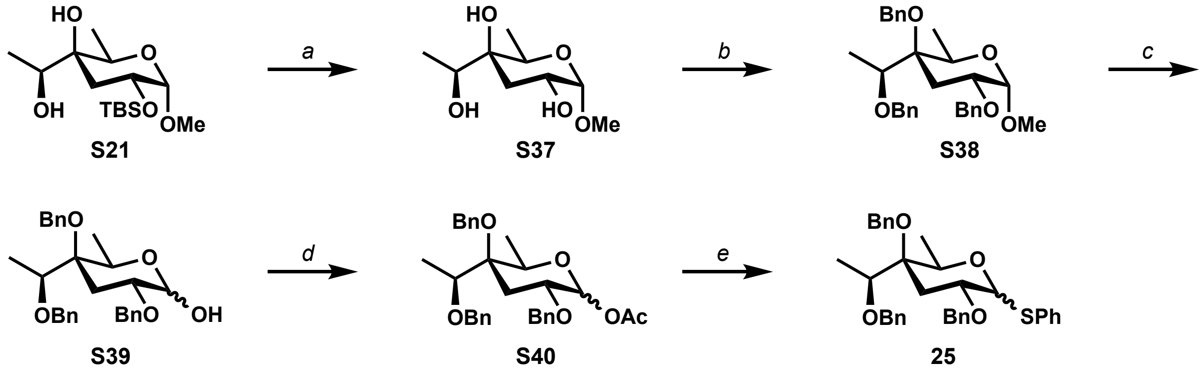

Reagents and conditions: a) HCl, MeOH (*quant.*); b) BnBr, NaH, DMF (53%); c) Ac_2O, H_2SO_4 (88%); d) PhSH, $BF_3{\cdot}OEt_2$, DCM (77%).

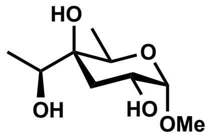

Methyl α-D-yersinioside (S37). Compound **S21** (1.0 g, 3.1 mmol) was dissolved in MeOH (62 mL, 0.05 M). 6 M aq. HCl was added (5.2 mL, 31 mmol, 10 eq.) and the mixture was stirred at room temperature for 3 h. Upon full conversion, the reaction was quenched with basic Amberlite IRA-67 resin (Sigma Aldrich Amberlite IRA-67 free base, pre-washed with MeOH). After filtration, the mixture was concentrated *in vacuo*. Purification by flash column chromatography (80:20 → 60:40; pentane:EtOAc) afforded the title compound (639 mg, 3.1 mmol, *quant.*) as a colorless oil. TLC: R_f 0.7 (acetone); $[\alpha]_D^{20}$ 74.9° (c 1.0, MeOH); IR (neat, cm^{-1}): 1035, 2939, 3383; ^1H NMR (400 MHz, $CDCl_3$, HH-COSY, HSQC): δ 4.67 (d, *J* = 3.9 Hz, 1H, H-1), 4.05 (q, *J* = 6.6 Hz, 1H, H-5), 3.92 (dddd, *J* = 10.9, 9.5, 6.7, 4.7 Hz, 1H, H-2), 3.69 (qd, *J* = 6.6, 3.6 Hz, 1H, H-7), 3.45 (s, 3H, CH_3 OMe), 2.24 (s, 1H, OH), 1.95 (d, *J* = 3.7 Hz, 1H, OH), 1.90 (d, *J* = 10.8 Hz, 1H, OH), 1.83 (ddd, *J* = 12.5, 5.6, 0.8 Hz, 1H, H-3), 1.71 (dd, *J* = 12.6, 11.7 Hz, 1H, H-3), 1.21 (m, 6H, H-6, H-8); ^{13}C NMR (101 MHz, $CDCl_3$, HSQC): δ 98.6 (C-1), 74.5 (C-7), 71.5 (C-3), 66.1 (C-5), 65.7 (C-2), 55.4 (CH_3 OMe), 34.7 (C-3), 17.1 (C-8), 14.0 (C-6); HRMS: $[M+Na]^+$ calcd for $C_9H_{18}O_5Na$ 229.1046, found 229.1049.

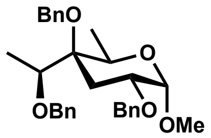

Methyl 2,4,7-tri-*O*-benzyl-α-D-yersinioside (S38). Glycoside **S37** (190 mg, 920 µmol) was dissolved in DMF (9.2 mL, 0.1 M) and BnBr (4.4 mL, 36.8 mmol, 40 eq.) and NaH (1.5 g, 36.8 mmol, 40 eq., 60% dispersion in mineral oil) were added at 0 °C. The mixture was stirred for 96 h at 40 °C and the reaction was quenched with H_2O at 0 °C. The organic phase was washed with sat. aq. $NaHCO_3$ and brine respectively, dried over $MgSO_4$, filtered, and concentrated *in vacuo*. Flash column chromatography (95:5 → 80:20; pentane:EtOAc) yielded the title compound (233 mg, 920 µmol, 53%) as a colorless oil. TLC: R_f 0.7 (pentane:EtOAc, 9:1, v:v); $[\alpha]_D^{20}$ 47.8° (c 1.0, $CHCl_3$); IR (neat, cm^{-1}): 734, 1046, 1454, 2936; ^1H NMR (400 MHz, $CDCl_3$, HH-COSY, HSQC): δ 7.38 – 7.22 (m, 15H, CH_{arom}), 4.72 – 4.53 (m, 5H, CH_2 Bn, CH_2 Bn, H-1), 4.36 (dd, *J* = 23.6, 11.6 Hz, 2H, CH_2 Bn), 4.14 (q, *J* = 6.5 Hz, 1H, H-7), 3.79 (ddd, *J* = 12.0, 5.0, 3.6 Hz, 1H, H-2), 3.53 (q, *J* = 6.3 Hz, 1H, H-5), 3.42 (s, 3H, CH_3 OMe), 2.22 – 2.14 (m, 1H, H-3), 2.07 (dd, *J* = 13.9, 12.0 Hz, 1H, H-3), 1.25 (d, *J* = 6.4 Hz, 3H, H-8), 1.21 (d, *J* = 6.6 Hz, 3H, H-6); ^{13}C NMR (101 MHz, $CDCl_3$, HSQC): δ 139.3, 138.6, 138.3 ($C_{q\text{-}arom}$), 128.6, 128.4, 128.3, 128.2, 128.1, 127.8, 127.7, 127.4, 127.3, 127.0, 127.0, 126.9 (CH_{arom}), 97.2 (C-1), 79.2 (C-4), 76.8 (C-7), 72.0 (C-2), 71.2, 71.0 (CH_2 Bn), 67.4 (C-5), 65.0 (CH_2 Bn), 55.0 (CH_3 OMe), 27.5 (C-3), 14.7 (C-8), 14.3 (C-6); HRMS: $[M+Na]^+$ calcd for $C_{30}H_{36}O_5Na$ 499.2460, found 499.2459.

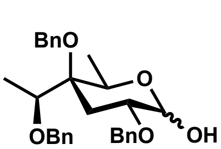

2,4,7-Tri-*O*-benzyl-D-yersinioside (S39). Compound **S38** (100 mg, 210 µmol) was dissolved in 80% aq. HCOOH (2.1 mL, 0.1 M) and $SrCl_2{\cdot}6H_2O$ (6.7 mg, 42 µmol, 0.2 eq.) was added. The mixture was stirred for 24 h at 40 °C, and the reaction was quenched with H_2O at 0 °C. The organic phase was washed with sat. aq. $NaHCO_3$ and brine respectively, dried over $MgSO_4$, filtered, and concentrated *in vacuo*. Flash column chromatography (90:10 → 70:30; pentane:EtOAc) yielded the title compound (43 mg, 94 µmol, 47%, α:β; 34:66) as a colorless oil. TLC: R_f 0.2 (pentane:EtOAc, 8:2, v:v); IR (neat, cm^{-1}): 696, 734, 1027, 1074, 1453, 3399; Data of the major stereoisomer (β-anomer): ^1H NMR (400 MHz, $CDCl_3$, HH-COSY, HSQC): δ 7.42 – 7.18 (m, 15H, CH_{arom}), 4.79 (d, *J* = 11.7 Hz, 2H, CH_2 Bn), 4.75 – 4.53 (m, 5H, CH_2 Bn, CH_2 Bn, H-1), 4.11 (q, *J* = 6.5 Hz, 1H, H-7), 3.68 – 3.60 (m, 1H, H-2), 3.48 (q, *J* = 6.1 Hz, 1H, H-5), 2.95 (d, *J* = 5.1 Hz, 1H, 1-OH), 2.35 (dd, *J* = 14.7, 5.7 Hz, 1H, H-3), 1.96 (dd, *J* = 14.6, 11.6 Hz, 1H, H-3), 1.24 (d, *J* = 6.2 Hz, 3H, H-8), 1.18 (d, *J* = 6.4 Hz, 3H, H-6); ^{13}C NMR (101 MHz,

$CDCl_3$, HSQC): δ 139.3, 138.6, 138.3 ($C_{q\text{-}arom}$), 128.5, 128.4, 128.3, 128.2, 128.1, 127.8, 127.7, 127.4, 127.3, 127.0, 127.0, 126.9 (CH_{arom}), 98.9 (C-1), 79.1 (C-4), 76.3 (C-2), 76.1 (C-7), 75.7 (C-5), 72.4, 72.2, 71.0 (CH_2 Bn), 31.9 (C-3), 14.6 (C-6), 14.3 (C-8); Diagnostic signals of the minor stereoisomer (α-anomer): ^{1}H NMR (400 MHz, $CDCl_3$, HH-COSY, HSQC): δ 5.29 (d, *J* = 3.6 Hz, 1H, H-1), 4.49 (d, *J* = 11.4 Hz, 1H, C*H*H Bn), 4.43 (q, *J* = 6.5 Hz, 1H, H-5), 4.33 (t, *J* = 12.2 Hz, 2H, CH_2 Bn), 3.84 (ddd, *J* = 11.4, 5.3, 3.6 Hz, 1H, H-2), 2.89 (s, 1H, 1-OH), 2.21 (dd, *J* = 13.9, 5.1 Hz, 1H, H-3), 2.16 – 2.02 (m, 1H, H-3), 1.27 (d, *J* = 6.3 Hz, 3H, H-6), 1.21 (d, *J* = 6.6 Hz, 3H, H-8); ^{13}C NMR (101 MHz, $CDCl_3$, HSQC): δ 128.5, 127.6, 127.4, 127.2, 126.9 (CH_{arom}), 90.1 (C-1), 70.7, 67.0, 65.6 (CH_2 Bn), 26.8 (C-3), 13.9 (C-8), 13.4 (C-6); HRMS: $[M+Na]^+$ calcd for $C_{29}H_{34}O_5Na$ 485.2298, found 485.2299.

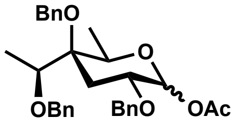

Acetyl 2,4,7-tri-*O*-benzyl-D-yersinioside (S40). Compound **S39** (69 mg, 150 µmol) was dissolved in pyridine (0.4 mL, 0.4 M) and Ac_2O (45 µL, 450 µmol, 3.0 eq.) was added at 0 °C. The reaction mixture was stirred for 24 h and quenched with sat. aq. $NaHCO_3$ upon full conversion. The organic phase was washed with sat. aq. $NaHCO_3$ and brine, respectively. The organic layer was dried over $MgSO_4$, filtered, and concentrated *in vacuo*. Flash column chromatography (90:10; pentane:EtOAc) yielded the title compound **74** (66 mg, 130 µmol, 87%, α:β; 24:76) as a colorless oil. TLC: R_f 0.5 (pentane:EtOAc, 9:1, v:v); IR (neat, cm^{-1}): 696, 734, 1053, 1088, 1228, 1453, 1748; Data of the major stereoisomer (β-anomer): ^{1}H NMR (500 MHz, $CDCl_3$, HH-COSY, HSQC): δ 7.42 – 7.16 (m, 15H, CH_{arom}), 5.61 (d, *J* = 8.1 Hz, 1H, H-1), 4.67 – 4.56 (m, 3H, C*H*H Bn, C*H*H Bn, CH*H* Bn), 4.46 (m, 1H, CH*H* Bn), 4.36 – 4.30 (m, 2H, C*H*H Bn, CH*H* Bn), 4.18 (q, *J* = 6.4 Hz, 1H, H-7), 3.79 (ddd, *J* = 11.5, 8.1, 5.7 Hz, 1H, H-2), 3.48 (q, *J* = 6.2 Hz, 1H, H-5), 2.39 (dd, *J* = 14.6, 5.7 Hz, 1H, H-3), 2.12 (s, 3H, $COCH_3$), 2.00 (dd, *J* = 14.7, 11.5 Hz, 1H, H-3), 1.23 (d, *J* = 6.2 Hz, 3H, H-6), 1.18 (d, *J* = 6.4 Hz, 3H, H-8); ^{13}C NMR (126 MHz, $CDCl_3$, HSQC): δ 169.7 (*C*OCH3), 139.3, 138.4, 138.2 ($C_{q\text{-}arom}$), 128.6, 128.6, 128.5, 128.4, 128.4, 128.0, 127.9, 127.9, 127.9, 127.8, 127.6, 127.4, 127.3, 127.3, 127.1 (CH_{arom}), 96.3 (C-1), 79.0 (C-4), 76.7 (C-7), 76.0 (C-5), 73.9 (C-2), 72.6, 70.9, 66.9 (CH_2 Bn), 32.2 (C-3), 21.4 (CO*C*H3), 14.4 (C-8), 13.6 (C-6); Diagnostic signals of the minor stereoisomer (α-anomer): ^{1}H NMR (500 MHz, $CDCl_3$, HH-COSY, HSQC): δ 6.33 (d, *J* = 3.6 Hz, 1H, H-1), 3.89 (ddd, *J* = 12.1, 5.2, 3.5 Hz, 1H, H-2), 3.56 (q, *J* = 6.3 Hz, 1H, H-5), 2.23 (ddd, *J* = 13.9, 4.6, 0.8 Hz, 1H, H-3), 2.12 (s, 3H, $COCH_3$), 1.28 (d, *J* = 6.3 Hz, 3H, H-6), 1.21 (d, *J* = 6.6 Hz, 3H, H-8);^{13}C NMR (126 MHz, $CDCl_3$, HSQC): δ 170.1 (*C*OCH3), 139.2, 138.6, 138.0 ($C_{q\text{-}arom}$), 89.6 (C-1), 79.0 (C-4), 76.8 (C-7), 71.5, 71.1 (CH_2 Bn), 71.0 (C-2), 70.3 (C-5), 65.7 (CH_2 Bn), 27.8 (C-3), 21.3 (CO*C*H3), 14.7 (C-8), 14.1 (C-6); HRMS: $[M+Na]^+$ calcd for $C_{31}H_{36}O_6Na$ 527.2404, found 527.2410.

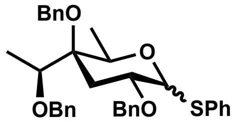

Phenyl 2,4,7-tri-*O*-benzyl-1-thio-D-yersinioside (25). Compound **S40** (66 mg, 130 µmol) was dissolved in DCM (1.3 mL, 0.1 M) and cooled to –80 °C upon which thiophenol (14.6 µL, 143 µmol, 1.1 eq.) and $BF_3 \cdot OEt_2$ (19.3 µL, 156 µmol, 1.2 eq.) were added. The reaction mixture was stirred for 2 h and was allowed to warm to room temperature. The reaction was quenched with sat. aq. $NaHCO_3$ and the organic phase was washed with sat. aq. $NaHCO_3$ and brine, respectively. The organic layer was dried over $MgSO_4$, filtered, and concentrated *in vacuo*. Flash column chromatography (99:1 → 95:5; pentane:EtOAc) yielded the title compound (55 mg, 100 µmol, 77%, α:β; 65:35) as a colorless oil. TLC: R_f 0.7 (pentane:EtOAc, 9.5:0.5, v:v); IR (neat, cm^{-1}): 694, 733, 1026, 1073, 1453; Data of the major stereoisomer (α-anomer): ^{1}H NMR (400 MHz, $CDCl_3$, HH-COSY, HSQC): δ 7.70 – 7.12 (m, 15H), 5.78 (d, *J* = 5.1 Hz, 1H, H-1), 4.75 – 4.61 (m, 3H, CH*H* Ph, C*H*H Ph, H-7), 4.57 (d, *J* = 10.0 Hz, 1H, CH*H* Ph), 4.50 (d, *J* = 10.0 Hz, 1H, C*H*H Ph), 4.47 – 4.40 (m, 1H, CH*H* Ph), 4.36 (d, *J* = 11.9 Hz, 1H, C*H*H Ph), 4.15 (dt, *J* = 11.9, 4.9 Hz, 1H, H-2), 3.60 (q, *J* = 6.2 Hz, 1H, H-5), 2.29 (dd, *J* = 14.3, 4.6 Hz, 1H, H-3),

2.07 (dd, J = 14.4, 11.9 Hz, 1H, H-3), 1.29 (d, J = 6.2 Hz, 3H, H-8), 1.23 – 1.17 (m, 3H, H-6); ^{13}C NMR (101 MHz, $CDCl_3$, HSQC): δ 139.2, 138.5, 137.8, 135.2 ($C_{q\text{-}arom}$), 132.1, 131.3, 128.8, 128.7, 128.5, 128.4, 128.4, 128.3, 128.2, 128.0, 127.9, 127.8, 127.7, 127.6, 127.4, 127.4, 127.2, 127.1, 127.1, 126.9 (CH_{arom}), 87.3 (C-1), 79.0 (C-4), 78.9 (C-7), 72.3 (CH_2 Bn), 72.0 (C-2), 70.9, 70.8 (CH_2 Bn), 69.1 (C-5), 29.7 (C-3), 14.6 (C-8), 14.1 (C-6); Diagnostic signals of the minor stereoisomer (β-anomer): ^{1}H NMR (400 MHz, $CDCl_3$, HH-COSY, HSQC): δ 4.65 (d, J = 8.3 Hz, 1H, H-1), 4.05 (q, J = 6.4 Hz, 1H, H-7), 3.72 (td, J = 10.3, 5.5 Hz, 1H, H-2), 3.45 (q, J = 6.2 Hz, 1H, H-5), 2.37 (dd, J = 14.5, 5.6 Hz, 1H, H-3), 1.97 (dd, J = 14.5, 11.0 Hz, 1H, H-3); ^{13}C NMR (101 MHz, $CDCl_3$, HSQC): δ 88.3 (C-1), 78.9 (C-4), 78.8 (C-7), 76.1 (C-5), 73.2 (C-2), 72.1, 70.7, 66.9 (CH_2 Bn), 33.4 (C-3), 14.7 (C-8), 13.3 (C-6); HRMS: $[M+Na]^+$ calcd for $C_{35}H_{38}O_4SNa$ 577.2383, found 577.2389.

Preparation of the target compounds

Synthesis of compound 1

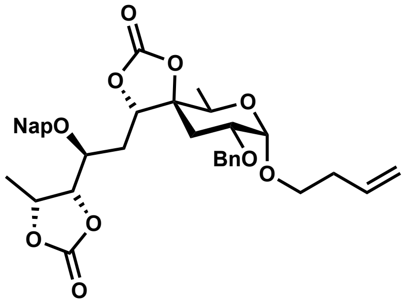

3-Butene 2-*O*-benzyl-4,7,10,11-di-*O*-carbonate-9-*O*-(2-methylnaphthalene)-α-D-caryophylloside (30). Compound **4** (1.68 g, 2.5 mmol) was dissolved in DCM (50 mL, 0.05 M) in a flame dried flask containing activated 3 Å molecular sieves (rods, size 1/16 in., Sigma Aldrich). Ph_2SO (650 mg, 3.25 mmol, 1.3 eq.), ethyl maleimide (625 mg 5.0 mmol, 2 eq) and TTBP (1.55 g, 6.25 mmol, 2.5 eq.) were added. The solution was stirred at room temperature for 30 min. The solution was cooled to –80 °C upon which Tf_2O (550 µL, 3.25 mmol, 1.3 eq.) was added slowly. Subsequently, the solution was allowed to attain to –65 °C to secure full activation of the donor followed by cooling back to –80 °C after which TBAI (7.4 g, 20 mmol, 8 eq.) was added. The solution was stirred for 5 min at –80 °C followed by the addition of the acceptor (5.4 mL, 62.5 mmol, 25 eq.) and triphenylphosphine oxide (4.17 g, 15 mmol, 6.0 eq.). The reaction was refluxed for 40 h upon which the reaction was quenched with sat. aq. $NaHCO_3$ followed by the dilution with EtOAc and sat. aq. $Na_2S_2O_3$. The aqueous layer was extracted three times with EtOAc. The organic layer was washed with H_2O and brine, dried over $MgSO_4$, filtered off and concentrated under reduced pressure. Flash column chromatography (80:20 → 60:40; pentane:EtOAc) yielded the title compound (943 mg, 1.49 mmol, 60%, α:β; >98:2) as a white foam. TLC: R_f 0.6 (pentane:EtOAc, 6:4, v:v); $[\alpha]_D^{20}$ 66.1° (c 0.5, $CHCl_3$); IR (neat, cm^{-1}): 1055, 1202, 1797; ^{1}H NMR (500 MHz, $CDCl_3$, HH-COSY, HSQC, HMBC): δ 7.89 – 7.27 (m, 12H, CH_{arom}), 5.83 (ddt, J = 17.1, 10.3, 6.8 Hz, 1H, H-15), 5.14 (dq, J = 17.2, 1.6 Hz, 1H, H-16), 5.09 (ddt, J = 10.2, 2.1, 1.2 Hz, 1H, H-16), 4.95 – 4.88 (m, 1H, H-11), 4.78 (d, J = 2.0 Hz, 2H, CH_2 Bn/Nap), 4.77 (d, J = 3.3 Hz, 1H, H-1), 4.63 (dd, J = 7.4, 6.4 Hz, 1H, H-10), 4.55 (d, J = 12.1 Hz, 1H, C*H*H Bn/Nap), 4.50 (d, J = 12.0 Hz, 1H, CH*H* Bn/Nap), 4.33 (dd, J = 11.4, 1.9 Hz, 1H, H-7), 4.02 (ddd, J = 8.5, 6.4, 3.1 Hz, 1H, H-9), 3.92 (q, J = 6.3 Hz, 1H, H-5), 3.75 (ddd, J = 11.7, 4.8, 3.3 Hz, 1H, H-2), 3.69 (dt, J = 9.8, 6.9 Hz, 1H, H-13), 3.55 (dt, J = 9.9, 6.4 Hz, 1H, H-13), 2.40 (ttd, J = 8.0, 6.7, 1.4 Hz, 2H, H-14), 2.13 – 2.03 (m, 2H, H-3, H-8), 1.92 (ddd, J = 14.9, 8.6, 2.0 Hz, 1H, H-8), 1.83 (dd, J = 13.5, 4.8 Hz, 1H, H-3), 1.46 (d, J = 6.7 Hz, 3H, H-12), 1.22 (d, J = 6.3 Hz, 3H, H-6); ^{13}C NMR (126 MHz, $CDCl_3$, HSQC, HMBC): δ 153.6, 153.5 (O(C=O)O), 137.9 ($C_{q\text{-}arom}$), 135.2 (C-15), 134.1, 133.3, 133.3 ($C_{q\text{-}arom}$), 128.9, 128.6, 128.1, 128.0, 127.9, 126.8, 126.8, 126.7, 125.4 (CH_{arom}), 117.1 (C-16), 95.5 (C-1), 84.9 (C-4), 81.0 (C-7), 78.9 (C-10), 75.8 (C-11), 73.8 (C-9), 73.7, 71.5 (CH_2 Bn/Nap), 71.5 (C-2), 67.7 (C-13), 64.8 (C-5), 34.0 (C-14), 33.7 (C-3), 29.7 (C-8), 15.3 (C-12), 14.9 (C-6); HRMS: $[M+Na]^+$ calcd for $C_{36}H_{40}O_{10}Na$ 655.2519, found 655.2514.

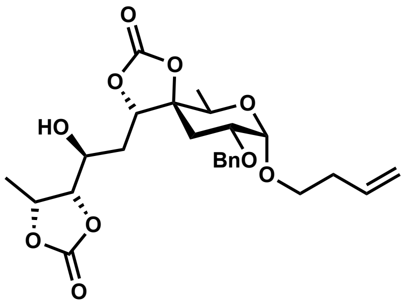

3-Butene 2-*O*-benzyl-4,7,10,11-di-*O*-carbonate-α-D-caryophylloside (31). Compound **30** (943 mg, 1.49 mmol) was divided into 15 equal portions of 0.1 mmol. Compound **30** (0.1 mmol, 63.3 mg, 1.0 eq.) was dissolved in 1:1 (v:v) DCM:HFIP (2.0 mL, 0.05 M) and TES (50 µL, 0.3 mmol, 3.0 eq.) was added. Then 0.5 M solution of HCl in HFIP (3.0 mL, 1.5 mmol, 15 eq.) was added and the reaction mixture was stirred for 2 h. Upon completion, the reaction was quenched with sat. aq. $NaHCO_3$. The aqueous layer was extracted twice with EtOAc followed by washing the combined organic layers with H_2O, sat. aq. $NaHCO_3$ and brine, respectively. Subsequently, the organic layer was dried over $MgSO_4$, filtered, and concentrated *in vacuo* to yield the crude product. Flash column chromatography (70:30 → 40:60; pentane:EtOAc) yielded the title compound (454 mg, 0.92 mmol, 61%) as a white foam. TLC: R_f 0.7 (pentane:EtOAc, 1:1, v:v); $[\alpha]_D^{20}$ 32.1° (c 1.0, $CHCl_3$); IR (neat, cm^{-1}): 1058, 1205, 1357, 1800, 2918, 3477; ^1H NMR (500 MHz, $CDCl_3$, HH-COSY, HSQC, HMBC): δ 7.38 – 7.28 (m, 5H, CH_{arom}), 5.83 (ddt, *J* = 17.1, 10.2, 6.8 Hz, 1H, H-15), 5.18 – 5.05 (m, 2H, H-16, H-16), 4.97 (p, *J* = 6.7 Hz, 1H, H-11), 4.80 (d, *J* = 3.3 Hz, 1H, H-1), 4.59 (d, *J* = 1.5 Hz, 2H, CH_2 Bn), 4.53 (dd, *J* = 11.9, 2.1 Hz, 1H, H-7), 4.40 (dd, *J* = 9.0, 7.3 Hz, 1H, H-10), 4.08 (q, *J* = 8.5 Hz, 1H, H-9), 3.93 (q, *J* = 6.3 Hz, 1H, H-5), 3.81 (ddd, *J* = 11.4, 5.0, 3.3 Hz, 1H, H-2), 3.71 (dt, *J* = 9.8, 6.9 Hz, 1H, H-13), 3.55 (dt, *J* = 9.8, 6.4 Hz, 1H, H-13), 2.99 (d, *J* = 7.0 Hz, 1H, 9-OH), 2.40 (tdt, *J* = 8.7, 7.9, 4.4, 1.3 Hz, 2H, H-14), 2.18 – 1.99 (m, 3H, H-3, H-3, H-8), 1.70 (ddd, *J* = 14.8, 10.5, 2.1 Hz, 1H, H-8), 1.49 (d, *J* = 6.7 Hz, 3H, H-12), 1.24 (d, *J* = 6.3 Hz, 3H, H-6); ^{13}C NMR (126 MHz, $CDCl_3$, HSQC, HMBC): δ 154.0, 153.9 (O(C=O)O), 137.9 ($C_{q\text{-}arom}$), 135.1 (C-15), 128.7, 128.2, 128.0 (CH_{arom}), 117.1 (C-16), 95.5 (C-1), 85.2 (C-4), 80.9 (C-7), 80.0 (C-10), 76.2 (C-11), 71.7 (CH_2 Bn), 71.7 (C-2), 67.7 (C-13), 65.1 (C-5), 64.8 (C-9), 34.1 (C-14), 33.7 (C-3), 32.1 (C-8), 15.0 (C-12), 14.8 (C-6); HRMS: $[M+Na]^+$ calcd for $C_{25}H_{32}O_{10}Na$ 515.1893, found 515.1888.

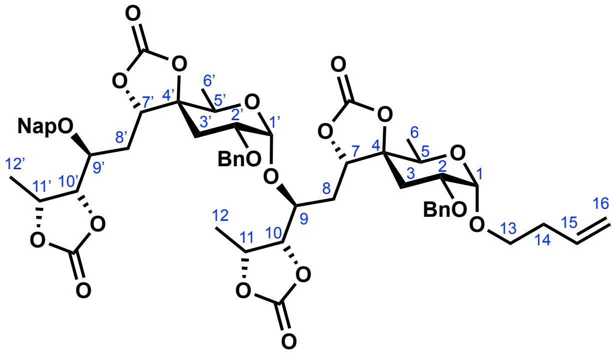

3-Butene 2-*O*-benzyl-4,7,10,11-di-*O*-carbonate-9-*O*-[2-*O*-benzyl-4,7,10,11-di-*O*-carbonate-9-*O*-(2-methylnaphthalene)-α-D-caryophyllosyl]-α-D-caryophylloside (32). Compound **4** (335 mg, 0.5 mmol, 1 eq.) was dissolved in DCM (10 mL, 0.05 M) in a flame dried flask containing activated 3 Å molecular sieves (rods, size 1/16 in., Sigma Aldrich). Ph_2SO (110 mg, 0.55 mmol, 1.1 eq.) and TTBP (310 mg, 1.25 mmol, 2.5 eq.) were added. The solution was stirred at room temperature for 30 min. The solution was cooled to –80 °C upon which Tf_2O (93.5 µL, 0.55 mmol, 1.1 eq.) was added slowly. Subsequently, the solution was allowed to attain to –65 °C to secure full activation of the donor followed by cooling back to –80 °C after which acceptor **31** (2.0 mL of a 0.5 M solution, 2.0 eq.) was added. The reaction was stirred for 20 h at –65 °C upon which the reaction was quenched with sat. aq. $NaHCO_3$ followed by the dilution with DCM. The aqueous layer was extracted three times with EtOAc. The organic layer was washed with H_2O and brine, dried over $MgSO_4$, filtered off and concentrated under reduced pressure. Size exclusion chromatography by isocratic elution with DCM:MeOH (1:1, v:v) followed by

flash column chromatography (80:20 → 70:30; pentane:acetone) yielded the title compound (262 mg, 249 µmol, 50%, α:β; >98:2) as a white foam. TLC: R_f 0.4 (pentane:acetone, 7:3, v:v); $[\alpha]_D^{20}$ 42.6° (c 0.5, $CHCl_3$); IR (neat, cm^{-1}): 754, 1062, 1201, 1802; ^1H NMR (400 MHz, $CDCl_3$, HH-COSY, HSQC, HMBC): δ 7.90 – 7.28 (m, 17H, CH_{arom}), 5.79 (ddt, *J* = 17.1, 10.2, 6.7 Hz, 1H, H-15), 5.14 – 5.04 (m, 2H, H-16, H-16), 4.99 – 4.87 (m, 3H, H-1, H-11, H-11'), 4.85 – 4.75 (m, 3H, C*H*H Bn/Nap, CH*H* Bn/Nap, H-10), 4.71 (d, *J* = 3.3 Hz, 1H, H-1'), 4.70 – 4.58 (m, 2H, H-7, H-10'), 4.53 – 4.32 (m, 5H, C*H*H Bn/Nap, C*H*H Bn/Nap, CH*H* Bn/Nap, CH*H* Bn/Nap, H-7'), 4.10 (td, *J* = 8.8, 3.0 Hz, 1H, H-9'), 4.04 – 3.93 (m, 2H, H-5', H-9), 3.83 – 3.75 (m, 2H, H-2', H-5), 3.65 (dt, *J* = 9.9, 6.9 Hz, 1H, H-13), 3.55 (dt, *J* = 11.8, 4.4 Hz, 1H, H-2), 3.48 (dt, *J* = 9.9, 6.4 Hz, 1H, H-13), 2.35 (q, *J* = 7.7 Hz, 2H, H-14), 2.19 – 1.96 (m, 3H, H-8, H-8', H-8'), 1.95 – 1.78 (m, 5H, H-3, H-3', H-3, H-3', H-8), 1.54 – 1.48 (m, 6H, H-12, H-12'), 1.25 (d, *J* = 6.3 Hz, 3H, H-6'), 1.16 (d, *J* = 6.3 Hz, 3H, H-6); ^{13}C NMR (101 MHz, $CDCl_3$, HSQC, HMBC): δ 153.7, 153.6, 153.4, 153.1 (O(C=O)O), 137.9, 137.1 ($C_{q\text{-}arom}$), 135.0 (C-15), 134.3, 133.2 ($C_{q\text{-}arom}$), 129.0, 128.7, 128.6, 128.4, 128.1, 128.0, 127.9, 127.8, 126.8, 126.7, 126.6, 125.5 (CH_{arom}), 117.0 (C-16), 98.3 (C-1'), 95.3 (C-1), 84.5 (C-4'), 84.0 (C-4), 80.9 (C-7'), 80.2 (C-7), 79.9 (C-10), 79.0 (C-10'), 76.3 (C-9), 75.9 (C-11/C-11'), 75.6 (C11'/C-11), 73.8 (C-9'), 73.8 (CH_2 Bn/Nap), 72.0 (C-2'), 71.9 (CH_2 Bn/Nap), 71.5 (C-2), 71.5 (CH_2 Bn/Nap), 67.7 (C-13), 66.3 (C-5'), 64.7 (C-5), 33.9 (C-14), 32.9 (C-3'/C-3), 32.8 (C-3/C-3'), 29.6 (C-8'), 29.3 (C-8), 15.3 (C-12'/C-12), 15.1 (C-12/C-12'), 14.9 (C-6, C-6'); HRMS: $[M+Na]^+$ calcd for $C_{57}H_{54}O_{19}Na$ 1075.3939, found 1075.3934.

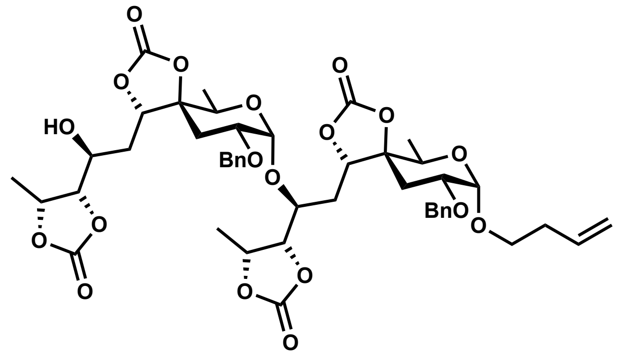

3-Butene 2-*O*-benzyl-4,7,10,11-di-*O*-carbonate-9-*O*-[2-*O*-benzyl-4,7,10,11-di-*O*-carbonate-α-D-caryophyllosyl]-α-D-caryophylloside (33). Compound **32** (84 mg, 80 µmol) was dissolved in 1:1 DCM:HFIP (1.6 mL, 0.05 M) and TES (40 µL, 240 µmol, 3.0 eq.) was added. Then 1.0 M solution of HCl in HFIP (2.4 mL, 2.4 mmol, 30 eq.) was added and the reaction mixture was stirred for 1.5 h. Upon completion the reaction was quenched with sat. aq. $NaHCO_3$. The aqueous layer was extracted twice with EtOAc followed by washing the combined organic layers with H_2O, sat. aq. $NaHCO_3$ and brine, respectively. Subsequently, the organic layer was dried over $MgSO_4$, filtered, and concentrated *in vacuo* to yield the crude product. Flash column chromatography (70:30 → 40:60; pentane:EtOAc) yielded the title compound (44 mg, 48 µmol, 60%) as a white foam. TLC: R_f 0.3 (toluene:EtOAc, 1:1, v:v); $[\alpha]_D^{20}$ –102.8° (c 0.25, $CHCl_3$); IR (neat, cm^{-1}): 753, 1052, 1201, 1368, 1804, 2923; ^1H NMR (500 MHz, $CDCl_3$, HH-COSY, HSQC): δ 7.40 – 7.27 (m, 10H, CH_{arom}), 5.80 (ddt, *J* = 17.1, 10.3, 6.7 Hz, 1H, H-15), 5.14 – 5.04 (m, 2H, H-16, H-16), 5.02 – 4.95 (m, 2H, H-11, H-11'), 4.92 (d, *J* = 3.4 Hz, 1H, H-1'), 4.86 (dd, *J* = 7.5, 3.8 Hz, 1H, H-10), 4.72 (d, *J* = 3.3 Hz, 1H, H-1), 4.63 – 4.55 (m, 3H, H-7, H-7', H-10'), 4.55 – 4.43 (m, 4H, C*H*H Bn, C*H*H Bn, CH*H* Bn, CH*H* Bn), 4.06 – 3.98 (m, 3H, H-5, H-5', H-9), 3.87 (ddd, *J* = 11.8, 4.9, 3.3 Hz, 1H, H-2'), 3.79 (q, *J* = 6.2 Hz, 1H, H-5), 3.66 (dt, *J* = 10.0, 6.9 Hz, 1H, H-13), 3.59 (ddd, *J* = 11.8, 4.8, 3.3 Hz, 1H, H-2), 3.50 (dt, *J* = 10.0, 6.4 Hz, 1H, H-13), 3.08 (d, *J* = 8.4 Hz, 1H, 9'-OH), 2.36 (dddd, *J* = 9.5, 7.8, 5.4, 1.3 Hz, 2H, H-14), 2.22 – 1.82 (m, 8H, H-3, H-3', H-3, H-3', H-8, H-8', H-8, H-8'), 1.56 – 1.48 (m, 6H, H-12, H-12'), 1.27 (d, *J* = 6.3 Hz, 3H, H-6'), 1.15 (d, *J* = 6.3 Hz, 3H, H-6); ^{13}C NMR (126 MHz, $CDCl_3$, HSQC): δ 154.2, 154.1, 153.6, 153.6 (O(C=O)O), 137.9, 137.3 ($C_{q\text{-}arom}$), 135.1 (C-15), 129.1, 129.0, 129.0, 128.7, 128.6, 128.4, 128.2, 128.1, 128.1, 128.0, 127.9, 127.9, 127.9 (CH_{arom}), 117.1 (C-16), 97.4 (C-1'), 95.5 (C-1), 84.8 (C-4'), 84.4 (C-4), 81.2 (C-7'), 80.7 (C-7), 80.0 (C-10), 79.9 (C-10'), 76.4 (C-11, C-11'), 75.9 (C-9), 72.2 (C-2'), 72.1, 71.6 (CH_2 Bn), 71.6 (C-2), 67.8 (C-13), 66.5 (C-9'), 65.2 (C-5'), 64.7 (C-5), 34.0 (C-14), 33.1 (C-3), 33.1, (C-3'), 32.5 (C-8'), 28.8 (C-8), 15.1

(C-12'/C-12), 15.0 (C-12/C-12'), 15.0 (C-6/C-6'), 14.9 (C-6'/C-6); HRMS: [M+Na]$^+$ calcd for $C_{46}H_{56}O_{19}Na$ 935.3313, found 935.3308.

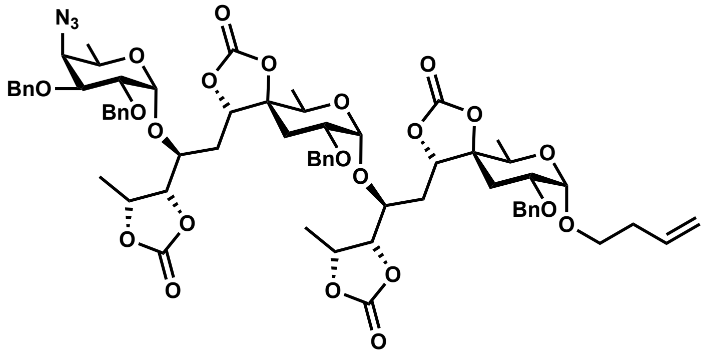

3-Butene 2-*O*-benzyl-4,7,10,11-di-*O*-carbonate-9-*O*-[2-*O*-benzyl-4,7,10,11-di-*O*-carbonate-9-*O*-[4-azido-2,3-di-*O*-benzyl-4,6-dideoxy-α-D-galactopyranosyl]-α-D-caryophyllosyl]-α-D-caryophylloside (34). Compound **3** (150 µmol, 69.2 mg, 3 eq.) was dissolved in DCM (1.0 mL, 0.05 M) in a flame dried flask containing activated 3 Å molecular sieves (rods, size 1/16 in., Sigma Aldrich). Ph_2SO (29 mg, 145 µmol, 2.9 eq.) and TTBP (31 mg, 125 µmol, 2.5 eq.) were added. The solution was stirred at room temperature for 30 min. The solution was cooled to –80 °C upon which Tf_2O (24.5 µL, 145 µmol, 2.9 eq.) was added slowly. Subsequently, the solution was allowed to attain to –65 °C to secure full activation of the donor followed by cooling back to –80 °C after which acceptor **33** (0.1 mL of a 0.5 M solution, 1.0 eq.) was added. The reaction was stirred for 20 h at –65 °C upon which the reaction was quenched with sat. aq. $NaHCO_3$ followed by the dilution with DCM. The aqueous layer was extracted three times with EtOAc. The organic layer was washed with H_2O and brine, dried over $MgSO_4$, filtered off and concentrated under reduced pressure. Size exclusion chromatography by isocratic elution with DCM:MeOH (1:1, v:v) followed by flash column chromatography (70:30 → 50:50; pentane:acetone) yielded the title compound (27.1 mg, 21.4 µmol, 43%, α:β; >98:2) as a white foam. TLC: R_f 0.6 (EtOAc:toluene, 1:1, v:v); ^1H NMR (500 MHz, $CDCl_3$, HH-COSY, HSQC, HMBC): δ 7.44 – 7.27 (m, 20H, CH_{arom}), 5.79 (ddt, *J* = 17.0, 10.3, 6.7 Hz, 1H, H-15), 5.13 – 5.04 (m, 2H, H-16, H-16), 4.97 (d, *J* = 3.9 Hz, 1H, H-1''), 4.94 – 4.82 (m, 5H, H-1', H-7', H-11, H-11', C*H*H Bn), 4.79 (s, 2H, CH_2 Bn), 4.72 – 4.68 (m, 3H, H-1, H-10, H-10'), 4.61 – 4.53 (m, 2H, H-7, CH*H* Bn), 4.48 (d, *J* = 11.8 Hz, 1H, C*H*H Bn), 4.42 (d, *J* = 11.9 Hz, 1H, CH*H* Bn), 4.38 (d, *J* = 10.7 Hz, 1H, C*H*H Bn), 4.32 (d, *J* = 10.8 Hz, 1H, CH*H* Bn), 4.09 – 4.03 (m, 2H, H-3'', H-5''), 3.99 – 3.91 (m, 3H, H-2'', H-9, H-9'), 3.82 – 3.73 (m, 3H, H-4'',H-5, H-5'), 3.65 (dt, *J* = 9.9, 6.8 Hz, 1H, H-2'), 3.57 (ddd, *J* = 12.0, 4.7, 3.5 Hz, 1H, H-13), 3.54 – 3.50 (m, 1H, H-2), 3.47 (dt, *J* = 10.0, 6.5 Hz, 1H, H-13), 2.35 (qd, *J* = 6.7, 6.2, 2.9 Hz, 2H, H-14), 1.94 – 1.89 (m, 2H, H-8', H-8'), 1.83 (ddd, *J* = 9.1, 6.1, 3.2 Hz, 2H, H-8, H-8), 1.76 (dd, *J* = 13.6, 11.9 Hz, 1H, H-3), 1.68 (dd, *J* = 13.5, 11.9 Hz, 1H, H-3'), 1.48 (d, *J* = 6.7 Hz, 6H, H-12, H-12'), 1.27 (d, *J* = 6.4 Hz, 3H, H-6''), 1.21 (d, *J* = 6.2 Hz, 3H, H-6'), 1.15 (d, *J* = 6.3 Hz, 3H, H-6); ^{13}C NMR (126 MHz, $CDCl_3$, HSQC): δ 153.7, 153.7, 153.4, 153.3 (O(C=O)O), 138.0, 138.0, 137.7, 137.3 ($C_{q\text{-}arom}$), 135.0 (C-15), 129.1, 129.1, 129.1, 128.8, 128.6, 128.6, 128.5, 128.5, 128.4, 128.3, 128.2, 128.1, 127.9, 127.9, 127.8, 127.8, 127.7 (CH_{arom}), 117.1 (C-16), 100.9 (C-1''), 98.3 (C-1'), 95.4 (C-1), 84.5 (C-4'), 83.8 (C-4), 80.4 (C-10, C-10'), 80.1 (C-7'), 80.0 (C-7), 78.3 (C-3''), 76.3 (C-9'/C-9), 76.0 (C-9/C-9'), 75.9 (C-2''), 75.9 (C-11'/C-11), 75.6 (C-11/C-11'), 75.3, 72.6 (CH_2 Bn), 72.2 (C-2'), 72.1 (CH_2 Bn), 71.6 (C-2), 71.5 (CH_2 Bn), 67.8 (C-13), 66.4 (C-5'), 66.0 (C-5''), 64.8 (C-4''), 64.1 (C-5), 33.9 (C-14), 32.9 (C-3), 32.0 (C-3'), 29.8 (C-8'), 29.3 (C-8), 17.6 (C-6''), 15.2 (C-12'/C-12), 15.1 (C-12/C-12'), 15.0 (C-6, C-6'); HRMS: [M+Na]$^+$ calcd for $C_{66}H_{77}O_{22}N_3Na$ 1286.4896, found 1286.4890.

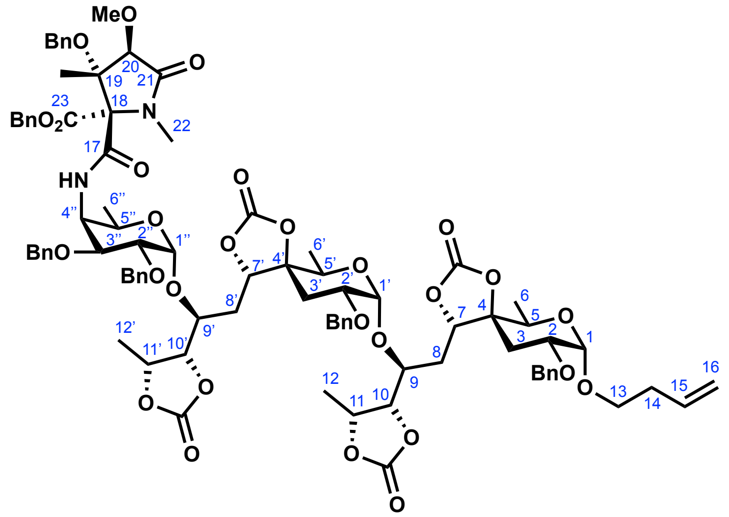

3-Butene 2-*O*-benzyl-4,7,10,11-di-*O*-carbonate-9-*O*-[2-*O*-benzyl-4,7,10,11-di-*O*-carbonate-9-*O*-[4-[(2'*S*,3'*S*,4'*R*)-3'-*O*-Benzyl-2'-(benzyloxycarbonyl)-4'-methoxy-1',3'-dimethyl-5'-oxopyrrolidine-2'-carboxamido]-2,3-di-*O*-benzyl-4,6-dideoxy-α-D-galactopyranosyl]-α-D-caryophyllosyl]-α-D-caryophylloside (35). Compound **34** (26.5 mg, 21 µmol, 1.0 eq.) was dissolved in THF (200 µL, 0.1 M) followed by the addition of trimethylphosphine (23.1 µL, 23.1 µmol, 1.1 eq. [1.0 M solution in THF, Sigma-Aldrich]). The mixture was stirred for 3 h at room temperature upon which H_2O (4.7 µL, 262 µmol, 12.5 eq.) was added and the reaction was stirred for another 18 h. Upon completion, the reaction was concentrated *in vacuo* to yield the crude galactosamine. To a stirred solution of pyrrolidone **2** (10.9 mg, 26.3 µmol, 1.25 eq.) and triethylamine (7.3 µL, 53 µmol, 2.5 eq.) in CH_3CN (0.2 mL, 0.1 M) was added HATU (10.5 mg, 27.7 µmol, 1.3 eq.). The solution was stirred for 30 min at room temperature followed by the addition of the galactosamine in 0.2 mL CH_3CN. The reaction was stirred for 3 h at room temperature upon which 1M HCl and EtOAc were added. The aqueous layer was extracted with EtOAc (3x) followed by washing the combined organic layers with H_2O, sat. aq. $NaHCO_3$ and brine, respectively. Subsequently, the organic layer was dried over $MgSO_4$, filtered, and concentrated *in vacuo* to yield the crude product as a colorless oil. Size exclusion chromatography by isocratic elution with DCM:MeOH (1:1, v:v) yielded the title compound (5 mg, 3 µmol, 15%, over 2 steps) as a colorless oil. TLC: R_f 0.5 (toluene:acetone, 7:3, v:v); ^{1}H NMR (600 MHz, $CDCl_3$, HH-COSY, HSQC): δ 8.08 (d, *J* = 10.1 Hz, 1H, NH), 7.39 – 7.13 (m, 30H, CH_{arom}), 5.78 (ddt, *J* = 17.1, 10.3, 6.7 Hz, 1H, H-15), 5.21 (d, *J* = 12.0 Hz, 1H, C*H*H Bn), 5.15 – 5.05 (m, 3H, CH*H* Bn, H-16, H-16), 4.96 (d, *J* = 4.1 Hz, 1H, H-1''), 4.93 – 4.82 (m, 5H, H-1', H-7', H-11', H-11, C*H*H Bn), 4.72 – 4.67 (m, 3H, H-1, H-10', H-10), 4.65 – 4.60 (m, 2H, H-4'', C*H*H Bn), 4.59 – 4.53 (m, 2H, H-7, CH*H* Bn), 4.51 – 4.46 (m, 2H, CH*H* Bn, C*H*H Bn), 4.41 (d, *J* = 11.8 Hz, 1H, CH*H* Bn), 4.37 (d, *J* = 11.6 Hz, 1H, CH*H* Bn), 4.34 (d, *J* = 10.6 Hz, 1H, C*H*H Bn), 4.29 (d, *J* = 10.5 Hz, 1H, CH*H* Bn), 4.23 (q, *J* = 6.6, 3.7 Hz, 1H, H-5''), 4.03 (dd, *J* = 10.2, 3.9 Hz, 1H, H-3''), 3.99 (q, *J* = 5.9 Hz, 1H, H-9'), 3.94 (dt, *J* = 10.0, 3.6 Hz, 1H, H-9), 3.91 (s, 1H, H-20), 3.79 (q, *J* = 6.2 Hz, 1H, H-5'), 3.73 (q, *J* = 6.2 Hz, 1H, H-5), 3.67 – 3.55 (m, 5H, H-2'', H-13, OCH_3), 3.55 – 3.42 (m, 3H, H-2, H-2', H-13), 2.58 (s, 3H, NCH_3), 2.34 (ddt, *J* = 6.4, 3.0, 1.4 Hz, 2H, H-14), 1.93 – 1.61 (m, 8H, H-3, H-3', H-3, H-3', H-8, H-8', H-8, H-8'), 1.52 (d, *J* = 6.6 Hz, 3H, H-12'), 1.47 (d, *J* = 6.6 Hz, 3H, H-12), 1.44 (s, 3H, 19-CH_3), 1.22 (d, *J* = 6.2 Hz, 3H, H-6'), 1.19 (d, *J* = 6.4 Hz, 3H, H-6''), 1.13 (d, *J* = 6.3 Hz, 3H, H-6); ^{13}C NMR (151 MHz, $CDCl_3$, HSQC): δ 172.7 (C=O ester), 169.1, 164.5 (C=O amide), 153.6, 153.4 (O(C=O)O), 138.3, 138.2, 137.9, 137.8, 137.2 ($C_{q\text{-}arom}$), 135.0 (C-15), 134.4 ($C_{q\text{-}arom}$), 129.2, 129.1, 129.0, 128.8, 128.7, 128.7, 128.6, 128.6, 128.6, 128.5, 128.4, 128.2, 128.2, 127.9, 127.8, 127.8, 127.7, 127.1 (CH_{arom}), 117.1 (C-16), 101.4 (C-1''), 97.8 (C-1'), 95.3 (C-1), 84.4 (C-4'), 84.0 (C-19), 83.8 (C-4), 82.5 (C-20), 80.5 (C-10'), 80.1 (C-10), 80.0 (C-7'), 79.8 (C-7), 78.9 (C-18), 77.6 (C-3''), 76.5 (C-9'/C-9), 76.3 (C-2''), 76.0 (C-9/C-9'), 75.9 (C-11'/C-11), 75.6 (CH_2 Bn), 75.6 (C-11/C-11'), 72.2 (C-2'), 72.1, 71.6 (CH_2 Bn), 71.5 (C-2), 71.4, 68.7, 67.8, 66.5 (CH_2 Bn), 66.5 (C-5'), 65.7 (C-5''), 64.7 (C-5), 59.5 (CH_3 OMe), 51.4 (C-4''), 33.9 (C-14), 32.8 (C-3'), 31.8 (C-3), 30.0 (C-8'), 29.8 (C-8), 29.2 (CH_3 NMe), 17.6 (C-6''), 15.3 (C-12'/C-12), 15.2 (C-12/C-12'), 15.0 (C-6'/C-6), 15.0 (C-6/C-6'), 14.9 (CH_3); HRMS: $[M+Na]^+$ calcd for $C_{89}H_{102}O_{28}N_2Na$ 1669.6517, found 1669.6511.

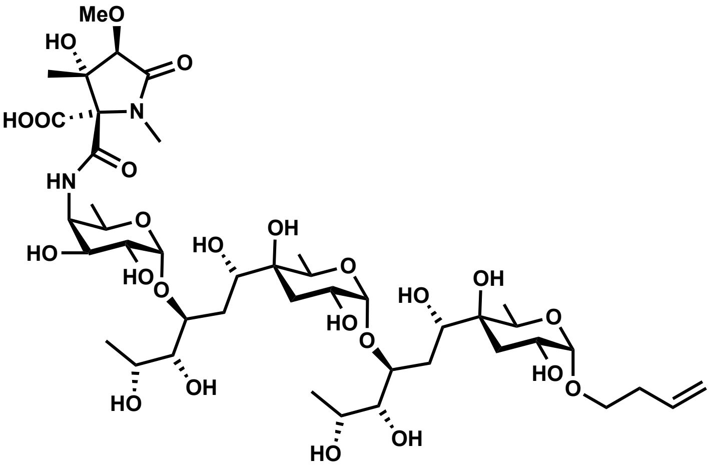

3-Butene 9-*O*-[9-*O*-[4-[(2'*S*,3'*S*,4'*R*)-2'-carboxyl-3'-hydroxy-4'-methoxy-1',3'-dimethyl-5'-oxopyrrolidine-2'-carboxamido]-4,6-dideoxy-α-D-galactopyranosyl]-α-D-caryophyllosyl]-α-D-caryophylloside (1). The protected target structure **35** (4.9 mg, 3 µmol) was dissolved in 0.6 mL 1:1 v:v THF:H_2O (0.005 M). LiOH·H_2O (12.6 mg, 300 µmol, 100 eq.) was added and the resulting mixture was stirred at room temperature for 20 h. Upon completion, 80% of the LiOH was quenched with 0.1 M HCl (2.4 mL) and the mixture was concentrated under reduced pressure to yield the crude product. The crude product was then co-evaporated twice with dry toluene. 3 mL ammonia was condensed at –70 °C, sodium (3.45 mg, 150 µmol, 50 eq.) was added and the resulting suspension was stirred for 30 min. The crude product was dissolved in 0.5 mL THF, hexene (50 µL, used for scales from 1-25 µmol) and *t*-BuOH (2.85 µL, 30 µmol, 10 eq.) and the solution was added to the suspension of sodium in ammonia. The reaction mixture was stirred at –70 °C for 15 min, upon which the reaction was quenched with water. The reaction mixture was then stirred at room temperature until all ammonia had evaporated. The mixture was concentrated *in vacuo* to yield the crude product as a colorless oil. Flash column chromatography (30:70 → 50:50; MeOH:DCM) followed by size exclusion over a 250x10 mm column filled with Biogel P2 media (Bio-Rad) yielded the title compound **1** (1.2 mg, 1.2 µmol, 40% over two steps) as a colorless oil. The NMR data showed the presence of four atropisomers in D_2O. Data for atropisomeric mixture: ^1H NMR (850 MHz, D_2O, HH-COSY, HSQC): δ 6.10 (td, *J* = 10.4, 6.7 Hz, 1H, H-15), 5.35 (d, *J* = 17.5, 1.7 Hz, 1H, H-16), 5.30 – 5.26 (m, 2H, H-1", H-16), 5.13 – 5.09 (m, 1H, H-1'), 4.98 (d, *J* = 3.7 Hz, 1H, H-1), 4.59 – 4.39 (m, 4H, H-4", H-5", H-5', H-5), 4.34 – 4.22 (m, 3H, H-3", H-9', H-9), 4.19 – 4.05 (m, 5H, H-2', H-2, H-7', H-7, H-20), 3.98 – 3.71 (m, 10H, H-2", H-10', H-10, H-11', H-11, H-13, H-13, CH_3 OMe), 3.01 – 2.93 (m, 3H, CH_3 NMe), 2.58 (p, *J* = 7.0 Hz, 2H, H-14), 2.19 – 2.05 (m, 4H, H-8', H-8, H-3', H-3), 2.00 – 1.91 (m, 2H, H-8', H-8), 1.84 – 1.74 (m, 2H, H-3', H-3), 1.51 – 1.36 (m, 9H, H-12', H-12, 19-CH_3), 1.35 – 1.23 (m, 9H, H-6", H-6', H-6); ^{13}C NMR (214 MHz, D_2O, HSQC): δ 172.2, 172.1, 170.0, 169.9 (C=O amide), 136.9 (C-15), 117.4 (C-16), 102.2 (C-1"), 101.0 (C-1'), 98.1 (C-1), 85.6, 85.1, 83.7 (C-20), 80.2, 80.2 (C-19), 78.9 (C-9', C-9), 78.9 (C-9, C-9'), 78.7 (C-10', C-10), 78.3 (C-10, C-10'), 75.9 (C-4', C-4), 75.8 (C-4, C-4'), 70.8, 70.5 (C-2"), 70.1, 70.0 (C-3"), 69.9 (C-7', C-7), 69.8 (C-7, C-7'), 68.5 (C-18), 68.2 (C-5', C-5), 68.2 (C-11', C-11), 68.1 (C-11, C-11'), 68.0 (C-13), 67.7 (C-5, C-5'), 66.8, 66.3 (C-5"), 65.9 (C-2', C-2), 65.5 (C-2, C-2'), 60.9, 60.8 (CH_3 OMe), 56.0, 55.5 (C-4"), 34.1 (C-14), 31.1 (C-8', C-8), 31.0 (C-8, C-8'), 30.7, 30.6, 30.5 (CH_3 NMe), 29.3 (C-3', C-3), 29.2 (C-3, C-3'), 20.1 (C-12', C-12), 19.9 (C-12, C-12'), 19.0, 17.2 (CH_3), 16.6 (C-6"), 13.0 (C-6', C-6), 12.9 (C-6, C-6'); HRMS: $[M+Na]^+$ calcd for $C_{43}H_{74}O_{24}N_2Na$ 1025.4529, found 1025.4524.

Synthesis of compound 36 and S44

Scheme S6. Synthesis of compound **36** and **S44**.

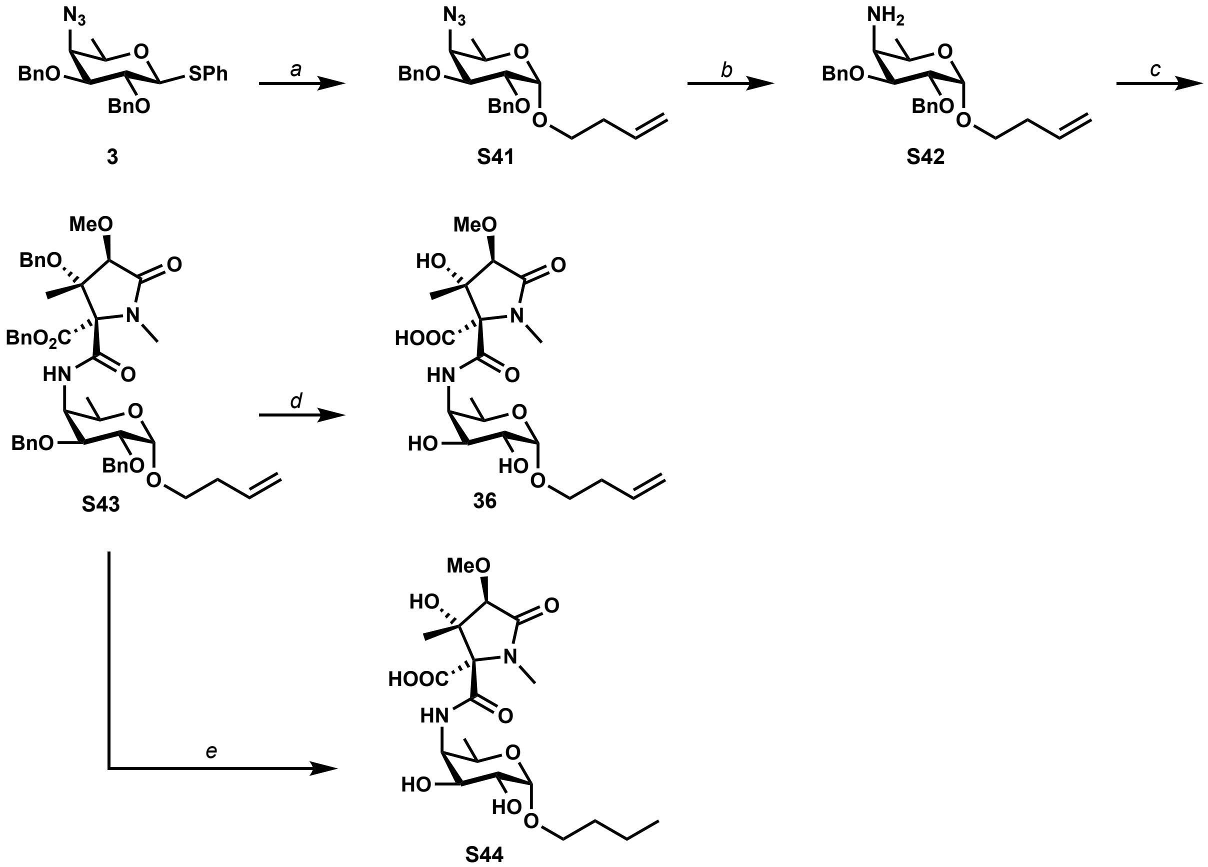

Reagents and conditions: a) Ph_2SO, TTBP, ethyl maleimide, Tf_2O, TBAI, 3-buten-1-ol (95%); b) triphenylphosphine, THF (79%); c) pyrralidone **2**, TEA, HATU, CH_3CN (88%); d) Na, NH_3, *t*-BuOH, THF (44%); e) H_2, $Pd(OH)_2/C$, THF, *t*-BuOH, H_2O (13%).

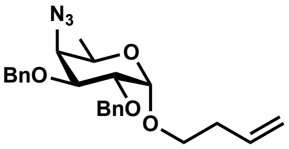

3-Butene 4-azido-2,3-di-*O*-benzyl-4,6-dideoxy-α-D-galactopyranoside (S41). To a solution of the donor **3** (23 mg, 50 µmol, 1 eq.) in DCM (1 mL, 0.05 M), Ph_2SO (13 mg, 65 µmol, 1.3 eq.), TTBP (31 mg, 125 µmol, 2.5 eq.) and ethyl maleimide (12.5 mg, 100 µmol, 2.0 eq) were added. The solution was stirred over activated 3 Å molecular sieves (rods, size 1/16 in., Sigma Aldrich) for 30 min. The solution was cooled to –80 °C upon which Tf_2O (11 µL, 65 µmol, 1.3 eq.) was added slowly. Subsequently, the solution was allowed to attain to –50 °C to secure full activation of the donor followed by cooling back to –80 °C after which TBAI (148 mg, 0.4 mmol, 8 eq.) was added. The solution was stirred for 15 min at –80 °C followed by the addition of the acceptor 3-buten-1-ol (0.2 mL of a 0.5 M solution, 2.0 eq.). The reaction was stirred for 16 h at 0 °C upon which the reaction was quenched with sat. aq. $NaHCO_3$ followed by the dilution with EtOAc. The organic layer was washed with H_2O and brine, dried over $MgSO_4$, filtered off and concentrated under reduced pressure. Flash column chromatography (95:5 → 92:8; pentane:Et_2O) yielded the title compound **S41** (19.1 mg, 45 µmol, 95%, α:β; >98:2) as a colorless oil. TLC: R_f 0.6 (pentane:Et_2O, 9:1, v:v); $[\alpha]_D^{20}$ 24.3° (c 1.0, $CHCl_3$); IR (neat, cm^{-1}): 697, 1045, 1105, 1709, 2109, 2916; ^1H NMR (400 MHz, $CDCl_3$, HH-COSY, HSQC): δ 7.42 – 7.27 (m, 10H, CH_{arom}), 5.81 (ddt, *J* = 17.0, 10.2, 6.7 Hz, 1H, H-9), 5.10 (dq, *J* = 17.2, 1.6 Hz, 1H, H-10), 5.08 – 5.00 (m, 1H, H-10), 4.85 (d, *J* = 11.7 Hz, 1H, C*H*H Bn), 4.81 (d, *J* = 12.0 Hz, 1H, C*H*H Bn), 4.74 (d, *J* = 11.7 Hz, 1H, CH*H*

Bn), 4.70 (d, *J* = 3.8 Hz, 1H, H-1), 4.64 (d, *J* = 12.0 Hz, 1H, CH*H* Bn), 4.03 (dd, *J* = 9.9, 3.7 Hz, 1H, H-3), 3.96 (qd, *J* = 6.5, 1.6 Hz, 1H, H-5), 3.83 (dd, *J* = 9.9, 3.8 Hz, 1H, H-2), 3.72 (dd, *J* = 3.8, 1.5 Hz, 1H, H-4), 3.56 (ddt, *J* = 44.4, 9.9, 7.0 Hz, 2H, H-7, H-7), 2.37 (qt, *J* = 7.0, 1.4 Hz, 2H, H-8, H-8), 1.21 (d, *J* = 6.5 Hz, 3H, H-6); ^{13}C NMR (101 MHz, $CDCl_3$, HSQC): δ 138.6, 138.4 ($C_{q\text{-}arom}$), 135.1 (C-9), 128.6, 128.5, 128.1, 127.9, 127.9, 127.8 (CH_{arom}), 116.8 (C-10), 97.5 (C-1), 78.2 (C-3), 76.2 (C-2), 73.6, 73.3 (CH_2 Bn), 67.7 (C-7), 65.2 (C-4), 64.5 (C-5), 34.0 (C-8), 17.4 (C-6). HRMS: $[M+Na]^+$ calcd for $C_{24}H_{29}O_4N_3Na$ 446.2056, found 446.2050.

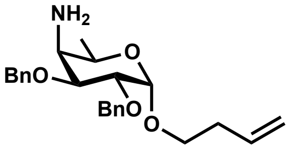

3-Butene 4-amine-2,3-di-*O*-benzyl-4,6-dideoxy-α-D-galactopyranoside (S42). Azide **S41** (42.4 mg, 0.1 mmol, 1 eq.) was dissolved in THF (250 µL, 0.4 M) followed by the addition of polymer bound triphenylphosphine (66.7 mg, 0.2 mmol, 2 eq.; 100-200 mesh, 3 mmol/gr). The mixture was stirred for 3 h at room temperature upon which H_2O (22.6 µL, 1.25 mmol, 12.5 eq.) was added and the reaction was stirred for another 16 h. Upon completion, the reaction was filtered, rinsed with $CHCl_3$, and concentrated *in vacuo* to yield the crude product. Flash column chromatography (10:90 → 0:100; pentane:EtOAc) yielded the title compound (31.3 mg, 78.7 µmol, 79%) as a colorless oil. TLC: R_f 0.1 (pentane:EtOAc, 1:9, v:v); $[\alpha]_D^{20}$ 39.6° (c 1.0, $CHCl_3$); IR (neat, cm^{-1}): 698, 1042, 1100, 2928; ^1H NMR (400 MHz, $CDCl_3$, HH-COSY, HSQC): δ 7.73 – 7.26 (m, 10H, CH_{arom}), 5.84 (ddt, *J* = 17.0, 10.2, 6.7 Hz, 1H, H-9), 5.11 (dq, *J* = 17.2, 1.6 Hz, 1H, H-10), 5.05 (ddt, *J* = 10.2, 2.1, 1.2 Hz, 1H, H-10), 4.79 (d, *J* = 12.1 Hz, 1H, C*H*H Bn), 4.77 – 4.73 (m, 2H, C*H*H Bn, H-1), 4.70 – 4.62 (m, 2H, CH*H* Bn, CH*H* Bn), 4.02 (qd, *J* = 6.6, 1.7 Hz, 1H, H-5), 3.86 (dd, *J* = 9.9, 4.0 Hz, 1H, H-3), 3.74 (dd, *J* = 10.0, 3.9 Hz, 1H, H-2), 3.59 (ddt, *J* = 55.2, 9.9, 7.0 Hz, 2H, H-7, H-7), 3.16 (dd, *J* = 4.1, 1.8 Hz, 1H, H-4), 2.40 (qt, *J* = 7.0, 1.4 Hz, 2H, H-8, H-8), 1.21 (d, *J* = 6.6 Hz, 3H, H-6); ^{13}C NMR (101 MHz, $CDCl_3$, HSQC): δ 138.8 ($C_{q\text{-}arom}$), 135.2 (C-9), 128.5, 128.5, 128.0, 127.8, 127.8 (CH_{arom}), 116.7 (C-10), 97.5 (C-1), 78.6 (C-3), 75.5 (C-2), 73.2, 72.5 (CH_2 Bn), 67.5 (C-7), 65.3 (C-5), 53.5 (C-4), 34.1 (C-8), 16.8 (C-6); HRMS: $[M+H]^+$ calcd for $C_{24}H_{32}O_4N$ 398.2331, found 398.2326.

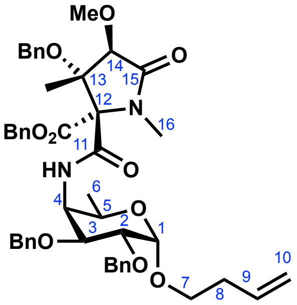

3-Butene 4-[(2'*S*,3'*S*,4'*R*)-3'-*O*-Benzyl-2'-(benzyloxycarbonyl)-4'-methoxy-1',3'-dimethyl-5'-oxopyrrolidine-2'-carboxamido] 2,3-di-*O*-benzyl-4,6-dideoxy-α-D-galactopyranoside (S43). To a stirred solution of pyrrolidone **2** (63.7 mg, 154 µmol, 1.25 eq.) and triethylamine (42.7 µL, 308 µmol, 2.5 eq.) in CH_3CN (0.4 mL, 0.15 M) was added HATU (62 mg, 163 µmol, 1.3 eq.). The solution was stirred for 30 min at room temperature followed by the addition of galactosamine **S42** in 0.4 mL CH_3CN. The reaction was stirred for 3 h at room temperature upon which 1M HCl and EtOAc were added. The aqueous layer was extracted with EtOAc (3x) followed by washing the combined organic layers with H_2O, sat. aq. $NaHCO_3$ and brine, respectively. Subsequently, the organic layer was dried over $MgSO_4$, filtered, and concentrated *in vacuo* to yield the crude product as a colorless oil. Flash column chromatography (80:20 → 60:40; pentane:EtOAc) yielded the title compound (86 mg, 108 µmol, 88%) as a colorless oil. TLC: R_f 0.6 (pentane:EtOAc, 1:1, v:v); $[\alpha]_D^{20}$ 79.4° (c 1.0, $CHCl_3$); IR (neat, cm^{-1}): 698, 1046, 1097, 1686, 1717, 2926; ^1H NMR (400 MHz, $CDCl_3$, HH-COSY, HSQC): δ 8.13 (d, *J* = 10.0 Hz, 1H, NH), 7.38 – 7.13 (m, 20H, CH_{arom}), 5.84 (ddt, *J* = 17.0, 10.2, 6.7 Hz, 1H, H-9), 5.17 (d, *J* = 12.1 Hz,

1H, C*H*H Bn), 5.15 – 5.04 (m, 3H, C*H*H Bn, H-10, H-10), 4.84 (d, *J* = 10.9 Hz, 1H, C*H*H Bn), 4.78 (d, *J* = 12.2 Hz, 1H, C*H*H Bn), 4.74 (d, *J* = 3.9 Hz, 1H, H-1), 4.61 (d, *J* = 6.3 Hz, 1H, CH*H* Bn), 4.60 – 4.51 (m, 3H, CH*H* Bn, CH*H* Bn, H-4), 4.35 (d, *J* = 11.6 Hz, 1H, CH*H* Bn), 4.18 (tt, *J* = 7.4, 3.5 Hz, 1H, H-5), 3.98 (dd, *J* = 10.1, 4.1 Hz, 1H, H-3), 3.84 (s, 1H, H-14), 3.72 – 3.62 (m, 1H, H-7), 3.59 – 3.48 (m, 5H, H-2, H-7, CH_3 OMe), 2.65 (s, 3H, CH_3 NMe), 2.40 (qt, *J* = 7.0, 1.4 Hz, 2H, H-8), 1.45 (s, 3H, CH_3), 1.15 (d, *J* = 6.5 Hz, 3H, H-6); ^{13}C NMR (101 MHz, $CDCl_3$, HSQC): δ 172.4 (C=O ester), 168.8, 164.7 (C=O amide), 138.8, 138.7, 137.8 ($C_{q\text{-}arom}$), 135.0 (C-9), 134.7($C_{q\text{-}arom}$), 128.8, 128.7, 128.5, 128.5, 128.4, 128.2, 128.1, 127.9, 127.7, 127.5, 127.1 (CH_{arom}), 116.9 (C-10), 97.6 (C-1), 83.9 (C-13), 82.4 (C-14), 79.5 (C-12), 77.6 (C-3), 75.6 (C-2), 73.4, 71.9, 68.3 (CH_2 Bn), 67.8 (C-7), 66.3 (CH_2 Ph), 64.2 (C-5), 59.5 (CH_3 OMe), 52.0 (C-4), 34.0 (C-8), 29.2 (CH_3 NMe), 17.4 (C-6), 14.8 (CH_3); HRMS: $[M+H]^+$ calcd for $C_{47}H_{55}O_{10}N_2$ 807.3857, found 807.3851.

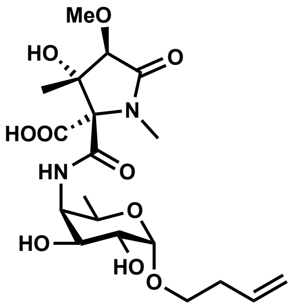

3-Butene 4-[(2'*S*,3'*S*,4'*R*)-2'-carboxyl-3'-hydroxy-4'-methoxy-1',3'-dimethyl-5'-oxopyrrolidine-2'-carboxamido]-4,6-dideoxy-α-D-galactopyranoside (36). The protected galactopyranoside **S43** (19.8 mg, 25 µmol) was co-evaporated twice with dry toluene. 20 mL ammonia was condensed at –70 °C, sodium (22.5 mg, 0.98 mmol, 40 eq.) was added and the resulting suspension was stirred for 30 min. Galactopyranoside was dissolved in 4 mL THF, 3-buten-1-ol (100 µL, used for scales from 10-100 µmol) and *t*-BuOH (24 µL, 250 µmol, 10 eq.) and the solution was added to the suspension of sodium in ammonia. The reaction mixture was stirred at –70 °C for 15 min, upon which the reaction was quenched with water. The reaction mixture was then stirred at room temperature until all ammonia had evaporated. The mixture was concentrated *in vacuo* to yield the crude product as a colorless oil. Flash column chromatography (30:70 → 50:50; MeOH:DCM) yielded the title compound (4.9 mg, 11 µmol, 44%) as a colorless oil. TLC: R_f 0.2 (DCM:MeOH, 7:3, v:v); The NMR data showed the presence of two atropisomers in D_2O in a 60:40 ratio. Data for atropisomeric mixture: ^1H NMR (500 MHz, D_2O, HH-COSY, HSQC: δ 5.89 (ddt, *J* = 17.1, 10.4, 6.7 Hz, 1H, H-9), 5.15 (dt, *J* = 17.3, 1.9 Hz, 1H, H-10), 5.11 – 5.06 (m, 1H, H-10), 4.98 – 4.93 (m, 1H, H-1), 4.30 (ddd, *J* = 13.0, 6.5, 1.6 Hz, 1H, H-5), 4.24 – 4.21 (m, 1H, H-4), 4.15 – 4.13 (m, 0.4H, H-14*), 4.03 (ddd, *J* = 10.8, 6.7, 4.2 Hz, 1H, H-3), 3.91 (t, *J* = 0.7 Hz, 0.6H, H-14), 3.74 (dt, *J* = 10.0, 6.8 Hz, 1H, H-7), 3.68 – 3.57 (m, 5H, H-2, H-7, OCH_3), 2.80 – 2.74 (m, 3H, NMe, 2.39 (d, *J* = 6.6 Hz, 2H, H-8), 1.56 (s, 1.8H, CH_3), 1.24 – 1.17 (m, 4.2H, H-6, CH_3*); ^{13}C NMR (126 MHz, D_2O, HSQC): δ 174.8 (C=O acid), 173.9 (C=O acid*), 171.7 (C=O amide), 171.3, 168.6 (C=O amide*), 167.9 (C=O amide), 135.7 (C-9), 116.7 (C-10), 98.4 (C-1), 84.7 (C-14*), 83.1 (C-14), 79.4 (C-13*), 76.2 (C-13), 69.2 (C-3*), 69.0 (C-3), 68.7 (OCH_3), 68.5 (OCH_3*), 67.8 (C-7), 65.7 (C-5), 65.2 (C-5*), 60.8 (C-2), 60.0 (C-2*), 54.9 (C-4*), 54.6 (C-4), 33.2 (C-8), 29.8 (NMe*), 29.7 (NMe), 22.5 (CH_3), 18.2 (CH_3*), 16.3 (C-6*), 16.0 (C-6); HRMS: $[M+H]^+$ calcd for $C_{19}H_{31}O_{10}N_2$ 447.1979, found 447.1973.

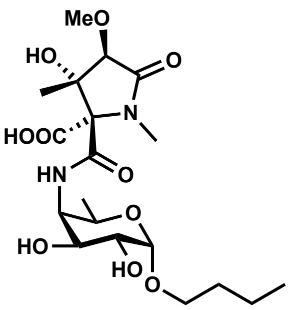

Butane 4-[(2'*S*,3'*S*,4'*R*)-2'-carboxyl-3'-hydroxy-4'-methoxy-1',3'-dimethyl-5'-oxopyrrolidine-2'-carboxamido]-4,6-dideoxy-α-D-galactopyranoside (S44). The protected galactopyranoside **S43** (19.8 mg, 25 µmol) was co-evaporated twice with dry toluene. It was then dissolved in 5 mL of a mixture of THF, *t*-BuOH and water (13:13:30). 3 drops of acetic acid were added and the solution was treated with palladium hydroxide on charcoal (52.7 mg, 20 % loading, Sigma-Aldrich) and subjected to hydrogen atmosphere for 20 h. The mixture was filtered through Celite© Hyflo Supercel (Merck) and the filtrate was concentrated *in vacuo*. Flash column chromatography (C18 column, gradient 100:0 → 50:50 CH_3OH–H_2O) yielded the title compound (1.5 mg, 3.4 µmol, 13%) as a white solid. TLC: R_f 0.2 (DCM:MeOH, 7:3, v:v); The NMR data showed the presence of two atropisomers in D_2O in a 60:40 ratio. Data for atropisomeric mixture: ^1H NMR (850 MHz, D_2O, HH-COSY, HSQC): δ 5.00 – 4.97 (m, 1H, H-1), 4.36 – 4.30 (m, 1H, H-5), 4.27 – 4.25 (m, 1H, H-4), 4.17 (s, 0.4H, H-14*), 4.09 – 4.05 (m, 1H, H-3), 3.95 (s, 0.6H, H-14), 3.75 – 3.55 (m, 6H, H-2, H-7, OMe), 2.83 – 2.80 (m, 3H, NMe), 1.68 – 1.58 (m, 3.8H, H-8, CH_3), 1.45 – 1.36 (m, 2H, H-9), 1.27 – 1.21 (m, 4.2H, H-6, CH_3*), 0.93 (t, *J* = 7.4 Hz, 3H, H-10); ^{13}C NMR (214 MHz, D_2O, HSQC): δ 174.7, 173.9 (C=O acid, C=O acid*), 171.6, 171.3, 168.5, 167.9 (C=O amide, C=O amide*), 98.3 (C-1*), 98.3 (C-1), 84.6 (C-14*), 83.1 (C-14), 81.3, 79.5 (C-14/C-13), 79.3, 76.2 (C-13/C-14, C-13*/C-14), 69.2 (C-3*), 69.0 (C-3), 68.6 (C-2), 68.4 (C-2*), 68.4 (C-7), 65.5 (C-5), 65.0 (C-5*), 60.8 (OCH_3), 59.9 (OCH_3*), 54.8 (C-4*), 54.6 (C-4), 30.7 (C-8), 29.7 (NMe*), 29.7 (NMe), 22.4 (CH_3), 18.7 (C-9), 18.1 (CH_3*), 16.3 (C-6*), 16.0 (C-6), 13.0 (C-10); HRMS: $[M+H]^+$ calcd for $C_{19}H_{33}O_{10}N_2$ 449.2135, found 449.2130.

Synthesis of compound 37

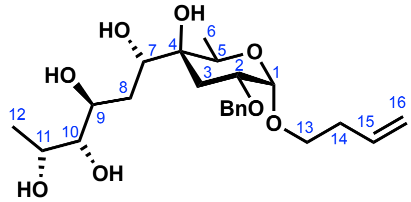

3-Butene caryophylloside (37). The protected glycoside **30** (33 mg, 50 µmol) was dissolved in 10 mL 1:1 v:v THF:H_2O (0.005 M). LiOH·H_2O (210 mg, 5.0 mmol, 100 eq.) was added and the resulting mixture was stirred at rt for 20 h. Upon completion 80% of the LiOH was quenched with 0.1 M HCl (40 mL) and the mixture was concentrated under reduced pressure to yield the crude product. The crude product was then co-evaporated twice with dry toluene. 3 mL ammonia was condensed at –70 °C, sodium (23 mg, 1.0 mmol, 20 eq.) was added and the resulting suspension was stirred for 30 min. The crude product was dissolved in 0.5 mL THF, 3-butenol (50 µL, 1.0 mmol, 20 eq.) and *t*-BuOH (50 µL, 500 µmol, 10 eq.) and the solution was added to the suspension of sodium in ammonia. The reaction mixture was stirred at –70 °C for 15 min, upon which the reaction was quenched with water. The reaction mixture was then stirred at room temperature until all ammonia had evaporated. The mixture was concentrated in vacuo to yield the crude product as a colourless oil. Flash column chromatography (5:95 → 20:80; MeOH:DCM) followed by size exclusion over a 250x10 mm column filled with Biogel P2 media (Bio-Rad) yielded the title compound (4.6 mg, 13 µmol, 26% over two steps) as a colourless oil. ^1H NMR (500 MHz, D_2O, HH-COSY, HSQC): δ 5.90 (ddt, *J* = 17.1, 10.3, 6.7 Hz, 1H, H-15), 5.16 (dq, *J* = 17.3, 1.7 Hz, 1H, H-16), 5.08 (dd, *J* = 10.3, 2.1 Hz, 1H, H-16), 4.80 (H-1, value from HSQC due to overlap with the solvent signal) 4.25 (q, *J* = 6.5 Hz, 1H, H-5), 3.98 (ddd, *J* = 12.3, 5.1, 3.7 Hz, 1H, H-2), 3.93 (dt, *J* = 12.5, 6.3 Hz, 1H, H-11), 3.81 (td, *J* = 7.4, 6.2, 2.2 Hz, 1H, H-9), 3.77 – 3.70 (m, 2H, H-7, H-13), 3.63

(dt, J = 9.8, 5.9 Hz, 1H, H-1'), 3.50 (t, J = 5.9 Hz, 1H, H-10), 2.43 – 2.36 (m, 2H, H-14), 1.93 (t, J = 12.6 Hz, 1H, H-3), 1.77 – 1.61 (m, 3H, H-3, H-8), 1.20 (d, J = 6.4 Hz, 3H, H-12), 1.13 (d, J = 6.6 Hz, 3H, H-6); ^{13}C NMR (126 MHz, D2O, HSQC): δ 135.9 (C-15), 116.6 (C-16), 97.1 (C-1), 77.4 (C-10), 74.8 (C-4), 69.6 (C-7), 67.9 (C-9), 67.5 (C-11), 67.1 (C-13), 66.9 (C-5), 64.6 (C-2), 33.3 (C-14), 31.9 (C-8), 30.4 (C-3), 16.9 (C-12), 11.3 (C-6); HRMS: $[M+Na]^+$ calcd for $C_{16}H_{30}O_8Na$ 373.1838, found 373.1833.

Preparation of model acceptors

Synthesis of acceptor 28

Scheme S7. Synthesis of compound **28**.

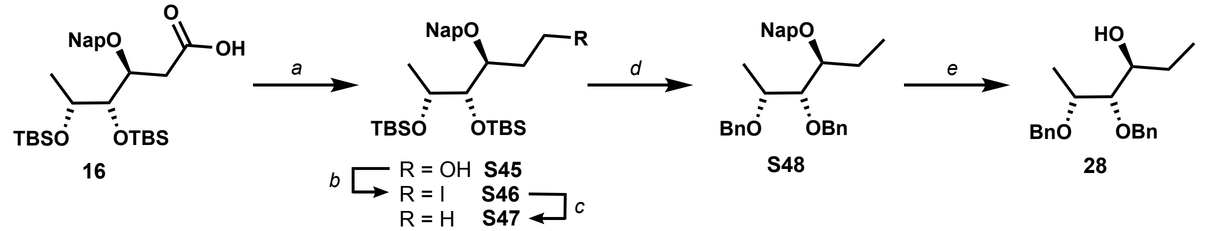

Reagents and conditions: a) $BH_3{\cdot}THF$, THF (75%); b) triphenylphosphine, imidazole, iodine, THF (92%); c) $LiAlH_4$, THF (72%); d) i: HF-pyridine, pyridine; ii: NaH, BnBr, DMF (91%); e) DDQ, DCM (67%).

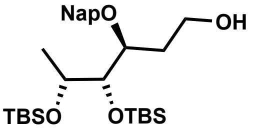

2,6-Dideoxy-3-*O*-(2-methylnaphthalene)-4,5-*O*-di-*tert*-butyldimethylsilyl-D-altritol (S45). Carboxylate **16** (2.96 g, 5.5 mmol) was dissolved in THF (10 mL), followed by adding $BH_3{\cdot}THF$ (17 mL, 1.0 M in THF, 3.0 eq) at 0 °C. The reaction mixture was left stirring at room temperature for 16 h, after which it was concentrated *in vacuo* to a thick syrup, which was absorbed on silica gel and chromatographed using pentane:Et_2O (75:25) as a mobile phase. The product was obtained as a clear oil (2.1 g, 75%). TLC: R_f 0.3 (pentane:Et_2O, 75:25); ^{1}H NMR (300 MHz, $CDCl_3$, HH-COSY, HSQC): δ 7.89 – 7.80 (m, 4H, CH_{arom}), 7.53 – 7.47 (m, 3H, CH_{arom}), 4.83 (d, J = 12.3 Hz, 1H, CH*H* Nap), 4.64 (d, J = 12.3 Hz, 1H, CH*H* Nap), 3.92 (m, 1H, H-3), 3.81 – 3.71 (m, 4H, H-5, H-4, H-1 x 2), 1.98 (m, 1H, H-2_a), 1.78 (m, 1H, H-2_b), 1.21 (d, J = 6.9 Hz, 3H, H-6), 0.93 (s, 9H, $C(CH_3)_3$), 0.87 (s, 9H, $C(CH_3)_3$), 0.16 – 0.03 (m, 12H, $SiCH_3$, $SiCH_3$, $SiCH_3$, $SiCH_3$); ^{13}C NMR (75 MHz, $CDCl_3$, HSQC): δ 135.5, 133.2, 133.0 ($C_{q\text{-}arom}$), 128.3, 127.9, 127.9, 127.7, 127.7, 127.7, 126.8, 126.8, 126.8, 126.1, 126.0, 125.9 (CH_{arom}), 79.5 (C-3), 77.8, 71.6 (CH_2 Nap), 69.6, 69.6, 60.8 (C-1), 31.1 (C-2), 26.1 ($C(CH_3)_3$), 26.1 ($C(CH_3)_3$), 25.8 ($C(CH_3)_3$), 20.8 (C-6), -3.9, -4.0, -4.4, -4.5, -4.8 ($SiCH_3$); HRMS: $[M+H]^+$ calcd for $C_{29}H_{51}O_4Si_2$ 519.3326, found 519.3323.

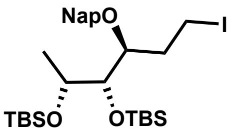

2,6-Dideoxy-1-deoxy-1-iodo-3-*O*-(2-methylnaphthalene)-4,5-*O*-di-*tert*-butyldimethylsilyl-D-altritol (S46). Alcohol **S45** (2.1 g, 4.04 mmol) was dissolved in THF (10 mL), followed by adding imidazole (884 mg, 13 mmol,1.5 eq), PPh_3 (1.75 g, 6.07 mmol, 1.5 eq.), and I_2 (1.5 g, 6.07 mmol, 1.5 eq.) sequentially at rt. The reaction mixture was heated at 60 °C for 1 h, after which it was quenched with sat. $Na_2S_2O_3$, diluted with CH_2Cl_2 and washed with H_2O. The organic layer was then dried over $MgSO_4$, filtered, and concentrated *in vacuo* to give the crude product as an oil, which was loaded on silica gel and chromatographed using pentane:Et_2O (90:10) as a mobile phase. The product was obtained as a clear oil (2.32 g, 92%). TLC: R_f 0.7 (pentane:Et_2O, 75:25); ^{1}H NMR (300 MHz, $CDCl_3$, HH-COSY, HSQC): δ

7.89 – 7.82 (m, 4H, CH_{arom}), 7.54 – 7.48 (m, 3H, CH_{arom}), 4.85 (d, *J* = 12.3 Hz, 1H, CH*H* Nap), 4.66 (d, *J* = 12.3 Hz, 1H, CH*H* Nap), 3.80 (m, 1H, H-5), 3.74 (m, 2H, H-4, H-3), 3.40 (m, 1H, C*H*H I), 3.28 (m, 1H, CH*H* I), 1.21 (d, *J* = 6.9 Hz, 3H, H-6), 0.93 (s, 9H, $C(CH_3)_3$), 0.87 (s, 9H, $C(CH_3)_3$), 0.16 – 0.03 (m, 12H, $SiCH_3$, $SiCH_3$, $SiCH_3$, $SiCH_3$); ^{13}C NMR (75 MHz, $CDCl_3$, HSQC): δ 135.9, 133.3, 132.9 ($C_{q\text{-}arom}$), 128.1, 127.9, 127.7, 126.5, 126.0, 126.0, 125.8 (CH_{arom}), 80.3, 78.0, 72.3 (CH_2 Nap), 69.7 (C-5), 34.2 (C-2), 26.1($C(CH_3)_3$), 26.0 ($C(CH_3)_3$), 20.5 (C-6), 4.1 (CH_2I), -3.9, -4.1, -4.4, -4.5 ($SiCH_3$); HRMS: $[M+H]^+$ calcd for $C_{29}H_{50}IO_3Si_2$ 629.2342, found 629.2337.

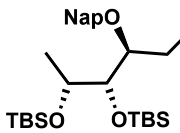

1,2,6-Trideoxy-3-*O*-(2-methylnaphthalene)-4,5-*O*-di-*tert*-butyldimethylsilyl-D-altritol (S47). Iodide **S46** (2.32 g, 3.75 mmol) was dissolved in dry THF (20 mL), and $LiAlH_4$ (1.5 mL, 4.0 M in Et_2O, 1.5 eq.) was added at 0 °C, and the reaction mixture was then left stirring at room temperature for 1 h. It was then carefully quenched with H_2O, after which saturated solution of Rochelle's salt was added, and stirring was continued at room temperature for 1 h. The reaction mixture was then diluted with Et_2O, and the organic layer was separated and dried over $MgSO_4$, filtered, and concentrated *in vacuo* to give the crude product as an oil, which was loaded on silica gel and chromatographed using pentane:Et_2O (90:10) as a mobile phase. The product was obtained as a clear oil (1.35 g, 72%). TLC: R_f 0.7 (pentane:Et_2O, 75:25); ^{1}H NMR (300 MHz, $CDCl_3$, HH-COSY, HSQC): δ 7.87 – 7.82 (m, 4H, CH_{arom}), 7.52 – 7.45 (m, 3H, CH_{arom}), 4.77 (d, *J* = 12.3 Hz, 1H, CH*H* Nap), 4.65 (d, *J* = 12.3 Hz, 1H, CH*H* Nap), 3.92 (m, 1H, H-5), 3.73 (dd, *J* = 4.3, 4.8 Hz, 1H, H-4), 3.50 (m, 1H, H-3), 1.65 (m, 2H, H-2), 1.17 (d, 3H, *J* = 5.8 Hz, H-6), 1.00 (t, *J* = 7.5 Hz, 3H, H-1), 0.93 (s, 9H, $C(CH_3)_3$), 0.87 (s, 9H, $C(CH_3)_3$), 0.16 – 0.03 (m, 12H, $SiCH_3$, $SiCH_3$, $SiCH_3$, $SiCH_3$); ^{13}C NMR (75 MHz, $CDCl_3$, HSQC): δ 136.5, 133.3, 132.9 ($C_{q\text{-}arom}$), 127.9, 127.7, 126.2, 126.0, 125.9, 125.6 (CH_{arom}), 81.7 (C-3), 78.2 (C-4), 71.9 (CH_2 Nap), 69.6 (C-5), 26.1 ($C(CH_3)_3$), 26.0 ($C(CH_3)_3$), 22.3 (C-2), 19.6 (C-6), 10.1 (C-1), -4.2, -4.2, -4.4, -4.7 ($SiCH_3$); HRMS: $[M+H]^+$ calcd for $C_{29}H_{51}O_3Si_2$ 503.3377, found 503.3374.

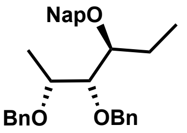

1,2,6-Trideoxy-3-*O*-(2-methylnaphthalene)-4,5-di-*O*-benzyl-D-altritol (S48). To a solution of compound **S47** (830 mg, 1.65 mmol) in pyridine (5 mL) was added a solution of HF-pyridine (5 mL, 5 mL of 70% HF-pyridine diluted with 5 mL of pyridine), and the reaction mixture was left stirring at rt for 16 h. The reaction mixture was diluted with CH_2Cl_2, washed with water, sat. $NaHCO_3$, and the organic phase was separated, dried over $MgSO_4$, filtered, and concentrated *in vacuo* to give the crude intermediate as an oil. This material was dissolved in DMF (10 mL), after which BnBr (600 μL, 5 mmol, 3.0 eq) and NaH (200 mg, 5.0 mmol, 3.0 eq) were added at 0 °C, and the reaction mixture was left stirring at room temperature for 4 h, after which it was quenched with methanol, concentrated *in vacuo*, loaded on silica gel, and chromatographed using hexane:EtOAc (90:10) as a mobile phase to give the desired product as a clear oil (710 mg, 91% over two steps). TLC: R_f 0.7 (pentane:EtOAc, 75:25); ^{1}H NMR (300 MHz, $CDCl_3$, HH-COSY, HSQC): δ 7.89 – 7.79 (m, 4H, CH_{arom}), 7.54 – 7.47 (m, 3H, CH_{arom}), 7.42 – 7.38 (m, 9H, CH_{arom}), 4.85 (d, *J* = 11.5 Hz, 1H, CH*H* Bn), 4.80 (d, *J* = 11.5 Hz, 1H, CH*H* Bn), 4.74 (s, 2H, CH_2Ar), 4.65 (d, *J* = 12.2 Hz, 1H, CH*H* Bn), 4.52 (d, *J* = 12.2 Hz, 1H, CH*H* Bn), 3.86 – 3.76 (m, 2H, H-4, H-5), 3.63 (m, 1H, H-3), 1.74 (m, 2H, H-2), 1.32 (d, *J* = 6.1 Hz, 2H, H-2), 0.99 (t, *J* = 7.4 Hz, 3H, H-1); ^{13}C NMR (75 MHz, $CDCl_3$) δ 138.9, 138.8, 136.2, 133.3, 132.9 ($C_{q\text{-}arom}$), 128.3, 128.3, 128.0, 128.0, 127.9, 127.7, 127.6, 127.5, 126.5, 126.1, 126.0, 125.8 (CH_{arom}), 81.6 (C-5), 80.5 (C-3), 75.5 (C-4), 73.9 (CH_2Ar), 71.8 (CH_2Ar), 70.8 (CH_2Ar), 22.7 (C-2), 15.4 (C-6), 9.8 (C-1); HRMS: $[M+H]^+$ calcd for $C_{31}H_{35}O_3$ 455.2586, found 455.2580.

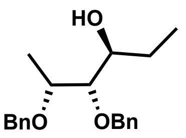

1,2,6-Trideoxy-4,5-di-*O*-benzyl-D-altritol (28). To a solution of **S48** (740 mg, 1.62 mmol) in CH_2Cl_2 (10 mL) was added water (1 mL) and DDQ (544 mg, 2.44 mmol, 1.5 eq) at rt. The reaction mixture was left stirring at that temperature for 1 h, after which it was quenched with sat. $NaHCO_3$. The organic phase was separated, dried over $MgSO_4$, and concentrated *in vacuo* to give a crude product. Column chromatography on silica gel using pentane:Et_2O (90:10) gave the title product as a clear oil (340 mg, 67%). TLC: R_f 0.7 (pentane:EtOAc, 75:25); ^1H NMR (300 MHz, $CDCl_3$, HH-COSY, HSQC): δ 7.39 – 7.29 (m, 10H, CH_{arom}), 4.77 (d, J = 11.4 Hz, 1H, CH*H*Bn), 4.69 (d, J = 5.5 Hz, 1H, CH*H*Bn), 4.65 (d, J = 5.5 Hz, 1H, CH*H*Bn), 4.51 (d, J = 11.4 Hz, 1H, CH*H*Bn), 3.83 (m, 1H, H-5), 3.71 (m, 1H, H-3), 3.42 (m, J = 5.1 Hz, 1H, H-4), 1.73 (m, 1H, H-2_a), 1.50 (m, 1H, H-2_b), 1.37 (d, J = 6.4 Hz, 3H, H-6), 1.00 (t, J = 7.0 Hz, 3H, H-1); ^{13}C NMR (75 MHz, $CDCl_3$): δ 128.4, 128.4, 127.9, 127.7 127.7, 127.6 (CH_{arom}), 84.1 (C-4), 76.6 (C-5), 74.2 (C-3), 74.1 (CH_2Ar), 70.7 (CH_2Ar), 26.0 (C-2), 16.1 (C-6), 10.3 (C-1); HRMS: $[M+H]^+$ calcd for $C_{20}H_{27}O_3$ 315.1960, found 319.1955.

Synthesis of acceptor 29

Scheme S8. Synthesis of compound **29**.

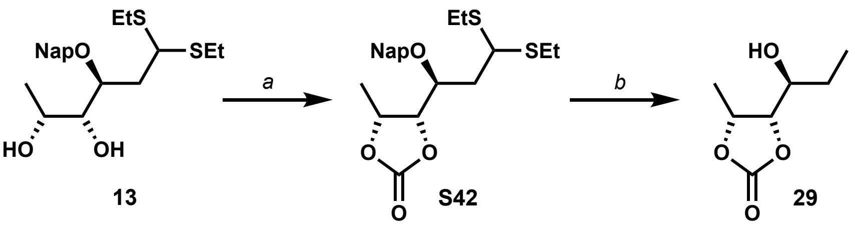

Reagents and conditions: a) $BH_3{\cdot}THF$, THF (75%); b) triphenylphosphine, imidazole, iodine, THF (92%); c) $LiAlH_4$, THF (72%); d) i: HF-pyridine, pyridine; ii: NaH, BnBr, DMF (91%); e) DDQ, DCM (67%).

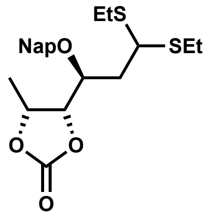

2,6-Dideoxy-1,1-diethyl-thioacetal-3-*O*-(2-methylnaphtalene)-4,5-*O*-carbonate-D-altritol (S42). A phosgene solution was prepared by diluting a 20% phosgene in hexane solution (1.35 mL) with dry THF (1.5 mL). **13** (190 mg, 0.50 mmol) was dissolved in THF (3.6 mL, 0.1 M) and Et_3N (346 µL, 2.5 mmol, 5.0 eq.) and cooled on ice. The phosgene solution was added dropwise after which the solution was stirred for 3 h at room temperature. The reaction was quenched by adding 1 mL of sat. aq. $NaHCO_3$ followed by diluting the mixture with Et_2O and water. The aqueous layer was extracted with Et_2O (3x) followed by washing the combined organic layers with H_2O, sat. aq. $NaHCO_3$ and brine respectively. Subsequently, the organic layer was dried over $MgSO_4$, filtered and concentrated *in vacuo* to yield the crude product as a colourless oil. Flash column chromatography (99:1 → 70:30; pentane:Et_2O) yielded the title compound (150 mg, 0.37 mmol, 74%) as a colourless oil. TLC: R_f 0.2 (pentane:Et_2O, 8:2, v:v); IR (neat, cm^{-1}): 817, 1092, 1125, 1348, 1804, 2870, 2928, 2970; ^1H NMR (500 MHz, $CDCl_3$, HH-COSY, HSQC): δ 7.92 – 7.38 (m, 7H, CH_{arom}), 4.98 – 4.87 (m, 1H, H-5), 4.85 (d, J = 11.5 Hz, 1H, CH*H*Nap), 4.76 (d, J = 11.5 Hz, 1H, CH*H*Nap), 4.72 (t, J = 7.1 Hz, 1H, H-4), 4.21 (ddd, J = 6.8, 6.1, 5.0 Hz, 1H, H-3), 3.98 (dd, J = 7.9, 6.8 Hz, 1H, H-1), 2.77 – 2.54 (m, 4H, SCH_2CH_3, SCH_2CH_3), 2.30 (ddd, J = 15.0, 6.8, 6.1 Hz, 1H, H-2), 2.18 (ddd, J = 15.0, 7.9, 5.0 Hz, 1H, H-2), 1.49 (d, J = 6.6 Hz, 3H, H-6), 1.25 (td, J = 7.4, 2.2 Hz, 6H, SCH_2CH_3, SCH_2CH_3); ^{13}C NMR (126 MHz, $CDCl_3$, HSQC): δ 154.1 (O(C=O)O),

134.8, 133.3, 133.2 ($C_{q\text{-arom}}$), 128.5, 128.0, 127.8, 126.8, 126.5, 126.3, 125.7 (CH_{arom}), 80.1 (C-4), 76.2 (C-5), 74.6 (C-3), 72.5 (CH_2 Nap), 47.3 (C-1), 38.5 (C-2), 24.5 (S*C*H_2CH_3), 24.1 (S*C*H_2CH_3), 15.2 (C-6), 14.4 (SCH_2*C*H_3); HRMS: $[M+Na]^+$ calcd for $C_{22}H_{28}O_4NaS_2$ 443.1321, found 443.1320.

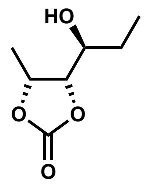

1,2,6-trideoxy-4,5-*O*-carbonate-D-altritol (29). S49 was converted to **29** according to a modified literature procedure.[Sommer, R.; Exner, T. E.; Titz, A. A Biophysical Study with Carbohydrate Derivatives Explains the Molecular Basis of Monosaccharide Selectivity of the Pseudomonas Aeruginosa Lectin LecB. *PLOS ONE* **2014**, 9 (11)] **S49** (100 mg, 0.24 mmol) was dissolved in 3 mL EtOH and 1 mL H_2O, followed by the addition of sodium hypophosphite monohydrate (0.25 g, 2.38 mmol, 10 eq.) in 1 mL EtOH. Subsequently, 20 spoon tips of pre-washed (with H_2O; pH ± 7) Raney®-Nickel (Sigma-Aldrich, W.R. Grace and Co. Raney® 2800, slurry, in H_2O, active catalyst) was added. The resulting suspension was stirred for 16 h at room temperature and the work-up was performed by filtration over Celite© Hyflo Supercel (Merck). After washing the Celite© with EtOH and H_2O, the filtrate was diluted with DCM. The aqueous layer was extracted with DCM (3x) followed by washing the combined organic layers with H_2O, sat. aq. $NaHCO_3$ and brine respectively. Subsequently, the organic layer was dried over $MgSO_4$, filtered and concentrated *in vacuo* to yield the crude product as a colourless oil. Flash column chromatography (10:90 → 40:60; pentane: EtOAc) yielded the title compound (33 mg, 0.21 mmol, 87%) as a colourless oil. TLC: R_f 0.3 (pentane:EtOAc, 8:2, v:v); IR (neat, cm^{-1}): 810, 1080, 1120, 1320, 1803, 2879, 2928; ^1H NMR (500 MHz, $CDCl_3$, HH-COSY, HSQC): δ 4.94 (p, *J* = 6.7 Hz, 1H, H-5), 4.41 (dd, *J* = 9.0, 7.2 Hz, 1H, H-4), 3.82 (tdd, *J* = 8.6, 5.3, 3.1 Hz, 1H, H-3), 1.87 (dqd, *J* = 14.4, 7.6, 3.0 Hz, 1H, H-2), 1.66 (d, *J* = 5.5 Hz, 1H, 3-OH), 1.59 – 1.51 (m, 1H, H-2), 1.51 (d, *J* = 6.6 Hz, 3H, H-6), 1.05 (t, *J* = 7.5 Hz, 3H, H-1); ^{13}C NMR (126 MHz, $CDCl_3$, HSQC): δ 154.3 (O(C=O)O), 80.0 (C-4), 76.4 (C-5), 69.9 (C-3), 27.4 (C-2), 15.0 (C-6), 8.9 (C-1); HRMS: $[M+Na]^+$ calcd for $C_7H_{12}O_4Na$ 183.0628, found 183.0623.

Model glycosylation reactions

Results of compound 4

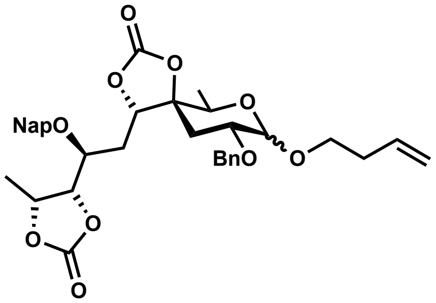

3-Butene 2-*O*-benzyl-4,7,10,11-di-*O*-carbonate-9-*O*-(2-methylnaphthalene)-α-D-caryophylloside (S51). The title compound was prepared according to general procedure III (30.6 mg, 48 µmol, 97%, α:β; 63:37). Flash column chromatography (80:20 → 60:40; pentane:EtOAc) yielded the title compound as a white foam. TLC: R_f 0.6 (pentane:EtOAc, 6:4, v:v); IR (neat, cm^{-1}): 1055, 1202, 1797; NMR data reported as a mixture of α- and β-anomers; ^1H NMR (500 MHz, $CDCl_3$, HH-COSY, HSQC, HMBC): ^1H NMR (500 MHz, $CDCl_3$, HH-COSY, HSQC): δ 7.89 – 7.27 (m, 19.2H, CH_{arom}), 5.89 – 5.76 (m, 1.6H, H-15_α, H-15_β), 5.23 – 4.97 (m, 3.2H, H-16_α, H-16_β), 4.92 – 4.86 (m, 1.6H, H-11_α, H-11_β), 4.79 – 4.75 (m, 4.2H, H-1_α, CH_2 Bn/Nap_α, CH_2 Bn/Nap_β), 4.65 – 4.60 (m, 1.6H, H-10_α, H-10_β), 4.57 – 4.43 (m, 3.8H, C*H*H Bn/Nap_α, C*H*H Bn/Nap_β, CH*H* Bn/Nap_α, CH*H* Bn/Nap_β, H-7_β), 4.36 – 4.30 (m, 1.6H, H-1_β, H-7_α), 4.05 – 3.88 (m, 3.2H, H-5_α, H-9_α, H-9_β, H-13_β), 3.77 – 3.51 (m, 4.8H, H-2_α, H-2_β, H-5_β, H-13_α, H-13_α, H-

$13_β$), 2.45 – 2.32 (m, 3.2H, H-$14_α$, H-$14_β$), 2.16 (dd, J = 14.3, 5.1 Hz, 0.6H, H-$3_β$), 2.11 – 2.02 (m, 3.2H, H-$3_α$, H-$8_α$, H-$8_β$, H-$8_β$), 1.92 (ddd, J = 14.9, 8.5, 2.0 Hz, 1H, H-$8_α$), 1.83 (dd, J = 13.5, 4.8 Hz, 1H, H-$3_α$), 1.74 (dd, J = 14.3, 10.2 Hz, 0.6H, H-$3_β$), 1.47 – 1.41 (m, 4.8H, H-$12_α$, H-$12_β$), 1.33 (d, J = 6.3 Hz, 1.8H, H-$6_β$), 1.22 (d, J = 6.3 Hz, 3H, H-$6_α$); ^{13}C NMR (126 MHz, $CDCl_3$, HSQC): δ 153.7, 153.6, 153.5, 153.3 (O(C=O)O), 138.1, 137.9 ($C_{q\text{-arom}}$), 135.2 (C-$15_α$), 135.0 (C-$15_β$), 134.2, 134.1, 133.3, 133.3 ($C_{q\text{-arom}}$), 132.4, 128.8, 128.8, 128.6, 128.6, 128.1, 128.1, 128.1, 127.9, 127.9, 127.9, 127.9, 127.0, 126.8, 126.7, 126.7, 126.6, 125.6, 125.5 (CH_{arom}), 117.1 (C-$16_α$), 116.9 (C-$16_β$), 103.6 (C-$1_β$), 95.6 (C-$1_α$), 84.9 (C-$4_α$), 84.0 (C-$4_β$), 81.0 (C-$7_α$), 80.2 (C-$7_β$), 78.9 (C-$10_α$), 78.7 (C-$10_β$), 75.8 (C-$11_β$), 75.8 (C-$11_α$), 73.9 (C-$9_β$), 73.8 (C-$9_α$), 73.7, 73.5 (CH_2 Bn/Nap), 73.3 (C-$2_β$), 73.2 (CH_2 Bn/Nap), 71.5 (C-$2_α$), 68.7 (C-$13_β$), 67.7 (C-$13_α$), 64.9 (C-$5_α$), 37.6 (C-$3_β$), 34.2 (C-$14_β$), 34.0 (C-$14_α$), 33.7 (C-$3_α$), 29.7 (C-$8_α$), 29.4 (C-$8_β$), 15.6 (C-$6_β$), 15.3 (C-$12_α$), 15.2 (C-$12_β$), 14.9 (C-$6_α$); HRMS: $[M+Na]^+$ calcd for $C_{36}H_{40}O_{10}Na$ 655.2519, found 655.2514.

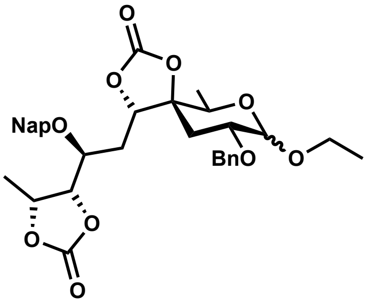

Ethyl 2-*O*-benzyl-4,7,10,11-di-*O*-carbonate-9-*O*-(2-methylnaphthalene)-D-caryophylloside (S52). The title compound was prepared according to general procedure III (28.5 mg, 47 µmol, 94%, α:β; 67:33). Flash column chromatography (80:20 → 50:50; pentane:EtOAc) yielded the title compound as a colorless oil. TLC: R_f 0.3 (pentane:EtOAc, 7:3, v:v); IR (neat, cm^{-1}): 756, 1059, 1090, 1202, 1382, 1802, 2929; NMR data reported as a mixture of α- and β-anomers; ^1H NMR (500 MHz, $CDCl_3$, HH-COSY, HSQC): δ 7.87 – 7.26 (m, 18H, CH_{arom}), 4.95 – 4.86 (m, 1.5H, H-$11_α$, H-$11_β$), 4.79 – 4.76 (m, 4H, H-$1_α$, CH_2 Bn/$Nap_α$, CH_2 Bn/$Nap_β$), 4.66 – 4.61 (m, 1.5H, H-$10_α$, H-$10_β$), 4.58 – 4.54 (m, 1.5H, C*H*H Bn/$Nap_α$, C*H*H Bn/$Nap_β$), 4.51 – 4.47 (m, 2H, CH*H* Bn/$Nap_α$, CH*H* Bn/$Nap_β$, H-$7_β$), 4.37 – 4.31 (m, 1.5H, H-$1_β$, H-$7_α$), 4.05 – 3.97 (m, 1.5H, H-$9_α$, H-$9_β$), 3.94 (dd, J = 9.5, 7.1 Hz, 1H, $CH_2CH_{3β}$), 3.89 (q, J = 6.4 Hz, 1H, H-$5_α$), 3.78 – 3.69 (m, 2.5H, H-$2_α$, H-$5_β$, $CH_2CH_{3α}$), 3.60 – 3.50 (m, 2H, H-$2_β$, $CH_2CH_{3α}$, $CH_2CH_{3β}$), 2.20 – 2.04 (m, 3H, H-$3_α$, H-$3_β$, H-$8_α$, H-$8_β$), 1.95 (ddd, J = 14.9, 8.6, 2.1 Hz, 1H, H-$8_α$), 1.84 (dd, J = 13.5, 4.8 Hz, 1H, H-$3_α$), 1.75 (dd, J = 14.3, 10.1 Hz, 0.5H, H-$3_β$), 1.47 – 1.42 (m, 4.5H, H-$12_α$, H-$12_β$), 1.34 (d, J = 6.3 Hz, 1.5H, H-$6_β$), 1.26 (m, 1.28 – 1.24, 4.5H, $CH_2CH_{3α}$, $CH_2CH_{3β}$), 1.23 (d, J = 6.3 Hz, 3H, H-$6_α$); ^{13}C NMR (126 MHz, $CDCl_3$, HSQC): δ 153.7 153.7, 153.5, 153.3 (O(C=O)O), 138.2, 137.9, 134.2, 134.1, 133.3 ($C_{q\text{-arom}}$), 128.8, 128.8, 128.7, 128.6, 128.6, 128.2, 128.1, 128.1, 128.1, 128.0, 127.9, 127.9, 127.9, 127.0, 126.8, 126.7, 126.7, 126.7, 126.6, 126.6, 125.6, 125.4 (CH_{arom}), 103.3 (C-$1_β$), 95.3 (C-$1_α$), 84.9 (C-$4_α$), 84.0 (C-$4_β$), 80.9 (C-$7_α$), 80.3 (C-$7_β$), 79.0 (C-$10_α$), 78.8 (C-$10_β$), 75.8 (C-$11_α$), 75.8 (C-$11_β$), 73.9 (C-$9_β$), 73.8 (C-$9_α$), 73.7 (CH_2 Bn/$Nap_α$), 73.5 (CH_2 Bn/$Nap_β$), 73.4 (C-$2_β$), 73.2 (CH_2 Bn/$Nap_β$), 71.5 (CH_2 Bn/$Nap_α$), 71.4 (C-$5_β$), 71.4 (C-$2_α$), 65.0 ($CH_2CH_{3β}$), 64.7 (C-$5_α$), 63.9 ($CH_2CH_{3α}$), 37.5 (C-$3_β$), 33.7 (C-$3_α$), 29.7 (C-$8_α$), 29.5 (C-$8_β$), 15.6 (C-$6_β$), 15.3 (C-$12_β$), 15.3 (C-$12_α$), 15.2 ($CH_2CH_{3α}$, $CH_2CH_{3β}$), 15.0 (C-$6_α$); HRMS: $[M+Na]^+$ calcd for $C_{34}H_{36}O_{10}Na$ 629.2363, found 629.2357.

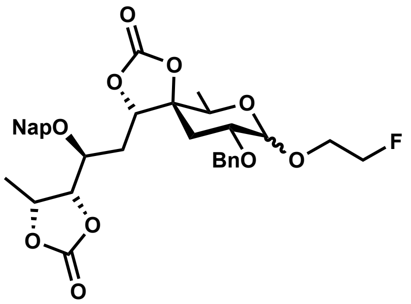

2-Fluoroethyl 2-*O*-benzyl-4,7,10,11-di-*O*-carbonate-9-*O*-(2-methylnaphthalene)-D-caryophylloside (S53). The title compound was prepared according to general procedure III (31 mg, 50 µmol, *quant.*, α:β; 83:17). Flash column chromatography (60:40 → 40:60; pentane:EtOAc) yielded the title compound as a colorless oil. TLC: R_f 0.4 (pentane:EtOAc, 1:1, v:v); IR (neat, cm^{-1}): 700, 753, 819, 1070, 1202, 1455, 1802; NMR data reported as a mixture of α- and β-anomers; ^{1}H NMR (500 MHz, $CDCl_3$, HH-COSY, HSQC): δ 7.89 – 7.27 (m, 14.4H, CH_{arom}), 4.99 – 4.85 (m, 1.2H, H-11$_α$, H-11$_β$), 4.81 (d, *J* = 3.2 Hz, 1H, H-1$_α$), 4.78 (s, 2H, CH_2 Bn/Nap$_α$), 4.74 – 4.46 (m, 6.6H, H-7$_β$, H-10$_α$, H-10$_β$, $CH_2F_α$, $CH_2F_β$, CH_2 Bn/Nap$_α$, CH_2 Bn/Nap$_β$, CH_2 Bn/Nap$_β$), 4.42 (d, *J* = 6.6 Hz, 0.2H, H-1$_β$), 4.32 (d, *J* = 11.0 Hz, 1H, H-7$_α$), 4.08 (dd, *J* = 12.5, 4.1 Hz, 0.2H, H-9$_β$), 4.06 – 3.95 (m, 2H, H-5$_α$, H-9$_α$), 3.95 – 3.66 (m, 3.6H, H-2$_α$, H-5$_β$, C*H*$_2$CH$_2$F$_α$, C*H*$_2$CH$_2$F$_β$), 3.61 (dt, *J* = 10.8, 5.8 Hz, 0.2H, H-2$_β$), 2.22 – 2.15 (m, 0.4H, H-3$_β$, H-8$_β$), 2.14 – 2.04 (m, 2.2H, H-3$_α$, H-8$_α$, H-8$_β$), 1.95 (dd, *J* = 14.8, 8.2 Hz, 1H, H-8$_α$), 1.84 (dd, *J* = 13.6, 4.9 Hz, 1H, H-3$_α$), 1.76 (dd, *J* = 14.4, 10.1 Hz, 0.2H, H-3$_β$), 1.46 (d, *J* = 6.7 Hz, 3H, H-12$_α$), 1.44 (d, *J* = 6.7 Hz, 0.6H, H-12$_β$), 1.34 (d, *J* = 6.2 Hz, 0.6H, H-6$_β$), 1.23 (d, *J* = 6.3 Hz, 3H, H-6$_α$); ^{13}C NMR (126 MHz, $CDCl_3$, HSQC): δ 153.7, 153.7, 153.5, 153.2 (O(C=O)O), 138.0, 137.8, 134.2, 134.1, 133.8, 133.3 ($C_{q\text{-}arom}$), 128.8, 128.8, 128.7, 128.7, 128.6, 128.2, 128.2, 128.1, 128.1, 128.1, 128.0, 128.0, 128.0, 127.9, 127.2, 127.0, 126.8, 126.8, 126.7, 126.7, 126.6, 126.6, 125.7, 125.6, 125.5 (CH_{arom}), 103.6 (C-1$_β$), 95.6 (C-1$_α$), 84.9 (C-4$_β$), 84.7 (C-4$_α$), 82.8 (d, *J* = 169.5 Hz, $CH_2F_α$), 82.7 (d, *J* = 169.8 Hz, $CH_2F_β$), 80.9 (C-7$_α$), 80.3 (C-7$_β$), 79.0 (C-10$_α$), 78.7 (C-10$_β$), 75.9 (C-11$_α$), 75.8 (C-11$_β$), 73.9 (C-9$_α$), 73.8 (C-9$_β$), 73.7 (CH_2 Bn/Nap$_α$), 73.5, 73.3 (CH_2 Bn/Nap$_β$), 73.2 (C-2$_β$), 71.6 (CH_2 Bn/Nap$_α$), 71.4 (C-2$_α$), 68.3 (d, *J* = 19.8 Hz, *C*H$_2$CH$_2$F$_β$), 67.0 (d, *J* = 19.5 Hz, *C*H$_2$CH$_2$F$_α$), 66.7 (C-5$_β$), 64.8 (C-5$_α$), 37.4 (C-3$_β$), 33.5 (C-3$_α$), 29.6 (C-8$_β$), 29.5 (C-8$_α$), 15.6 (C-12$_β$), 15.2 (C-12$_α$), 15.2 (C-6$_α$), 14.9 (C-12$_β$); HRMS: $[M+Na]^+$ calcd for $C_{34}H_{37}FO_{10}Na$ 647.2268, found 647.2263.

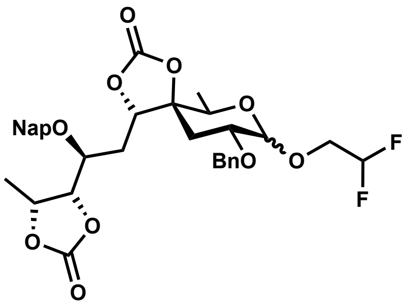

2,2-Difluoroethyl 2-*O*-benzyl-4,7,10,11-di-*O*-carbonate-9-*O*-(2-methylnaphthalene)-D-caryophylloside (S54). The title compound was prepared according to general procedure III (20.1 mg, 31 µmol, 63%, α:β; 87:13). Flash column chromatography (80:20 → 60:40; pentane:EtOAc) yielded the title compound as a colorless oil. TLC: R_f 0.7 (pentane:EtOAc, 1:1, v:v); IR (neat, cm^{-1}): 700, 754, 819, 1063, 1202, 1364, 1802; Data of the major stereoisomer (α-anomer): ^{1}H NMR (400 MHz, $CDCl_3$, HH-COSY, HSQC): δ 7.93 – 7.28 (m, 12H, CH_{arom}), 5.95 (tt, *J* = 55.3, 4.2 Hz, 1H, CHF_2), 4.93 (h, *J* = 6.6 Hz, 1H, H-11), 4.82 – 4.74 (m, 3H, H-1, CH_2 Bn/Nap), 4.62 (t, *J* = 7.0 Hz, 1H, H-10), 4.57 (d, *J* = 12.1 Hz, 1H, C*H*H Bn/Nap), 4.48 (d, *J* = 12.0 Hz, 1H, CH*H* Bn/Nap), 4.33 (dd, *J* = 11.1, 2.1 Hz, 1H, H-7), 4.03 (ddd, *J* = 8.2, 6.5, 3.2 Hz, 1H, H-9), 3.91 (q, *J* = 6.3 Hz, 1H, H-5), 3.82 – 3.69 (m, 3H, H-2, C*H*$_2$CHF$_2$), 2.15 – 2.01 (m, 2H, H-3, H-8), 1.93 (ddd, *J* = 14.7, 8.1, 2.1 Hz, 1H, H-8), 1.85 (dd, *J* = 13.6, 4.9 Hz, 1H, H-3), 1.46 (d, *J* = 6.6 Hz, 3H, H-12), 1.24 (d, *J* = 6.3 Hz, 3H, H-6); ^{13}C NMR (126 MHz, $CDCl_3$, HSQC): δ 153.6, 153.3 (O(C=O)O), 137.7, 134.1, 133.3, 133.3 ($C_{q\text{-}arom}$), 128.8, 128.7, 128.7, 128.3, 128.1, 128.0,

127.9, 127.9, 126.9, 126.7, 126.6, 125.5 (CH_{arom}), 114.1 (t, J = 241.2 Hz, CHF_2), 96.3 (C-1), 84.5 (C-4), 80.9 (C-7), 78.8 (C-10), 75.8 (C-11), 73.8 (C-9), 73.7, 71.8 (CH_2 Bn/Nap), 71.3 (C-2), 67.1 (t, J = 28.2 Hz, CH_2CHF_2), 65.3 (C-5), 33.4 (C-3), 29.5 (C-8), 15.2 (C-12), 14.9 (C-6); Diagnostic signals of the minor stereoisomer (β-anomer): ^{1}H NMR (400 MHz, $CDCl_3$, HH-COSY, HSQC): δ 5.91 (tt, J = 55.4, 4.1 Hz, 1H, CHF_2), 3.59 (q, J = 6.4 Hz, 1H, H-5), 1.41 (d, J = 6.6 Hz, 3H, H-12), 1.11 (d, J = 6.4 Hz, 3H, H-6); ^{13}C NMR (126 MHz, $CDCl_3$, HSQC): δ 96.7 (C-1), 84.7 (C-4), 78.9 (C-10), 72.7 (C-9), 71.9, 71.9 (CH_2 Bn/Nap), 71.6 (C-2), 31.3 (C-3), 29.8 (C-8), 14.8 (C-12), 13.3 (C-6); HRMS: $[M+Na]^+$ calcd for $C_{34}H_{36}F_2O_{10}Na$ 665.2174, found 665.2169.

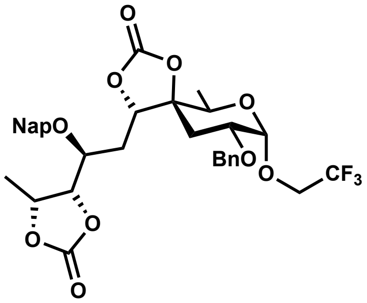

2,2,2-Trifluoroethyl 2-*O*-benzyl-4,7,10,11-di-*O*-carbonate-9-*O*-(2-methylnaphthalene)-α-D-caryophylloside (S55). The title compound was prepared according to general procedure III (25 mg, 38 µmol, 76%, α:β; >98:2). Flash column chromatography (90:10 → 70:30; pentane:EtOAc) yielded the title compound as a colorless oil. TLC: R_f 0.8 (pentane:EtOAc, 1:1, v:v); $[\alpha]_D^{20}$ 21.0° (c 1.0, $CHCl_3$); IR (neat, cm^{-1}): 701, 753, 819, 1067, 1155, 1279, 1804; ^{1}H NMR (400 MHz, $CDCl_3$, HH-COSY, HSQC): δ 7.91 – 7.27 (m, 12H, CH_{arom}), 4.91 (p, J = 6.8 Hz, 1H, H-11), 4.83 (d, J = 3.3 Hz, 1H, H-1), 4.78 (d, J = 2.5 Hz, 2H, CH_2 Bn/Nap), 4.62 (dd, J = 7.3, 6.5 Hz, 1H, H-10), 4.55 (d, J = 11.8 Hz, 1H, C*H*H Bn/Nap), 4.49 (d, J = 11.8 Hz, 1H, CH*H* Bn/Nap), 4.32 (dd, J = 11.2, 2.1 Hz, 1H, H-7), 4.03 (ddd, J = 8.5, 6.5, 3.1 Hz, 1H, H-9), 3.99 – 3.85 (m, 3H, H-5, CH_2CF_3), 3.78 (ddd, J = 11.7, 4.9, 3.3 Hz, 1H, H-2), 2.14 – 2.00 (m, 2H, H-3, H-8), 1.92 (ddd, J = 14.9, 8.5, 2.1 Hz, 1H, H-8), 1.85 (dd, J = 13.7, 4.9 Hz, 1H, H-3), 1.46 (d, J = 6.7 Hz, 3H, H-12), 1.24 (d, J = 6.4 Hz, 3H, H-6); ^{13}C NMR (101 MHz, $CDCl_3$, HSQC): δ 153.6, 153.3 (O(C=O)O), 137.6, 134.2, 133.3, 133.3 ($C_{q\text{-}arom}$), 128.9, 128.7, 128.7, 128.2, 128.1, 128.0, 127.9, 126.8, 126.8, 126.6, 125.5 (CH_{arom}), 123.8 (d, J = 278.7 Hz, CF_3), 96.4 (C-1), 84.3 (C-4), 81.0 (C-7), 79.0 (C-10), 75.8 (C-11), 73.9 (C-9), 73.8, 71.7 (CH_2 Bn/Nap), 71.1 (C-2), 65.7 (C-5), 65.1 (d, J = 35.0 Hz, CH_2CF_3), 33.3 (C-3), 29.7 (C-8), 15.3 (C-12), 14.9 (C-6); HRMS: $[M+Na]^+$ calcd for $C_{34}H_{35}F_3O_{10}Na$ 683.2080, found 683.2075.

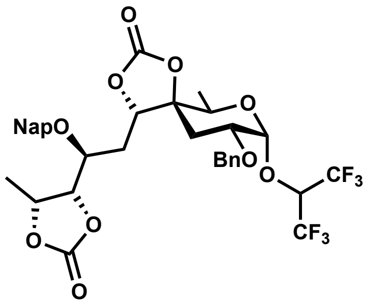

1,1,1,3,3,3-Hexafluoropropyl 2-*O*-benzyl-4,7,10,11-di-*O*-carbonate-9-*O*-(2-methylnaphthalene)-α-D-caryophylloside (S56). The title compound was prepared according to general procedure III (5.2 mg, 7.2 µmol, 16%, α:β; >98:2). Flash column chromatography (90:10 → 70:30; pentane:EtOAc) yielded the title compound as a colorless oil. TLC: R_f 0.8 (pentane:EtOAc, 1:1, v:v); IR (neat, cm^{-1}): 700, 754, 819, 1066, 1204, 1311, 1803; ^{1}H NMR (500 MHz, $CDCl_3$, HH-COSY, HSQC): δ 7.89 – 7.27 (m, 12H, CH_{arom}), 5.10 (d, J = 3.4 Hz, 1H, H-1), 4.92 (p, J = 6.8 Hz, 1H, H-11), 4.81 (d, J = 11.6 Hz, 1H, C*H*H Bn/Nap), 4.75 (d, J = 11.6 Hz, 1H, CH*H* Bn/Nap), 4.59 (t, J = 7.0 Hz, 1H, H-10), 4.55 (d, J = 11.5 Hz, 1H, C*H*H Bn/Nap), 4.52 – 4.43 (m, 2H, CH*H* Bn/Nap, $CH(CF_3)_2$), 4.31 (dd, J = 11.5, 2.0 Hz, 1H, H-7), 4.04 (ddd, J = 9.3, 6.7, 3.1 Hz, 1H, H-9), 3.99 (q, J = 6.2 Hz, 1H, H-5), 3.82 (ddd, J = 11.4, 4.7, 3.6 Hz, 1H, H-2), 2.07 – 1.99 (m, 2H, H-3, H-8), 1.92 – 1.87 (m, 1H, H-8), 1.84 (dd, J = 13.8, 4.7 Hz, 1H, H-3), 1.47 (d, J

= 6.7 Hz, 3H, H-12), 1.25 – 1.23 (m, 3H, H-6); ^{13}C NMR (126 MHz, $CDCl_3$, HSQC): δ 137.3, 134.2, 133.3, 133.3 ($C_{q\text{-}arom}$), 128.9, 128.7, 128.3, 128.1, 127.9, 127.8, 126.9, 126.8, 126.7, 125.5 (CH_{arom}), 97.9 (C-1), 84.0 (C-4), 81.2 (C-7), 79.0 (C-10), 75.8 (C-11), 74.0 (C-9), 74.0, 71.8 (CH_2 Bn/Nap), 70.5 (C-2), 66.7 (C-5), 33.3 (C-3), 29.8 (C-8), 15.3 (C-12), 14.8 (C-6); HRMS: $[M+Na]^+$ calcd for $C_{35}H_{34}F_6O_{10}Na$ 751.1954, found 751.1948.

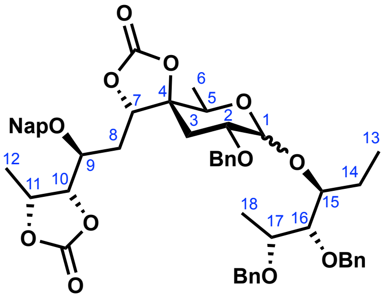

1,2,6-Trideoxy-4,5-di-*O*-benzyl-D-altritol-2-*O*-benzyl-4,7,10,11-di-*O*-carbonyl-9-*O*-(2-methylnaphthalene)-α-D-caryophylloside (S57). The title compound was prepared according to the general procedure III giving the product as a white solid (11.5 mg, 50%, α:β; 77:23) TLC: R_f 0.8 (pentane:EtOAc, 3:2, v:v); Data of the major stereoisomer (α-anomer): ^1H NMR (600 MHz, $CDCl_3$, HH-COSY, HSQC): δ 7.87 – 7.14 (m, 22H, CH_{arom}), 4.98 (d, J = 3.2 Hz, 1H, H-1), 4.79 (m, 1H, H-11), 4.76 – 4.64 (m, 2H, CH_2 Bn/Nap), 4.56 – 4.48 (m, 2H, CH_2 Bn/Nap), 4.37 (t, J = 6.8 Hz, H-10), 4.27 (dd, J = 11.6, 1.6 Hz, 1H, H-7), 4.22 (q, J = 6.8 Hz, 1H, H-17), 3.92 (m, 1H, H-9), 3.85 (m, 1H, H-15), 3.78 (m, 1H, H-2), 3.67 (m, 1H, H-16), 2.22 (m, 1H, H-8), 2.04 –1.96 (m, 2H, H-3, H-8), 1.87 (dd, J = 13.6, 4.5 Hz, 1H, H-3), 1.82 (m, 1H, H-2), 1.43 (d, J = 6.4 Hz, 3H, H-12), 1.41 (d, J = 6.4 Hz, 3H, H-6), 1.07 (d, J = 6.4 Hz, 3H, H-18), 0.95 (t, J = 8.2 Hz, 1H, H-13); ^{13}C NMR (101 MHz, $CDCl_3$, HSQC): δ 153.5, 153.4, 138.7, 137.8, 134.0, 133.2 ($C_{q\text{-}arom}$), 128.7, 128.5, 128.4, 128.0, 128.0, 127.9, 127.8, 127.6, 127.6, 127.5, 127.5, 127.0, 126.5, 126.4, 125.6 (CH_{arom}), 93.0 (C-1), 82.2 (C-16), 80.6 (C-7), 78.3 (C-10), 78.2, 75.7 (CH_2 Bn/Nap), 75.6 (C-11), 75.0 (C-15), 73.5 (C-9), 71.4 (C-2), 70.8 (CH_2 Bn/Nap), 64.8 (C-5), 33.7 (C-3), 29.7 (C-8), 22.7 (C-14), 16.3 (C-6), 15.0 (C-12), 14.9 (C-18), 10.0 (C-13); Diagnostic signals of the minor stereoisomer (β-anomer): ^1H NMR (600 MHz, $CDCl_3$, HH-COSY, HSQC): δ 4.45 (d, J = 7.0 Hz, 1H, H-1); ^{13}C NMR (126 MHz, $CDCl_3$, HSQC): δ 102.3 (C-1); HRMS: $[M+Na]^+$ calcd for $C_{52}H_{58}O_{12}N_a$ 897.3826, found 897.3816.

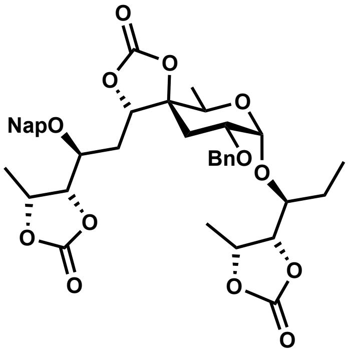

1,2,6-Trideoxy-4,5-di-*O*-carbonyl-D-altritol-2-*O*-benzyl-4,7,10,11-di-*O*-carbonyl-9-*O*-(2-methylnaphthalene)-α-D-caryophylloside (S58). The title compound was prepared according to the general procedure III giving the product as a white solid (12.0 mg, 54%, α:β; >98:2). TLC: R_f 0.4 (pentane:EtOAc, 3:2, v:v); ^1H NMR (600 MHz, $CDCl_3$, HH-COSY, HSQC): δ 7.88 – 7.89 (m, 4H, CH_{arom}), 7.39 (m, 2H, CH_{arom}), 7.44 (m, 2H, CH_{arom}), 7.37 – 7.30 (m, 4H, CH_{arom}), 5.01 – 4.95 (m, 3H, H-1, H-5, H-11), 4.90 (d, J = 11.9 Hz, 1H, C*H*H Bn/Nap), 4.79 (d, J = 11.9 Hz, 1H, C*H*H Bn/Nap), 4.71 (m, 2H, H-10, H-16), 4.52 (d, J = 11.9 Hz, 1H, C*H*H Bn/Nap), 4.35 (dd, J = 11.8, 1.42 Hz, 1H, H-7), 4.16 (m, 1H, H-9), 4.00 (m, 2H, H-17, H-15), 3.81 (m, 1H, H-2), 2.22 (m, 1H, H-8), 2.04 -1.96 (m, 2H, H-3, H-8), 1.87 (dd, J = 13.6, 4.5 Hz, 1H, H-3), 1.82 (m, 1H, H-14), 1.66 (d, J = 6.3 Hz, H-6), 1.60 (m, 3H, H-14), 1.56 (d, J = 7.1 Hz, 1H, H-12), 1.27 (d, J = 7.1 Hz, 1H, H-18), 1.02 (t, J = 8.2 Hz, 1H, H-13); ^{13}C NMR (151

MHz, $CDCl_3$, HSQC): δ 155.8, 155.0, 154.4, 138.7, 135.6, 134.2, 134.1($C_{q\text{-}arom}$), 129.5, 129.5, 129.0, 128.9, 128.7, 128.5, 127.8, 127.4, 127.3, 126.6 (CH_{arom}), 93.4 (C-1), 82.3 (C-7), 80.5 (C-16), 79.6 (C-10), 78.1 (C-15), 77.3 (C-11/C-17) 77.2 (C-17/C-11), 75.0 (C-9), 74.7, 72.3 (CH_2 Bn/Nap), 72.2 (C-2), 66.6 (C-5), 34.1 (C-3), 30.0 (C-8), 23.0 (C-14), 16.4 (C-12), 16.3 (C-6), 15.9 (C-18), 11.5 (C-13); HRMS: $[M+Na]^+$ calcd for $C_{39}H_{44}O_{13}N_a$ 743.2680, found 743.2672.

Results of compound 23

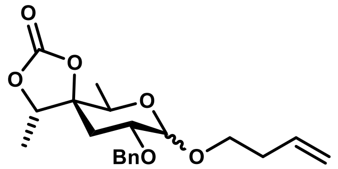

But-3-ylene 2-*O*-benzyl-4,7-carbonate-D-yersinioside (S59). The title compound was prepared according to general procedure III (15.6 mg, 43 µmol, 86%, α:β; 59:41) as a colorless oil. The title compound was also prepared according to general procedure IV (10 mg, 27 µmol, 55%, α:β; 67:33). The title compound was also prepared according to general procedure V (11 mg, 30 µmol, 61%, α:β; >98:2). TLC: R_f 0.4 (pentane:EtOAc, 8:2, v:v); $[\alpha]_D^{20}$ 28.3° (c 0.5, $CHCl_3$; α-anomer); IR (neat, cm^{-1}): 746, 1008, 1066, 1089, 1808, 2925; Data of the α-anomer: ^1H NMR (400 MHz, $CDCl_3$): δ 7.39 – 7.28 (m, 5H, CH_{arom}), 5.83 (ddt, *J* = 17.1, 10.2, 6.8 Hz, 1H, H-11), 5.13 (dq, *J* = 17.2, 1.7 Hz, 1H, H-12), 5.06 (ddt, *J* = 10.2, 2.1, 1.2 Hz, 1H, H-12), 4.78 (d, *J* = 3.3 Hz, 1H, H-1), 4.61 (d, *J* = 12.1 Hz, 1H, C*H*H Bn), 4.56 (d, *J* = 12.0 Hz, 1H, CH*H* Bn), 4.33 (q, *J* = 6.9 Hz, 1H, H-5), 3.95 (q, *J* = 6.3 Hz, 1H, H-7), 3.81 (ddd, *J* = 11.8, 5.0, 3.3 Hz, 1H, H-2), 3.73 – 3.51 (m, 2H, H-9), 2.55 – 2.28 (m, 2H, H-10), 2.11 (dd, *J* = 13.5, 11.8 Hz, 1H, H-3), 1.97 (dd, *J* = 13.5, 4.9 Hz, 1H, H-3), 1.43 (d, *J* = 6.9 Hz, 3H, H-6), 1.25 (d, *J* = 6.3 Hz, 3H, H-8); ^{13}C NMR (101 MHz, $CDCl_3$): δ 154.2 (O(C=O)O), 138.0 ($C_{q\text{-}arom}$), 135.2 (C-11), 129.5, 128.7, 128.1, 127.9 (CH_{arom}), 117.0 (C-12), 95.6 (C-1), 84.9 (C-4), 81.5 (C-5), 71.8 (CH_2 Bn), 71.7 (C-2), 67.6 (C-9), 65.0 (C-7), 34.1 (C-10), 33.7 (C-3), 14.7 (C-8), 13.1 (C-6); Diagnostic signals of the β-anomer: ^1H NMR (500 MHz, $CDCl_3$): δ 4.36 (d, *J* = 7.2 Hz, 1H, H-1); ^{13}C NMR (126 MHz): δ 104.1 (C-1), 73.8 (CH_2 Bn), 68.9 (C-9); HRMS: $[M+Na]^+$ calcd for $C_{20}H_{26}O_6Na$ 385.1627, found 385.1622.

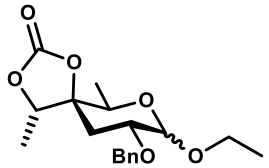

Ethyl 2-*O*-benzyl-4,7-carbonate-D-yersinioside (S60). The title compound was prepared according to general procedure III (10 mg, 30 µmol, 60%, α:β; 50:50) as a colorless oil. The title compound was also prepared according to general procedure IV (12 mg, 36 µmol, 72%, α:β; 63:37). The title compound was also prepared according to general procedure V (10 mg, 30 µmol, 60%, α:β; >98:2). TLC: R_f 0.5 (pentane:EtOAc, 7:3, v:v); $[\alpha]_D^{20}$ 69.8° (c 0.5, $CHCl_3$; α-anomer); IR (neat, cm^{-1}): 1007, 1066, 1804, 2923; NMR data reported as a mixture of α- and β-anomers; ^1H NMR (400 MHz, $CDCl_3$): δ 7.38 – 7.28 (m, 10H, CH_{arom}), 4.86 (d, *J* = 11.6 Hz, 1H, C*H*H Bn), 4.79 (d, *J* = 3.3 Hz, 1H, H-1_α), 4.65 – 4.59 (m, 2H, C*H*H Bn, CH*H* Bn), 4.56 (d, *J* = 12.1 Hz, 1H, , CH*H* Bn), 4.47 – 4.30 (m, 3H, H-1_β, H-7_β, H-7_α), 4.02 – 3.90 (m, 2H, H-9_β, H-9_α), 3.81 (ddd, *J* = 11.8, 5.0, 3.4 Hz, 1H, H-2_α), 3.76 – 3.68 (m, 2H, H-5_β, H-5_α), 3.65 – 3.50 (m, 3H, H-2_β, H-9_β, H-9_α), 2.25 (dd, *J* = 14.2, 5.2 Hz, 1H, H-3_β), 2.16 – 2.04 (m, 1H, H-3_α), 1.97 (dd, *J* = 13.5, 5.0 Hz, 1H, H-3_α), 1.76 (dd, *J* = 14.3, 10.8 Hz, 1H, H-3_β), 1.48 – 1.42 (m, *J* = 6.9, 3.3 Hz, 6H, H-8_β, H-8_α), 1.36 (d, *J* = 6.3 Hz, 3H H-10_α/H-10_β), 1.27 – 1.24 (m, 9H, H-6_β, H-6_α, H-10_β/H-10_α); ^{13}C NMR (101 MHz, $CDCl_3$): δ 138.0, 137.9 ($C_{q\text{-}arom}$), 131.2, 129.5, 128.7, 128.6, 128.1, 128.0, 128.0, 127.9, 124.9 (CH_{arom}), 104.0 (C-1_β), 95.3 (C-1_α), 85.0 (C-4_α/C-4_β), 84.2(C-4_β/C-4_α), 81.5 (C-7_α/C-7_β), 80.6 (C-7_β/C-7_α), 73.4(C-2_β), 73.4 (CH_2 Bn), 71.9(C-2_α), 71.7 (CH_2 Bn), 65.1 (C-9_β/C-9_α), 64.9 (C-5_β/C-5_α), 63.8 (C-9_α/C-9_β), 38.3 (C-3_β), 33.8 (C-3_α), 15.4 (C-6_β/C-6_α), 15.4 (C-6_α/C-6_β), 15.2 (C-10_α/C-10_β), 14.8 (C-10_β/C-10_α), 13.2 (C-8_β/C-8_α), 13.2 (C-8_α/C-8_β); HRMS: $[M+Na]^+$ calcd for $C_{18}H_{24}O_6Na$ 359.1471, found 359.1465.

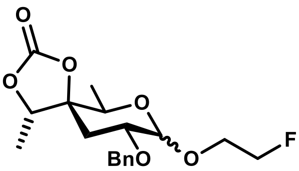

2-Fluoroethyl 2-*O*-benzyl-4,7-carbonate-D-yersinioside (S61). The title compound was prepared according to general procedure III (13 mg, 38 µmol, 76%, α:β; 66:34) as a colorless oil. The title compound was also prepared according to general procedure IV (14 mg, 42 µmol, 85%, α:β; 81:19). The title compound was also prepared according to general procedure V (11 mg, 33 µmol, 65%, α:β; >98:2); TLC: R_f 0.1 (pentane:EtOAc, 8:2, v:v); $[\alpha]_D^{20}$ 57.6° (c 0.5, $CHCl_3$; α-anomer); IR (neat, cm^{-1}): 1008, 1066, 1793, 1805; Data of the anomer: ^1H NMR (500 MHz, $CDCl_3$): δ 7.45 – 7.19 (m, 5H, CH_{arom}), 4.82 (d, *J* = 3.2 Hz, 1H, H-1), 4.72 – 4.50 (m, 4H, CH_2F, CH*H* Bn, C*H*H Bn), 4.34 (q, *J* = 6.9 Hz, 1H, H-7), 4.01 (q, *J* = 6.4 Hz, 1H, H-5), 3.96 – 3.68 (m, 3H, H-3, C*H*$_2$$CH_2F$), 2.14 (dd, *J* = 13.5, 11.9 Hz, 1H, H-3), 1.99 (dd, *J* = 13.5, 4.9 Hz, 1H, H-3), 1.43 (d, *J* = 6.9 Hz, 3H, H-8), 1.25 (d, *J* = 6.3 Hz, 3H, H-6); ^{13}C NMR (126 MHz, $CDCl_3$): δ 154.1 (O(C=O)O), 137.9 ($C_{q\text{-}arom}$), 128.7, 128.6, 128.2, 128.0, 128.0 (CH_{arom}), 95.8 (C-1), 84.8 (C-4), 82.7 (d, *J* = 169.8 Hz, CH_2F), 81.6 (C-7), 72.0 (C-2), 71.8 (C-5), 71.7 (CH_2 Bn), 67.1 (d, *J* = 19.7 Hz, C*H*$_2$$CH_2F$), 33.6 (C-3), 14.8 (C-6), 13.1 (C-8); Diagnostic signals of the β-isomer: ^1H NMR (500 MHz, $CDCl_3$): δ 4.86 (d, *J* = 11.5 Hz, 1H, C*H*H Bn), 4.46 – 4.39 (m, 2H, H-1, H-7), 3.65 (ddd, *J* = 11.1, 6.9, 5.3 Hz, 2H, H-2), 2.27 (dd, *J* = 14.3, 5.3 Hz, 1H, H-3), 1.78 (dd, *J* = 14.3, 10.7 Hz, 1H, H-3), 1.36 (d, *J* = 6.3 Hz, 3H, H-6); ^{13}C NMR (126 MHz): δ 153.8 (O(C=O)O), 138.2 ($C_{q\text{-}arom}$), 104.2 (C-1), 84.1 (C-4), 82.84 (d, *J* = 169.8 Hz, CH_2F), 80.6 (C-7), 73.5 (CH_2 Bn), 73.2 (C-2), 68.27 (d, *J* = 20.0 Hz, *C*H_2CH_2F), 65.0 (C-5), 38.1 (C-3), 15.3 (C-6), 13.2 (C-8); HRMS: $[M+Na]^+$ calcd for $C_{18}H_{23}O_6Na$ 377.1376, found 377.1368.

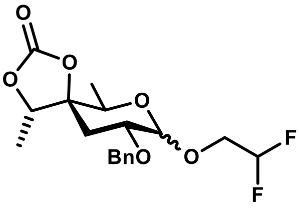

2,2-Di-fluoroethyl 2-*O*-benzyl-4,7-carbonate-D-yersinioside (S62). The title compound was prepared according to general procedure III (18 mg, 50 µmol, *quant.*, α:β; 80:20) as a colorless oil. The title compound was also prepared according to general procedure IV (18 mg, 50 µmol, *quant.*, α:β; 88:12). The title compound was also prepared according to general procedure V (3.0 mg, 8 µmol, 16%, α:β; >98:2); TLC: R_f 0.2 (pentane:EtOAc, 8:2, v:v); IR (neat, cm^{-1}): 696, 1009, 1063, 1091, 1793, 1808; Data of the major stereoisomer (α-anomer): ^1H NMR (500 MHz, $CDCl_3$): δ 8.10 – 7.16 (m, 15H, CH_{arom}), 5.94 (tt, *J* = 55.4, 4.2 Hz, 1H, CHF_2), 4.79 (d, *J* = 3.3 Hz, 1H, H-1), 4.63 (d, *J* = 12.1 Hz, 1H, CH*H* Bn), 4.55 (d, *J* = 12.0 Hz, 1H, C*H*H Bn), 4.35 (q, *J* = 6.9 Hz, 1H, H-7), 3.95 (q, *J* = 6.3 Hz, 1H, H-5), 3.87 – 3.70 (m, 3H, H-2, C*H*$_2$$CHF_2$), 2.09 (d, *J* = 11.8 Hz, 1H, H-3), 2.00 (dd, *J* = 13.6, 5.0 Hz, 1H, H-3), 1.43 (d, *J* = 6.9 Hz, 3H, H-8), 1.26 (d, *J* = 6.3 Hz, 3H, H-6); ^{13}C NMR (126 MHz, $CDCl_3$): δ 154.0 (O(C=O)O), 137.8 ($C_{q\text{-}arom}$), 131.2, 129.5, 128.7, 128.0, 124.9 (CH_{arom}), 114.1 (t, *J* = 241.5 Hz, CHF_2), 96.4 (C-1), 84.5 (C-4), 81.5 (C-7), 71.9 (CH_2 Bn), 71.6 (C-2), 67.2 (t, *J* = 28.5 Hz, *C*H_2CHF_2), 65.5 (C-5), 33.5 (C-3), 14.8 (C-6), 13.1 (C-8); Diagnostic signals of the minor stereoisomer (β-isomer): ^1H NMR (500 MHz, $CDCl_3$): δ 4.46 – 4.39 (m, 2H, H-1, H-7), 3.64 (ddd, *J* = 10.8, 7.1, 5.3 Hz, 1H), 2.26 (dd, *J* = 14.4, 5.3 Hz, 1H, H-3), 1.78 (dd, *J* = 14.3, 10.8 Hz, 1H, H-3), 1.36 (d, *J* = 6.2 Hz, 1H, H-6); ^{13}C NMR (126 MHz, $CDCl_3$): δ 145.8 (O(C=O)O), 138.0 ($C_{q\text{-}arom}$), 104.3 (C-1), 83.9 (C-4), 80.6 (C-7), 73.5 (CH_2 Bn), 73.1 (C-2), 72.2 (C-5), 38.0 (C-3), 15.3 (C-6), 13.2 (C-8); HRMS: $[M+Na]^+$ calcd for $C_{18}H_{22}O_6F_2Na$ 395.1282, found 395.1284.

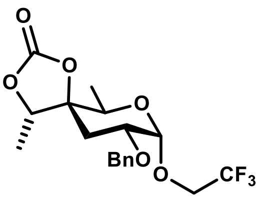

2,2,2-Tri-fluoroethyl 2-*O*-benzyl-4,7-carbonate-α-D-yersinioside (S63). The title compound was prepared according to general procedure III (15 mg, 38 µmol, 77%, α:β; >98:2) as a colorless oil. The title compound was also prepared according to general procedure IV (7.0 mg, 18 µmol, 36%, α:β; >98:2). TLC: R_f 0.8 (pentane:EtOAc, 7:3, v:v); $[\alpha]_D^{20}$ 25.7° (c 0.5, $CHCl_3$); IR (neat, cm^{-1}): 1009, 1063, 1275, 1793, 1809, 2925; ^1H NMR (400 MHz, $CDCl_3$): δ 7.38 – 7.28 (m, 5H, CH_{arom}), 4.83 (d, *J* = 3.3 Hz, 1H, H-1), 4.63 (d, *J* = 11.9 Hz, 1H, C*H*H Bn), 4.56 (d, *J* = 11.9 Hz, 1H, CH*H* Bn), 4.36 (q, *J* = 6.9 Hz, 1H, H-7), 3.98 – 3.80 (m, 4H, H-2, H-5, C$H_2$$CF_3$), 2.13 (dd, *J* = 13.6, 11.8 Hz, 1H, H-3), 2.02 (ddd, *J* = 13.6, 5.1, 0.8 Hz, 1H, H-3), 1.44 (d, *J* = 5.2 Hz, 3H, H-8), 1.27 (d, *J* = 6.4 Hz, 3H, H-6); ^{13}C NMR (101 MHz, $CDCl_3$): δ 153.9 (O(C=O)O), 137.8 ($C_{q\text{-}arom}$), 131.2, 129.5, 128.7, 128.3, 128.0, 125.7, 124.9 (CH_{arom}), 96.4 (C-1), 84.4 (C-4), 81.5 (C-7), 71.9 (CH_2 Bn), 71.4 (C-2), 65.8 (C-5), 65.3, 65.0 (C-9), 33.4 (C-3), 14.7 (C-6), 13.1 (C-8); HRMS: $[M+Na]^+$ calcd for $C_{18}H_{21}F_3O_6Na$ 413.1188, found 413.1182.

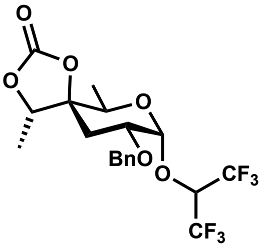

1,1,1,3,3,3-hexafluoropropyl 2-*O*-benzyl-4,7-carbonate-α-D-yersinioside (S64). The title compound was prepared according to general procedure III yieldingthe title compound (6.5 mg, 14 µmol, 28%, α:β; >98:2). Flash column chromatography (80:20 → 60:40; pentane:Et_2O) yielded the title compound as a colourless oil. TLC: R_f 0.8 (pentane:EtOAc, 8:2, v:v); $[\alpha]_D^{20}$ 42.5° (c 0.5, $CHCl_3$); IR (neat, cm^{-1}): 1008, 1064, 1105, 1197, 1796, 1813, 2923; ^1H NMR (400 MHz, $CDCl_3$, HH-COSY, HSQC): δ 7.39 – 7.29 (m, 5H, CH_{arom}), 5.15 (d, *J* = 3.4 Hz, 1H, H-1), 4.63 (d, *J* = 11.5 Hz, 1H, C*H*H Bn), 4.54 (d, *J* = 11.5 Hz, 1H, CH*H* Bn), 4.53 – 4.45 (m, 1H, H-9), 4.39 (q, *J* = 6.9 Hz, 1H, H-7), 4.04 (q, *J* = 6.3 Hz, 1H, H-5), 3.90 (ddd, *J* = 11.6, 5.2, 3.4 Hz, 1H, H-2), 2.14 (dd, *J* = 13.7, 11.6 Hz, 1H, H-3), 2.06 (dd, *J* = 13.7, 4.7 Hz, 1H, H-3), 1.43 (d, *J* = 2.9 Hz, 3H, H-8), 1.28 (d, *J* = 6.3 Hz, 3H, H-6); ^{13}C NMR (101 MHz, $CDCl_3$, HSQC): δ 137.3 ($C_{q\text{-}arom}$), 128.7, 128.3, 127.8 (CH_{arom}), 97.9 (C-1), 84.1 (C-4), 81.6 (C-7), 73.4, 73.1 (C-9), 71.7 (CH_2 Bn), 70.7 (C-2), 66.8 (C-5), 33.2 (C-3), 14.7 (C-6), 13.0 (C-8); HRMS: $[M+Na]^+$ calcd for $C_{19}H_{20}F_6O_6Na$ 481.1062, found 481.1056.

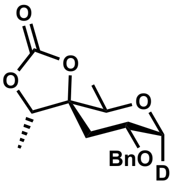

2-*O*-benzyl-4,7-carbonate-1-α-deuterio-D-yersinioside (S65). The title compound was prepared according to general procedure III yielding the title compound (7.9 mg, 27 µmol, 54%, α:β; >98:2). Flash column chromatography (80:20 → 60:40; pentane:Et_2O) yielded the title compound as a colorless oil. TLC: R_f 0.5 (pentane:EtOAc, 8:2, v:v); $[\alpha]_D^{20}$ 12.5° (c 0.5, $CHCl_3$); IR (neat, cm^{-1}): 1008, 1065, 1093, 1793, 2923; ^1H NMR (400 MHz, $CDCl_3$, HH-COSY, HSQC): δ 7.38 – 7.29 (m, 5H, CH_{arom}), 4.56 (s, 2H, CH_2 Bn), 4.49 (q, *J* = 6.8 Hz, 1H, H-7), 4.00 (dd, *J* = 4.5, 1.8 Hz, 1H, H-1), 3.81 (dt, *J* = 9.2, 4.5 Hz, 1H, H-2), 3.64 (q, *J* = 6.3 Hz, 1H, H-5), 2.31 (ddd, *J* = 13.8, 4.6, 1.9 Hz, 1H, H-3), 1.76 (dd, *J* = 13.7, 9.5 Hz, 1H, H-3), 1.47 (d, *J* = 6.8 Hz, 3H, H-8), 1.31 (d, *J* = 6.3 Hz, 3H, H-6); ^{13}C NMR (101 MHz, $CDCl_3$, HSQC): δ 154.0 (O(C=O)O), 137.9 ($C_{q\text{-}arom}$), 128.7, 128.2, 127.8 (CH_{arom}), 83.8 (C-4), 81.8 (C-7), 73.4 (C-5), 71.5

(CH_2 Bn), 70.5 (C-2), 67.8, 67.6, 67.4 (C-1), 38.3 (C-3), 14.9 (C-6), 13.4 (C-8); HRMS: $[M+Na]^+$ calcd for $C_{16}H_{19}DO_5Na$ 316.1271, found 316.1266.

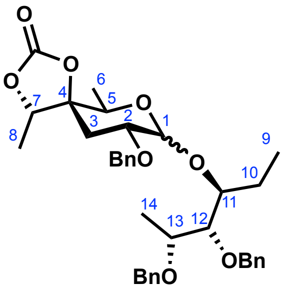

1,2,6-Trideoxy-4,5-di-*O*-benzyl-D-altritol-2-*O*-benzyl-4,7-carbonyl-D-yersinioside (S66). The title compound was prepared according to the general procedure III giving the product as a white solid (10.4 mg, 63%, α:β; 61:39). TLC: R_f 0.5 (pentane:EtOAc, 4:1, v:v) for α-isomer. TLC: R_f 0.2 (pentane:EtOAc, 4:1, v:v) for β-isomer; NMR data reported as a mixture of α- and β-anomers; ^{1}H NMR (850 MHz, $CDCl_3$, HH-COSY, HSQC): δ 7.41 – 7.17 (m, 15H, CH_{arom}), 4.95 (d, J = 3.3 Hz, 1H, H-1$_\alpha$), 4.76 (m, 2H, CH*H* Bn$_\alpha$, CH*H* Bn$_\beta$), 4.71 (d, J = 11.8 Hz, 1H, CH*H* Bn$_\beta$), 4.67 – 4.49 (m, 7H, CH*H* Bn$_\alpha$, CH*H* Bn$_\alpha$, CH*H* Bn$_\beta$, C*H*H Bn$_\alpha$, C*H*H Bn$_\alpha$, C*H*H Bn$_\alpha$, C*H*H Bn$_\beta$, C*H*H Bn$_\beta$), 4.43 (d, J = 7.5 Hz, 1H, H-1$_\beta$), 4.38 – 4.33 (m, 1H, C*H*H Bn$_\beta$, H-13$_\beta$), 4.22 (q, J = 6.9 Hz, 1H, H-7$_\alpha$/H-5$_\alpha$), 4.13 – 4.05 (m, 2H, H-5$_\alpha$/H-7$_\alpha$, H-5$_\beta$/H-7$_\beta$), 3.91 (dt, J = 8.3, 3.6 Hz, 1H, H-11$_\beta$), 3.87 (ddd, J = 6.9, 5.3, 3.8 Hz, 1H, H-11$_\alpha$), 3.82 – 3.77 (m, 2H, H-2$_\alpha$, H-12$_\alpha$), 3.69 – 3.67 (m, 1H, H-12$_\beta$), 3.62 (dd, J = 6.2, 3.6 Hz, 1H, H-13$_\alpha$), 3.61 – 3.58 (m, 1H, H-2$_\beta$, H-5$_\beta$/H-7$_\beta$), 3.55 (q, J = 6.2 Hz, 1H, H-5$_\beta$/H-7$_\beta$), 2.07 – 1.98 (m, 2H, H-3$_\alpha$), 1.92 (ddd, J = 13.4, 4.8, 1.0 Hz, 1H, H-3$_\alpha$), 1.77 – 1.66 (m, 2H, H-10$_\alpha$, H-10$_\beta$, H-10$_\beta$), 1.64 – 1.59 (m, 3H, H-10$_\alpha$, H-3$_\beta$, H-3$_\beta$), 1.39 – 1.37 (m, 2H, H-14$_\beta$), 1.36 – 1.32 (m, 4H, H-14$_\alpha$), 1.29 – 1.27 (m, 5H, H-6$_\beta$/H-8$_\beta$), 1.12 (d, J = 6.9 Hz, 3H, H-6$_\alpha$/H-8$_\alpha$), 1.00 (d, J = 6.3 Hz, 3H, H-6$_\alpha$/H-8$_\alpha$), 0.97 (t, J = 7.4 Hz, 2H, H-9$_\beta$), 0.92 (t, J = 7.5 Hz, 3H, H-9$_\alpha$), 0.88 (td, J = 7.2, 0.9 Hz, 2H, H-6$_\beta$/H-8$_\beta$); ^{13}C NMR (214 MHz, $CDCl_3$, HSQC): δ 154.2, 154.1, 152.3, 147.2, 139.0, 138.8, 138.7, 138.2, 138.0, 136.0 ($C_{q\text{-}arom}$), 131.3, 128.6, 128.5, 128.4, 128.3, 128.1, 128.0, 127.8, 127.7, 127.6, 127.5, 124.9, 114.2 (CH_{arom}), 102.4 (C-1$_\beta$), 93.3 (C-1$_\alpha$), 85.0, 84.5, 83.2, 81.7 (C-12), 81.6 (C-5$_\alpha$), 80.6, 80.6, 80.2, 80.1, 77.9 (C-11), 77.9, 75.4 (C-13), 75.4, 75.1, 74.2, 73.8 (C-2$_\beta$), 73.6, 73.0, 72.0 (C-13), 72.0, 71.9 (C-2$_\alpha$), 71.9, 71.6, 71.4, 71.4, 71.0, 70.8, 70.8, 70.8, 65.1 (C-5$_\beta$), 64.8, 38.8 (C-3$_\beta$), 36.7, 34.4, 33.9 (C-3$_\alpha$), 32.1, 31.6, 31.2, 30.5, 30.3, 29.8, 29.7, 29.5, 29.4, 29.3, 29.1, 28.8, 26.1, 23.4, 22.8, 22.6, 16.1, 16.0, 15.2, 14.6 (C-6$_\beta$), 14.3, 13.1, 12.9, 12.9 (C-6$_\alpha$), 10.5 (C-9), 9.9 (C-9); HRMS: $[M+Na]^+$ calcd for $C_{36}H_{44}O_8N_a$ 627.2934, found 627.2928.

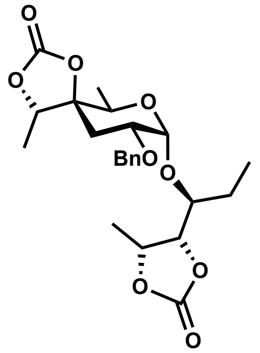

1,2,6-Trideoxy-4,5-*O*-carbonate-D-altritol-2-*O*-benzyl-4,7-carbonate-α-D-yersinioside (S67). The title compound was prepared according to general procedure III (17 mg, 37 μmol, 74%, α:β; >98:2). Flash column chromatography (80:20 → 60:40; pentane:EtOAc) yielded the title compound as a colorless oil. TLC: R_f 0.3 (pentane:EtOAc, 7:3, v:v); $[\alpha]_D^{20}$ 48.2° (c 1.0, $CHCl_3$); IR (neat, cm^{-1}): 1006, 1063, 1790, 2923; ^{1}H NMR (500 MHz, $CDCl_3$, HH-COSY, HSQC, HMBC): δ 7.37 – 7.27 (m, 5H, CH_{arom}), 4.98 (d, J = 3.4 Hz, 1H, H-1), 4.95 – 4.88 (m, 1H, H-13), 4.70 (dd, J = 7.5, 3.5 Hz, 1H, H-12), 4.58 (d, J = 11.8 Hz, 1H, C*H*H Bn), 4.53 (d, J = 11.8 Hz, 1H, CH*H* Bn), 4.37 (q, J = 6.8 Hz, 1H, H-7), 4.03 (q, J =

6.3 Hz, 1H, H-5), 3.95 (ddd, *J* = 7.5, 5.6, 3.4 Hz, 1H, H-9), 3.86 (ddd, *J* = 11.2, 5.4, 3.4 Hz, 1H, H-2), 2.07 – 2.01 (m, 2H, H-3, H-3), 1.81 (dqd, *J* = 15.2, 7.6, 4.9 Hz, 1H, H-10'), 1.62 – 1.58 (m, 4H, H-10, H-14), 1.50 (d, *J* = 6.9 Hz, 3H, H-8), 1.27 (d, *J* = 6.3 Hz, 3H, H-6), 1.01 (t, *J* = 7.5 Hz, 3H, H-11); ^{13}C NMR (126 MHz, $CDCl_3$, HSQC): δ 154.6, 154.1 (O(C=O)O), 137.9 ($C_{q\text{-}arom}$), 128.6, 128.0, 127.6 (CH_{arom}), 93.0 (C-1), 82.0 (C-7), 78.7 (C-12), 77.2 (C-9), 76.1 (C-13), 71.7 (C-2), 71.6 (CH_2 Bn), 65.9 (C-5), 33.5 (C-3), 22.2 (C-10), 15.3 (C-14), 14.9 (C-6), 13.1 (C-8), 10.3 (C-11); HRMS: $[M+Na]^+$ calcd for $C_{23}H_{30}O_9Na$ 473.1788, found 473.1782.

Results of compound 24

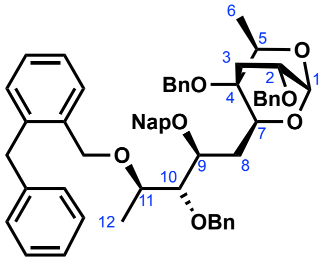

(1*R*,3*S*,4*R*,5*R*,7*S*)-3-((2*S*,3*R*,4*R*)-4-((2-Benzylbenzyl)oxy)-3-*O*-benzyl-2-*O*-2-methylnaphthalene)pentyl)-4,7-di-*O*-benzyl-5-methyl-2,6-dioxabicyclo[2.2.2]octane (26). The title compound was prepared according to general procedure III (16.7 mg, 19 µmol, 85%). Flash column chromatography (90:10 → 80:20; pentane:Et_2O) yielded the title compound as a colorless oil. TLC: R_f 0.3 (pentane:EtOAc, 9:1, v:v); $[\alpha]_D^{20}$ 5.6° (c 1.0, $CHCl_3$); IR (neat, cm^{-1}): 696, 734, 804, 1027, 1071, 1088, 1260, 1453; ^{1}H NMR (500 MHz, $CDCl_3$, HH-COSY, HSQC): δ 7.87 – 6.95 (m, 31H, CH_{arom}), 4.89 – 4.81 (m, 2H, CH*H* Ph, C*H*H Ph), 4.74 (d, *J* = 2.3 Hz, 1H, H-1), 4.65 (m, 2H, CH*H* Ph, C*H*H Ph), 4.56 (d, *J* = 10.3 Hz, 1H, CH*H* Ph), 4.52 (d, *J* = 11.7 Hz, 1H, H-7), 4.43 (d, *J* = 11.7 Hz, 1H, CH*H* Ph), 4.36 (d, *J* = 11.7 Hz, 1H, C*H*H Ph), 4.30 – 4.20 (m, 5H, H-5, H-9, CH*H* Ph, C*H*H Ph, C*H*H Ph), 3.97 (s, 2H, CH_2Bn), 3.75 – 3.66 (m, 2H, H-2, H-10), 3.42 (dq, *J* = 7.7, 6.0 Hz, 1H, H-11), 2.31 – 2.22 (m, 2H, H-3, H-8), 2.15 (dd, *J* = 14.4, 10.8 Hz, 1H, H-8), 1.98 (ddd, *J* = 13.8, 2.8, 1.5 Hz, 1H, H-3), 1.24 (d, *J* = 6.4 Hz, 3H, H-6), 1.20 (d, *J* = 6.1 Hz, 3H, H-12); ^{13}C NMR (126 MHz, $CDCl_3$, HSQC): δ 140.9, 139.3, 139.0, 138.0, 137.9, 136.8, 136.7 ($C_{q\text{-}arom}$), 133.4, 132.9, 130.2, 129.8, 129.1, 128.6, 128.5, 128.4, 128.1, 128.0, 127.8, 127.8, 127.8, 127.5, 126.5, 126.1, 126.1, 125.9, 125.8, 125.7 (CH_{arom}), 90.4 (C-1), 82.4 (C-10), 77.9 (C-9), 75.5 (C-11), 75.1 (C-7), 74.2 (CH_2 Bn), 73.6 (C-5), 72.7 (C-2), 72.3 (CH_2 Bn), 71.7 (C-4), 70.7, 69.3, 64.4, 38.1 (CH_2 Bn), 30.4 (C-8), 28.4 (C-3), 16.9 (C-12), 15.8 (C-6); HRMS: $[M+Na]^+$ calcd for $C_{58}H_{60}O_7Na$ 891.4237, found 891.4240.

Results of compound S36

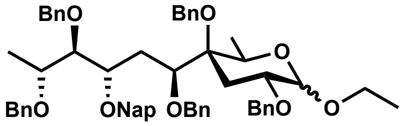

Ethyl 2,4,7,10,11-penta-*O*-benzyl-9-*O*-2-methylnaphthalene-D-caryophylloside (S68). The title compound was prepared according to general procedure III (20.6 mg, 22.5 µmol, *quant.*, α:β; 25:75). Flash column chromatography (95:5 → 90:10; pentane:Et_2O) yielded the title compound as a colorless oil. TLC: R_f 0.8 (pentane:EtOAc, 8:2, v:v); IR (neat, cm^{-1}): 696, 733, 1028, 1051, 1073, 1093, 1453; Data of the major stereoisomer (β-anomer): ^{1}H NMR (500 MHz, $CDCl_3$, HH-COSY, HSQC): δ 7.94 – 6.68 (m, 32H, CH_{arom}), 4.89 – 4.40 (m, 11H, CH*H* Ph, CH*H* Ph, CH*H* Ph, CH*H* Ph, C*H*H Ph, C*H*H Ph, CH*H* Ph, C*H*H Ph, CH*H* Ph, C*H*H Ph, C*H*H Ph), 4.30 (d, *J* = 7.7 Hz, 1H, H-1), 4.21 (d, *J* = 11.5 Hz, 1H, C*H*H Ph), 4.05 – 3.94 (m, 2H, H-9, CH_2CH_3), 3.86 – 3.71 (m, 2H, H-5, H-10), 3.66 (ddd, *J* = 11.5, 7.9, 6.3 Hz, 1H, H-2), 3.60 – 3.54 (m, 2H, H-7, CH_2CH_3), 3.47 (dd, *J* = 7.7, 6.1 Hz, 1H, H-11), 2.30 (dd, *J* = 14.6, 5.6 Hz, 1H, H-3), 2.24 (dd, *J* = 14.5, 10.7 Hz, 1H, H-8), 1.96 (dd, *J* = 14.6, 11.7 Hz, 1H, H-3), 1.63 (ddd, *J* = 14.8, 9.8, 1.6 Hz, 1H, H-8), 1.34 – 1.25 (m, 9H, CH_2CH_3, H-6, H-12); ^{13}C NMR (126 MHz, $CDCl_3$, HSQC): δ 139.6, 139.0, 138.8, 138.8, 138.4, 136.2, 133.4 ($C_{q\text{-}arom}$), 128.5, 128.5, 128.4, 128.3, 128.3, 128.2,

128.2, 128.2, 128.2, 128.1, 128.1, 128.1, 128.0, 127.8, 127.8, 127.8, 127.7, 127.7, 127.6, 127.6, 127.5, 127.5, 127.3, 127.1, 127.1, 127.0, 126.9, 126.8, 126.6, 126.4, 126.4, 126.3, 126.2, 126.1 (CH_{arom}), 105.5 (C-1), 81.9 (C-10), 80.0 (C-4), 78.4 (C-7), 76.6 (C-9), 75.6 (C-5), 74.8 (C-2), 74.8 (C-11), 74.1, 73.5, 73.1, 70.9, 70.4, 66.7 (CH_2 Bn), 65.1 (*C*H_2CH_3), 33.2 (C-3), 31.9 (C-8), 16.8 (CH_2*C*H_3), 15.5 (C-12), 15.2 (C-6); Diagnostic signals of the minor stereoisomer (α-isomer): ^{1}H NMR (500 MHz, $CDCl_3$, HH-COSY, HSQC): δ 4.82 (d, *J* = 4.2 Hz, 1H, H-1); ^{13}C NMR (126 MHz, $CDCl_3$, HSQC): δ 95.8 (H-1), 80.3 (C-4), 32.4 (C-3), 27.9 (C-8), 16.9 (CH_2*C*H_3), 15.3 (C-12), 15.2 (C-6); HRMS: $[M+Na]^+$ calcd for $C_{60}H_{66}O_8Na$ 937.4655, found 937.4667.

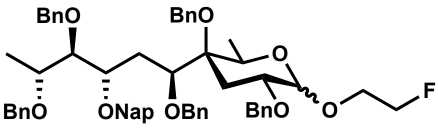

2-Fluoroethyl 2,4,7,10,11-penta-*O*-benzyl-9-*O*-2-methylnaphthalene-D-caryophylloside (S69). The title compound was prepared according to general procedure III (14.6 mg, 15.6 µmol, 70%, α:β; 33:67). Flash column chromatography (90:10 → 80:20; pentane:Et_2O) yielded the title compound as a colorless oil. TLC: R_f 0.6 (pentane:EtOAc, 9:1, v:v); IR (neat, cm^{-1}): 696, 734, 1028, 1071, 1094, 1453; Data of the major stereoisomer (β-anomer): ^{1}H NMR (500 MHz, $CDCl_3$, HH-COSY, HSQC): δ 8.02 – 6.63 (m, 32H, CH_{arom}), 4.90 – 4.40 (m, 13H, CH_2F, CH*H*Ph, CH*H*Ph, CH*H*Ph, CH*H*Ph, CH*H*Ph, CH*H*Ph, C*H*H Ph, C*H*H Ph, C*H*H Ph, C*H*H Ph, C*H*H Ph), 4.35 (d, *J* = 7.7 Hz, 1H, H-1), 4.21 (d, *J* = 11.5 Hz, 1H, C*H*H Ph), 4.08 – 3.96 (m, 3H, H-5, C*H*$_2$$CH_2F$), 3.88 – 3.71 (m, 4H, H-9, H-10), 3.67 (ddd, *J* = 13.1, 7.6, 5.7 Hz, 1H, H-2), 3.59 (d, *J* = 9.3 Hz, 1H, H-7), 3.51 – 3.44 (m, 1H, H-11), 2.32 (dd, *J* = 14.7, 5.7 Hz, 1H, H-3), 2.27 – 2.20 (m, 1H, H-8), 1.95 (dd, *J* = 14.6, 11.7 Hz, 1H, H-3), 1.63 (dd, *J* = 13.8, 10.2 Hz, 1H, H-8), 1.32 – 1.28 (m, 6H, H-6, H-12); ^{13}C NMR (126 MHz, $CDCl_3$, HSQC): δ 139.5, 138.9, 138.7, 138.4, 136.2, 136.1, 133.4, 133.1 ($C_{q\text{-}arom}$), 128.6, 128.5, 128.5, 128.4, 128.4, 128.3, 128.2, 128.2, 128.2, 128.1, 128.1, 127.9, 127.8, 127.8, 127.8, 127.7, 127.7, 127.6, 127.6, 127.5, 127.3, 127.1, 127.1, 127.1, 127.0, 126.9, 126.8, 126.7, 126.6, 126.4, 126.4, 126.3, 126.2, 126.1 (CH_{arom}), 105.8 (C-1), 83.0 (d, *J* = 169.4 Hz, CH_2F), 81.9 (C-9/C-10), 79.9 (C-4), 78.4 (C-7), 76.6 (C-5), 75.8 (C-9/C-10), 74.8 (C-2), 74.6, 74.1, 73.2, 70.9, 68.3, 66.7 (CH_2 Bn), 33.1 (C-3), 31.9 (C-8), 16.8 (C-12), 15.2 (C-6); Diagnostic signals of the minor stereoisomer (α-isomer): ^{1}H NMR (500 MHz, $CDCl_3$, HH-COSY, HSQC): δ 4.85 (d, *J* = 3.8 Hz, 1H, H-1); ^{13}C NMR (126 MHz, $CDCl_3$, HSQC): δ 96.5 (C-1), 82.8 (d, *J* = 169.5 Hz, CH_2F), 80.3 (C-4); HRMS: $[M+Na]^+$ calcd for $C_{60}H_{65}O_8FNa$ 955.4561, found 955.4578.

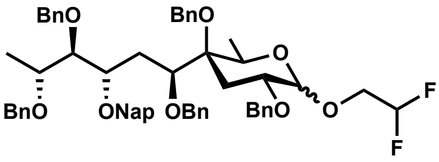

2,2-Difluoroethyl 2,4,7,10,11-penta-*O*-benzyl-9-*O*-2-methylnaphthalene-D-caryophylloside (S70). The title compound was prepared according to general procedure III (18.4 mg, 19.3 µmol, 86%, α:β; 63:37). Flash column chromatography (95:5 → 80:20; pentane:Et_2O) yielded the title compound as a colourless oil. TLC: R_f 0.5 (pentane:EtOAc, 9:1, v:v); IR (neat, cm^{-1}): 696, 732, 1028, 1070, 1453; Data of the major stereoisomer (α-anomer): ^{1}H NMR (500 MHz, $CDCl_3$, HH-COSY, HSQC): δ 8.28 – 6.49 (m, 32H, CH_{arom}), 5.86 (tt, *J* = 55.7, 4.4 Hz, 1H, CHF_2), 4.89 – 4.83 (m, 2H, C*H*H Ph, C*H*H Ph), 4.81 (d, *J* = 2.7 Hz, 1H, H-1), 4.71 – 4.41 (m, 8H, C*H*H Ph, C*H*H Ph, C*H*H Ph, C*H*H Ph), 4.24 – 4.16 (m, 2H, CH*H* Ph, CH*H* Ph), 4.08 – 3.93 (m, 4H, H-5, H-9), 3.81 – 3.67 (m, 6H, H-2, H-10, C*H*$_2$$CHF_2$), 3.54 (d, *J* = 9.3 Hz, 1H, H-7), 3.50 – 3.40 (m, 1H, H-11), 2.24 – 2.16 (m, 3H, H-8, H-3), 2.10 – 2.04 (m, 1H, H-3), 1.36 – 1.25 (m, 6H, H-6, H-12); ^{13}C NMR (126 MHz, $CDCl_3$, HSQC): δ 139.3, 138.8, 138.8, 138.5, 138.3, 136.1, 133.4, 133.1 ($C_{q\text{-}arom}$), 128.6, 128.5, 128.4, 128.4, 128.4, 128.3, 128.3, 128.2, 128.2, 128.1, 128.1, 128.0, 127.8, 127.8, 127.8, 127.8, 127.7, 127.6, 127.6, 127.5, 127.4, 127.2, 127.1, 127.1, 126.9, 126.8, 126.7, 126.6, 126.4, 126.4, 126.3, 126.2, 126.2 (CH_{arom}), 114.5 (t, *J* = 241.2 Hz, CHF_2), 97.0 (C-1), 82.0 (C-10), 80.2 (C-4), 79.0 (C-7), 76.7 (C-5/C-9), 75.0 (C-11), 74.1, 73.9 (CH_2 Bn), 71.9 (C-2), 71.4, 71.0, 70.7 (CH_2 Bn), 68.9 (C-5/C-9), 66.8 (d, *J* = 45.7 Hz, *C*H_2CHF_2), 65.3 (CH_2 Bn), 32.5 (C-8), 27.9 (C-3), 16.9

(C-12), 15.3 (C-6); Diagnostic signals of the minor stereoisomer (β-isomer): ^{1}H NMR (500 MHz, $CDCl_3$, HH-COSY, HSQC): δ 4.34 (d, J = 7.6 Hz, 1H, H-1), 3.64 (ddd, J = 13.3, 7.7, 5.8 Hz, 2H, H-2), 3.59 (d, J = 9.1 Hz, 1H, H-7), 2.32 (dd, J = 14.6, 5.7 Hz, 1H, H-3), 1.92 (dd, J = 14.6, 11.7 Hz, 1H, H-3), 1.65 (dd, J = 13.8, 9.7 Hz, 2H, H-8); ^{13}C NMR (126 MHz, $CDCl_3$, HSQC): δ 114.6 (t, J = 241.2 Hz, CHF_2), 105.9 (C-1), 79.8 (C-4), 66.99 (d, J = 57.6 Hz), 33.0 (C-8), 31.9 (C-3), 16.8 (C-12), 15.2 (C-6); HRMS: $[M+Na]^+$ calcd for $C_{60}H_{64}O_8F_2Na$ 973.4467, found 973.4478.

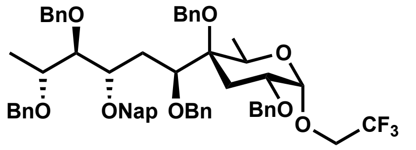

2,2,2-Trifluoroethyl 2,4,7,10,11-penta-*O*-benzyl-9-*O*-2-methylnaphthalene-D-caryophylloside (S71). The title compound was prepared according to general procedure III (14.3 mg, 15.3 µmol, 68%, α:β; >98:2). Flash column chromatography (95:5 → 90:10; pentane:EtOAc) yielded the title compound as a colorless oil. TLC: R_f 0.8 (pentane:EtOAc, 8:2, v:v); $[\alpha]_D^{20}$ 3.2° (c 1.0, $CHCl_3$); IR (neat, cm^{-1}): 696, 734, 1028, 1071, 1095, 1159, 1278, 1453; ^{1}H NMR (500 MHz, $CDCl_3$, HH-COSY, HSQC): δ 7.89 – 6.74 (m, 32H, CH_{arom}), 4.89 (d, J = 3.4 Hz, 1H, H-1), 4.88 – 4.81 (m, 2H, C*H*H Ph, C*H*H Ph), 4.68 – 4.41 (m, 9H, C*H*H Ph, C*H*H Ph, C*H*H Ph, C*H*H Ph, CH*H* Ph, CH*H* Ph, CH*H* Ph, CH*H* Ph, CH*H* Ph), 4.17 (d, J = 11.5 Hz, 1H, CH*H* Ph), 4.05– 3.92 (m, 2H, H-5, H-9), 3.88 (dd, J = 11.7, 8.8 Hz, 1H, CH_2CF_3), 3.77 (ddd, J = 12.2, 4.7, 3.6 Hz, 1H, H-2), 3.74 – 3.71 (m, 1H, H-10), 3.54 (d, J = 9.1 Hz, 1H, H-7), 3.43 (dt, J = 11.6, 5.8 Hz, 1H, H-11), 2.27 – 2.17 (m, 2H, H-3, H-8), 2.10 (dd, J = 13.8, 12.4 Hz, 1H, H-3), 1.65 (dd, J = 13.7, 9.6 Hz, 1H, H-8), 1.30 (d, J = 1.3 Hz, 3H, H-12), 1.29 (d, J = 1.9 Hz, 3H, H-6); ^{13}C NMR (126 MHz, $CDCl_3$) δ 139.2, 138.8, 138.7, 138.5, 138.3, 136.0, 133.3, 133.1 ($C_{q\text{-}arom}$), 130.2, 129.8, 129.1, 128.6, 128.6, 128.5, 128.4, 128.4, 128.4, 128.3, 128.2, 128.1, 128.1, 128.0, 128.0, 128.0, 127.9, 127.8, 127.8, 127.7, 127.6, 127.5, 127.4, 127.1, 127.0, 126.8, 126.7, 126.6, 126.4, 126.2, 126.1, 126.1, 125.9, 125.8, 125.7 (CH_{arom}), 96.9 (C-1), 82.0 (C-10), 80.1 (C-4), 78.9 (C-7), 76.7 (C-9), 75.0 (C-11), 74.1, 73.9 (CH_2 Bn), 71.7 (C-2), 71.2, 71.0, 70.7 (CH_2 Bn), 69.2 (C-5), 65.3 (CH_2 Bn), 64.7 (dd, J = 69.1, 38.4 Hz, CH_2CF_3), 32.5 (C-8), 27.8 (C-3), 16.9 (C-12), 15.3 (C-6); HRMS: $[M+Na]^+$ calcd for $C_{60}H_{63}O_8F_3Na$ 991.4373, found 991.4390.

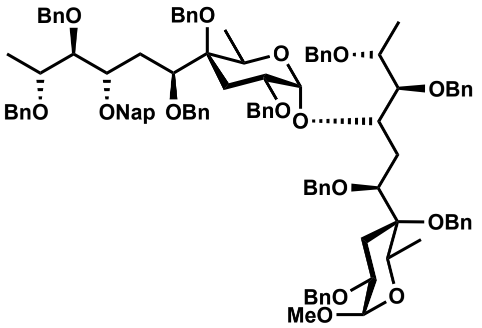

Methyl 2,4,7,10,11-penta-*O*-benzyl-9-*O*-[2,4,7,10,11-penta-*O*-benzyl-9-*O*-2-methylnaphthalene-α-D-caryophyllosyl]-α-D-caryophylloside (S72). The title compound was prepared according to general procedure VI with acceptor **S33** (1.2 eq. acceptor used instead of 2.0 eq.) yielding title compound (7.3 mg, 4.5 µmol, 20%, α:β; >98:2). Flash column chromatography (95:5 → 80:20; pentane:EtOAc) yielded the title compound as a colorless oil. TLC: R_f 0.7 (pentane:EtOAc, 9:1, v:v); $[\alpha]_D^{20}$ 2.1° (c 0.4, $CHCl_3$); IR (neat, cm^{-1}): 696, 734, 1028, 1072, 1096, 1453; ^{1}H NMR (500 MHz, $CDCl_3$, HH-COSY, HSQC): δ 7.84 – 6.74 (m, 57H, CH_{arom}), 5.02 (d, J = 3.5 Hz, 1H, H-1'), 4.82 – 4.72 (m, 2H, CH*H* Ph, CH*H* Ph), 4.67 (d, J = 3.4 Hz, 1H, H-1), 4.63 – 4.32 (m, 20H, CH*H* Ph, CH*H* Ph, CH*H* Ph, CH*H* Ph, CH*H* Ph, CH*H* Ph, CH*H* Ph, CH*H* Ph, C*H*H Ph, C*H*H Ph, C*H*H Ph, C*H*H Ph, C*H*H Ph, C*H*H Ph, C *H*H Ph, C*H*H Ph, C*H*H Ph, C*H*H Ph, C*H*H Ph, CH*H* Ph), 4.28 – 4.19 (m, 3H, H-5, H-9, H-9'), 4.10 (m, *2*H, H-10, H-10'), 3.99 (m, *2*H, H-5', H-7'), 3.80 (dd, J = 9.8, 5.9 Hz, 1H, H-2'), 3.76 – 3.66 (m, 1H, H-2), 3.60 (d, J = 9.6 Hz, 1H, H-7), 3.45 (p, J = 6.3 Hz, 1H, H-11), 3.35 (td, J = 6.7, 6.3, 3.3 Hz, 1H, H-11'), 3.31 (s, 3H, CH_3 OMe), 2.40 – 2.32 (m, 3H, H-3, H-3', H-8'), 2.26 (dd, J = 14.6, 11.1 Hz, 1H, H-8), 2.09 – 1.98 (m, 2H, H-3, H-

3'), 1.83 (dd, J = 14.1, 9.6 Hz, 1H, H-8'), 1.65 (dd, J = 14.0, 9.7 Hz, 1H, H-8), 1.30 (d, J = 6.5 Hz, 3H, H-6), 1.30 (d, J = 6.4 Hz, 3H, H-6'), 1.21 (d, J = 6.1 Hz, 3H, H-12'), 1.15 (d, J = 6.2 Hz, 3H, H-12); ^{13}C NMR (126 MHz, $CDCl_3$) δ 139.4, 139.3, 139.1, 139.0, 138.9, 138.9, 138.6, 138.5, 138.4, 136.0, 135.3, 133.0 ($C_{q\text{-}arom}$), 128.7, 128.5, 128.4, 128.4, 128.4, 128.4, 128.4, 128.3, 128.3, 128.3, 128.2, 128.2, 128.2, 128.1, 128.1, 128.1, 128.0, 128.0, 127.9, 127.8, 127.8, 127.7, 127.7, 127.6, 127.6, 127.5, 127.4, 127.3, 127.3, 127.2, 127.2, 127.1, 127.1, 127.0, 127.0, 127.0, 126.9, 126.9, 126.8, 126.5, 126.4, 126.3, 126.3, 126.3, 126.2, 126.1, 125.4 (CH_{arom}), 98.3 (C-1), 97.5 (C-1'), 85.3 (C-2'), 81.7 (C-2), 80.6, 80.4 (C-4, C-4'), 80.2 (C-5'), 79.7 (C-5), 79.4 (C-7), 76.7 (C-7'), 75.2, 74.9 (C-11, C-11'), 74.9, 74.2, 74.0, 73.8, 72.6 (CH_2 Bn), 72.5, 72.0 (C-10, C-10'), 71.2, 70.9, 70.8, 70.7, 70.6 (CH_2 Bn), 69.8, 68.8 (C-9, C-9'), 65.7, 65.2 (CH_2 Bn), 55.0 (CH_3 OMe), 33.4 (C-8'), 32.7 (C-8), 27.6, 22.5 (C-3, C-3'), 16.8 (C-12'), 16.7 (C-12), 15.5, 15.3 (C-6, C-6'); HRMS: $[M+Na]^+$ calcd for $C_{106}H_{116}O_{15}Na$ 1651.8212, found 1651.8168.

Results of compound 25

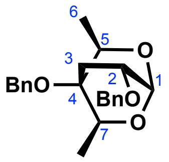

(1*R*,3*R*,4*R*,5*S*,7*S*)-4,7-Di-*O*-benzyl-3,5-dimethyl-2,6-dioxabicyclo[2.2.2]octane (S73). The title compound was prepared according to general procedure III (on a 30 µmol scale) yielding title compound (8.8 mg, 25 µmol, 83%). Flash column chromatography (95:5 → 80:20; pentane:Et_2O) yielded the title compound as a colorless oil. TLC: R_f 0.2 (pentane:Et_2O, 8:2, v:v); $[\alpha]_D^{20}$ –2.4° (c 1.0, $CHCl_3$); IR (neat, cm^{-1}): 696, 1027, 1066, 1091, 1127; ^{1}H NMR (500 MHz, $CDCl_3$, HH-COSY, HSQC): δ 7.40 – 7.19 (m, 10H, CH_{arom}), 4.86 (d, J = 2.1 Hz, 1H, H-1), 4.58 (s, 2H, CH_2Bn), 4.44 (d, J = 10.6 Hz, 1H, C*H*H Ph), 4.41 (d, J = 10.6 Hz, 1H, CH*H* Ph), 4.32 (qd, J = 6.4, 2.0 Hz, 1H, H-7), 4.25 (qd, J = 6.4, 1.6 Hz, 1H, H-5), 3.74 (ddd, J = 10.0, 3.3, 2.1 Hz, 1H, H-2), 2.29 (ddd, J = 13.7, 10.0, 2.1 Hz, 1H, H-3), 2.02 (ddd, J = 13.7, 3.3, 1.7 Hz, 1H, H-3), 1.46 (d, J = 6.4 Hz, 3H, H-8), 1.26 (d, J = 6.4 Hz, 3H, H-6); ^{13}C NMR (126 MHz, $CDCl_3$, HSQC): δ 138.1, 137.8 ($C_{q\text{-}arom}$), 128.7, 128.6, 128.0, 127.9, 127.8, 127.5 (CH_{arom}), 90.7 (C-1), 75.1 (C-7), 74.3 (C-5), 72.6 (C-2), 72.3 (C-4), 70.7 (4-OCH_2Bn), 64.9 (2-OCH_2Bn), 27.2 (C-3), 16.1 (C-6), 15.4 (C-8); HRMS: $[M+Na]^+$ calcd for $C_{22}H_{26}O_4Na$ 377.1729, found 377.1729.

Results of compound 3

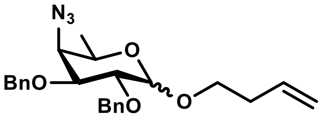

3-Butene 4-azido-2,3-di-*O*-benzyl-4,6-dideoxy-D-galactopyranoside (S74). The title compound was prepared according to general procedure III (18 mg, 42 µmol, 85%, α:β; 39:61) as a colorless oil. The title compound was also prepared according to general procedure IV (13 mg, 31 µmol, 61%, α:β; 62:38). The title compound was also prepared according to general procedure V (19 mg, 45 µmol, 95%, α:β; >98:2). TLC: R_f 0.6 (pentane:Et_2O, 9:1, v:v); $[\alpha]_D^{20}$ 24.3° (c 1.0, $CHCl_3$, α-anomer); IR (neat, cm^{-1}): 697, 1045, 1105, 1709, 2109, 2916; Data of the α-anomer: ^{1}H NMR (400 MHz, $CDCl_3$): δ 7.42 – 7.27 (m, 10H, CH_{arom}), 5.81 (ddt, J = 17.0, 10.2, 6.7 Hz, 1H, H-9), 5.10 (dq, J = 17.2, 1.6 Hz, 1H, H-10), 5.08 – 5.00 (m, 1H, H-10), 4.85 (d, J = 11.7 Hz, 1H, C*H*H Bn), 4.81 (d, J = 12.0 Hz, 1H, C*H*H Bn), 4.74 (d, J = 11.7 Hz, 1H, CH*H* Bn), 4.70 (d, J = 3.8 Hz, 1H, H-1), 4.64 (d, J = 12.0 Hz, 1H, CH*H* Bn), 4.03 (dd, J = 9.9, 3.7 Hz, 1H, H-3), 3.96 (qd, J = 6.5, 1.6 Hz, 1H, H-5), 3.83 (dd, J = 9.9, 3.8 Hz, 1H, H-2), 3.72 (dd, J = 3.8, 1.5 Hz, 1H, H-4), 3.56 (ddt, J = 44.4, 9.9, 7.0 Hz, 2H, H-7), 2.37 (qt, J = 7.0, 1.4 Hz, 2H, H-8, H-8), 1.21 (d, J = 6.5 Hz, 3H, H-6); ^{13}C NMR (101 MHz, $CDCl_3$): δ 138.6, 138.4 ($C_{q\text{-}arom}$), 135.1 (C-9), 128.6, 128.5, 128.1, 127.9, 127.9, 127.8 (CH_{arom}), 116.8 (C-10), 97.5 (C-1), 78.2 (C-3), 76.2 (C-2), 73.6, 73.3 (CH_2 Bn), 67.7 (C-7), 65.2 (C-4), 64.5 (C-5), 34.0 (C-8), 17.4 (C-6); Diagnostic signals of the β-anomer: ^{1}H NMR (500 MHz, $CDCl_3$): δ 4.30 (d, J = 7.6 Hz, 1H, H-1); ^{13}C NMR (101 MHz, $CDCl_3$): δ 103.7 (C-1), 73.6, 73.3 (CH_2 Bn), 67.7 (C-7); HRMS: $[M+Na]^+$ calcd for $C_{24}H_{29}O_4N_3Na$ 446.2056, found 446.2050.

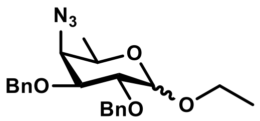

Ethyl 4-azido-2,3-di-*O*-benzyl-4,6-dideoxy-D-galactopyranoside (S75). The title compound was prepared according to general procedure III (18 mg, 44 µmol, 87%, α:β; 36:64) as a colorless oil. The title compound was also prepared according to general procedure IV (19 mg, 47 µmol, 93%, α:β; 62:38). The title compound was also prepared according to general procedure V (15 mg, 38 µmol, 75%, α:β; >98:2). TLC: R_f 0.7 (pentane:EtOAc, 9:1, v:v); IR (neat, cm^{-1}): 696, 1028, 1045, 1061, 2102; Data of the β-anomer: ^1H NMR (500 MHz, $CDCl_3$): δ 7.48 – 7.23 (m, 10H, CH_{arom}), 4.91 (d, *J* = 10.8 Hz, 1H, C*H*H Ph), 4.76 (d, *J* = 10.2 Hz, 1H, C*H*H Ph), 4.74 (d, *J* = 10.9 Hz, 1H, CH*H* Ph), 4.64 (d, *J* = 12.1 Hz, 1H, CH*H* Ph), 4.29 (d, *J* = 7.3 Hz, 1H, H-1), 3.96 (dq, *J* = 9.4, 7.1 Hz, 1H, CH_2CH_3), 3.66 – 3.62 (m, 3H, H-2, H-3, H-4), 3.56 (dd, *J* = 9.5, 7.1 Hz, 1H, CH_2CH_3), 3.53 – 3.49 (m, 1H, H-5), 1.30 (d, *J* = 6.3 Hz, 3H, H-6), 1.26 (t, *J* = 7.1 Hz, 3H, CH_2CH_3); ^{13}C NMR (126 MHz, $CDCl_3$, HSQC): δ 138.7, 138.1 ($C_{q\text{-}arom}$), 128.6, 128.5, 128.4, 128.2, 128.1, 127.9, 127.9, 127.7 (CH_{arom}), 103.5 (C-1), 81.1 (C-2/C-3), 79.3 (C-2/C-3), 75.4, 73.1 (CH_2 Bn), 68.9 (C-5), 65.5 (CH_2CH_3), 64.0 (C-4), 17.7 (C-6), 15.4 (CH_2CH_3); Diagnostic signals of the α-anomer: ^1H NMR (500 MHz, $CDCl_3$): δ 4.85 (d, *J* = 11.7 Hz, 1H, C*H*H Ph), 4.82 (d, *J* = 12.1 Hz, 1H, C*H*H Ph), 4.74 (d, *J* = 10.9 Hz, 1H, CH*H* Ph), 4.69 (d, *J* = 3.8 Hz, 1H, H-1), 4.04 (dd, *J* = 9.9, 3.7 Hz, 1H, H-3), 3.82 (dd, *J* = 9.9, 3.8 Hz, 1H, H-2), 3.72 (dd, *J* = 3.8, 1.5 Hz, 1H, H-4), 1.22 (t, *J* = 7.1 Hz, 3H, CH_2CH_3), 1.21 (d, *J* = 6.5 Hz, 2H, H-6); ^{13}C NMR (126 MHz, $CDCl_3$, HSQC): δ 138.5, 138.4 ($C_{q\text{-}arom}$), 97.1 (C-1), 78.3 (C-3), 76.2 (C-2), 73.6, 73.3 (CH_2 Bn), 65.2 (C-4), 64.3 (C-5), 63.6 (CH_2CH_3), 17.4 (C-6), 15.1 (CH_2CH_3); HRMS: $[M+Na]^+$ calcd for $C_{22}H_{27}O_4N_3Na$ 420.1899, found 420.1892.

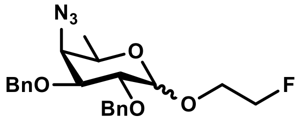

2-Mono-fluoroethyl 4-azido-2,3-di-*O*-benzyl-4,6-dideoxy-D-galactopyranoside (S76). The title compound was prepared according to general procedure III (21 mg, 50 µmol, *quant.*, α:β; 48:52) as a colorless oil. The title compound was also prepared according to general procedure IV (21 mg, 50 µmol, *quant.*, α:β; 81:19). The title compound was also prepared according to general procedure V (17 mg, 38 µmol, 79%, α:β; >98:2). TLC: R_f 0.3 (pentane:EtOAc, 9:1, v:v); IR (neat, cm^{-1}): 1041, 1089, 2102; Data of the β-anomer: ^1H NMR (500 MHz, $CDCl_3$): δ 7.75 – 7.12 (m, 10H, CH_{arom}), 4.93 (d, *J* = 10.6 Hz, 1H, C*H*H Ph), 4.85 (d, *J* = 11.7 Hz, 1H, C*H*H Ph), 4.77 (m, 1H, CH*H* Ph), 4.72 (d, *J* = 10.8 Hz, 1H, CH*H* Ph), 4.70 – 4.48 (m, 2H, CH_2F), 4.36 (d, *J* = 7.0 Hz, 1H, H-1), 4.13 – 3.98 (m, 1H, CH_2CH_2F), 3.88 – 3.62 (m, 4H, H-2, H-3, H-4, CH_2CH_2F), 3.53 (qd, *J* = 6.3, 1.1 Hz, 1H, H-5), 1.31 (d, *J* = 6.3 Hz, 3H, H-6); ^{13}C NMR (126 MHz) δ 138.5, 138.0 ($C_{q\text{-}arom}$), 131.2, 129.4, 128.6, 128.5, 128.4, 128.1, 127.8, 127.7 (CH_{arom}), 103.8 (C-1), 83.38 (d, *J* = 5.1 Hz, CH_2F), 81.0 (C-2/C-3), 79.0 (C-2/C-3), 73.3, 73.2 (CH_2 Bn), 69.0 (C-5), 67.21 (d, *J* = 20.0 Hz, CH_2CHF_2), 63.8 (C-4), 17.6 (C-6); Diagnostic signals of the α-anomer: ^1H NMR (500 MHz, $CDCl_3$): δ 4.72 (d, *J* = 5.8 Hz, 1H, H-1), 1.21 (d, *J* = 6.5 Hz, 3H, H-6); ^{13}C NMR (126 MHz, $CDCl_3$) δ 138.4, 138.3 ($C_{q\text{-}arom}$), 97.8 (C-1), 82.0 (d, *J* = 5.2 Hz, CH_2F), 78.1 (C-3), 76.1 (C-2), 75.4, 73.7 (CH_2 Bn), 68.7 (d, *J* = 20.2 Hz, CH_2CHF_2), 65.0 (C-4), 64.6 (C-5), 17.4 (C-6); HRMS: $[M+Na]^+$ calcd for $C_{22}H_{26}O_4N_3FNa$ 438.1805, found 438.1800.

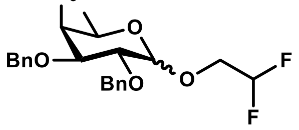

2,2-Di-fluoroethyl 4-azido-2,3-di-*O*-benzyl-4,6-dideoxy-D-galactopyranoside (S77). The title compound was prepared according to general procedure III (20 mg, 46 µmol, 91%, α:β; 77:23) as a colorless oil. The title compound was also prepared according to general procedure IV (19 mg, 43 µmol, 85%, α:β; >98:2). The title compound was also prepared according to general procedure V (17 mg, 41

μmol, 81%, α:β; >98:2). TLC: R_f 0.7 (pentane:EtOAc, 9:1, v:v); IR (neat, cm^{-1}): 696, 1046, 1067, 1091, 2104; Data of the α-anomer: ^1H NMR (500 MHz, $CDCl_3$): δ 7.80 – 7.22 (m, 10H, CH_{arom}), 5.92 (tt, J = 55.6, 4.4 Hz, 1H, CHF_2), 4.85 (m, 1H, CH*H* Bn), 4.82 (m, 1H, CH*H* Bn), 4.74 (d, J = 11.7 Hz, 1H, C*H*H Bn), 4.69 (d, J = 3.8 Hz, 1H, H-1), 4.62 (d, J = 11.9 Hz, 1H, CH*H* Bn), 4.02 (dd, J = 9.9, 3.6 Hz, 1H, H-3), 3.95 (qd, J = 6.6, 1.5 Hz, 1H, H-5), 3.86 (dd, J = 9.9, 3.8 Hz, 1H, H-2), 3.74 – 3.64 (m, 3H, CH_2CHF_2, H-4), 1.22 (d, J = 6.5 Hz, 3H, H-6); ^{13}C NMR (126 MHz, $CDCl_3$): δ 138.3, 138.2 ($C_{q\text{-}arom}$), 128.6, 128.5, 128.4, 128.1, 128.0, 127.9, 127.9, 127.7 (CH_{arom}), 114.1 (t, J = 241.3 Hz, CHF_2), 98.5 (C-1), 77.9 (C-3), 75.9 (C-2), 73.9, 73.3 (CH_2 Bn), 67.4 (t, J = 28.7 Hz, CH_2CHF_2), 65.0 (C-5), 64.8 (C-4), 17.3 (C-6); Diagnostic signals of the β-anomer: ^1H NMR (500 MHz, $CDCl_3$): δ 5.85 (tt, J = 55.1, 4.1 Hz, 1H, CHF_2), 4.34 (d, J = 7.2 Hz, 1H, H-1), 4.14 (tdd, J = 13.7, 11.9, 3.9 Hz, 1H, CH_2CHF_2), 3.53 (qd, J = 6.3, 1.1 Hz, 1H, H-5), 1.31 (d, J = 6.3 Hz, 3H, H-6); ^{13}C NMR (126 MHz) δ 138.3, 137.8 ($C_{q\text{-}arom}$), 114.3 (dd, J = 242.3, 239.7 Hz, CHF_2), 103.9 (C-1), 80.9 (C-3), 78.8 (C-2), 75.5, 73.2 (CH_2 Bn), 68.5 (dd, J = 30.8, 26.4 Hz, CH_2CHF_2), 68.3 (C-5), 63.7 (C-4), 17.5 (C-6); HRMS: $[M+Na]^+$ calcd for $C_{22}H_{25}O_4N_3F_2Na$ 456.1711, found 456.1704.

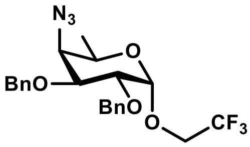

2,2,2-Tri-fluoroethyl 4-azido-2,3-di-*O*-benzyl-4,6-dideoxy-α-D-galactopyranoside (S78). The title compound was prepared according to general procedure III (16 mg, 35 μmol, 70%, α:β; >98:2) as a colorless oil. The title compound was also prepared according to general procedure IV (17 mg, 37 μmol, 73%, α:β; >98:2). TLC: R_f 0.8 (pentane:EtOAc, 9:1, v:v); IR (neat, cm^{-1}): 1103, 1156, 1277, 2109; ^1H NMR (400 MHz, $CDCl_3$): δ 7.59 – 7.28 (m, 10H, CH_{arom}), 4.84 (m, 2H, C*H*H Bn, CH*H* Bn), 4.76 (d, J = 5.5 Hz, 1H, H-1), 4.74 (m, 1H, CH*H* Bn), 4.63 (d, J = 11.9 Hz, 1H, C*H*H Bn), 4.04 (dd, J = 9.9, 3.6 Hz, 1H, H-3), 3.95 (qd, J = 6.5, 1.3 Hz, 1H, H-5), 3.92 – 3.82 (m, 3H, H-2, CH_2CF_3), 3.75 (dd, J = 3.6, 1.4 Hz, 1H, H-4), 1.23 (d, J = 6.5 Hz, 3H, H-6); ^{13}C NMR (101 MHz): δ 138.3, 138.2 ($C_{q\text{-}arom}$), 129.6, 129.4, 129.1, 128.9, 128.7, 128.6, 128.6, 128.1, 128.0, 127.9 (CH_{arom}), 122.5 (CF_3), 98.5 (C-1), 77.8 (C-3), 75.8 (C-2), 73.8, 73.4 (CH_2 Bn), 65.3 (C-5), 65.2 (q, J = 34.9 Hz, CH_2CF_3), 64.8 (C-4), 17.3 (C-6); ^{19}F NMR (471 MHz, $CDCl_3$): δ –73.7 (t, J = 8.3 Hz); HRMS: $[M+Na]^+$ calcd for $C_{22}H_{24}O_4N_3F_3Na$ 474.1617, found 474.1614.

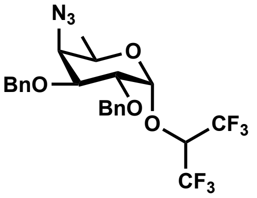

1,1,1,3,3,3-Hexafluoropropyl 2,3-di-*O*-benzyl-4-azido-4,6-dideoxy-α-D-galactopyranoside (S79). The title compound was prepared according to general procedure III (18 mg, 35 μmol, 69%, α:β; >98:2) as a colorless oil. Flash column chromatography (95:5 → 80:20; pentane:Et_2O) yielded the title compound. TLC: R_f 0.8 (pentane:EtOAc, 9:1, v:v); $[\alpha]_D^{20}$ 47.0° (c 1.0, $CHCl_3$); IR (neat, cm^{-1}): 1105, 1196, 1287, 2110; ^1H NMR (500 MHz, $CDCl_3$, HH-COSY, HSQC): δ 7.90 – 7.28 (m, 10H), 5.06 (d, J = 3.9 Hz, 1H, H-1), 4.84 (d, J = 11.5 Hz, 1H, CH*H* Bn), 4.76 (m, 1H, C*H*H Bn), 4.74 (m, 1H, CH*H* Bn), 4.69 (d, J = 11.5 Hz, 1H, C*H*H Bn), 4.41 (hept, J = 5.9 Hz, 1H, $CH(CF_3)_2$), 4.10 – 4.00 (m, 2H, H-3, H-4), 3.94 (dd, J = 10.0, 3.9 Hz, 1H, H-2), 3.79 (dd, J = 3.4, 1.3 Hz, 1H, H-5), 1.24 (d, J = 6.4 Hz, 3H, H-6); ^{13}C NMR (126 MHz, $CDCl_3$, HSQC): δ 138.0, 137.9 ($C_{q\text{-}arom}$), 136.7, 135.5, 133.7, 131.7, 130.4, 129.6, 129.1, 128.9, 128.6, 127.7 (CH_{arom}), 100.1 (C-1), 77.6 (C-3/C-4), 75.1 (C-2), 73.8, 73.4 (CH_2 Bn), 73.1 (p, J = 33.0 Hz, $CH(CF_3)$)), 66.4 (C-3/C-4), 64.5 (C-5), 17.2 (C-6); HRMS: $[M+Na]^+$ calcd for $C_{23}H_{23}O_4N_3F_6Na$ 542.1490, found 542.1489.

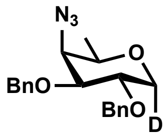

2,3-Di-*O*-benzyl-4-azido-1,4,6-trideoxy-1-α-deuterio-D-galactopyranoside (S80). The title compound was prepared according to general procedure III (15 mg, 41 µmol, 82%, α:β; >98:2) as a colorless oil. Flash column chromatography (90:10 → 80:20; pentane:EtOAc) yielded the title compound. TLC: R_f 0.5 (pentane:EtOAc, 9:1, v:v); $[\alpha]_D^{20}$ 11.4° (c 1.0, $CHCl_3$); IR (neat, cm^{-1}): 1093, 1124, 2108; ^1H NMR (500 MHz, $CDCl_3$, HH-COSY, HSQC): δ 7.44 – 7.26 (m, 10H, CH_{arom}), 4.87 – 4.76 (m, 3H, CH_2 Bn), 4.64 (d, *J* = 11.5 Hz, 1H, CH_2 Bn), 3.97 (d, *J* = 5.6 Hz, 1H, H-1), 3.89 (dd, *J* = 9.0, 5.6 Hz, 1H, H-2), 3.72 (dd, *J* = 3.7, 1.3 Hz, 1H, H-4), 3.67 (dd, *J* = 9.1, 3.7 Hz, 1H, H-3), 3.47 (qd, *J* = 6.3, 1.3 Hz, 1H, H-5), 1.27 (d, *J* = 6.3 Hz, 3H, H-6); ^2H NMR (77 MHz, $CDCl_3$) δ 3.12 (s, 1H); ^{13}C NMR (126 MHz, $CDCl_3$, HSQC): δ 138.4, 138.1 ($C_{q\text{-}arom}$), 129.5, 128.6, 128.6, 128.0, 127.9, 127.9, 127.9, 124.9 (CH_{arom}), 82.8 (C-3), 74.6 (C-2), 73.9 (CH_2 Bn), 73.8 (C-5), 72.7 (CH_2 Bn), 68.34 (t, *J* = 21.5 Hz, C-1), 64.3 (C-4), 18.0 (C-6); HRMS: $[M+Na]^+$ calcd for $C_{19}H_{20}O_3N_3DNa$ 377.1700, found 377.1697.

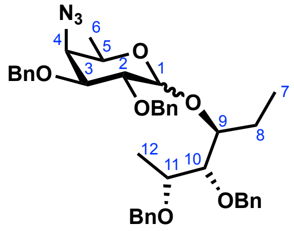

1,2,6-Trideoxy-4,5-di-*O*-benzyl-D-altritol-2,3-di-*O*-benzyl-4-azido-4,6-dideoxy-D-galactopyranoside (S81). The title compound was prepared according to the general procedure III giving title glycoside as a white solid (25.2 mg, 76%, α:β; 58:42). TLC: R_f 0.5 (pentane:Et_2O, 4:1, v:v) for α-isomer; TLC: R_f 0.2 (pentane:Et_2O, 4:1, v:v); NMR data reported as a mixture of α- and β-anomers; ^1H NMR (500 MHz, $CDCl_3$, HH-COSY, HSQC): δ 7.39 – 7.20 (m, 40H, CH_{arom}), 4.87 (d, *J* = 4.0 Hz, 1H, H-1_α), 4.83 – 4.65 (m, 2H, CH_2 Bn), 4.59 (d, *J* = 11.6 Hz, 1H, CH*H* Bn), 4.51 – 4.47 (m, 2H, CH_2 Bn), 4.37 (d, *J* = 7.1 Hz, H-1_β), 4.32 (d, *J* = 11.6 Hz, 1H, CH*H* Bn), 3.94 (m, 1H, H-9_β), 3.91 (dd, *J* = 3.6, 9.9 Hz, 1H, H-3_α), 3.85 – 3.81 (m, 2H, H-9_α, H-2_α), 3.77 (m, 1H, H-5_β), 3.72 (m, 1H, H-9), 3.70 – 3.65 (m, 2H, H-11, H-5_β), 3.65 – 3.59 (m, 2H, H-2_β, H-4_β), 3.58 (dd, *J* = 3.4, 1.5 Hz, 1H, H-4_α), 3.46 (m, 1H, H-5_α), 1.74 – 1.56 (m, 4H, H-8_β, H-8_β, H-8_α, H-8_α),1.29 – 1.65 (m, 6H, H-6_α, H-12), 1.04 (d, *J* = 6.4 Hz, 3H, H-6_β), 0.98 (t, *J* = 7.4 Hz, 3H, H-7), 0.91 (t, *J* = 7.6 Hz, 3H, H-7); ^{13}C NMR (126 MHz, $CDCl_3$, HSQC): δ 139.0, 138.9, 138.8, 138.8, 138.7, 138.5, 138.3, 137.8 ($C_{q\text{-}arom}$), 128.6, 128.5, 128.4, 128.4, 128.4, 128.3, 128.0, 128.0, 127.9, 127.9, 127.8, 127.7, 127.7, 127.6, 127.6, 127.6, 127.5, 127.4 (CH_{arom}), 102.0 (C-1_β), 96.1 (C-1_α), 83.0, 81.9, 81.6, 80.7, 79.1, 78.5 (C-9), 77.8 (C-3_α), 76.2 (C-2_α), 75.4, 75.2, 75.1, 74.0, 73.7, 73.4, 72.9, 70.7, 70.6, 68.9, 65.1 (C-4_α), 64.9 (C-5_β), 63.7, 29.8, 23.5, 22.3 (C-8), 17.6 (C-6_β), 17.3, 15.8, 15.5, 10.3 (C-7), 9.6 (C-7); HRMS: $[M+Na]^+$ calcd for $C_{40}H_{47}N_3O_6Na$ 688.3363, found 688.3357.

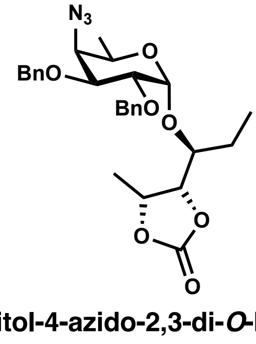

1,2,6-Trideoxy-4,5-*O*-carbonate-D-altritol-4-azido-2,3-di-*O*-benzyl-4,6-dideoxy-α-D-galactopyranoside (S82). The title compound was prepared according to general procedure III (17 mg, 33 µmol, 66%, α:β; >98:2). Flash column chromatography (90:10 → 70:30; pentane:EtOAc) yielded the title compound as a colorless oil. TLC: R_f 0.6 (pentane:EtOAc, 7:3, v:v); $[\alpha]_D^{20}$ 45.2° (c 1.0, $CHCl_3$); IR

(neat, cm^{-1}): 698, 737, 1012, 1053, 1093, 1800, 2106, 2928; ^{1}H NMR (500 MHz, $CDCl_3$, HH-COSY, HSQC, HMBC): δ 7.43 – 7.30 (m, 10H, CH_{arom}), 4.89 (d, J = 3.9 Hz, 1H, H-1), 4.88 – 4.82 (m, 2H, C*H*H Ph, H-9), 4.81 – 4.74 (m, 2H, CH_2 Bn), 4.67 – 4.61 (m, 2H, CH*H* Bn, H-8), 4.05 – 3.95 (m, 2H, H-3, H-5), 3.87 (dd, J = 10.0, 3.8 Hz, 1H, H-2), 3.83 (q, J = 5.6 Hz, 1H, H-7), 3.78 (dd, J = 3.6, 1.6 Hz, 1H, H-4), 1.75 (dqd, J = 14.9, 7.5, 5.8 Hz, 1H, H-11), 1.65 (dqd, J = 14.8, 7.4, 5.9 Hz, 1H, H-11'), 1.50 (d, J = 6.7 Hz, 3H, H-10), 1.24 (d, J = 6.5 Hz, 3H, H-6), 1.00 (t, J = 7.5 Hz, 3H, H-12); ^{13}C NMR (126 MHz, $CDCl_3$, HSQC): δ 154.5 (O(C=O)O), 138.4, 138.0 ($C_{q\text{-}arom}$), 131.2, 129.5, 128.7, 128.6, 128.5, 128.0, 127.9, 127.9, 124.9 (CH_{arom}), 96.3 (C-1), 78.8 (C-8), 78.0 (C-3), 77.2 (C-7), 76.1 (C-9), 75.8 (C-2), 74.1, 73.0 (CH_2 Bn), 65.5 (C-5), 64.7 (C-4), 22.7 (C-11), 17.5 (C-6), 15.1 (C-10), 9.6 (C-12); HRMS: $[M+Na]^+$ calcd for $C_{27}H_{33}O_7Na$ 534.2216, found 534.2211.

Structural proofs

Compound 21

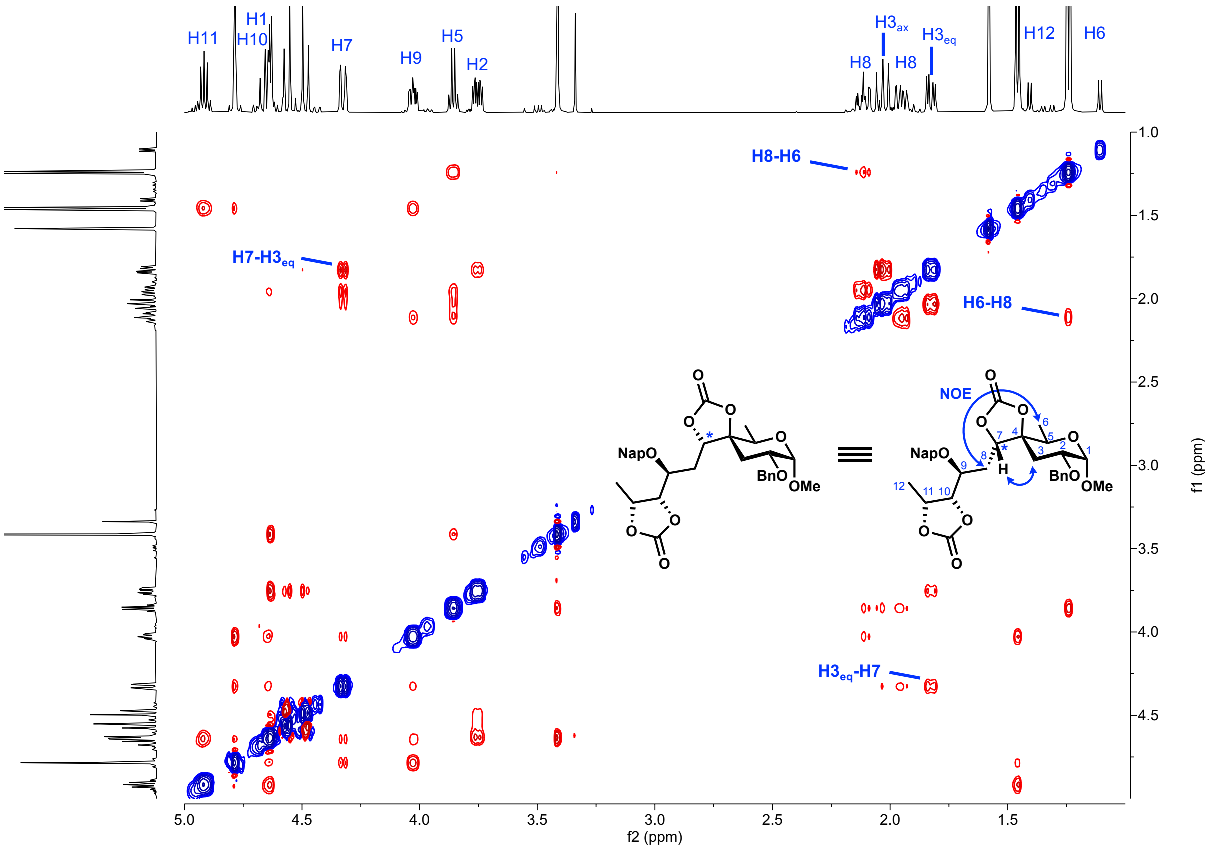

Figure S12. NOESY spectrum of compound **21**. The key NOE interactions for **21** can be found between $H3_{eq}$-H7 and H6-H8.

Compound S29 and S30

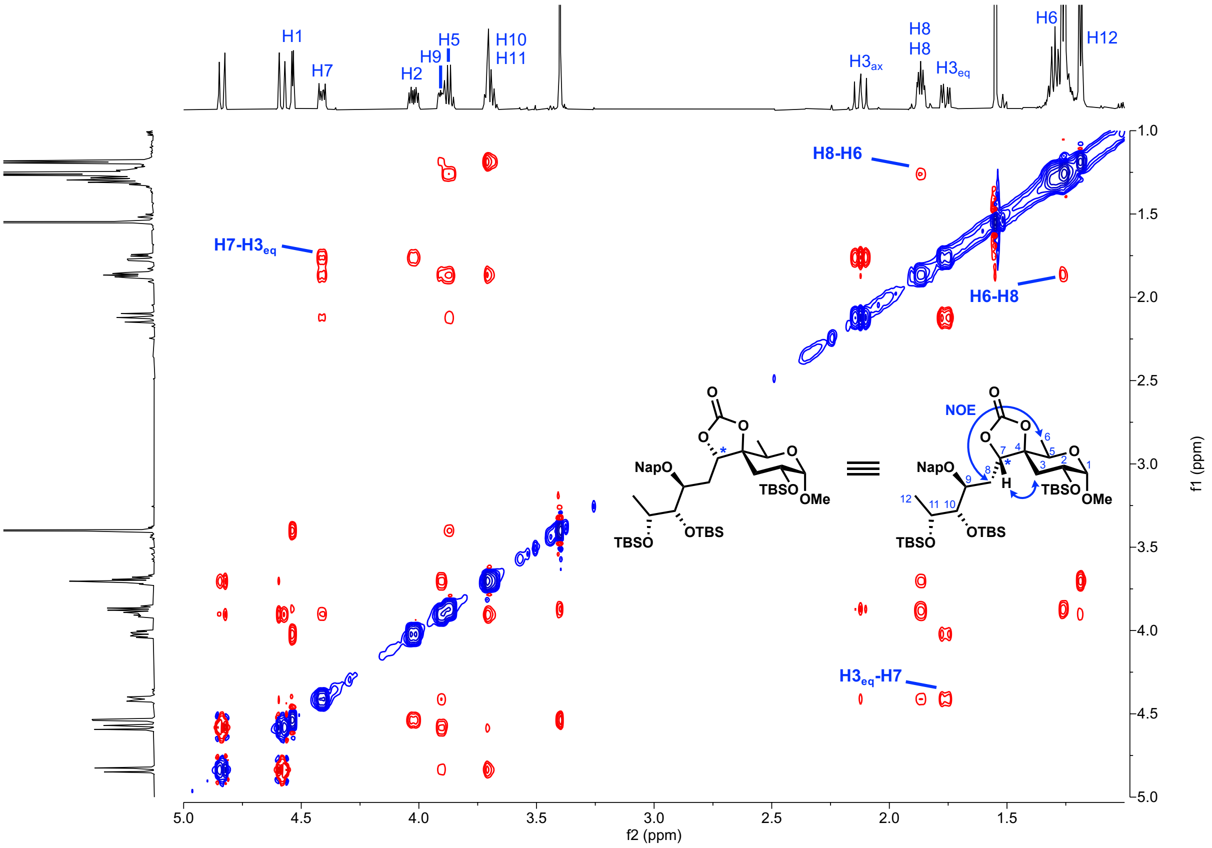

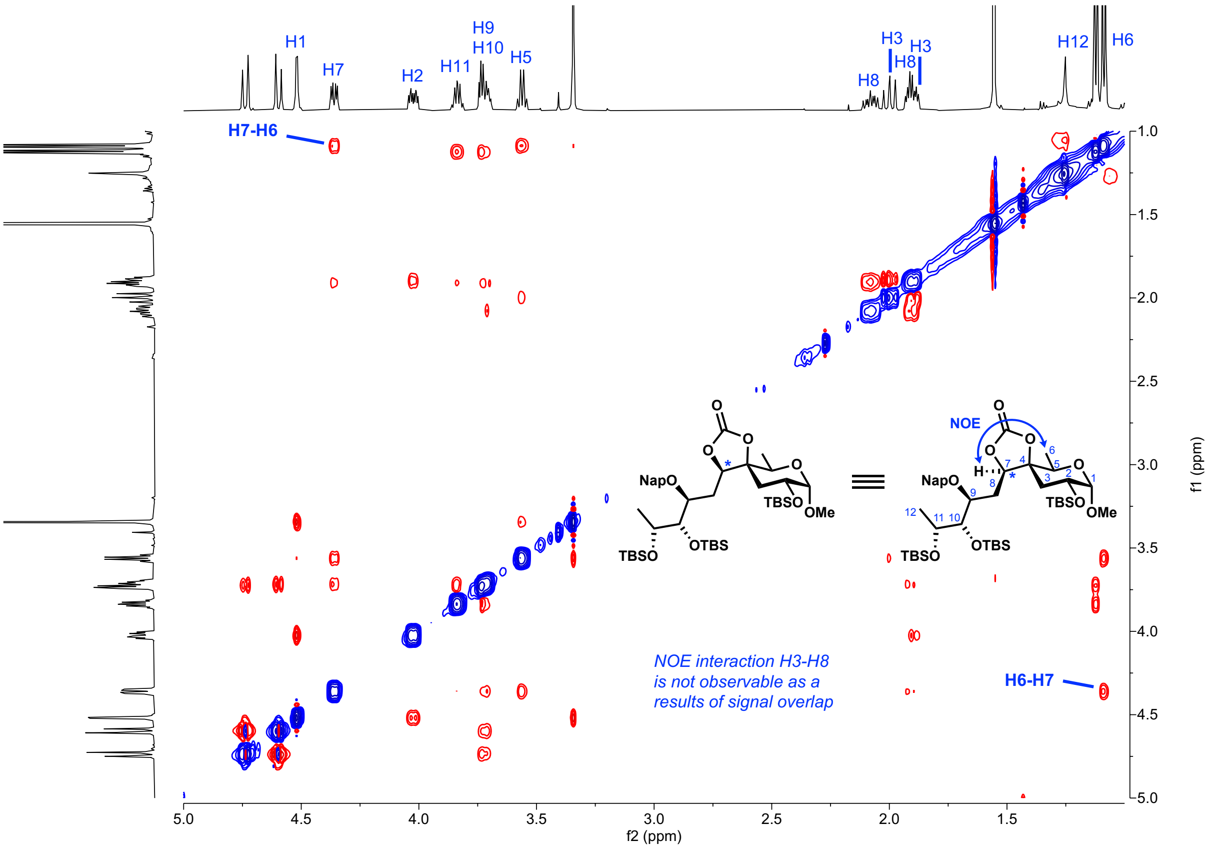

Figure S13. NOESY spectra of compound **S29** and **S30**. (A) The key NOE interactions for **S29** can be found between $H3_{eq}$-H7 and H6-H8. (B) The key NOE interaction for **S30** can be found between H6-H7.

Compound S22

^{1}H NMR H-H coupling constants

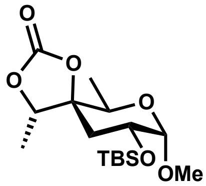

**H-1:** d, J = 3.4 Hz (eq^{H-1}-ax^{H-2})	
**H-2:** ddd, J = 3.5 Hz (ax^{H-2}-eq^{H-1}), 5.0 Hz (ax^{H-2}-eq^{H-3}), 11.6 Hz (ax^{H-2}-ax^{H-3})	
**H-3$_{ax}$:** dd, J = 11.6 Hz (ax^{H-3}-ax^{H-2}), 13.5 Hz (ax^{H-3}-eq^{H-3})	
**H-3$_{eq}$:** dd, J = 4.9 Hz (eq^{H-3}-ax^{H-2}), 13.5 Hz (eq^{H-3}-ax^{H-3})	
**H-5:** q, J = 6.4 Hz (ax^{H-5}-H-6)	

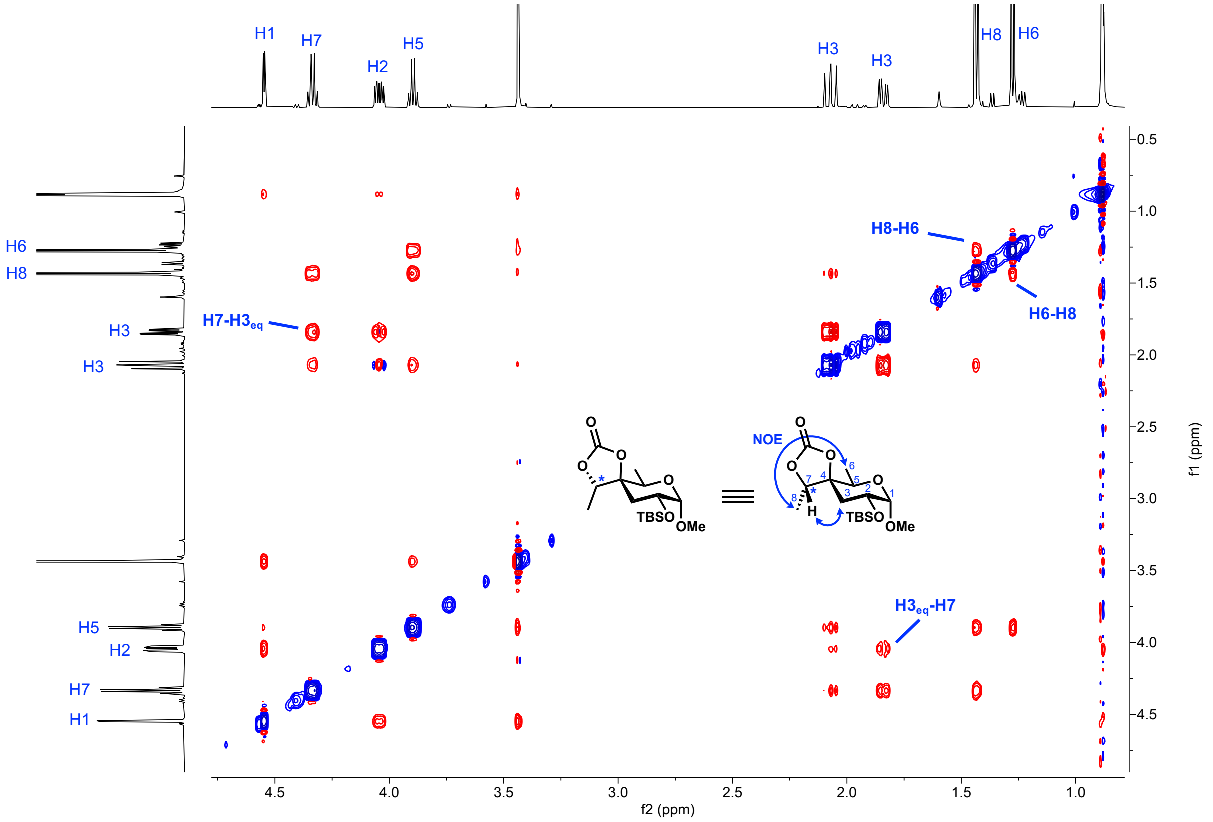

Figure S14. NOESY spectra of compound **S22**. The key NOE interactions for **S22** can be found between H3$_{eq}$-H7 and H6-H8.

Compound 24

¹H NMR H-H coupling constants

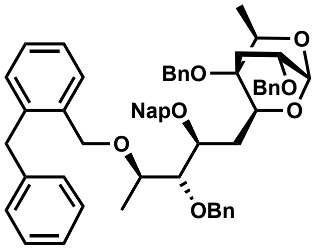

**H-1:**	d, $J = 2.3$ Hz (eq^{H-1}-ax^{H-2})
**H-2:**	overlaps with H-10
**H-3$_{ax}$:**	overlaps with H-8
**H-3$_{eq}$:**	dd, $J = 2.8$ Hz (eq^{H-3}-ax^{H-2}), 13.8 Hz (eq^{H-3}-ax^{H-3})
**H-5:**	overlaps with H-9

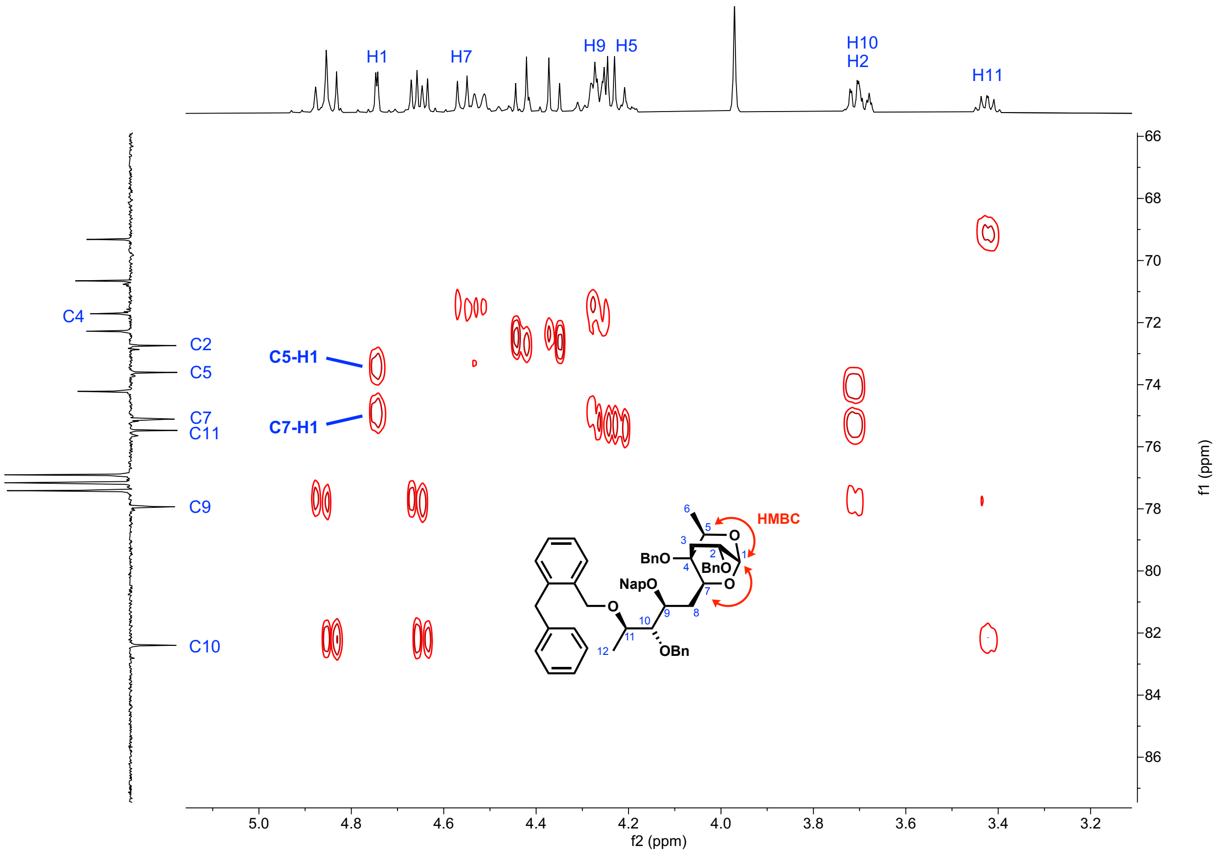

Figure S15. HMBC spectrum of compound **24**. The key long-range heteronuclear correlation for **24** can be found between C5-H1 and C7-H1.

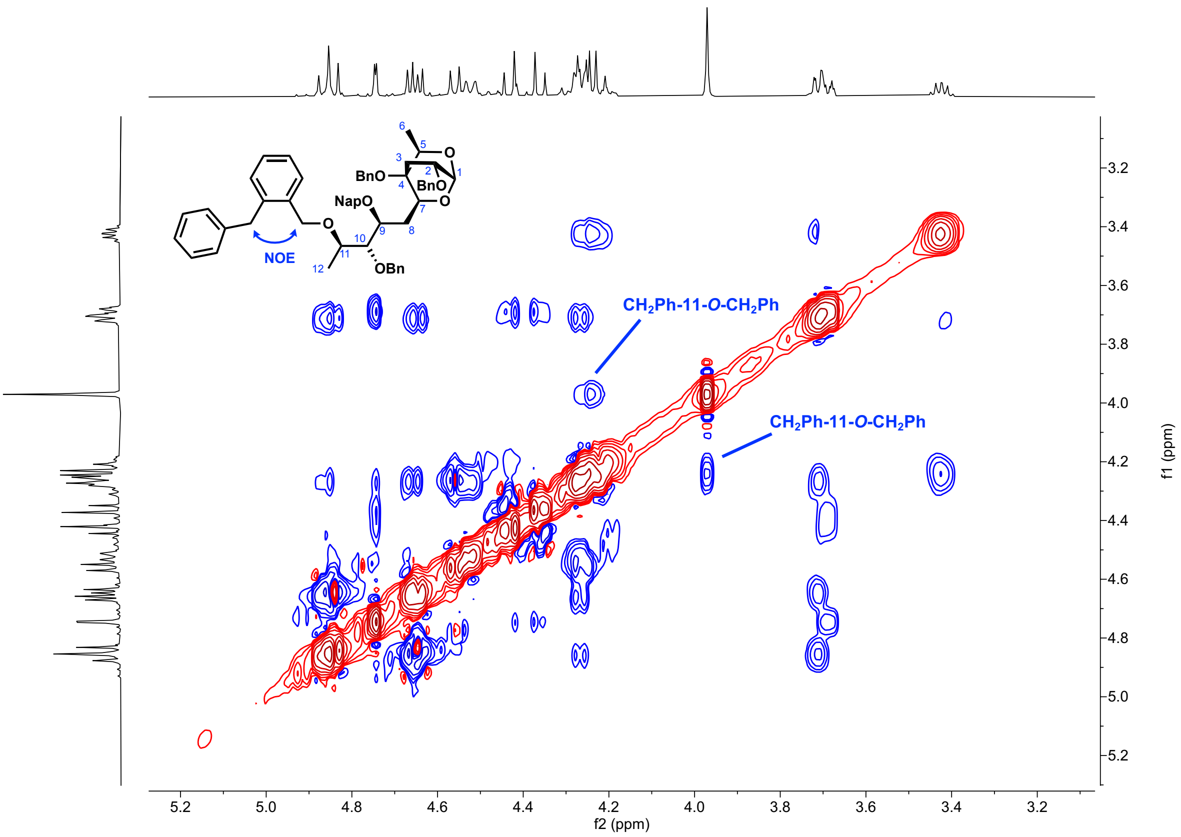

Figure S16. NOESY spectra of compound **24**. The key NOE interactions for **24** can be found between CH_2Bn and 11-*O*-CH_2Bn.

Compound S68-α

¹H NMR H-H coupling constants

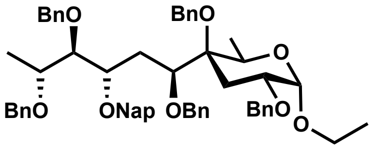

H-1: d, J = 2.1 Hz (eq$^{H\text{-}1}$-ax$^{H\text{-}2}$)

H-2: ddd, J = 2.1 Hz (ax$^{H\text{-}2}$-eq$^{H\text{-}1}$), 3.3 Hz (ax$^{H\text{-}2}$-eq$^{H\text{-}3}$), 10.0 Hz (ax$^{H\text{-}2}$-ax$^{H\text{-}3}$)

H-3$_{ax}$: dd, J = 10.0 Hz (ax$^{H\text{-}3}$-ax$^{H\text{-}2}$), 13.7 Hz (ax$^{H\text{-}3}$-eq$^{H\text{-}3}$)

H-3$_{eq}$: dd, J = 3.3 Hz (eq$^{H\text{-}3}$-ax$^{H\text{-}2}$), 13.7 Hz (eq$^{H\text{-}3}$-ax$^{H\text{-}3}$)

H-5: qd, J = 6.4 Hz (ax$^{H\text{-}5}$-H-6), 1.6 Hz (ax$^{H\text{-}5}$-eq$^{H\text{-}3}$)

Compound S68-β

¹H NMR H-H coupling constants

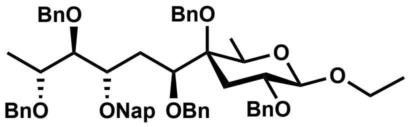

H-1: d, J = 7.7 Hz (ax$^{H\text{-}1}$-ax$^{H\text{-}2}$)

H-2: ddd, J = 7.9 Hz (ax$^{H\text{-}2}$-ax$^{H\text{-}1}$), 6.3 Hz (ax$^{H\text{-}2}$-eq$^{H\text{-}3}$), 11.5 Hz (ax$^{H\text{-}2}$-ax$^{H\text{-}3}$)

H-3$_{ax}$: dd, J = 11.7 Hz (ax$^{H\text{-}3}$-ax$^{H\text{-}2}$), 14.6 Hz (ax$^{H\text{-}3}$-eq$^{H\text{-}3}$)

H-3$_{eq}$: dd, J = 5.6 Hz (eq$^{H\text{-}3}$-ax$^{H\text{-}2}$), 14.6 Hz (eq$^{H\text{-}3}$-ax$^{H\text{-}3}$)

H-5: overlaps with other signals

Compound S71-α

¹H NMR H-H coupling constants

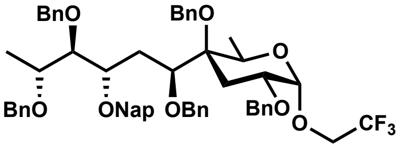

H-1: d, J = 3.4 Hz (eq$^{H\text{-}1}$-ax$^{H\text{-}2}$)

H-2: ddd, J = 3.6 Hz (ax$^{H\text{-}2}$-eq$^{H\text{-}1}$), 4.7 Hz (ax$^{H\text{-}2}$-eq$^{H\text{-}3}$), 12.2 Hz (ax$^{H\text{-}2}$-ax$^{H\text{-}3}$)

H-3$_{ax}$: overlaps with H-8

H-3$_{eq}$: dd, J = 3.3 Hz (eq$^{H\text{-}3}$-ax$^{H\text{-}2}$), 13.7 Hz (eq$^{H\text{-}3}$-ax$^{H\text{-}3}$)

H-5: overlaps with H-9

Compound S73

¹H NMR H-H coupling constants

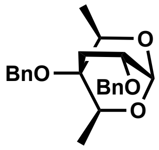

H-1: d, J = 2.1 Hz (eq^{H-1}-ax^{H-2})

H-2: ddd, J = 2.1 Hz (ax^{H-2}-eq^{H-1}), 3.3 Hz (ax^{H-2}-eq^{H-3}), 10.0 Hz (ax^{H-2}-ax^{H-3})

H-3$_{ax}$: ddd, J = 10.0 Hz (ax^{H-3}-ax^{H-2}), 13.7 Hz (ax^{H-3}-eq^{H-3}), 2.1 Hz (ax^{H-3}-ax^{H-7})

H-3$_{eq}$: ddd, J = 3.3 Hz (eq^{H-3}-ax^{H-2}), 13.7 Hz (eq^{H-3}-ax^{H-3}), 1.7 Hz (eq^{H-3}-ax^{H-5})

H-5: qd, J = 6.4 Hz (ax^{H-5}-H-6), 1.6 Hz (ax^{H-5}-eq^{H-3})

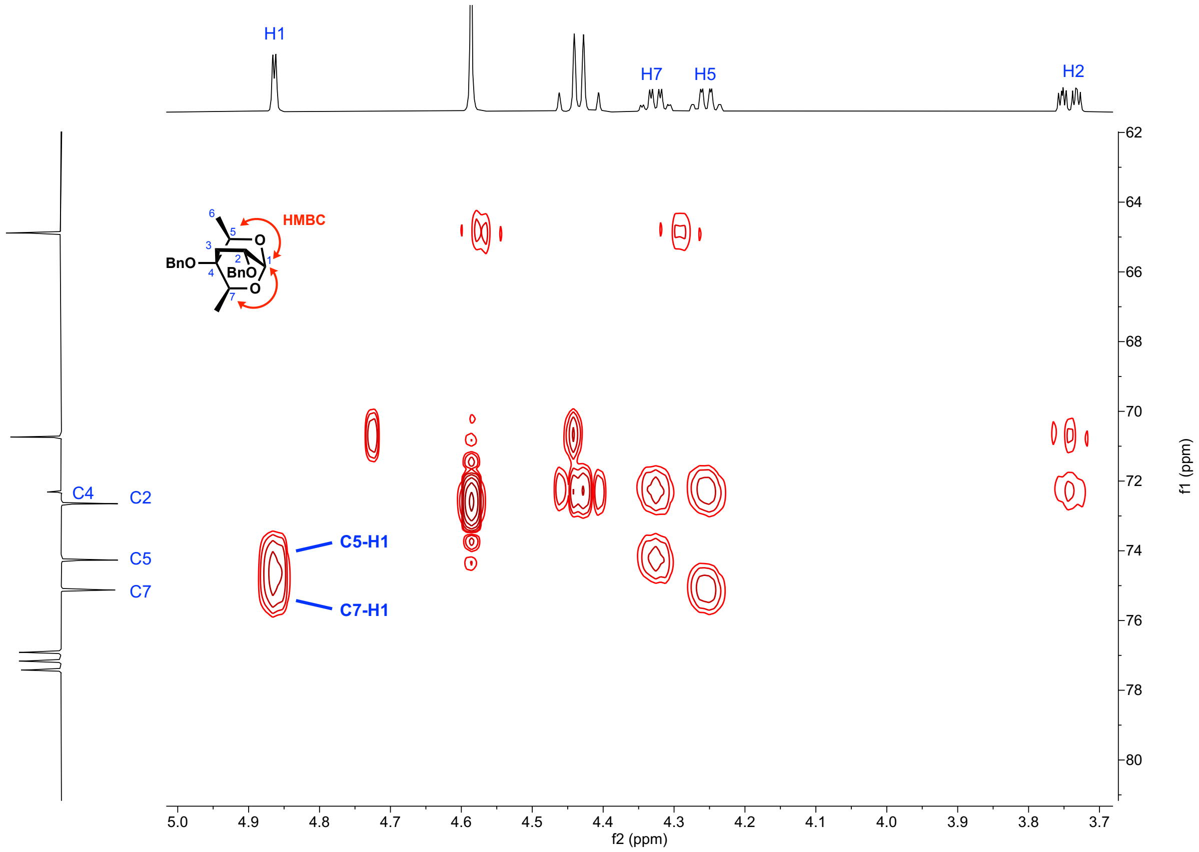

Figure S17. HMBC spectrum of compound **S73**. The key long-range heteronuclear correlation for **S73** can be found between C5-H1 and C7-H1.

Compound S80

¹H NMR H-H coupling constants

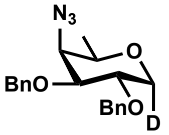

**H-1:** d, J = 5.6 Hz (eq$^{H\text{-}1}$-ax$^{H\text{-}2}$)
**H-2:** dd, J = 5.6 Hz (ax$^{H\text{-}2}$-eq$^{H\text{-}1}$), 9.0 Hz (ax$^{H\text{-}2}$-ax$^{H\text{-}3}$)
**H-3:** dd, J = 9.0 Hz (ax$^{H\text{-}3}$-ax$^{H\text{-}2}$), 3.7 Hz (ax$^{H\text{-}3}$-eq$^{H\text{-}4}$)
**H-4:** dd, J = 3.7 Hz (eq$^{H\text{-}4}$-ax$^{H\text{-}3}$), 1.3 Hz (eq$^{H\text{-}4}$-ax$^{H\text{-}5}$)
**H-5:** qd, J = 1.3 Hz (ax$^{H\text{-}5}$-eq$^{H\text{-}4}$), 6.3 Hz (ax$^{H\text{-}5}$–H-6)

Compound S75-α

¹H NMR H-H coupling constants

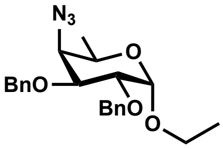

**H-1:** d, J = 3.8 Hz (eq$^{H\text{-}1}$-ax$^{H\text{-}2}$)
**H-2:** dd, J = 3.8 Hz (ax$^{H\text{-}2}$-eq$^{H\text{-}1}$), 9.9 Hz (ax$^{H\text{-}2}$-ax$^{H\text{-}3}$)
**H-3:** dd, J = 9.9 Hz (ax$^{H\text{-}3}$-ax$^{H\text{-}2}$), 3.7 Hz (ax$^{H\text{-}3}$-eq$^{H\text{-}4}$)
**H-4:** dd, J = 3.8 Hz (eq$^{H\text{-}4}$-ax$^{H\text{-}3}$), 1.5 Hz (eq$^{H\text{-}4}$-ax$^{H\text{-}5}$)
**H-5:** overlaps with other signals

Compound S75-β

¹H NMR H-H coupling constants

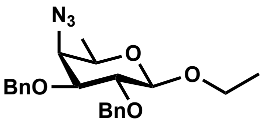

**H-1:** d, J = 7.3 Hz (eq$^{H\text{-}1}$-ax$^{H\text{-}2}$)
**H-2:** dd, J = 7.1 Hz (ax$^{H\text{-}2}$-eq$^{H\text{-}1}$), 9.5 Hz (ax$^{H\text{-}2}$-ax$^{H\text{-}3}$)
**H-3:** overlaps with H-4
**H-4:** overlaps with H-3
**H-5:** overlaps with other signals

NMR spectra of new and selected compounds

^{1}H NMR, 400 MHz, $CDCl_3$ of **7**

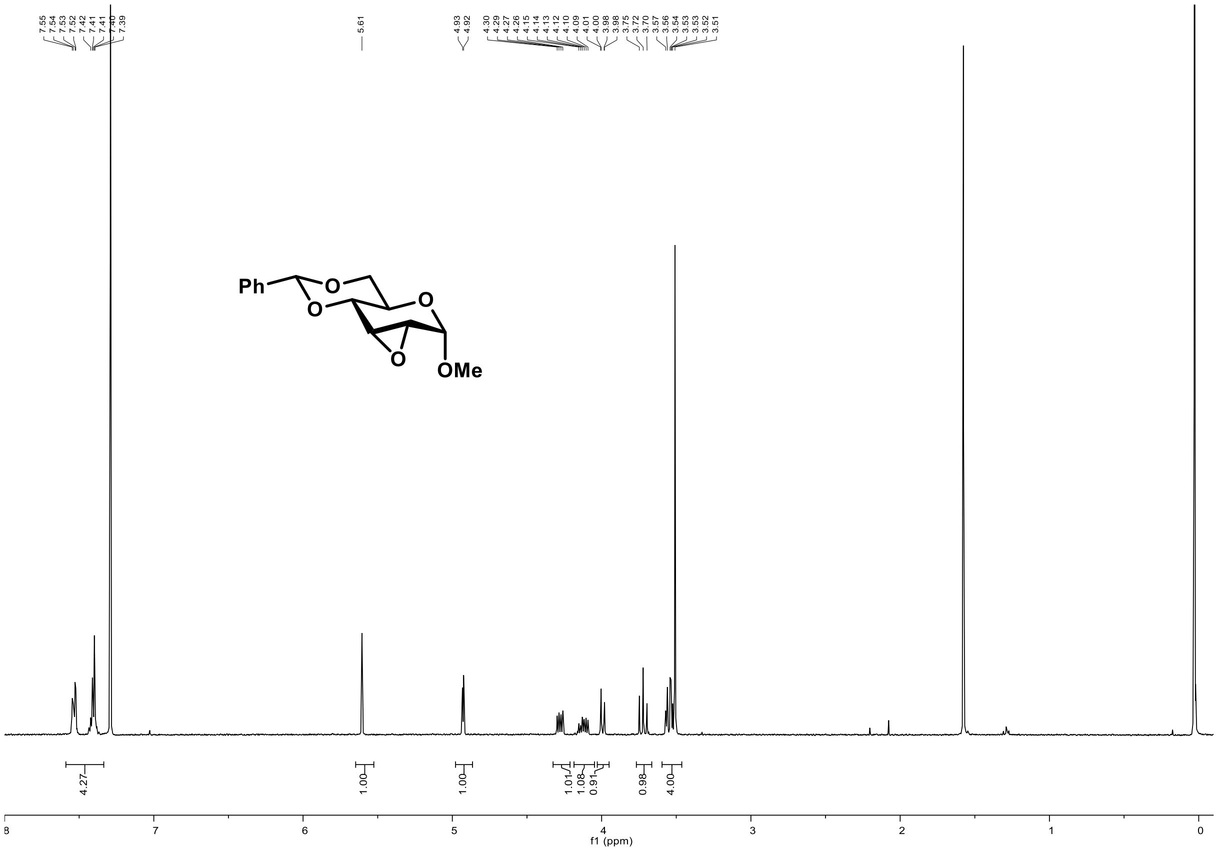

^{13}C NMR, 101 MHz, $CDCl_3$ of **7**

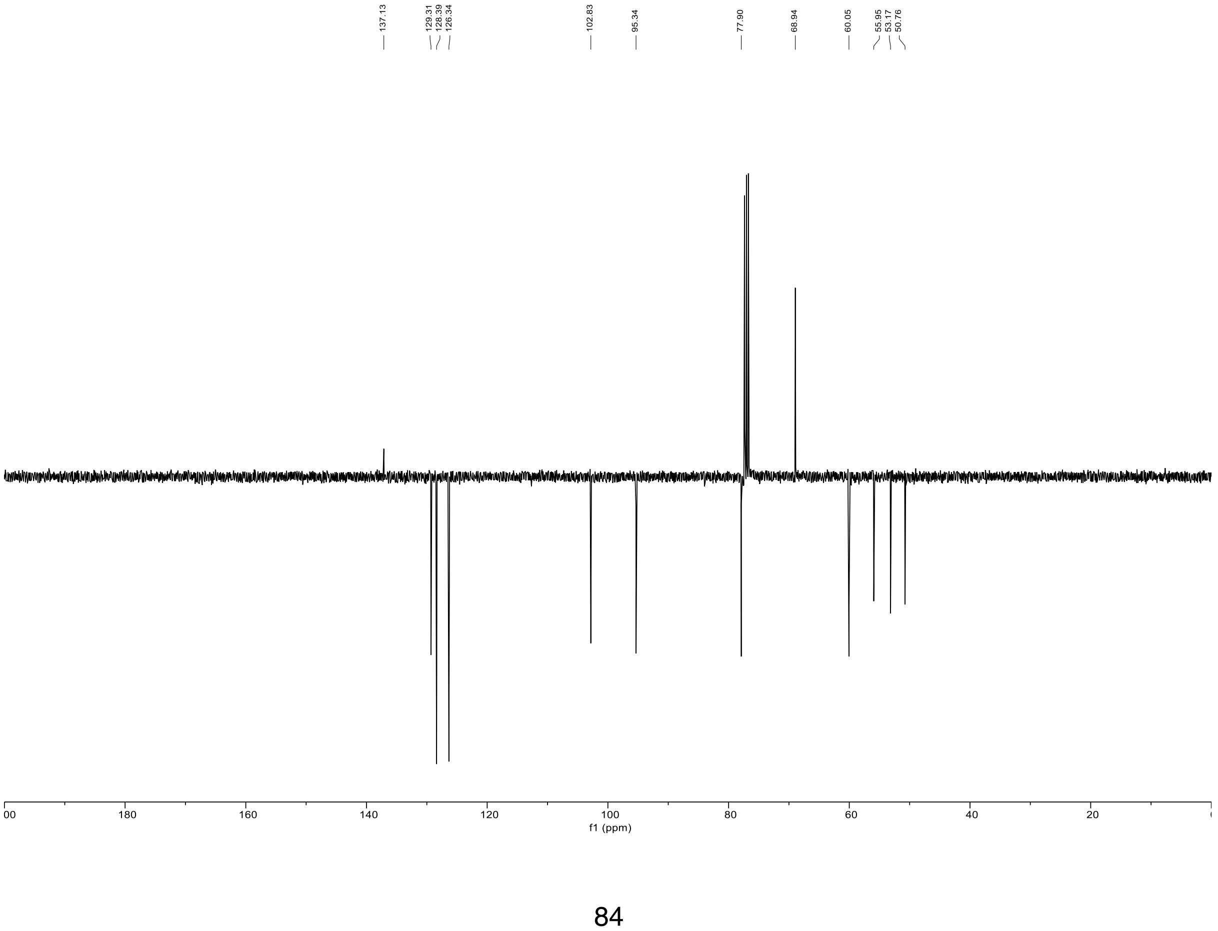

HH-COSY NMR, CDCl$_3$ of **7**

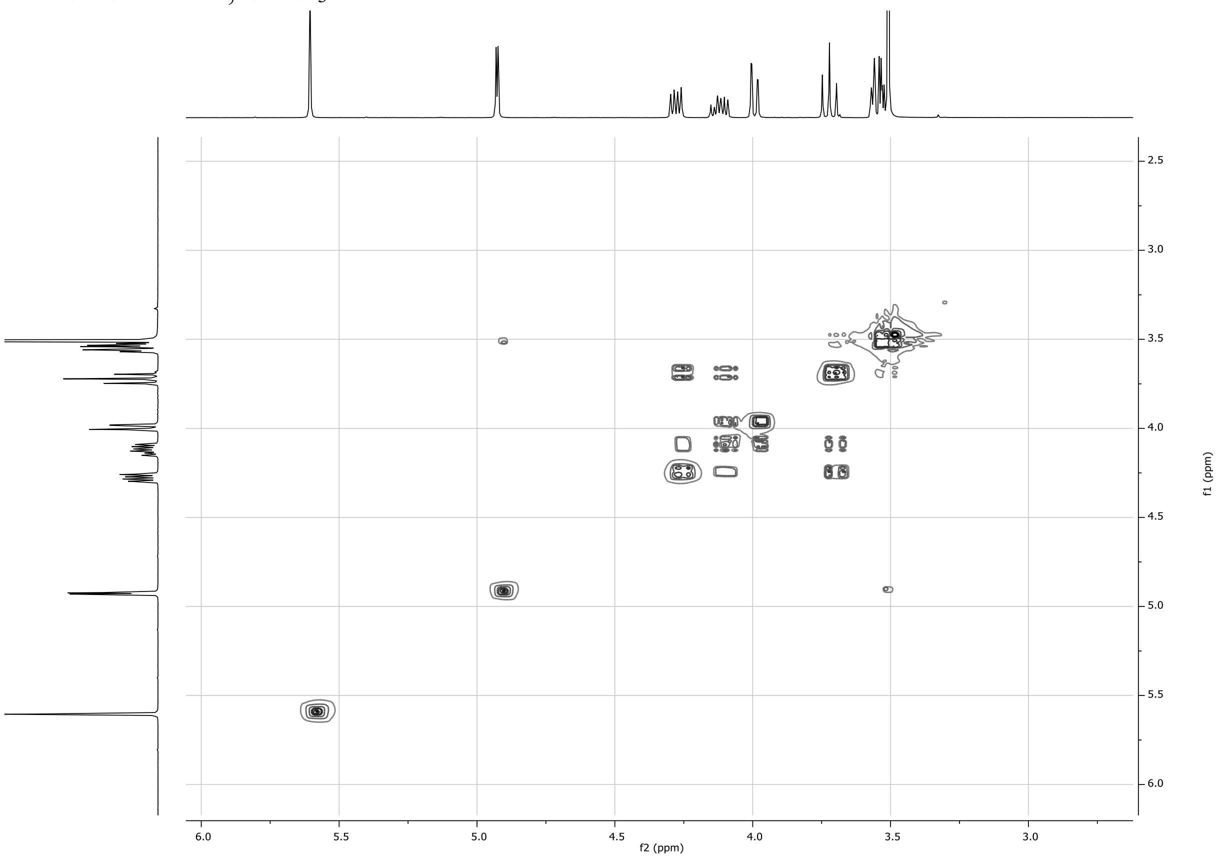

HSQC NMR, CDCl$_3$ of **7**

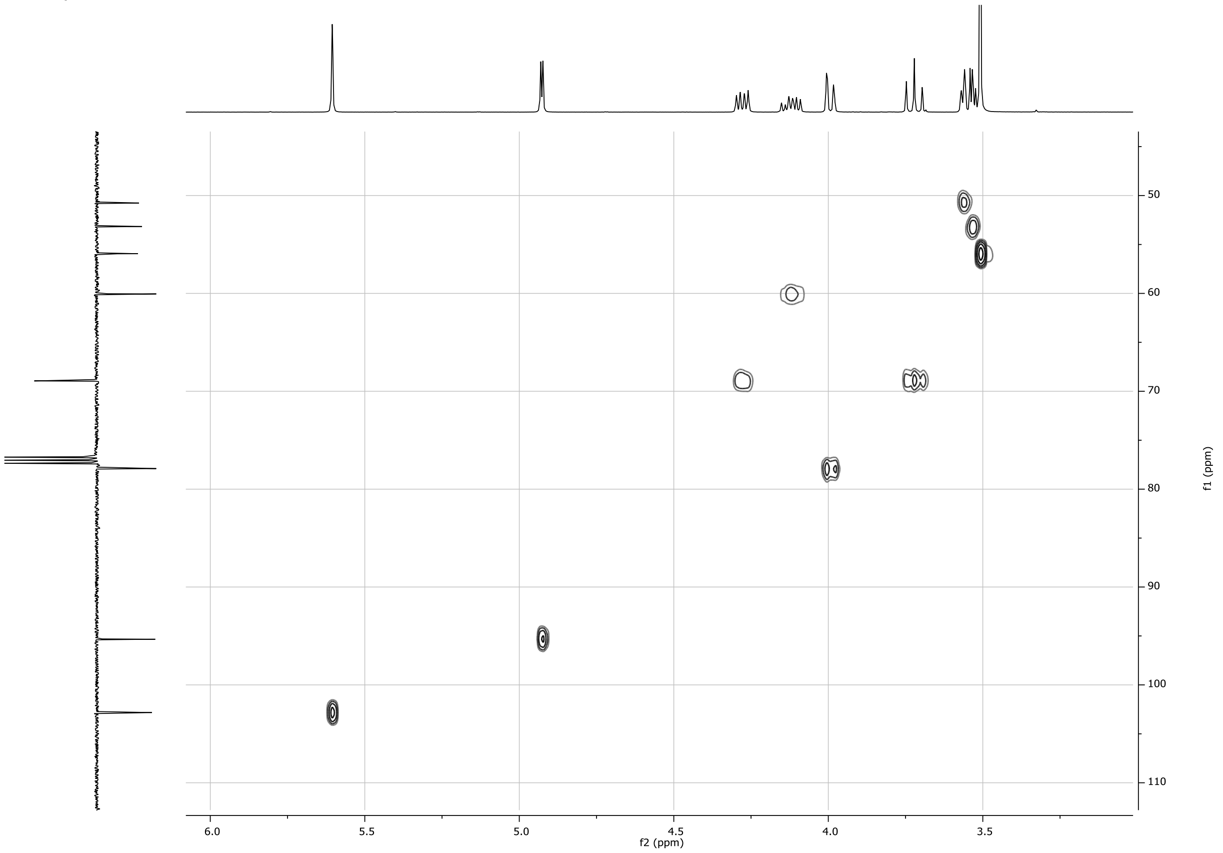

^{1}H NMR, 400 MHz, $CDCl_3$ of **8**

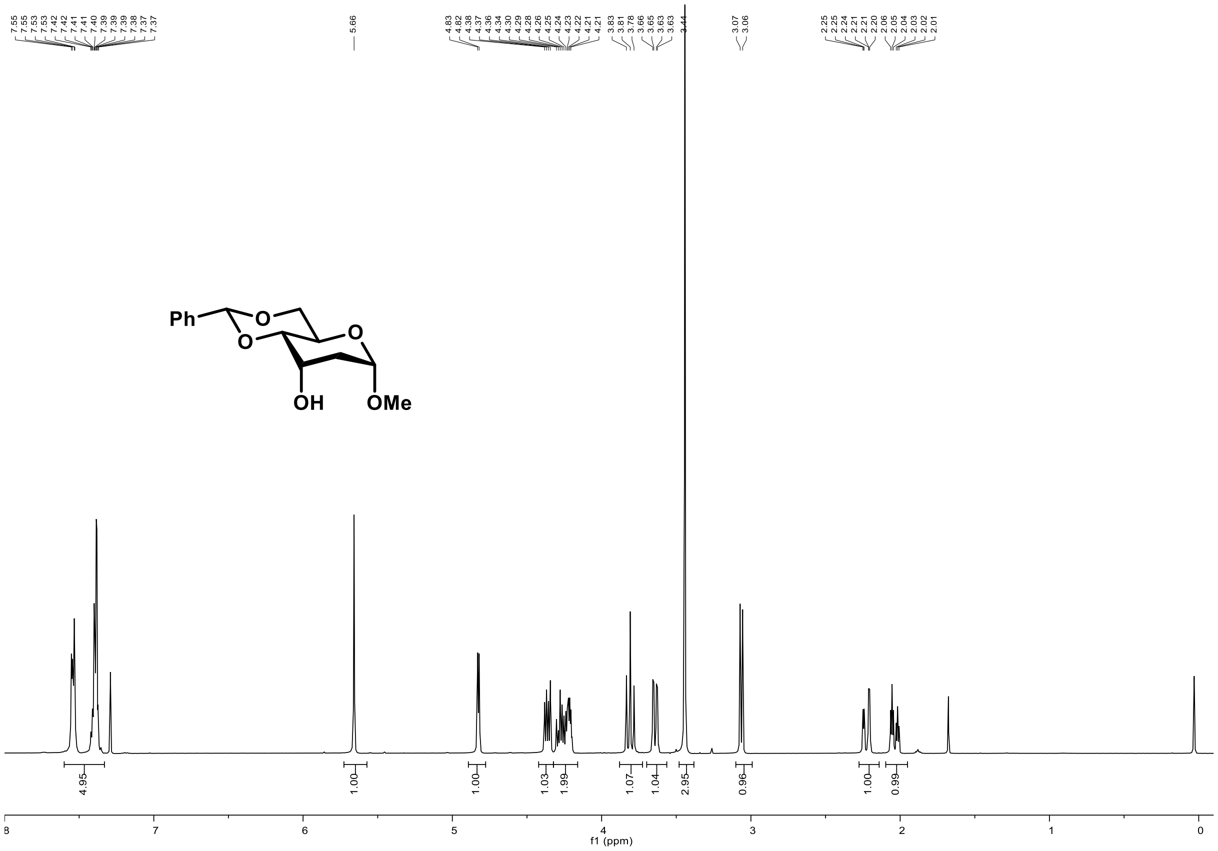

^{13}C NMR, 101 MHz, $CDCl_3$ of **8**

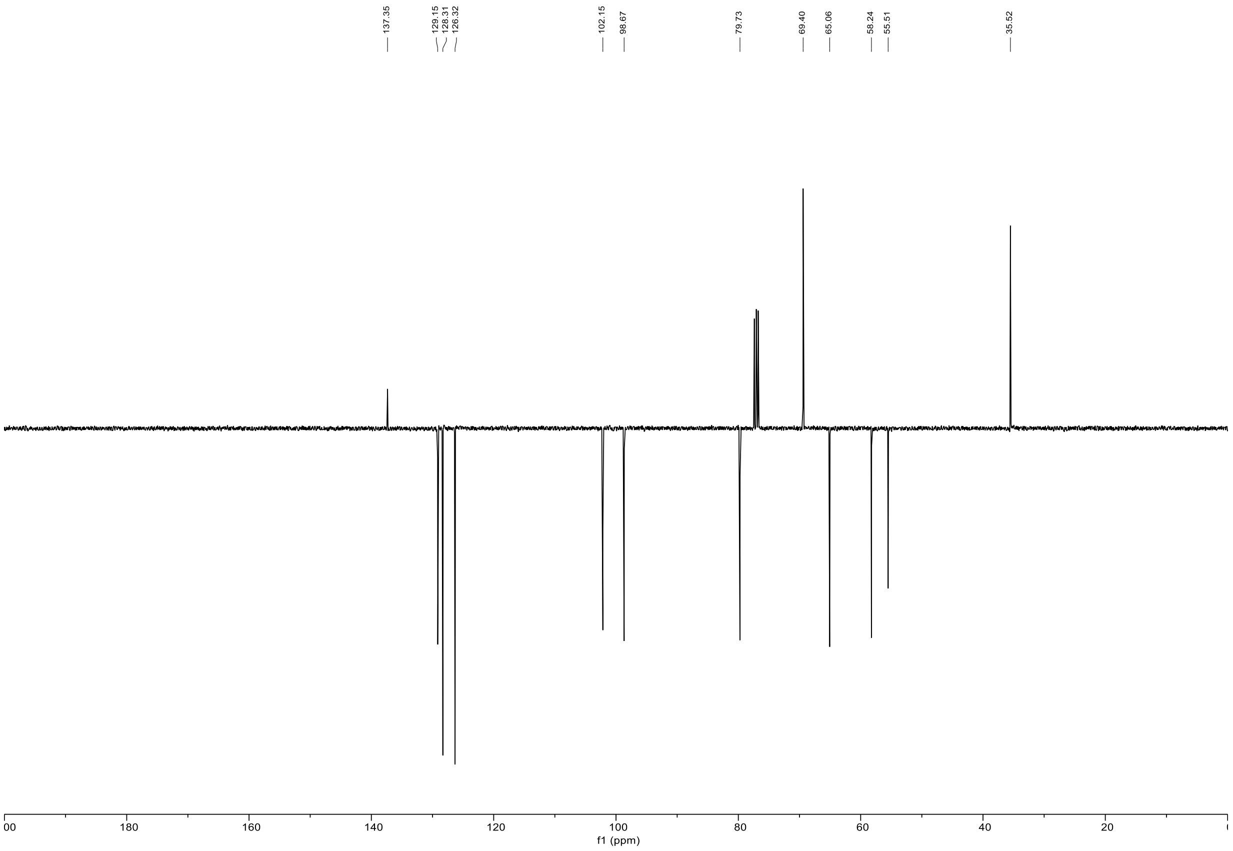

HH-COSY NMR, $CDCl_3$ of **8**

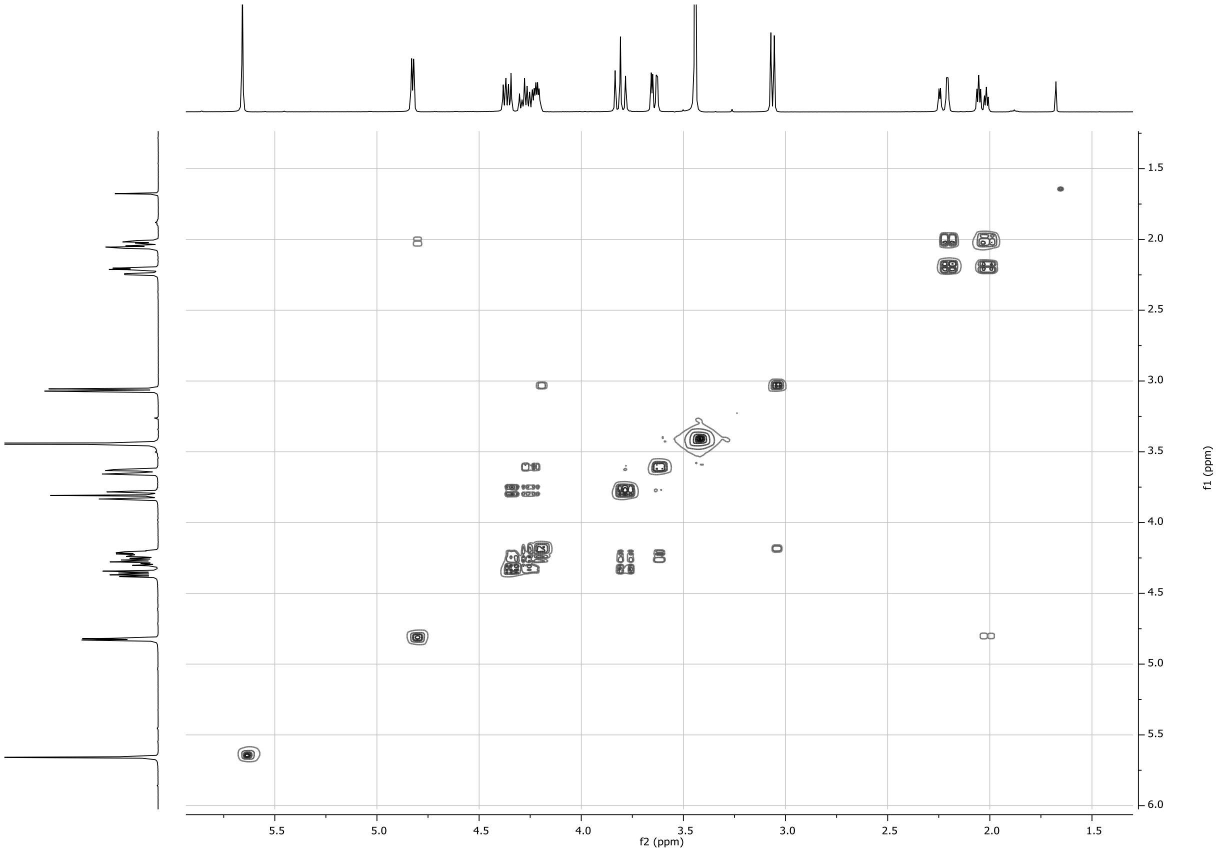

HSQC NMR, $CDCl_3$ of **8**

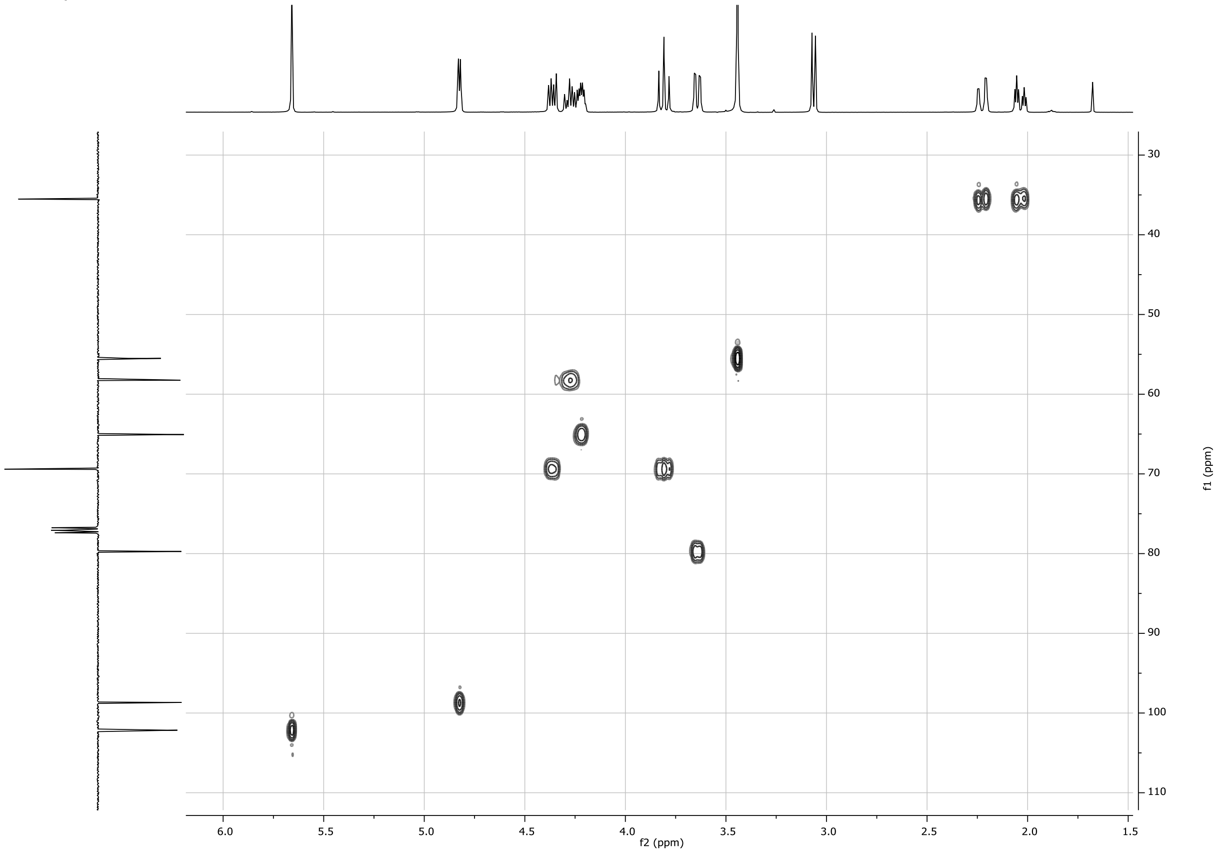

^{1}H NMR, 400 MHz, $CDCl_3$ of **9**

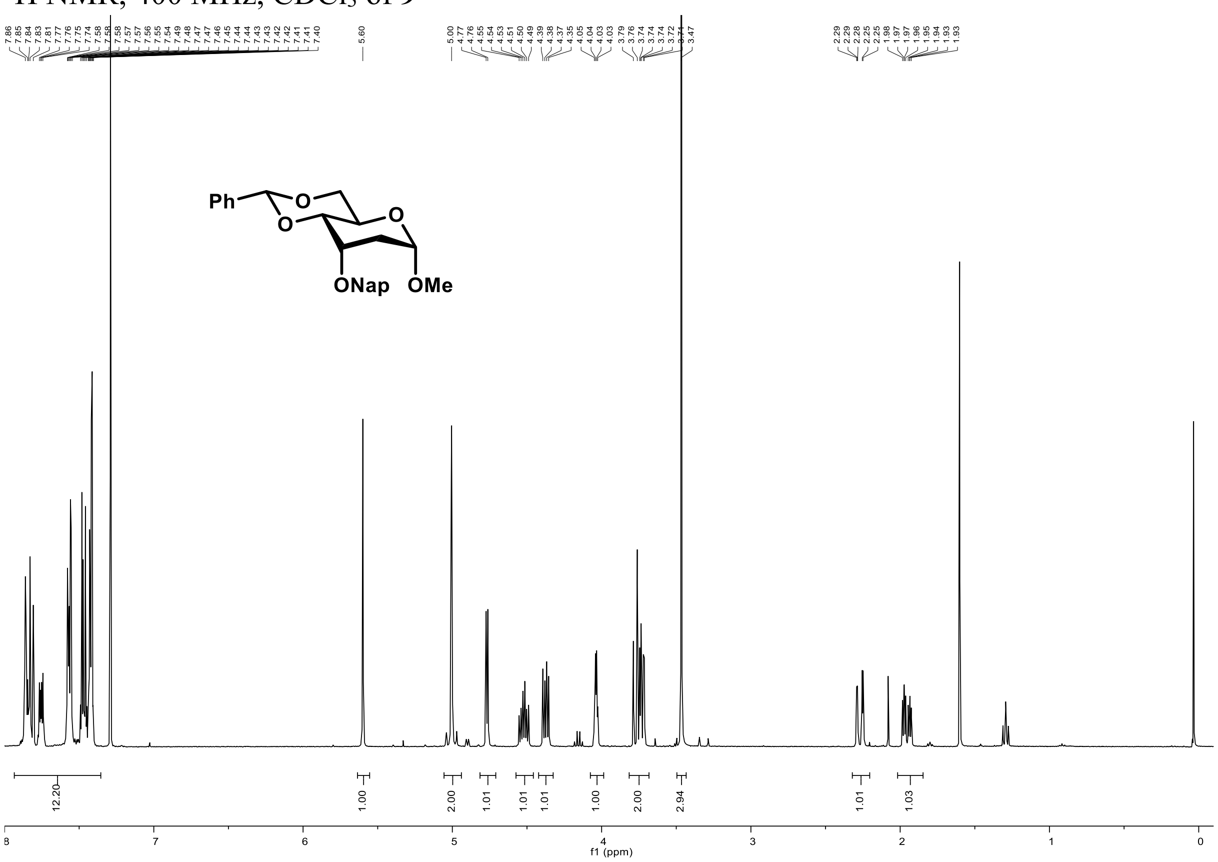

^{13}C NMR, 101 MHz, $CDCl_3$ of **9**

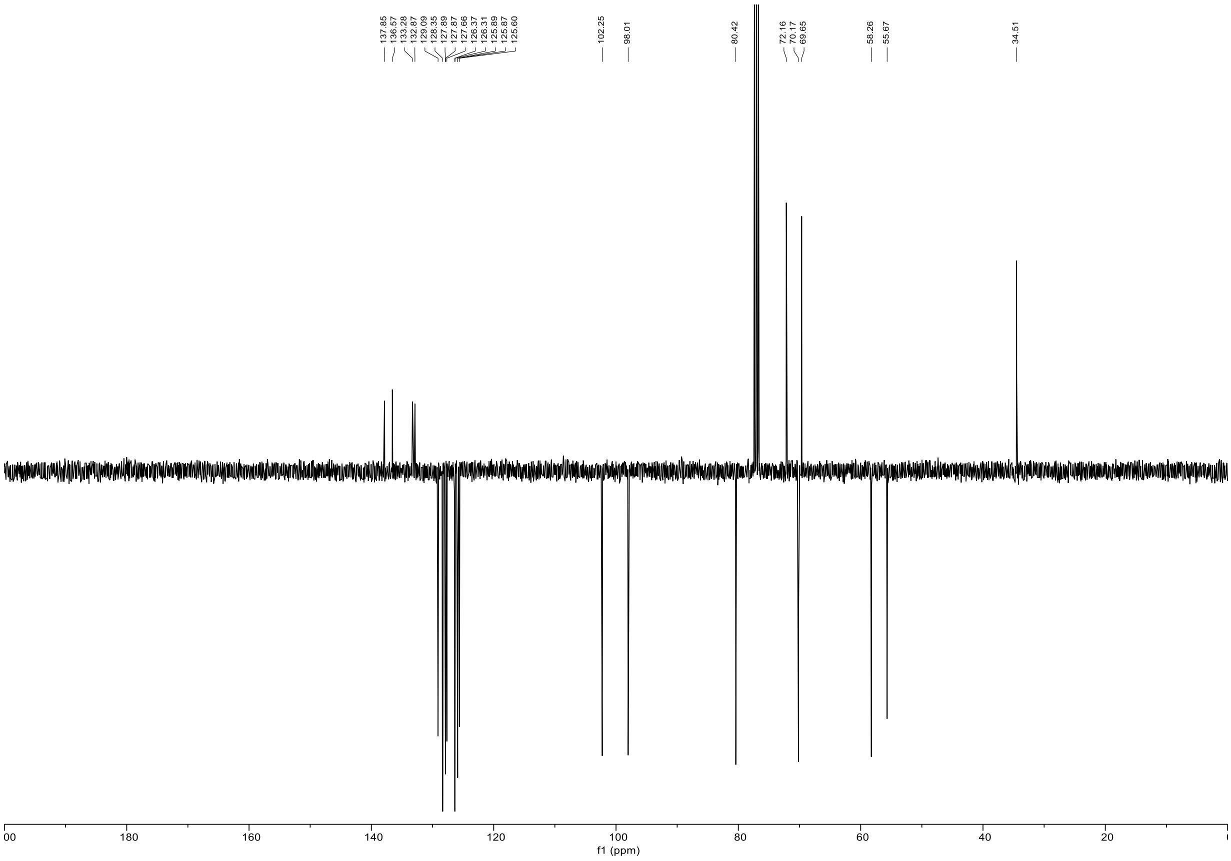

HH-COSY NMR, $CDCl_3$ of **9**

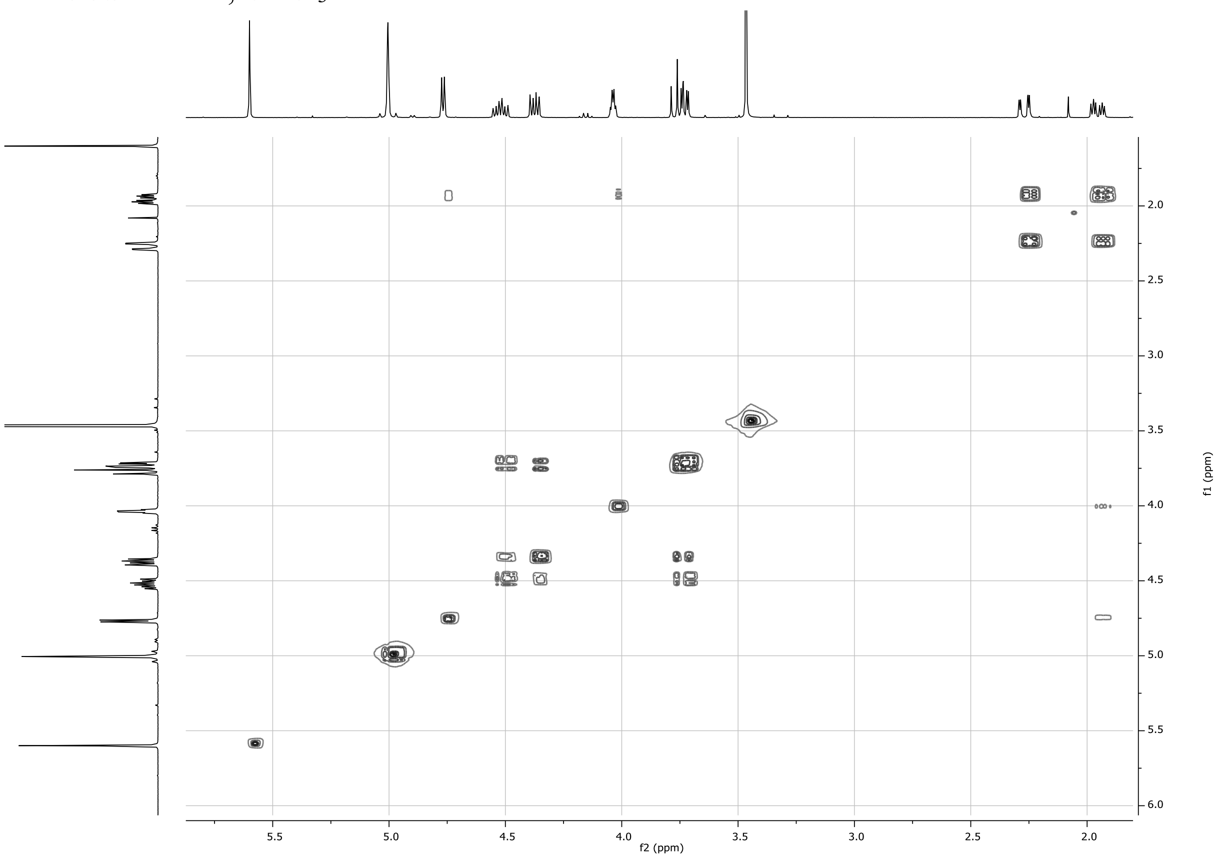

HSQC NMR, $CDCl_3$ of **9**

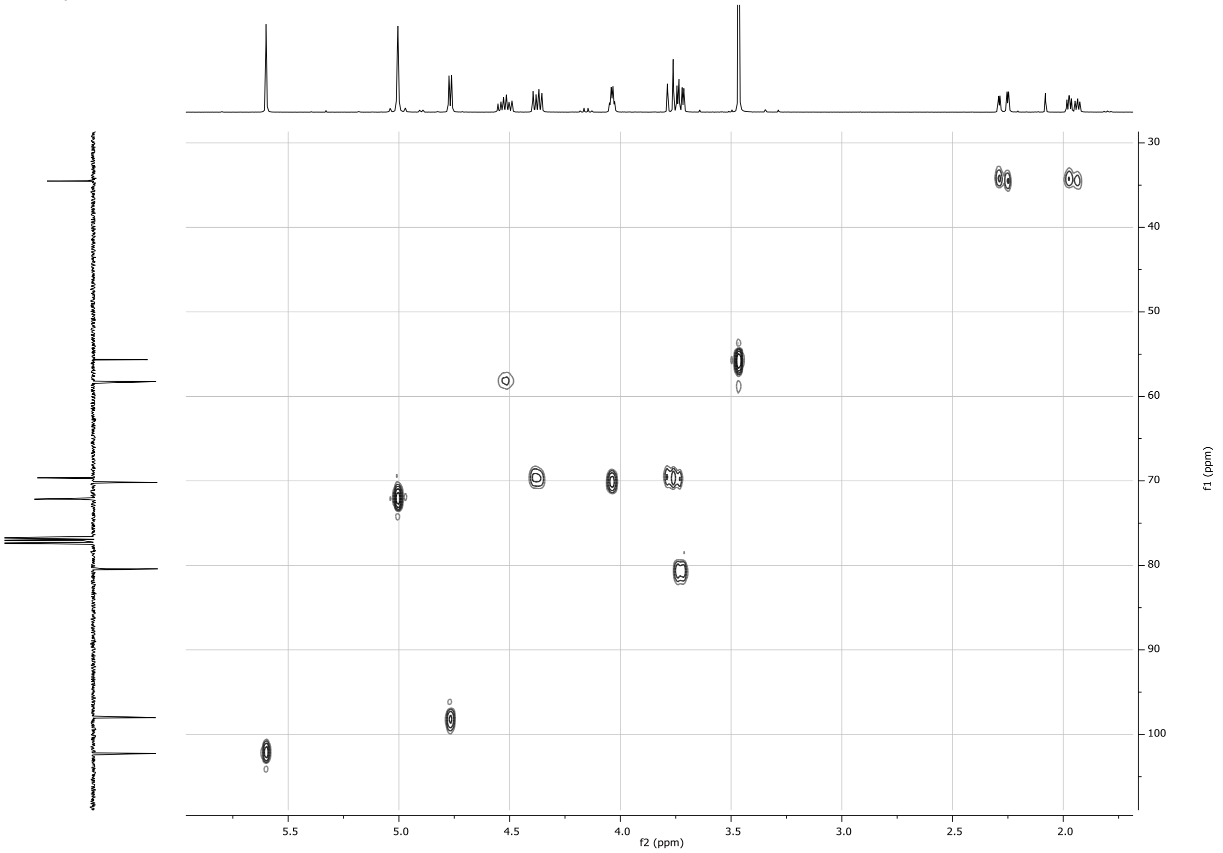

^{1}H NMR, 400 MHz, $CDCl_3$ of **10**

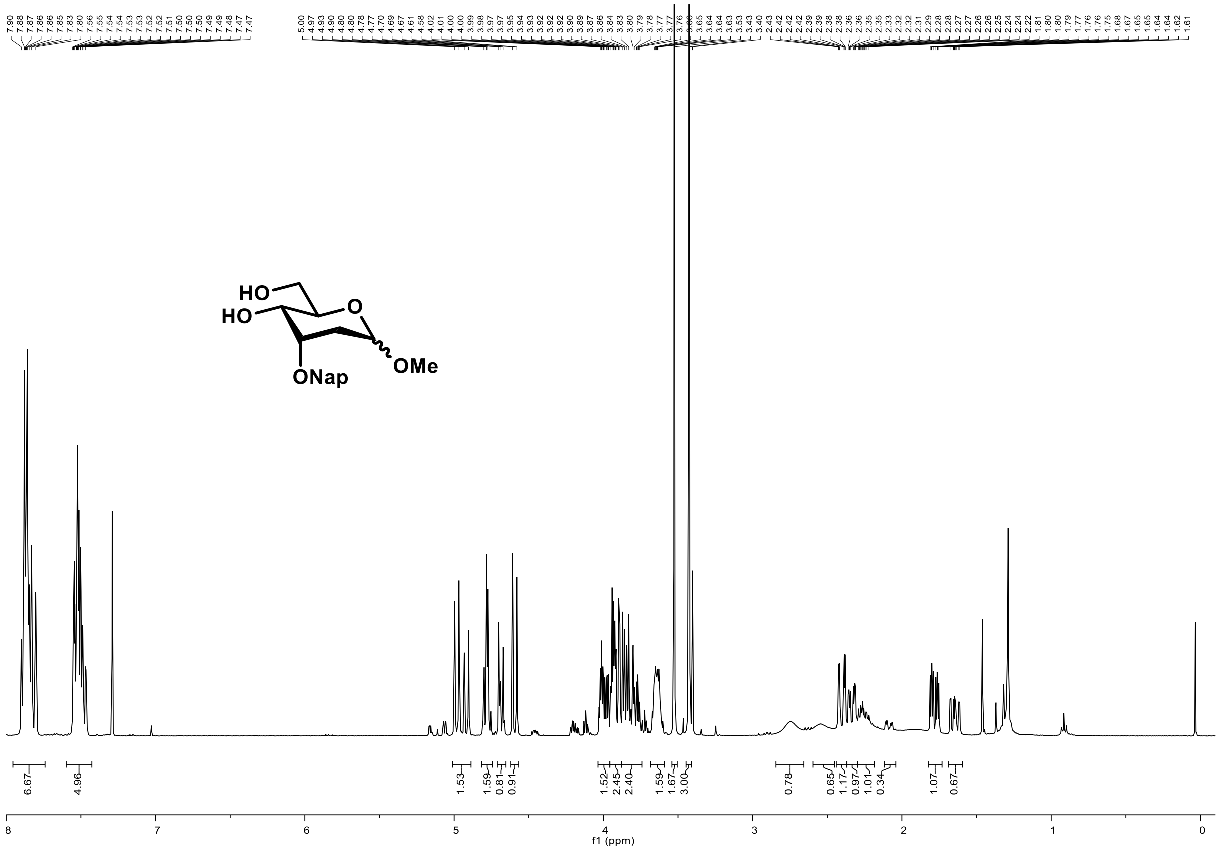

^{13}C NMR, 101 MHz, $CDCl_3$ of **10**

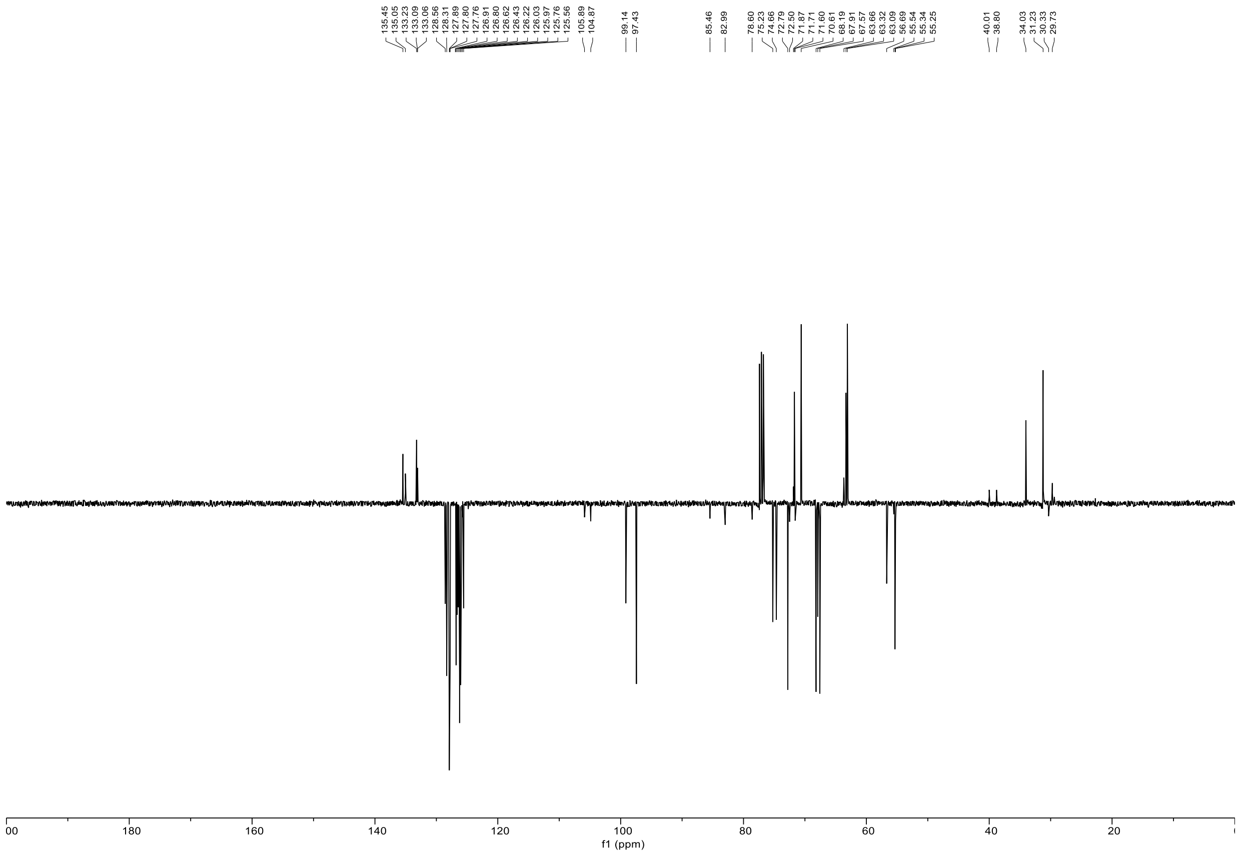

HH-COSY NMR, $CDCl_3$ of **10**

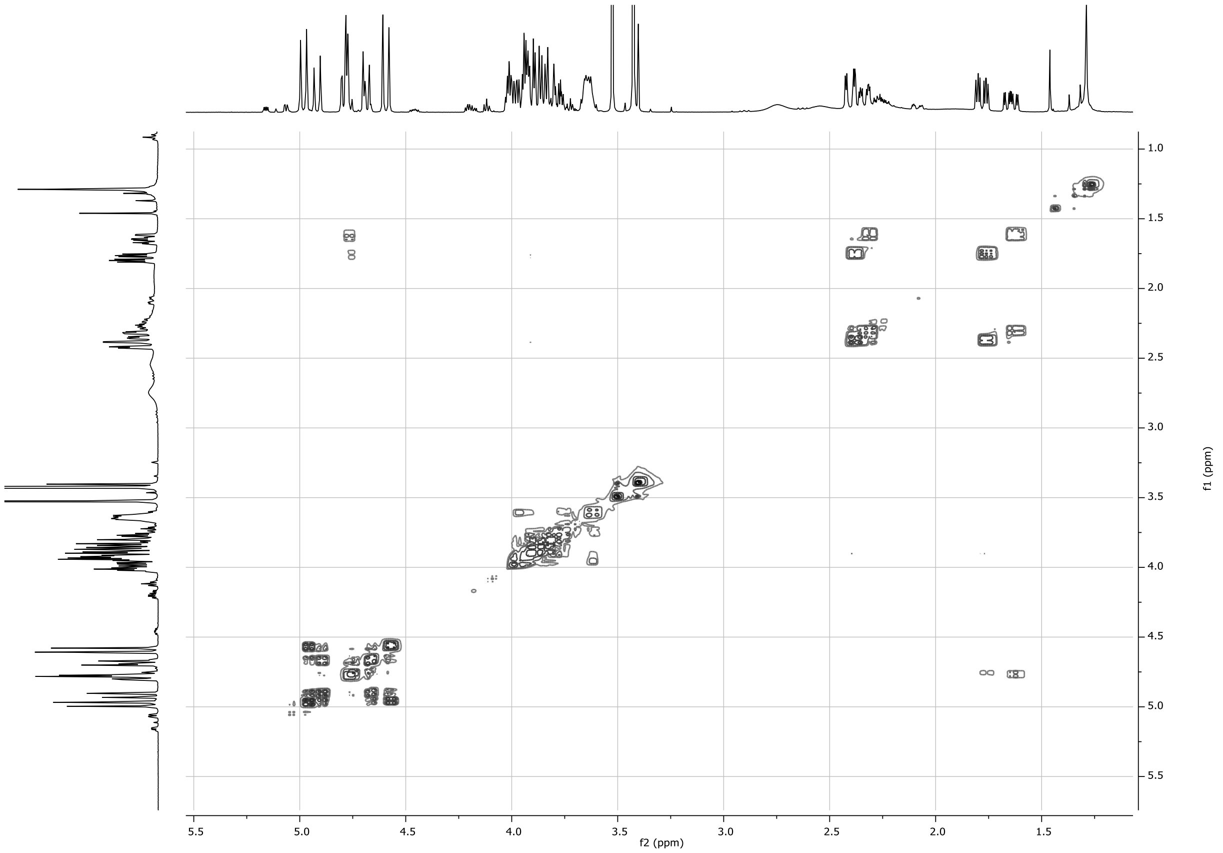

HSQC NMR, $CDCl_3$ of **10**

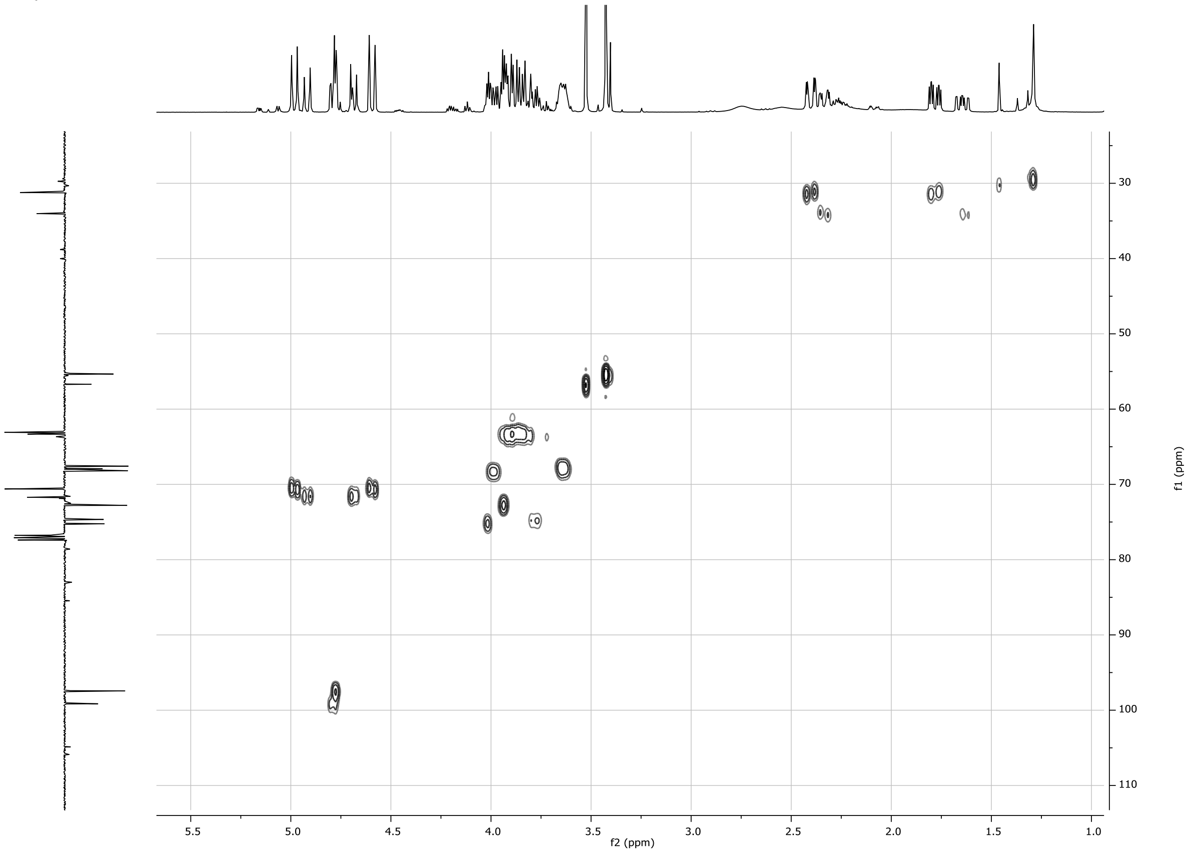

^{1}H NMR, 400 MHz, $CDCl_3$ of **11**

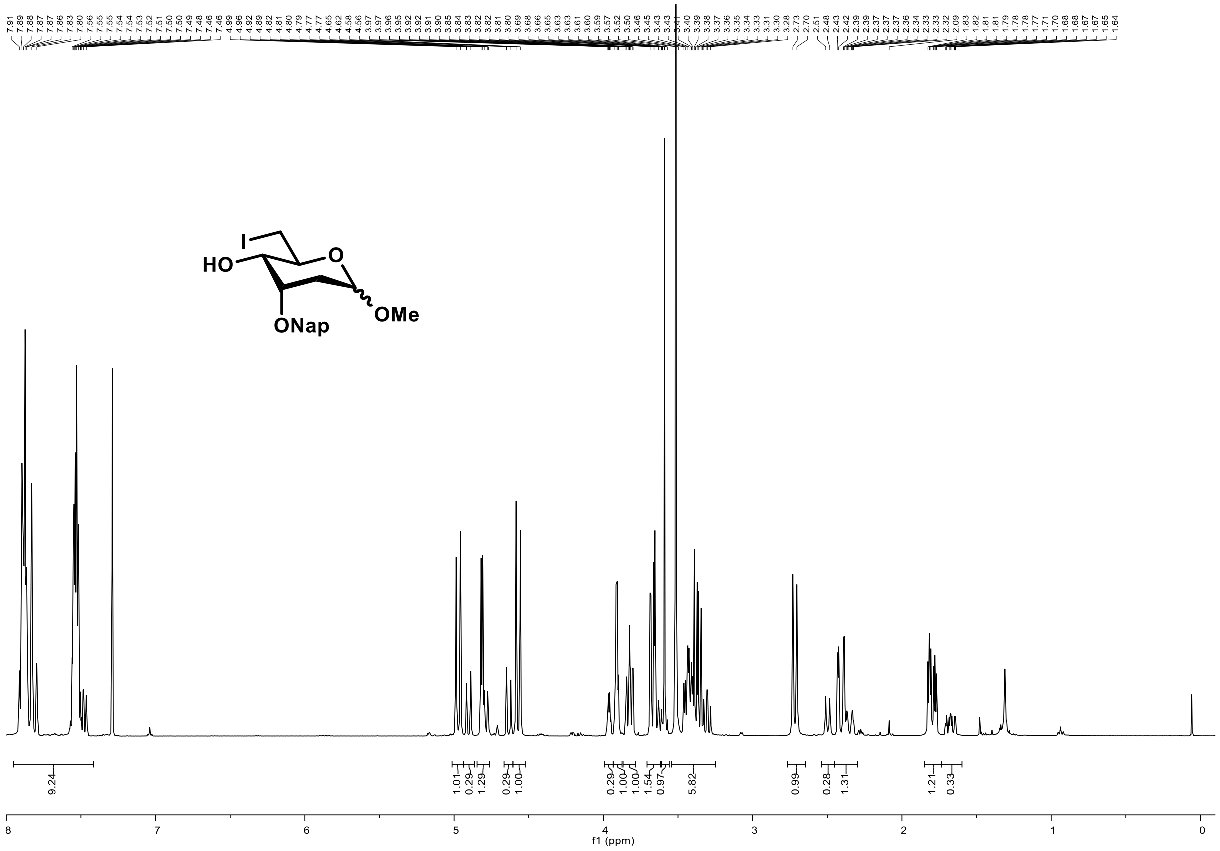

^{13}C NMR, 101 MHz, $CDCl_3$ of **11**

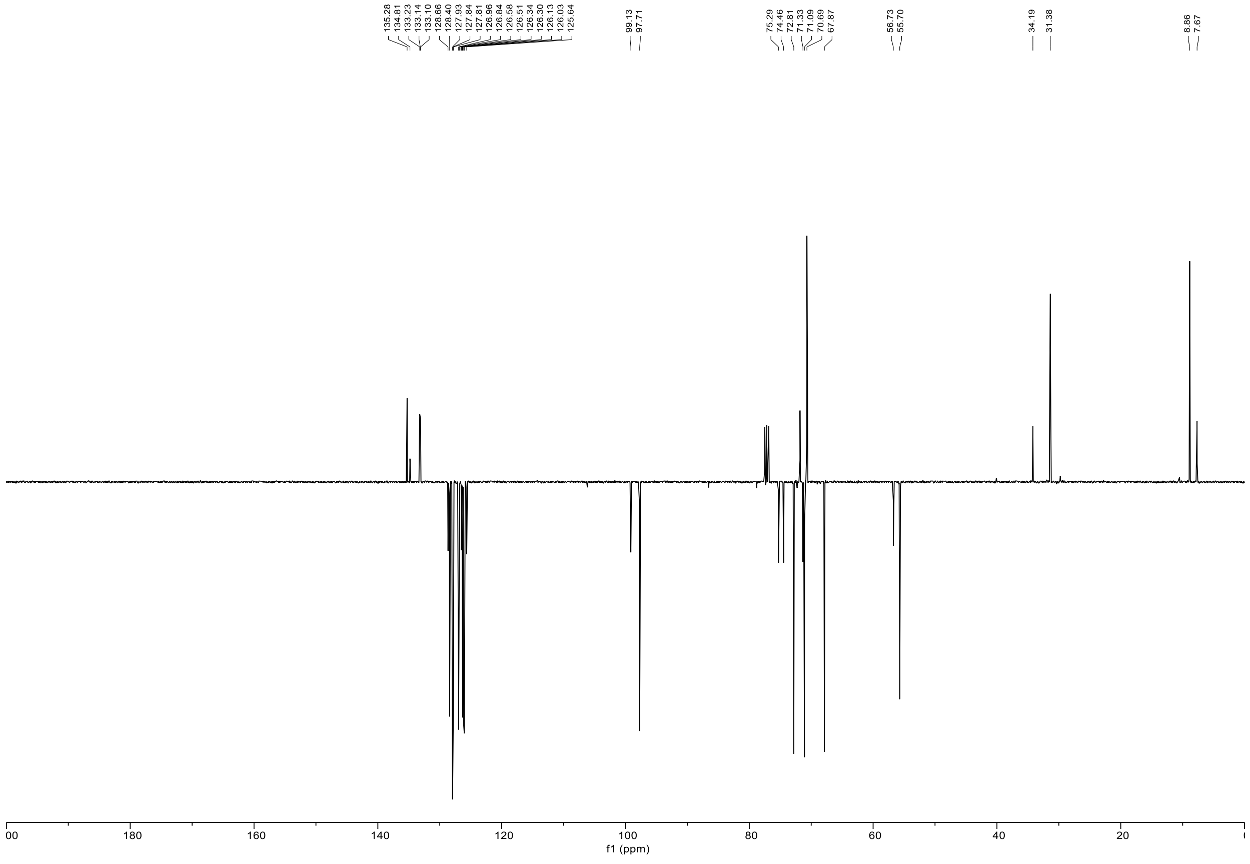

HH-COSY NMR, $CDCl_3$ of **11**

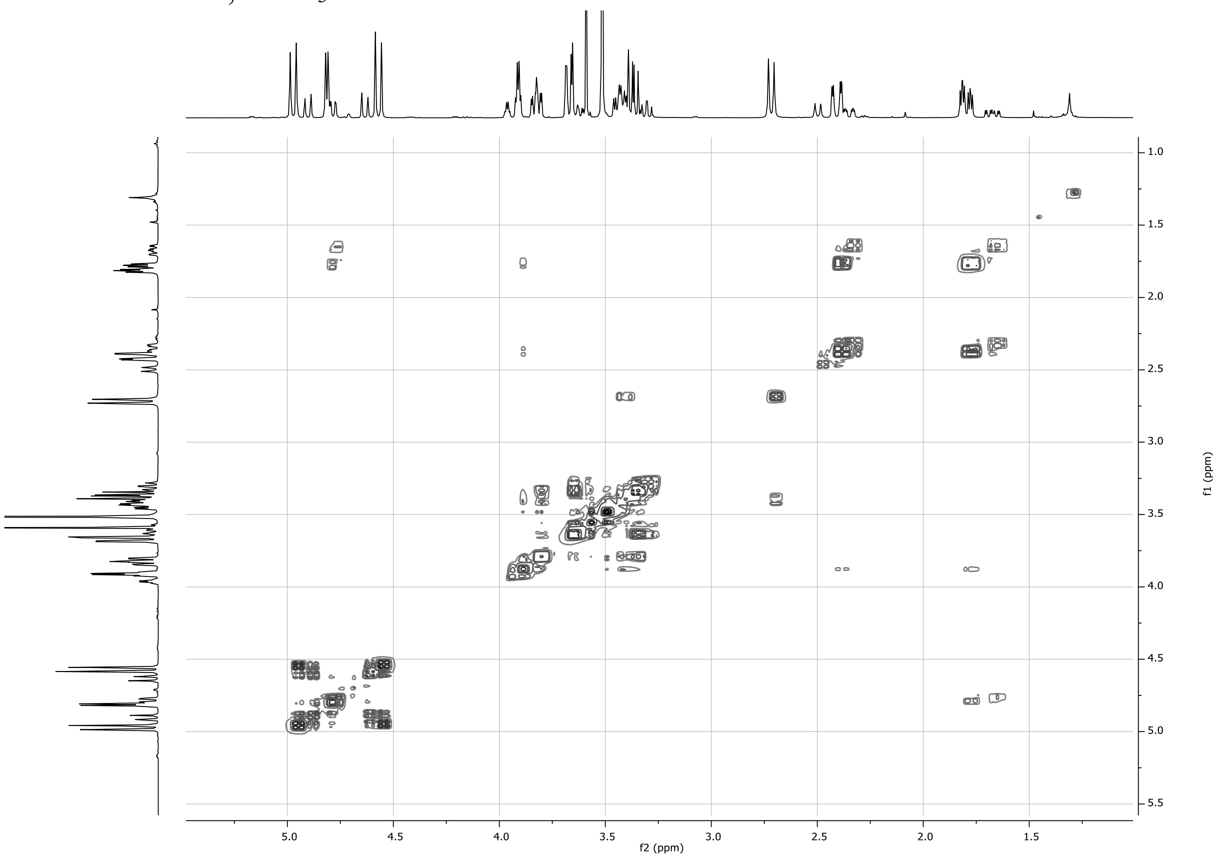

HSQC NMR, $CDCl_3$ of **11**

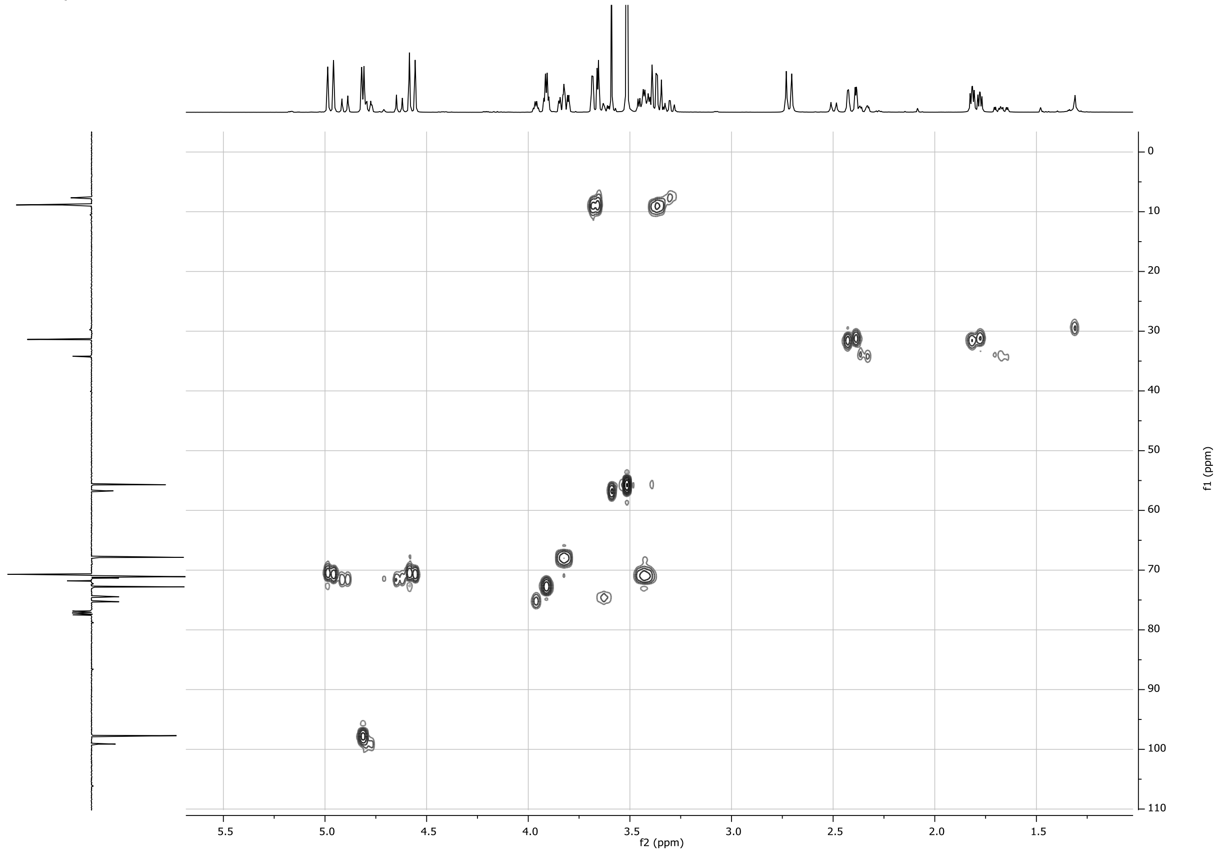

^{1}H NMR, 400 MHz, $CDCl_3$ of **12**

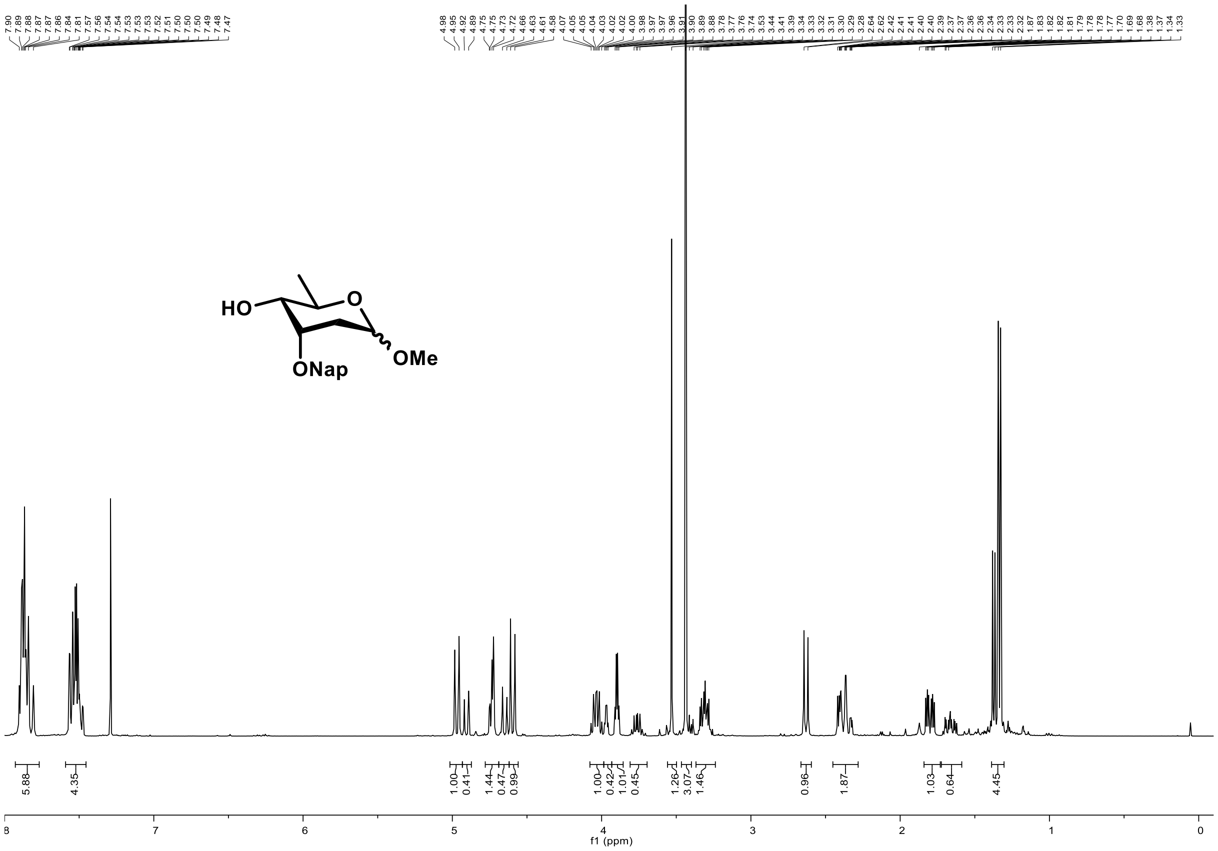

^{13}C NMR, 101 MHz, $CDCl_3$ of **12**

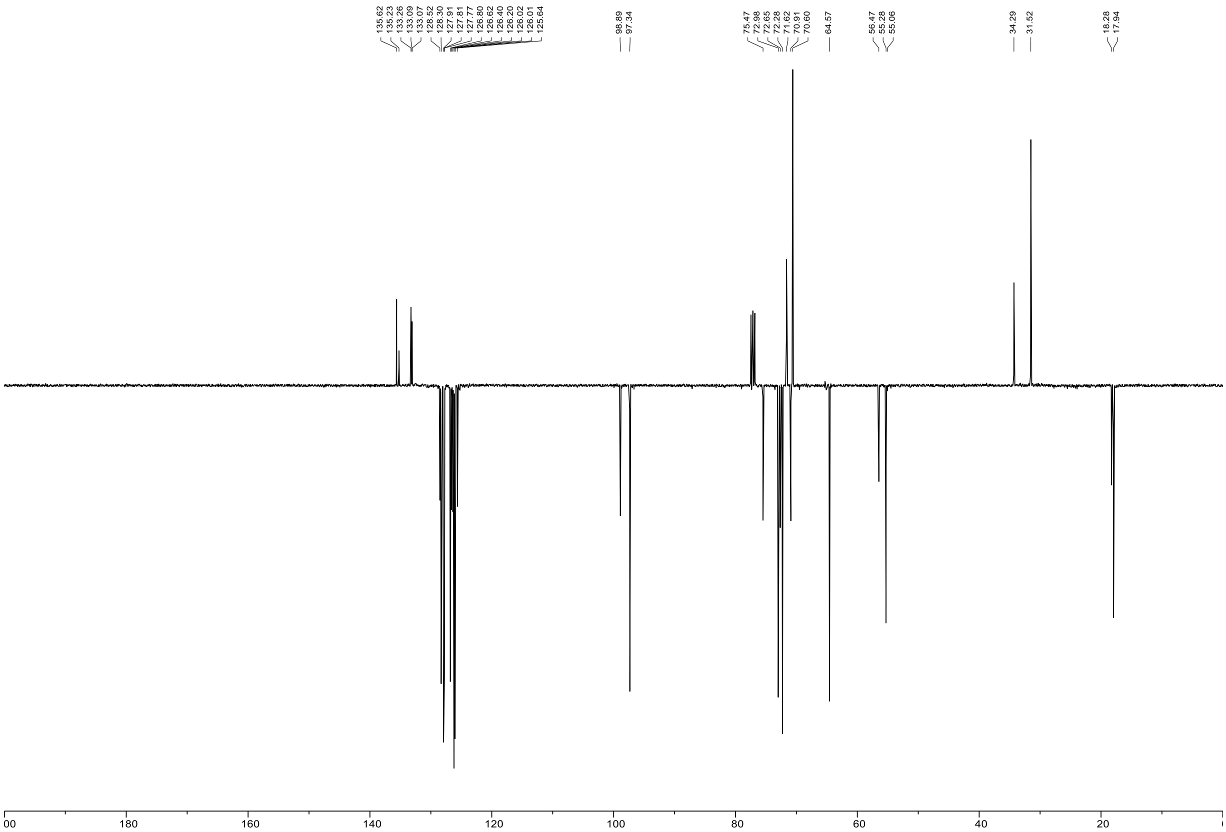

HH-COSY NMR, $CDCl_3$ of **12**

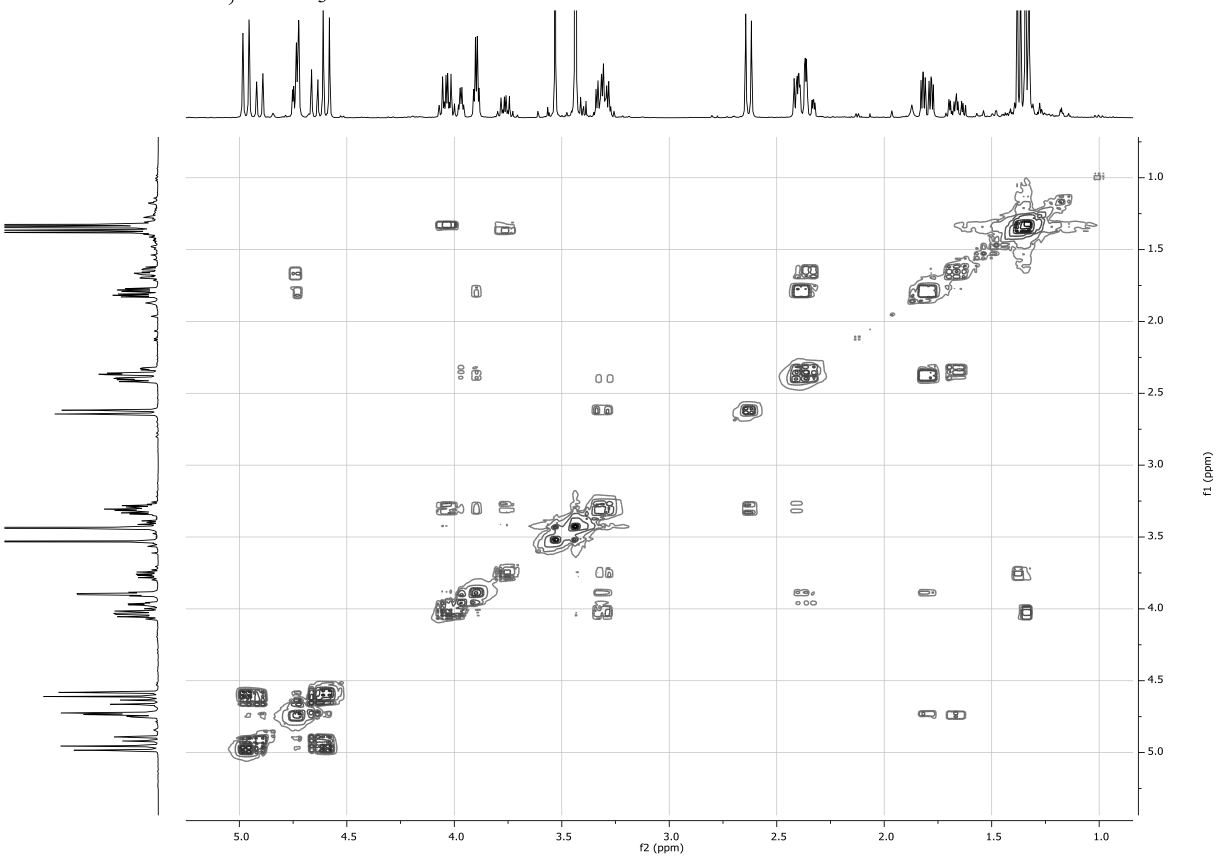

HSQC NMR, $CDCl_3$ of **12**

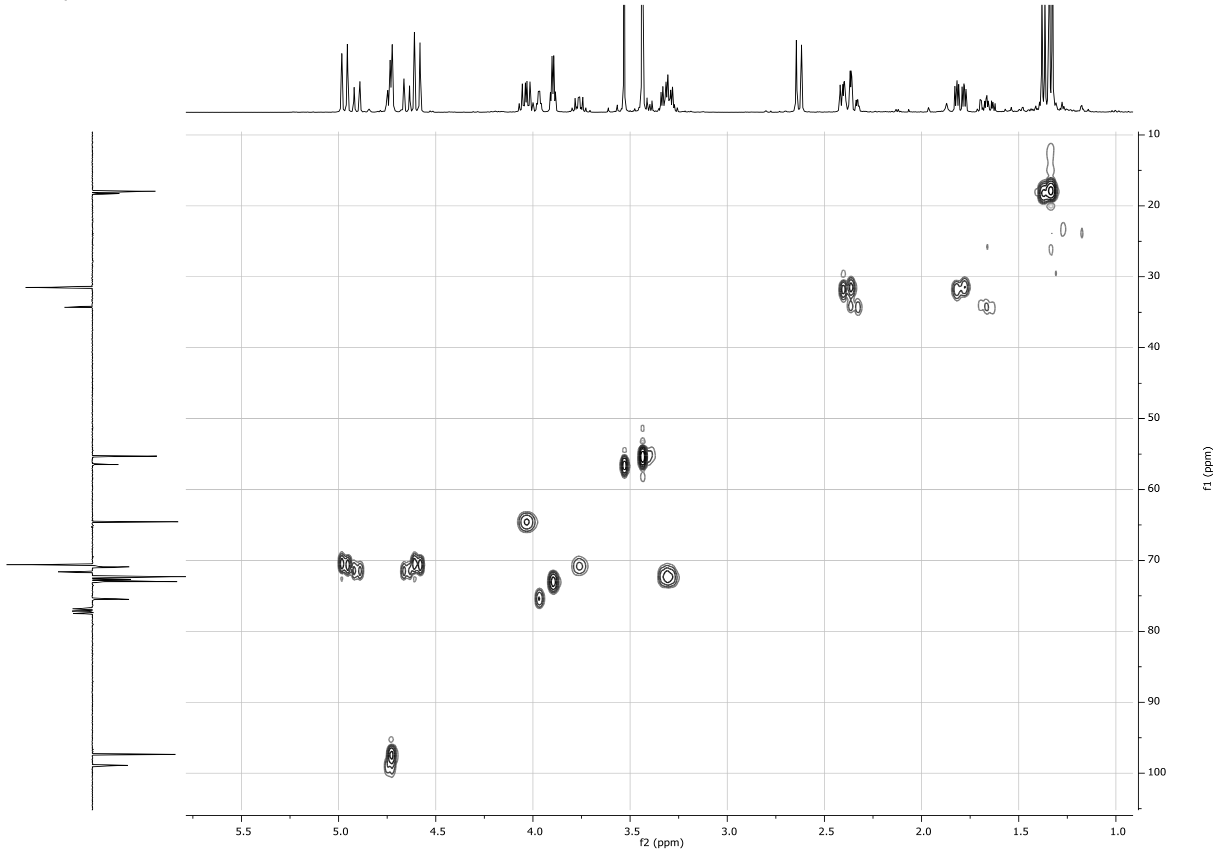

^{1}H NMR, 400 MHz, $CDCl_3$ of **13**

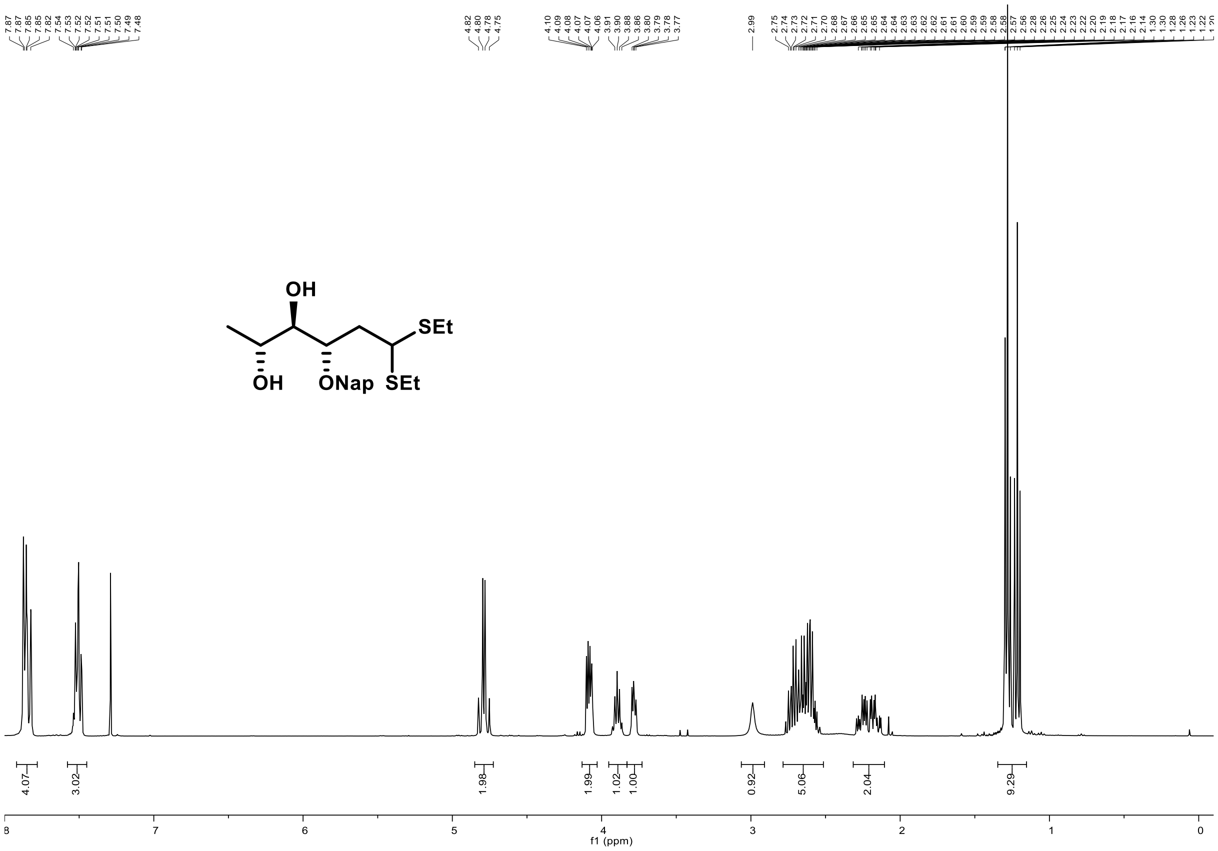

^{13}C NMR, 101 MHz, $CDCl_3$ of **13**

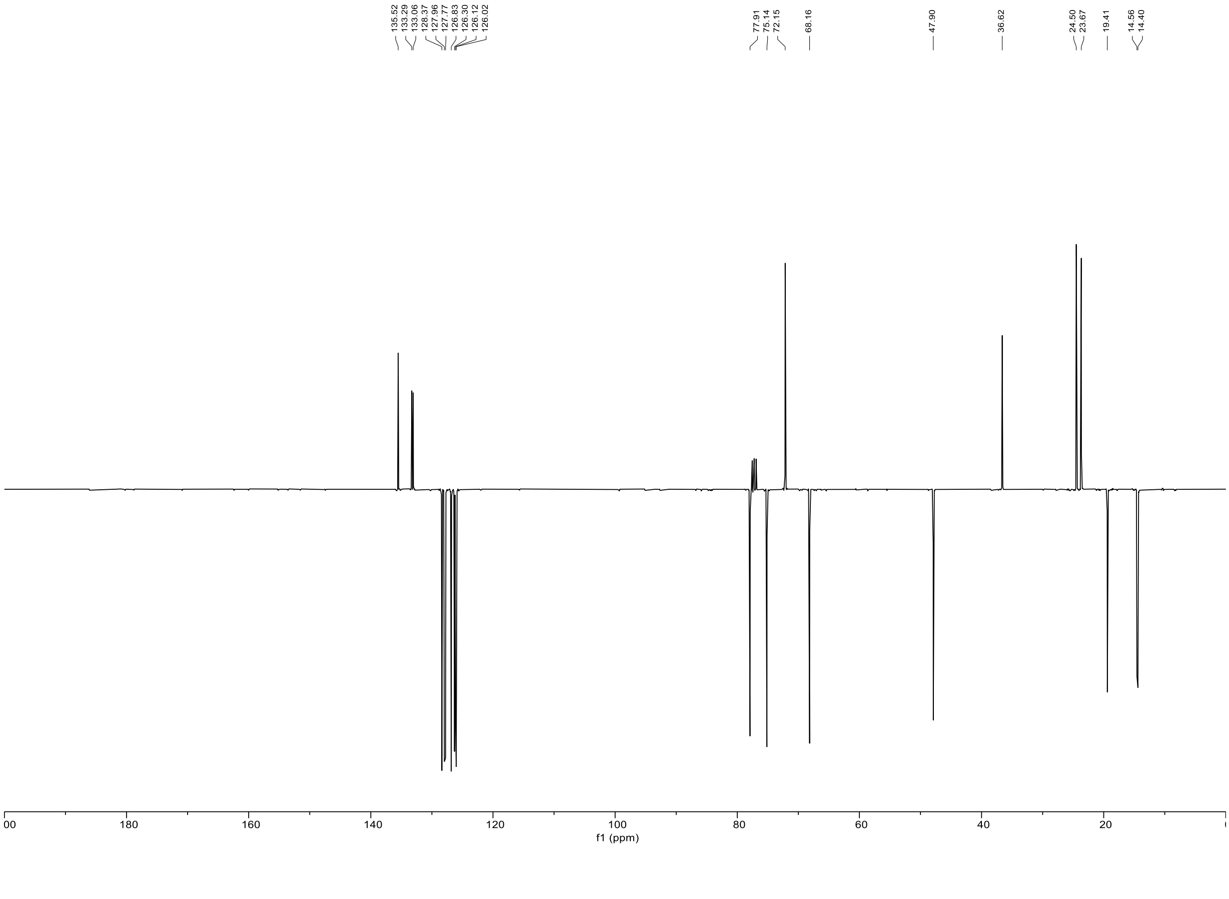

HH-COSY NMR, $CDCl_3$ of **13**

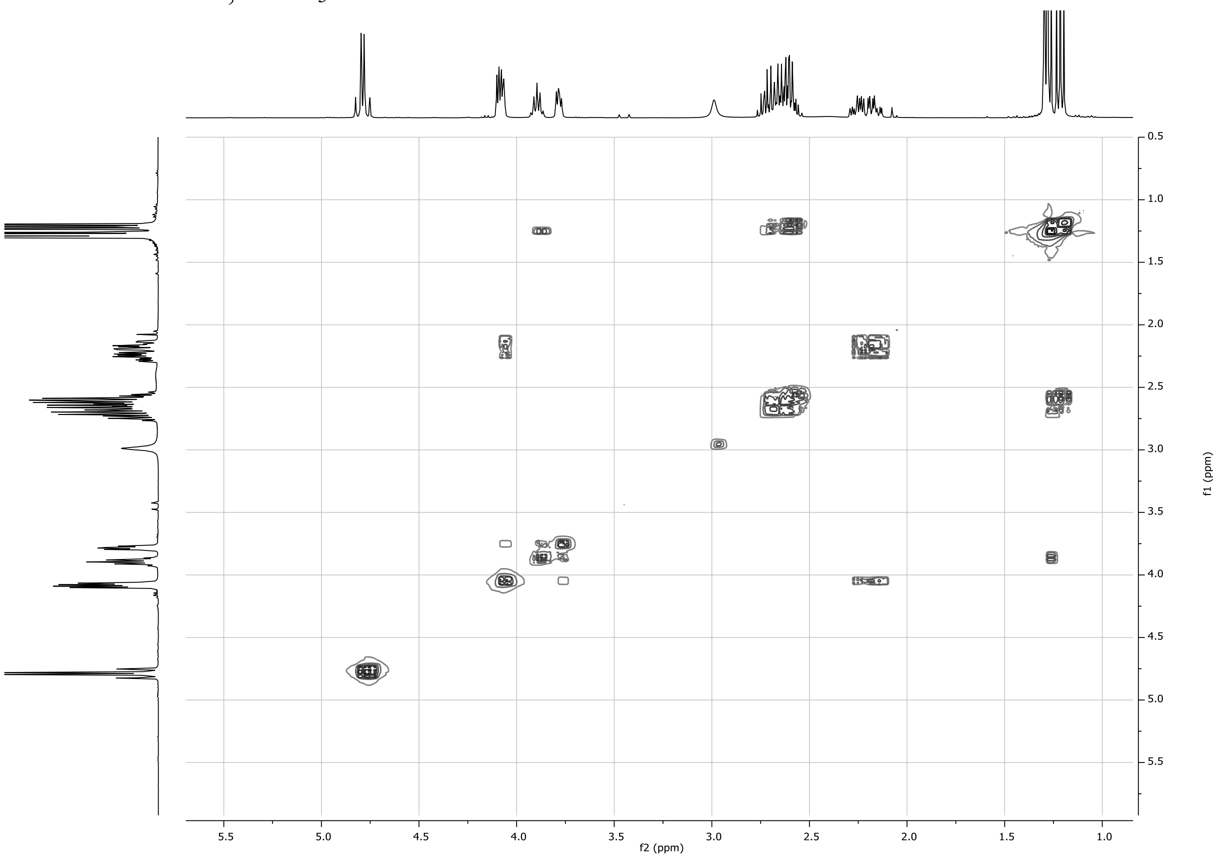

HSQC NMR, $CDCl_3$ of **13**

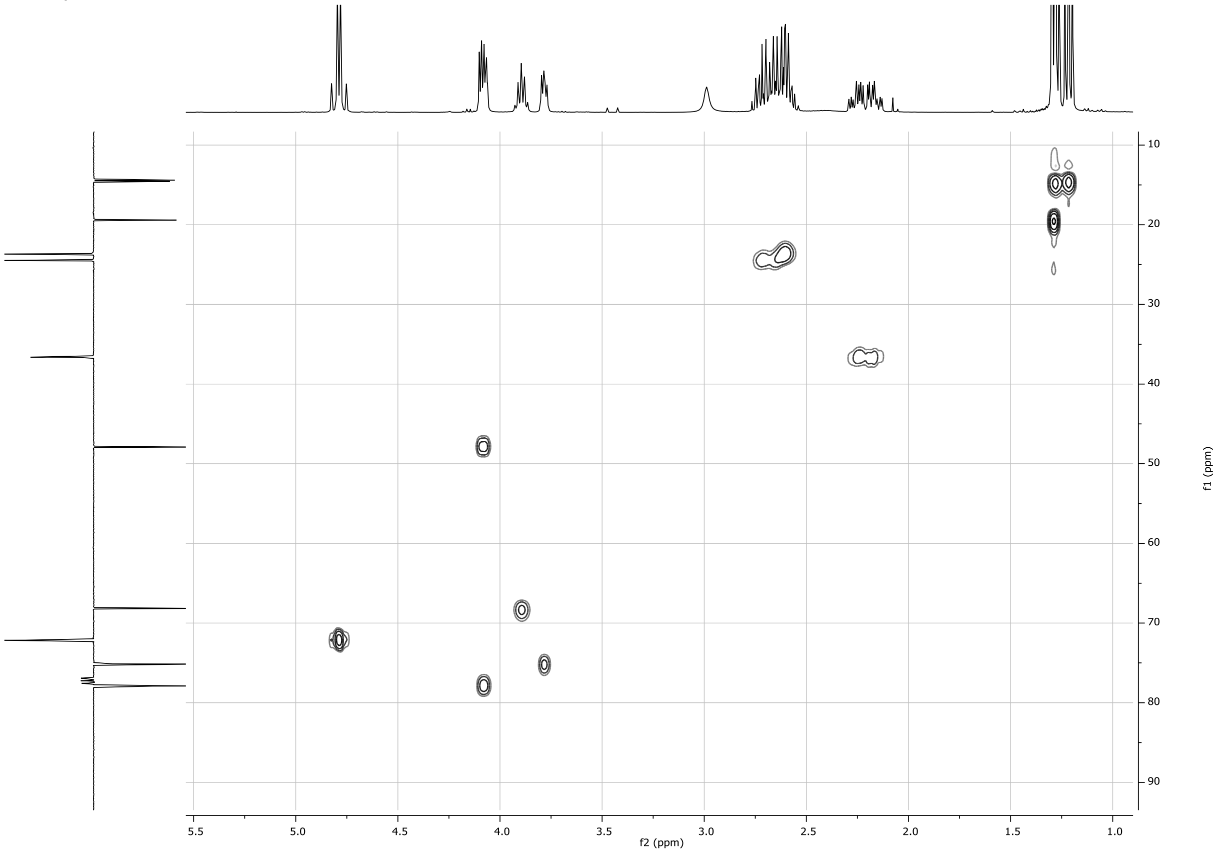

^{1}H NMR, 400 MHz, $CDCl_3$ of **14**

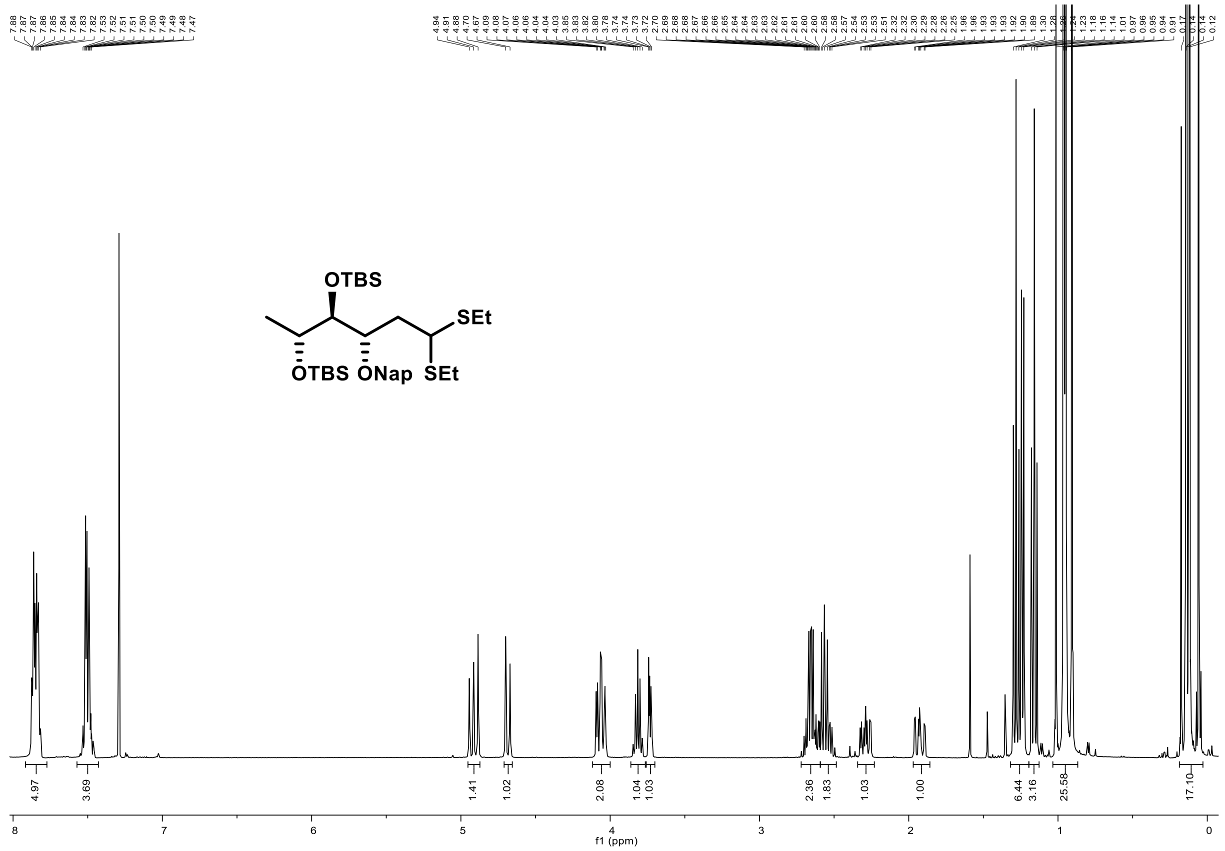

^{13}C NMR, 101 MHz, $CDCl_3$ of **14**

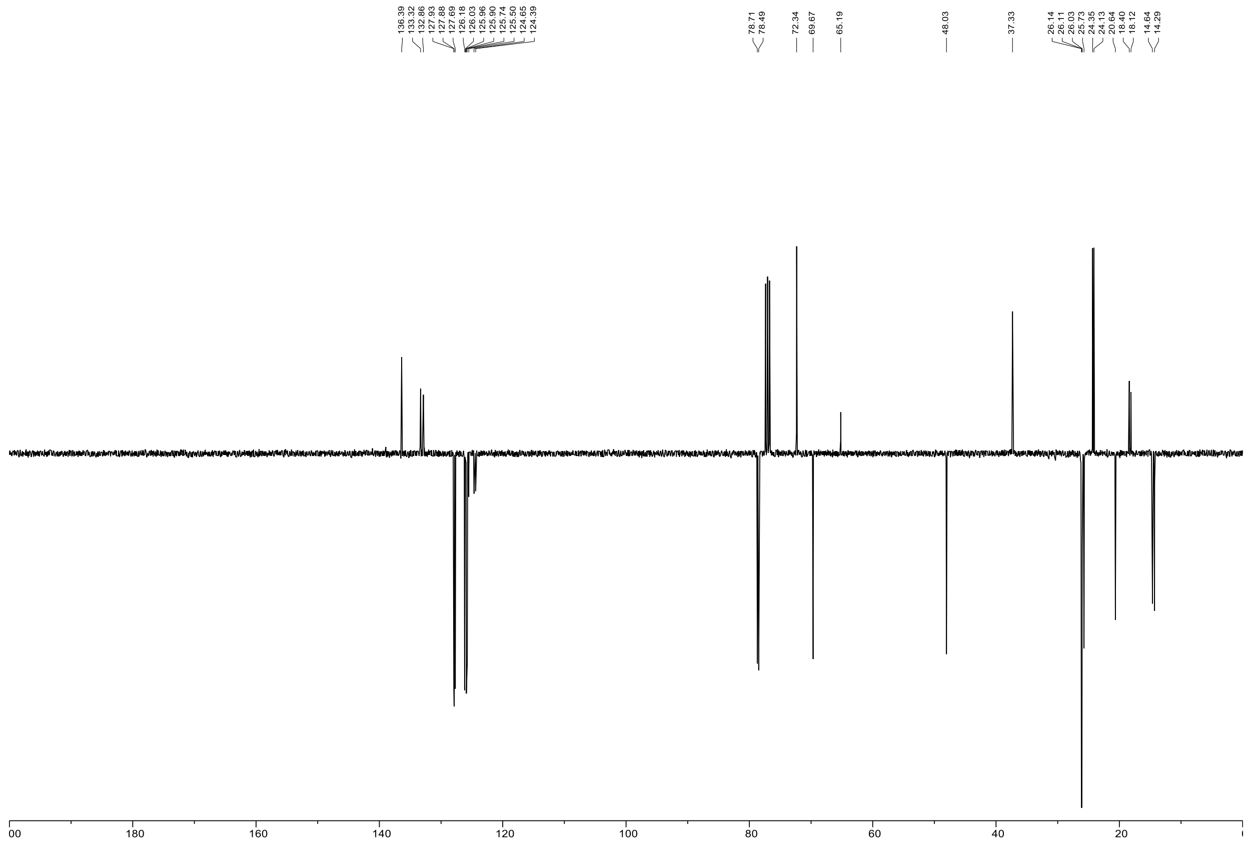

HH-COSY NMR, $CDCl_3$ of **14**

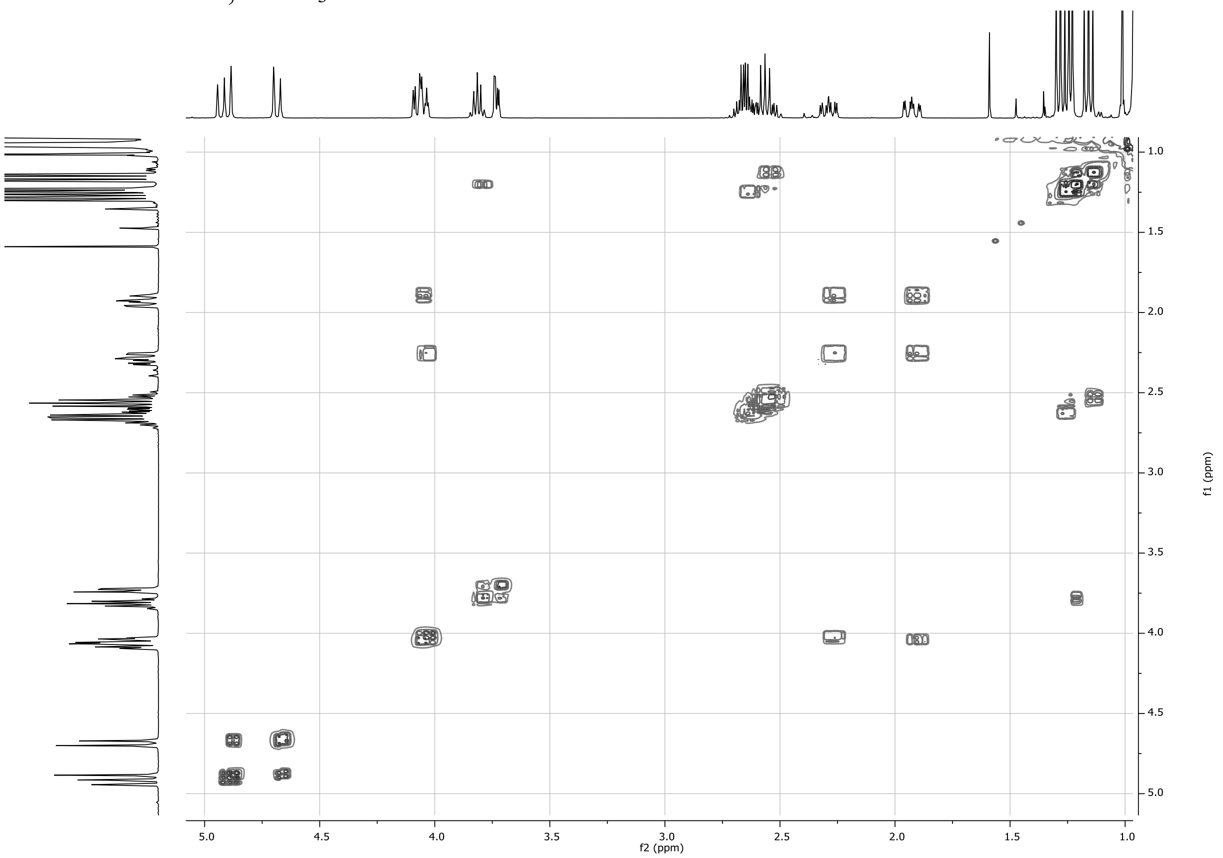

HSQC NMR, $CDCl_3$ of **14**

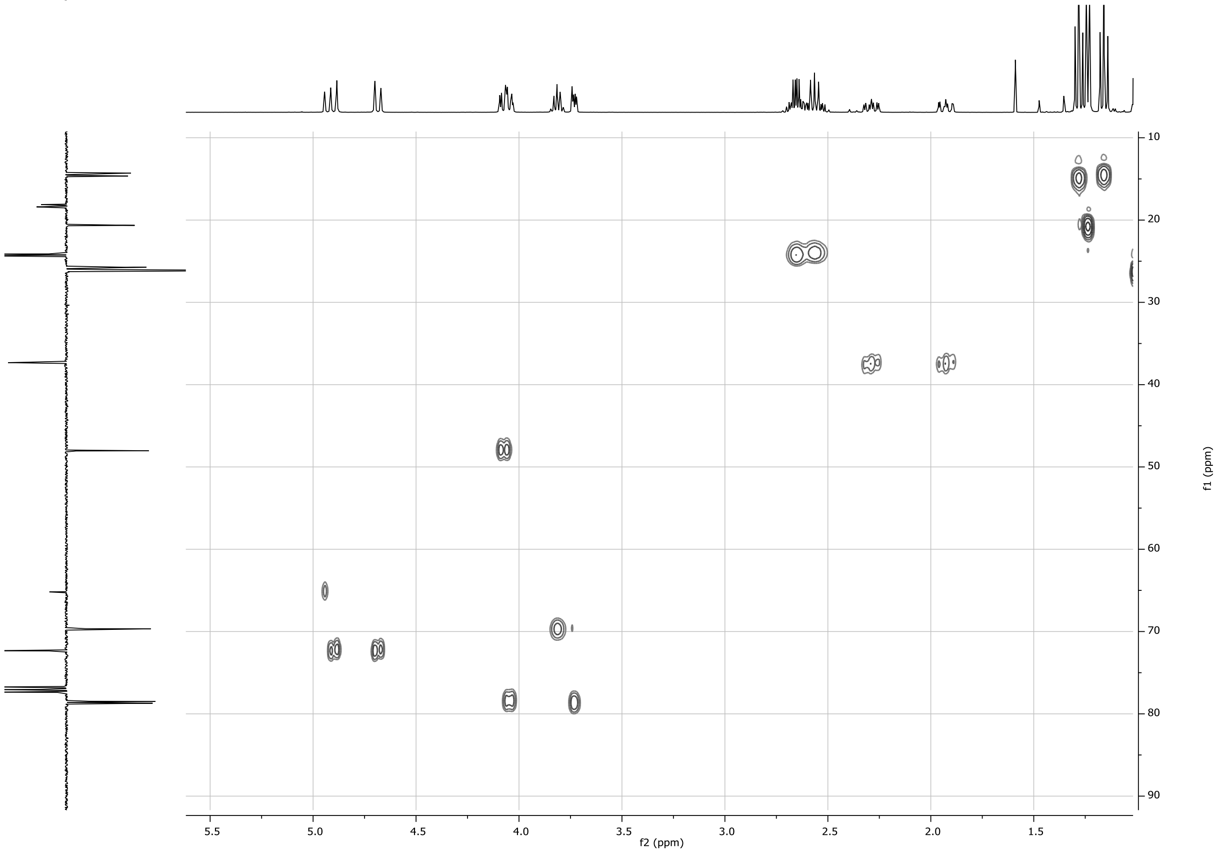

^{1}H NMR, 400 MHz, $CDCl_3$ of **15**

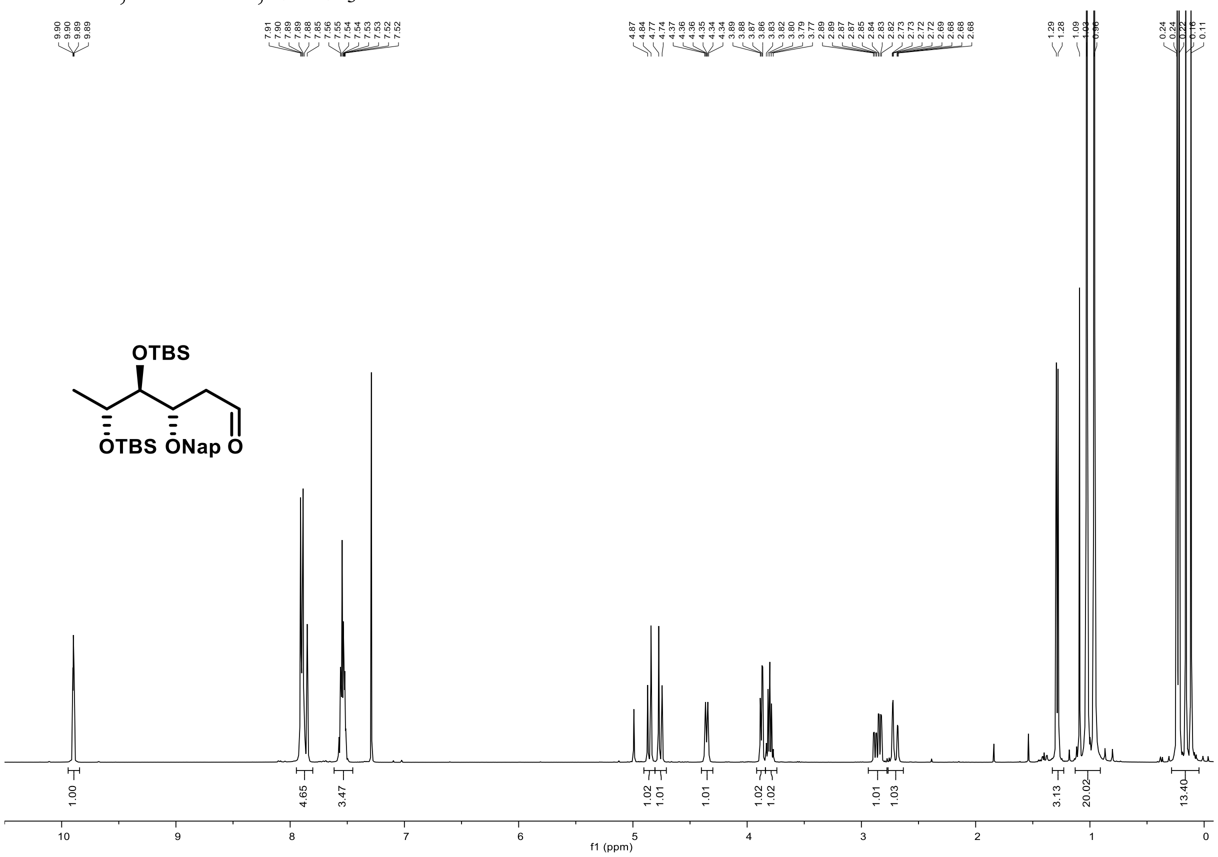

^{13}C NMR, 101 MHz, $CDCl_3$ of **15**

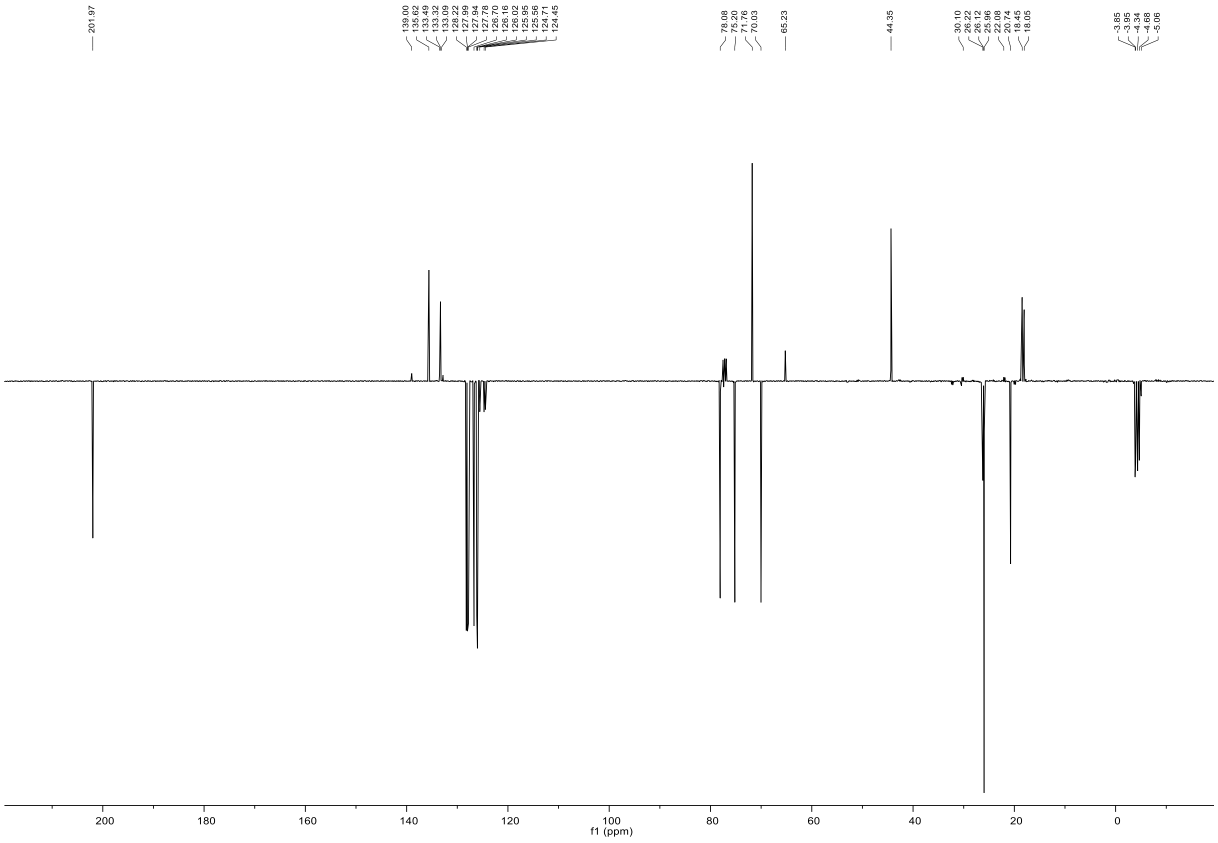

HH-COSY NMR, $CDCl_3$ of **15**

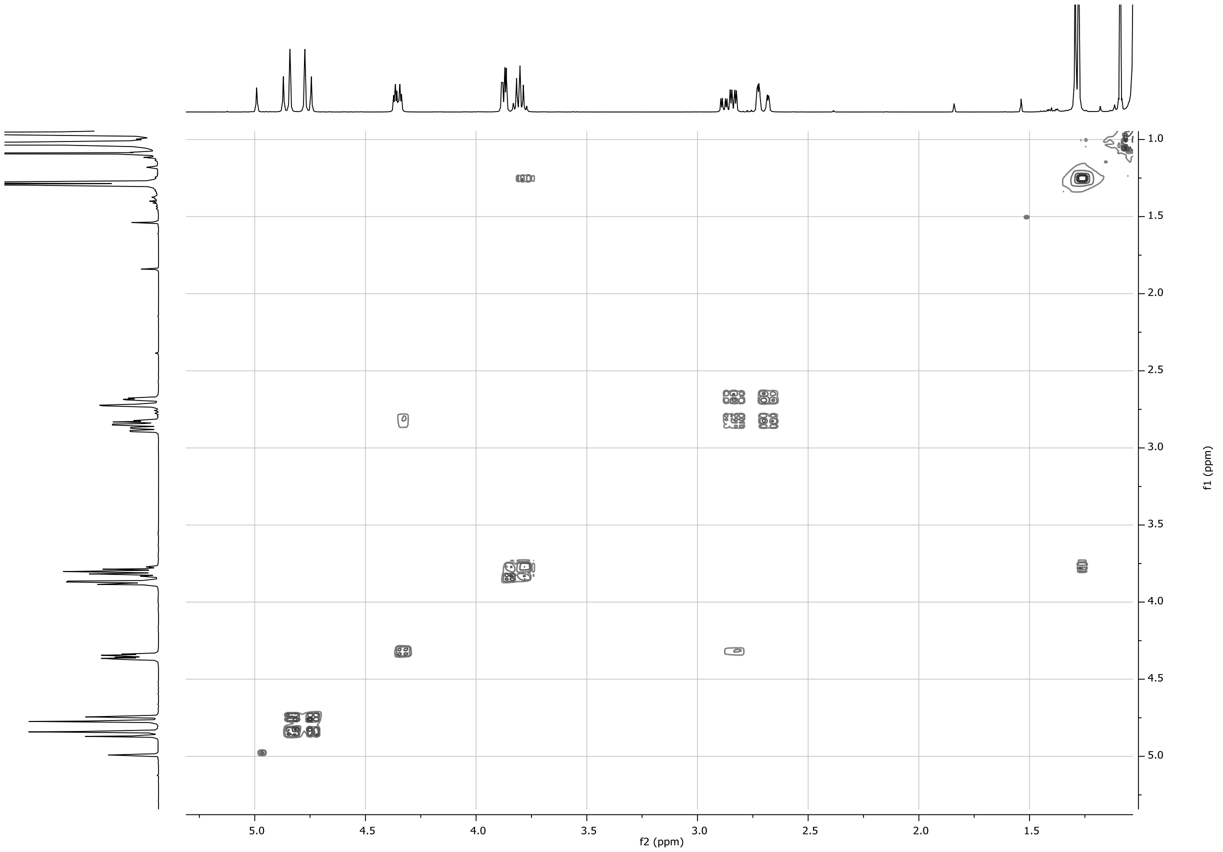

HSQC NMR, $CDCl_3$ of **15**

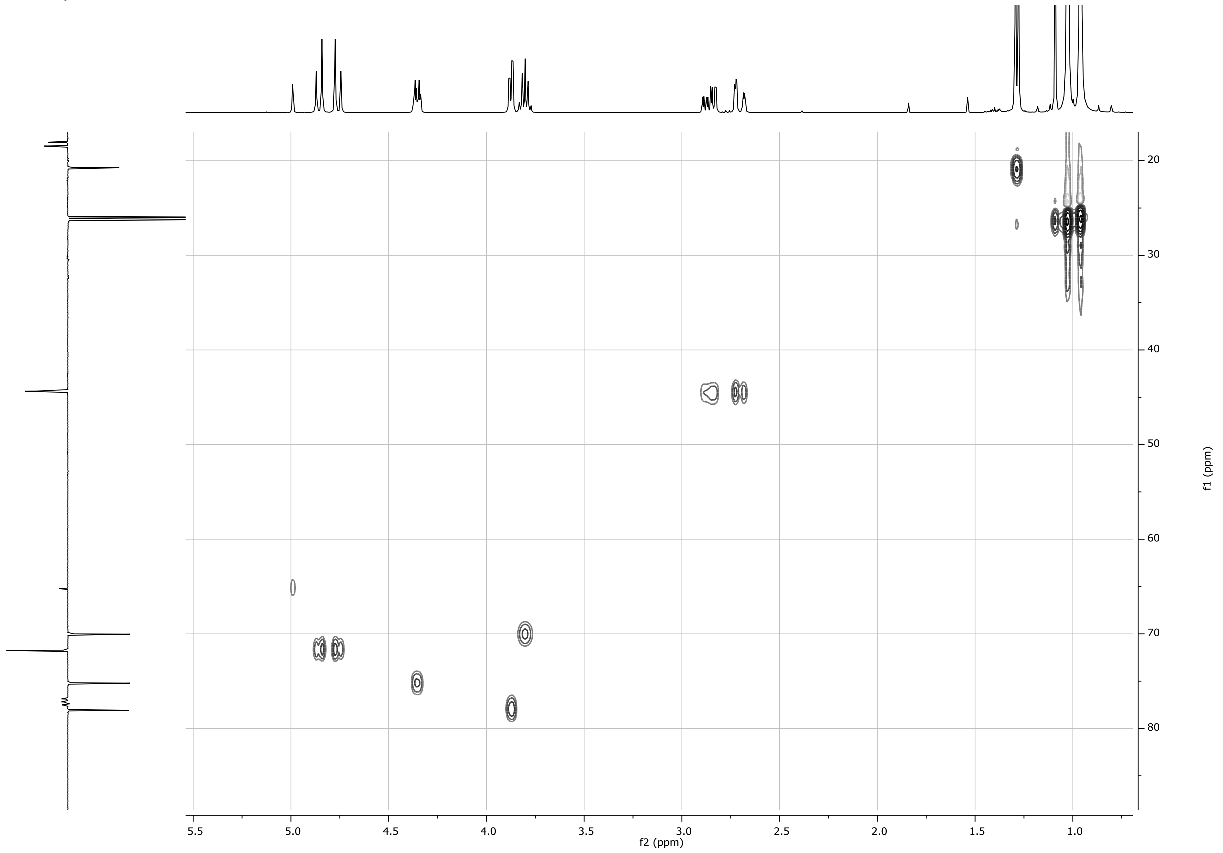

^{1}H NMR, 400 MHz, $CDCl_3$ of **16**

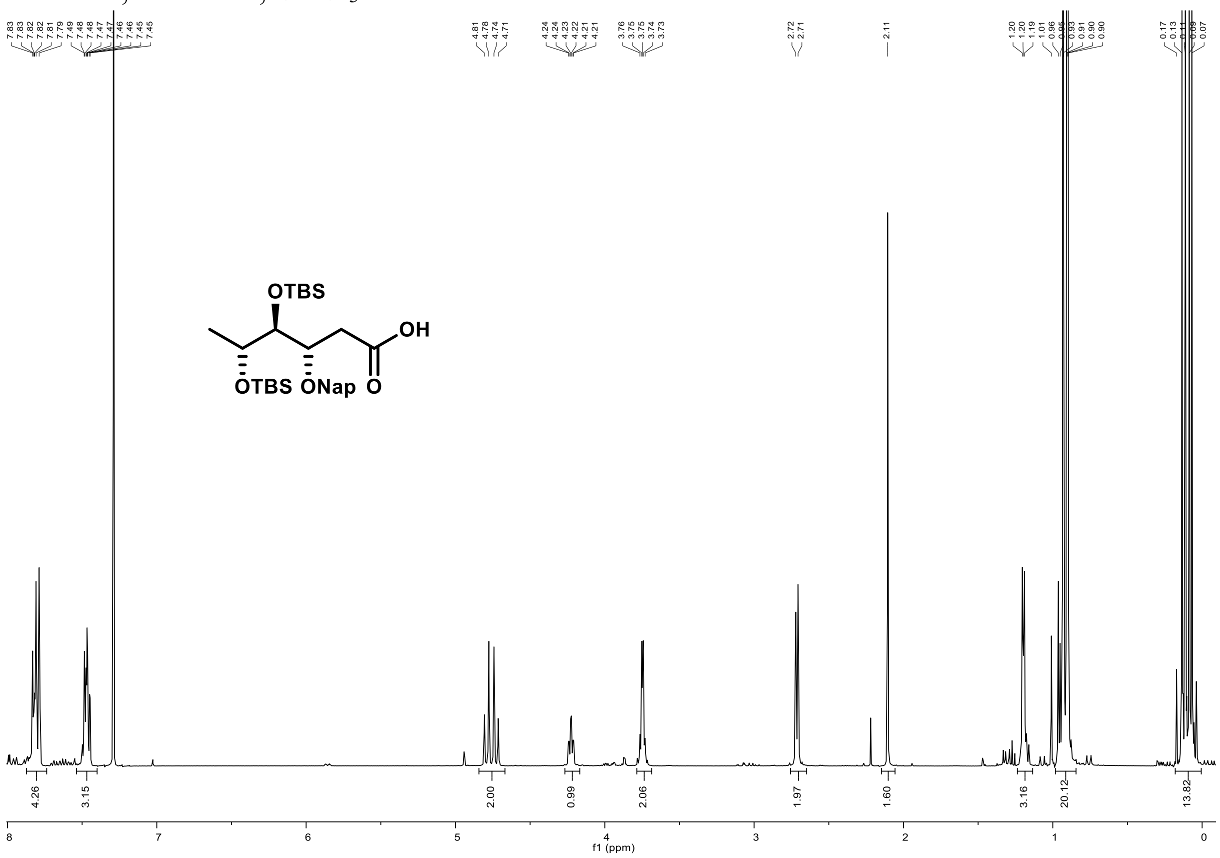

^{13}C NMR, 101 MHz, $CDCl_3$ of **16**

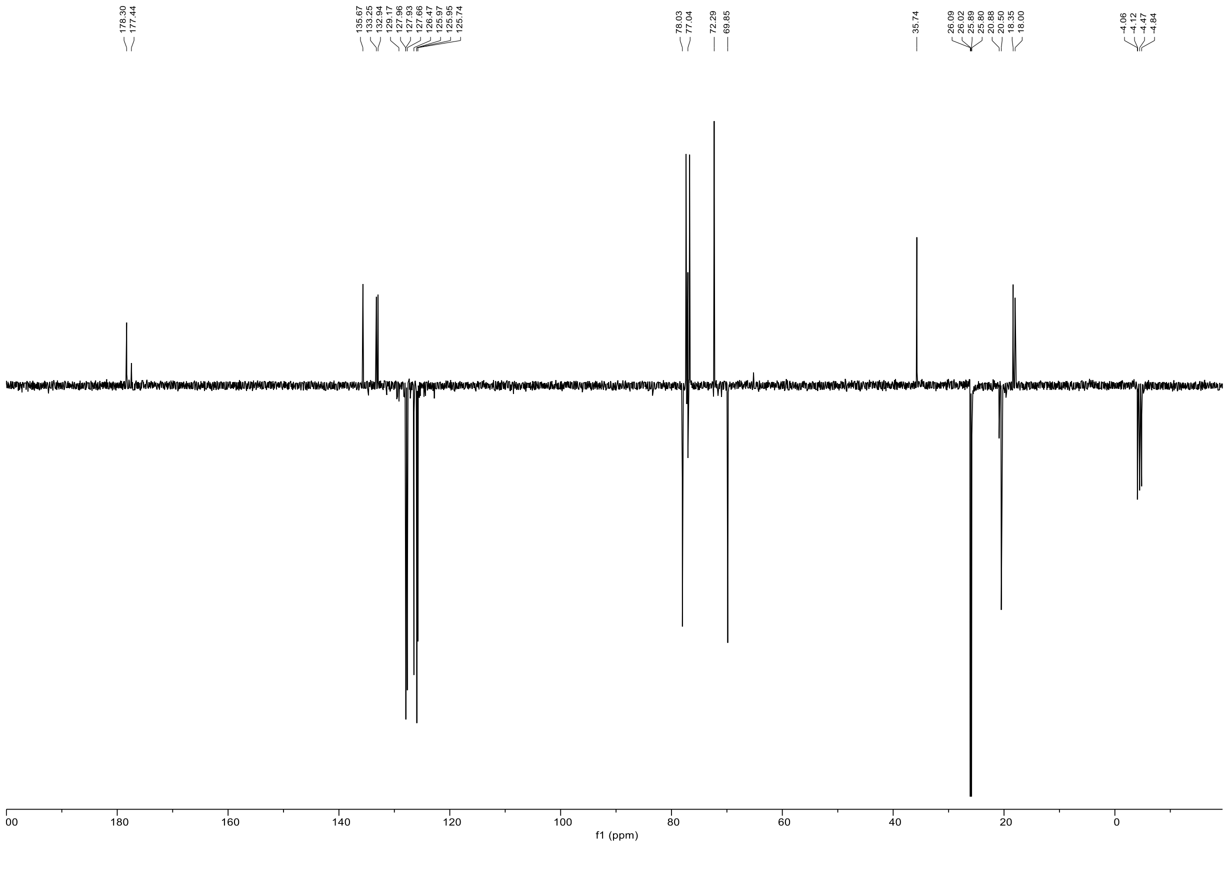

HH-COSY NMR, $CDCl_3$ of **16**

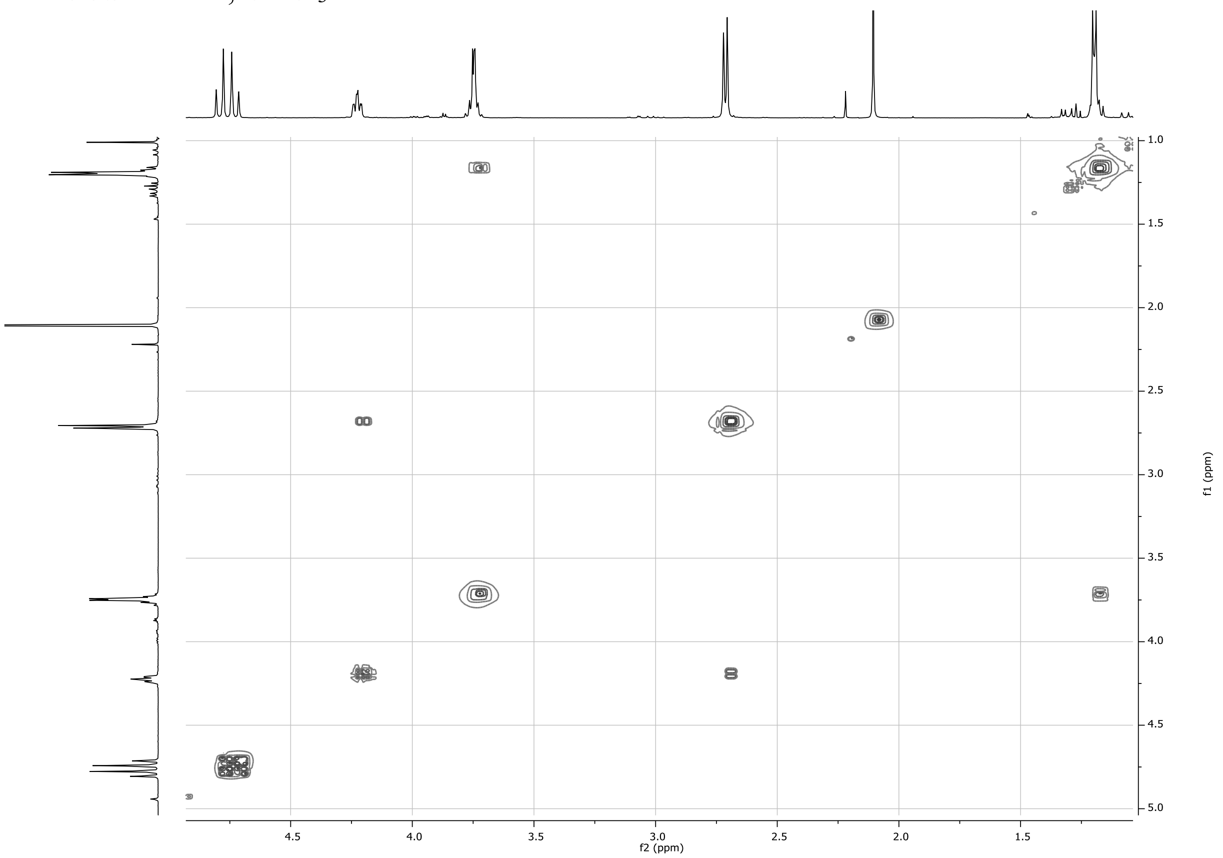

HSQC NMR, $CDCl_3$ of **16**

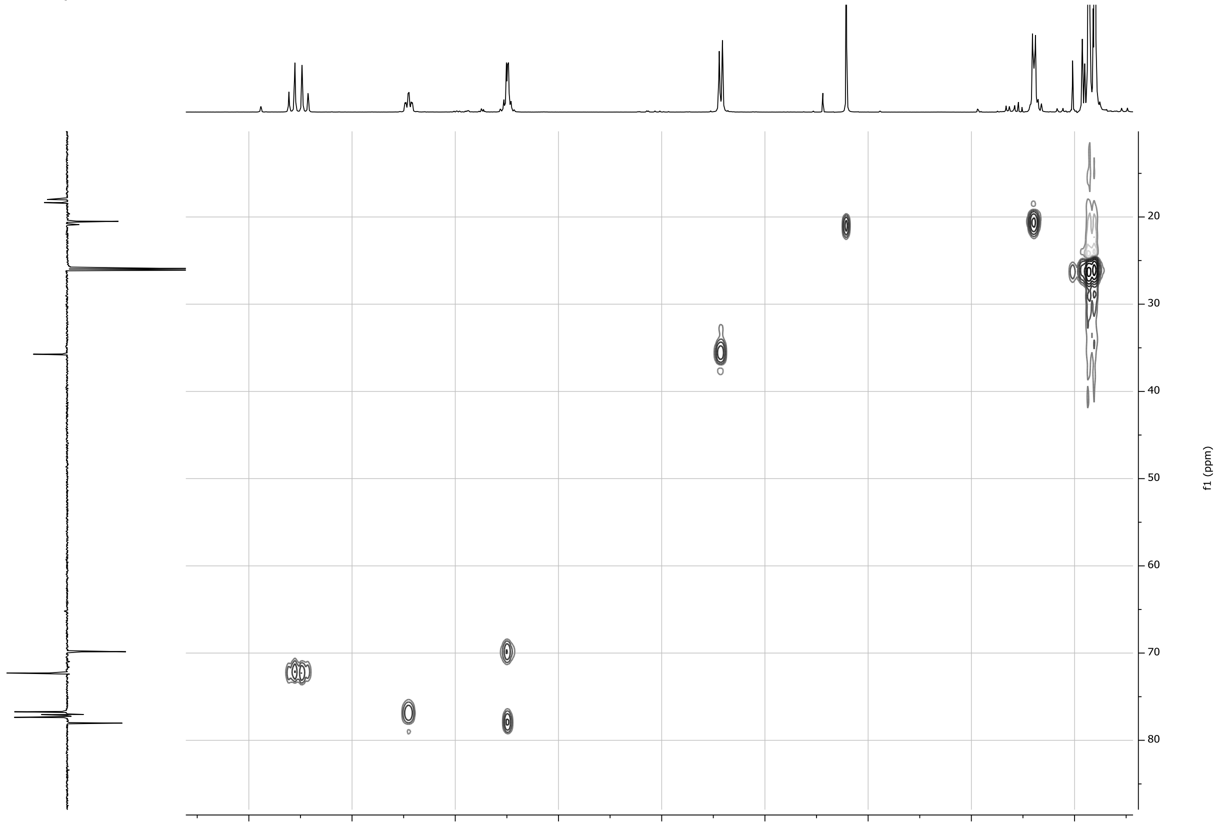

^{1}H NMR, 400 MHz, $CDCl_3$ of **17**

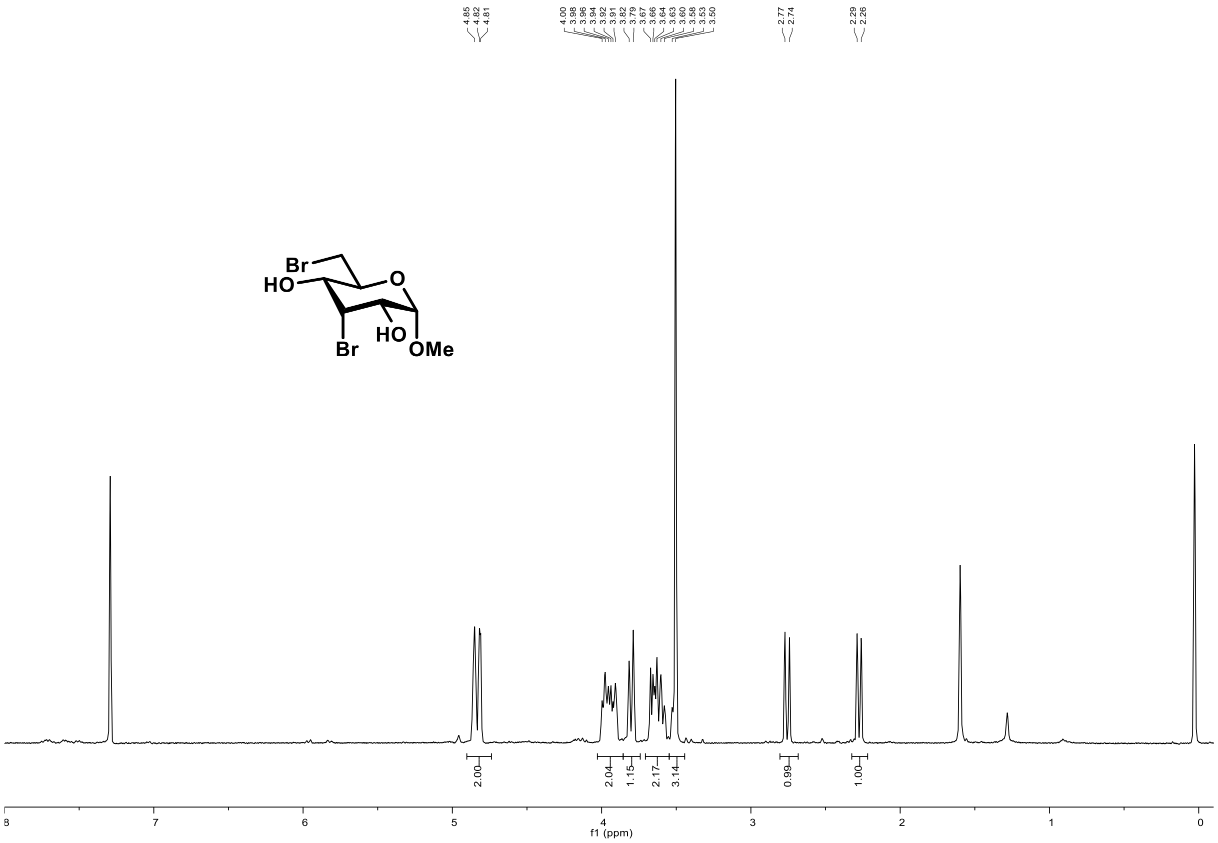

^{13}C NMR, 101 MHz, $CDCl_3$ of **17**

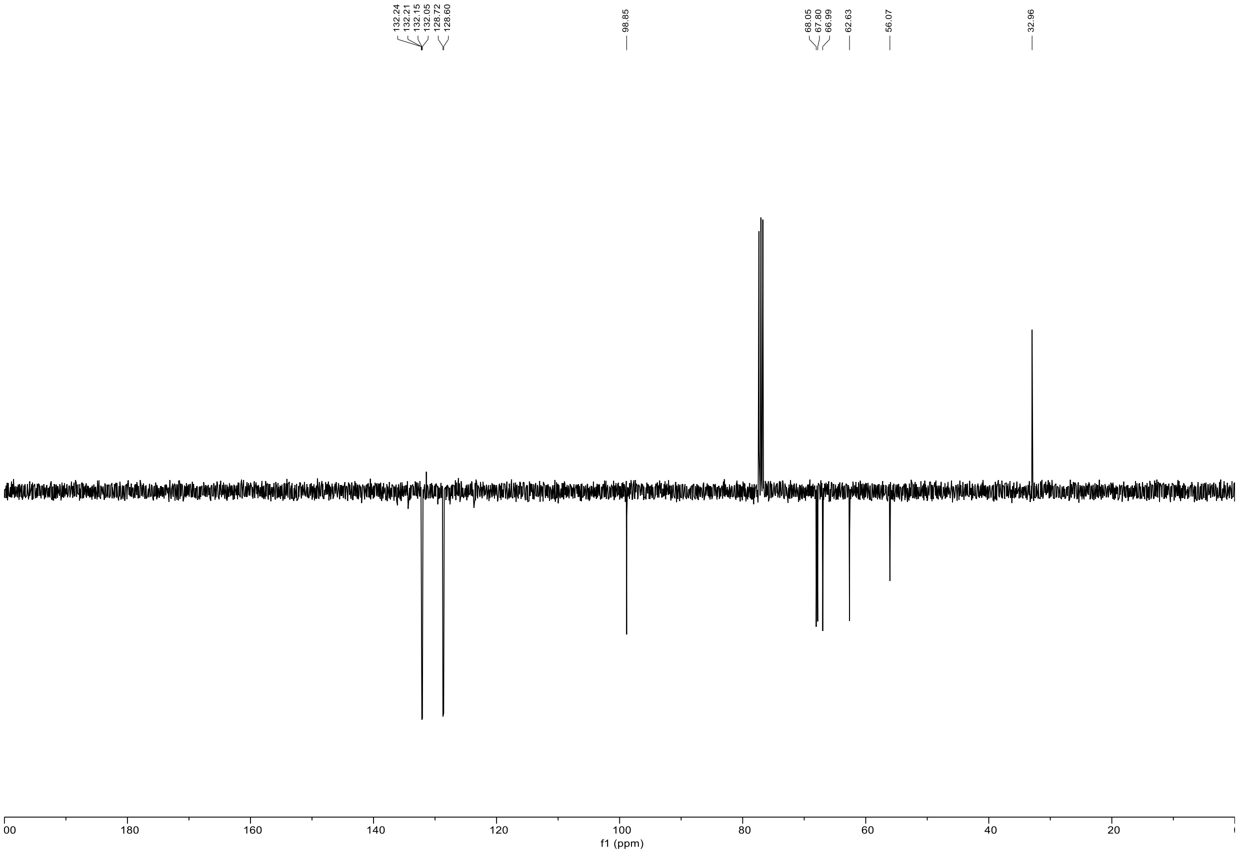

HH-COSY NMR, $CDCl_3$ of **17**

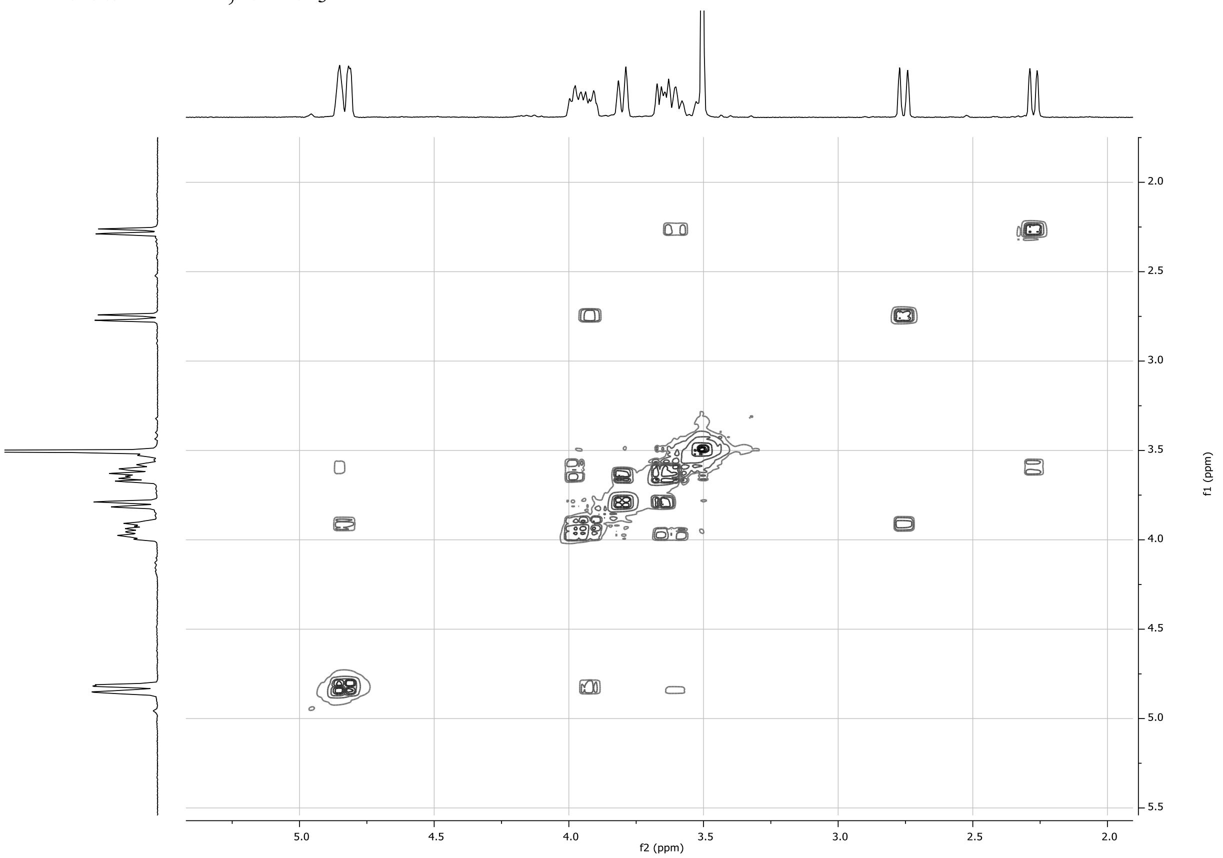

HSQC NMR, $CDCl_3$ of **17**

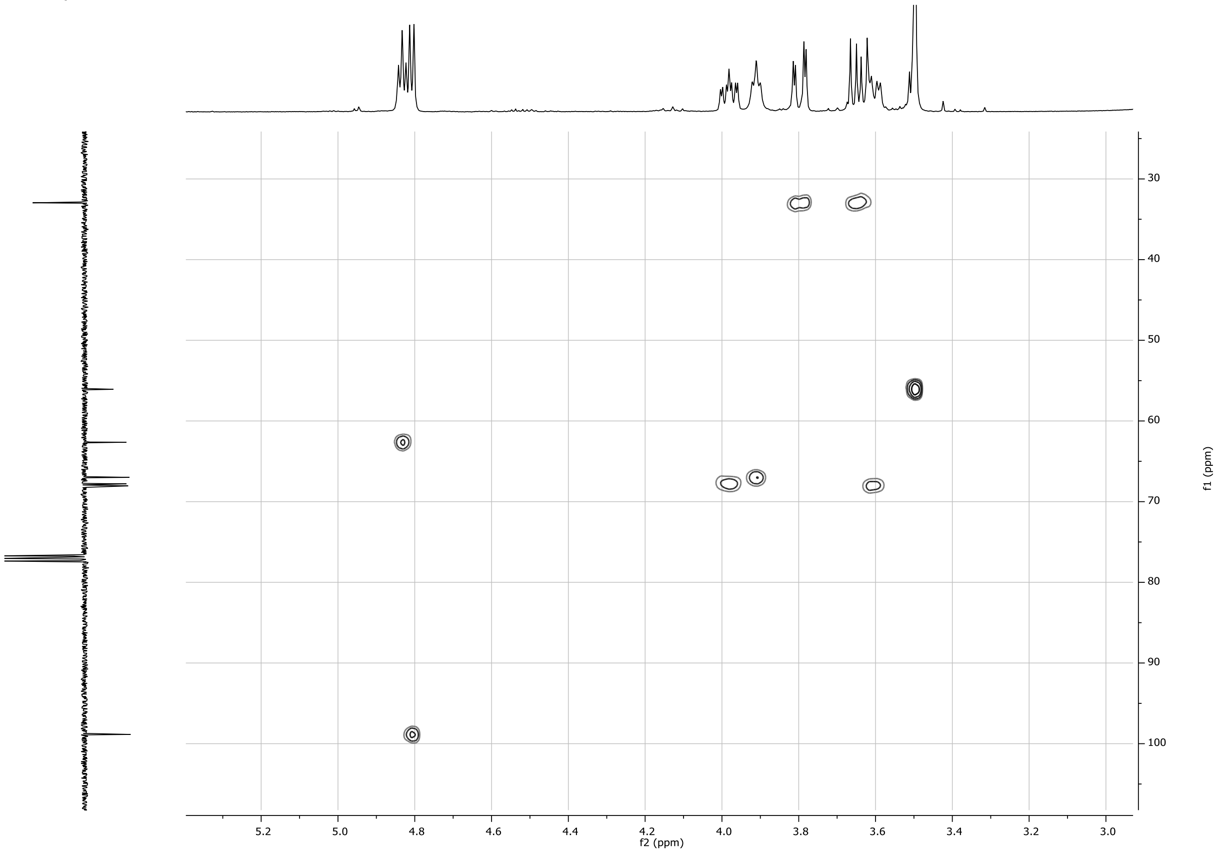

^{1}H NMR, 400 MHz, $CDCl_3$ of **18**

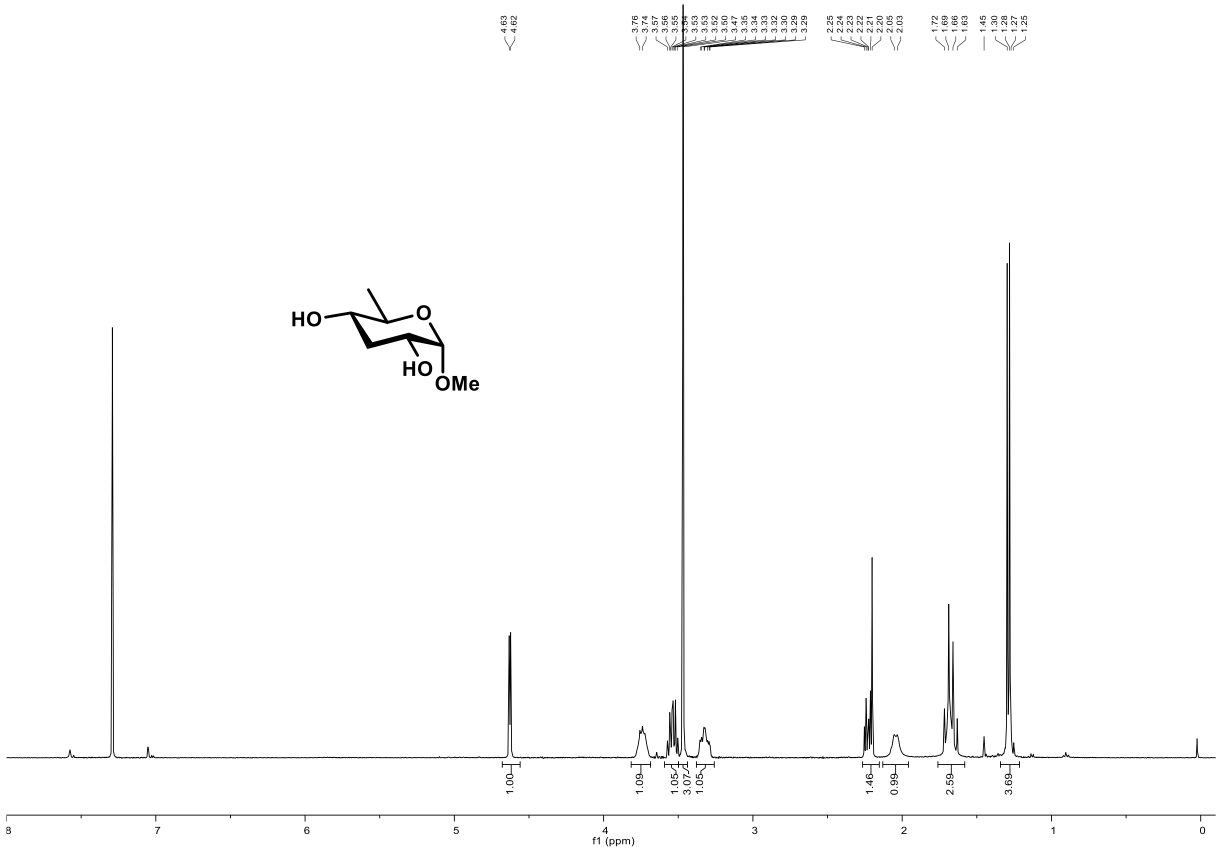

^{13}C NMR, 101 MHz, $CDCl_3$ of **18**

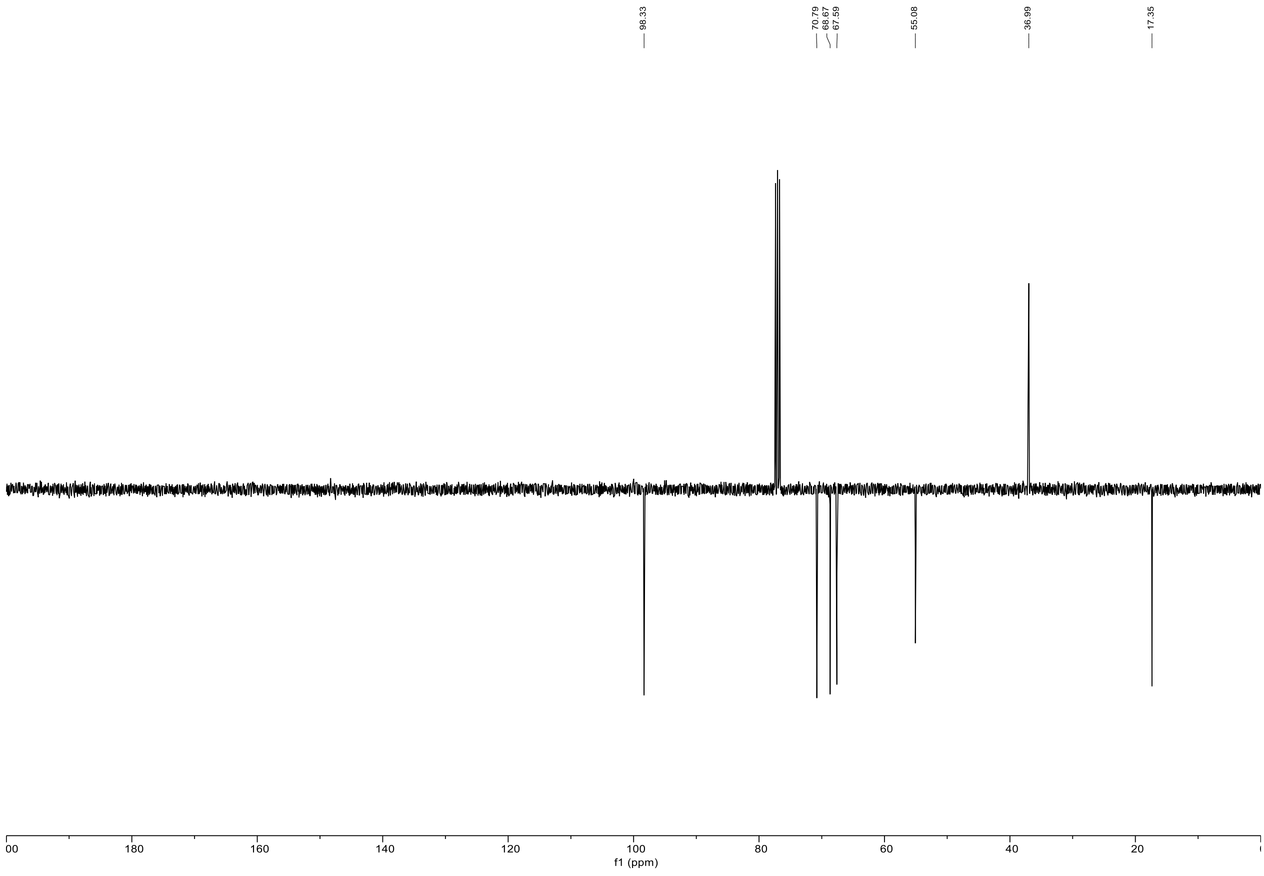

HH-COSY NMR, $CDCl_3$ of **18**

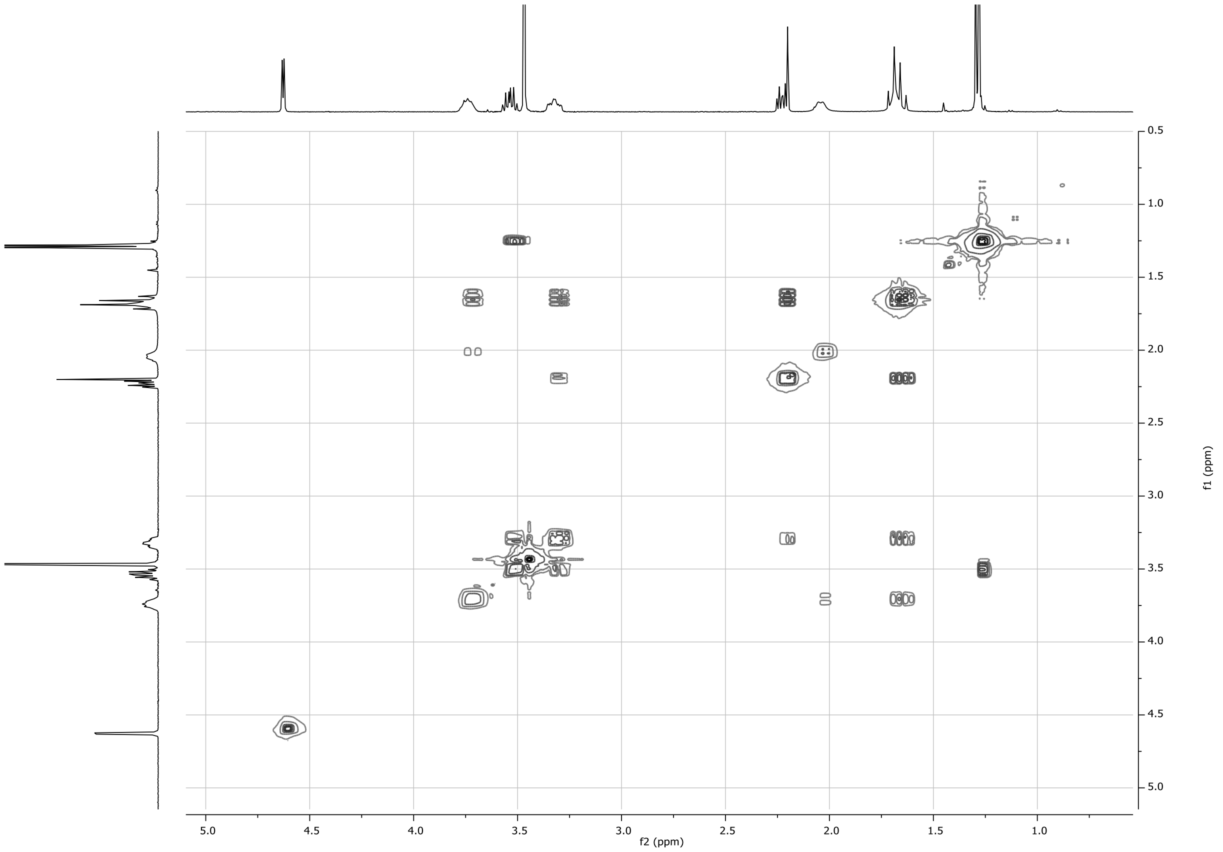

HSQC NMR, $CDCl_3$ of **18**

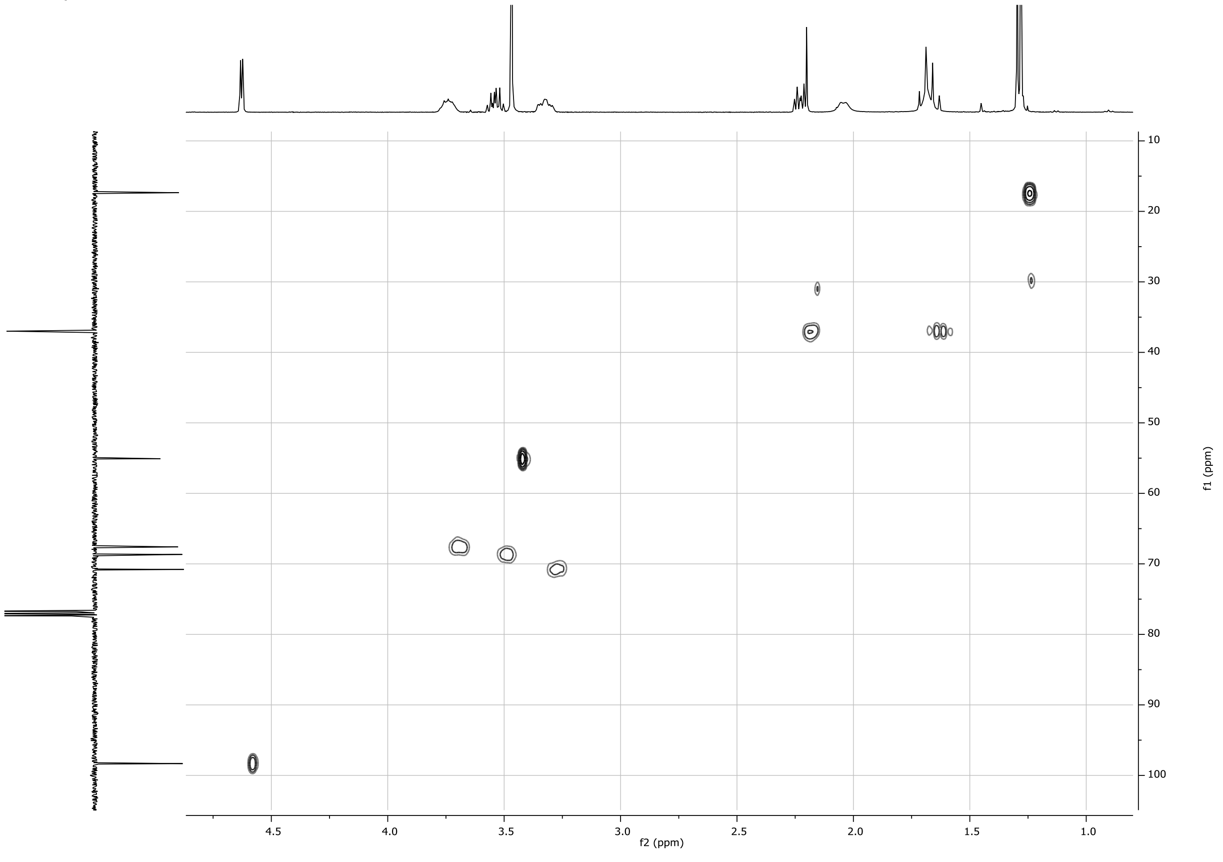

^{1}H NMR, 400 MHz, $CDCl_3$ of **19**

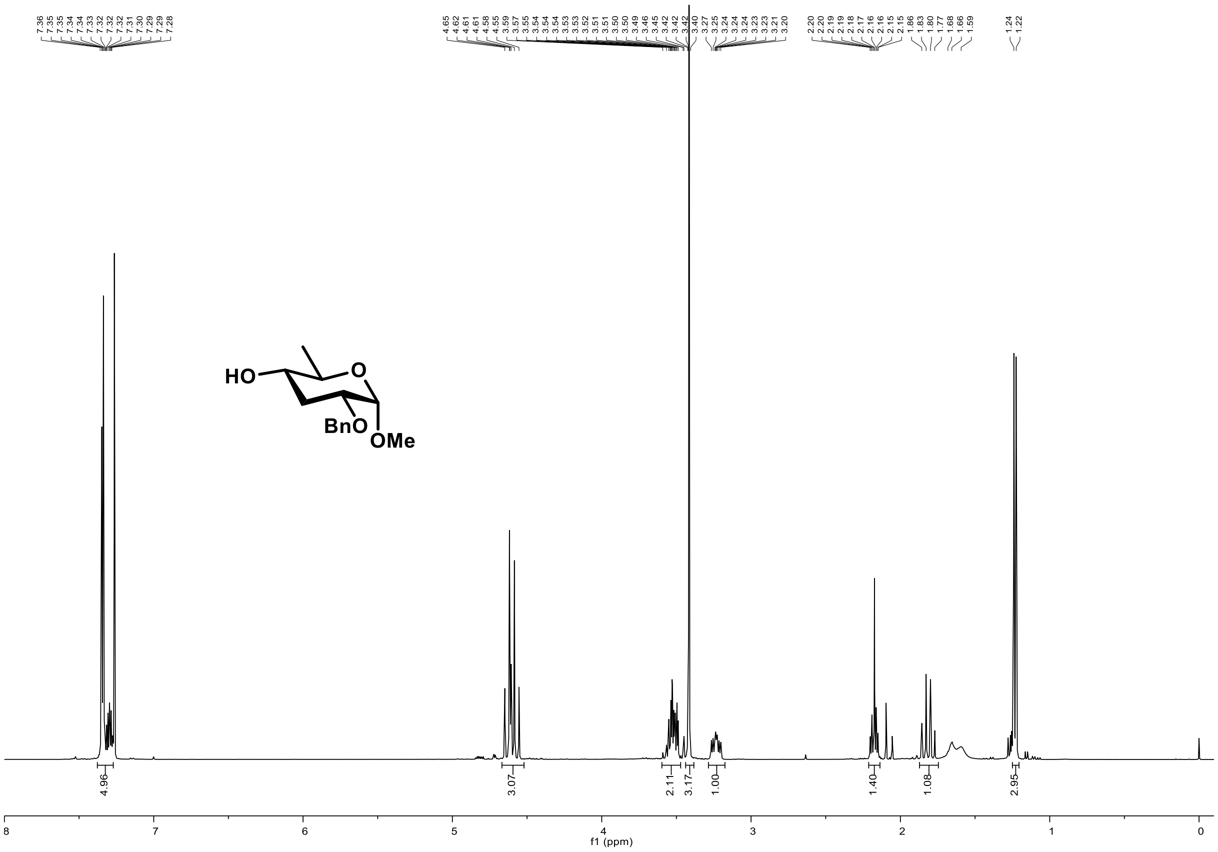

^{13}C NMR, 101 MHz, $CDCl_3$ of **19**

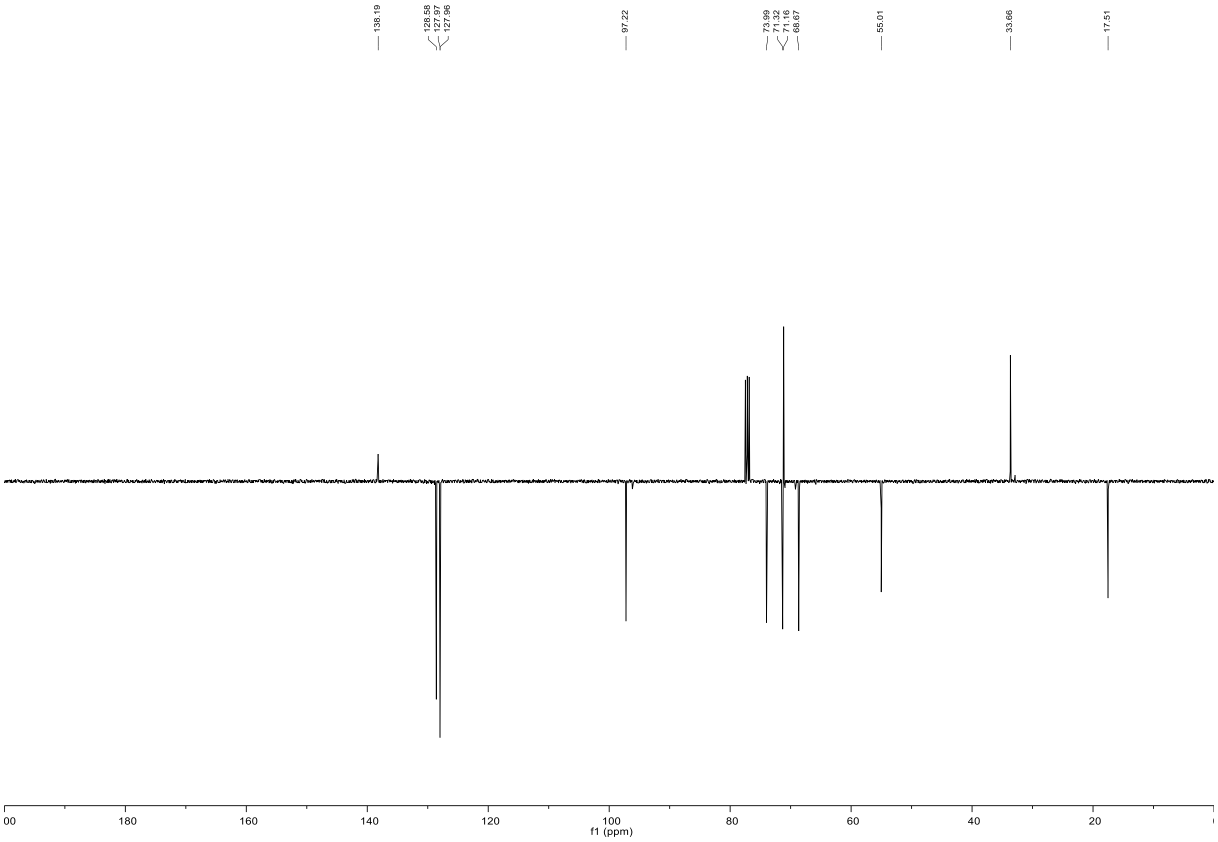

HH-COSY NMR, $CDCl_3$ of **19**

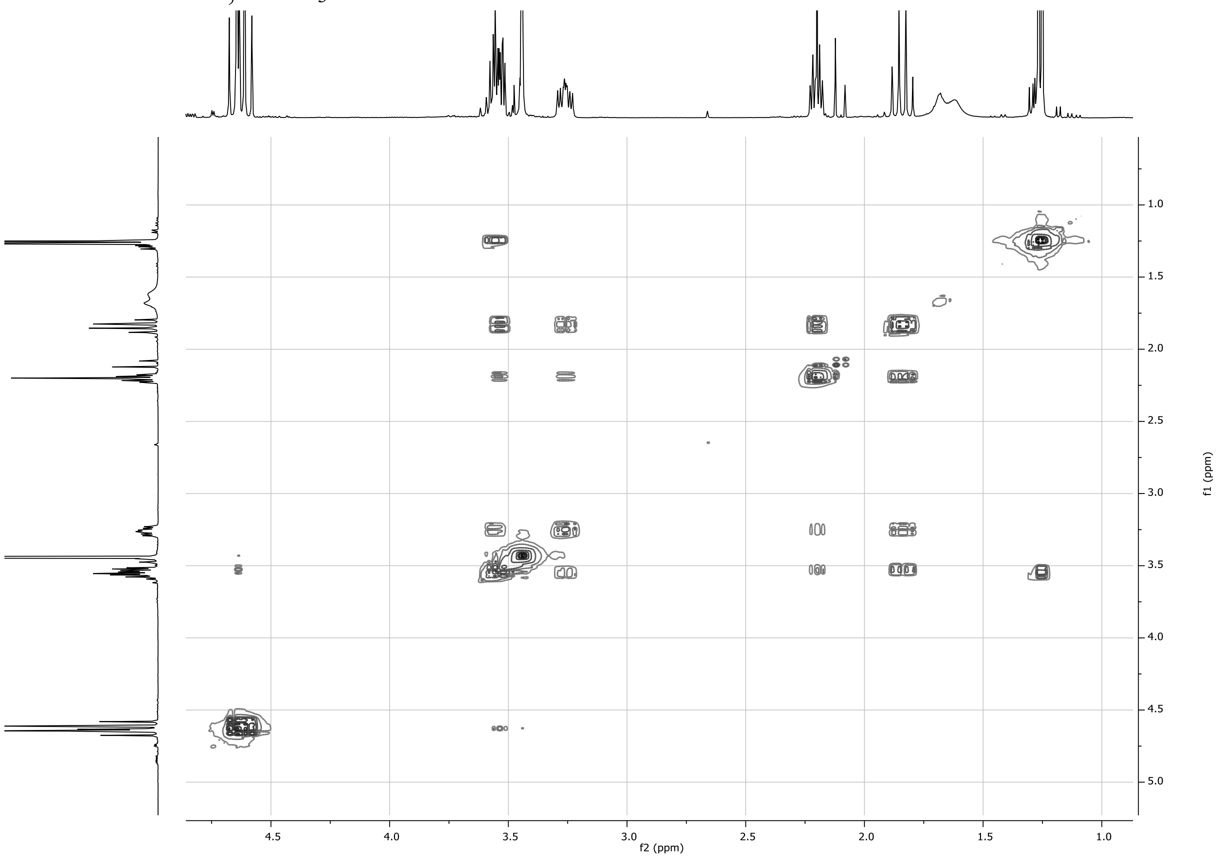

HSQC NMR, $CDCl_3$ of **19**

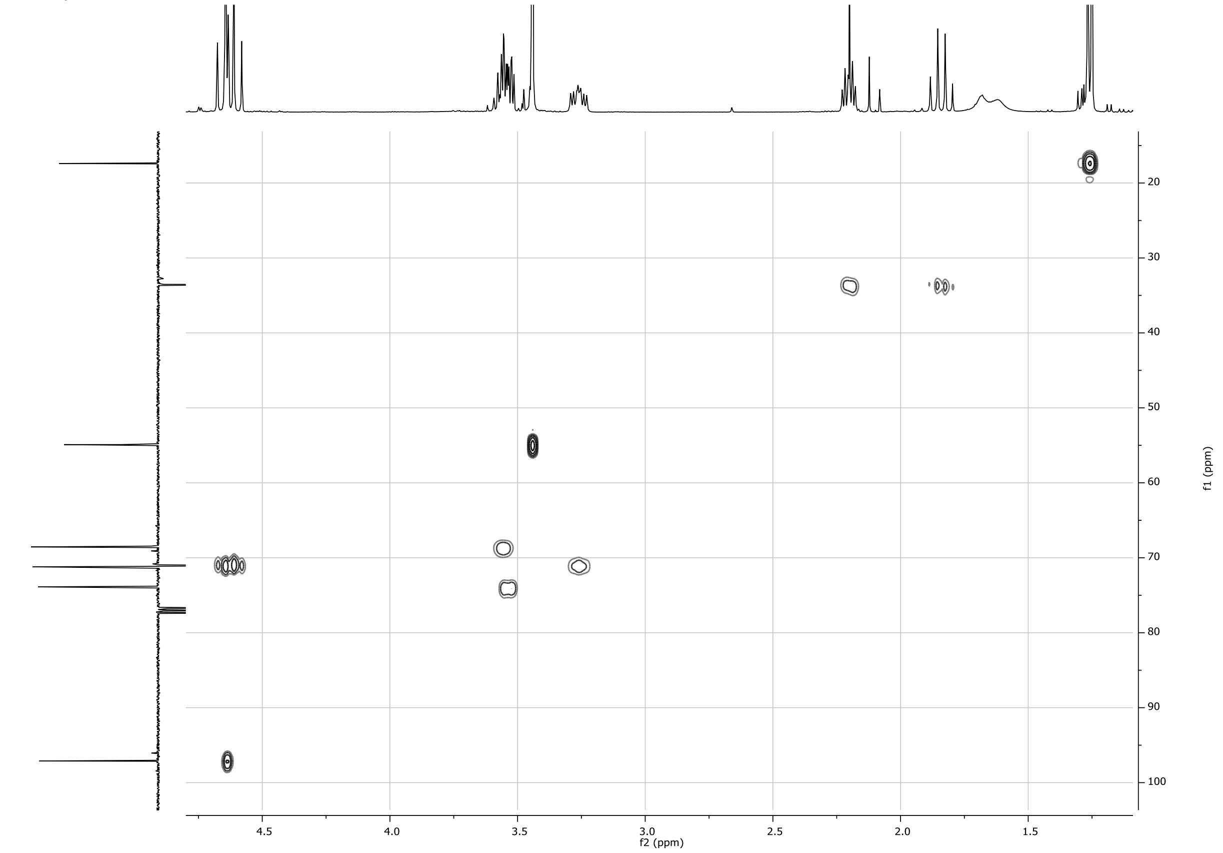

^{1}H NMR, 400 MHz, $CDCl_3$ of **6**

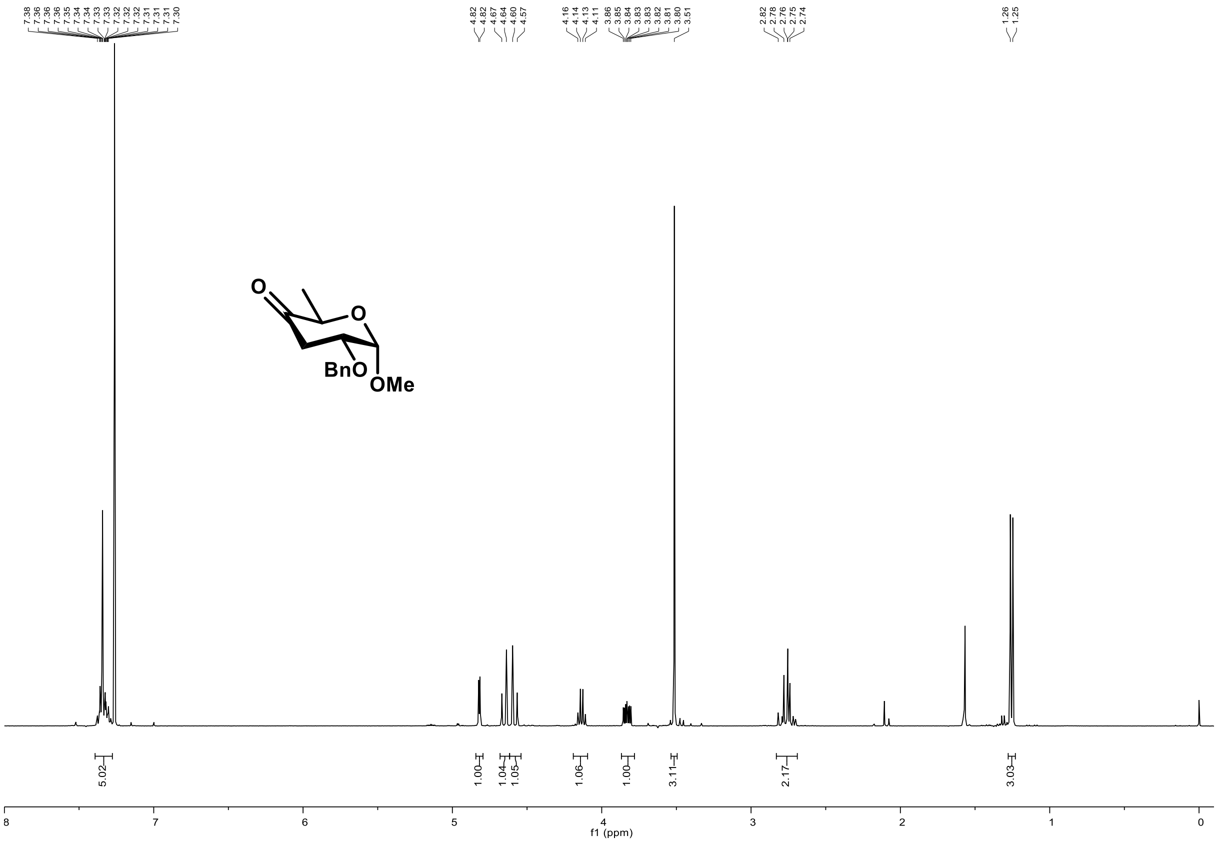

^{13}C NMR, 101 MHz, $CDCl_3$ of **6**

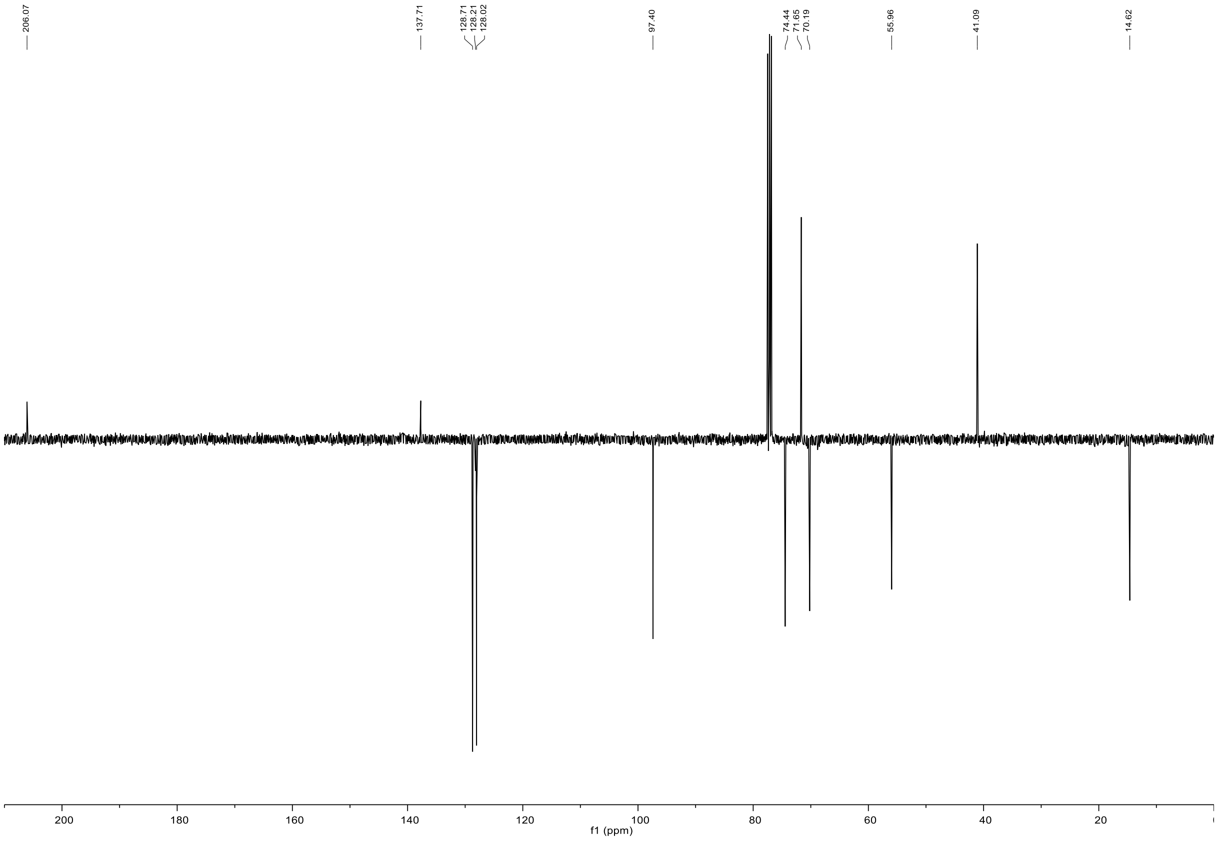

HH-COSY NMR, $CDCl_3$ of **6**

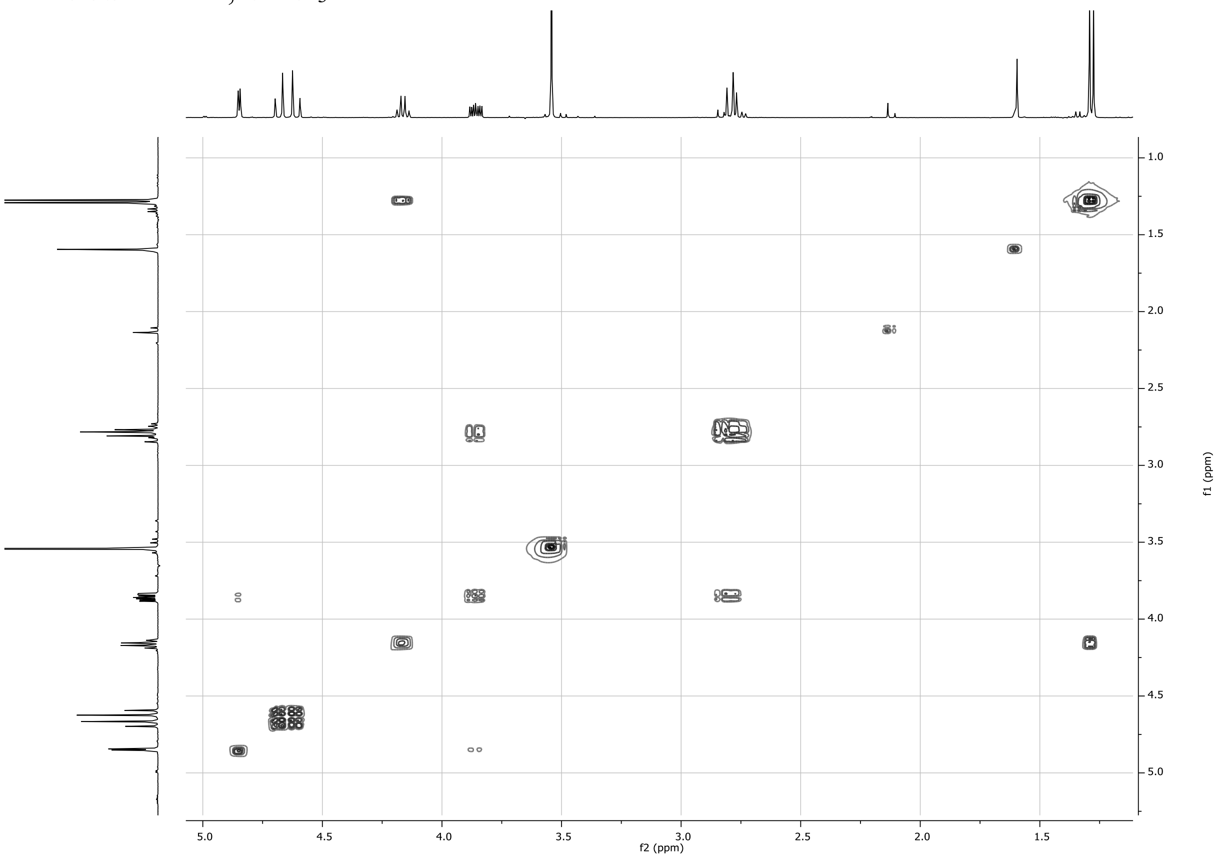

HSQC NMR, $CDCl_3$ of **6**

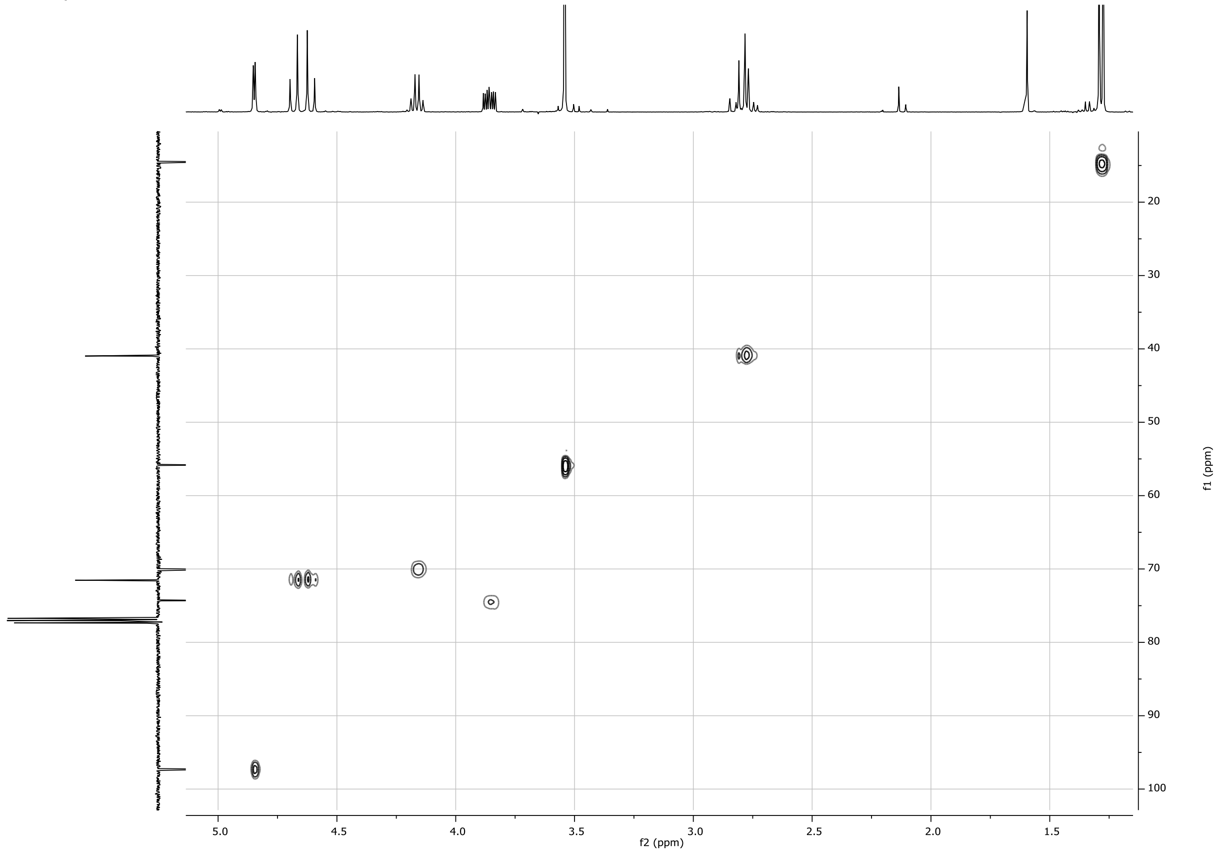

^{1}H NMR, 400 MHz, $CDCl_3$ of **20**

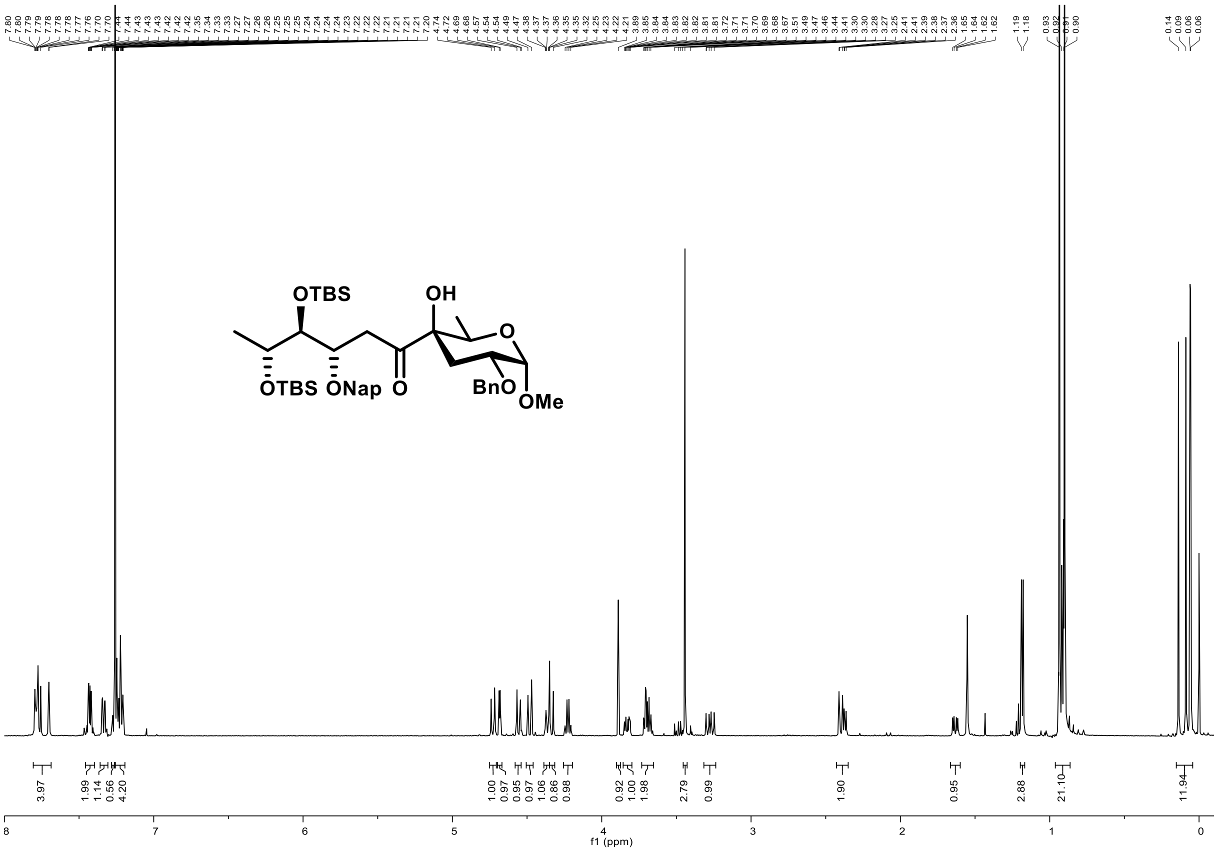

^{13}C NMR, 101 MHz, $CDCl_3$ of **20**

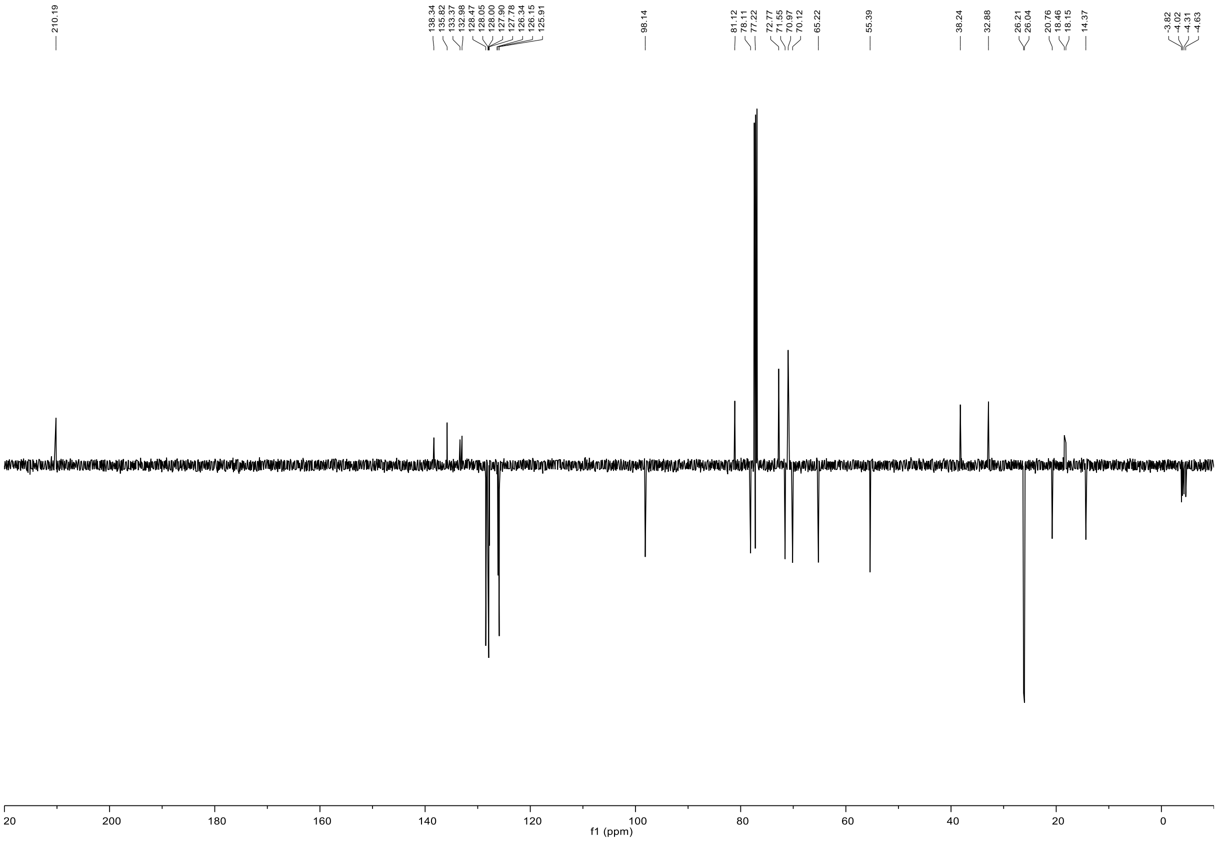

HH-COSY NMR, $CDCl_3$ of **20**

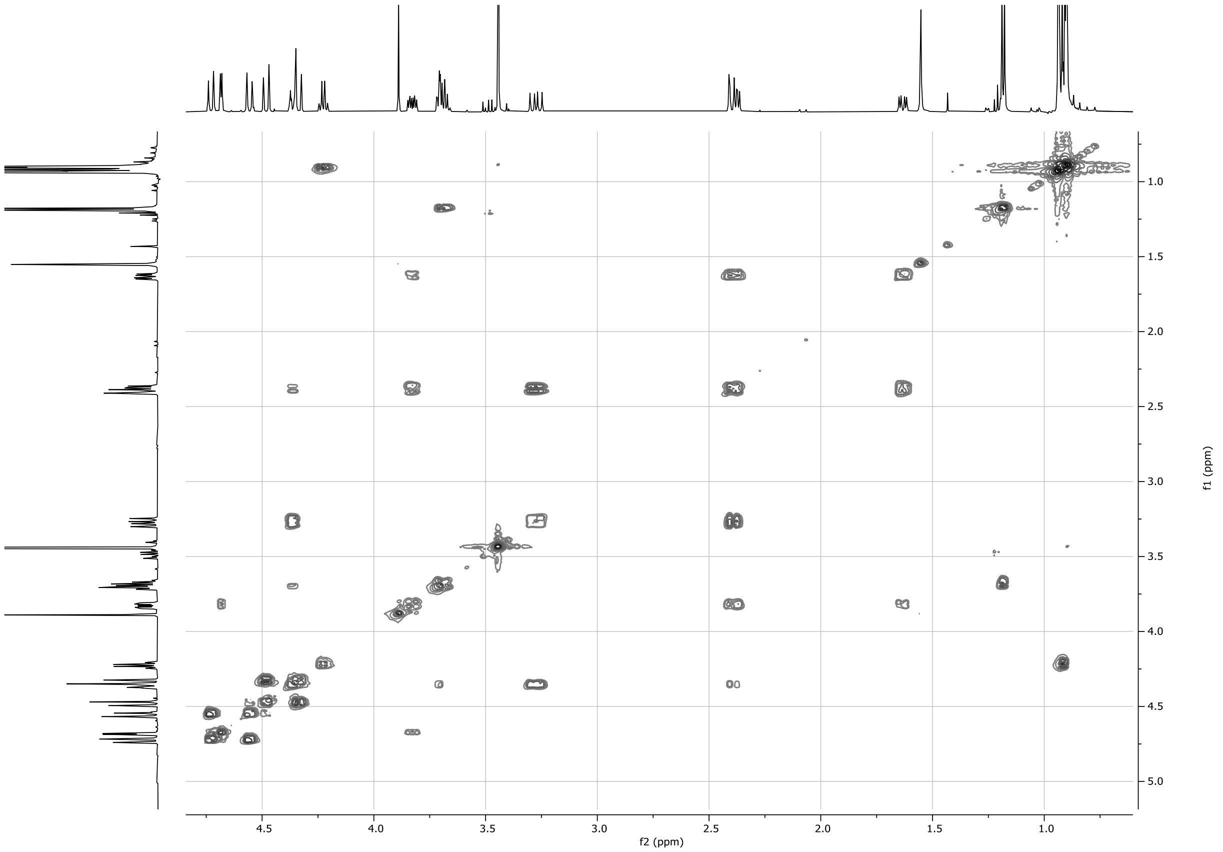

HSQC NMR, $CDCl_3$ of **20**

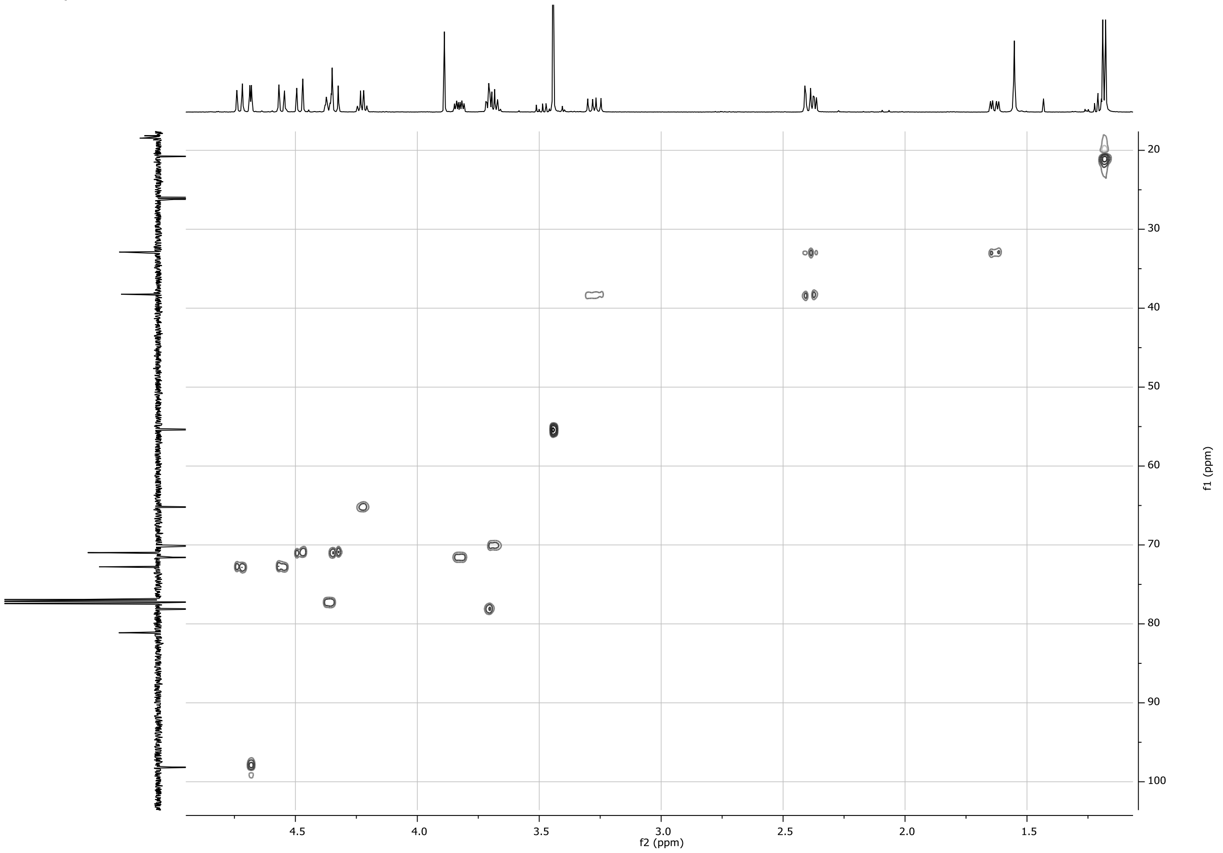

NOESY NMR, $CDCl_3$ of **20**

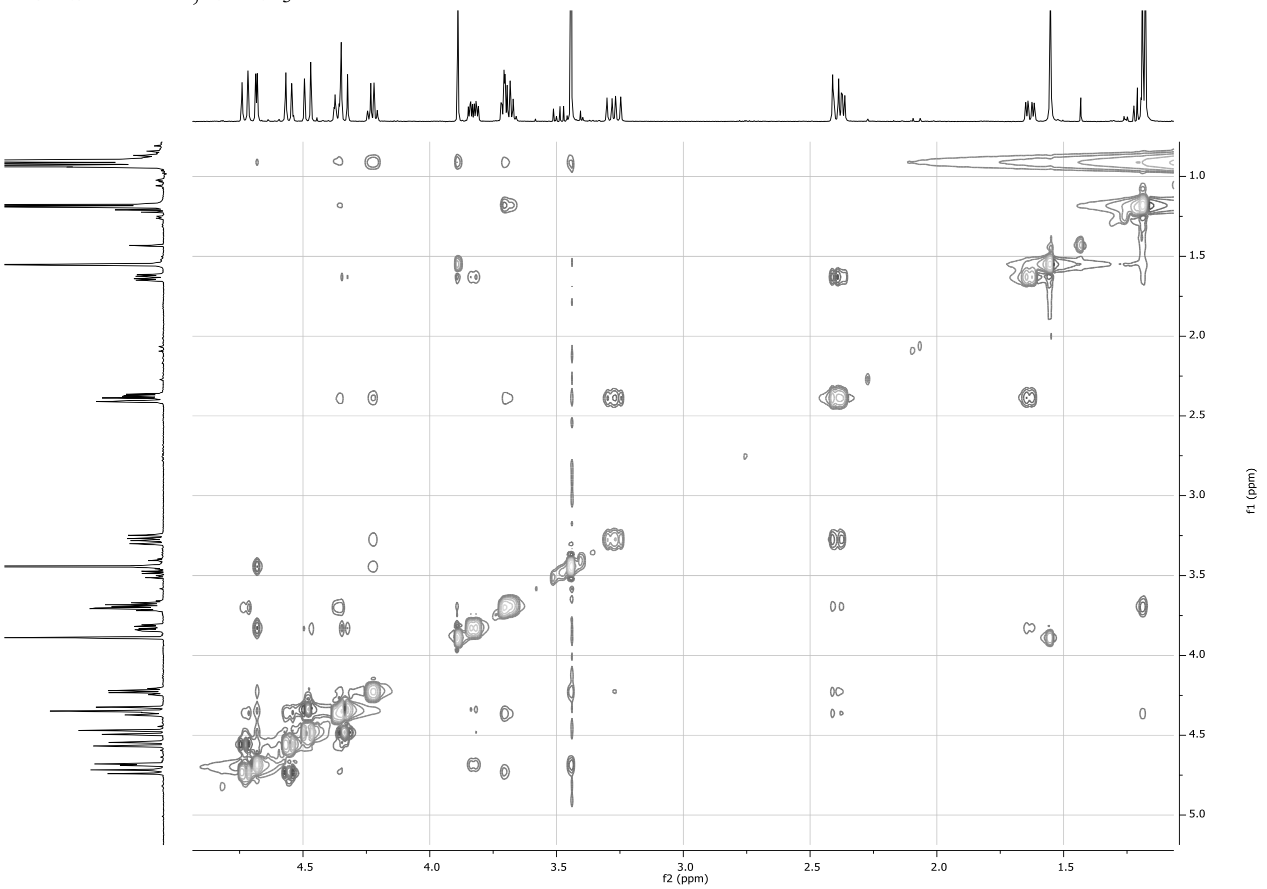

^{1}H NMR, 500 MHz, $CDCl_3$ of **21**

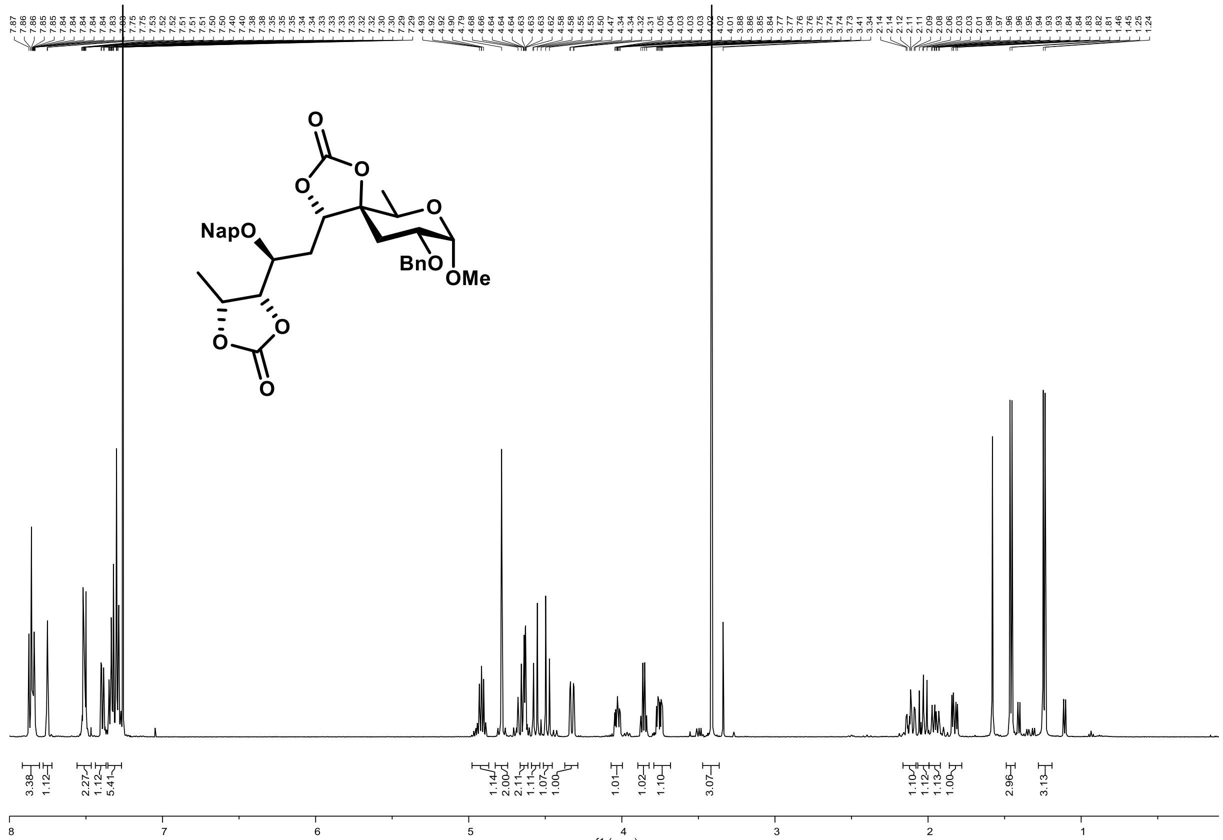

^{13}C NMR, 126 MHz, $CDCl_3$ of **21**

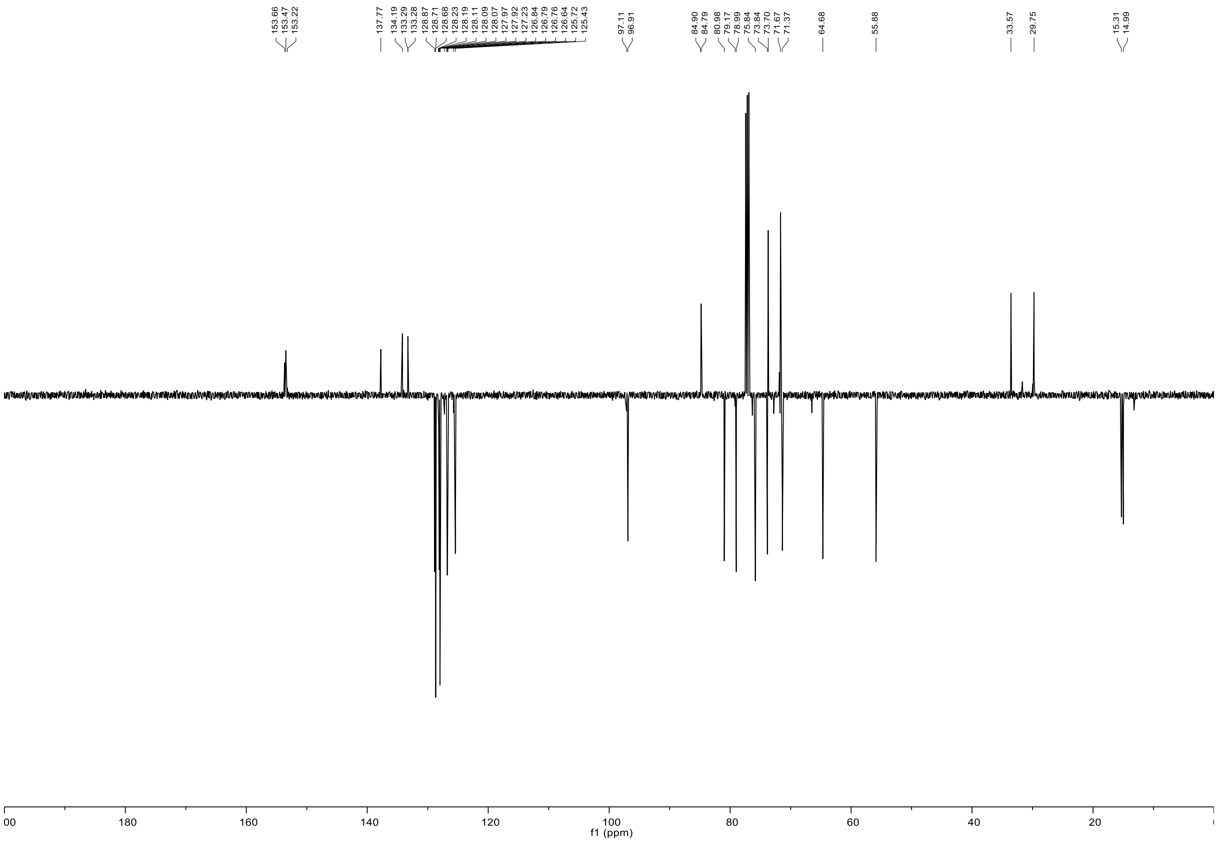

HH-COSY NMR, $CDCl_3$ of **21**

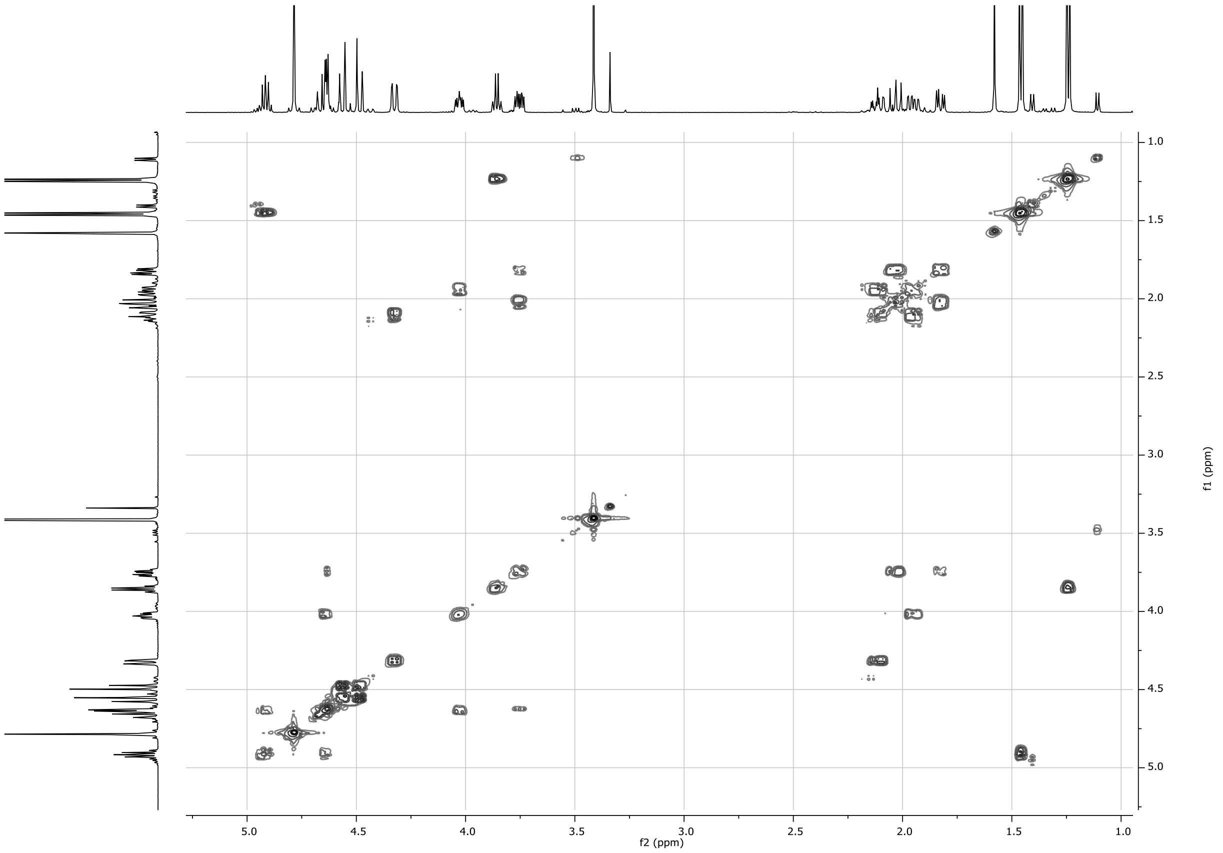

HSQC NMR, $CDCl_3$ of **21**

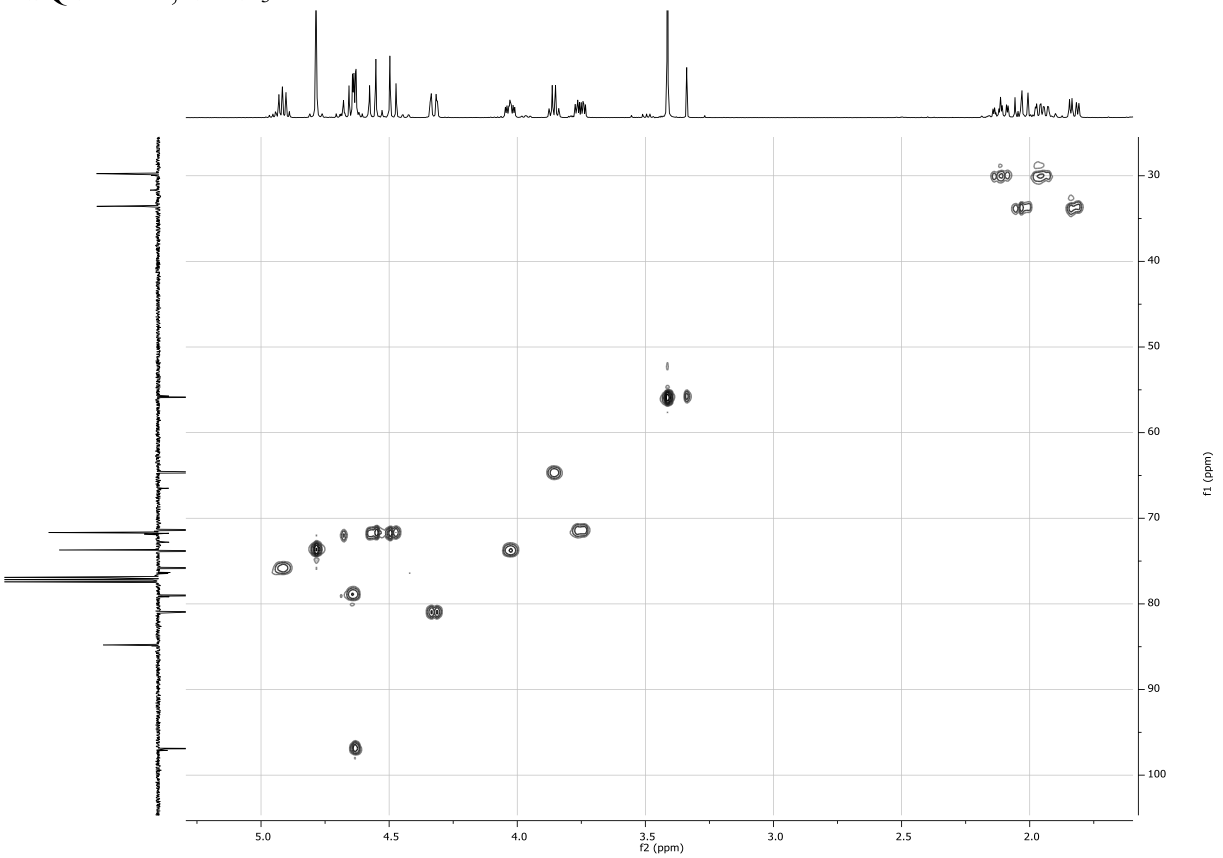

NOESY NMR, $CDCl_3$ of **21**

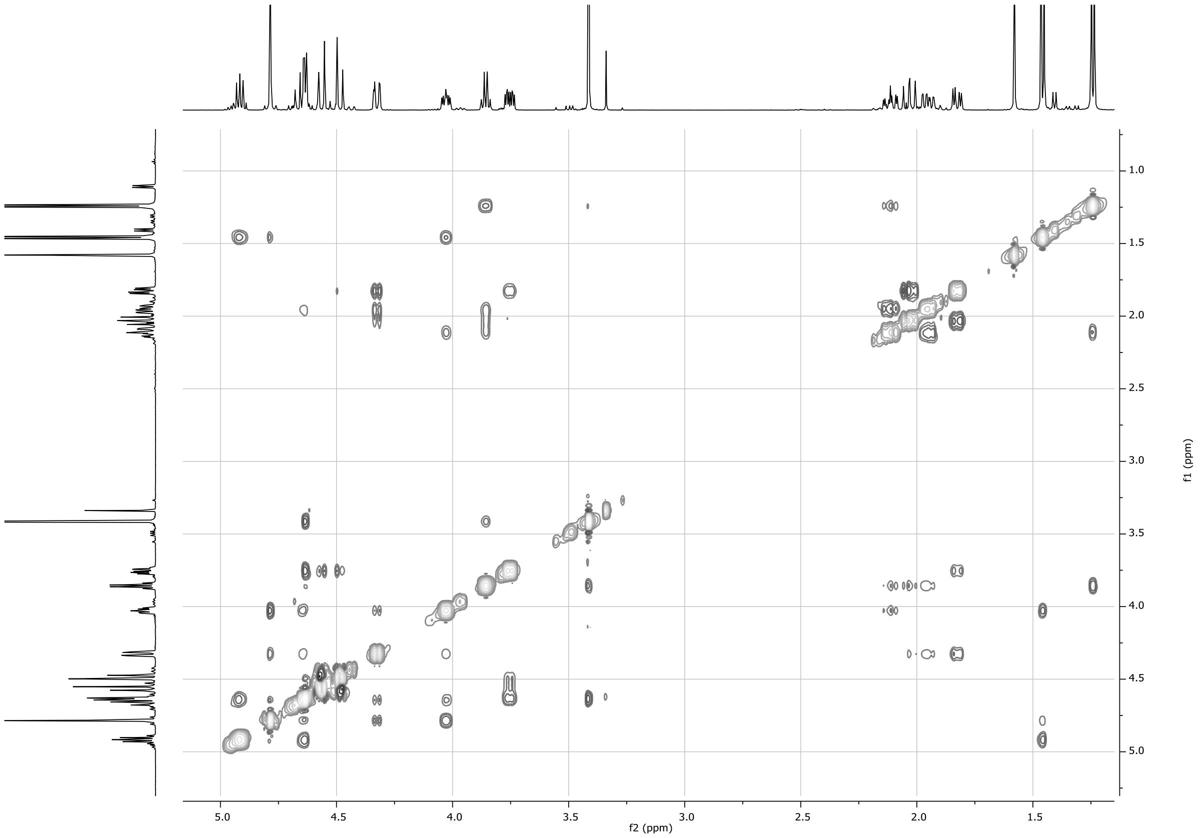

^{1}H NMR, 400 MHz, $CDCl_3$ of **22**

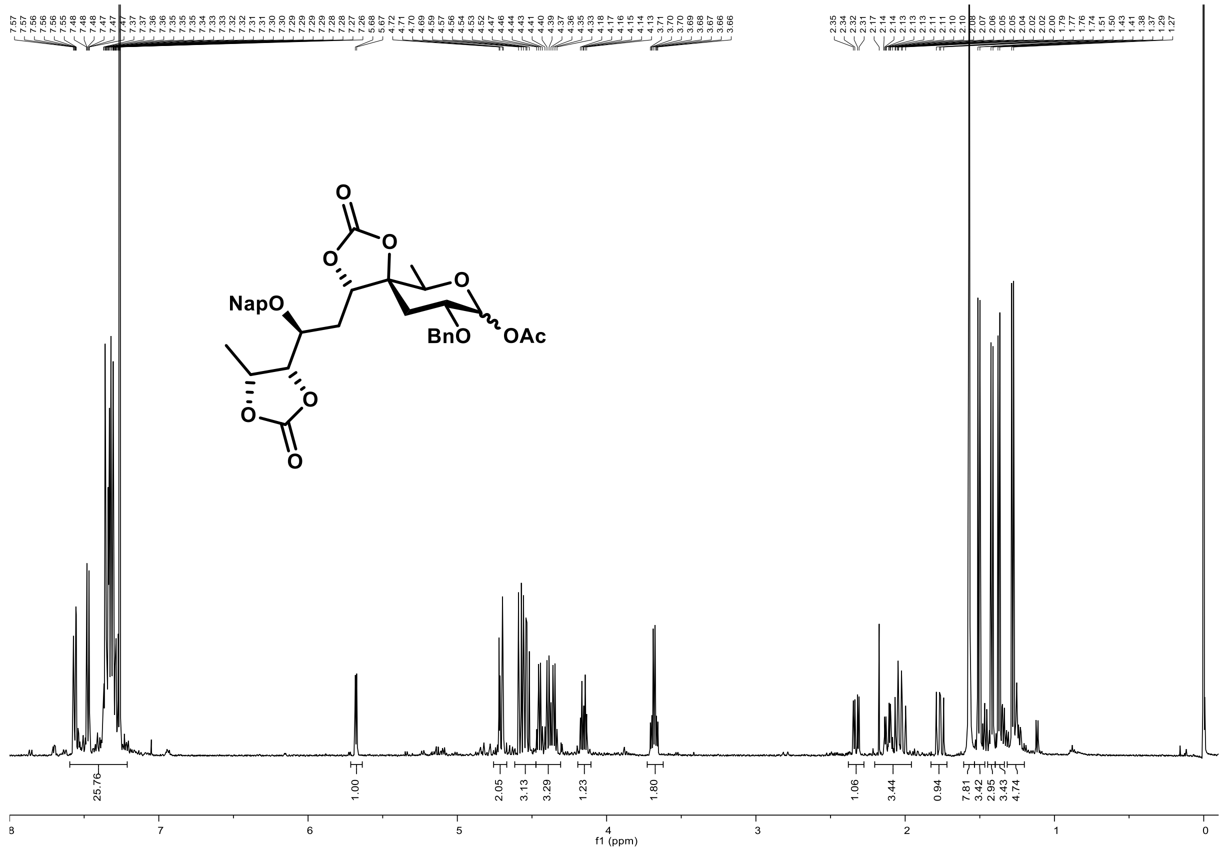

^{13}C NMR, 101 MHz, $CDCl_3$ of **22**

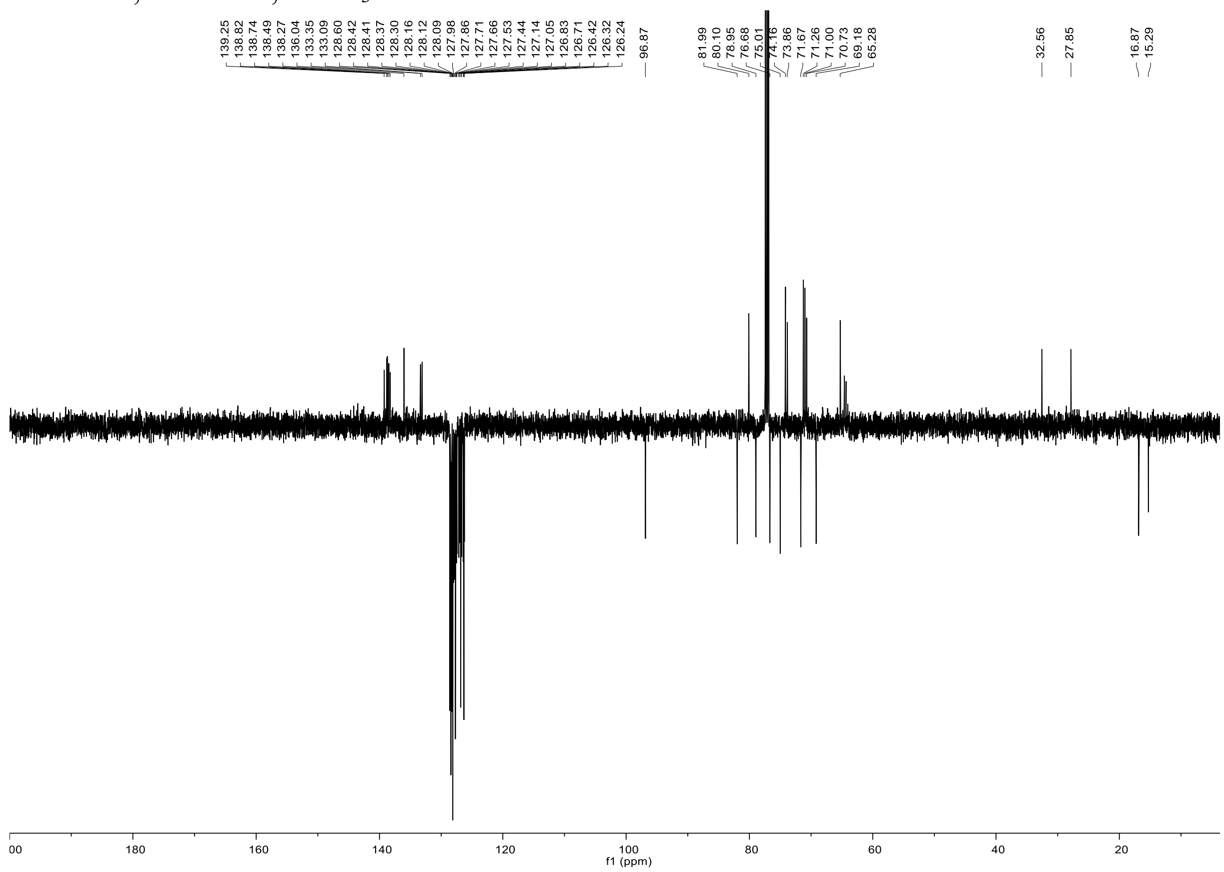

HH-COSY NMR, $CDCl_3$ of **22**

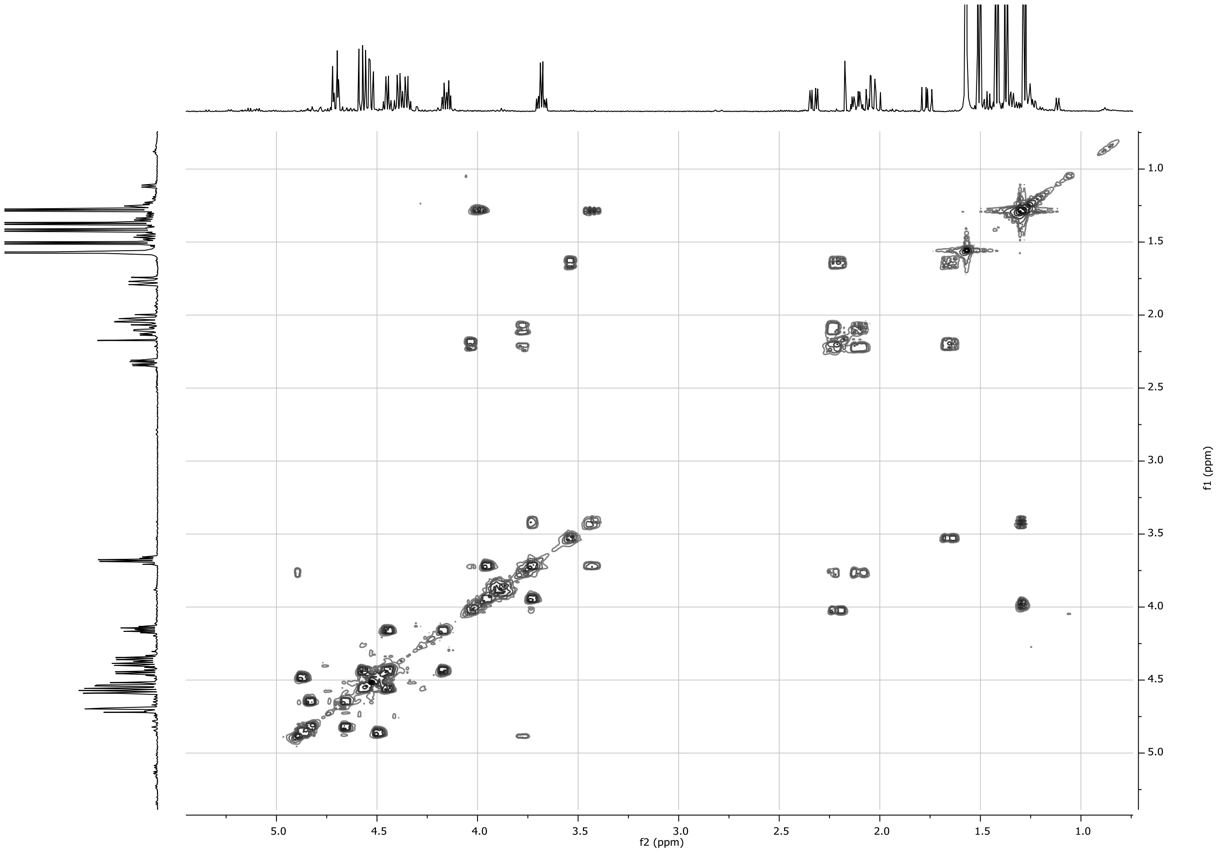

HSQC NMR, $CDCl_3$ of **22**

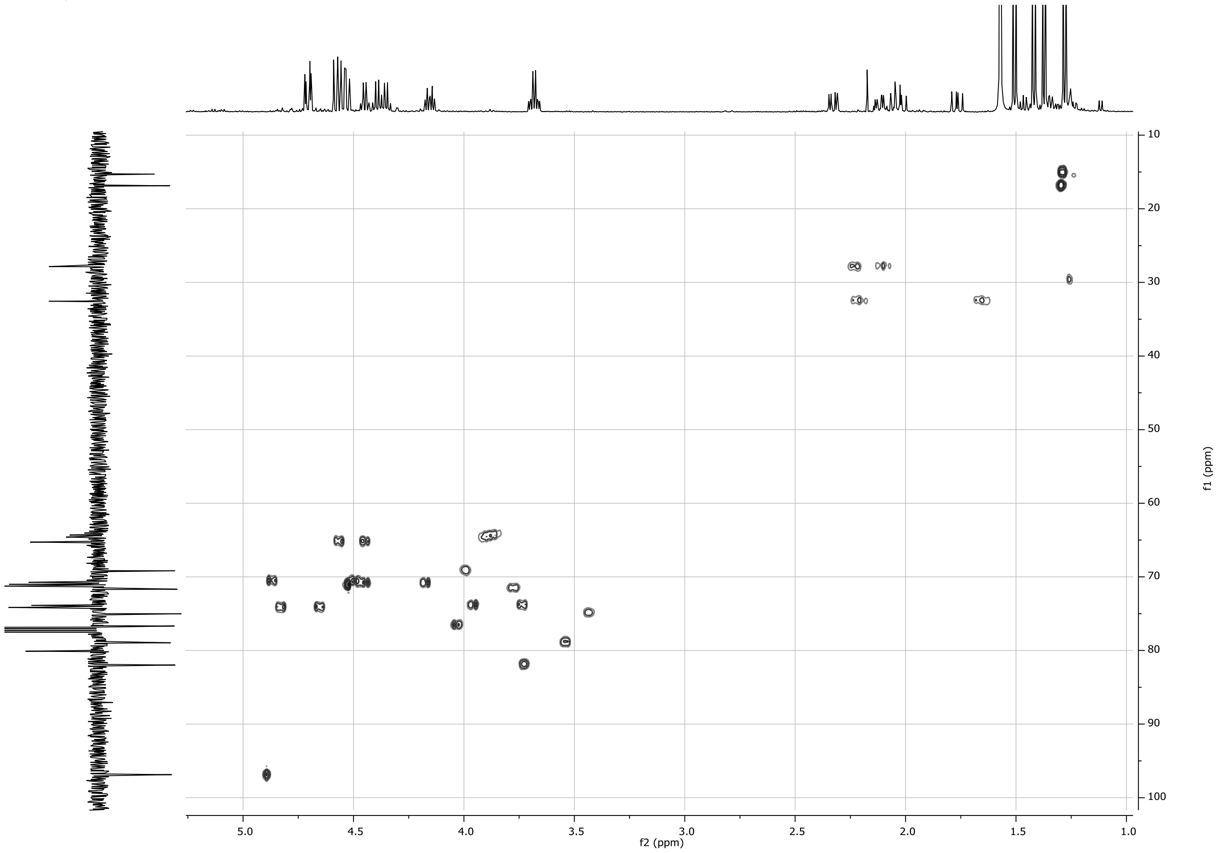

^{1}H NMR, 500 MHz, $CDCl_3$ of **4**

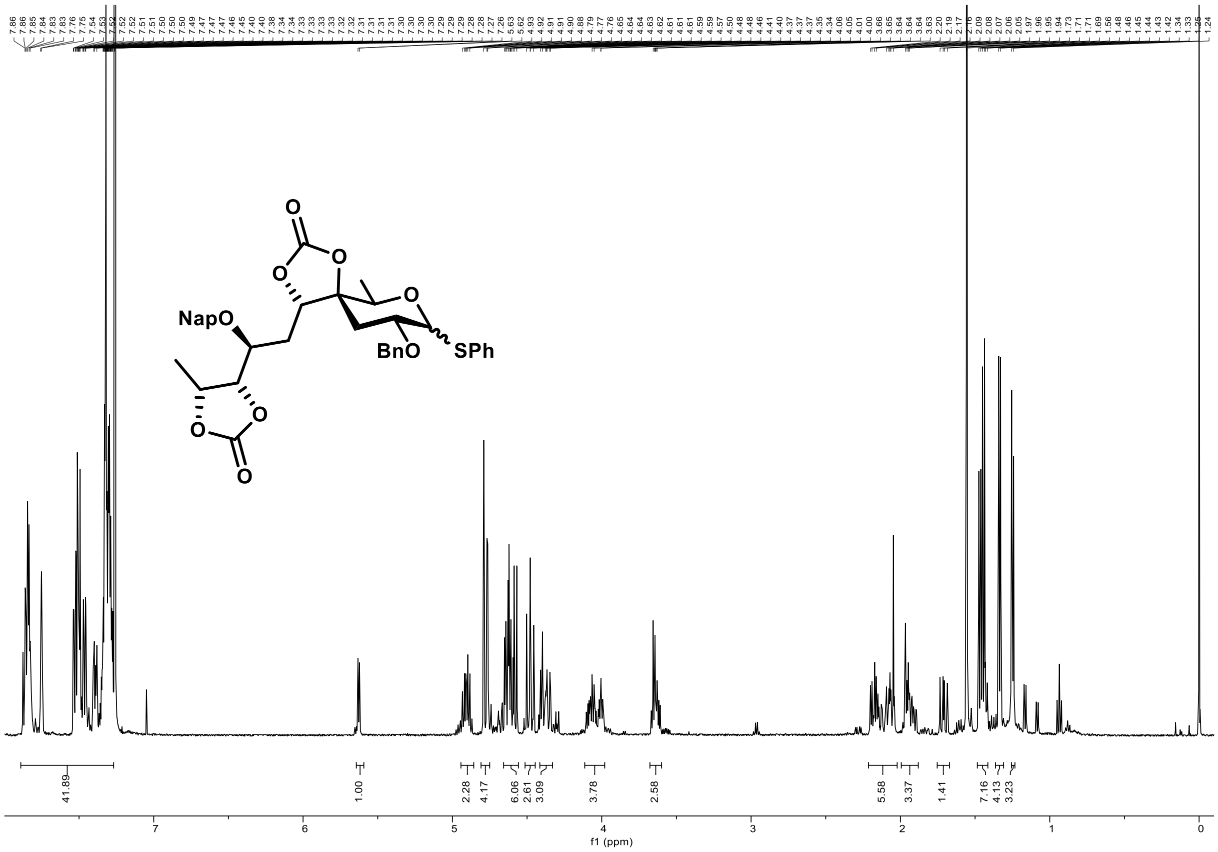

^{13}C NMR, 126 MHz, $CDCl_3$ of **4**

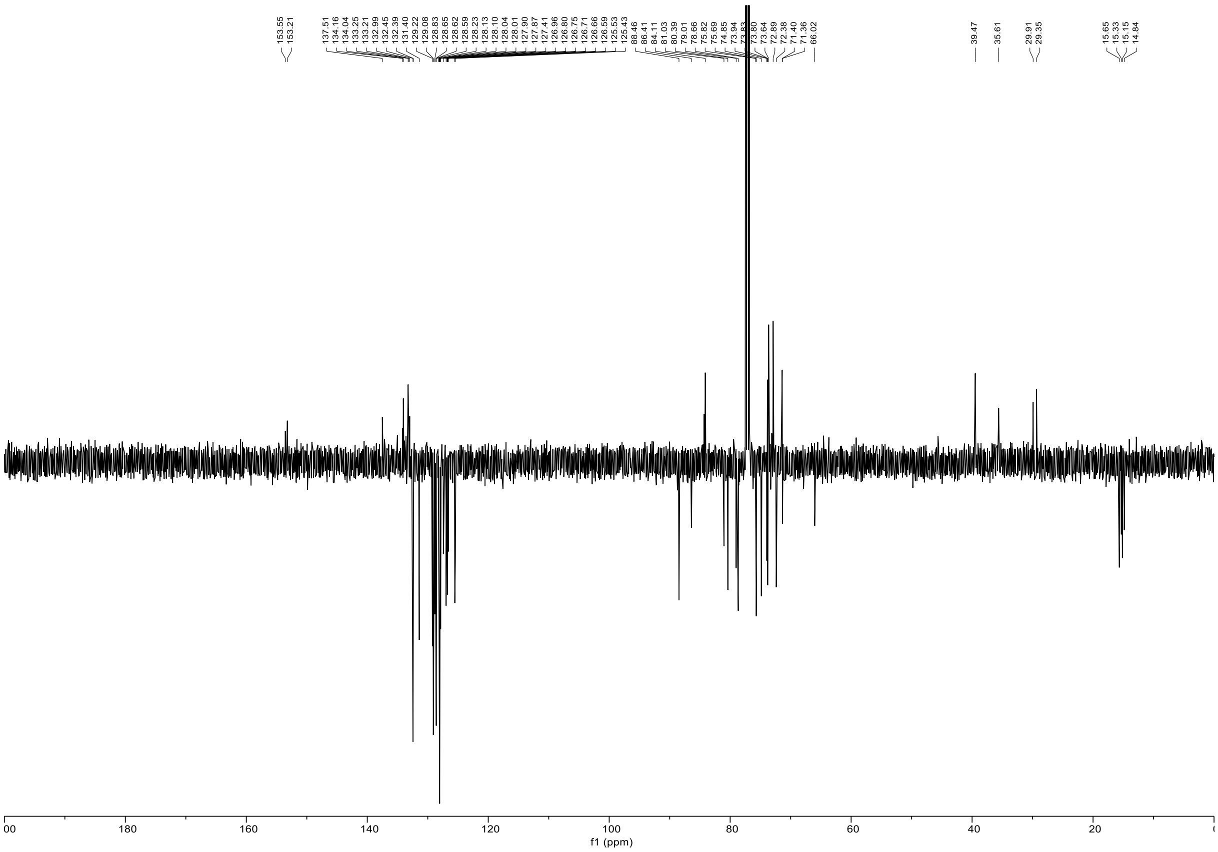

HH-COSY NMR, $CDCl_3$ of **4**

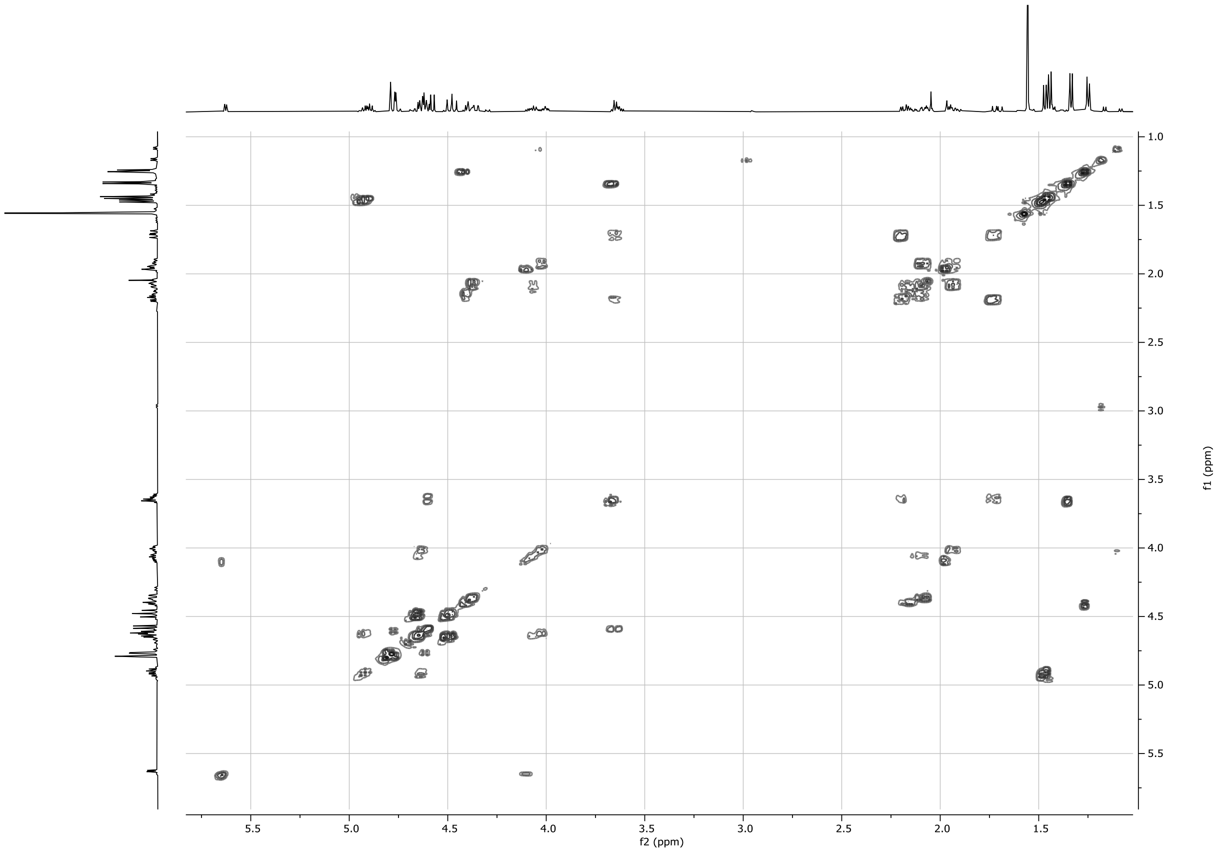

HSQC NMR, $CDCl_3$ of **4**

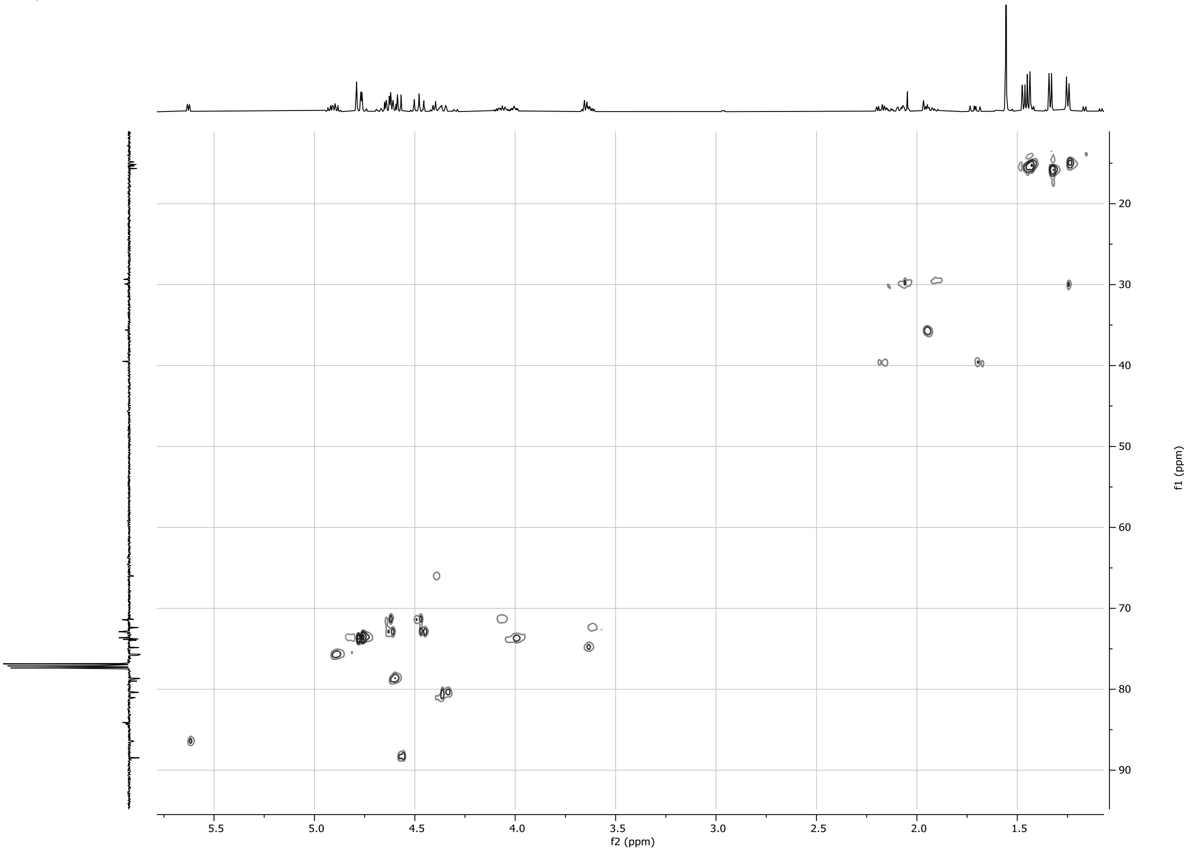

¹H NMR, 500 MHz, $CDCl_3$ of **S51**

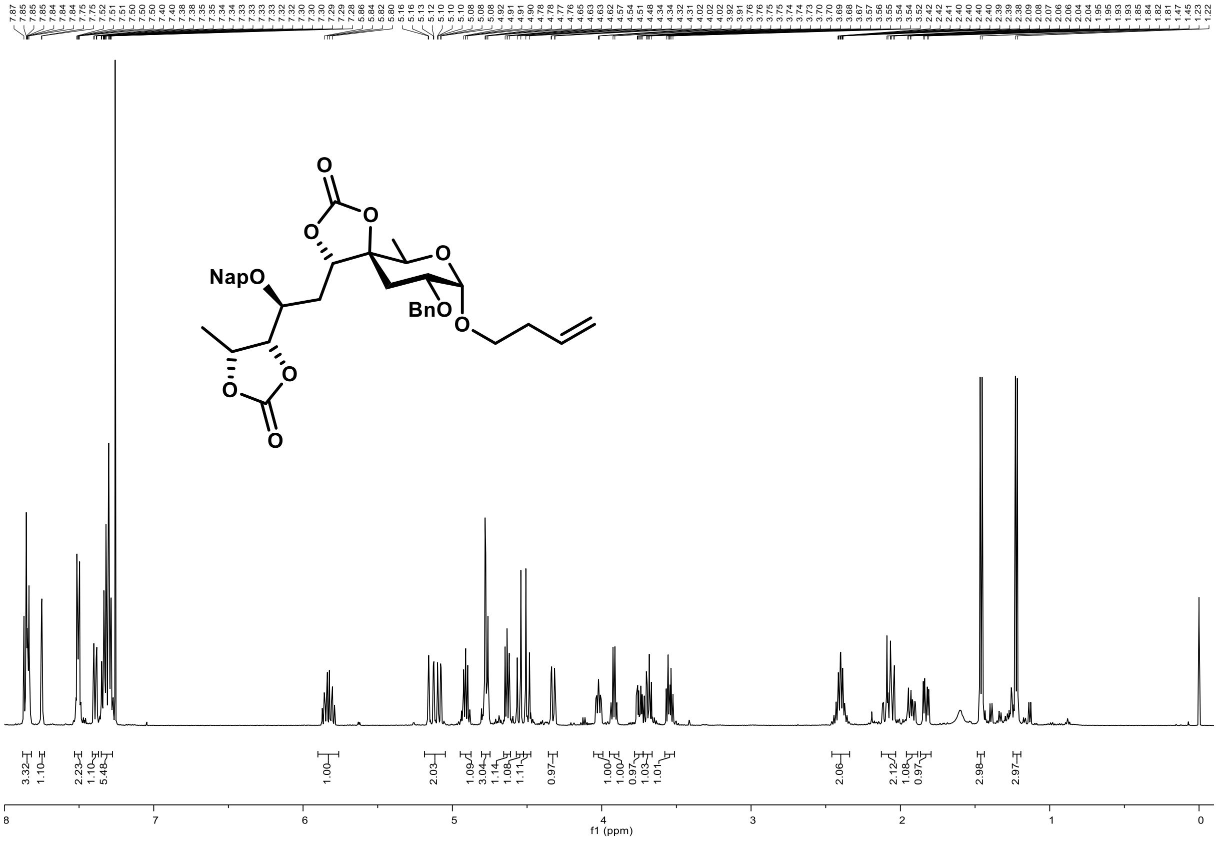

¹³C NMR, 126 MHz, $CDCl_3$ of **S51**

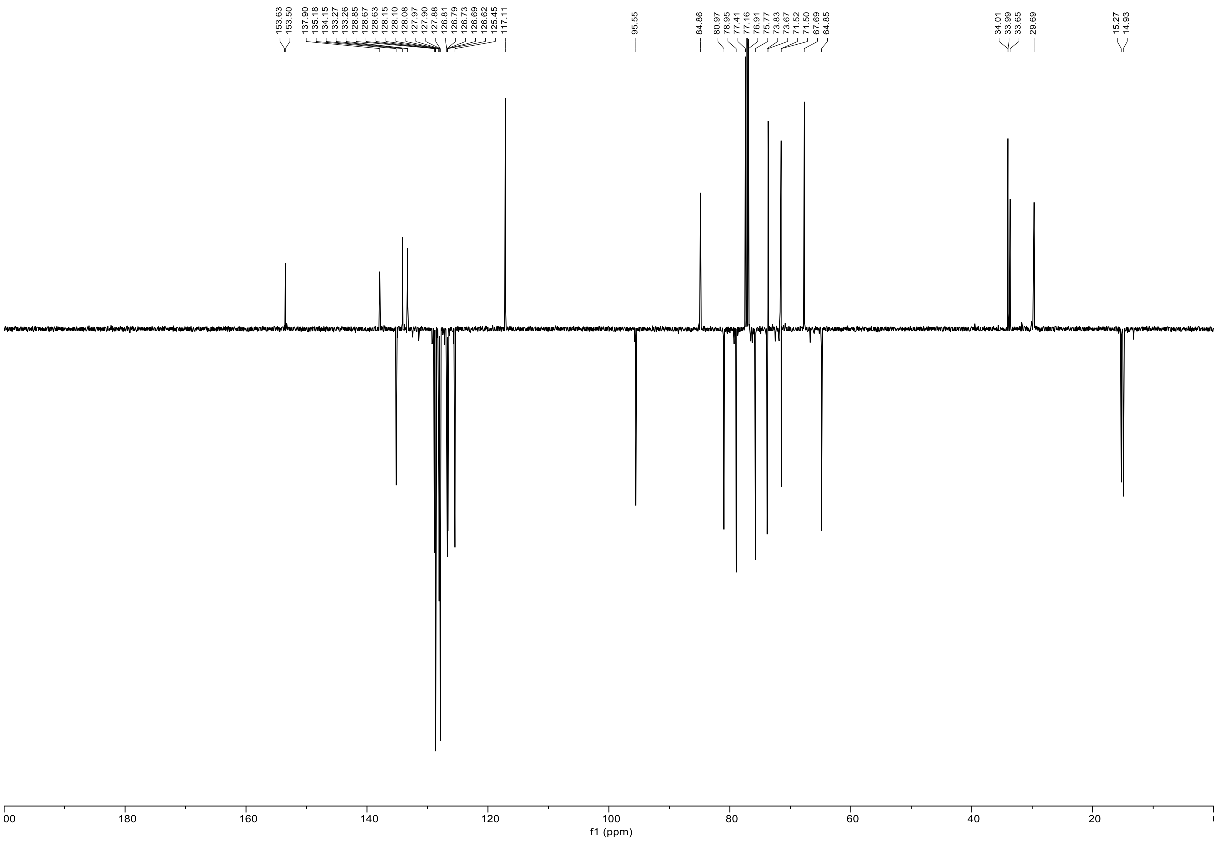

HH-COSY NMR, $CDCl_3$ of **S51**

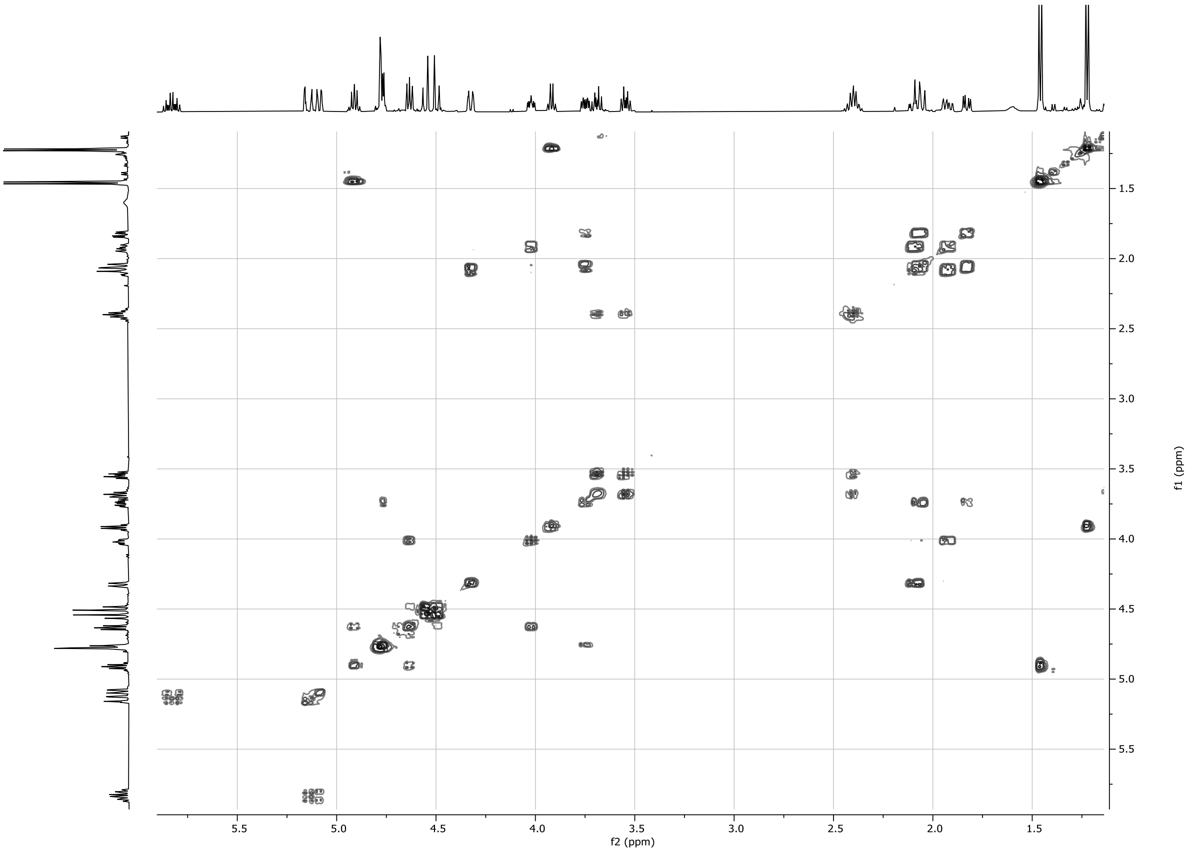

HSQC NMR, $CDCl_3$ of **S51**

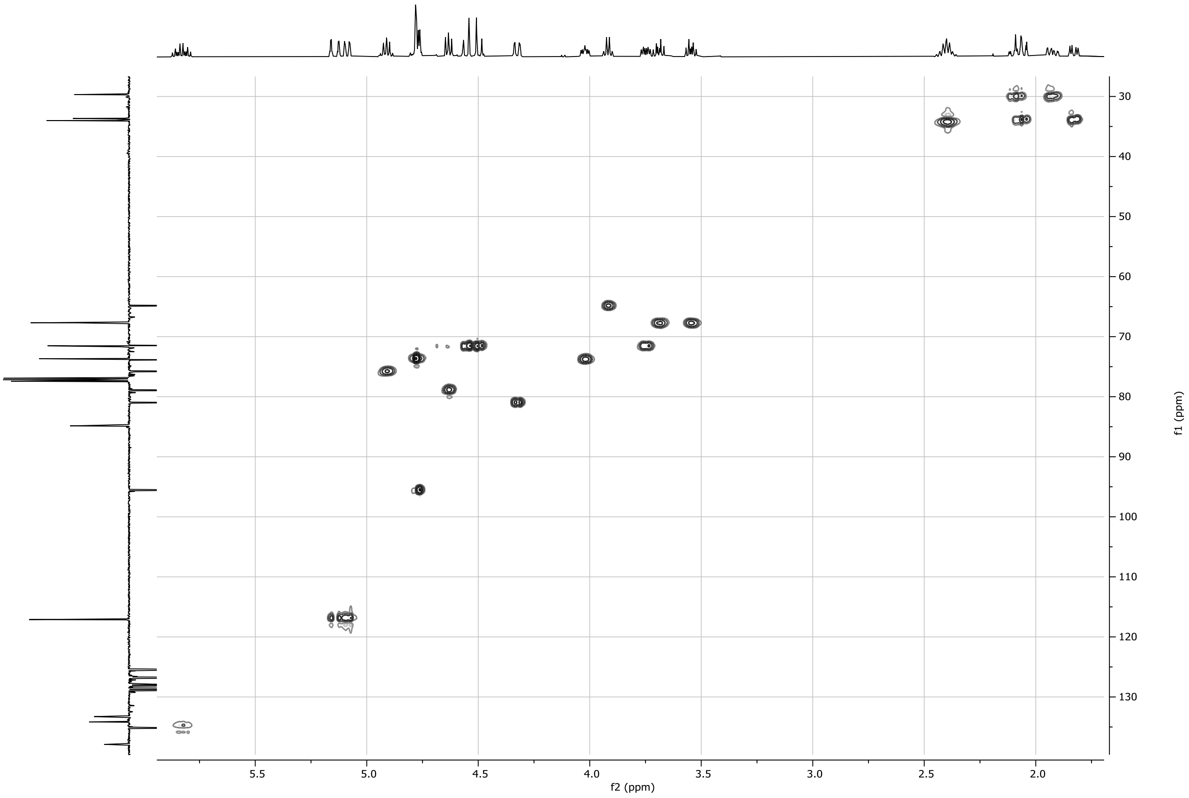

HMBC NMR, $CDCl_3$ of **S51**

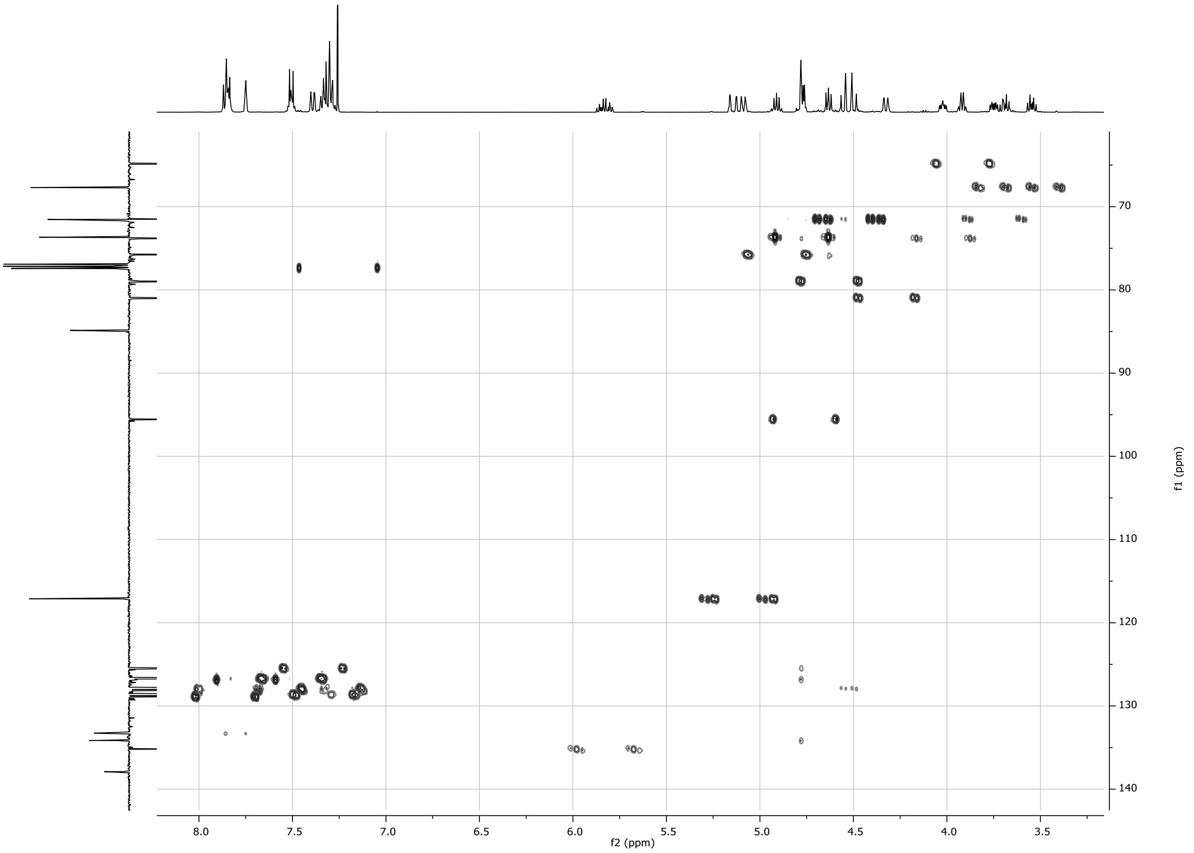

^{1}H NMR, 400 MHz, $CDCl_3$ of **S1**

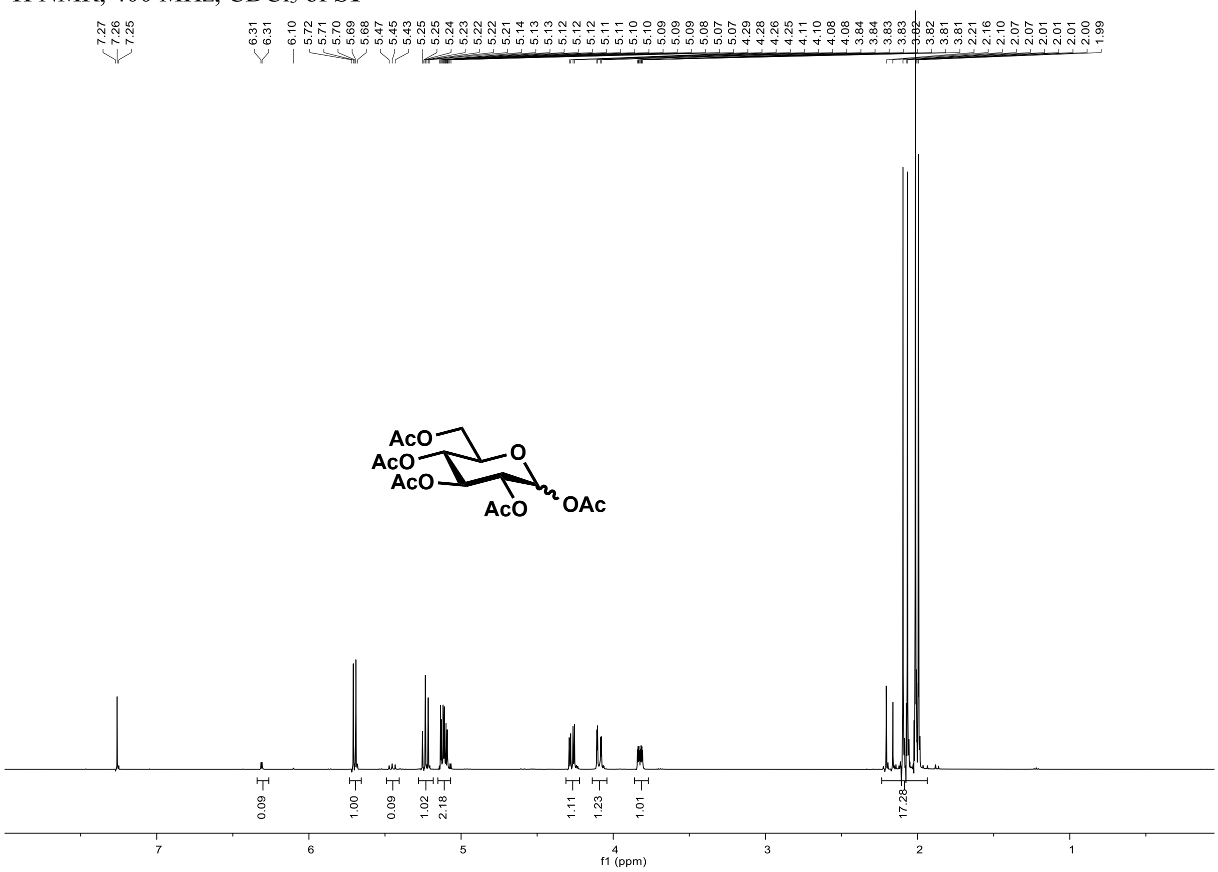

^{13}C NMR, 101 MHz, $CDCl_3$ of **S1**

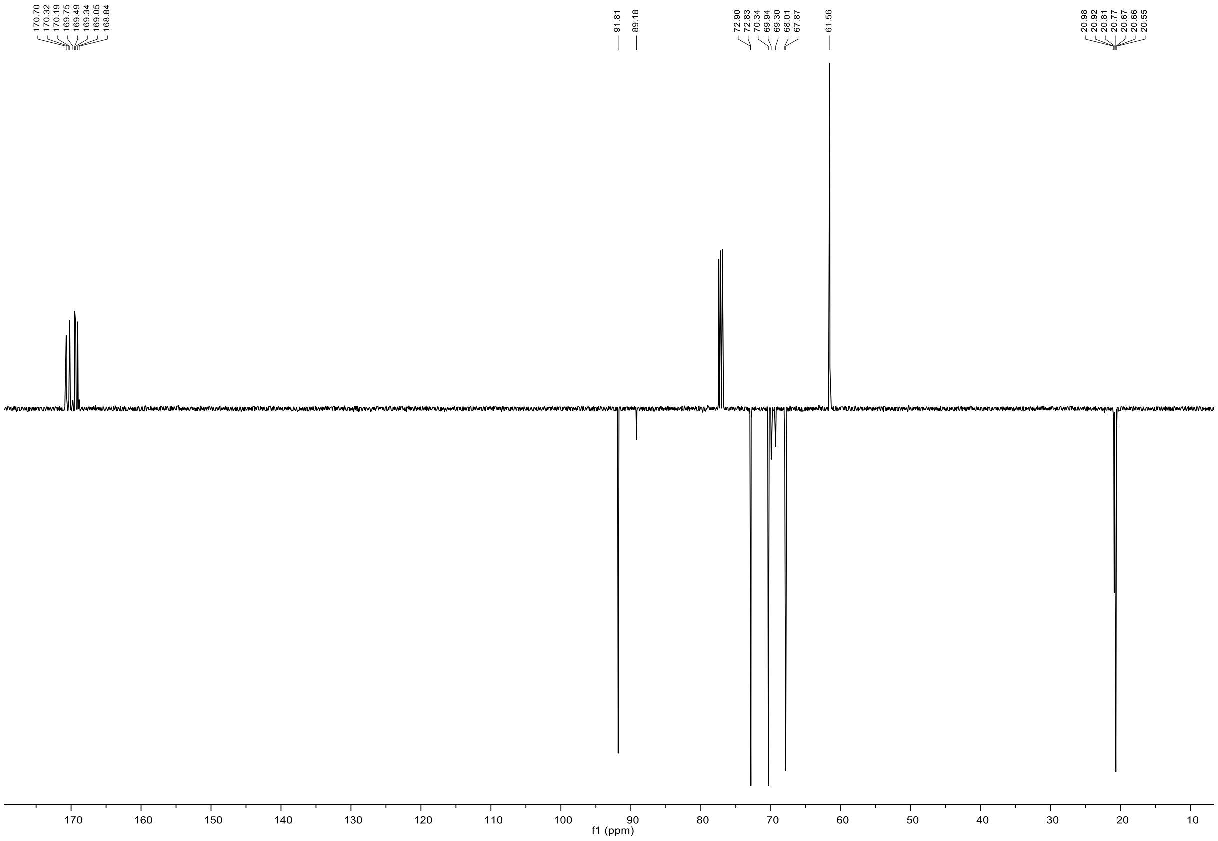

HH-COSY NMR, $CDCl_3$ of **S1**

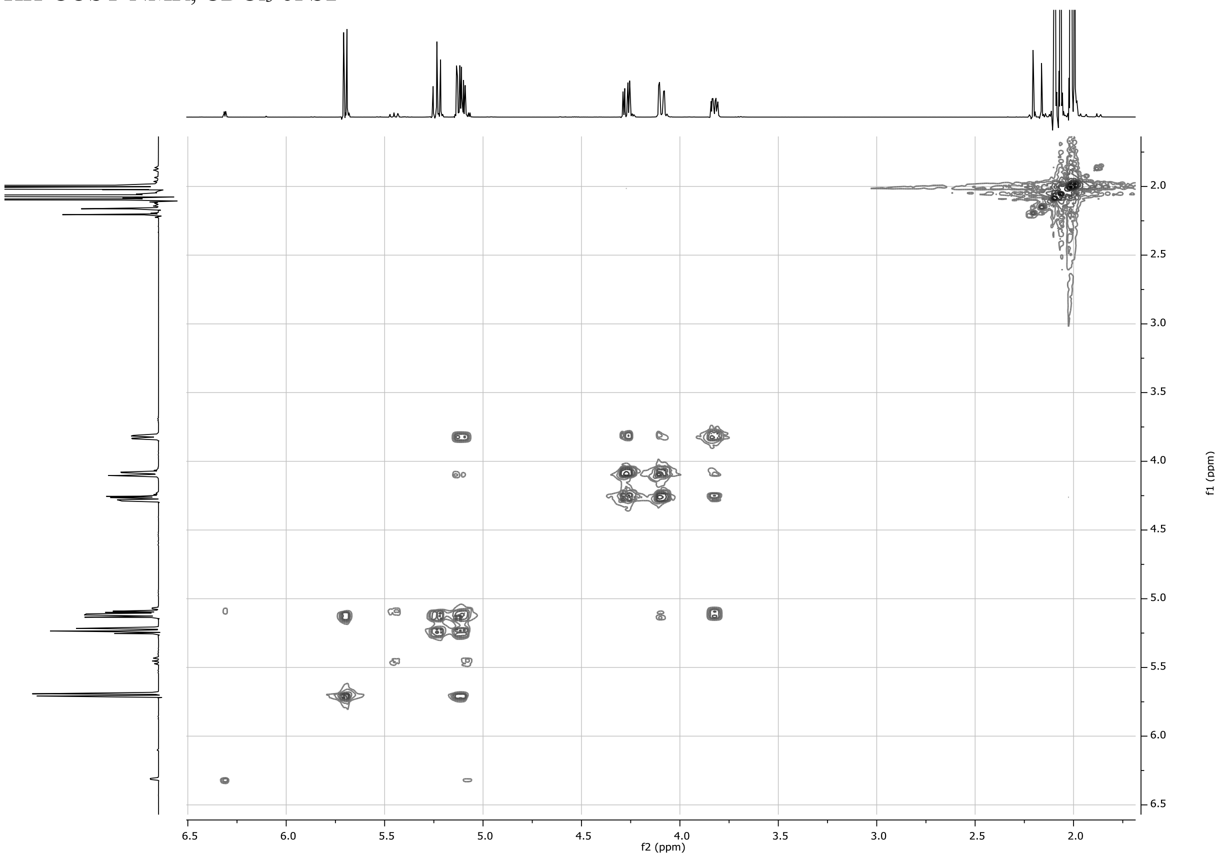

HSQC NMR, $CDCl_3$ of **S1**

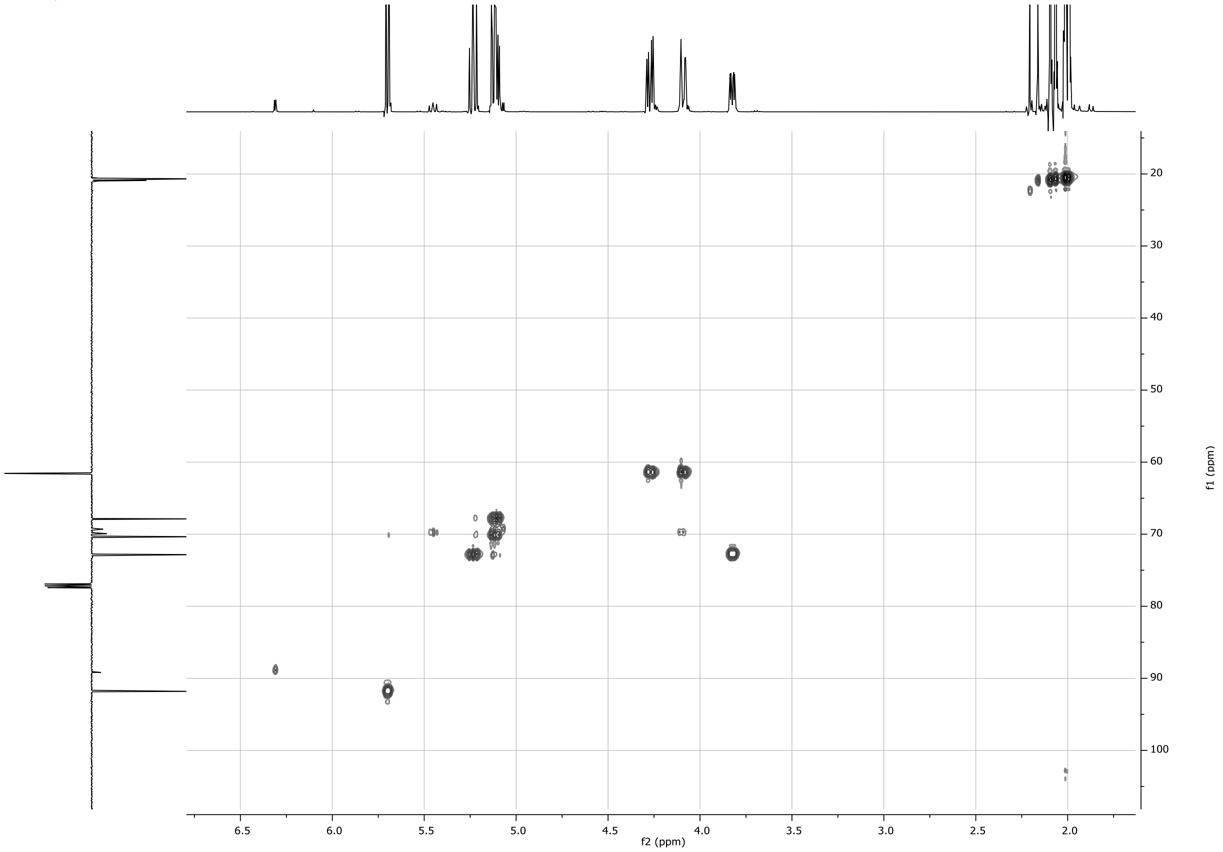

^{1}H NMR, 400 MHz, $CDCl_3$ of **S2**

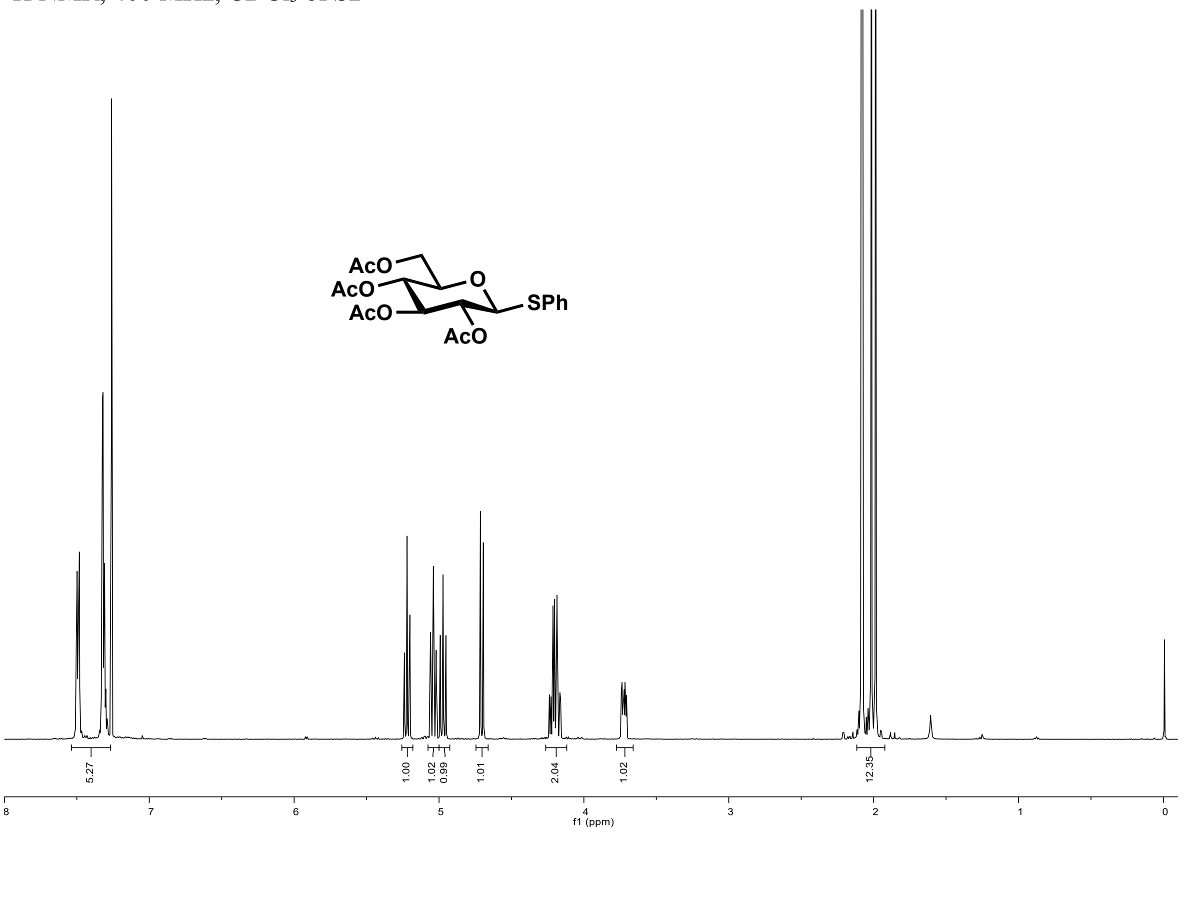

^{13}C NMR, 101 MHz, $CDCl_3$ of **S2**

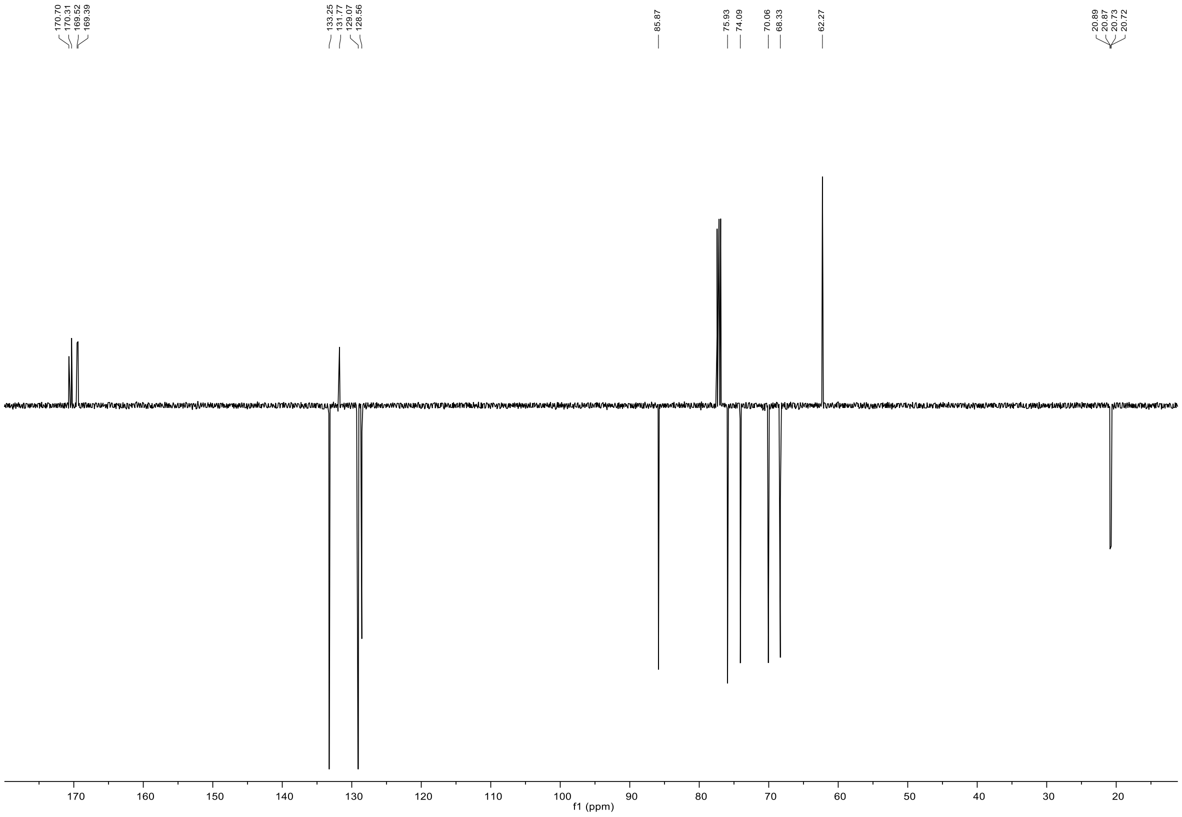

HH-COSY NMR, $CDCl_3$ of **S2**

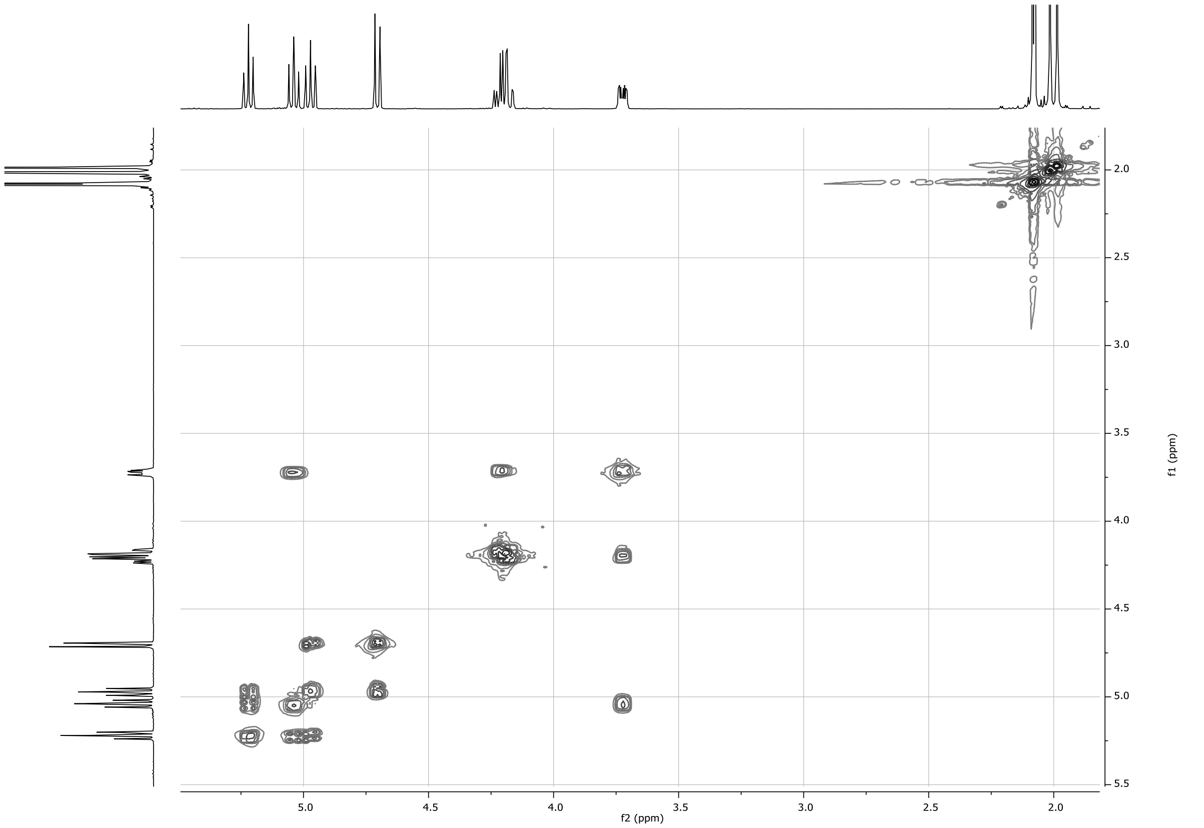

HSQC NMR, $CDCl_3$ of **S2**

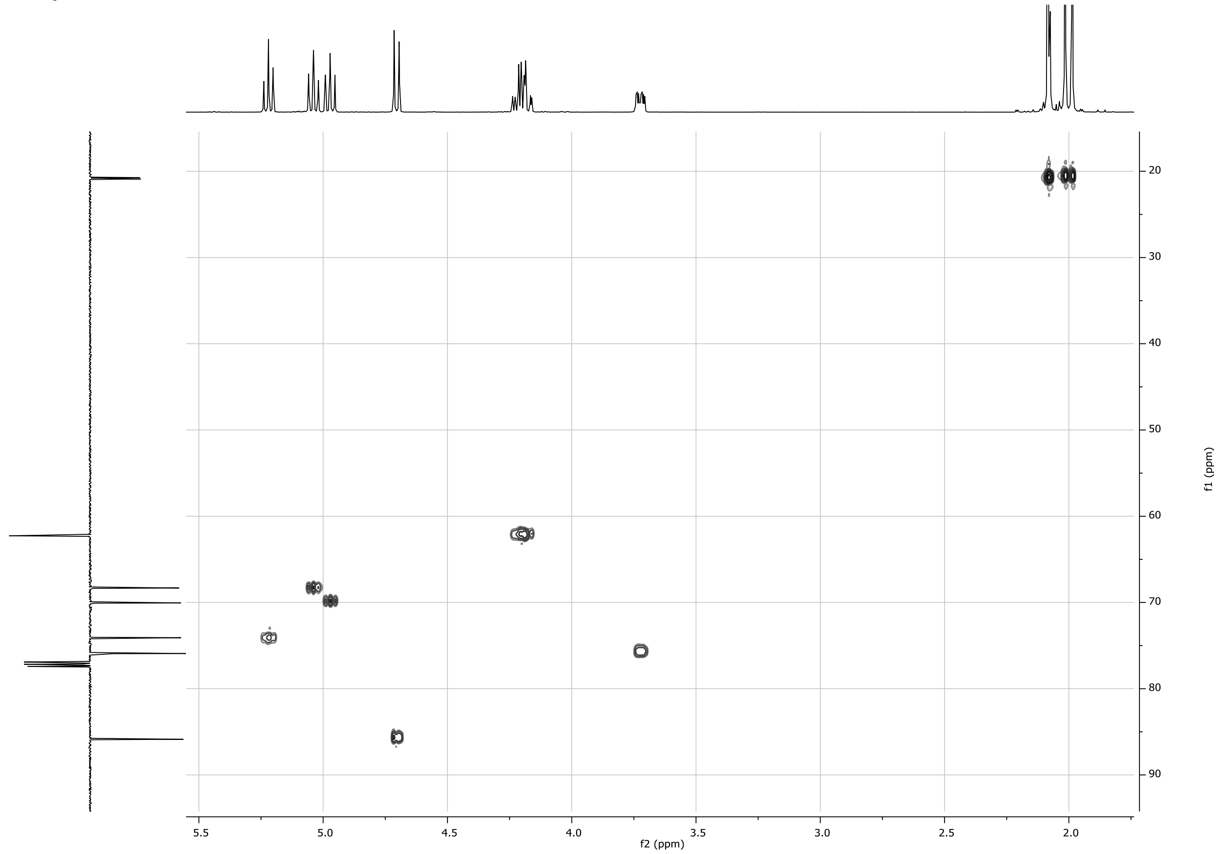

^{1}H NMR, 400 MHz, $CDCl_3$ of **S3**

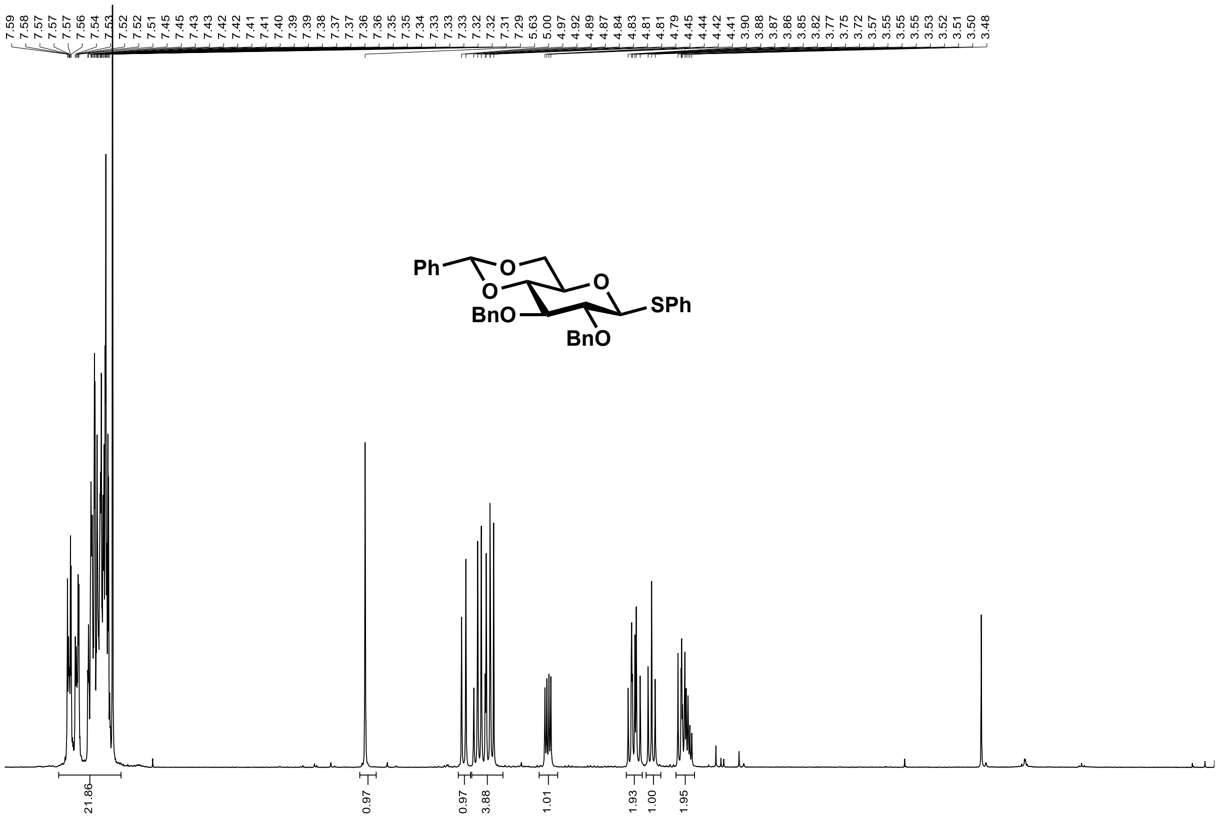

^{13}C NMR, 101 MHz, $CDCl_3$ of **S3**

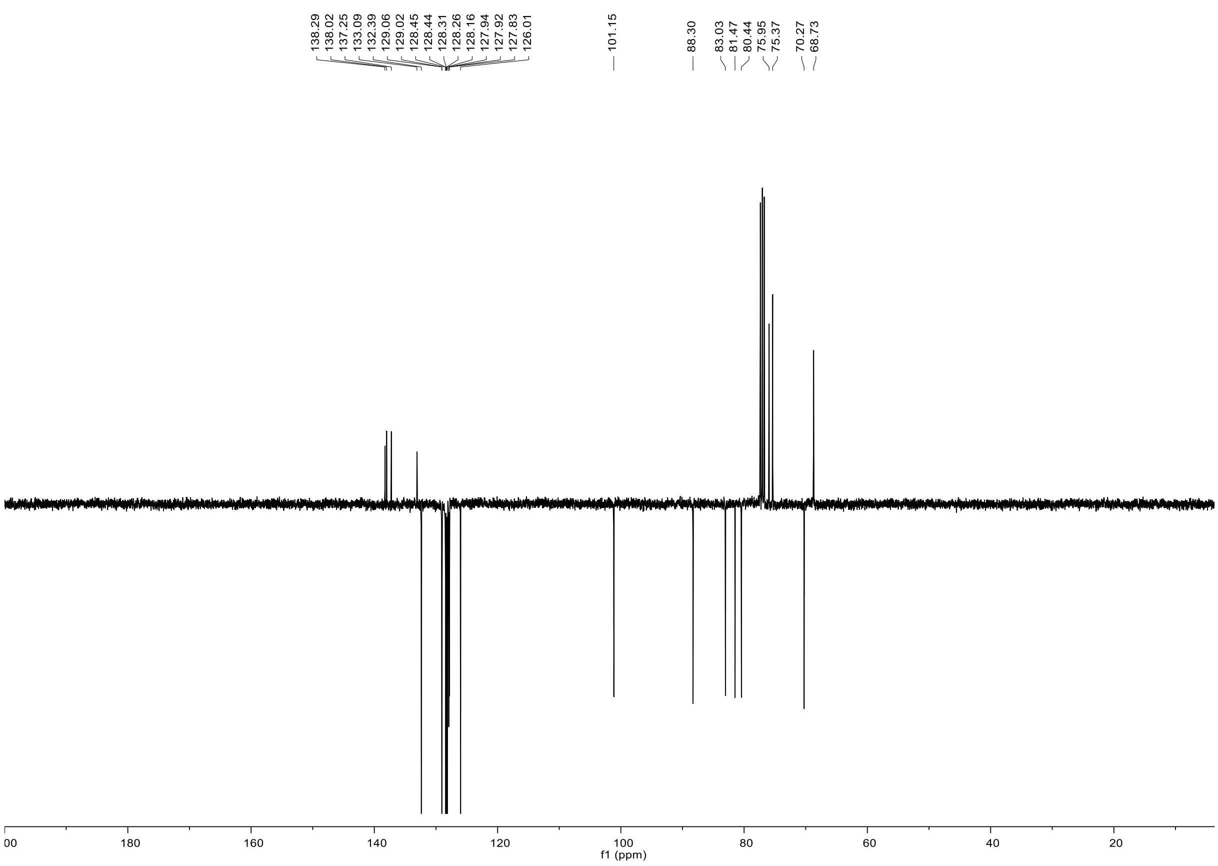

HH-COSY NMR, $CDCl_3$ of **S3**

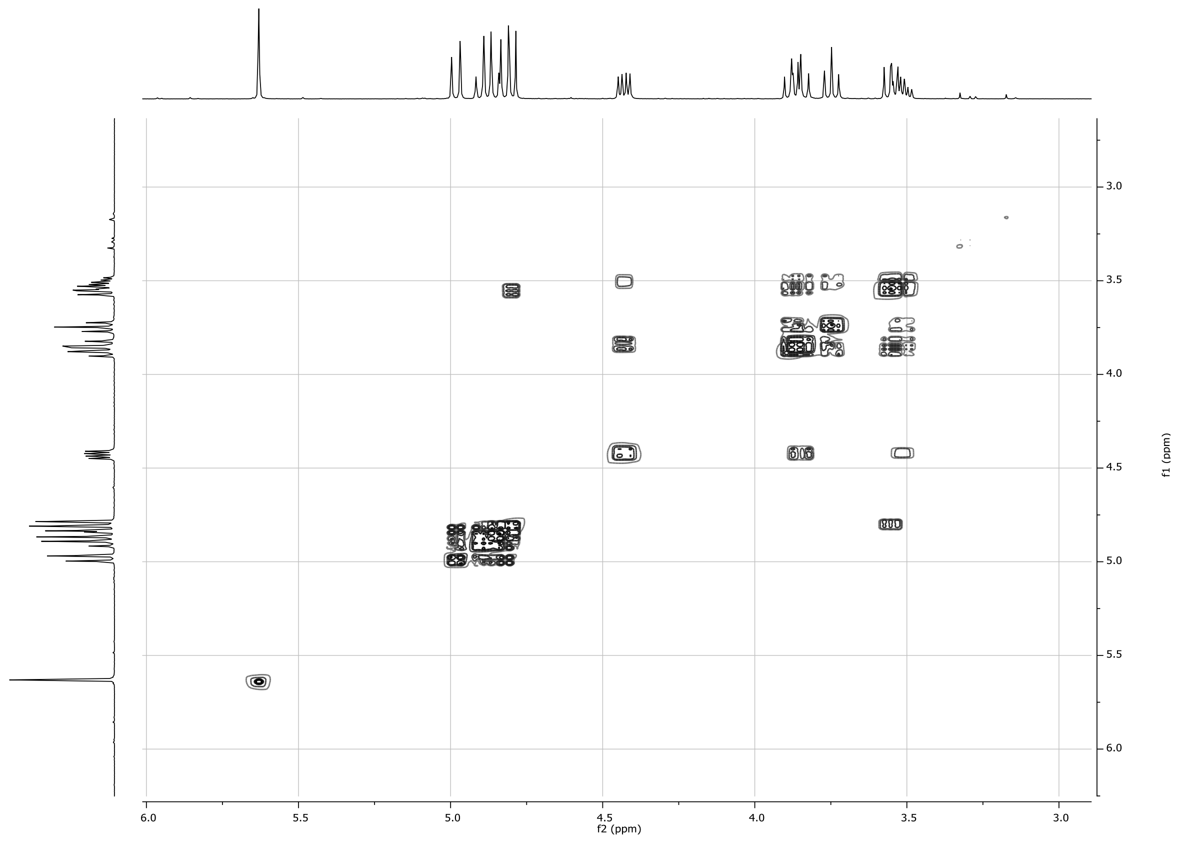

HSQC NMR, $CDCl_3$ of **S3**

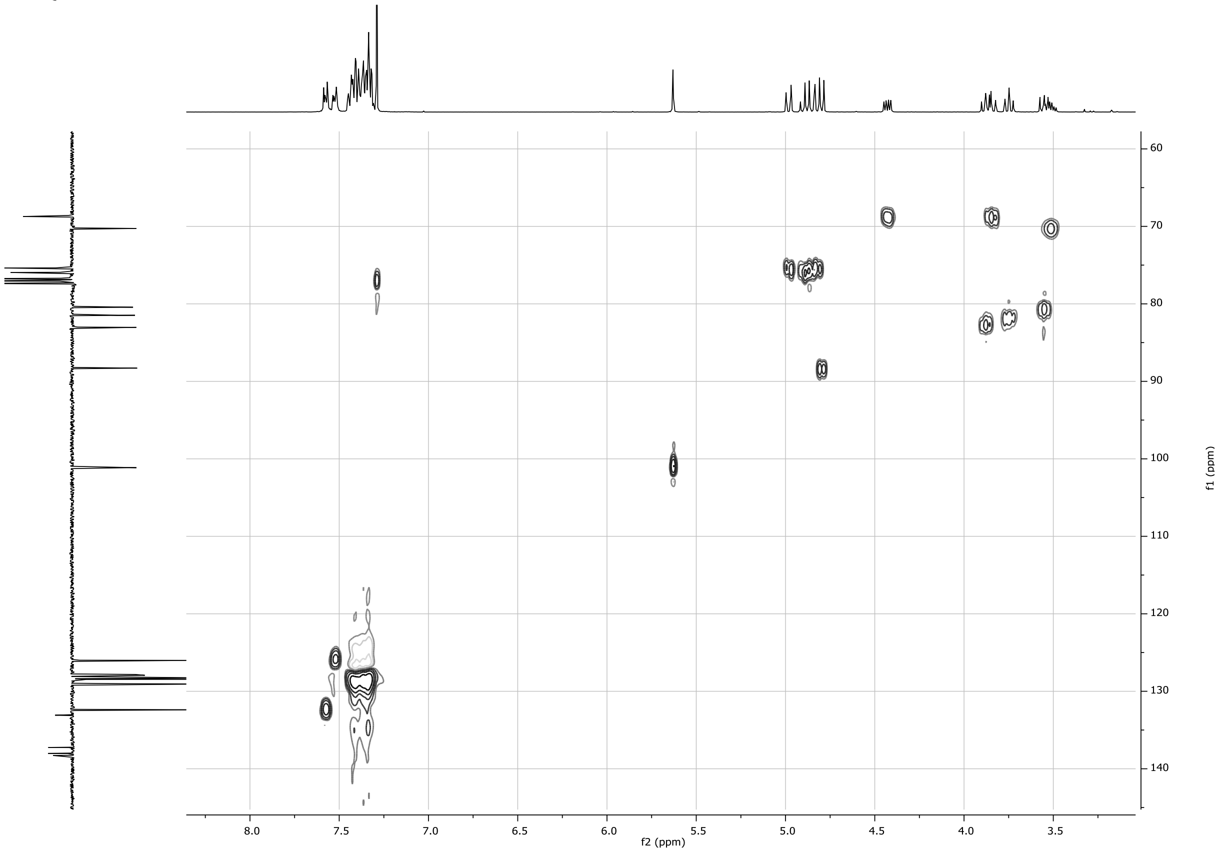

^{1}H NMR, 400 MHz, $CDCl_3$ of **S4**

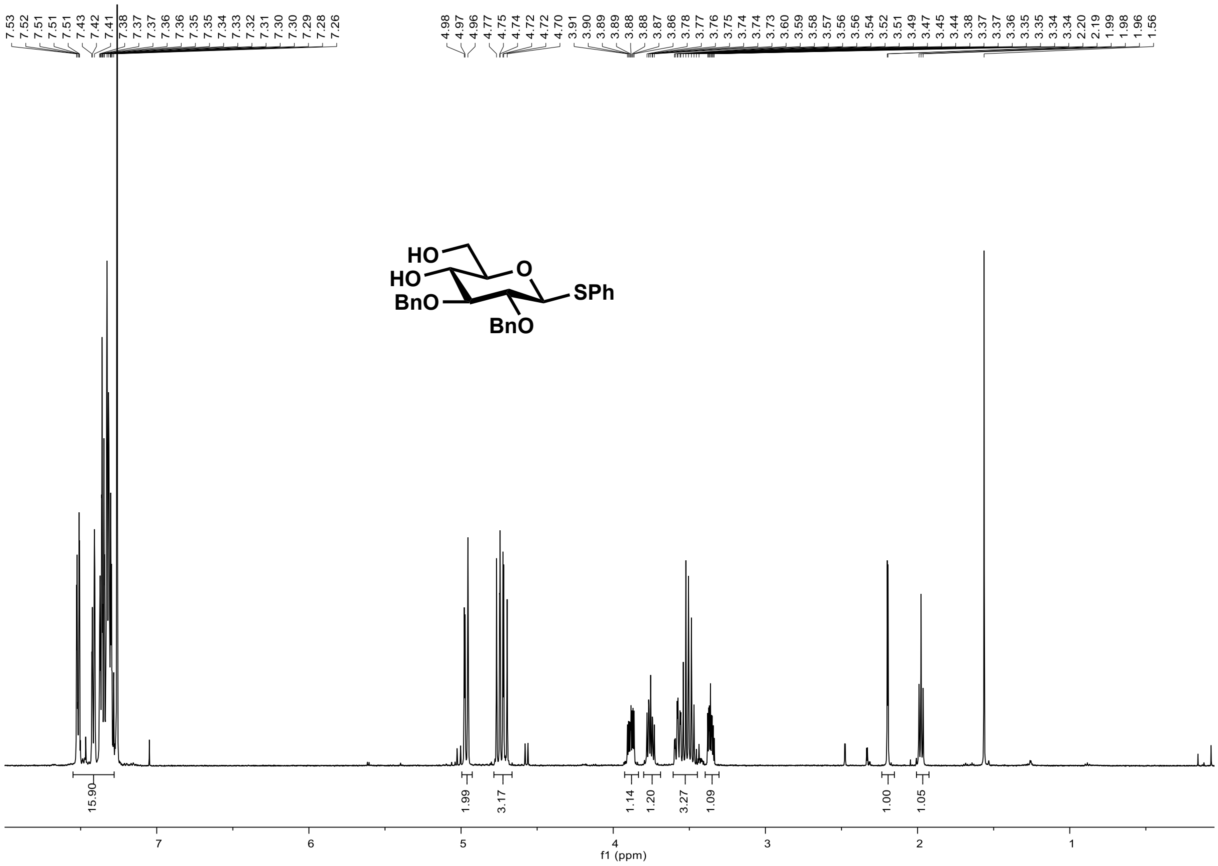

^{13}C NMR, 101 MHz, $CDCl_3$ of **S4**

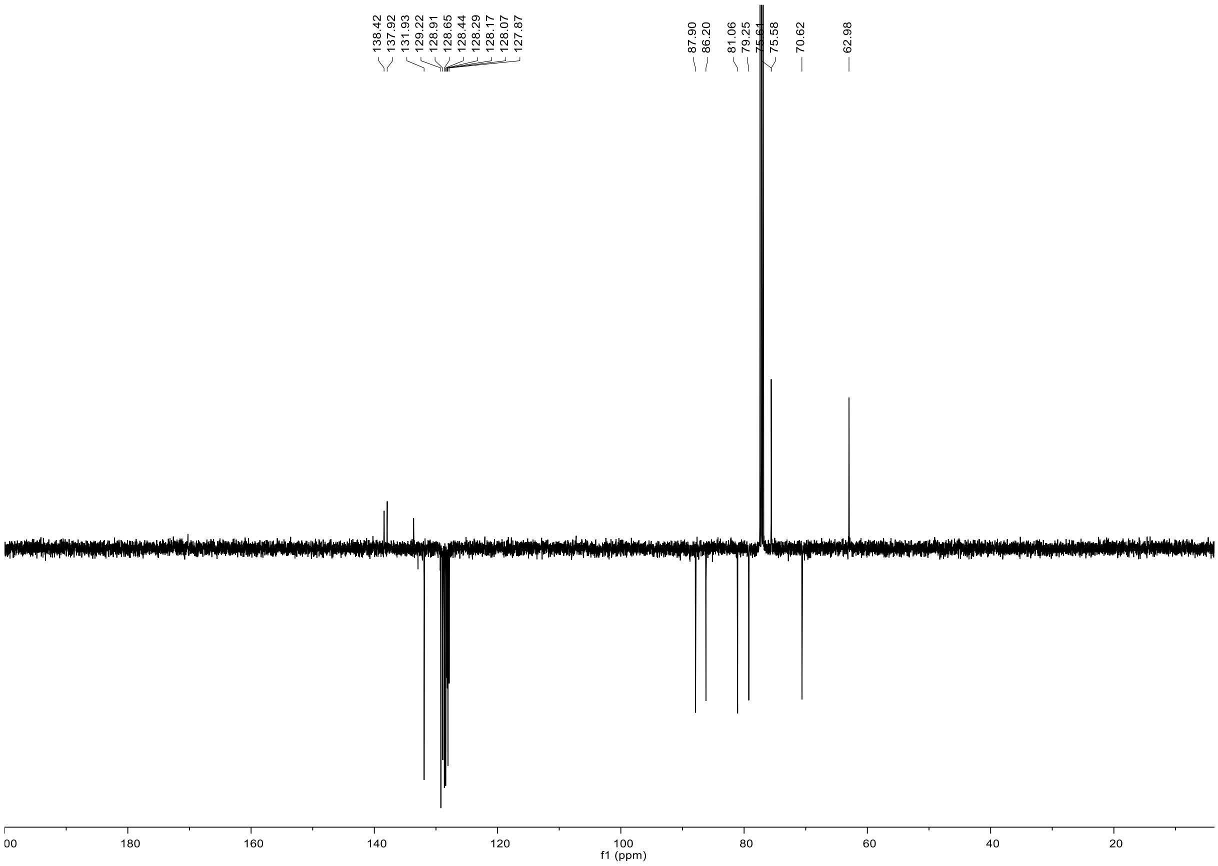

HH-COSY NMR, $CDCl_3$ of **S4**

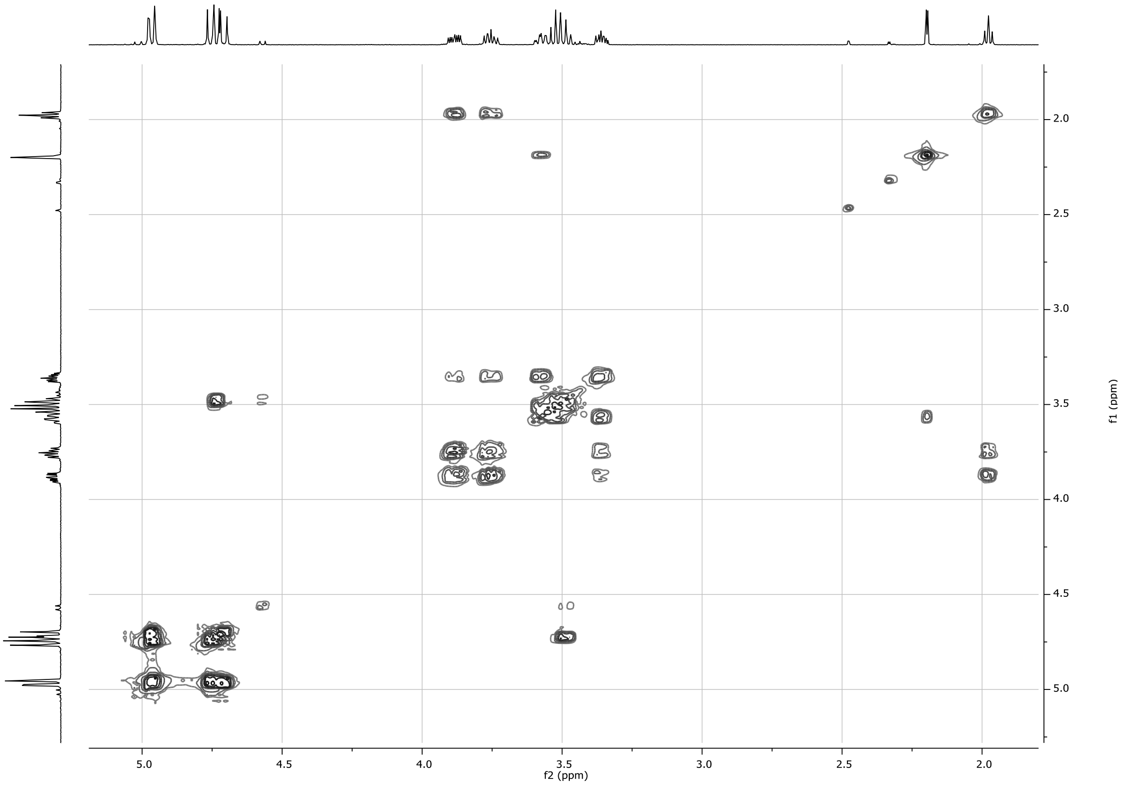

HSQC NMR, $CDCl_3$ of **S4**

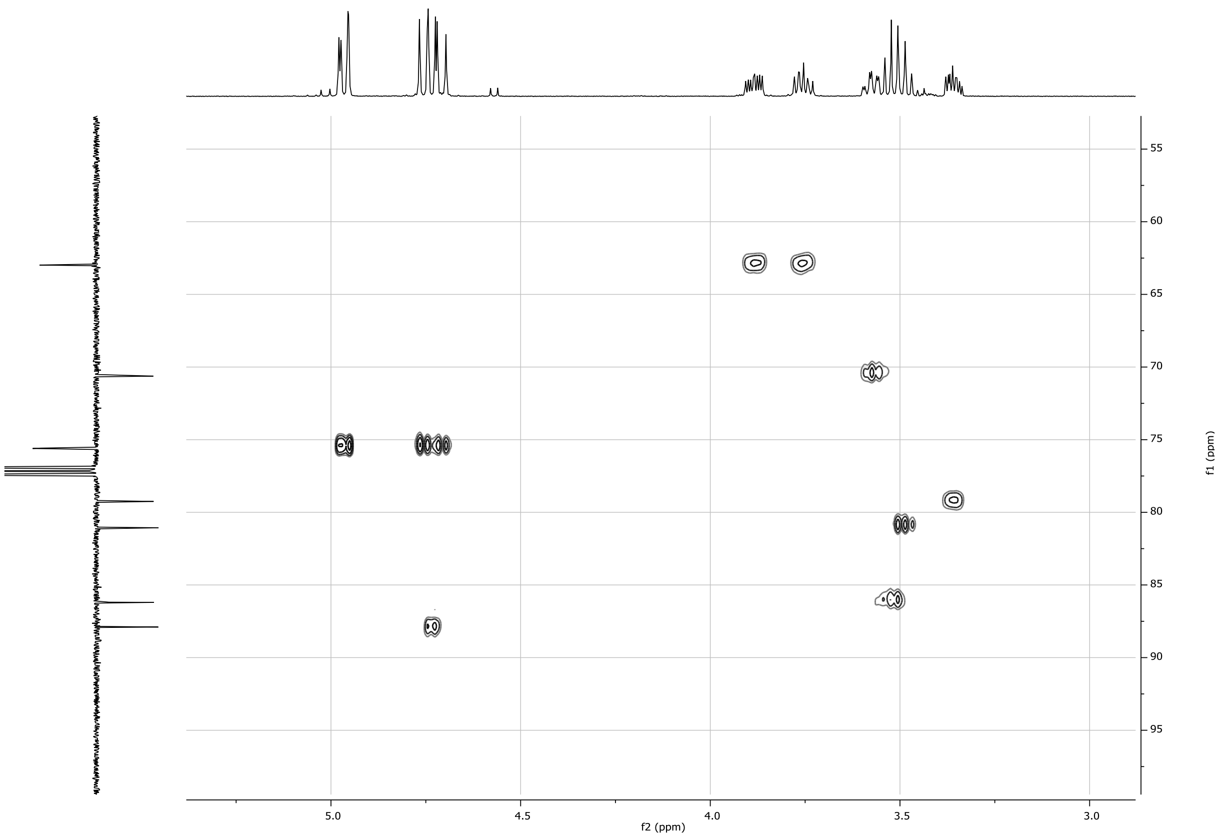

^{1}H NMR, 400 MHz, $CDCl_3$ of **S5**

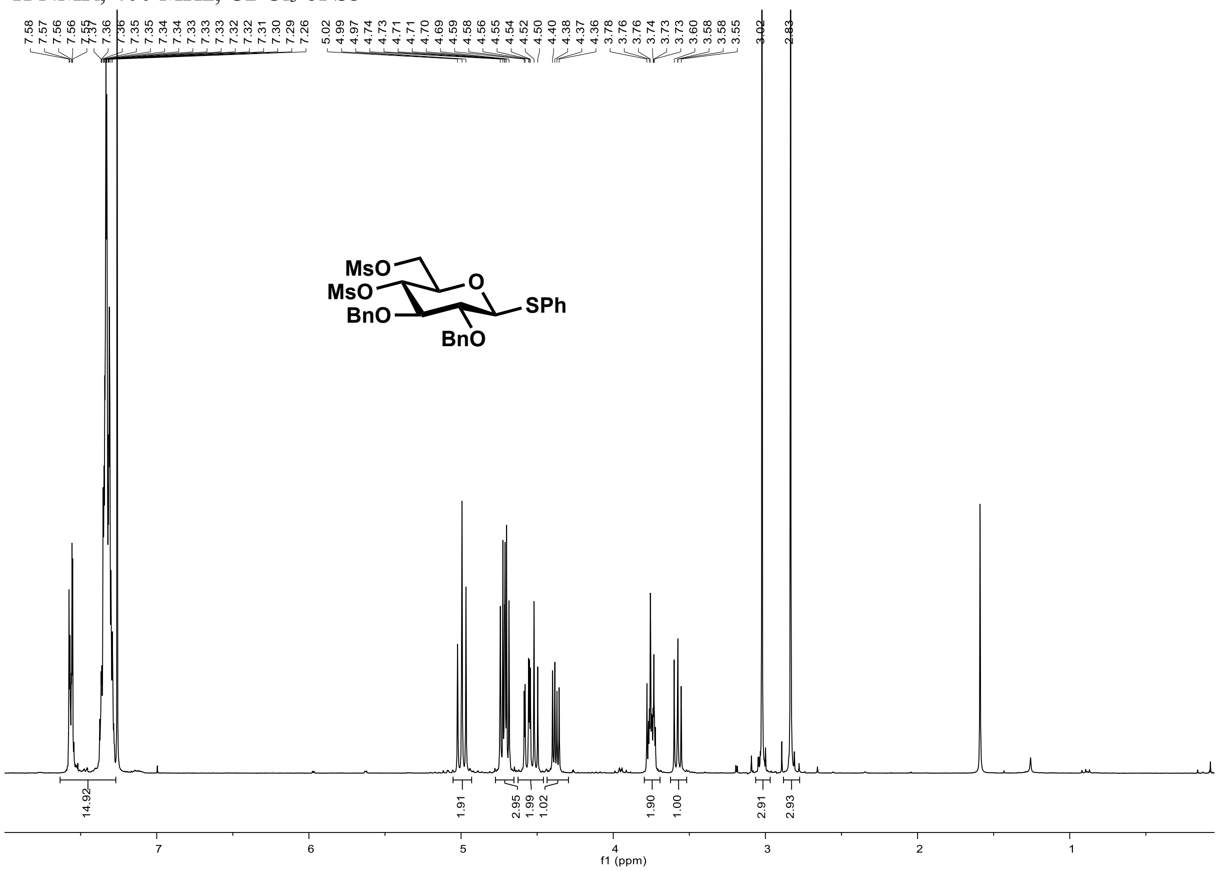

^{13}C NMR, 101 MHz, $CDCl_3$ of **S5**

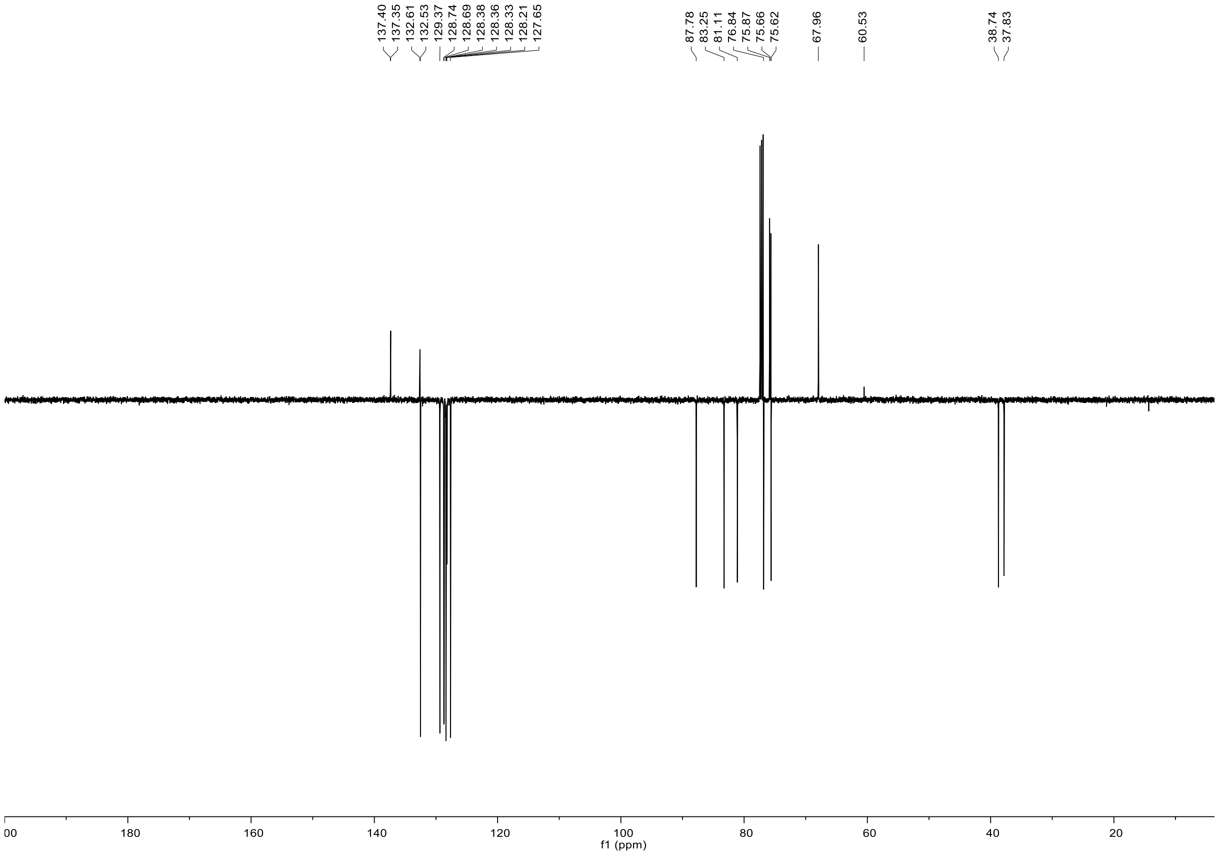

HH-COSY NMR, $CDCl_3$ of **S5**

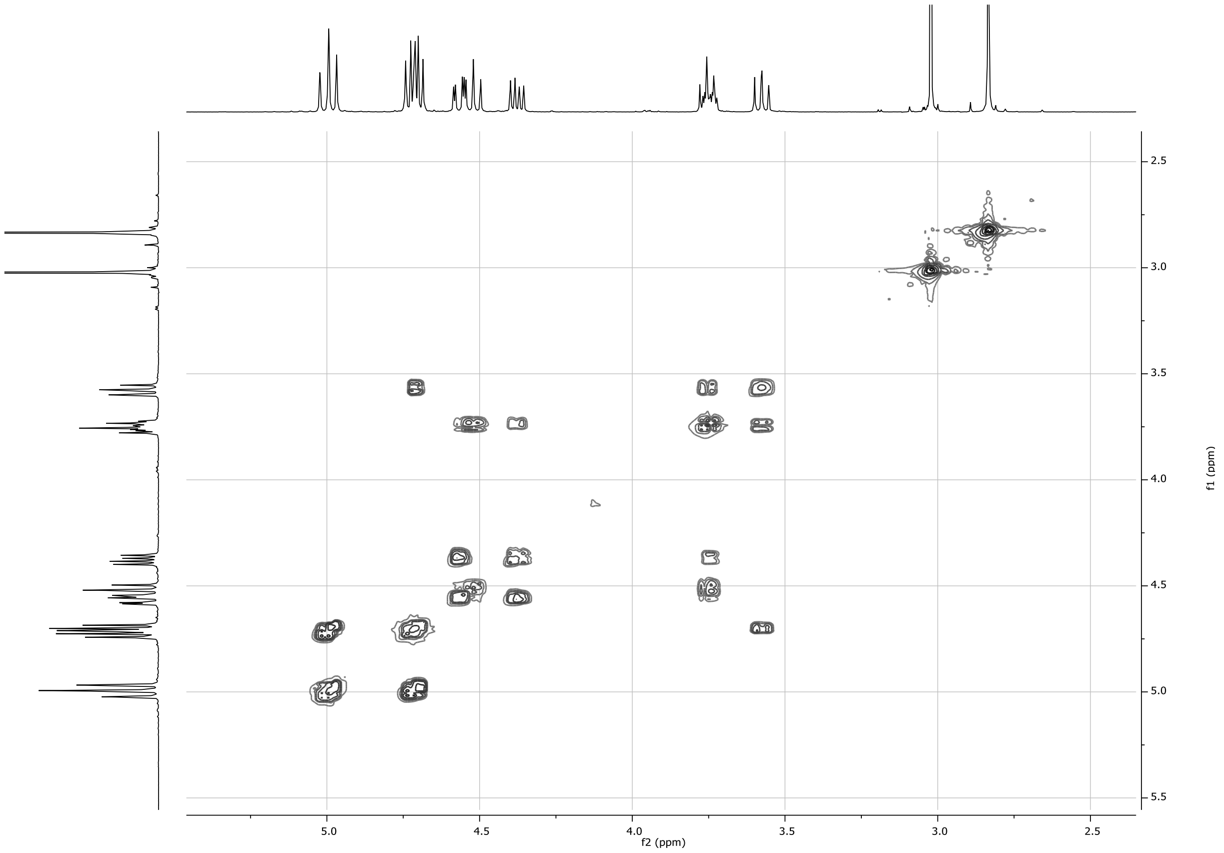

HSQC NMR, CDCl3 of **S5**

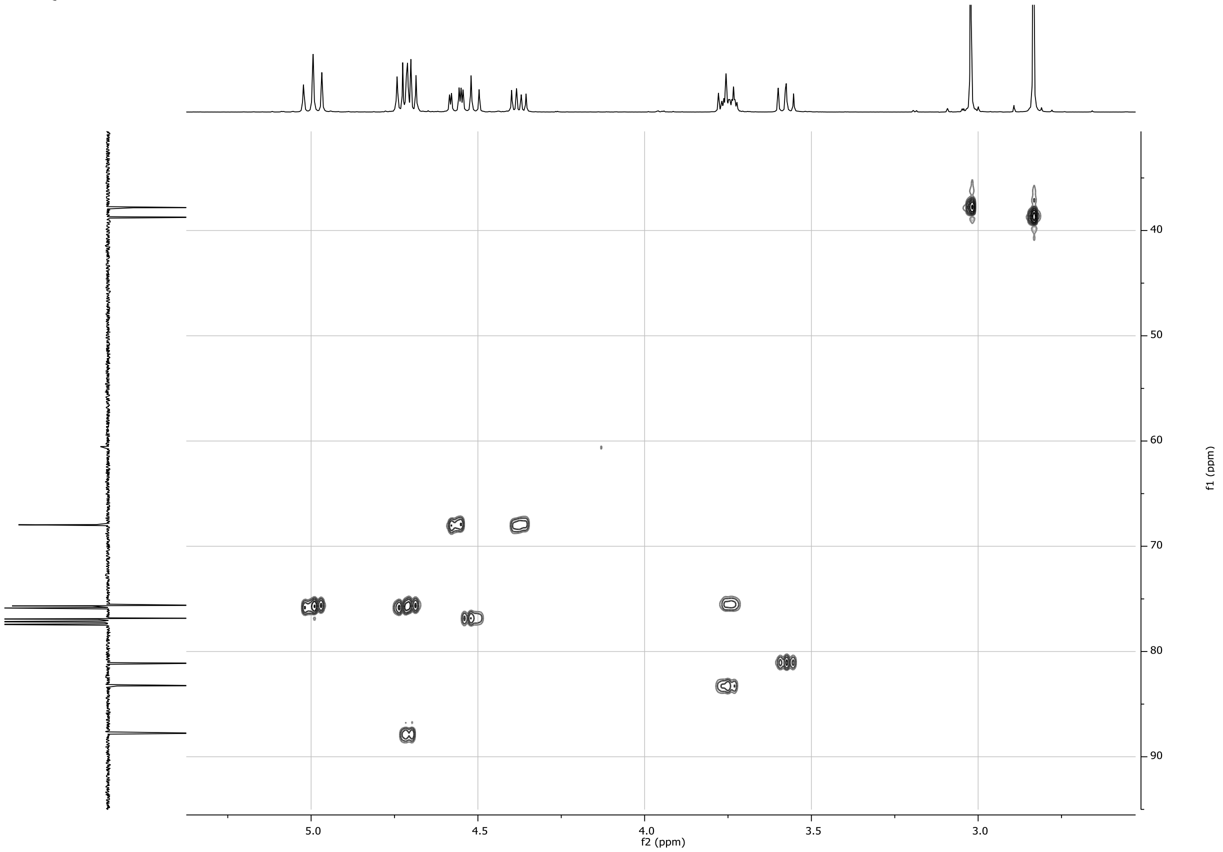

^{1}H NMR, 400 MHz, CDCl3 of **S6**

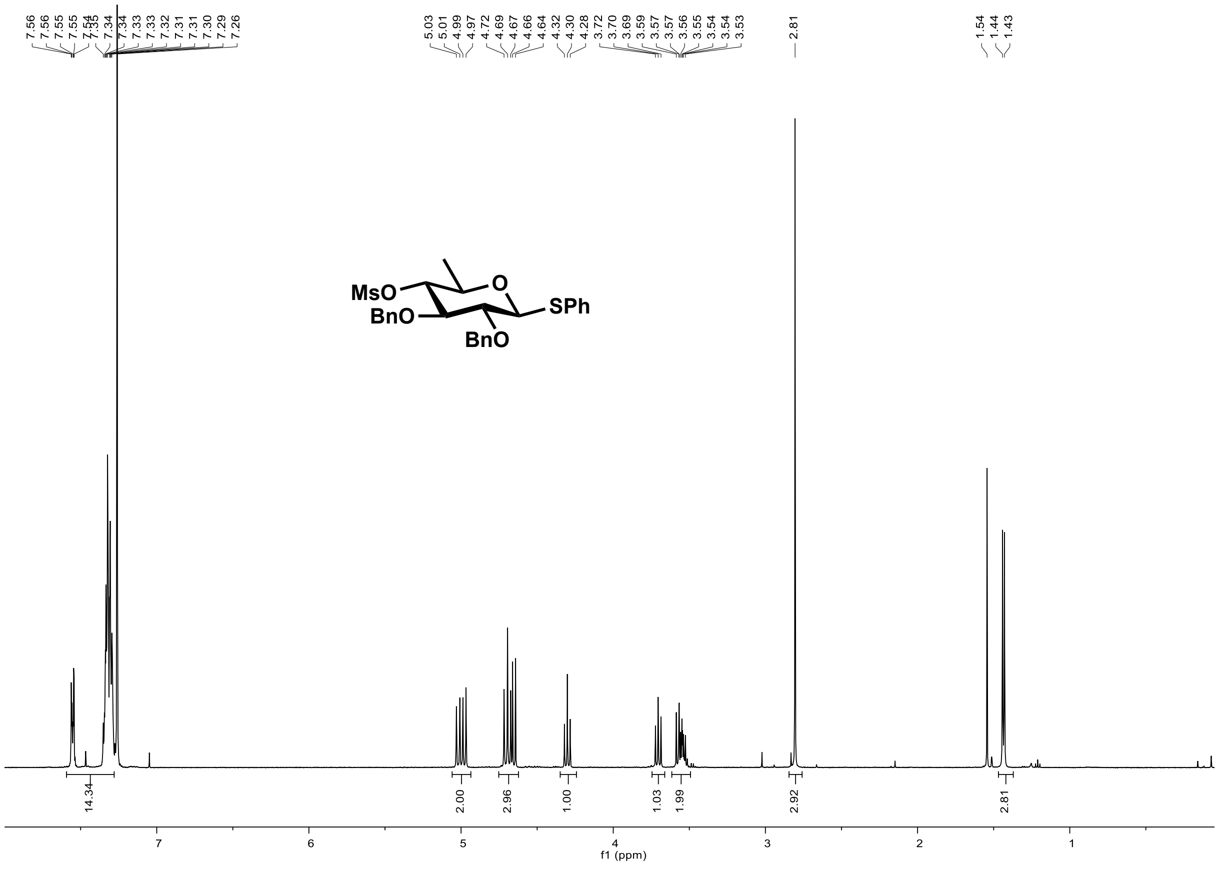

^{13}C NMR, 101 MHz, $CDCl_3$ of **S6**

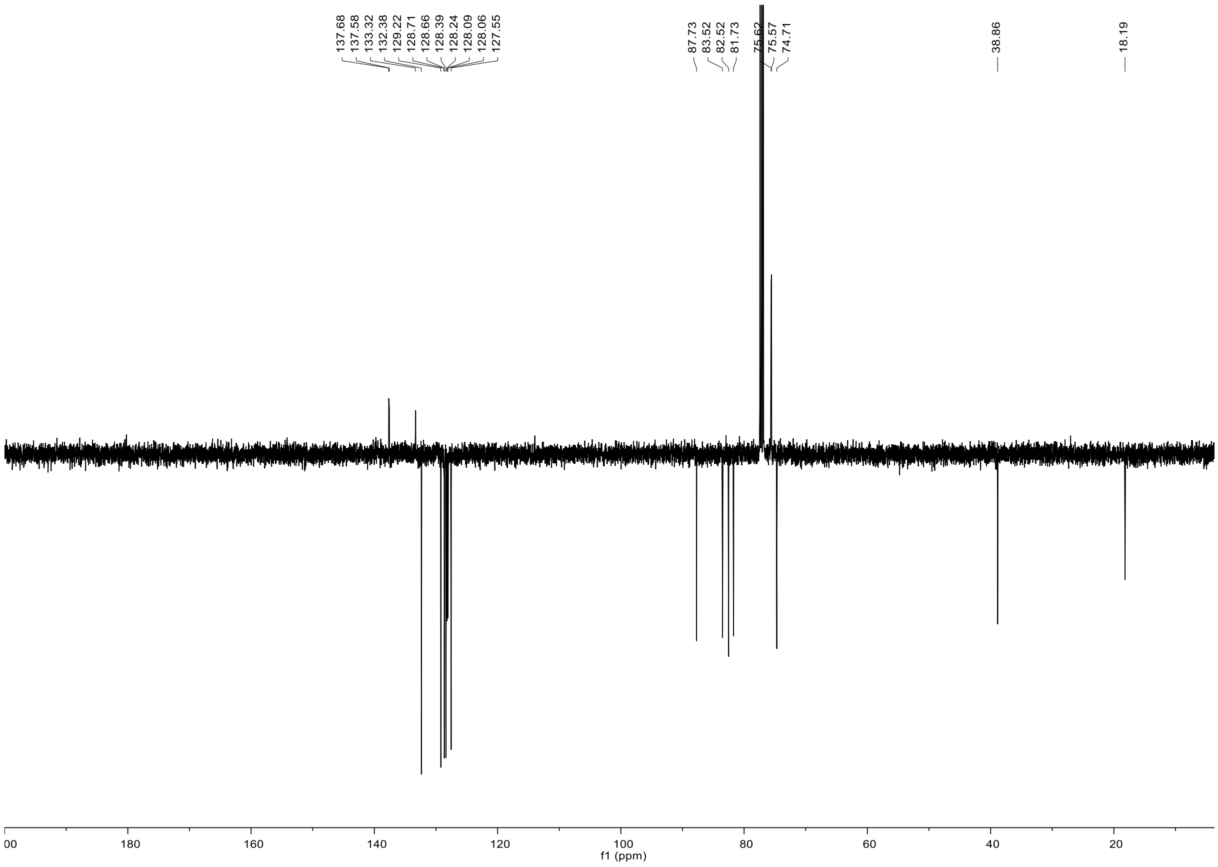

HH-COSY NMR, $CDCl_3$ of **S6**

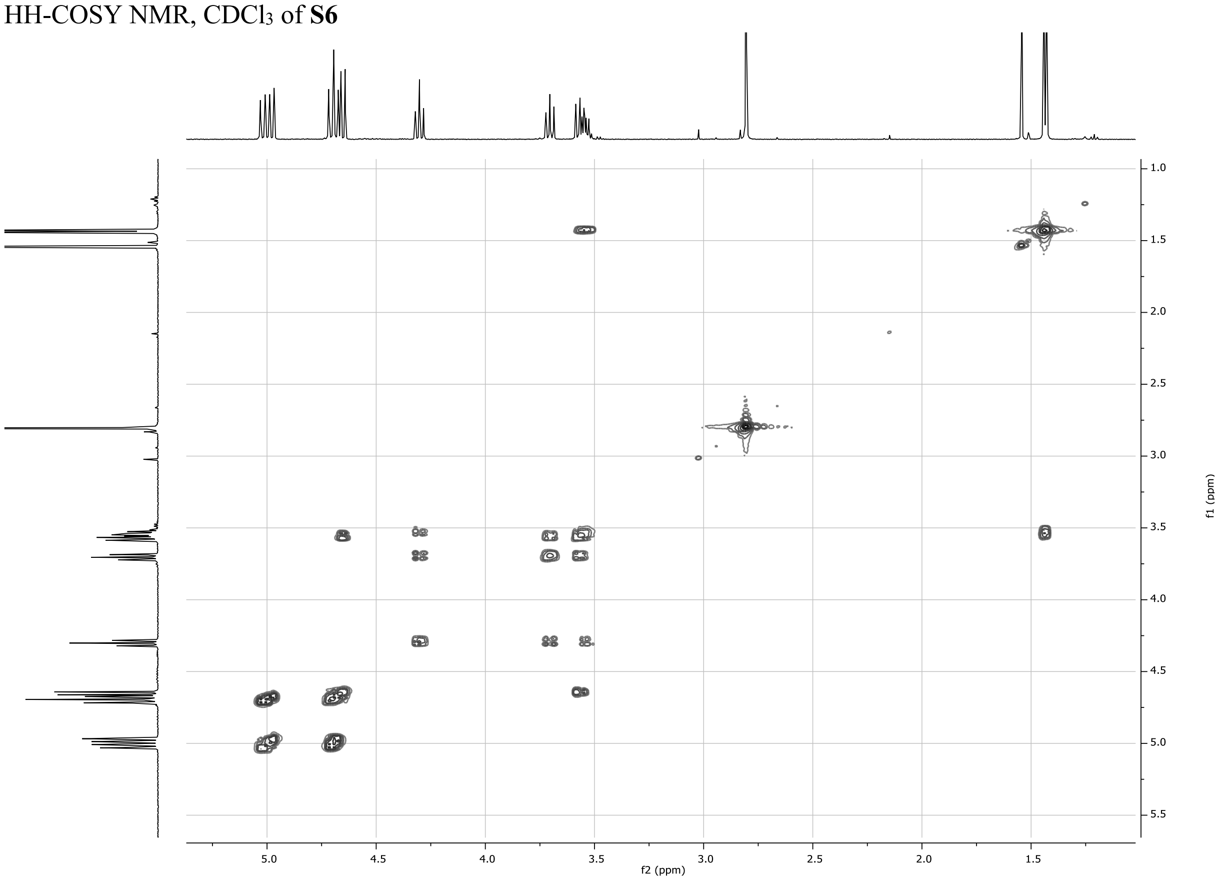

HSQC NMR, $CDCl_3$ of **S6**

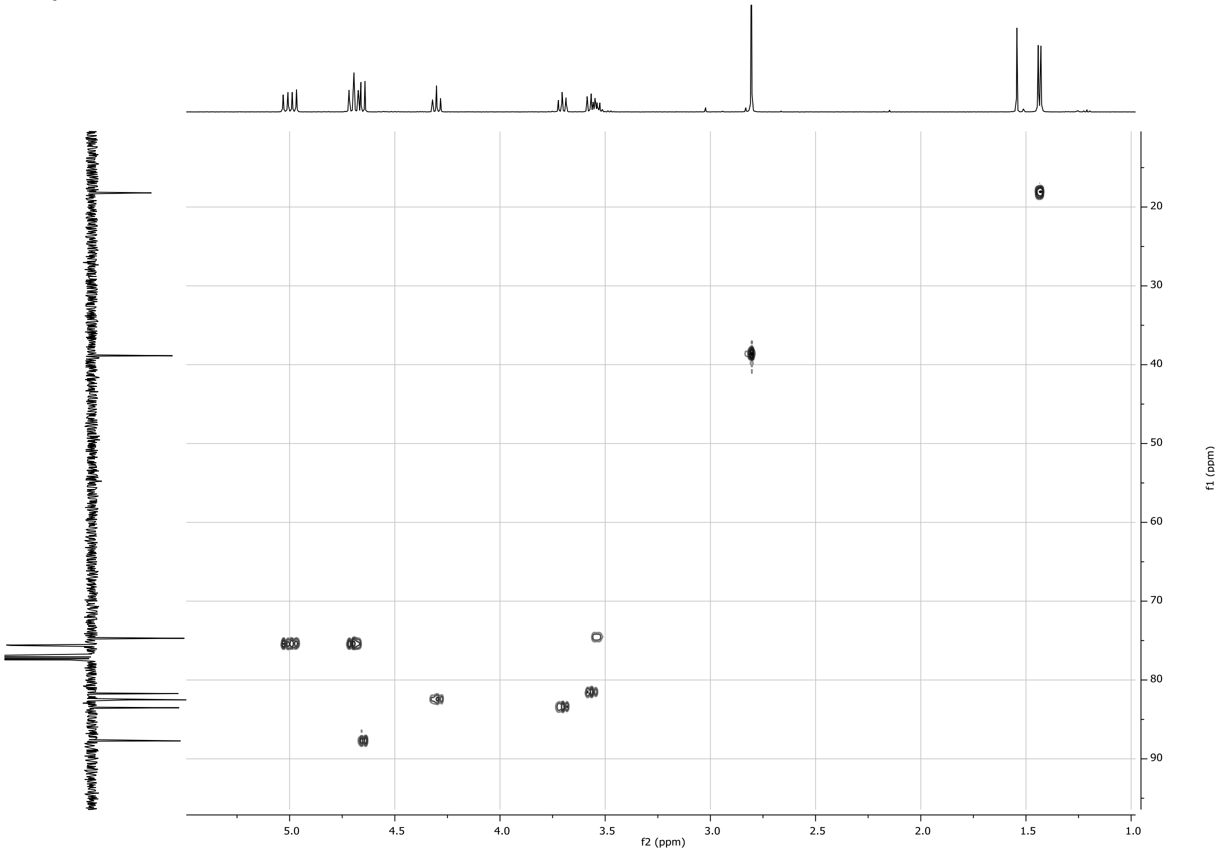

^{1}H NMR, 400 MHz, $CDCl_3$ of **3**

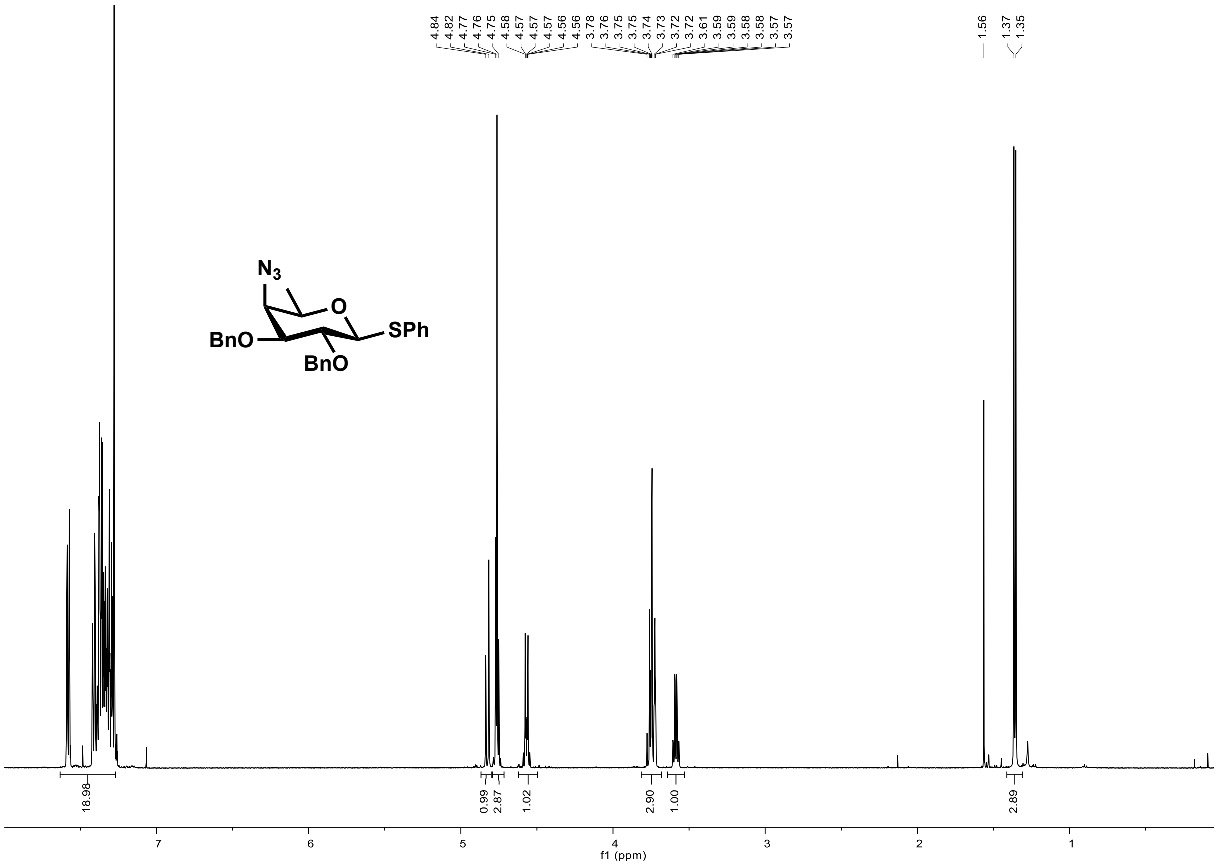

^{13}C NMR, 101 MHz, $CDCl_3$ of **3**

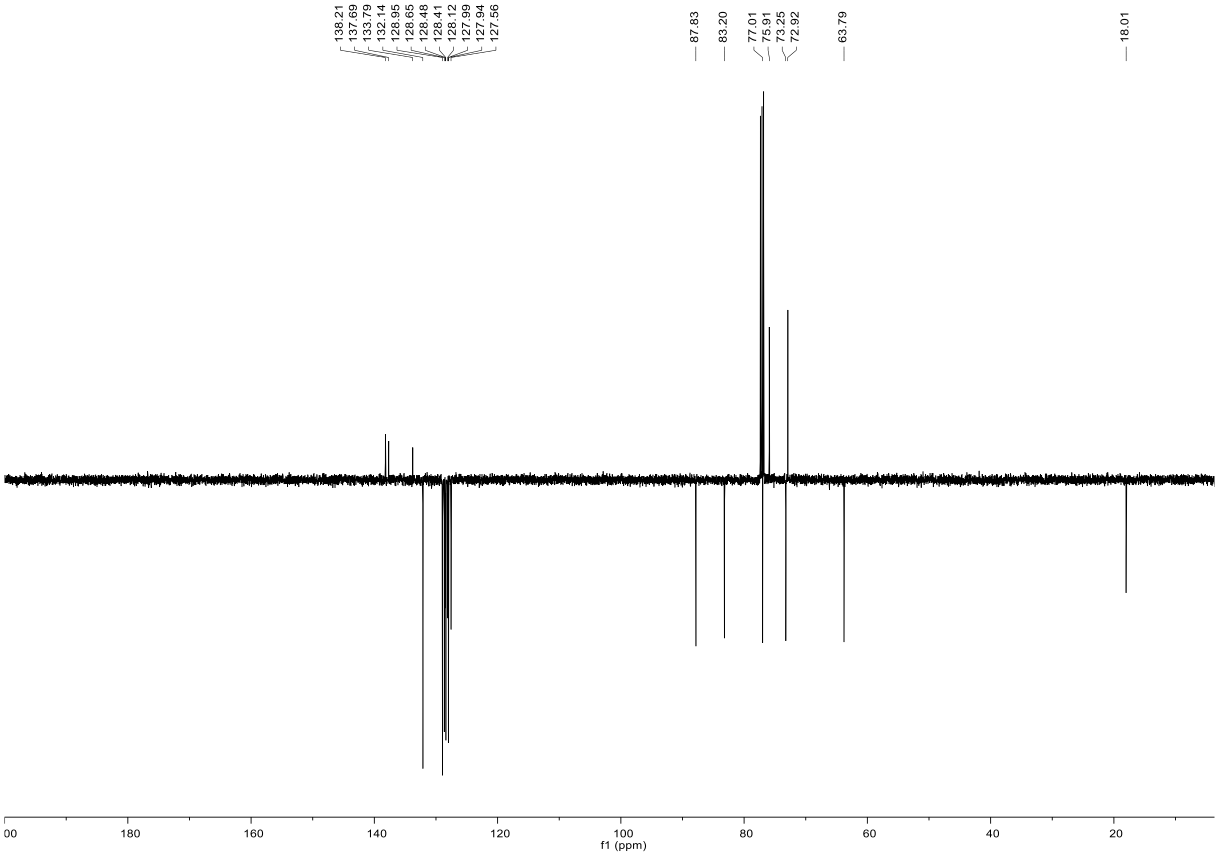

H
H-COSY NMR, $CDCl_3$ of **3**

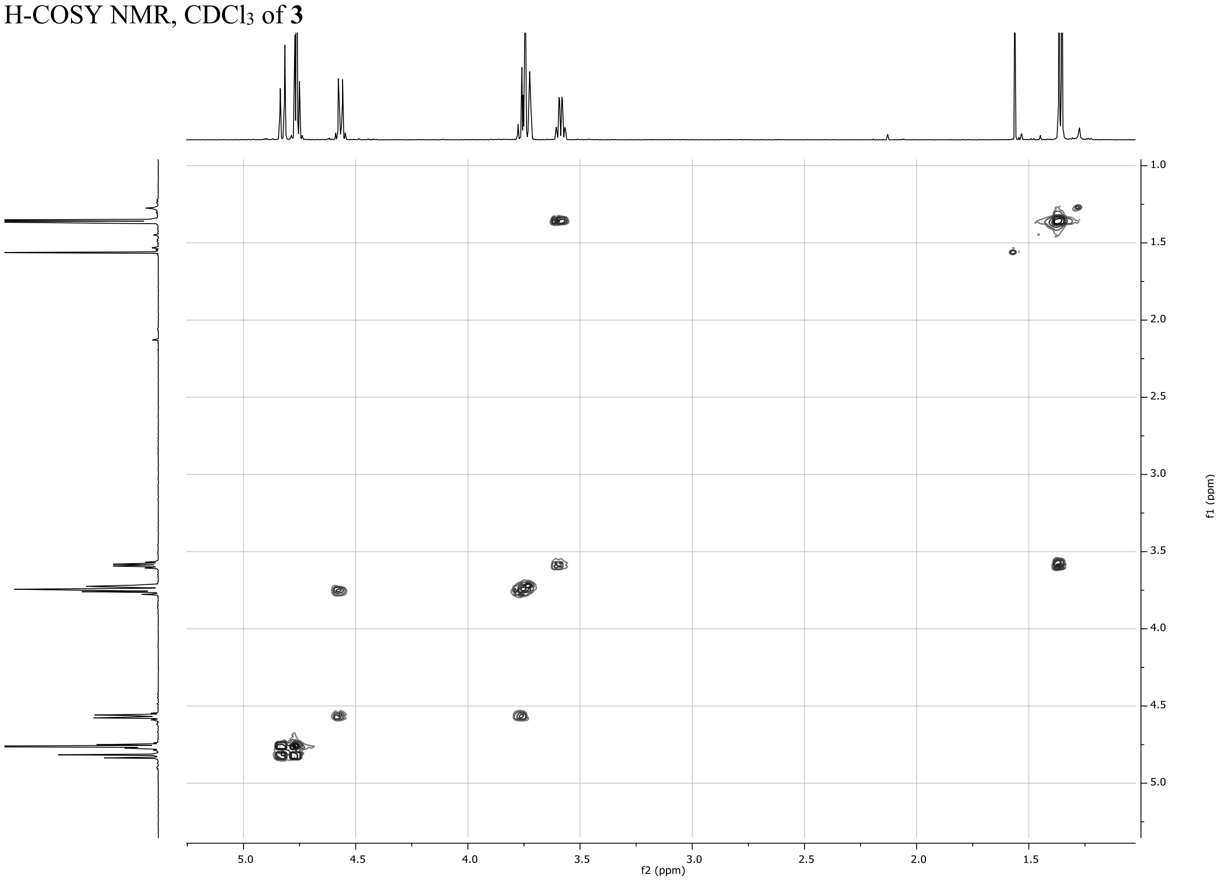

HSQC NMR, $CDCl_3$ of **3**

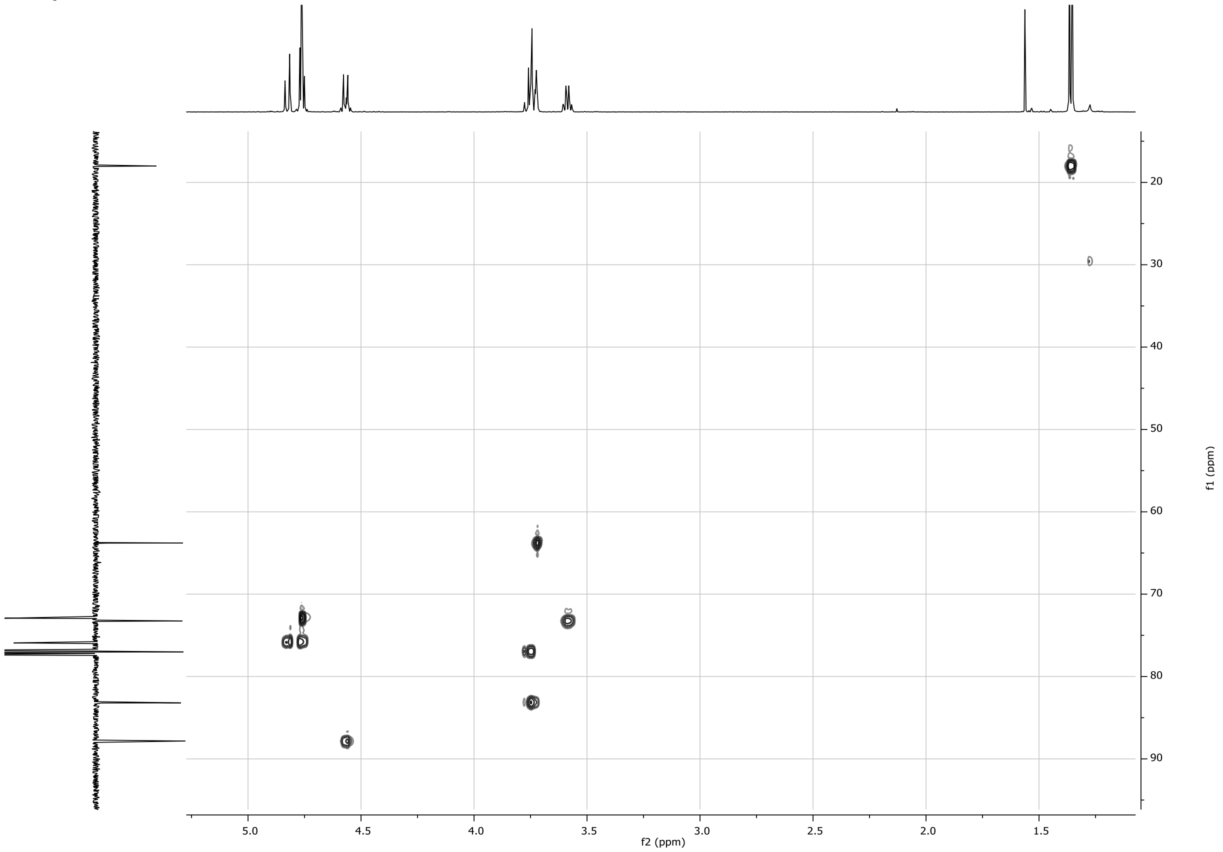

^{1}H NMR, 400 MHz, $CDCl_3$ of **S7**

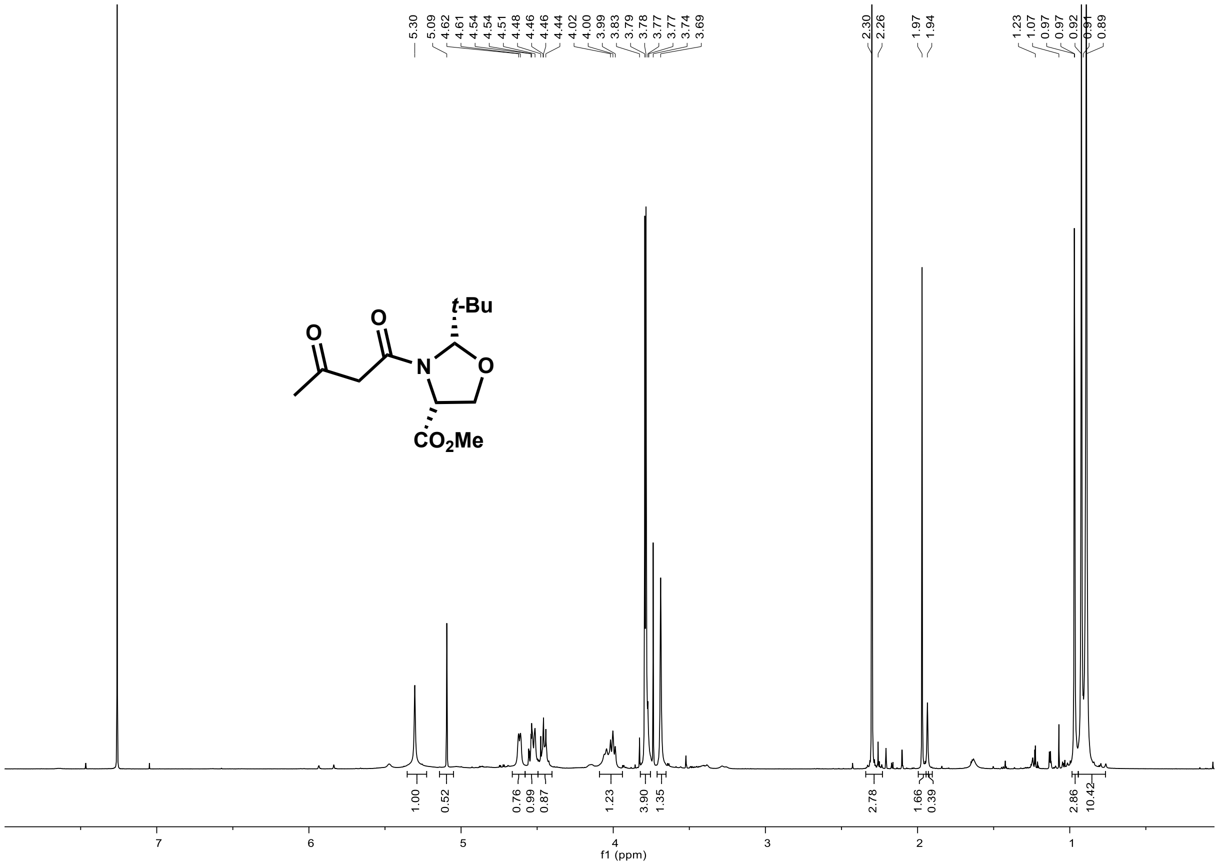

^{13}C NMR, 101 MHz, $CDCl_3$ of **S7**

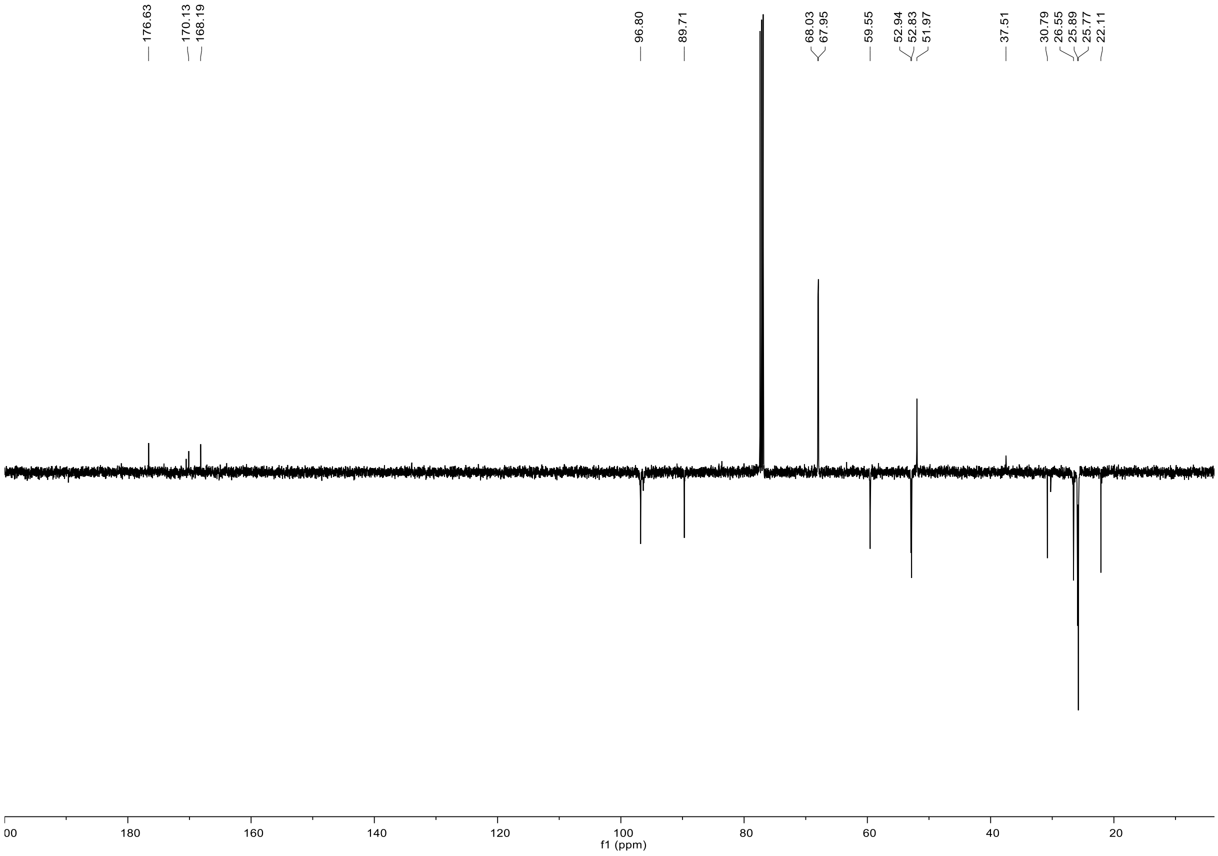

HH-COSY NMR, $CDCl_3$ of **S7**

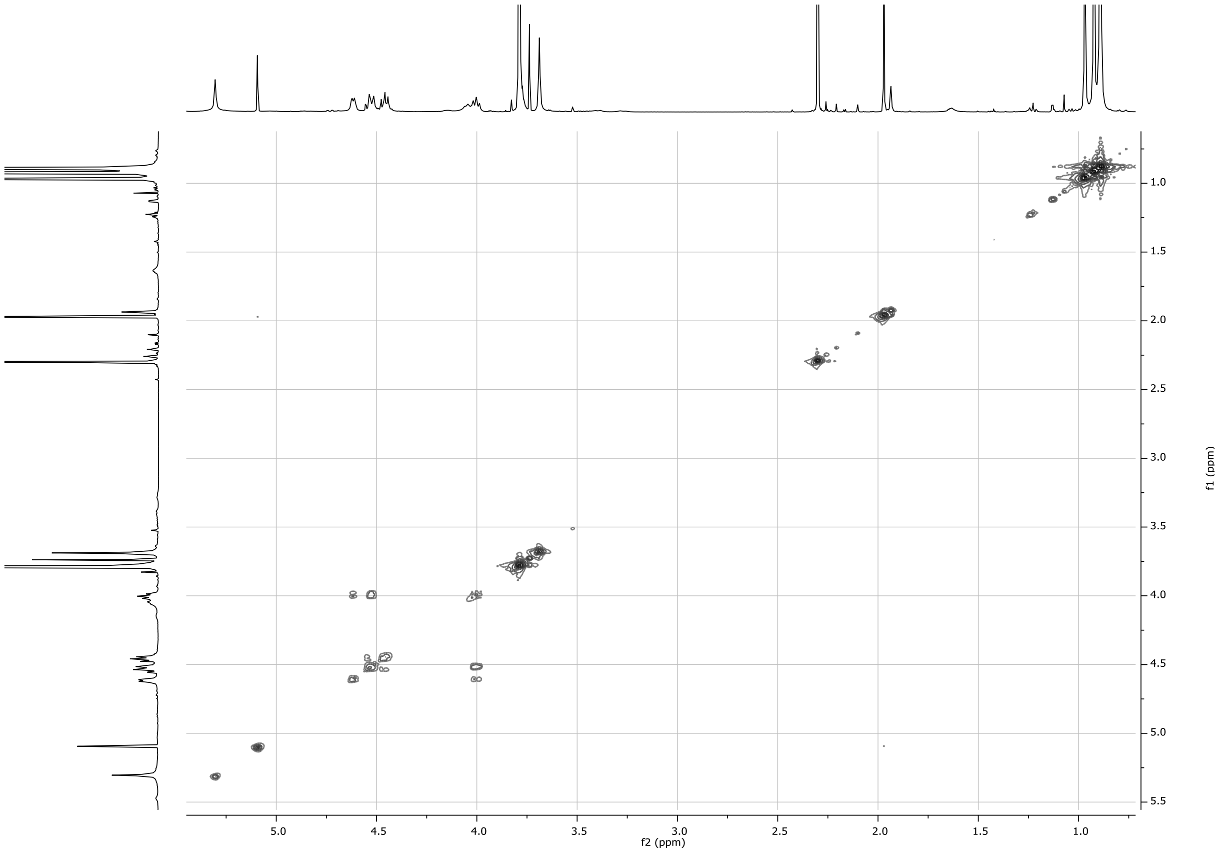

HSQC NMR, $CDCl_3$ of **S7**

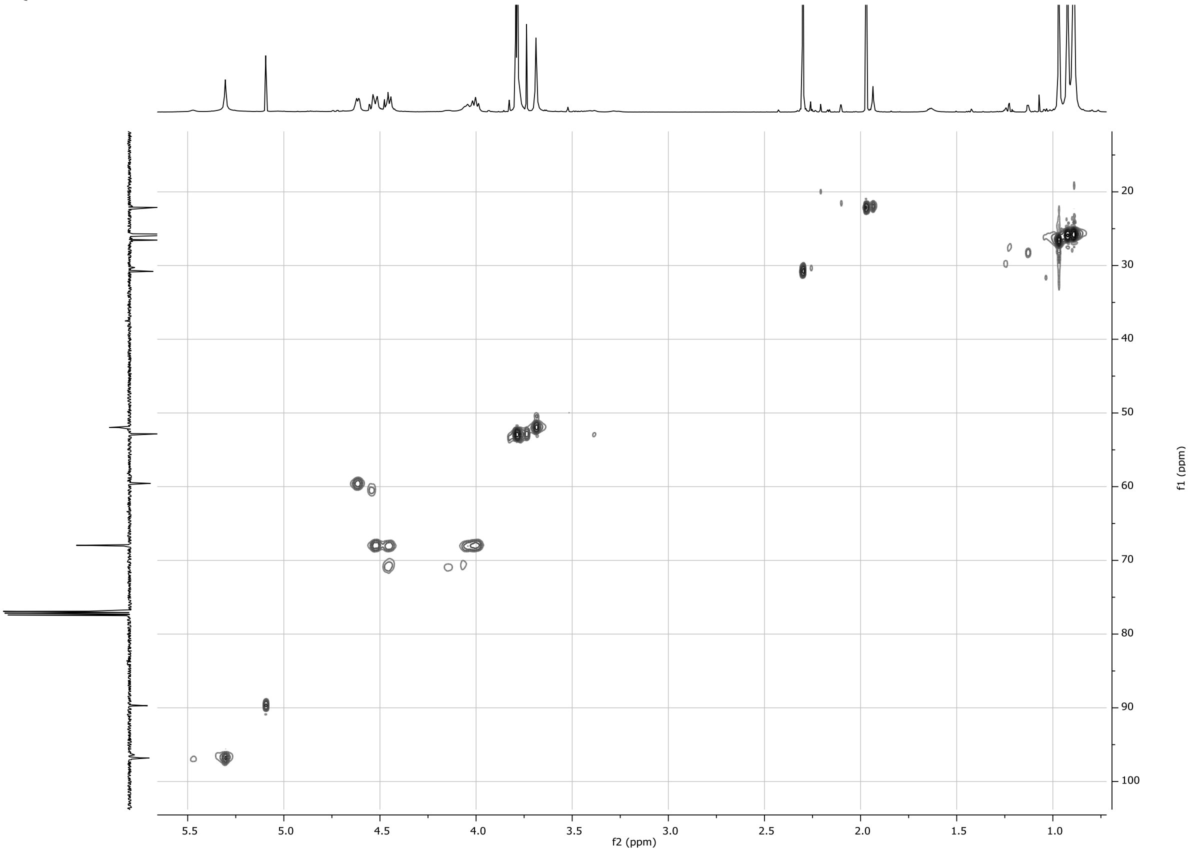

^{1}H NMR, 400 MHz, $CDCl_3$ of **S8**

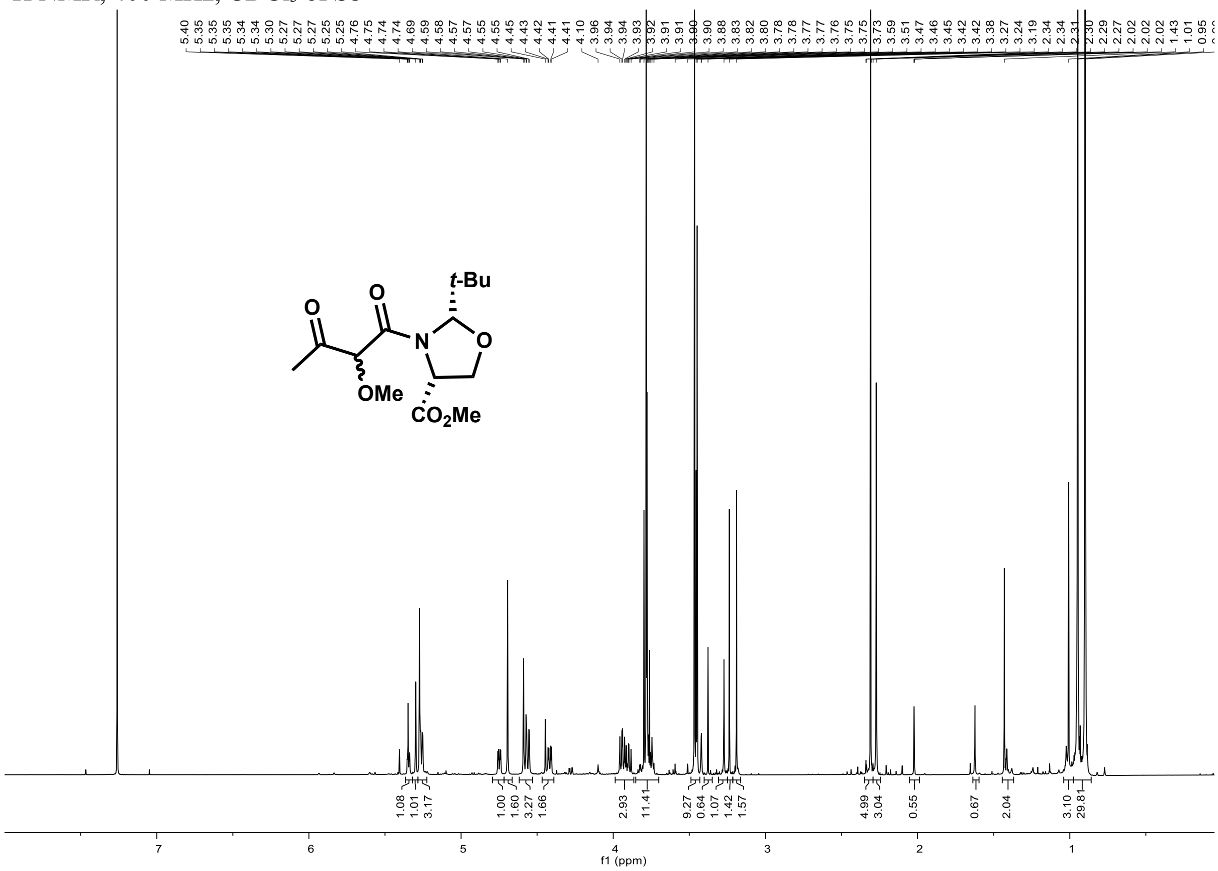

^{13}C NMR, 101 MHz, $CDCl_3$ of **S8**

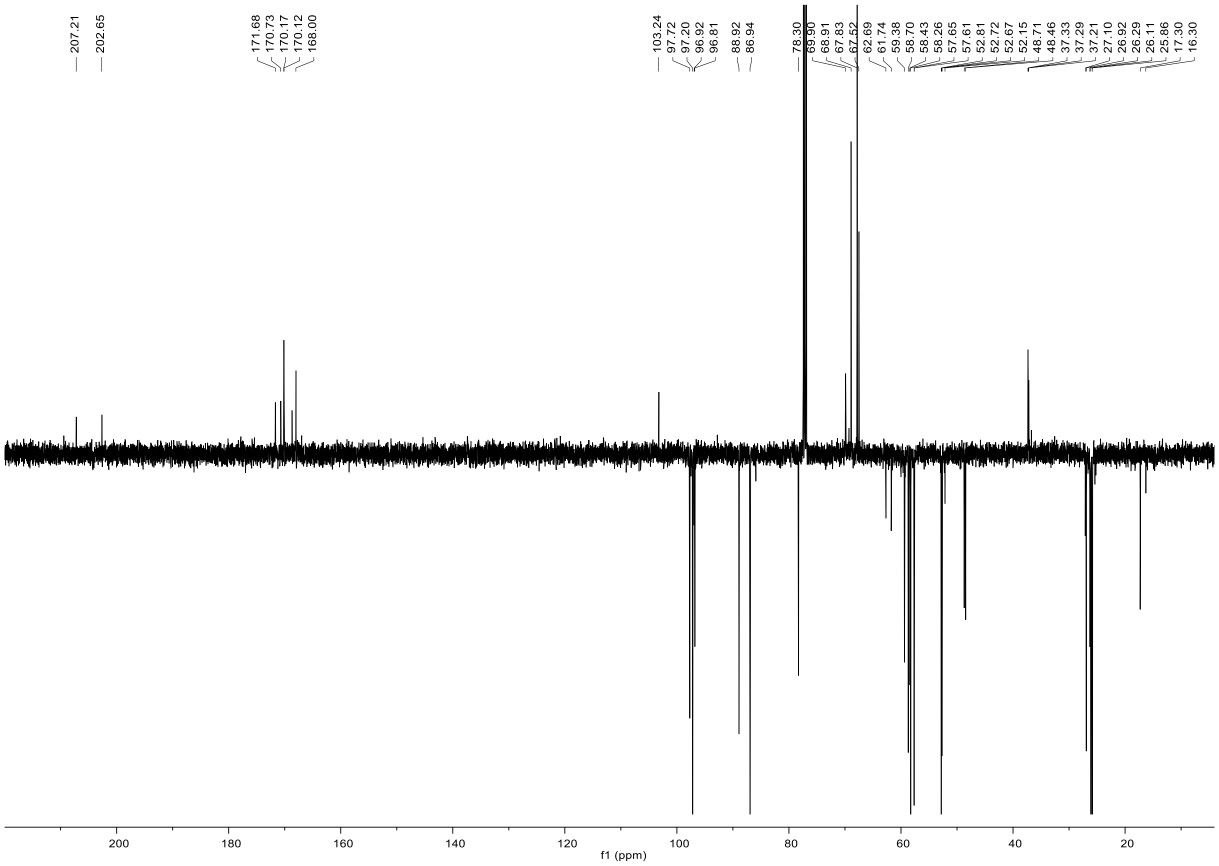

HH-COSY NMR, $CDCl_3$ of **S8**

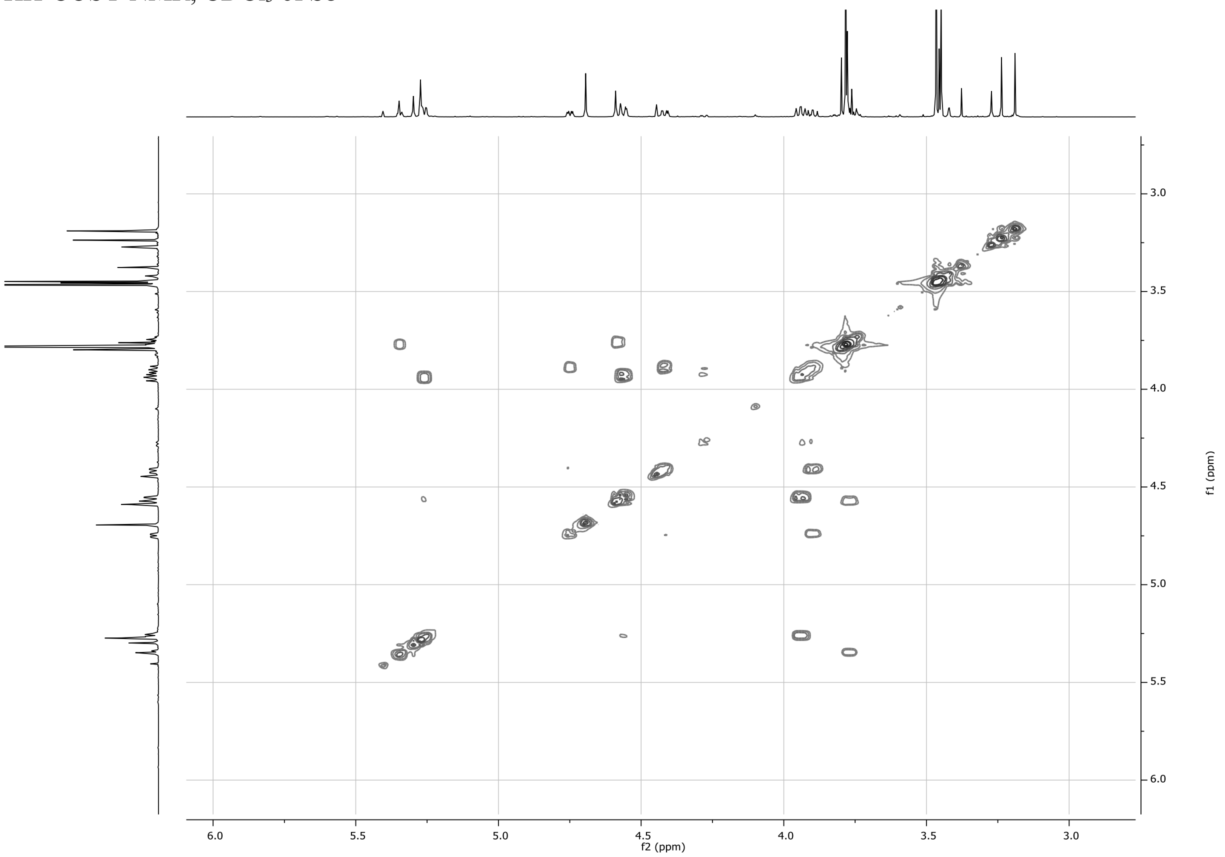

HSQC NMR, $CDCl_3$ of **S8**

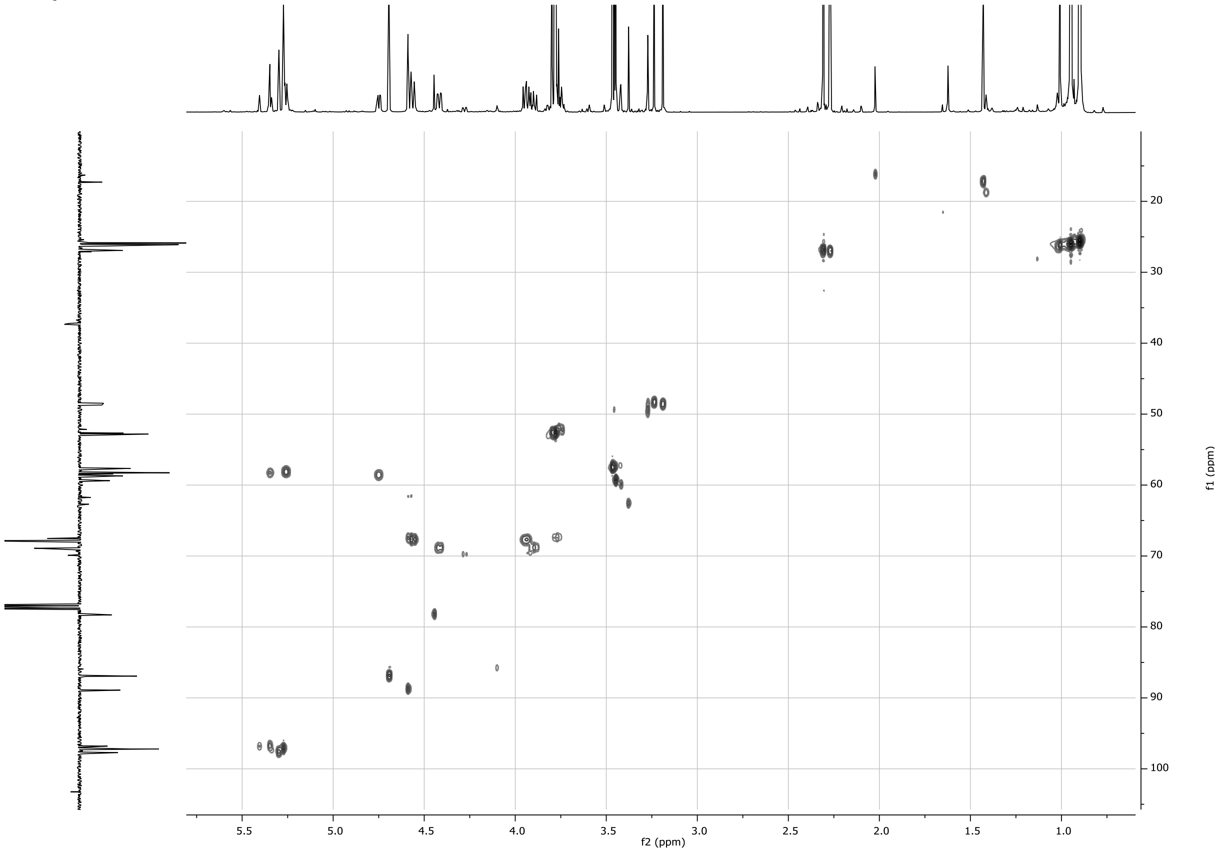

^{1}H NMR, 400 MHz, $CDCl_3$ of **S9**

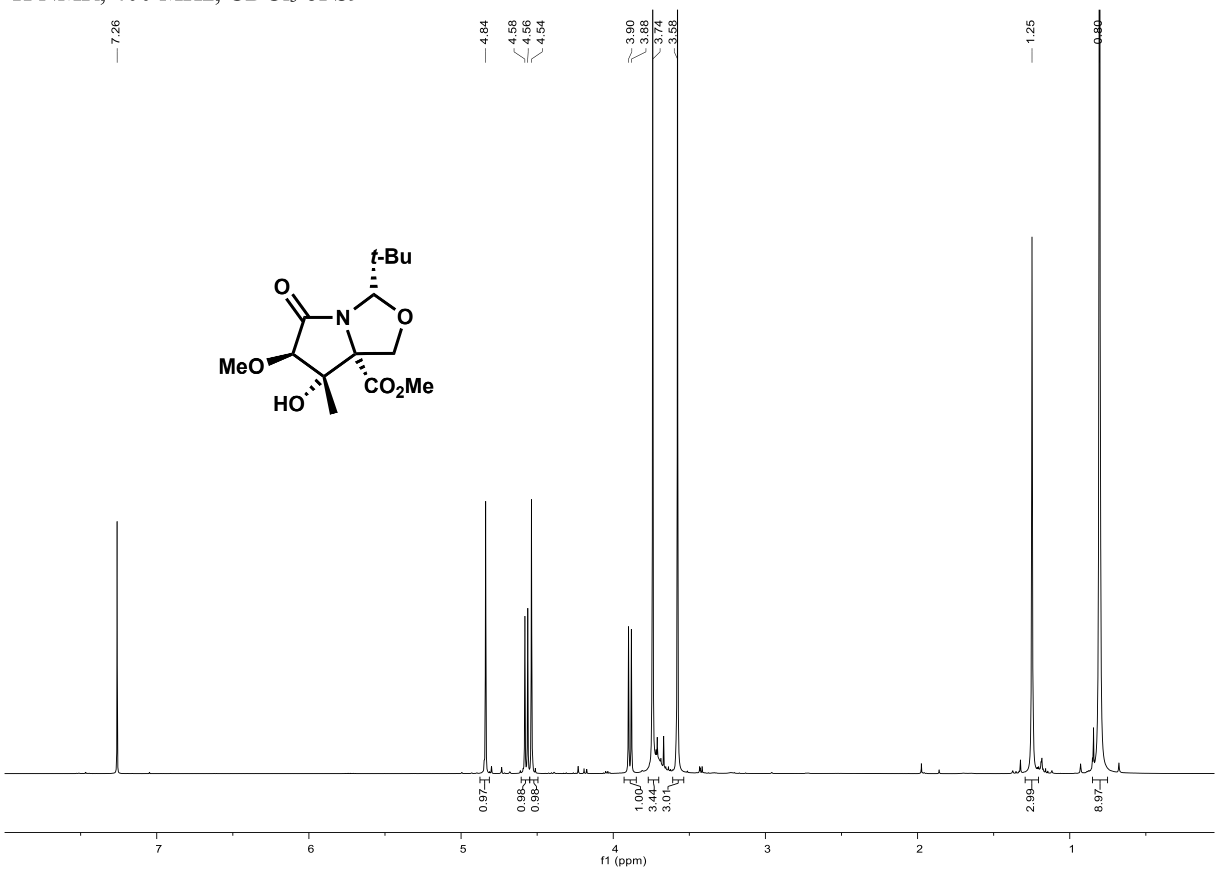

^{13}C NMR, 101 MHz, $CDCl_3$ of **S9**

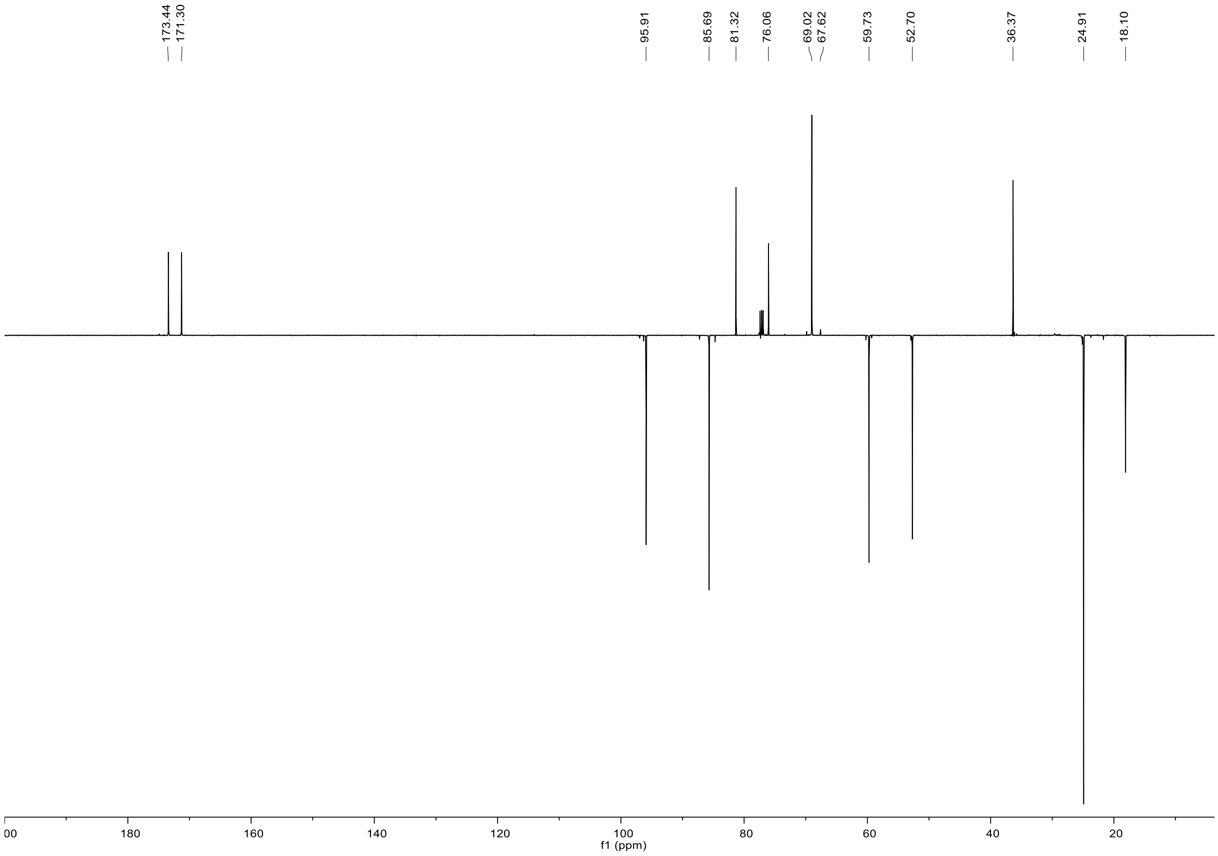

HH-COSY NMR, $CDCl_3$ of **S9**

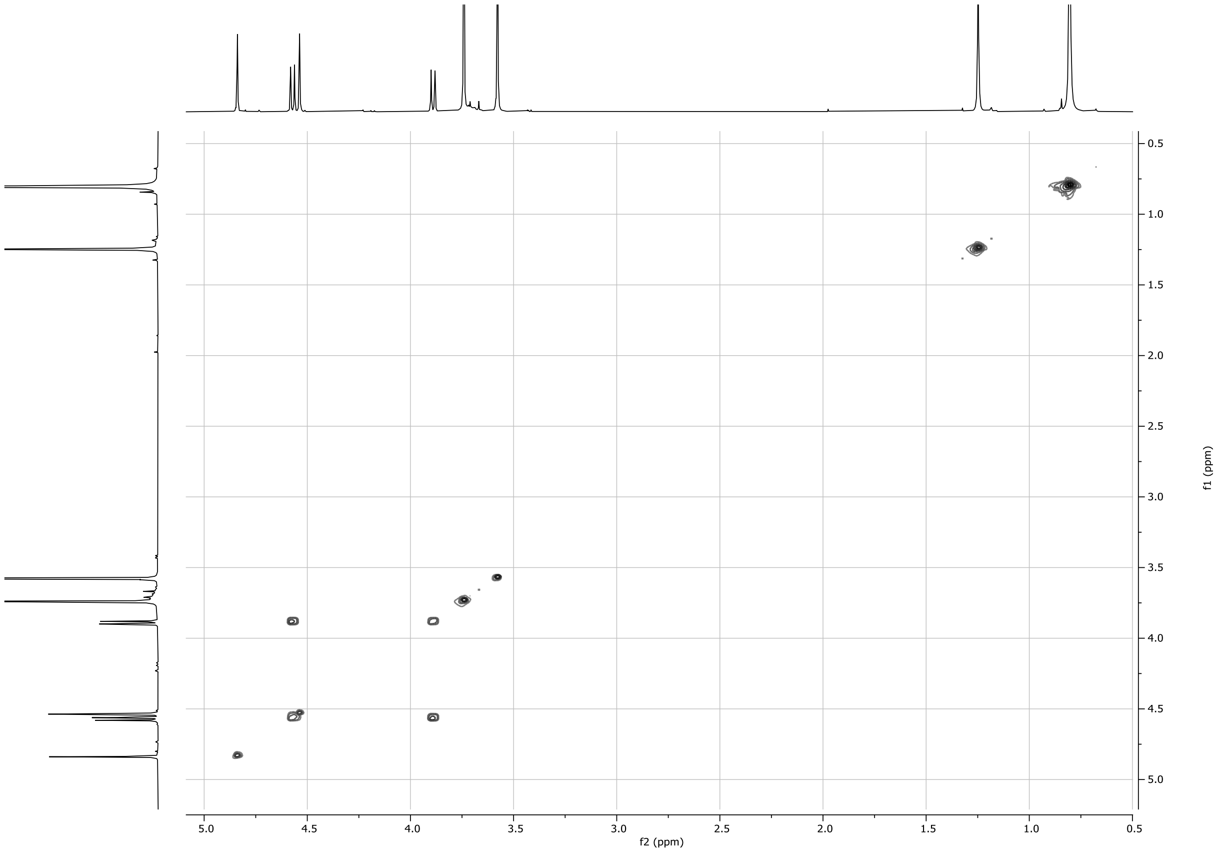

HSQC NMR, $CDCl_3$ of **S9**

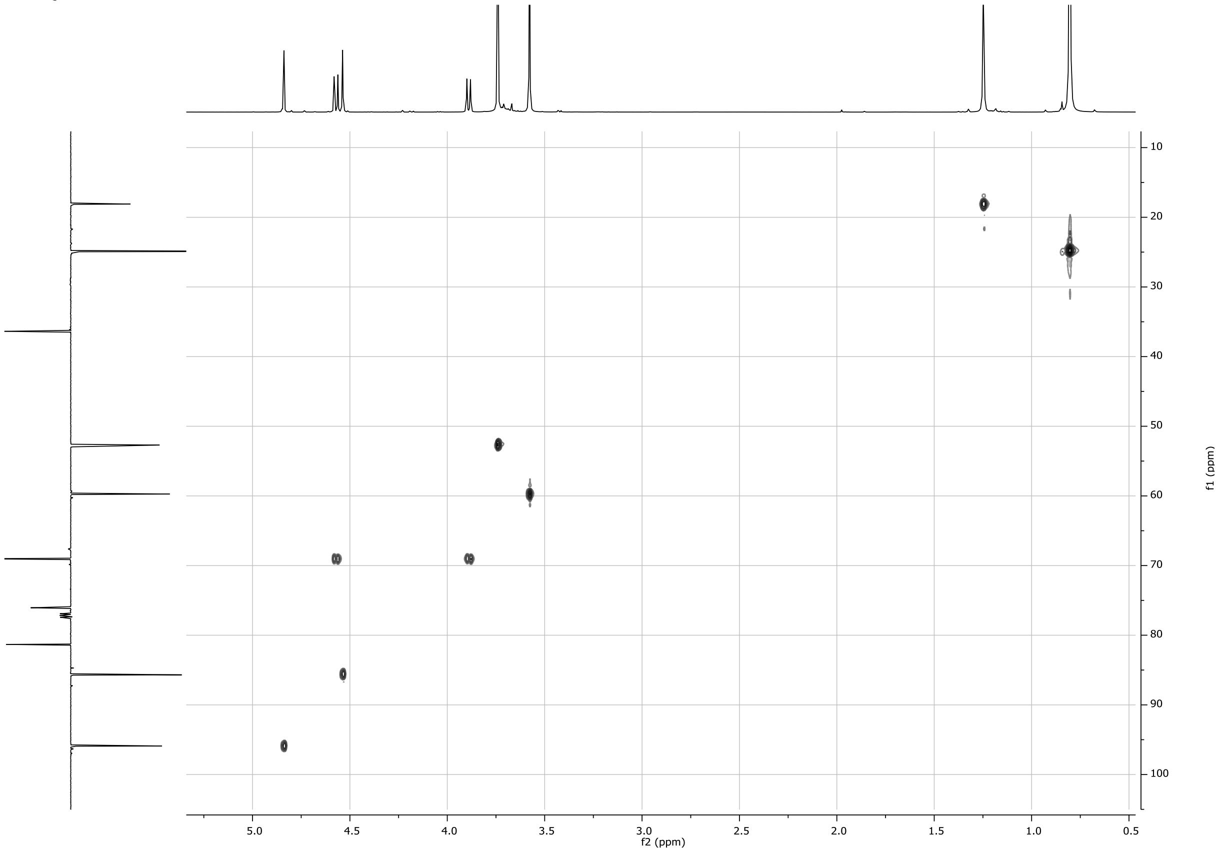

^{1}H NMR, 500 MHz, $CDCl_3$ of **S10**

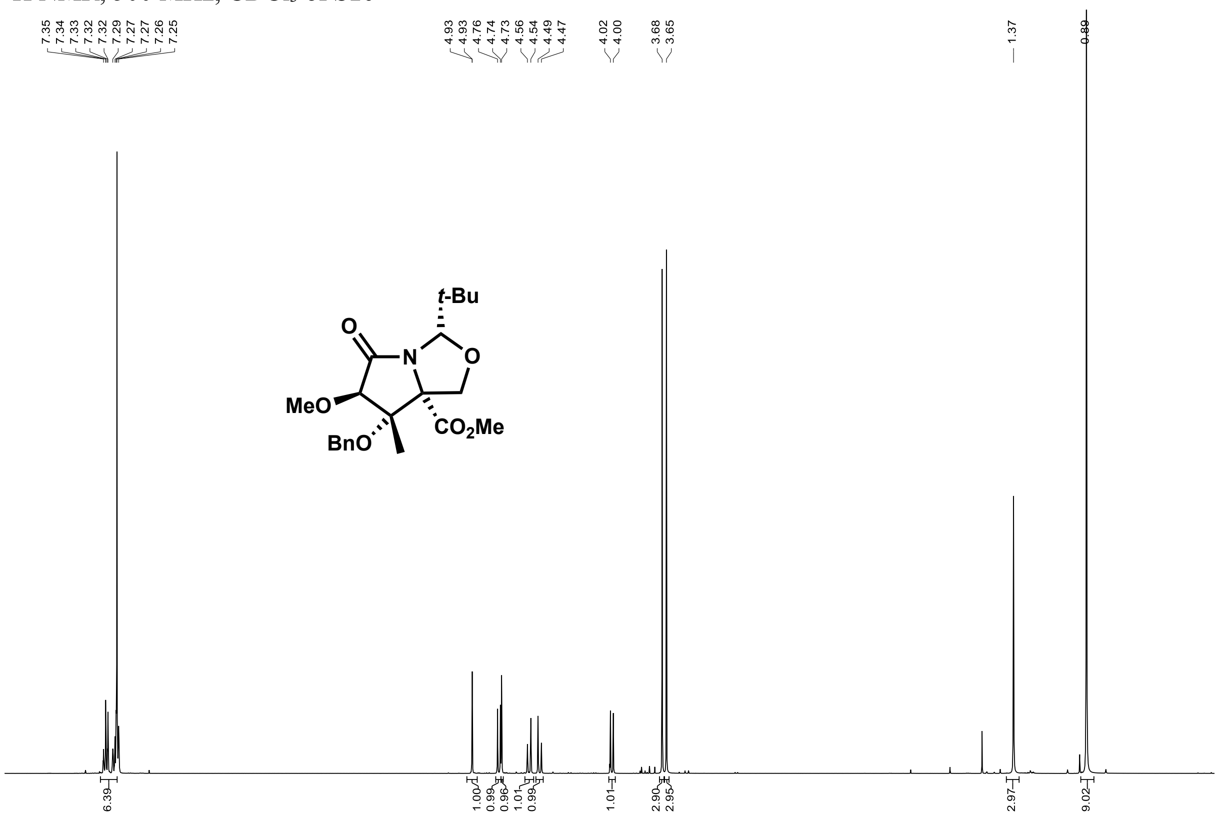

^{13}C NMR, 126 MHz, $CDCl_3$ of **S10**

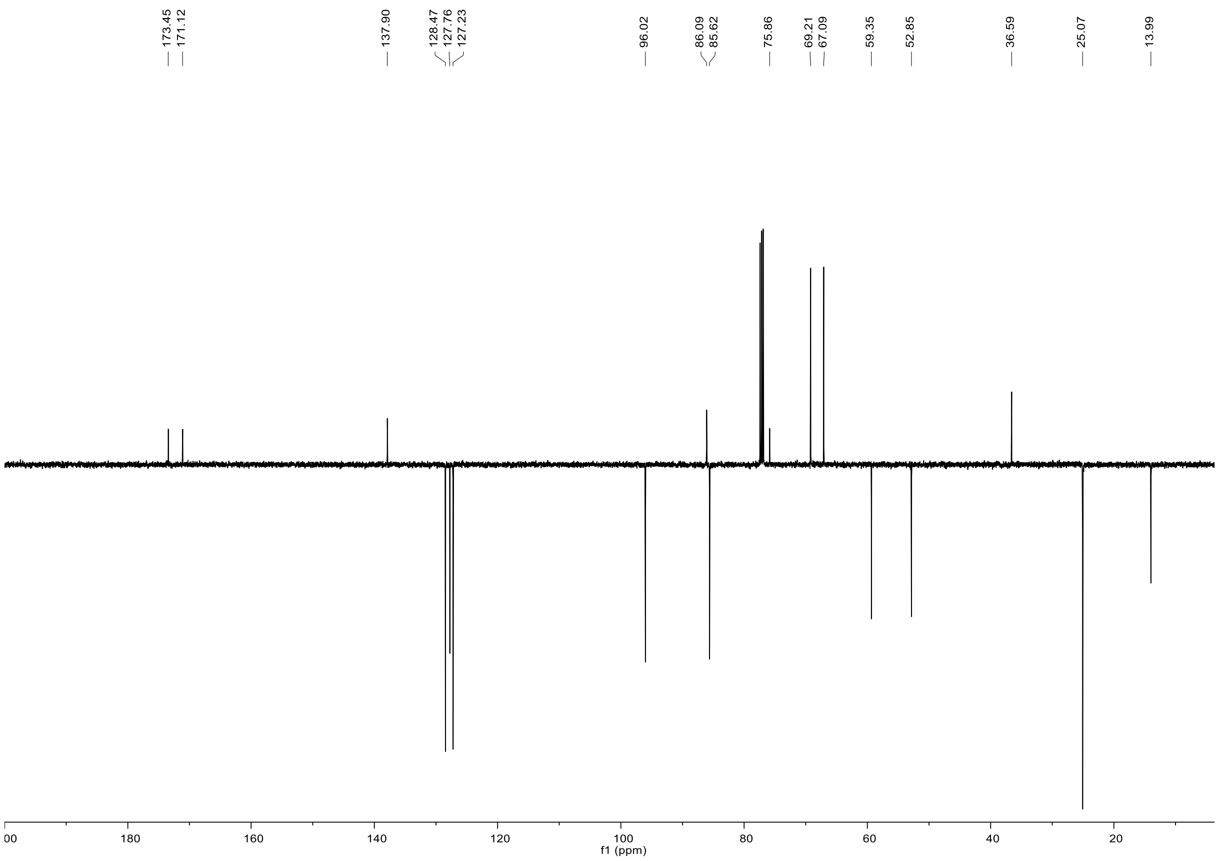

HH-COSY NMR, $CDCl_3$ of **S10**

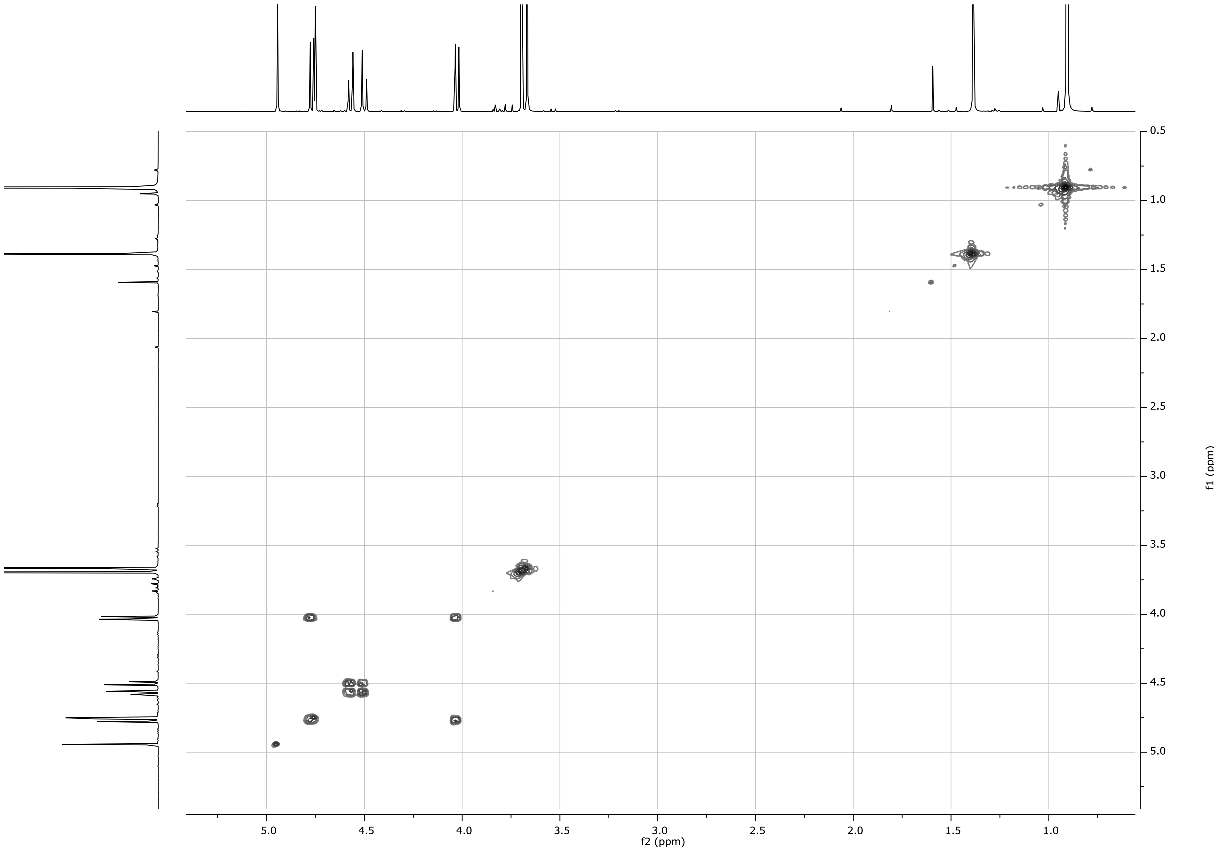

HSQC NMR, $CDCl_3$ of **S10**

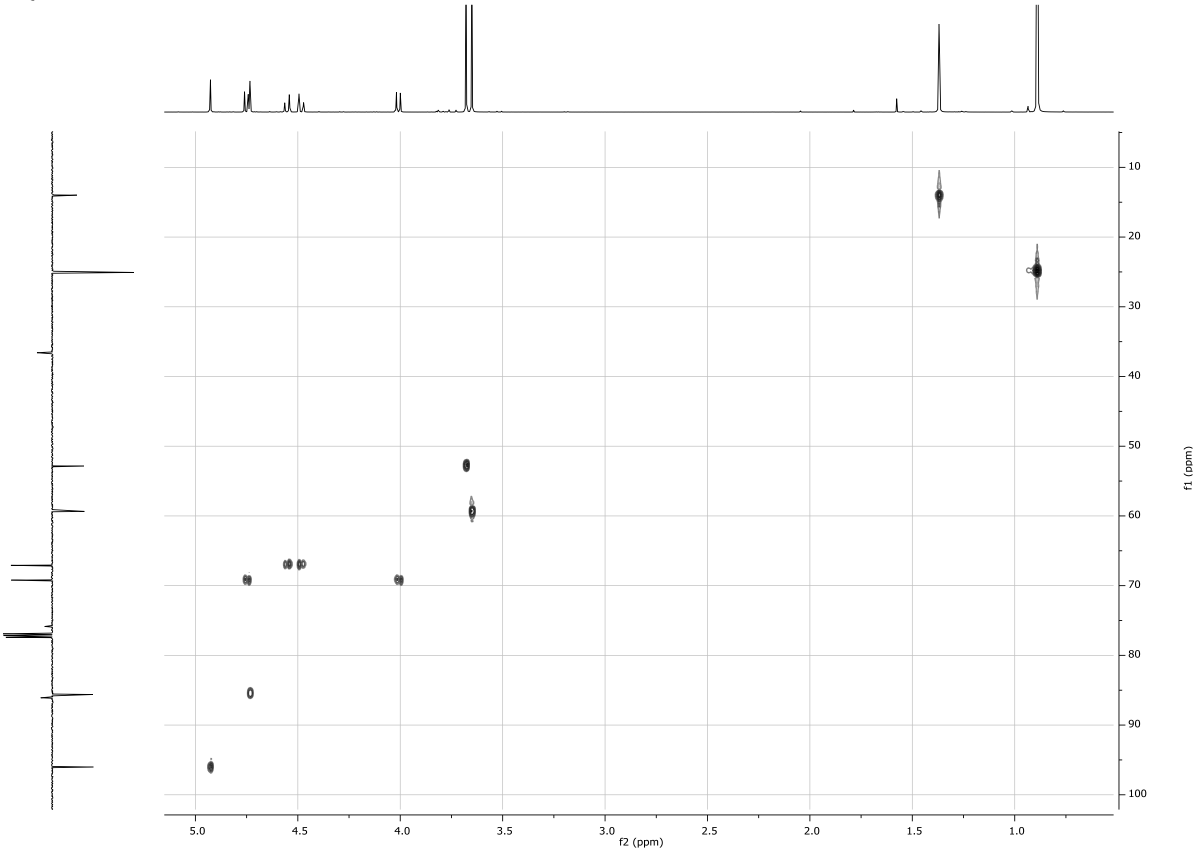

^1H NMR, 400 MHz, $CDCl_3$ of **S11**

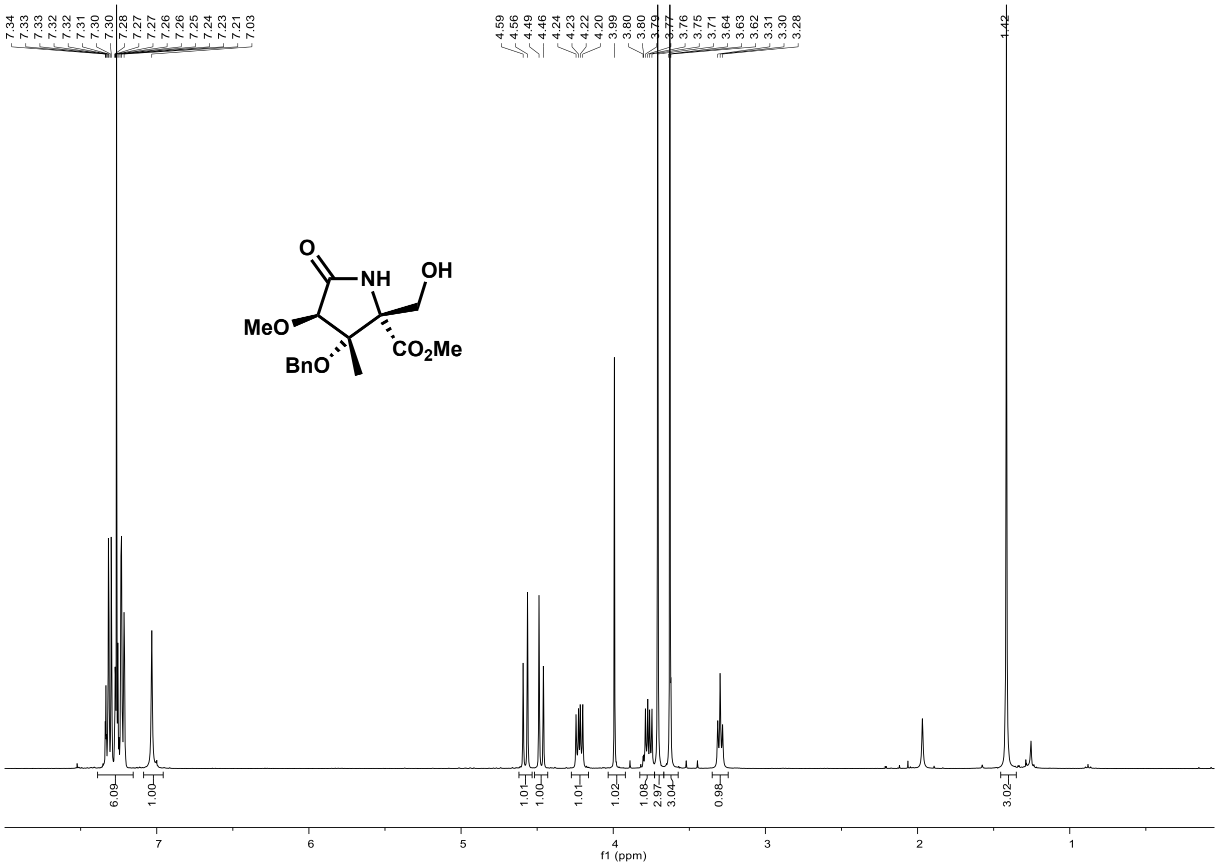

^{13}C NMR, 101 MHz, $CDCl_3$ of **S11**

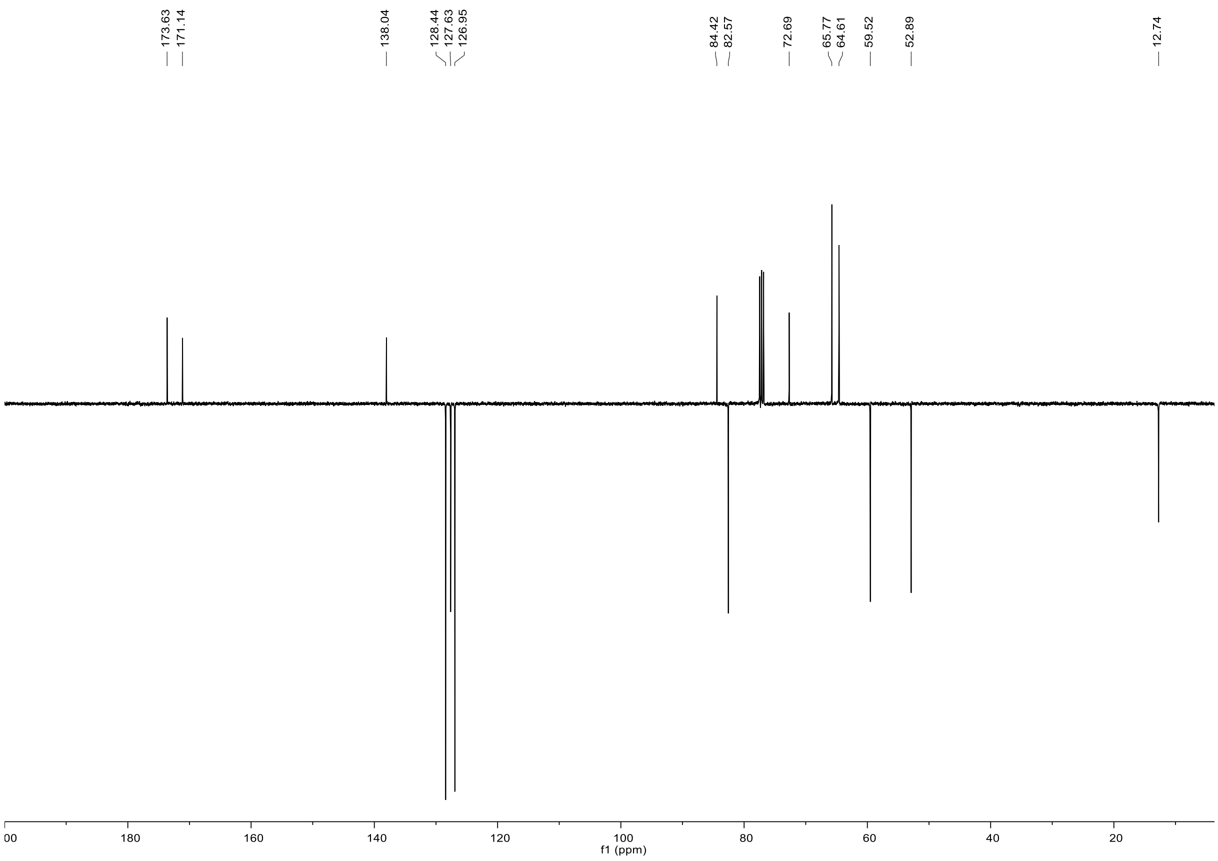

HH-COSY NMR, $CDCl_3$ of **S11**

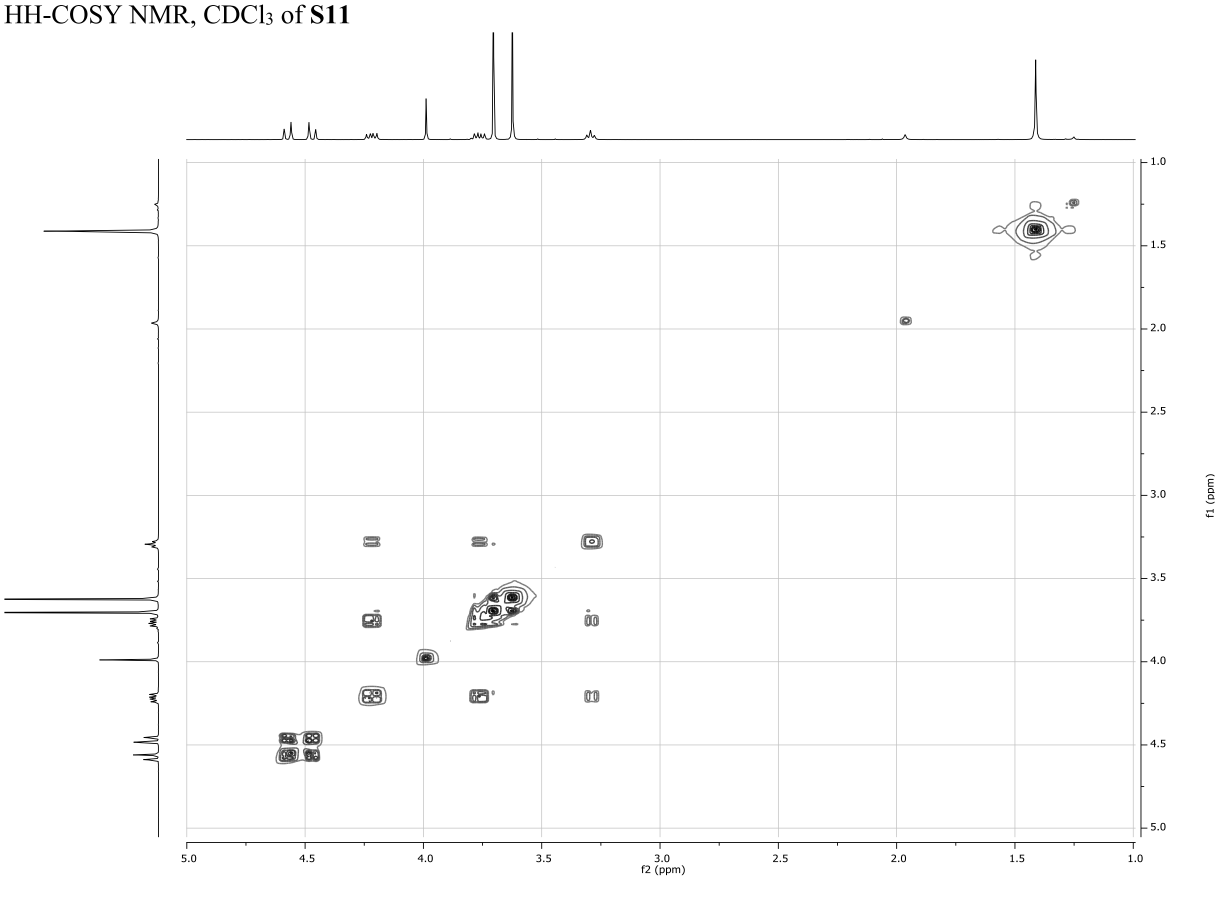

HSQC NMR, $CDCl_3$ of **S11**

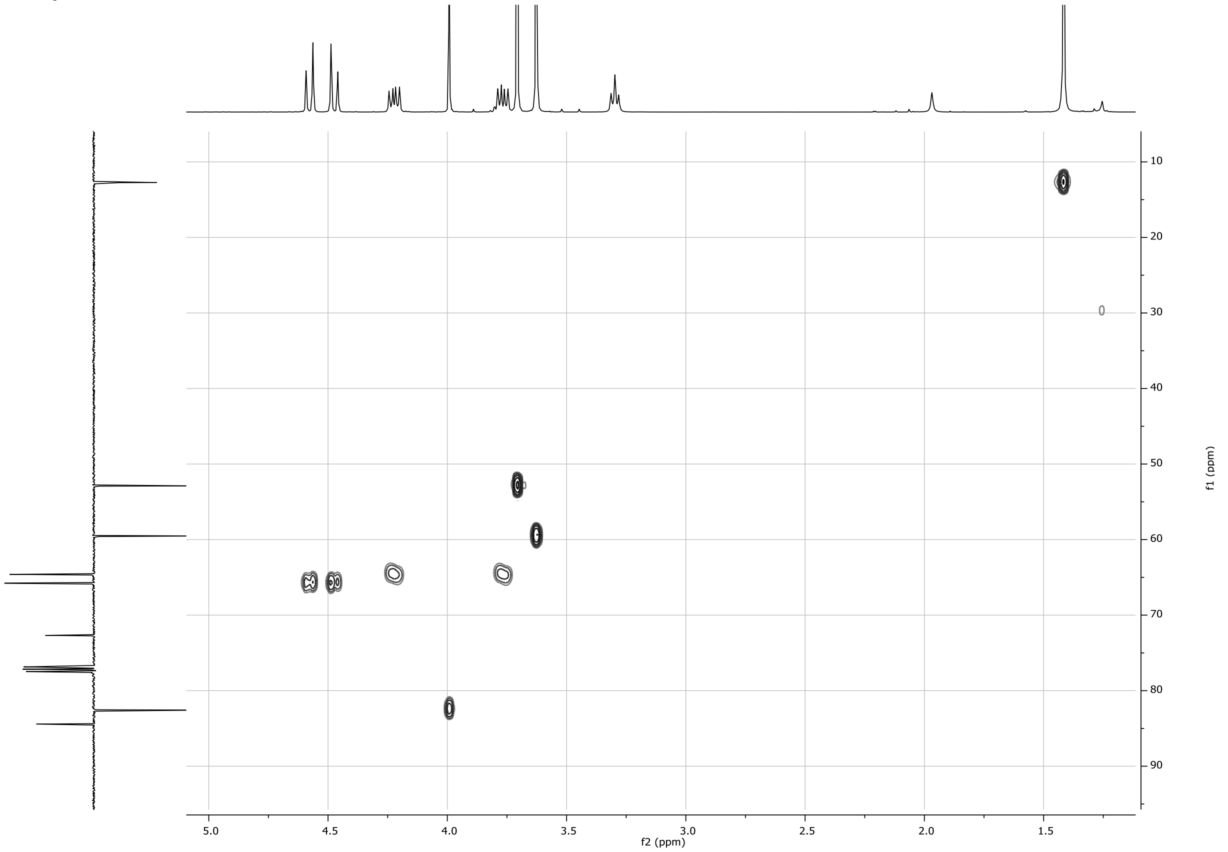

^{1}H NMR, 400 MHz, $CDCl_3$ of **S12**

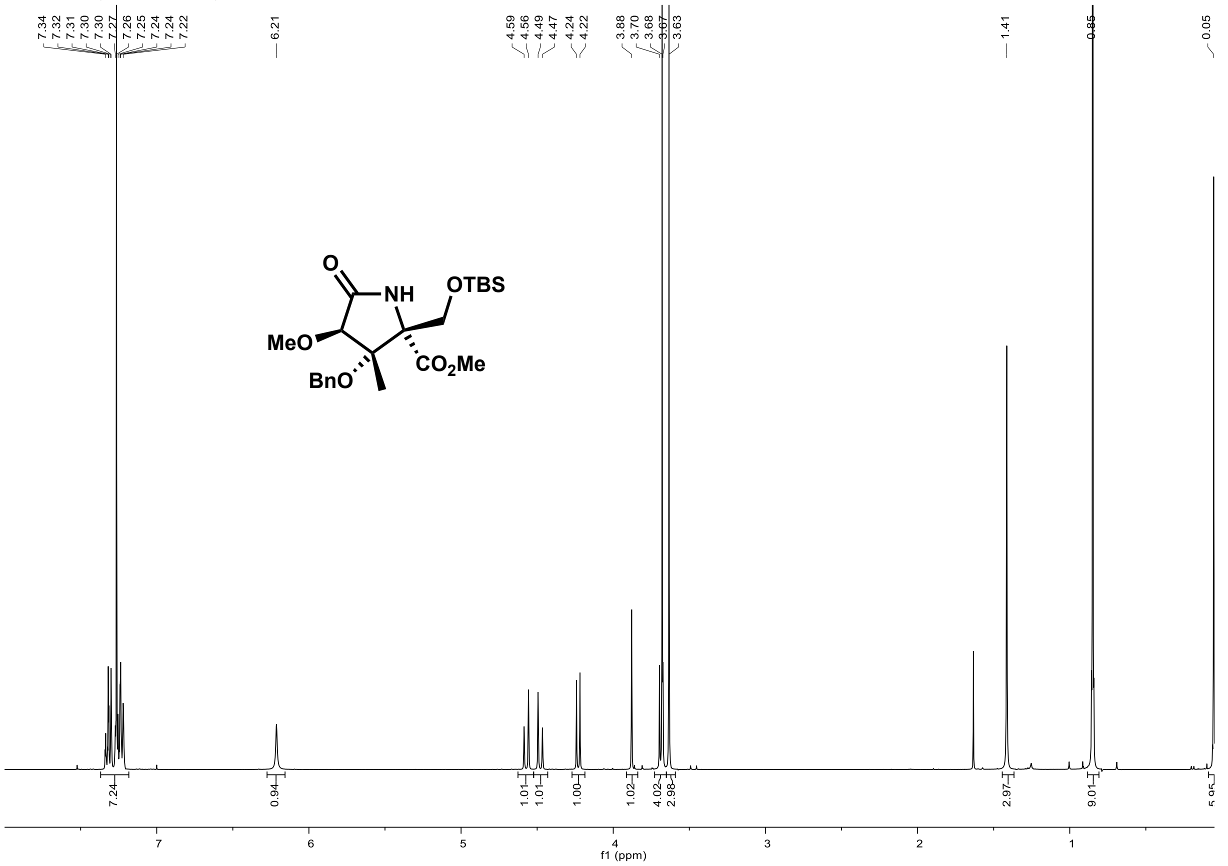

^{13}C NMR, 101 MHz, $CDCl_3$ of **S12**

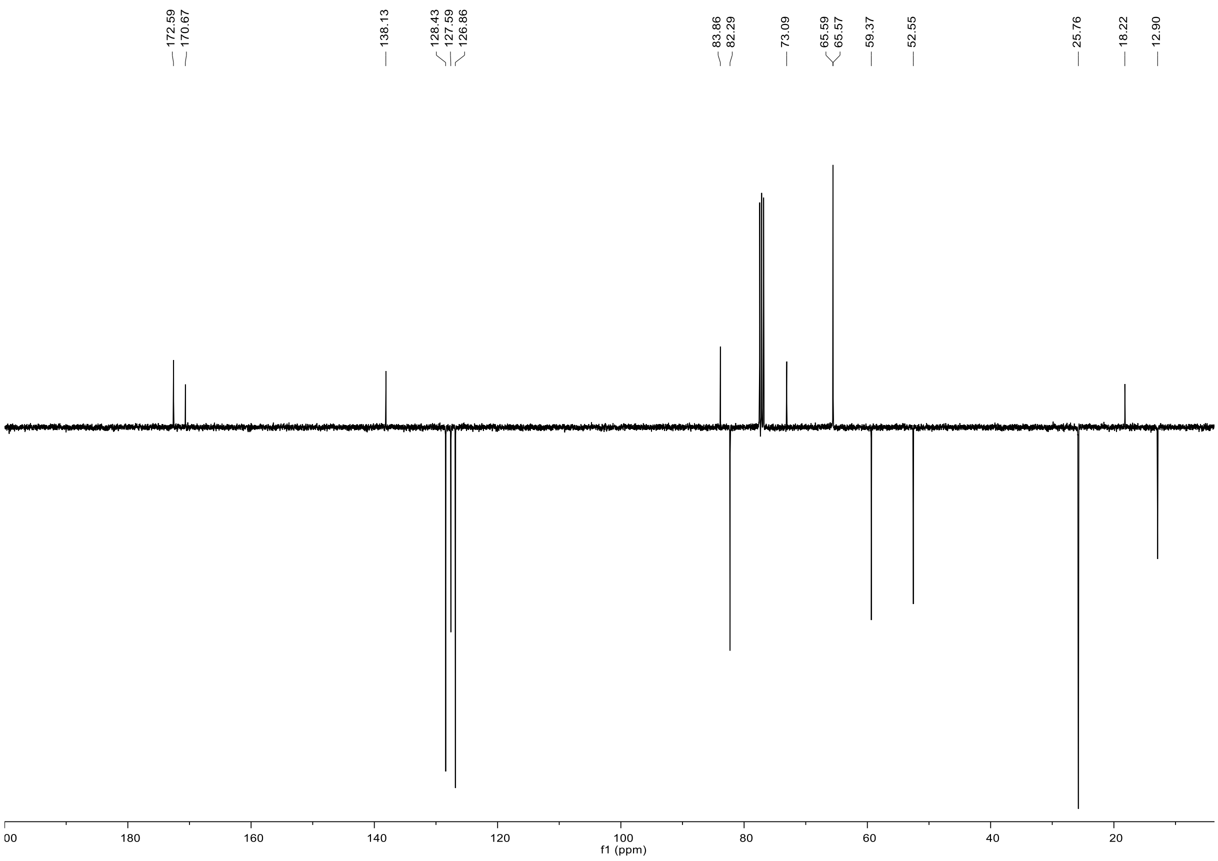

HH-COSY NMR, $CDCl_3$ of **S12**

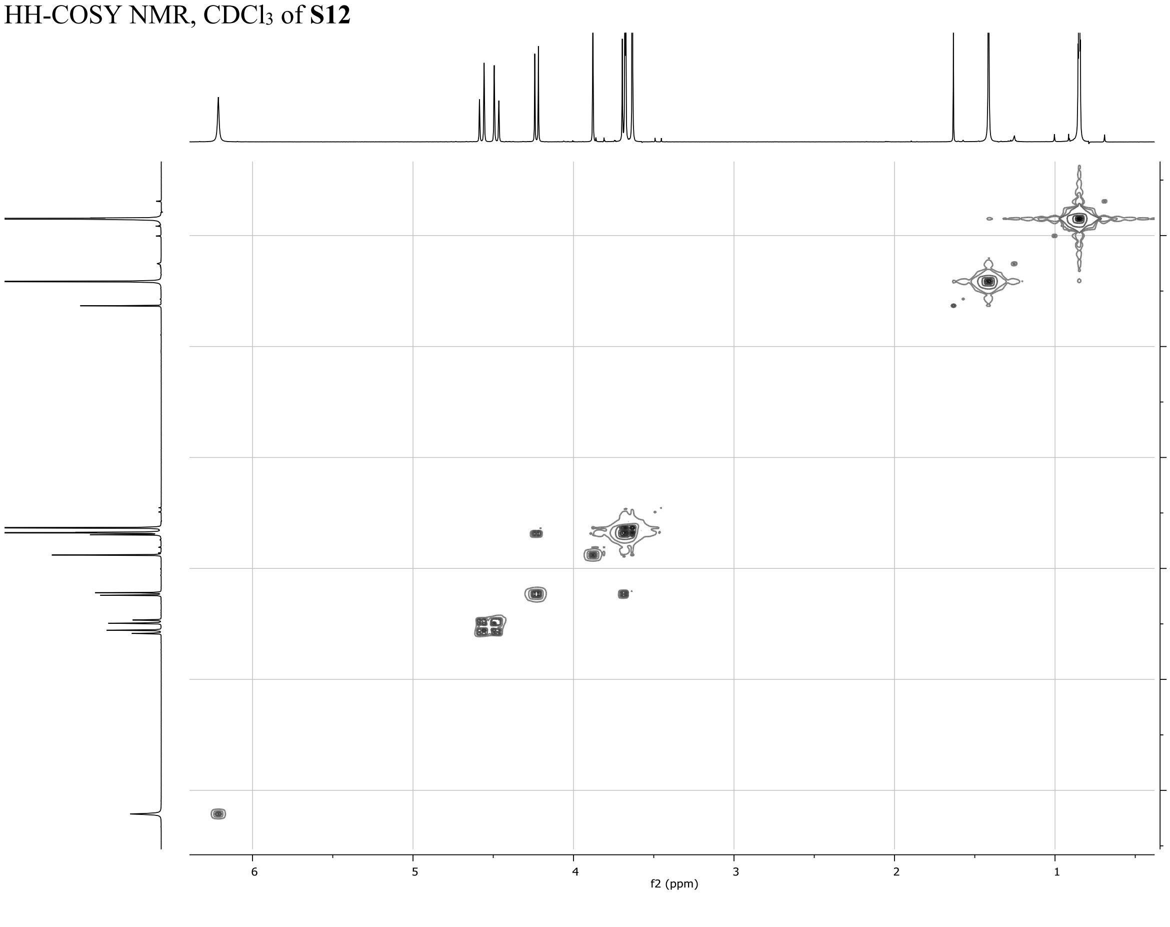

HSQC NMR, $CDCl_3$ of **S12**

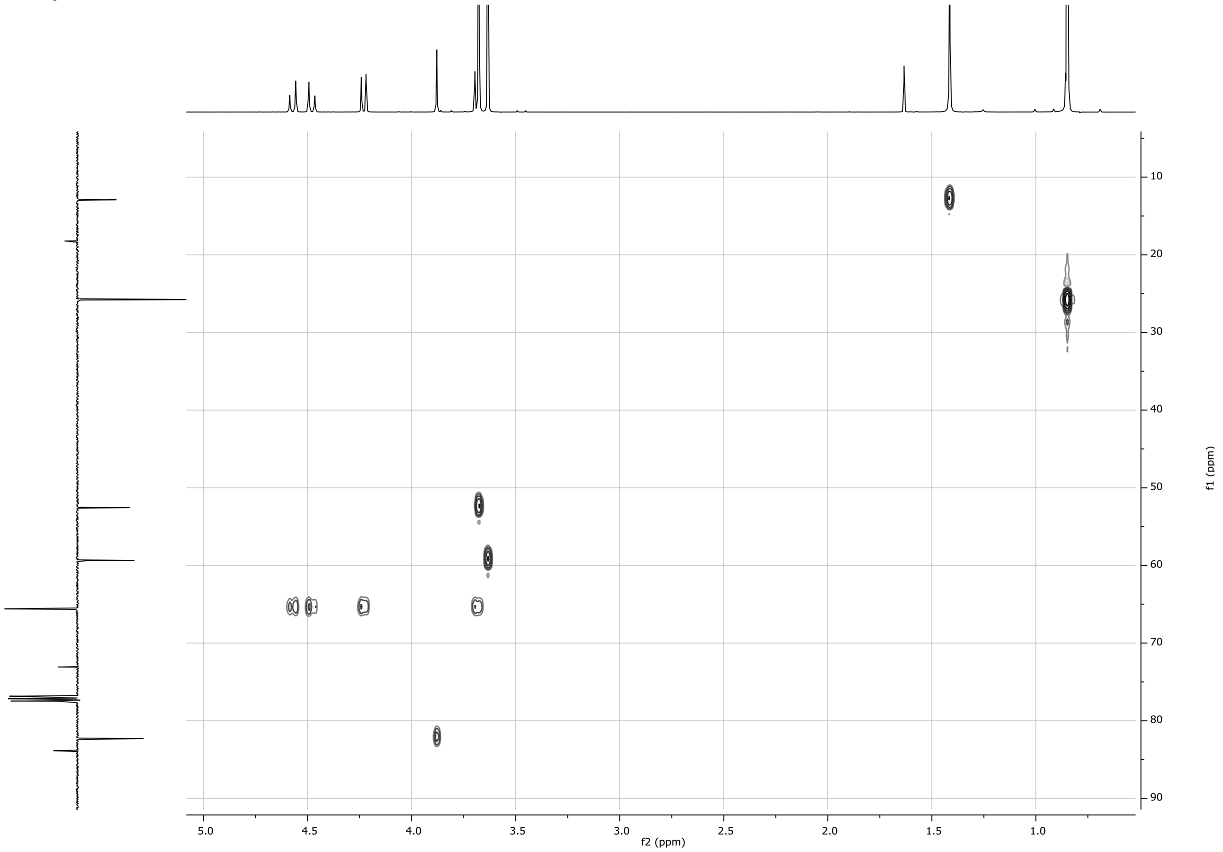

^{1}H NMR, 400 MHz, $CDCl_3$ of **S13**

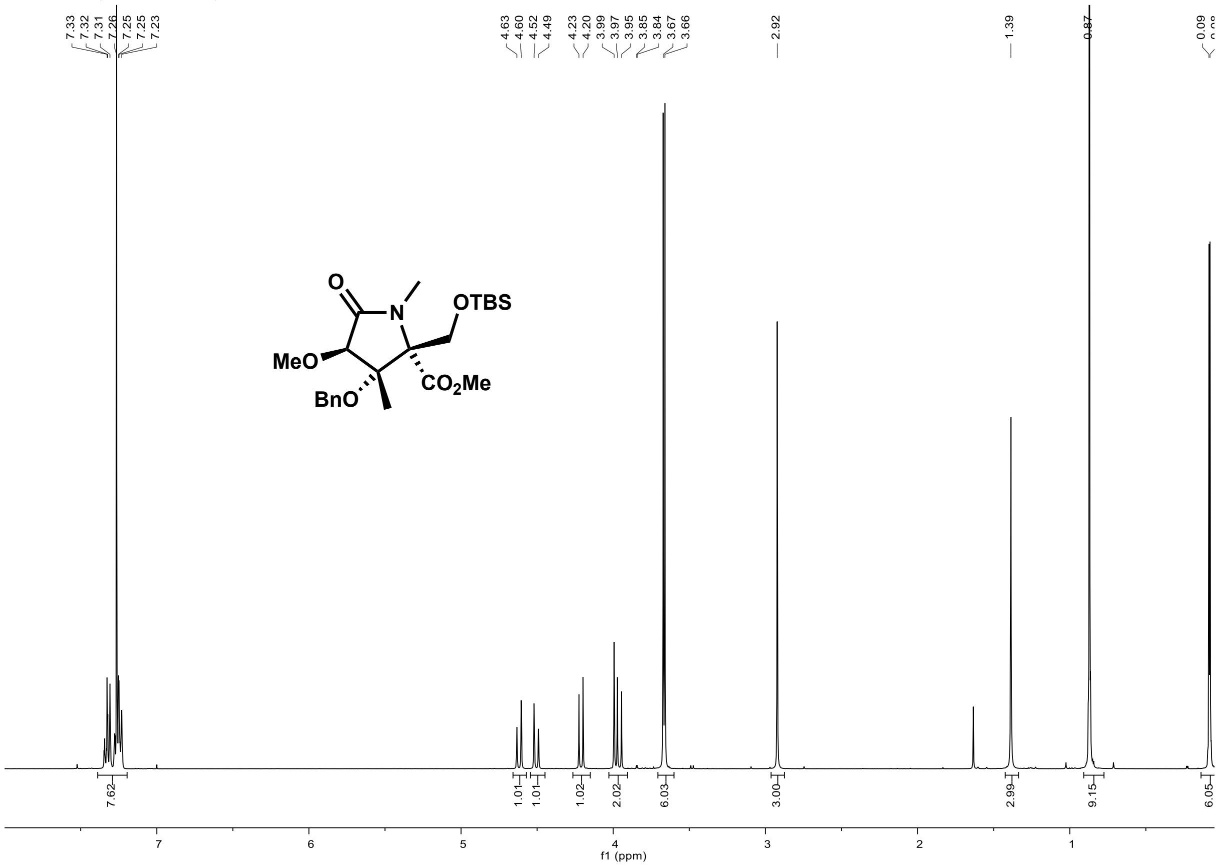

^{13}C NMR, 101 MHz, $CDCl_3$ of **S13**

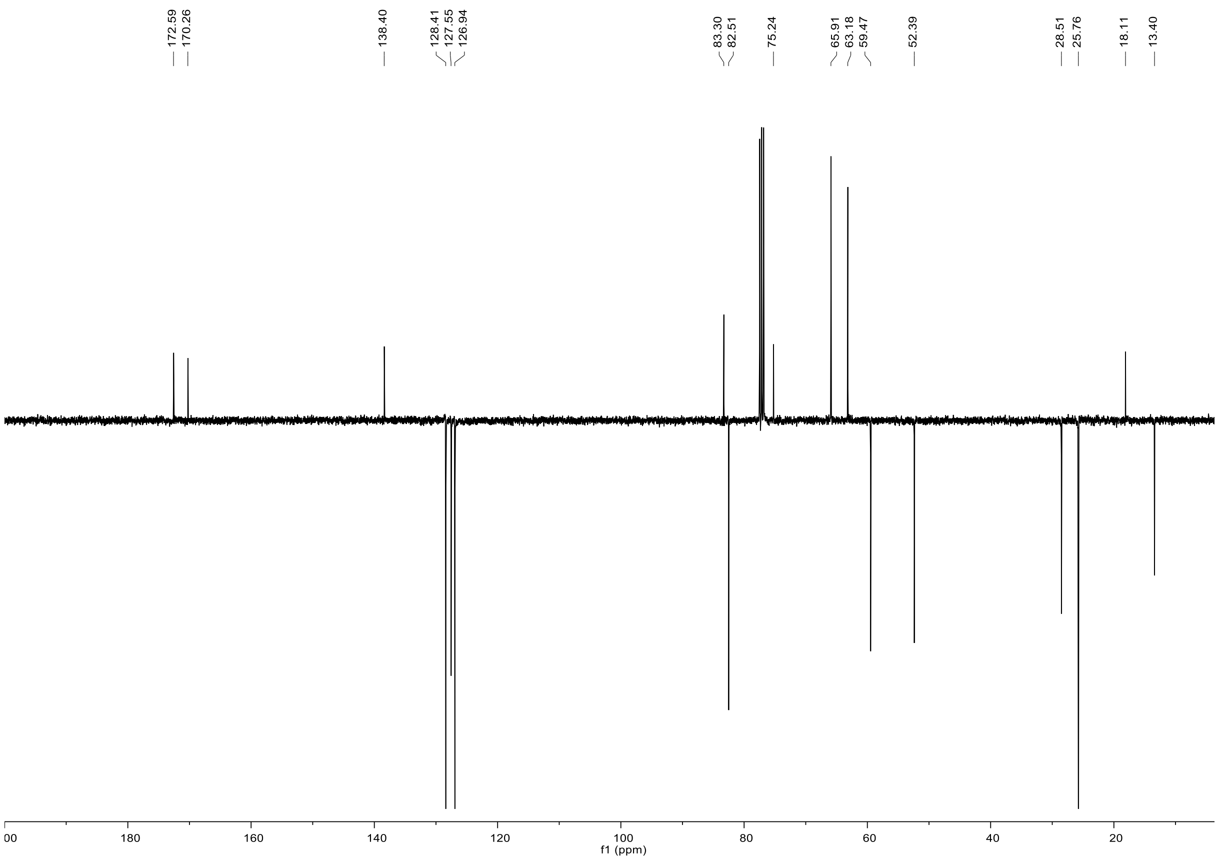

HH-COSY NMR, $CDCl_3$ of **S13**

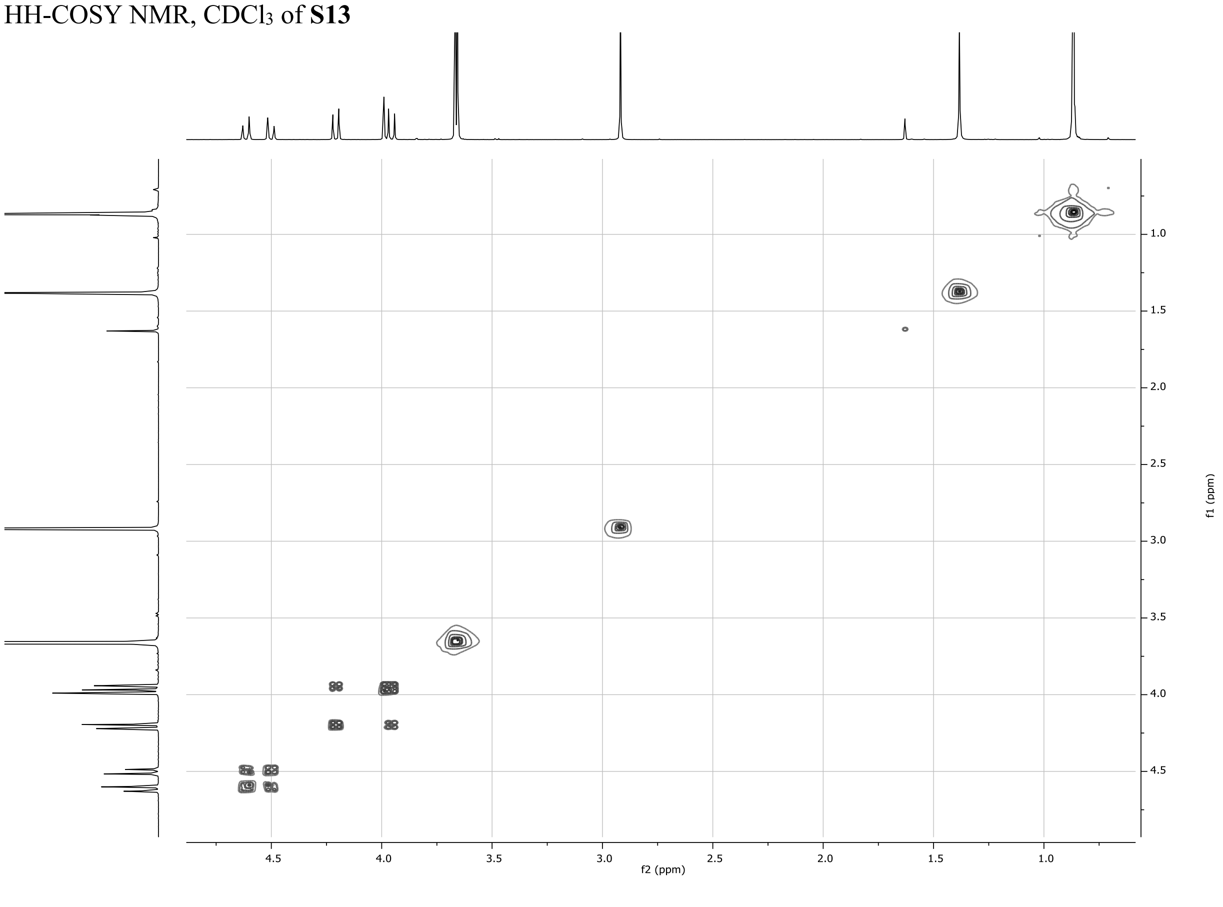

HSQC NMR, $CDCl_3$ of **S13**

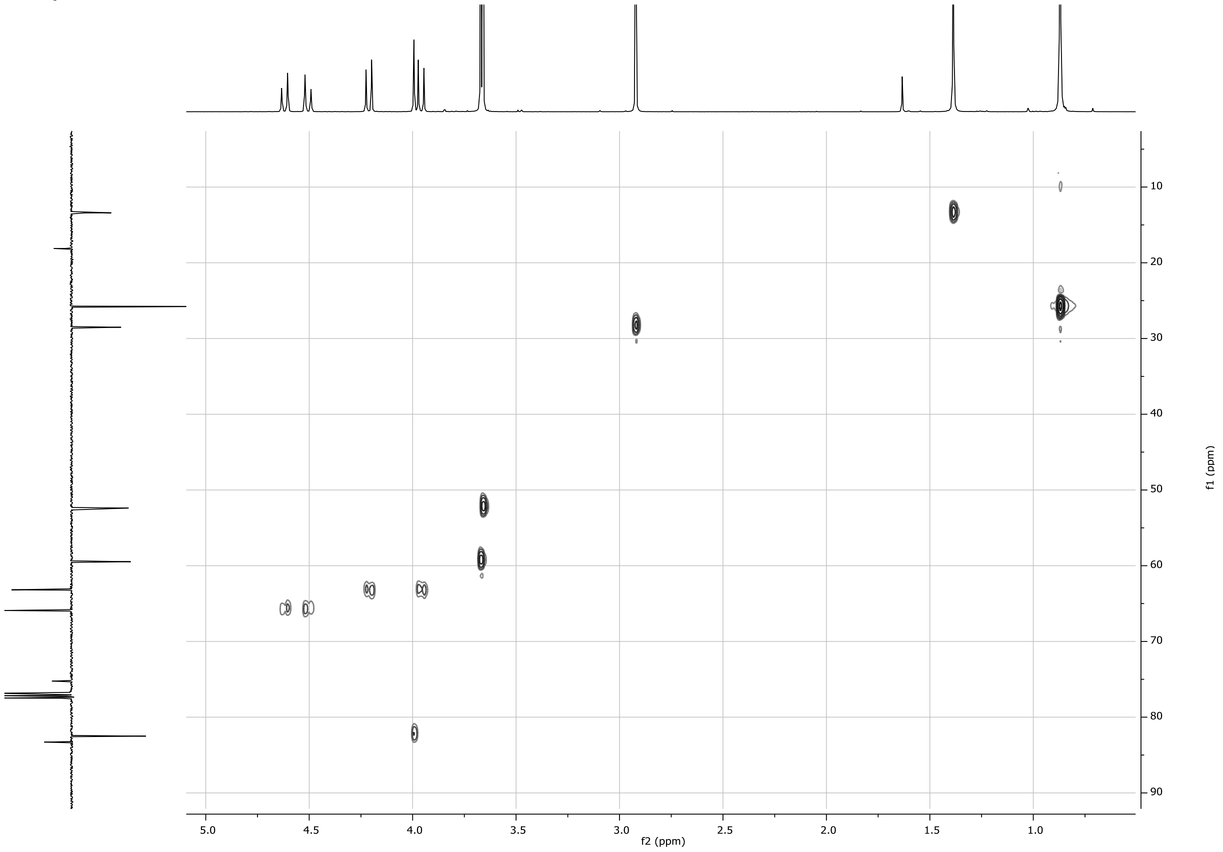

^{1}H NMR, 400 MHz, $CDCl_3$ of **S14**

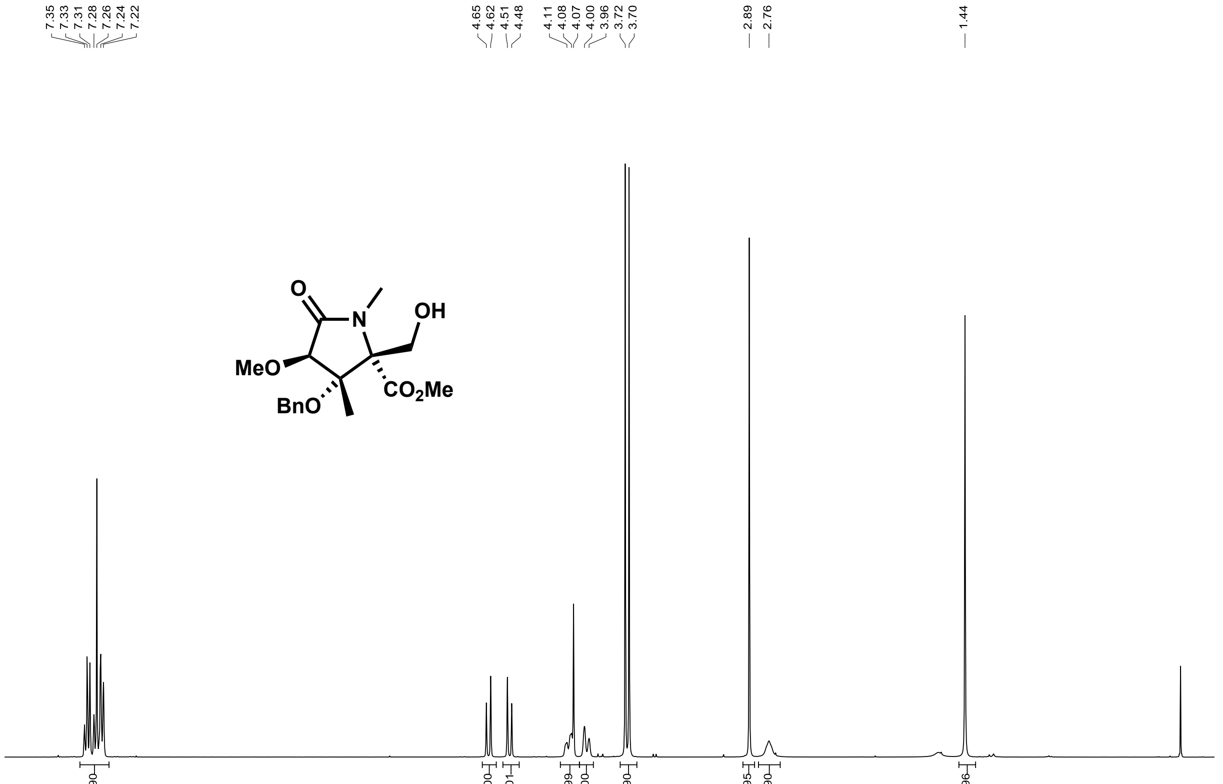

^{13}C NMR, 101 MHz, $CDCl_3$ of **S14**

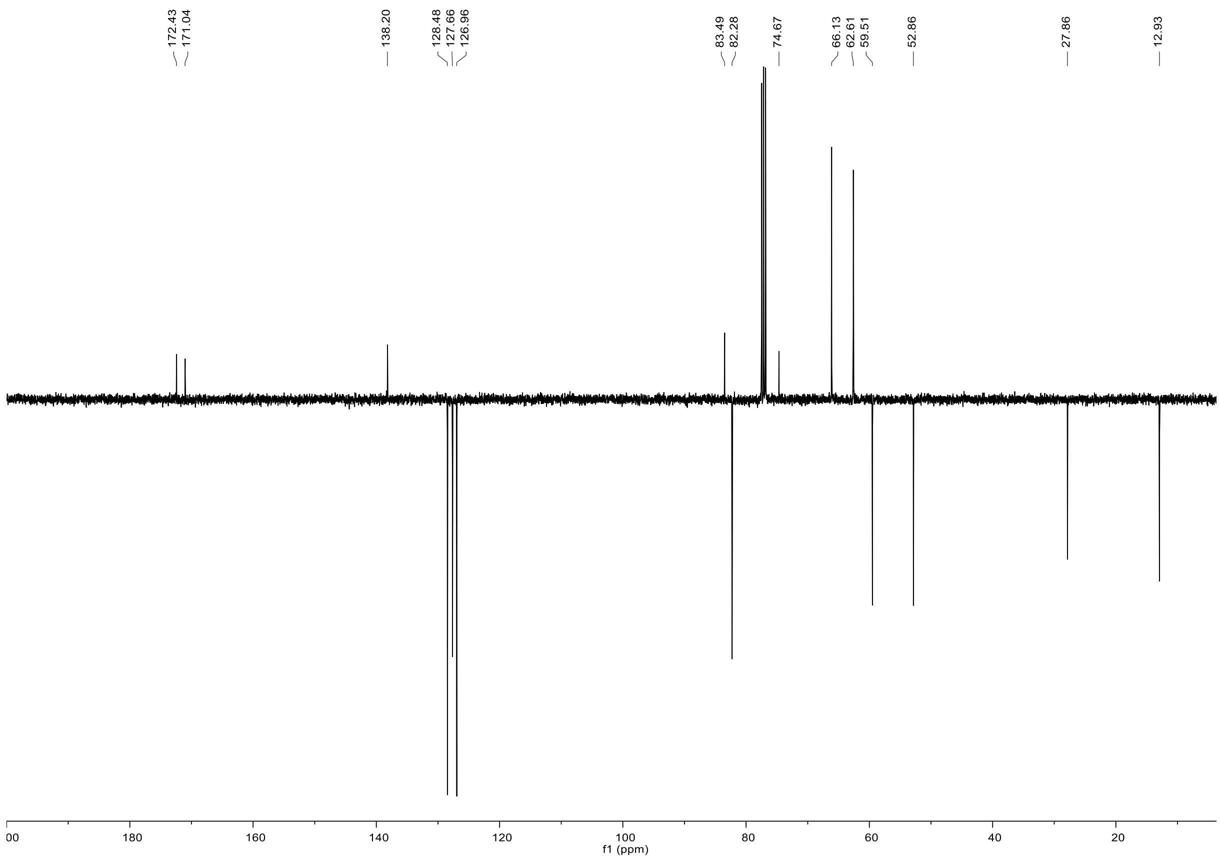

HH-COSY NMR, $CDCl_3$ of **S14**

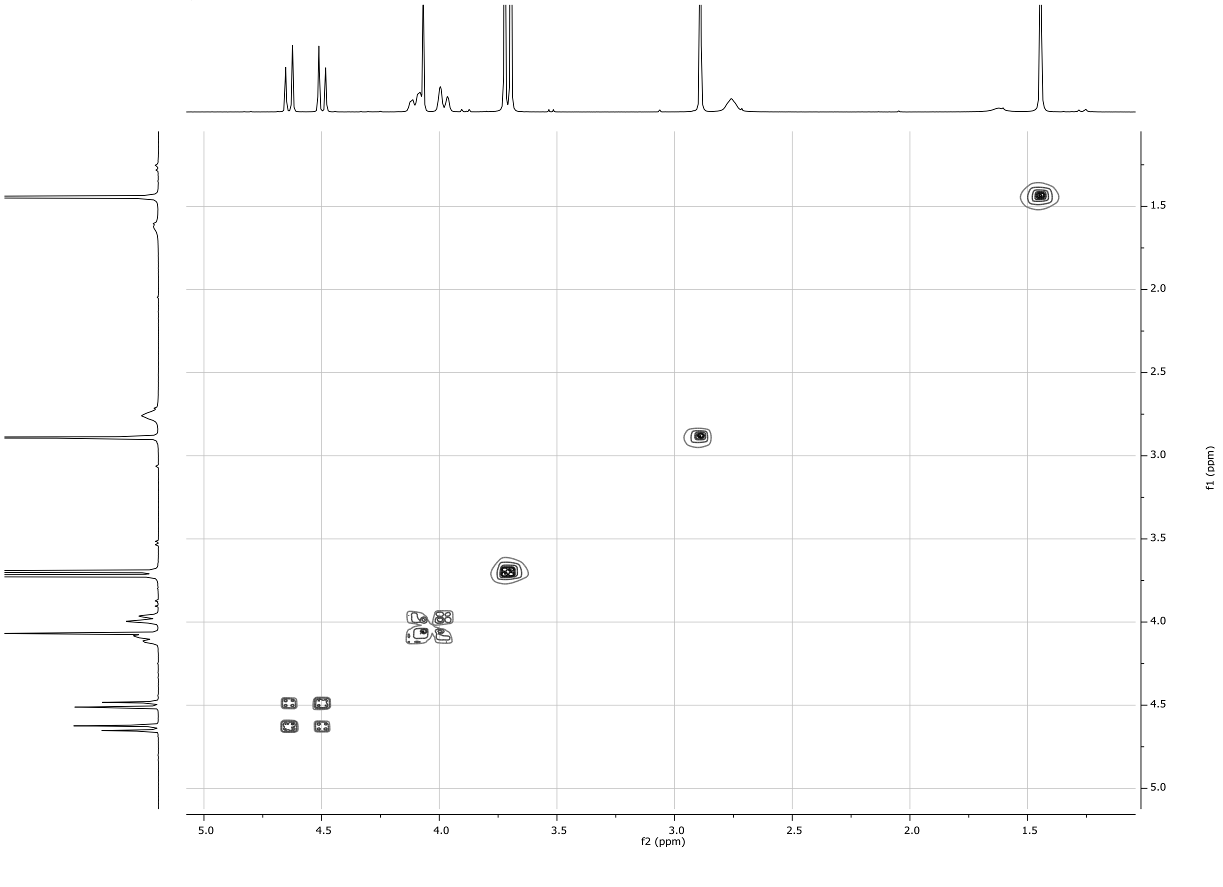

HSQC NMR, $CDCl_3$ of **S14**

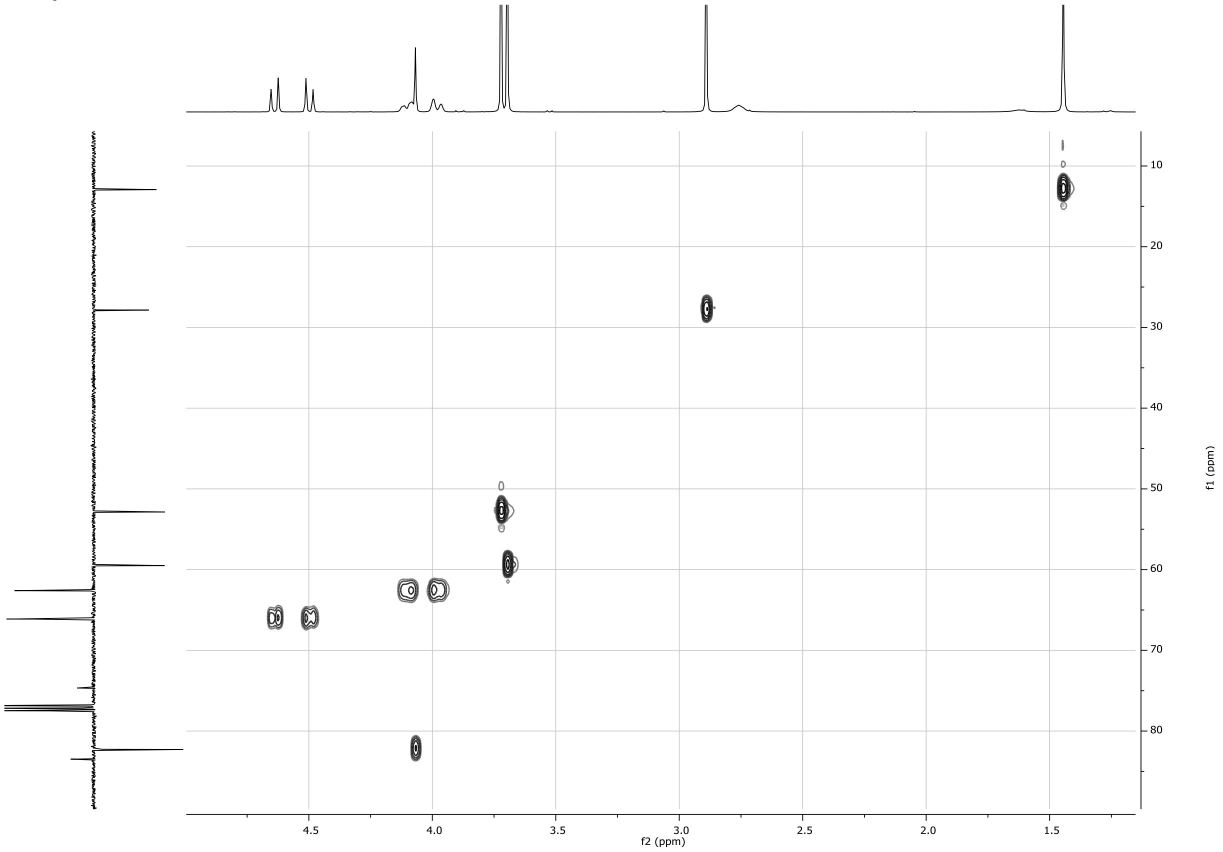

^{1}H NMR, 400 MHz, $CDCl_3$ of **S15**

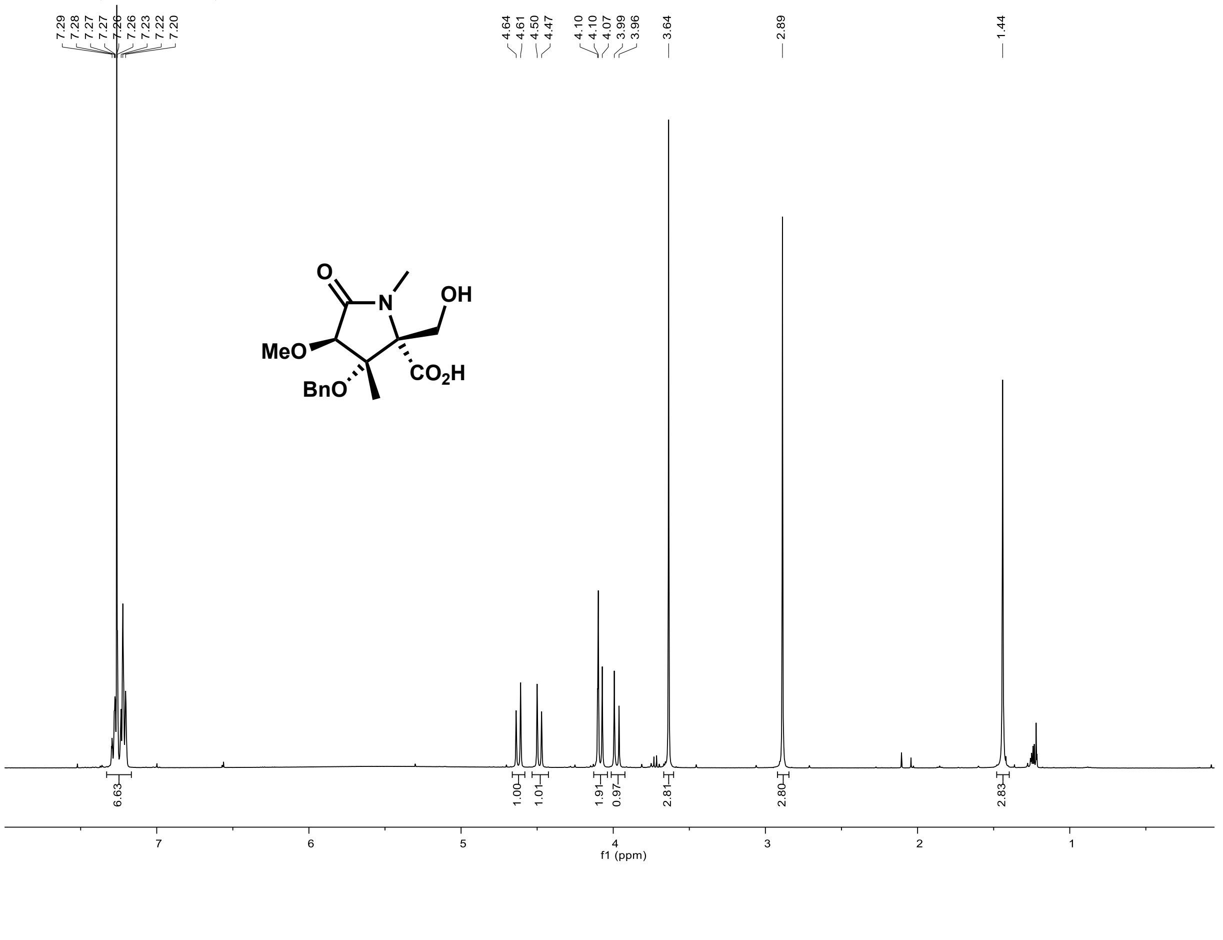

^{13}C NMR, 101 MHz, $CDCl_3$ of **S15**

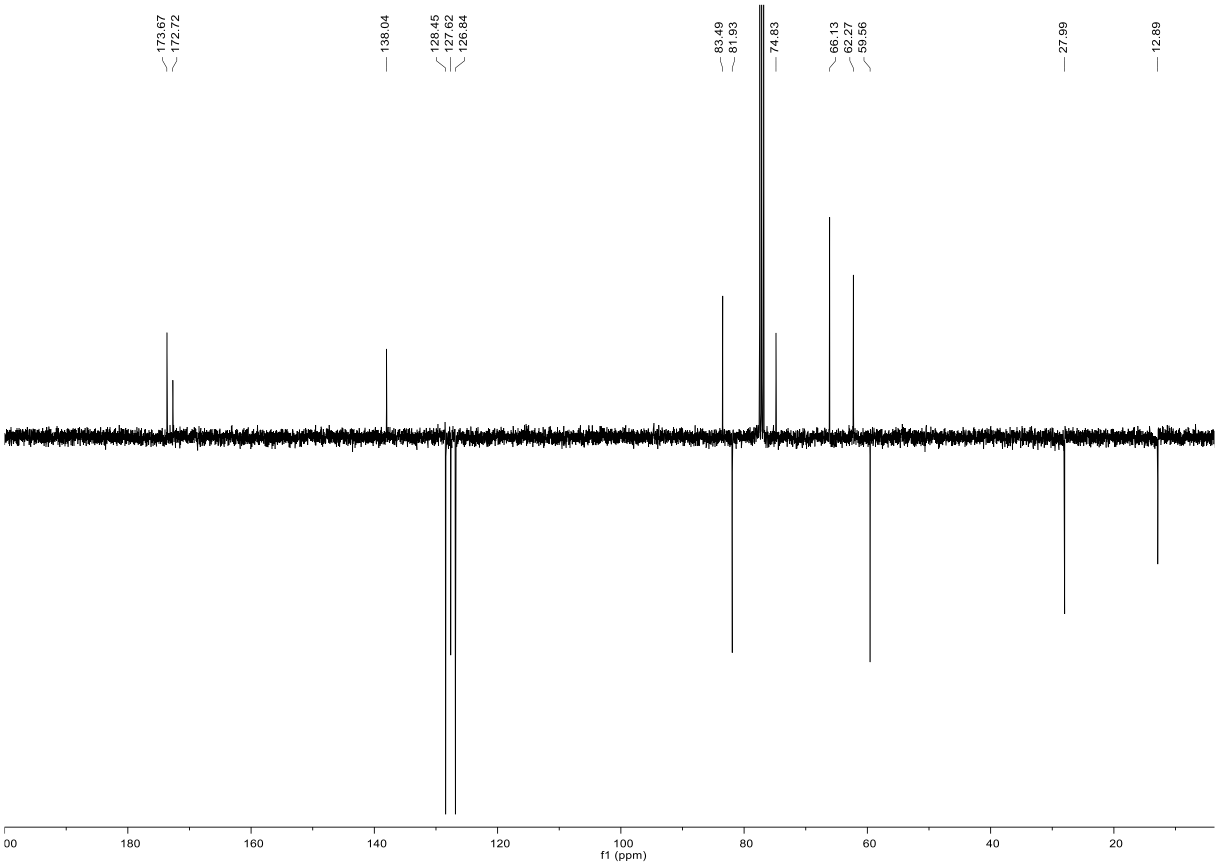

HH-COSY NMR, $CDCl_3$ of **S15**

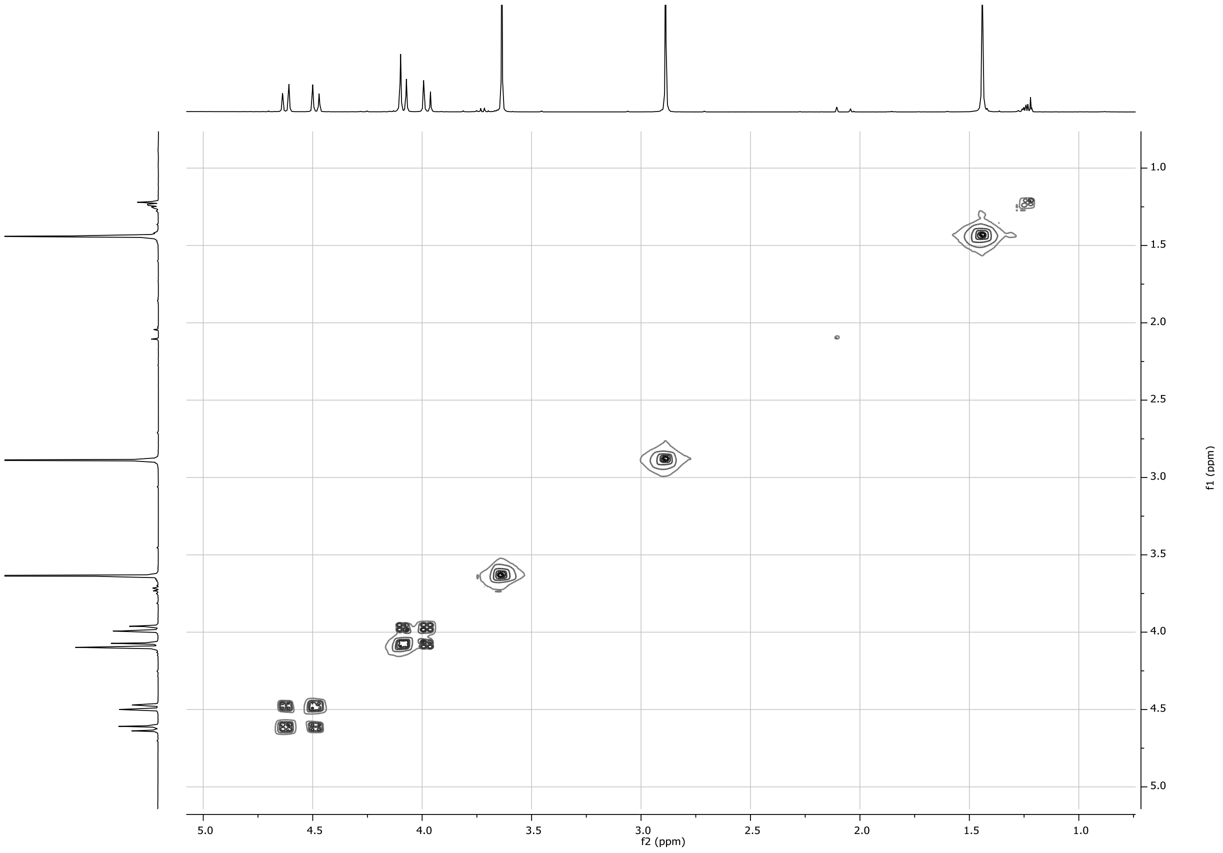

HSQC NMR, $CDCl_3$ of **S15**

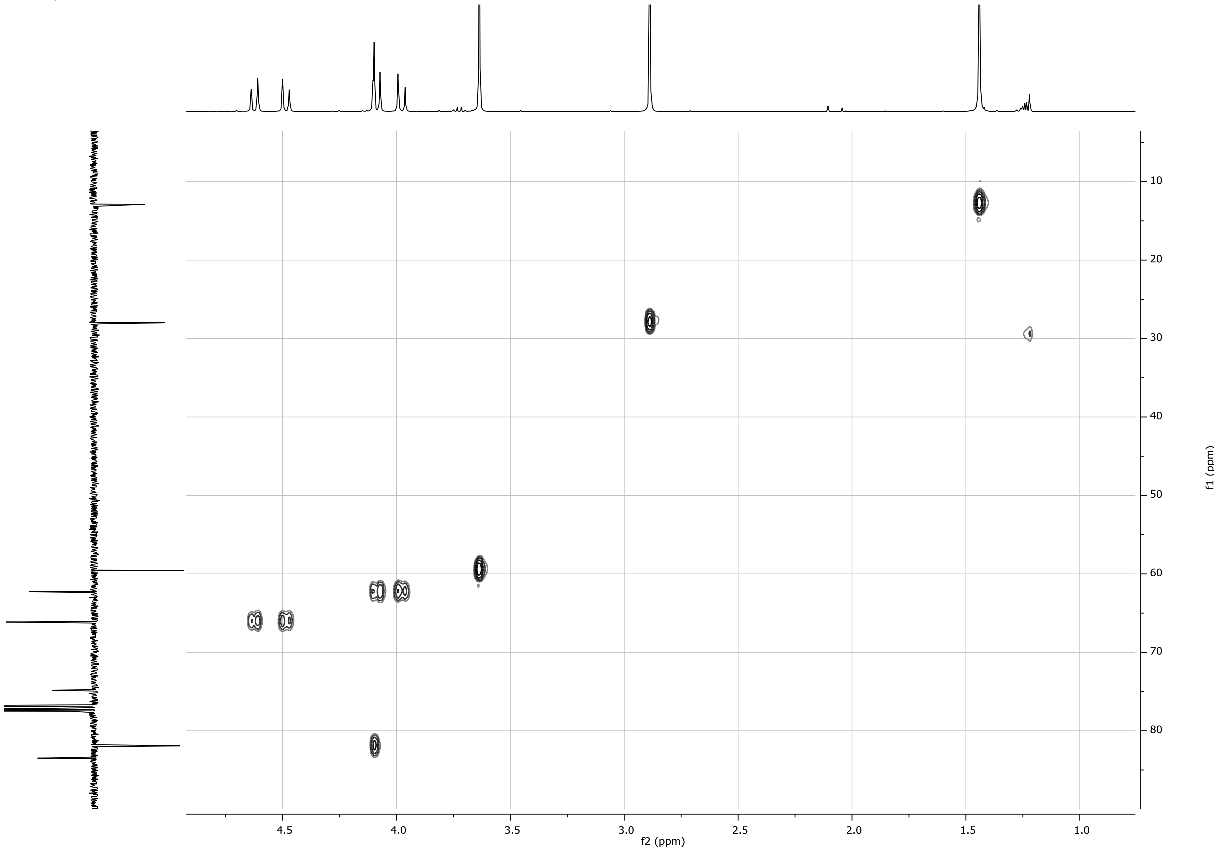

^{1}H NMR, 400 MHz, $CDCl_3$ of **S16**

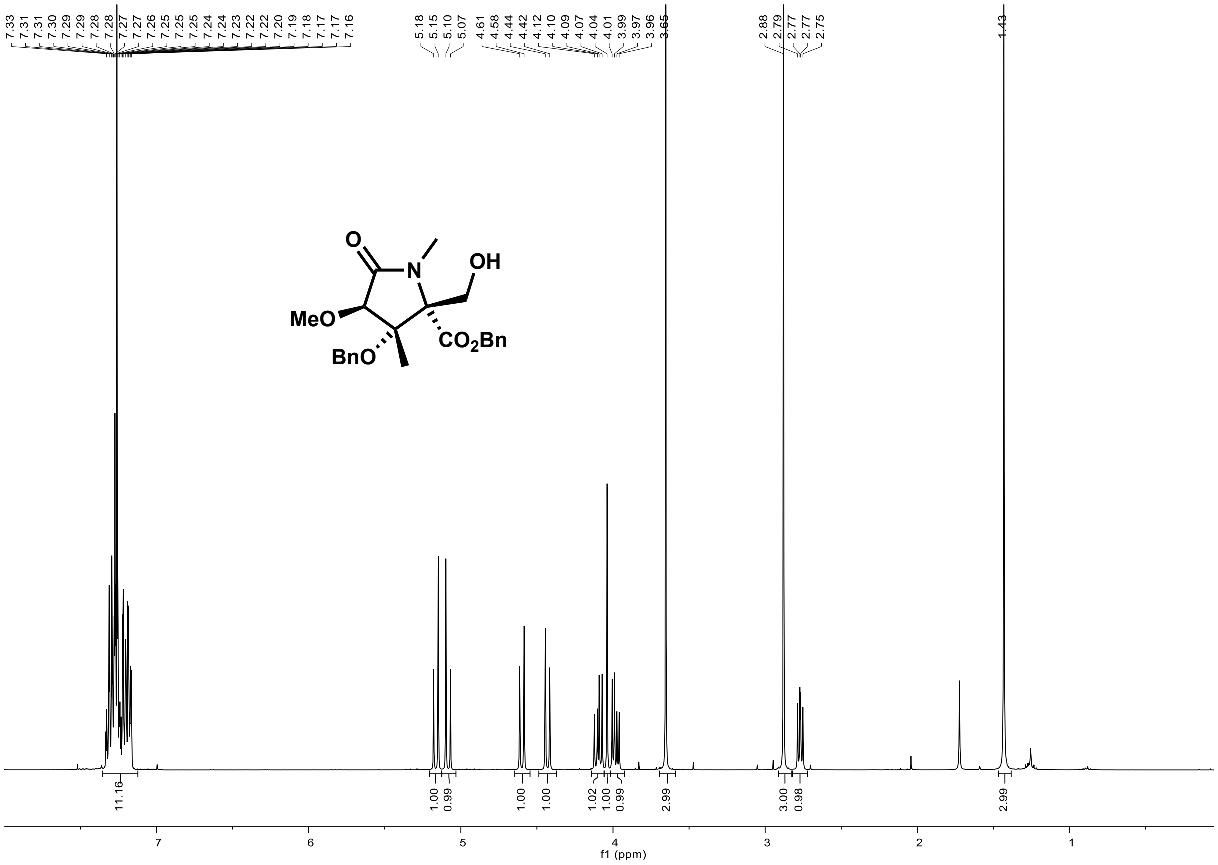

^{13}C NMR, 101 MHz, $CDCl_3$ of **S16**

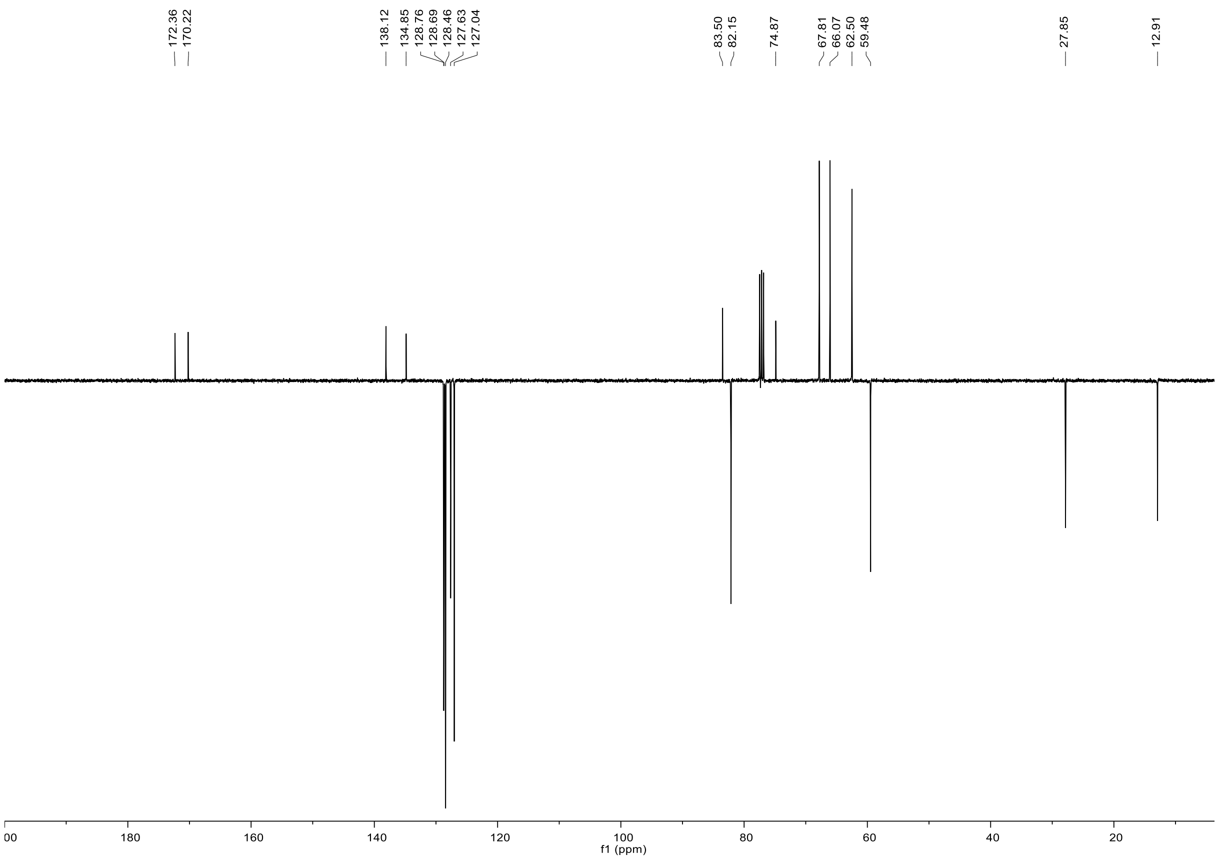

HH-COSY NMR, $CDCl_3$ of **S16**

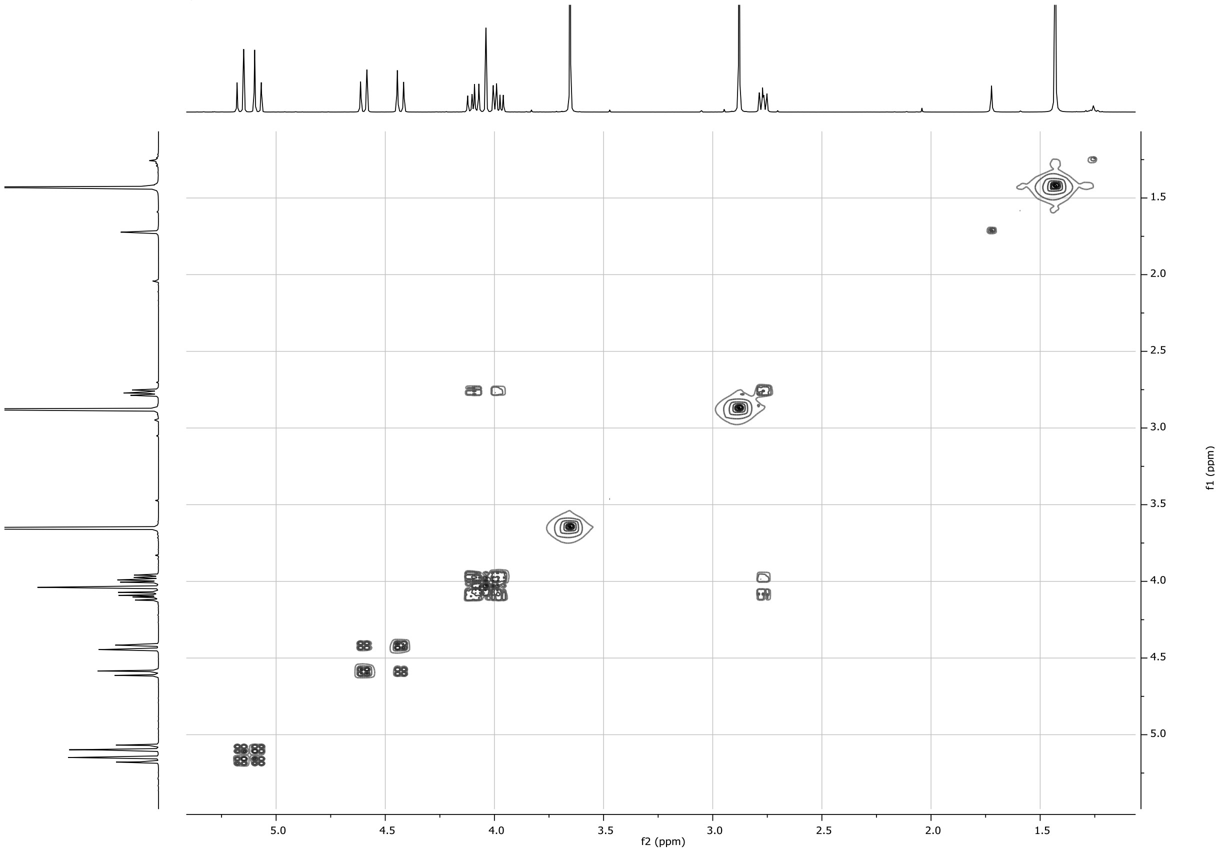

HSQC NMR, $CDCl_3$ of **S16**

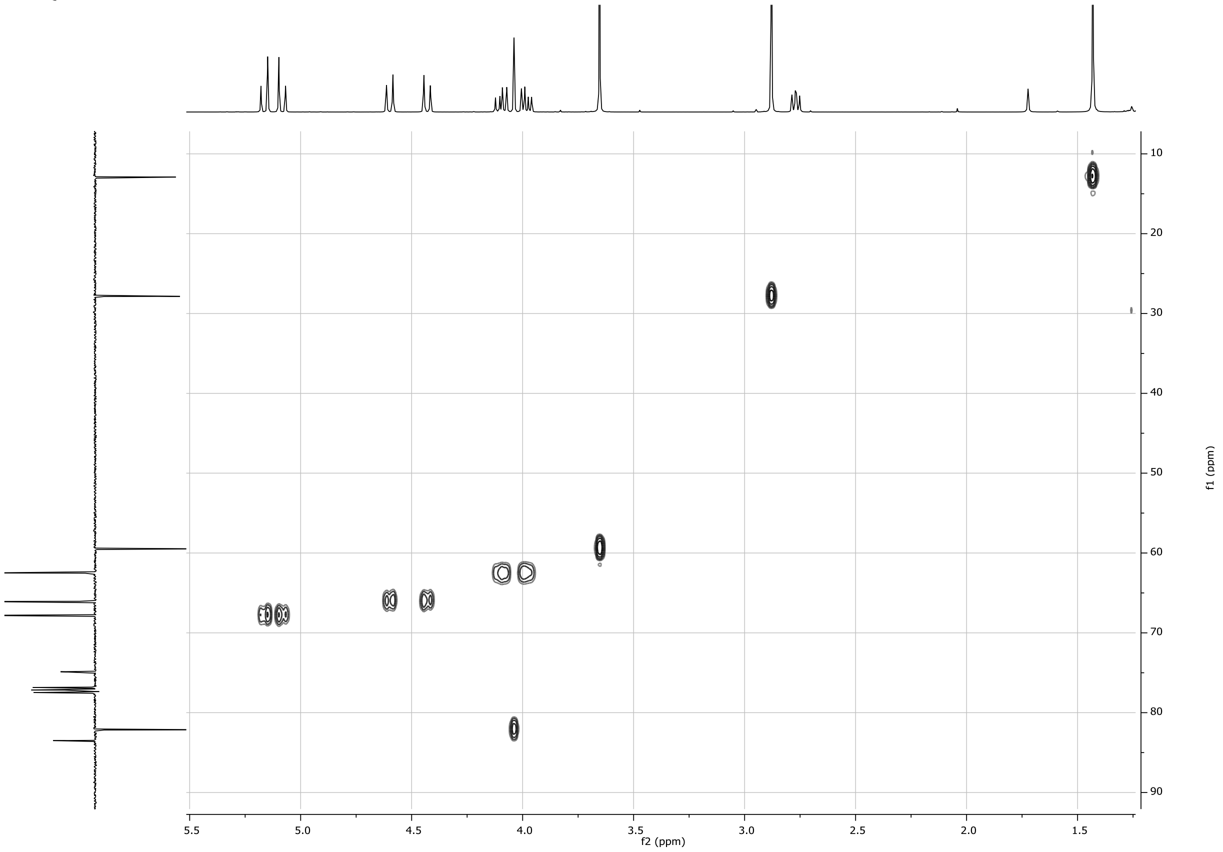

^{1}H NMR, 400 MHz, $CDCl_3$ of **S17**

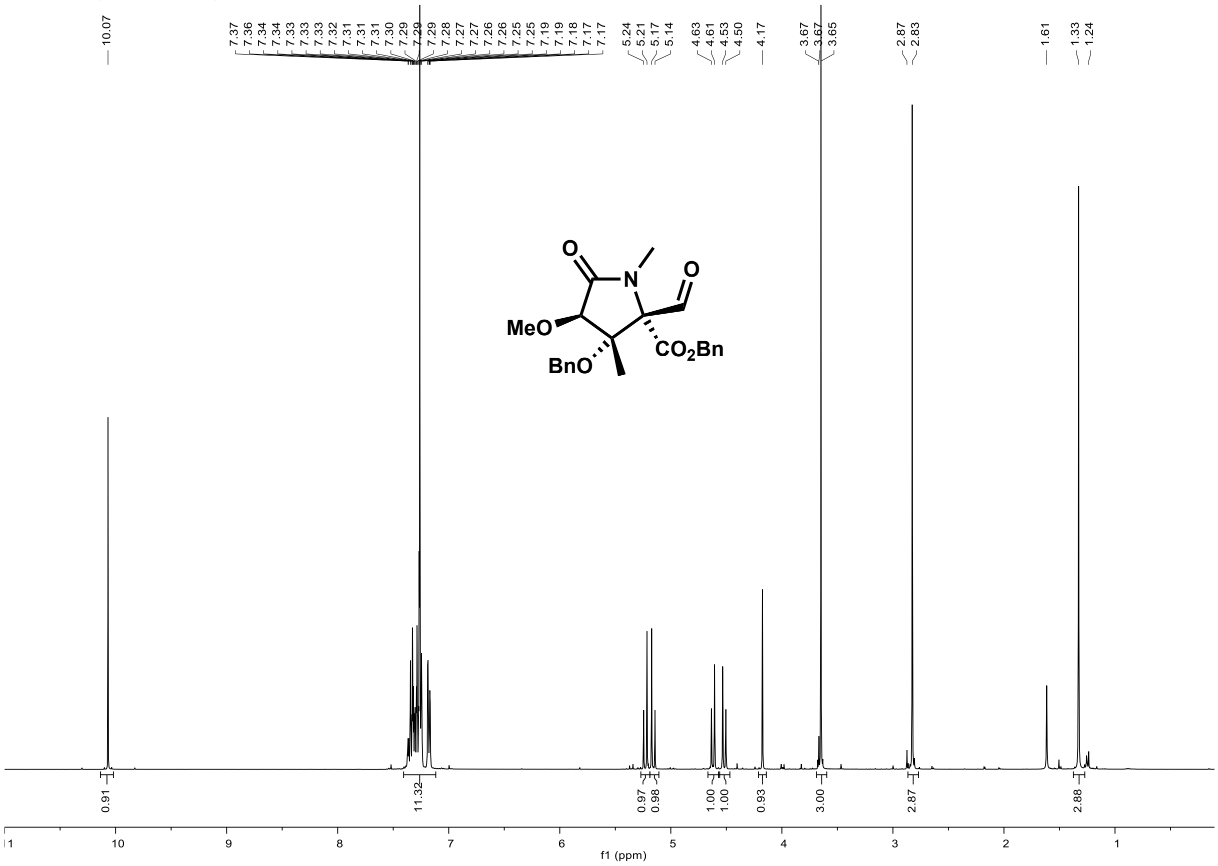

^{13}C NMR, 101 MHz, $CDCl_3$ of **S17**

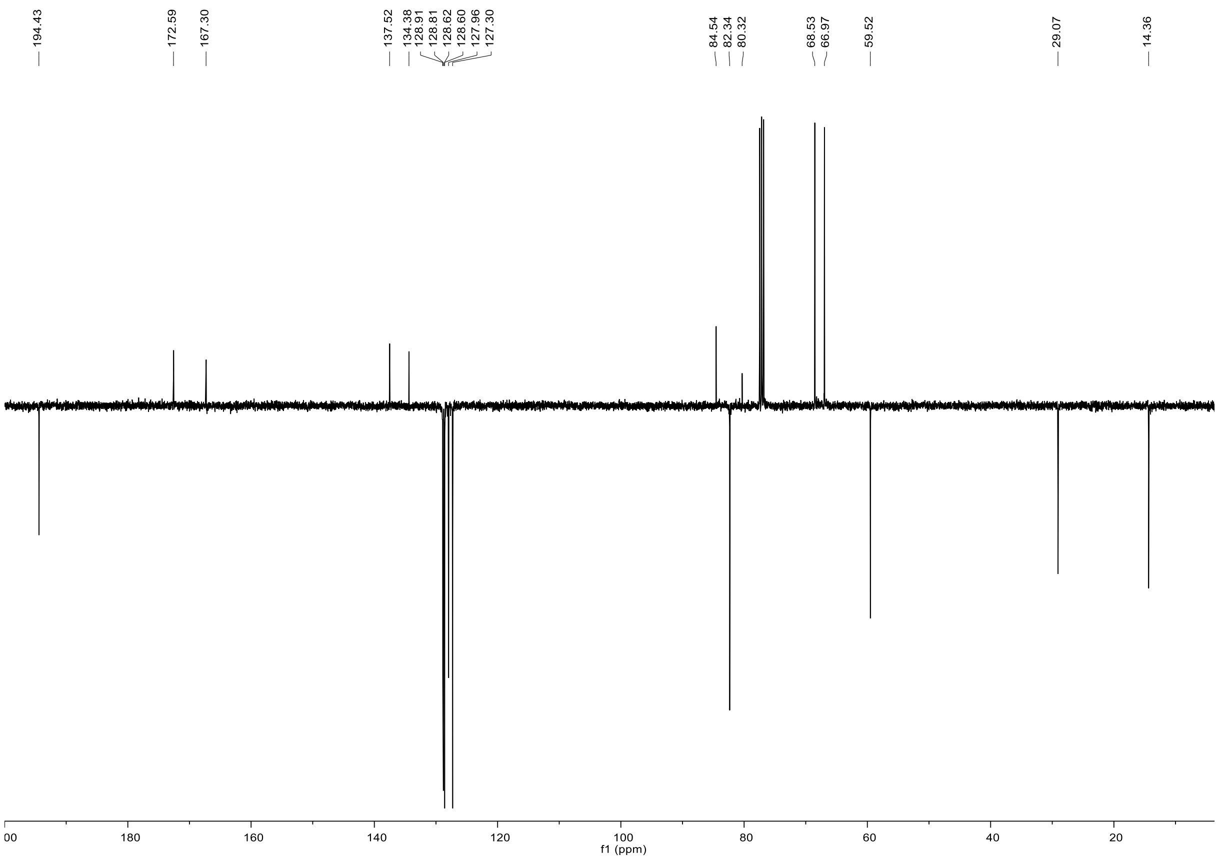

HH-COSY NMR, $CDCl_3$ of **S17**

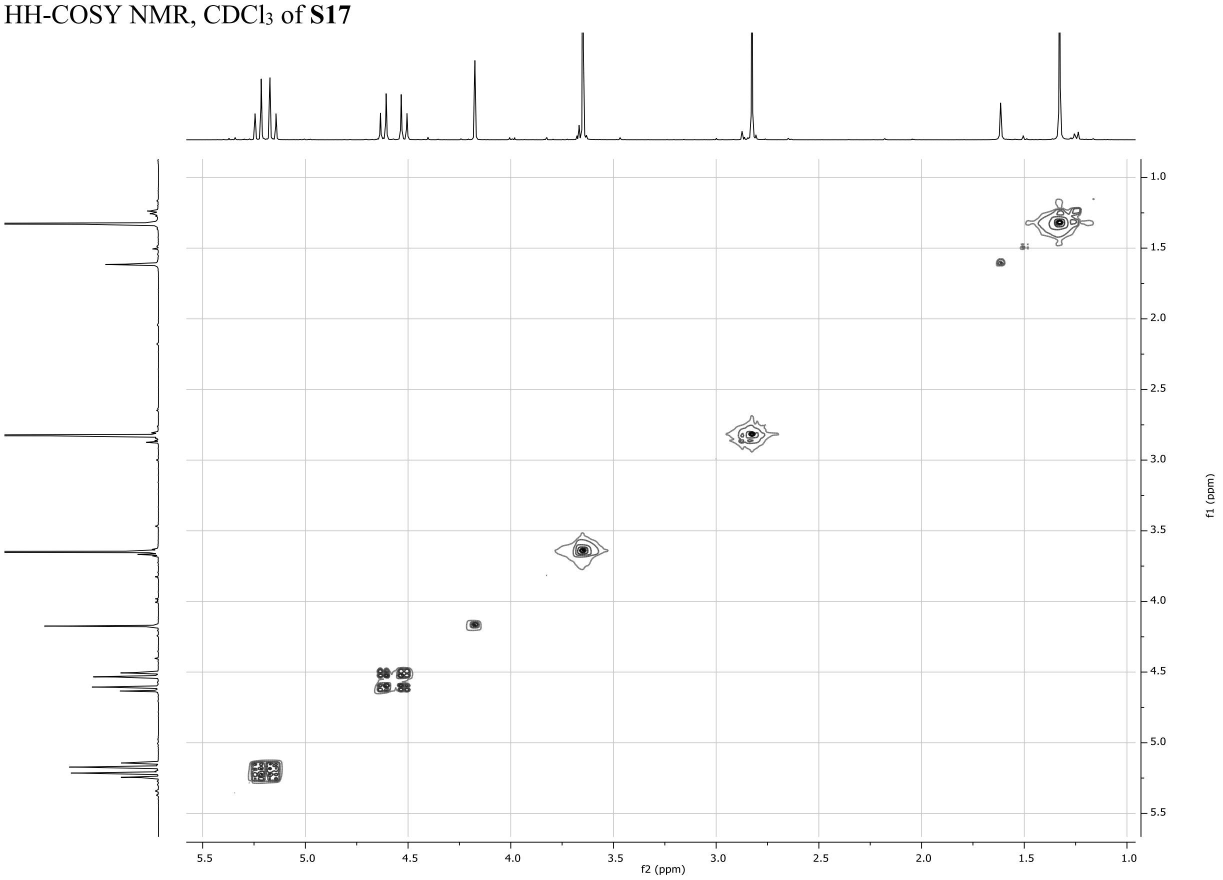

HSQC NMR, $CDCl_3$ of **S17**

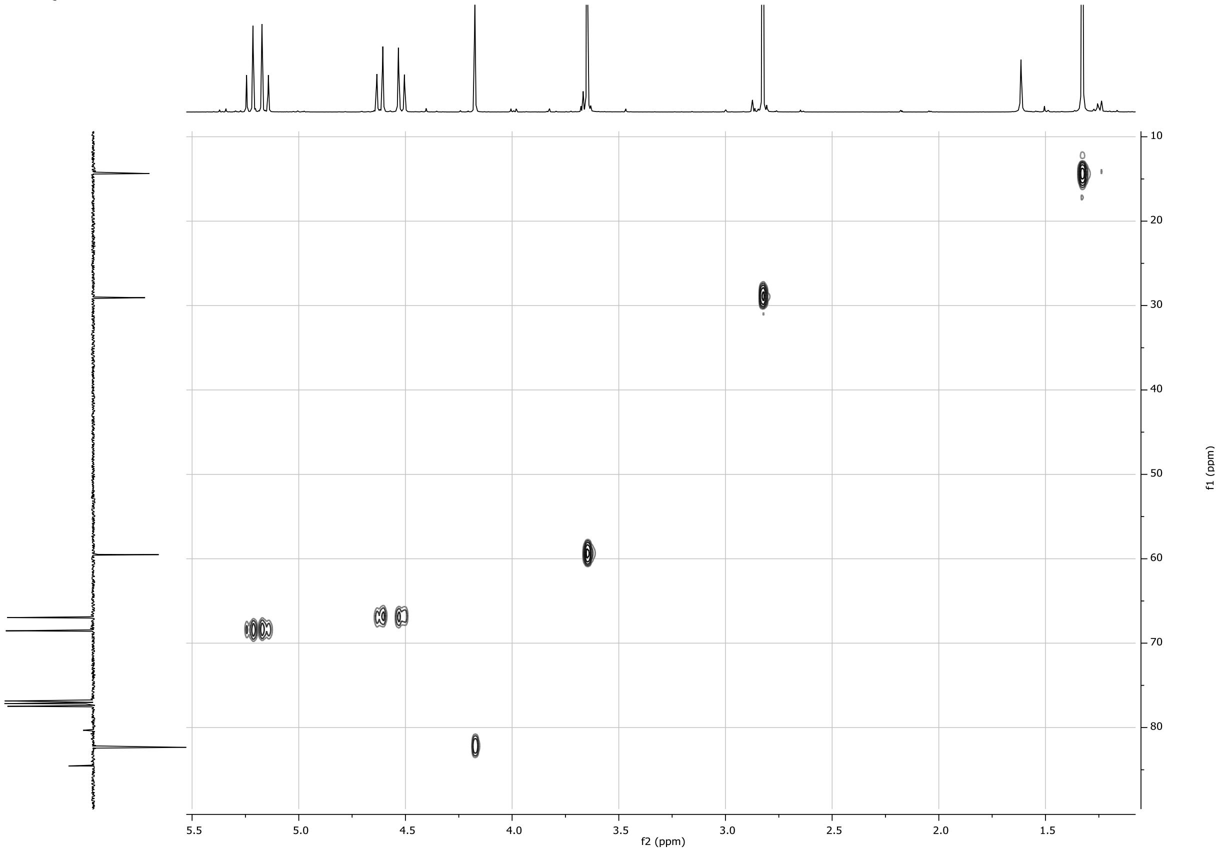

^{1}H NMR, 400 MHz, $CDCl_3$ of **2**

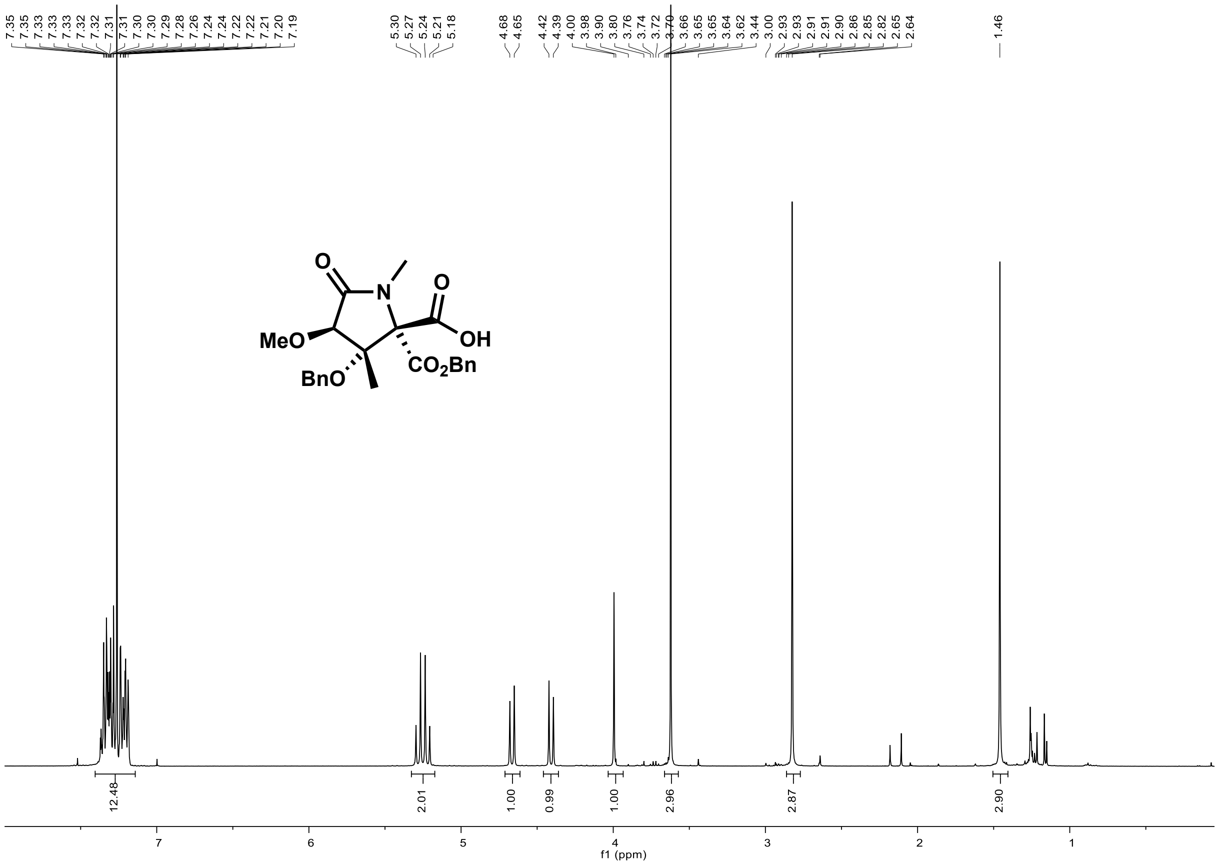

^{13}C NMR, 101 MHz, $CDCl_3$ of **2**

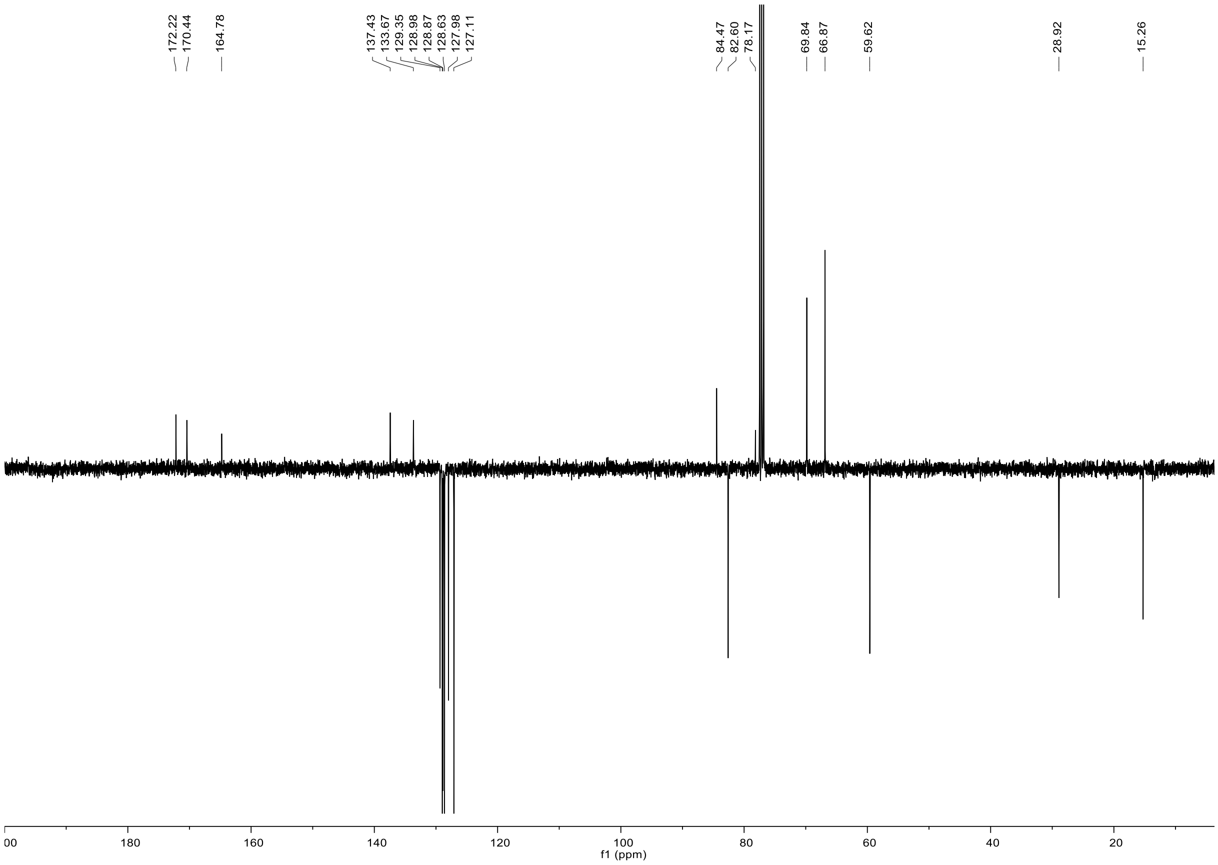

HH-COSY NMR, $CDCl_3$ of **2**

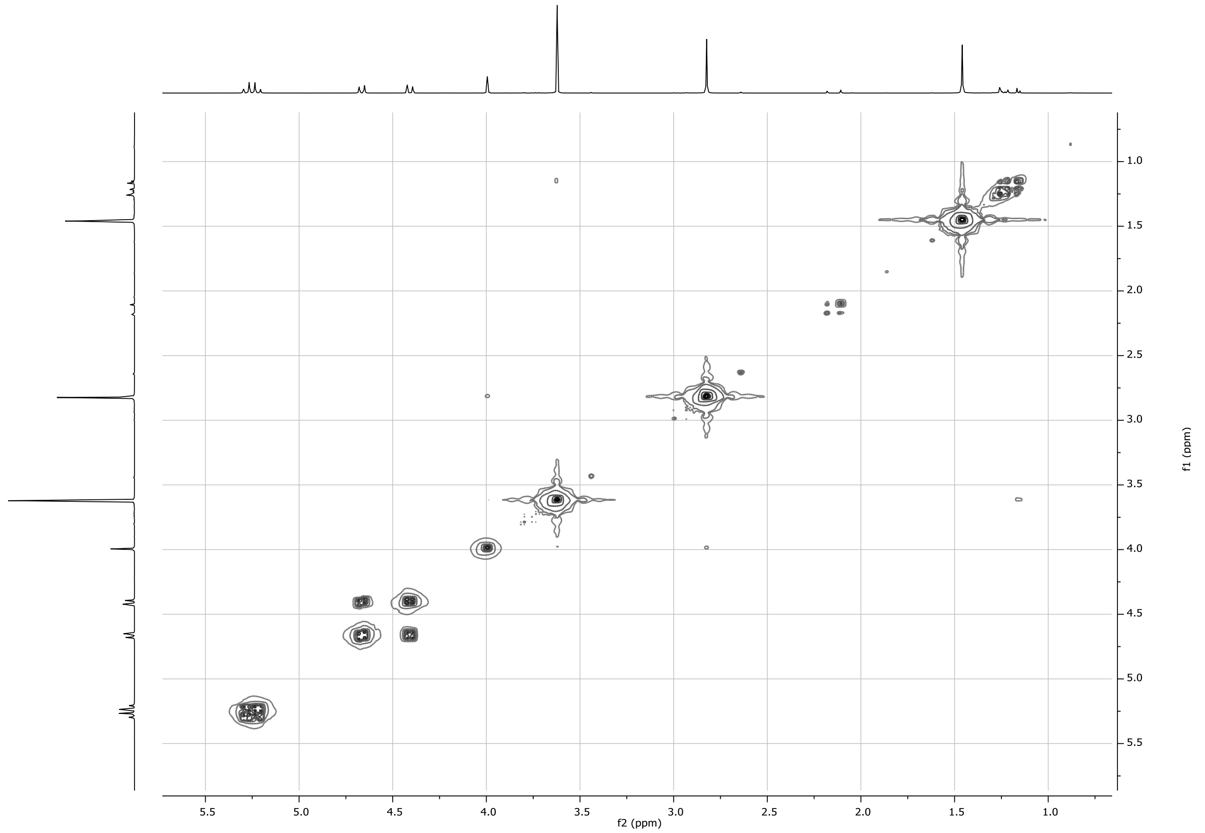

HSQC NMR, $CDCl_3$ of **2**

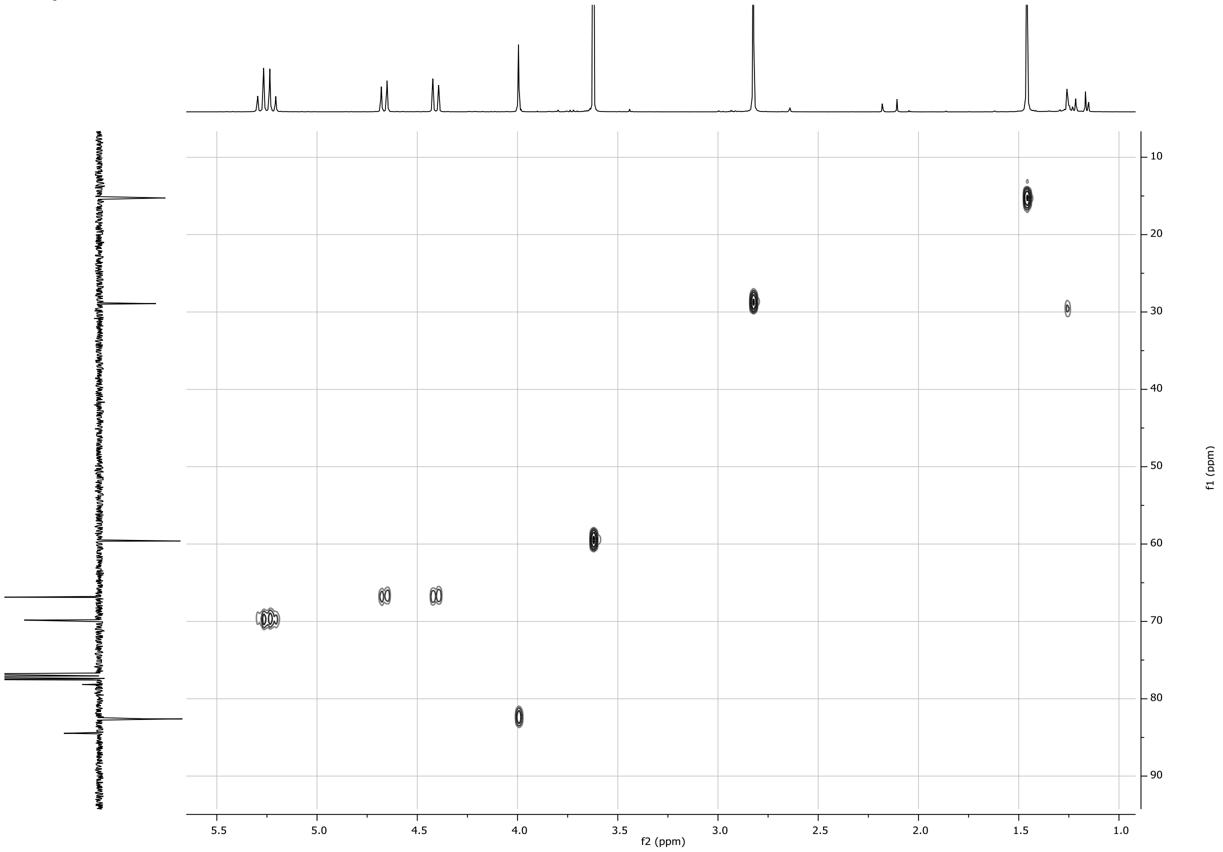

^{1}H NMR, 400 MHz, $CDCl_3$ of **S18**

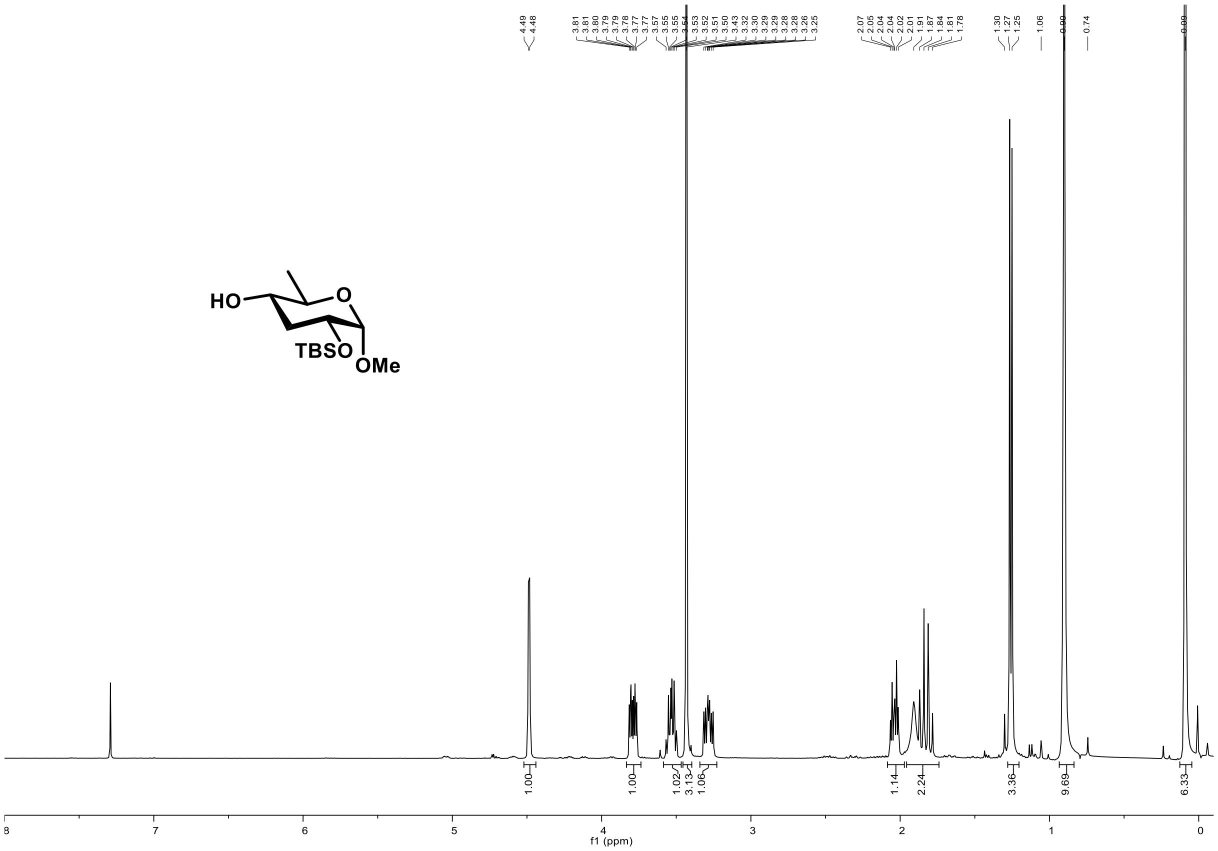

^{13}C NMR, 101 MHz, $CDCl_3$ of **S18**

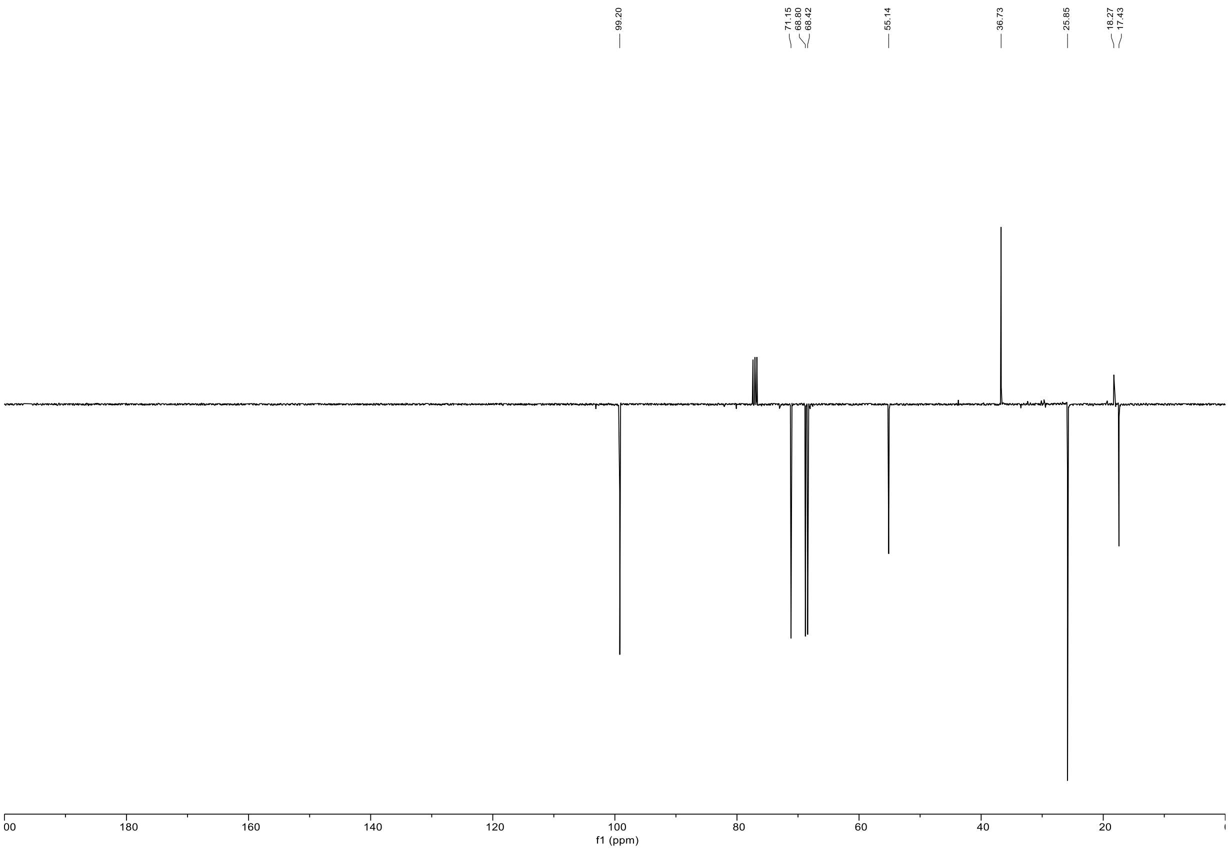

HH-COSY NMR, $CDCl_3$ of **S18**

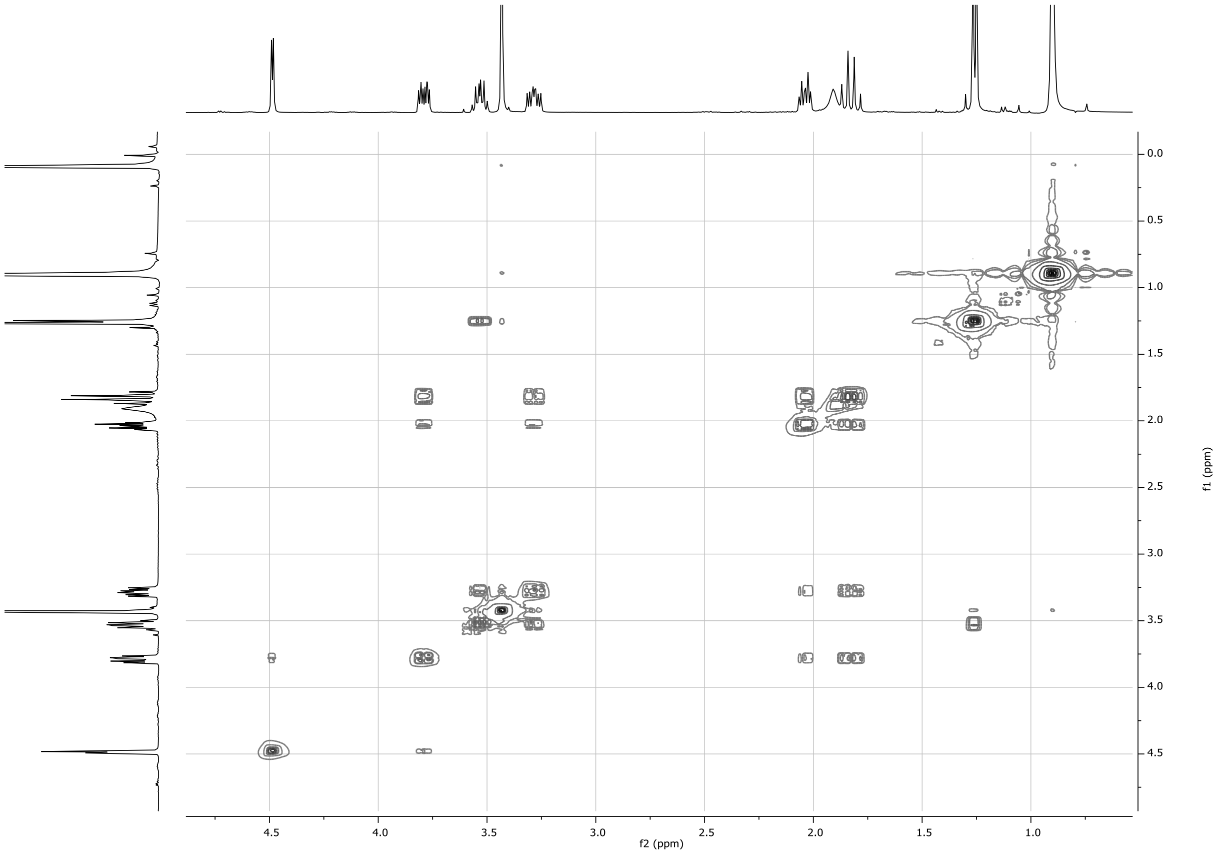

HSQC NMR, $CDCl_3$ of **S18**

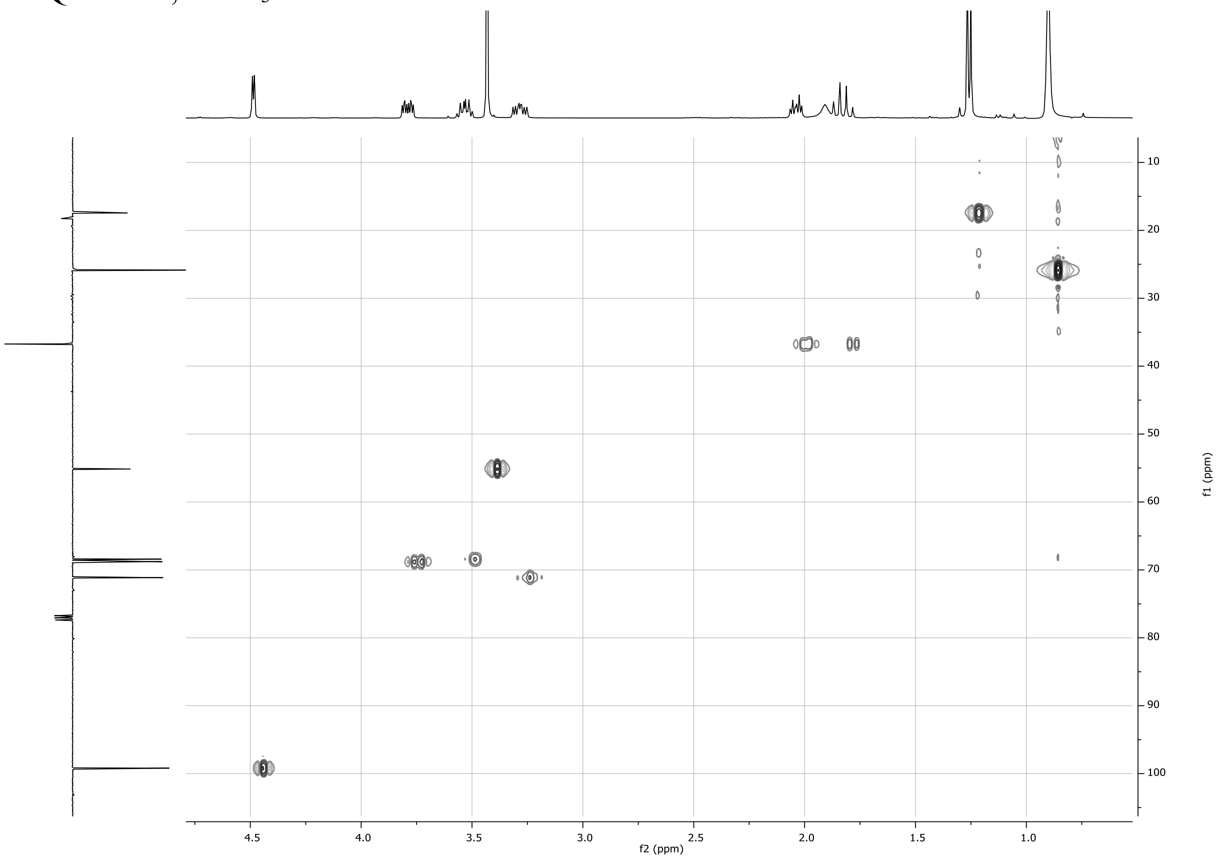

^{1}H NMR, 400 MHz, $CDCl_3$ of **S19**

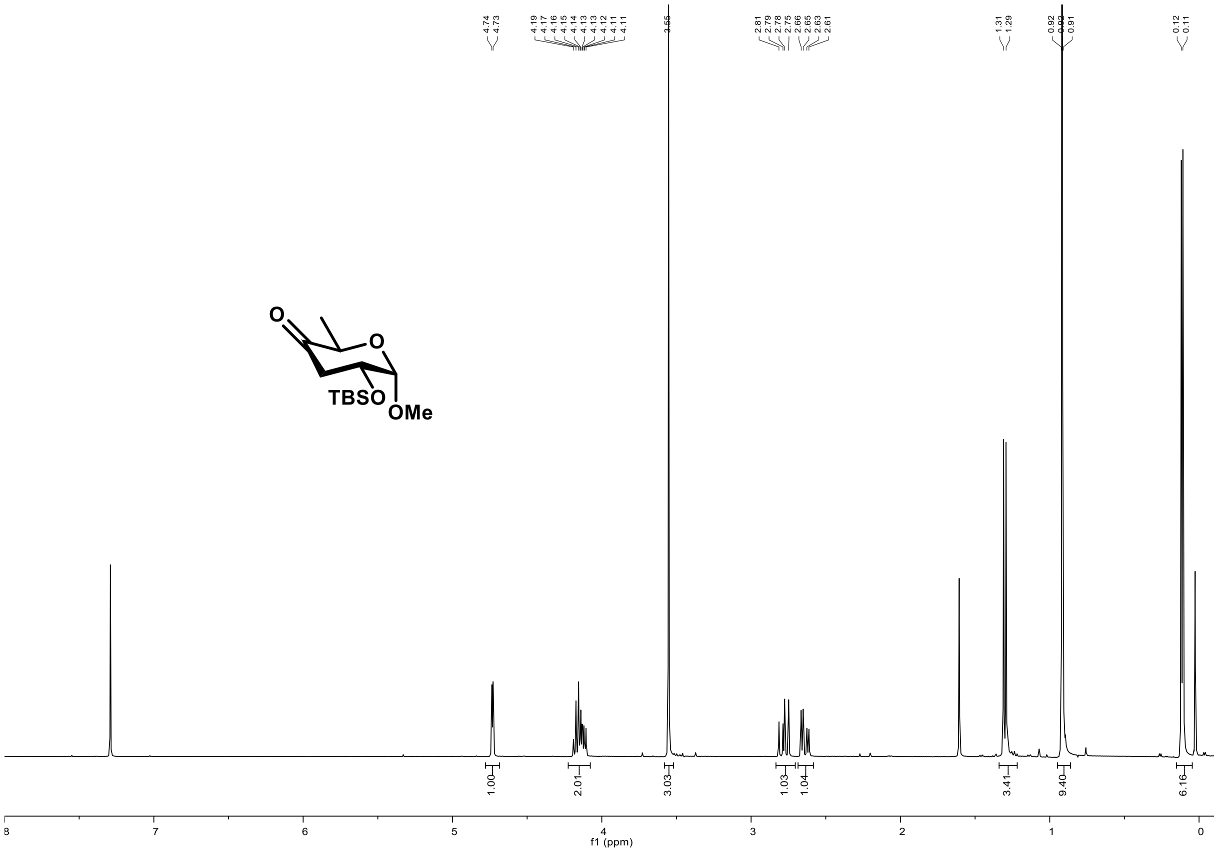

^{13}C NMR, 101 MHz, $CDCl_3$ of **S19**

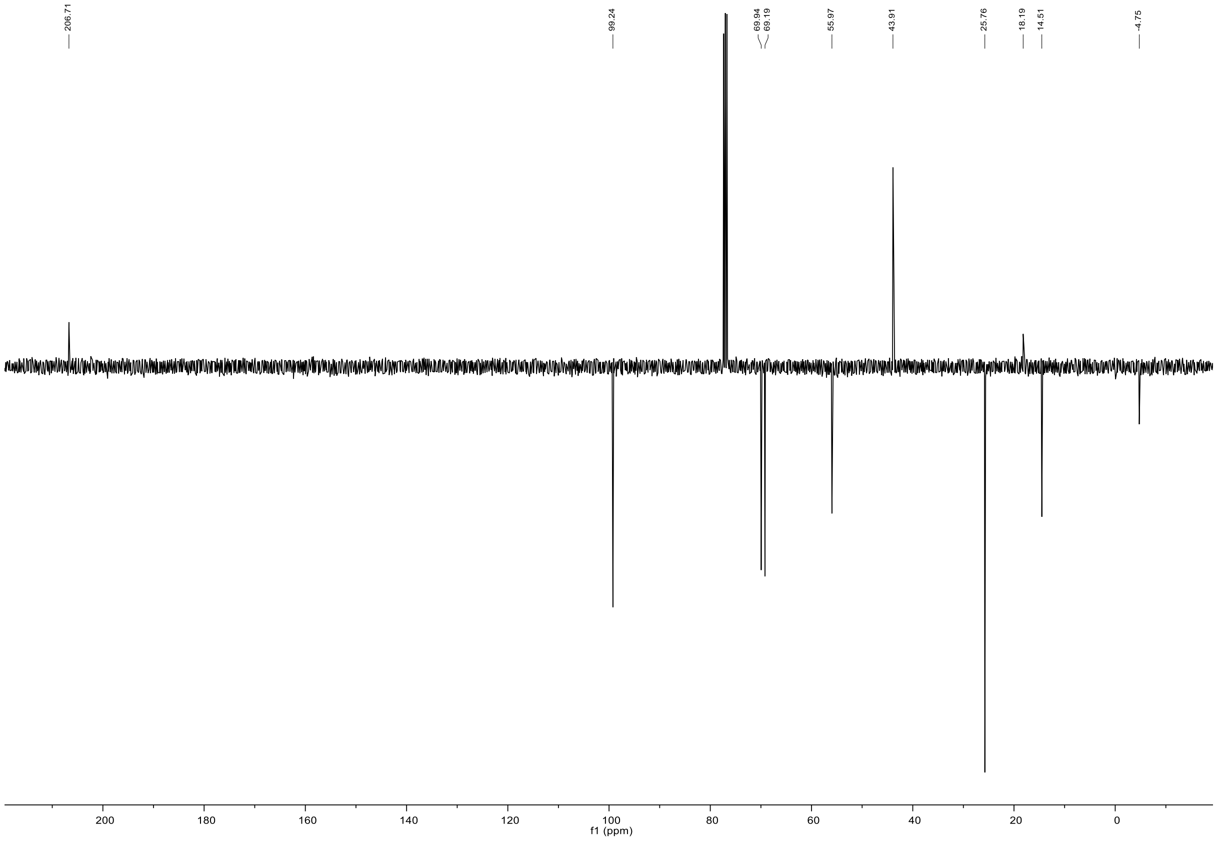

HH-COSY NMR, $CDCl_3$ of **S19**

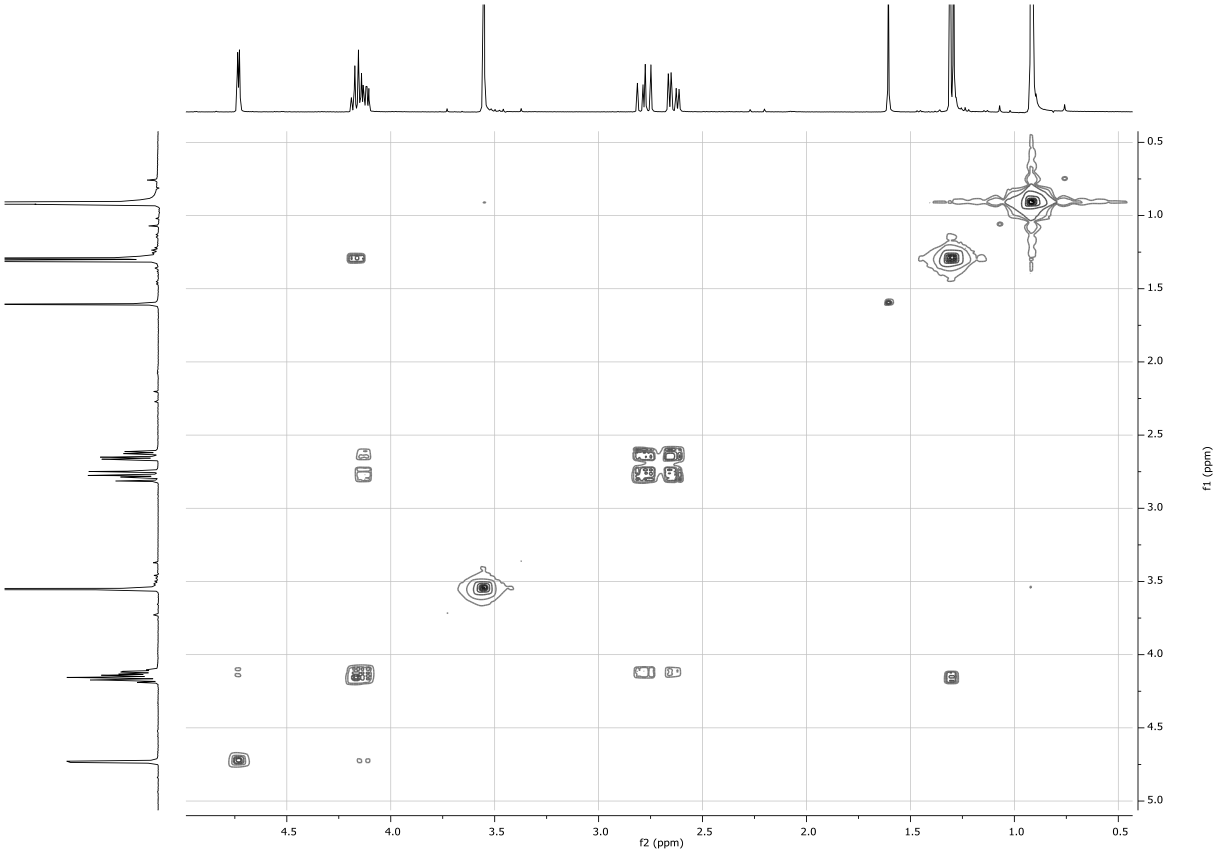

HSQC NMR, $CDCl_3$ of **S19**

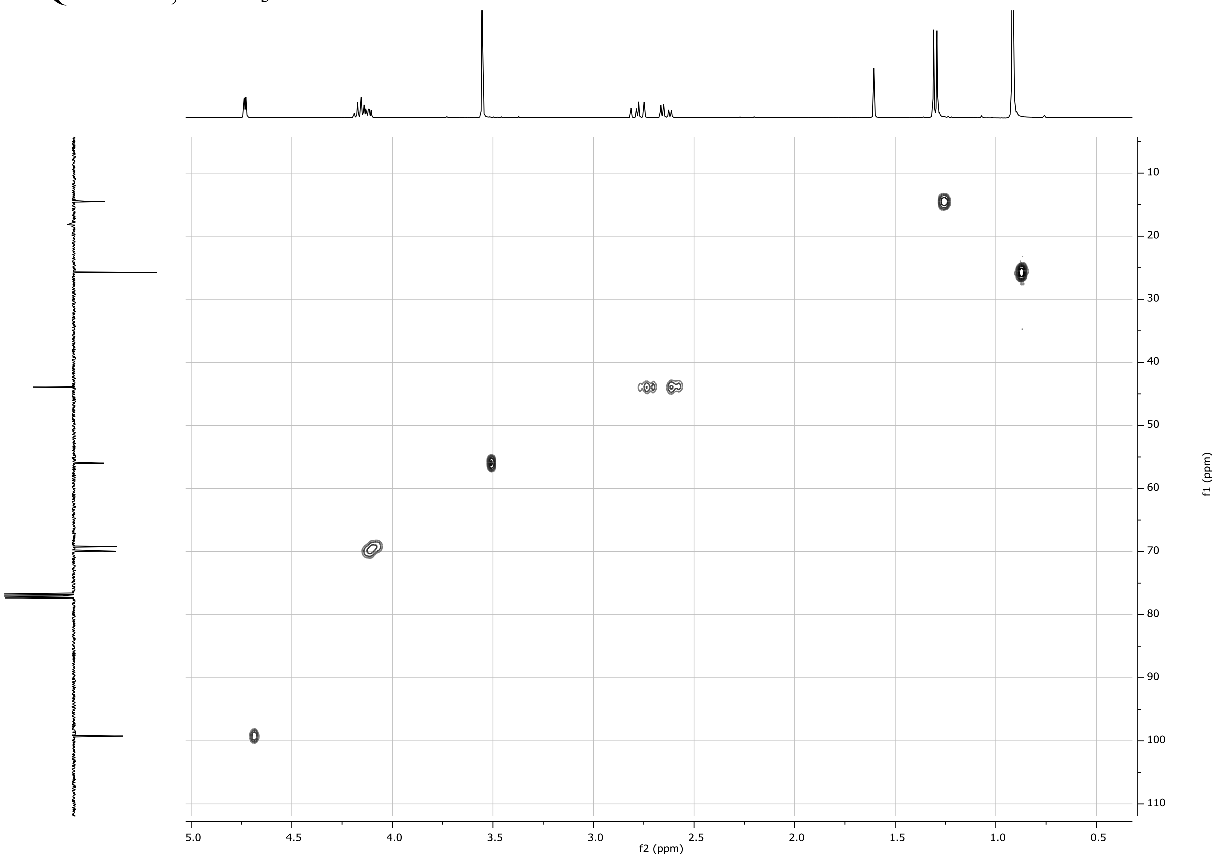

^1H NMR, 400 MHz, $CDCl_3$ of **S20**

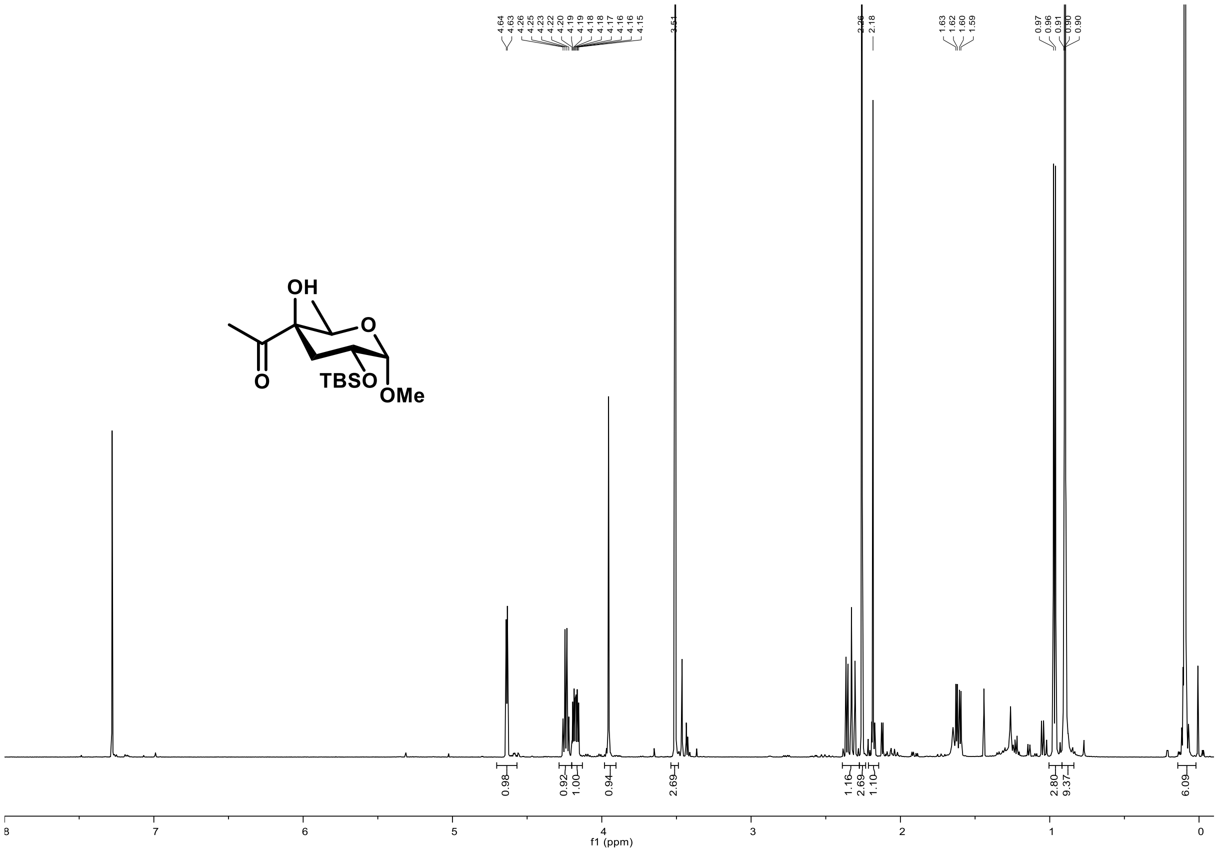

^{13}C NMR, 101 MHz, $CDCl_3$ of **S20**

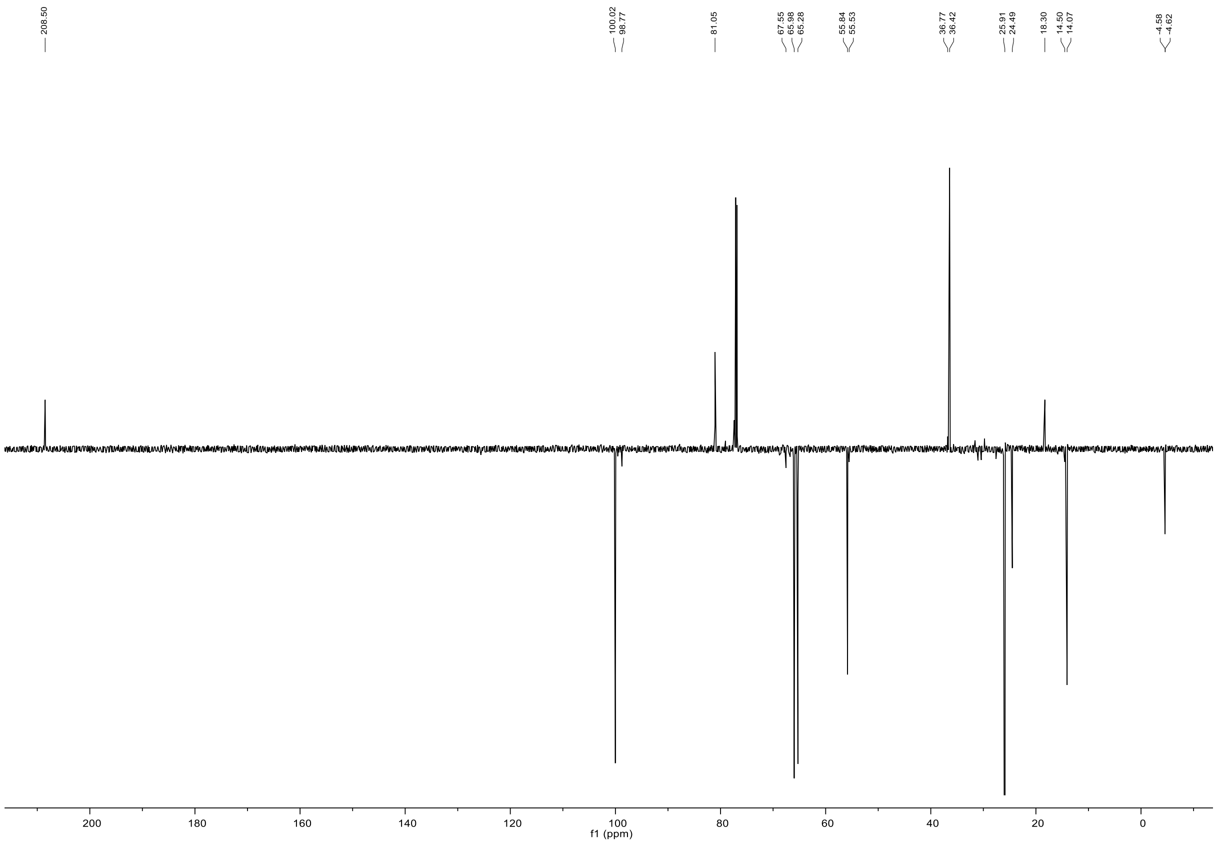

HH-COSY NMR, $CDCl_3$ of **S20**

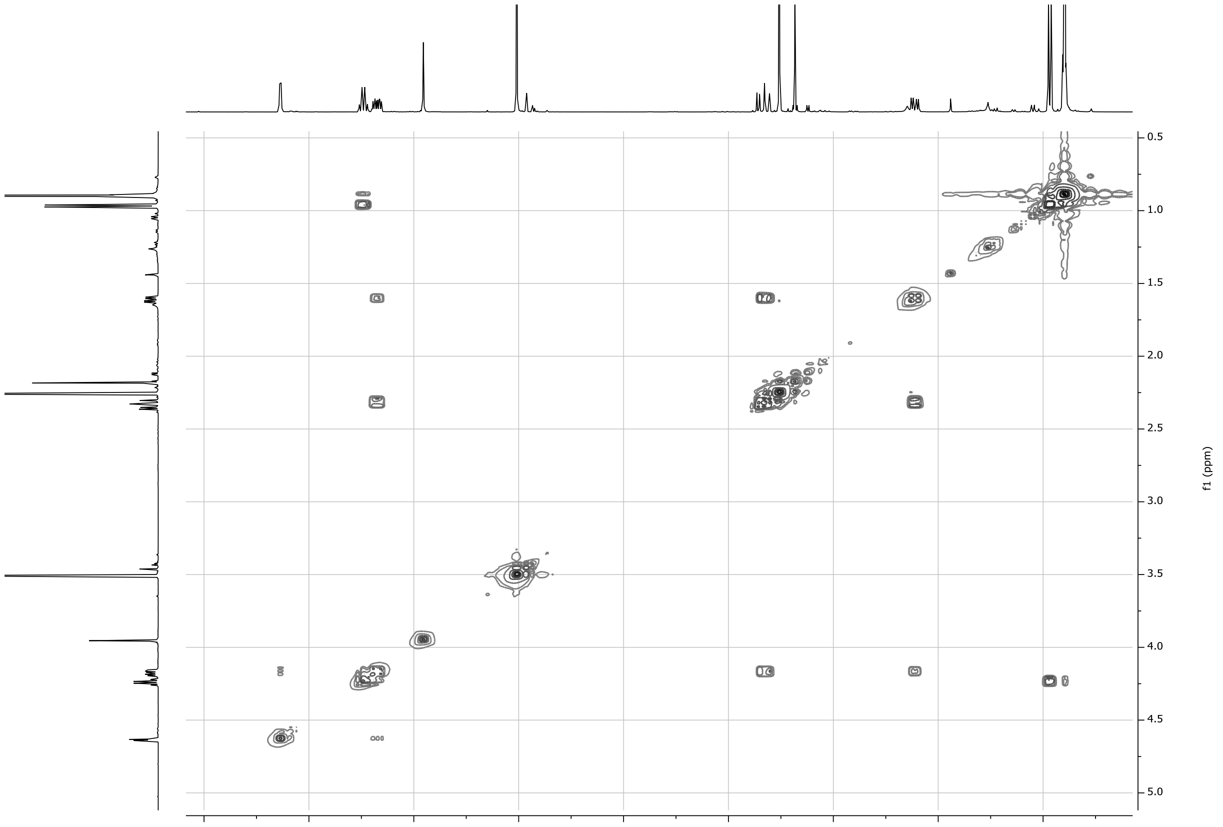

HSQC NMR, $CDCl_3$ of **S20**

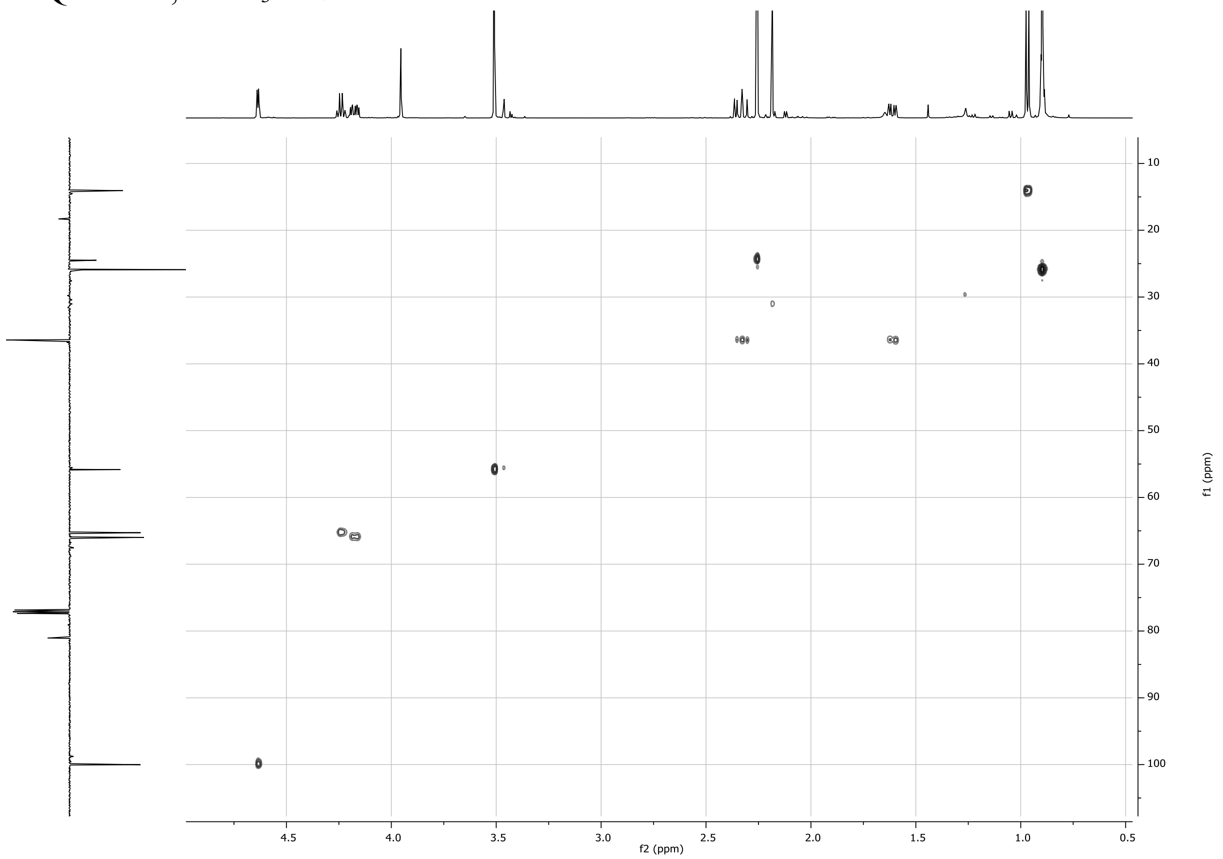

^1H NMR, 400 MHz, $CDCl_3$ of **S21**

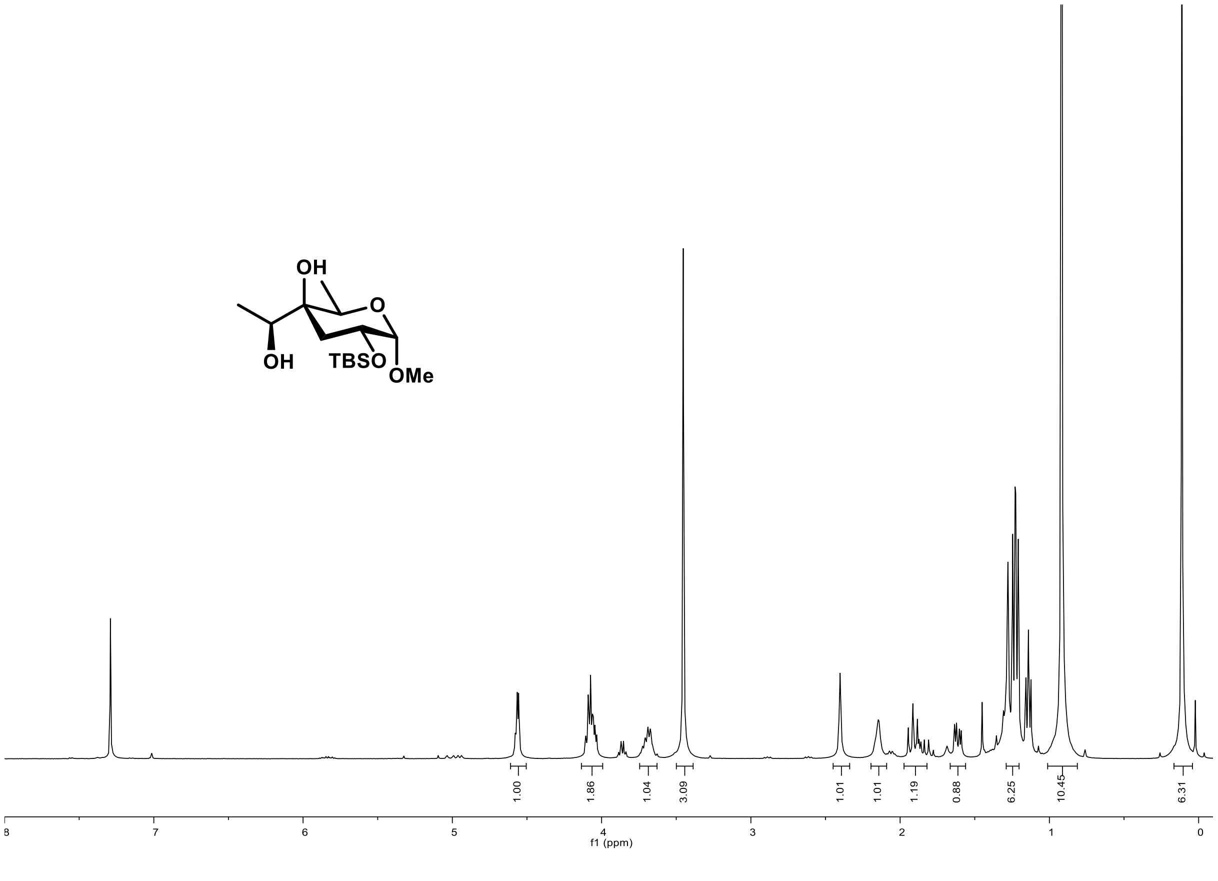

^{13}C NMR, 101 MHz, $CDCl_3$ of **S21**

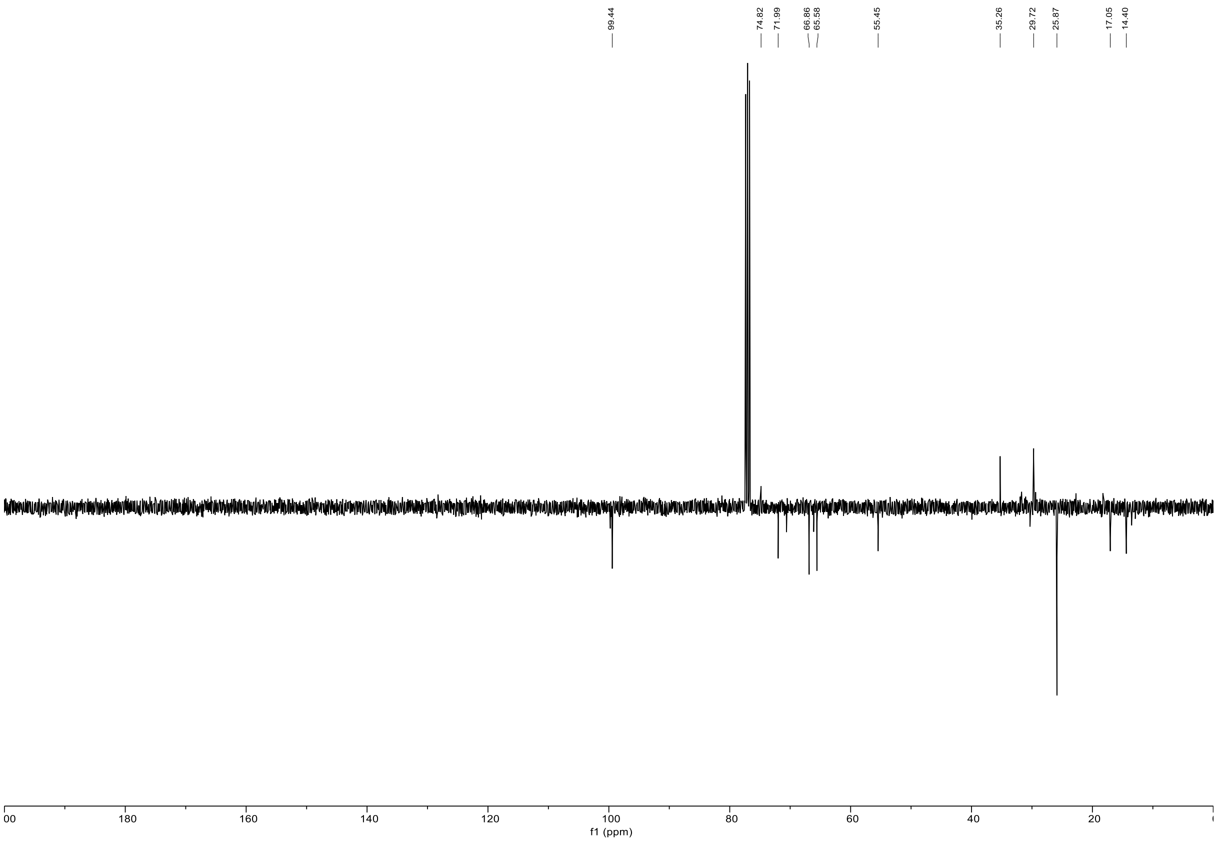

HH-COSY NMR, $CDCl_3$ of **S21**

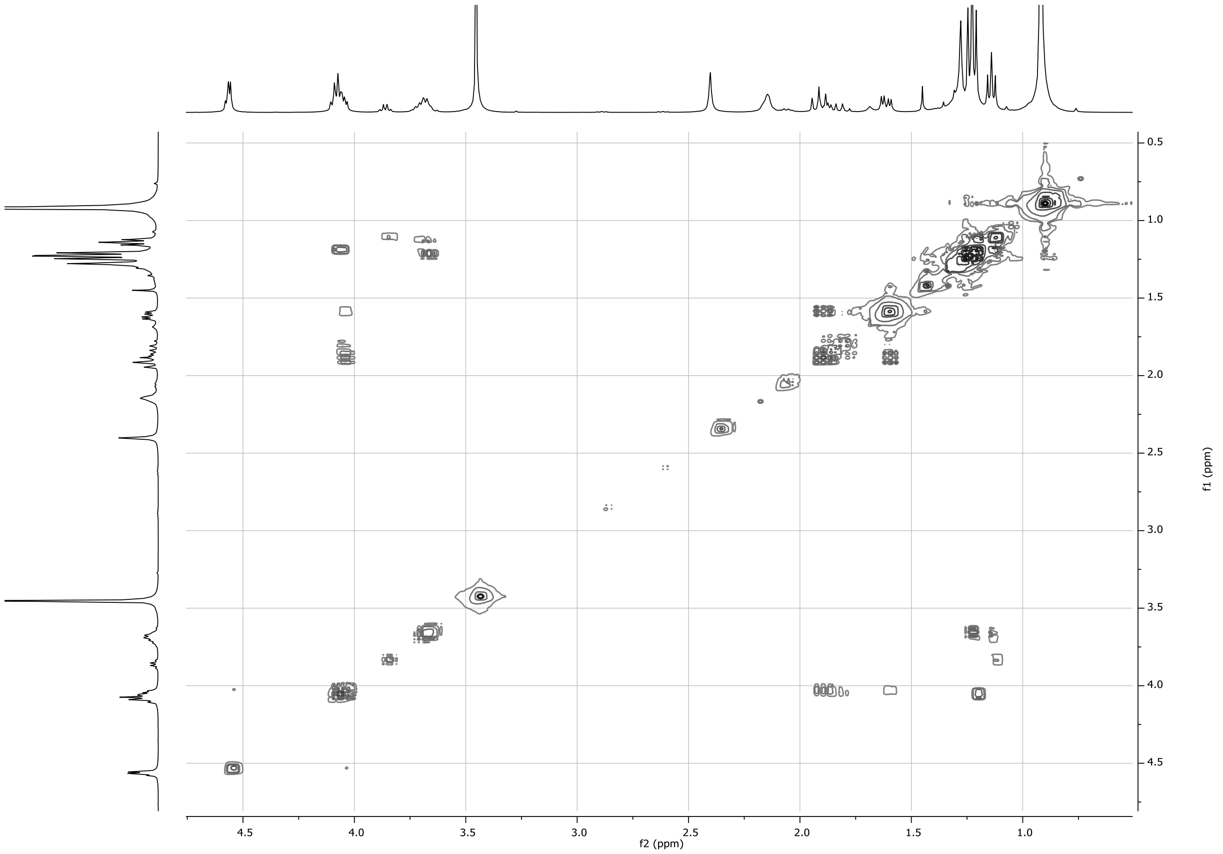

HSQC NMR, $CDCl_3$ of **S21**

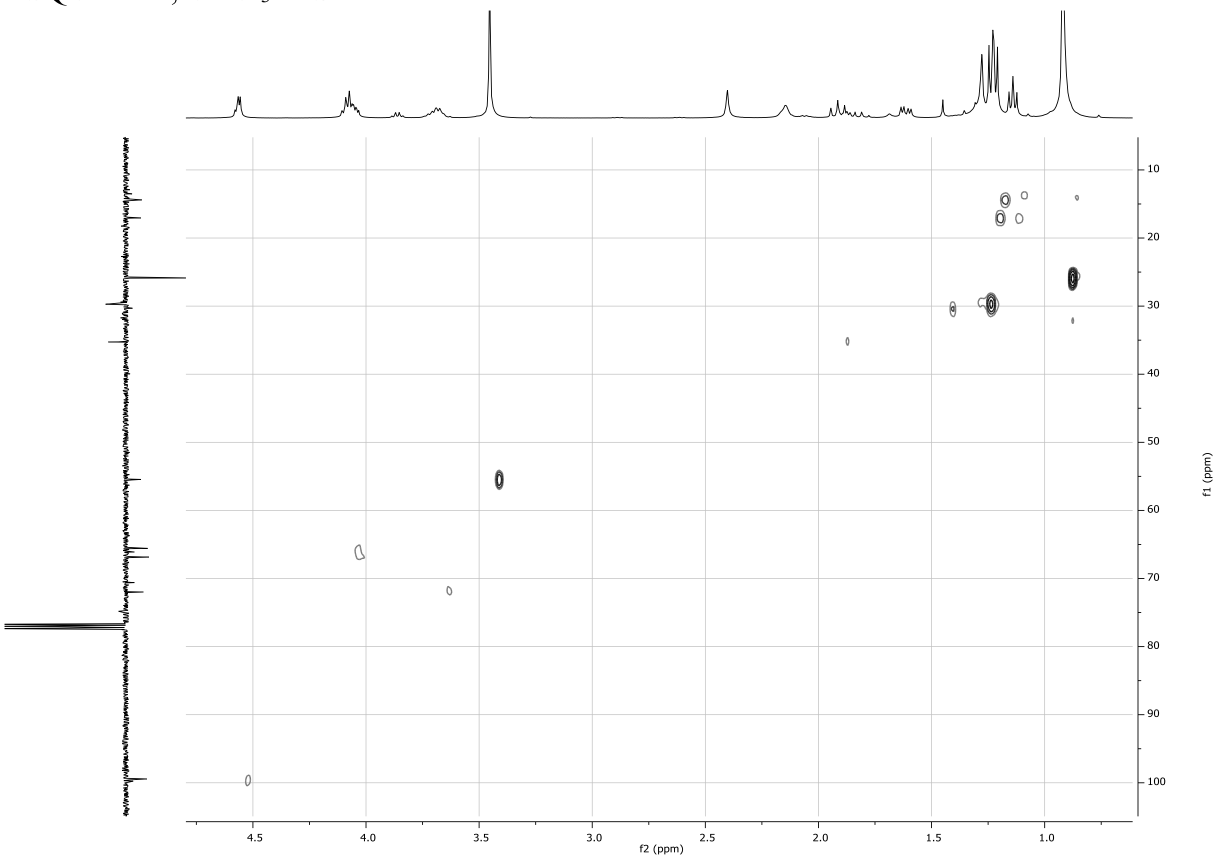

^1H NMR, 400 MHz, $CDCl_3$ of **S22**

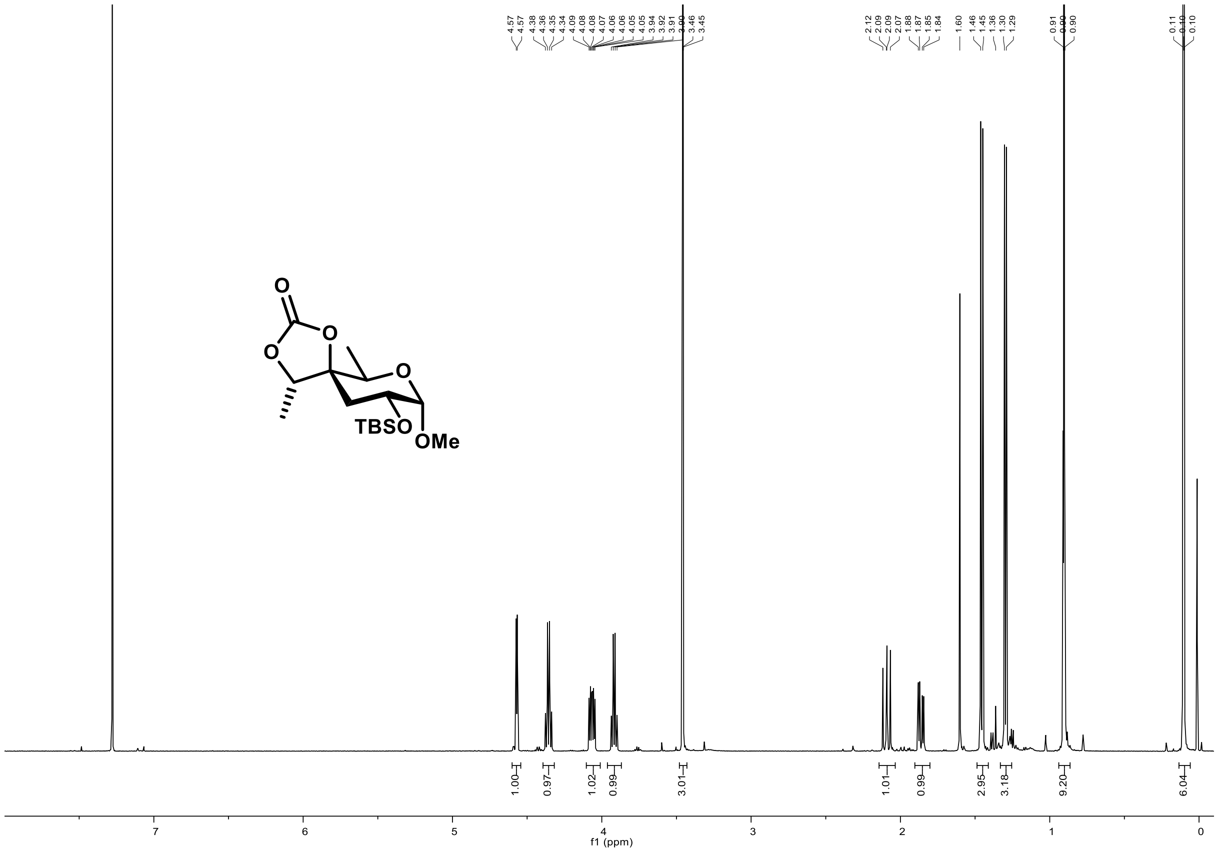

^{13}C NMR, 101 MHz, $CDCl_3$ of **S22**

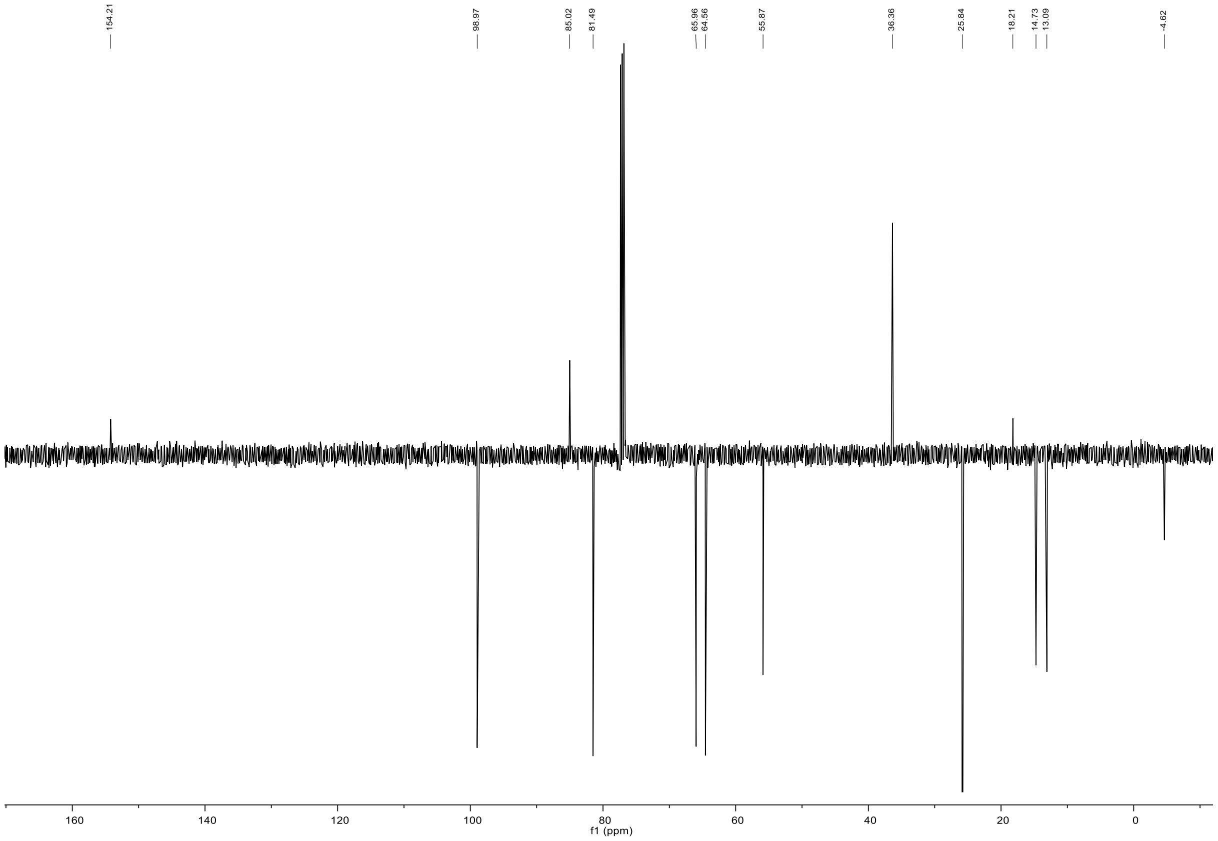

HH-COSY NMR, $CDCl_3$ of **S22**

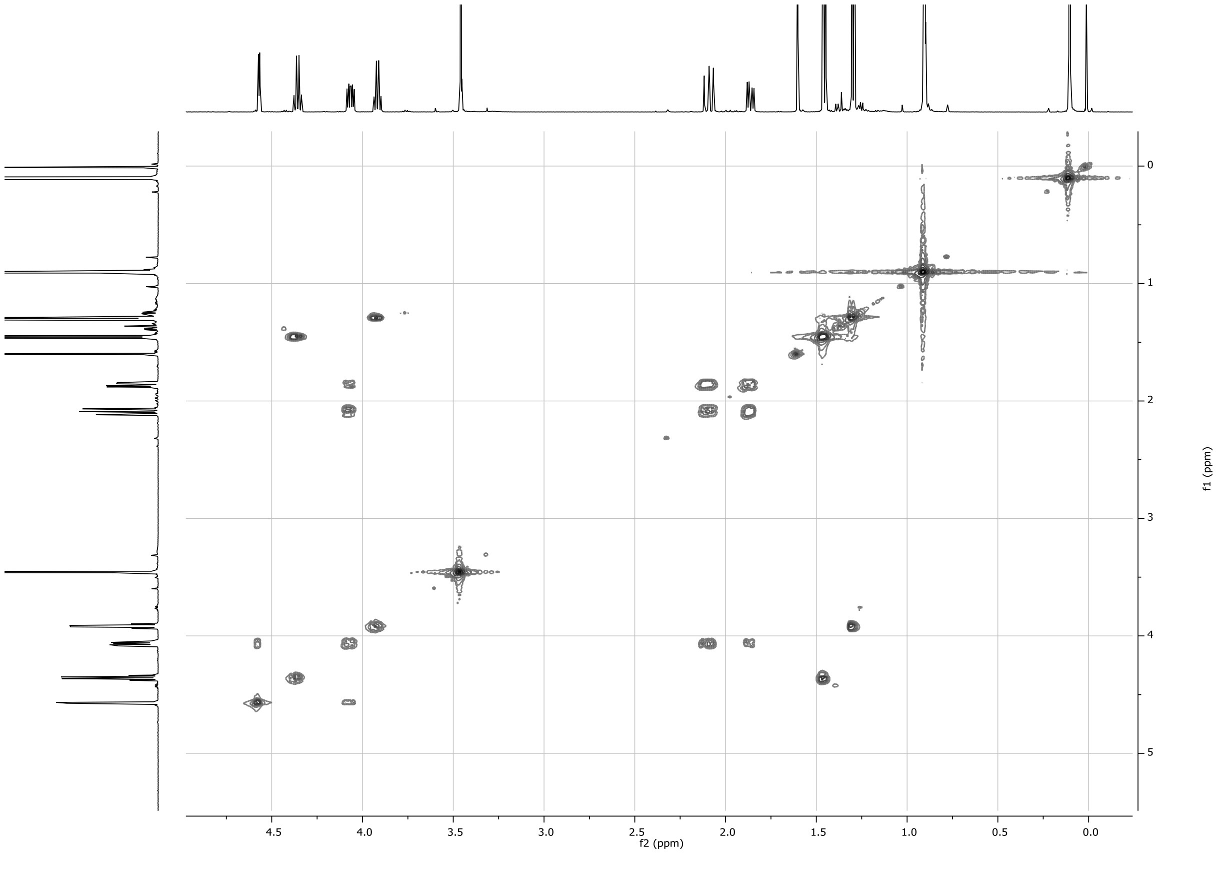

HSQC NMR, $CDCl_3$ of **S22**

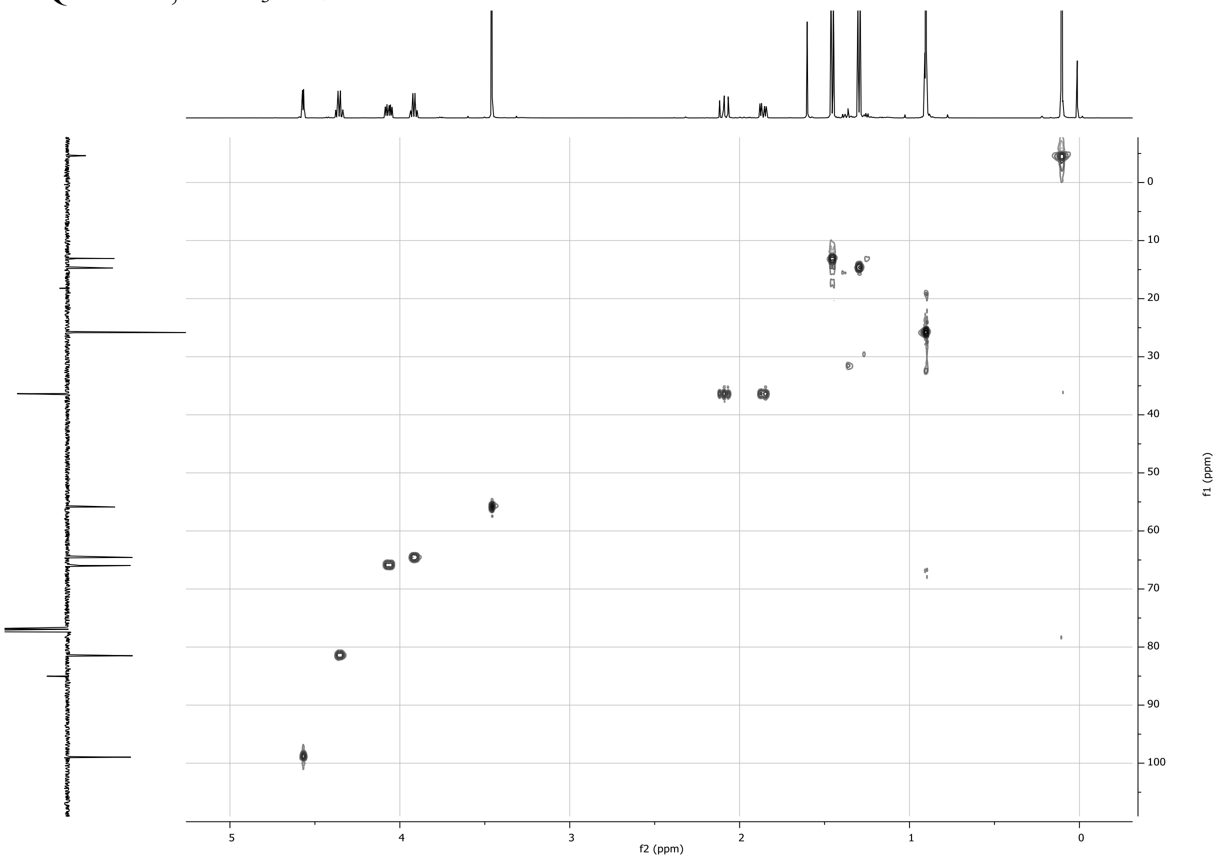

^{1}H NMR, 400 MHz, $CDCl_3$ of **S23**

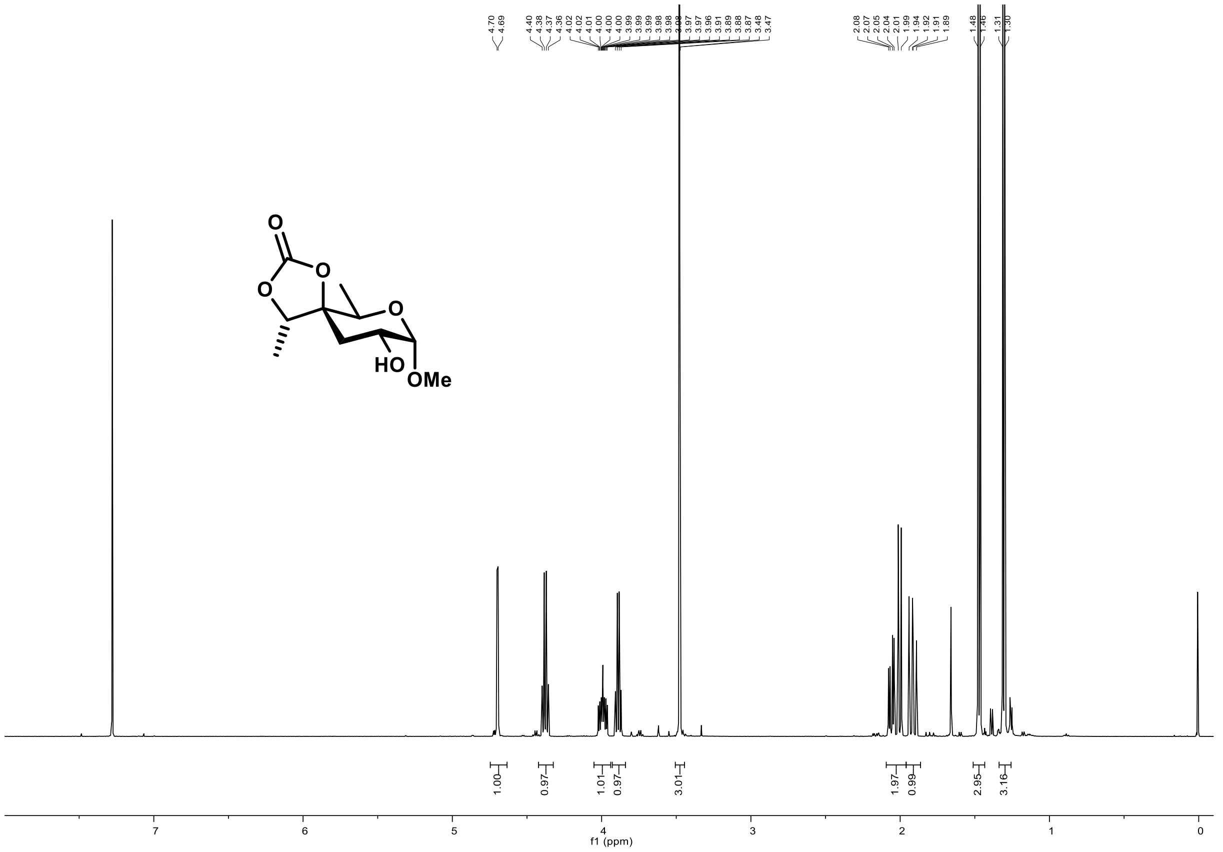

^{13}C NMR, 101 MHz, $CDCl_3$ of **S23**

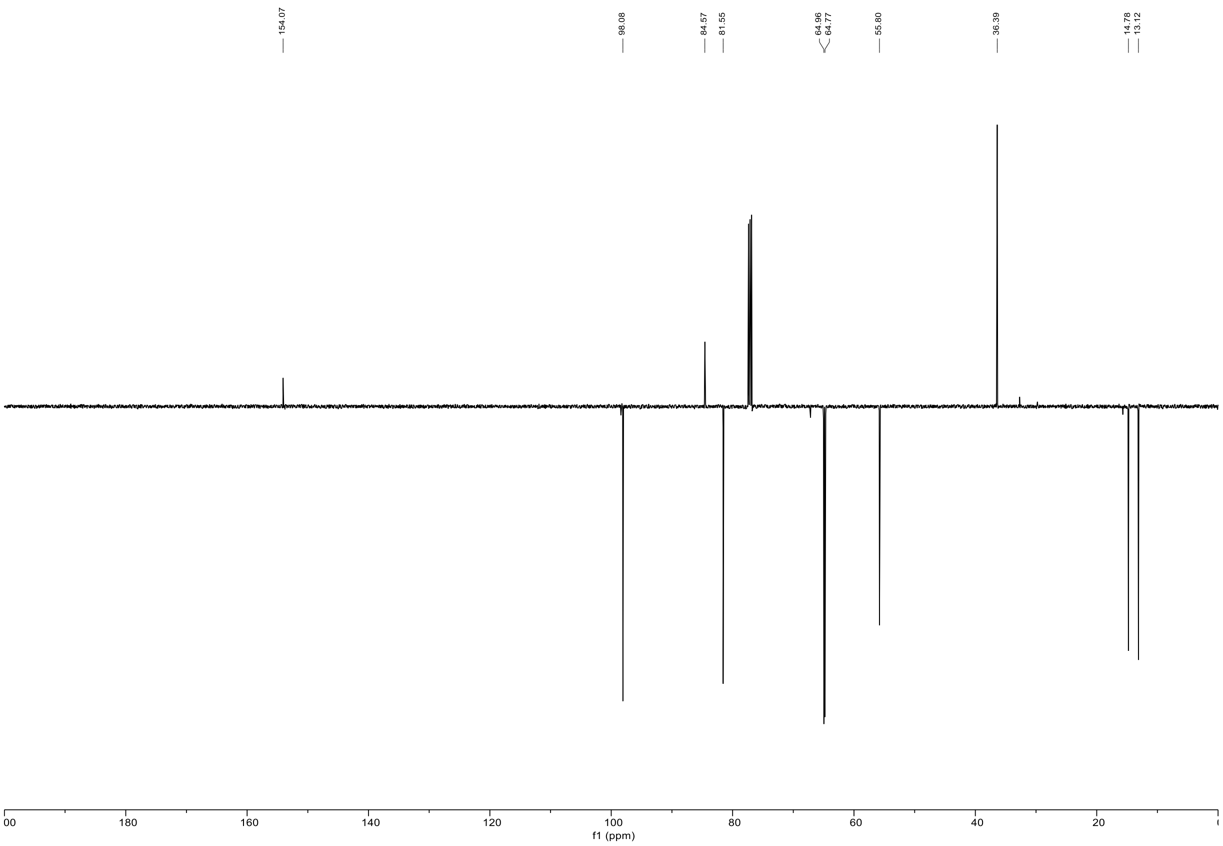

HH-COSY NMR, $CDCl_3$ of **S23**

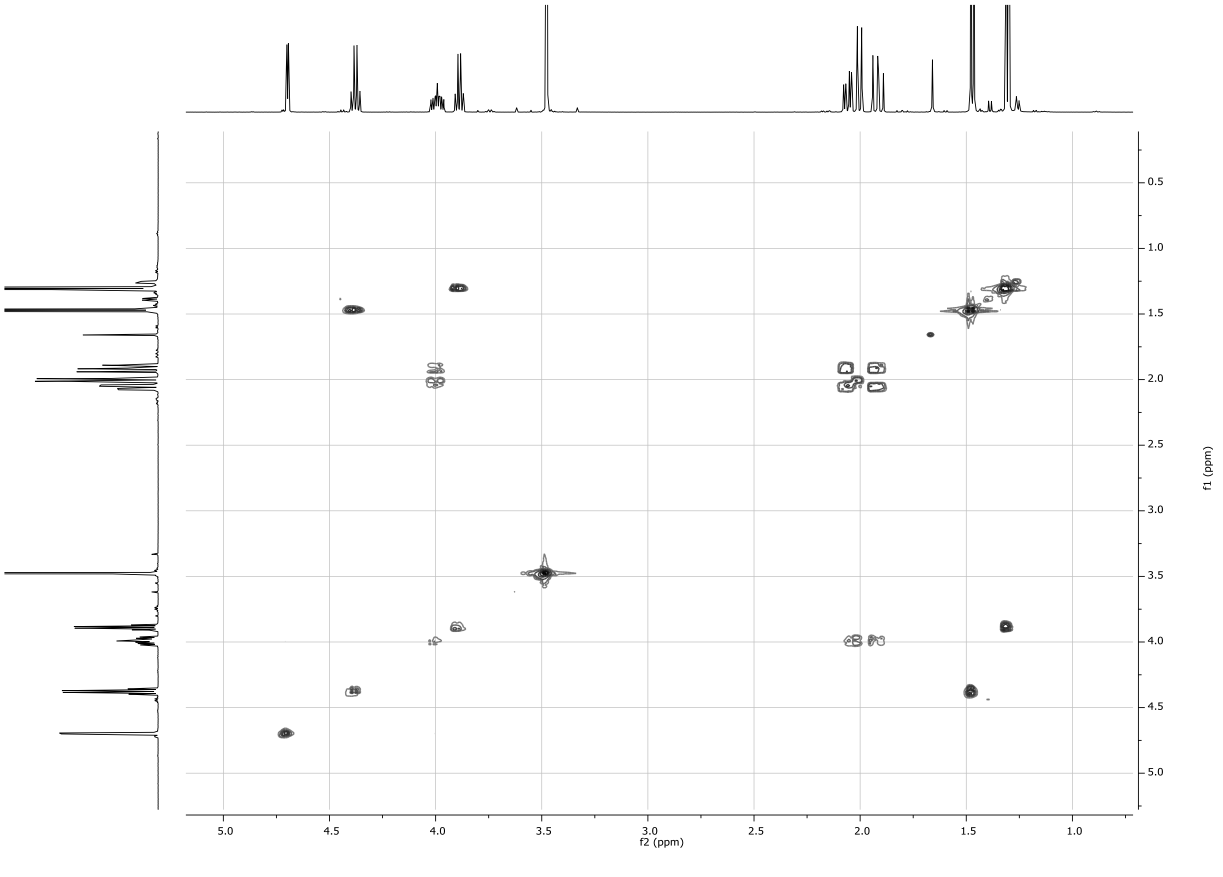

HSQC NMR, $CDCl_3$ of **S23**

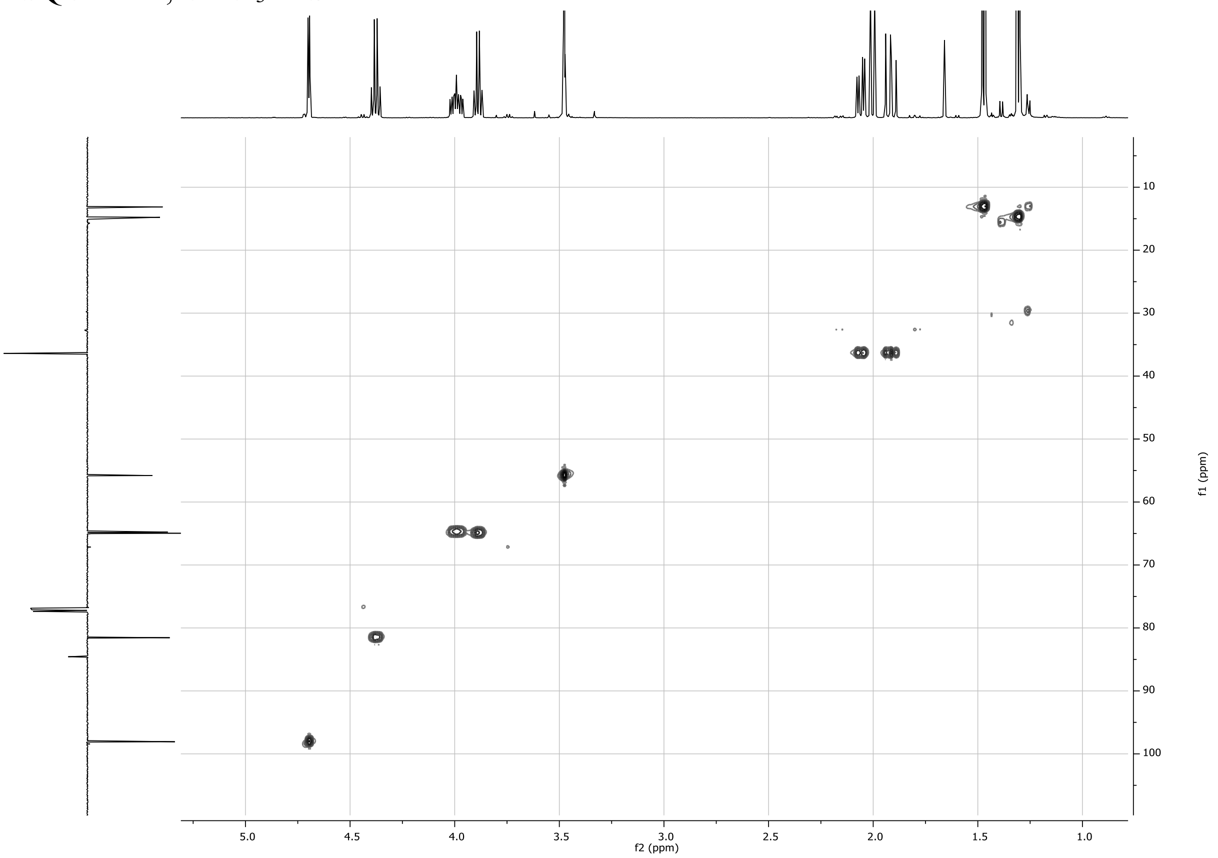

^{1}H NMR, 400 MHz, $CDCl_3$ of **S24**

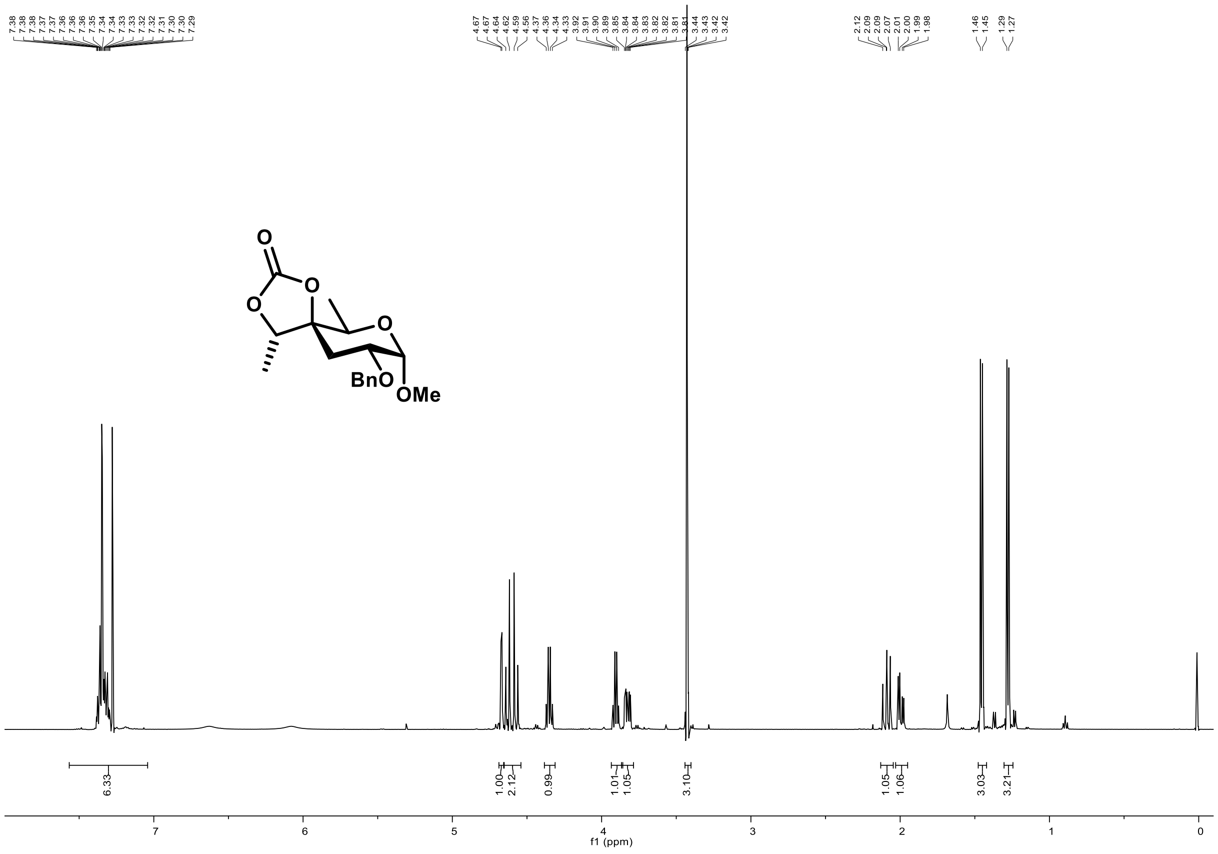

^{13}C NMR, 101 MHz, $CDCl_3$ of **S24**

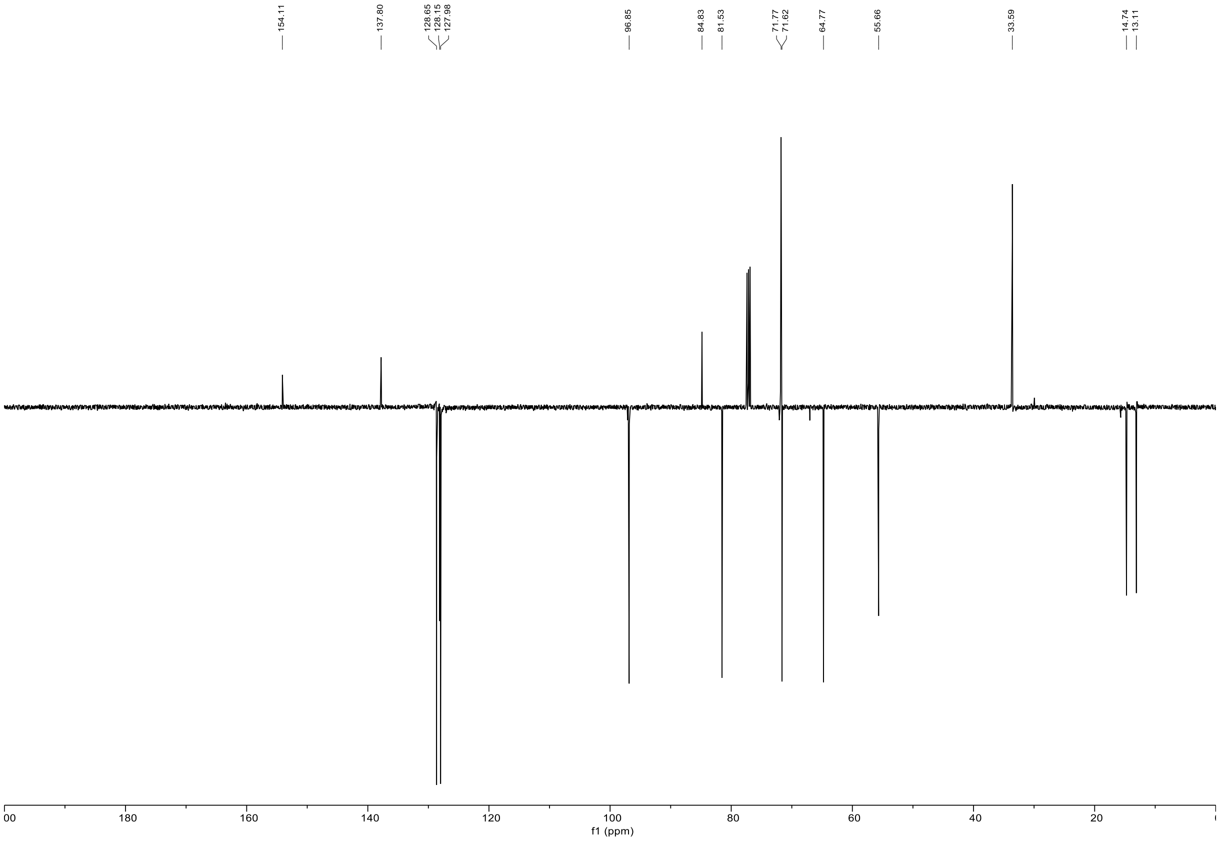

HH-COSY NMR, $CDCl_3$ of **S24**

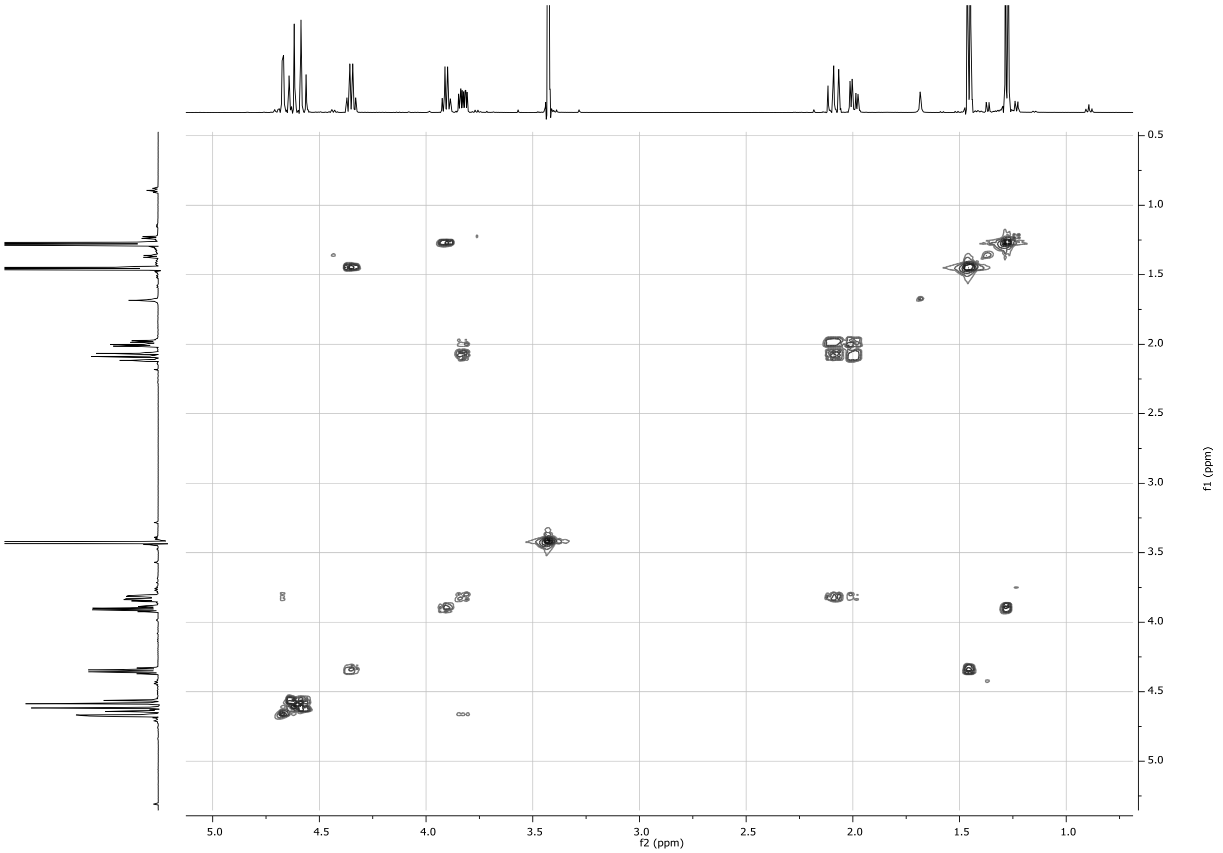

HSQC NMR, $CDCl_3$ of **S24**

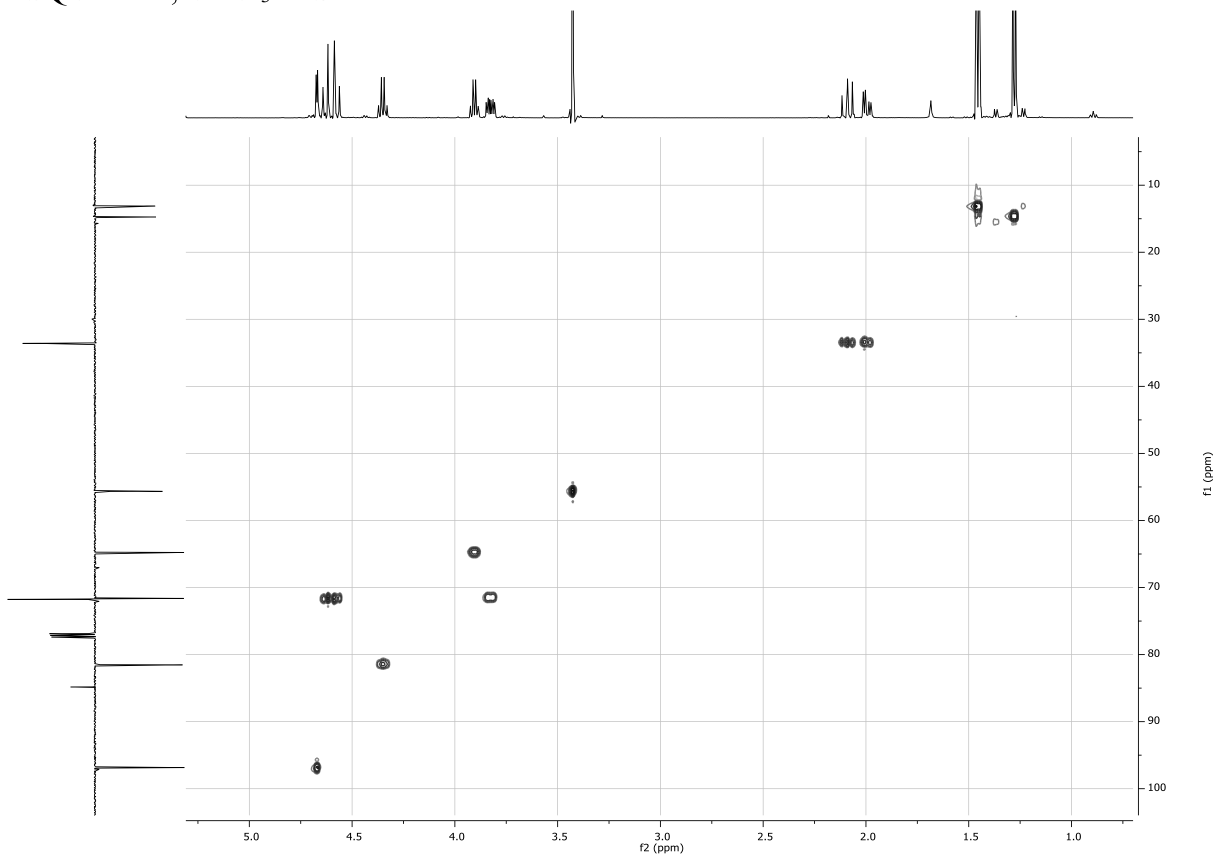

^{1}H NMR, 400 MHz, $CDCl_3$ of **S25**

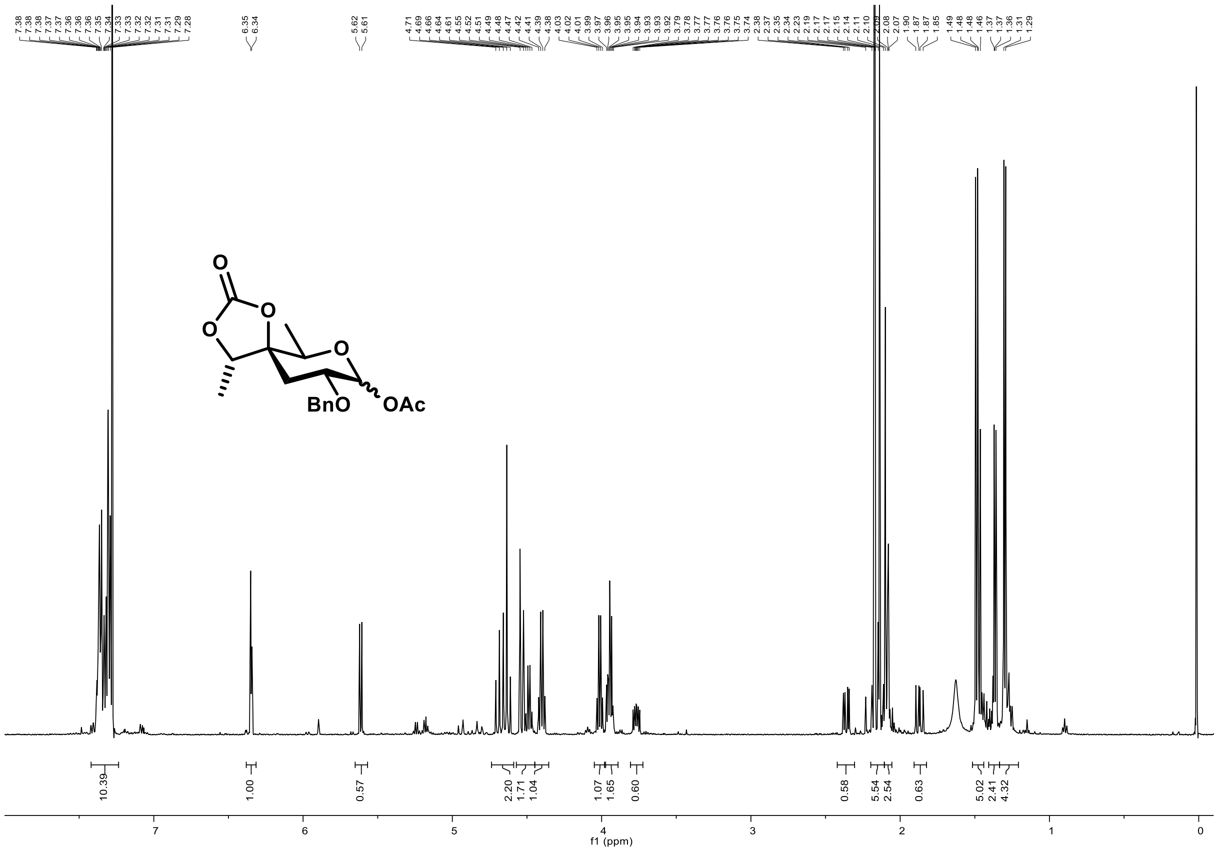

^{13}C NMR, 101 MHz, $CDCl_3$ of **S25**

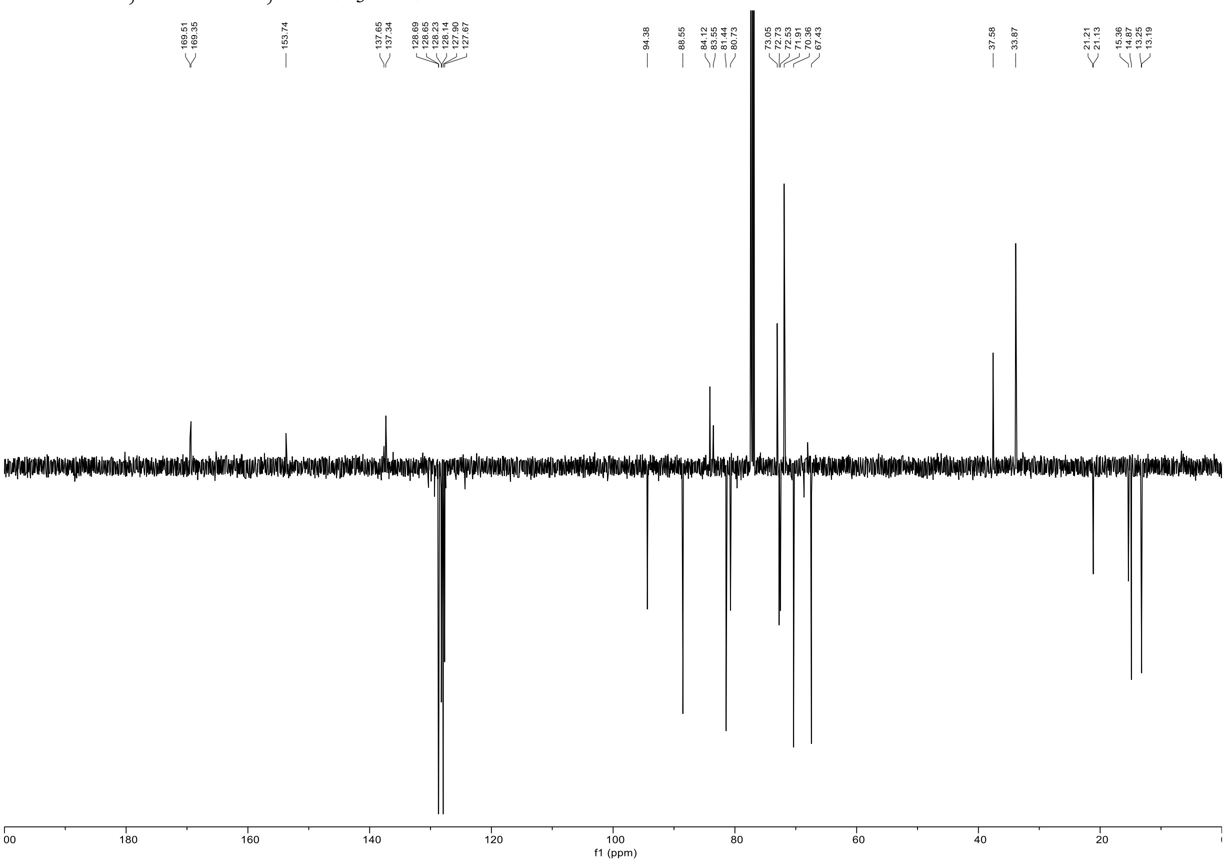

HH-COSY NMR, $CDCl_3$ of **S25**

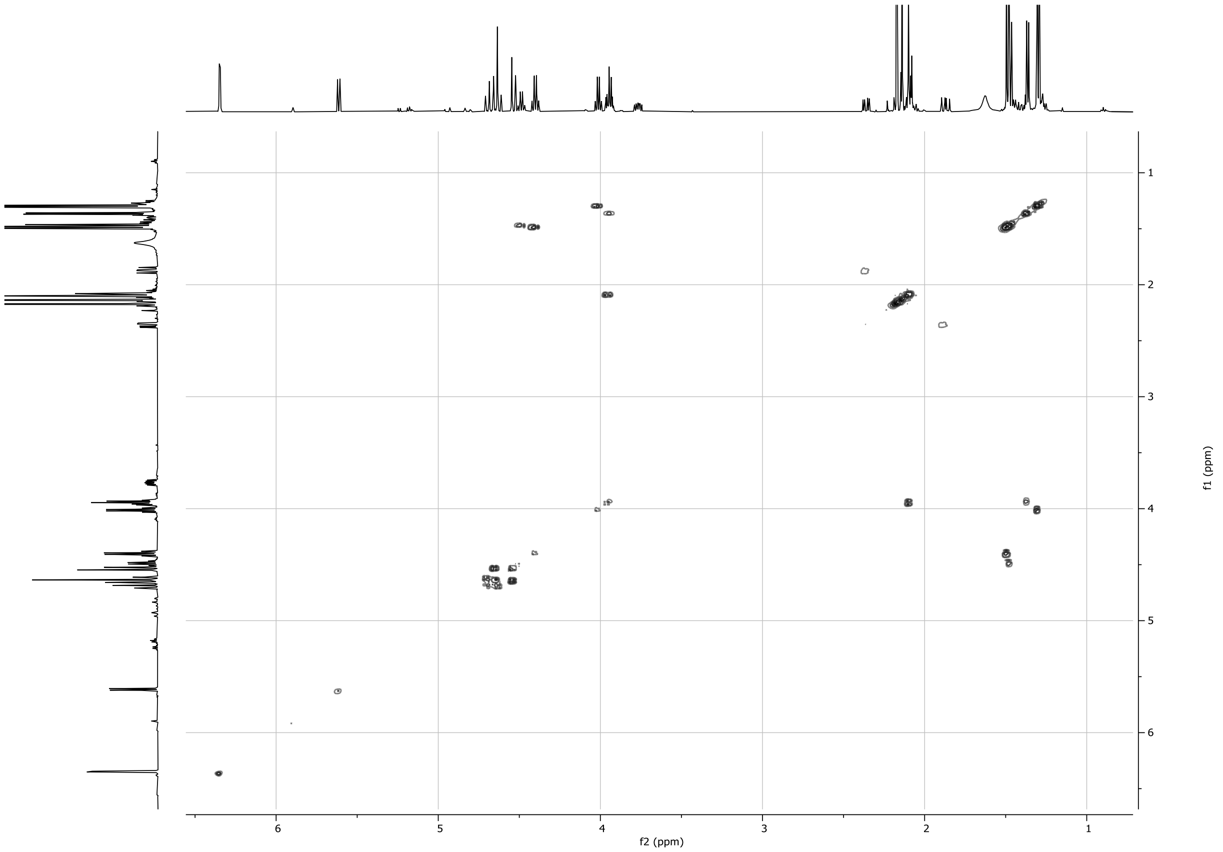

HSQC NMR, $CDCl_3$ of **S25**

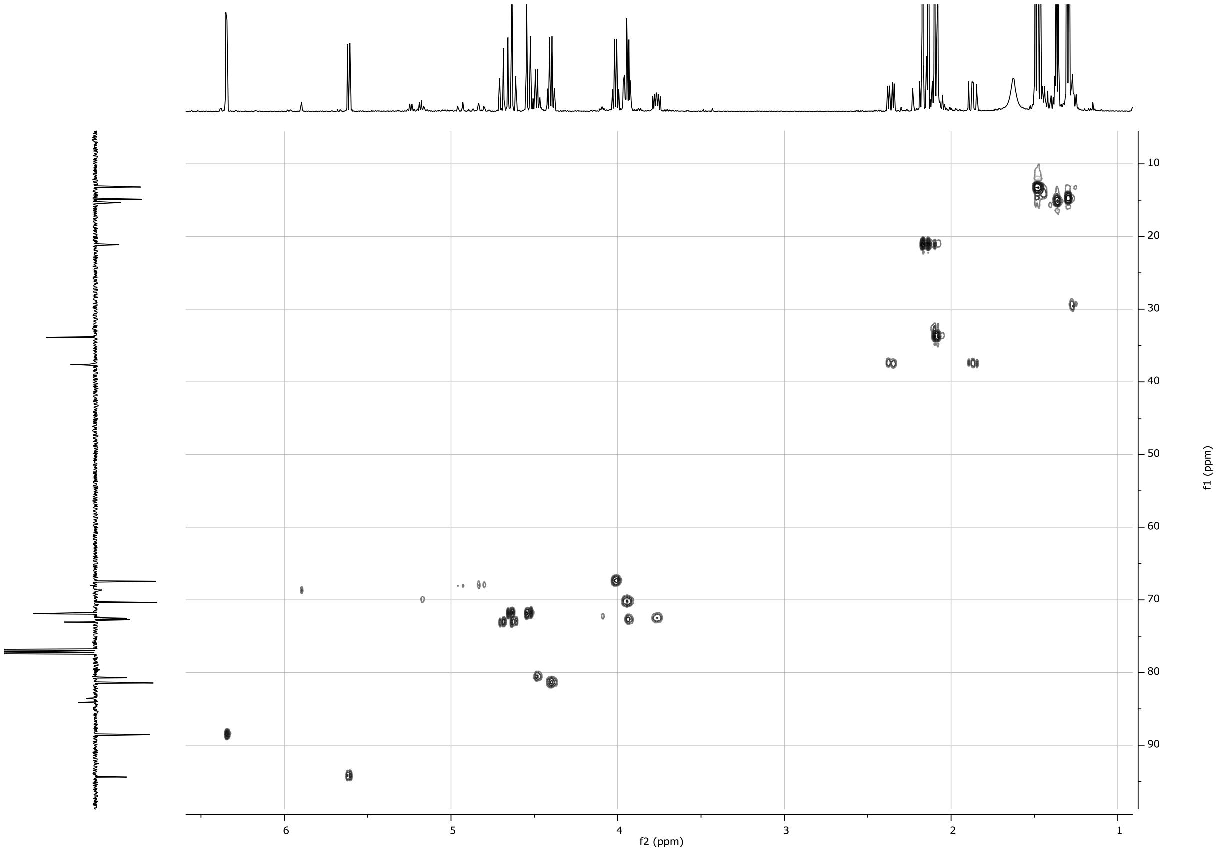

^{1}H NMR, 400 MHz, $CDCl_3$ of **23**

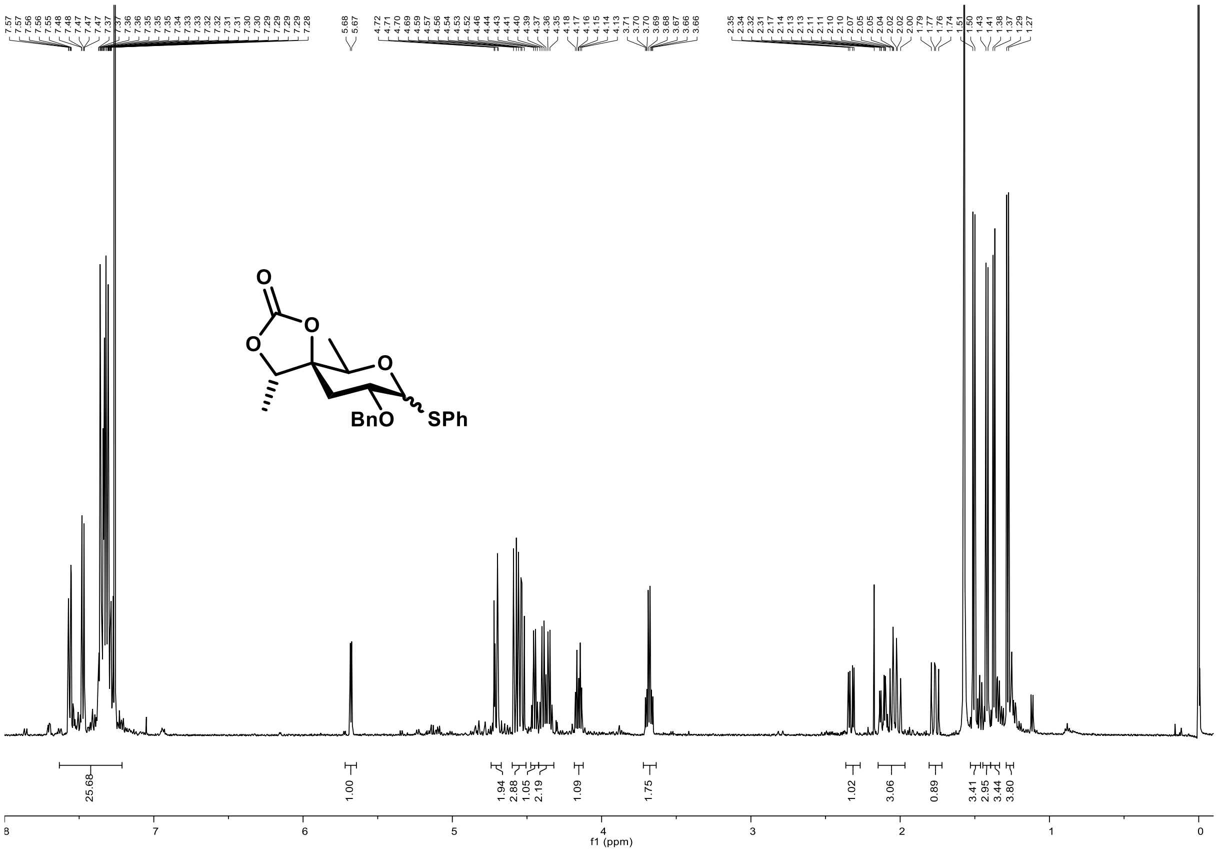

^{13}C NMR, 101 MHz, $CDCl_3$ of **23**

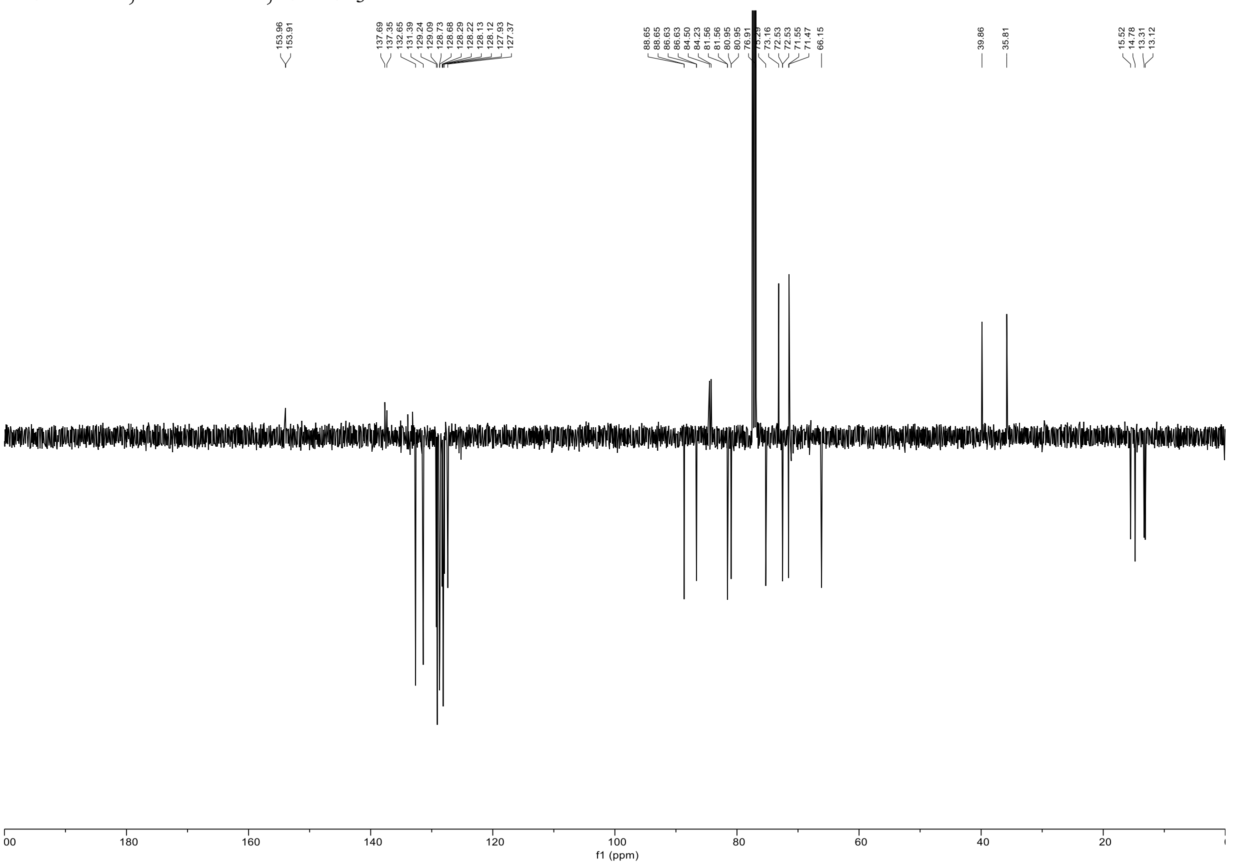

HH-COSY NMR, $CDCl_3$ of **23**

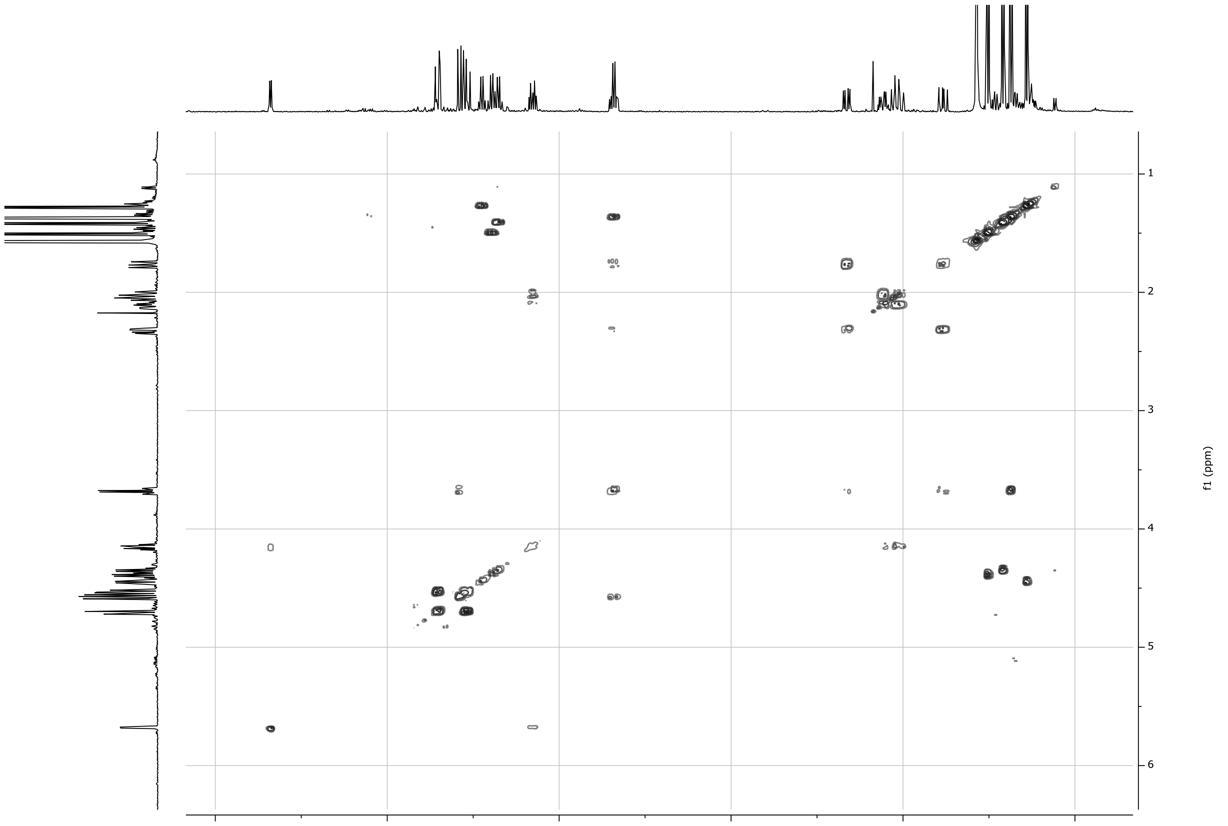

HSQC NMR, CDCl$_3$ of **23**

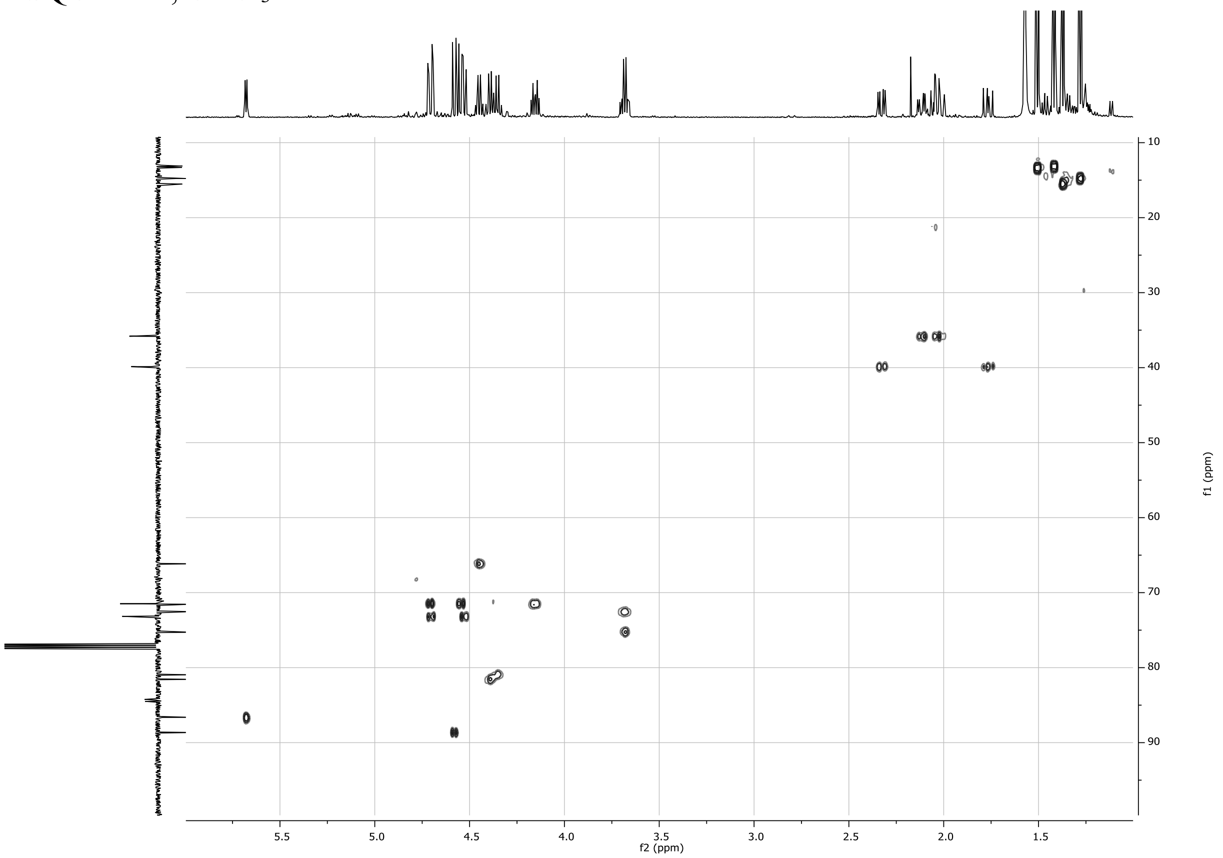

^{1}H NMR, 400 MHz, CDCl$_3$ of **S26**

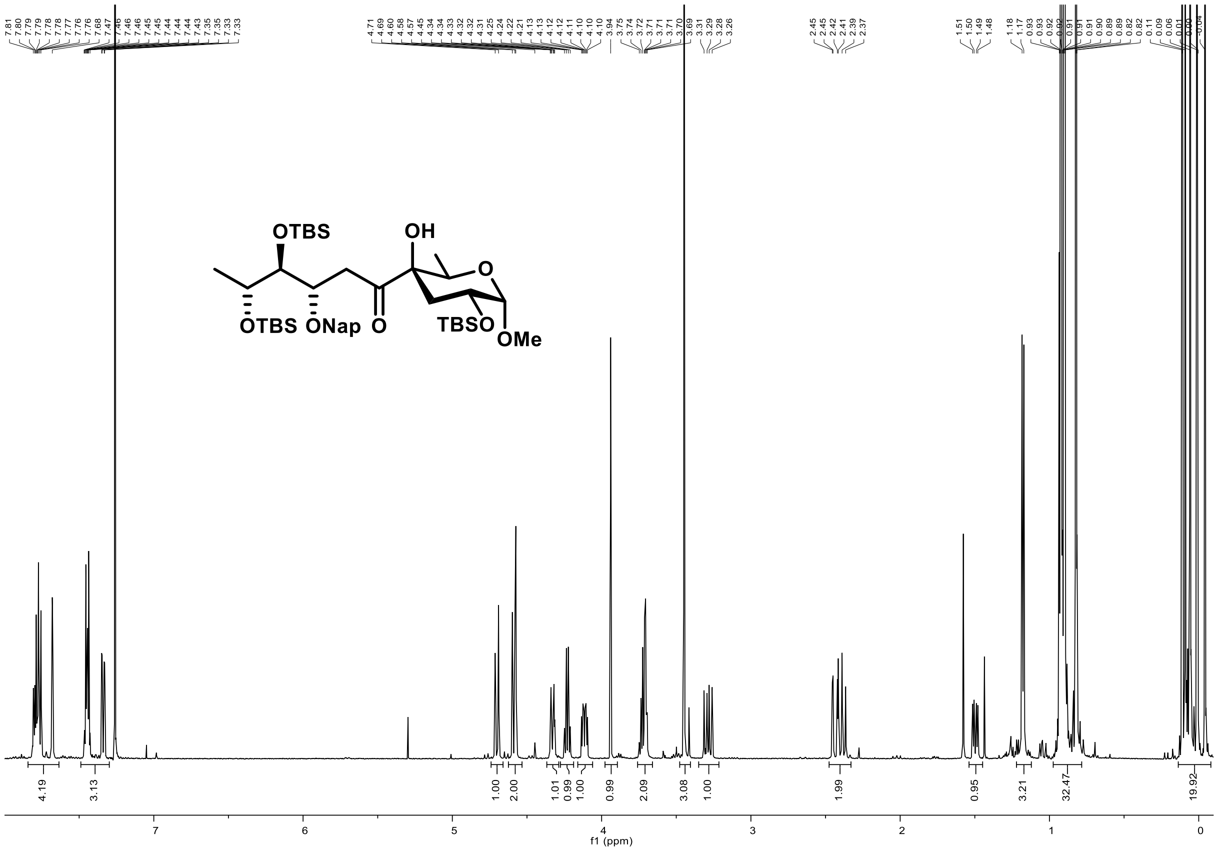

^{13}C NMR, 101 MHz, $CDCl_3$ of **S26**

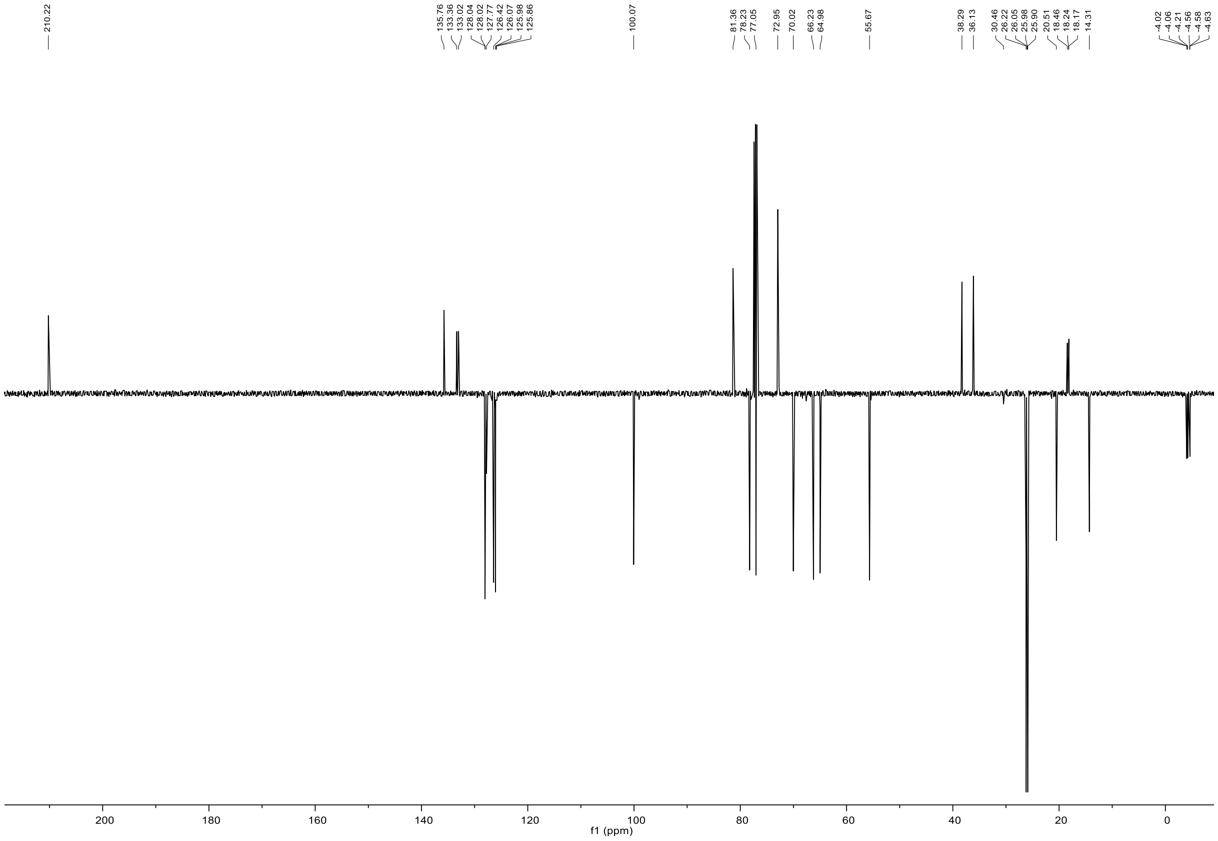

HH-COSY NMR, $CDCl_3$ of **S26**

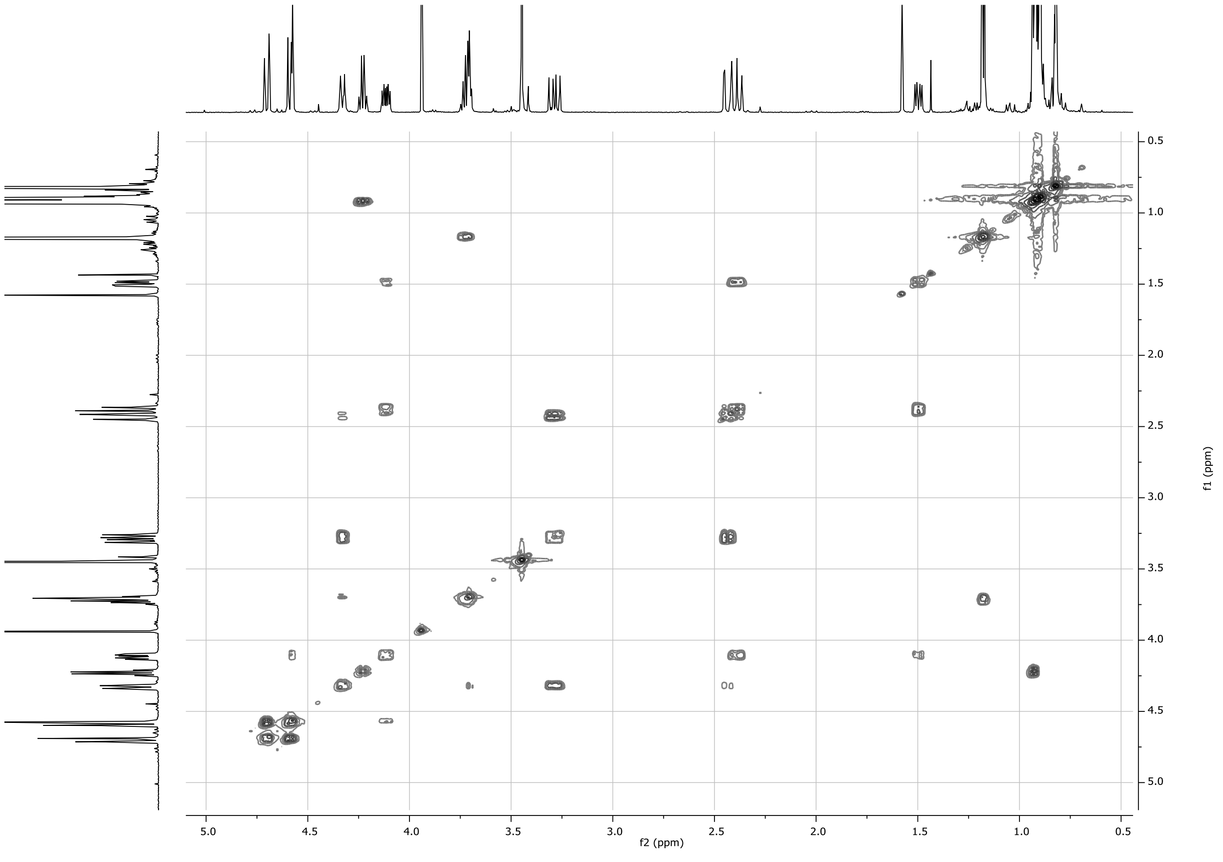

HSQC NMR, $CDCl_3$ of **S26**

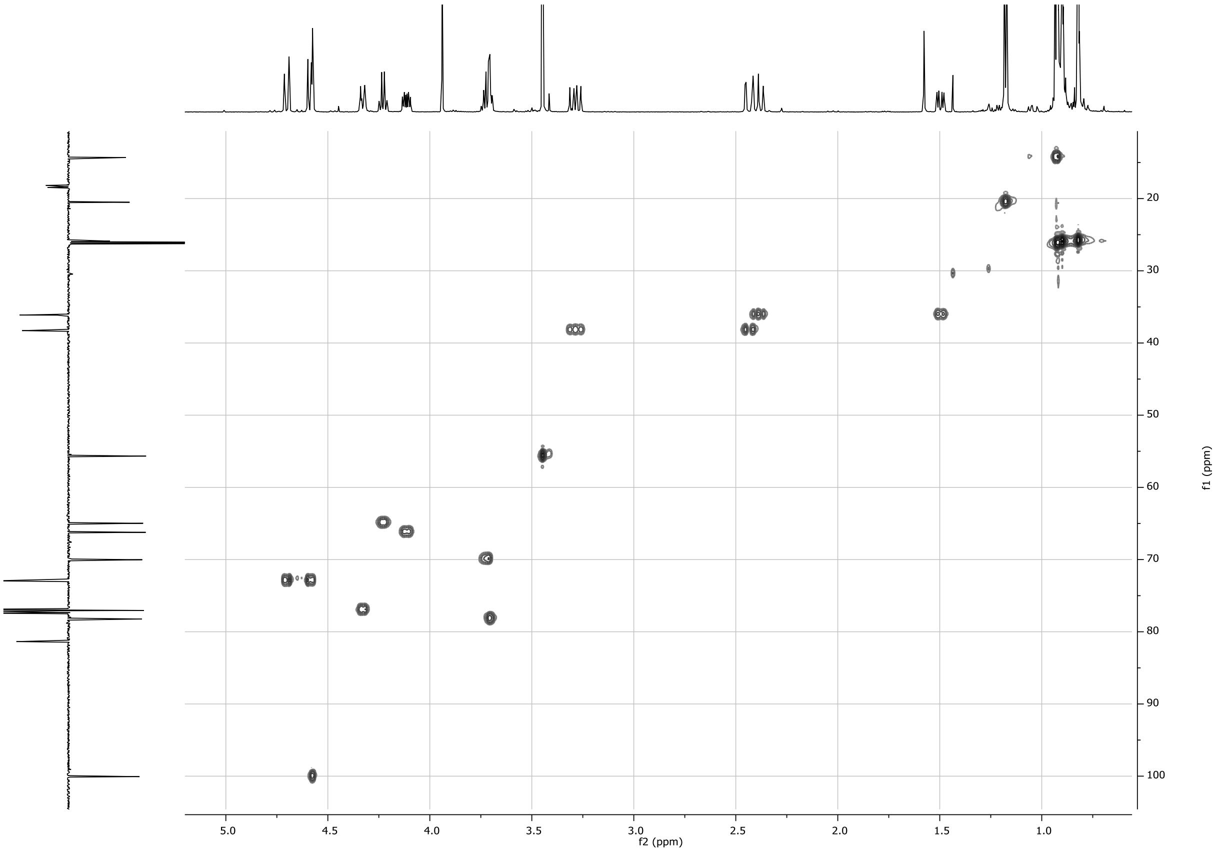

NOESY NMR, $CDCl_3$ of **S26**

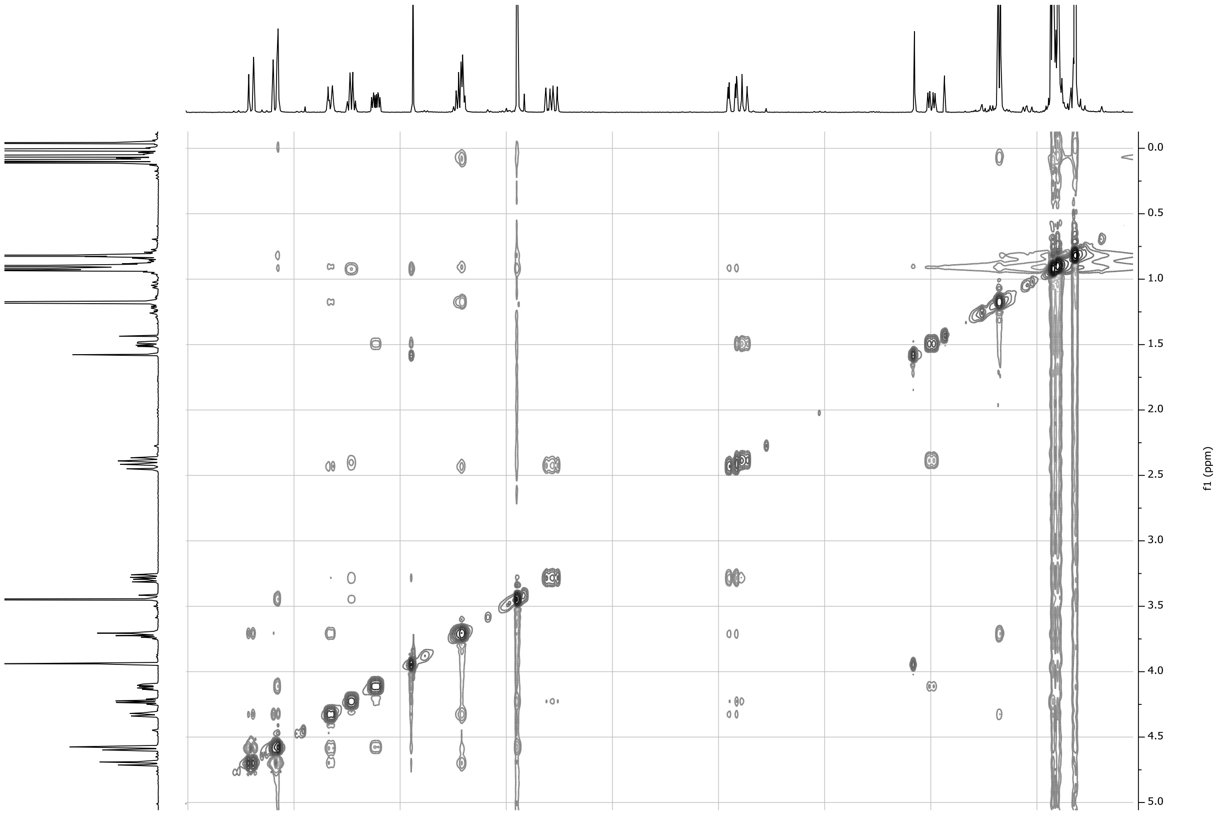

^{1}H NMR, 400 MHz, $CDCl_3$ of **S26-C4-*epi***

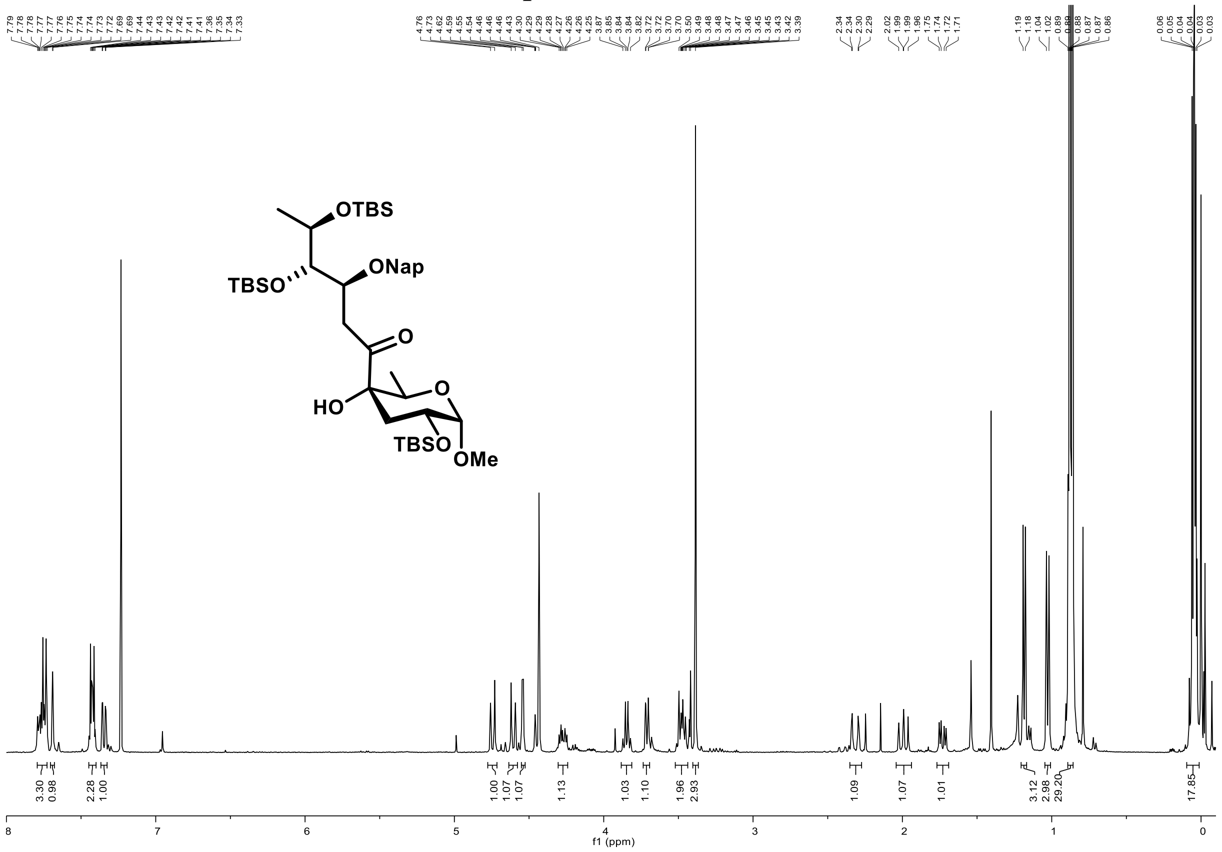

^{13}C NMR, 101 MHz, $CDCl_3$ of **S26-C4-*epi***

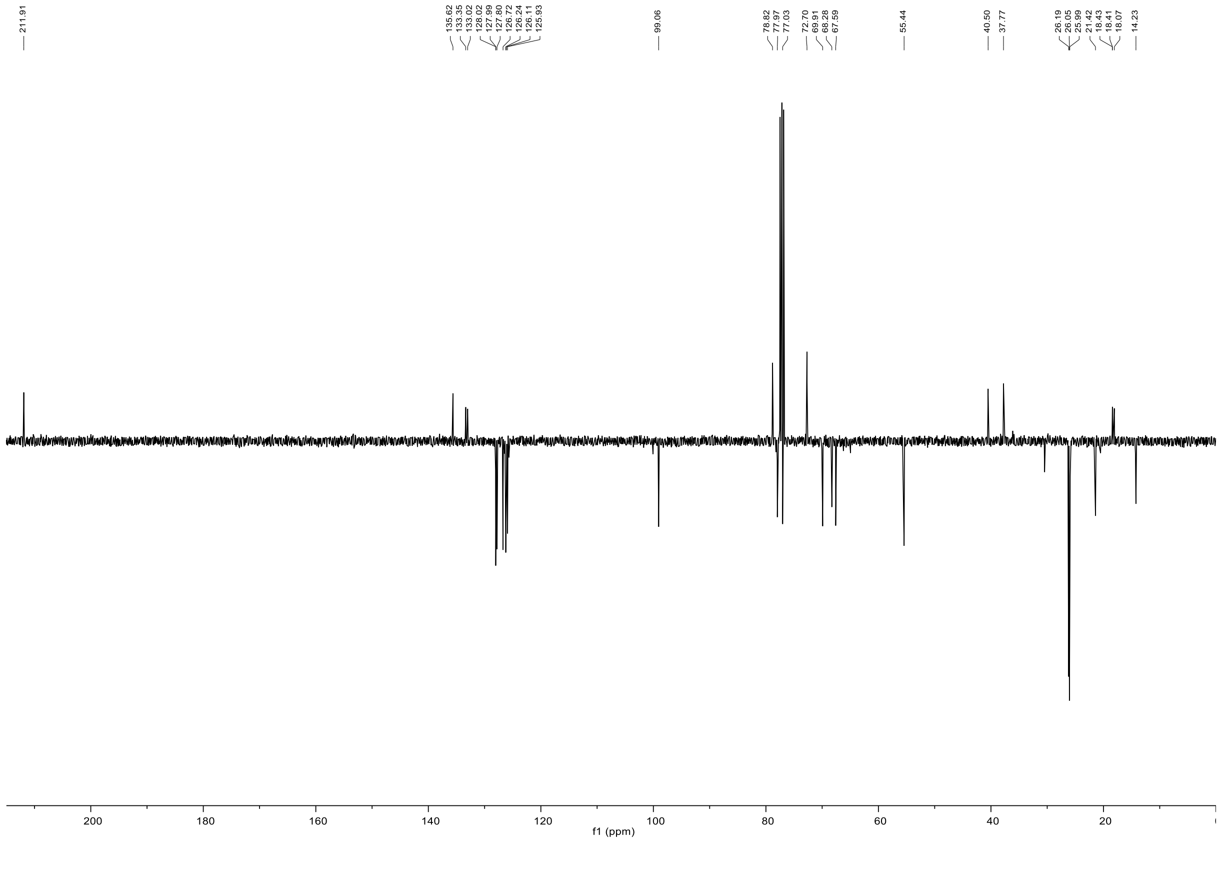

HH-COSY NMR, $CDCl_3$ of **S26-C4-*epi***

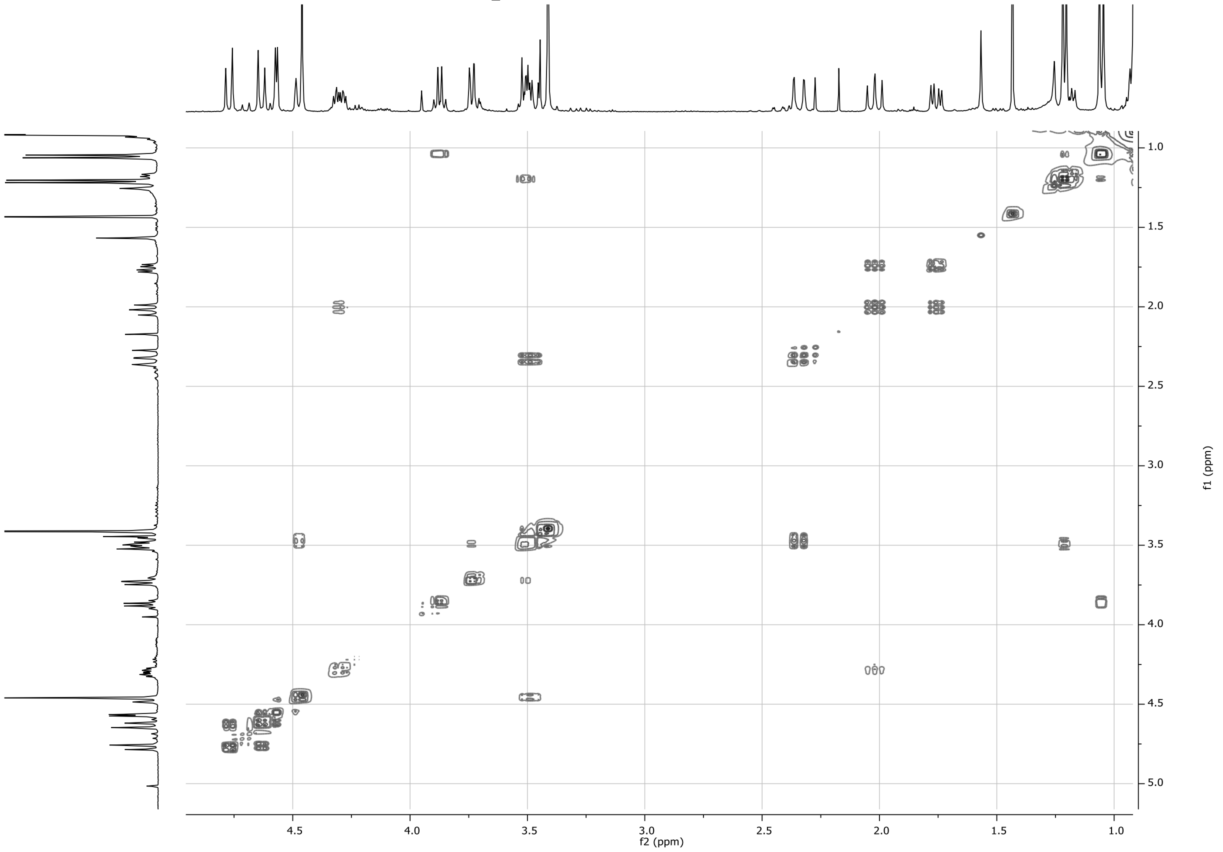

HSQC NMR, $CDCl_3$ of **S26-C4-*epi***

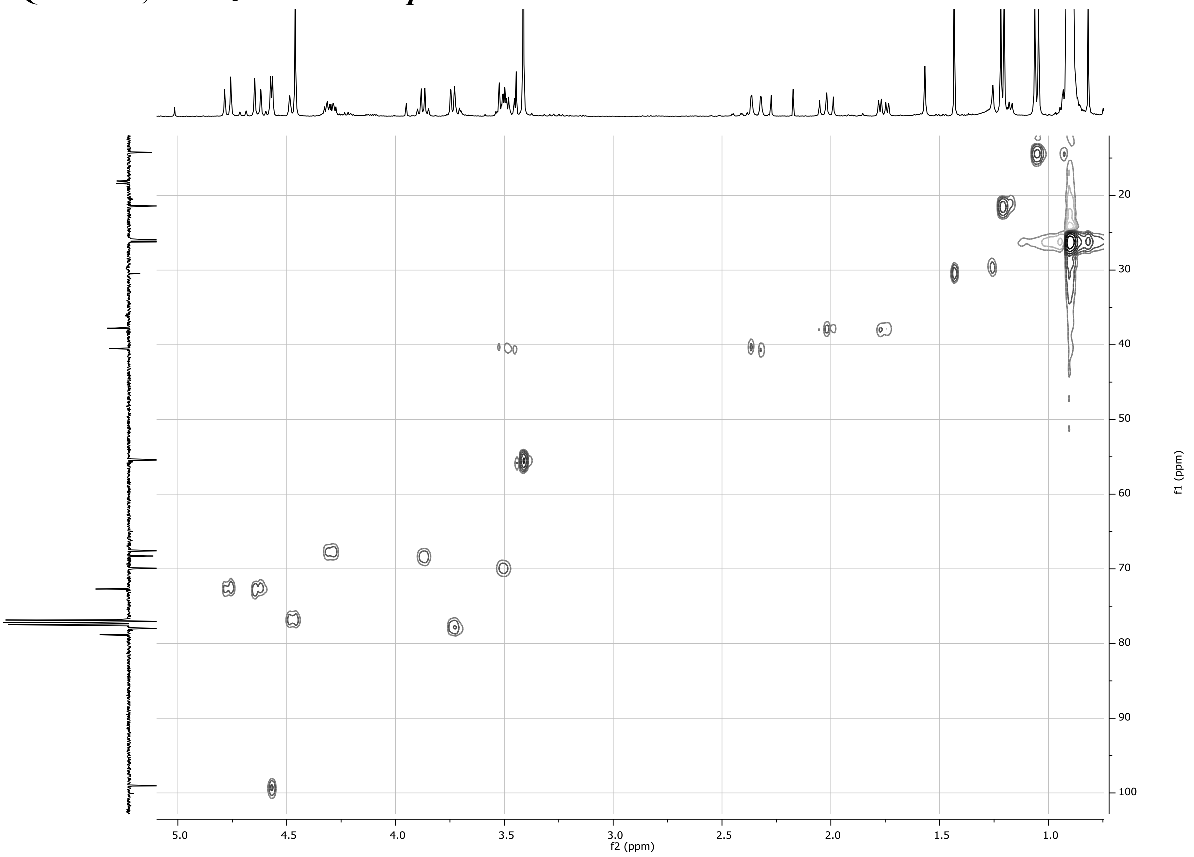

NOESY NMR, CDCl$_3$ of **S26-C4-*epi***

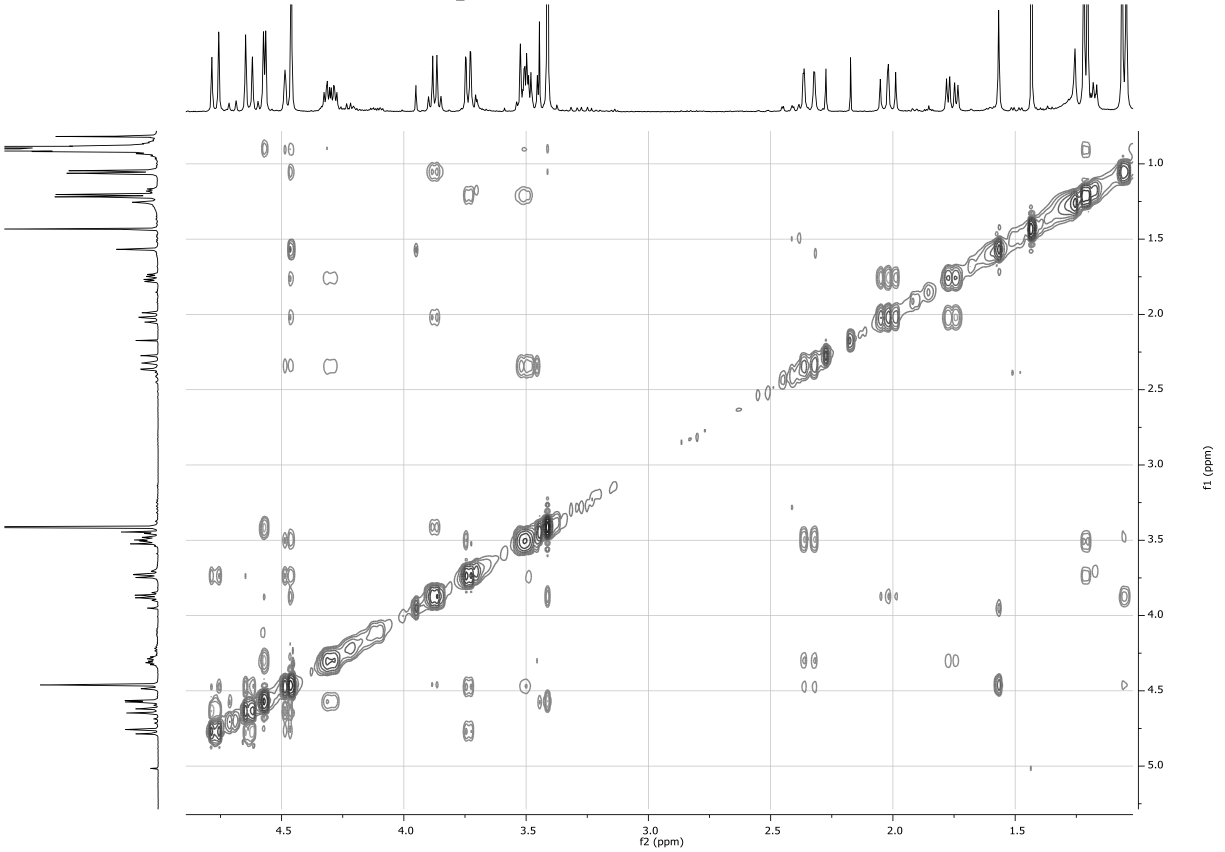

^{1}H NMR, 400 MHz, CDCl$_3$ of **S27**

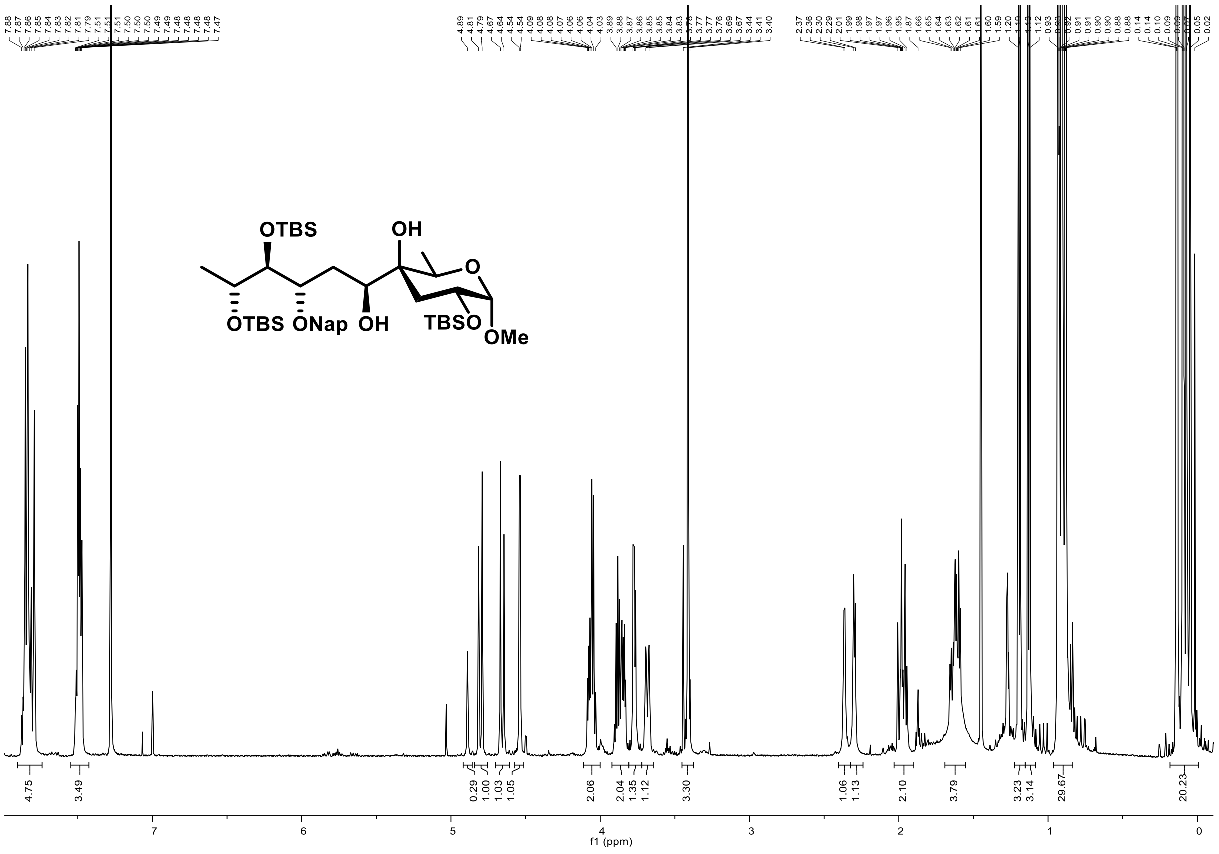

^{13}C NMR, 101 MHz, $CDCl_3$ of **S27**

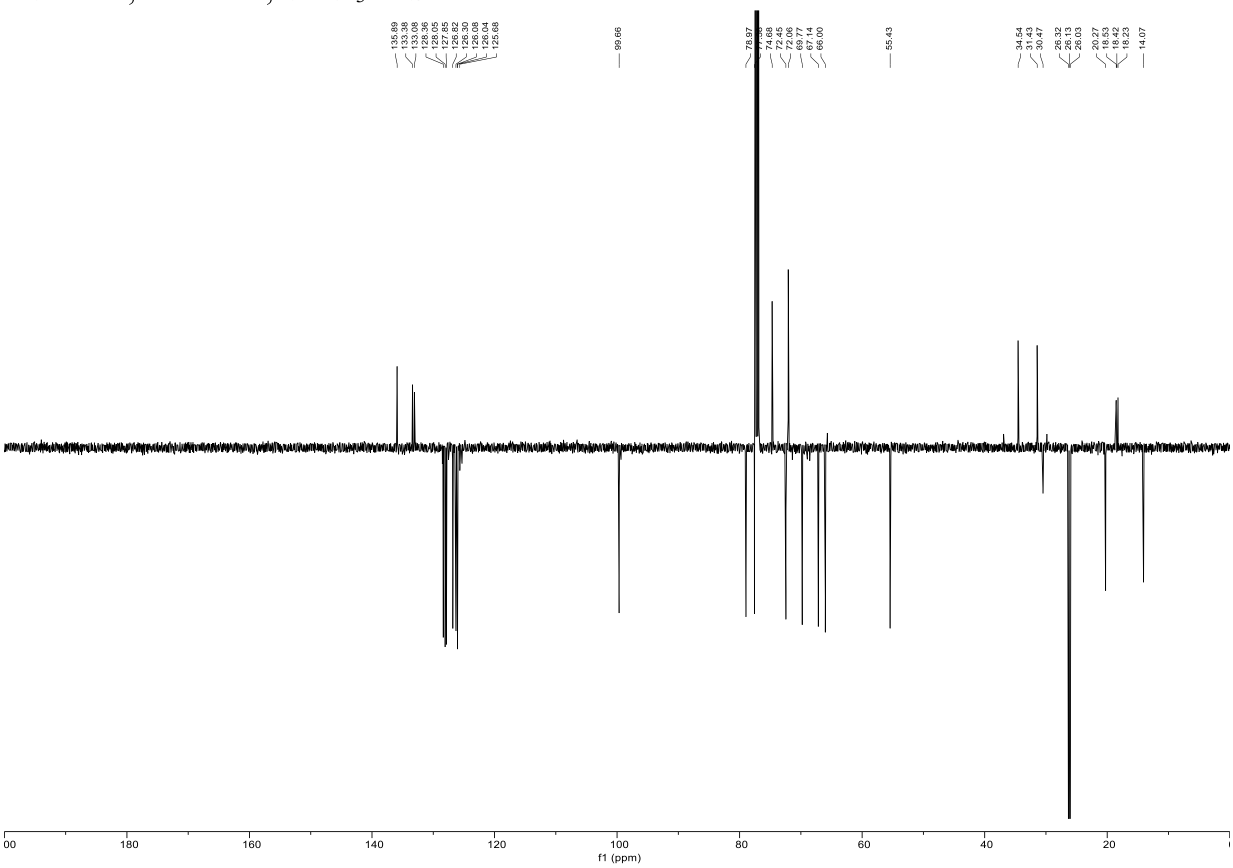

HH-COSY NMR, $CDCl_3$ of **S27**

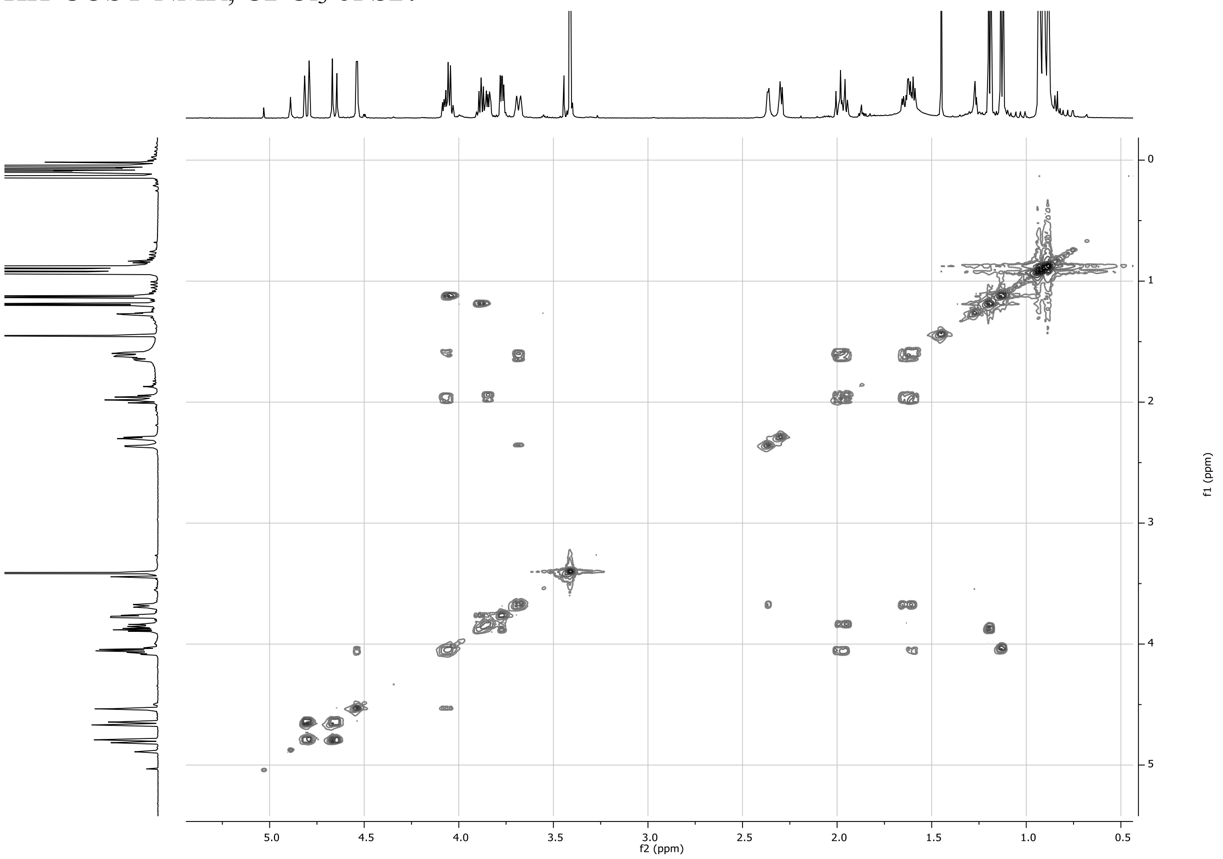

HSQC NMR, $CDCl_3$ of **S27**

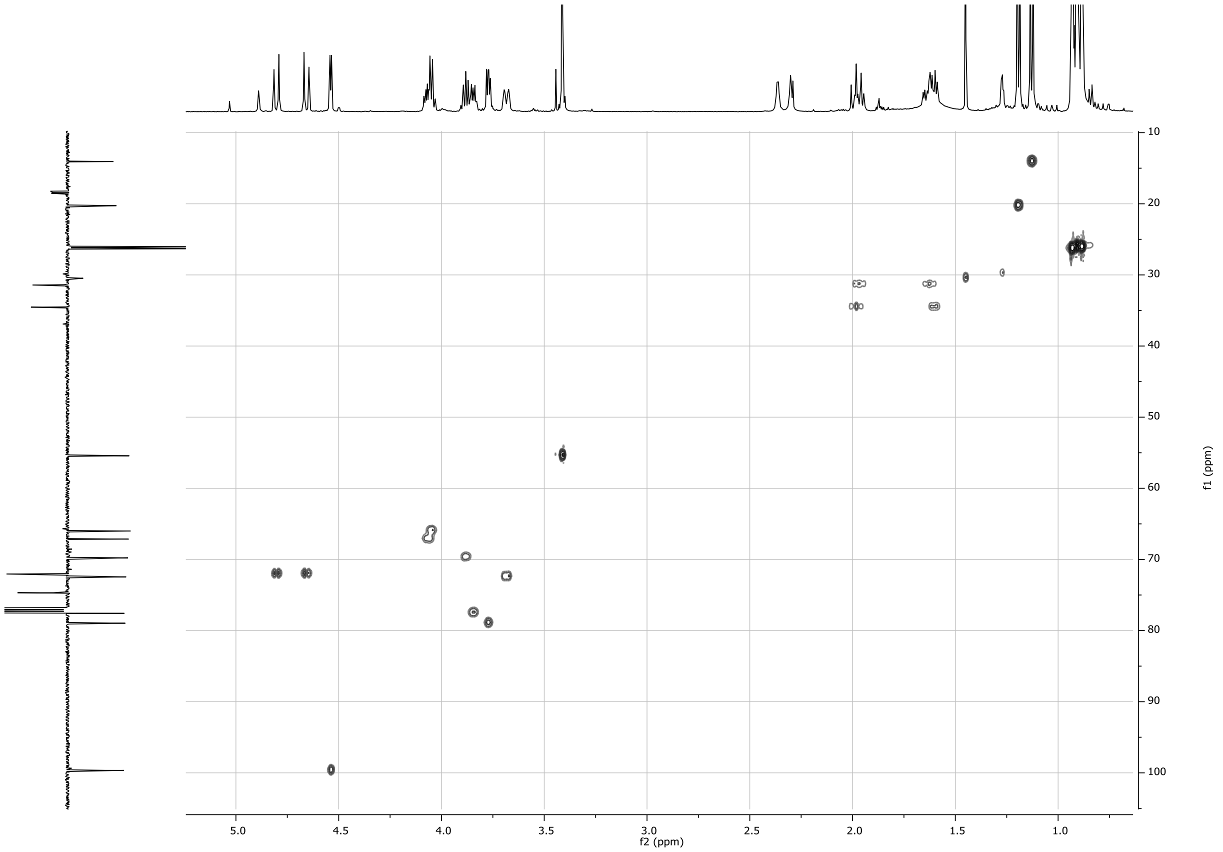

NOESY NMR, $CDCl_3$ of **S27**

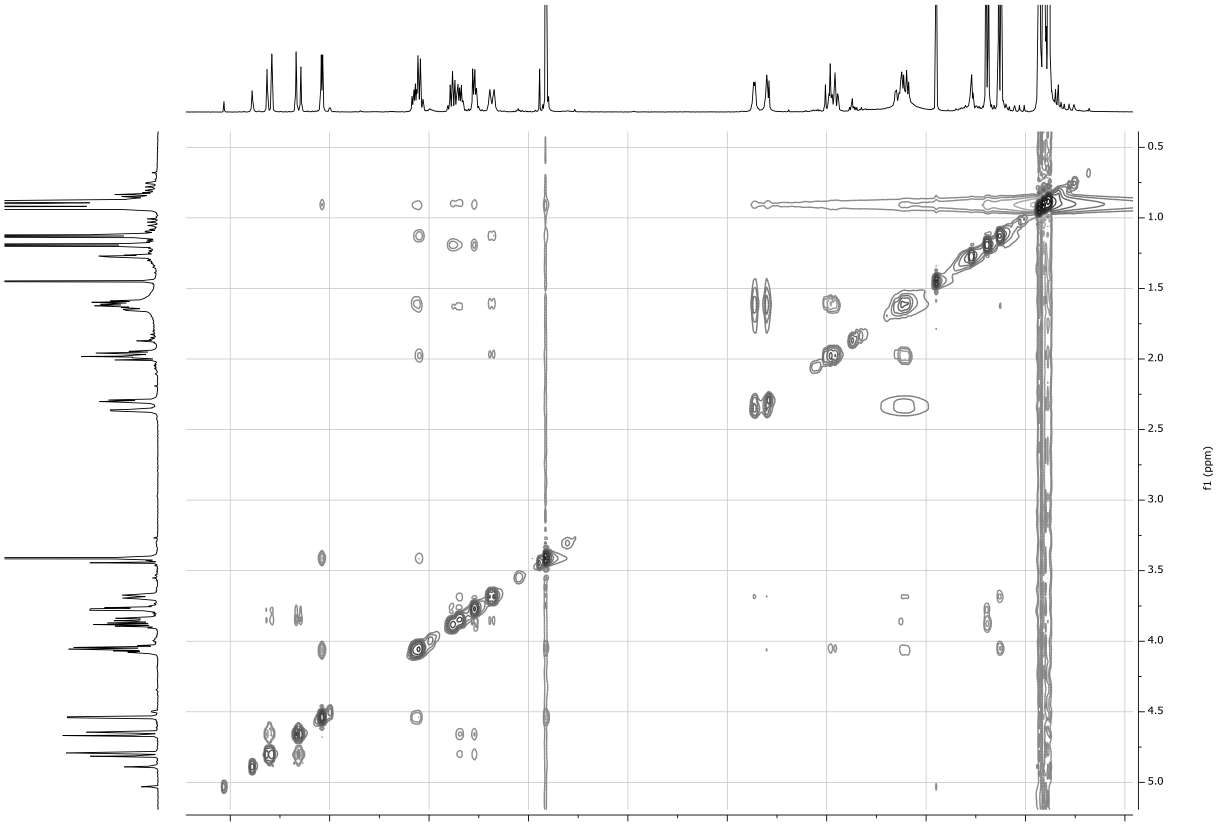

^{1}H NMR, 400 MHz, $CDCl_3$ of **S29**

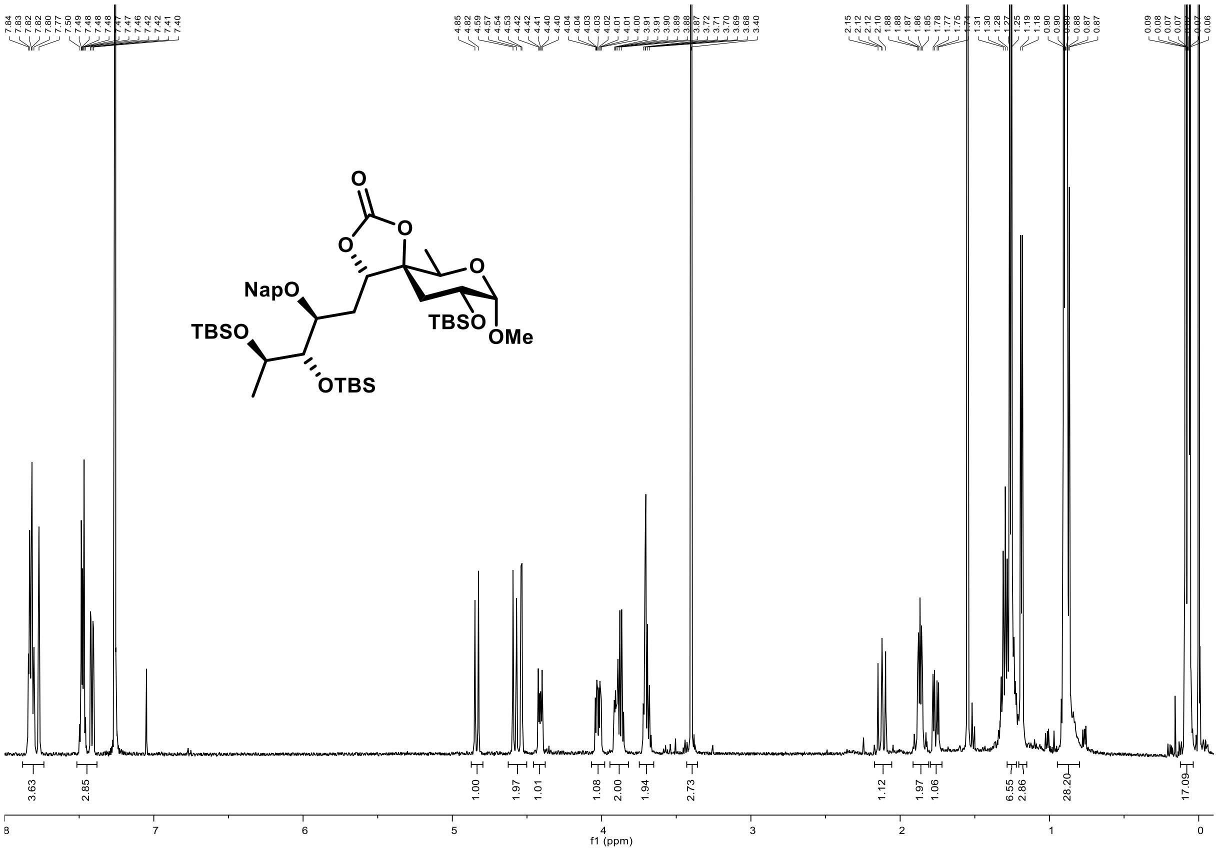

^{13}C NMR, 101 MHz, $CDCl_3$ of **S29**

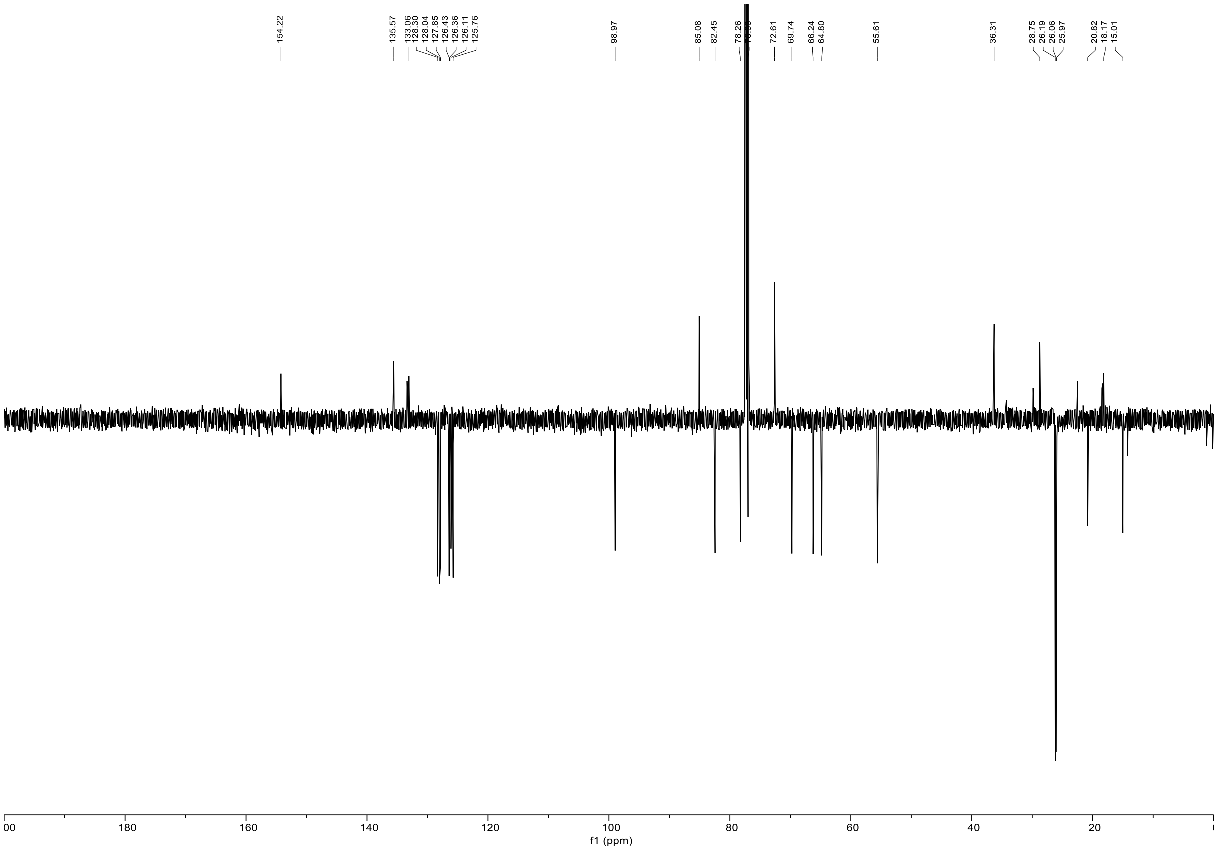

HH-COSY NMR, $CDCl_3$ of **S29**

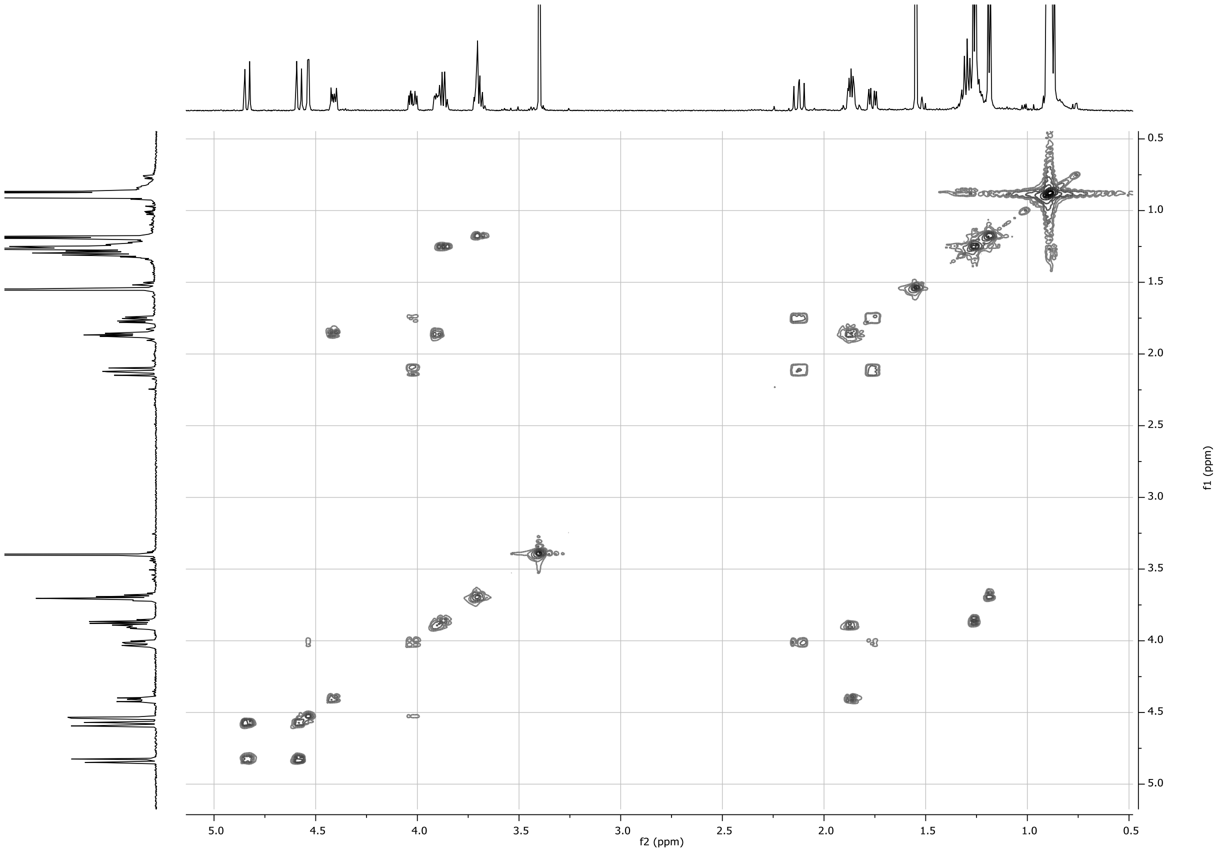

HSQC NMR, $CDCl_3$ of **S29**

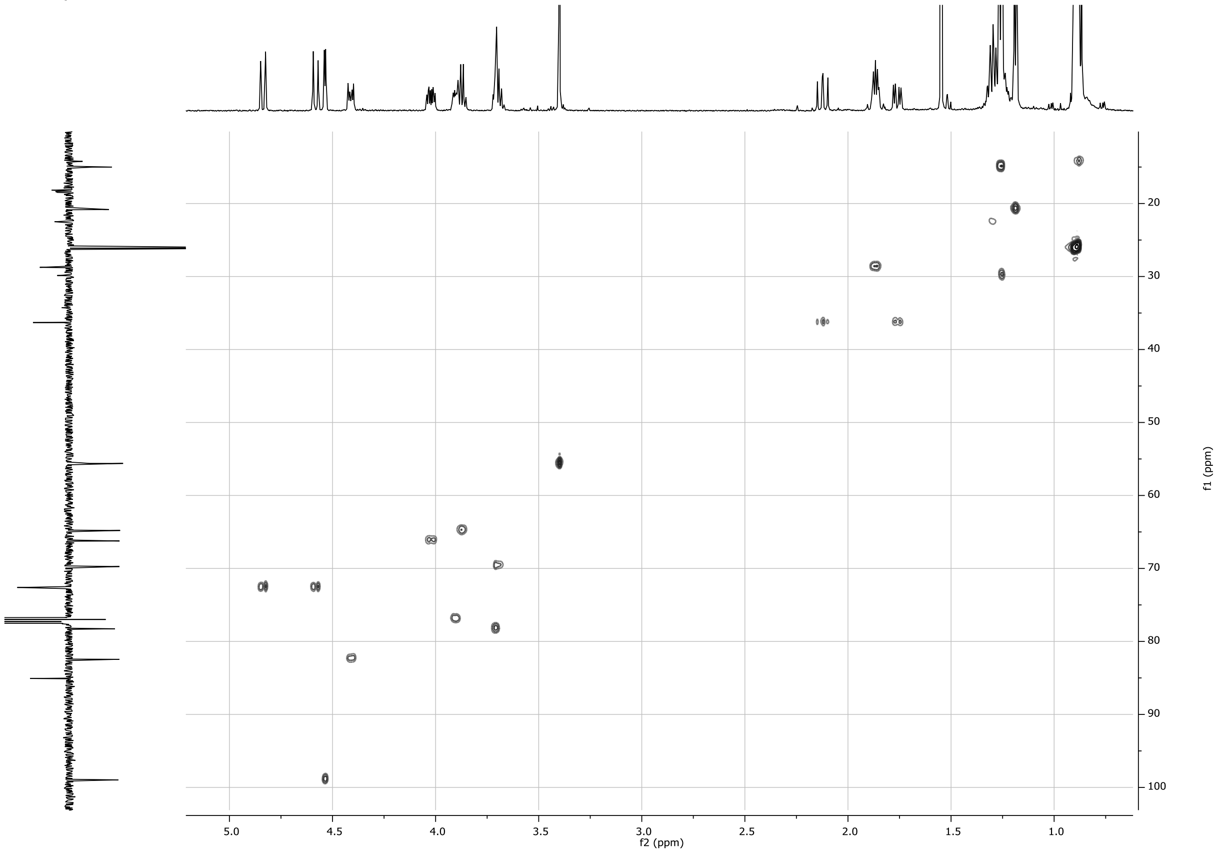

NOESY NMR, $CDCl_3$ of **S29**

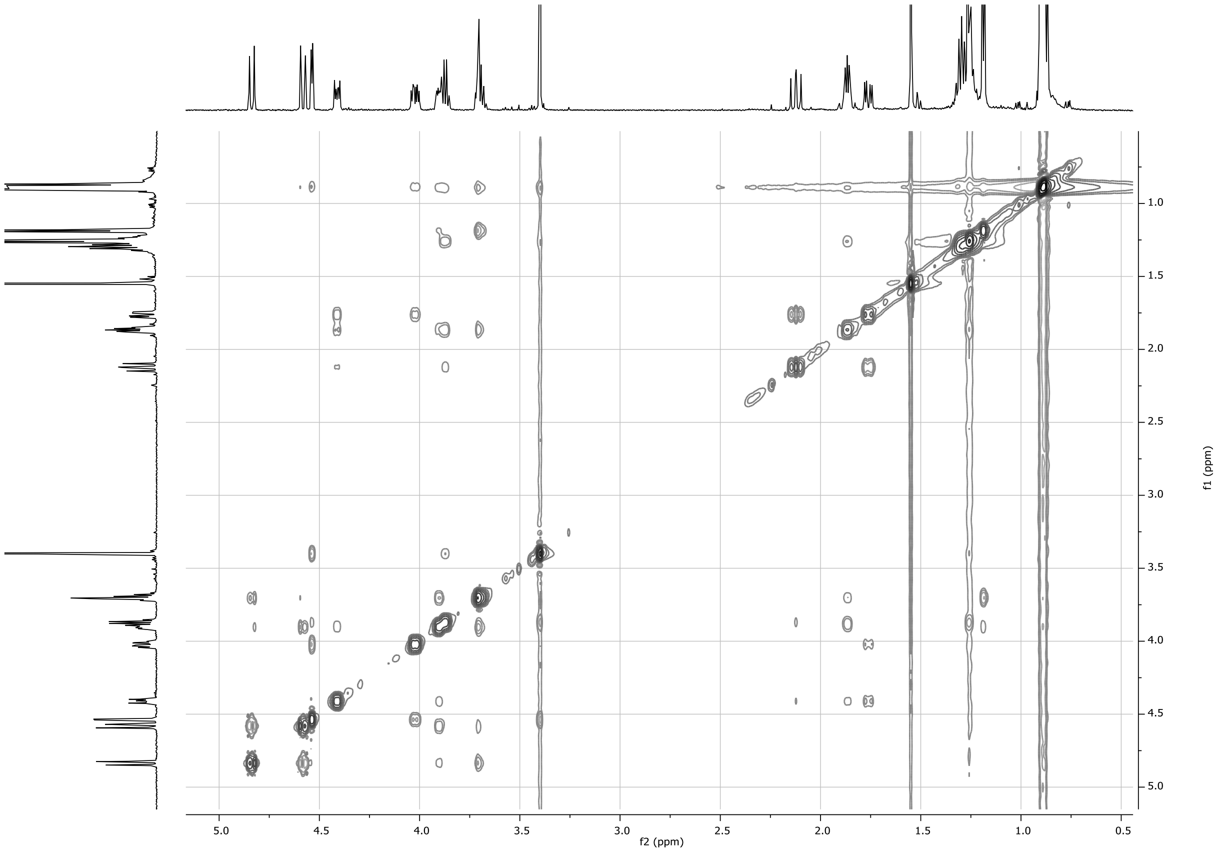

^{1}H NMR, 400 MHz, $CDCl_3$ of **S34**

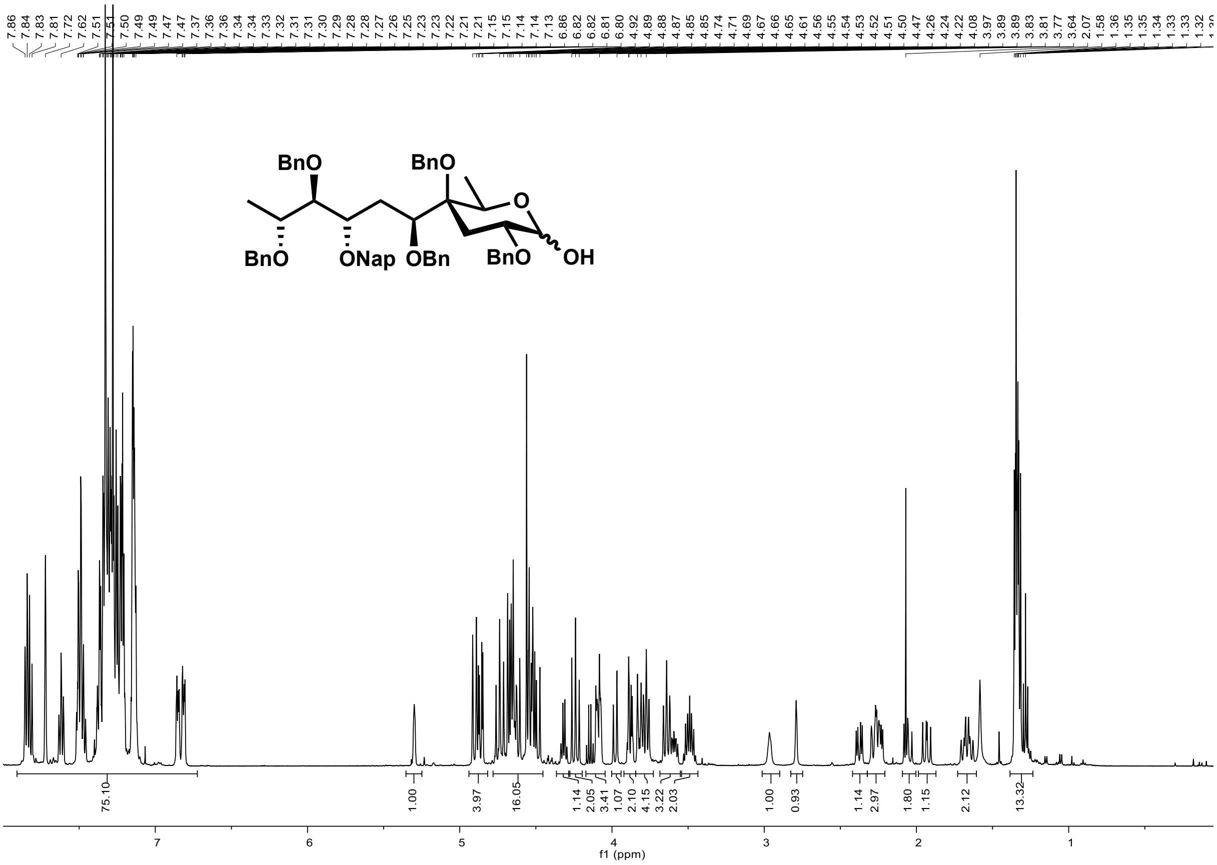

^{13}C NMR, 101 MHz, $CDCl_3$ of **S34**

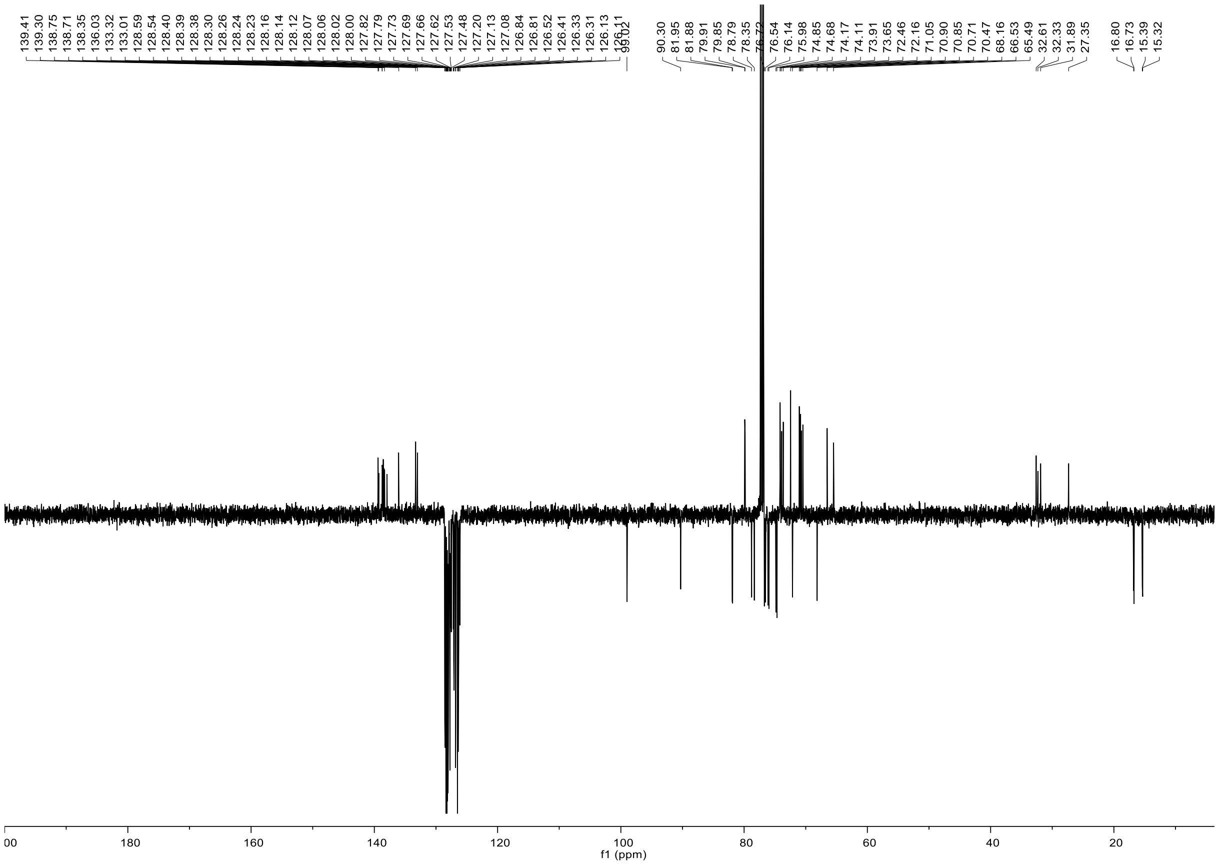

HH-COSY NMR, $CDCl_3$ of **S34**

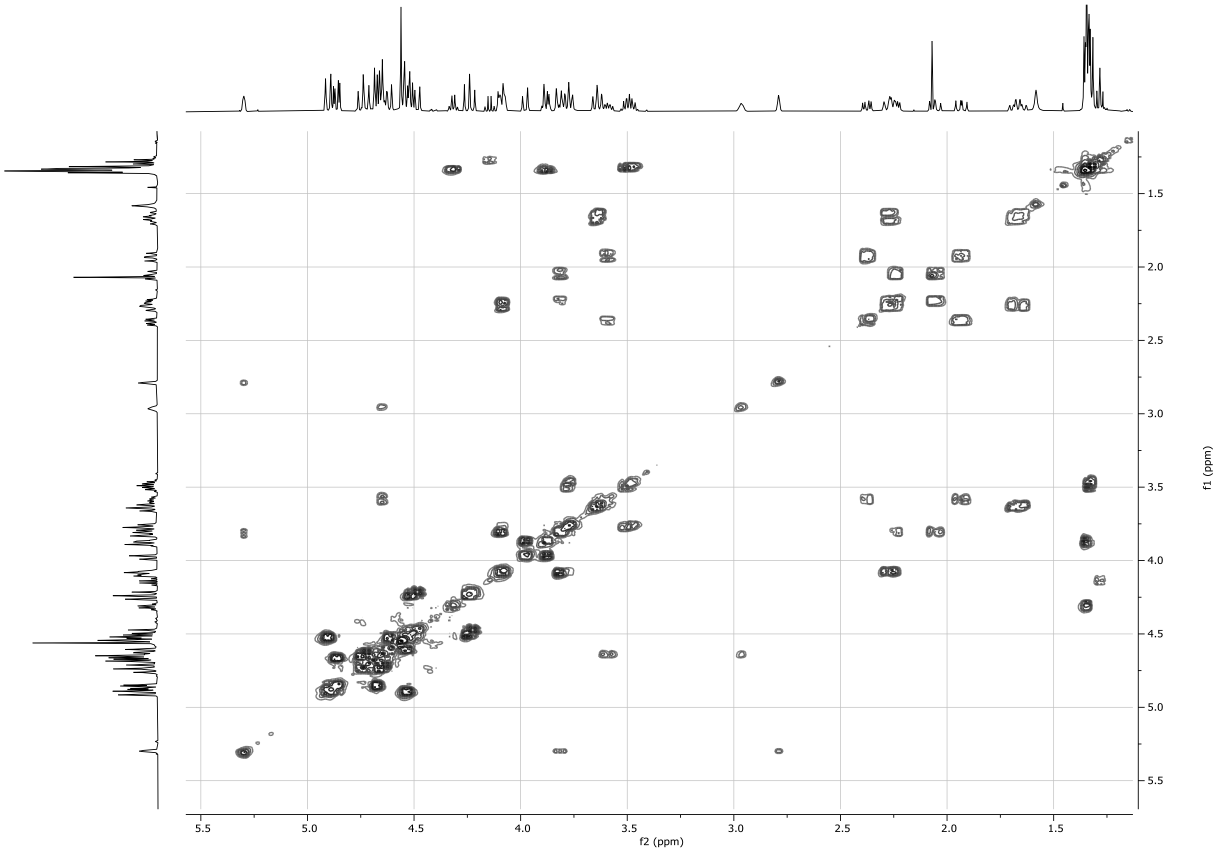

HSQC NMR, $CDCl_3$ of **S34**

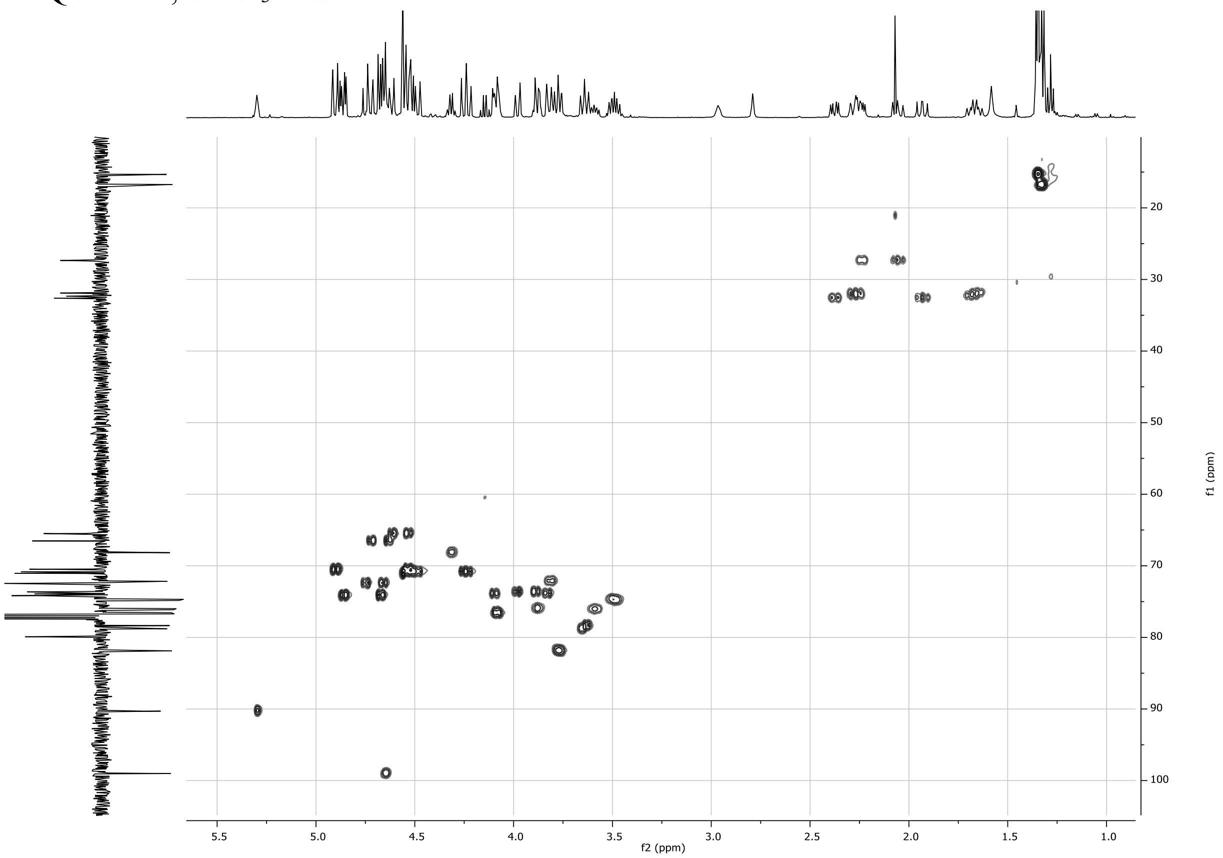

^{1}H NMR, 400 MHz, toluene-d_8 of **S36** (T = 233 K)

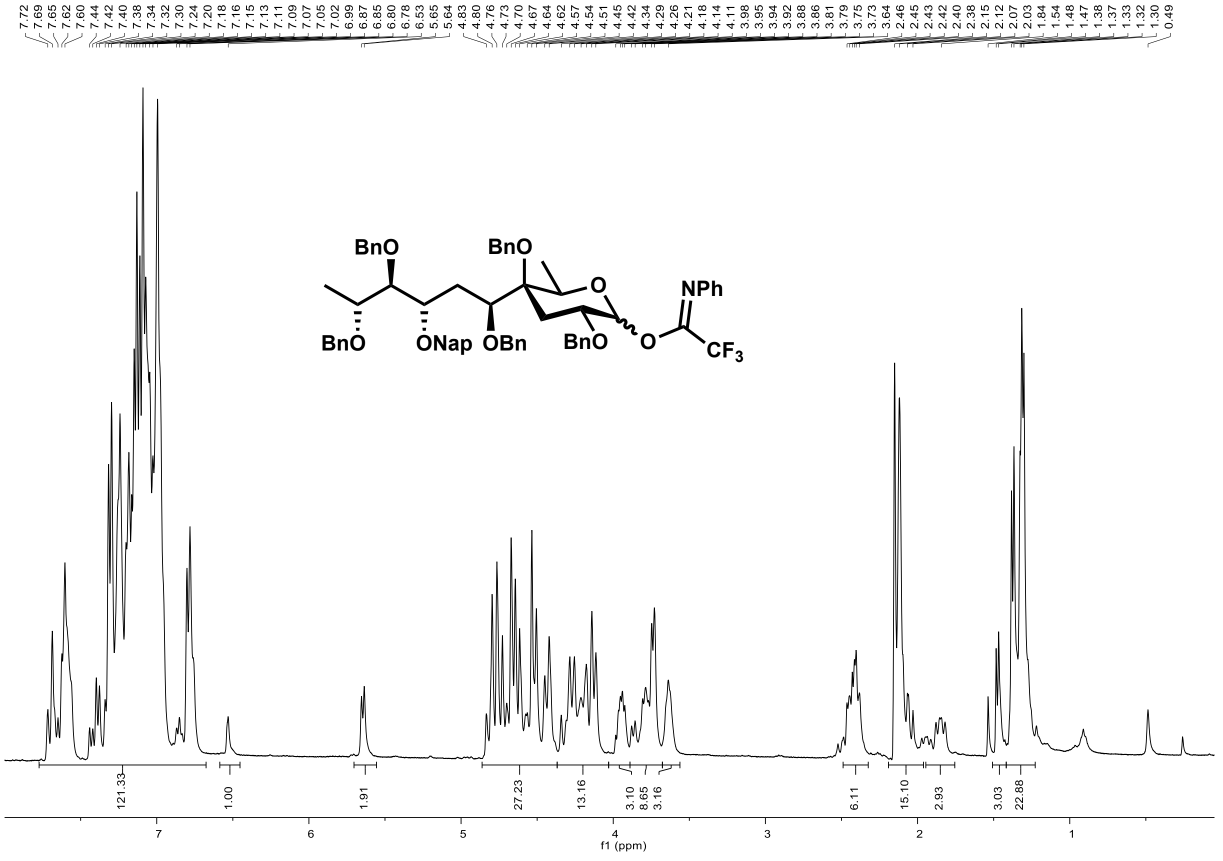

^{13}C NMR, 101 MHz, toluene-d_8 of **S36** (T = 233 K)

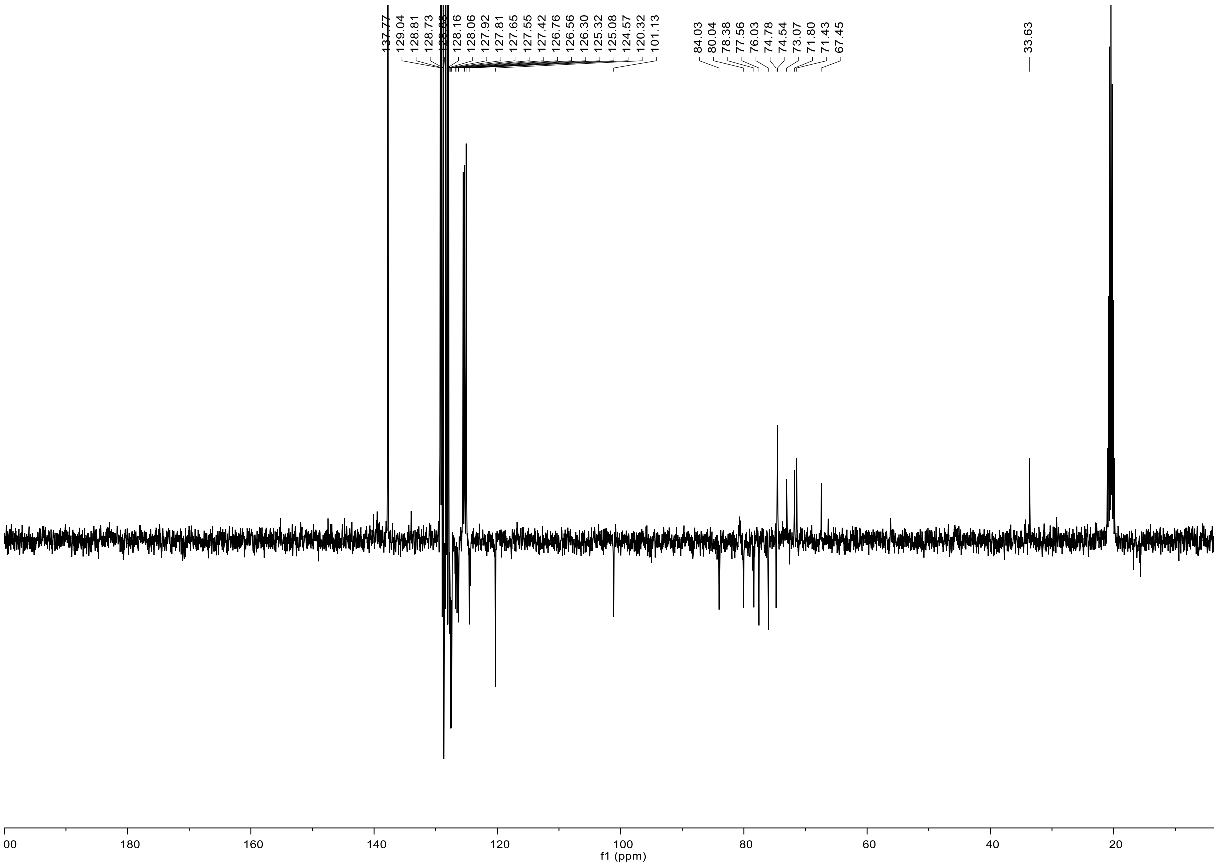

HH-COSY NMR, toluene-d_8 of **S36** (T = 233 K)

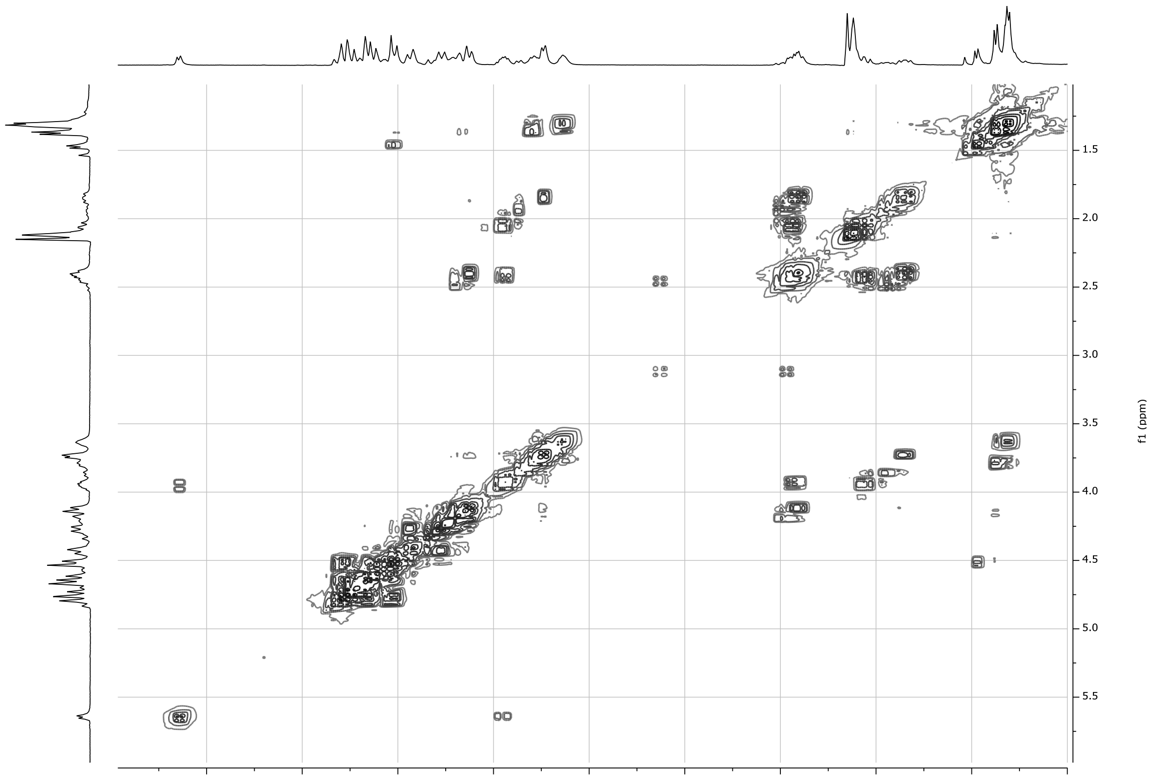

HSQC NMR, toluene-d_8 of **S36** (T = 233 K)

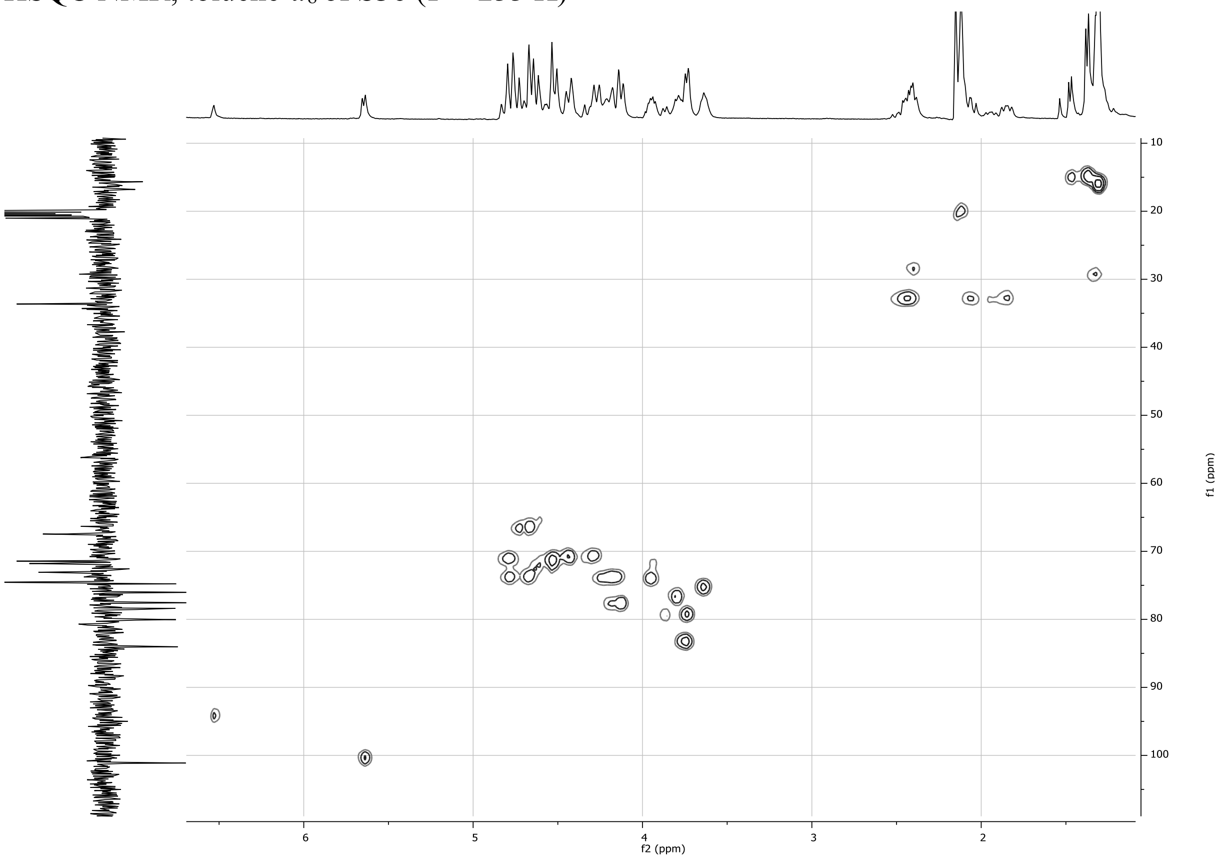

^{1}H NMR, 500 MHz, $CDCl_3$ of **31**

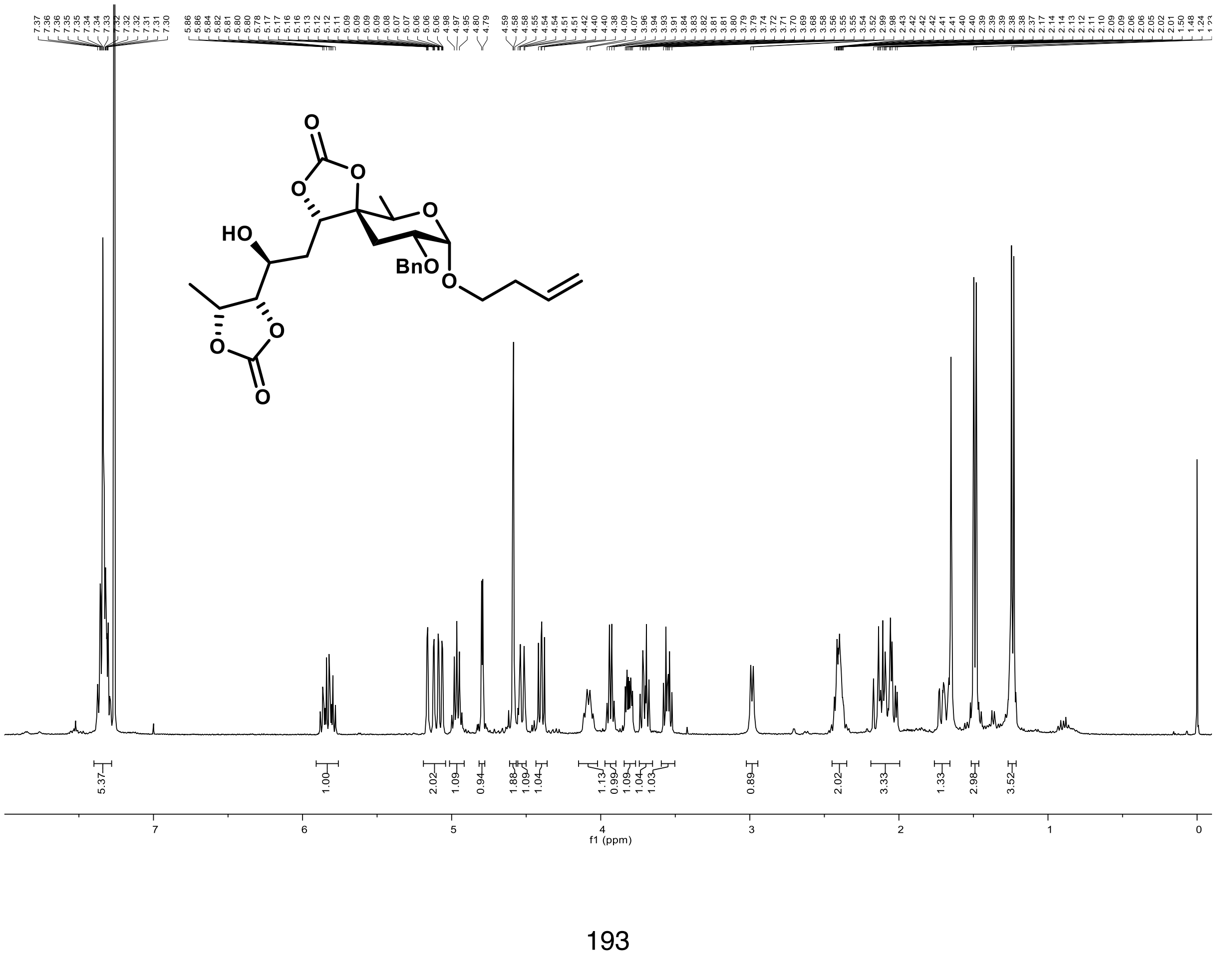

^{13}C NMR, 126 MHz, $CDCl_3$ of **31**

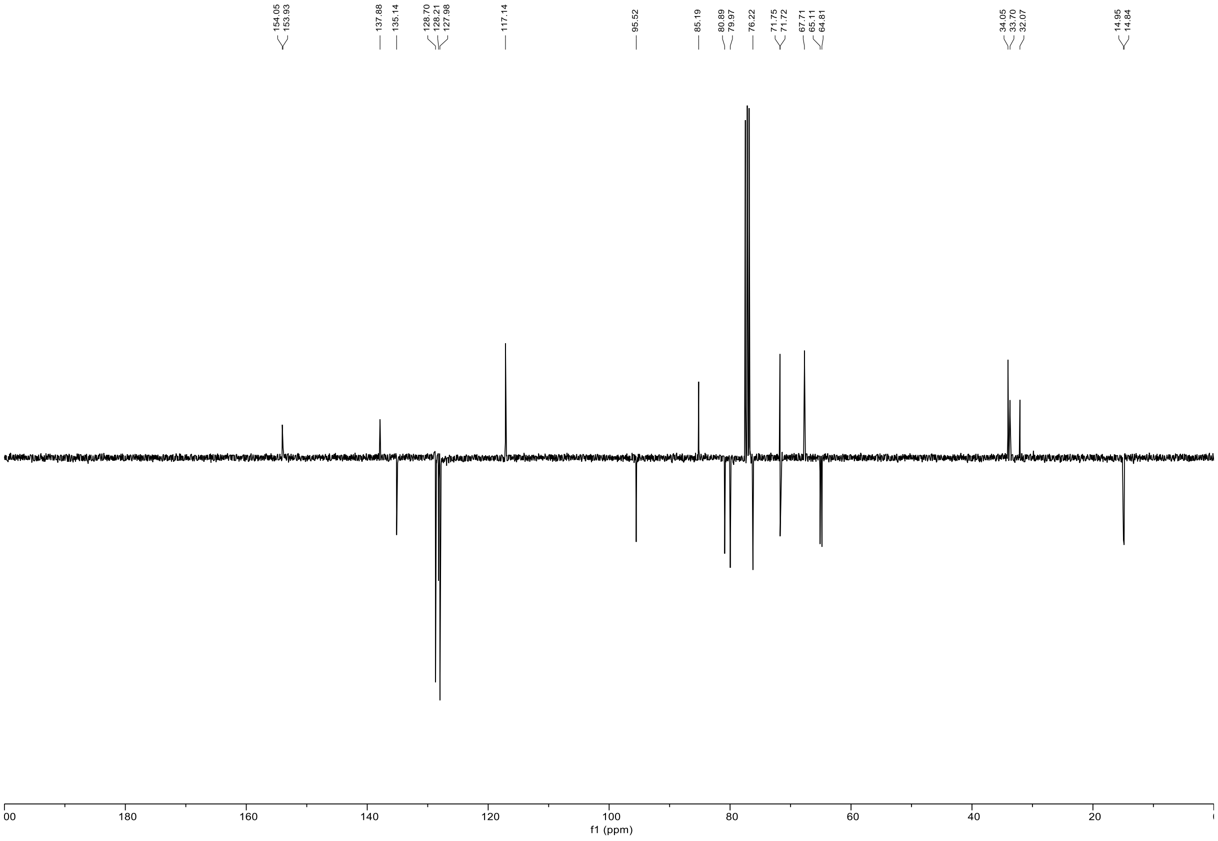

HH-COSY NMR, $CDCl_3$ of **31**

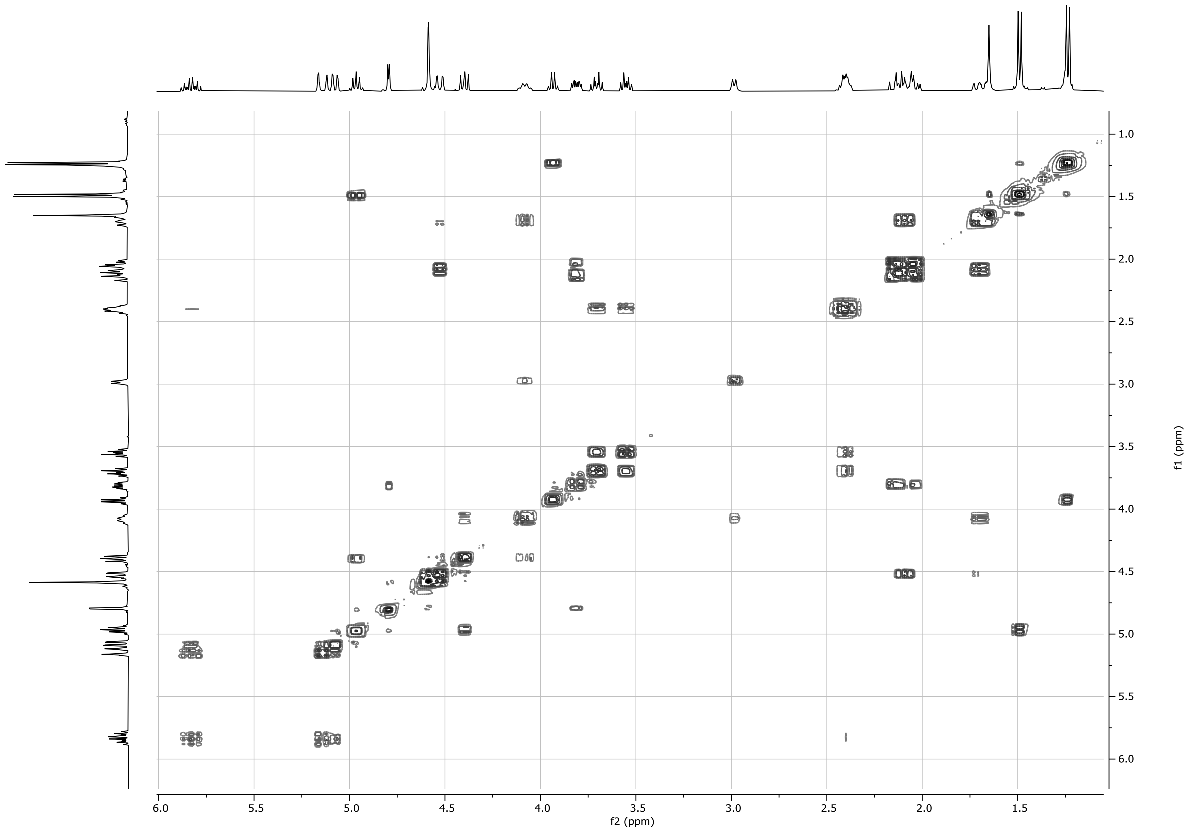

HSQC NMR, $CDCl_3$ of **31**

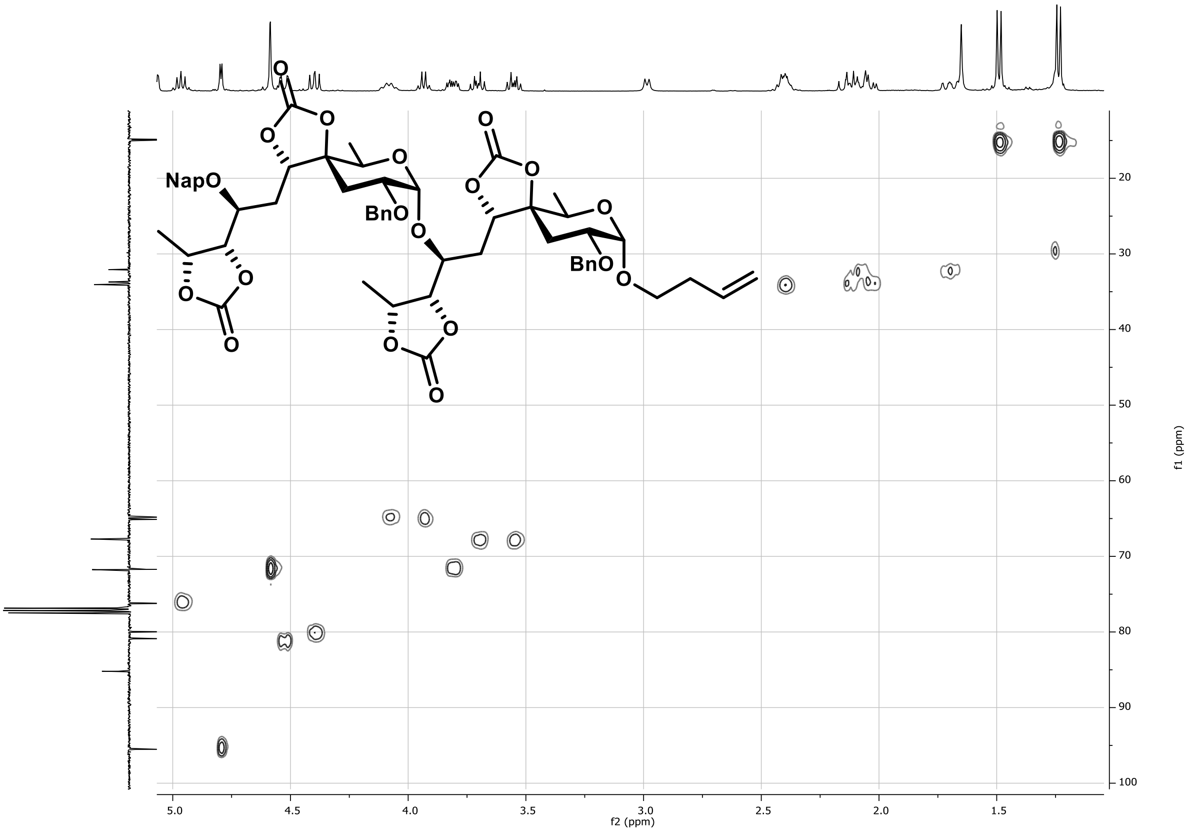

^{1}H NMR, 400 MHz, $CDCl_3$ of **32**

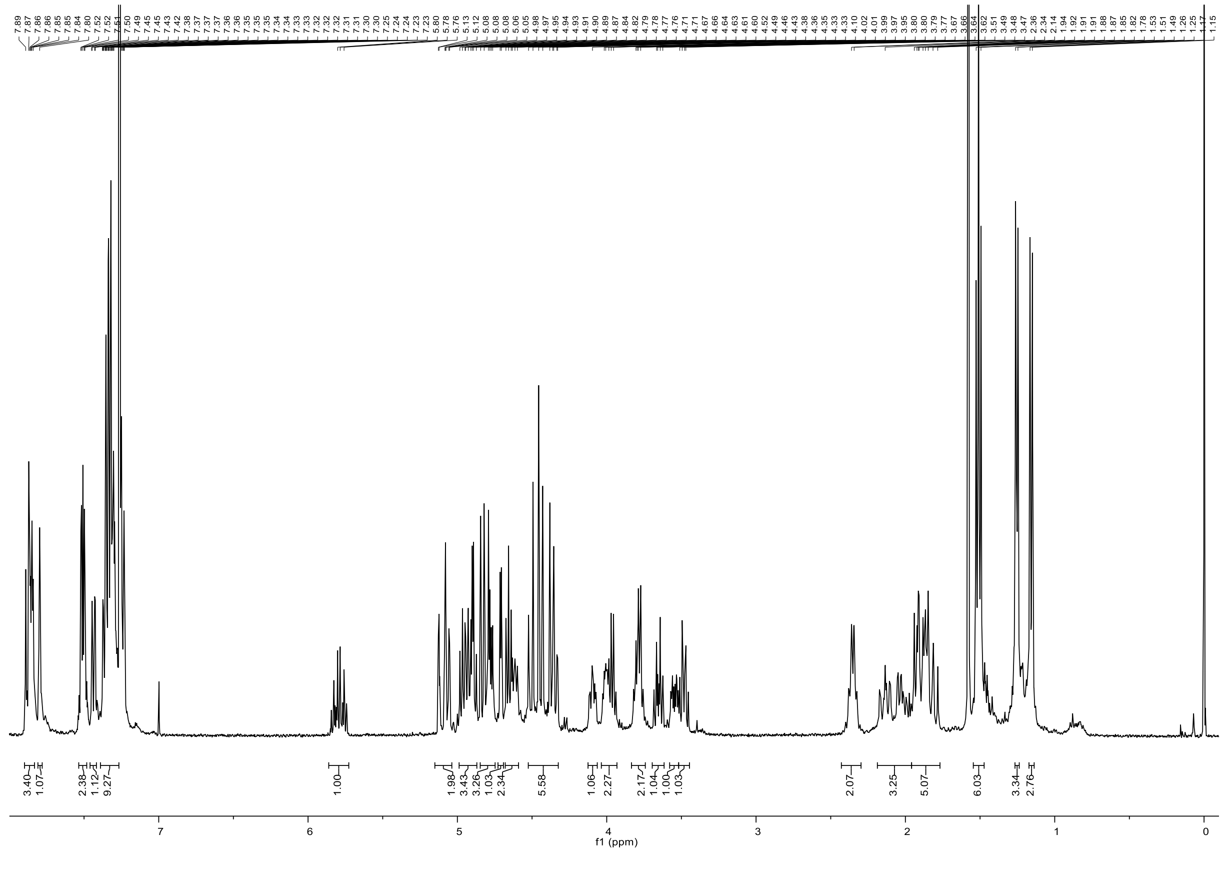

^{13}C NMR, 101 MHz, $CDCl_3$ of **32**

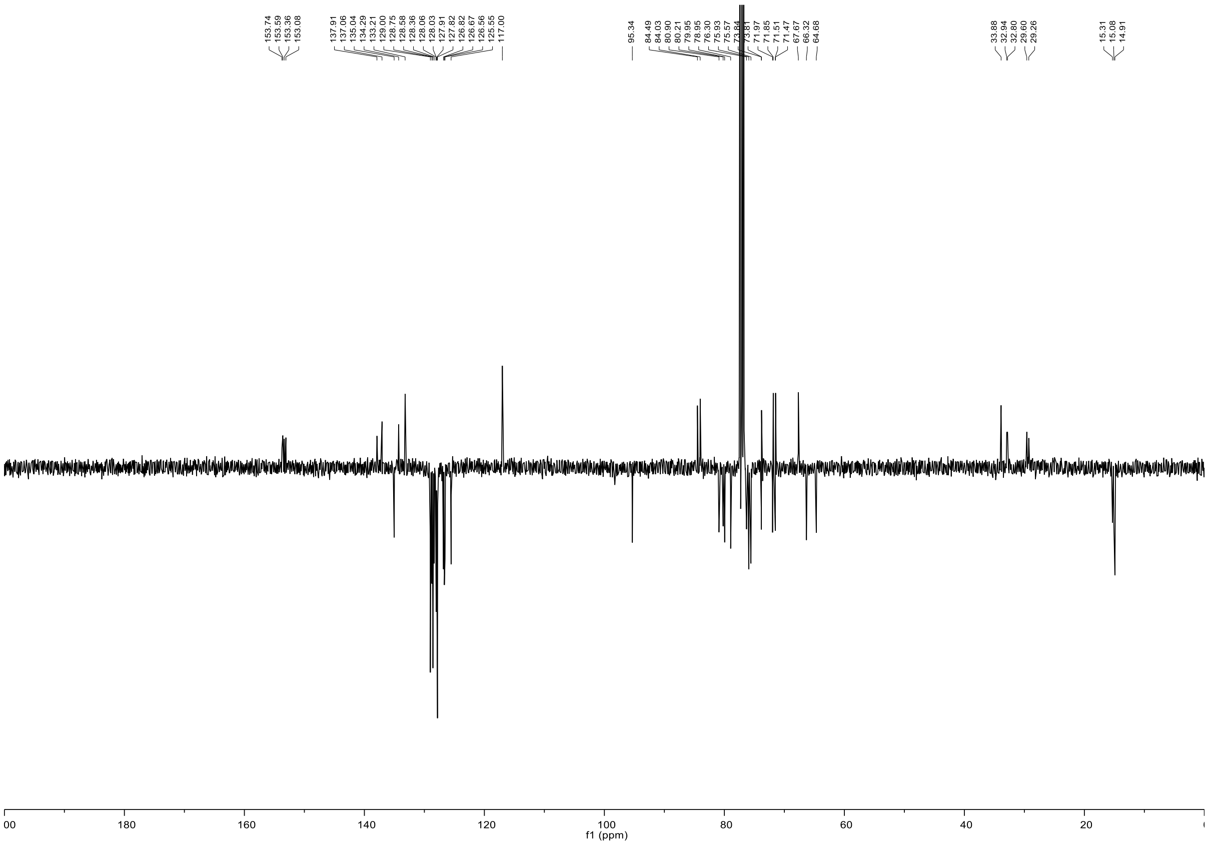

HH-COSY NMR, $CDCl_3$ of **32**

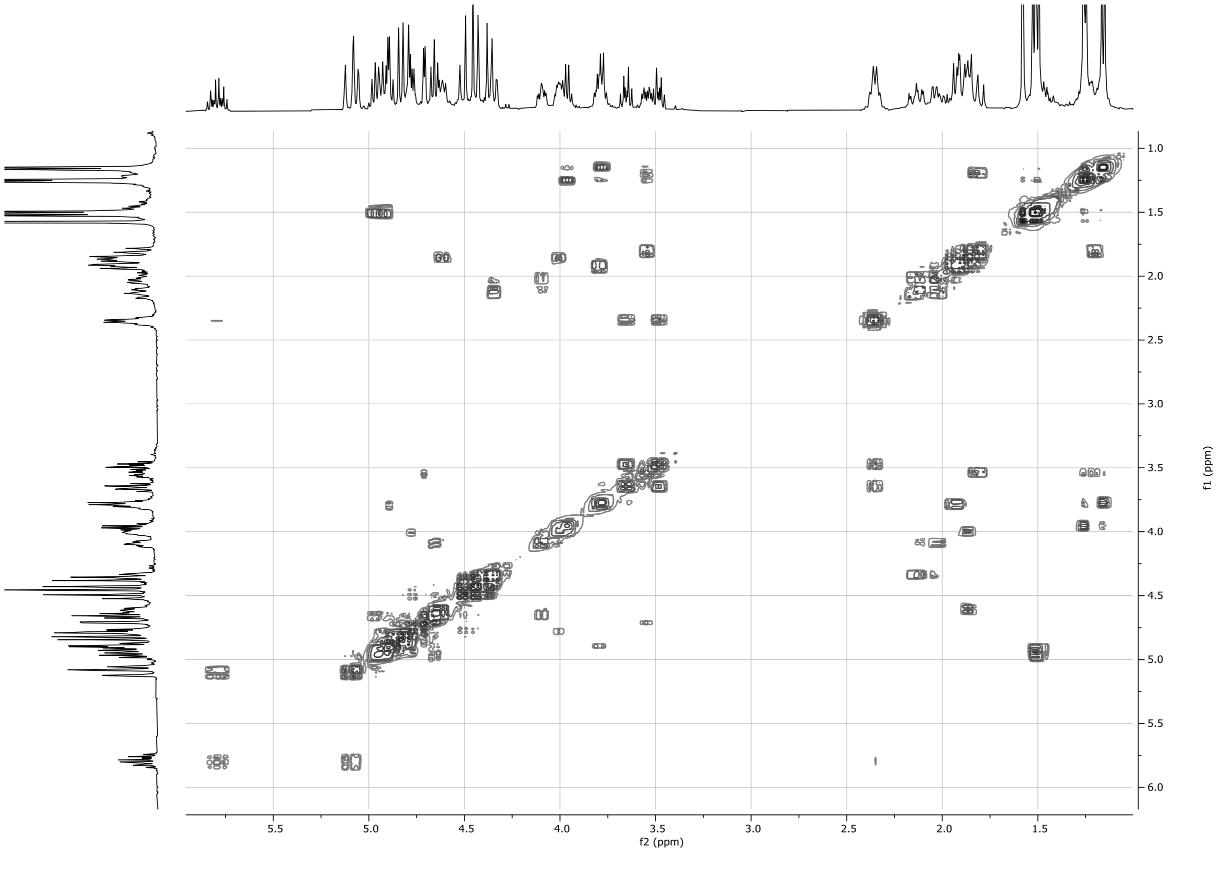

HSQC NMR, $CDCl_3$ of **32**

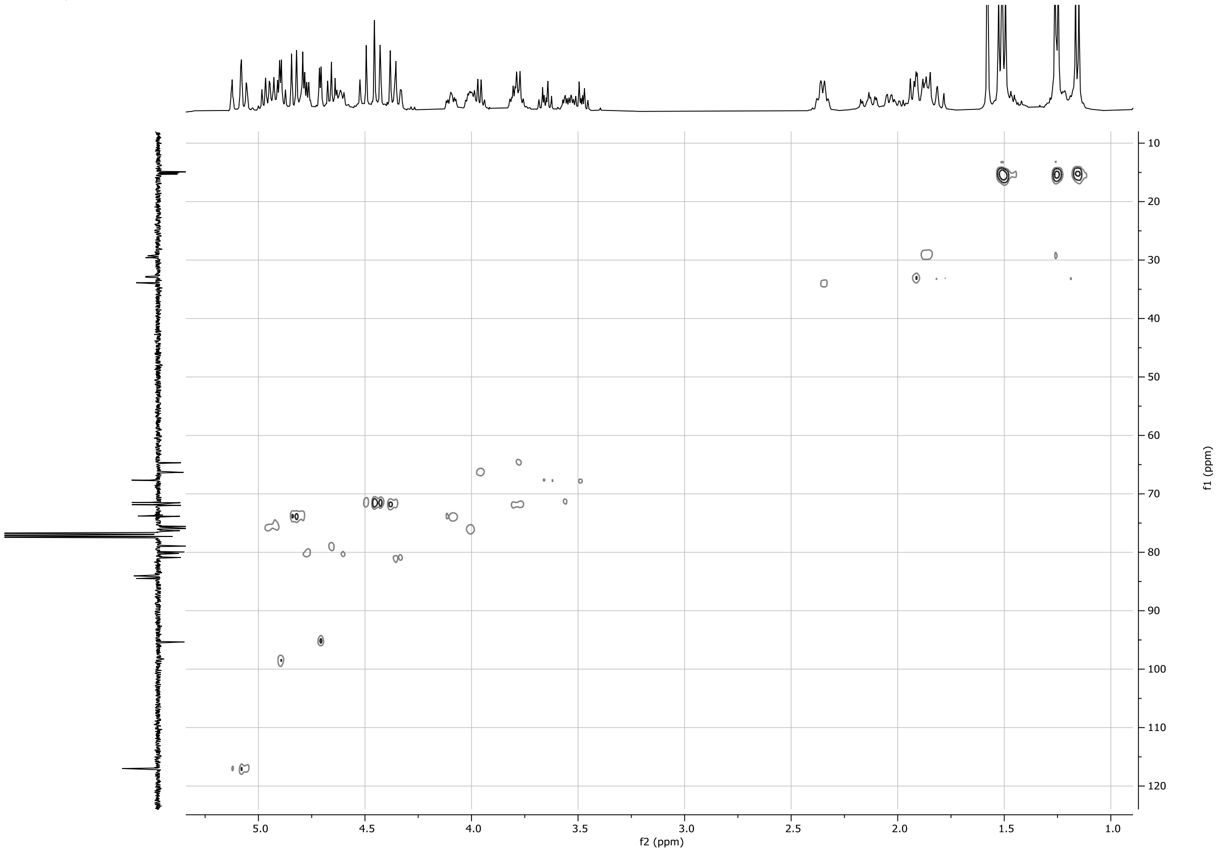

HMBC NMR, $CDCl_3$ of **32**

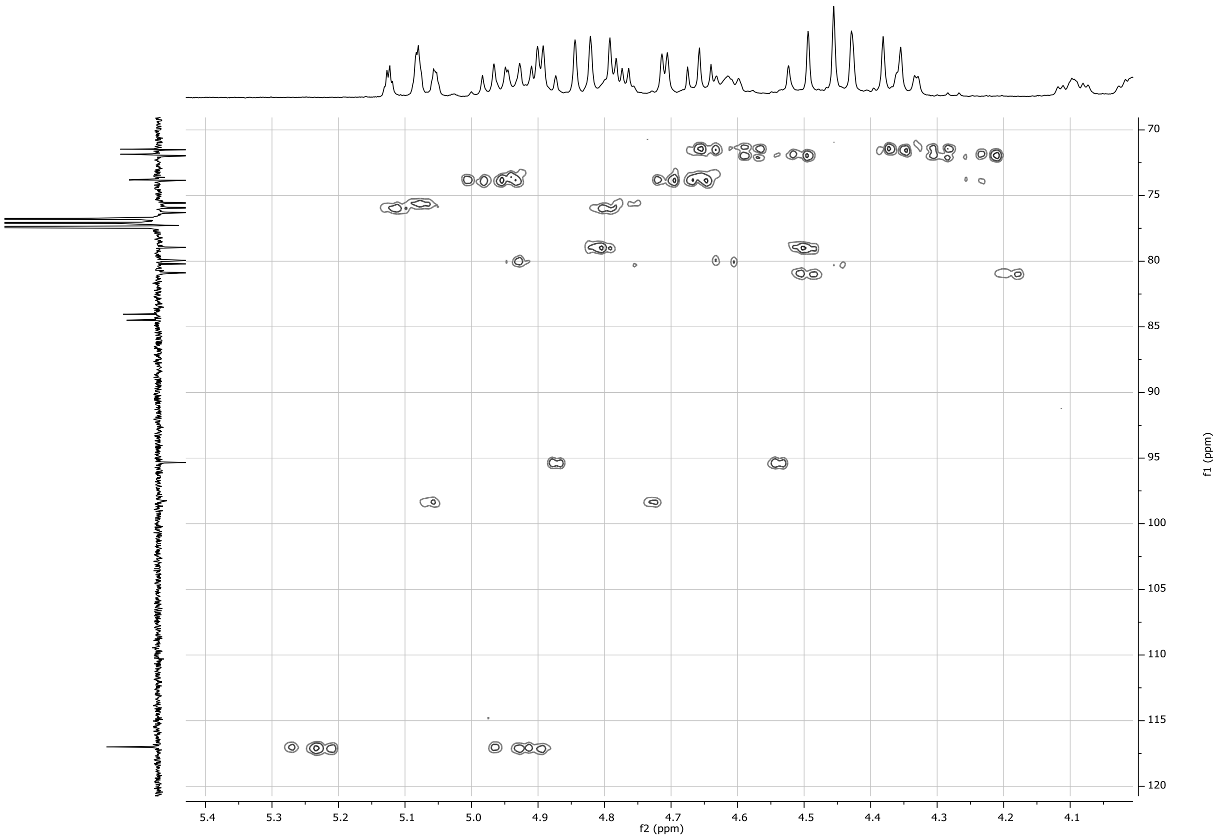

^{1}H NMR, 500 MHz, $CDCl_3$ of **33**

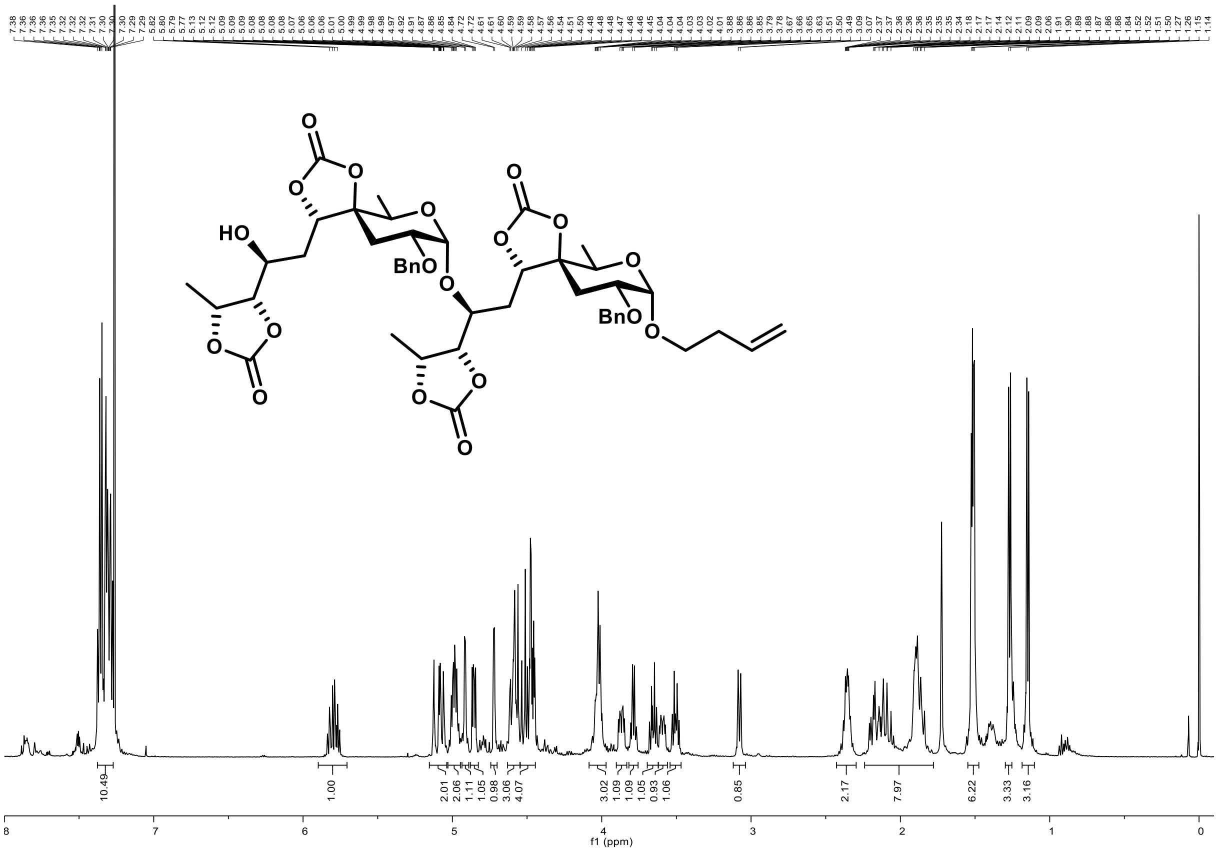

^{13}C NMR, 126 MHz, $CDCl_3$ of **33**

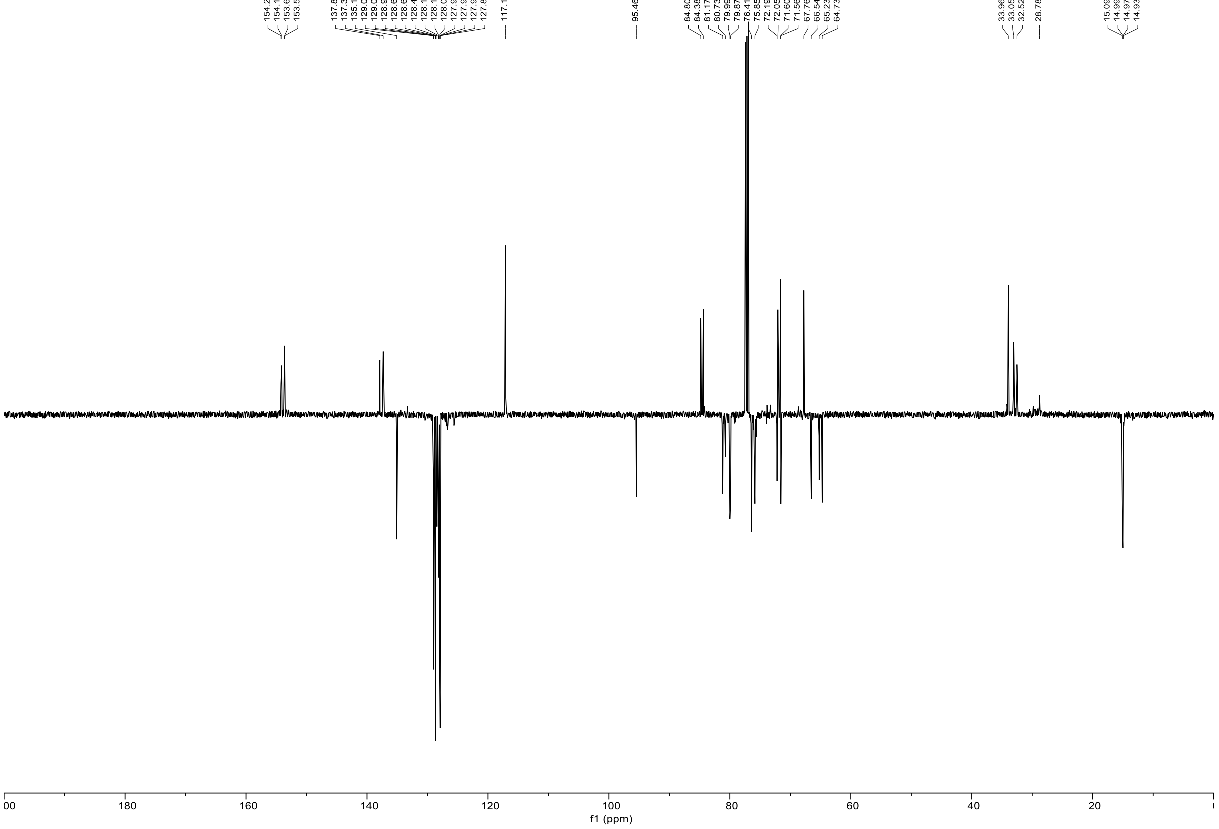

HH-COSY NMR, $CDCl_3$ of **33**

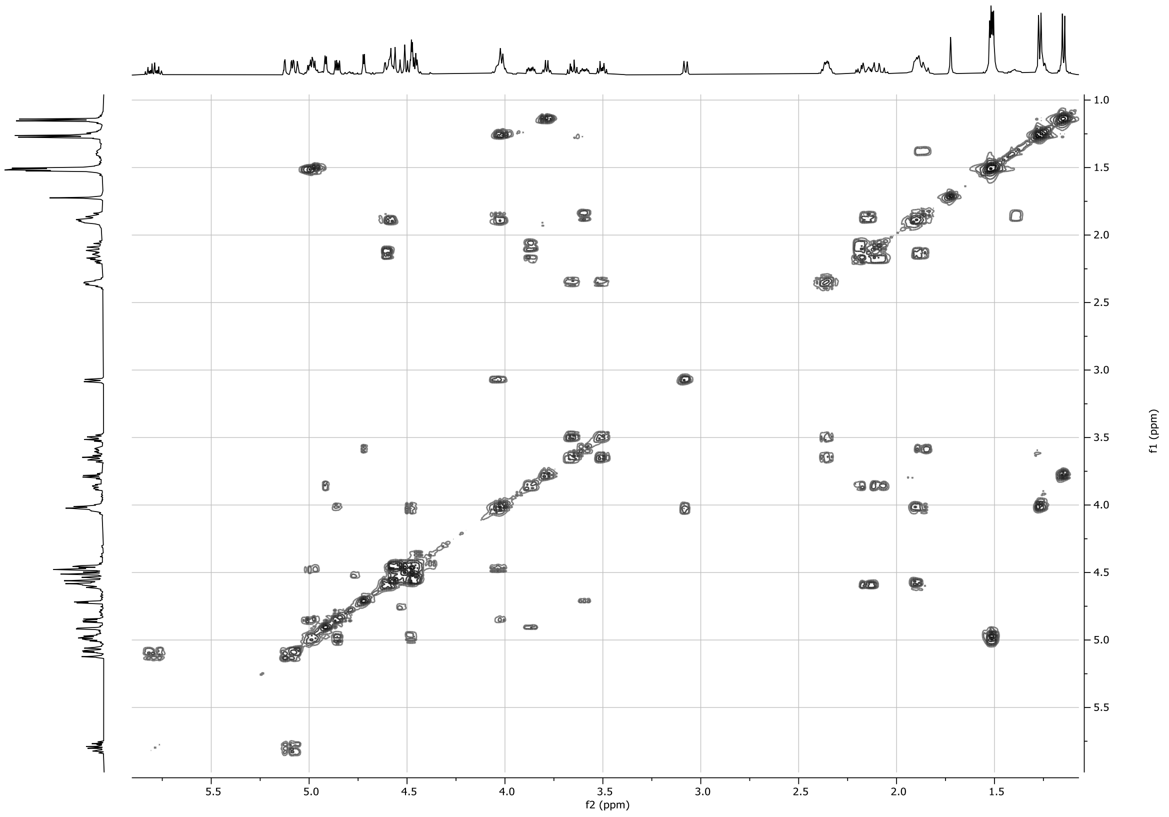

HSQC NMR, $CDCl_3$ of **33**

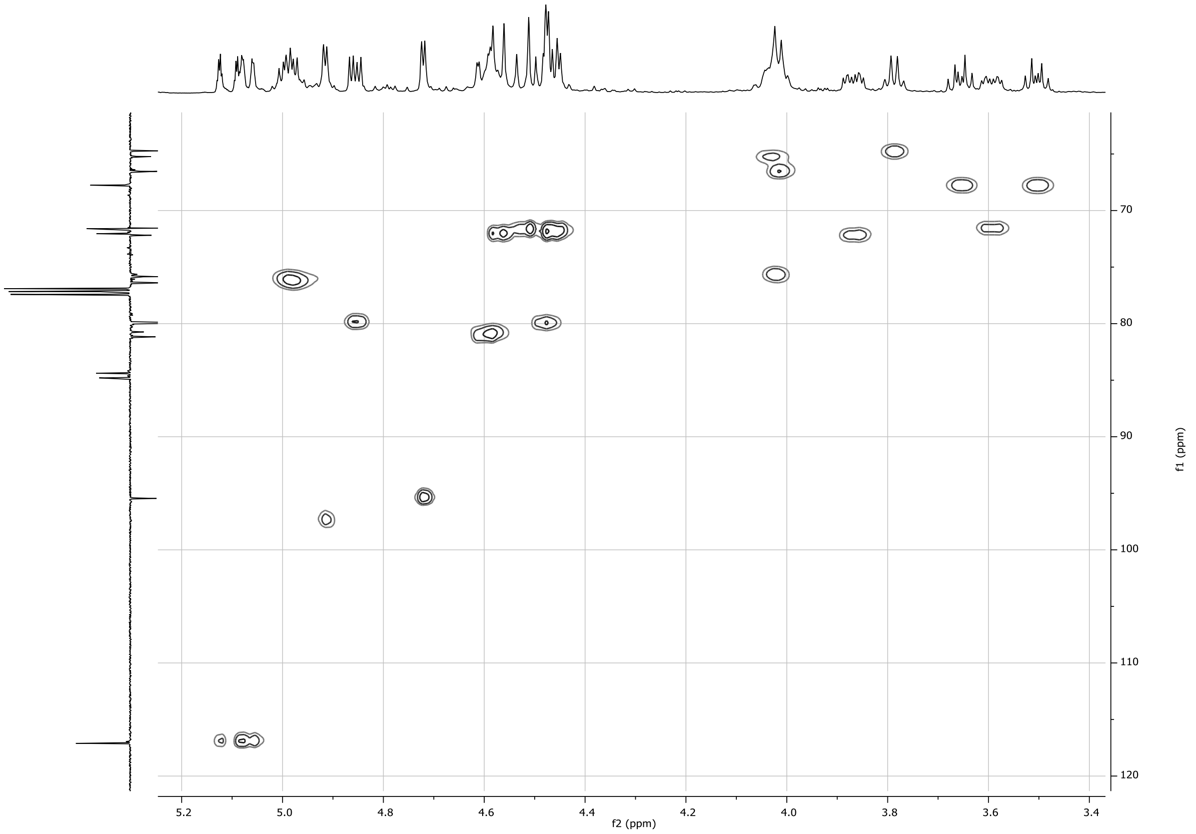

^{1}H NMR, 500 MHz, $CDCl_3$ of **34**

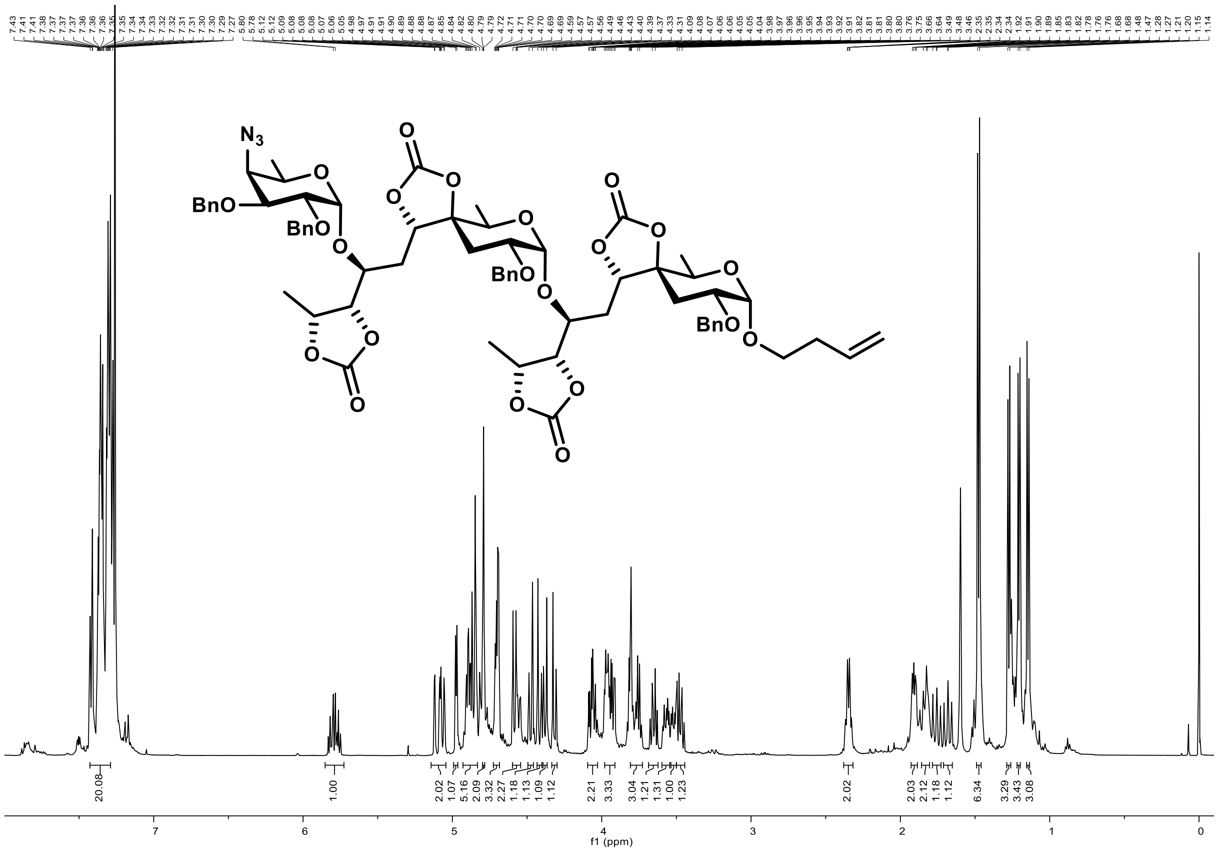

^{13}C NMR, 126 MHz, $CDCl_3$ of **34**

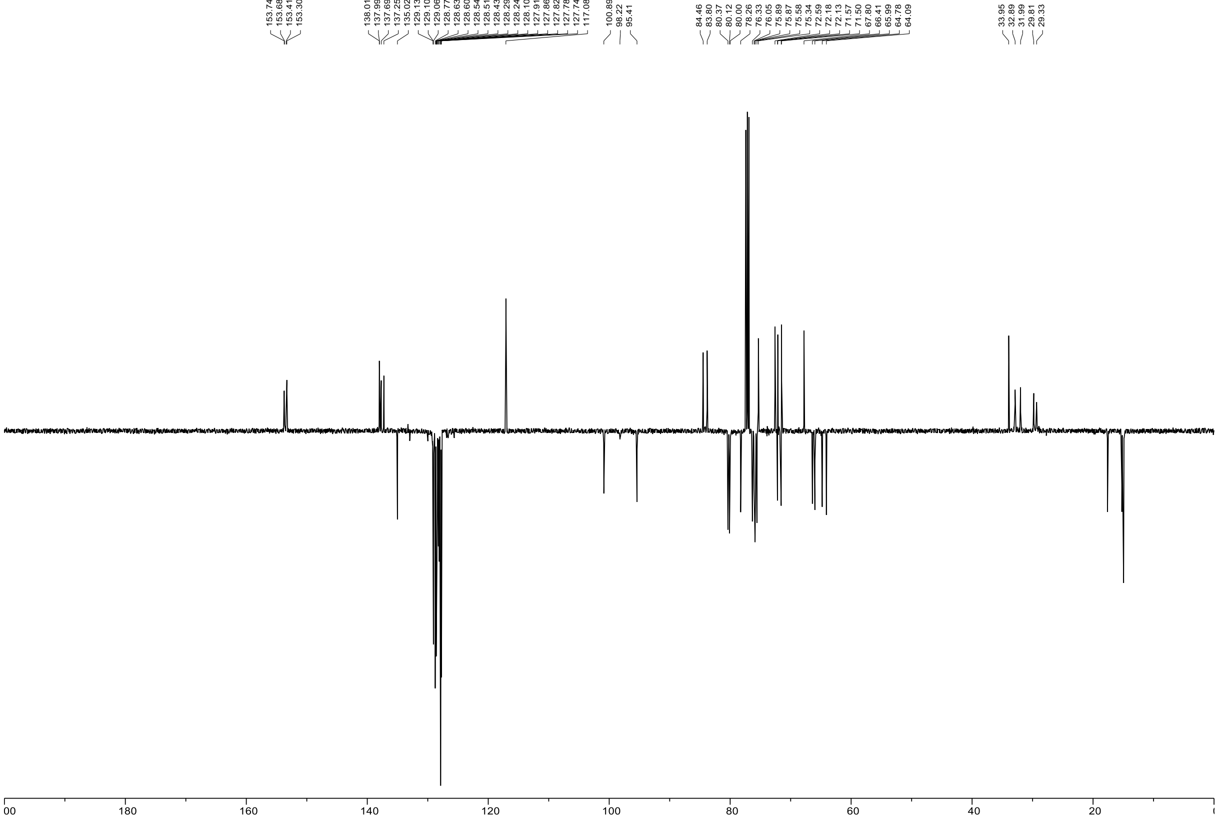

HH-COSY NMR, $CDCl_3$ of **34**

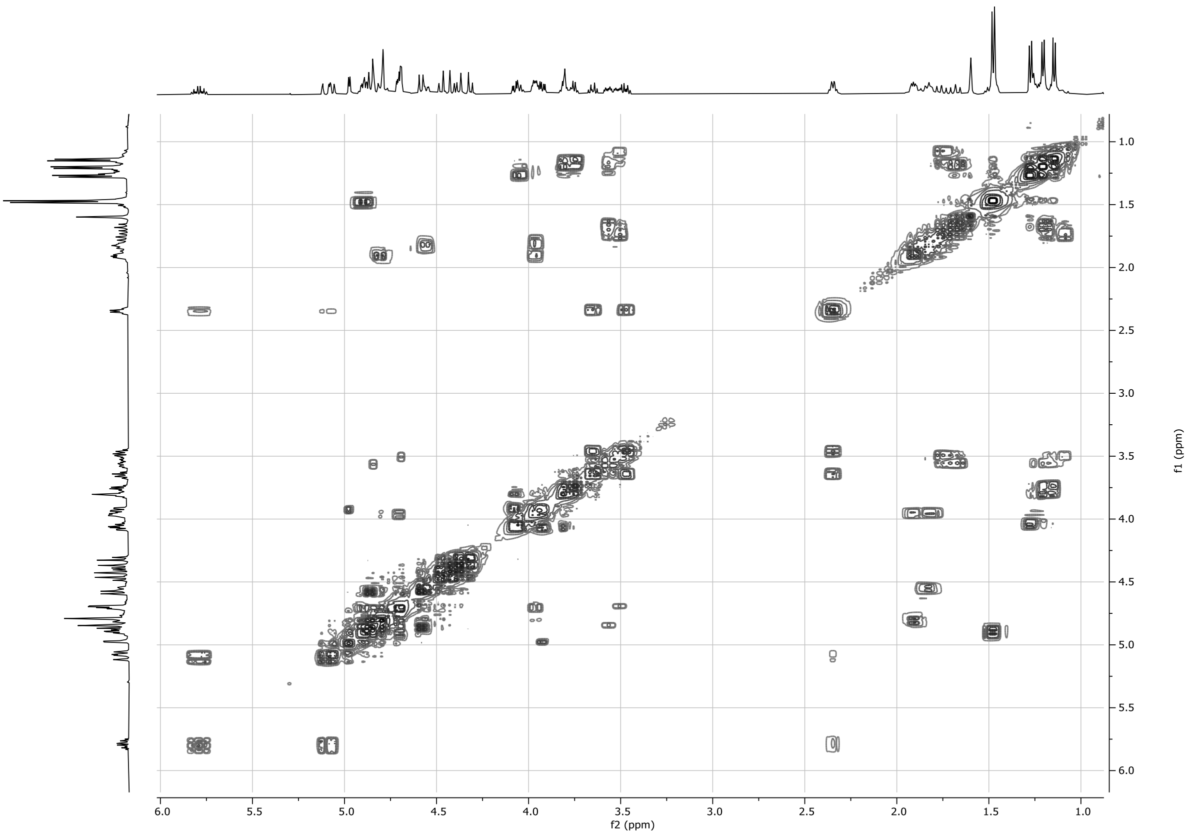

HSQC NMR, $CDCl_3$ of **34**

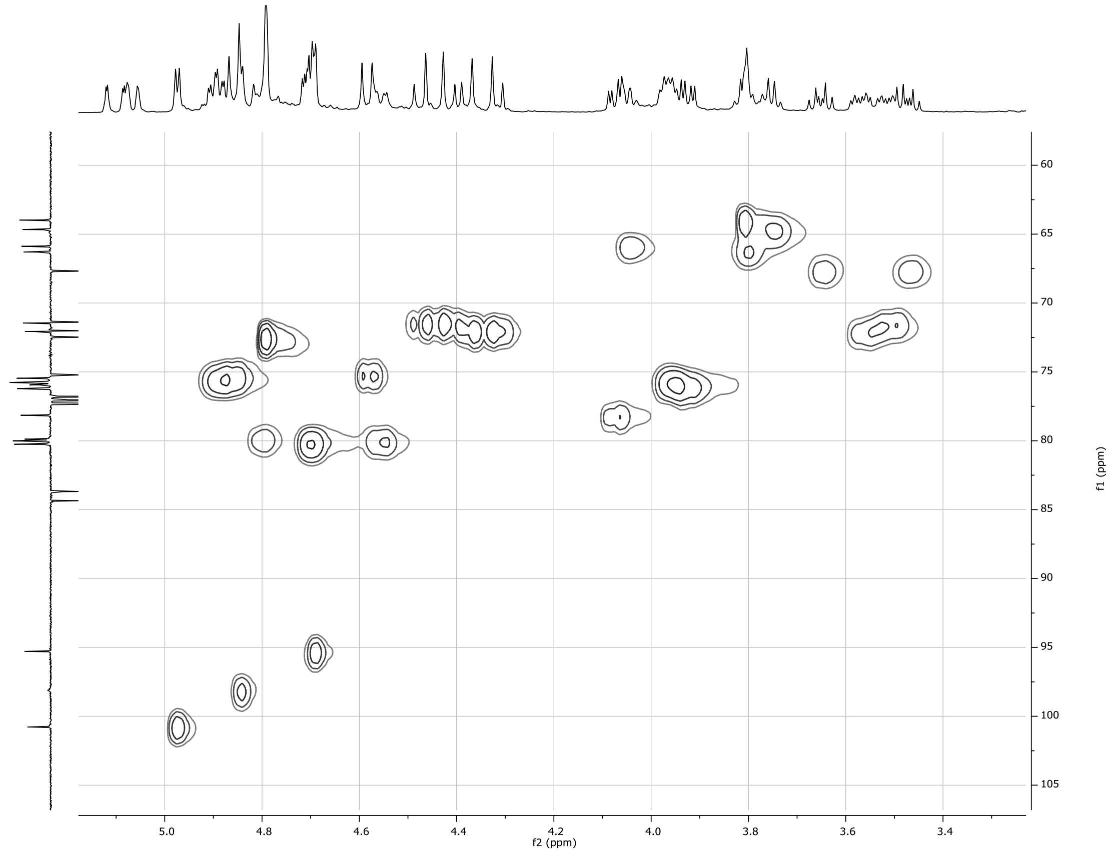

HMBC-GATED NMR, $CDCl_3$ of **34**

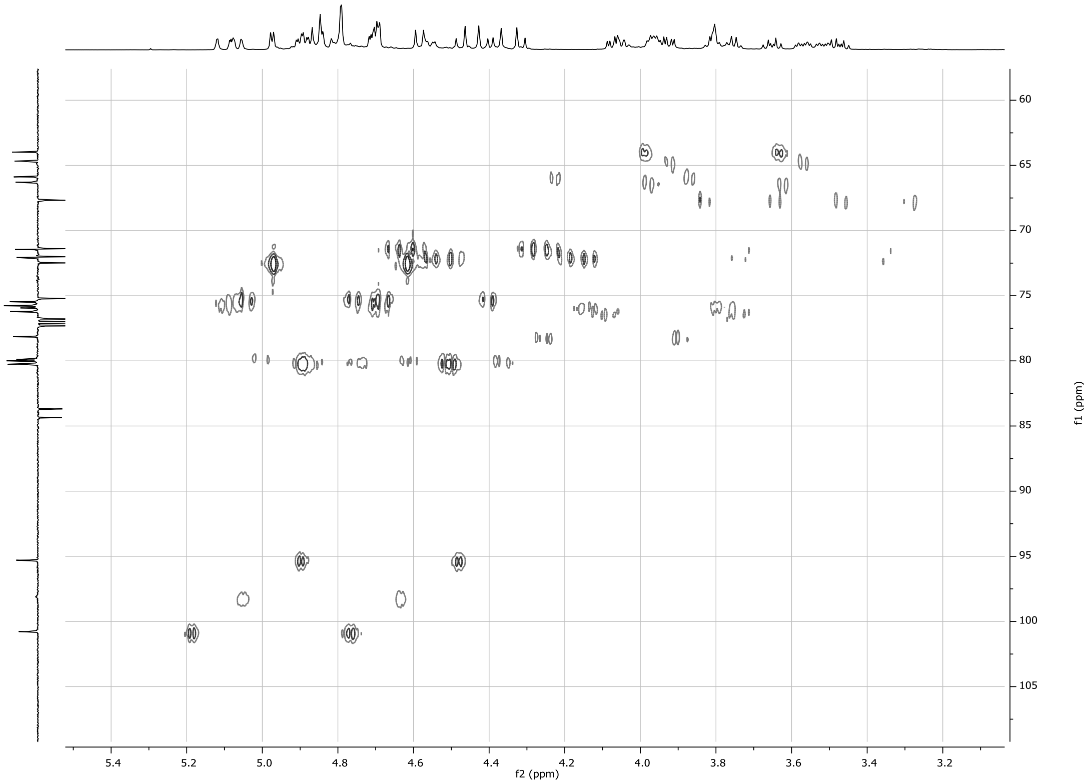

^{1}H NMR, 600 MHz, $CDCl_3$ of **35**

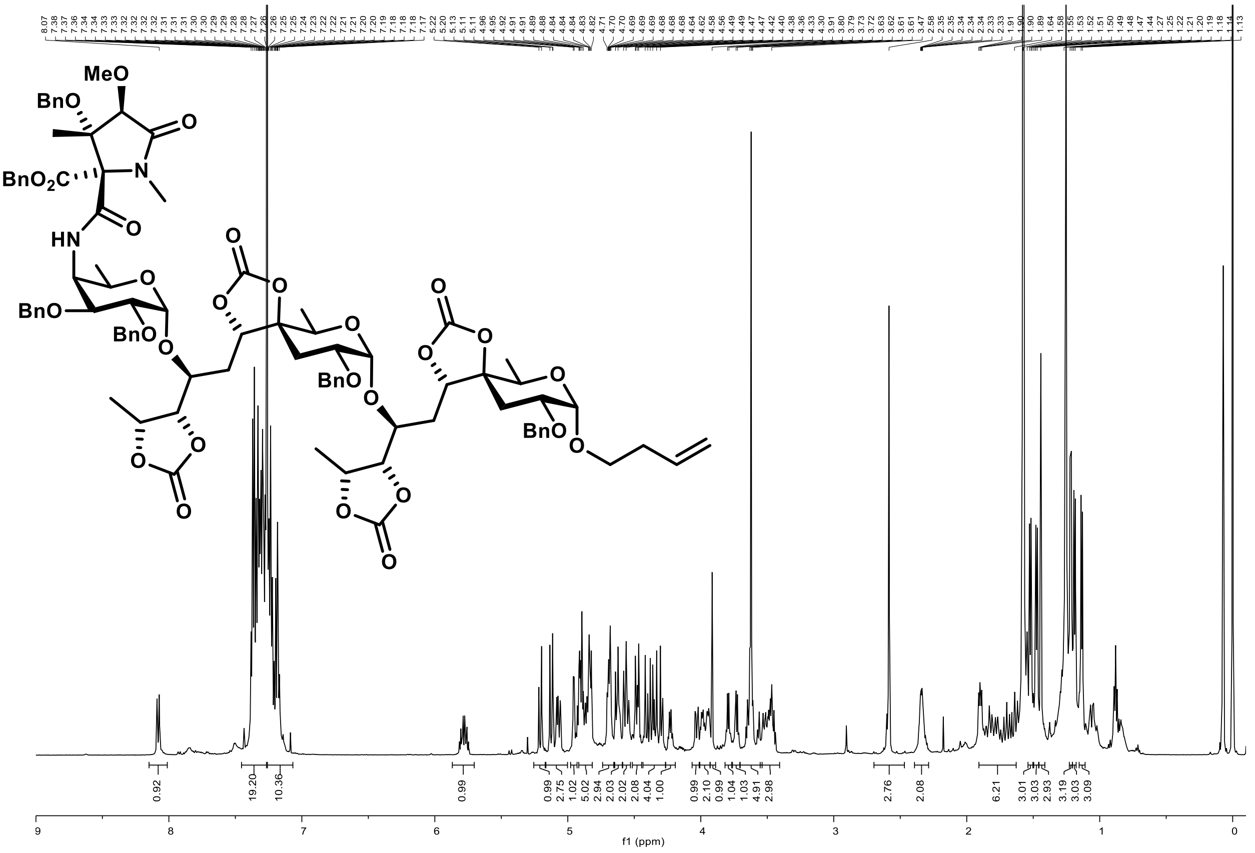

^{13}C NMR, 151 MHz, $CDCl_3$ of **35**

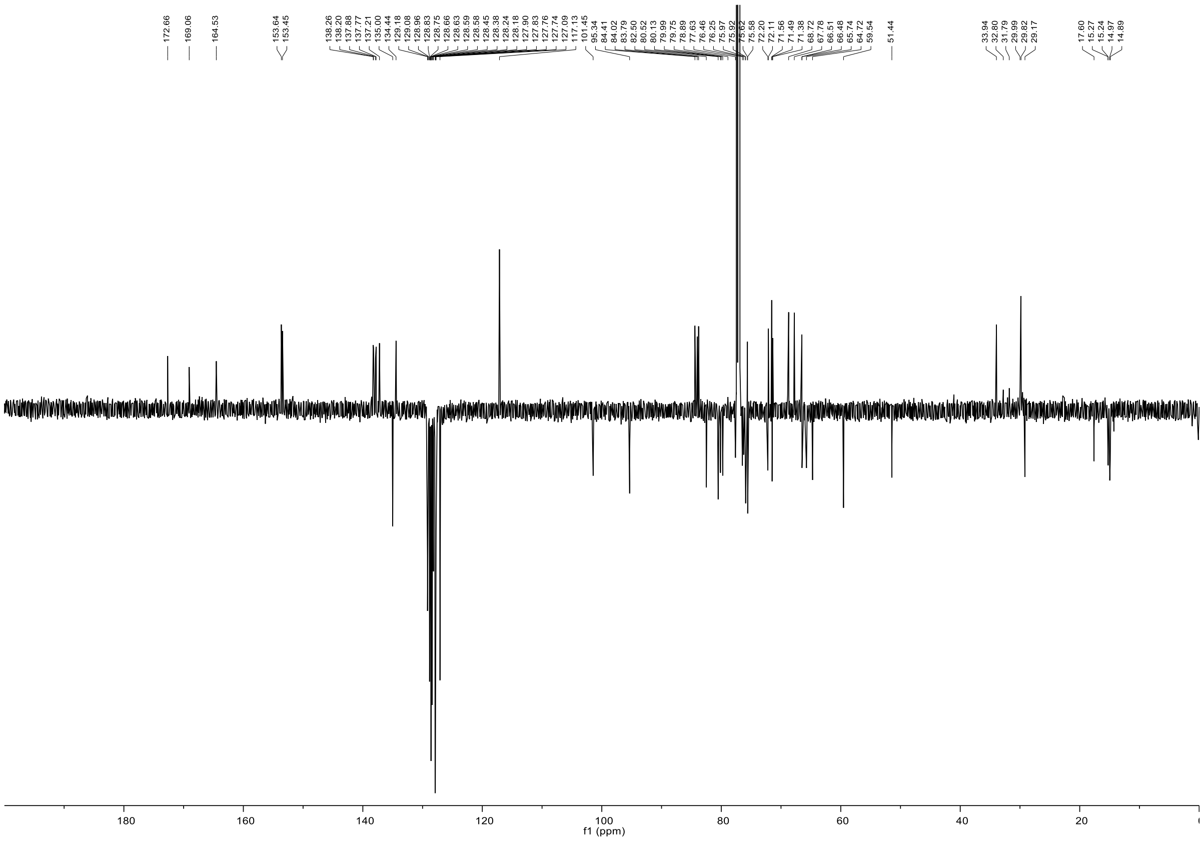

HH-COSY NMR, $CDCl_3$ of **35**

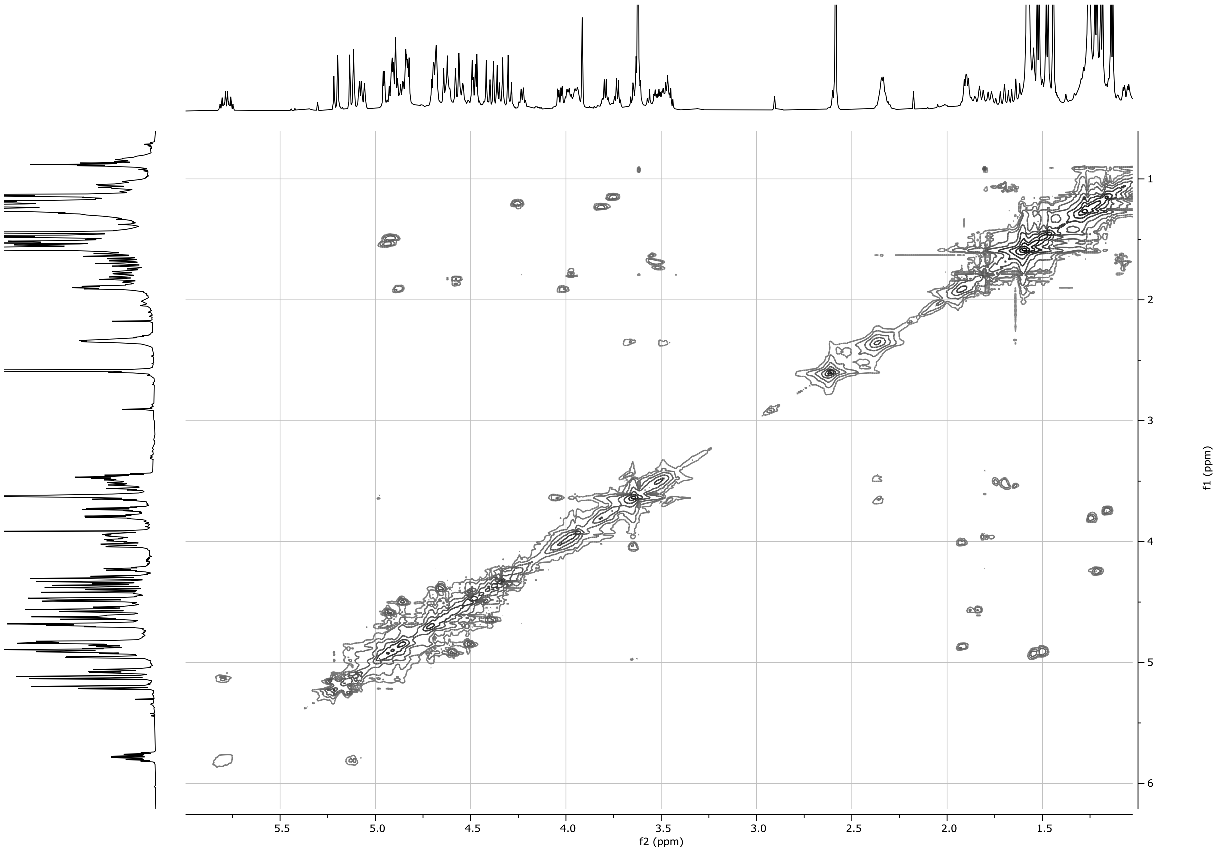

HSQC NMR, $CDCl_3$ of **35**

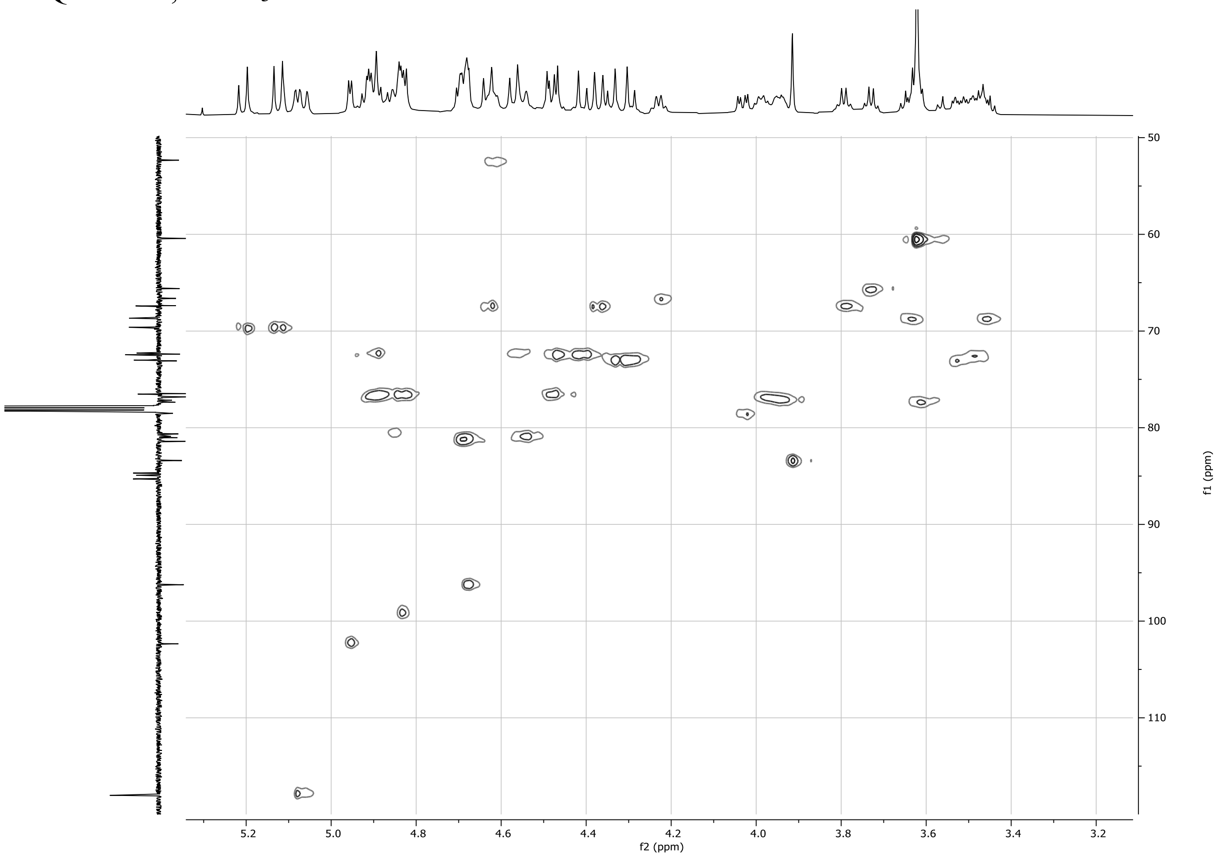

^{1}H NMR, 850 MHz, D_2O of **1**

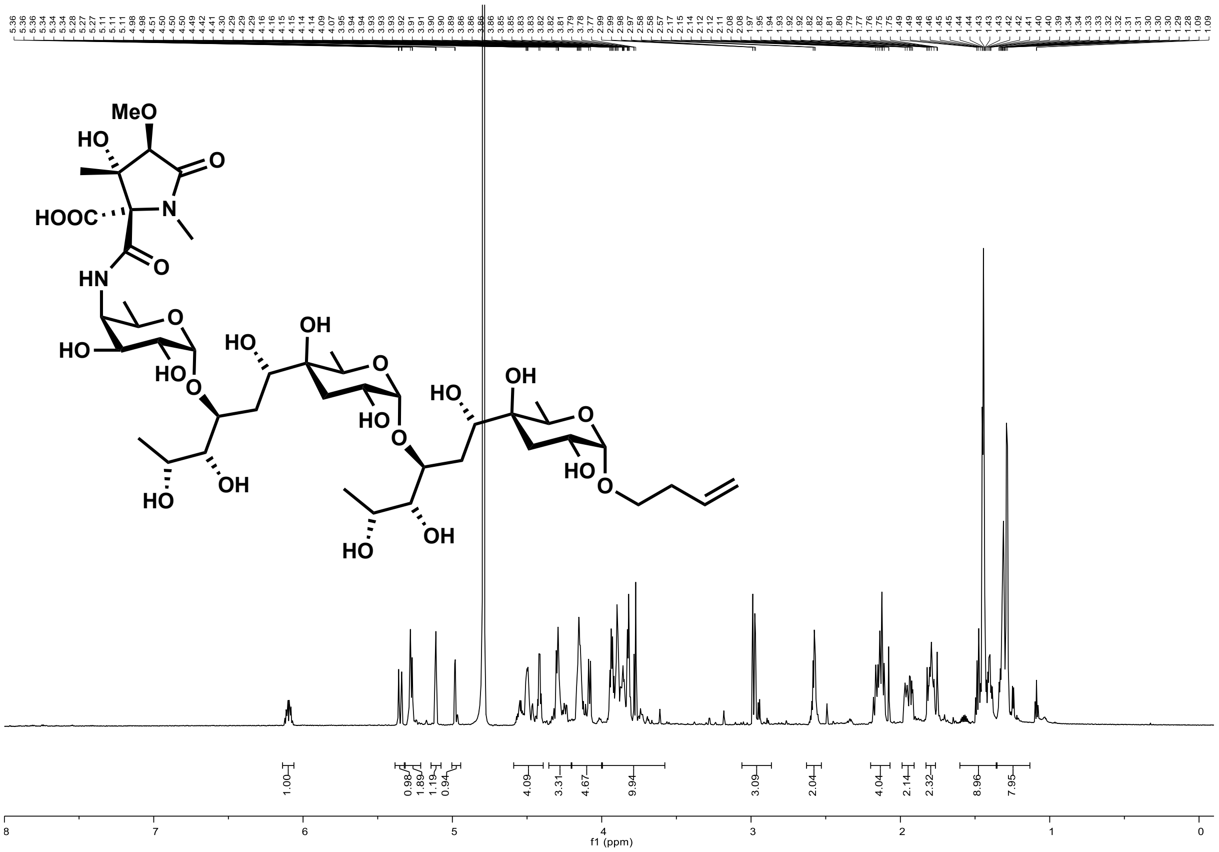

^{13}C NMR, 214 MHz, D_2O of **1**

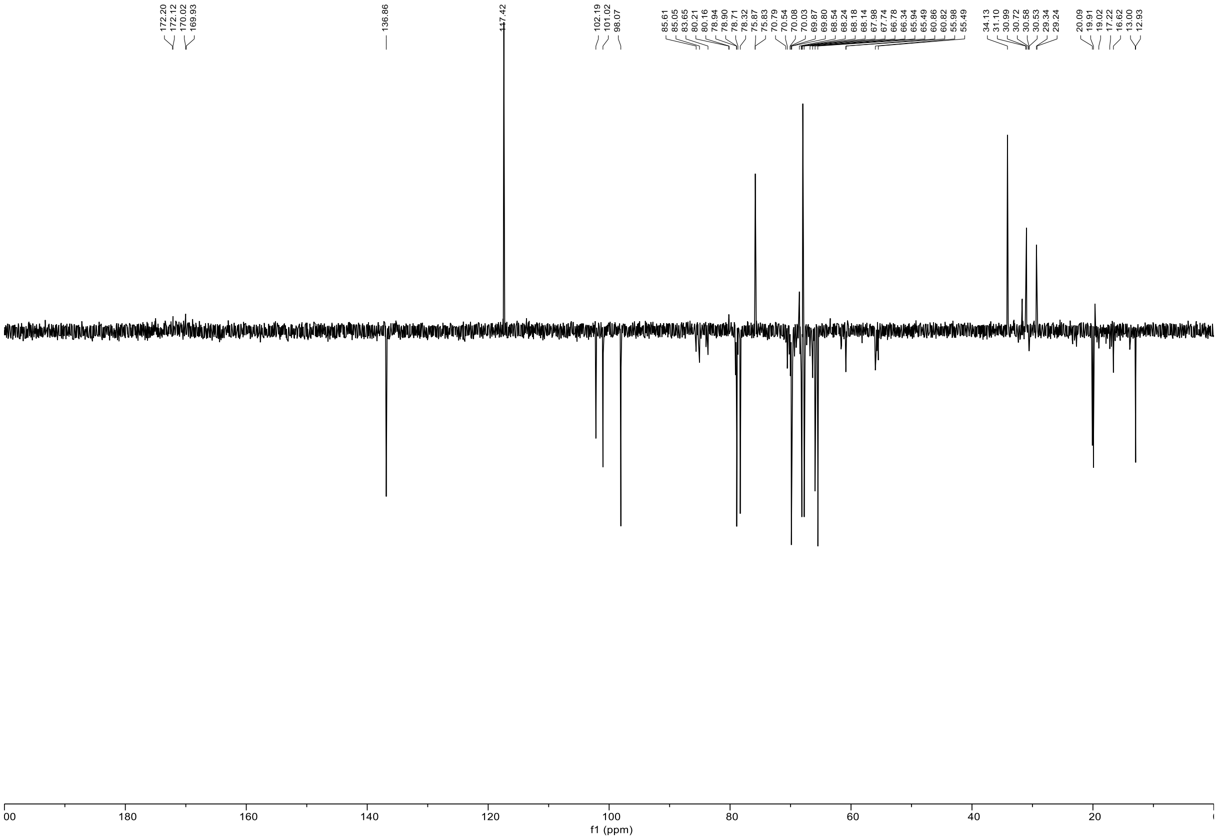

HH-COSY NMR, D_2O of **1**

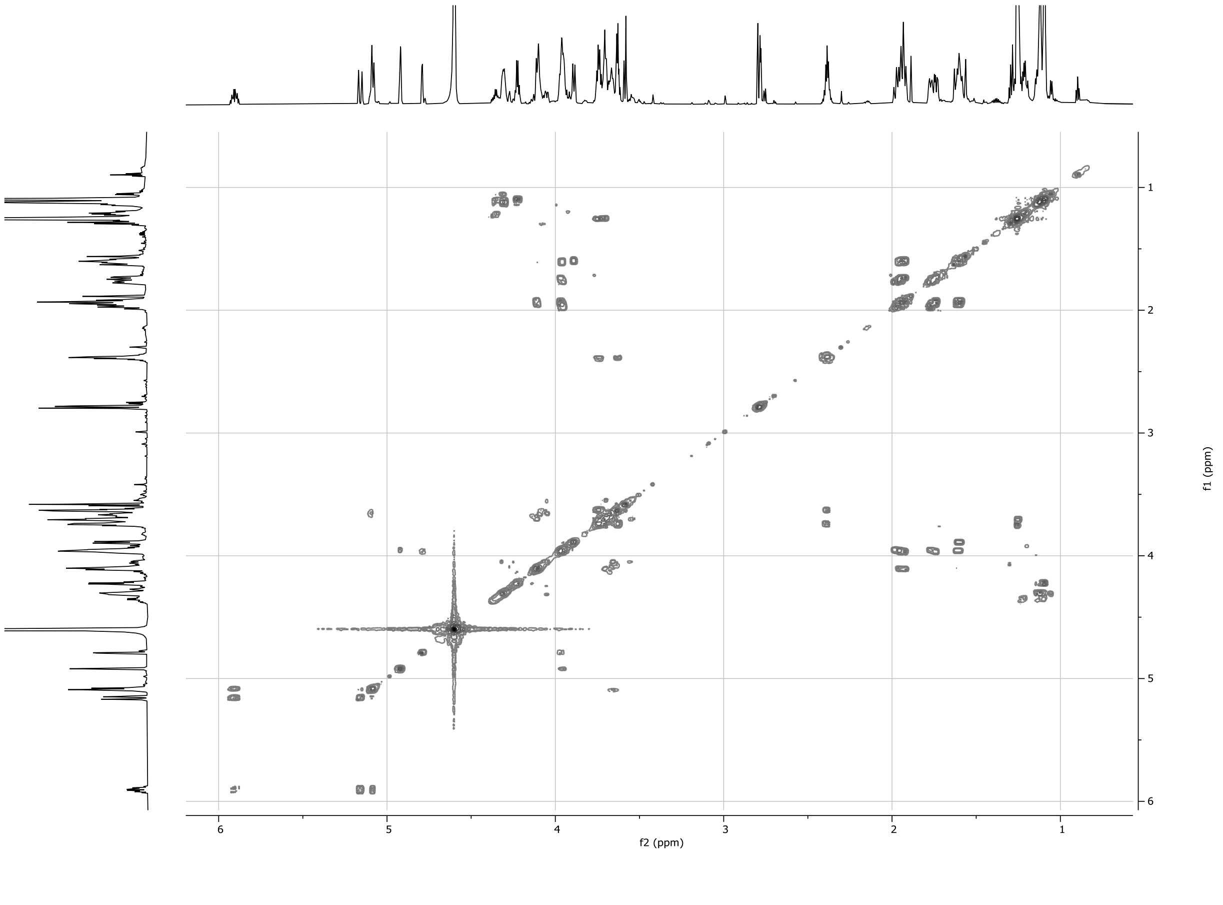

HSQC NMR, D_2O of **1**

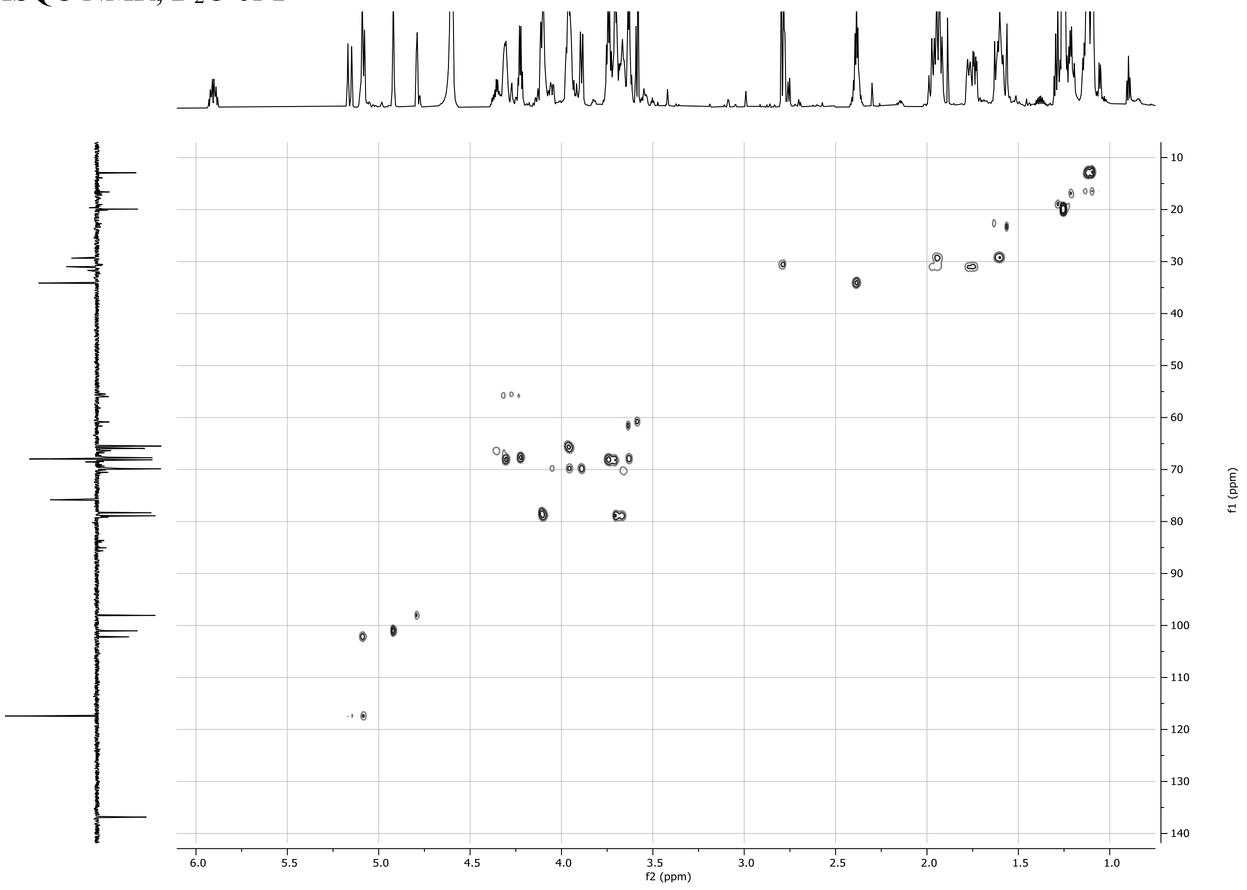

^{1}H NMR, 400 MHz, $CDCl_3$ of **S41**

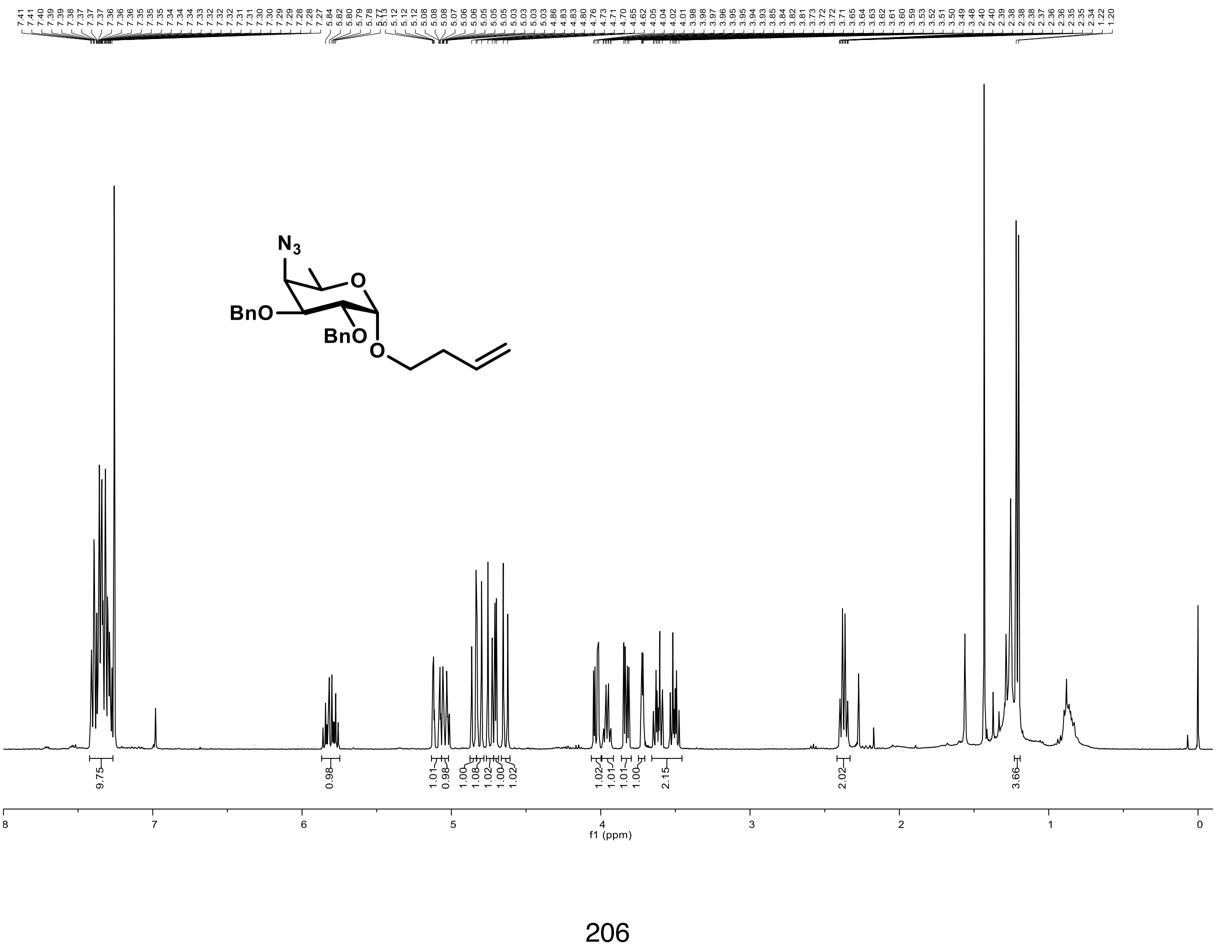

^{13}C NMR, 101 MHz, $CDCl_3$ of **S41**

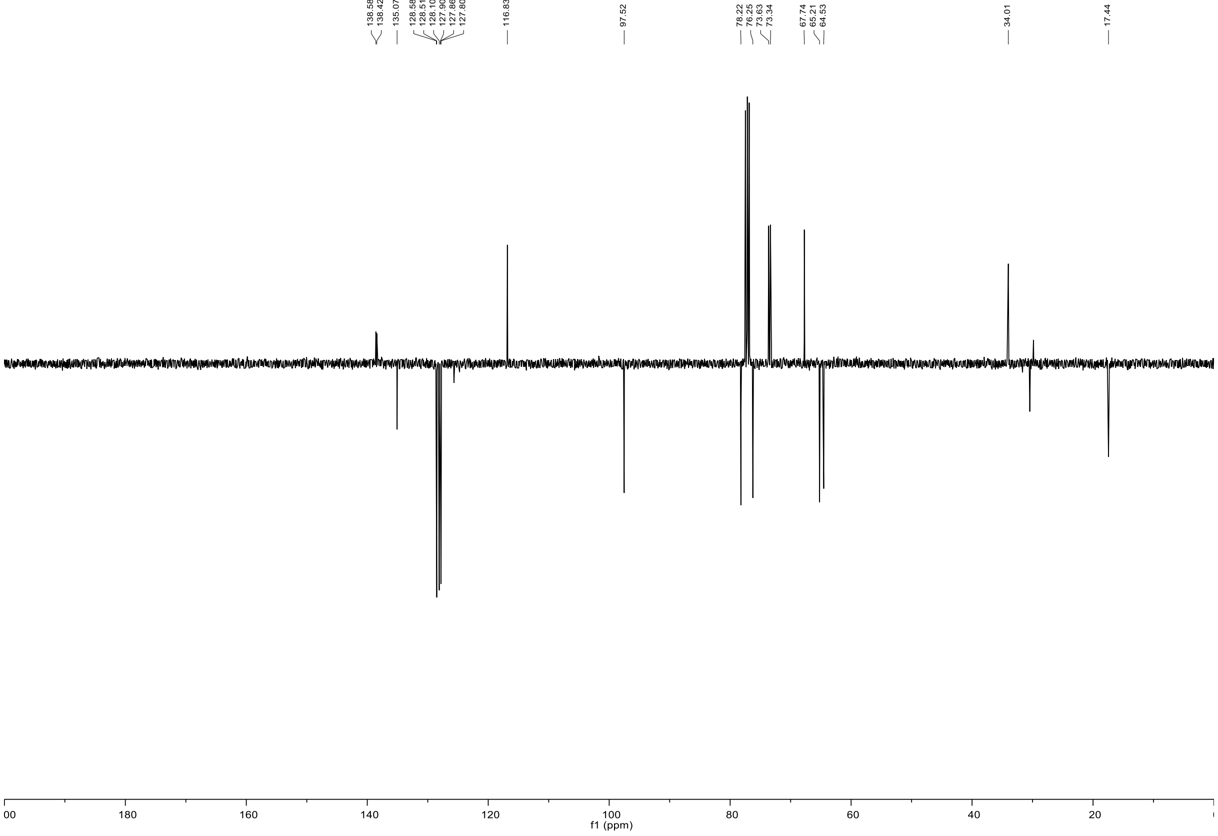

HH-COSY NMR, $CDCl_3$ of **S41**

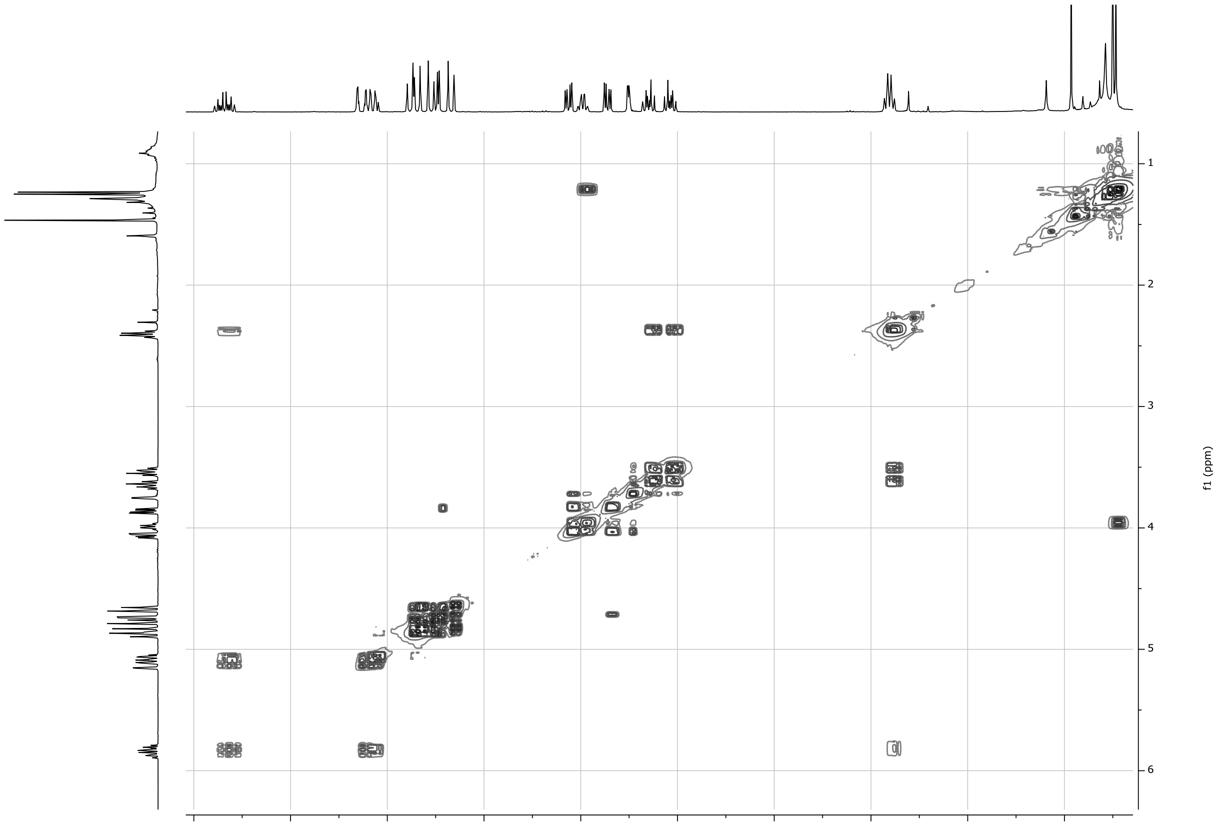

HSQC NMR, $CDCl_3$ of **S41**

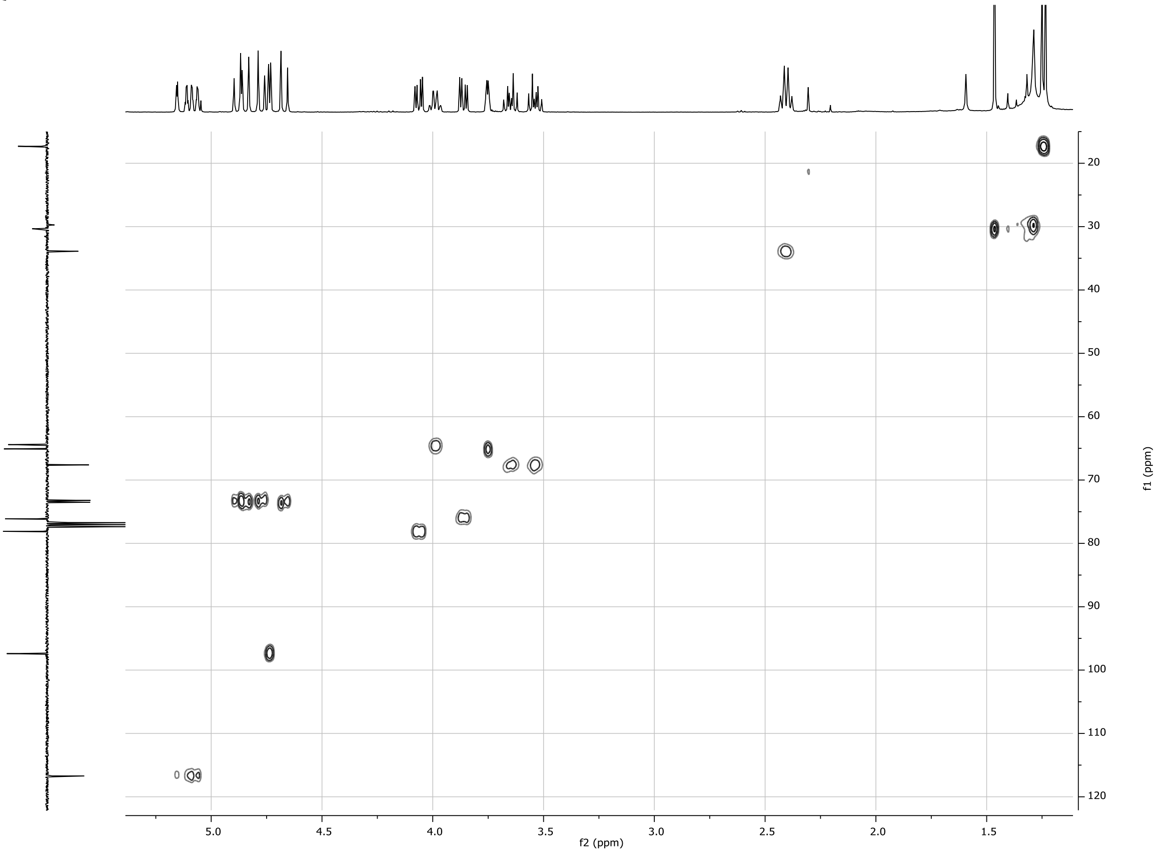

^{1}H NMR, 400 MHz, $CDCl_3$ of **S42**

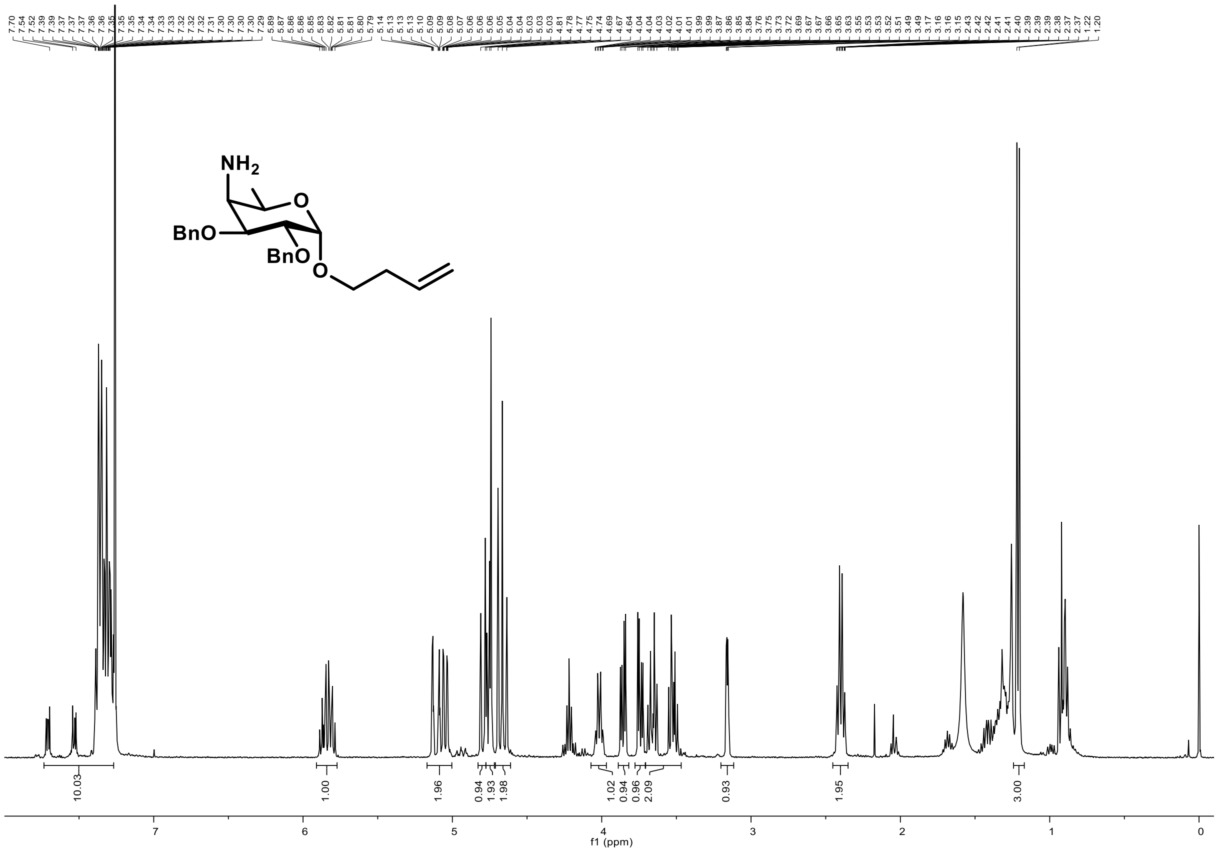

^{13}C NMR, 101 MHz, $CDCl_3$ of **S42**

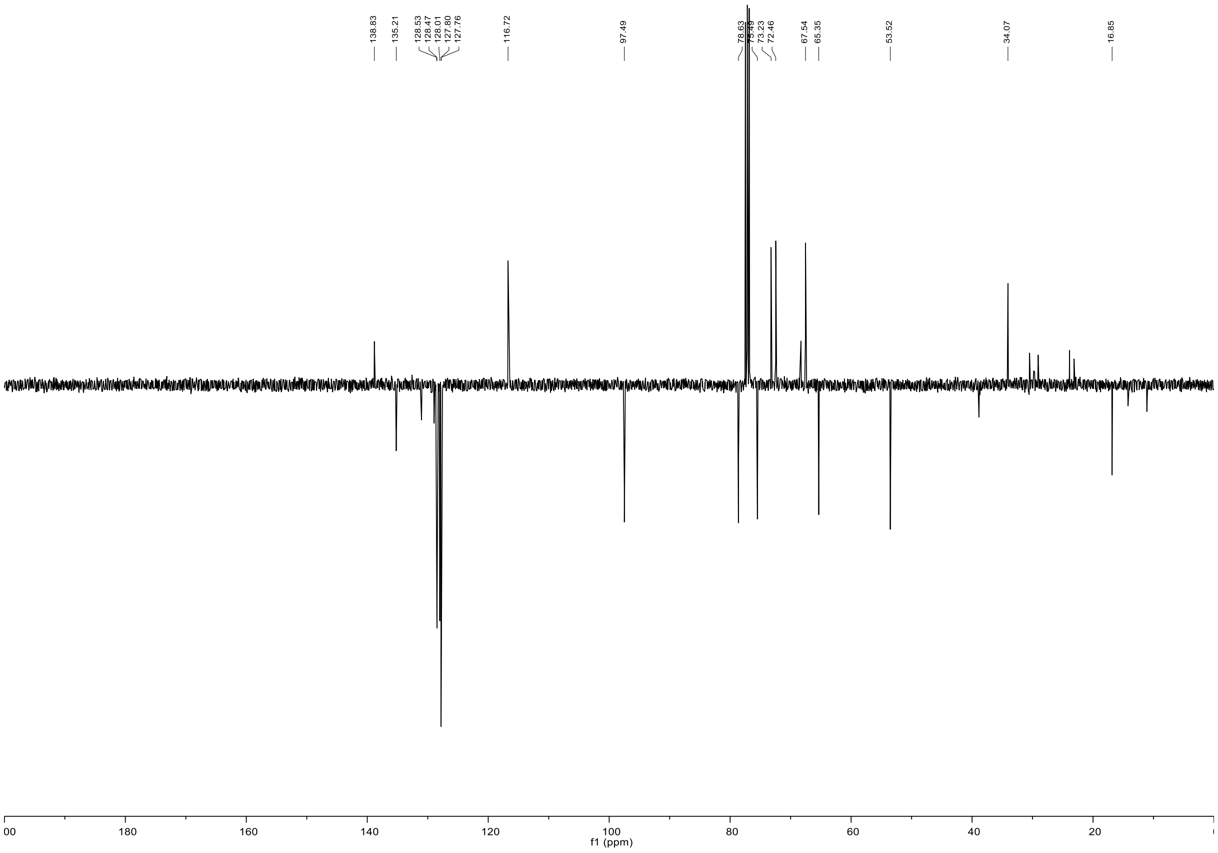

HH-COSY NMR, $CDCl_3$ of **S42**

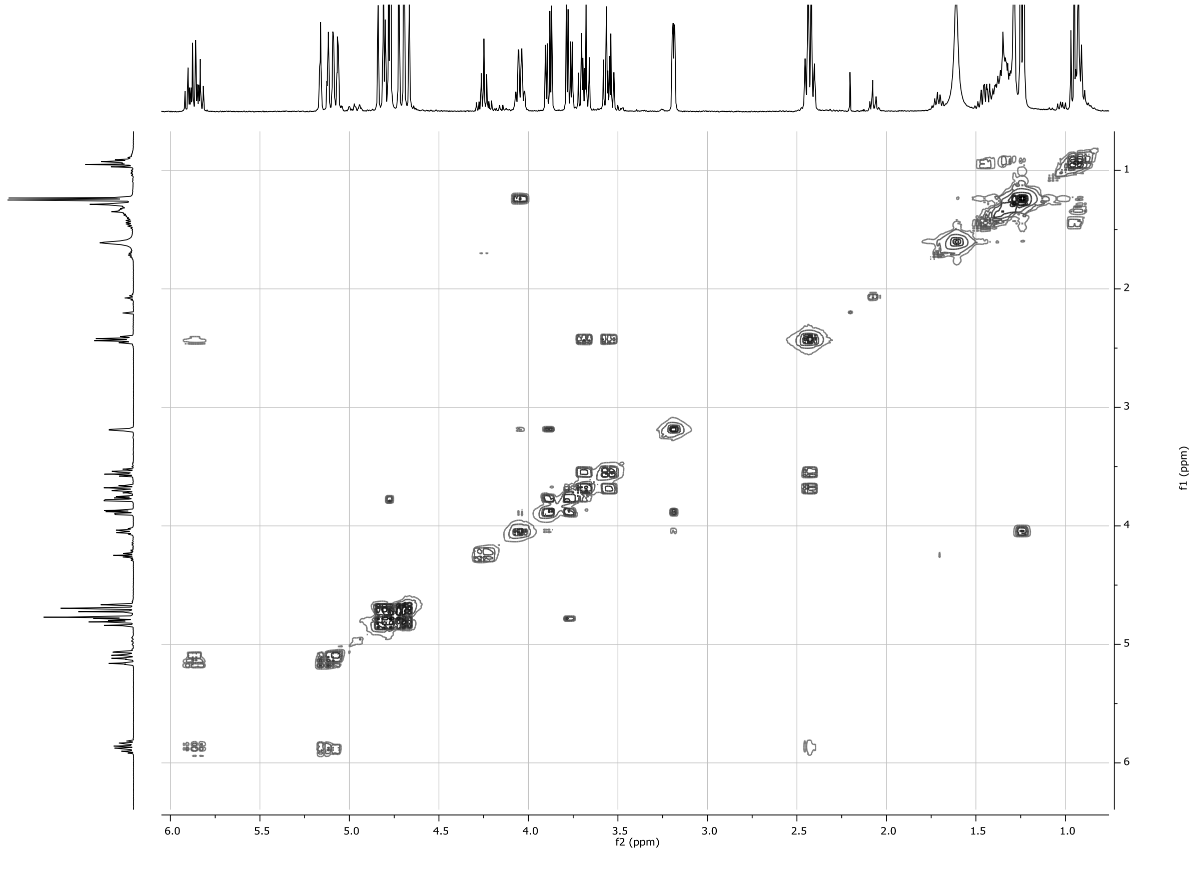

HSQC NMR, $CDCl_3$ of **S42**

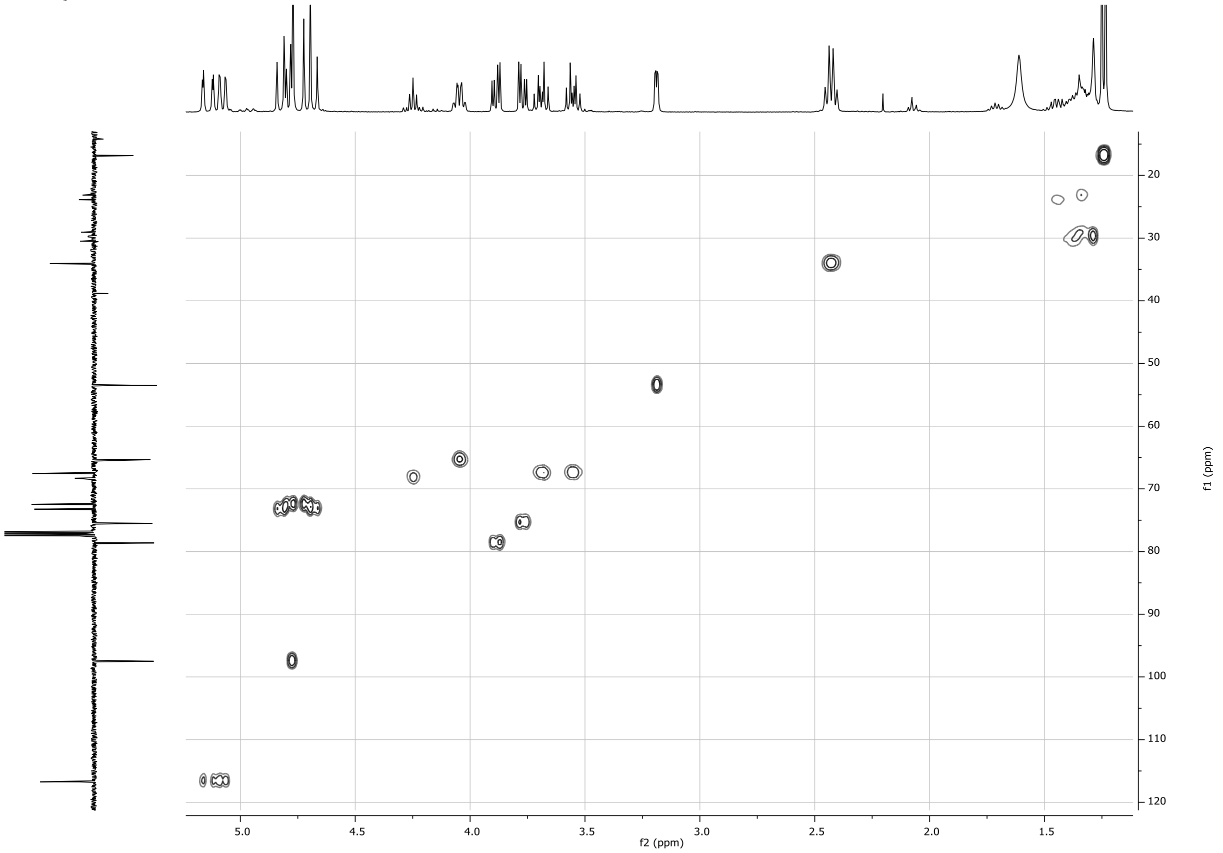

^{1}H NMR, 400 MHz, $CDCl_3$ of **S43**

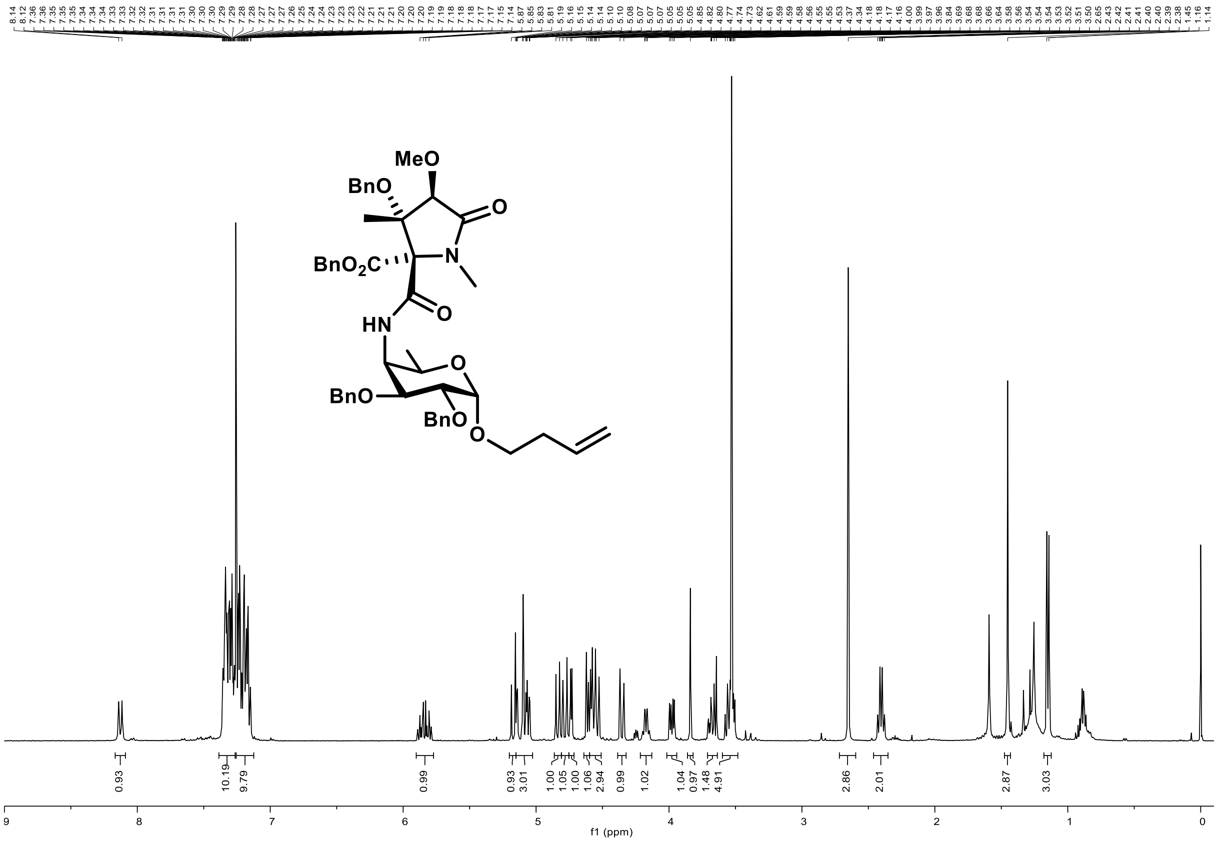

^{13}C NMR, 101 MHz, $CDCl_3$ of **S43**

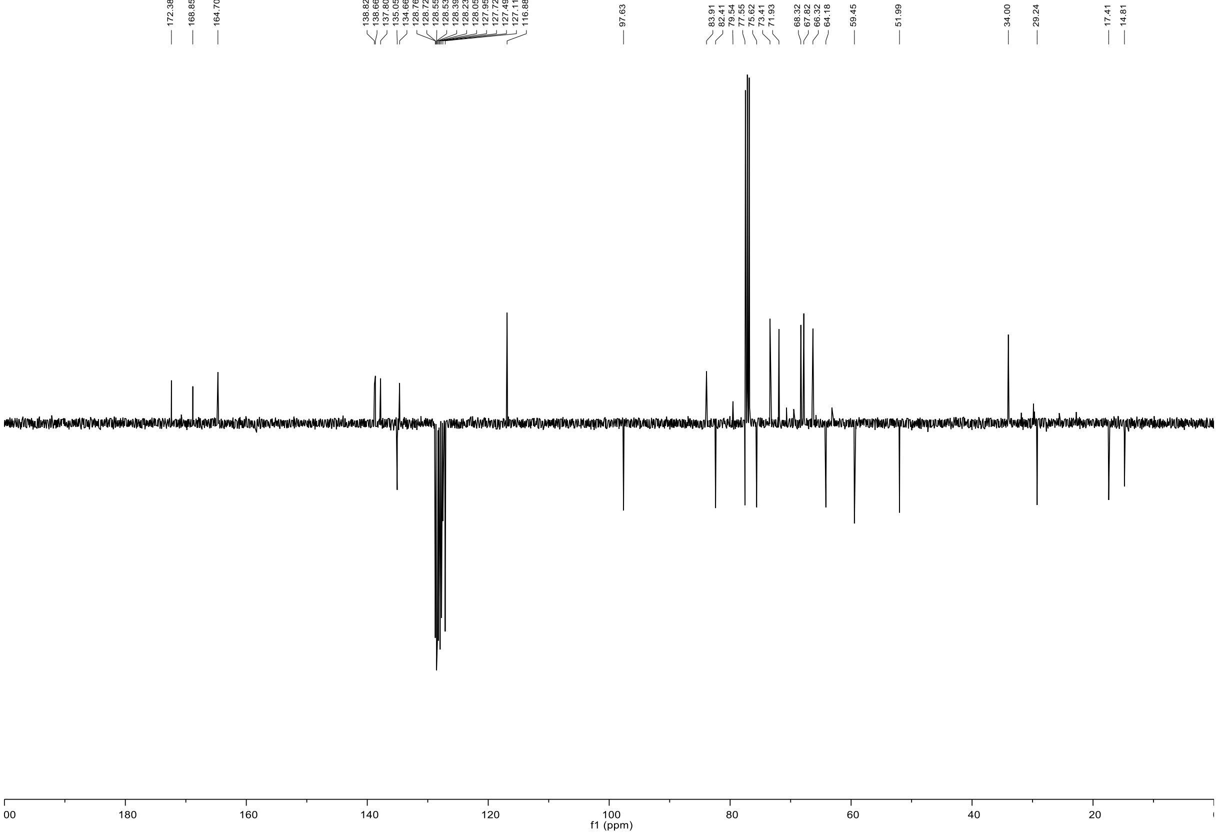

HH-COSY NMR, $CDCl_3$ of **S43**

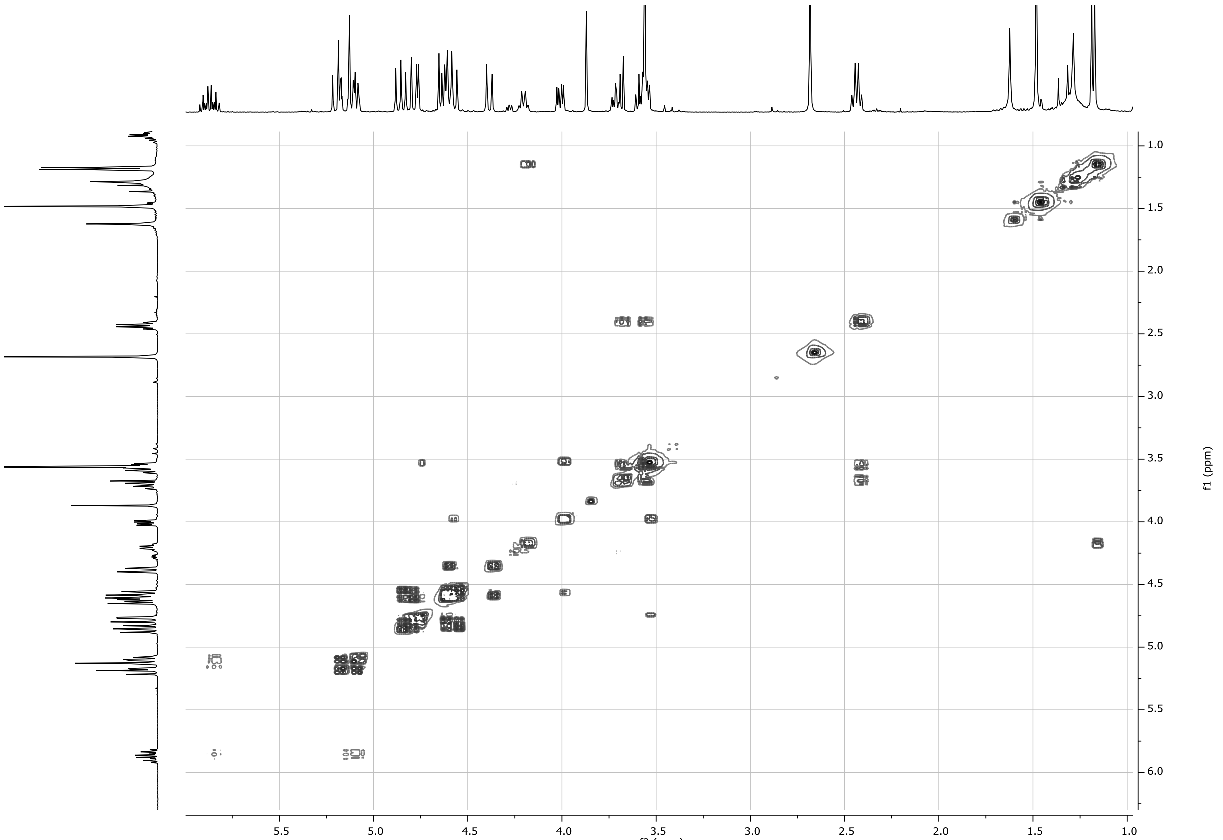

HSQC NMR, $CDCl_3$ of **S43**

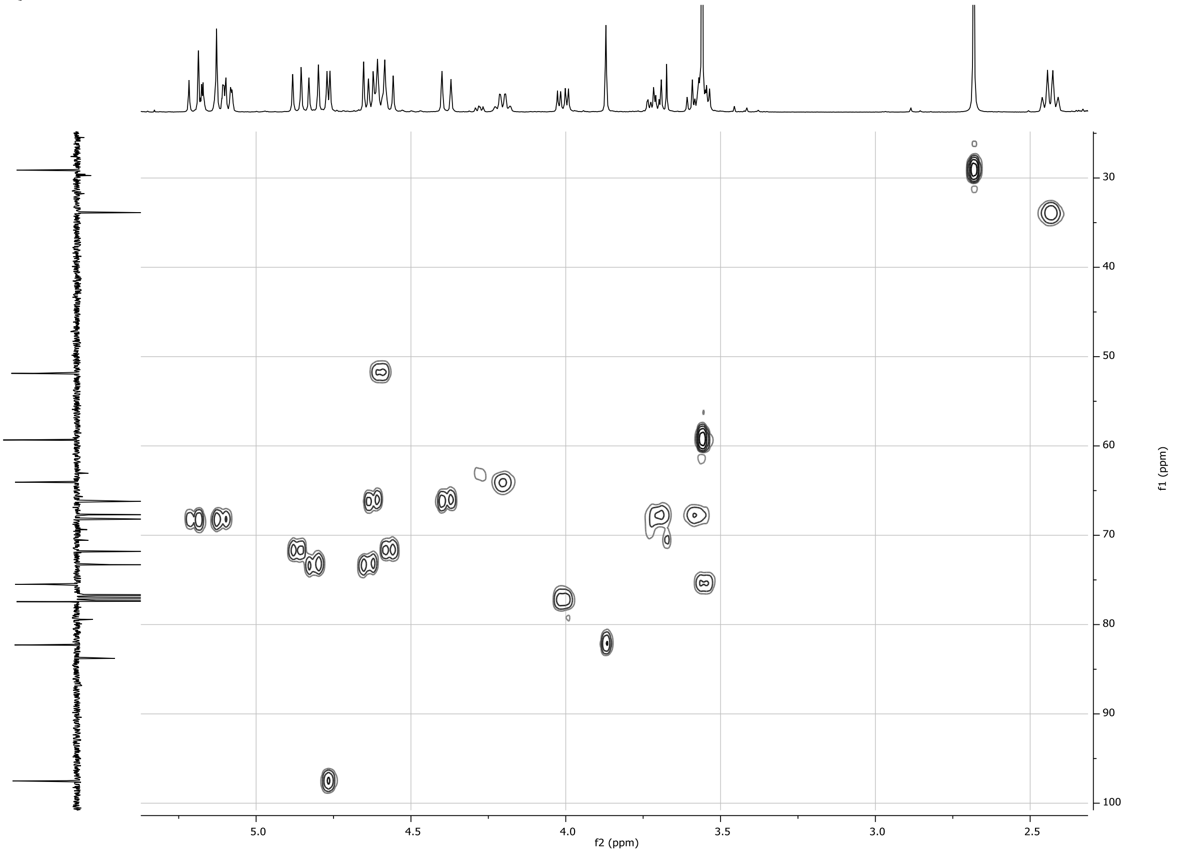

^1H NMR, 500 MHz, D_2O of **36**

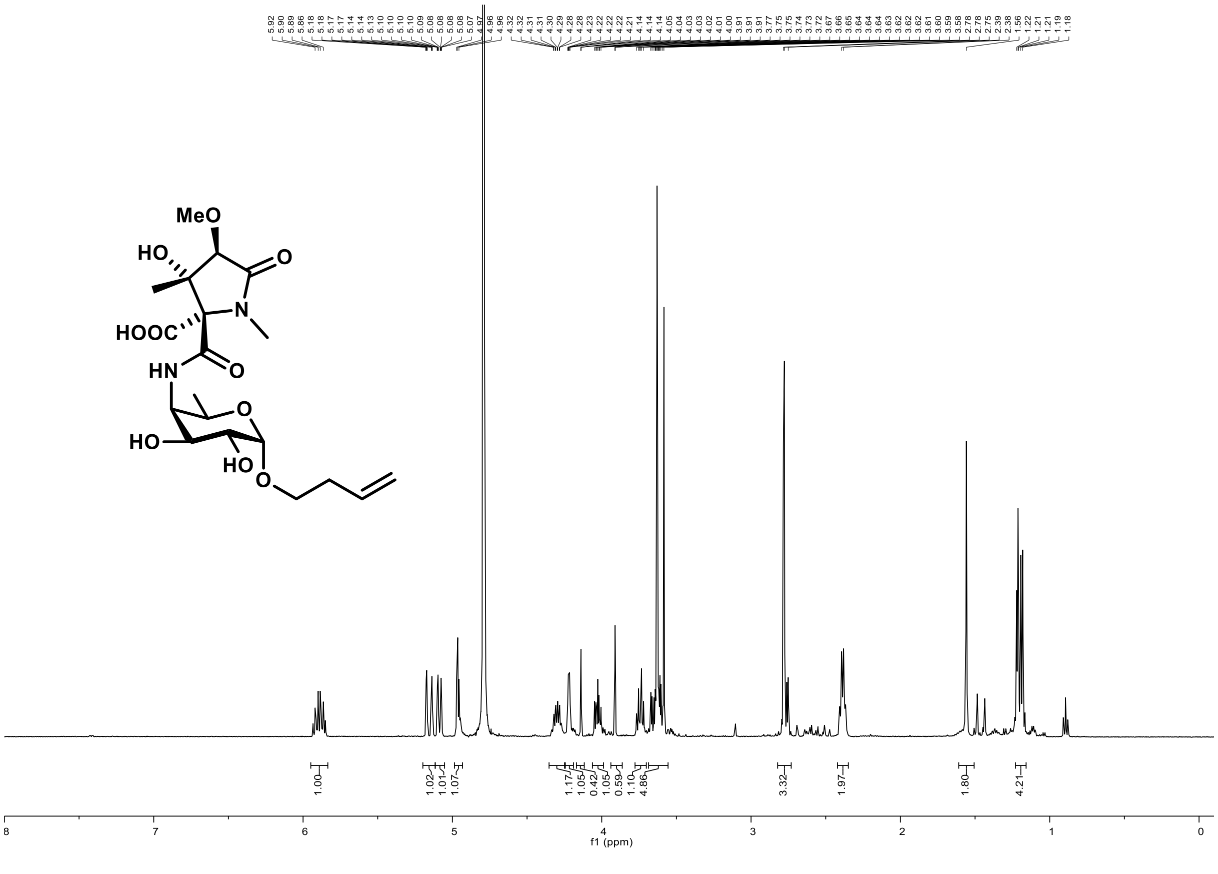

^{13}C NMR, 126 MHz, D_2O of **36**

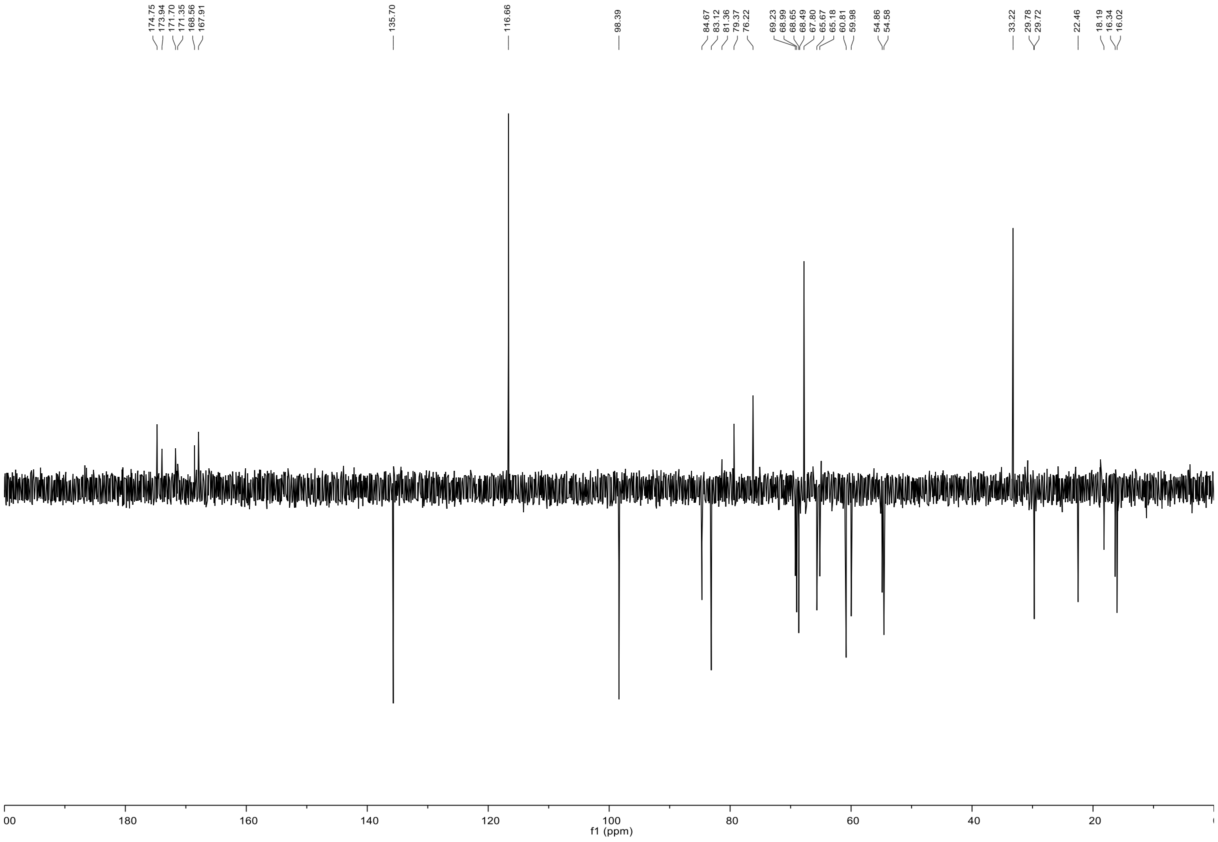

HH-COSY NMR, D_2O of **36**

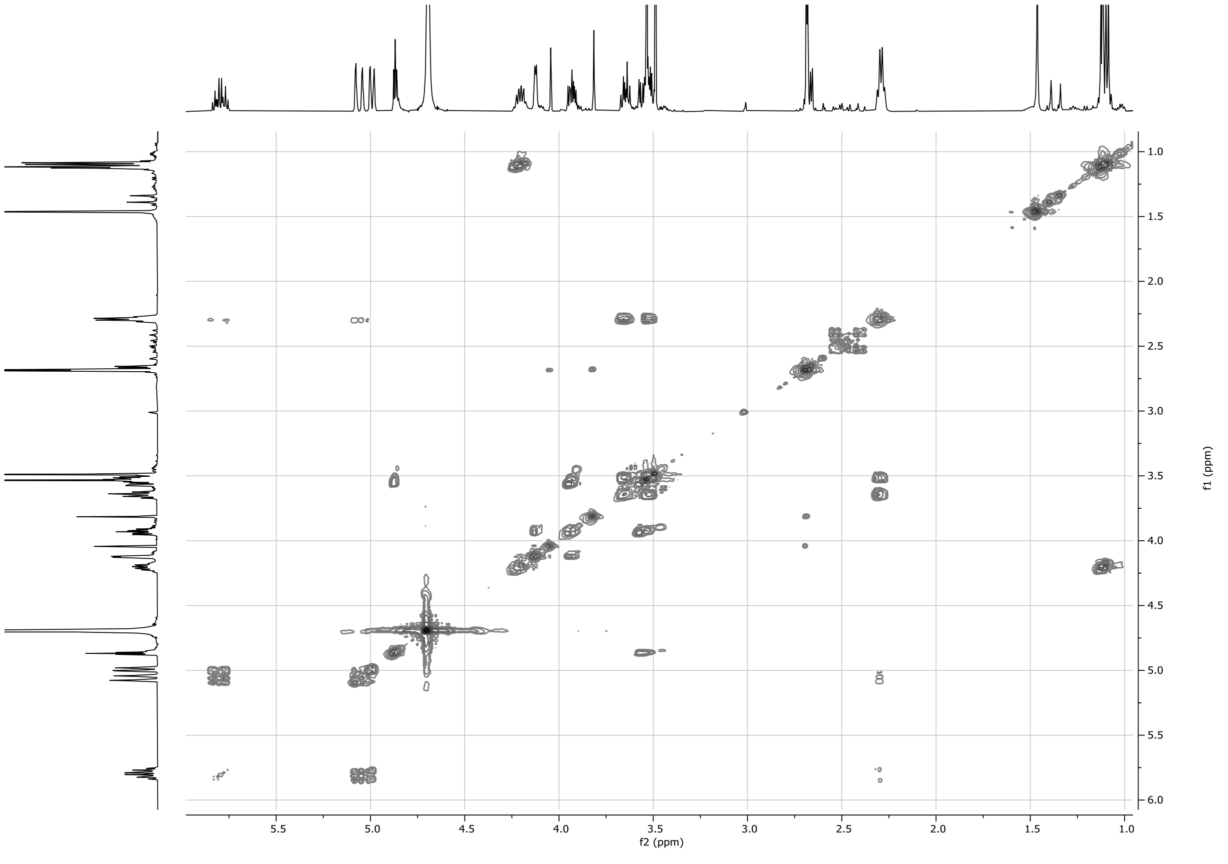

HSQC NMR, D_2O of **36**

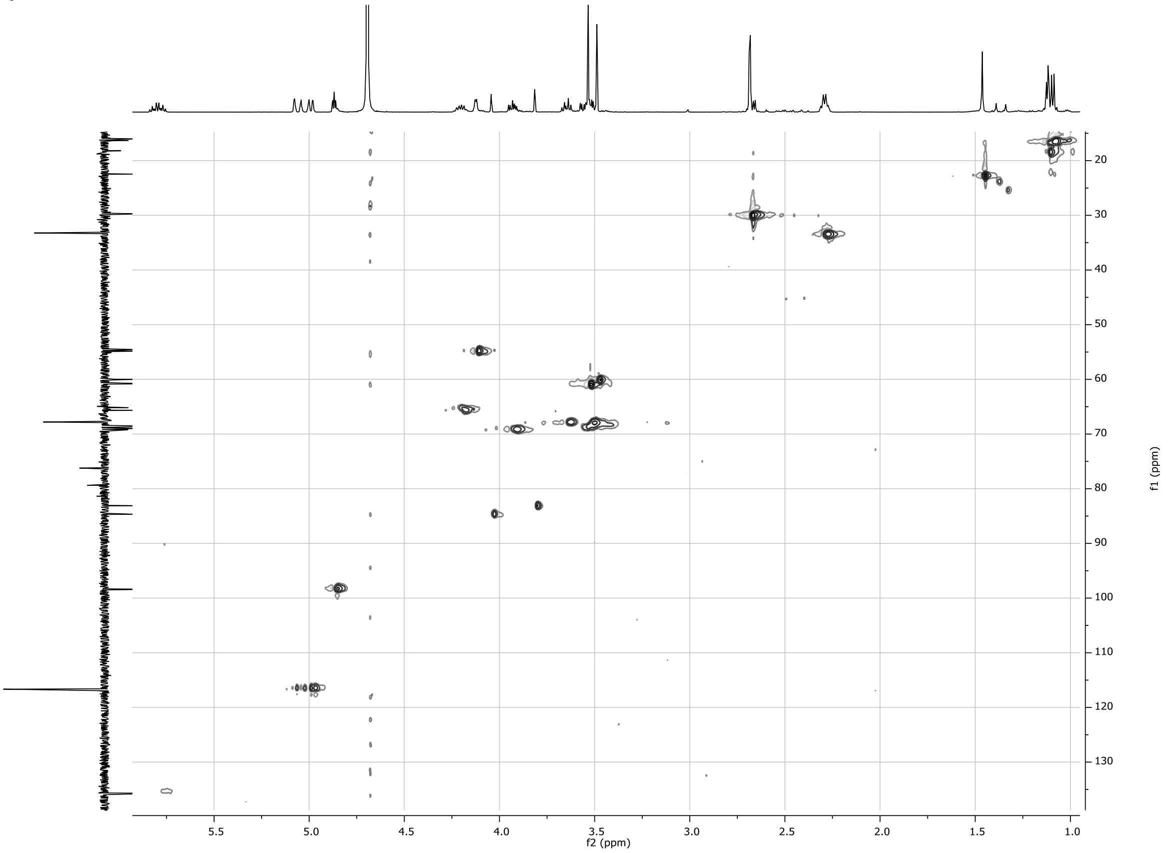

^{1}H NMR, 850 MHz, D_2O of **S44**

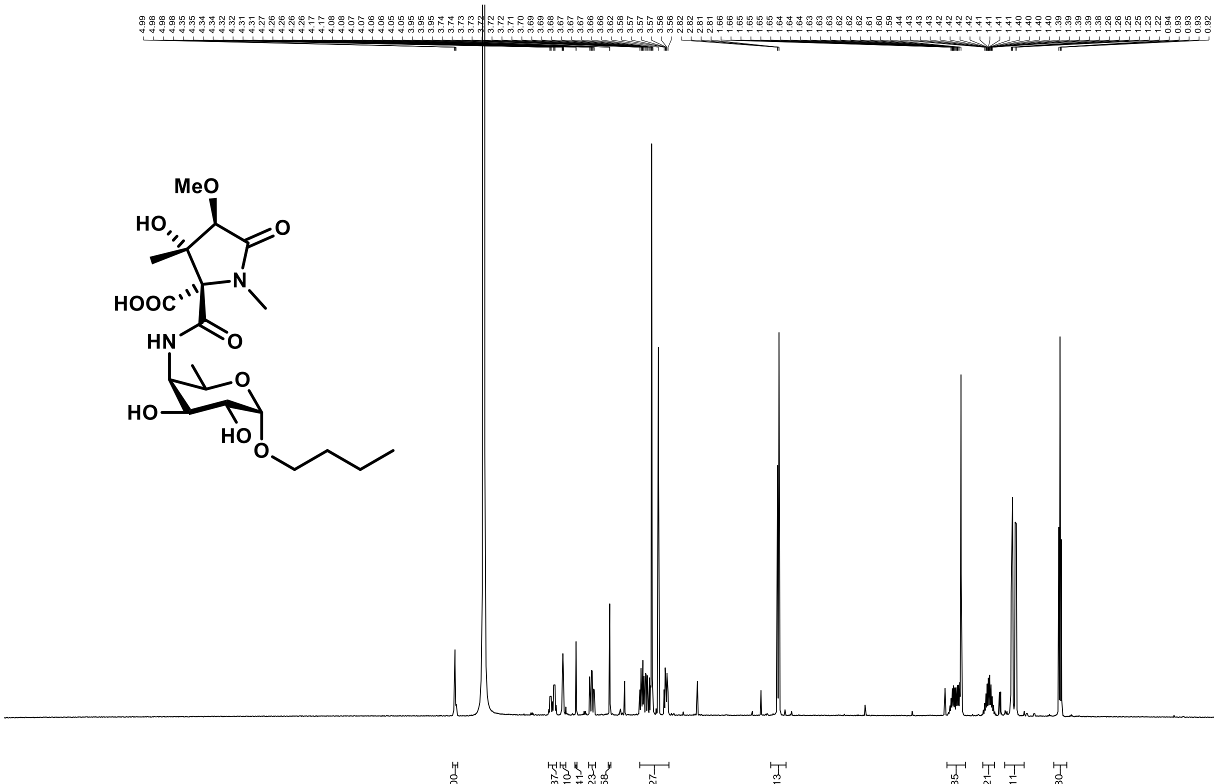

^{13}C NMR, 214 MHz, D_2O of **S44**

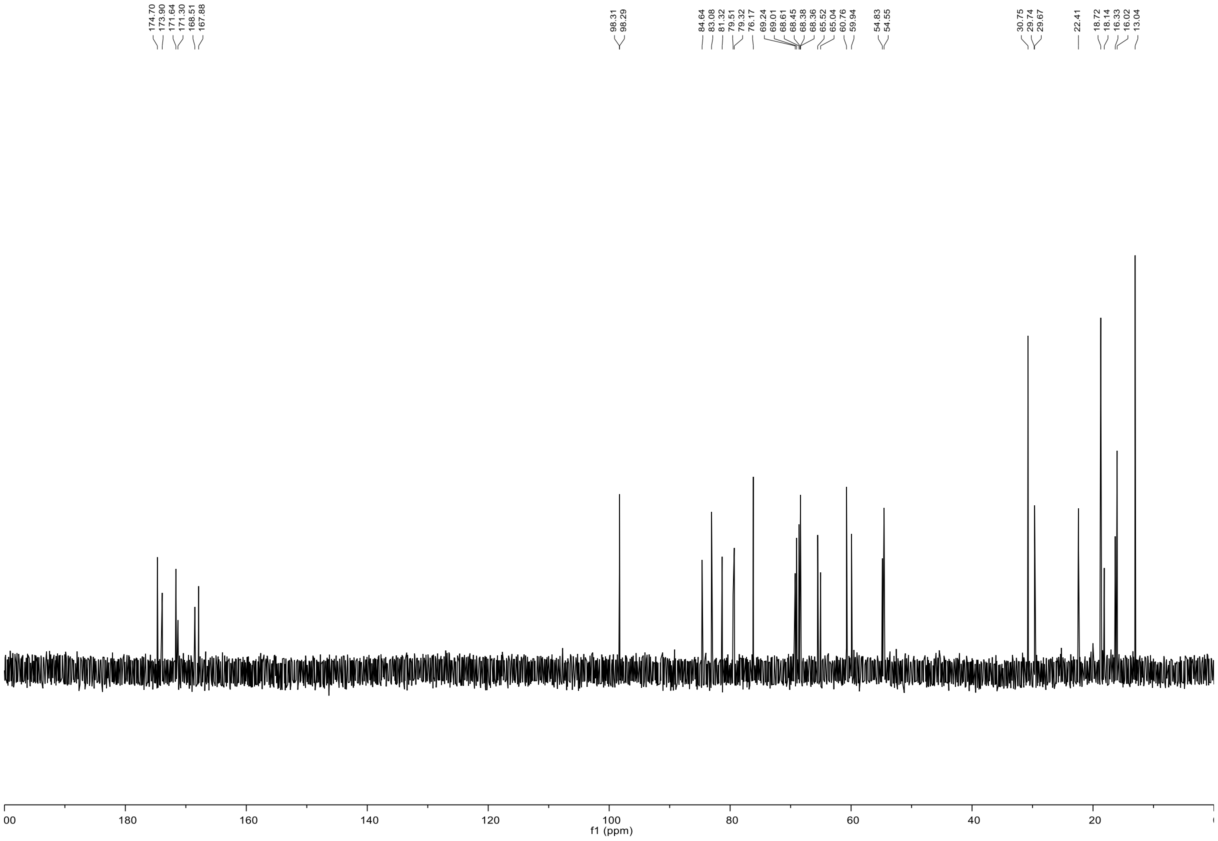

HH-COSY NMR, D_2O of **S44**

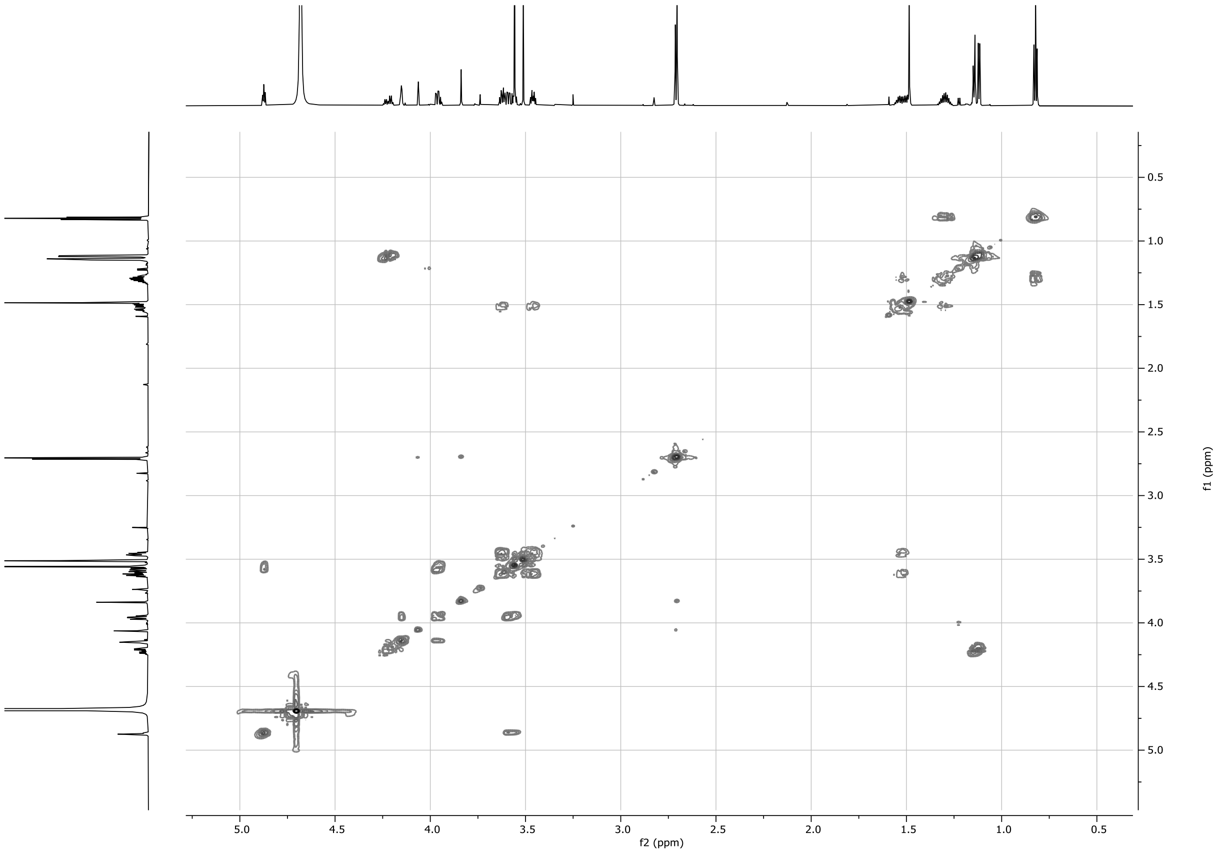

HSQC NMR, D_2O of **S44**

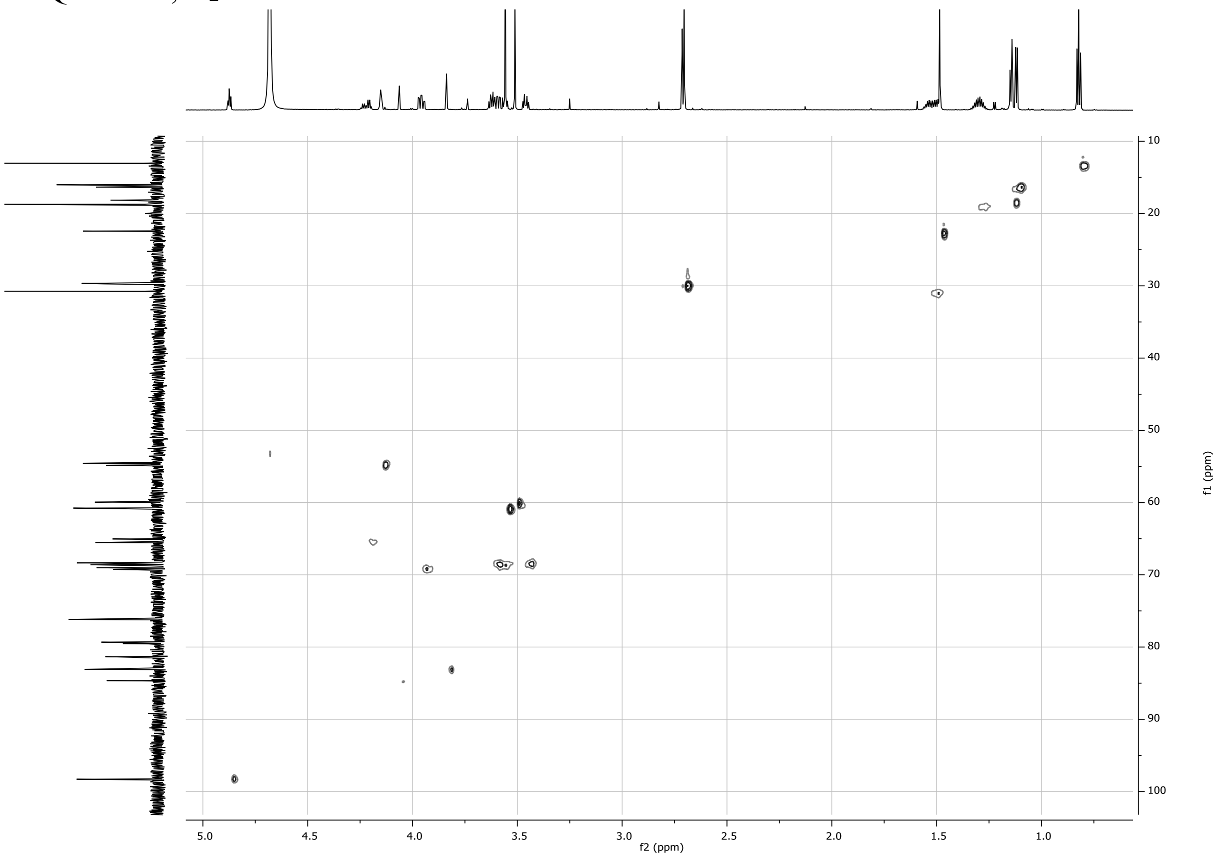

^1H NMR, 500 MHz, D_2O of **37**

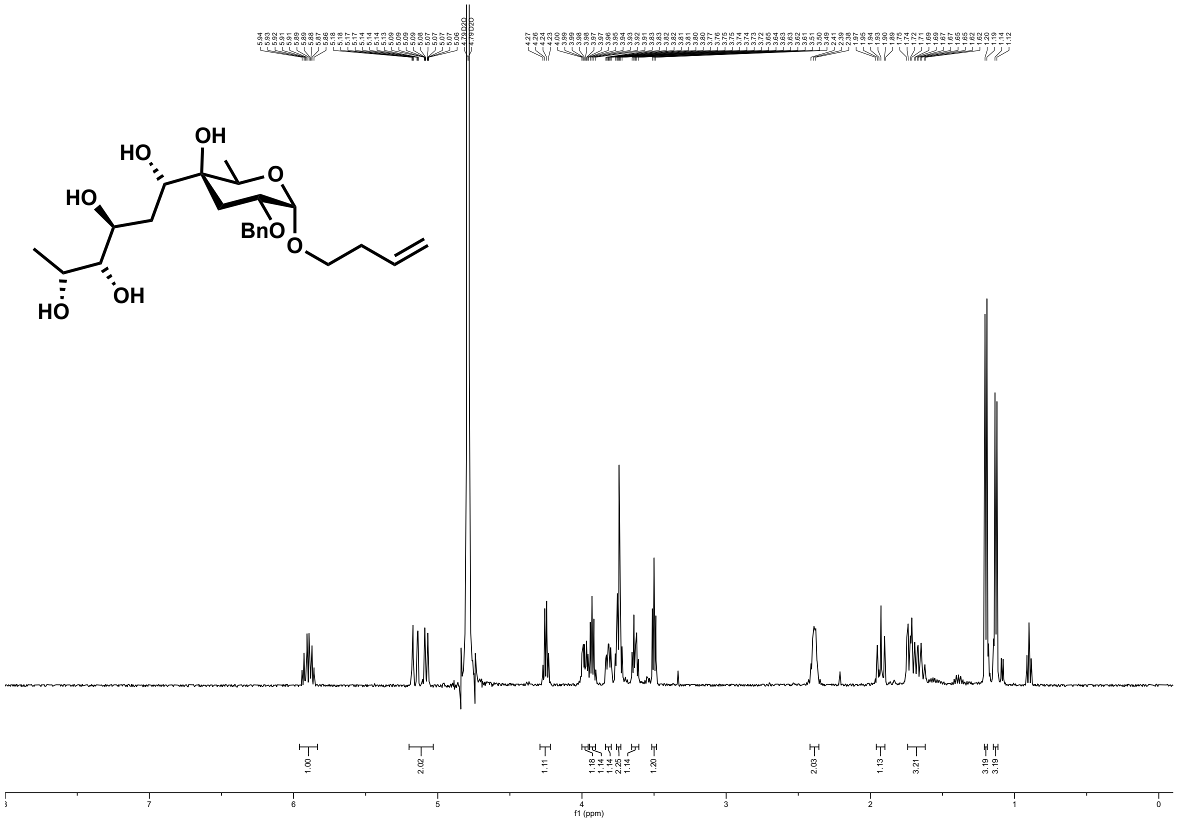

^{13}C NMR, 126 MHz, D_2O of **37**

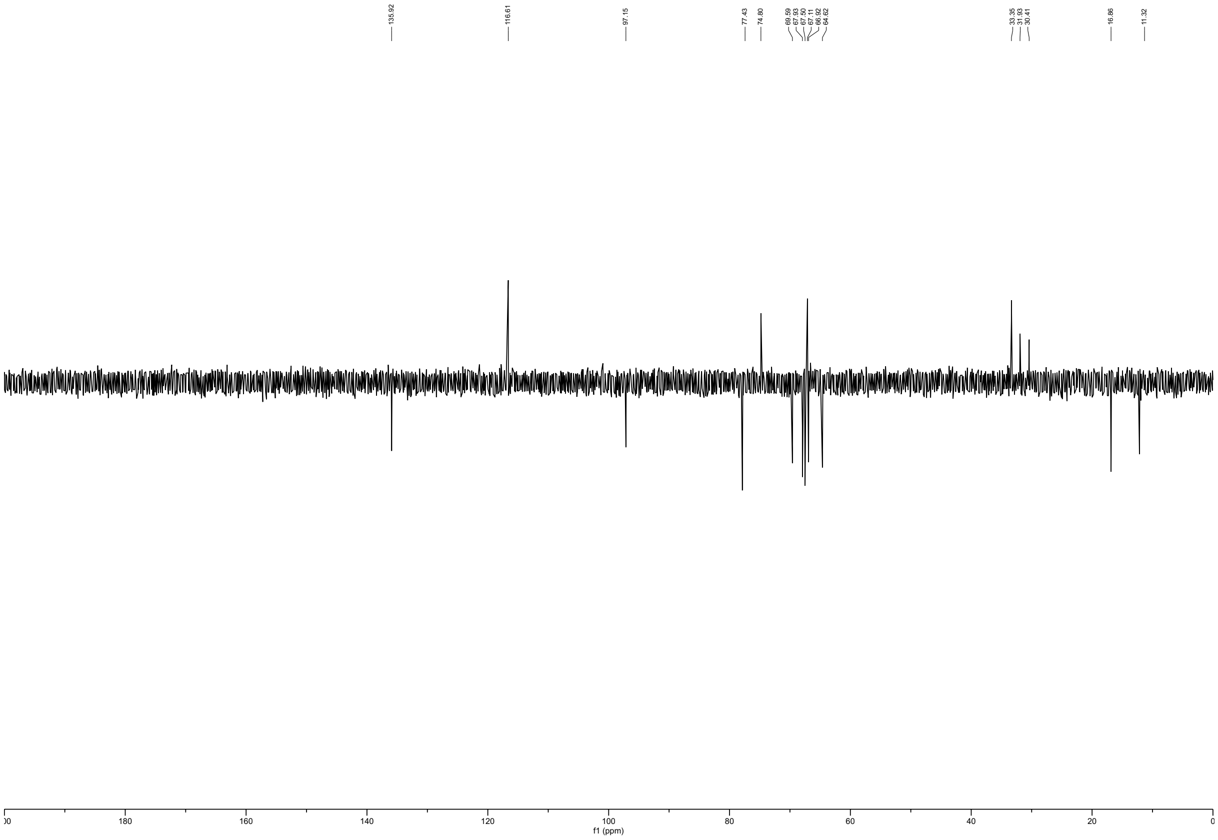

HH-COSY NMR, D_2O of **S37**

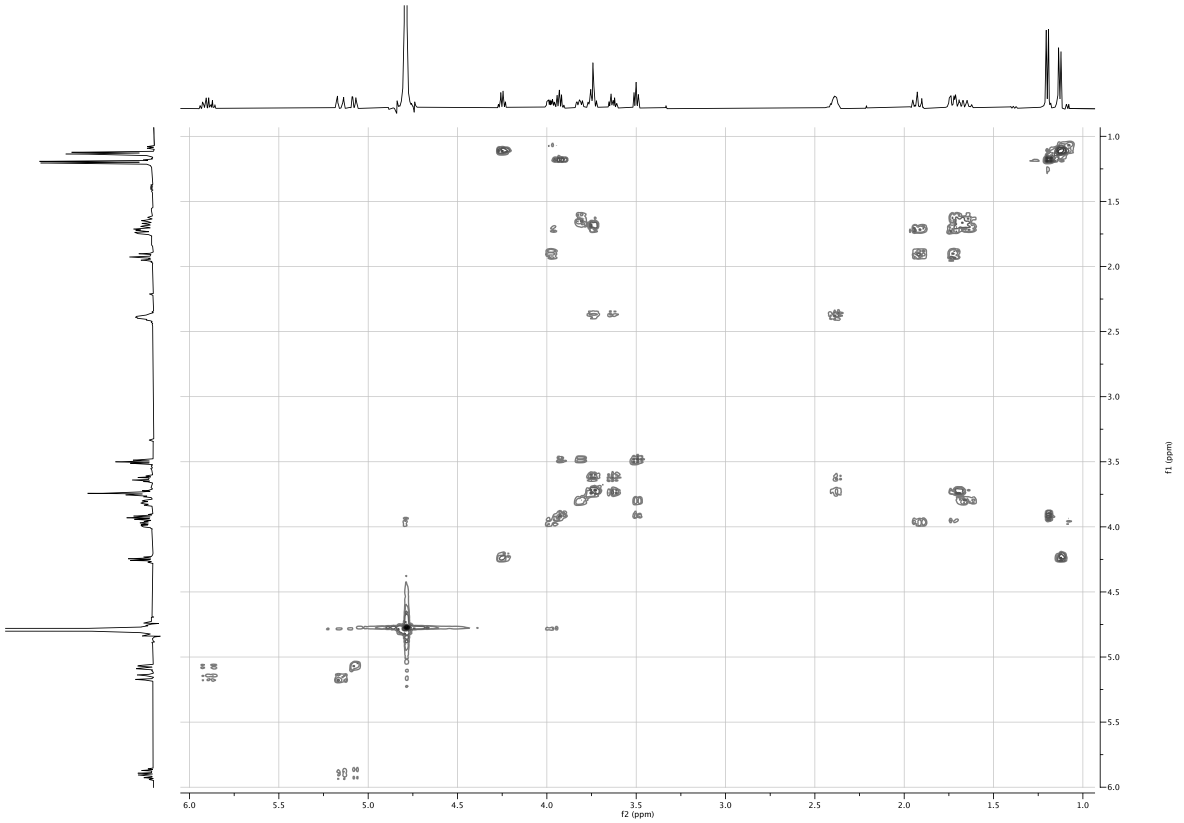

HSQC NMR, D_2O of **S37**

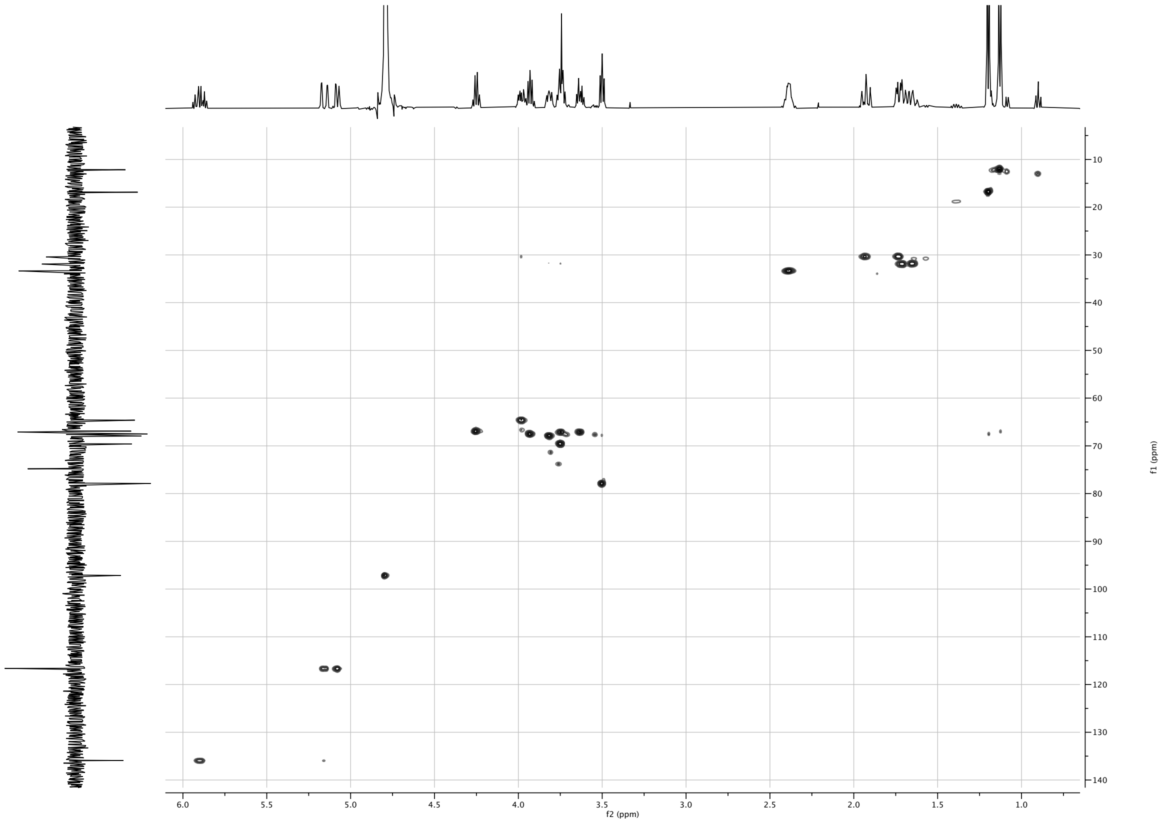

^{1}H NMR, 500 MHz, $CDCl_3$ of **S45**

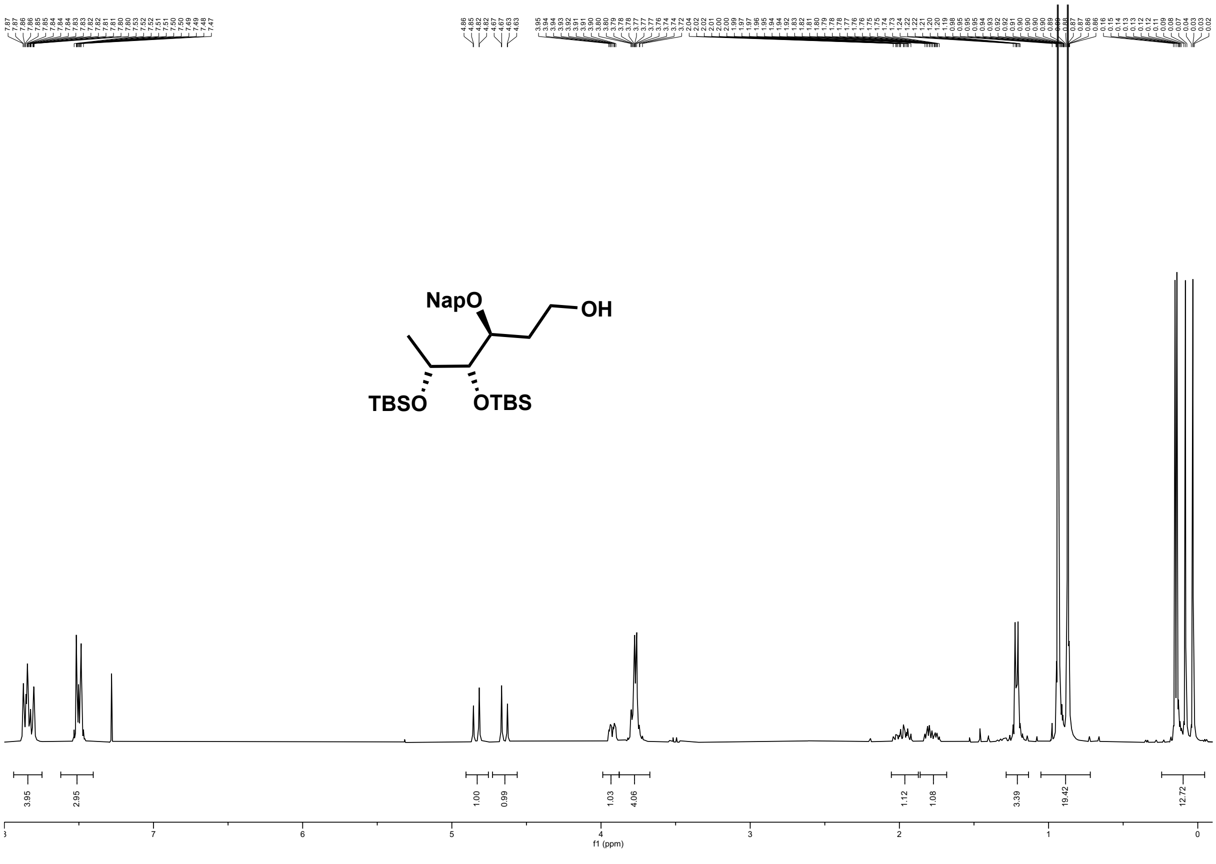

^{13}C NMR, 126 MHz, $CDCl_3$ of **S45**

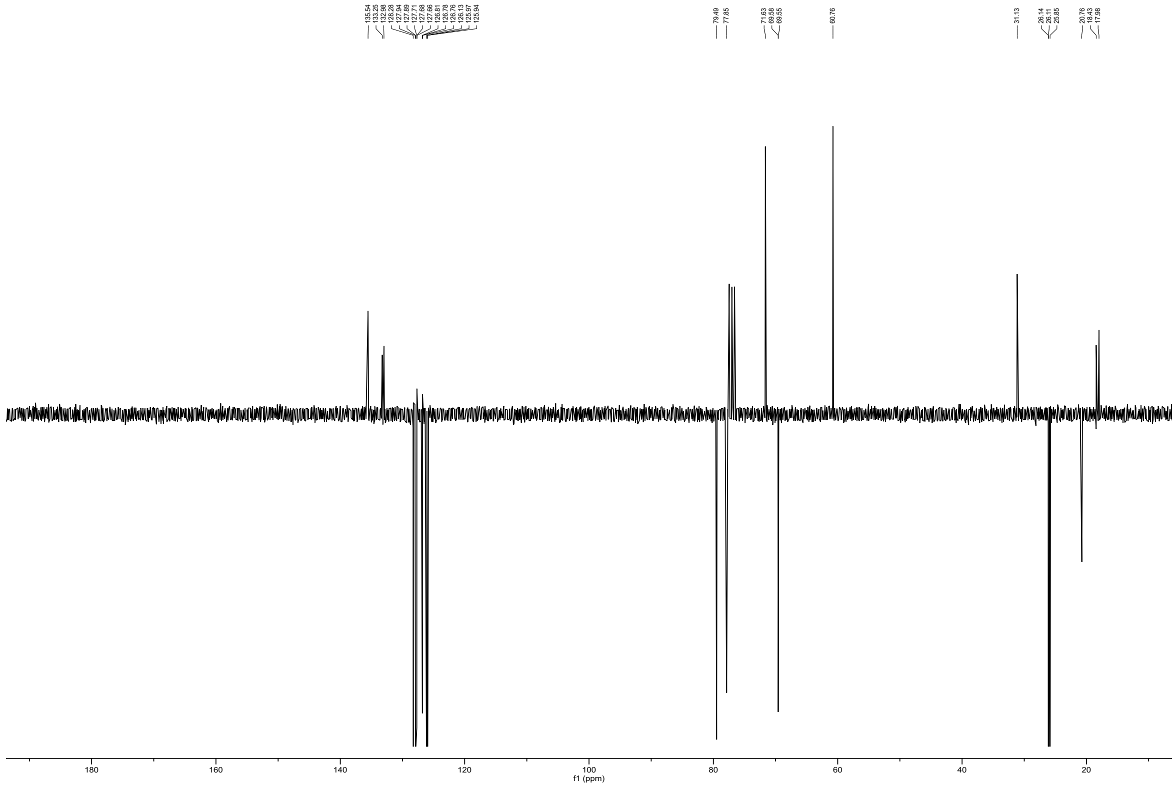

HH-COSY NMR, $CDCl_3$ of **S45**

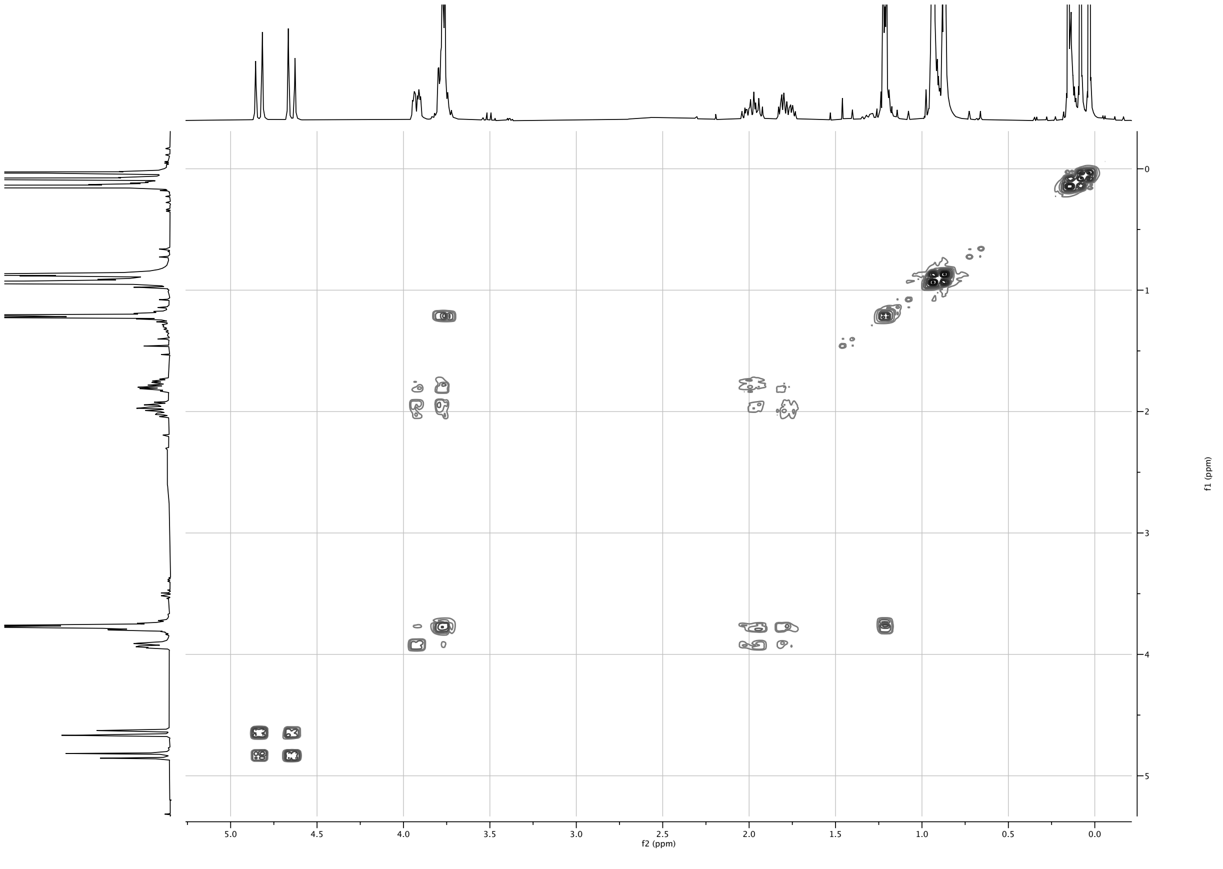

HSQC NMR, $CDCl_3$ of **S45**

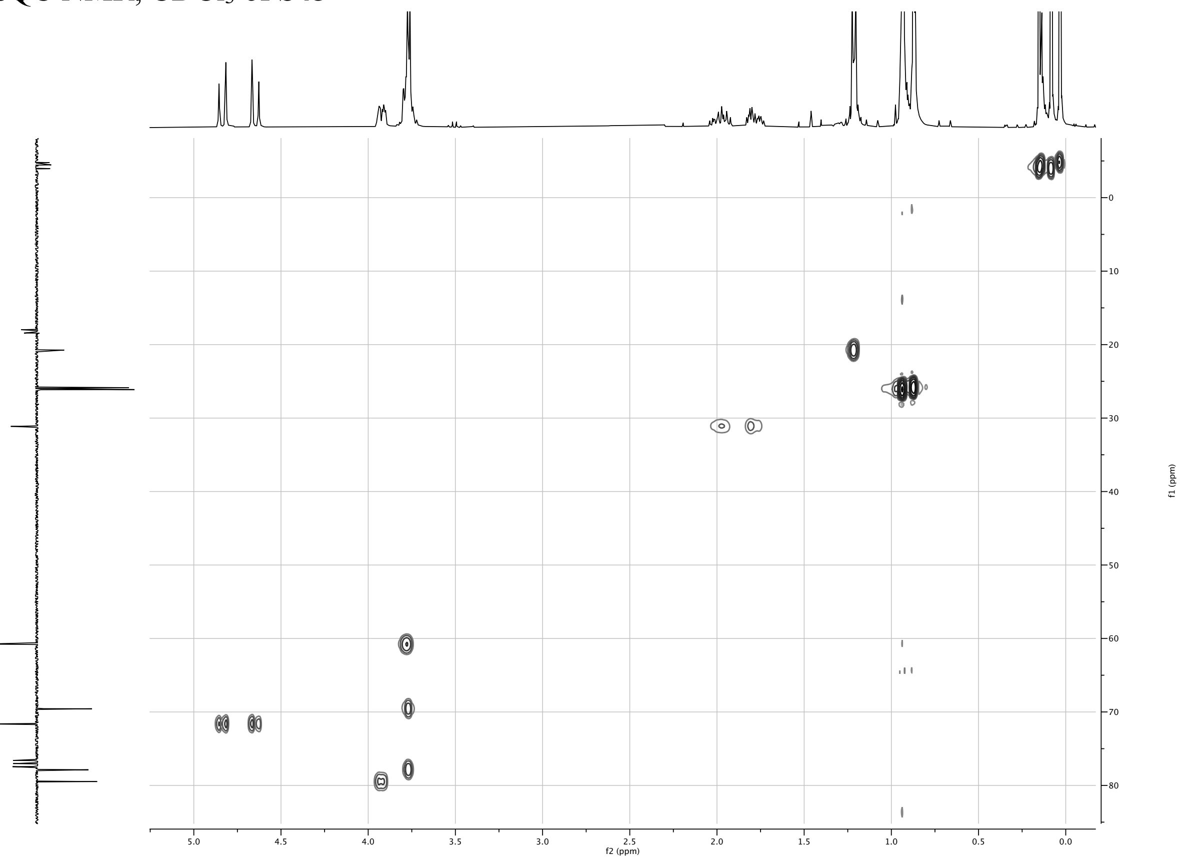

^{1}H NMR, 500 MHz, $CDCl_3$ of **S46**

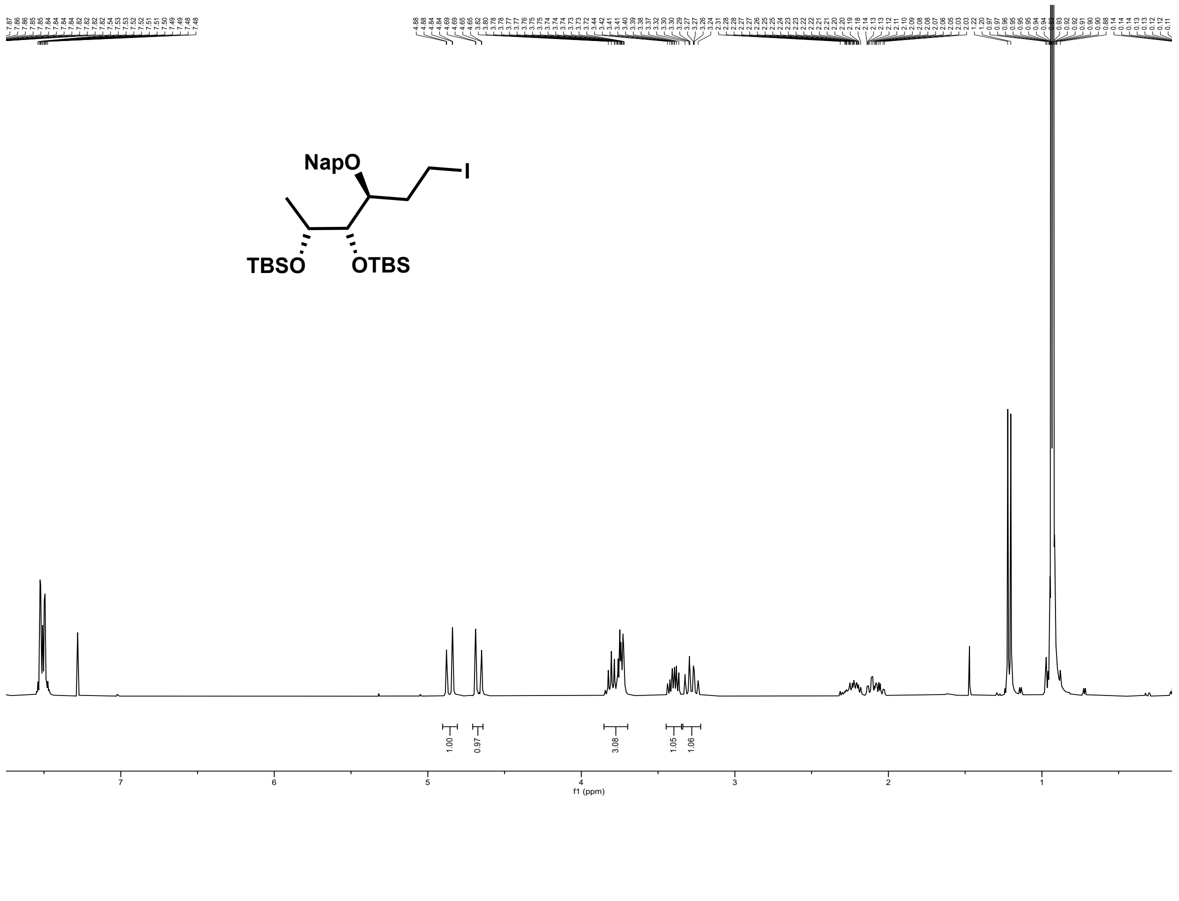

^{13}C NMR, 126 MHz, $CDCl_3$ of **S46**

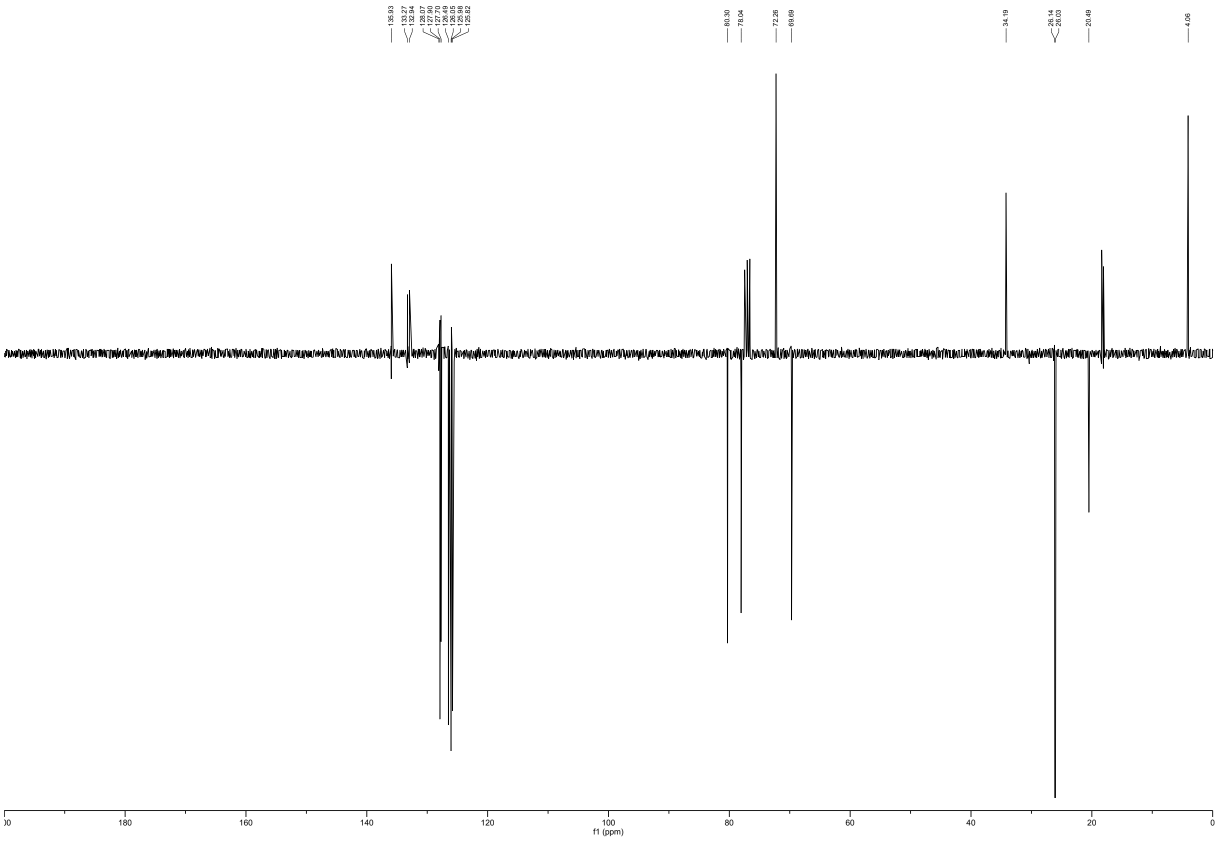

HH-COSY NMR, $CDCl_3$ of **S46**

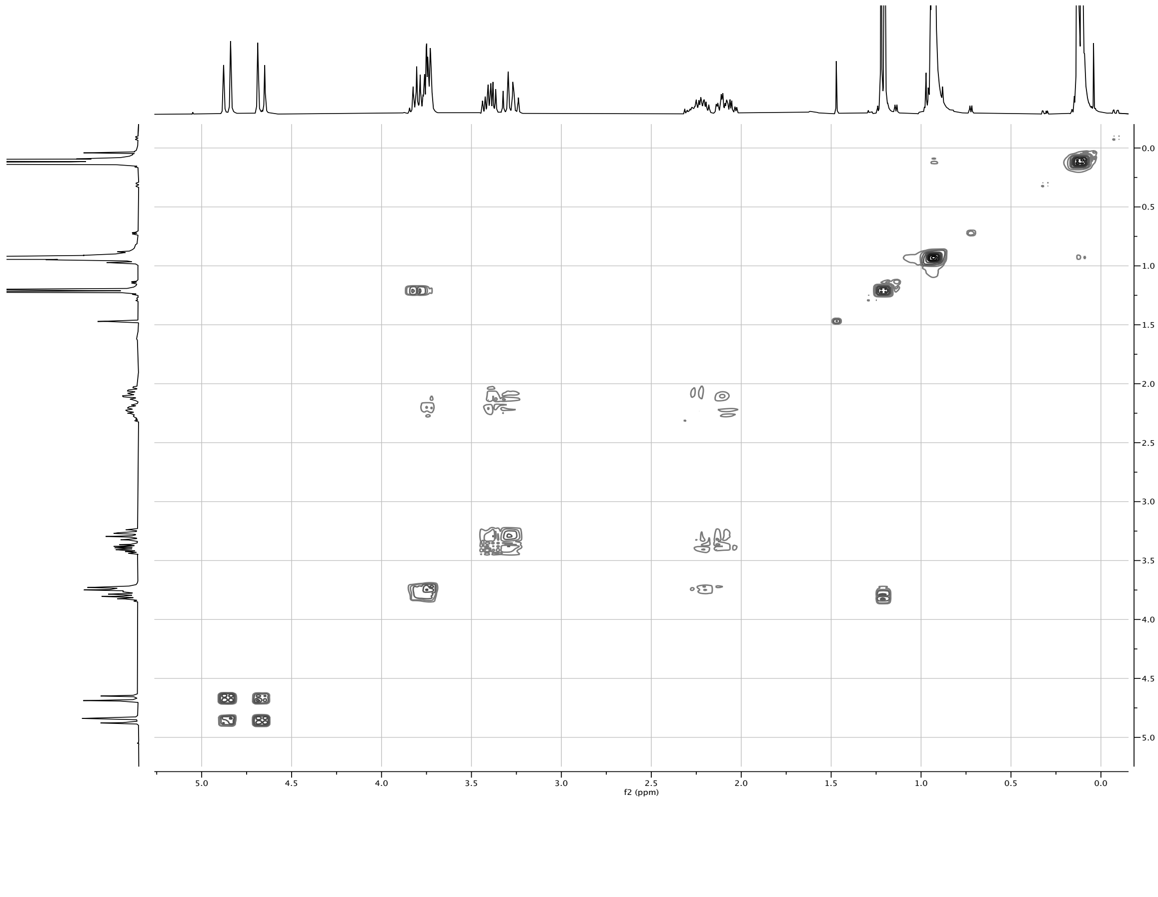

HSQC NMR, $CDCl_3$ of **S46**

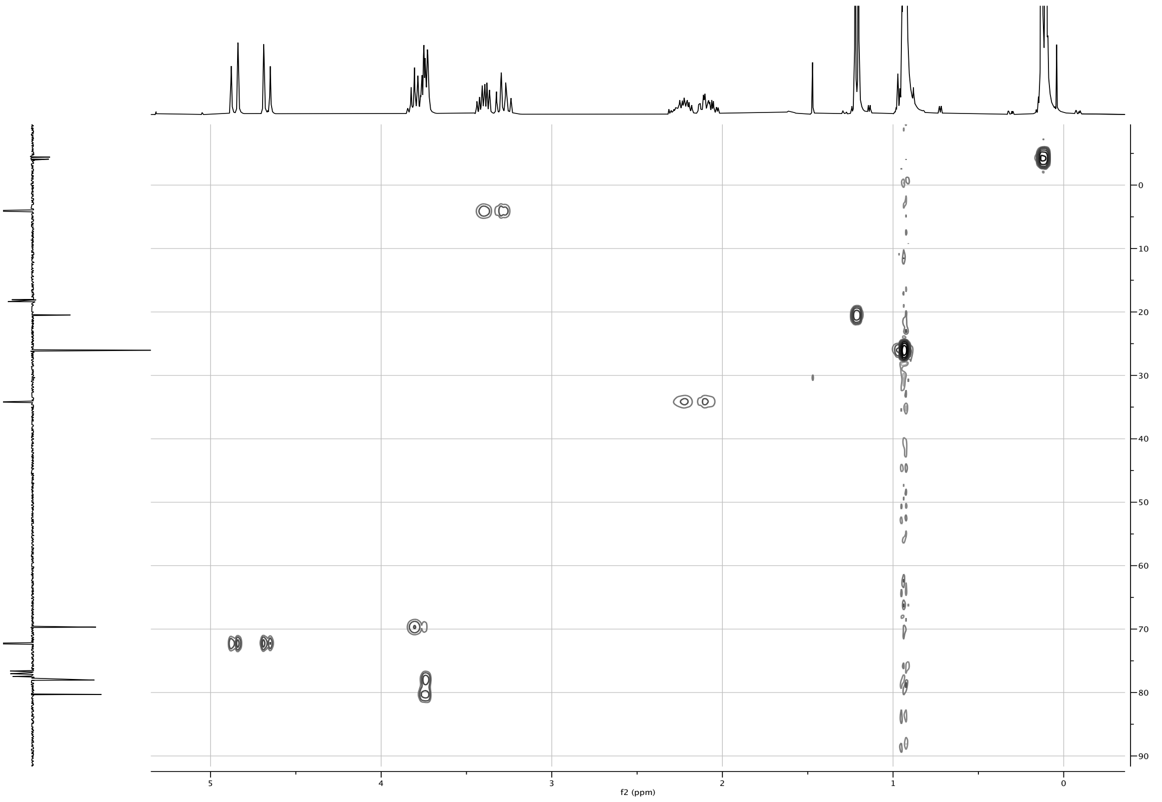

^{1}H NMR, 500 MHz, $CDCl_3$ of **S47**

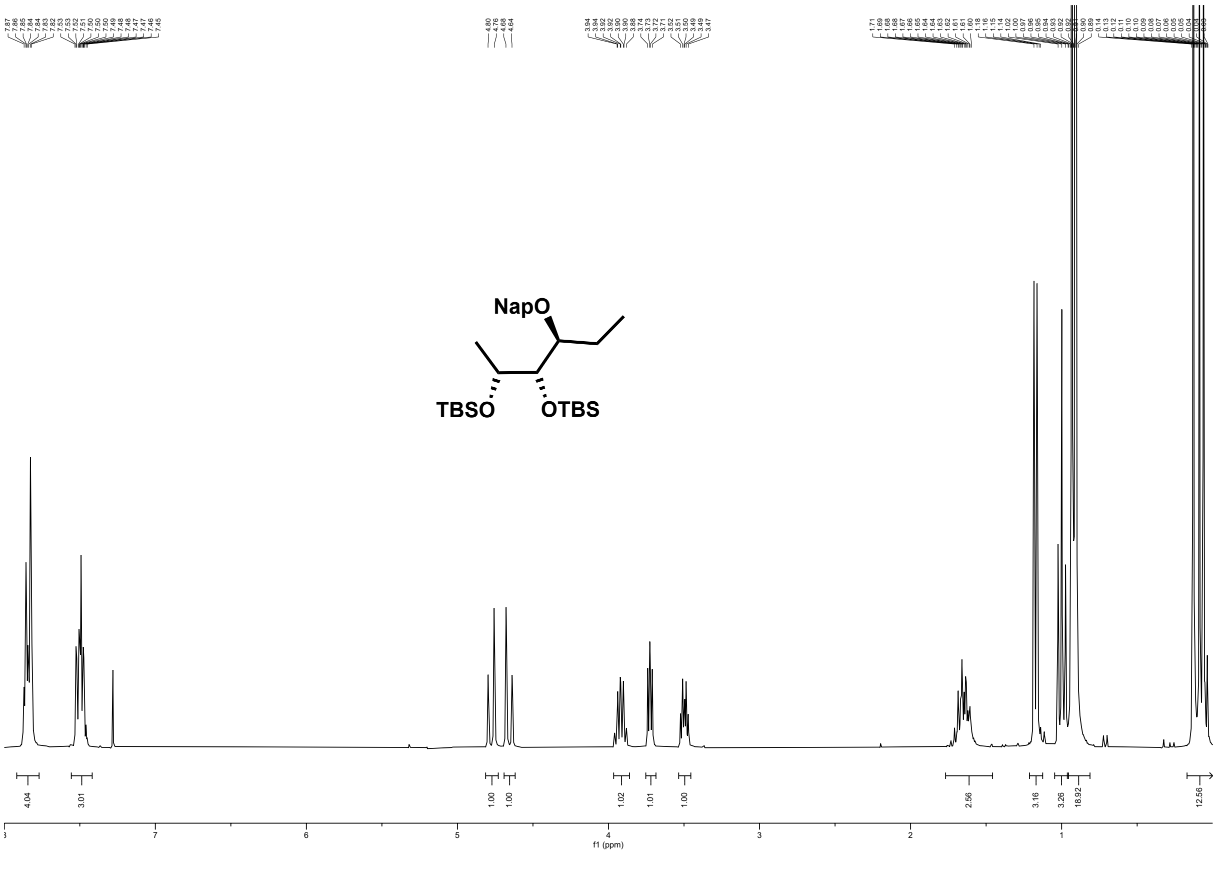

^{13}C NMR, 126 MHz, $CDCl_3$ of **S47**

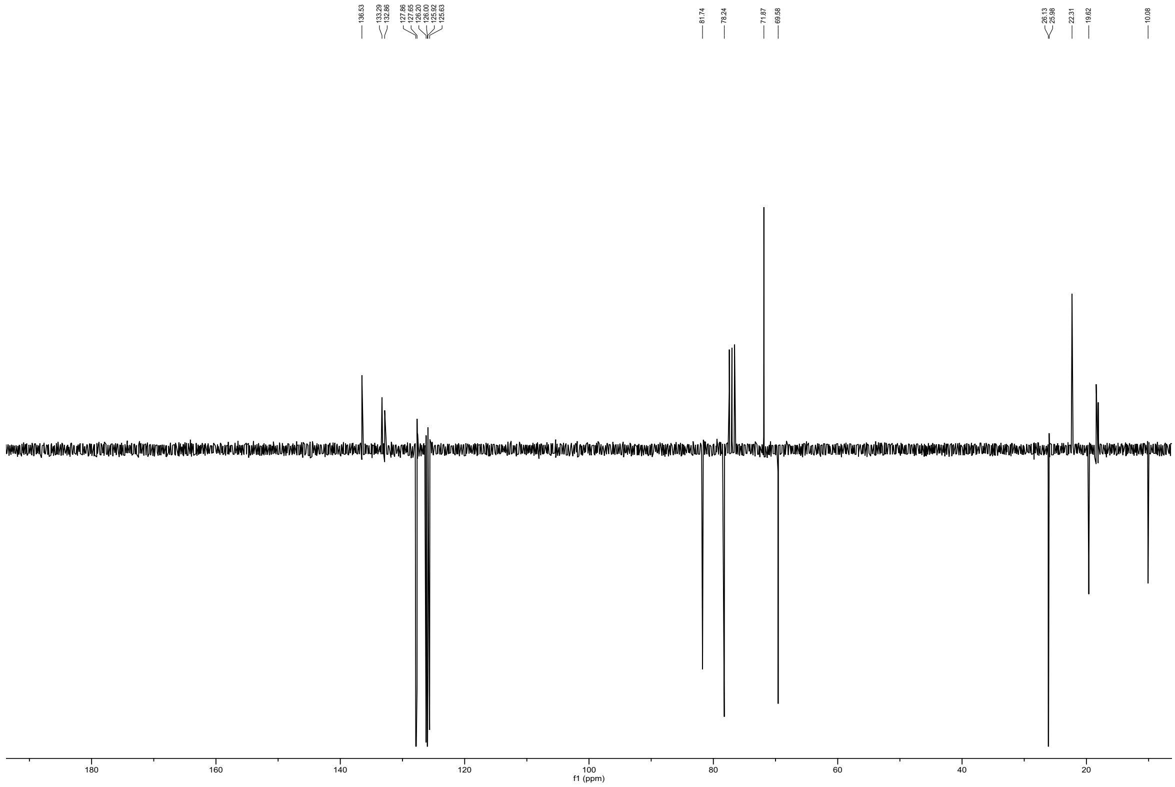

HH-COSY NMR, $CDCl_3$ of **S47**

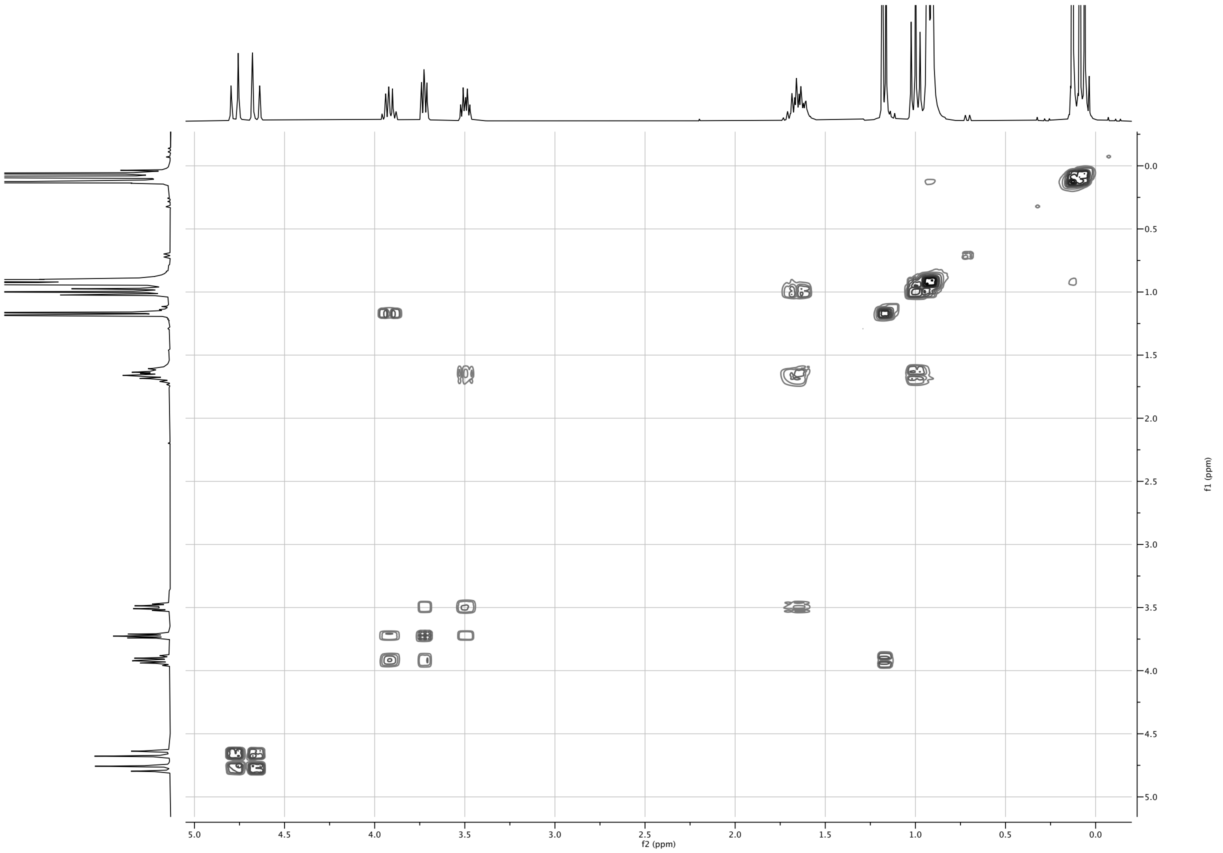

HSQC NMR, $CDCl_3$ of **S47**

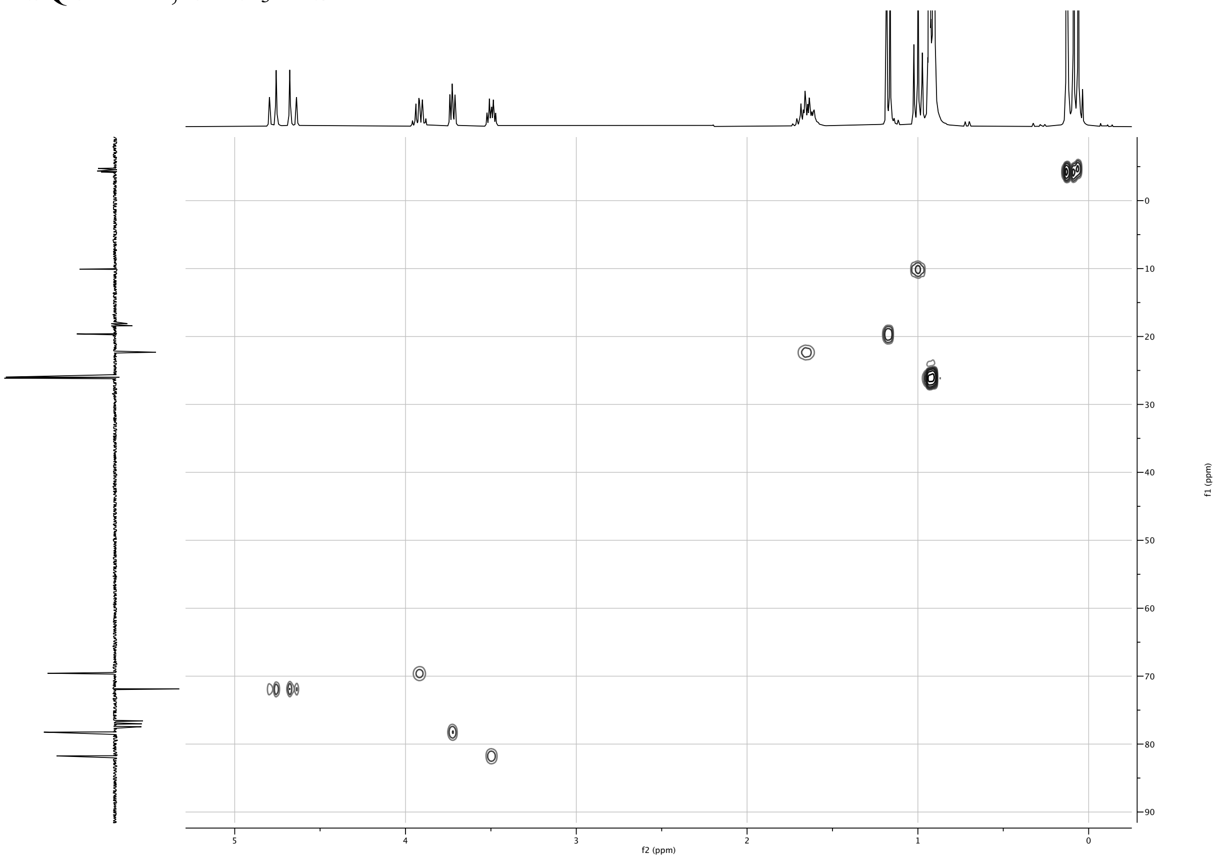

^{1}H NMR, 500 MHz, DCl_3 of **S48**

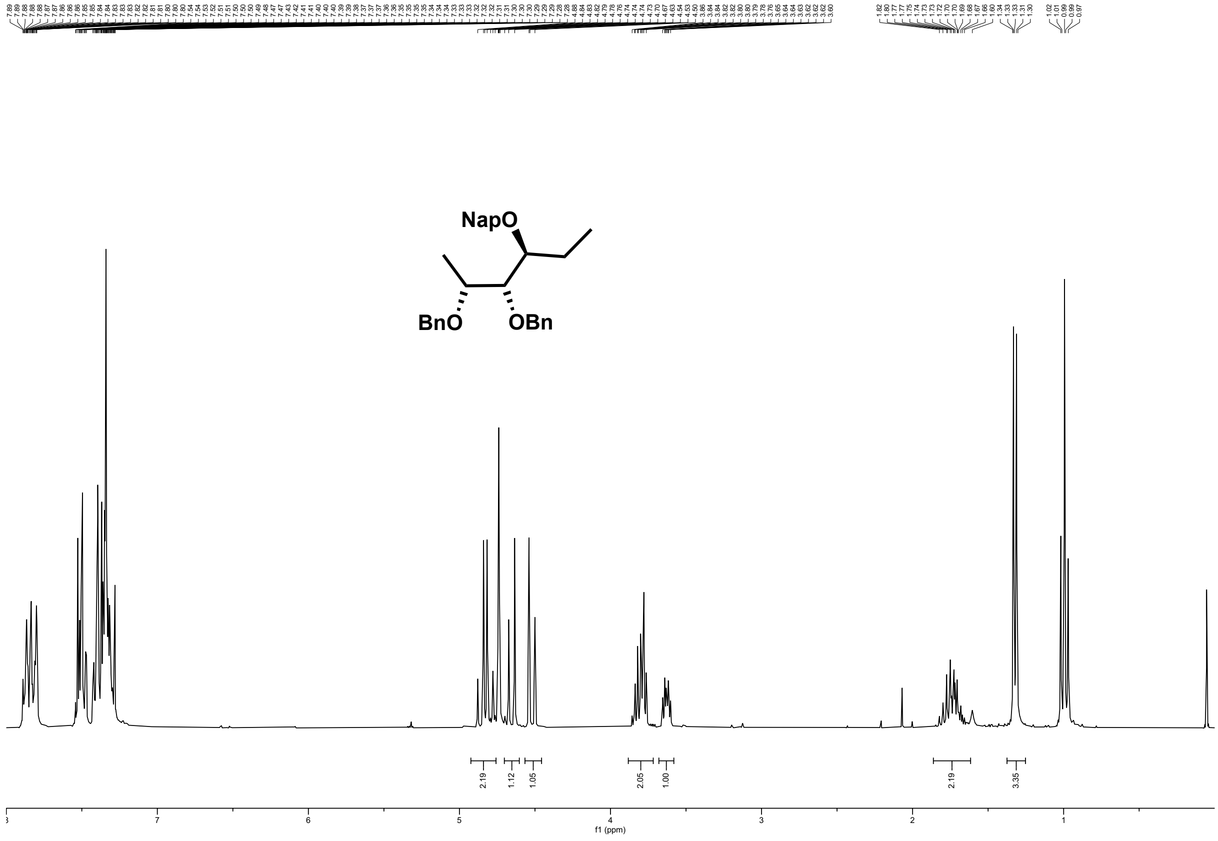

^{13}C NMR, 126 MHz, $CDCl_3$ of **S48**

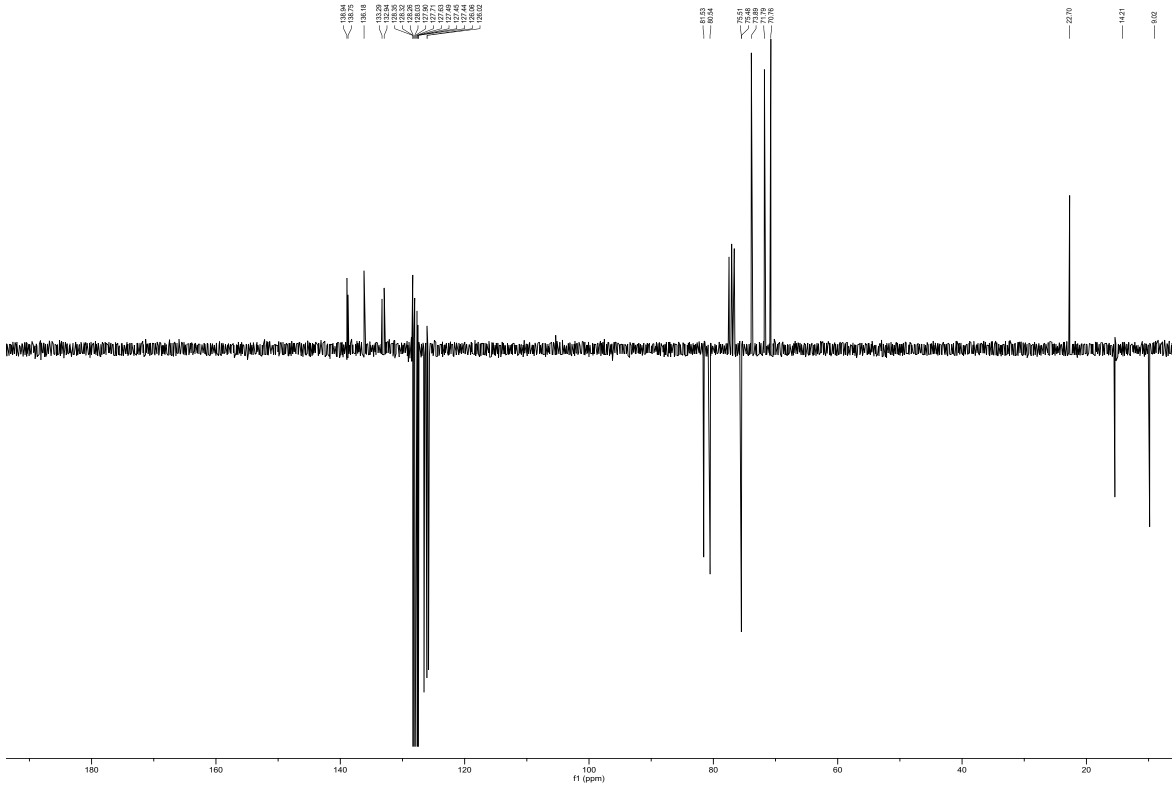

HH-COSY NMR, $CDCl_3$ of **S48**

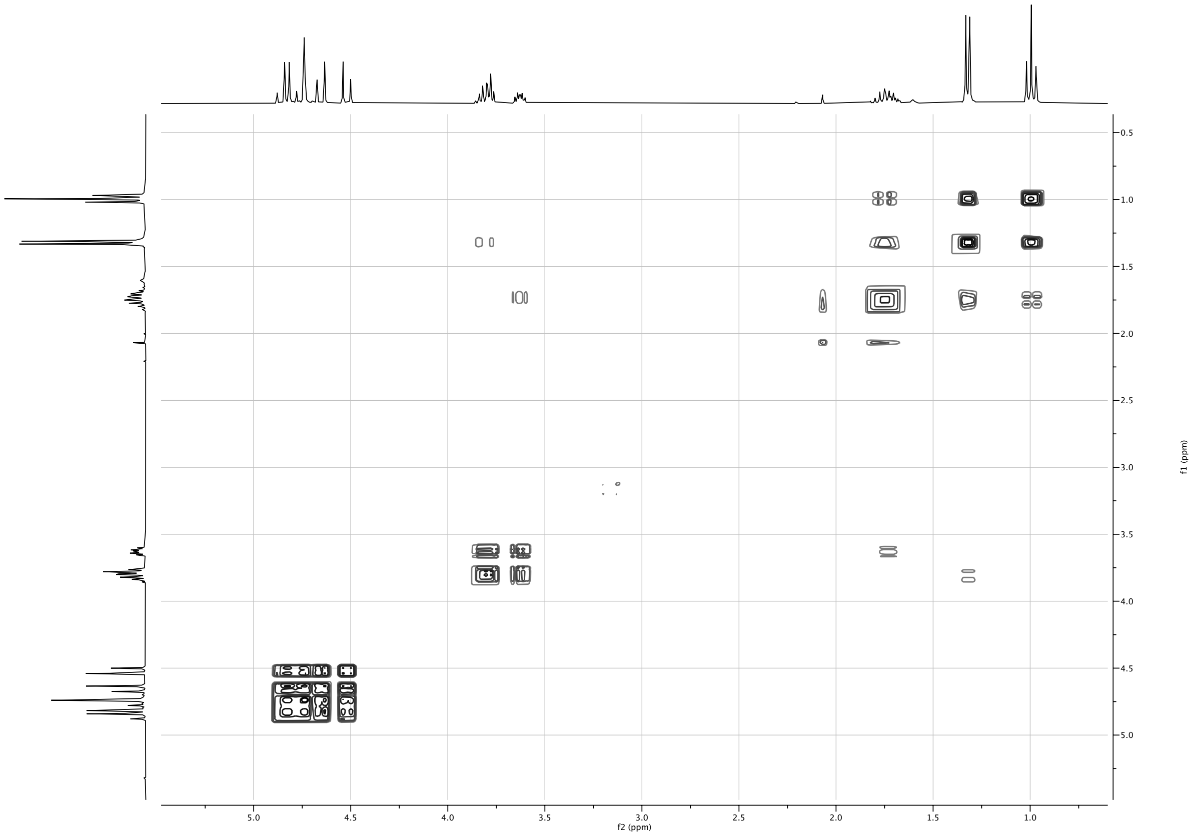

HSQC NMR, $CDCl_3$ of **S48**

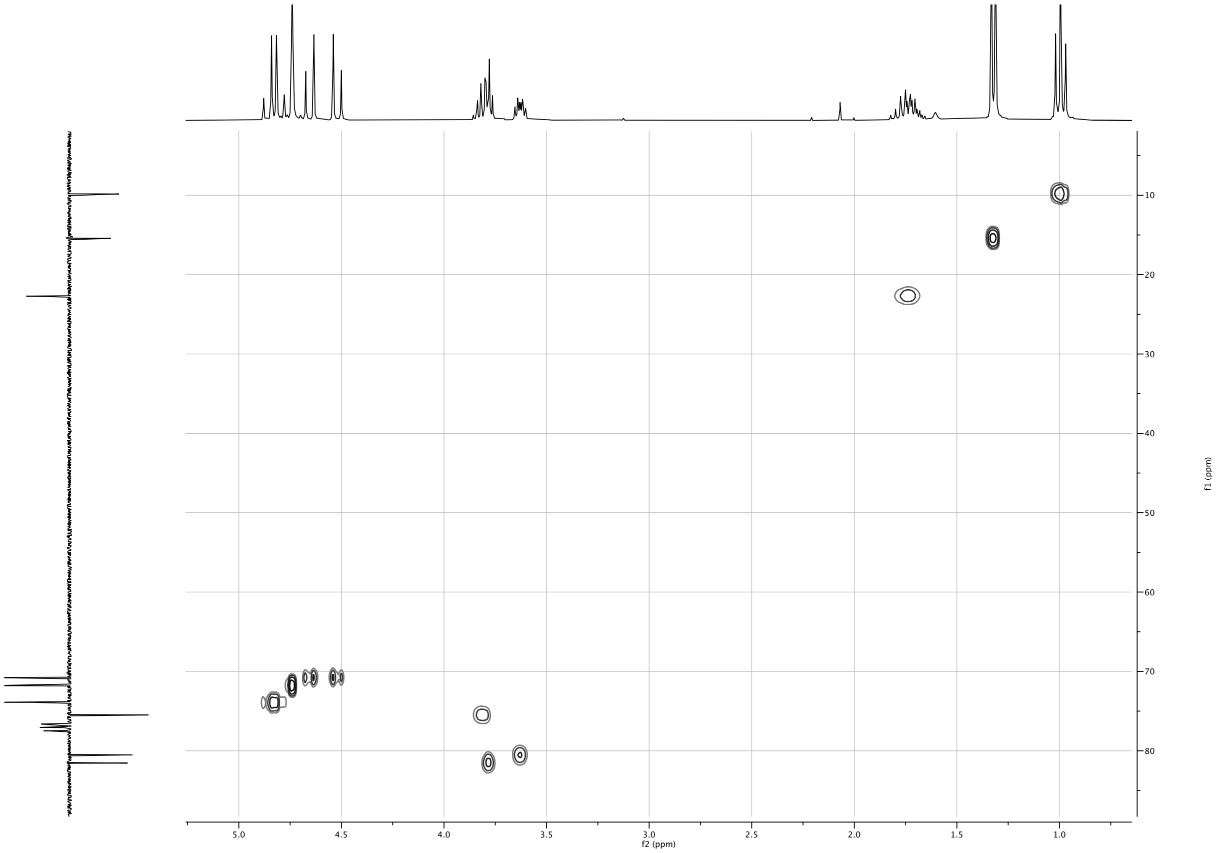

^{1}H NMR, 500 MHz, $CDCl_3$ of **28**

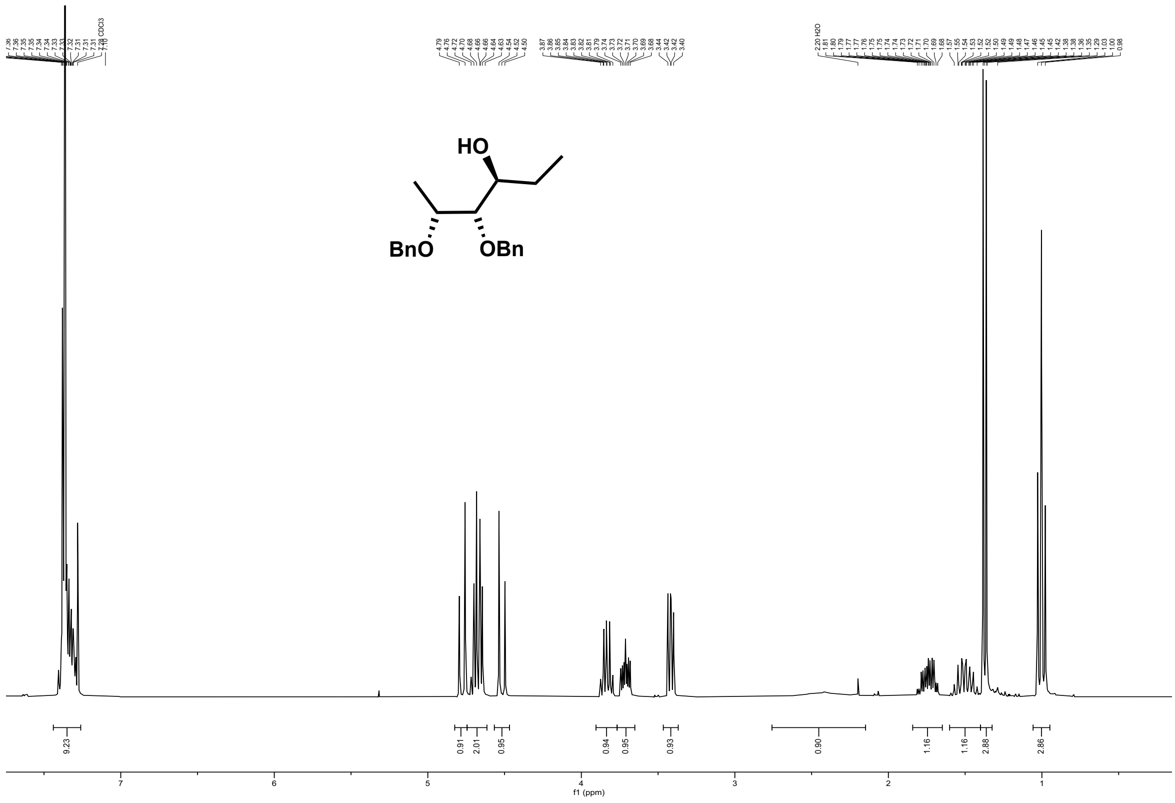

^{13}C NMR, 126 MHz, $CDCl_3$ of **28**

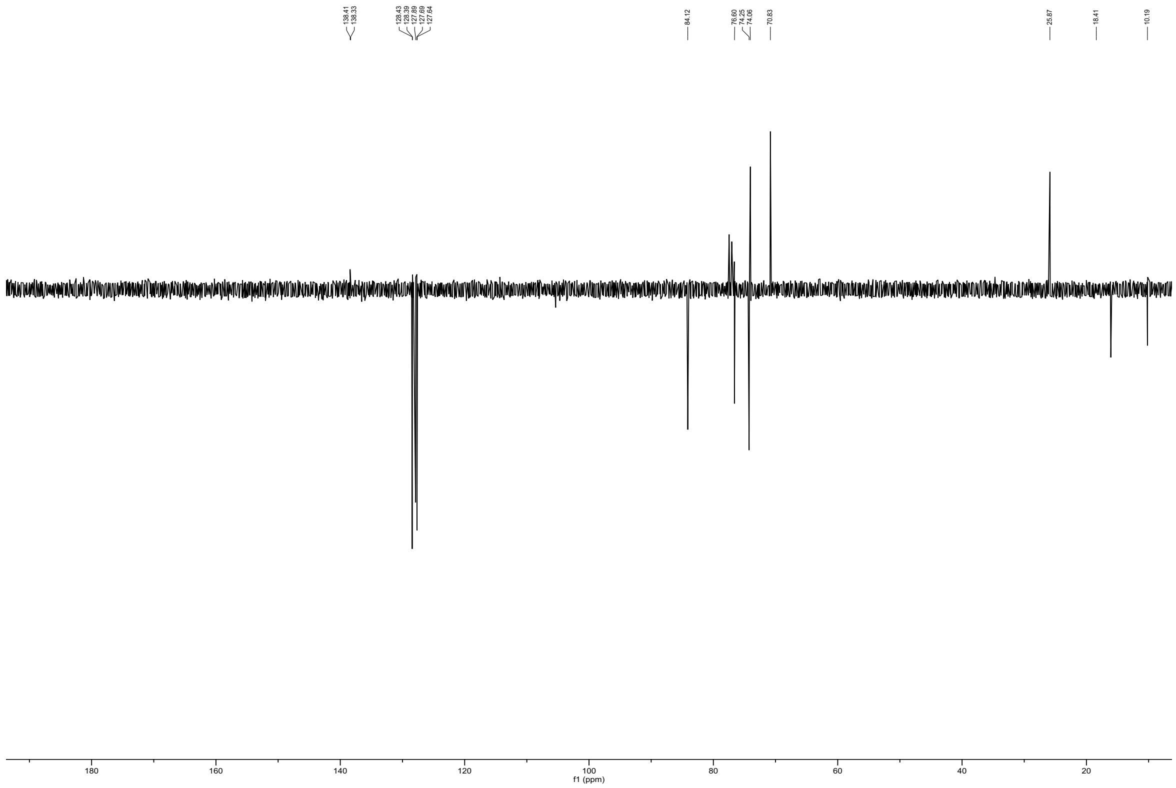

HH-COSY NMR, $CDCl_3$ of **28**

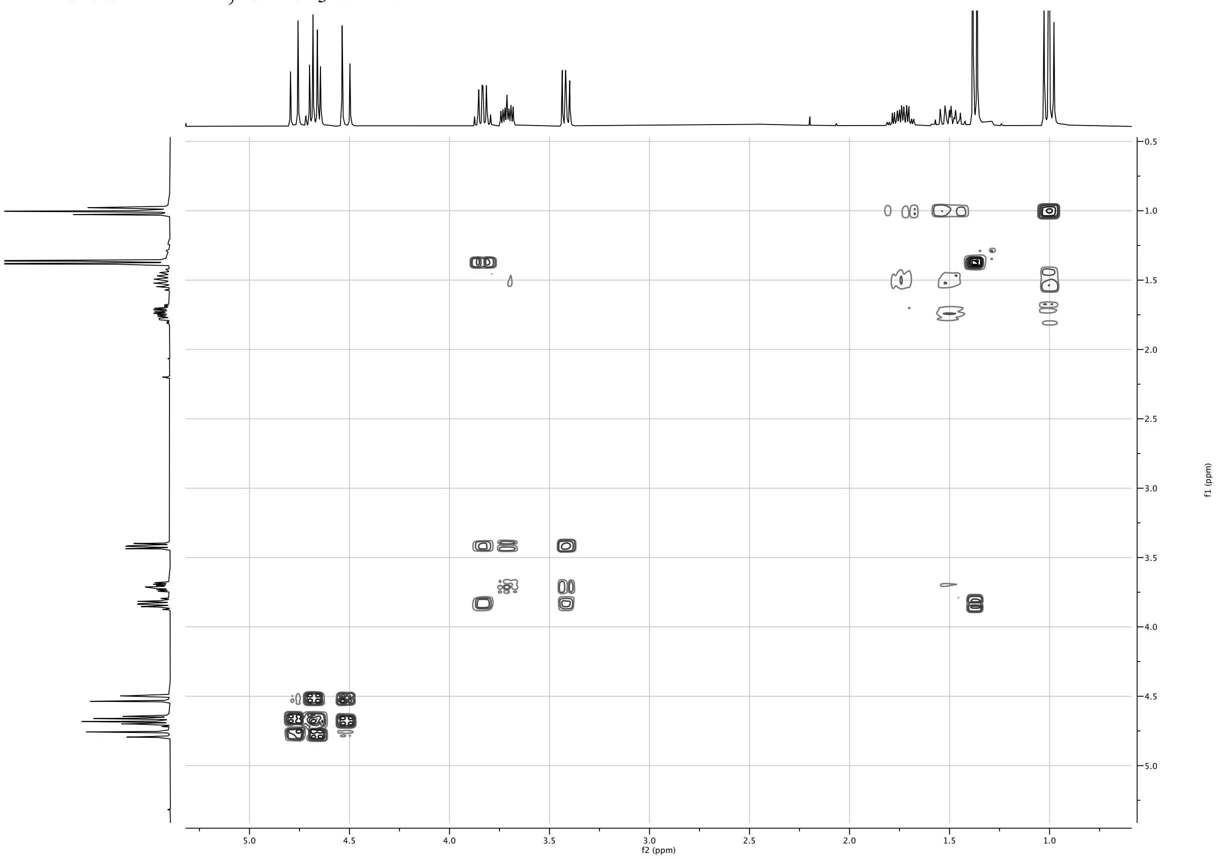

HSQC NMR, $CDCl_3$ of **28**

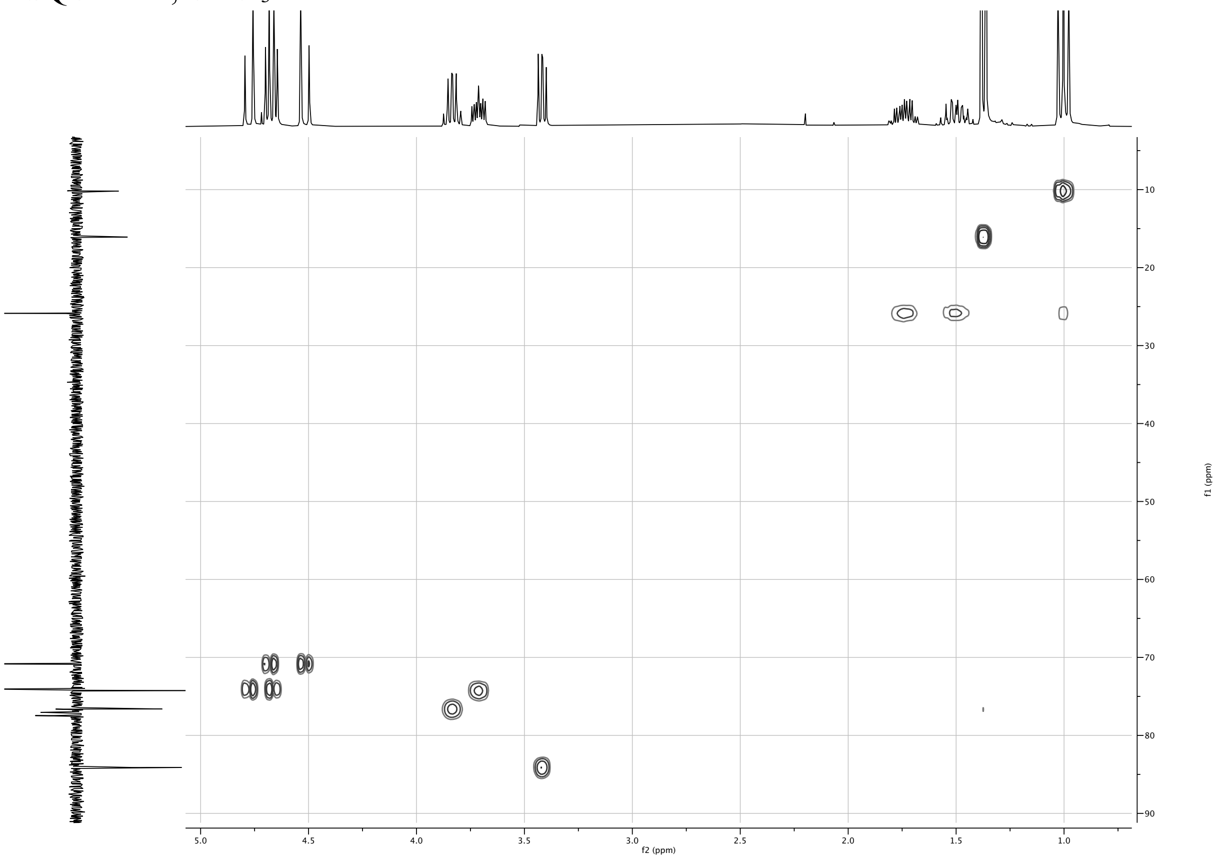

^{1}H NMR, 500 MHz, $CDCl_3$ of **S49**

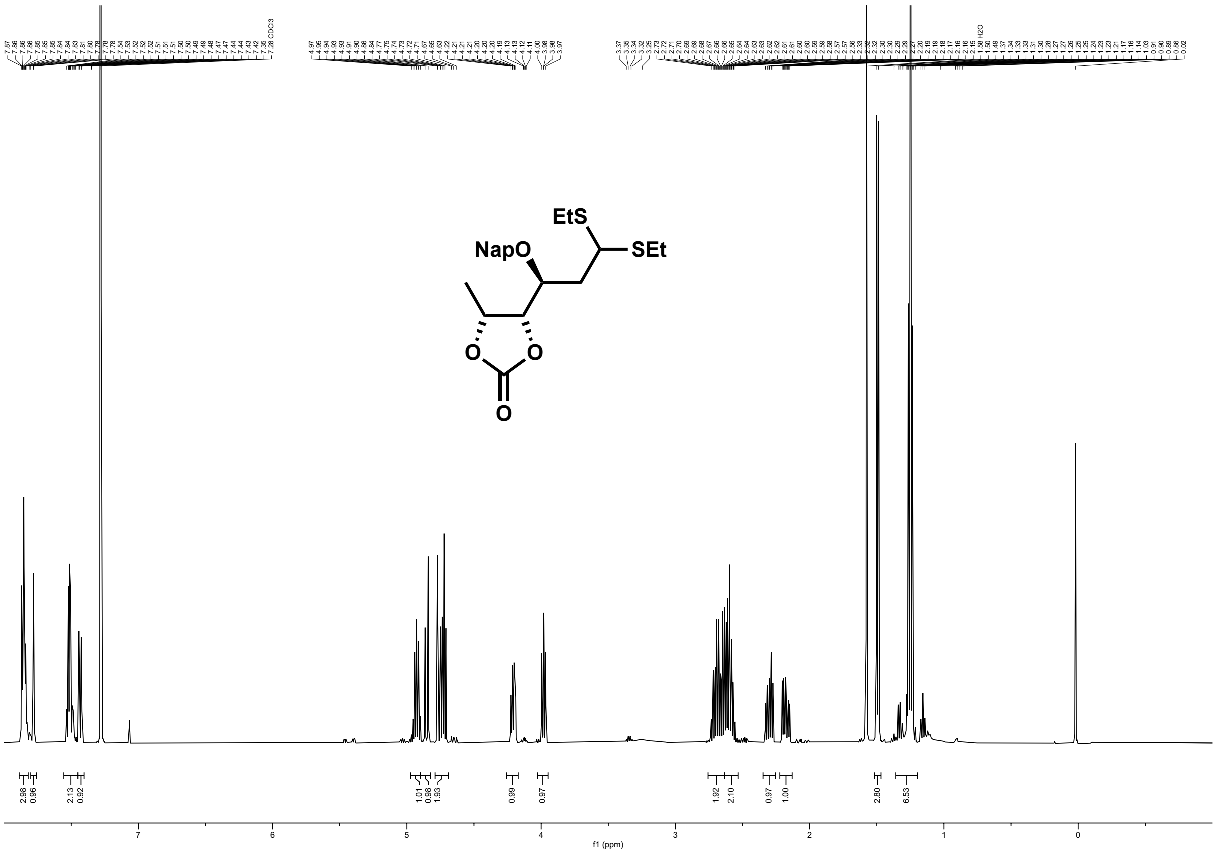

^{13}C NMR, 126 MHz, $CDCl_3$ of **S49**

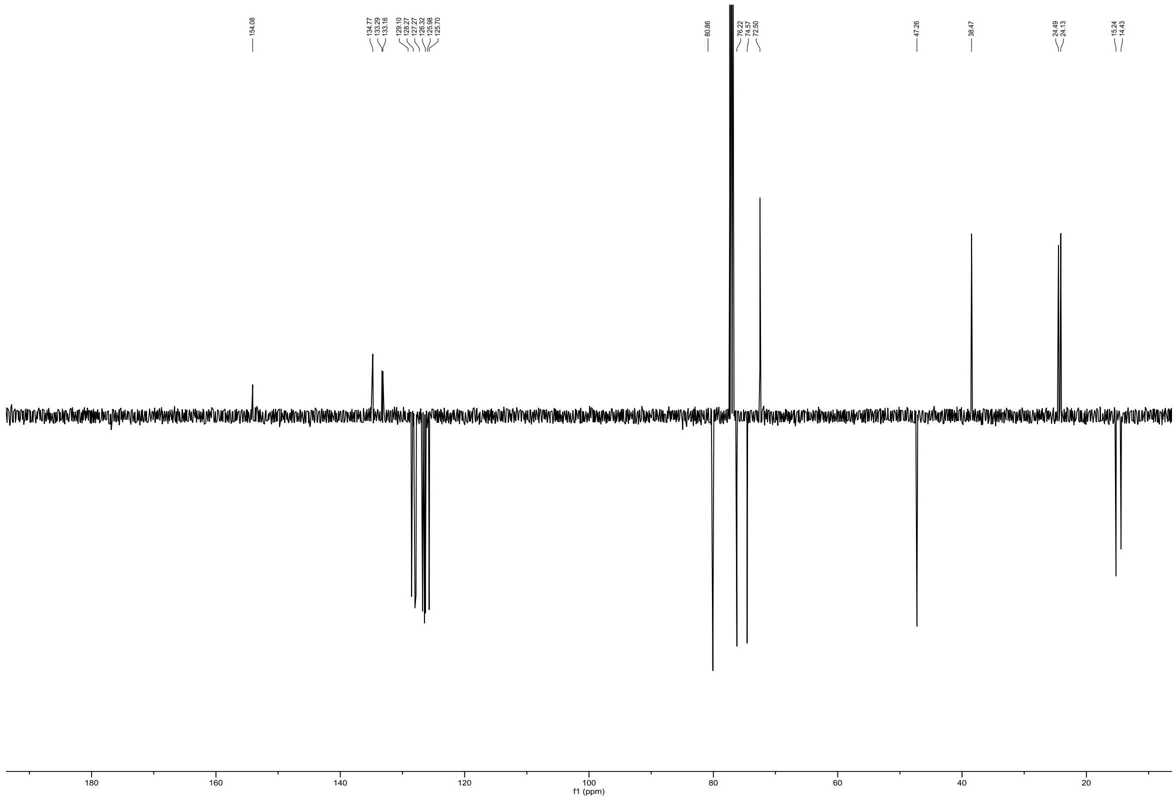

HH-COSY NMR, $CDCl_3$ of **S49**

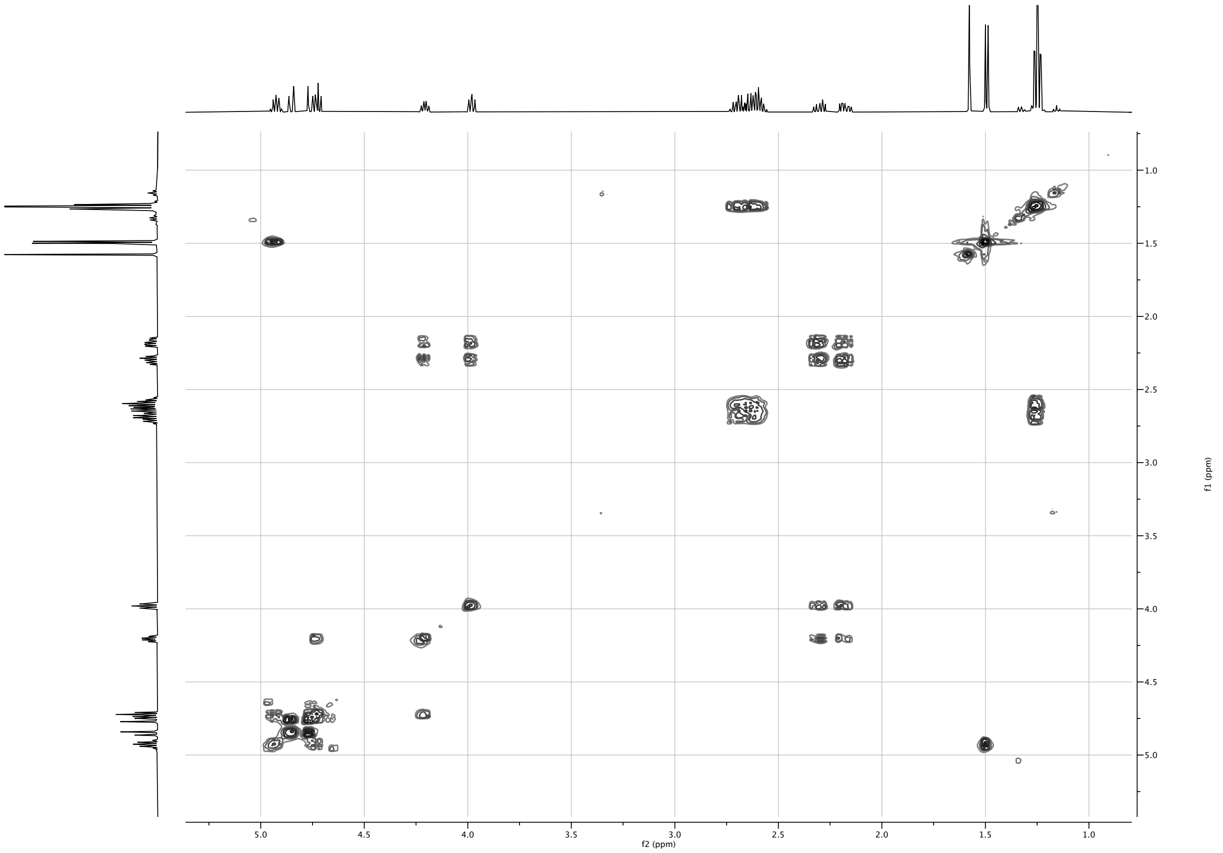

HSQC NMR, $CDCl_3$ of **S49**

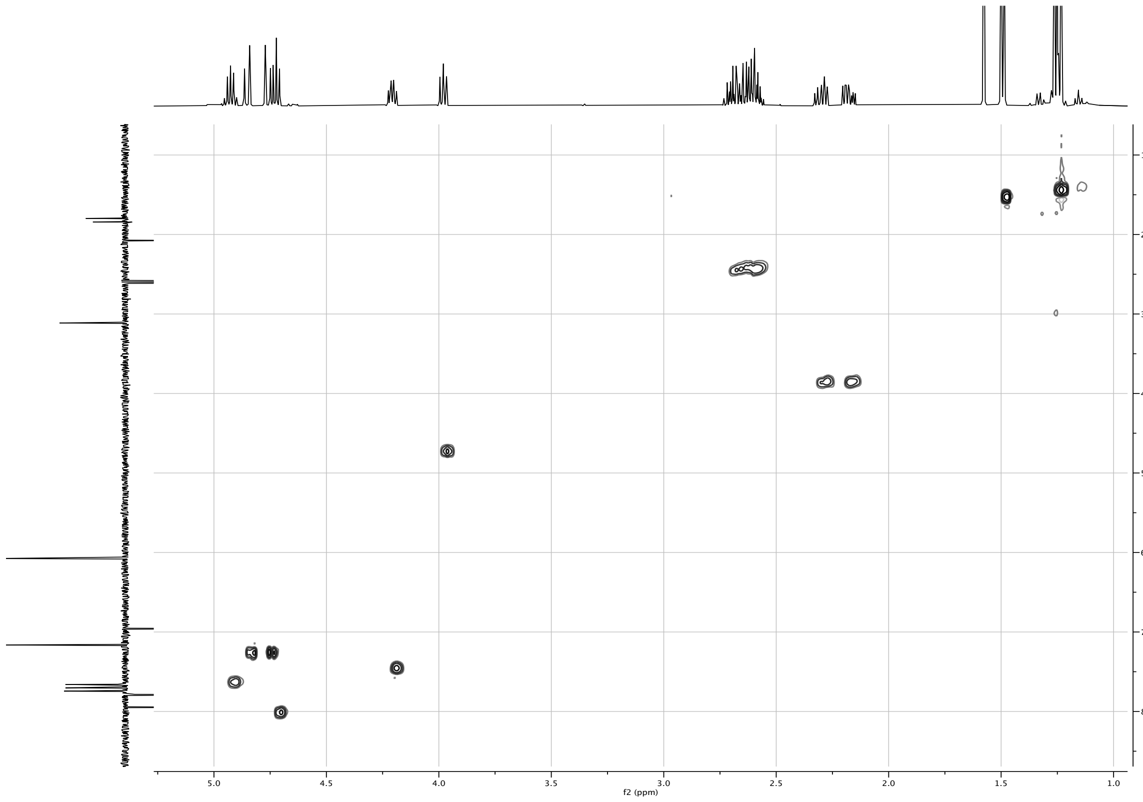

^1H NMR, 500 MHz, $CDCl_3$ of **S50**

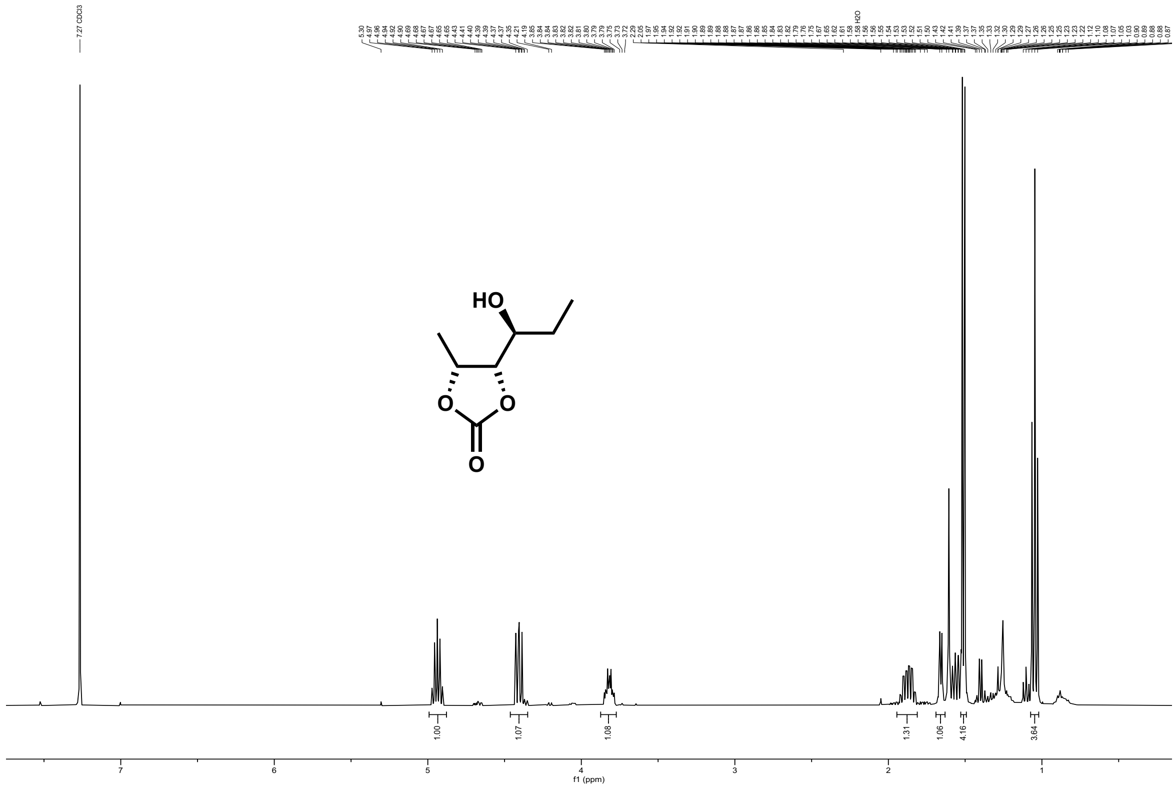

^{13}C NMR, 126 MHz, $CDCl_3$ of **S50**

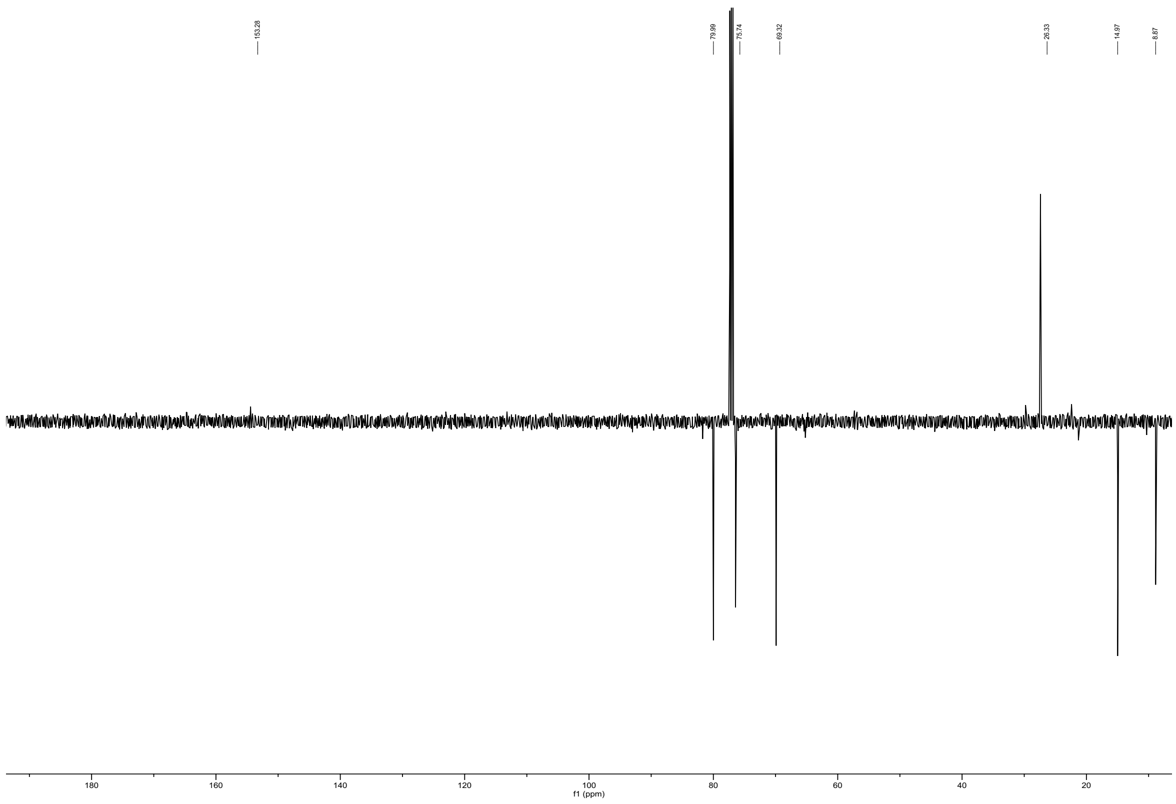

HH-COSY NMR, $CDCl_3$ of **S50**

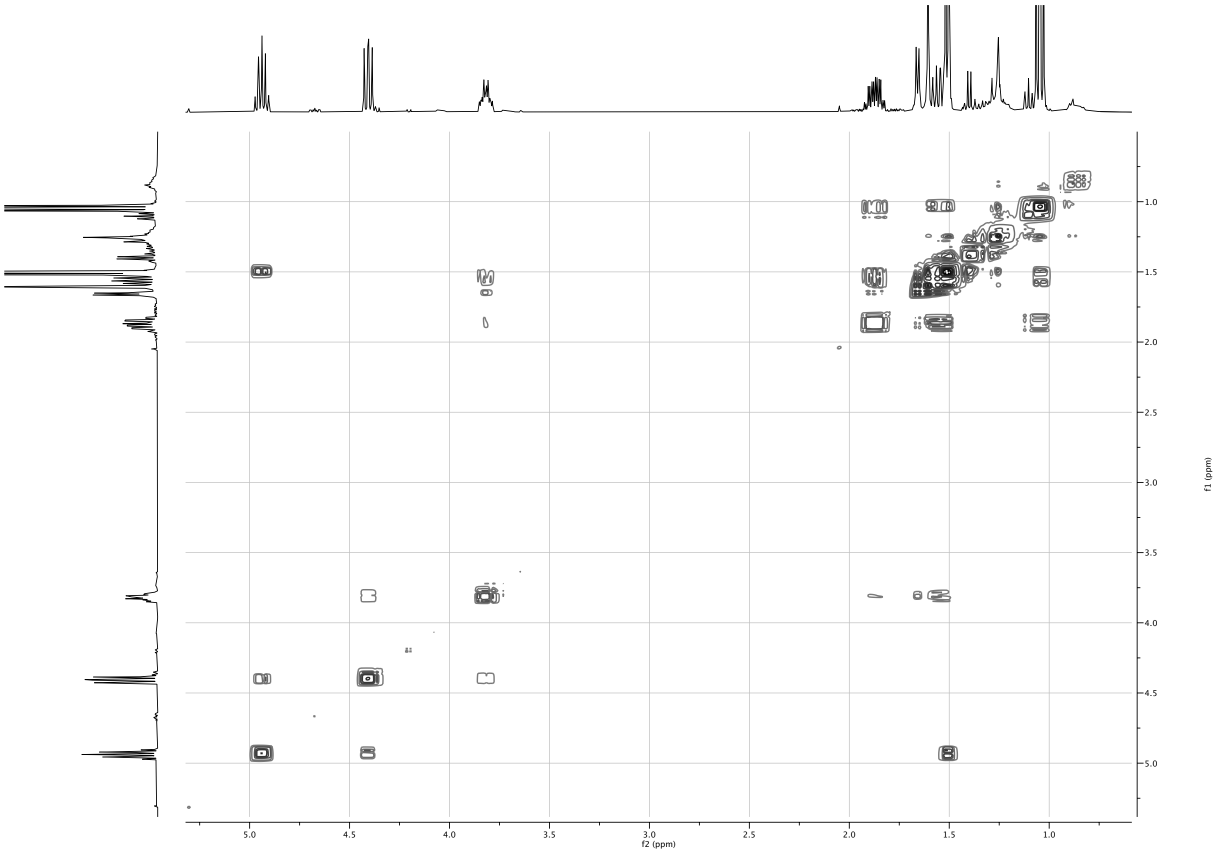

HSQC NMR, $CDCl_3$ of **S50**

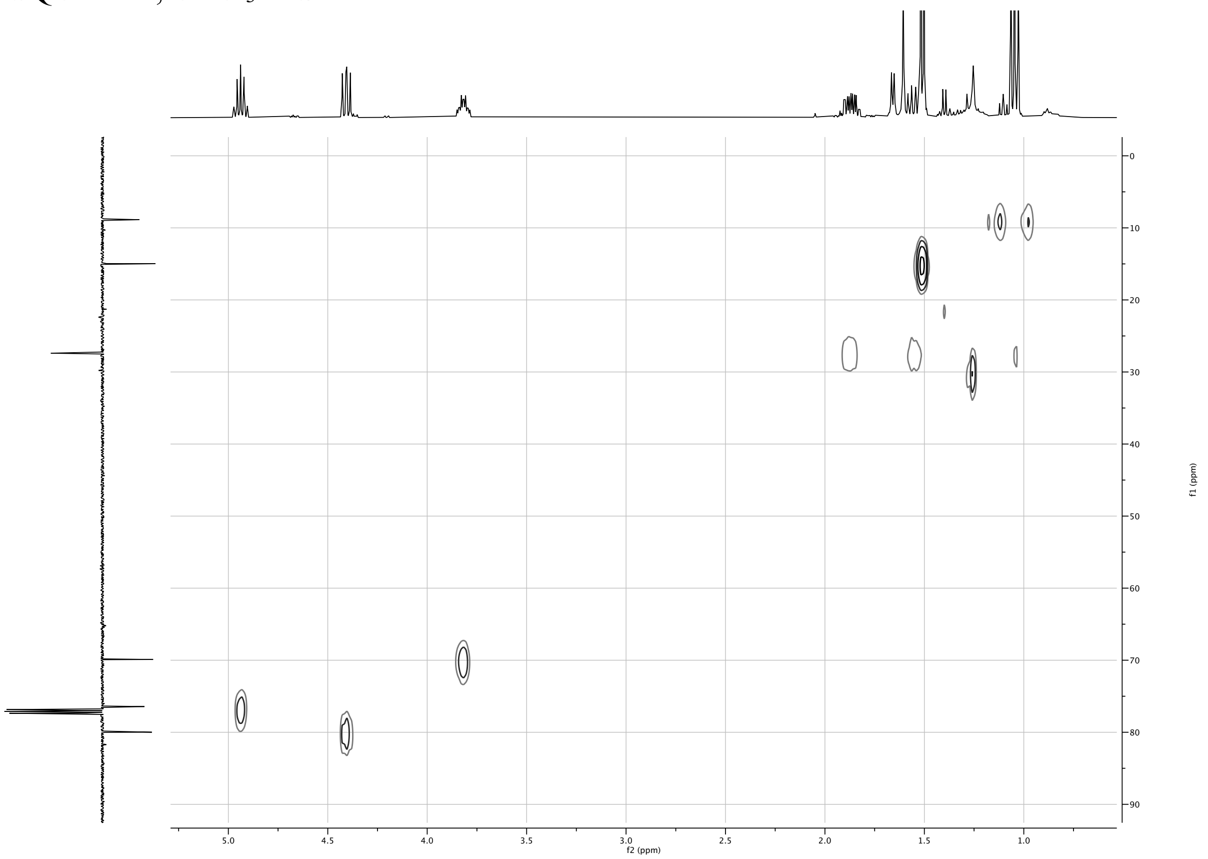

HMBC NMR, $CDCl_3$ of **S50**

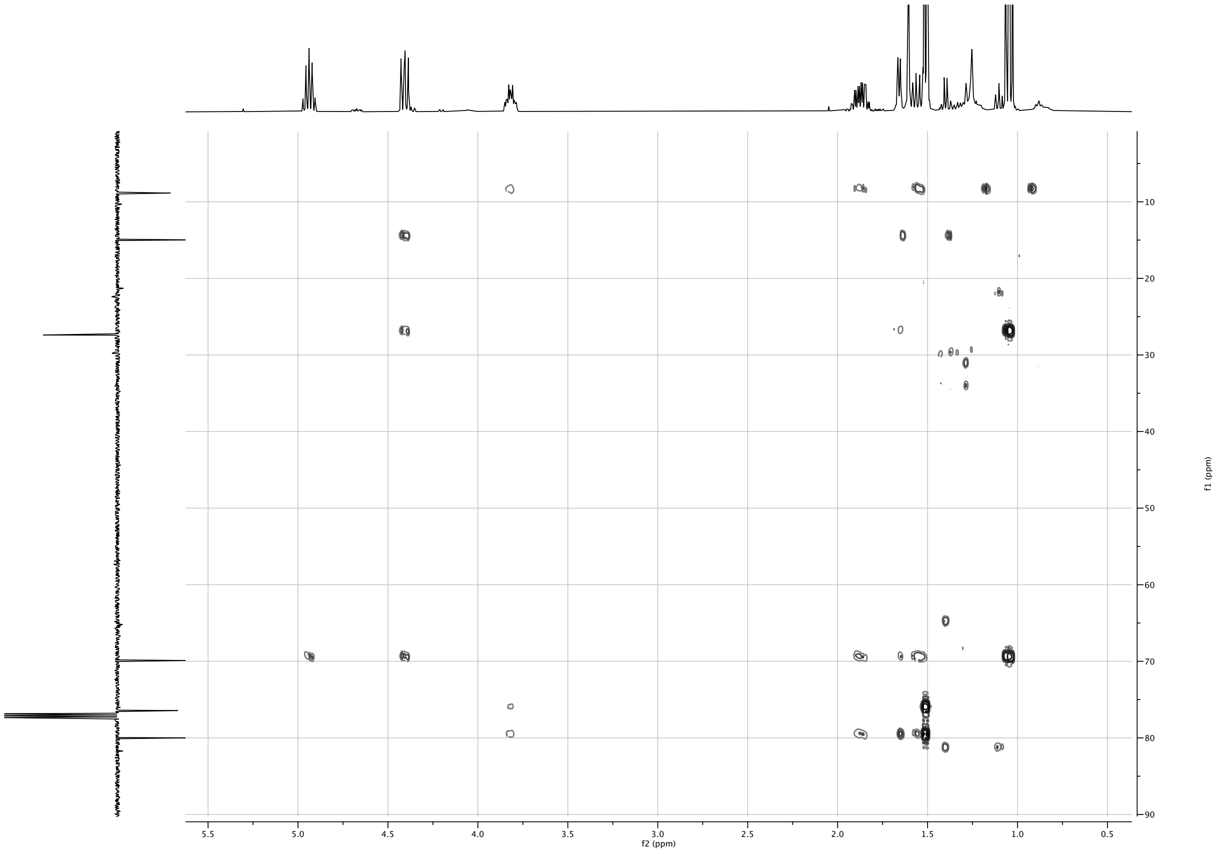

^{1}H NMR, 500 MHz, $CDCl_3$ of **S52**

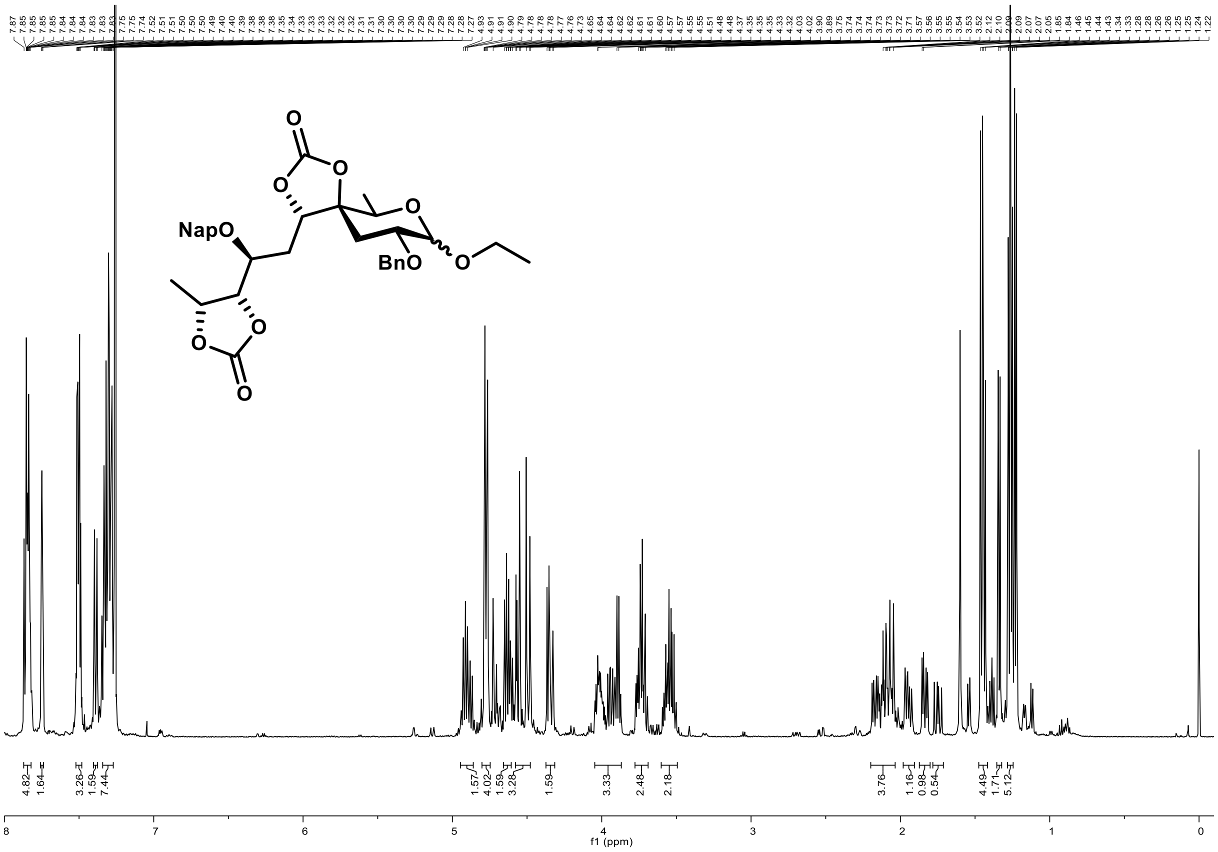

^{13}C NMR, 126 MHz, $CDCl_3$ of **S52**

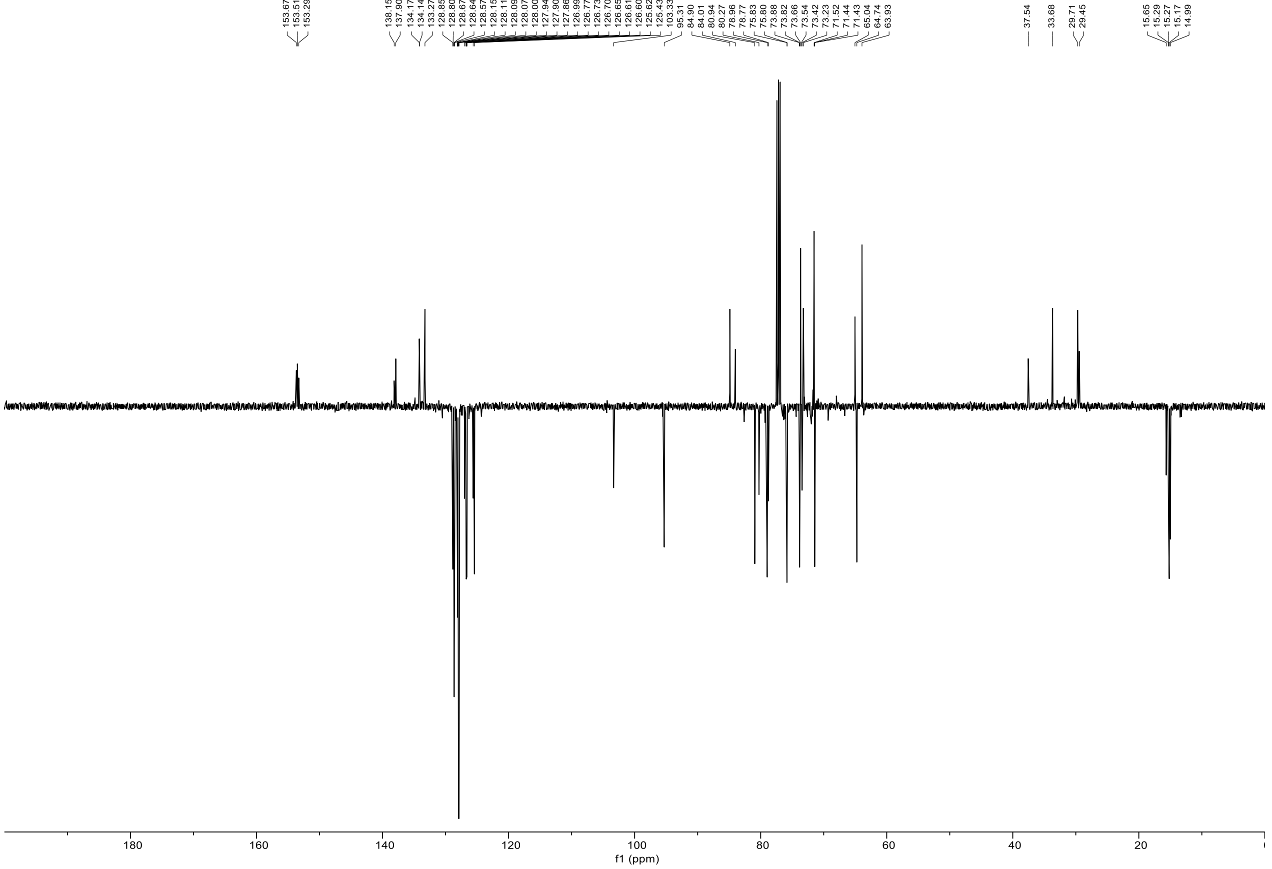

HH-COSY NMR, $CDCl_3$ of **S52**

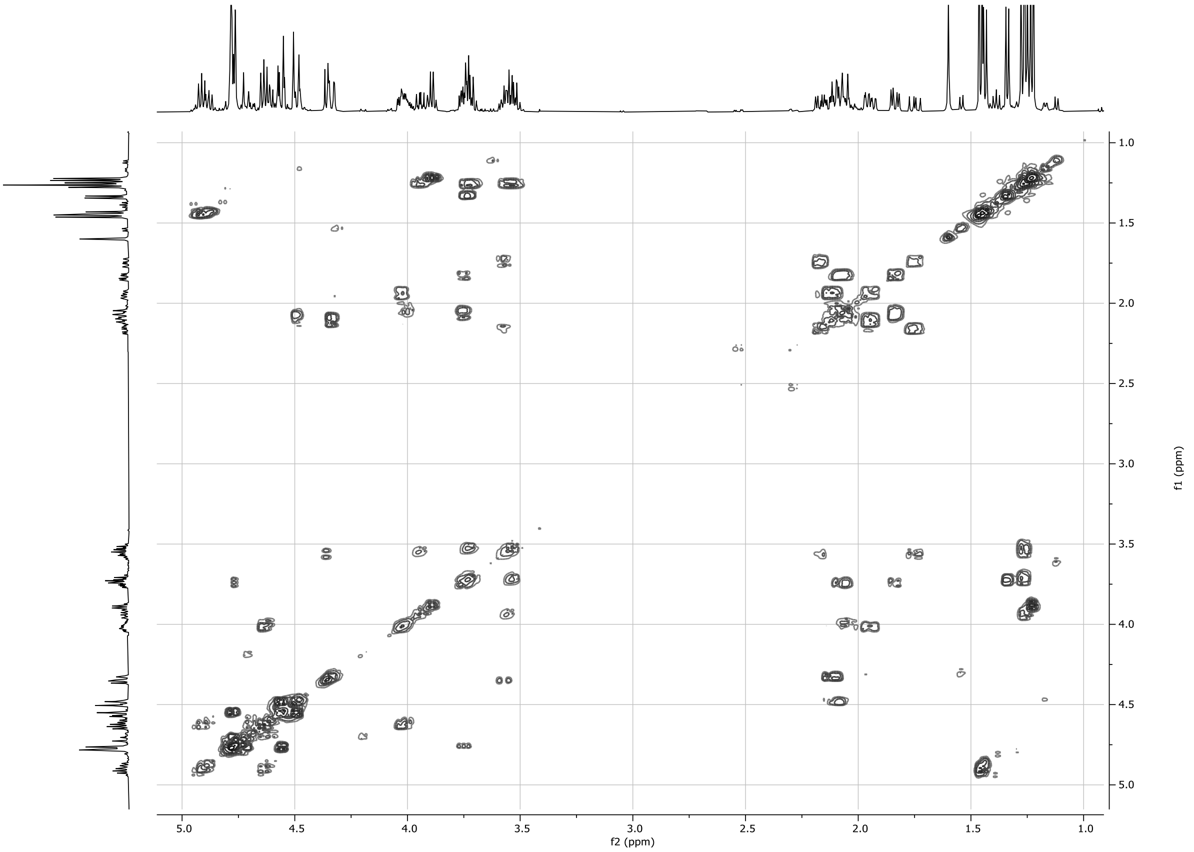

HSQC NMR, $CDCl_3$ of **S52**

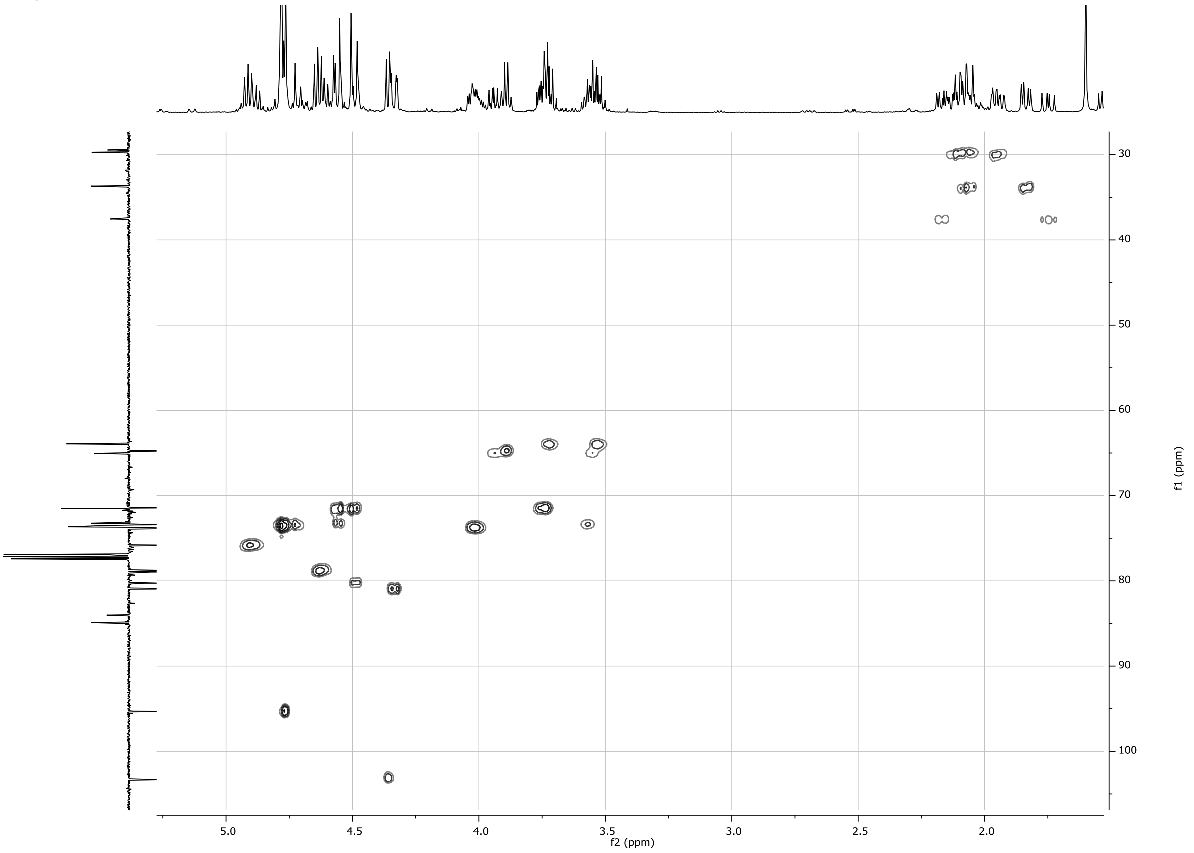

^{1}H NMR, 500 MHz, $CDCl_3$ of **S53**

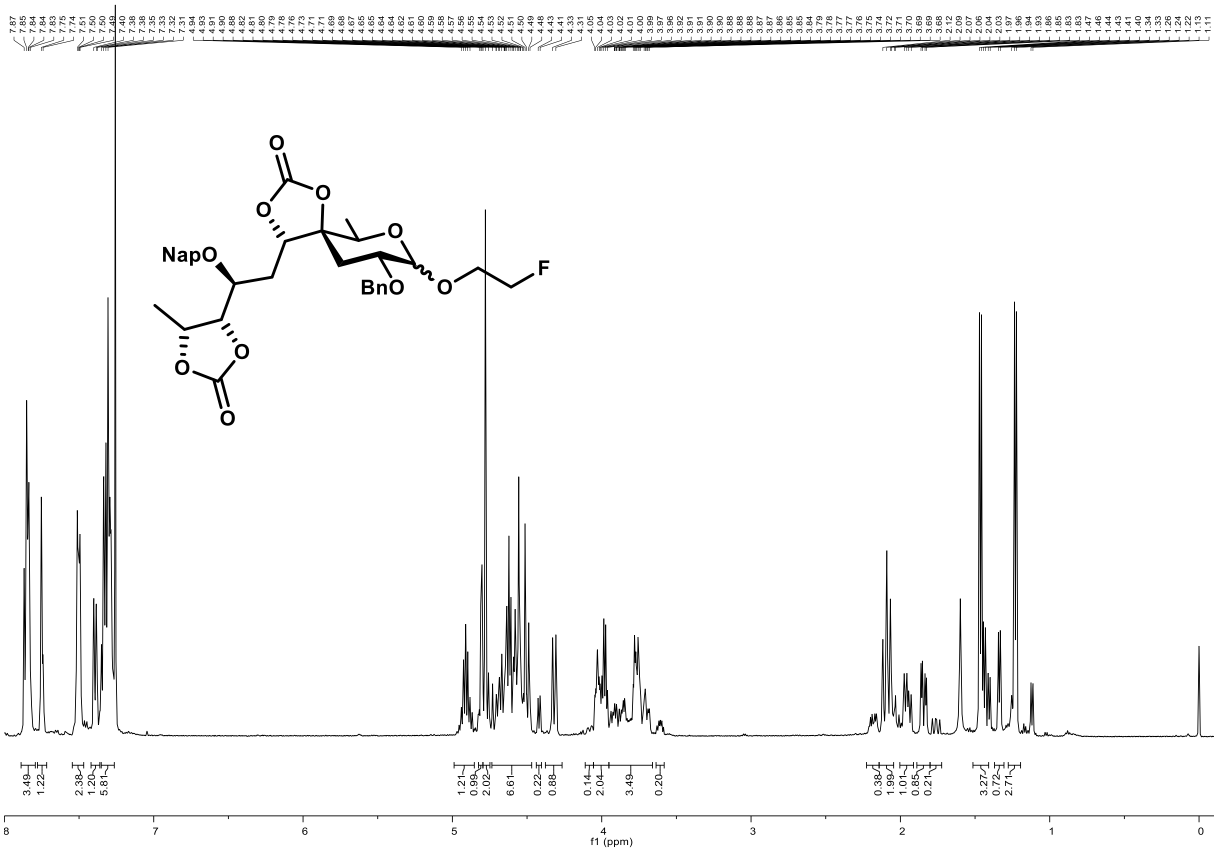

^{13}C NMR, 126 MHz, $CDCl_3$ of **S53**

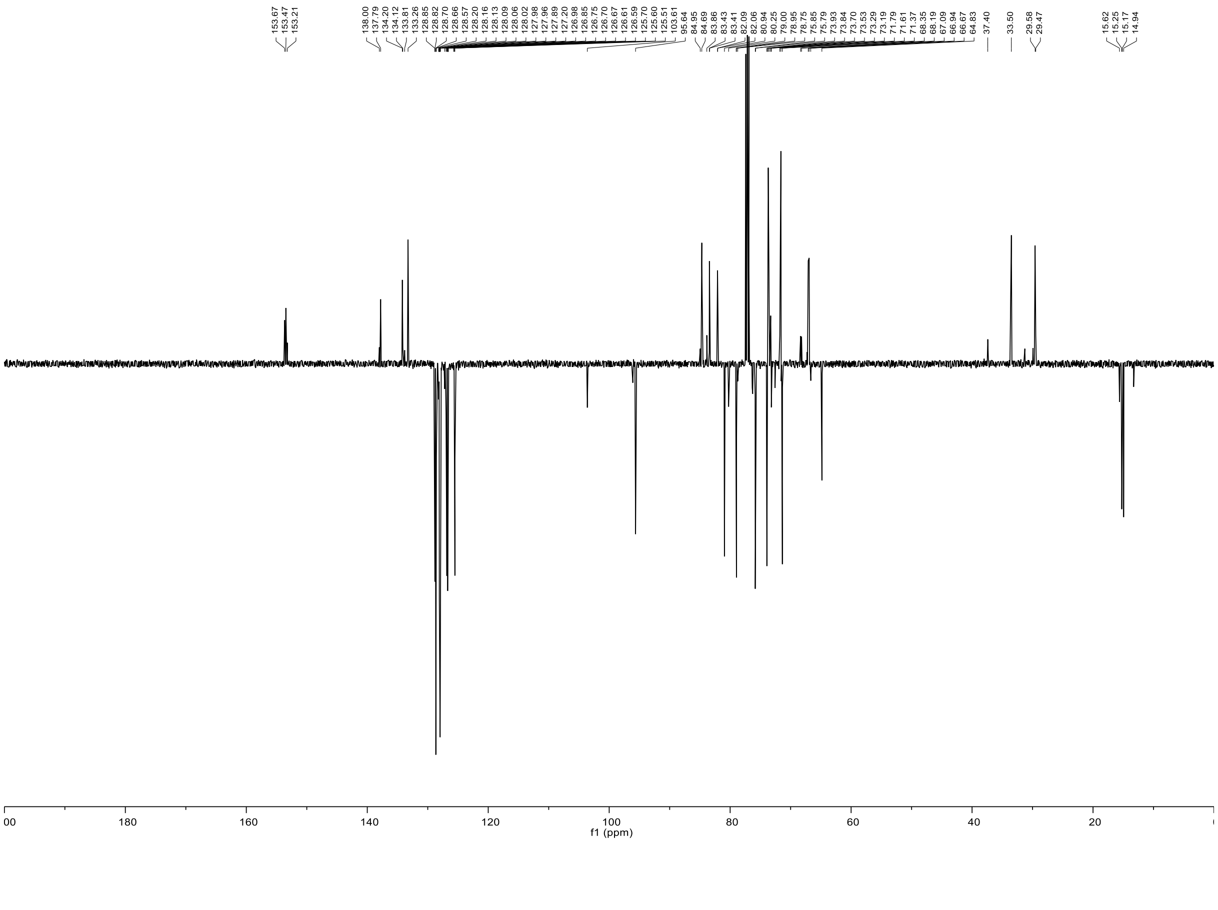

HH-COSY NMR, $CDCl_3$ of **S53**

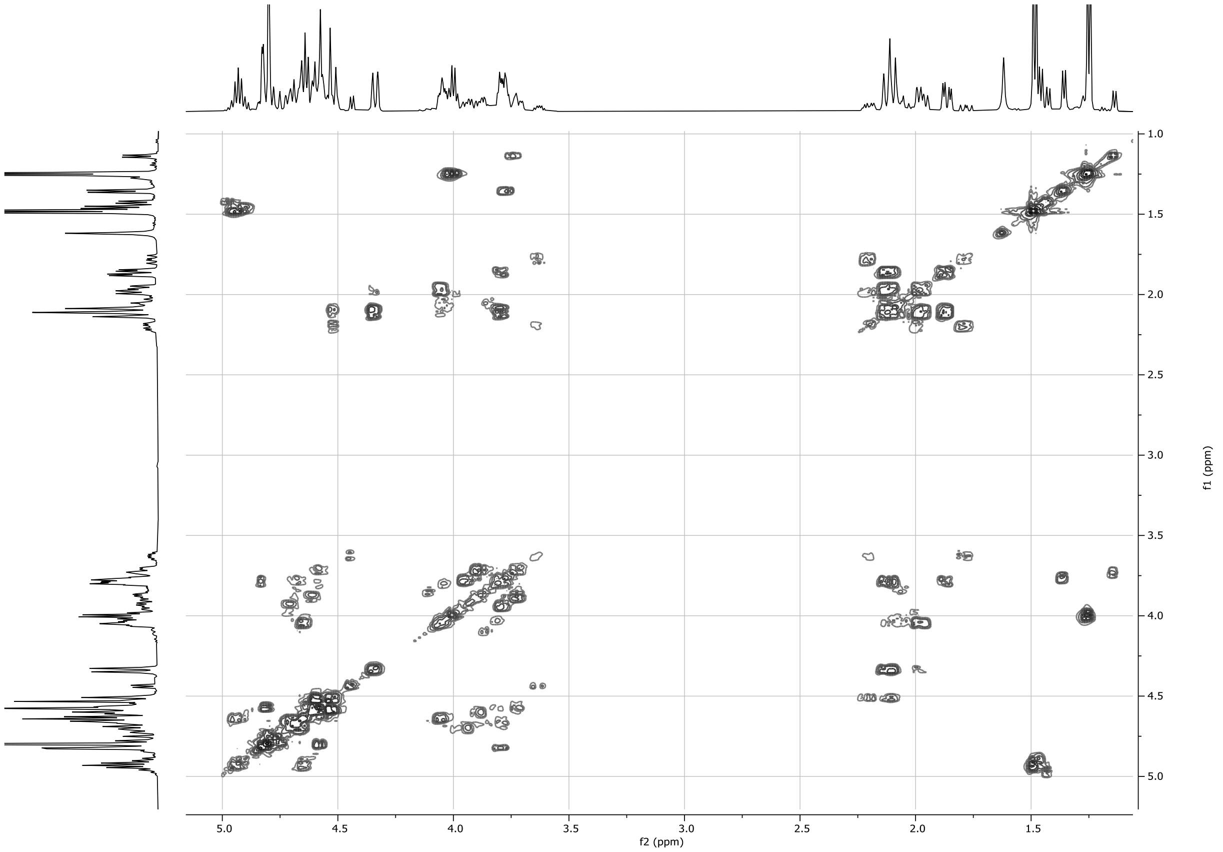

HSQC NMR, $CDCl_3$ of **S53**

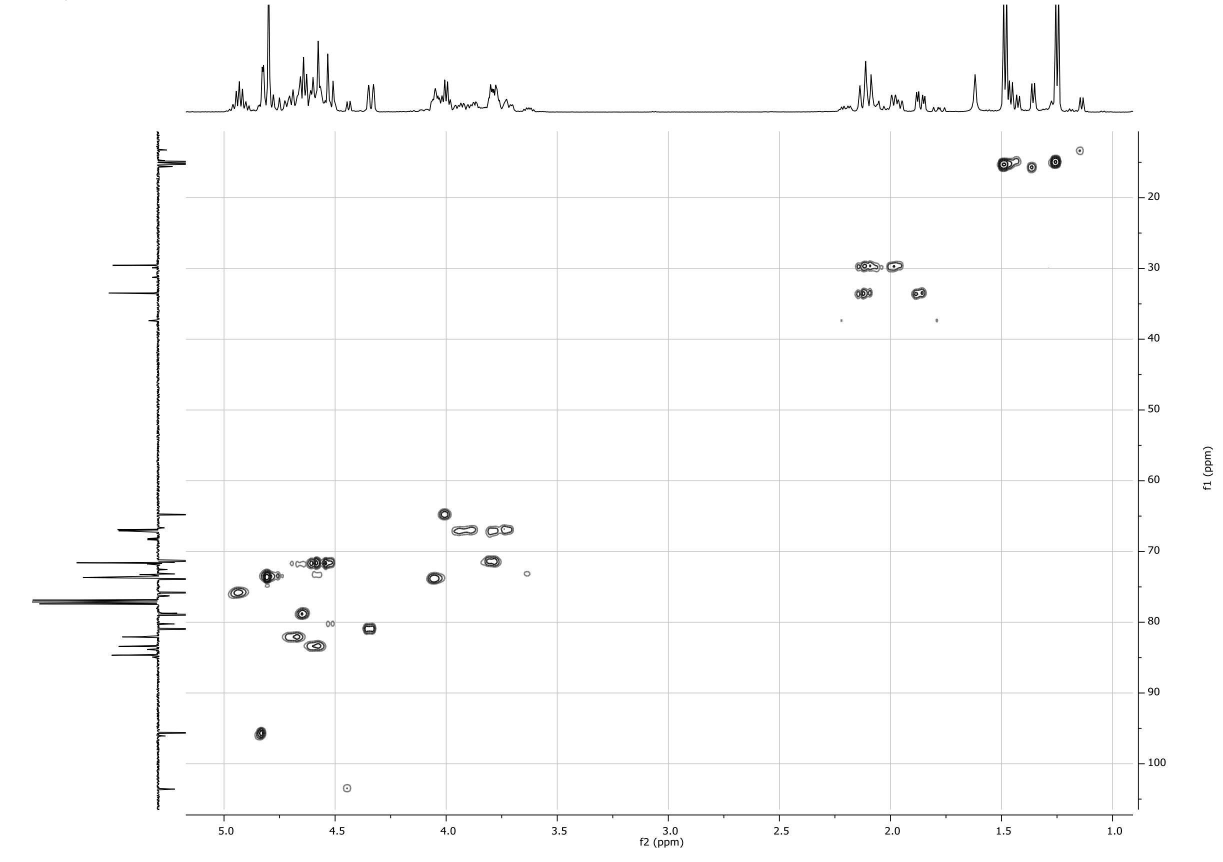

^{1}H NMR, 400 MHz, $CDCl_3$ of **S54**

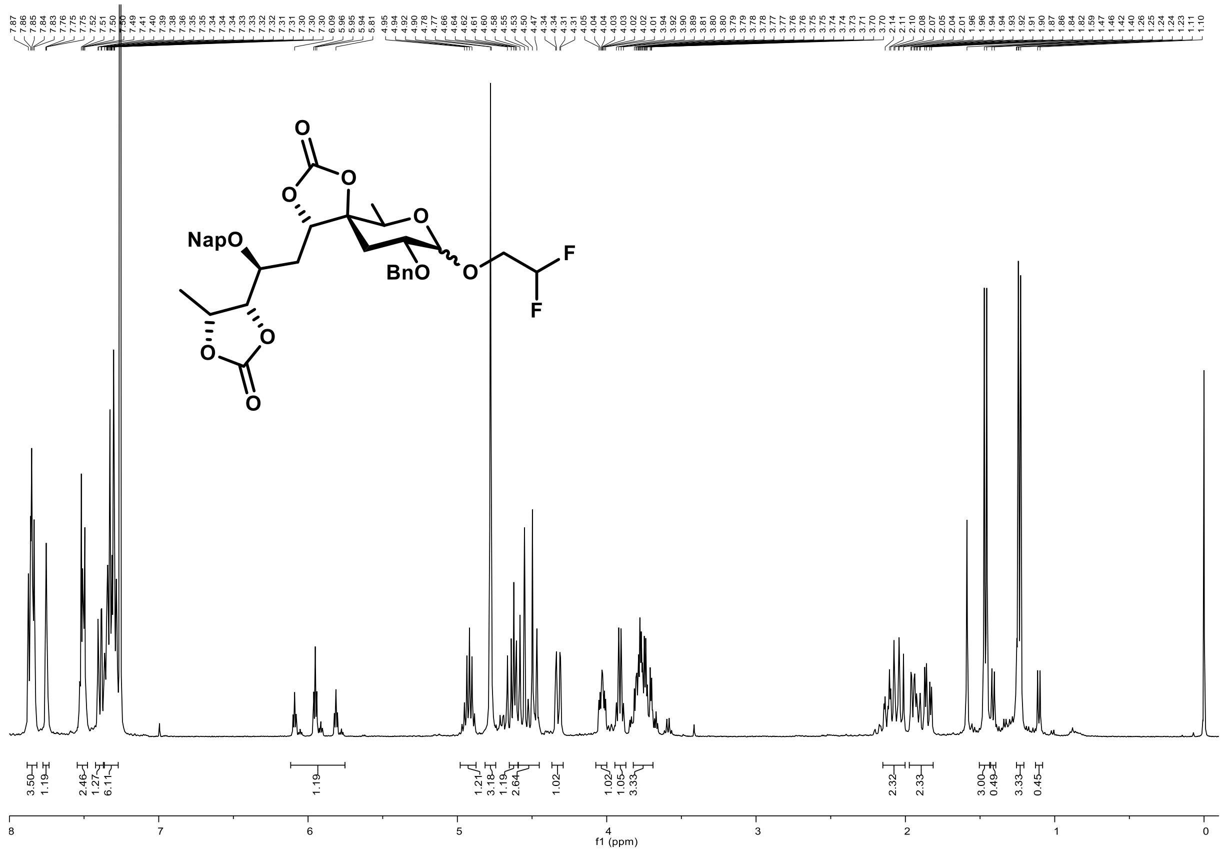

^{13}C NMR, 101 MHz, $CDCl_3$ of **S54**

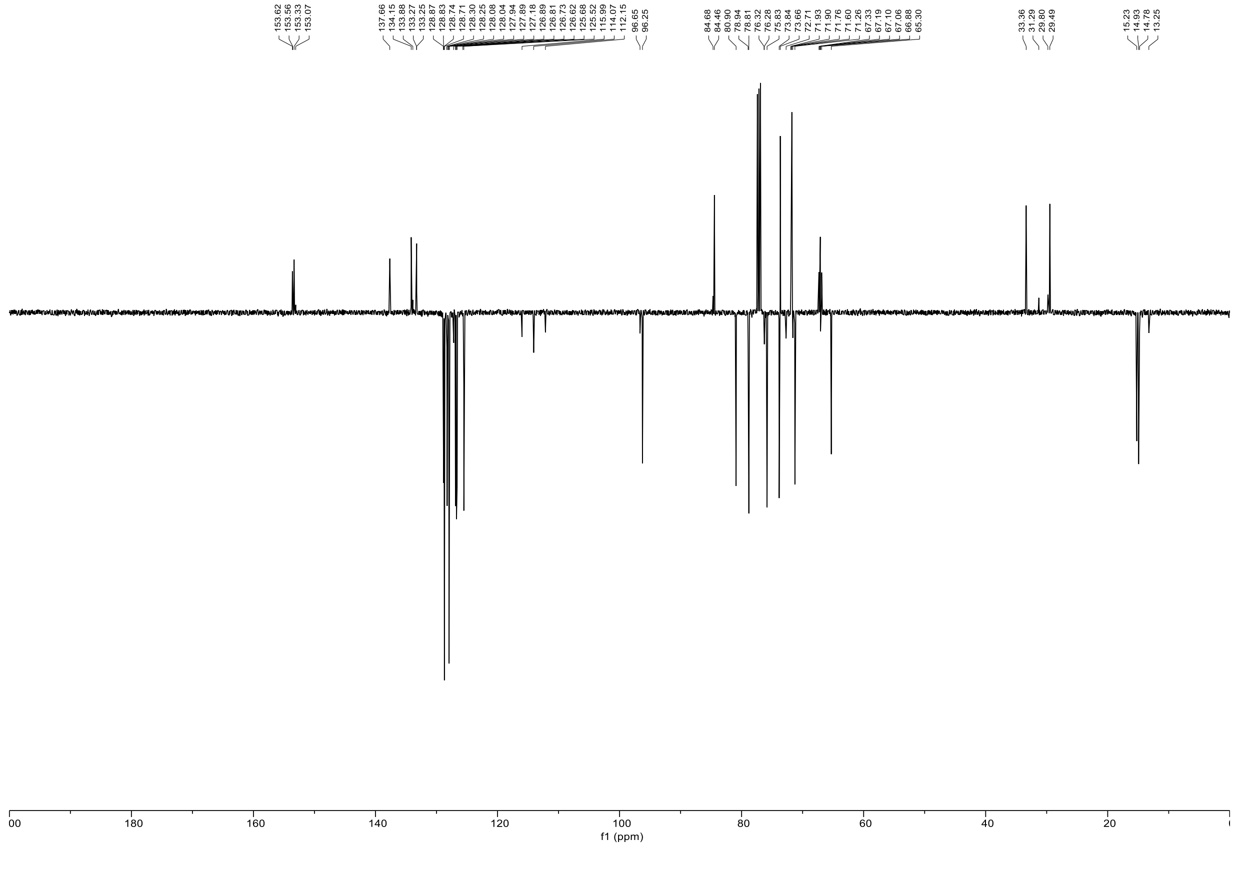

HH-COSY NMR, CDCl$_3$ of **S54**

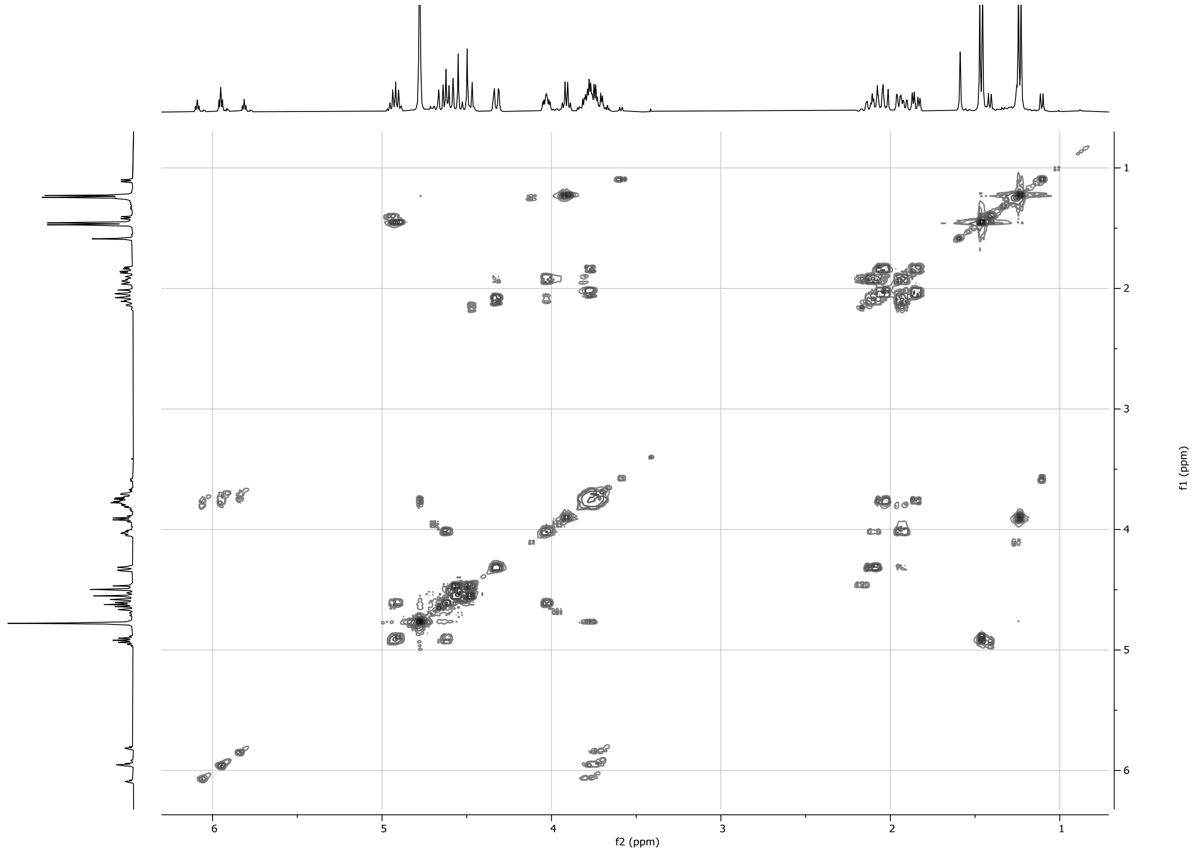

HSQC NMR, CDCl$_3$ of **S54**

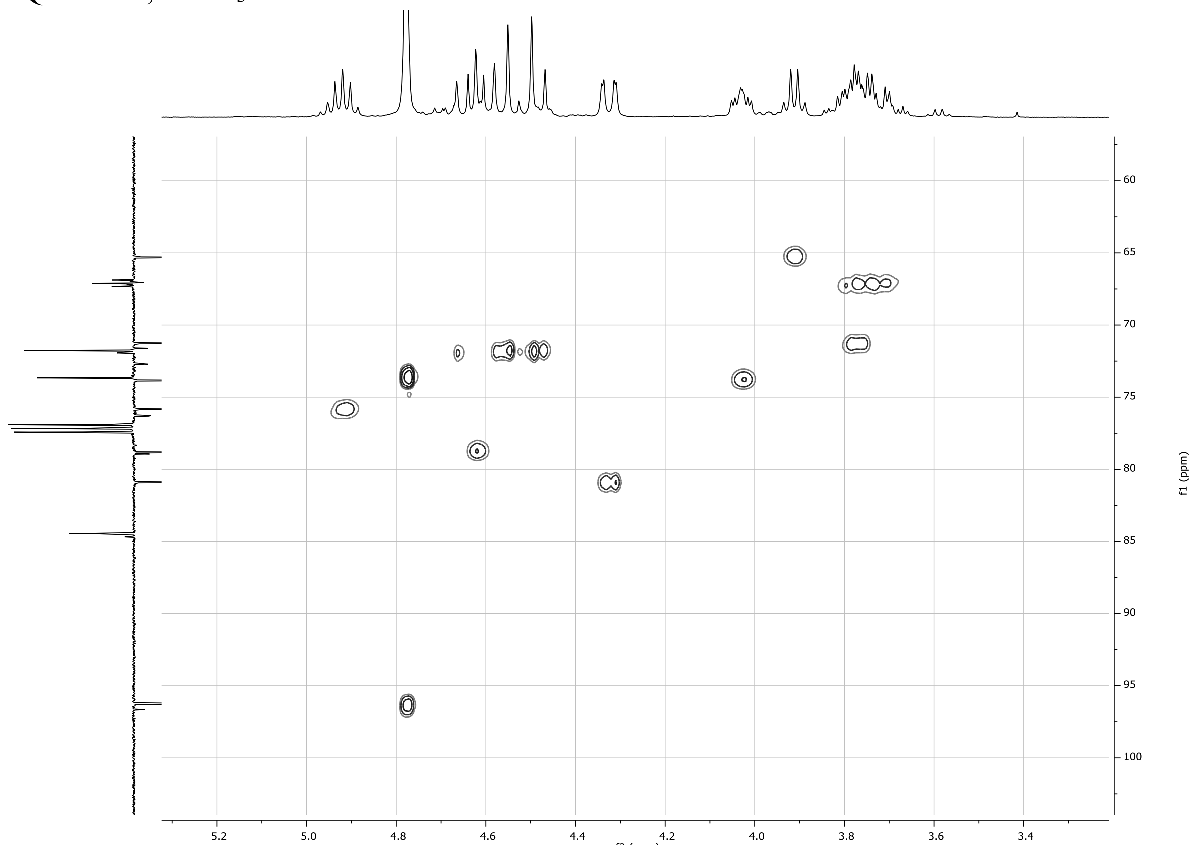

¹H NMR, 400 MHz, $CDCl_3$ of **S55**

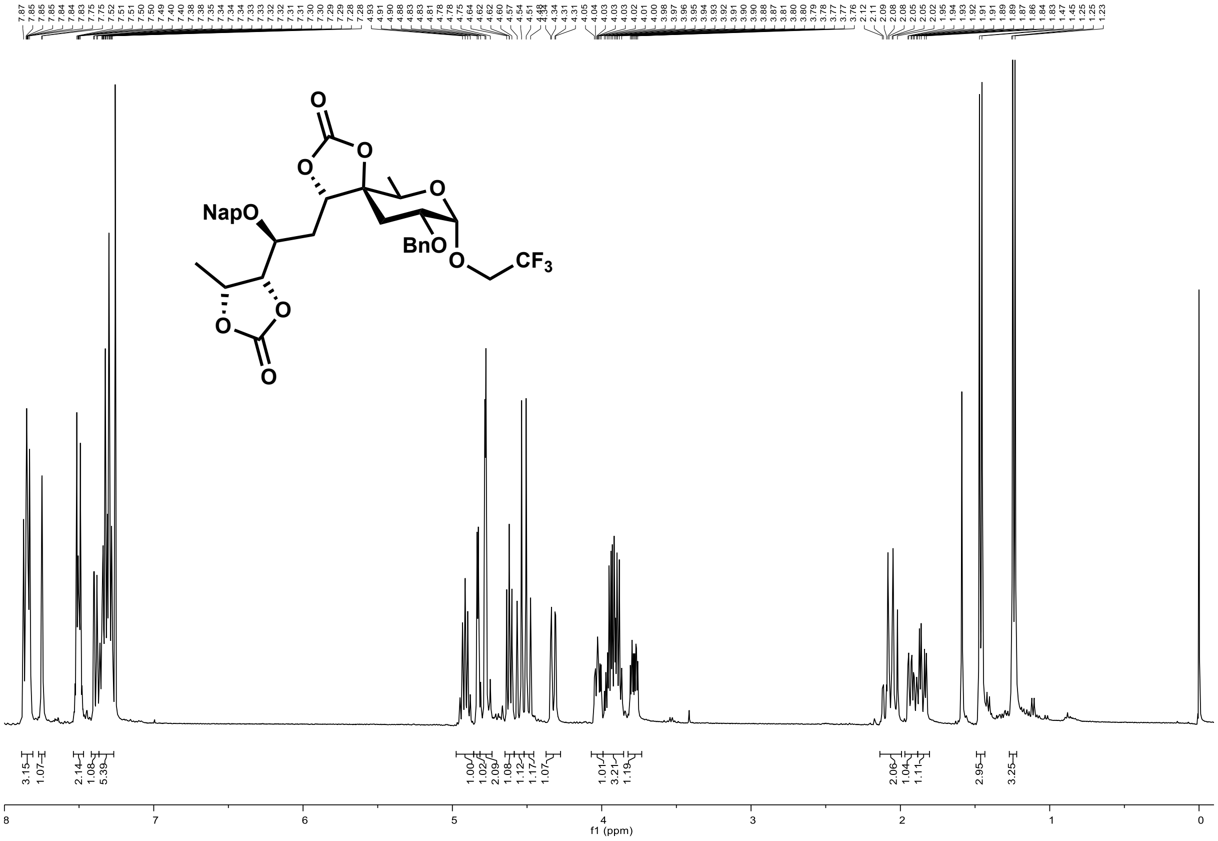

¹³C NMR, 101 MHz, $CDCl_3$ of **S55**

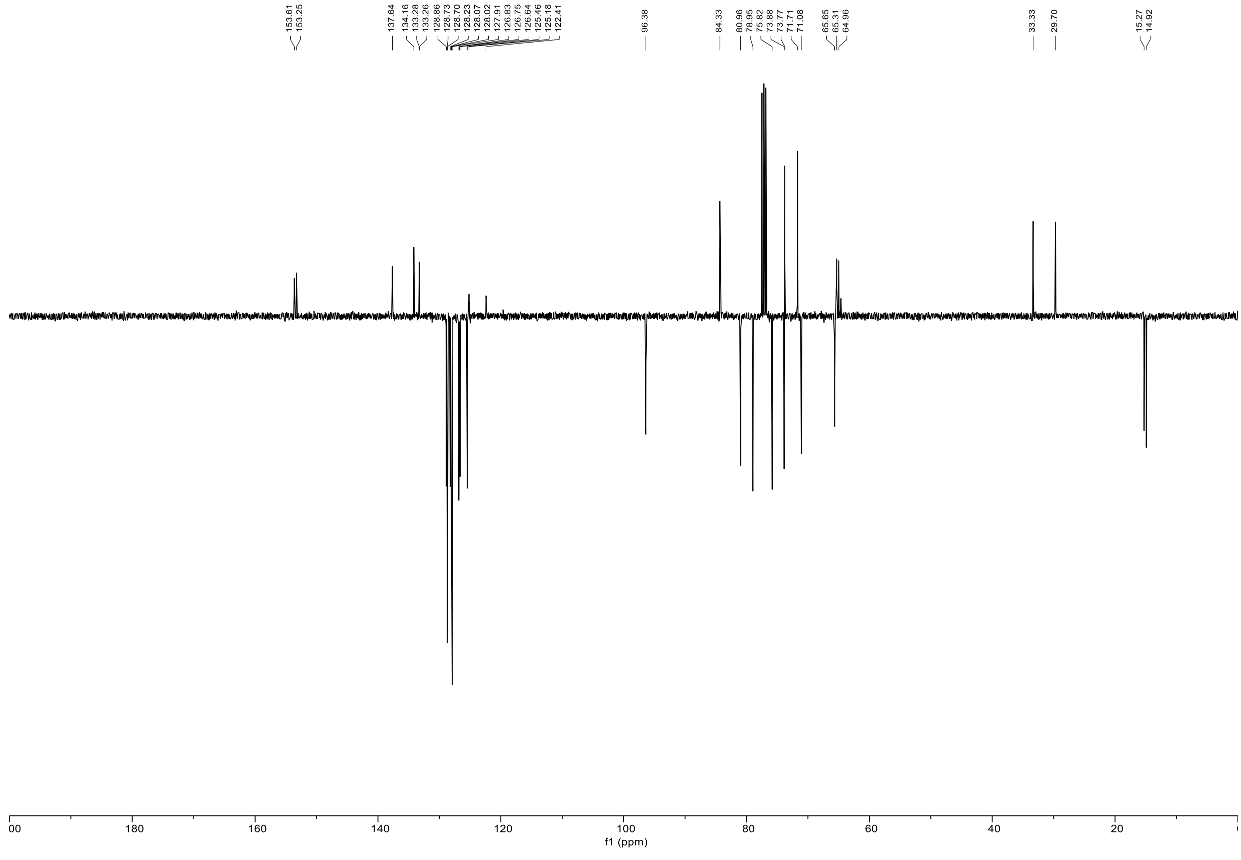

HH-COSY NMR, $CDCl_3$ of **S55**

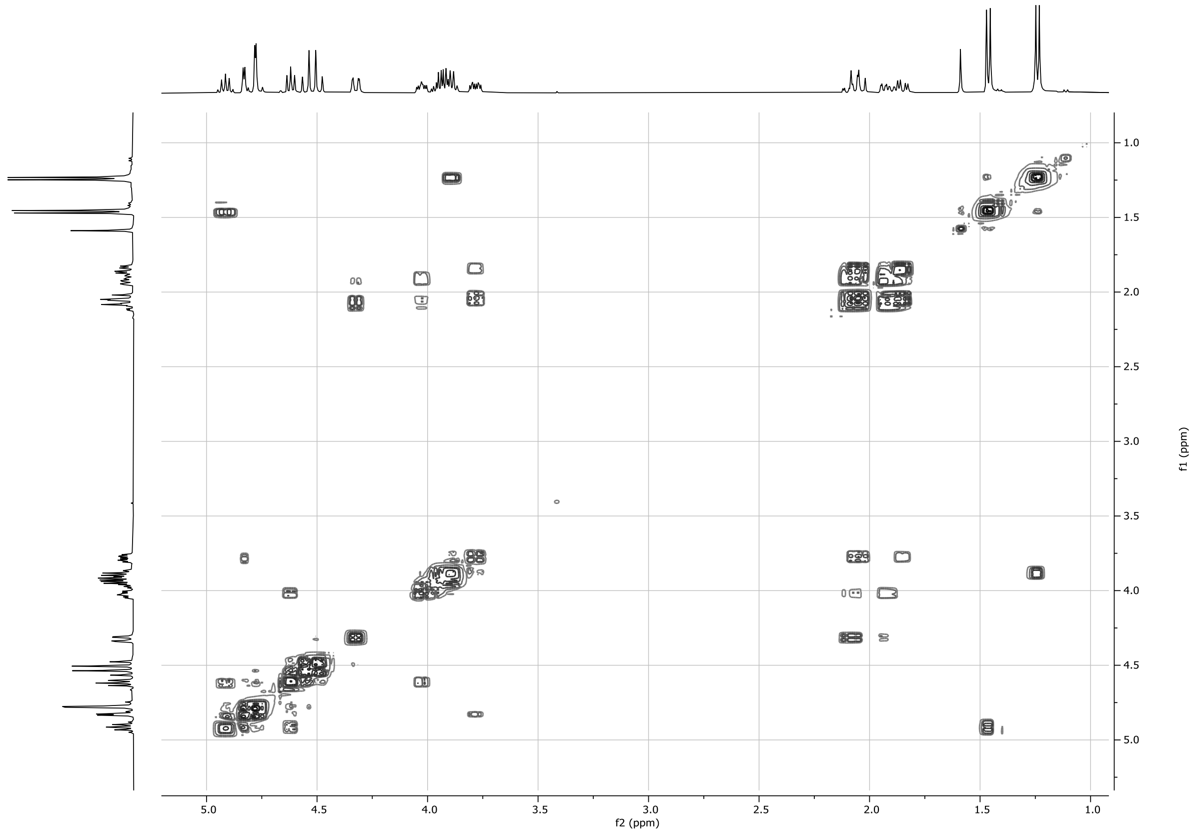

HSQC NMR, $CDCl_3$ of **S55**

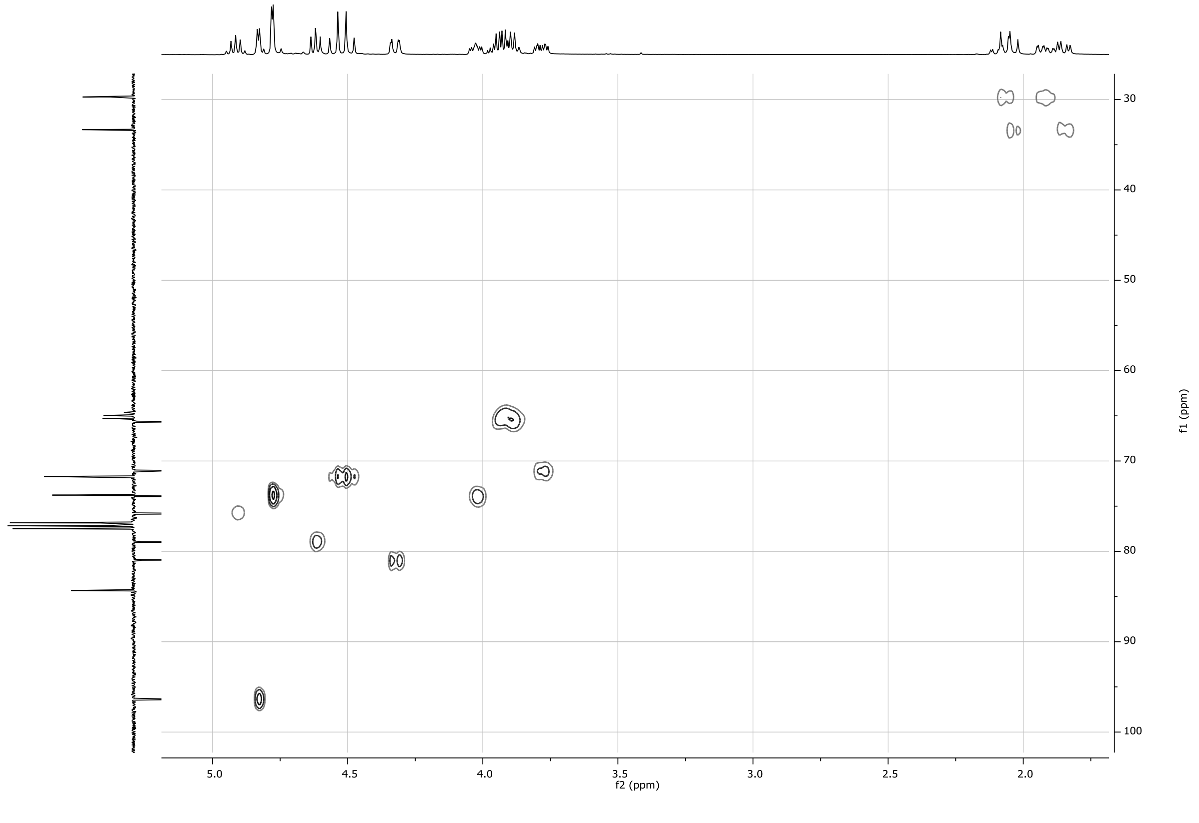

^{1}H NMR, 500 MHz, $CDCl_3$ of **S56**

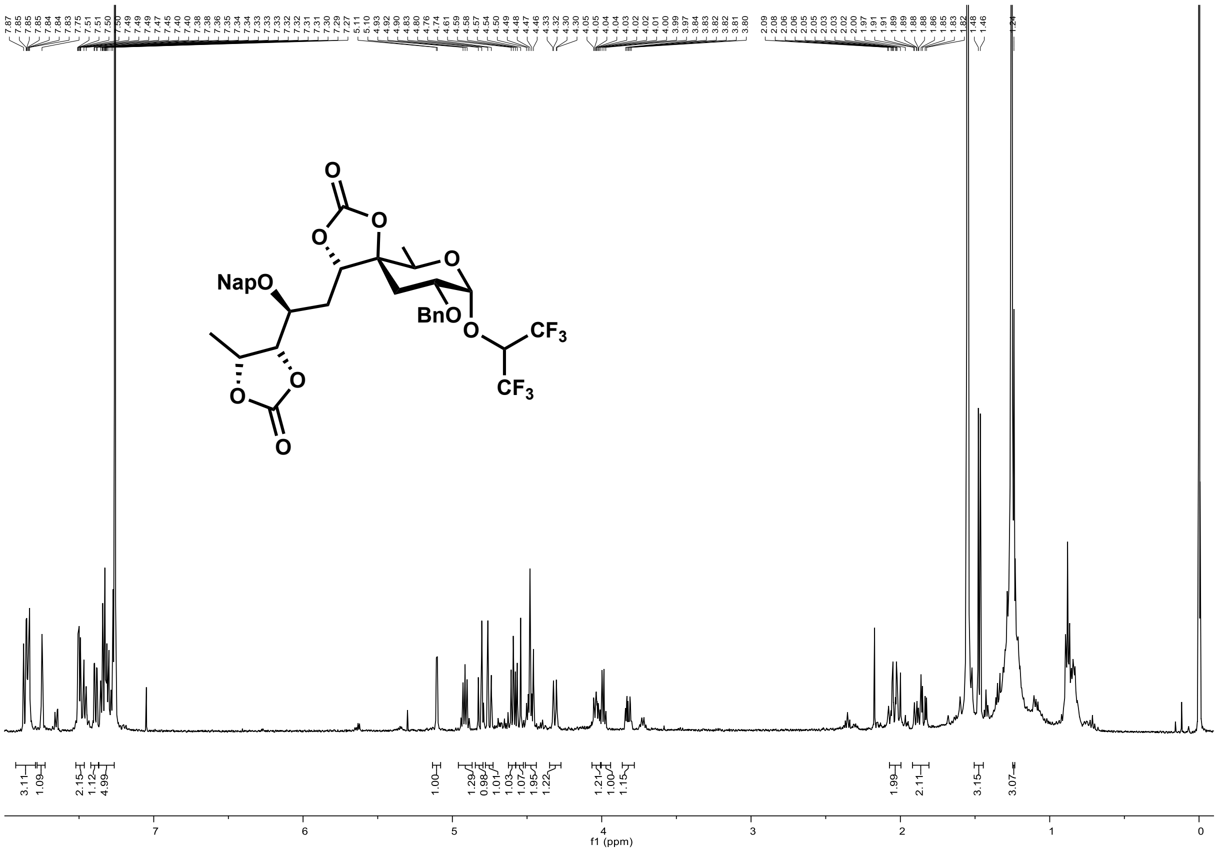

^{13}C NMR, 126 MHz, $CDCl_3$ of **S56**

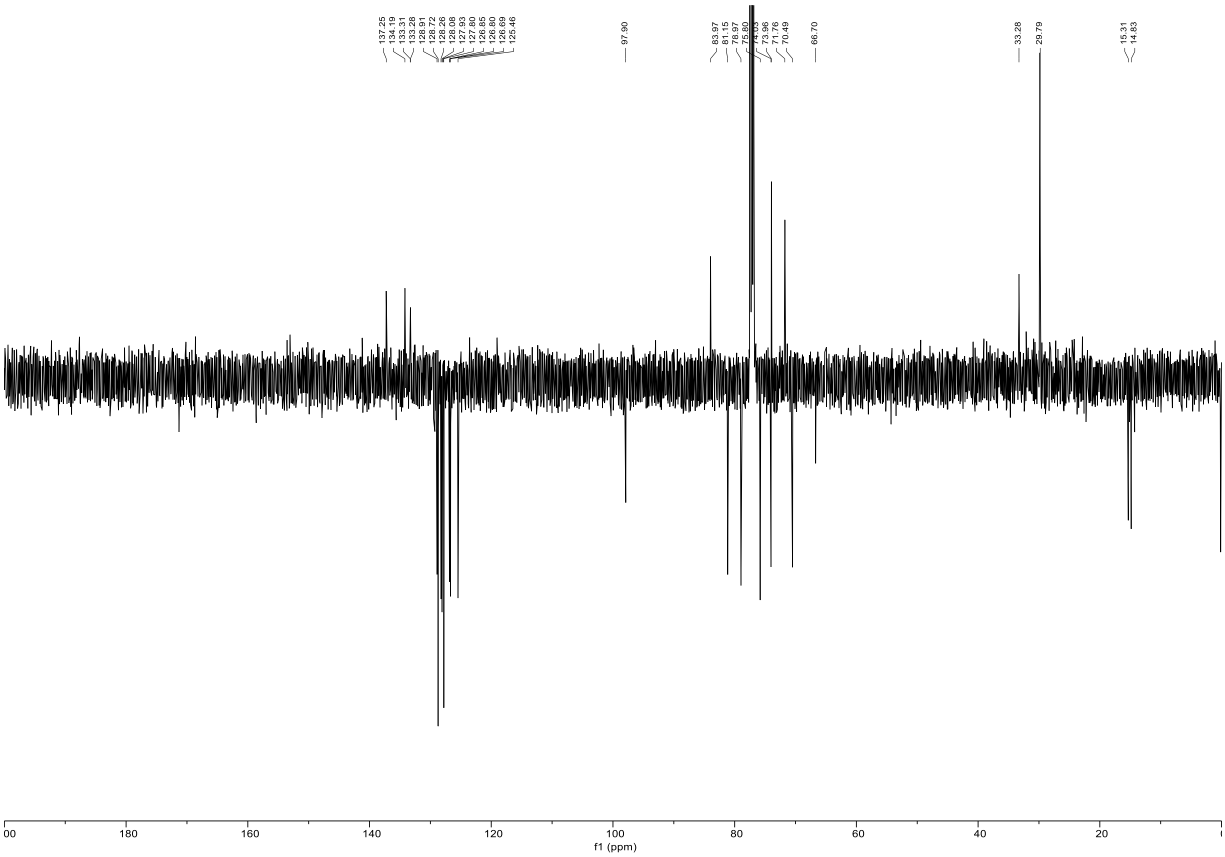

HH-COSY NMR, $CDCl_3$ of **S56**

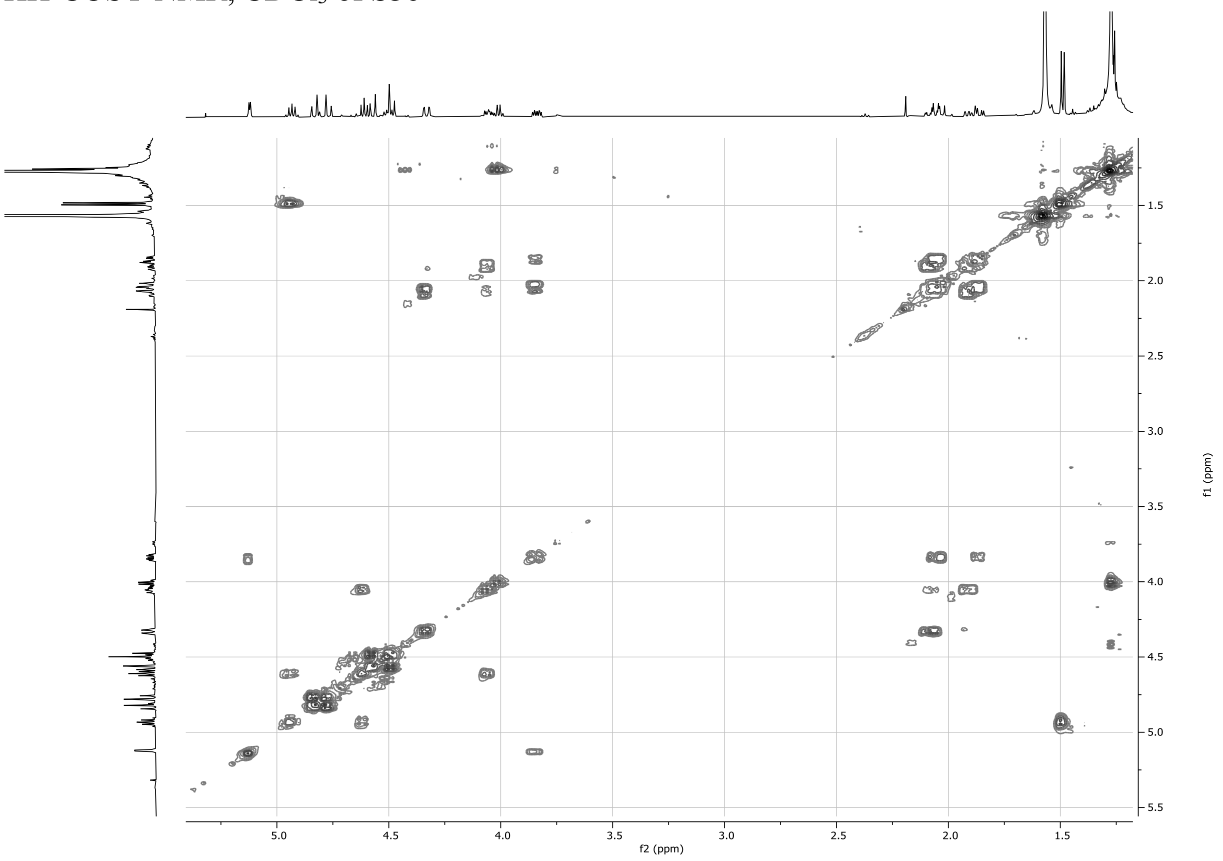

HSQC NMR, $CDCl_3$ of **S56**

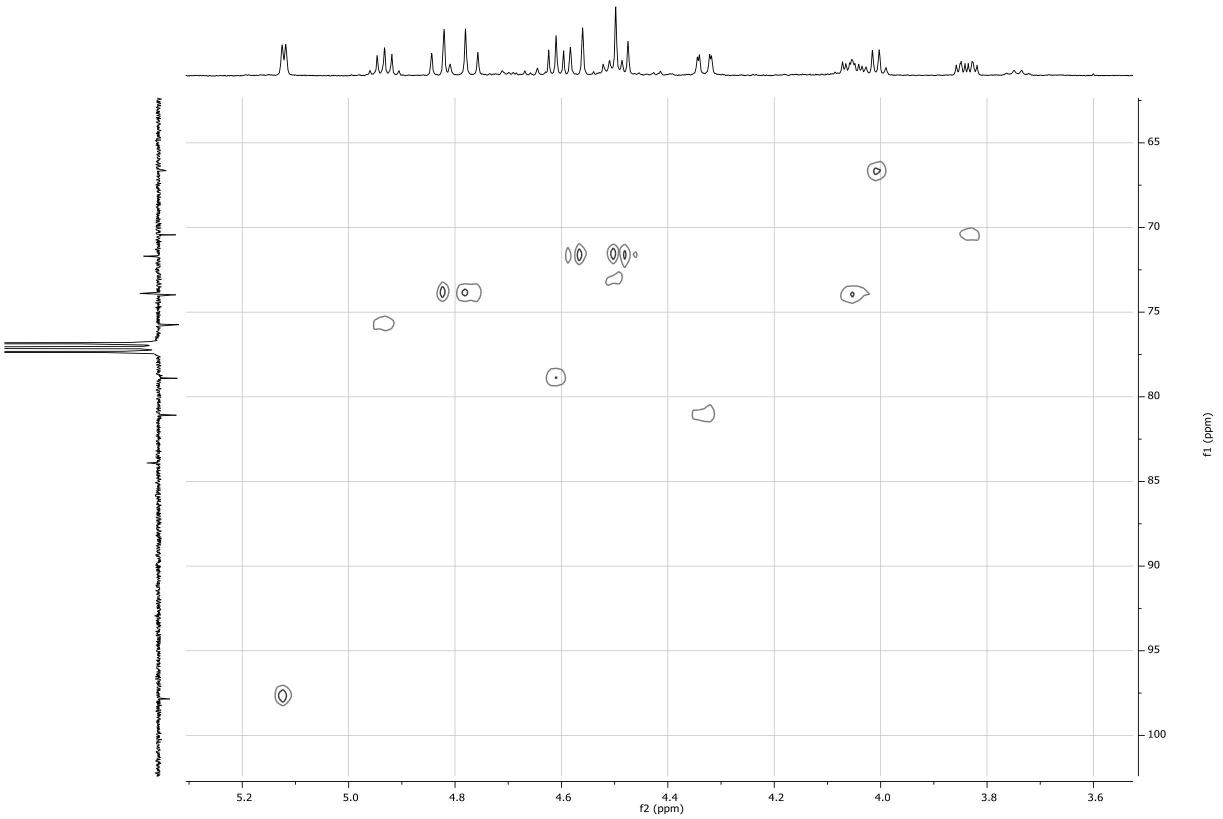

^{1}H NMR, 500 MHz, $CDCl_3$ of **S57**

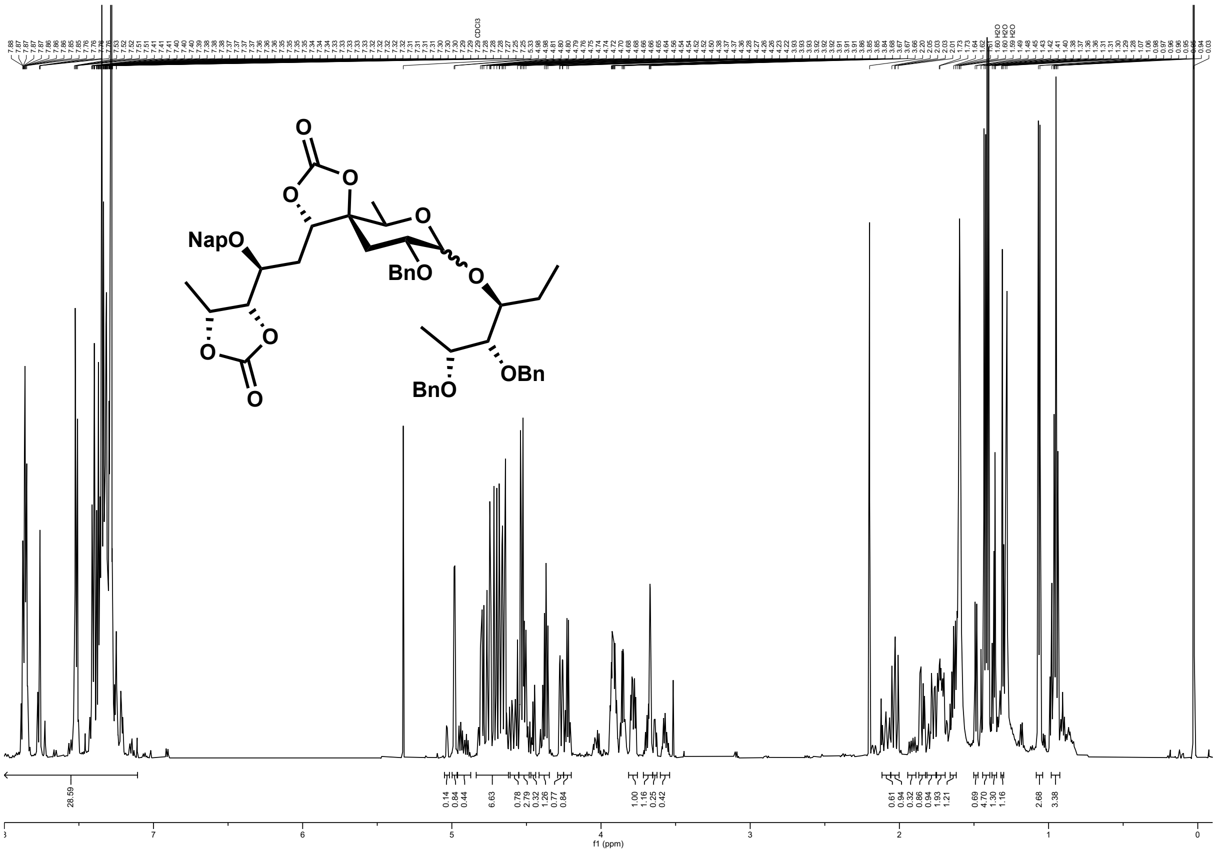

^{13}C NMR, 126 MHz, $CDCl_3$ of **S57**

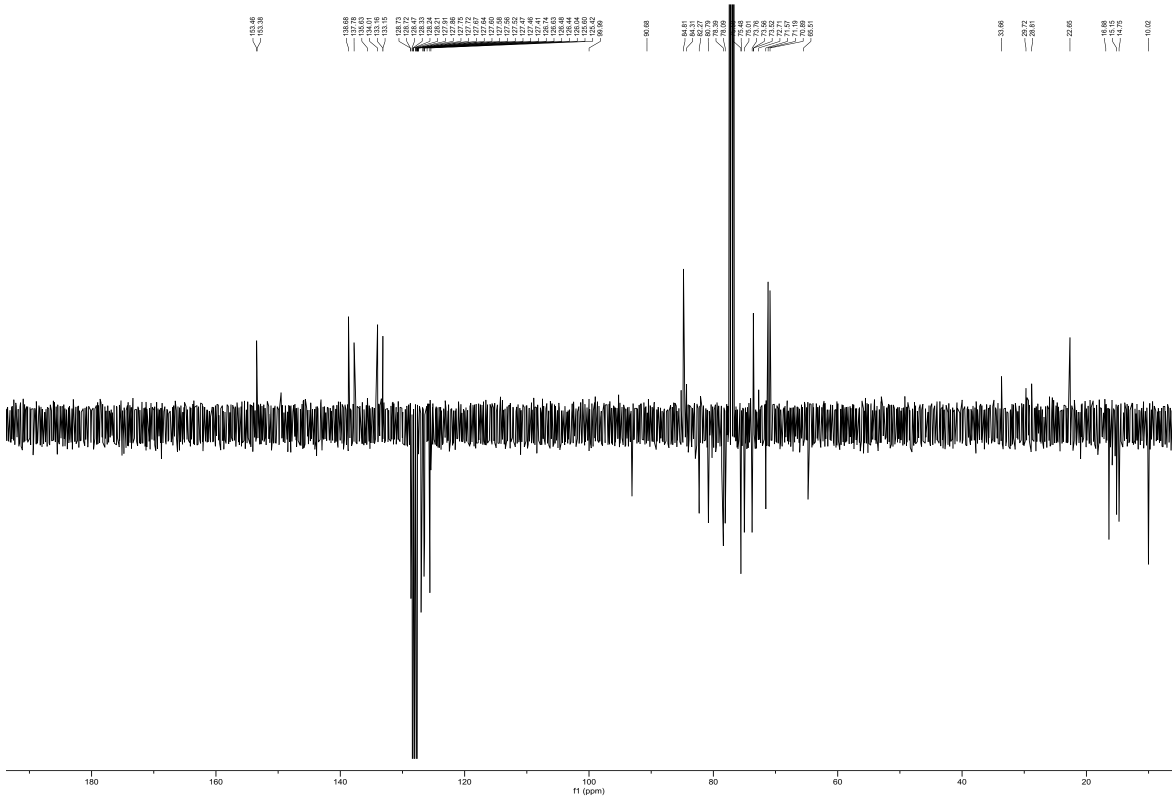

HH-COSY NMR, CDCl₃ of **S57**

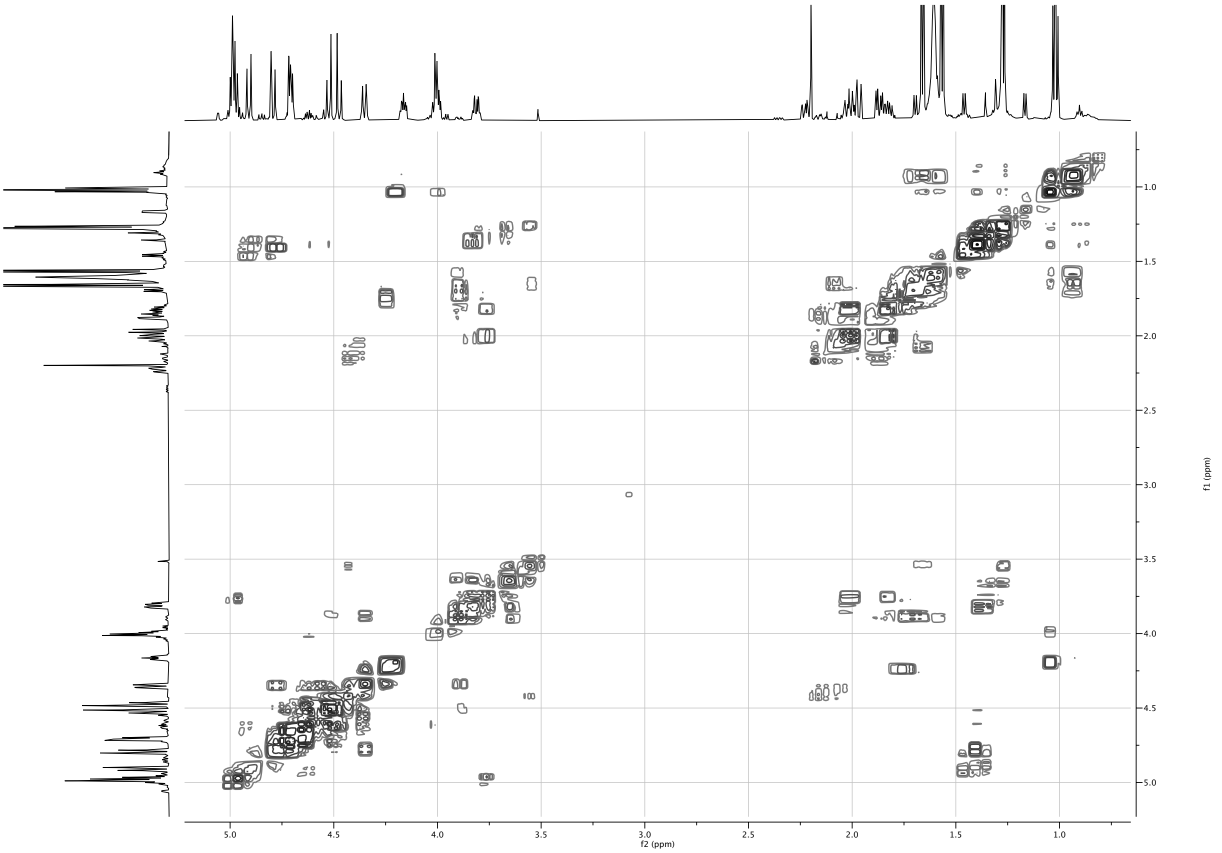

HSQC NMR, CDCl₃ of **S57**

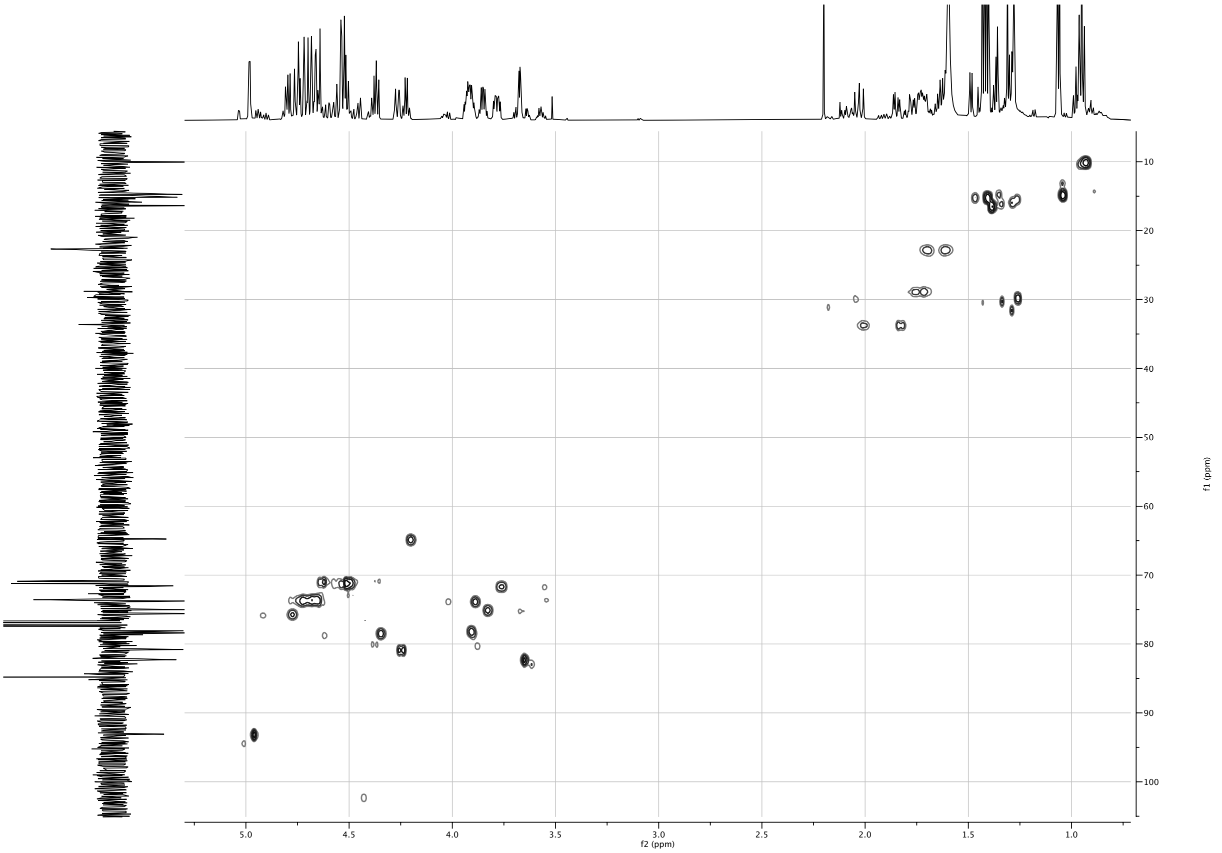

^{1}H NMR, 500 MHz, $CDCl_3$ of **S58**

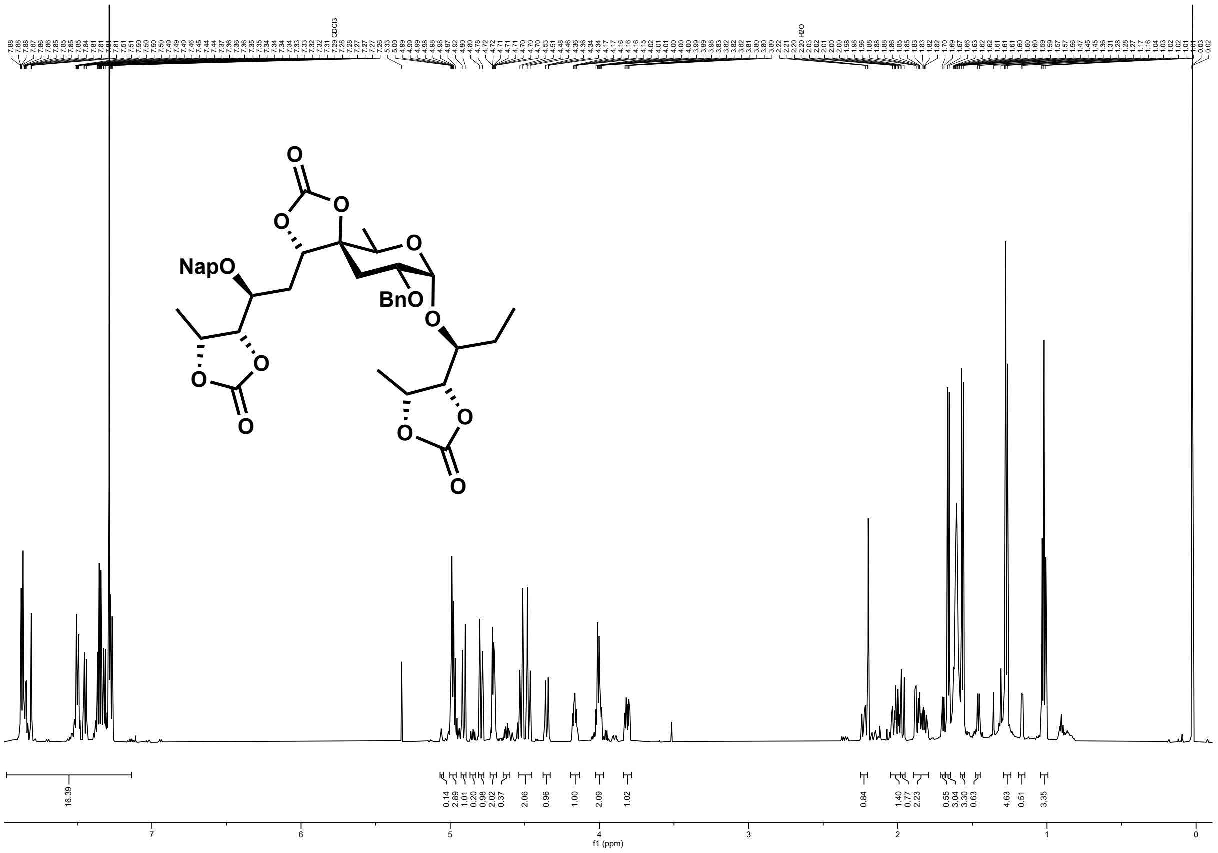

^{13}C NMR, 126 MHz, $CDCl_3$ of **S58**

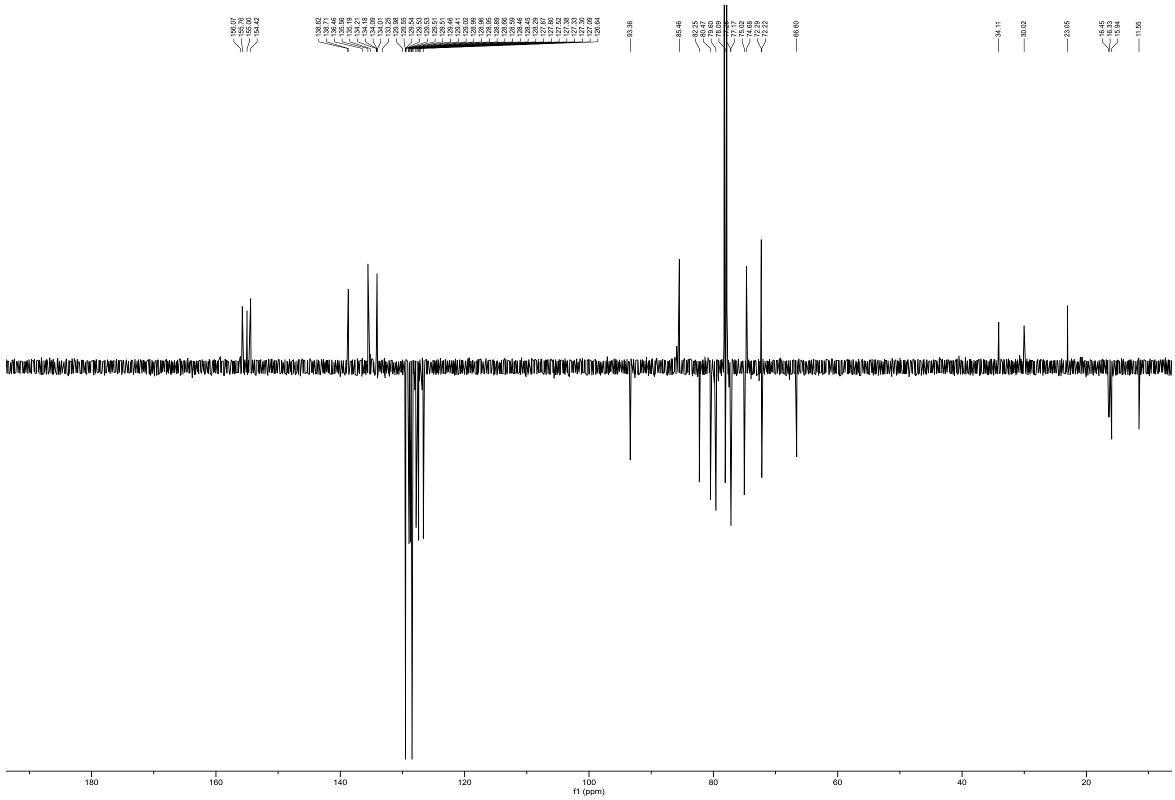

HH-COSY NMR, $CDCl_3$ of **S58**

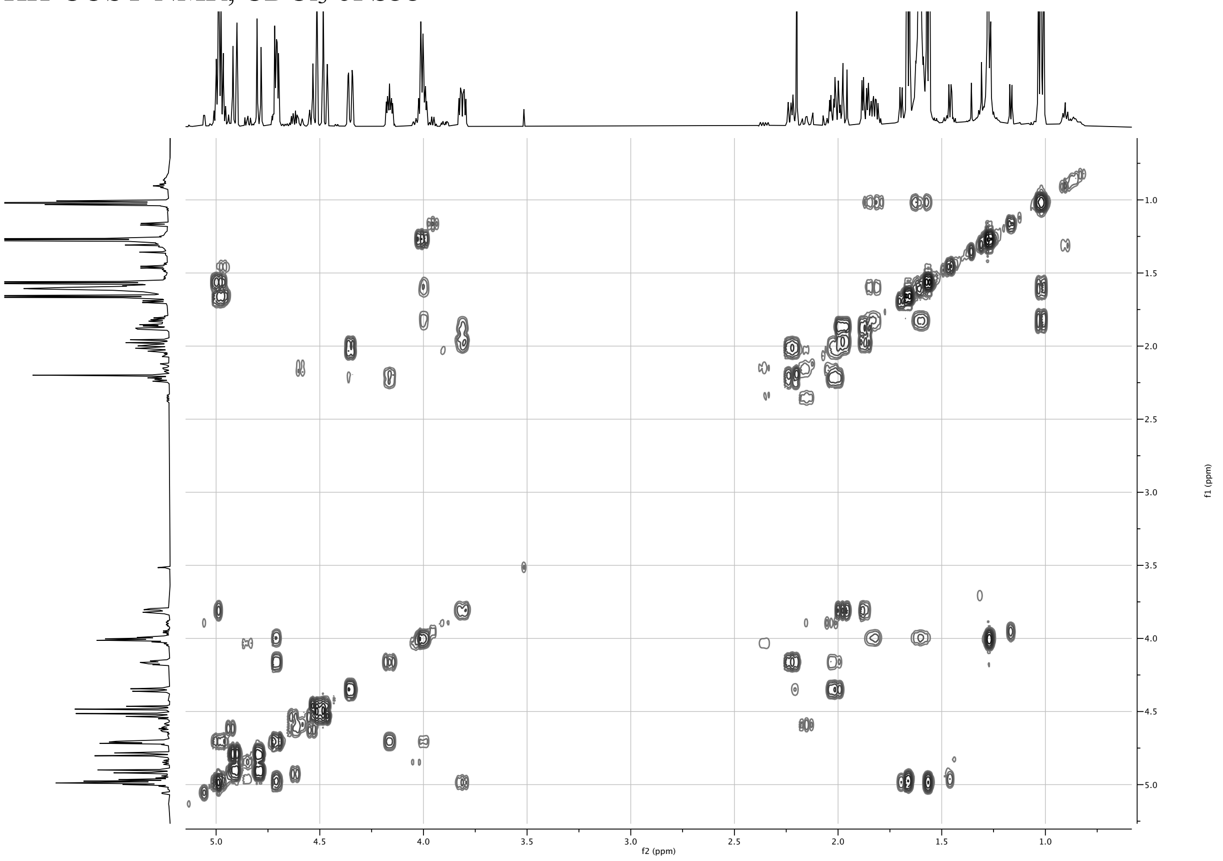

HSQC NMR, $CDCl_3$ of **S58**

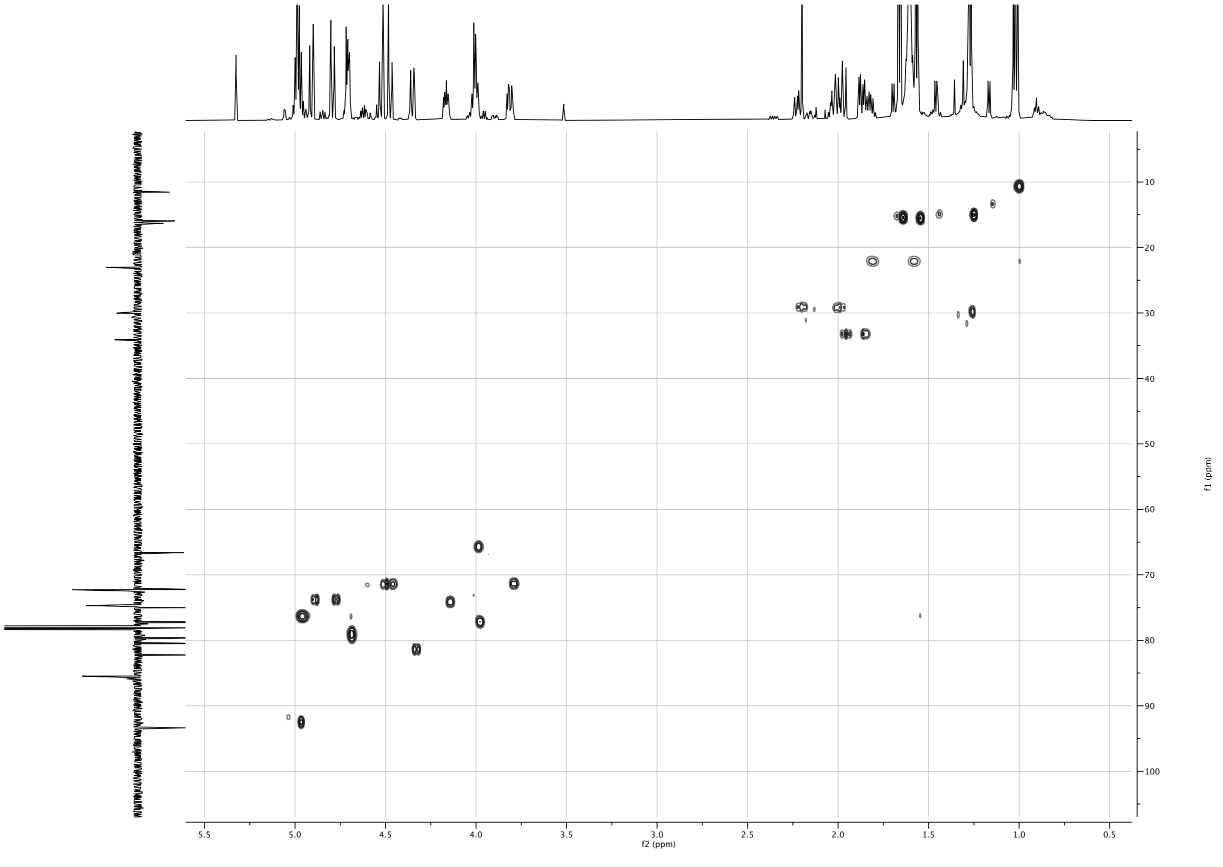

^{1}H NMR, 400 MHz, $CDCl_3$ of **S60**

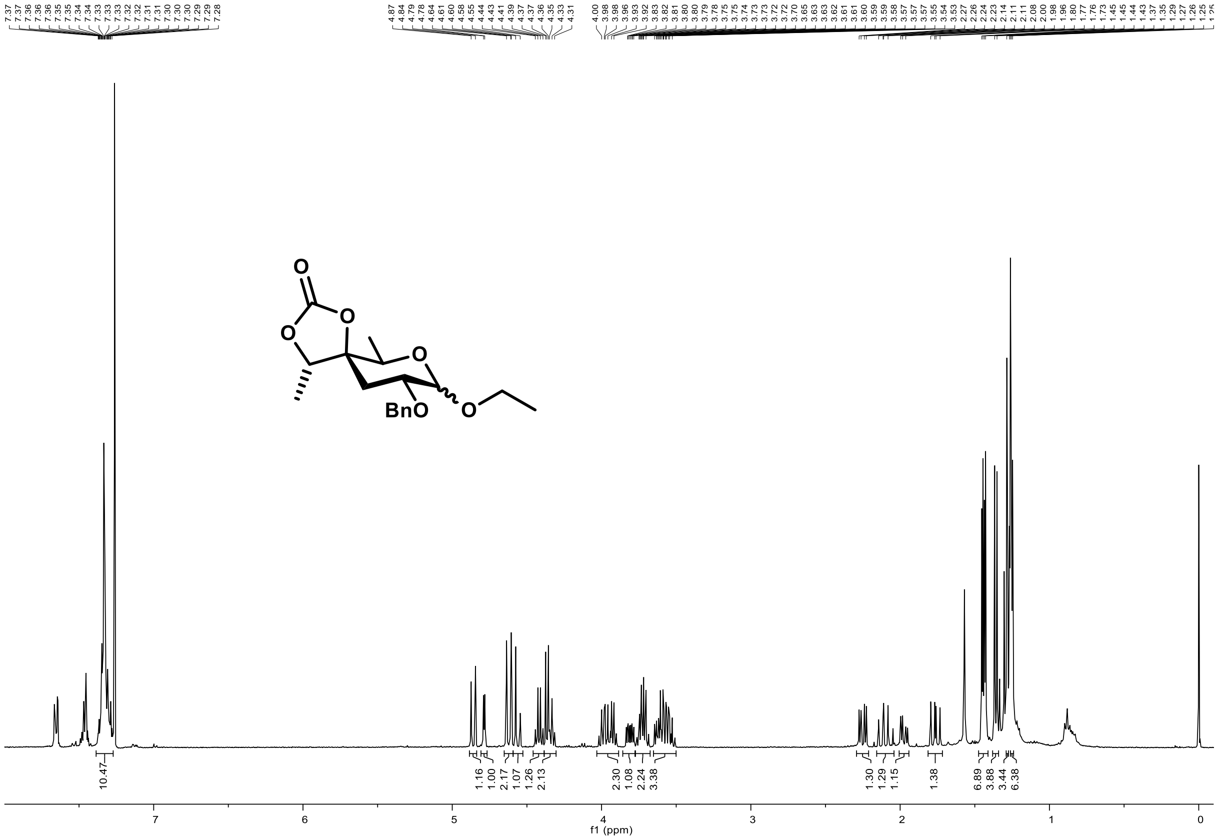

^{13}C NMR, 101 MHz, $CDCl_3$ of **S60**

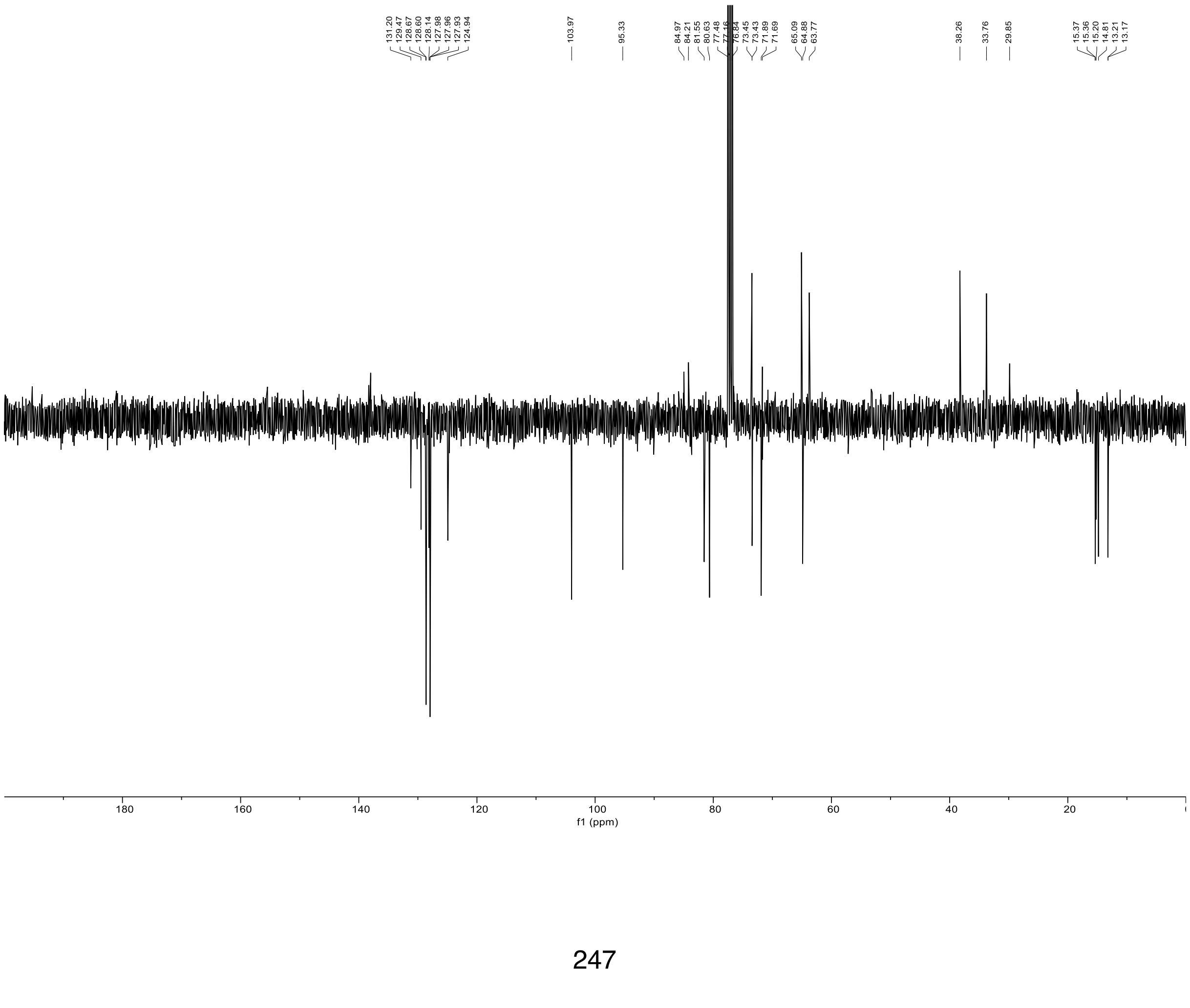

HH-COSY NMR, CDCl$_3$ of **S60**

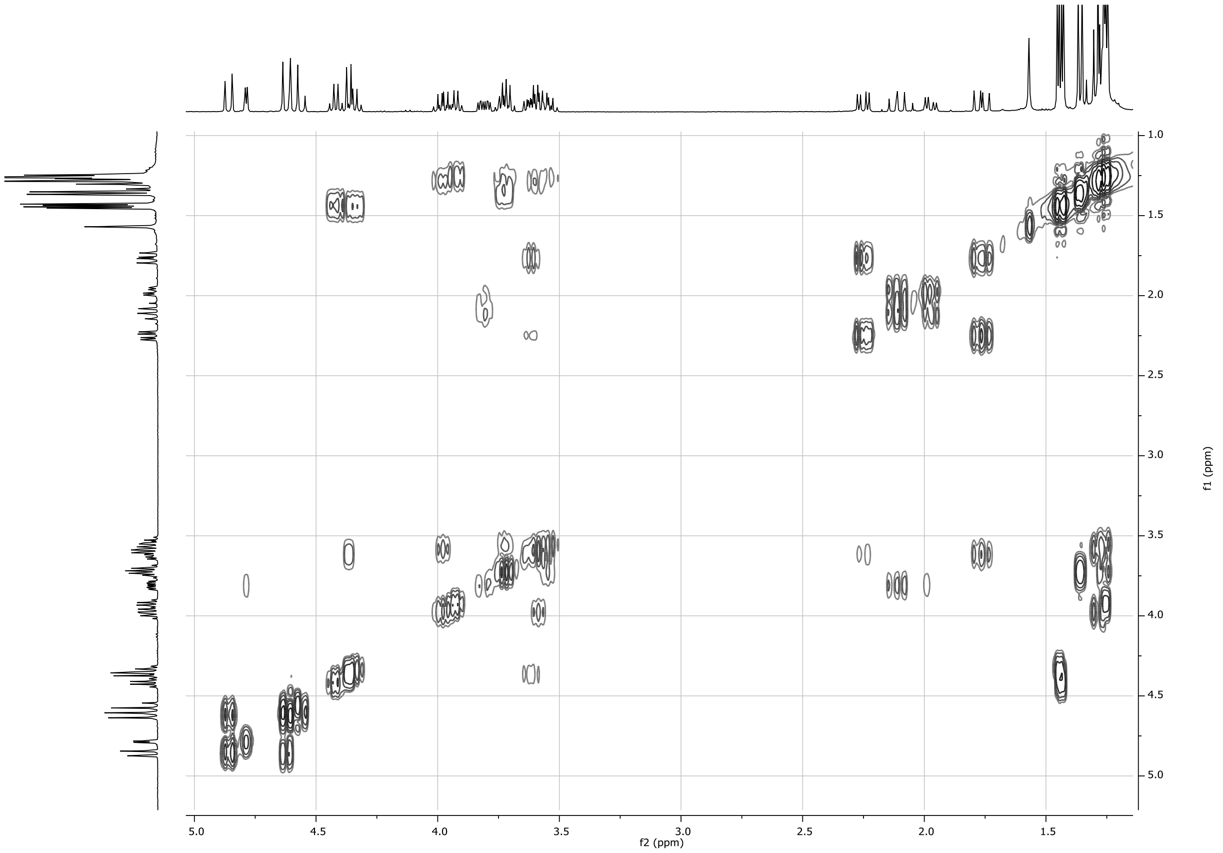

HSQC NMR, CDCl$_3$ of S**60**

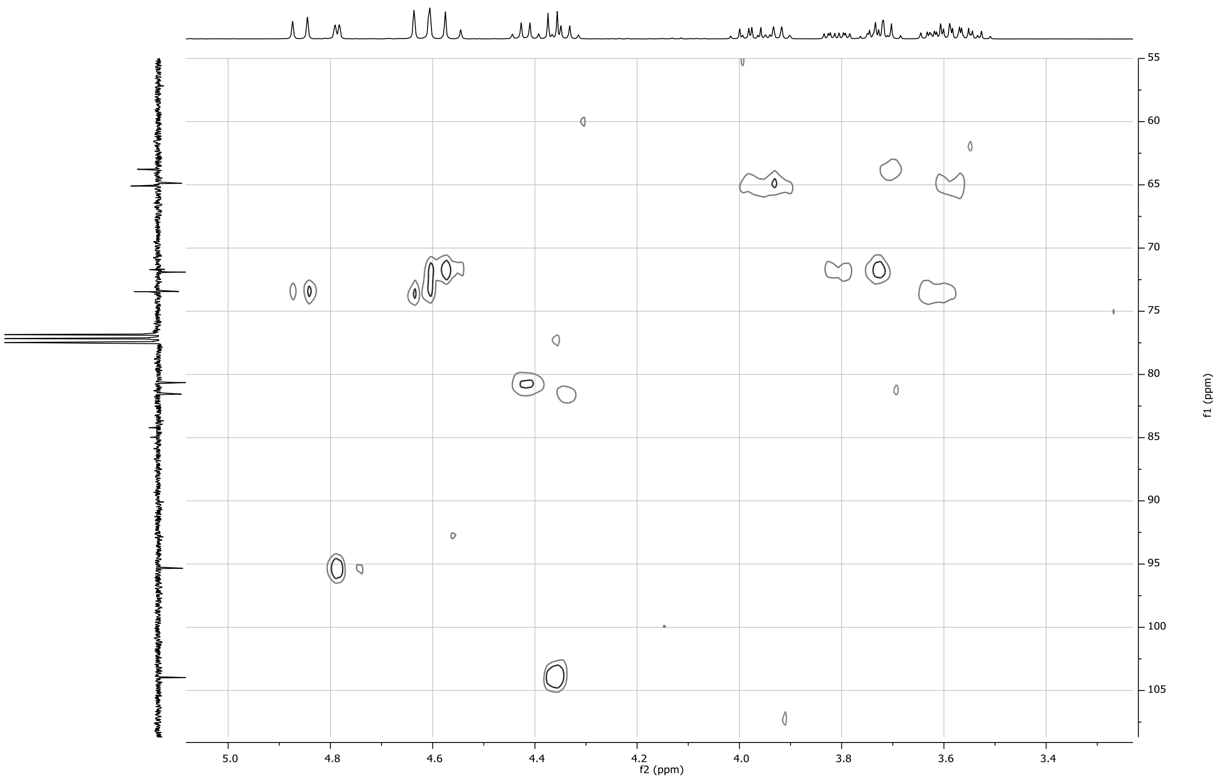

^{1}H NMR, 400 MHz, $CDCl_3$ of **S61**

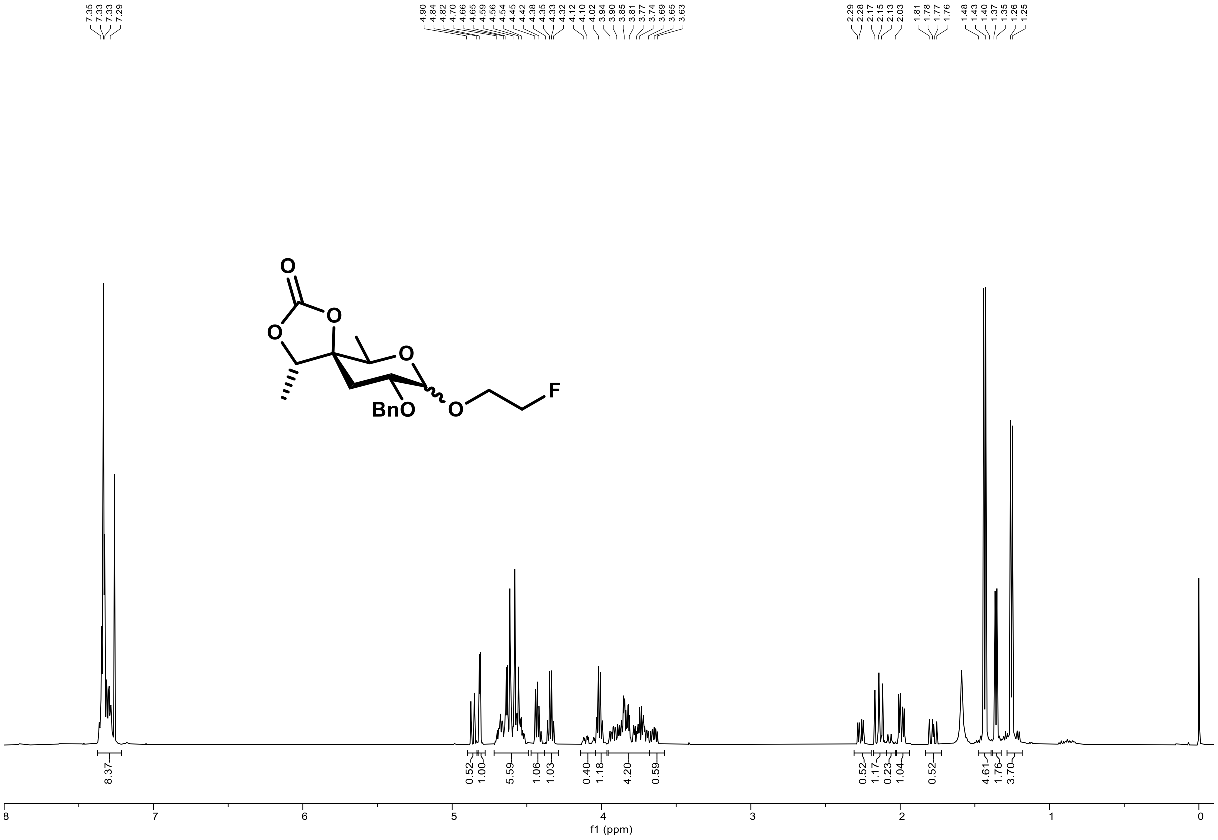

^{13}C NMR, 101 MHz, $CDCl_3$ of **S61**

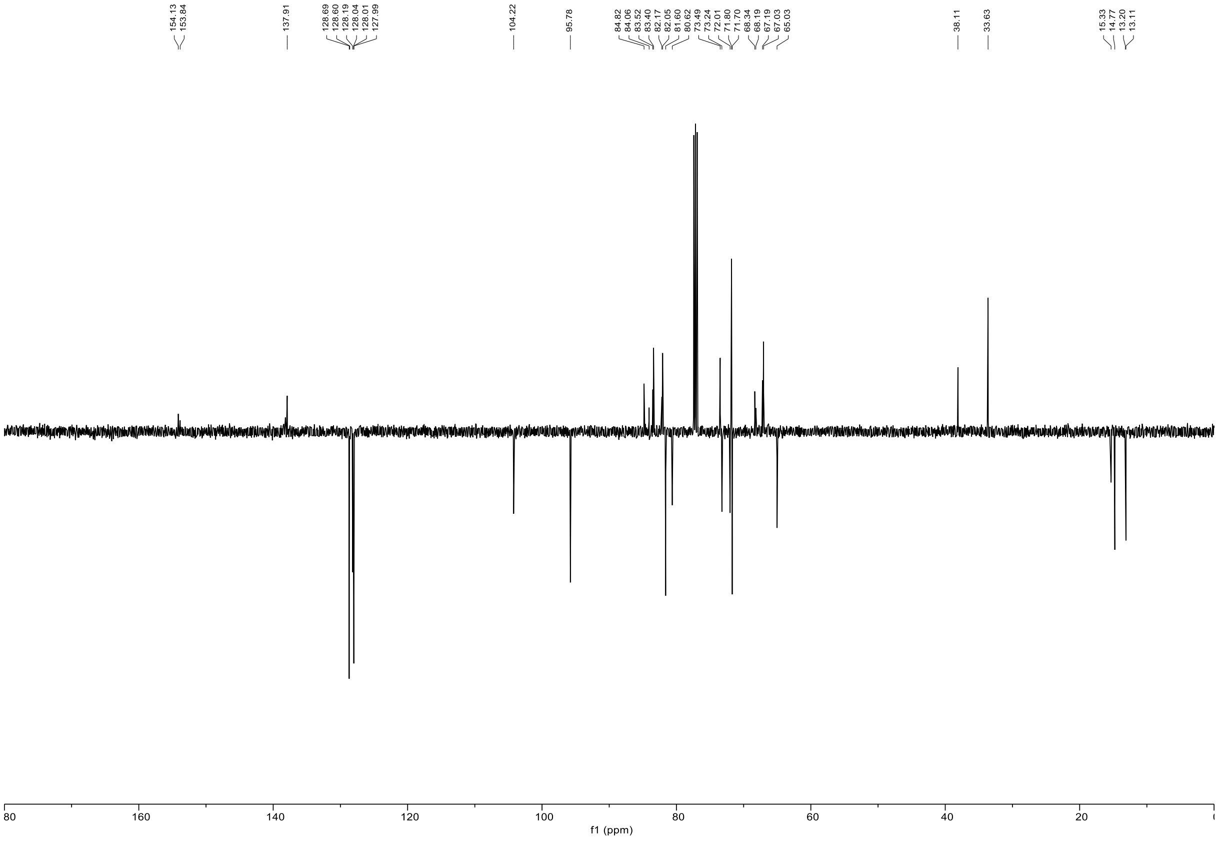

HH-COSY NMR, $CDCl_3$ of **S61**

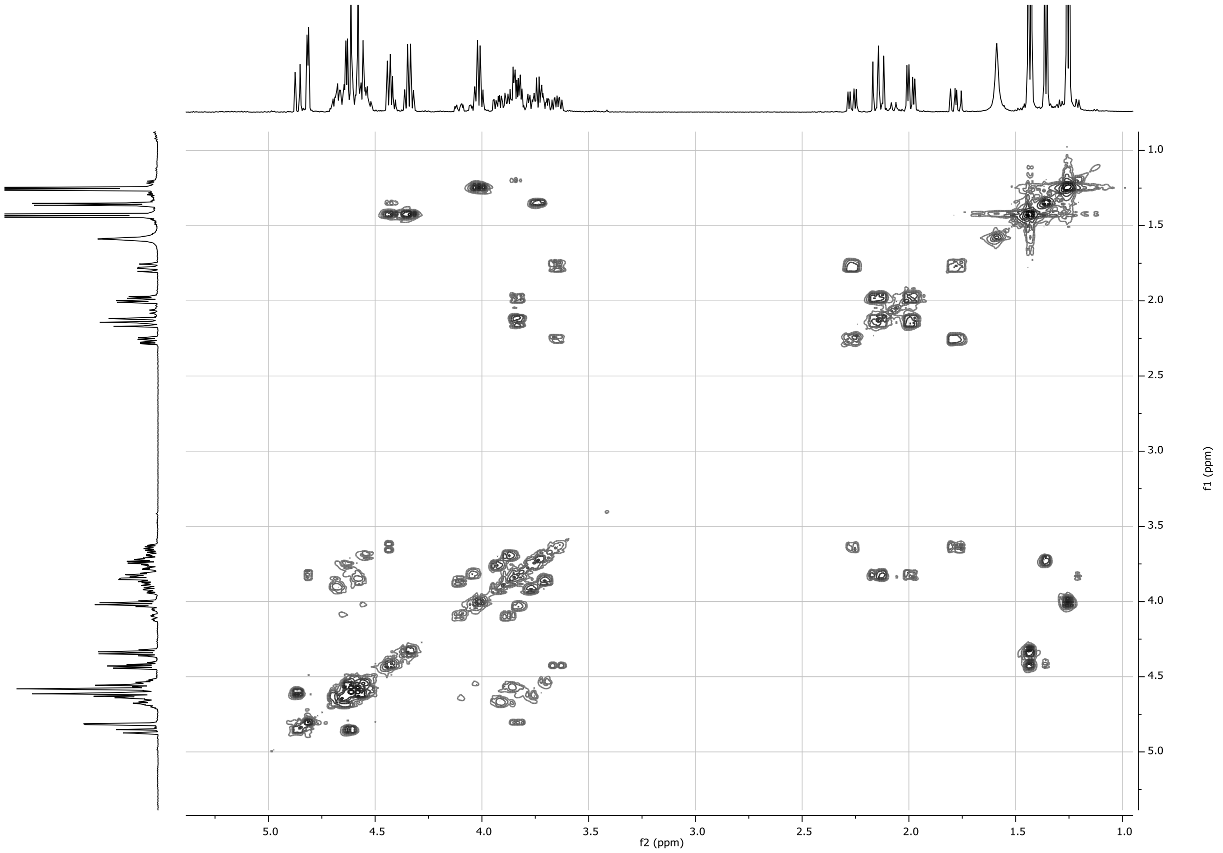

HSQC NMR, $CDCl_3$ of **S61**

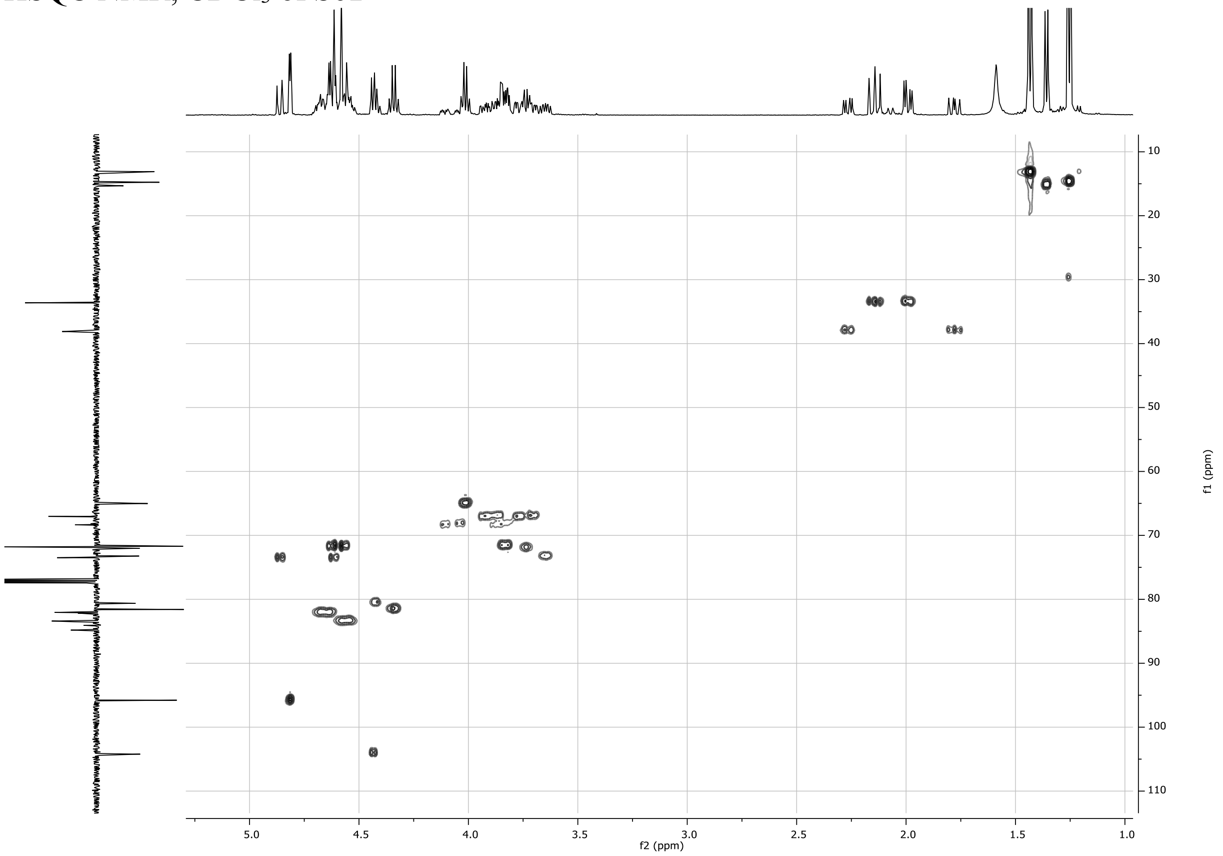

^{1}H NMR, 400 MHz, $CDCl_3$ of **S62**

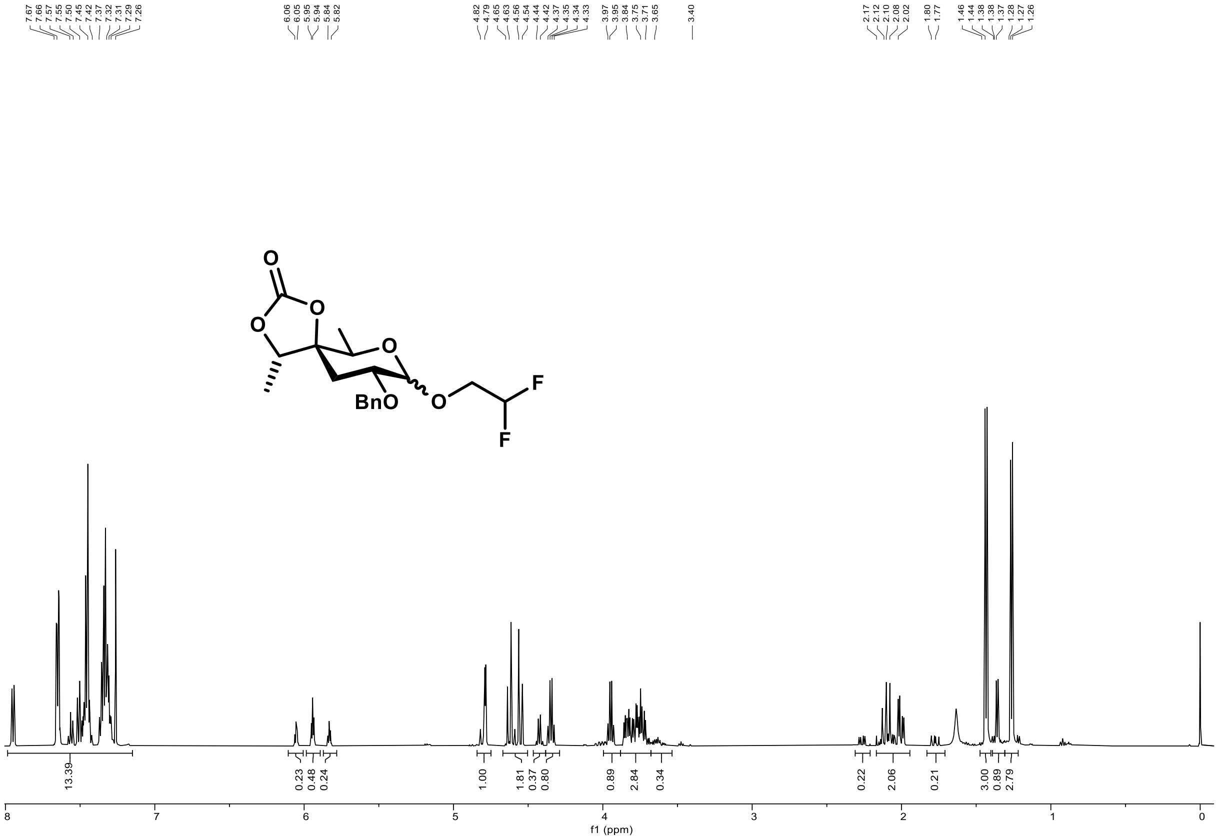

^{13}C NMR, 101 MHz, $CDCl_3$ of **S62**

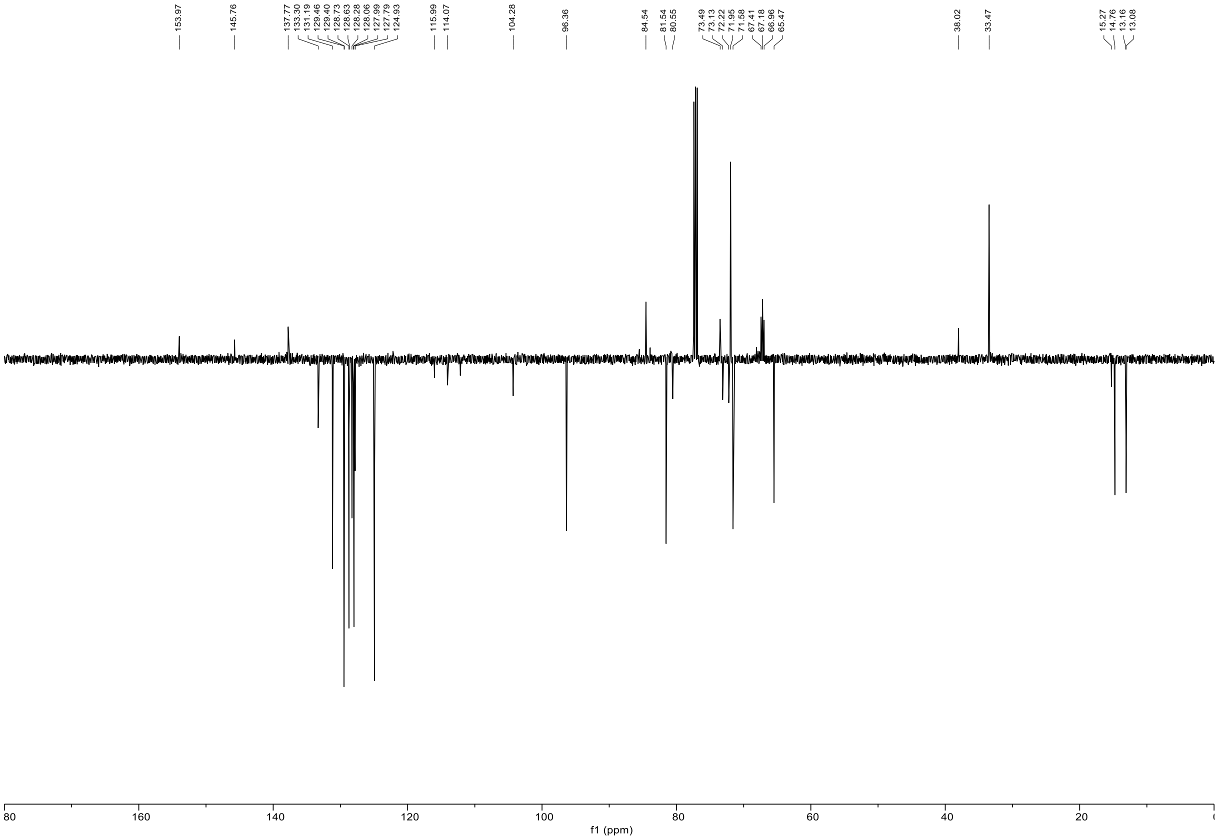

HH-COSY NMR, $CDCl_3$ of **S62**

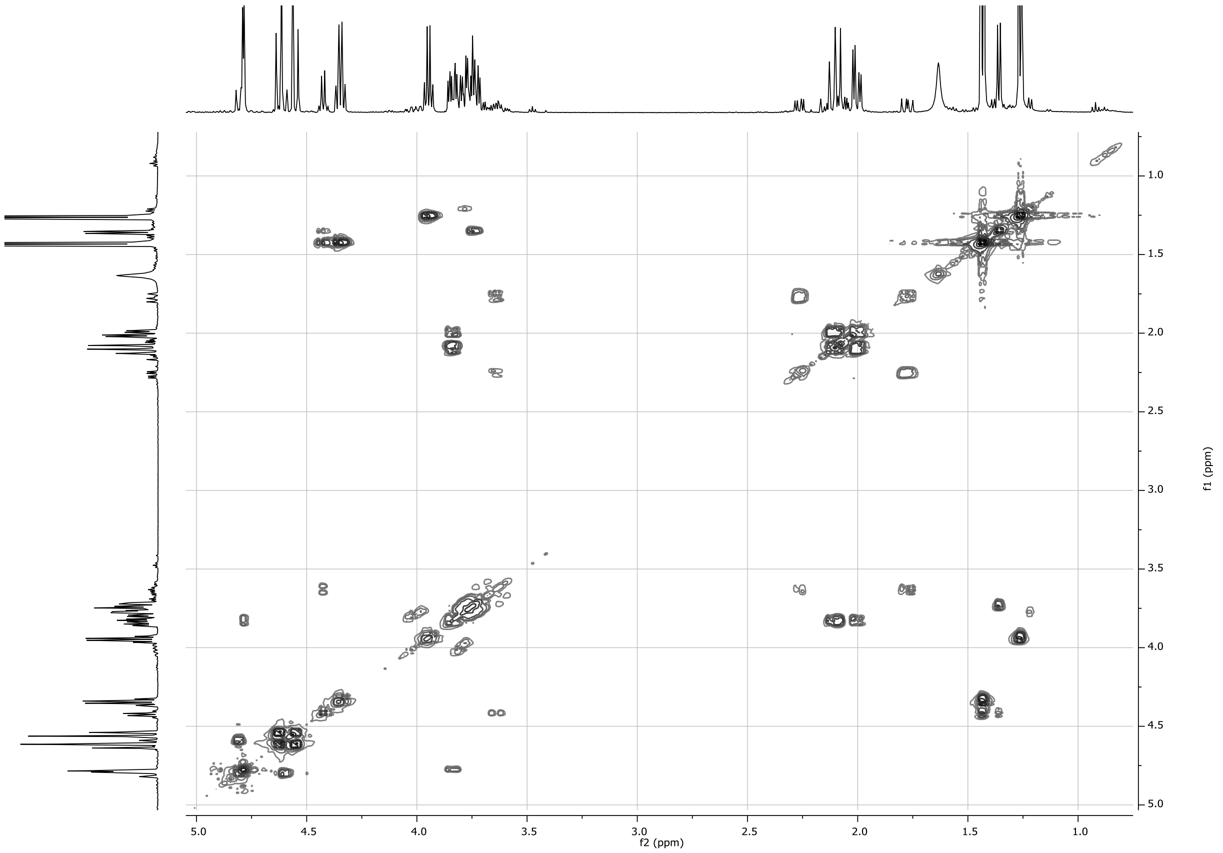

HSQC NMR, $CDCl_3$ of **S62**

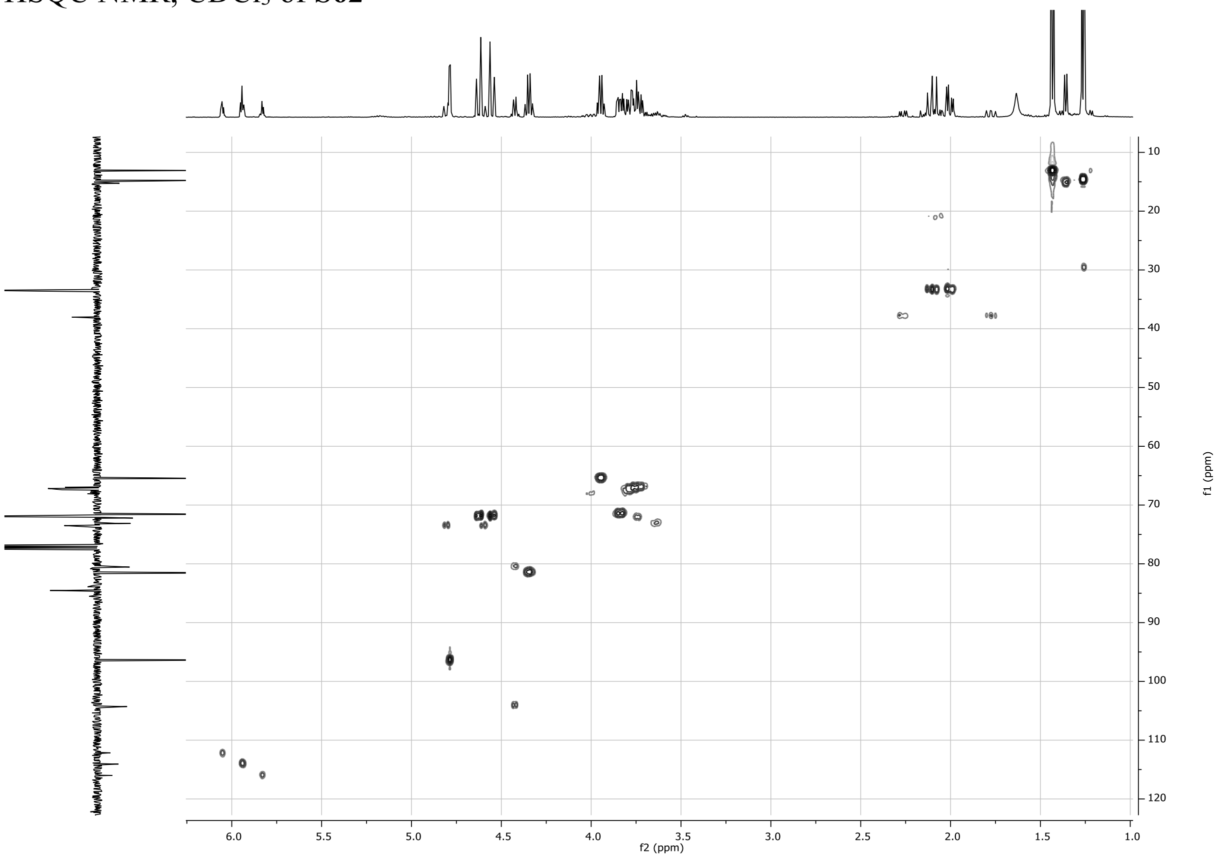

^{1}H NMR, 400 MHz, $CDCl_3$ of **S63**

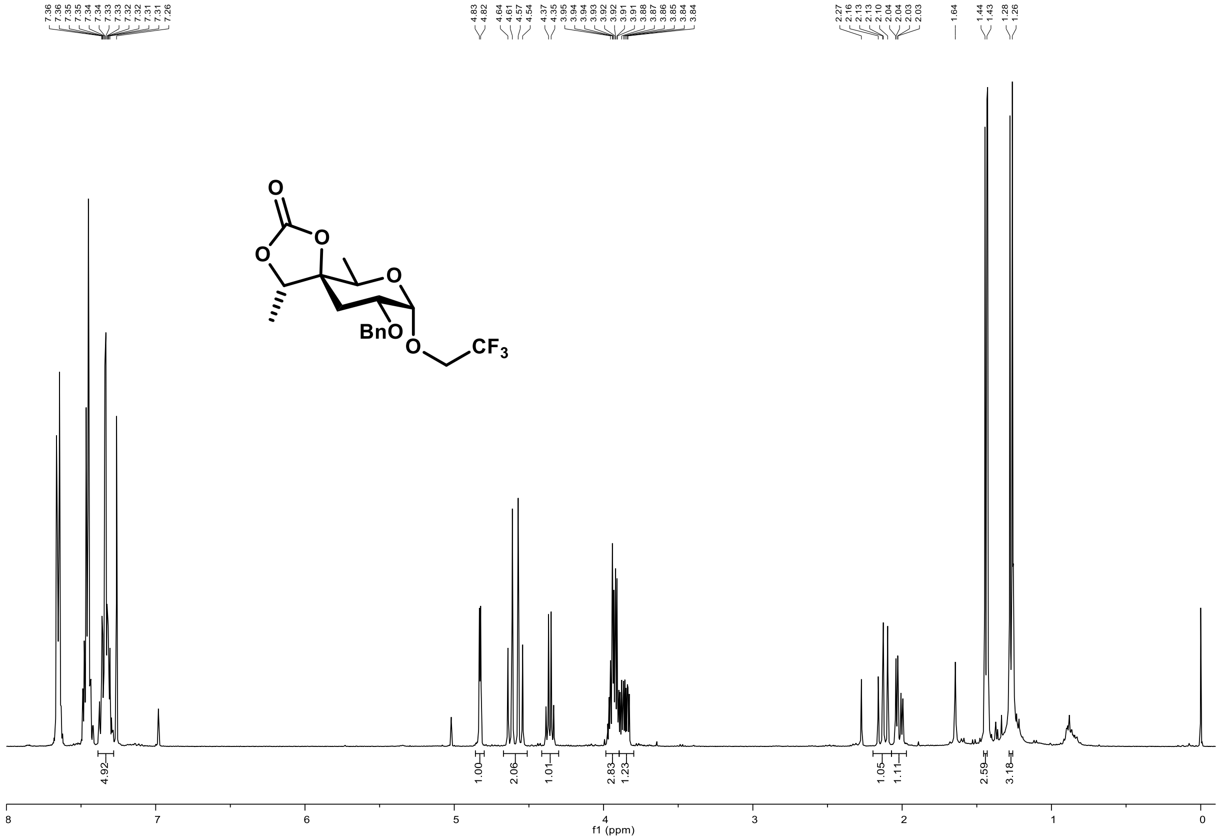

^{13}C NMR, 101 MHz, $CDCl_3$ of **S63**

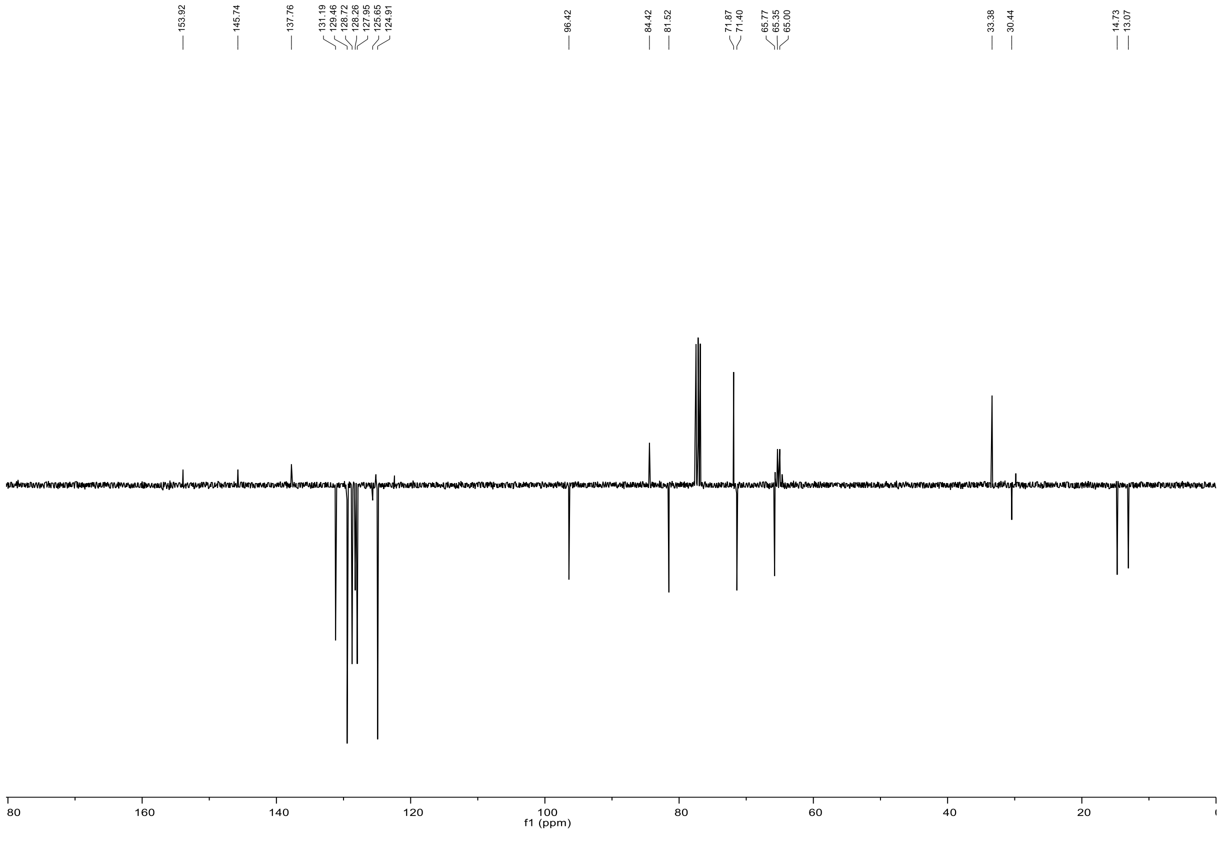

HH-COSY NMR, $CDCl_3$ of **S63**

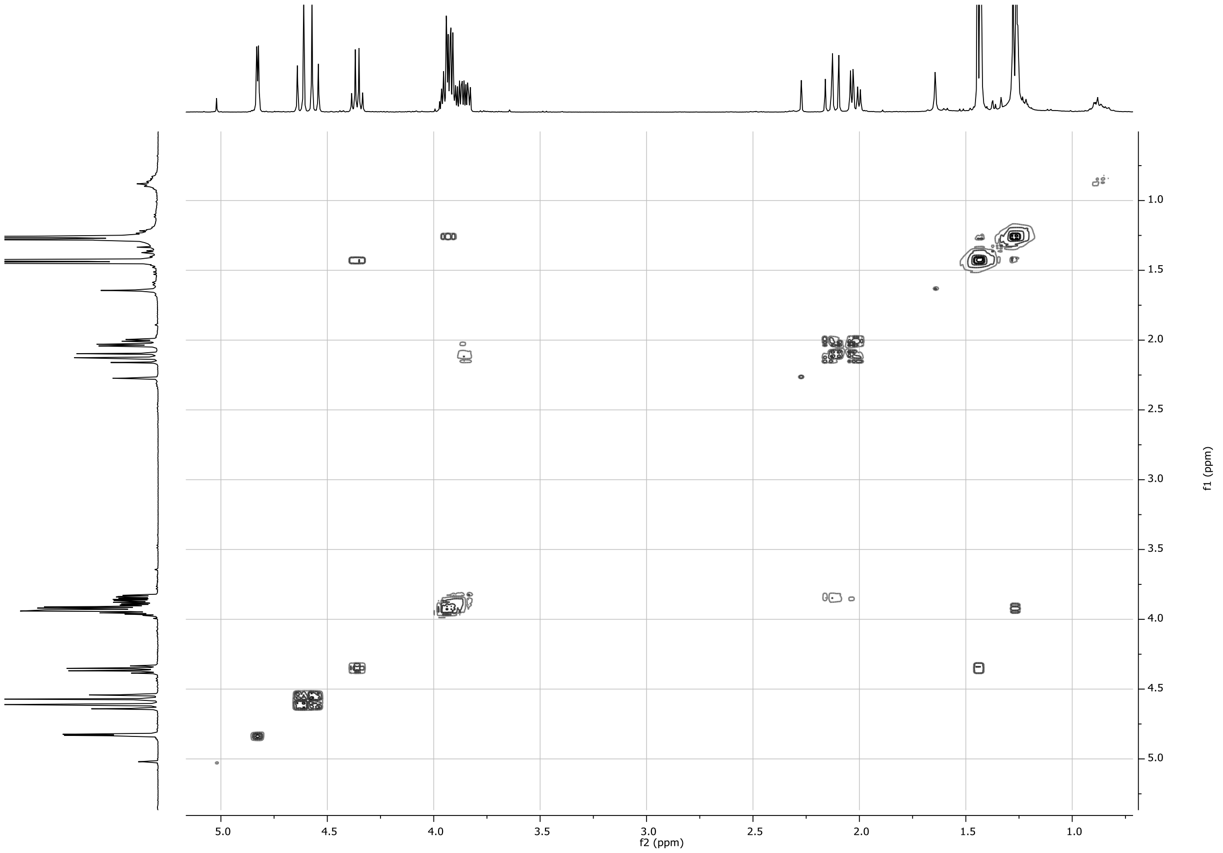

HSQC NMR, $CDCl_3$ of **S63**

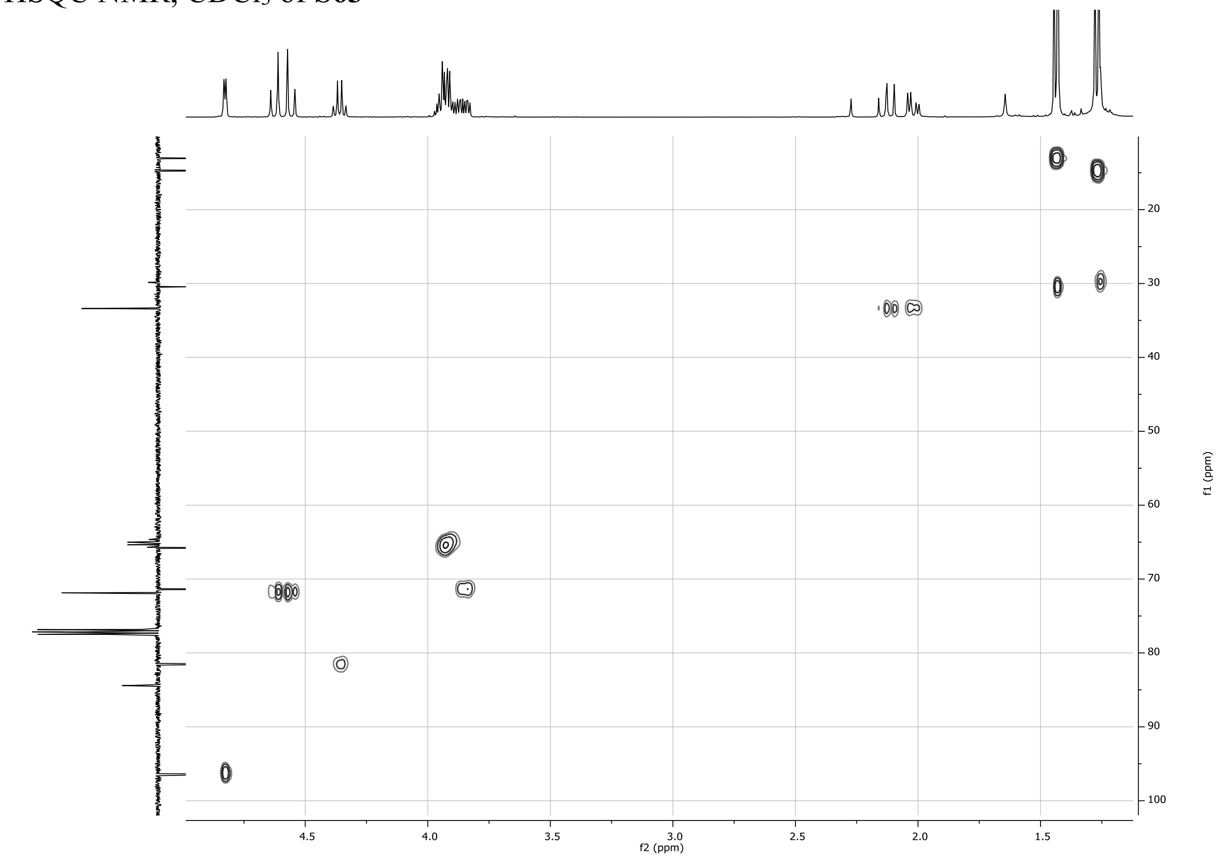

^{1}H NMR, 500 MHz, $CDCl_3$ of **S59**

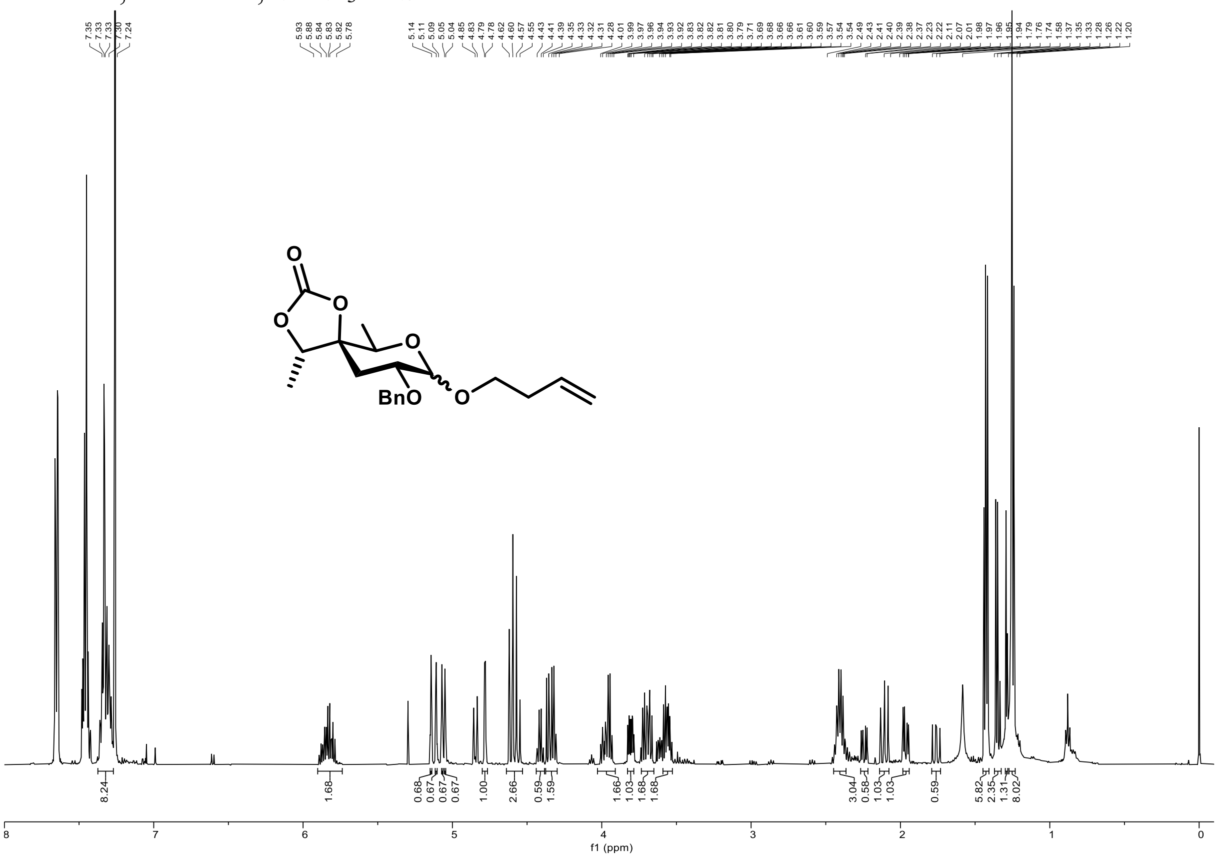

^{13}C NMR, 125 MHz, $CDCl_3$ of **S59**

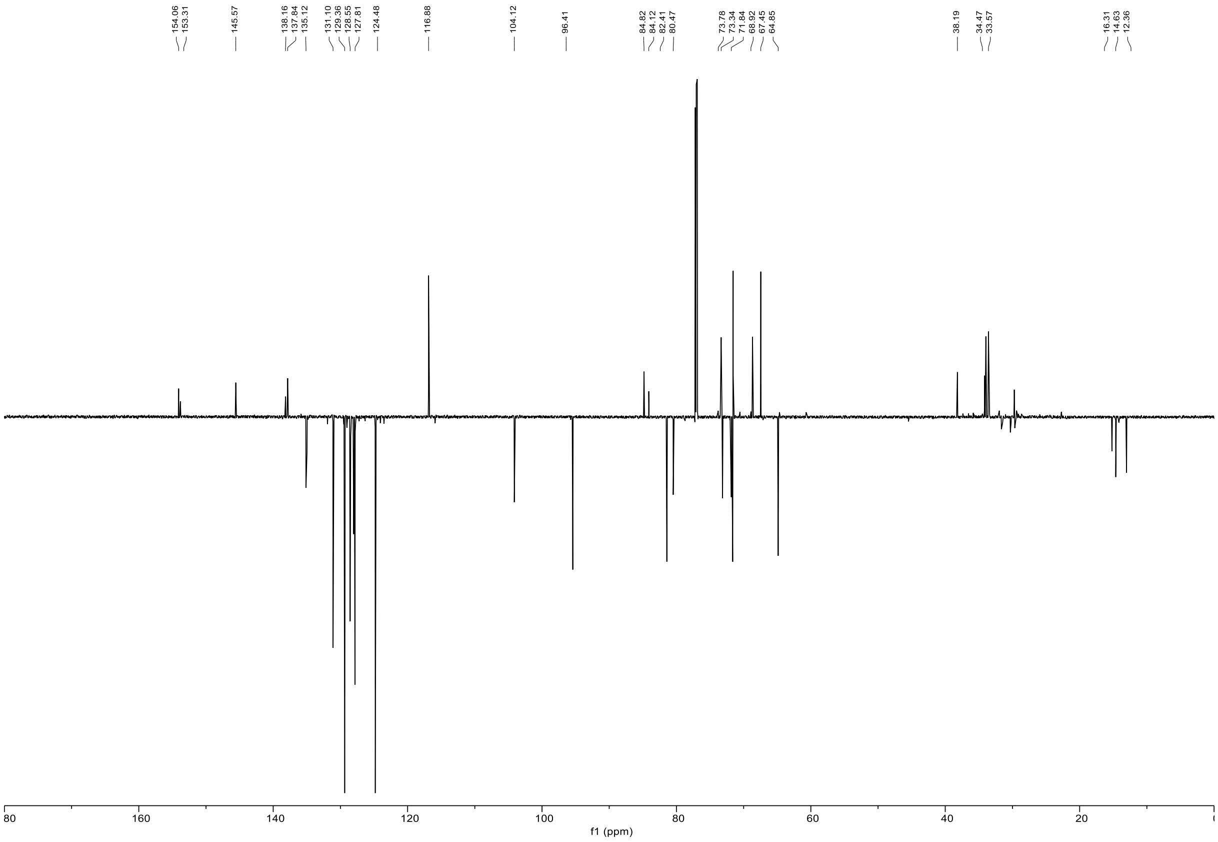

HH-COSY NMR, $CDCl_3$ of **S59**

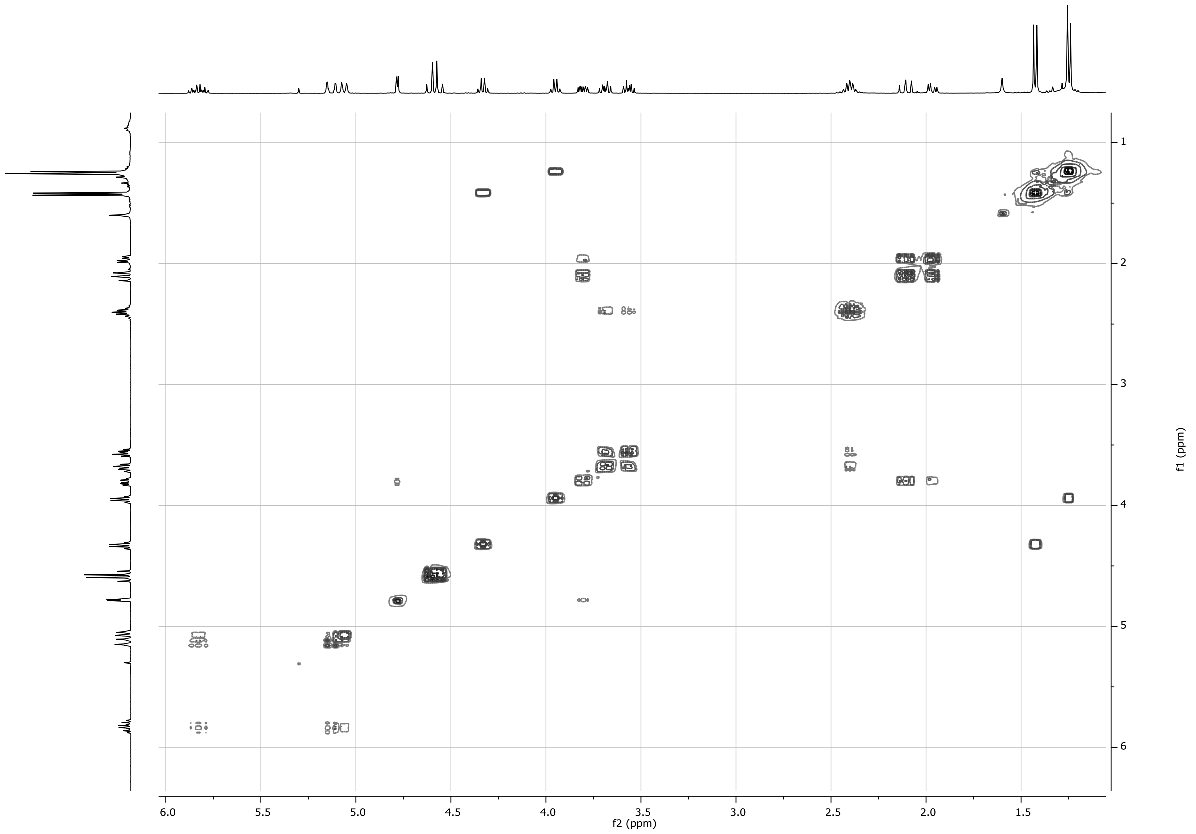

HSQC NMR, $CDCl_3$ of **S59**

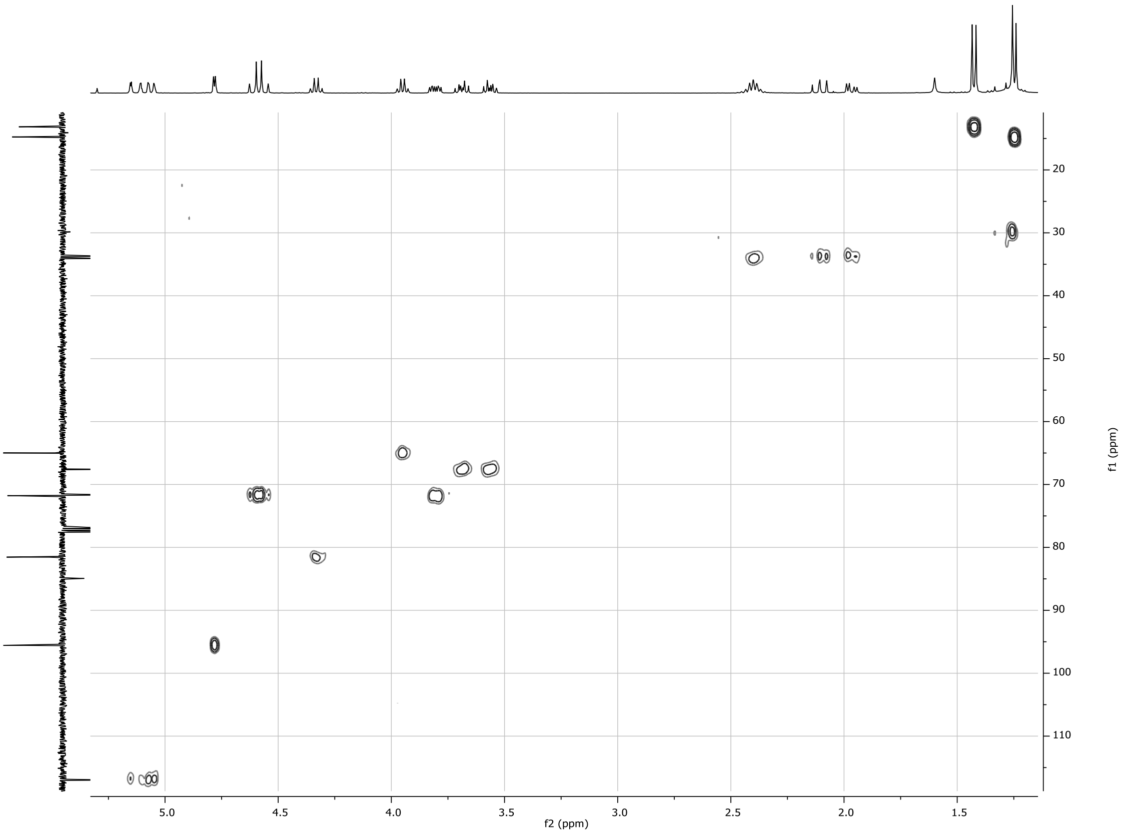

^{1}H NMR, 400 MHz, $CDCl_3$ of **S64**

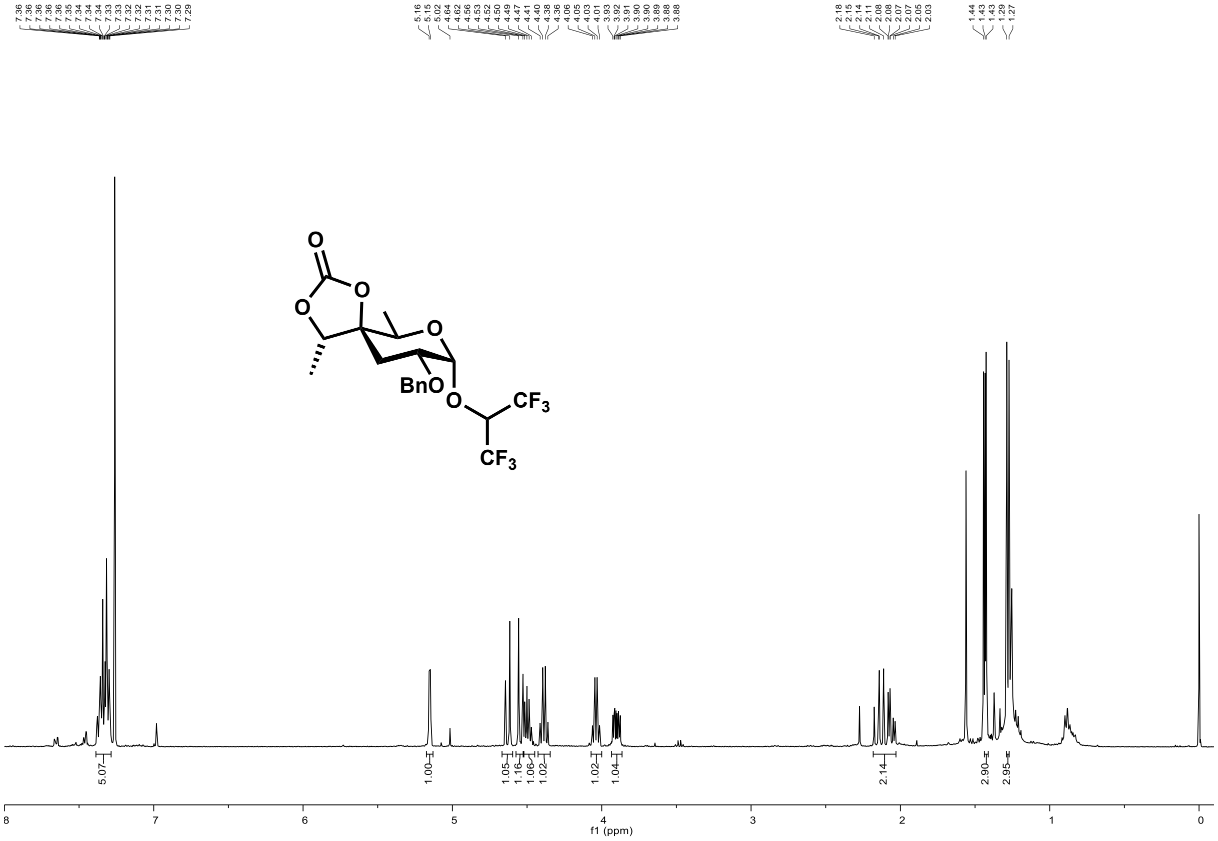

^{13}C NMR, 101 MHz, $CDCl_3$ of **S64**

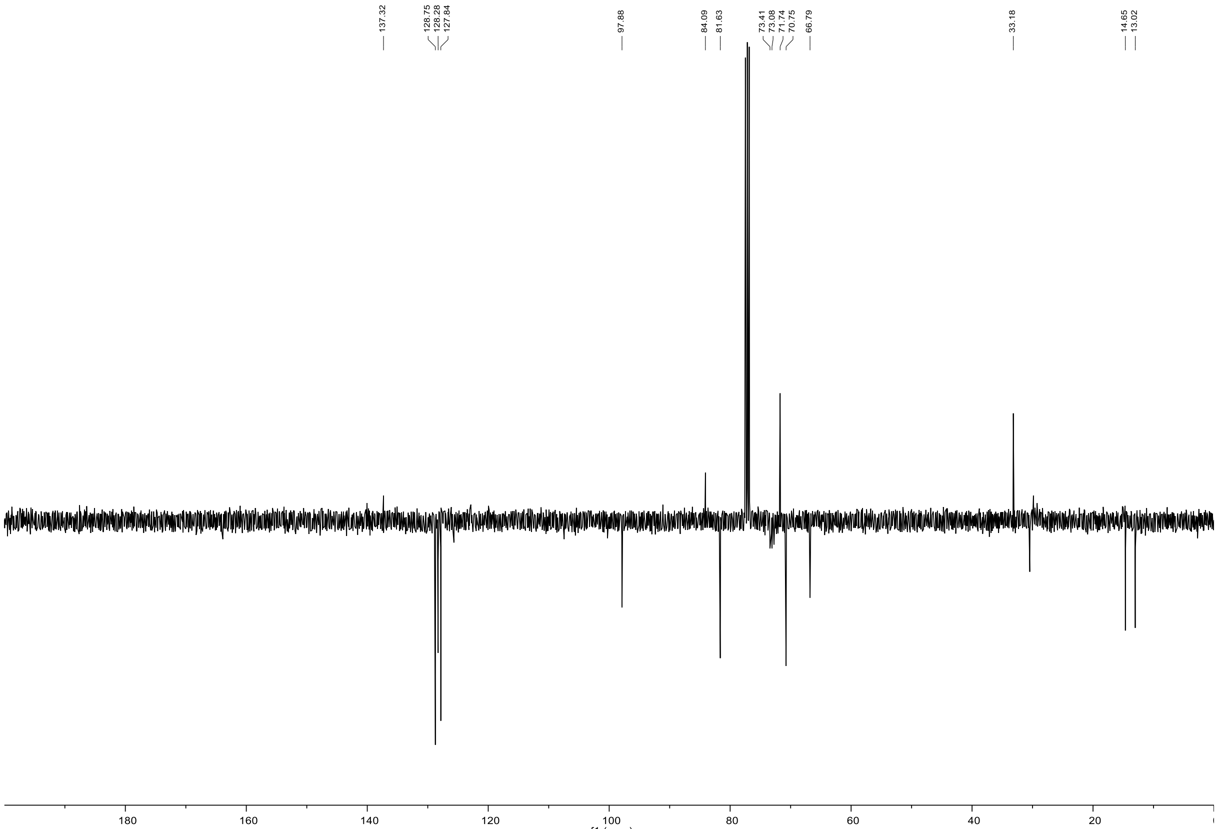

HH-COSY NMR, CDCl$_3$ of **S64**

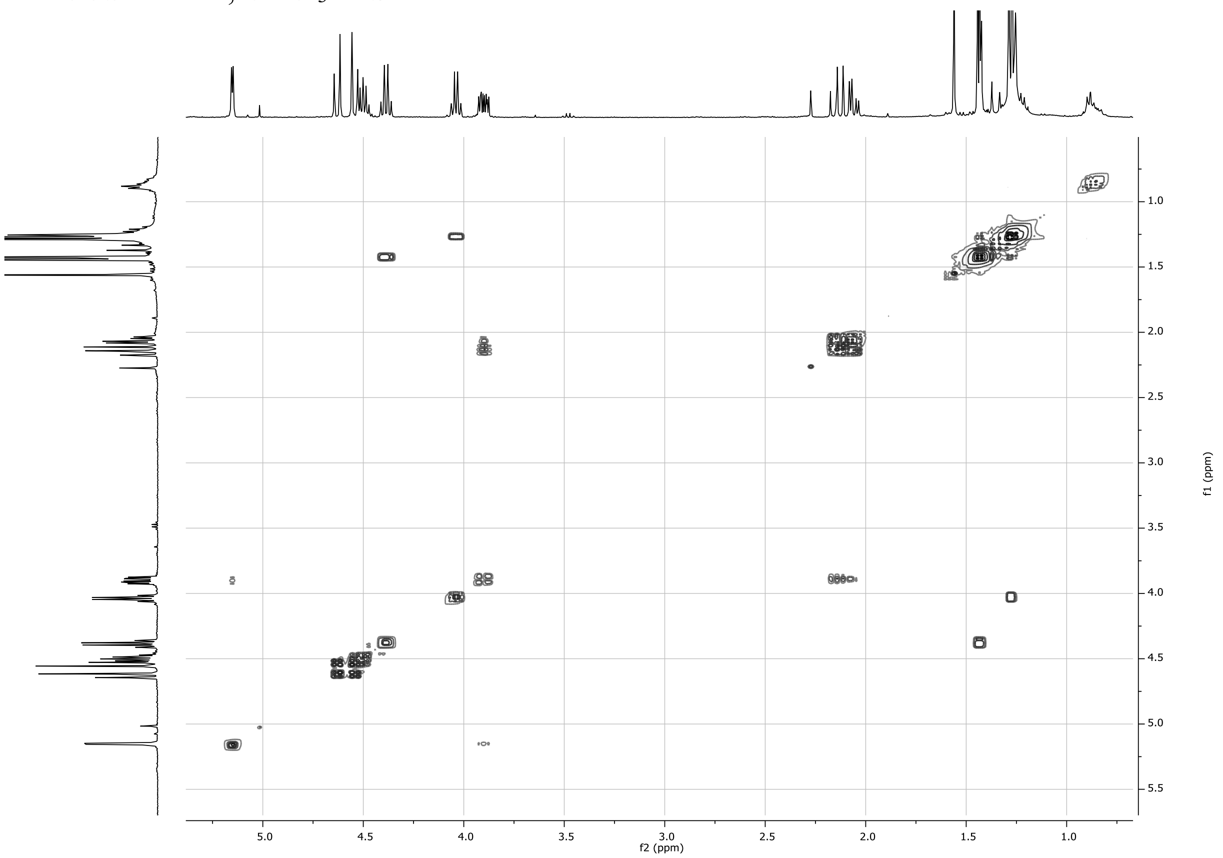

HSQC NMR, CDCl$_3$ of **S64**

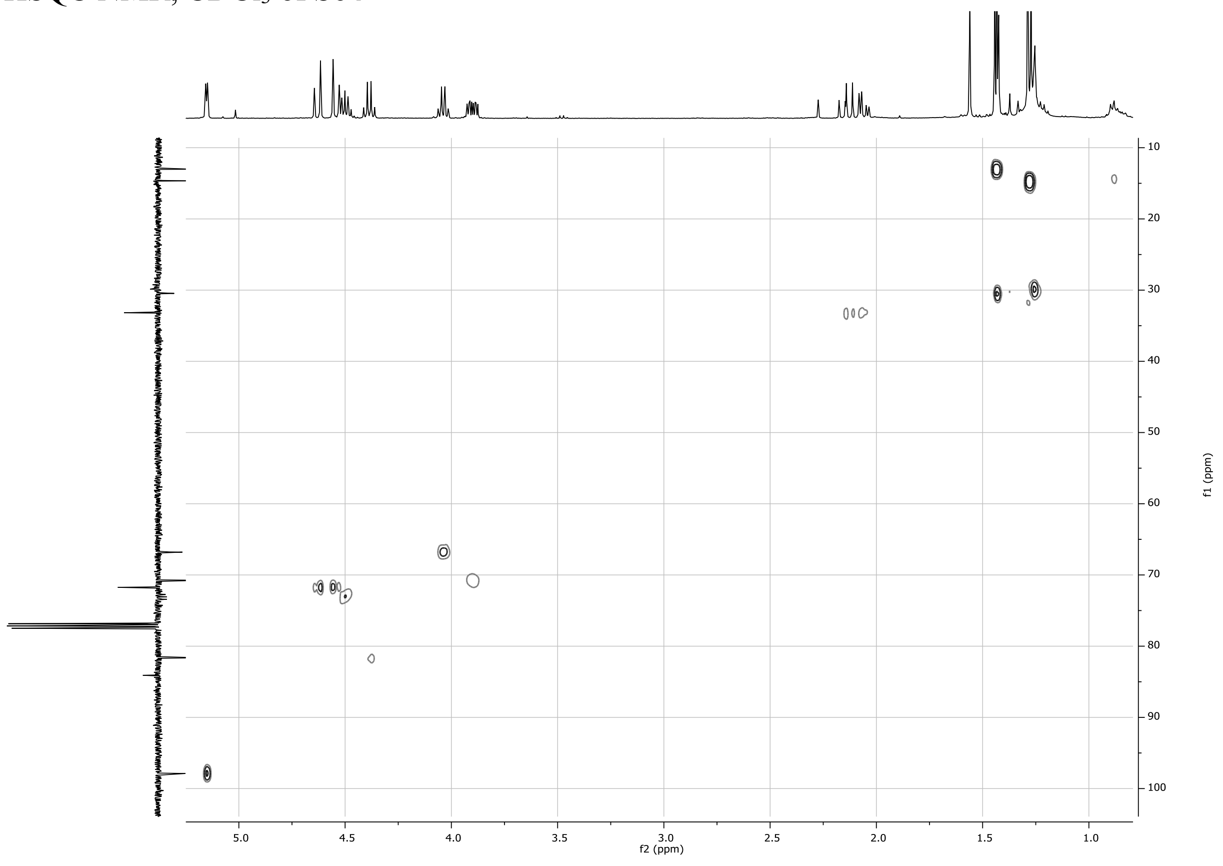

^{1}H NMR, 400 MHz, $CDCl_3$ of **S65**

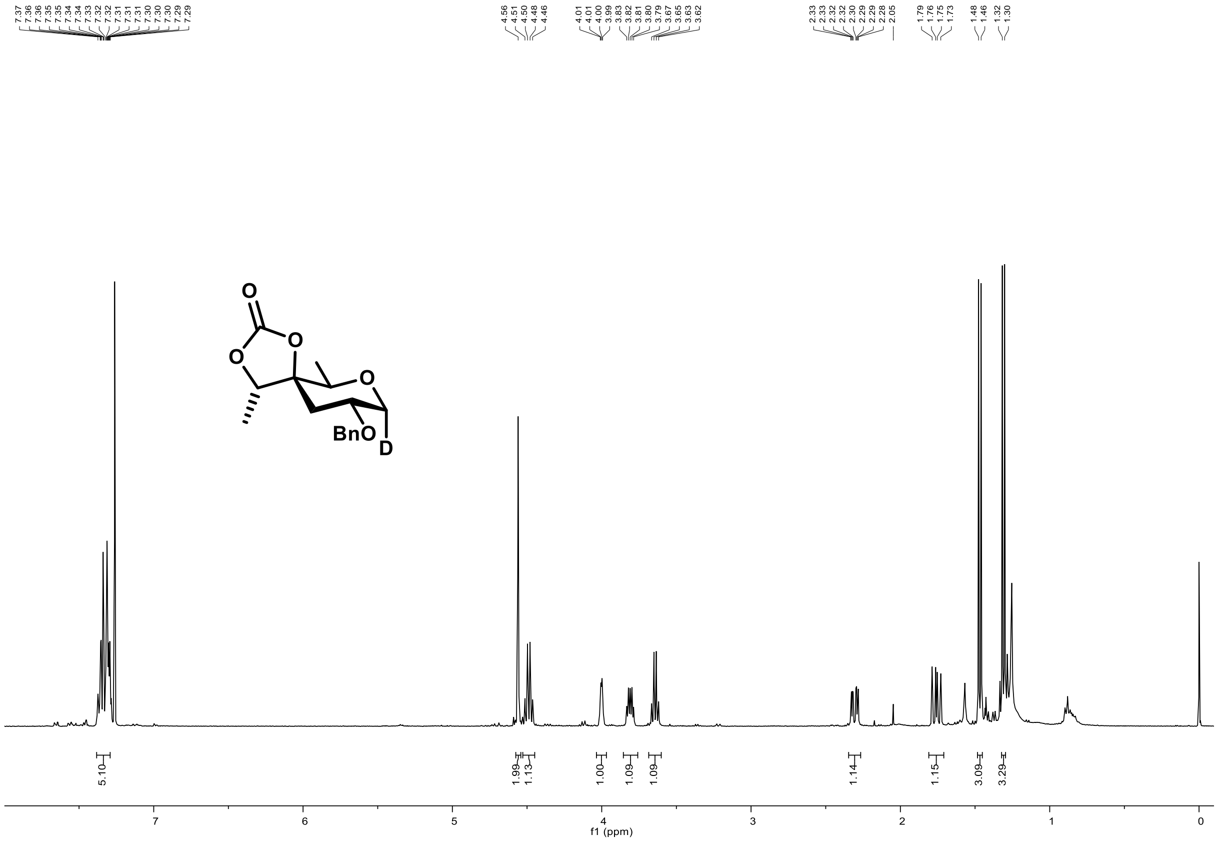

^{13}C NMR, 101 MHz, $CDCl_3$ of **S65**

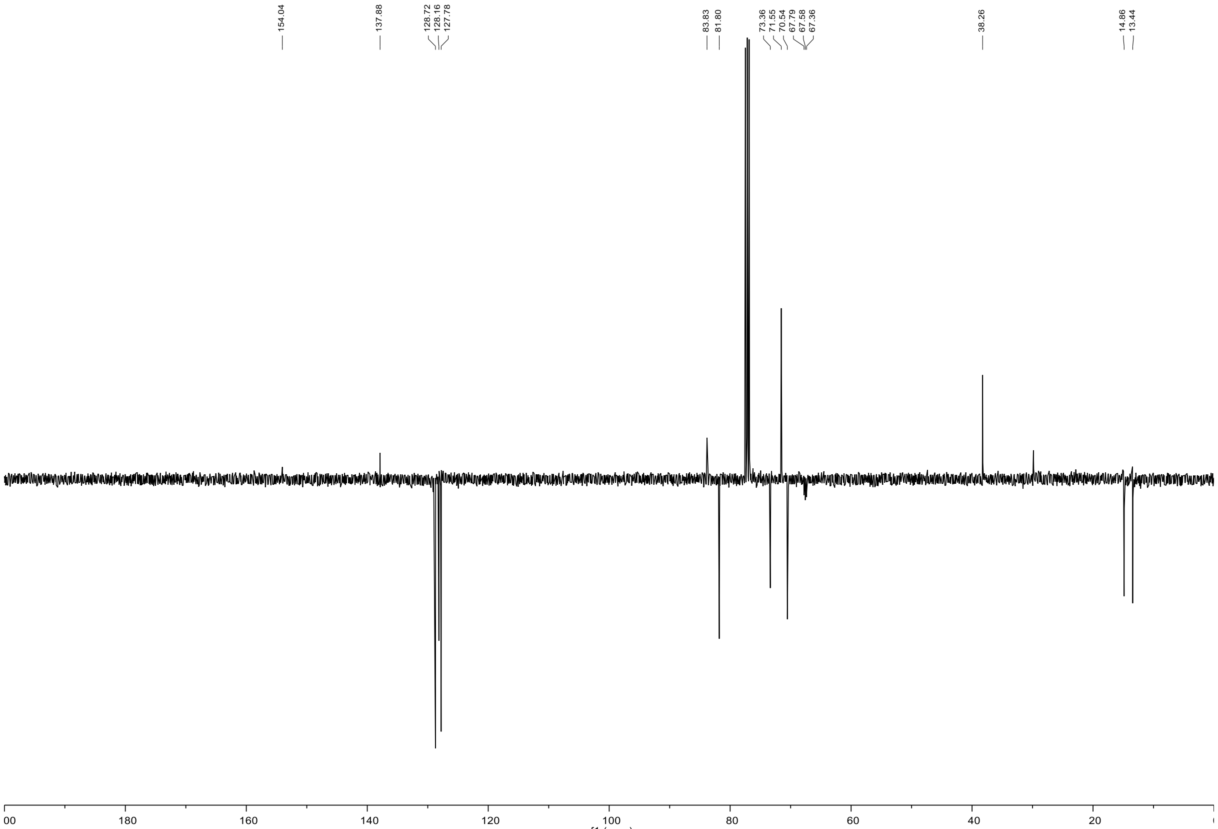

HH-COSY NMR, $CDCl_3$ of **S65**

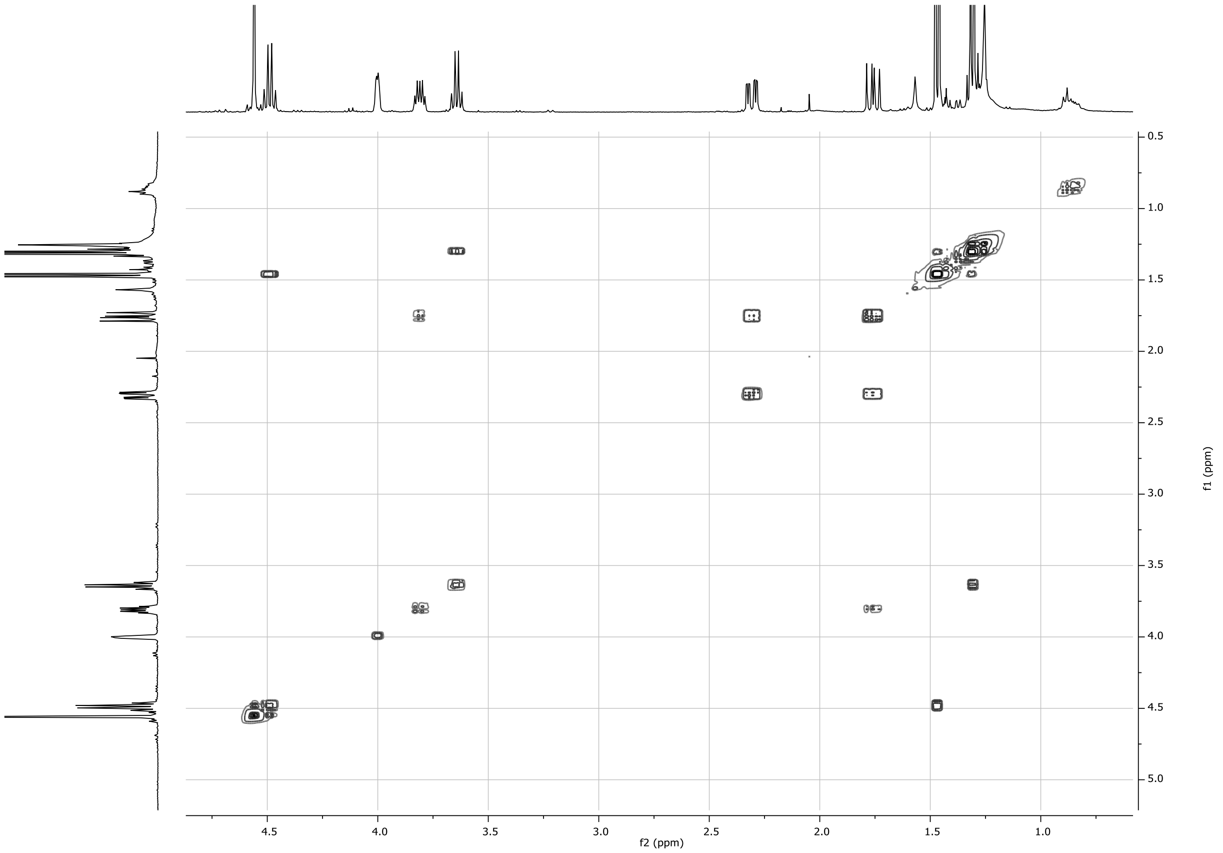

HSQC NMR, $CDCl_3$ of **S65**

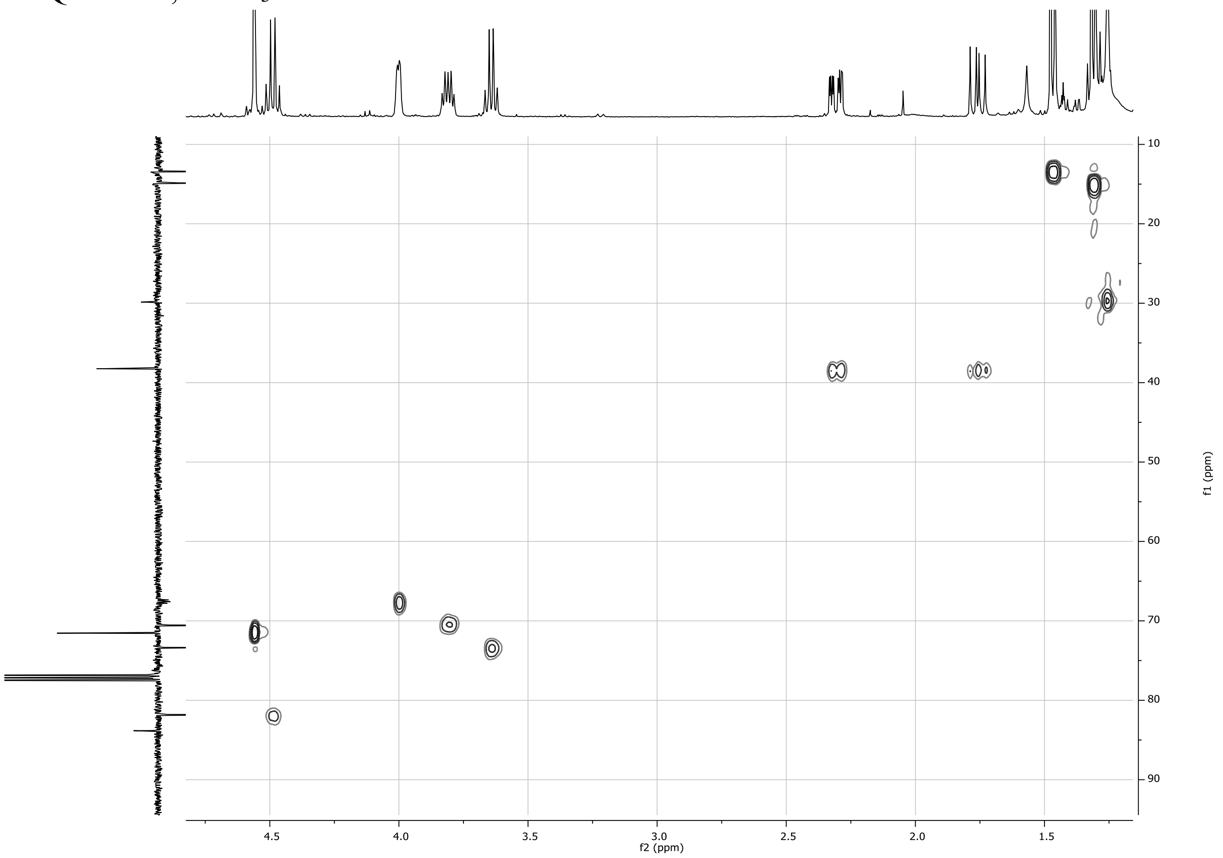

^{1}H NMR, 500 MHz, $CDCl_3$ of **S66**

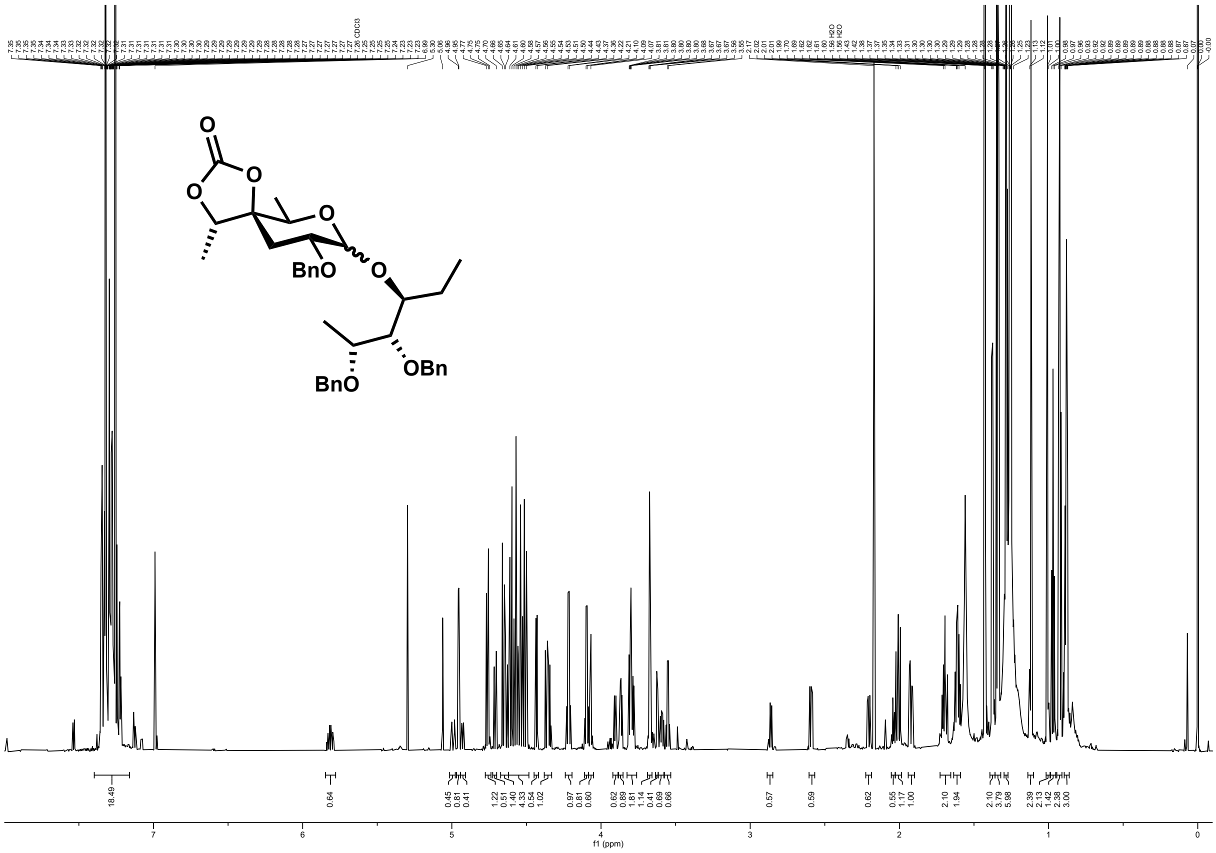

^{13}C NMR, 126 MHz, $CDCl_3$ of **S66**

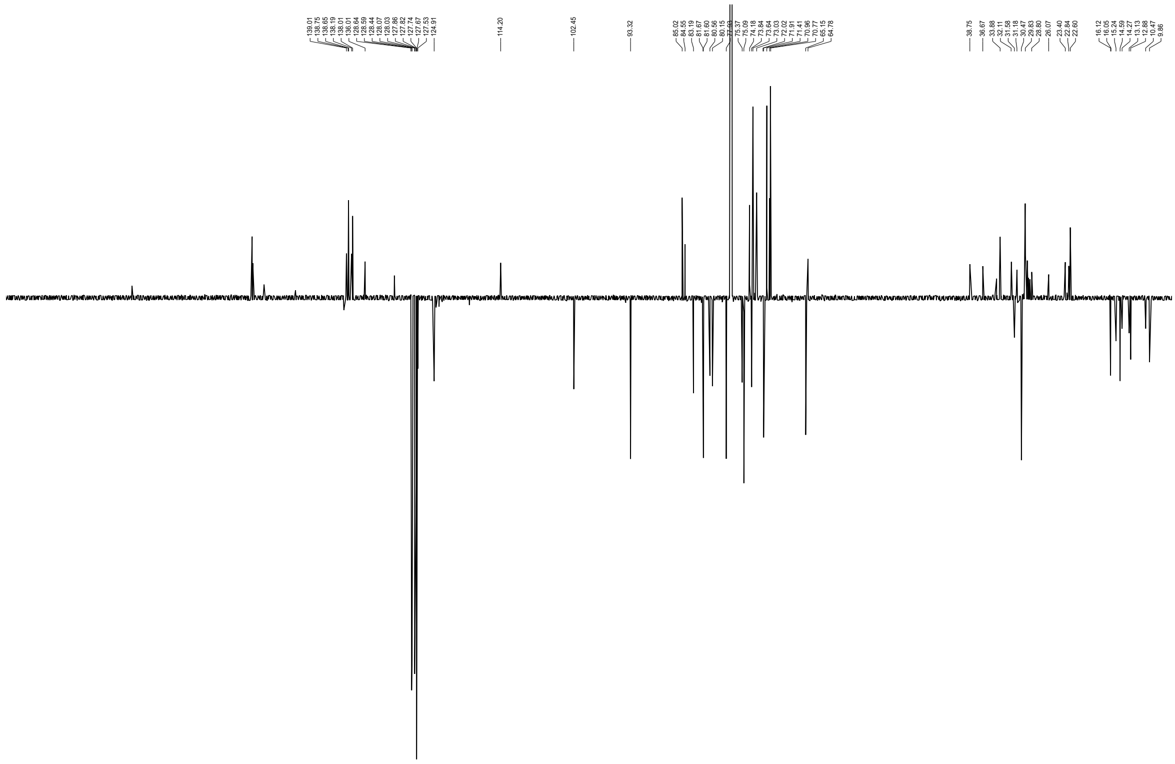

HH-COSY NMR, $CDCl_3$ of **S66**

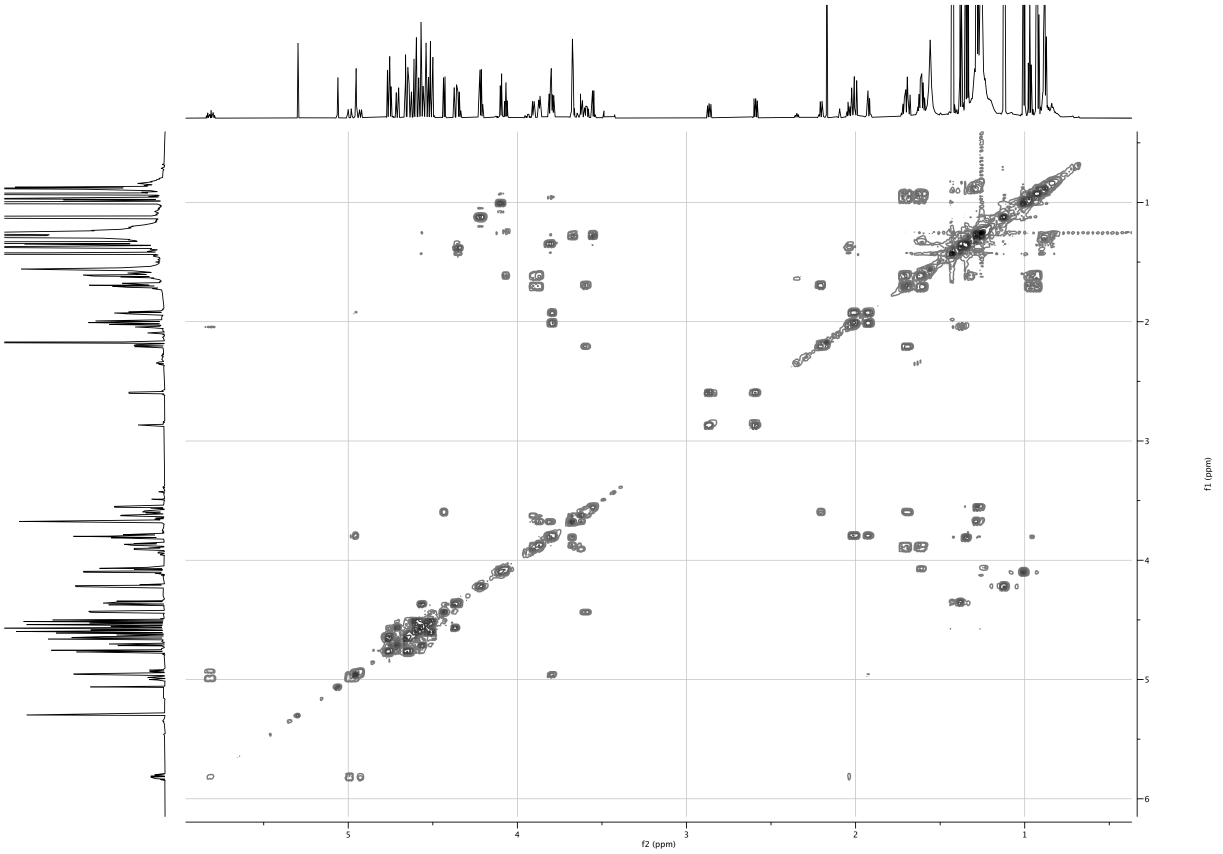

HSQC NMR, $CDCl_3$ of **S66**

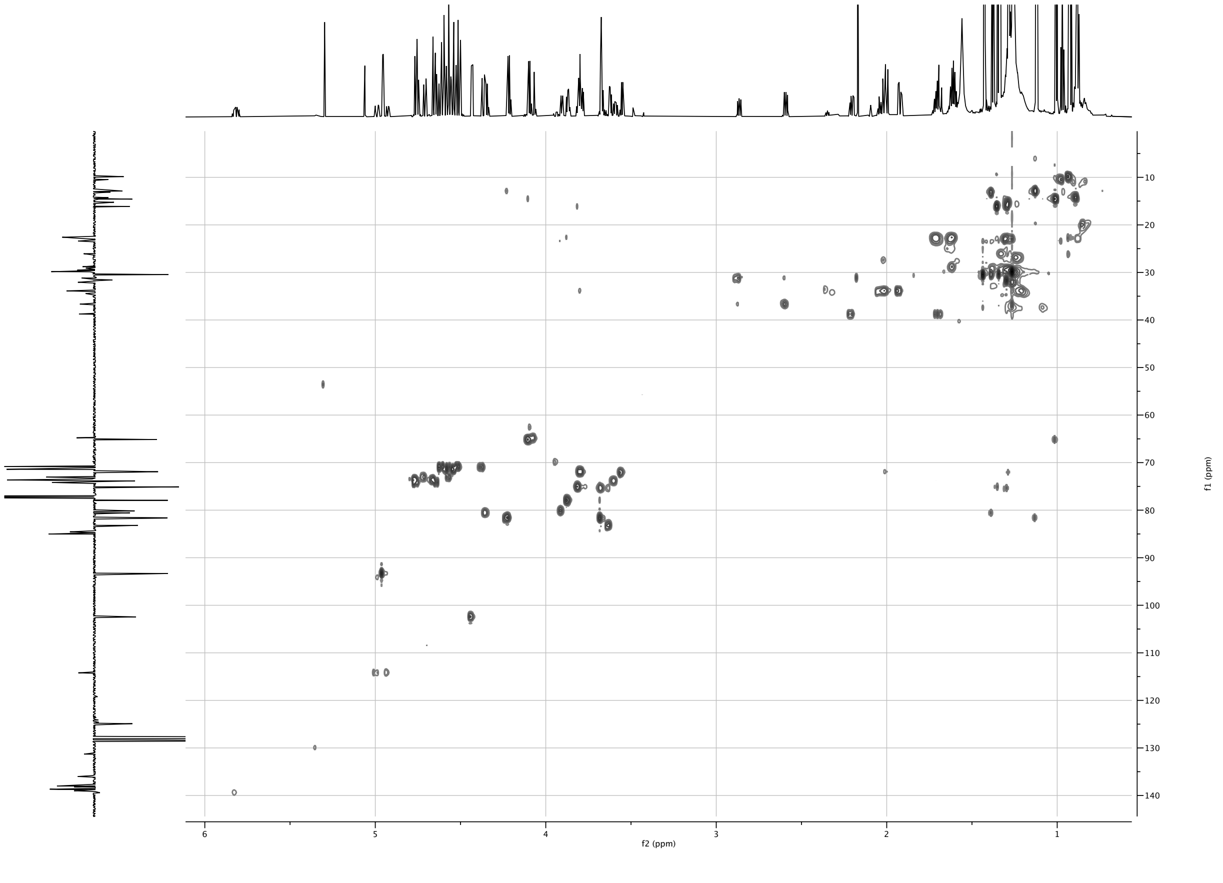

^{1}H NMR, 500 MHz, $CDCl_3$ of **S67**

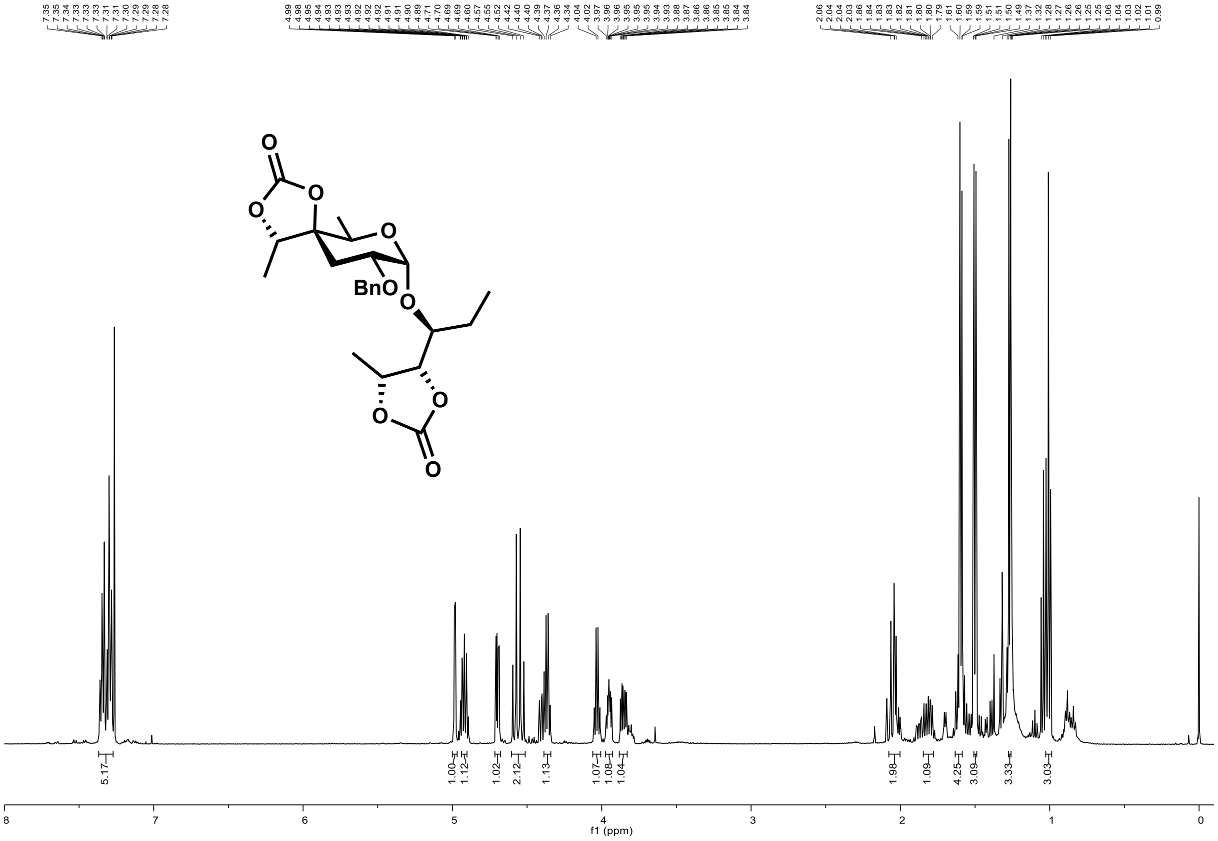

^{13}C NMR, 126 MHz, $CDCl_3$ of **S67**

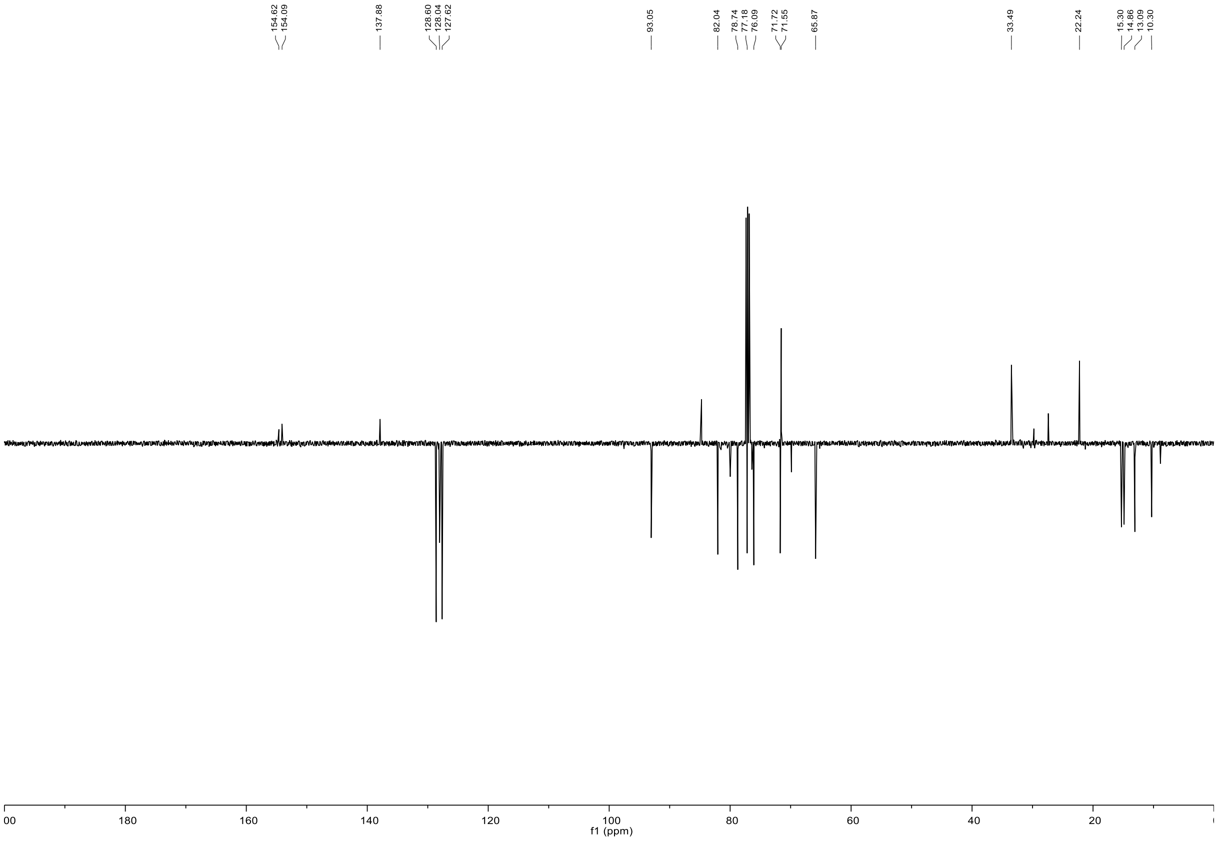

HH-COSY NMR, $CDCl_3$ of **S67**

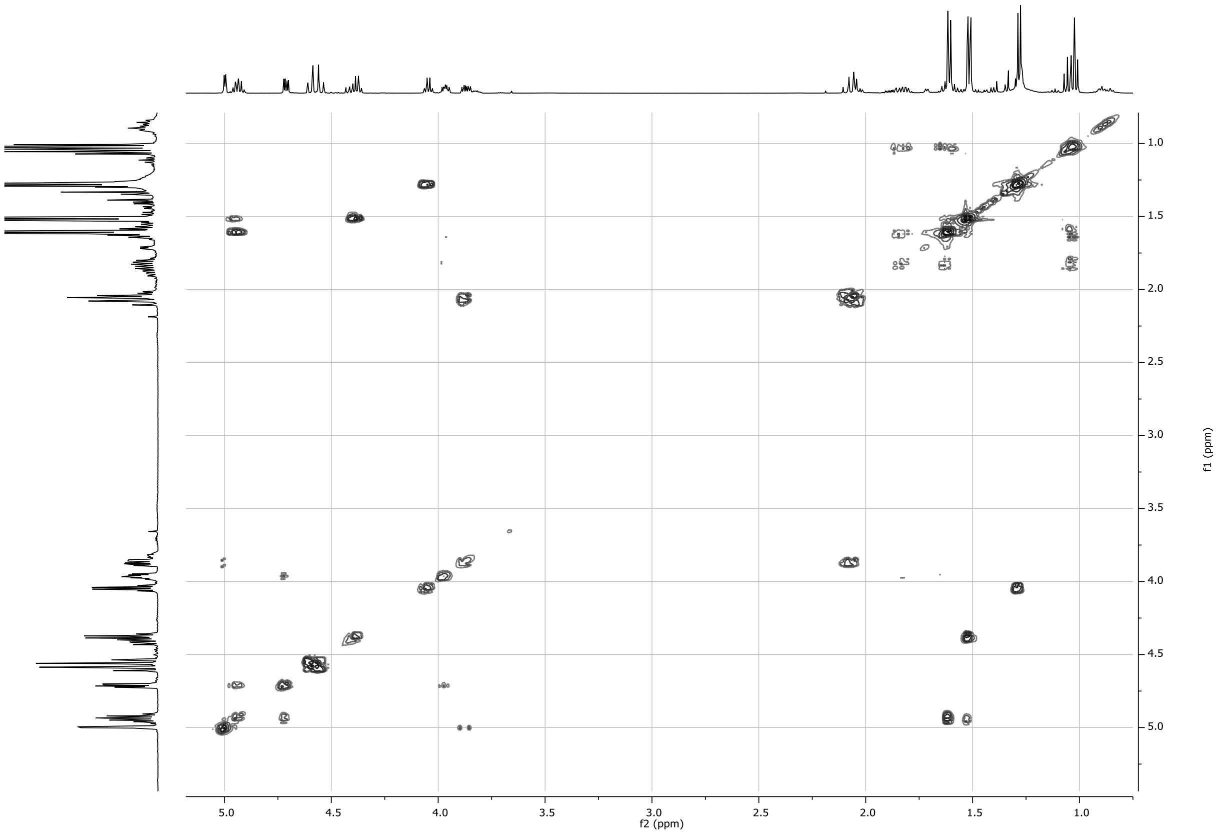

HSQC NMR, $CDCl_3$ of **S67**

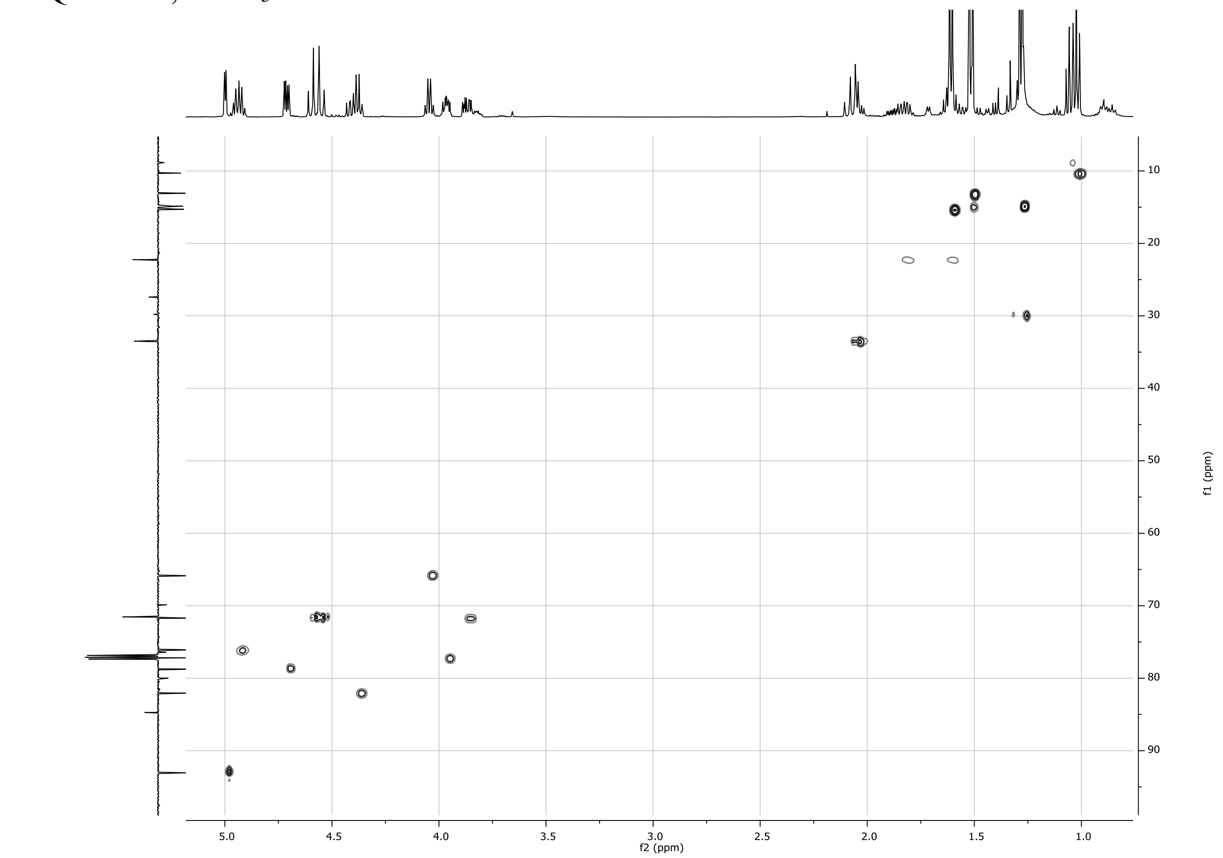

HMBC NMR, $CDCl_3$ of **S67**

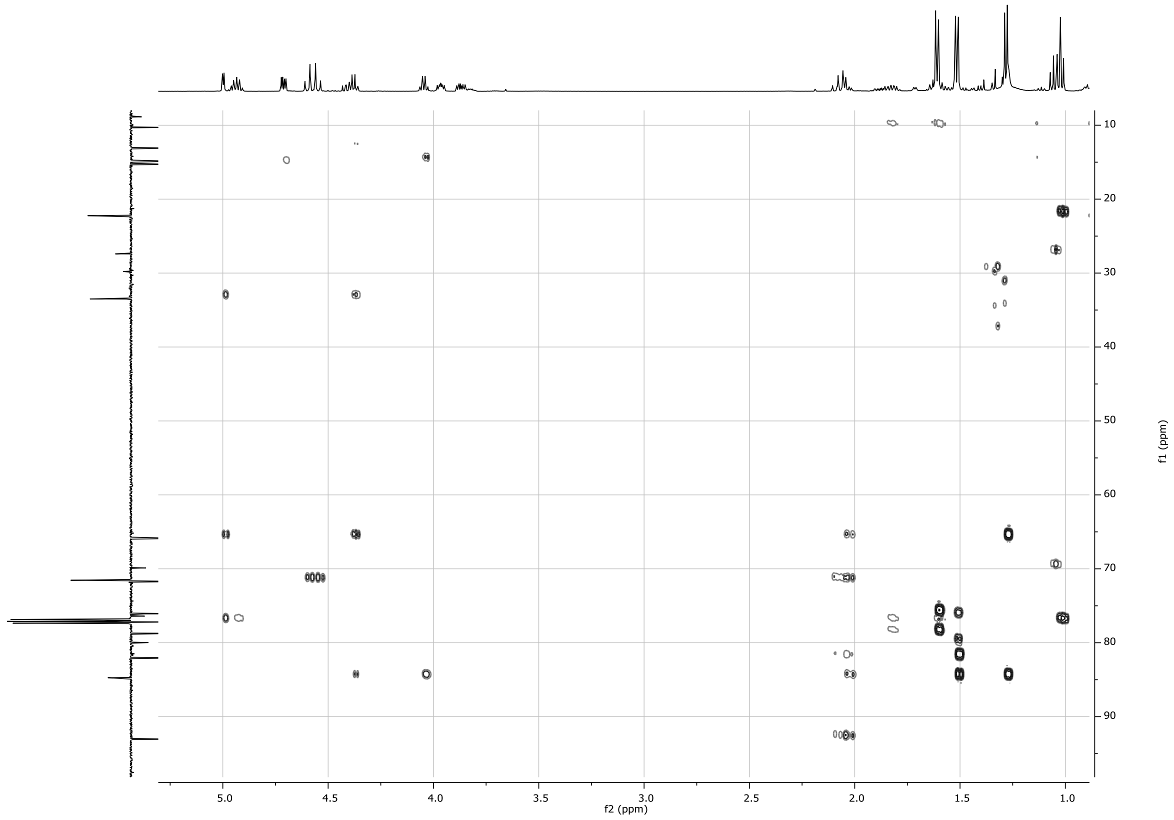

^{1}H NMR, 500 MHz, $CDCl_3$ of **S60 (+DMF)**

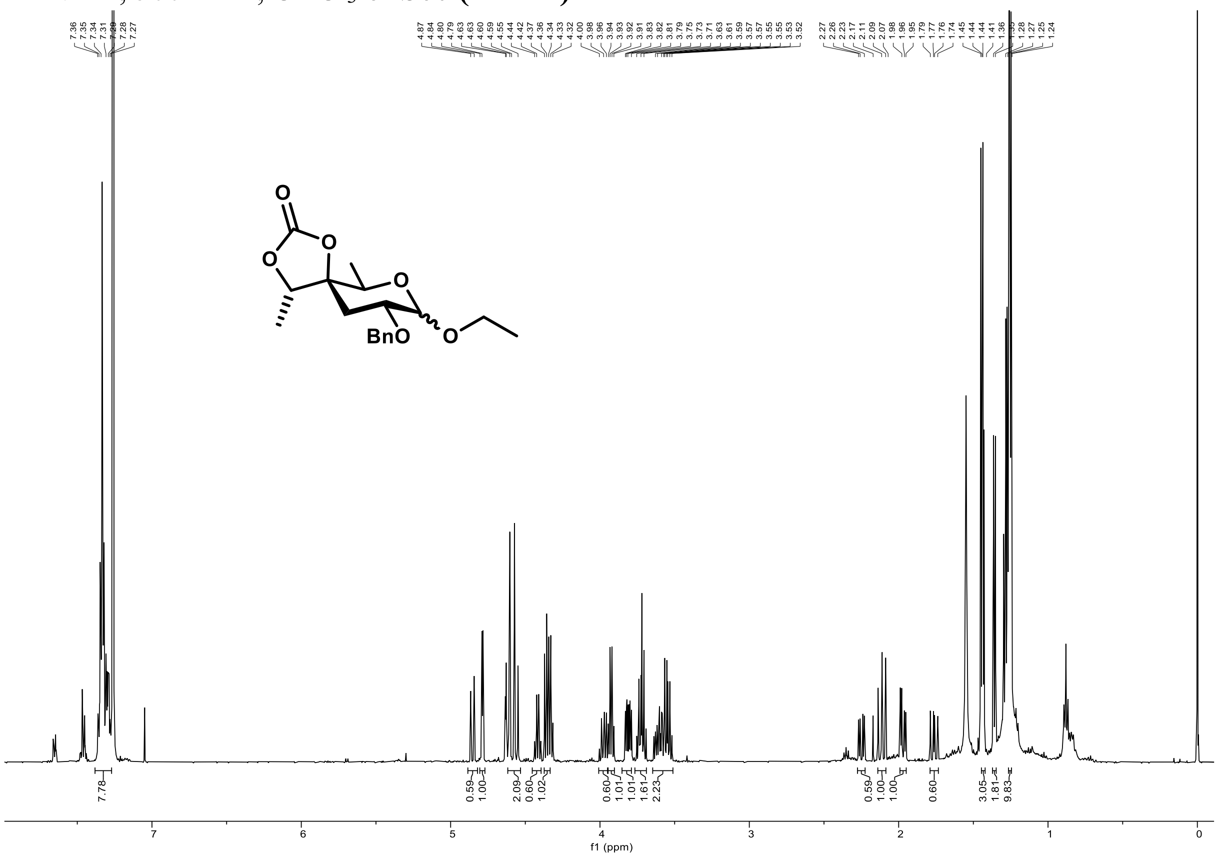

^{13}C NMR, 125 MHz, $CDCl_3$ of **S60 (+DMF)**

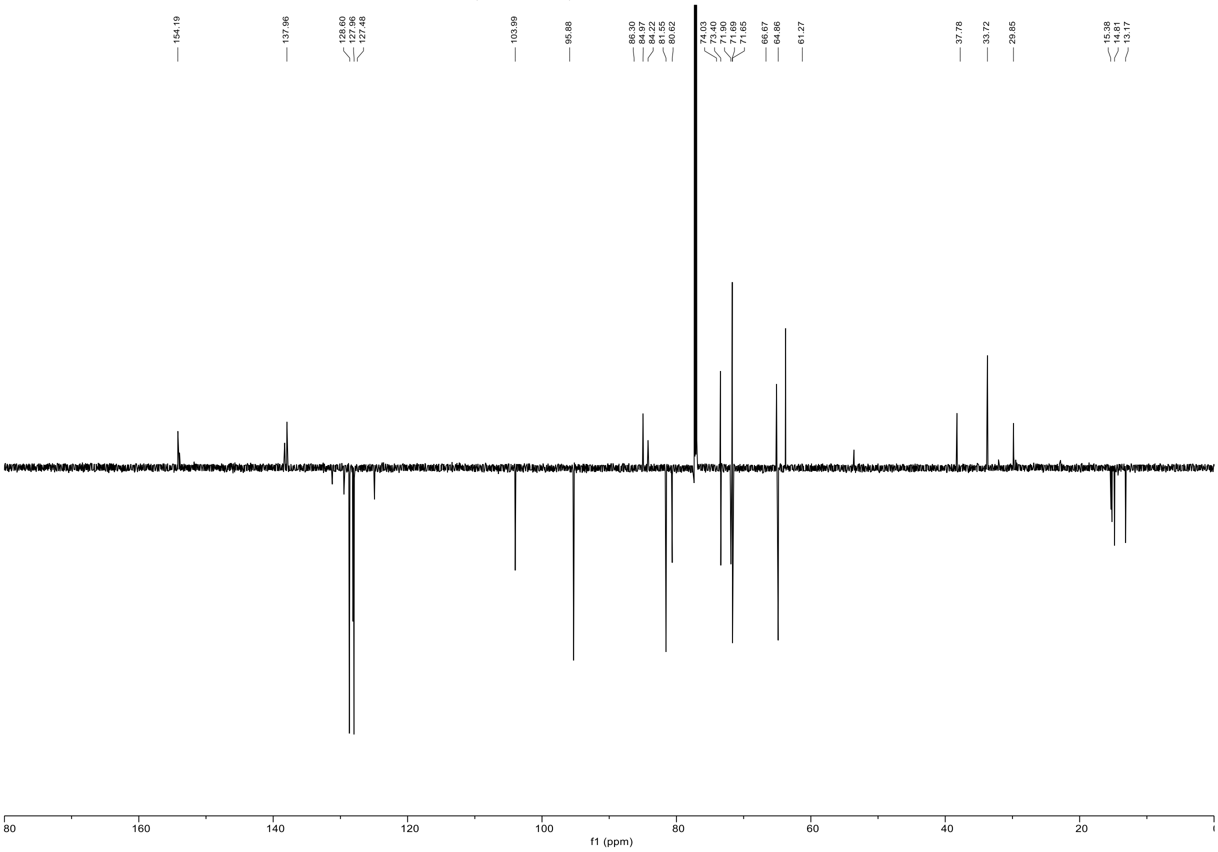

^{1}H NMR, 500 MHz, $CDCl_3$ of **S61 (+DMF)**

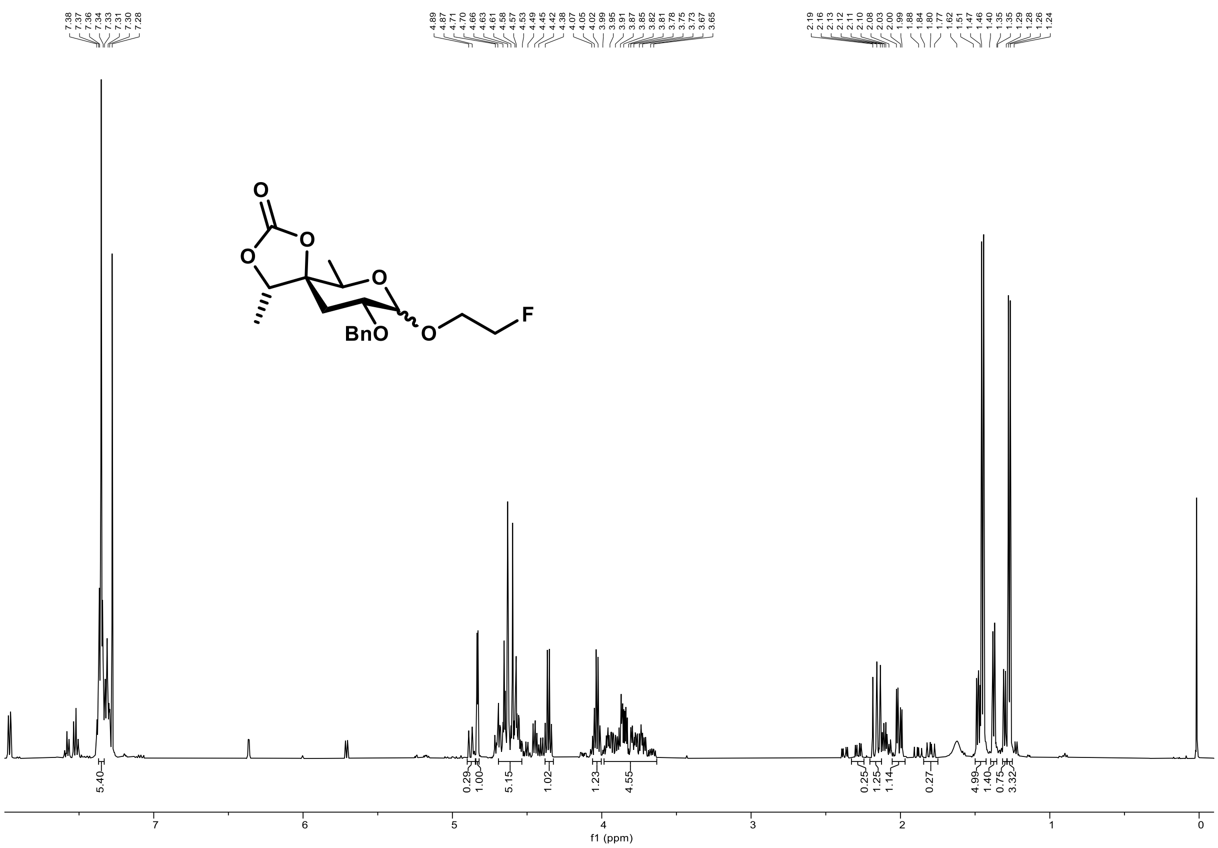

^{13}C NMR, 126 MHz, $CDCl_3$ of **S61 (+DMF)**

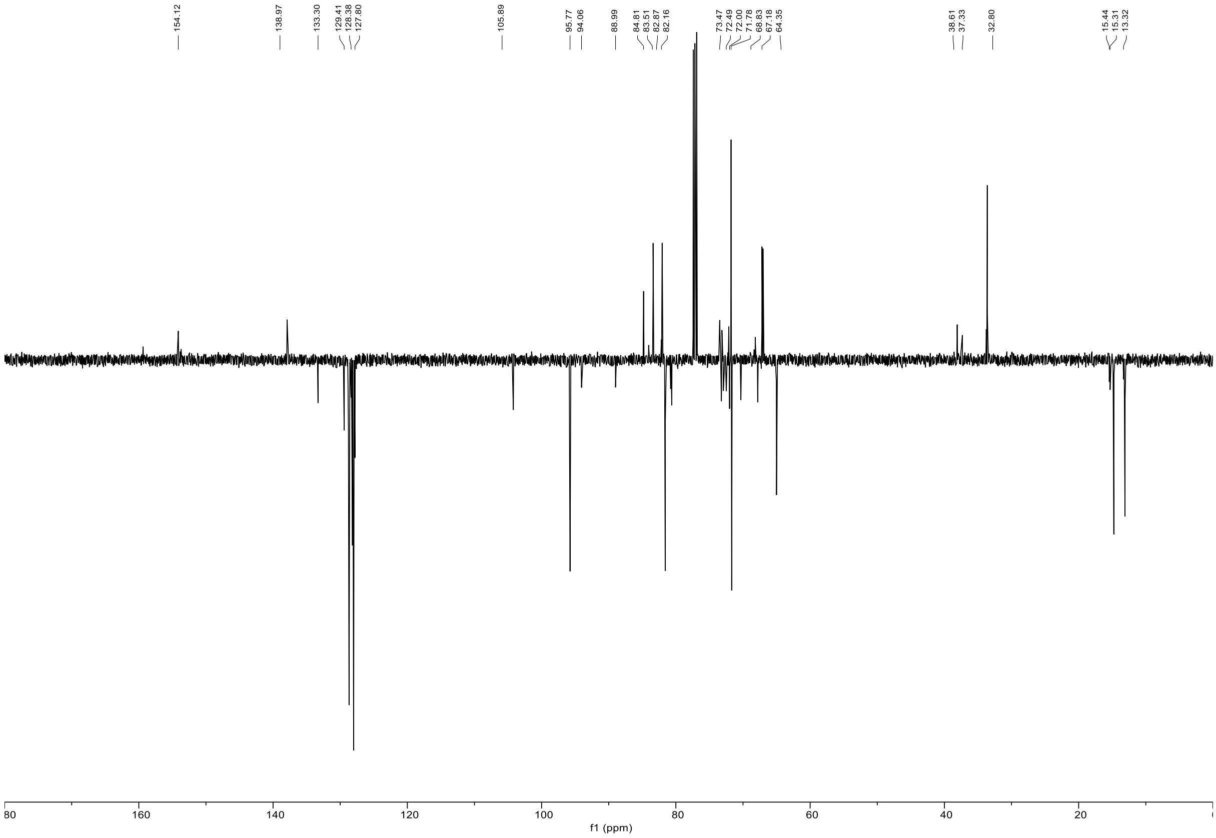

^{1}H NMR, 500 MHz, $CDCl_3$ of **S62 (+DMF)**

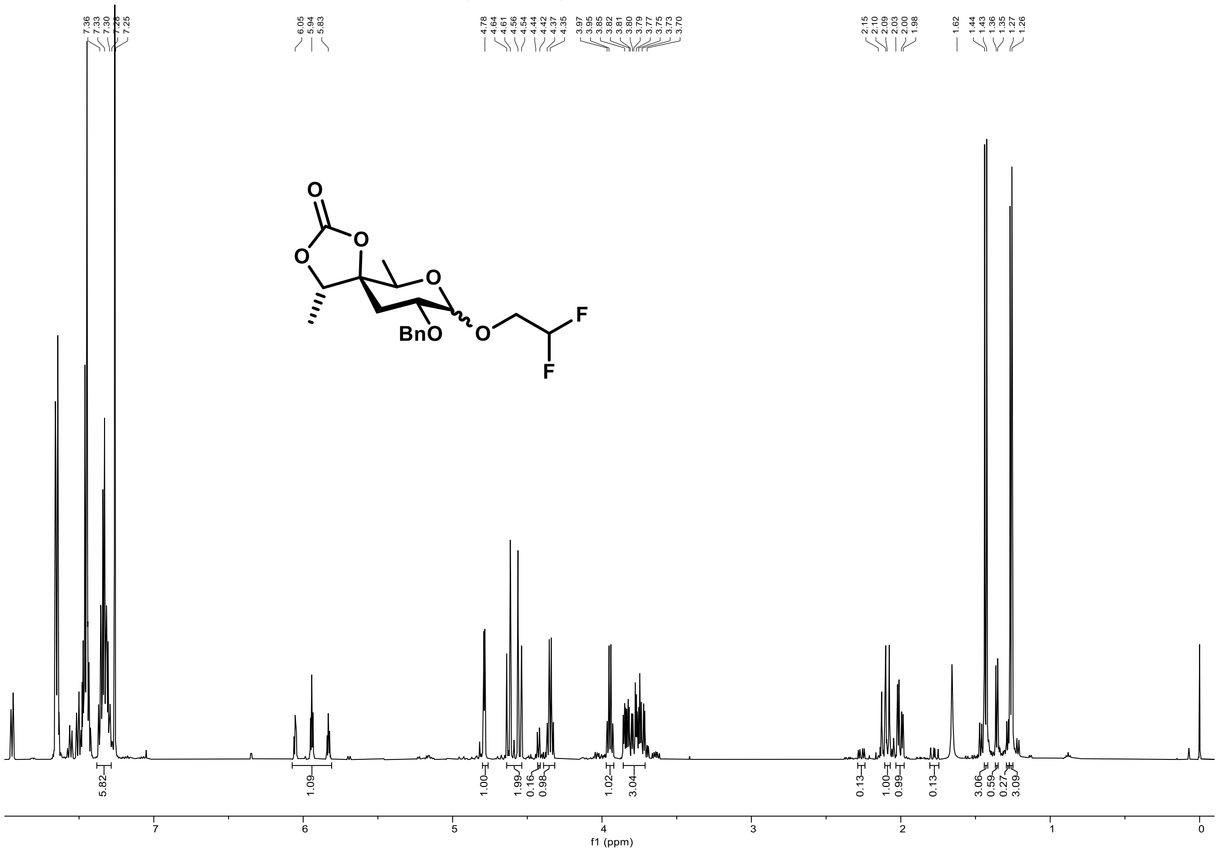

^{13}C NMR, 126 MHz, $CDCl_3$ of **S62 (+DMF)**

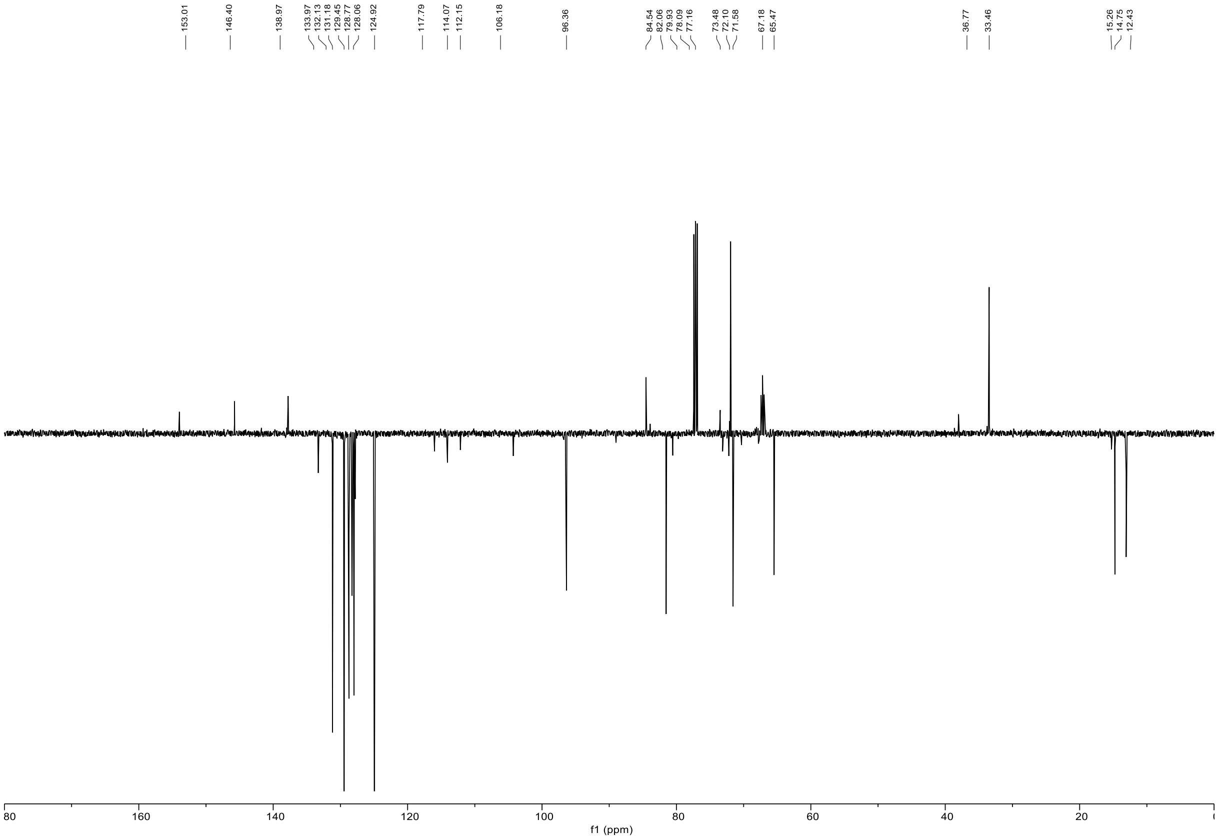

^{1}H NMR, 500 MHz, $CDCl_3$ of **S63 (+DMF)**

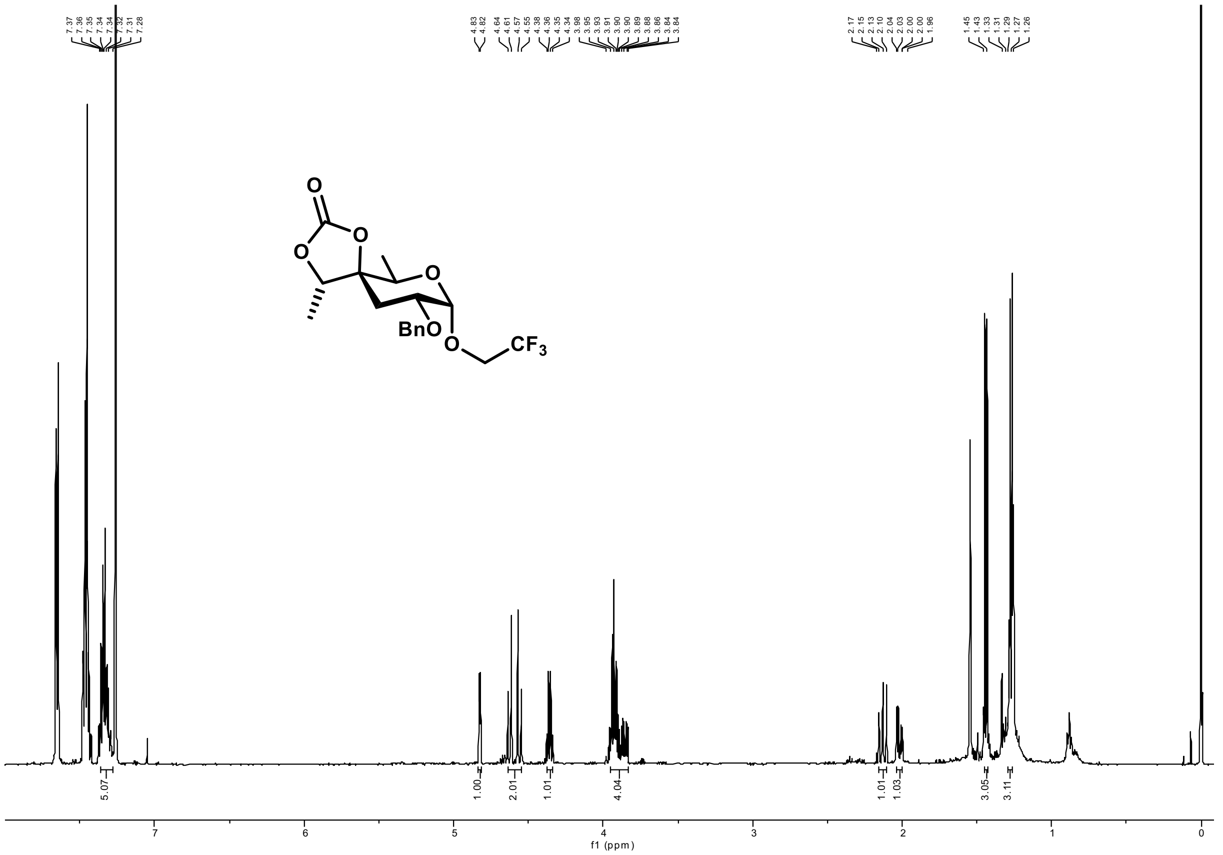

^{13}C NMR, 214 MHz, $CDCl_3$ of **S63 (+DMF)**

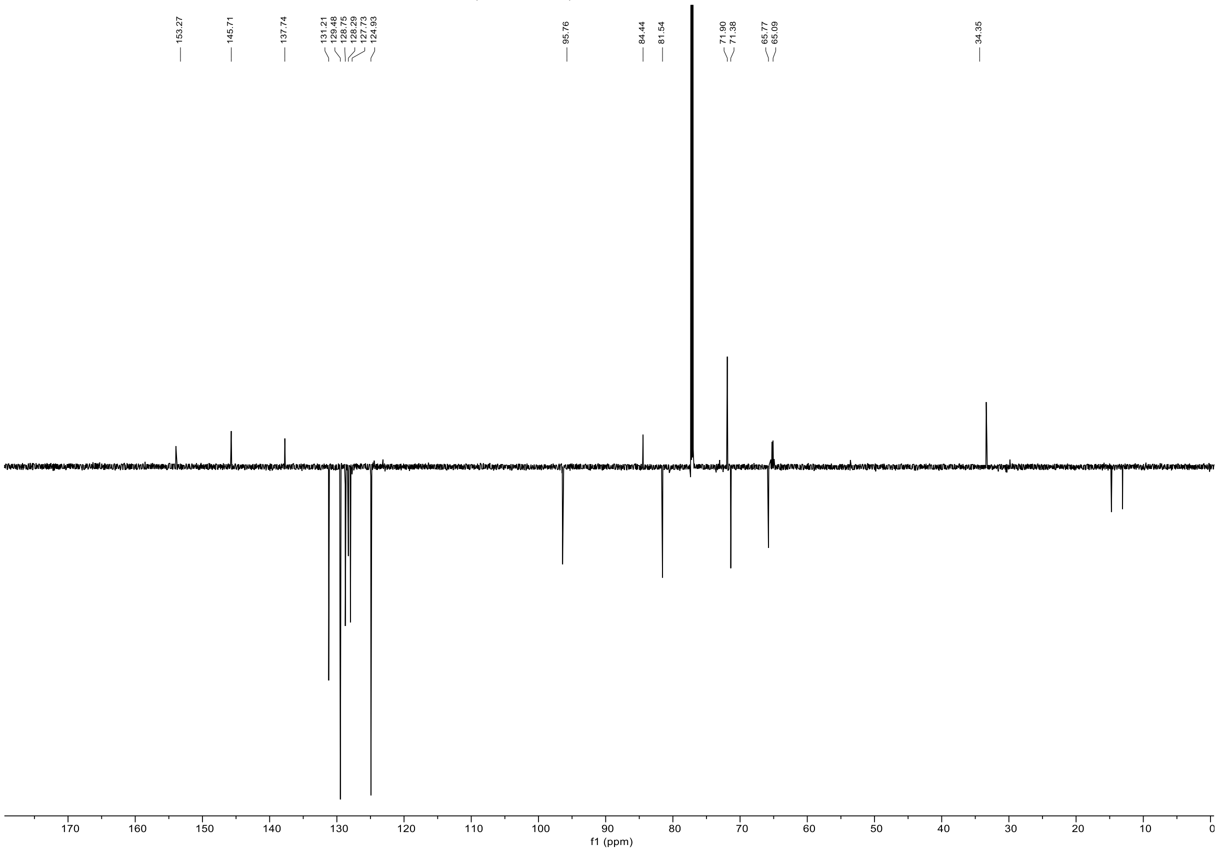

^{1}H NMR, 850 MHz, $CDCl_3$ of **S59 (+DMF)**

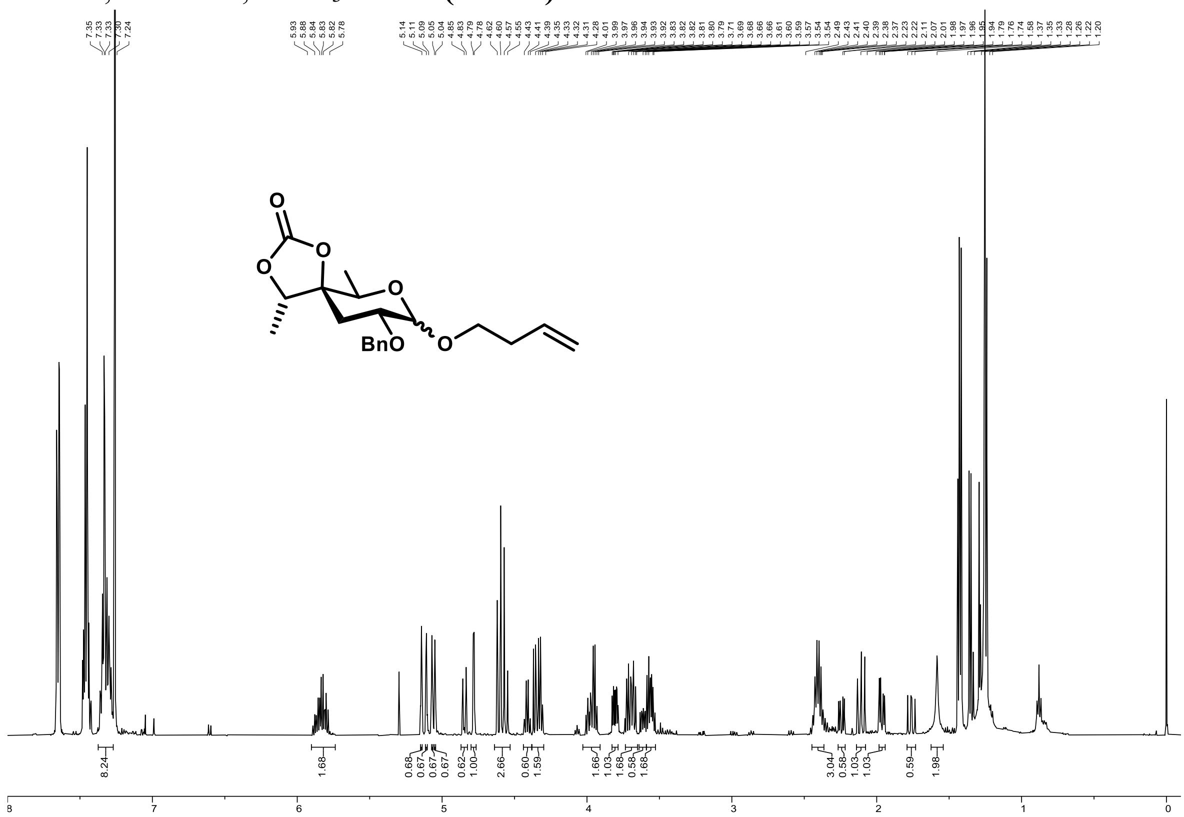

^{13}C NMR, 214 MHz, $CDCl_3$ of **S59 (+DMF)**

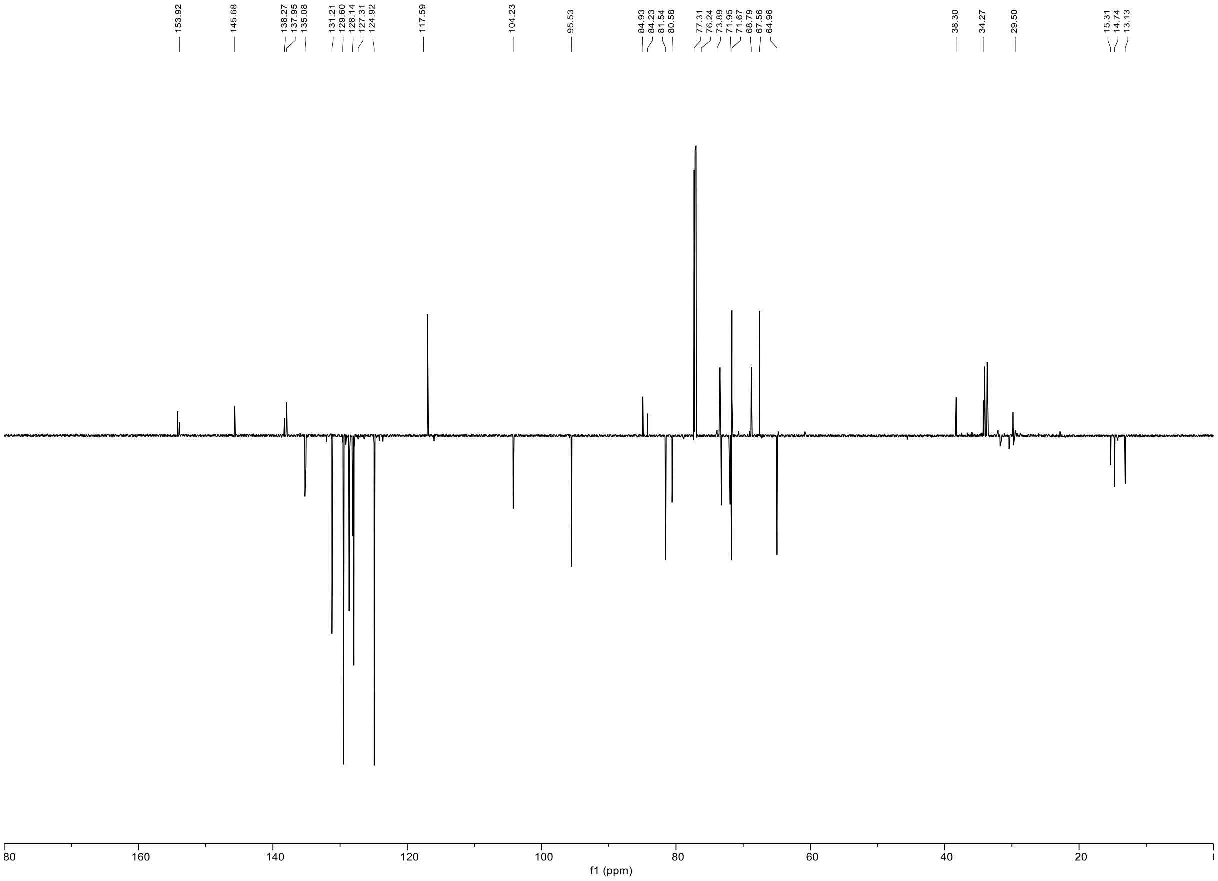

^{1}H NMR, 500 MHz, $CDCl_3$ of **S60 (+TBAI)**

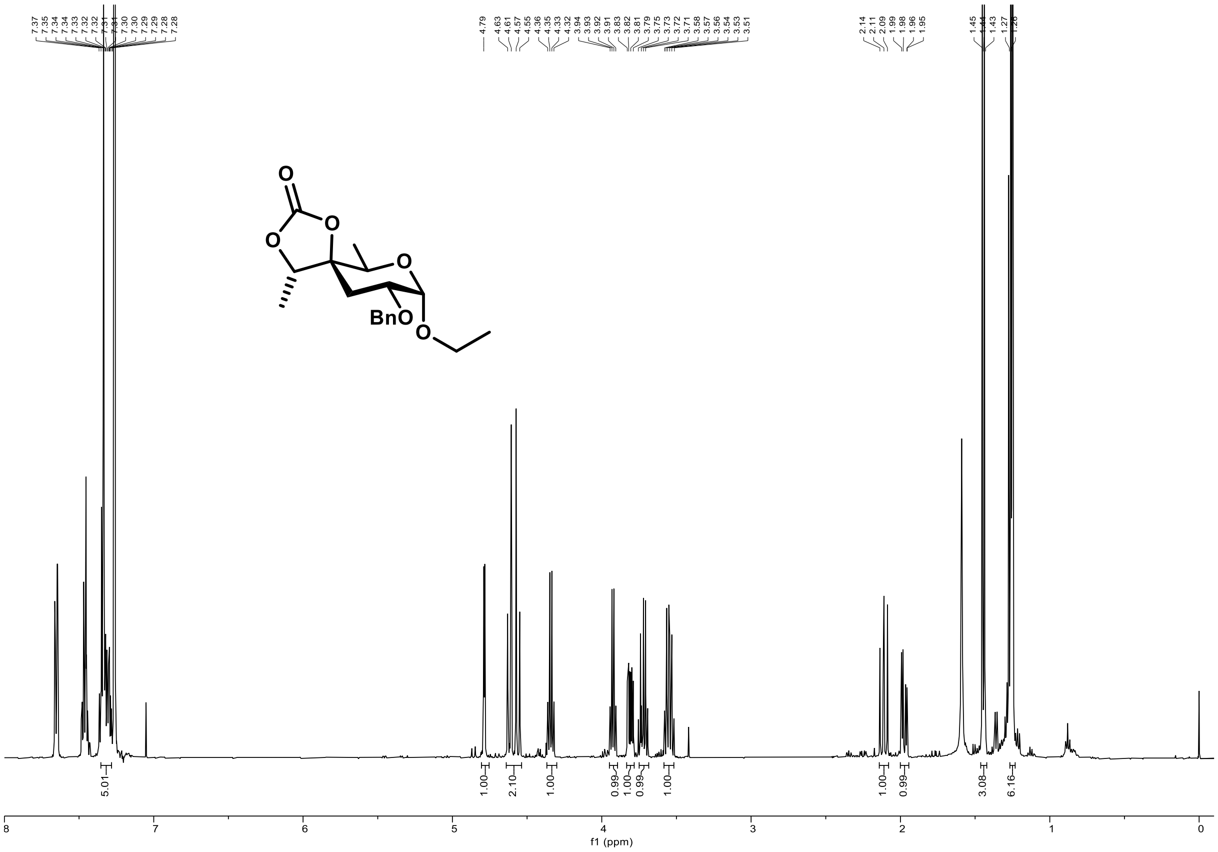

^{13}C NMR, 126 MHz, $CDCl_3$ of **S60 (+TBAI)**

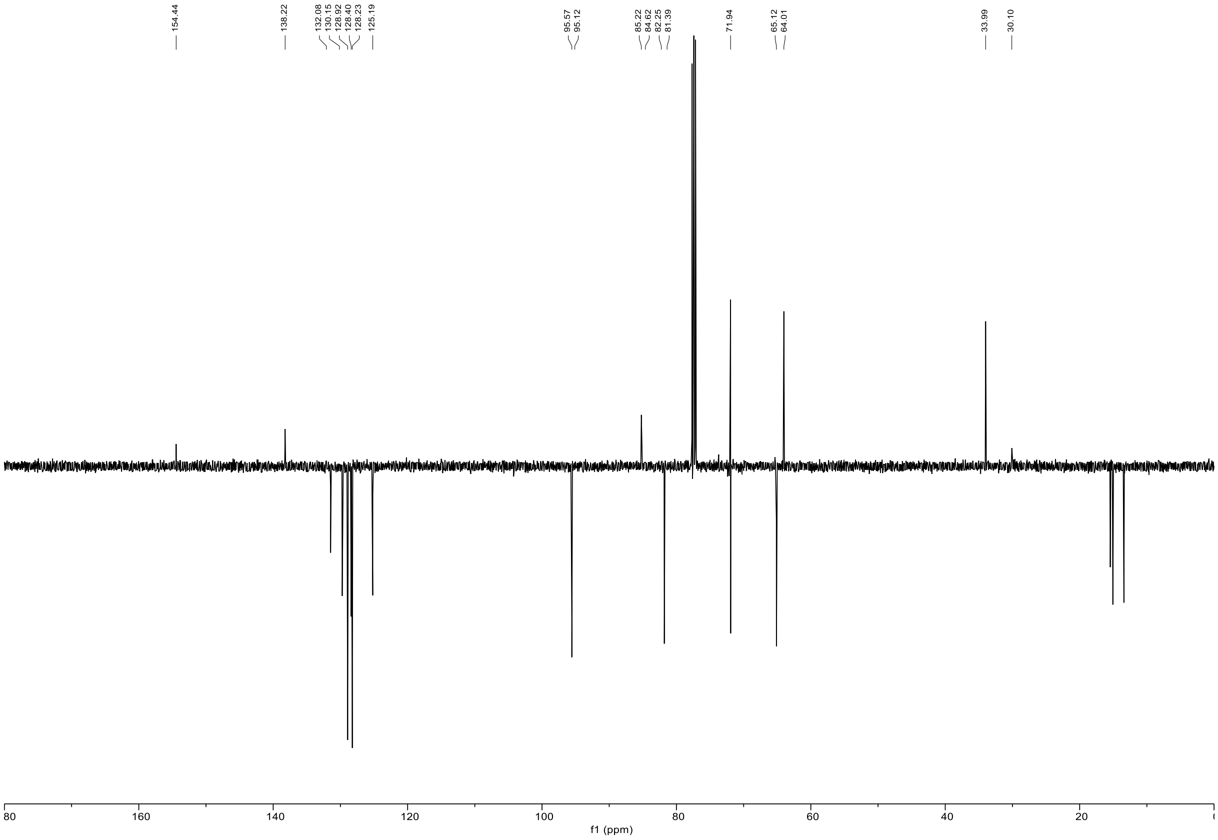

^{1}H NMR, 500 MHz, $CDCl_3$ of **S61 (+TBAI)**

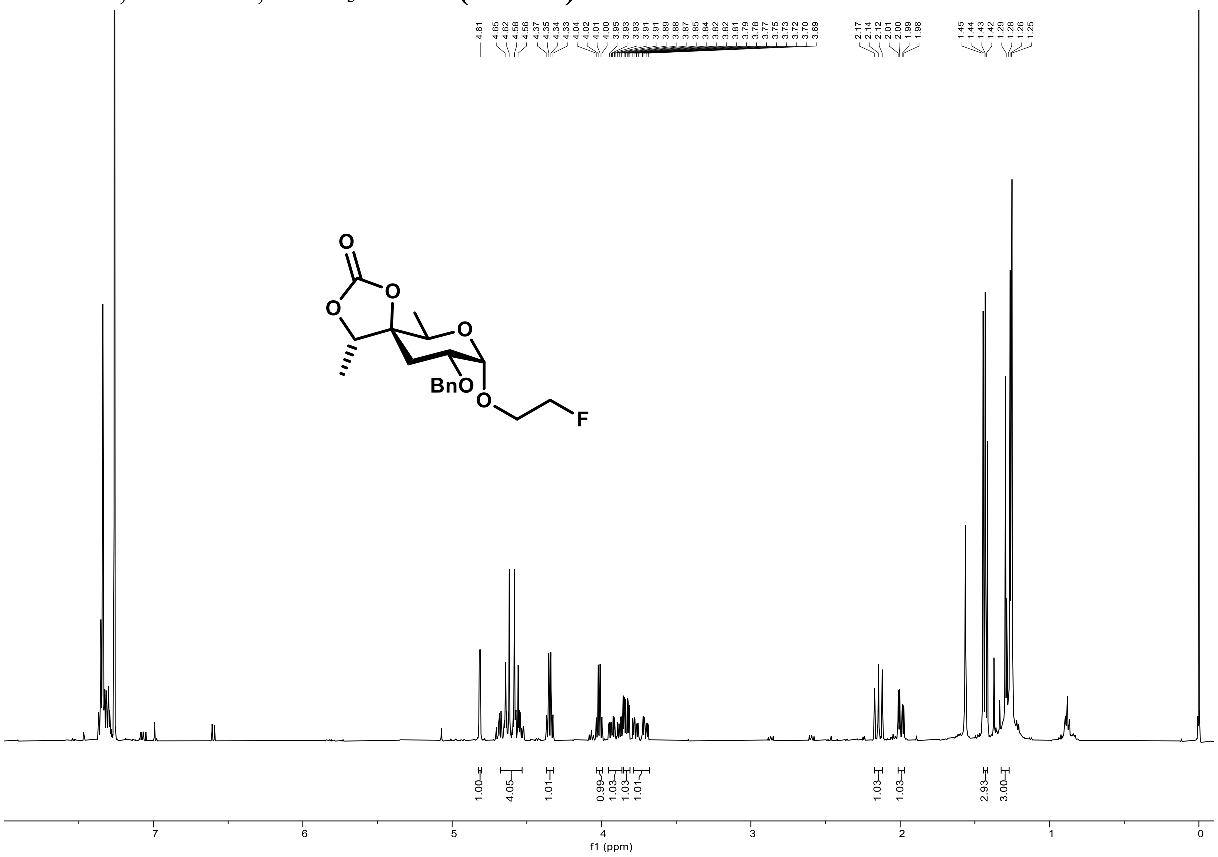

^{13}C NMR, 126 MHz, $CDCl_3$ of **S61 (+TBAI)**

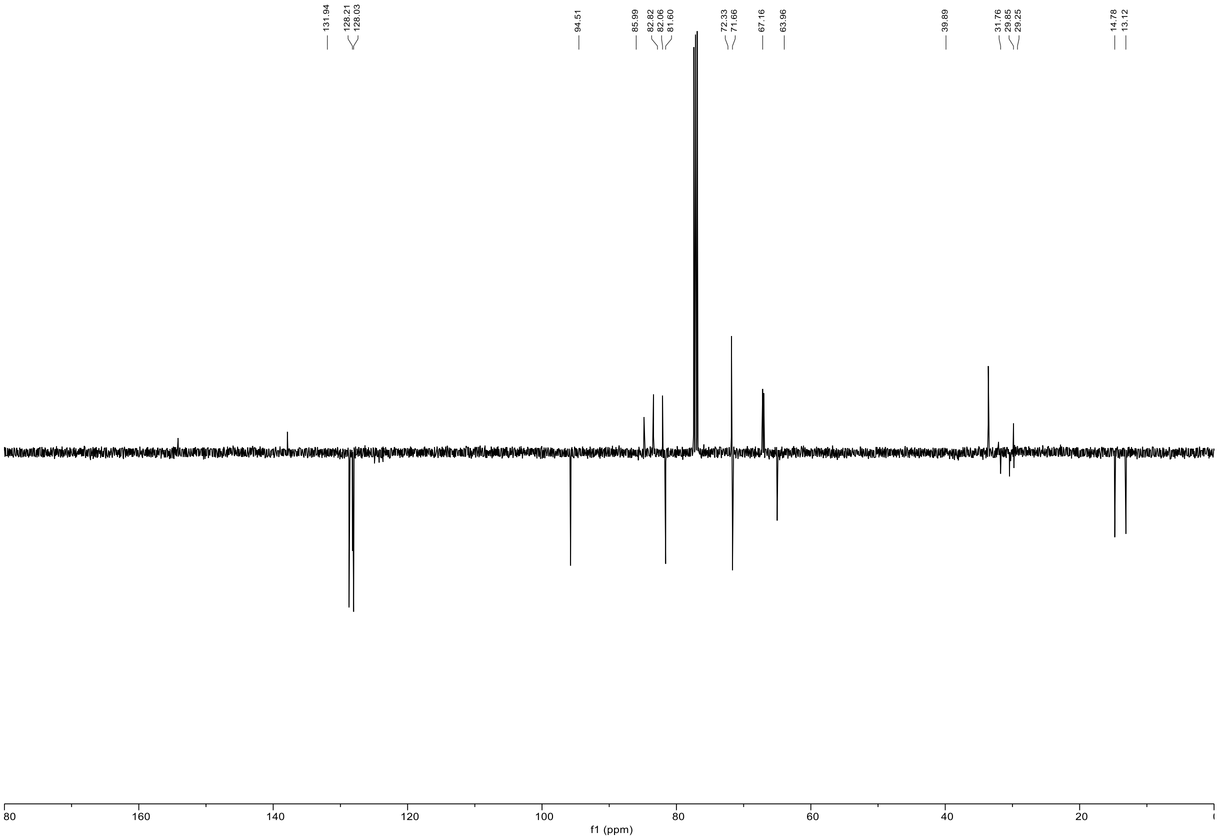

^{1}H NMR, 500 MHz, $CDCl_3$ of **S62 (+TBAI)**

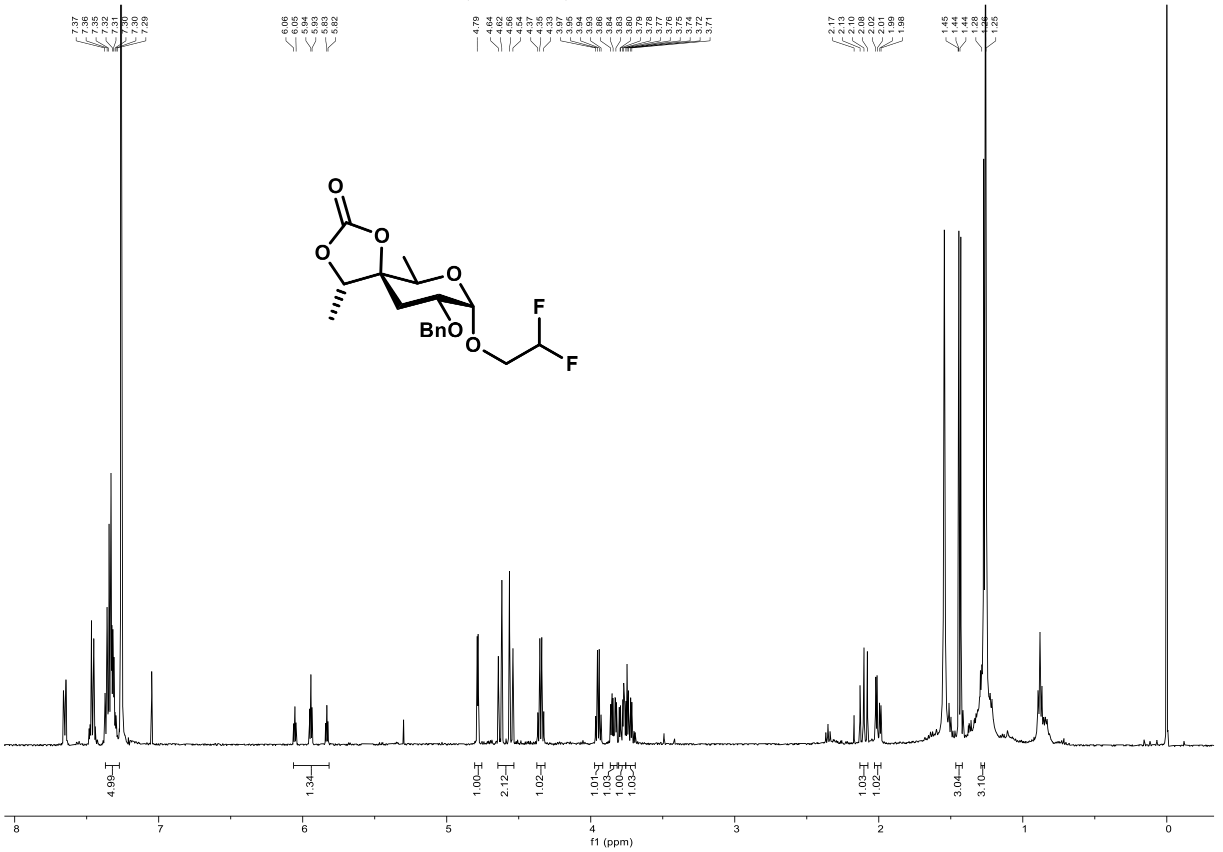

^{13}C NMR, 126 MHz, $CDCl_3$ of **S62 (+TBAI)**

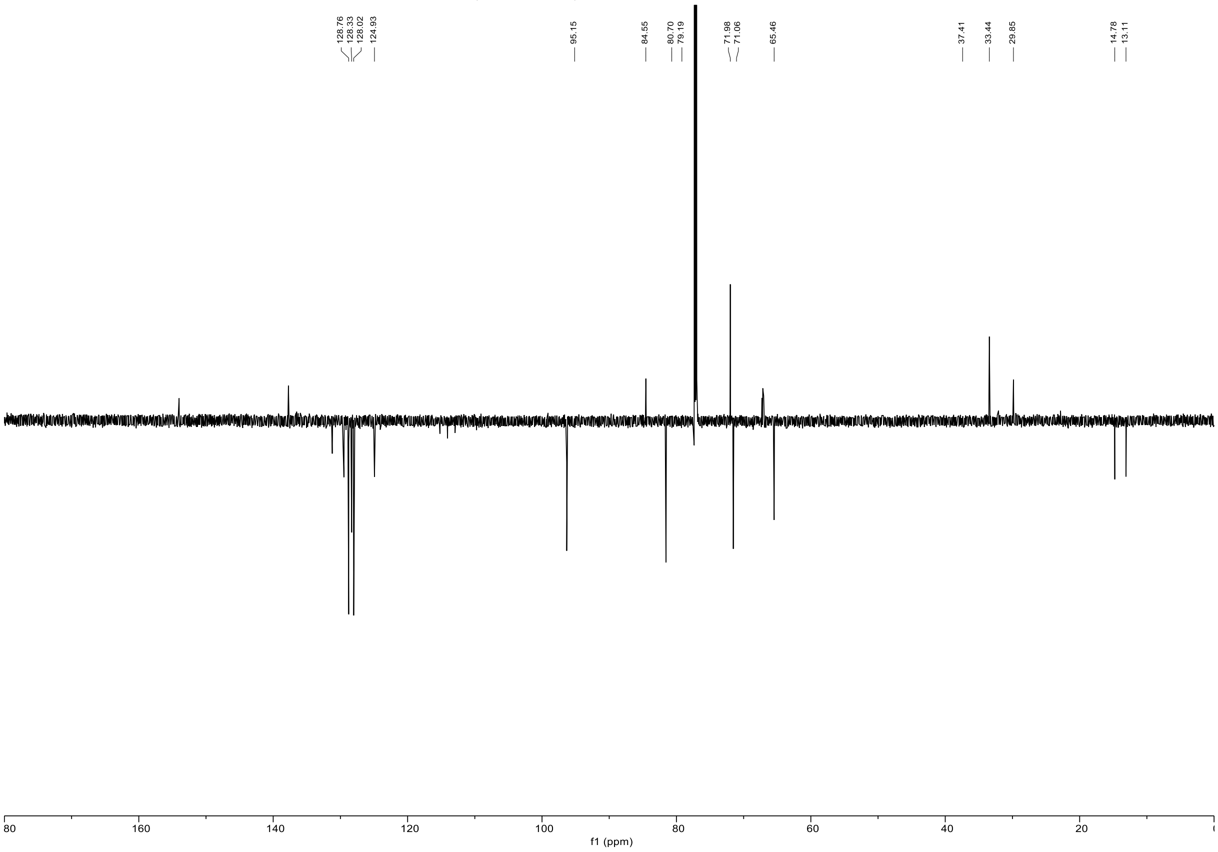

^{1}H NMR, 400 MHz, $CDCl_3$ of **S59 (+TBAI)**

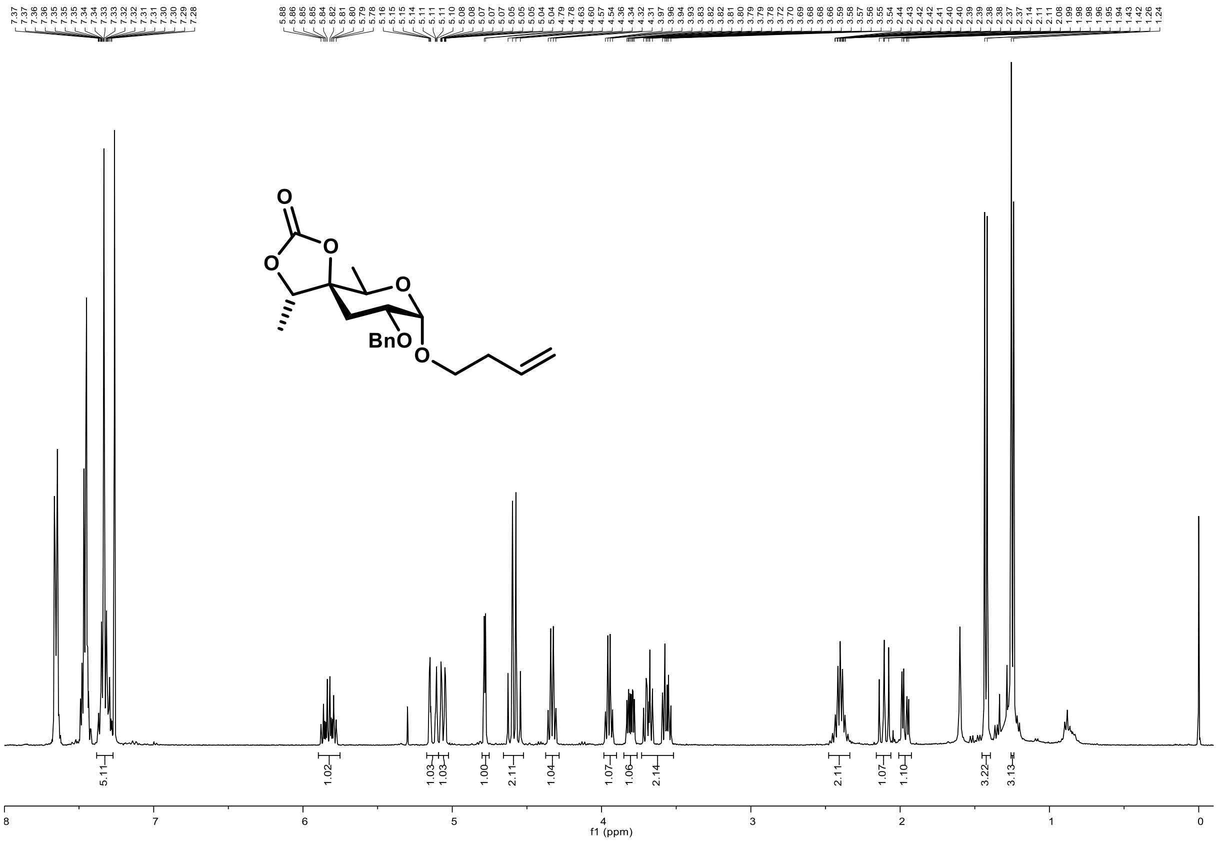

^{13}C NMR, 101 MHz, $CDCl_3$ of **S59 (+TBAI)**

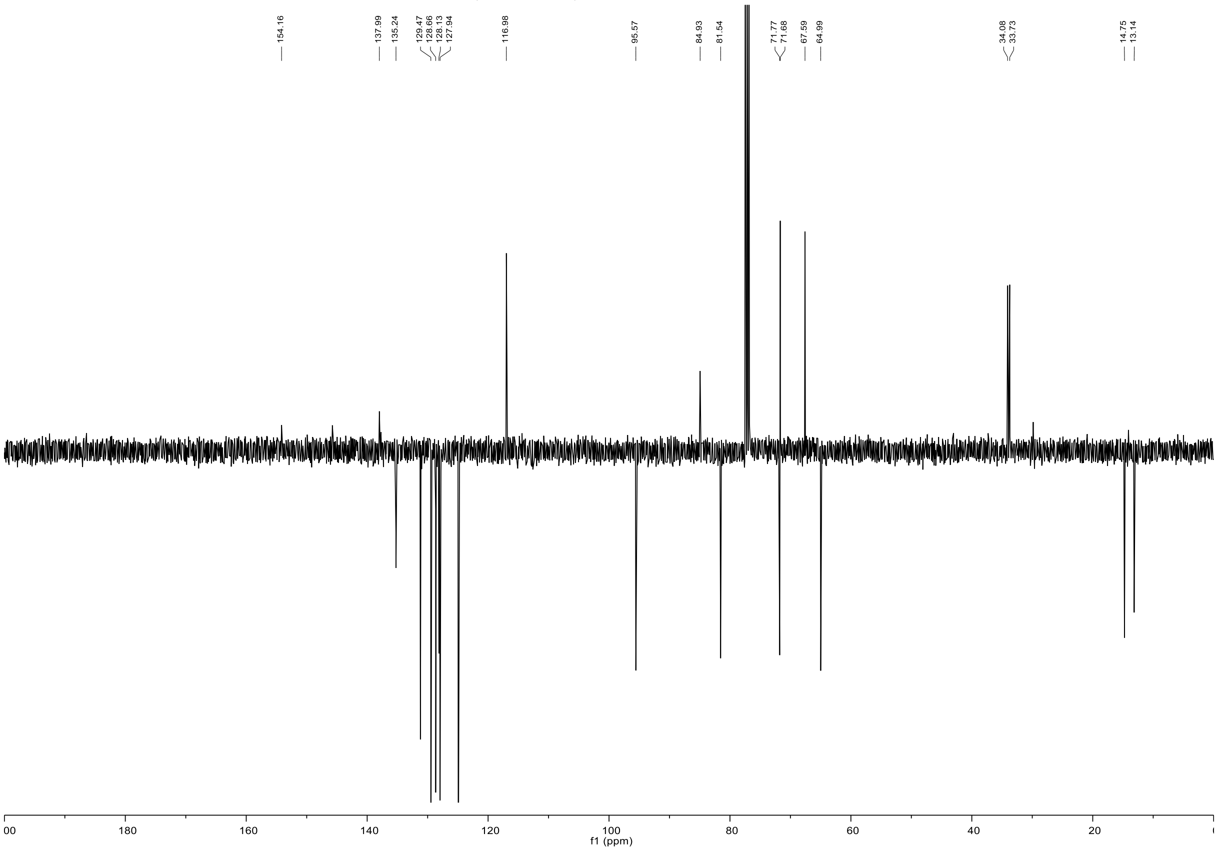

^{1}H NMR, 400 MHz, $CDCl_3$ of **26**

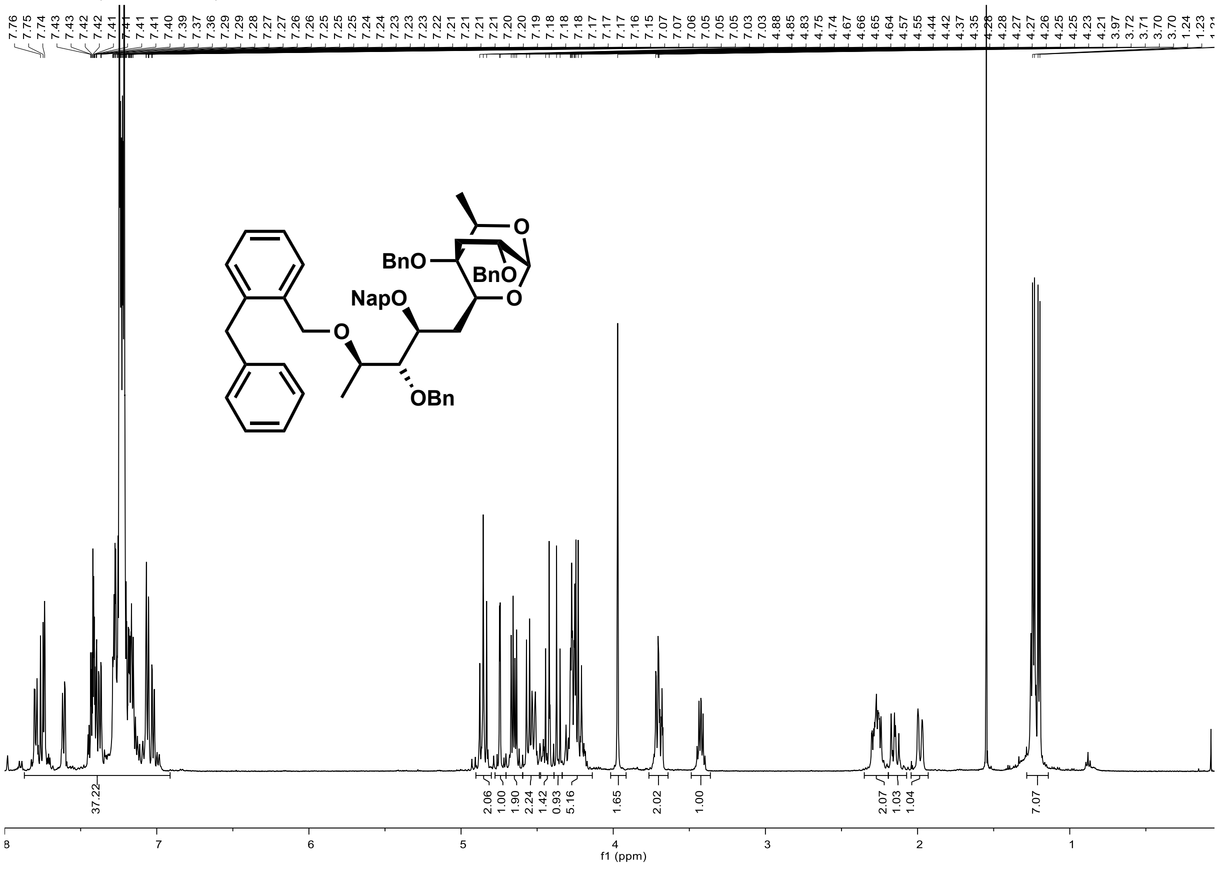

^{13}C NMR, 101 MHz, $CDCl_3$ of **26**

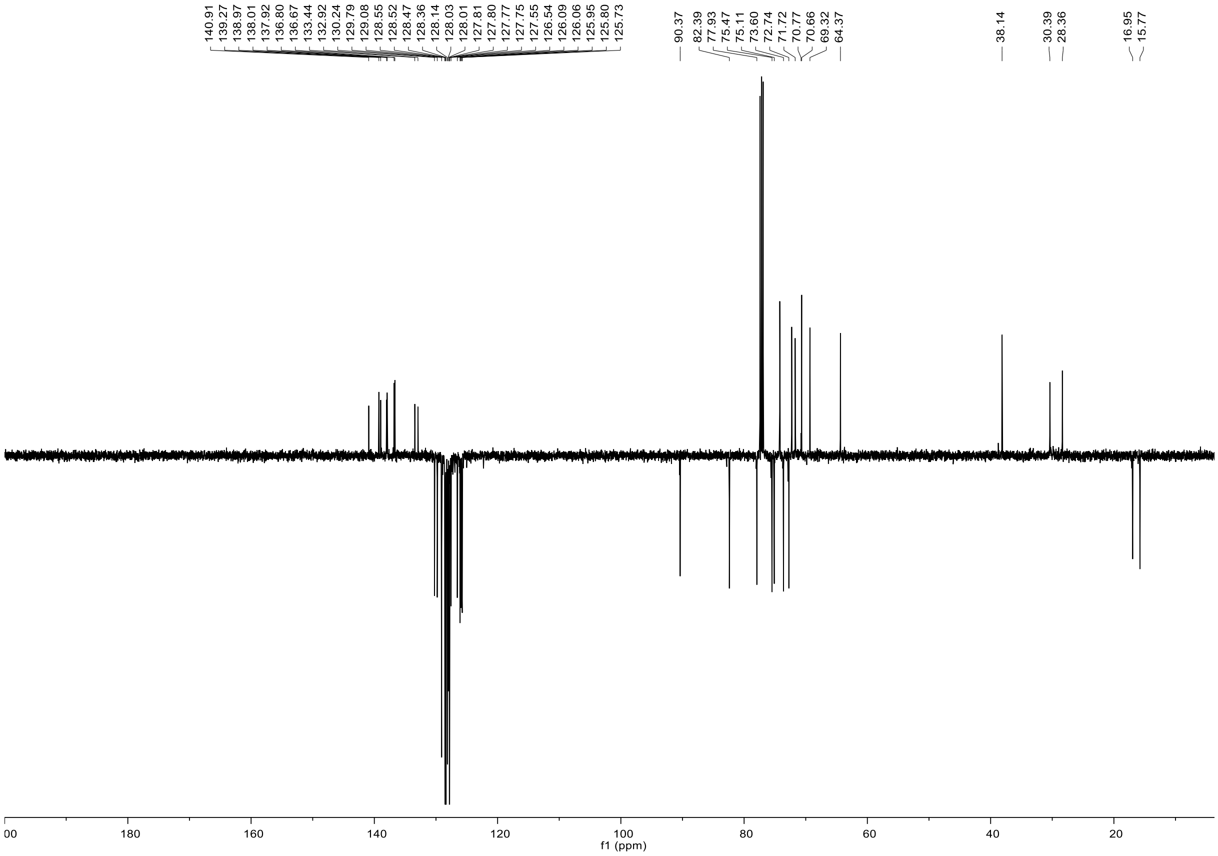

HH-COSY NMR, $CDCl_3$ of **26**

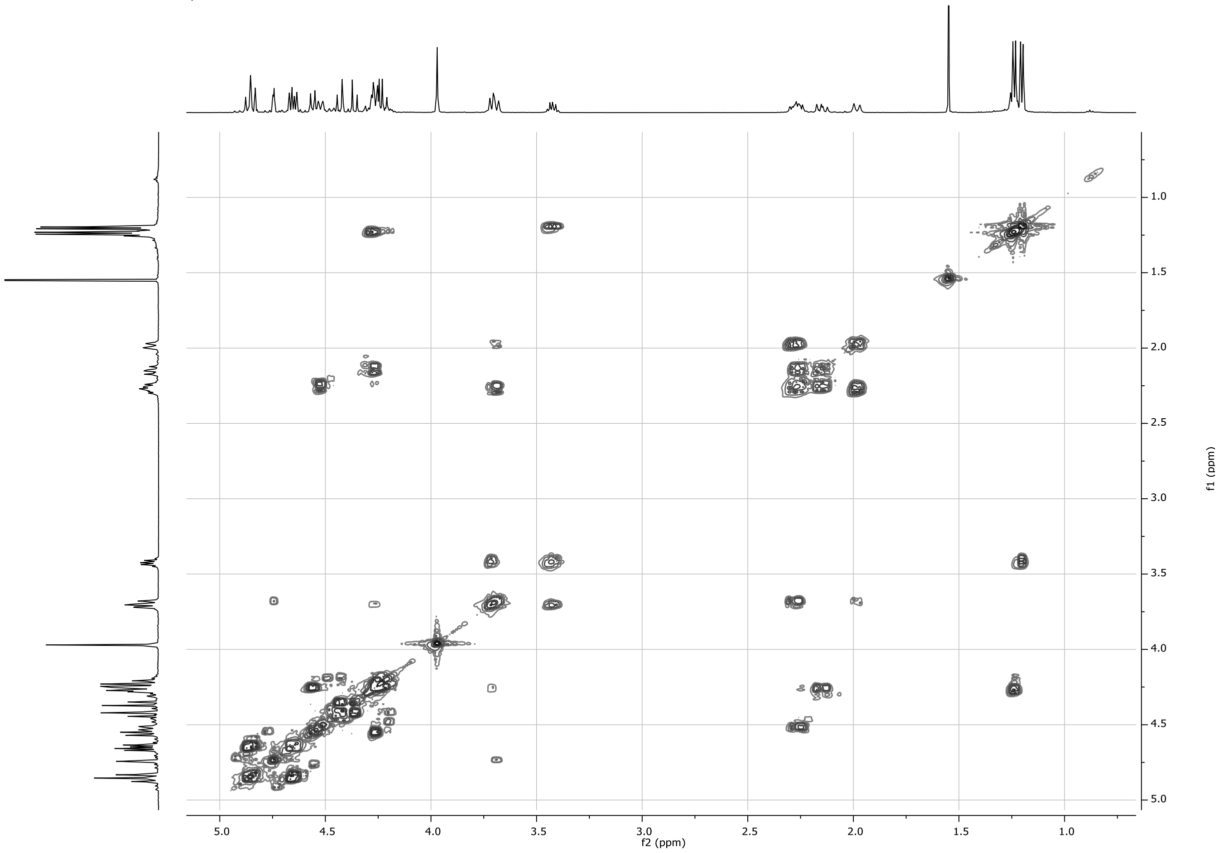

HSQC NMR, $CDCl_3$ of **26**

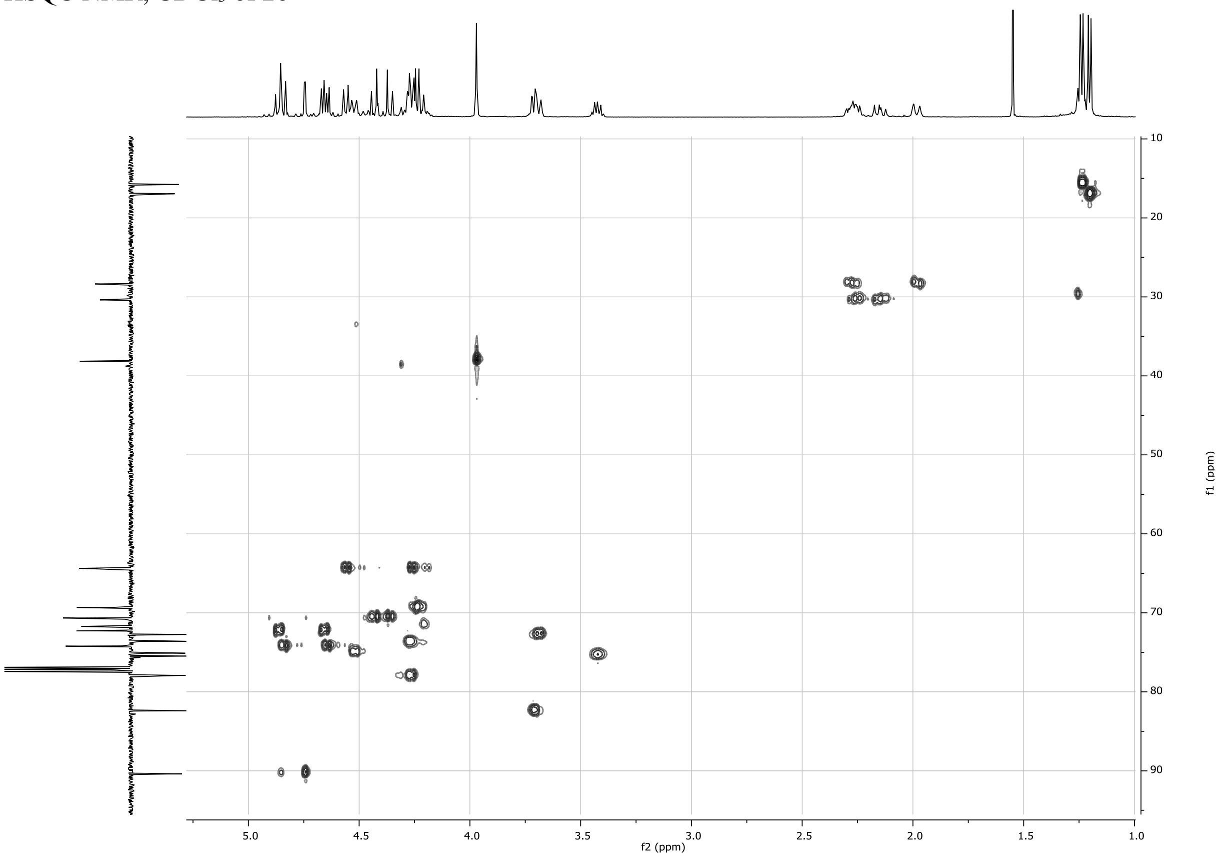

NOESY NMR, $CDCl_3$ of **26**

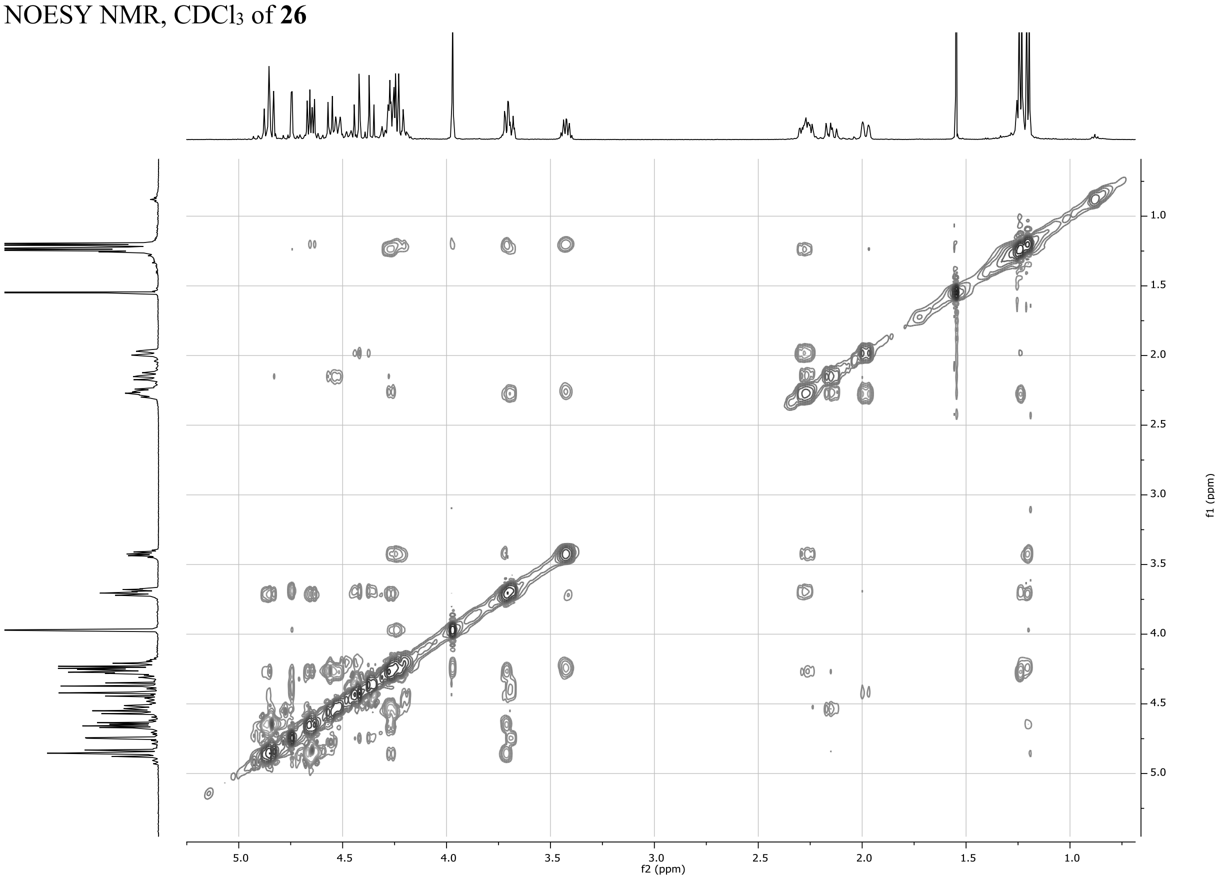

HMBC NMR, $CDCl_3$ of **26**

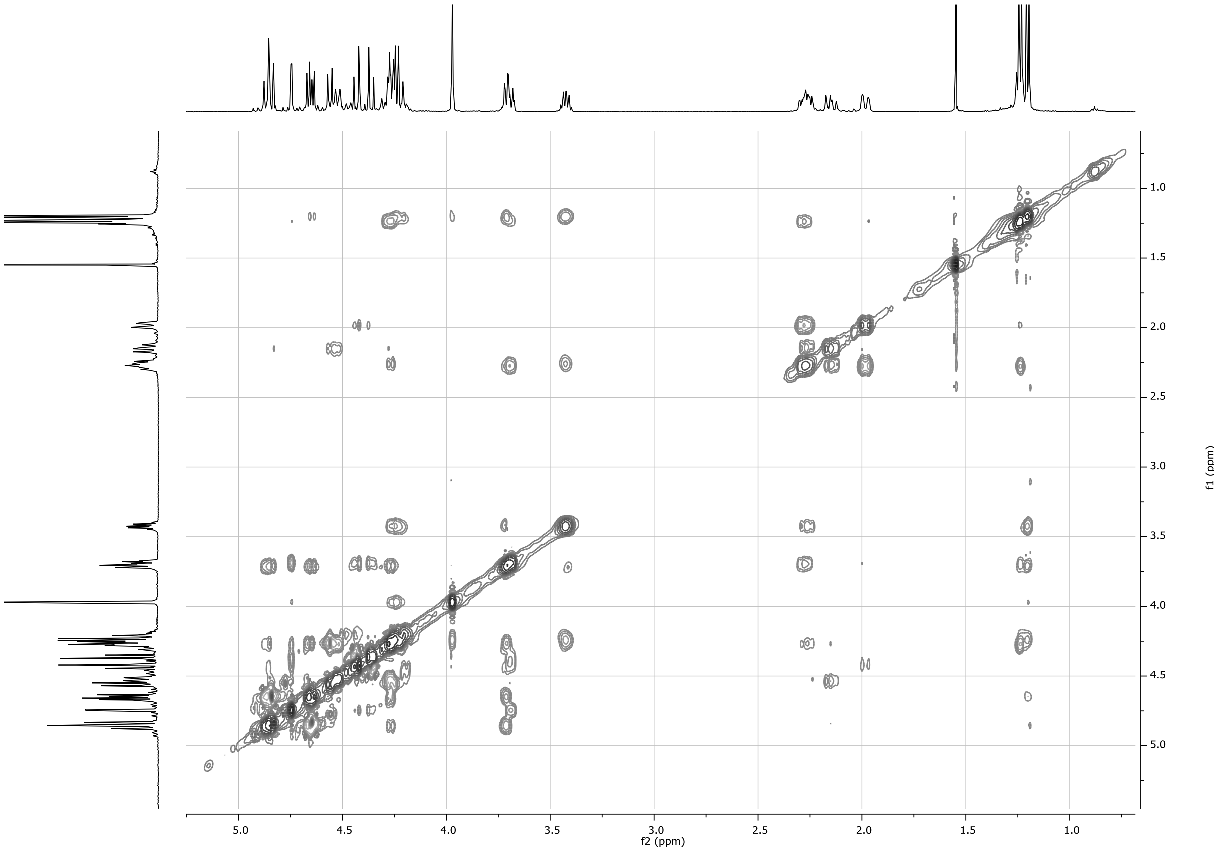

^{1}H NMR, 400 MHz, $CDCl_3$ of **S68**

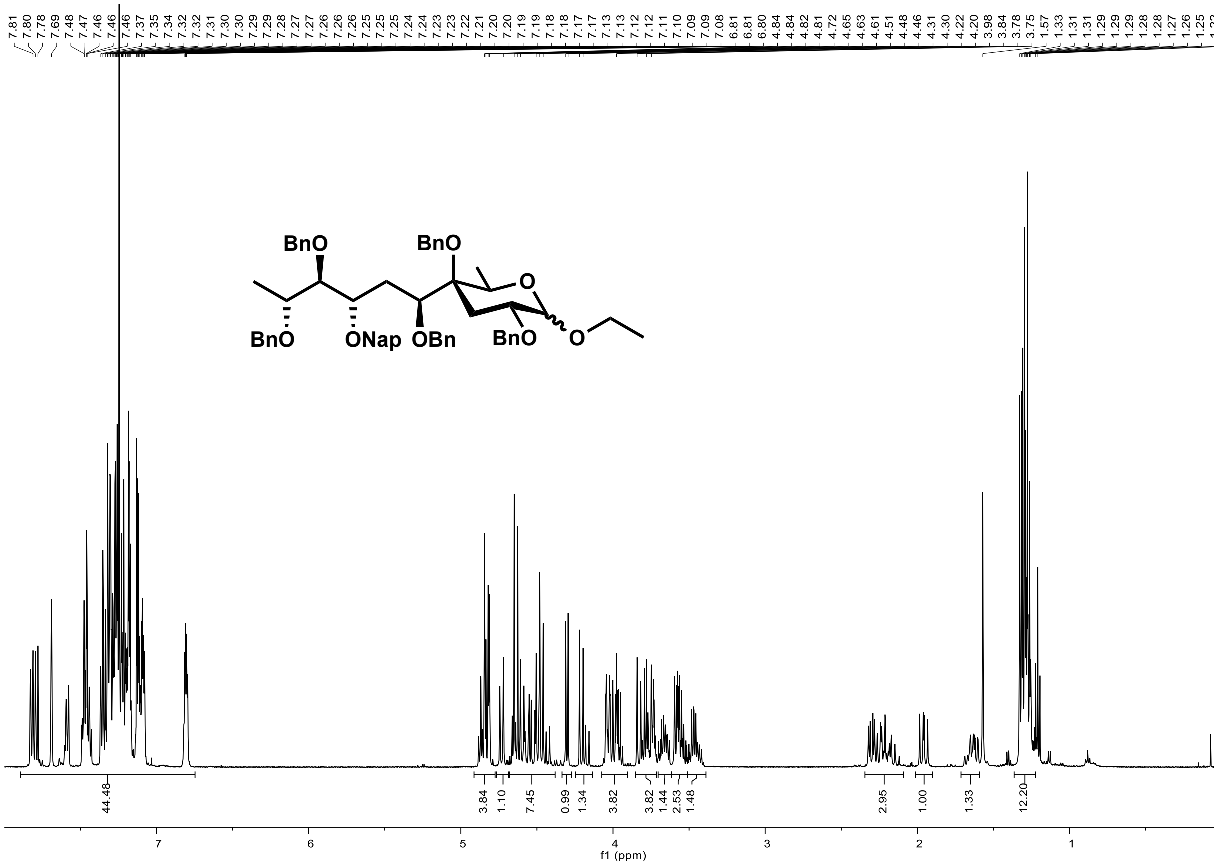

^{13}C NMR, 101 MHz, $CDCl_3$ of **S68**

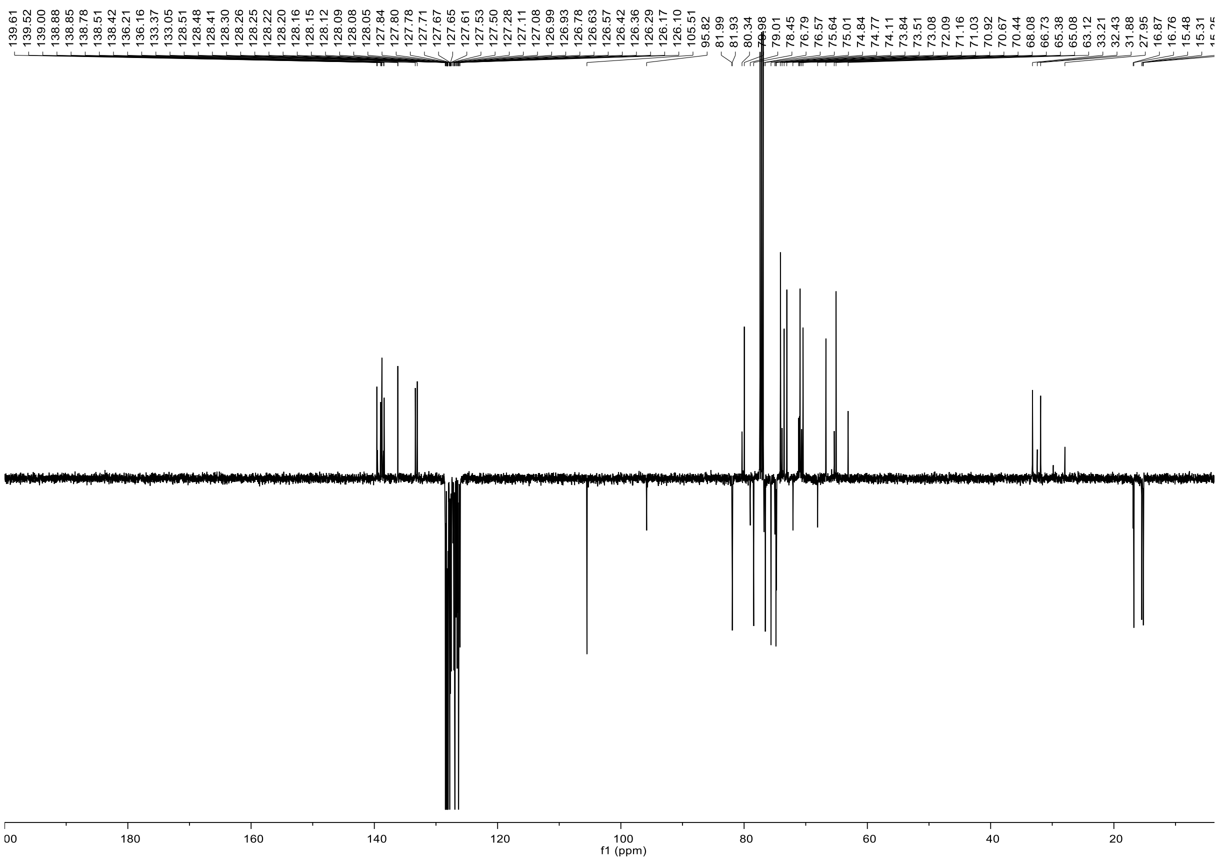

HH-COSY NMR, $CDCl_3$ of **S68**

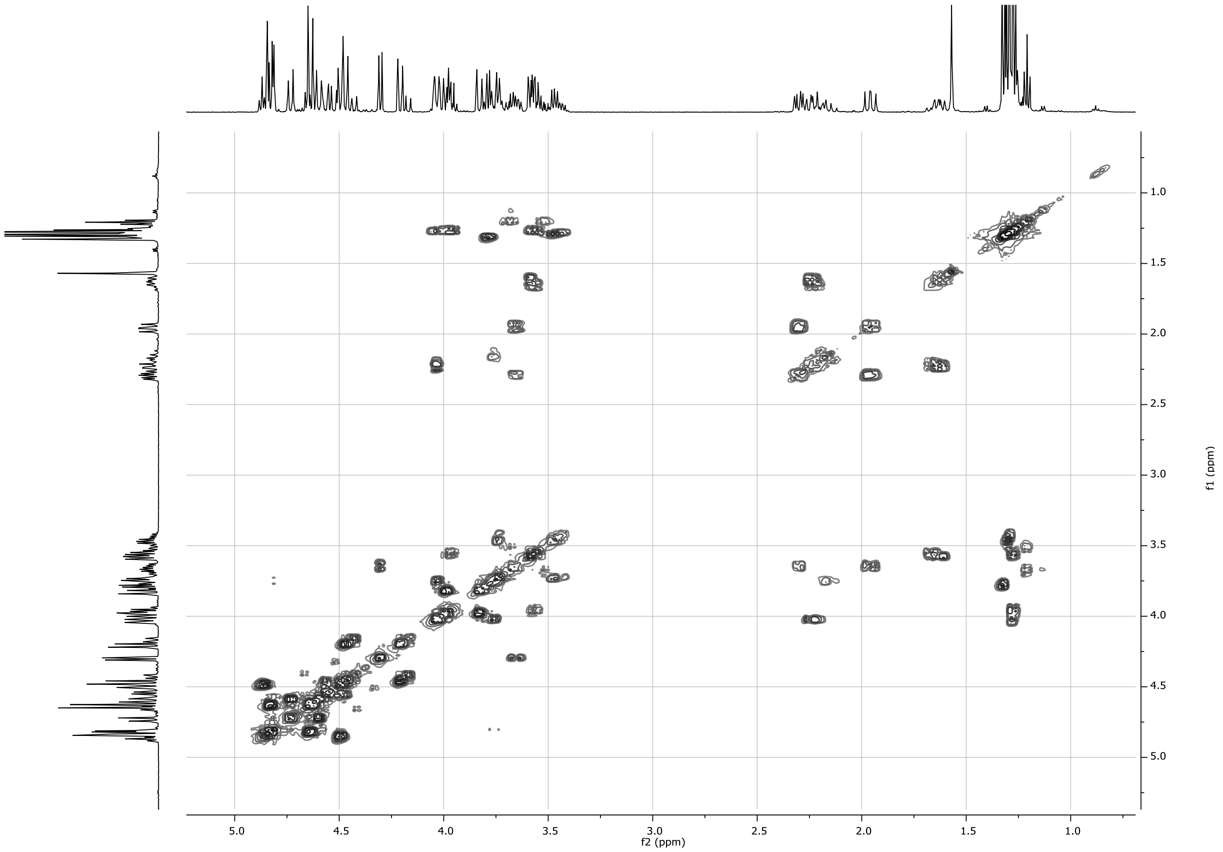

HSQC NMR, $CDCl_3$ of **S68**

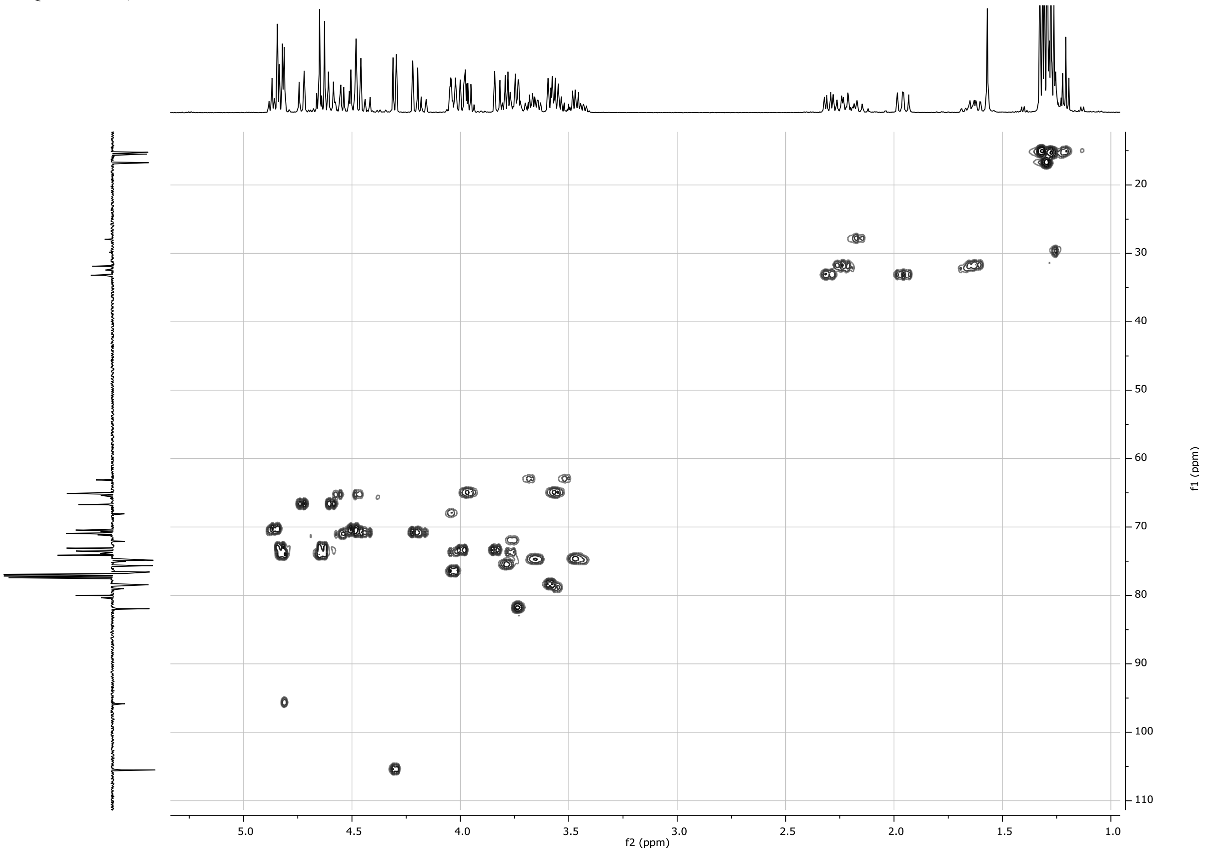

^{1}H NMR, 400 MHz, $CDCl_3$ of **S69**

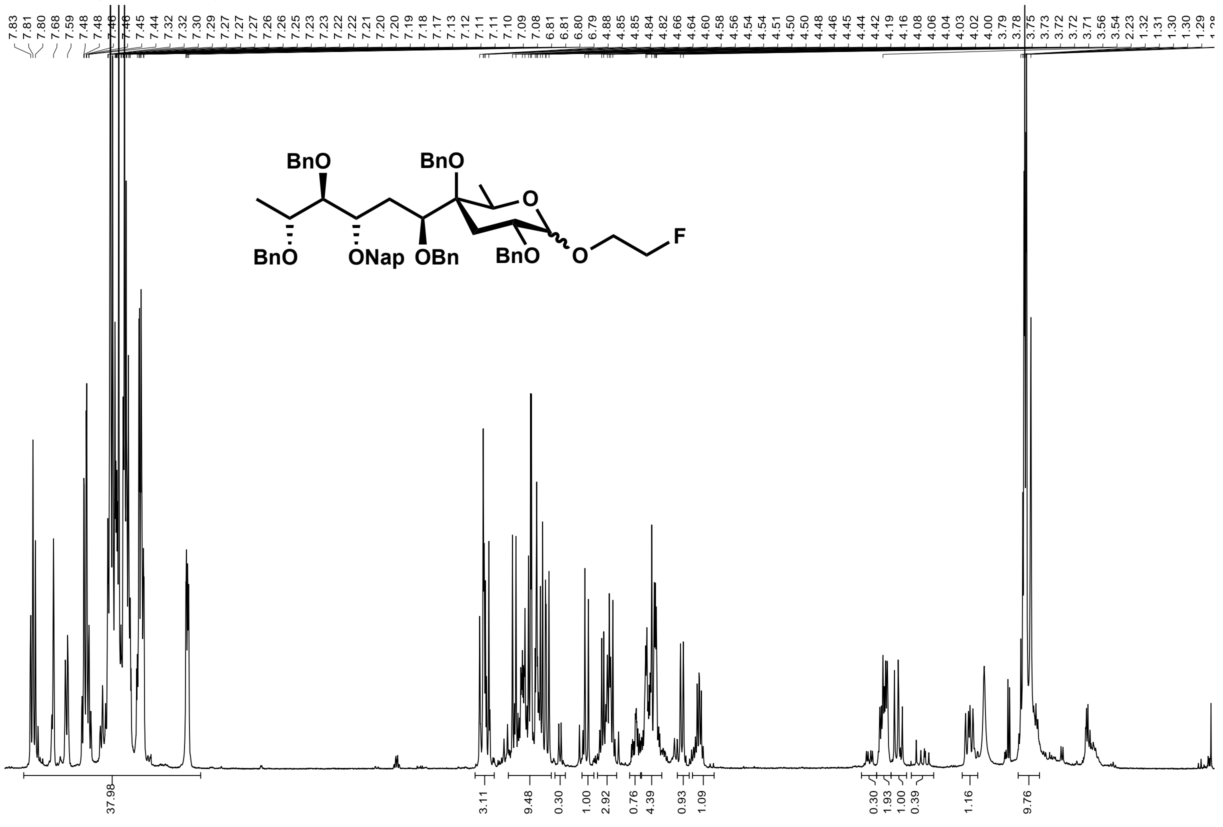

^{13}C NMR, 101 MHz, $CDCl_3$ of **S69**

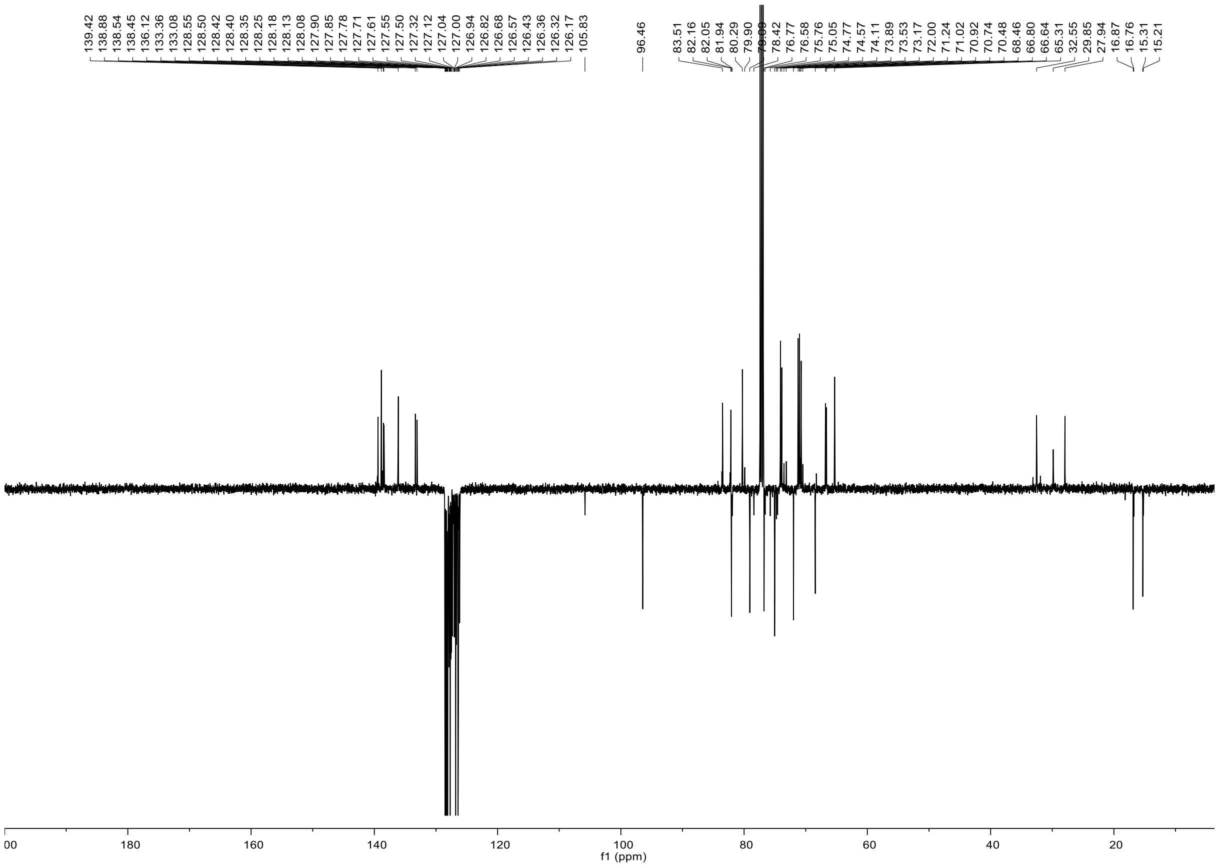

HH-COSY NMR, $CDCl_3$ of **S69**

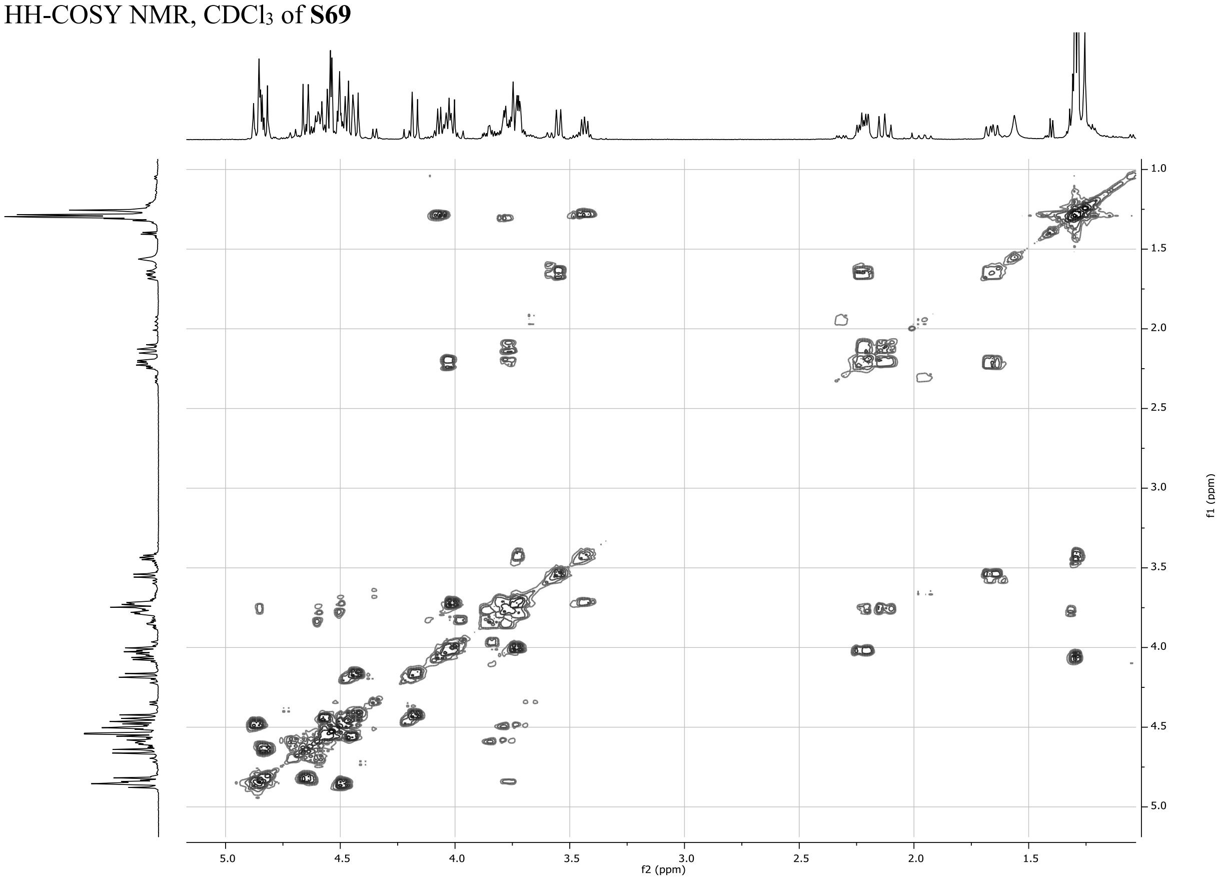

HSQC NMR, $CDCl_3$ of **S69**

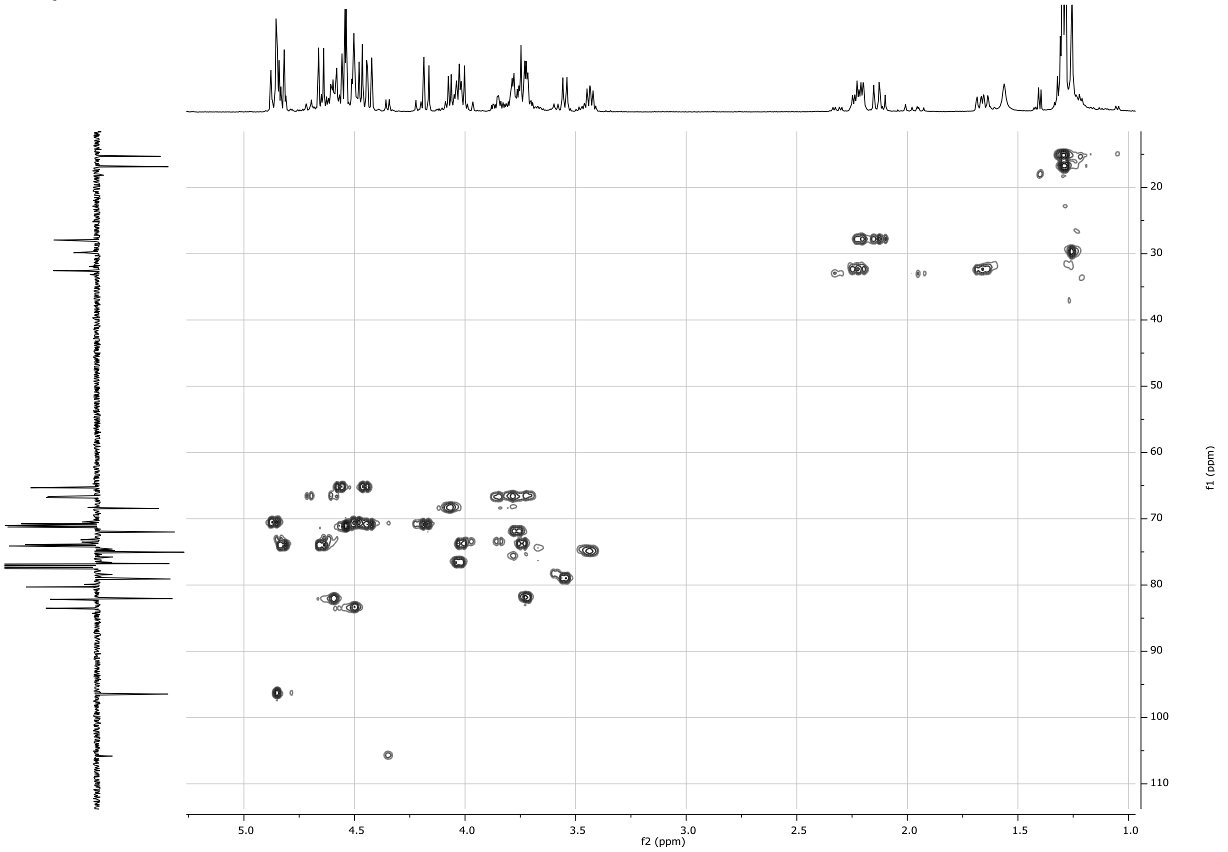

^{1}H NMR, 400 MHz, $CDCl_3$ of **S70**

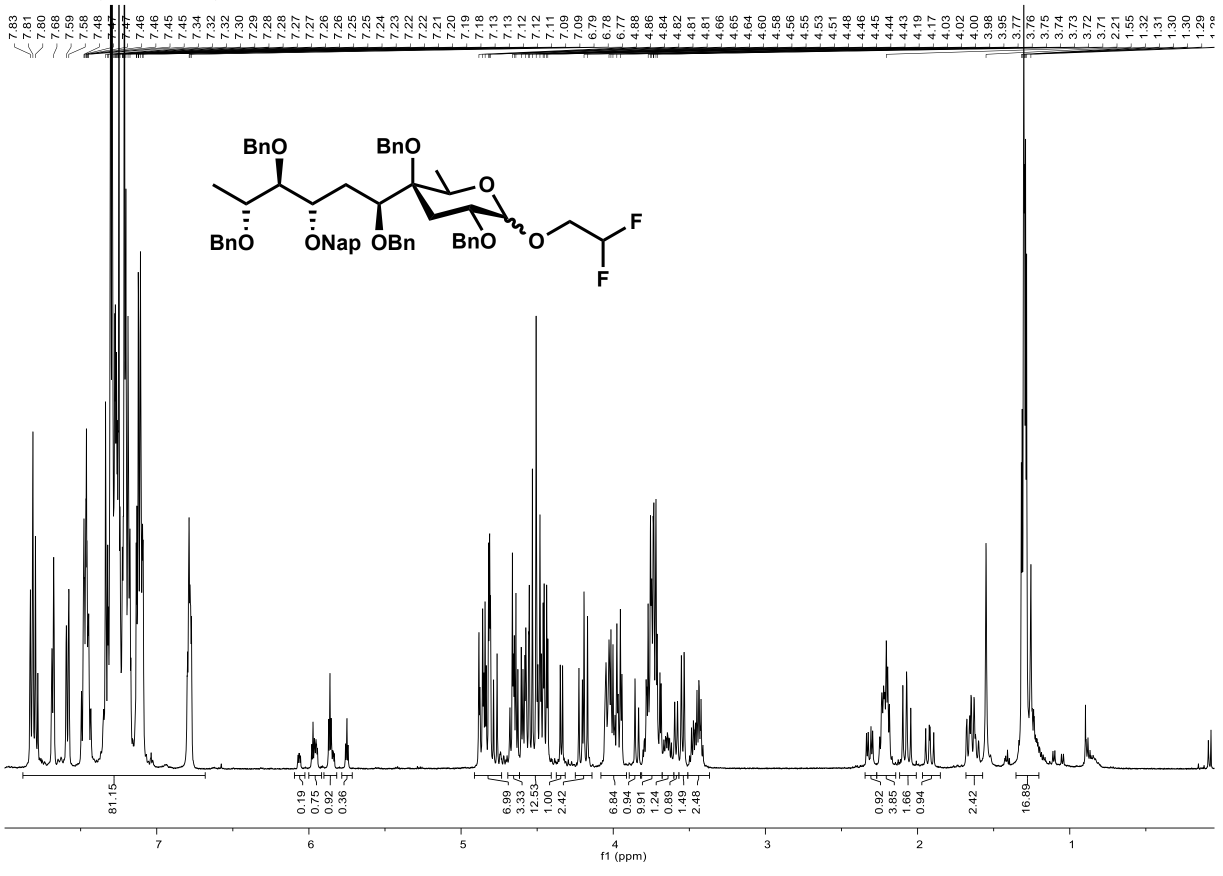

^{13}C NMR, 101 MHz, CDCl$_3$ of **S70**

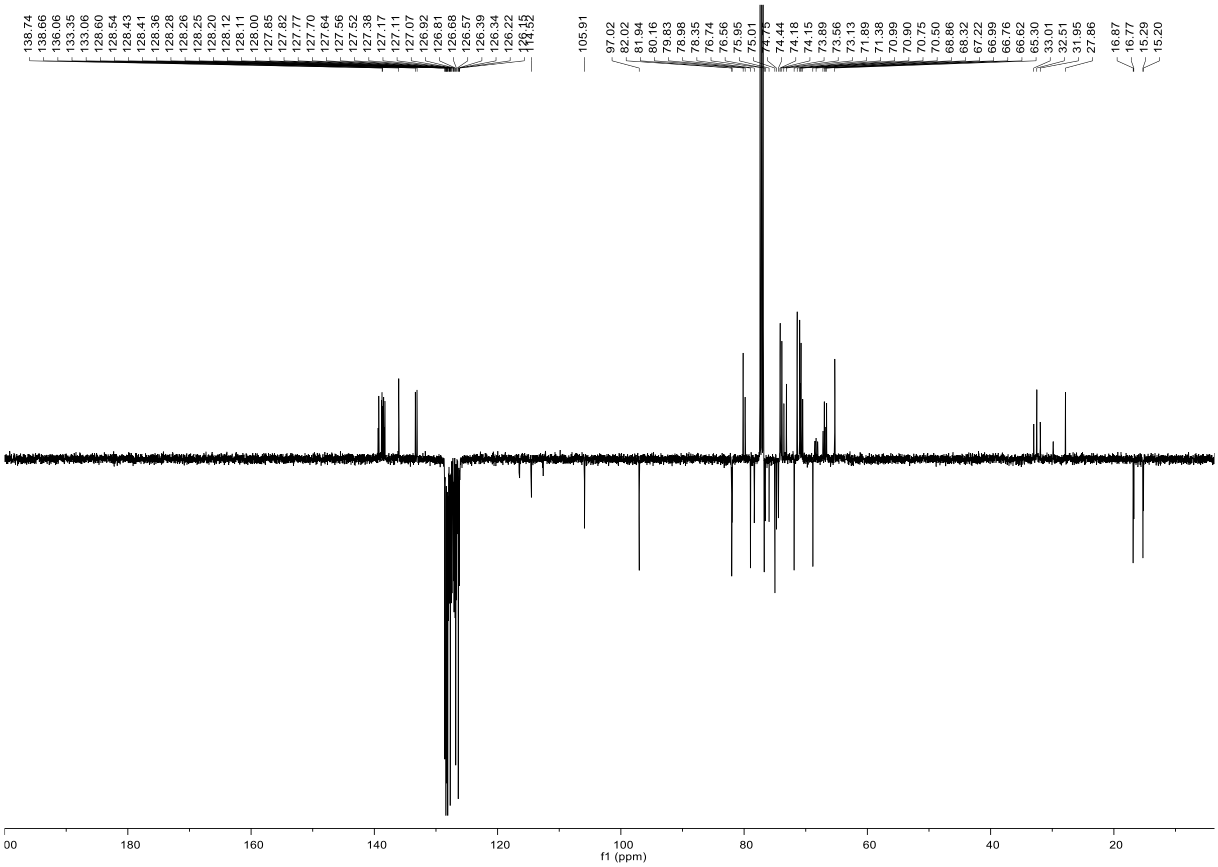

HH-COSY NMR, CDCl$_3$ of **S70**

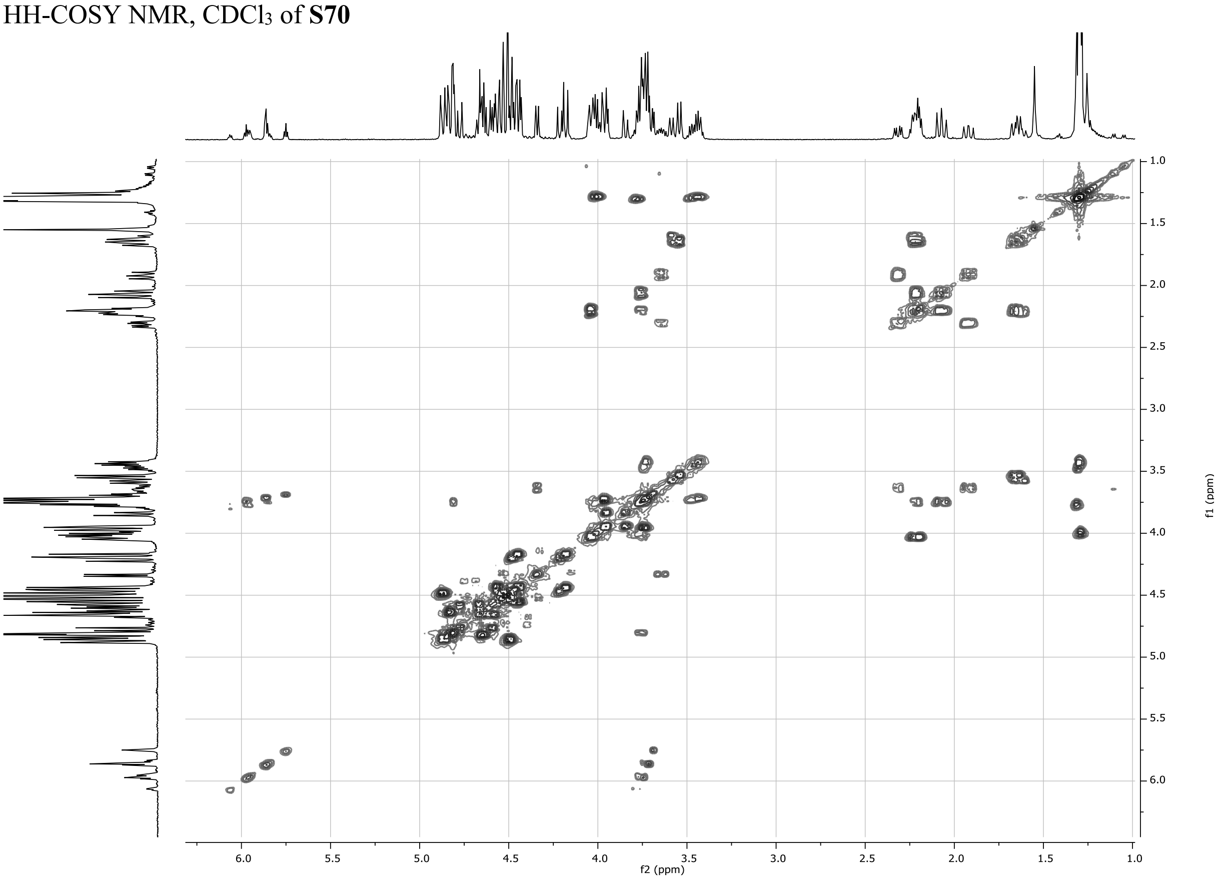

HSQC NMR, $CDCl_3$ of **S70**

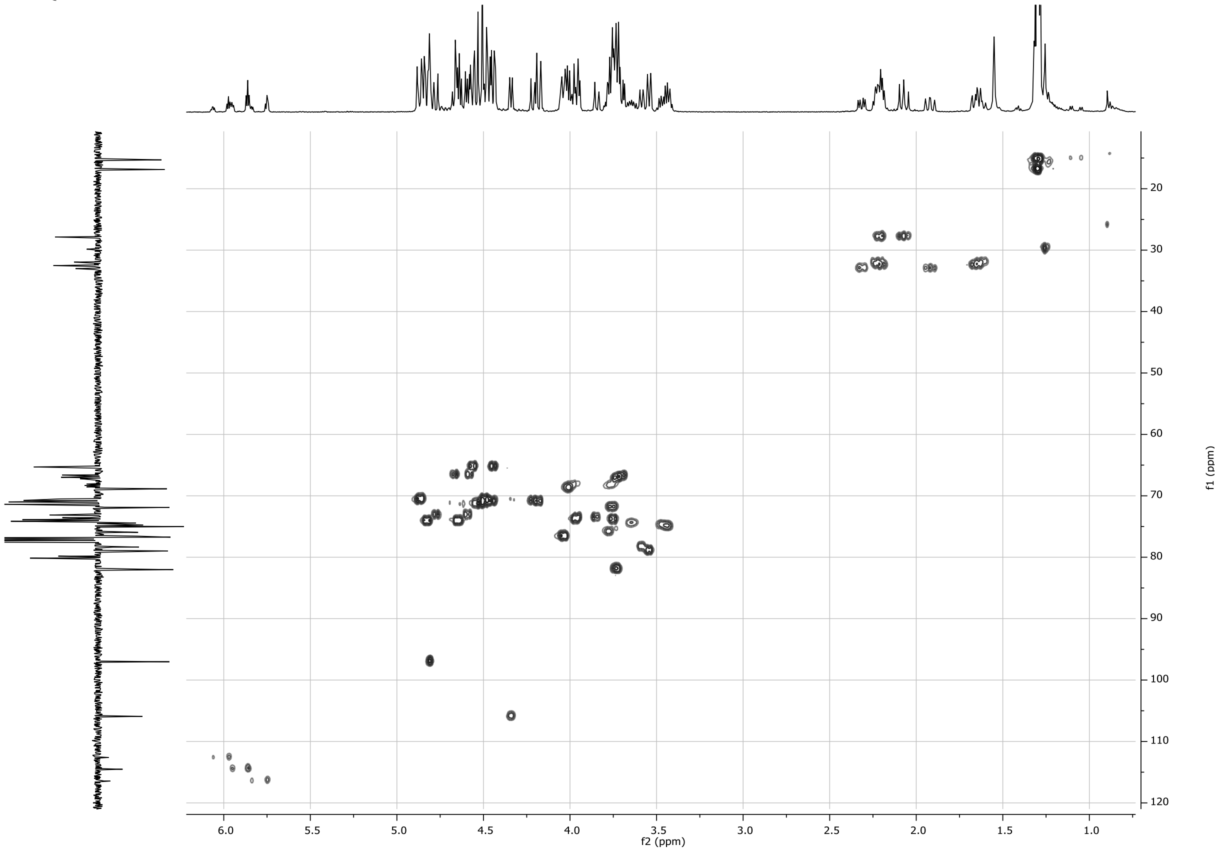

^{1}H NMR, 400 MHz, $CDCl_3$ of **S71**

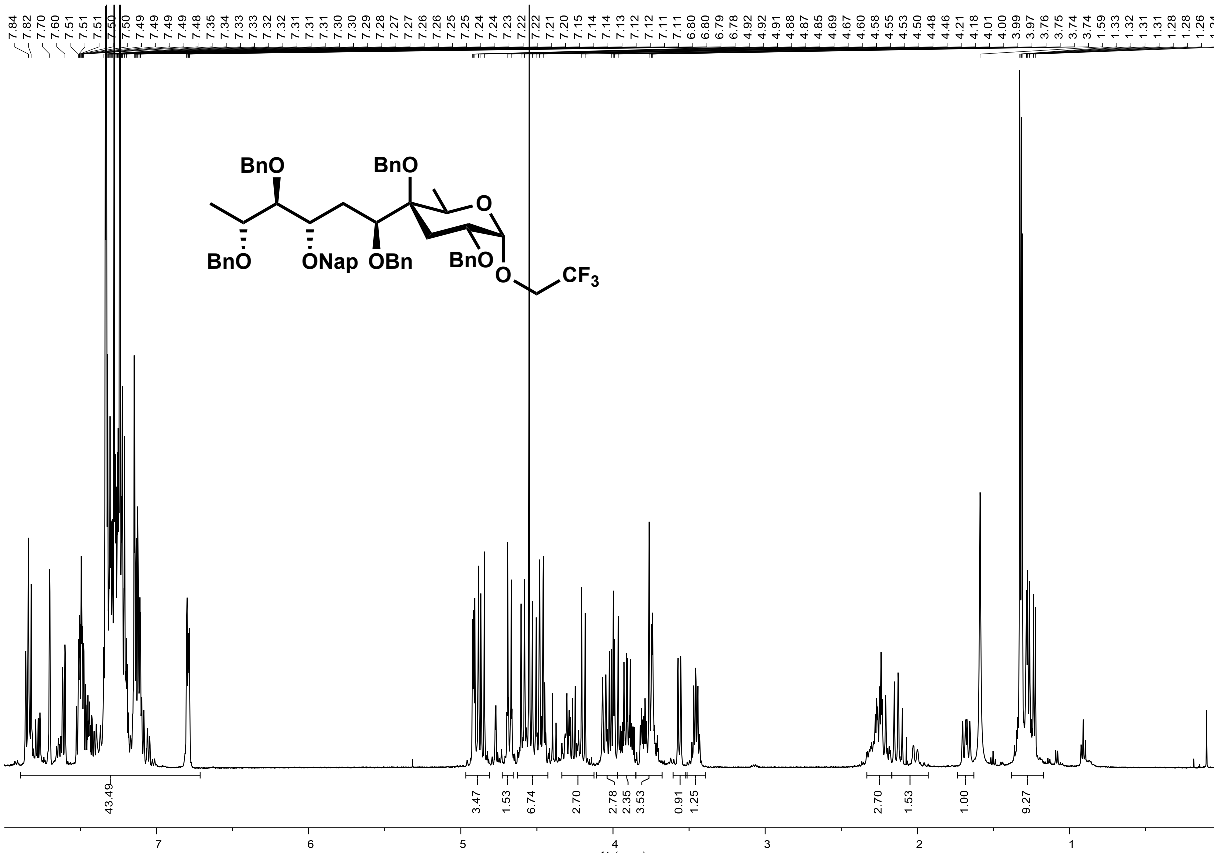

^{13}C NMR, 101 MHz, $CDCl_3$ of **S71**

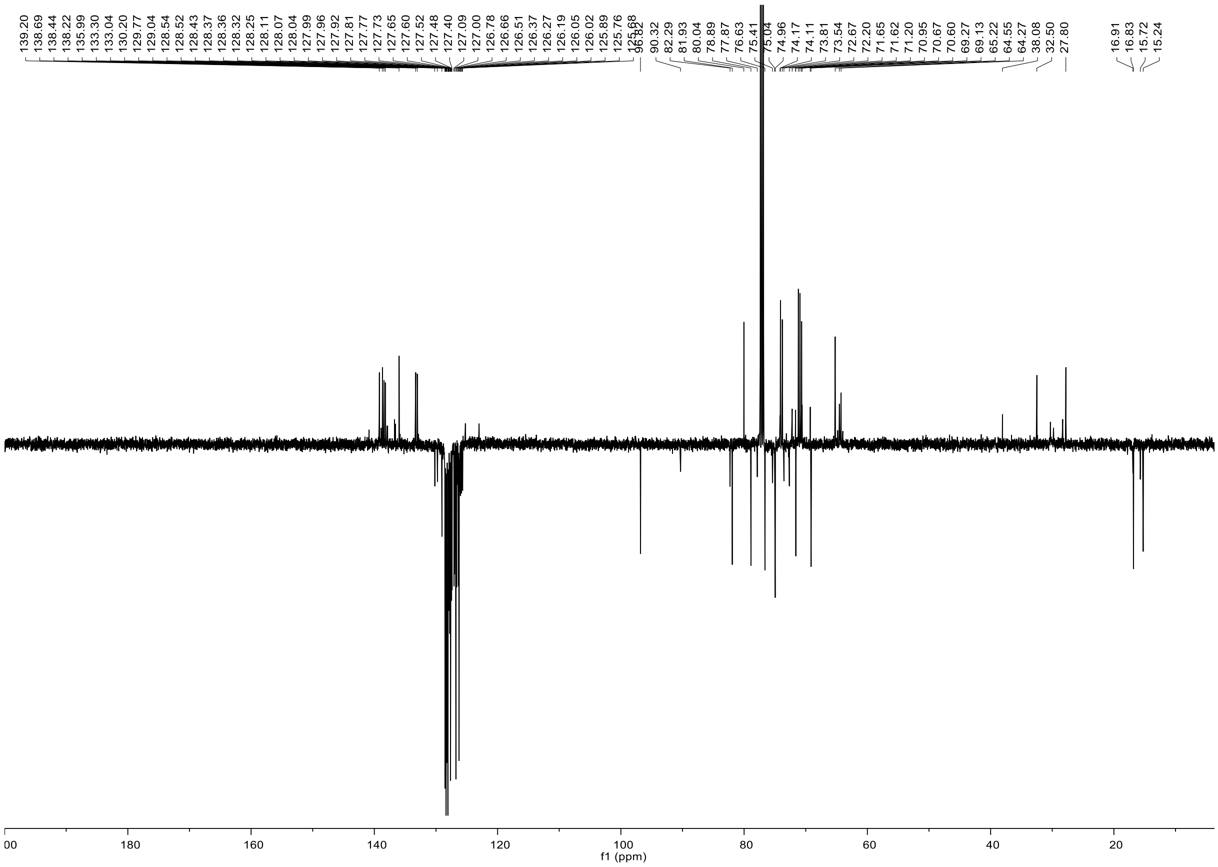

HH-COSY NMR, $CDCl_3$ of **S71**

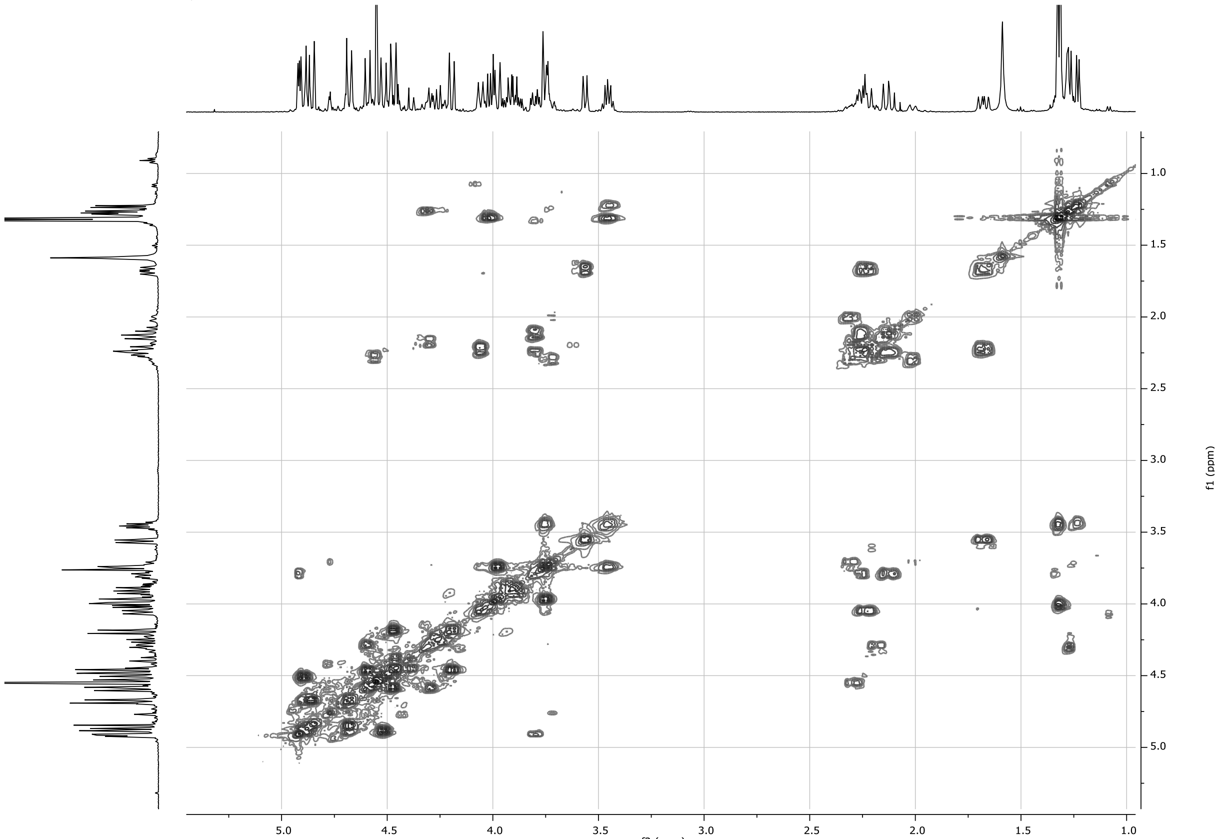

HSQC NMR, $CDCl_3$ of **S71**

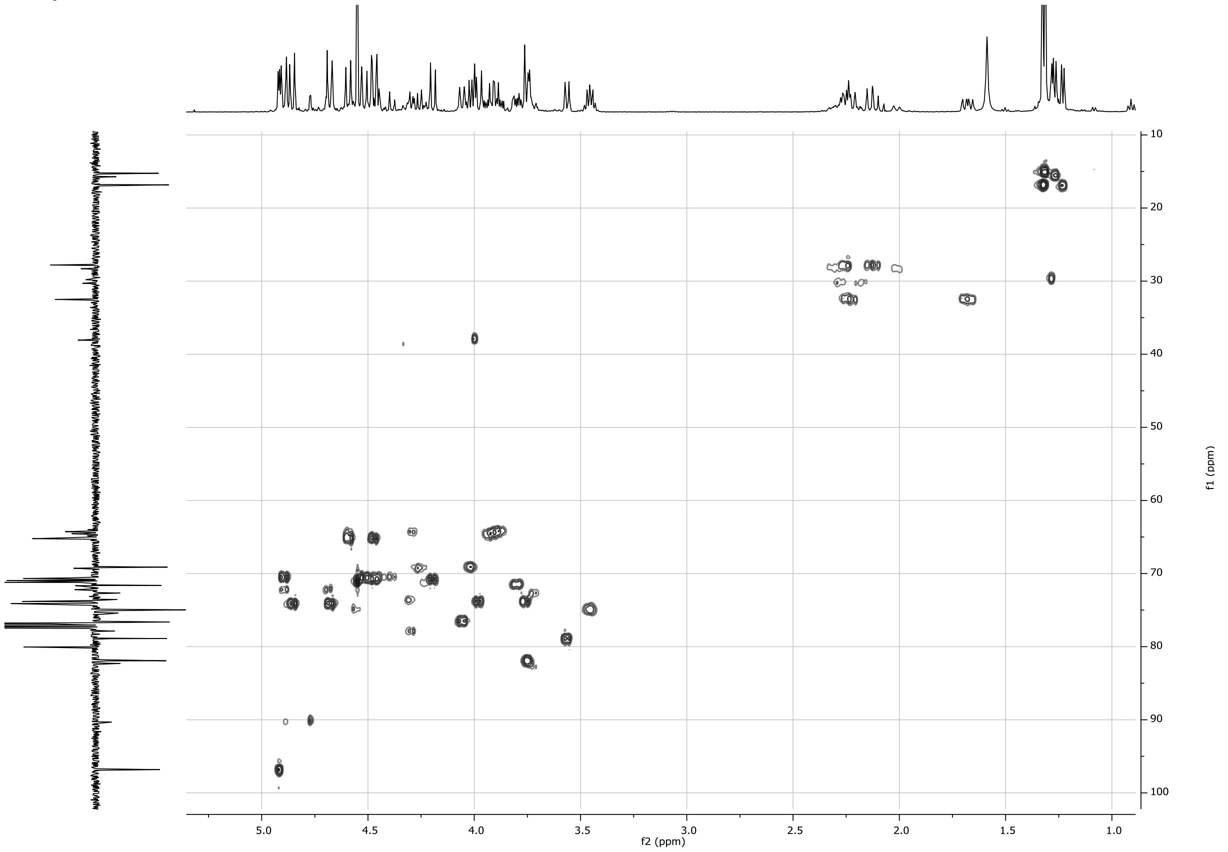

^{1}H NMR, 400 MHz, $CDCl_3$ of **S72**

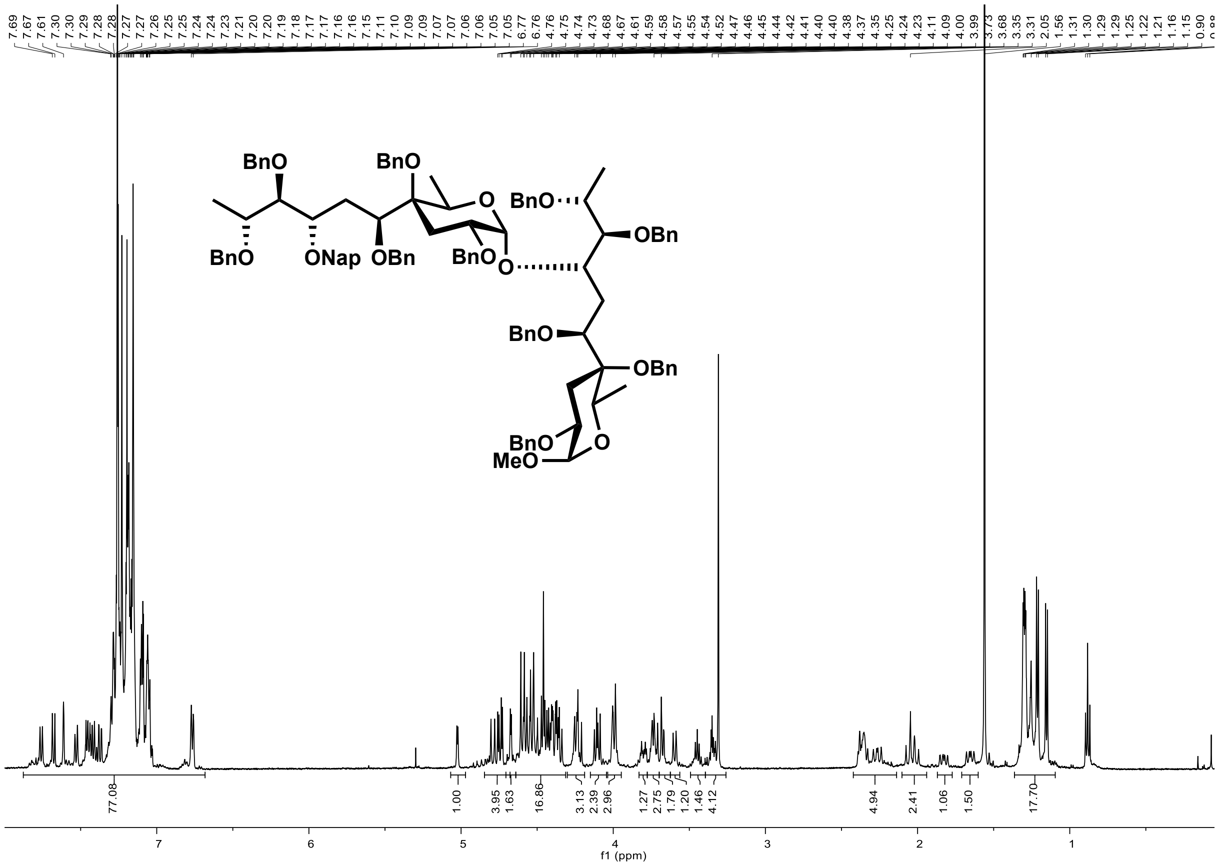

^{13}C NMR, 101 MHz, $CDCl_3$ of **S72**

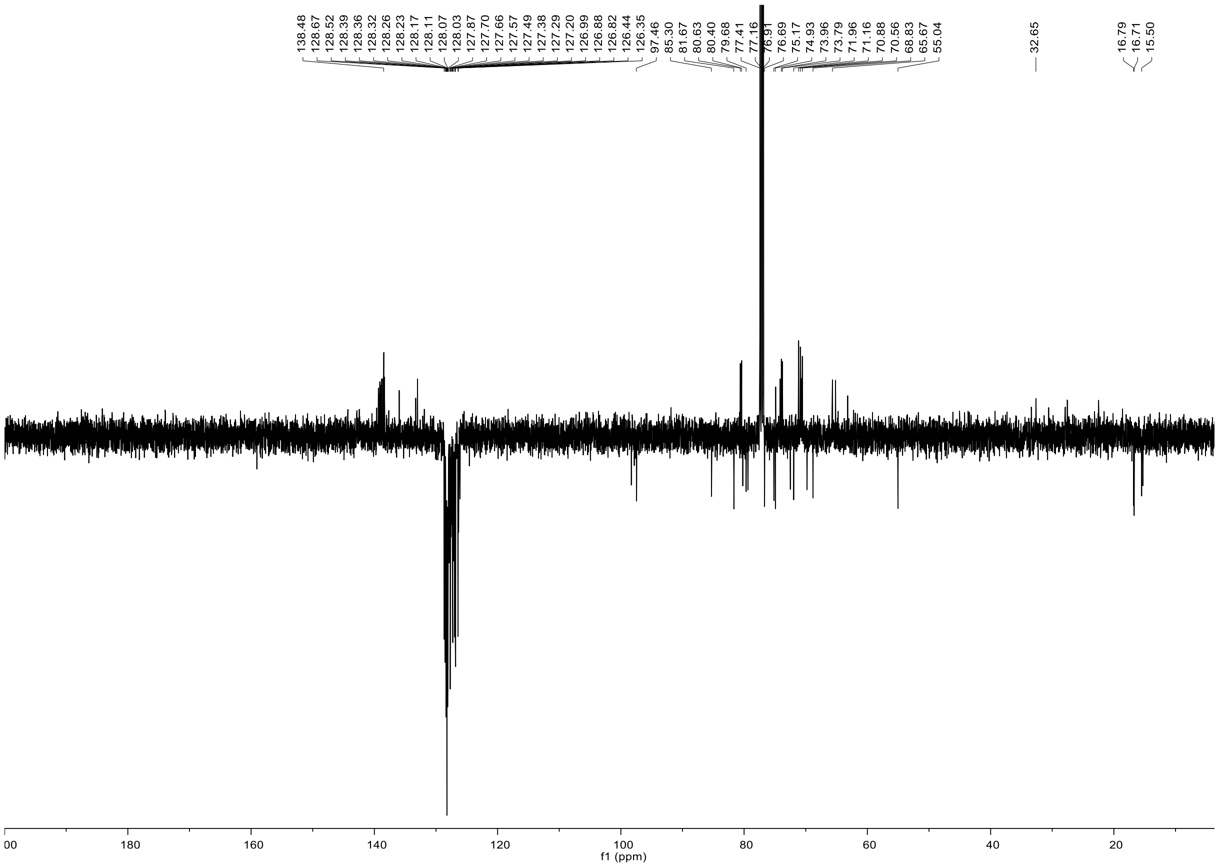

HH-COSY NMR, $CDCl_3$ of **S72**

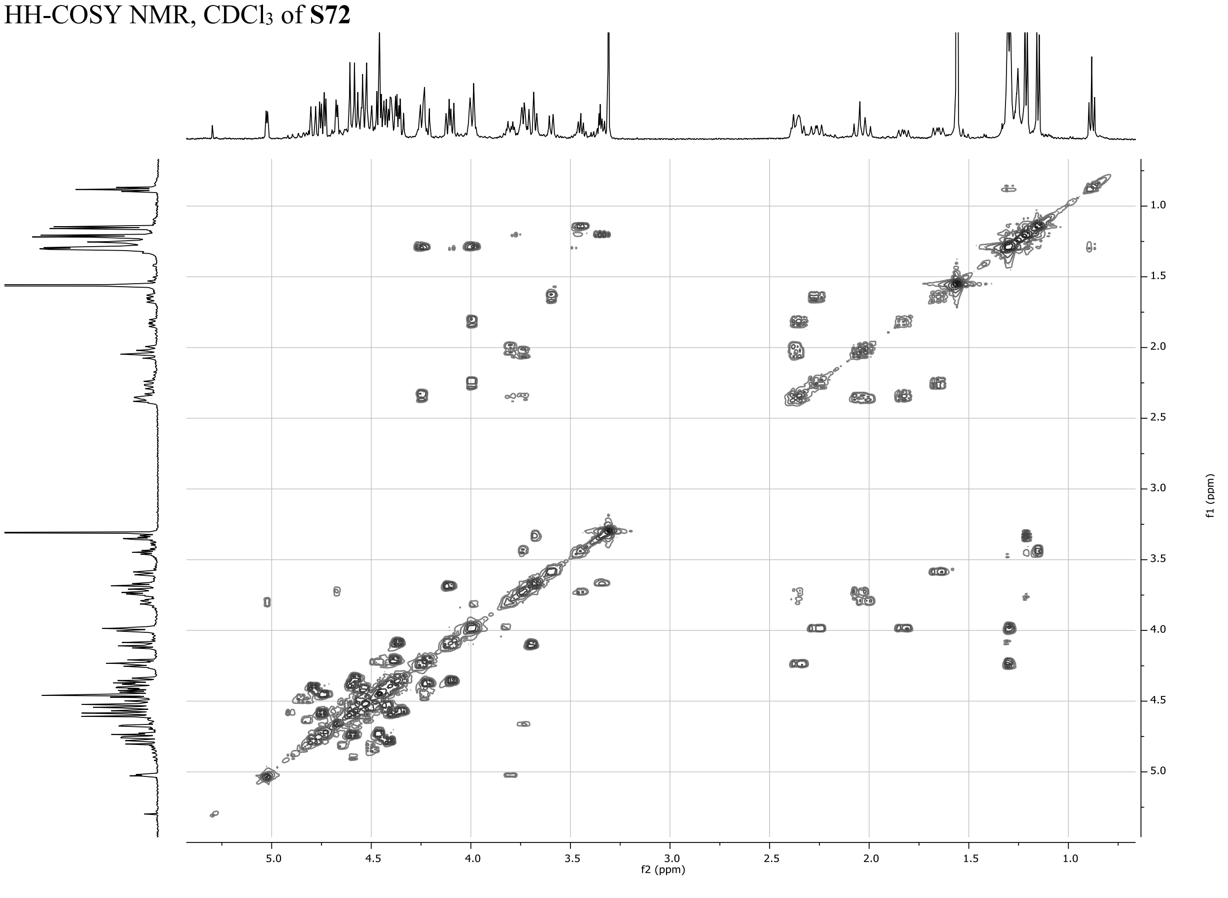

HSQC NMR, $CDCl_3$ of **S72**

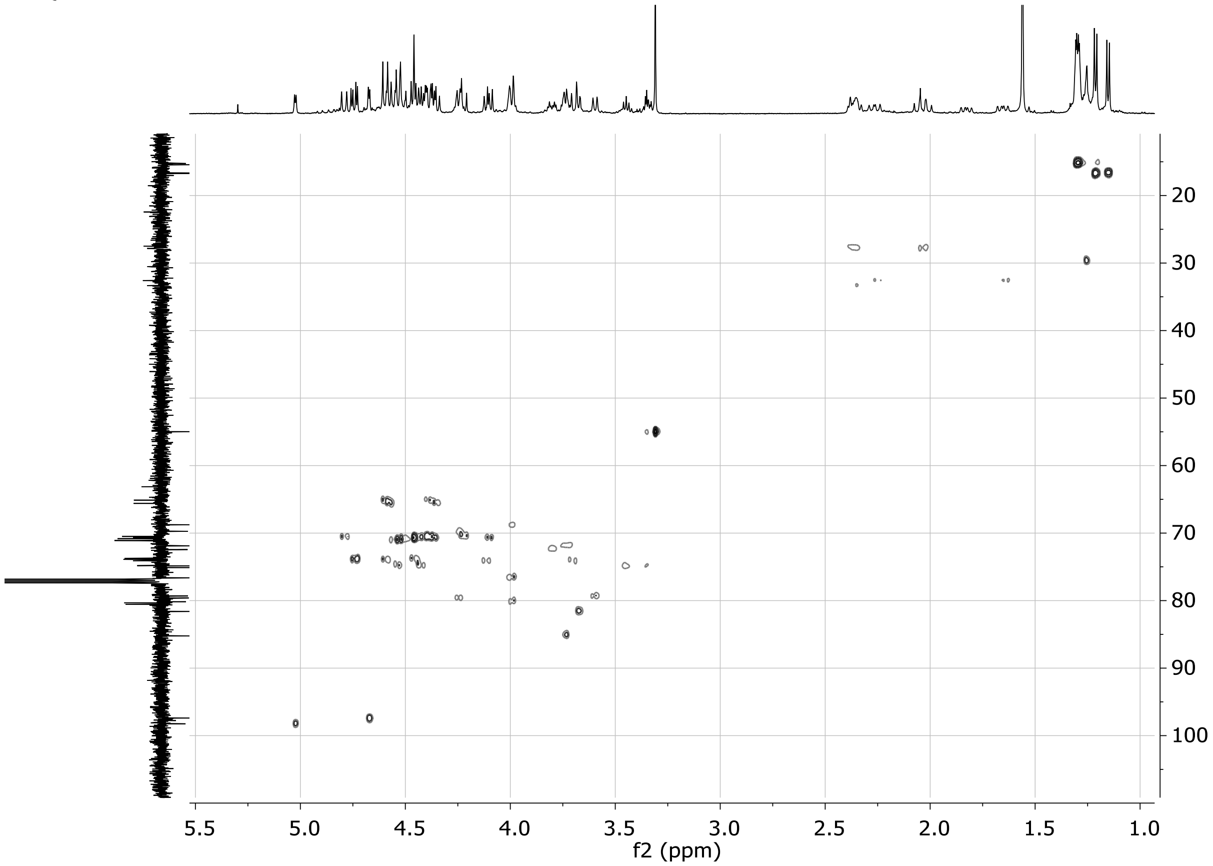

HMBC NMR, $CDCl_3$ of **S72**

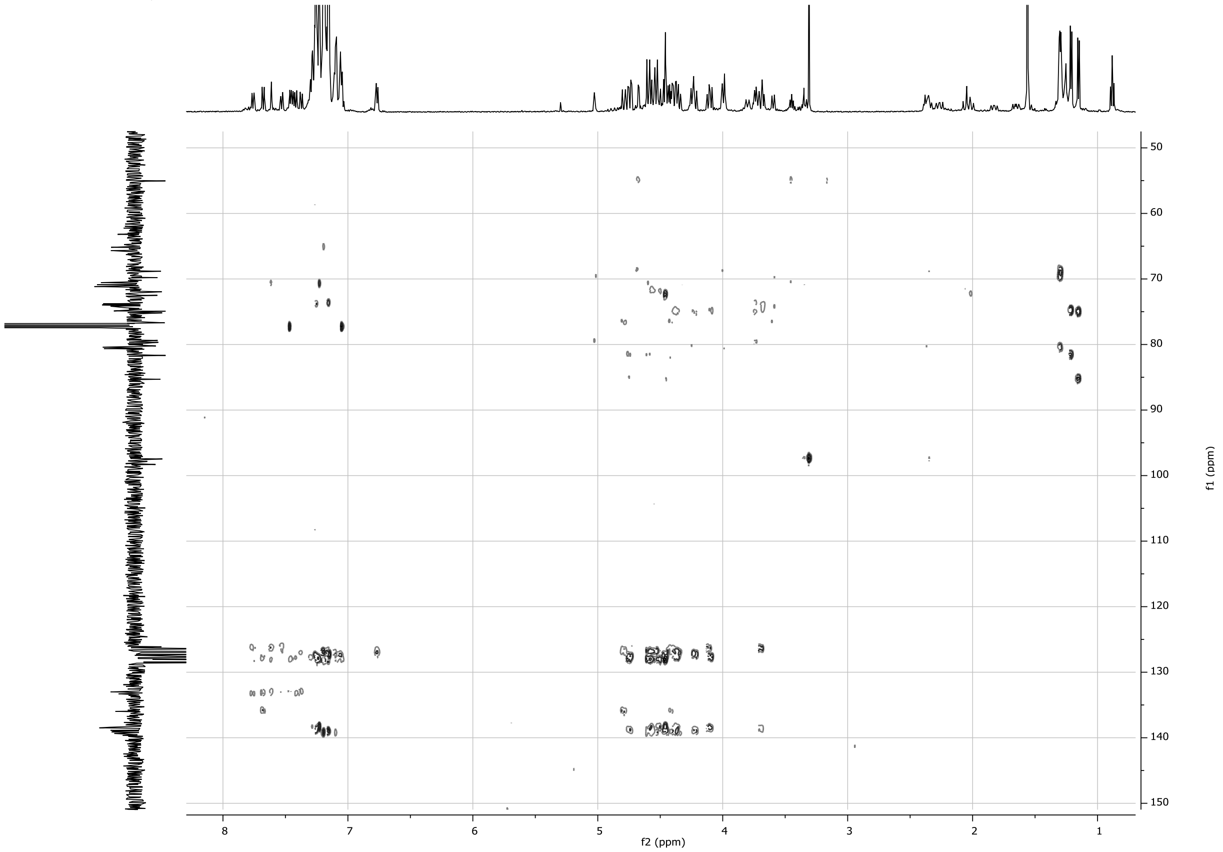

HMBC-GATED NMR, $CDCl_3$ of **S72**

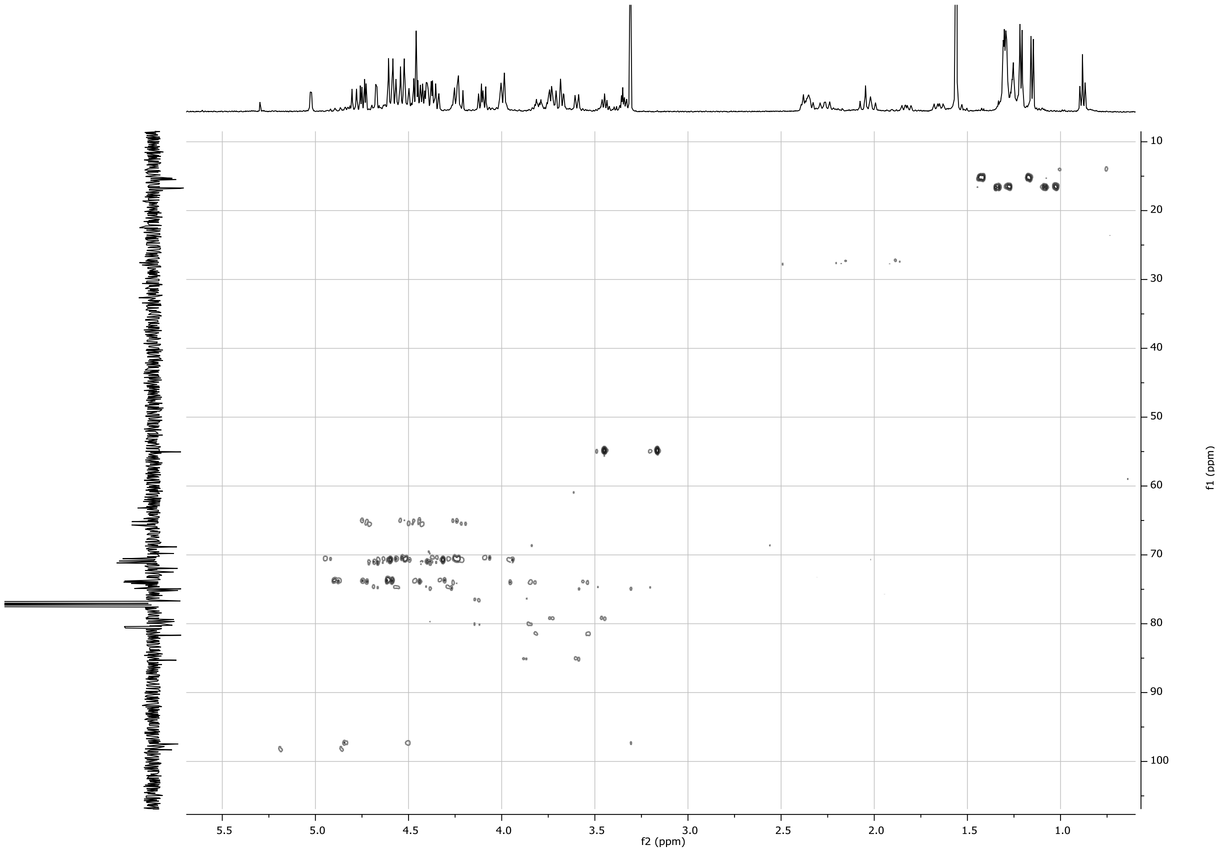

^{1}H NMR, 400 MHz, $CDCl_3$ of **S73**

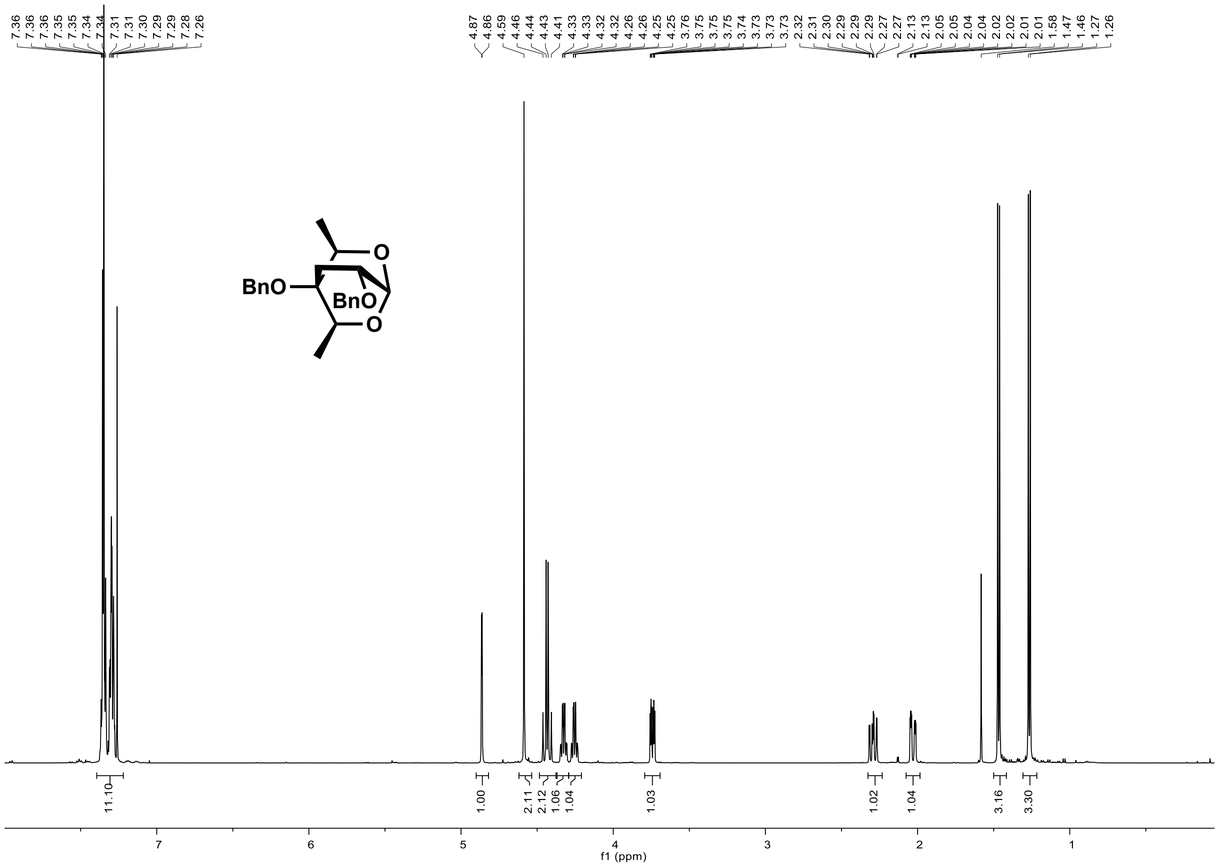

^{13}C NMR, 101 MHz, $CDCl_3$ of **S73**

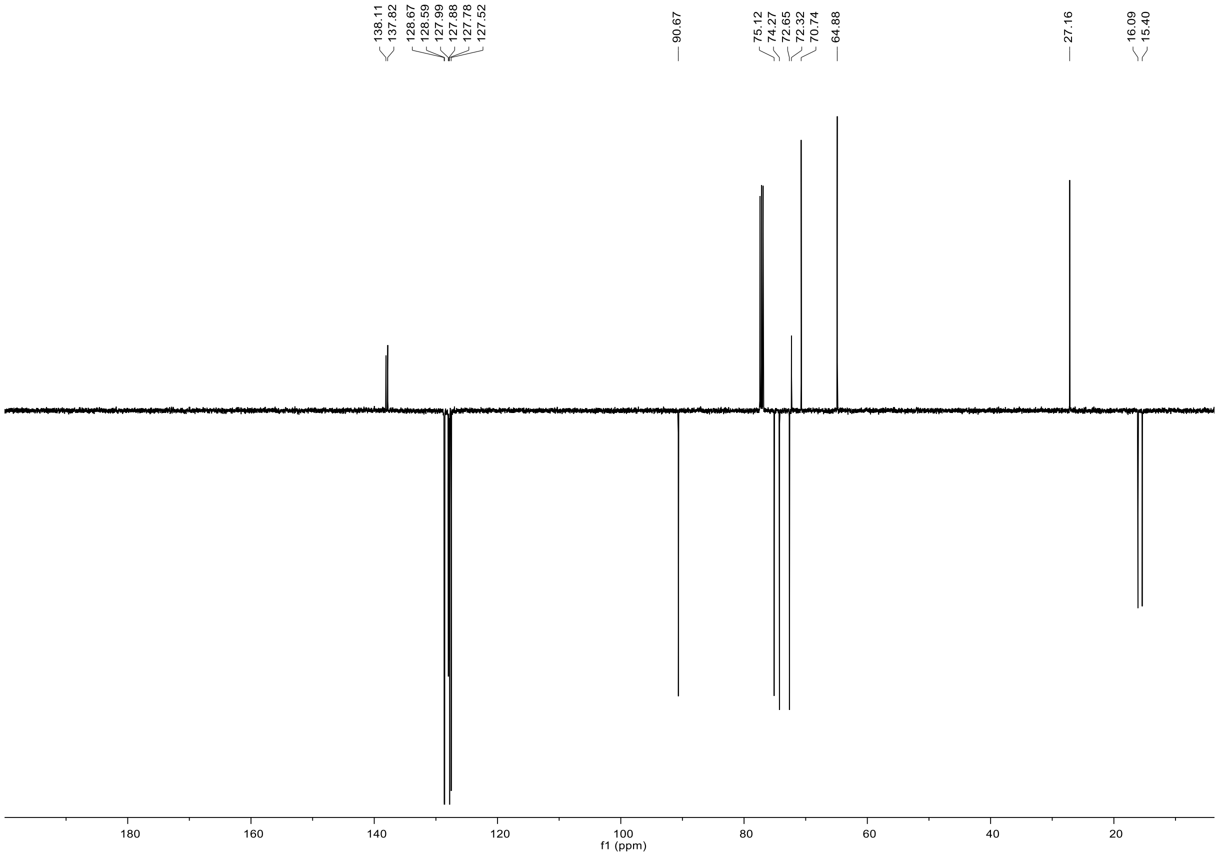

HH-COSY NMR, $CDCl_3$ of **S73**

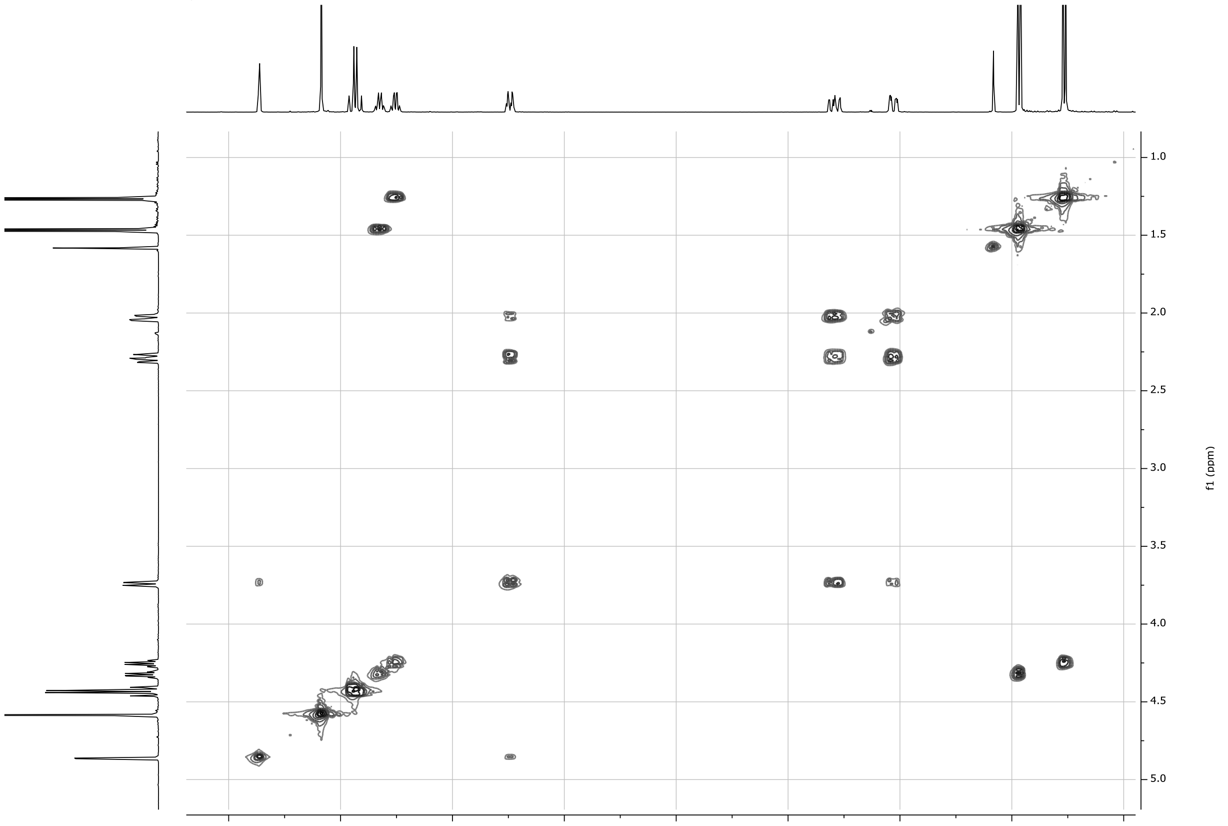

HSQC NMR, $CDCl_3$ of **S73**

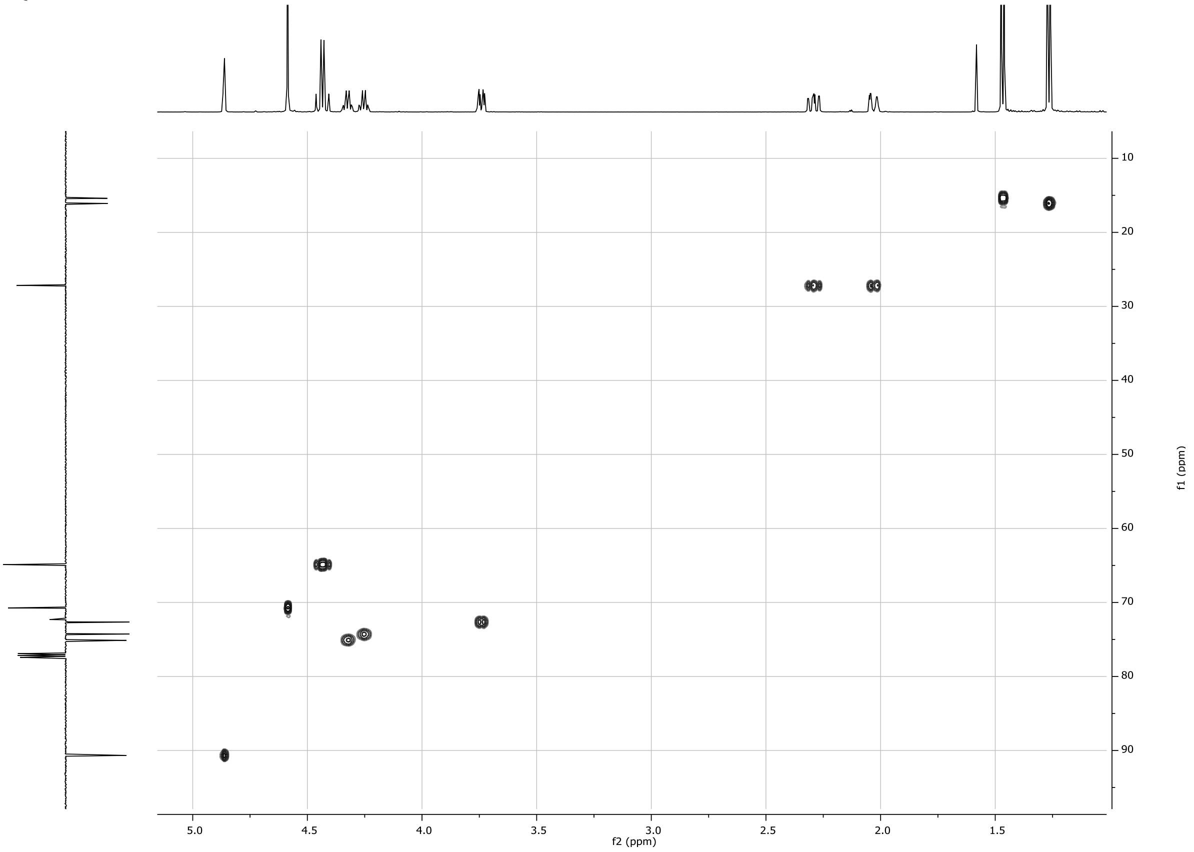

NOESY NMR, $CDCl_3$ of **S73**

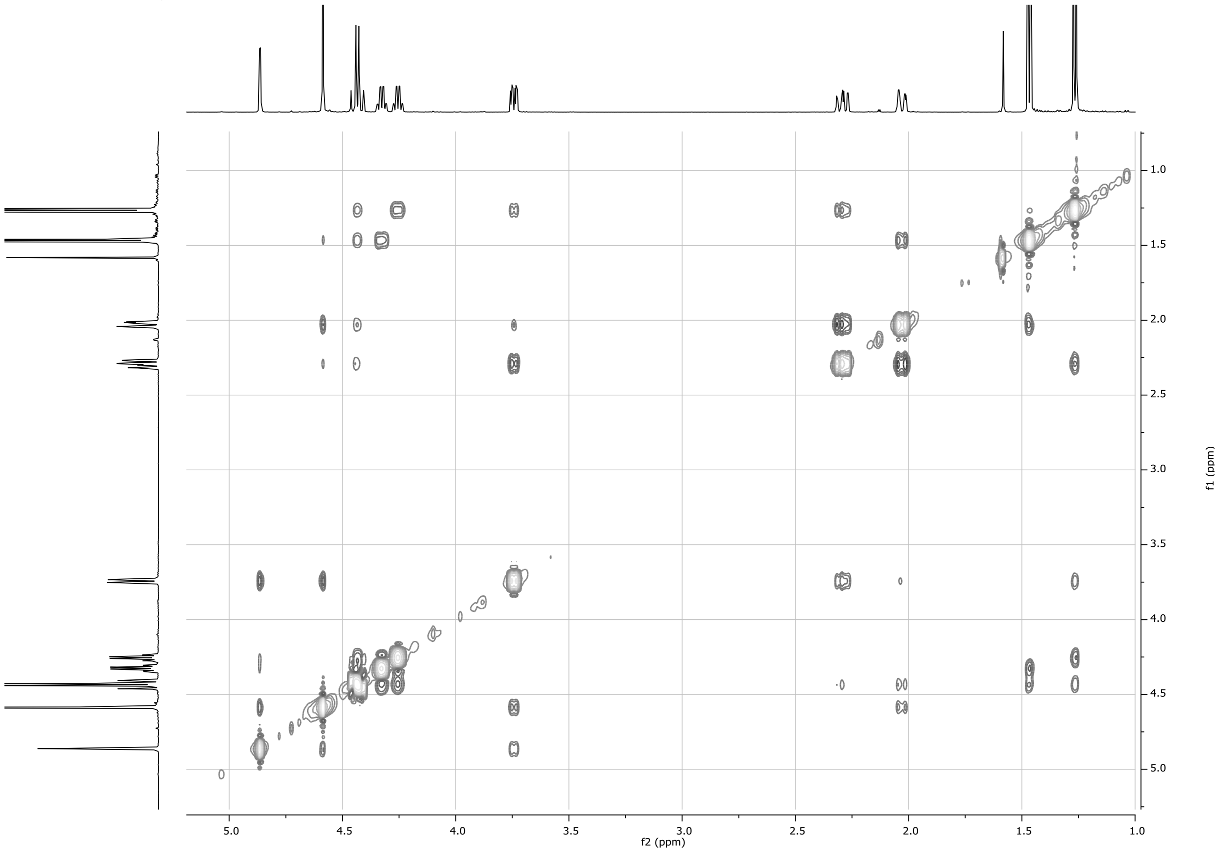

HMBC NMR, $CDCl_3$ of **S73**

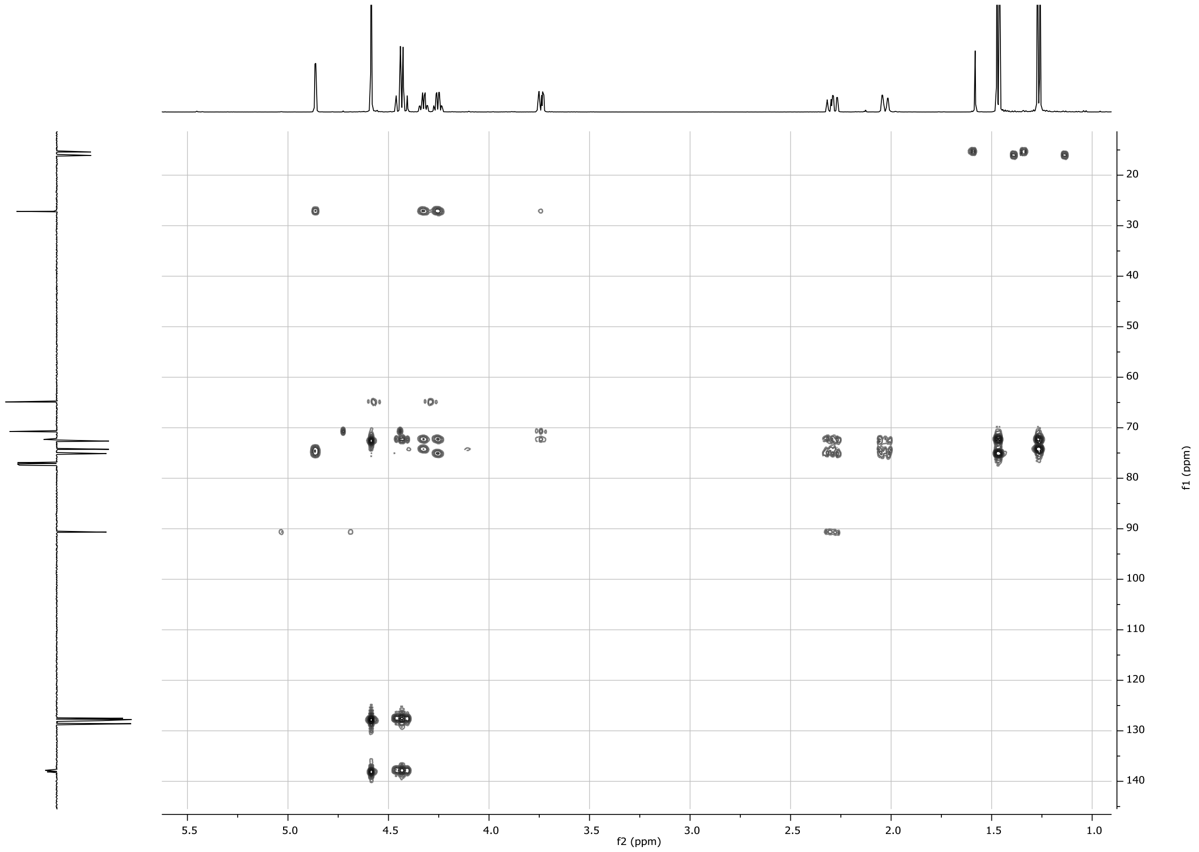

^1H NMR, 400 MHz, $CDCl_3$ of **S75**

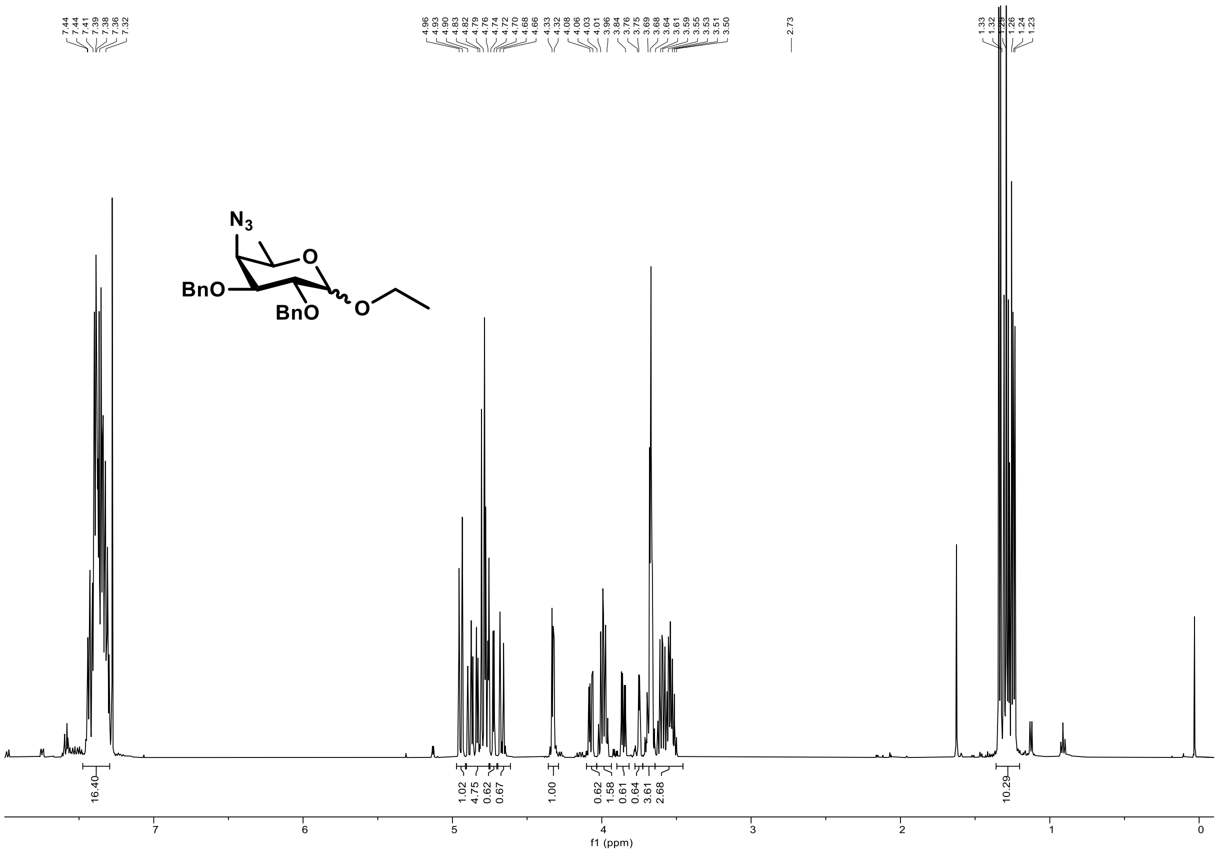

^{13}C NMR, 101 MHz, $CDCl_3$ of **S75**

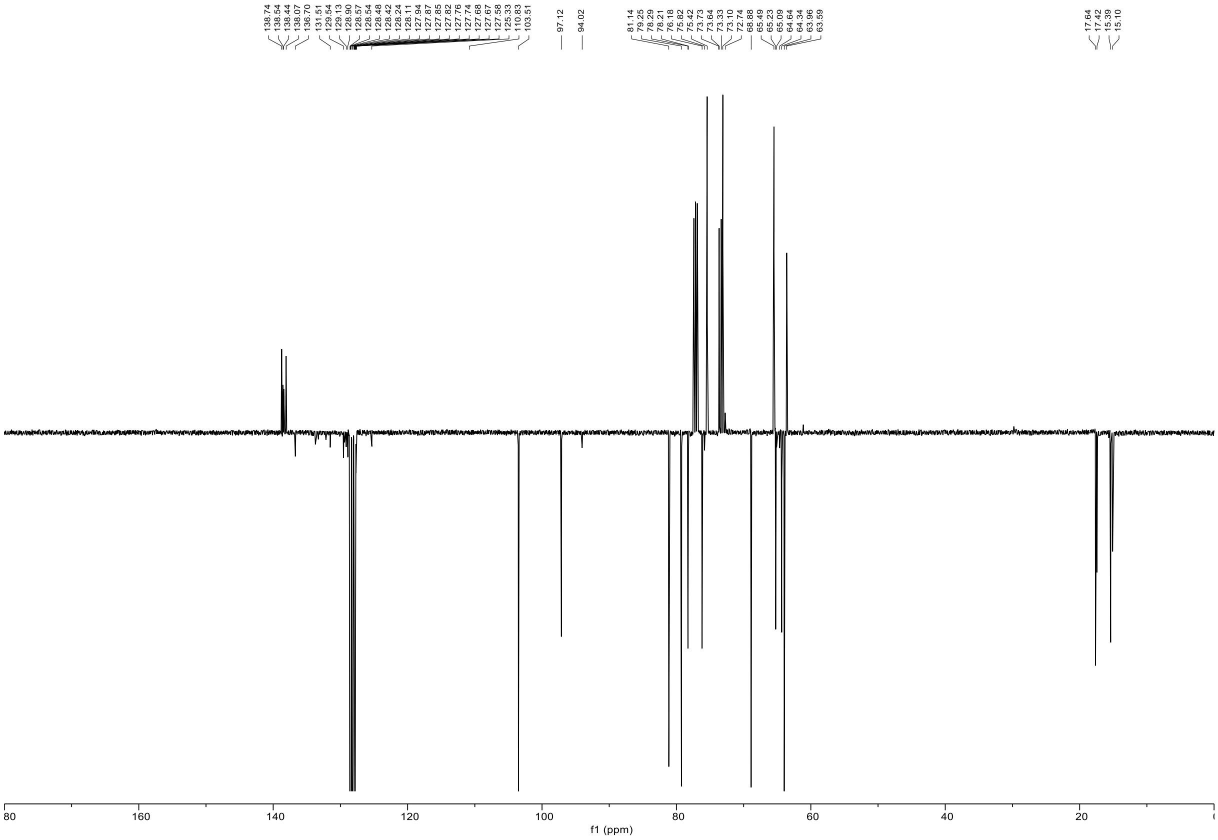

^1H NMR, 400 MHz, $CDCl_3$ of **S76**

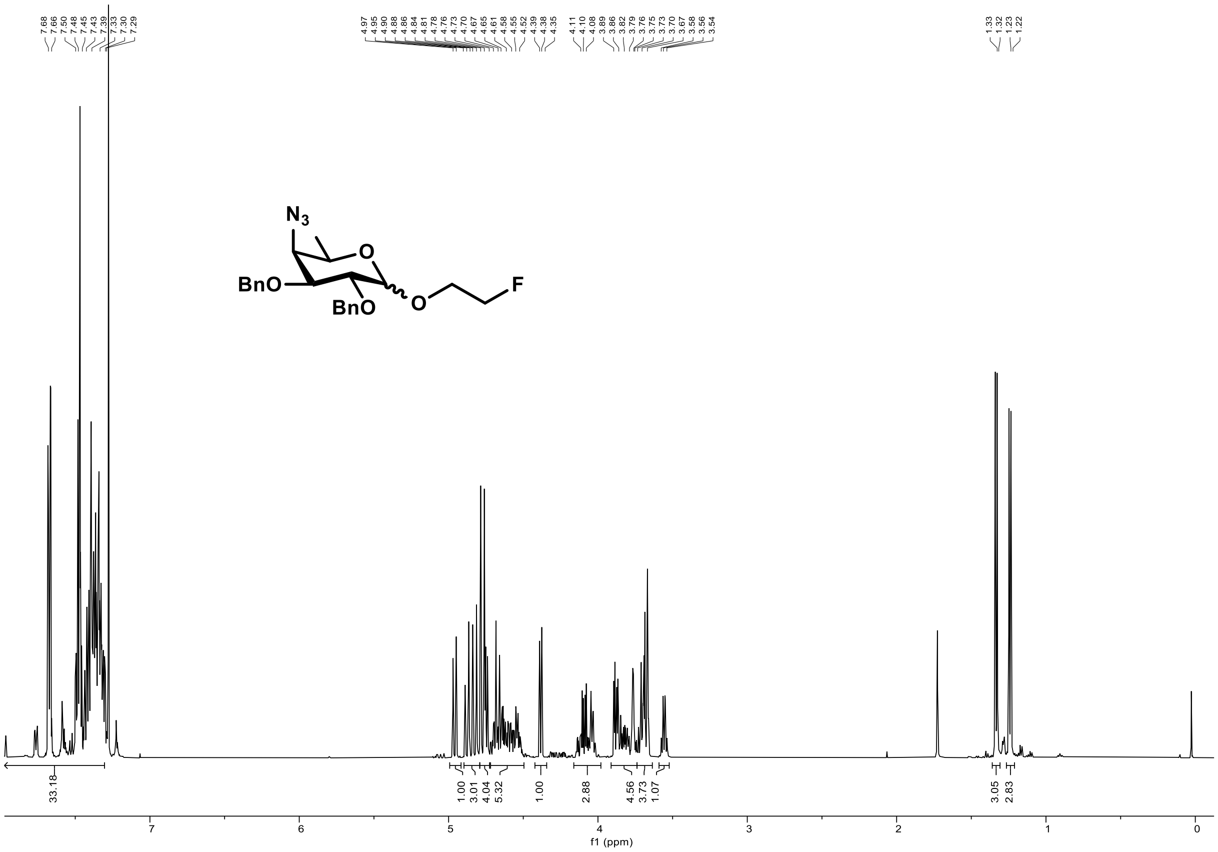

^{13}C NMR, 101 MHz, $CDCl_3$ of **S76**

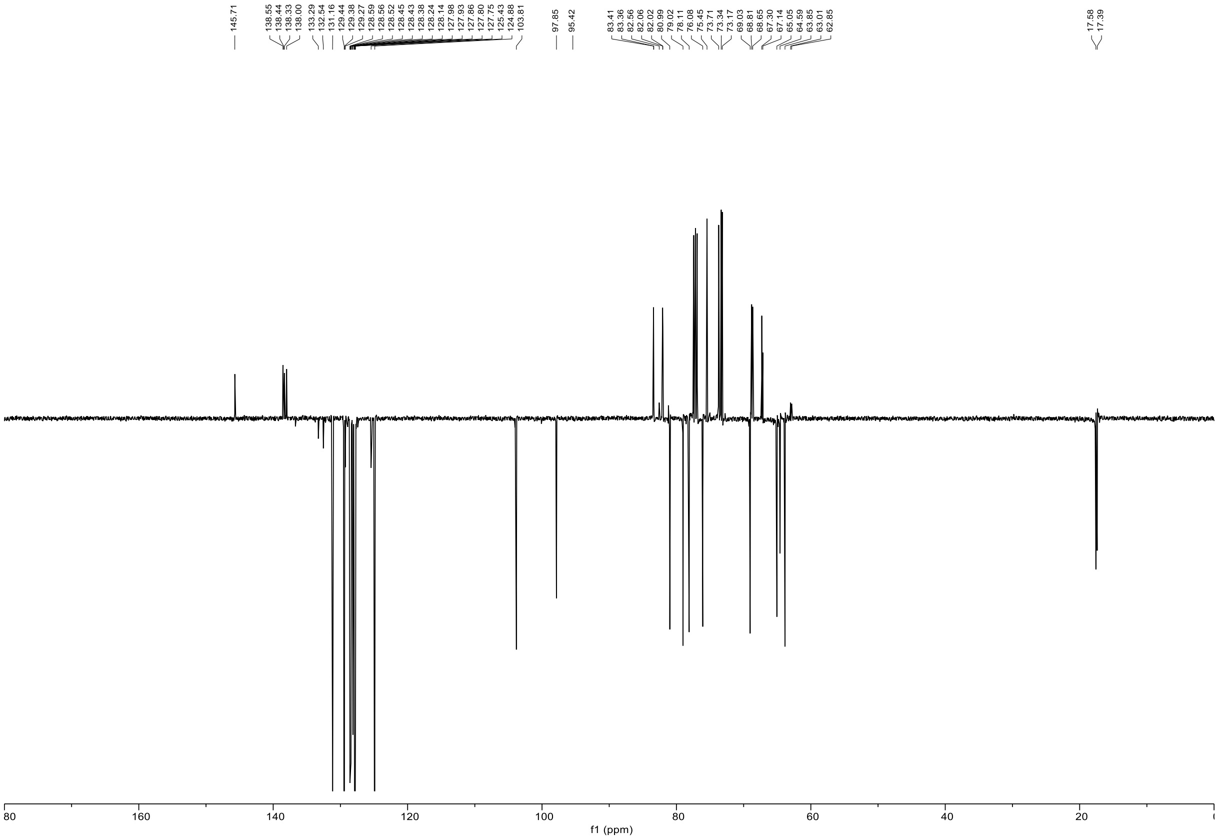

^{1}H NMR, 400 MHz, $CDCl_3$ of **S77**

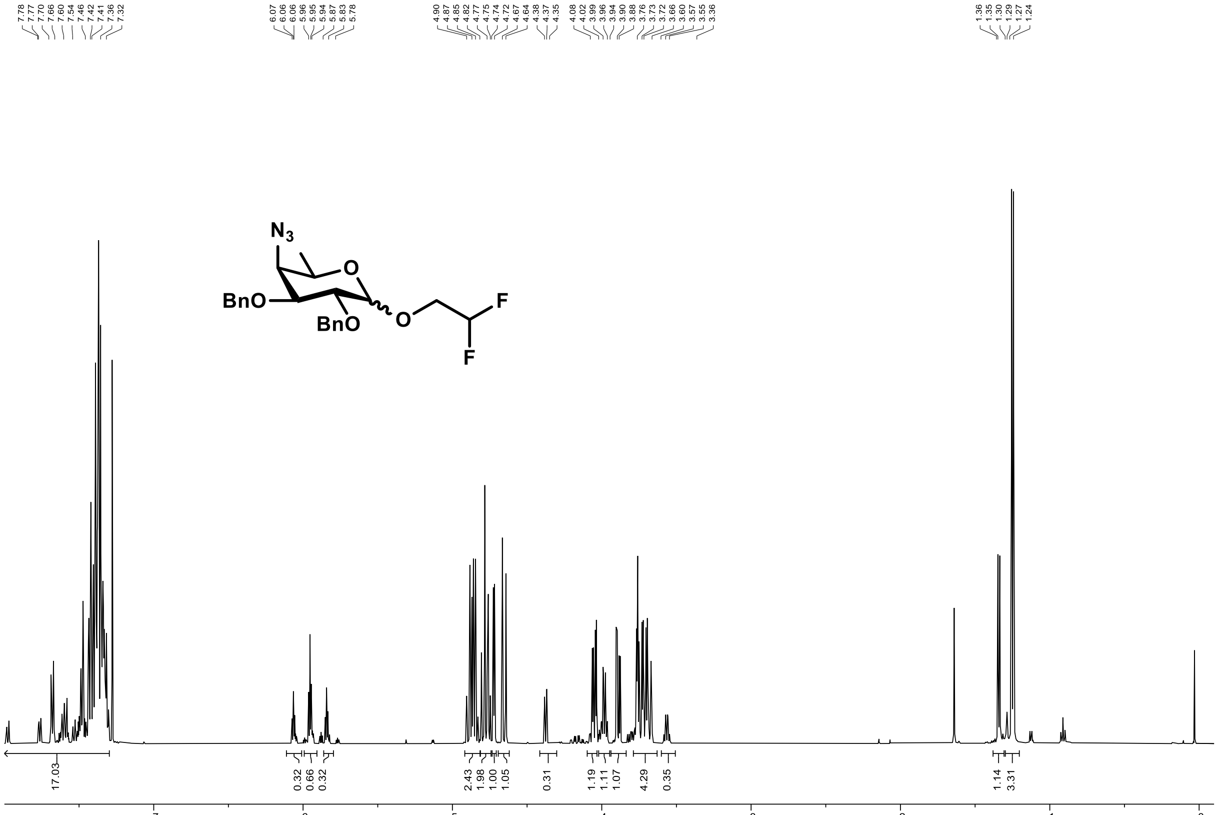

^{13}C NMR, 101 MHz, $CDCl_3$ of **S77**

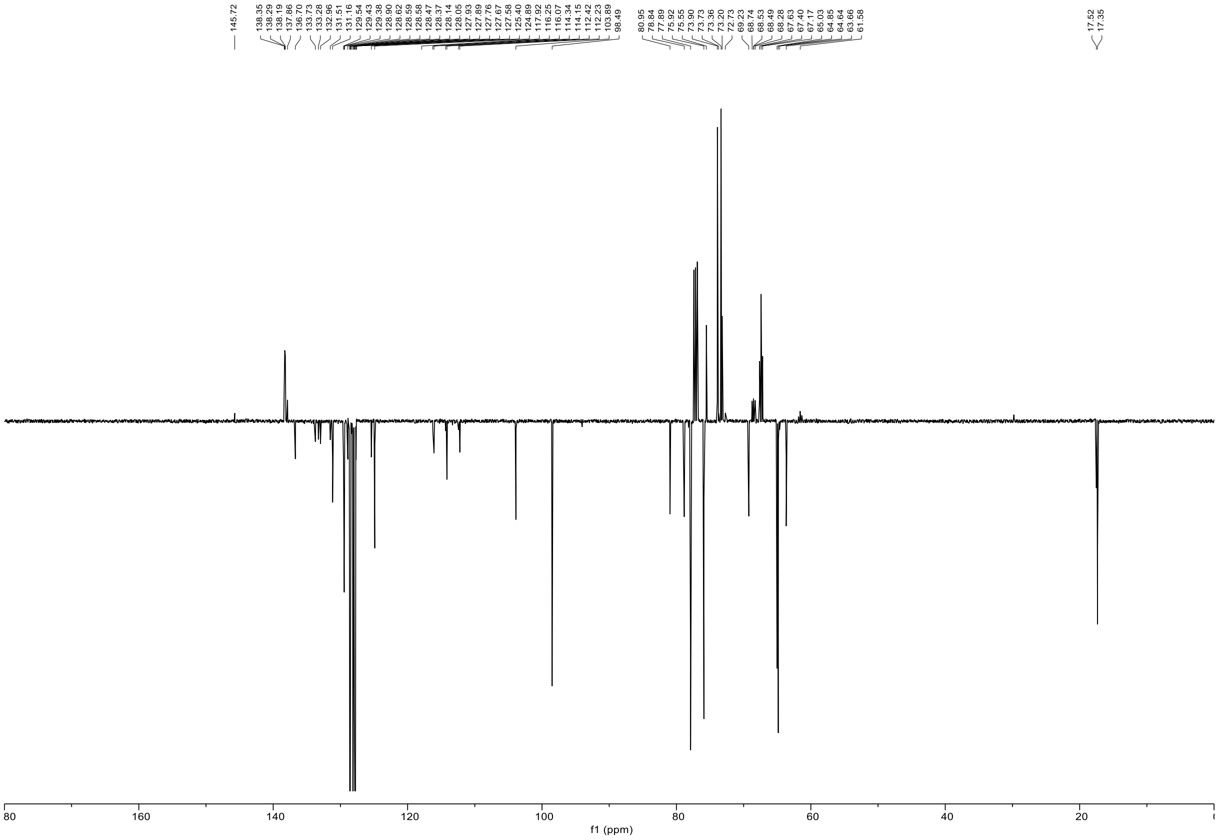

^{1}H NMR, 400 MHz, $CDCl_3$ of **S78**

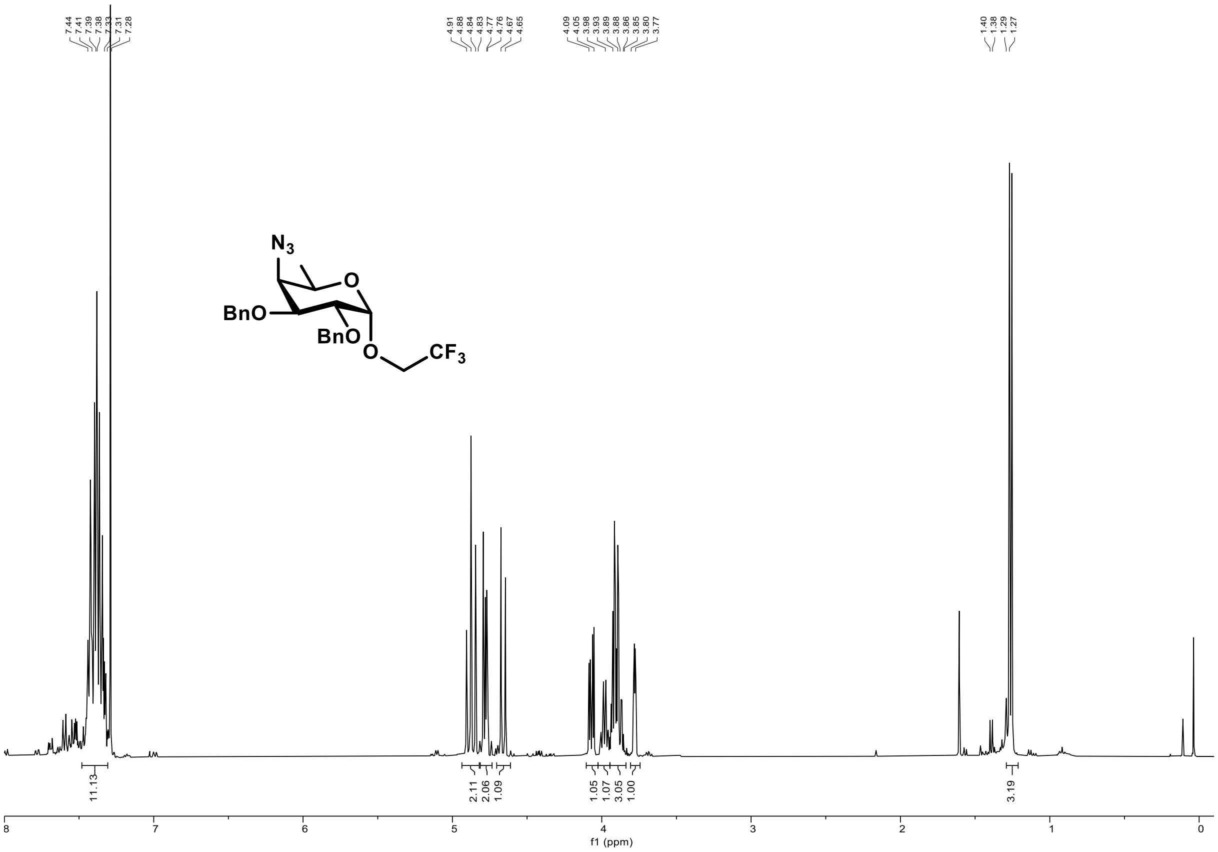

^{13}C NMR, 101 MHz, $CDCl_3$ of **S78**

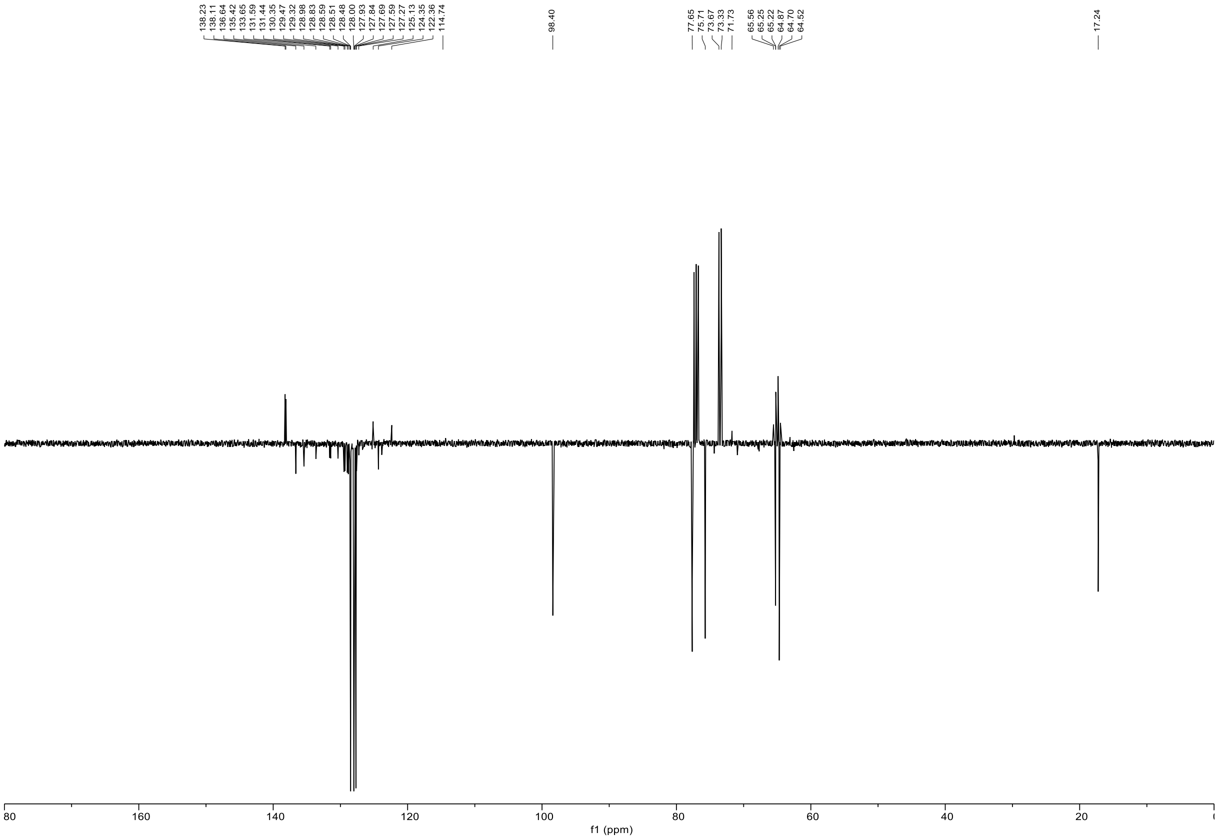

^{1}H NMR, 400 MHz, $CDCl_3$ of **S80**

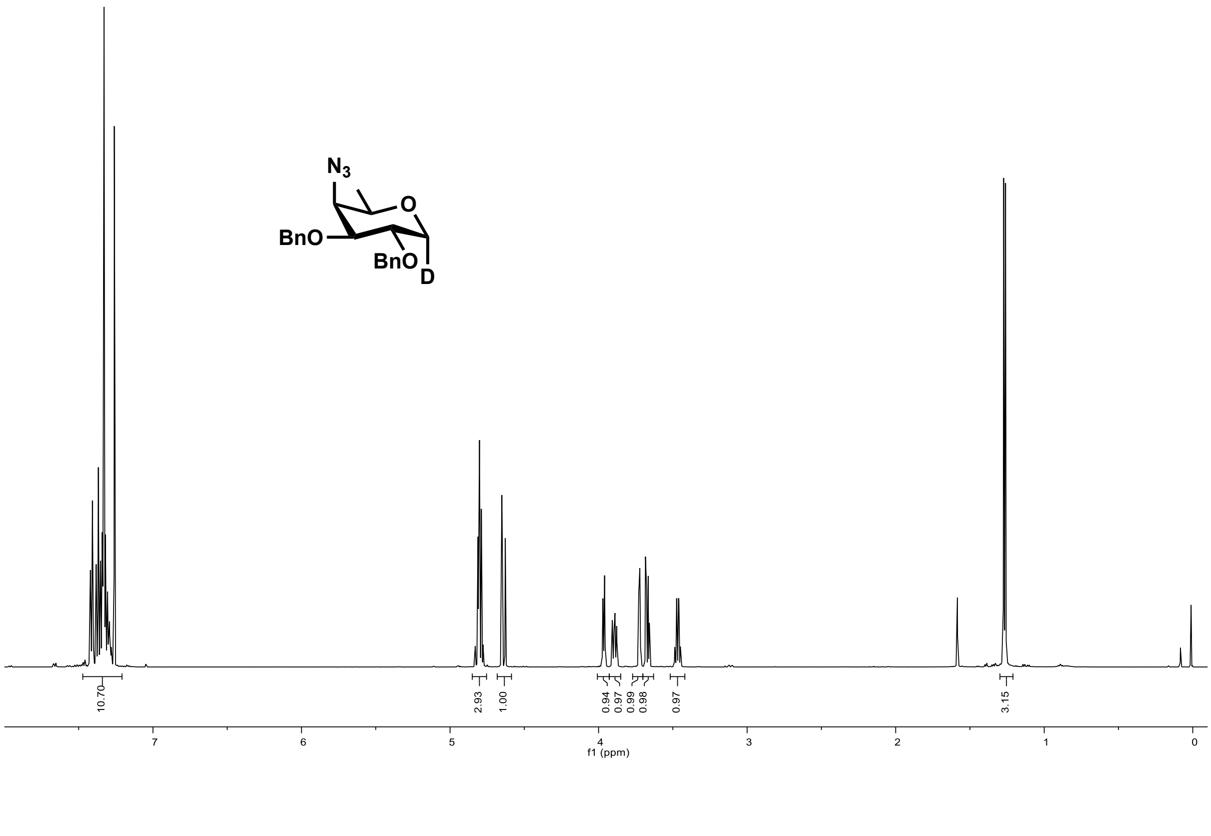

^{13}C NMR, 101 MHz, $CDCl_3$ of **S80**

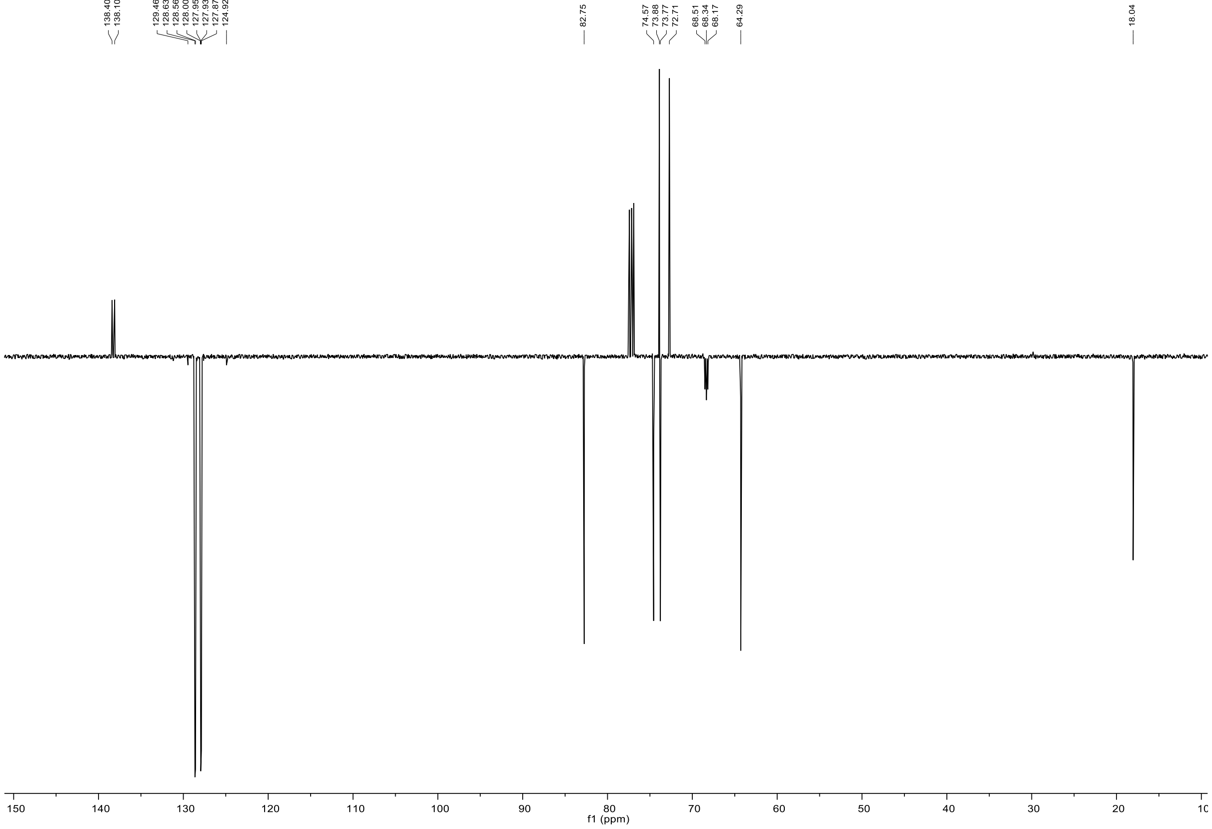

HH-COSY NMR, $CDCl_3$ of **S80**

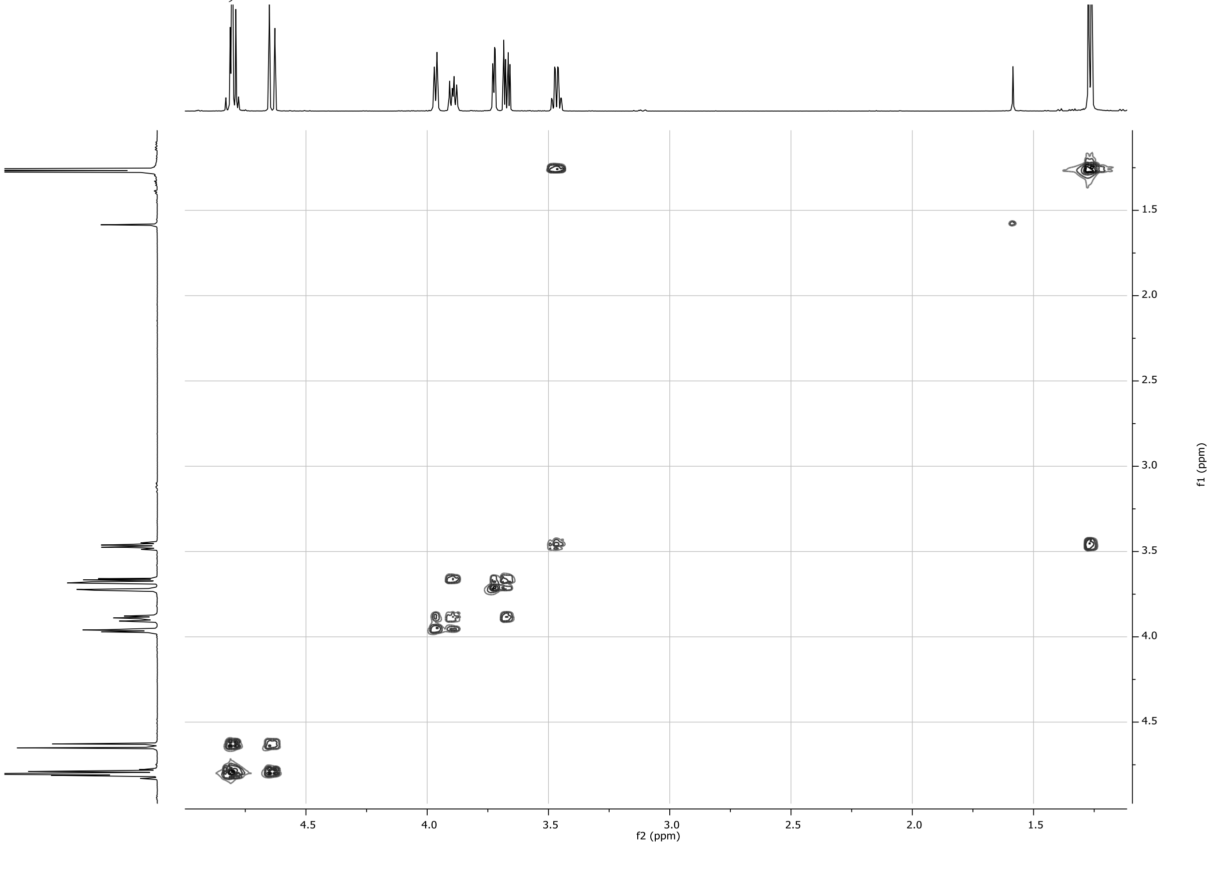

HSQC NMR, $CDCl_3$ of **S80**

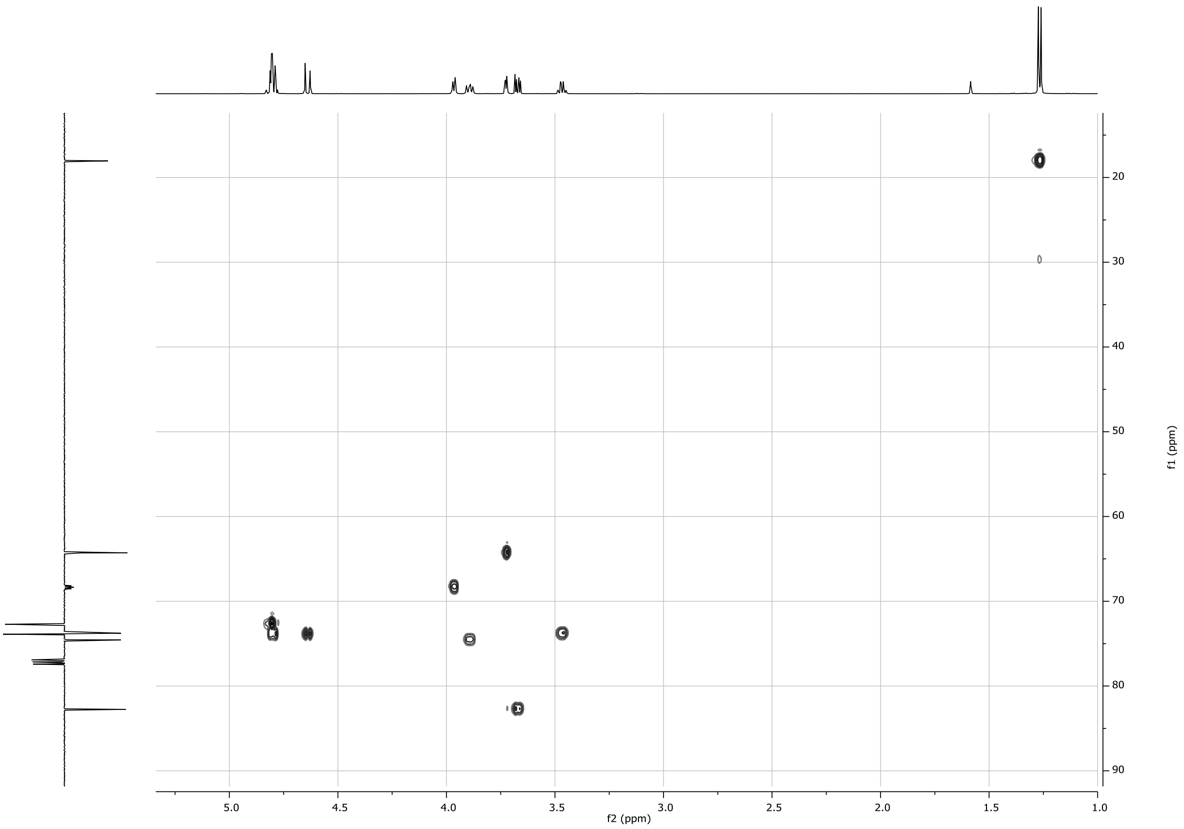

^{2}H NMR, $CDCl_3$ of **S80**

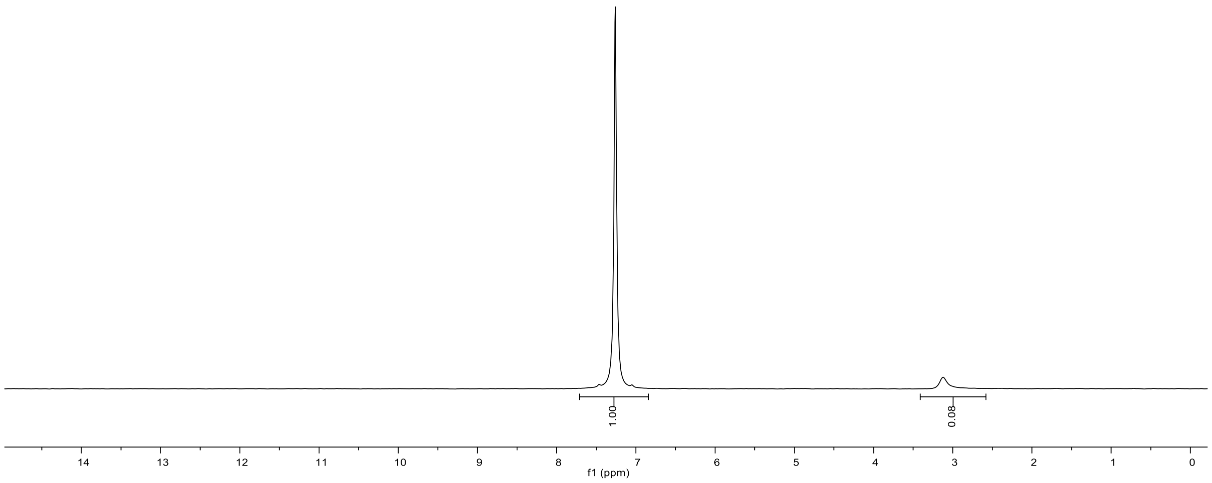

^{1}H NMR, 400 MHz, $CDCl_3$ of **S79**

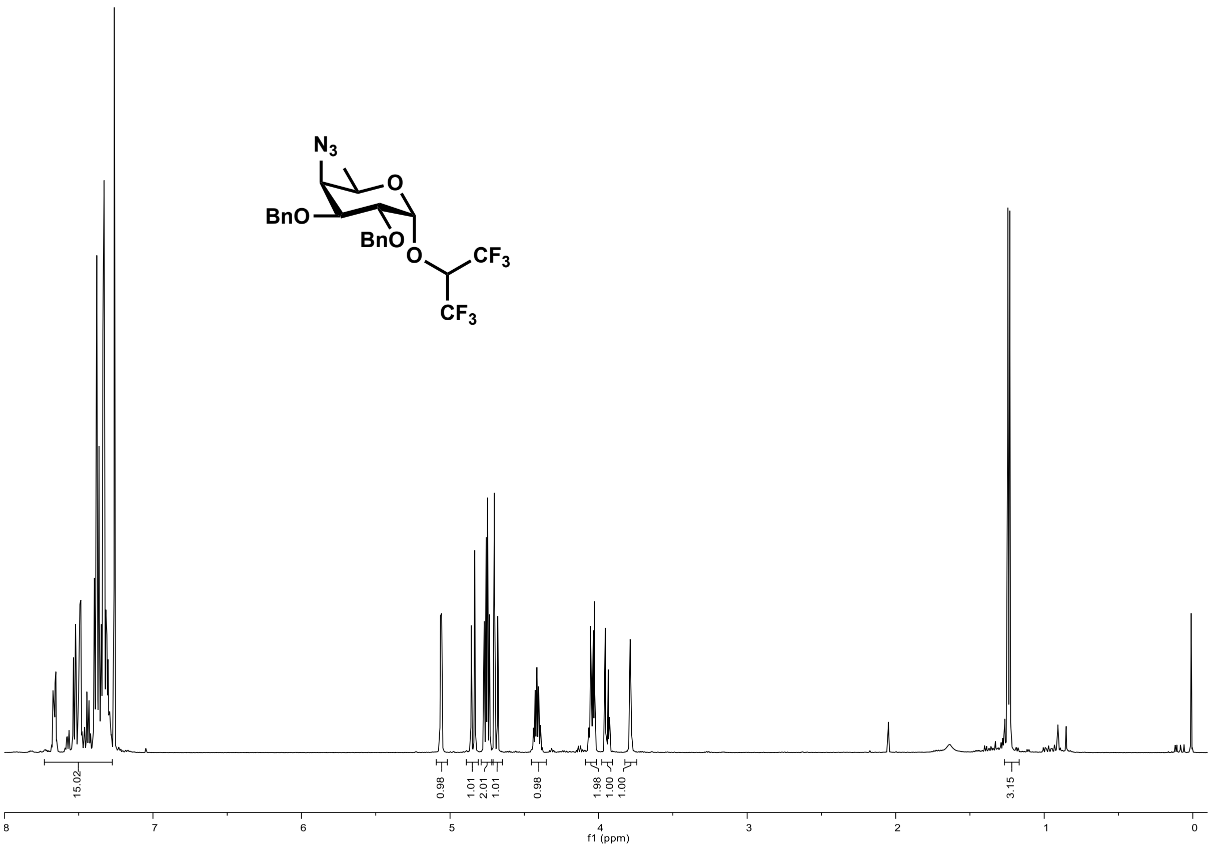

^{13}C NMR, 101 MHz, $CDCl_3$ of **S79**

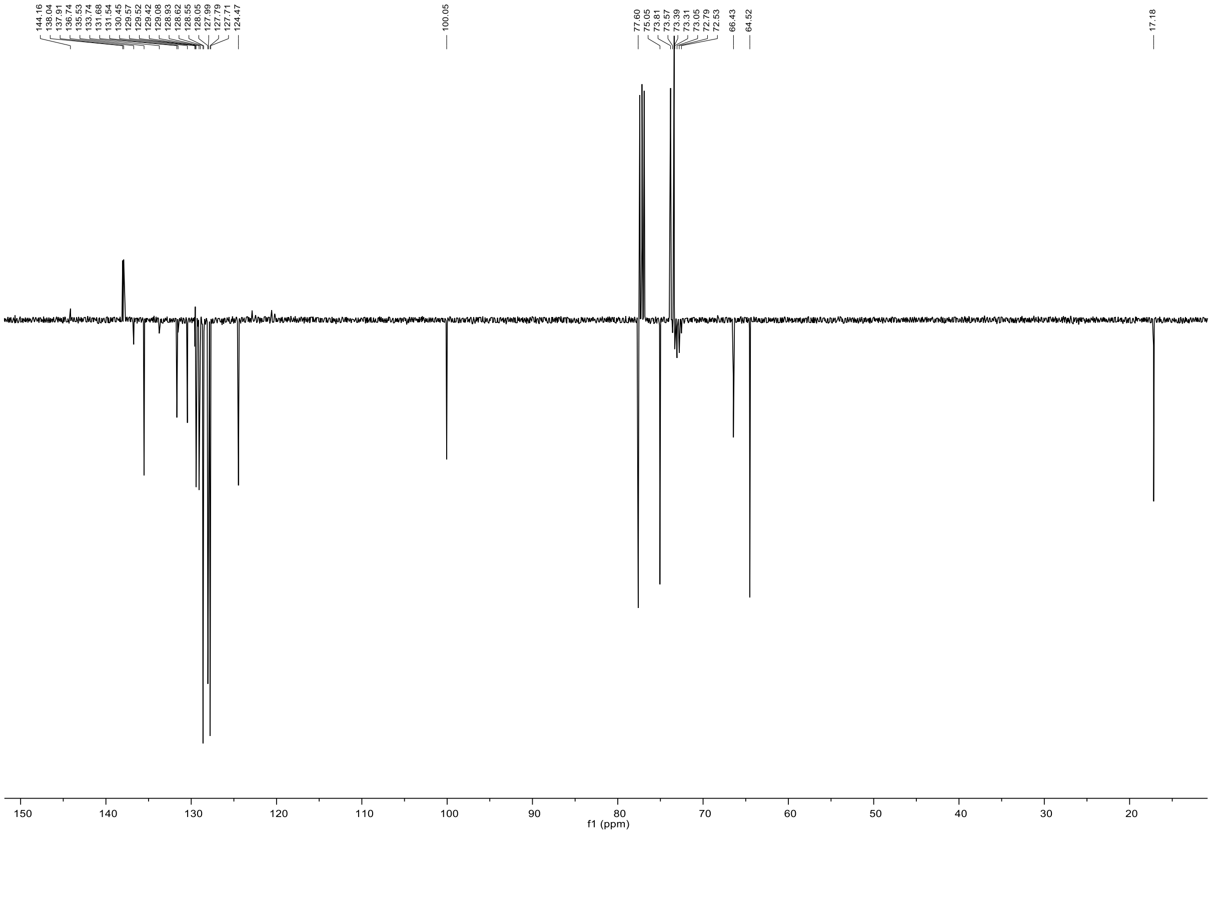

HH-COSY NMR, $CDCl_3$ of **S79**

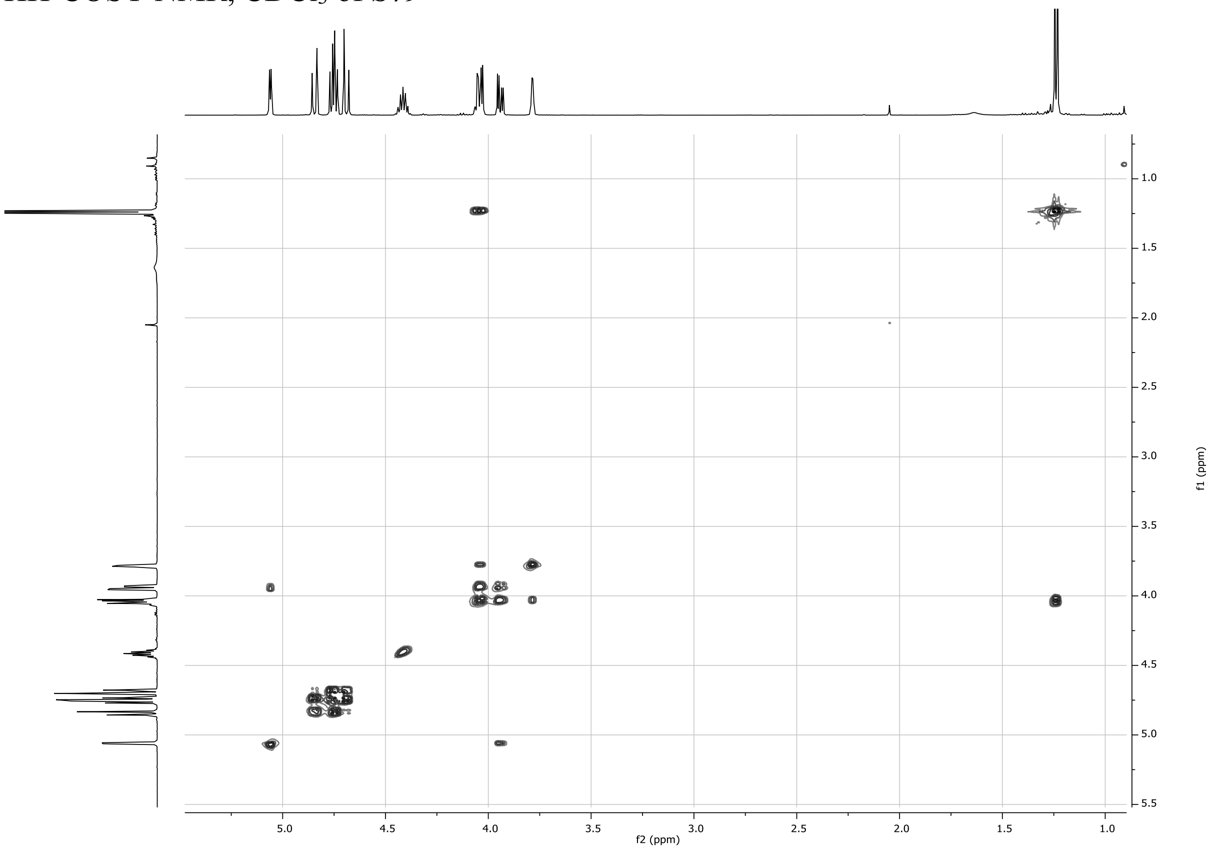

HSQC NMR, $CDCl_3$ of **S79**

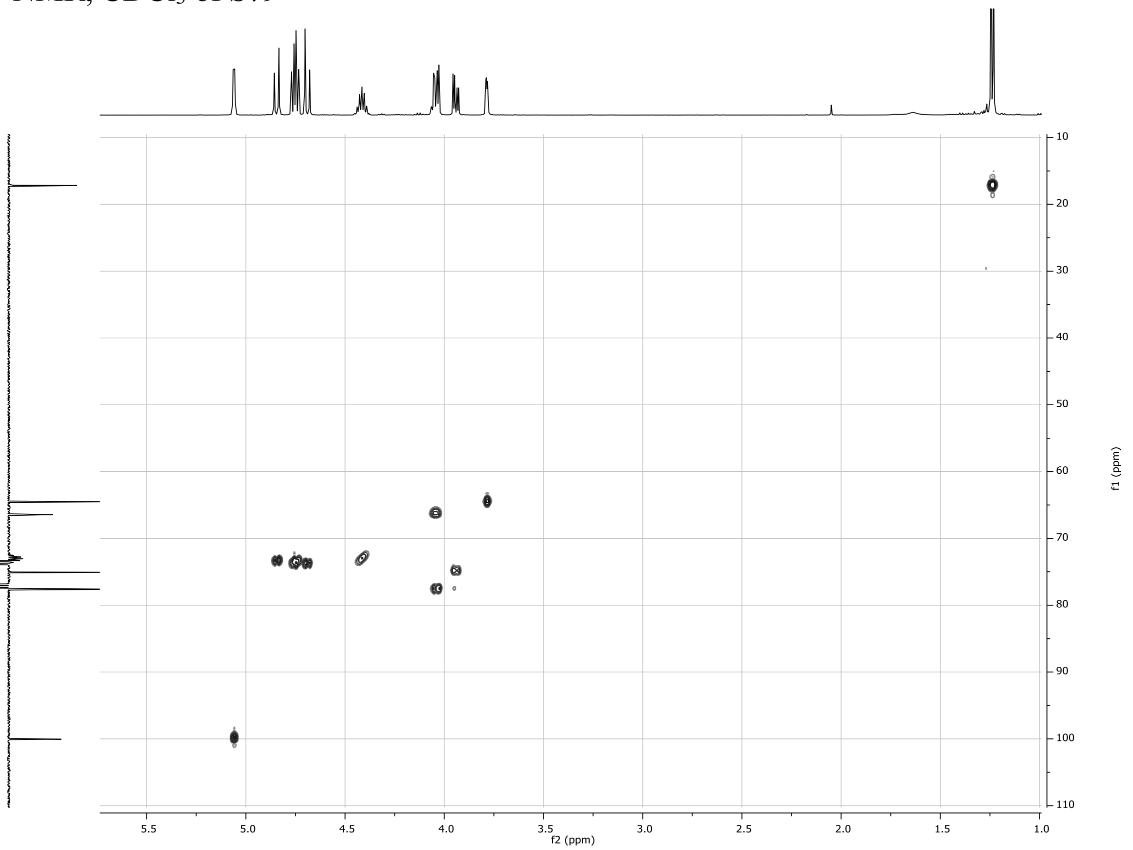

^{1}H NMR, 850 MHz, $CDCl_3$ of **S74**

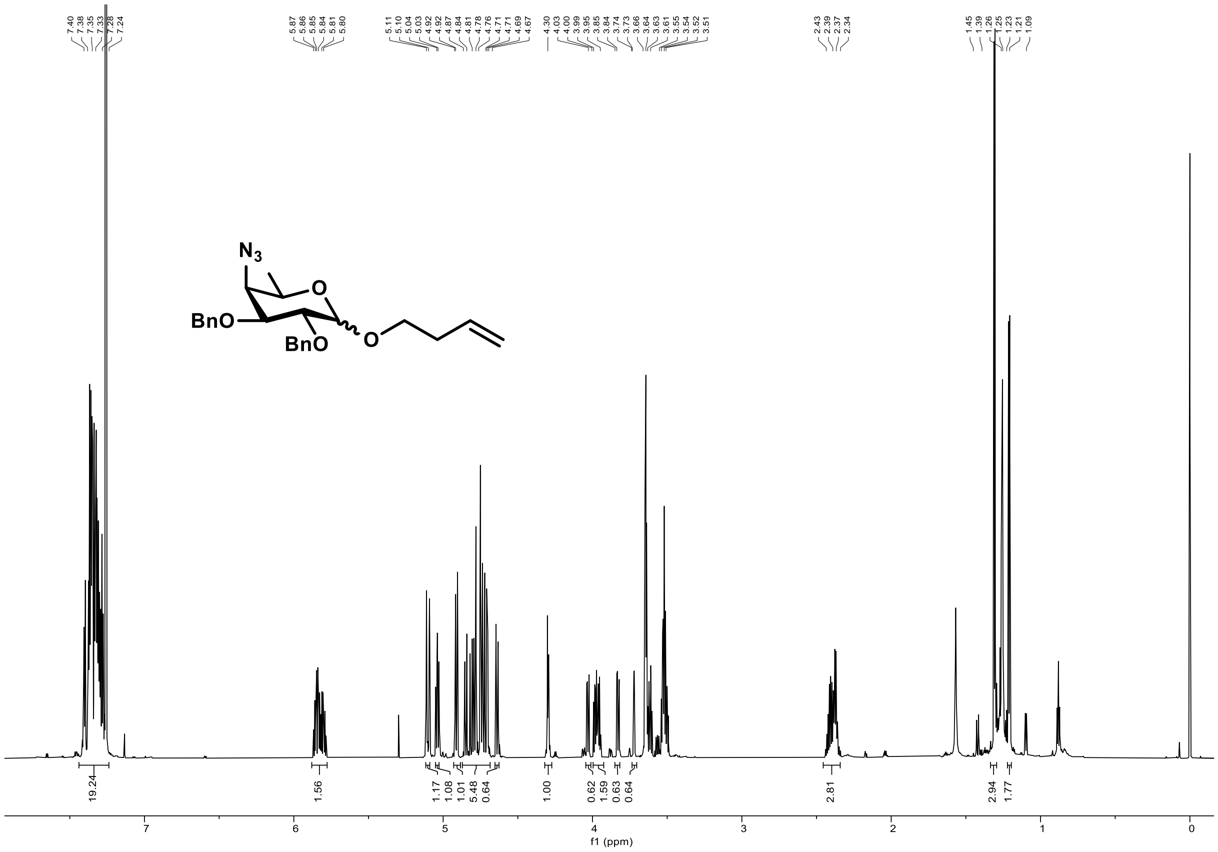

^{13}C NMR, 214 MHz, $CDCl_3$ of **S74**

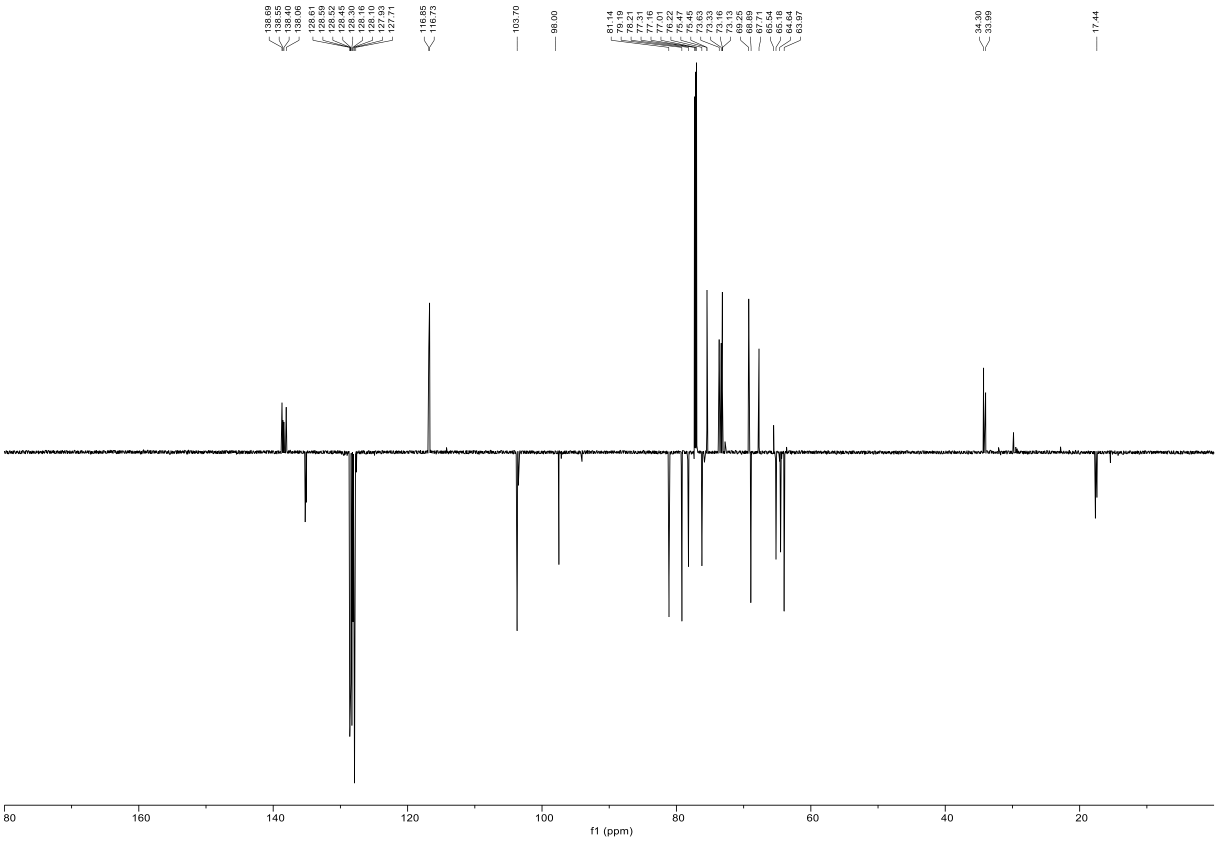

^{1}H NMR, 500 MHz, CDCl$_3$ of **S81**

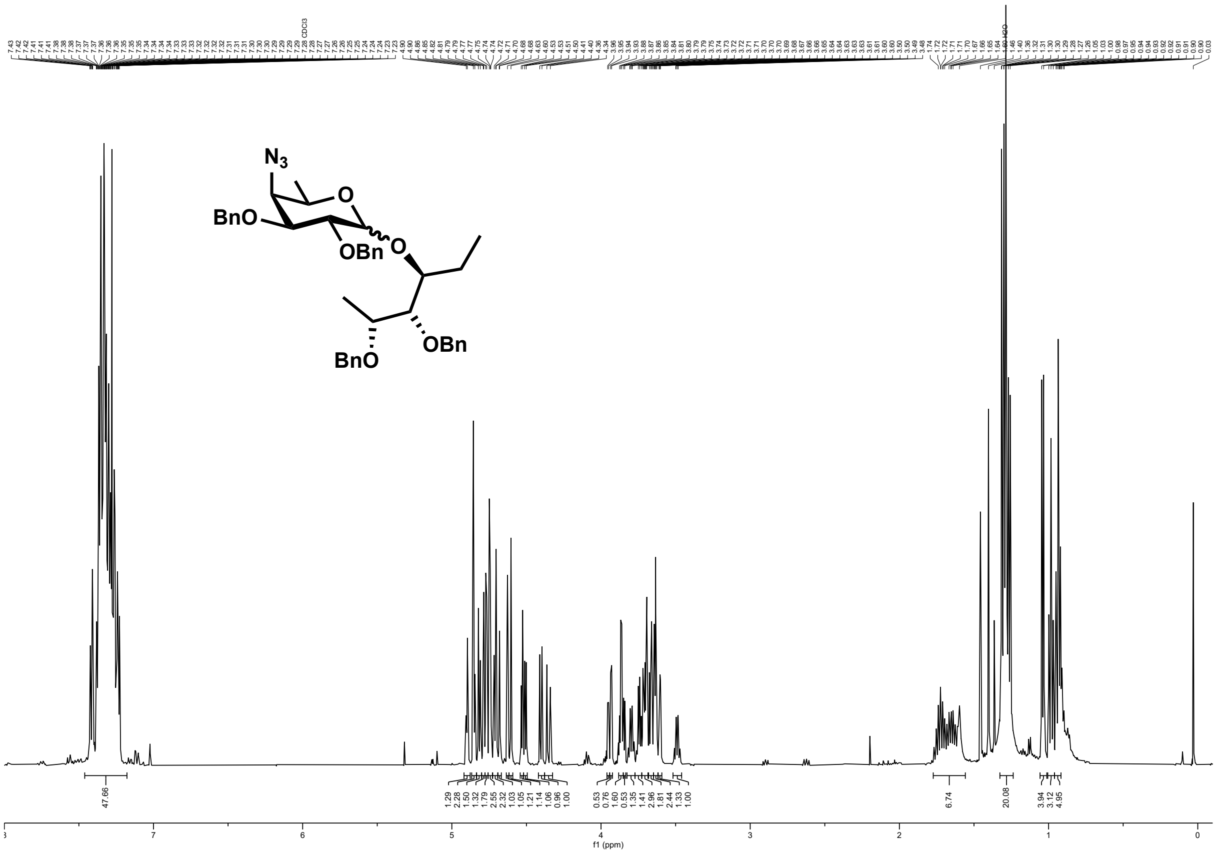

^{13}C NMR, 126 MHz, CDCl$_3$ of **S81**

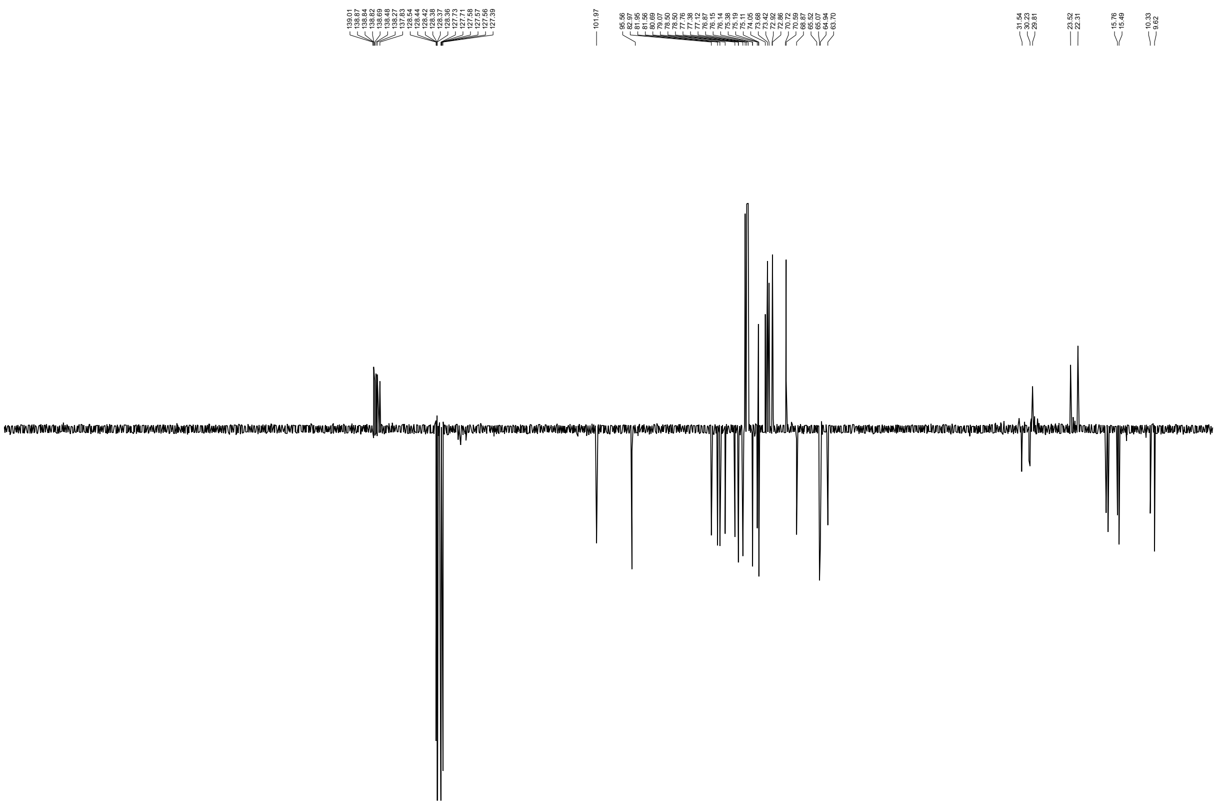

HH-COSY NMR, $CDCl_3$ of **S81**

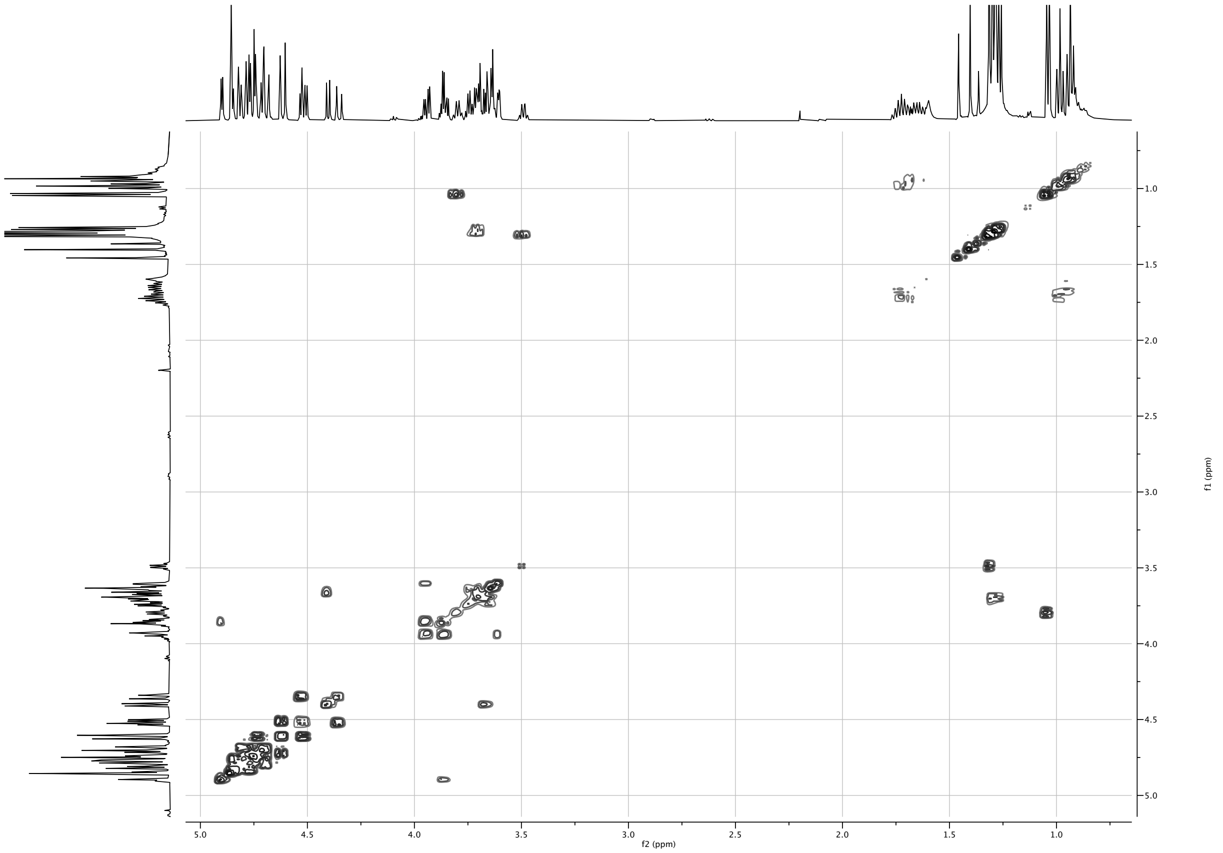

HSQC NMR, $CDCl_3$ of **S81**

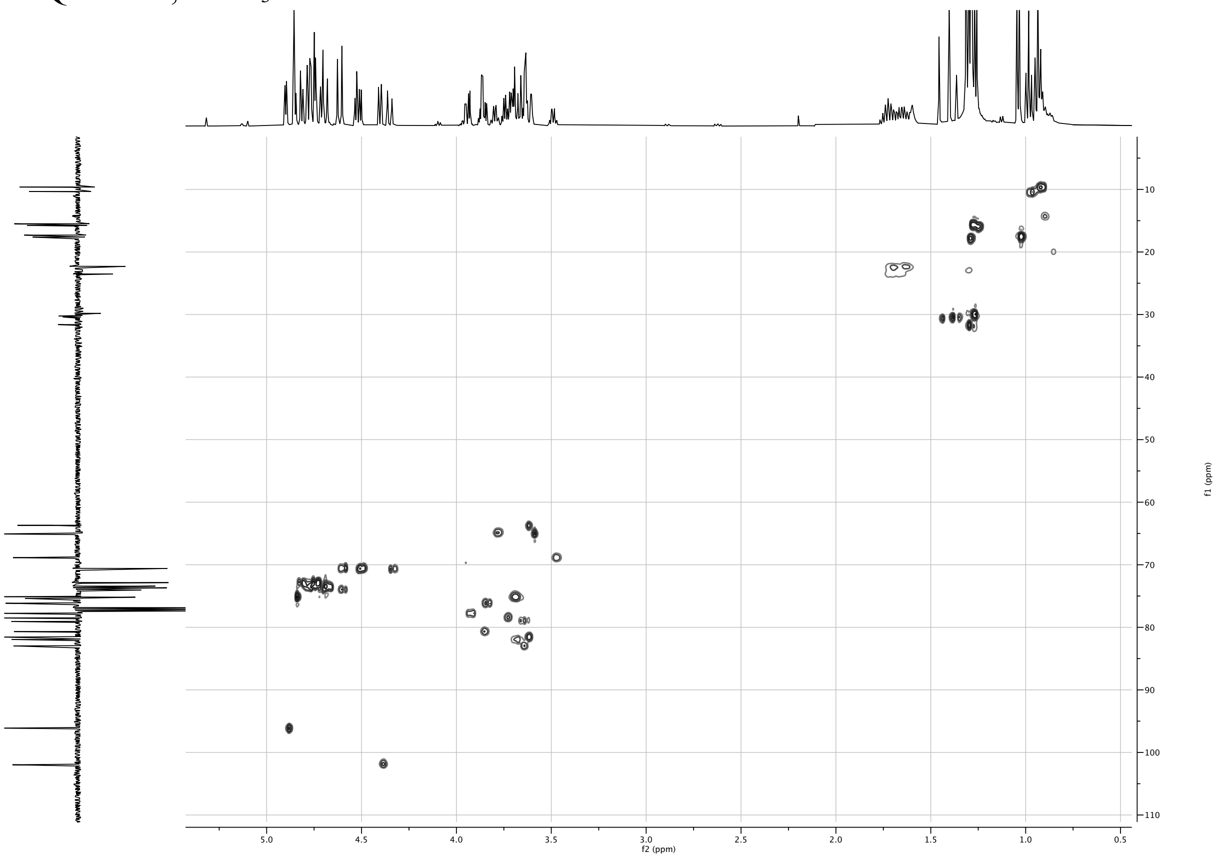

^{1}H NMR, 500 MHz, $CDCl_3$ of **S82**

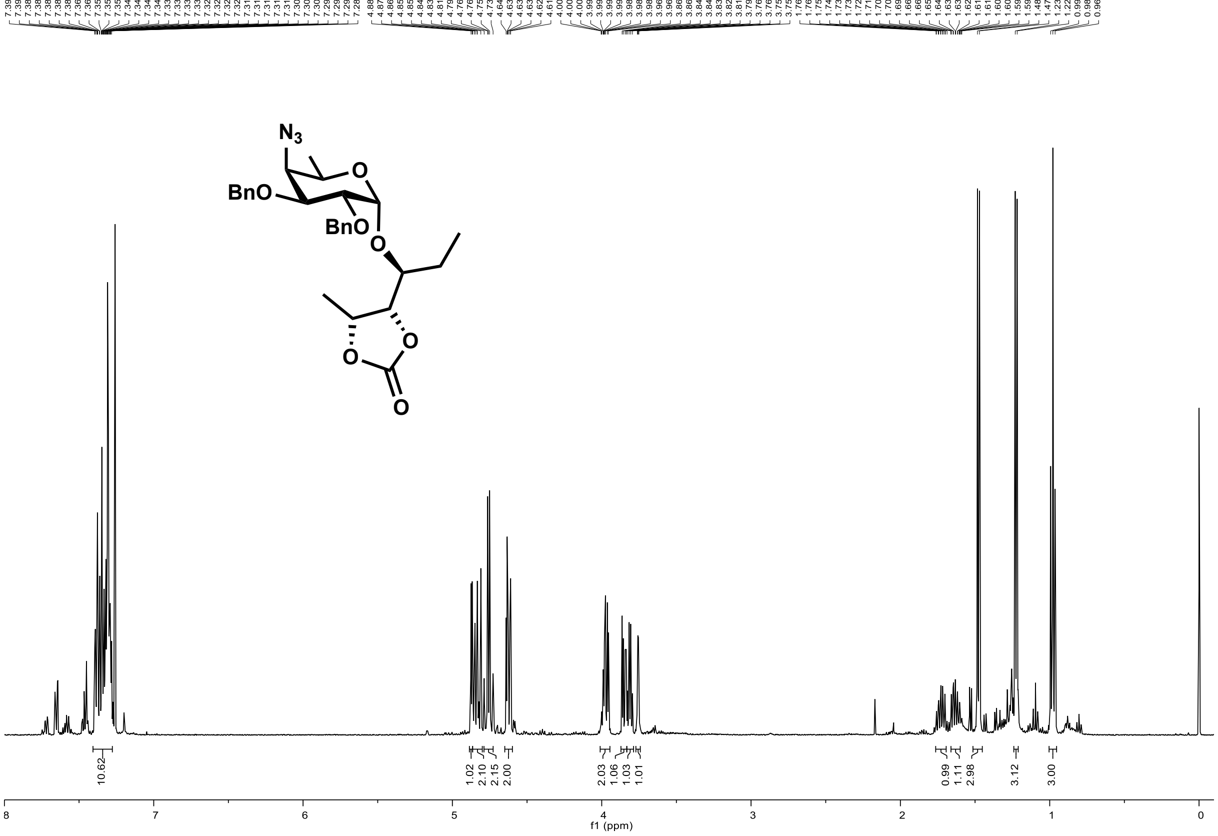

^{13}C NMR, 126 MHz, $CDCl_3$ of **S82**

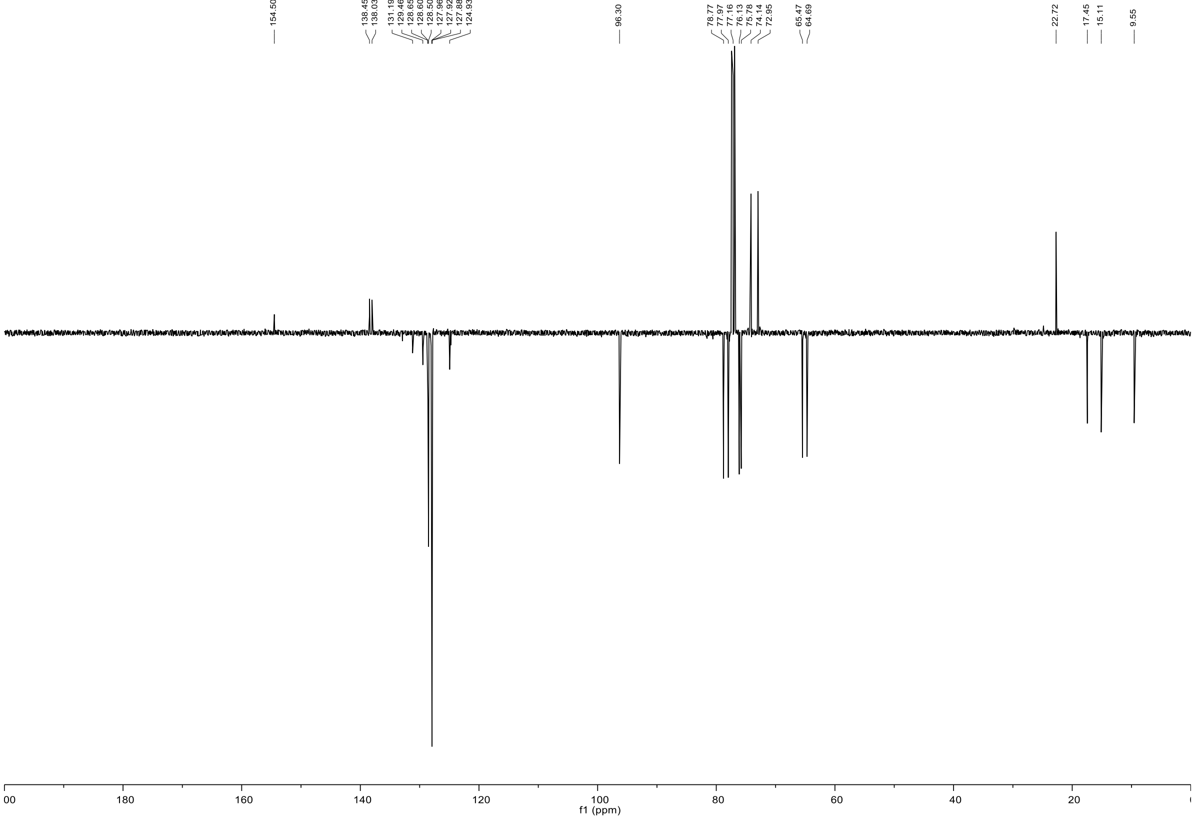

HH-COSY NMR, $CDCl_3$ of **S82**

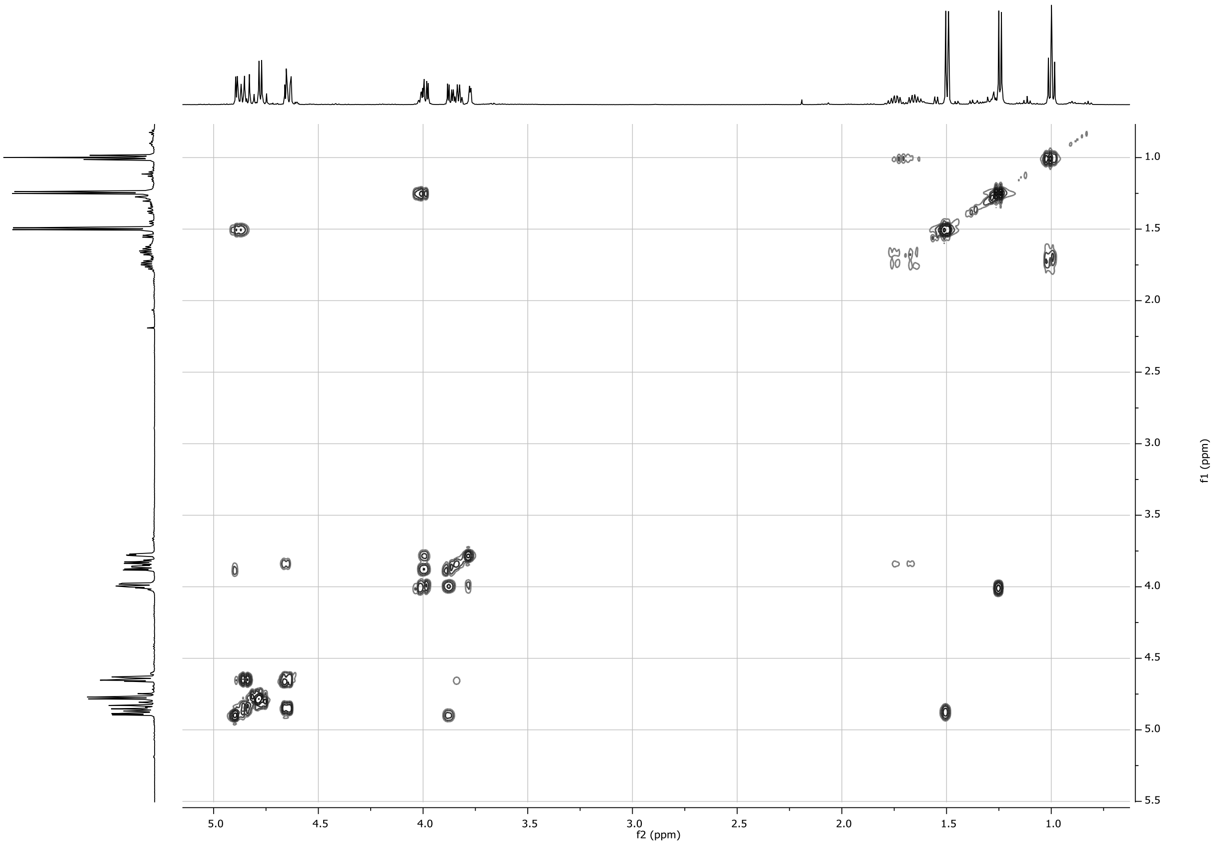

HSQC NMR, $CDCl_3$ of **S82**

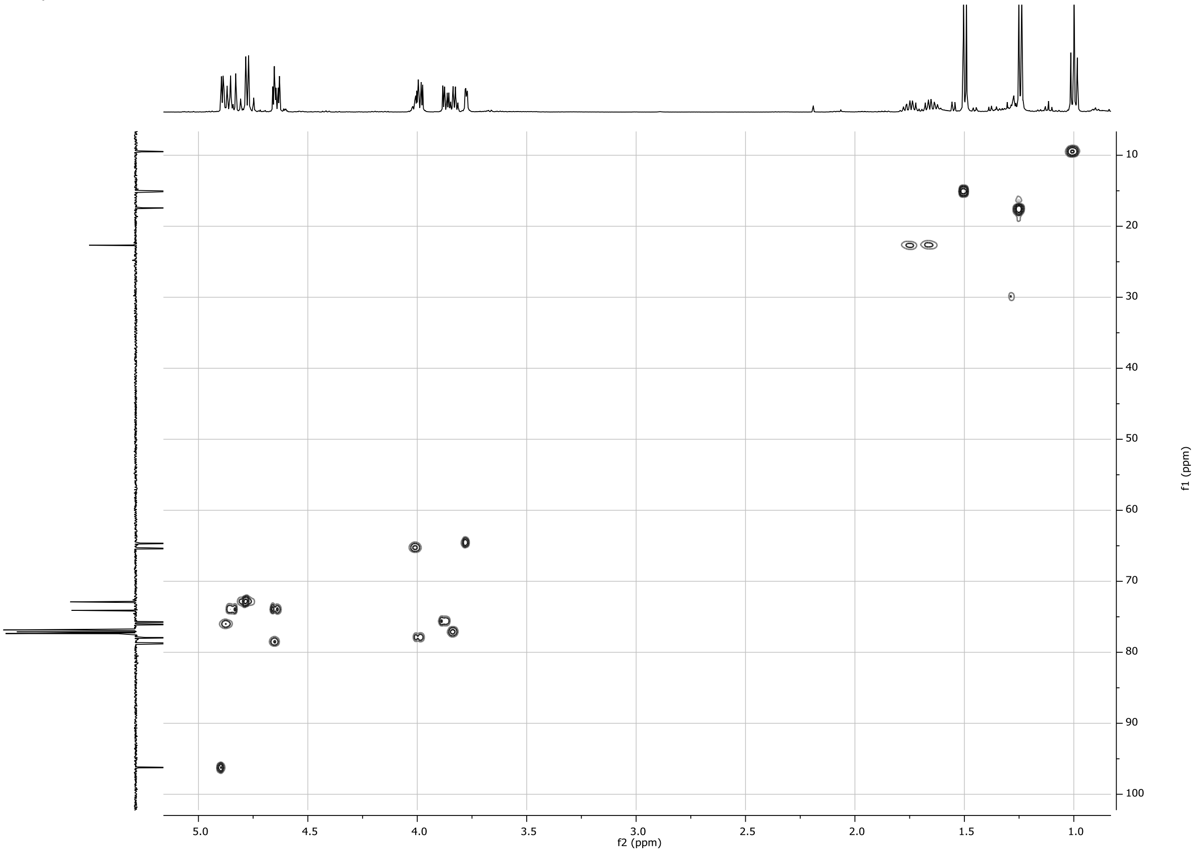

HMBC NMR, $CDCl_3$ of **S82**

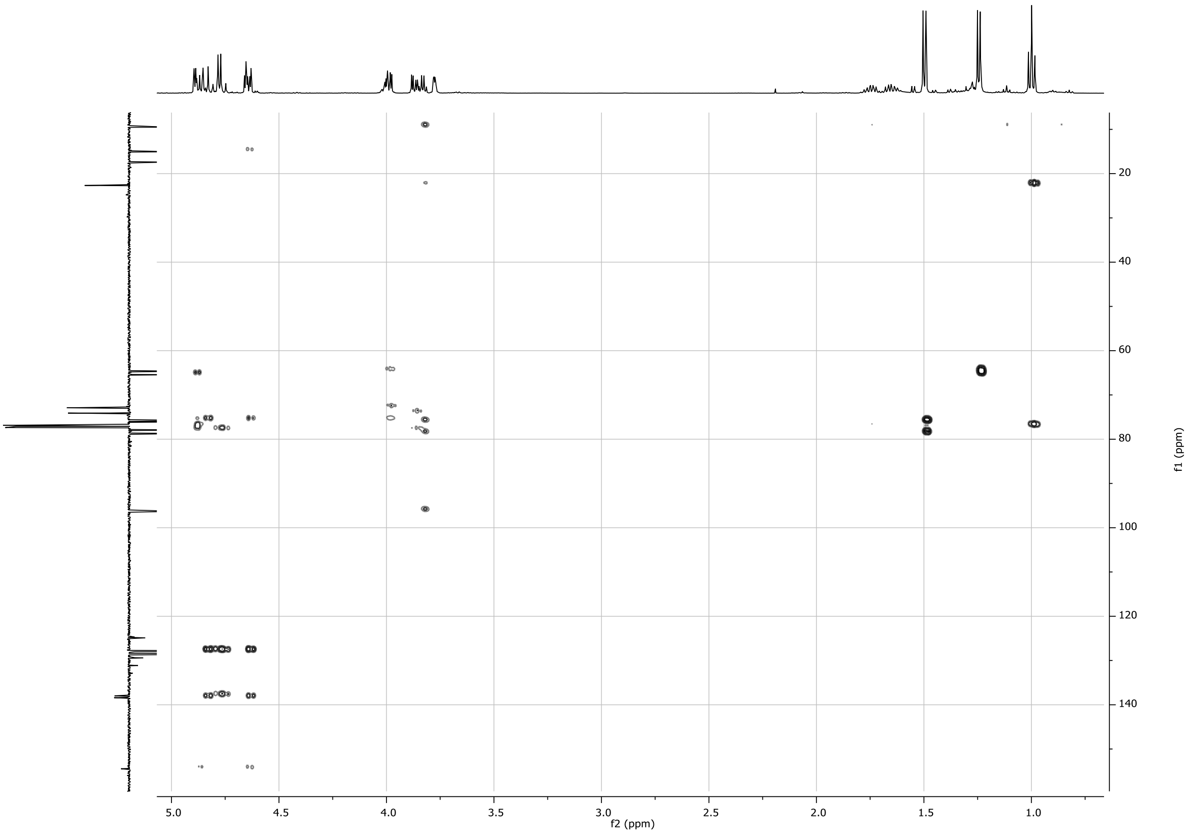

^{1}H NMR, 400 MHz, $CDCl_3$ of **S75 (+DMF)**

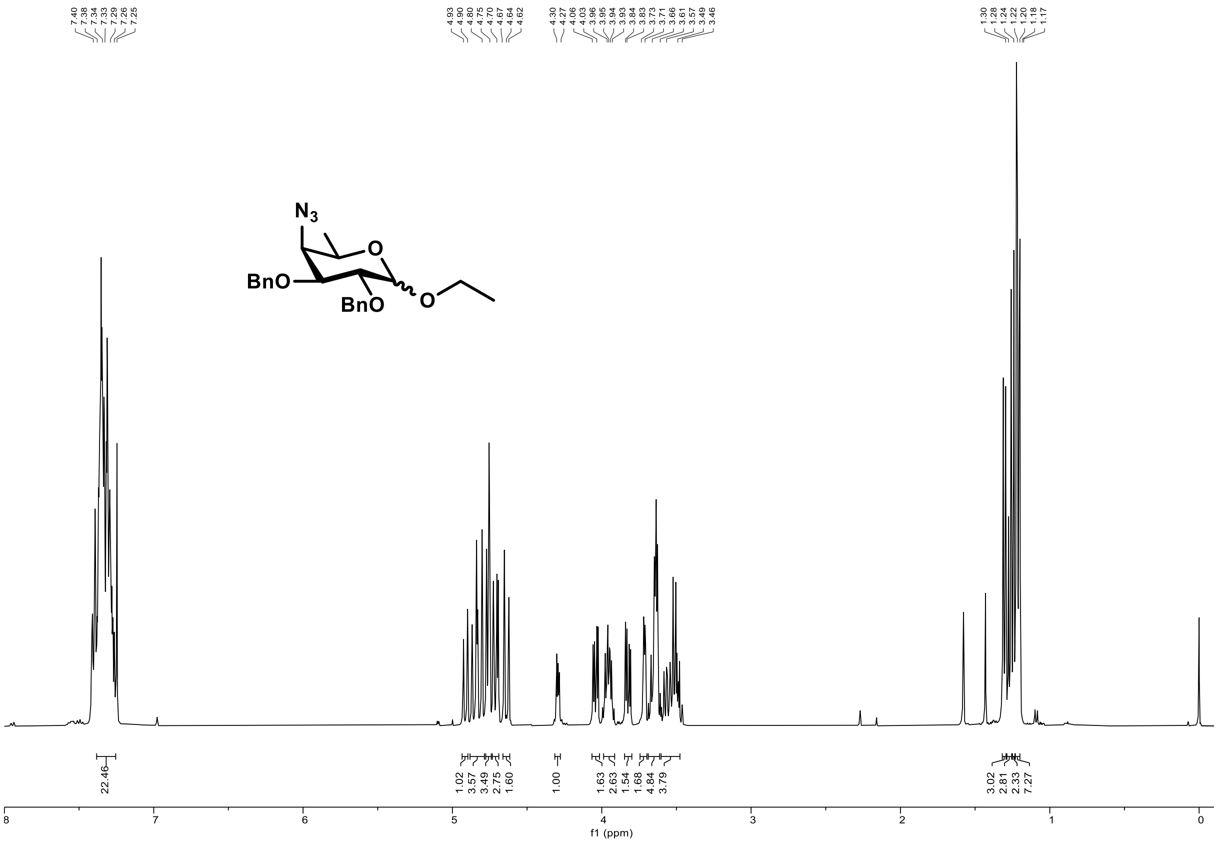

^{13}C NMR, 101 MHz, $CDCl_3$ of **S75 (+DMF)**

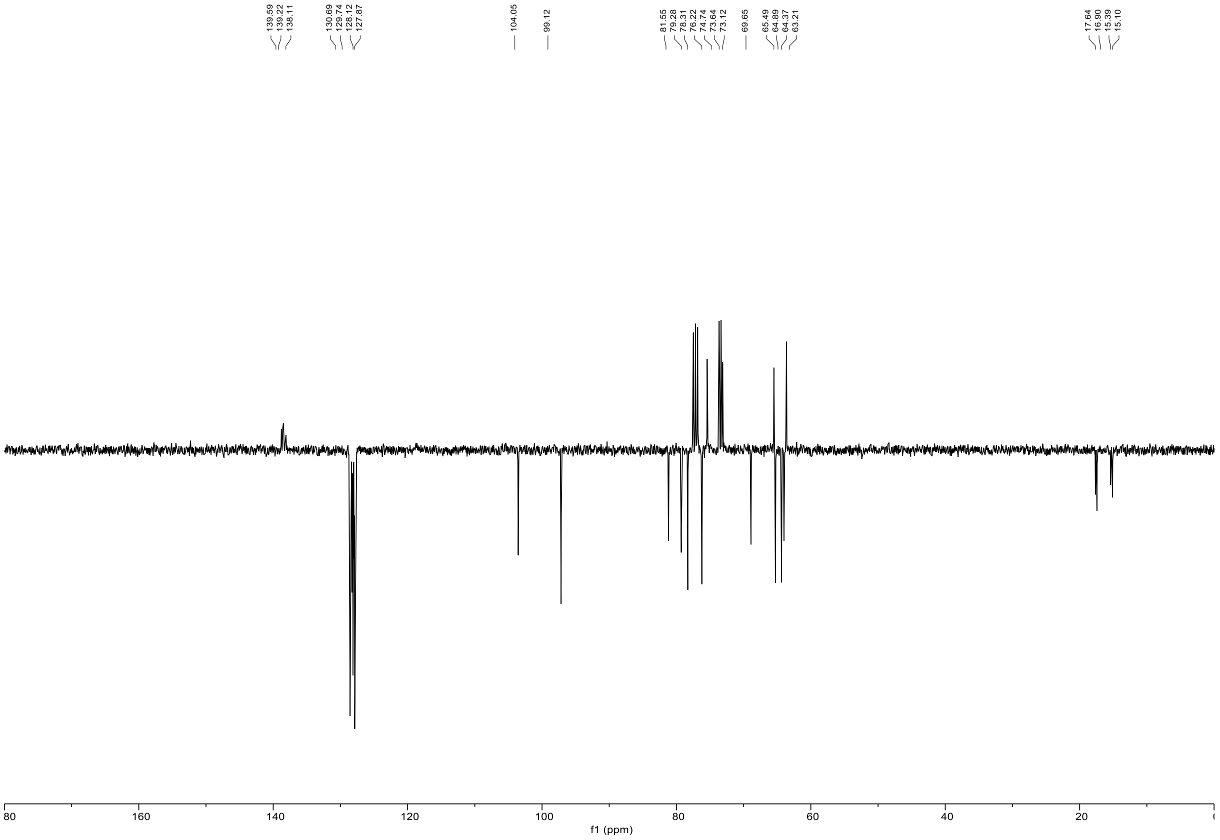

^{1}H NMR, 400 MHz, $CDCl_3$ of **S76 (+DMF)**

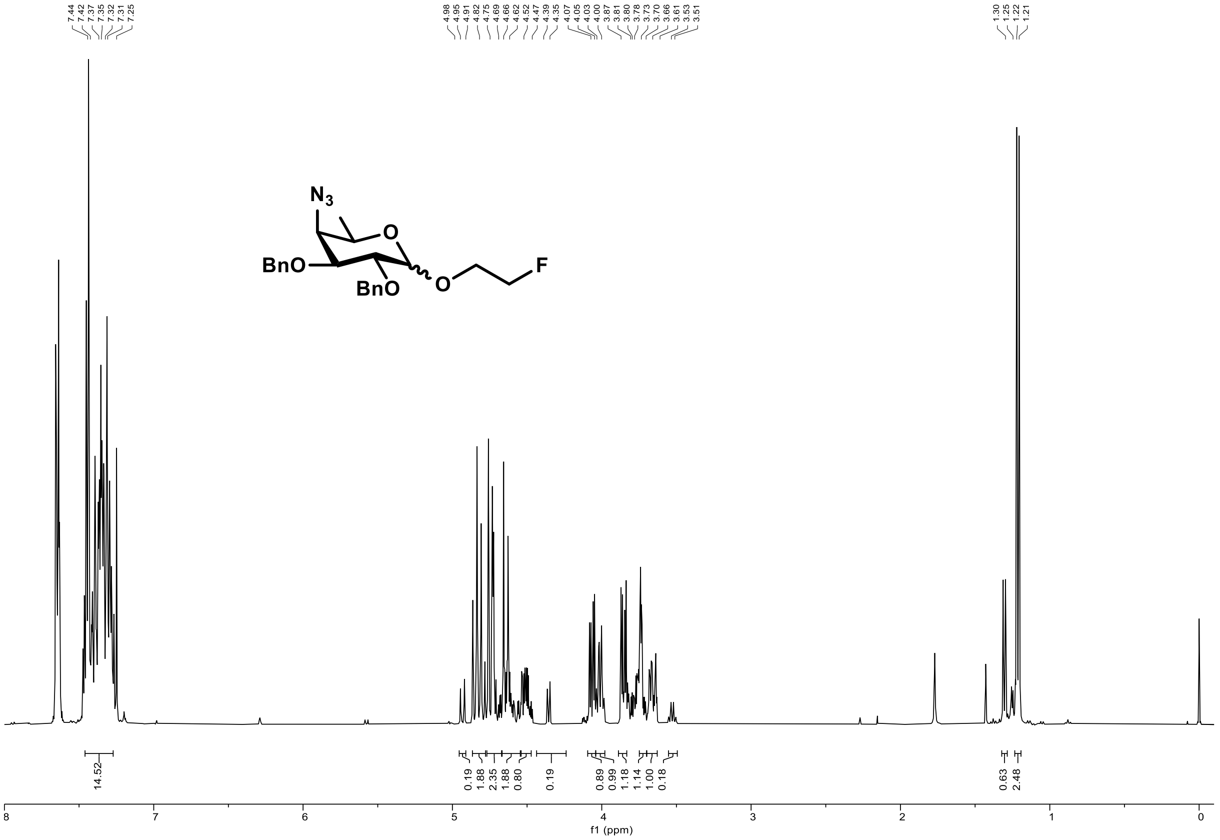

^{13}C NMR, 101 MHz, $CDCl_3$ of **S76 (+DMF)**

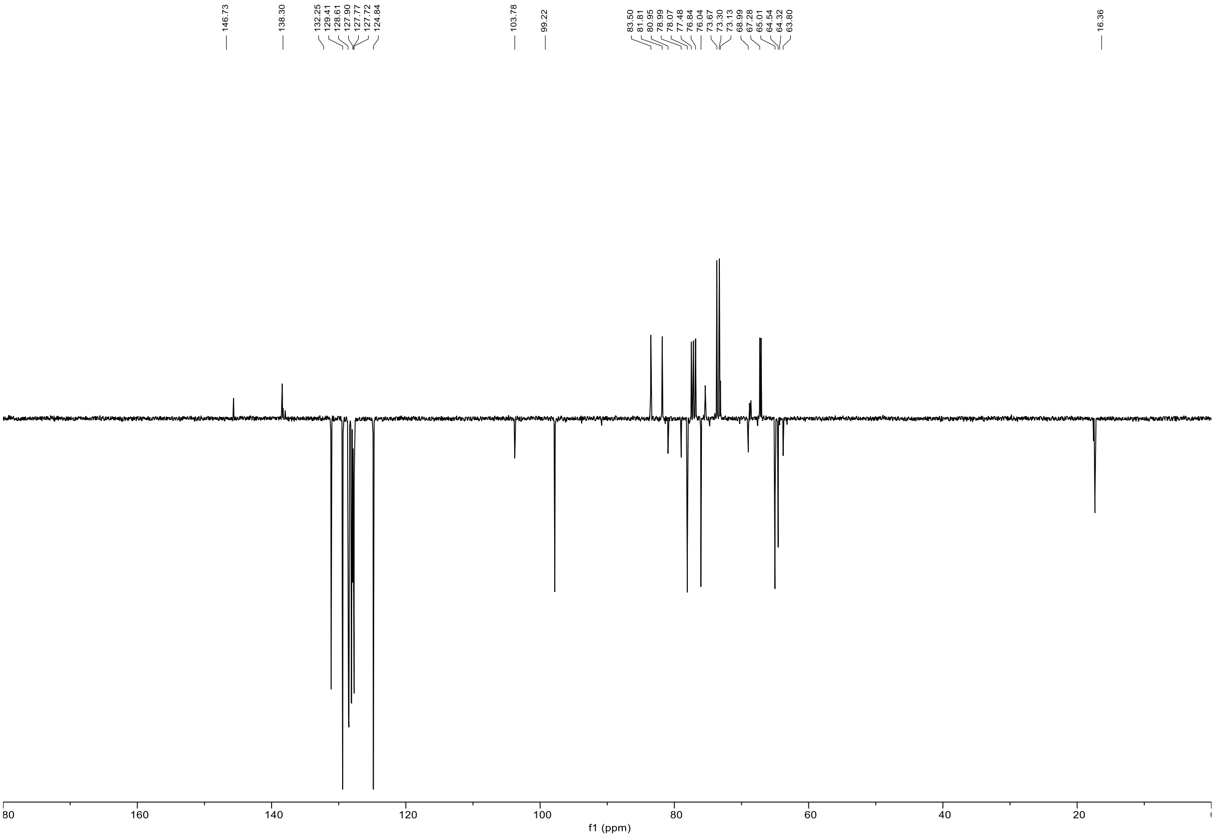

^{1}H NMR, 400 MHz, $CDCl_3$ of **S77 (+DMF)**

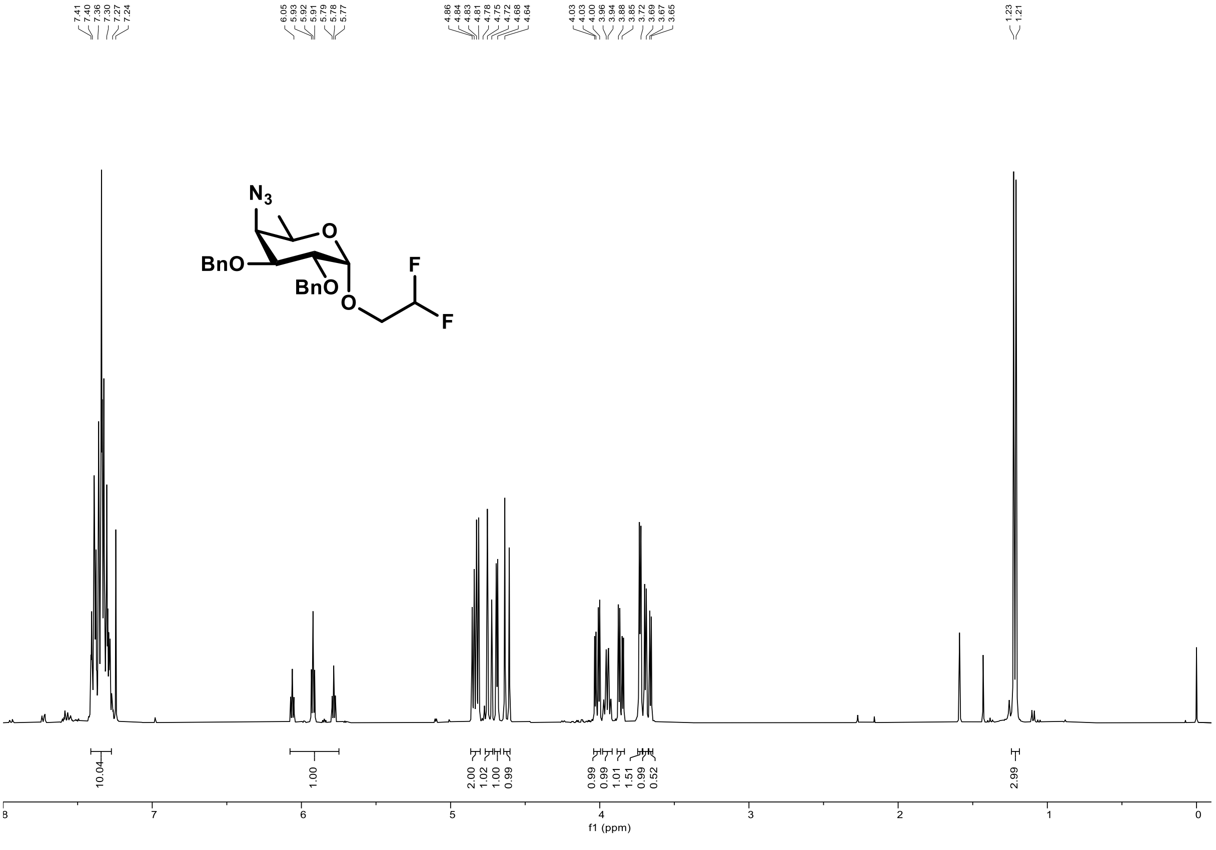

^{13}C NMR, 101 MHz, $CDCl_3$ of **S77 (+DMF)**

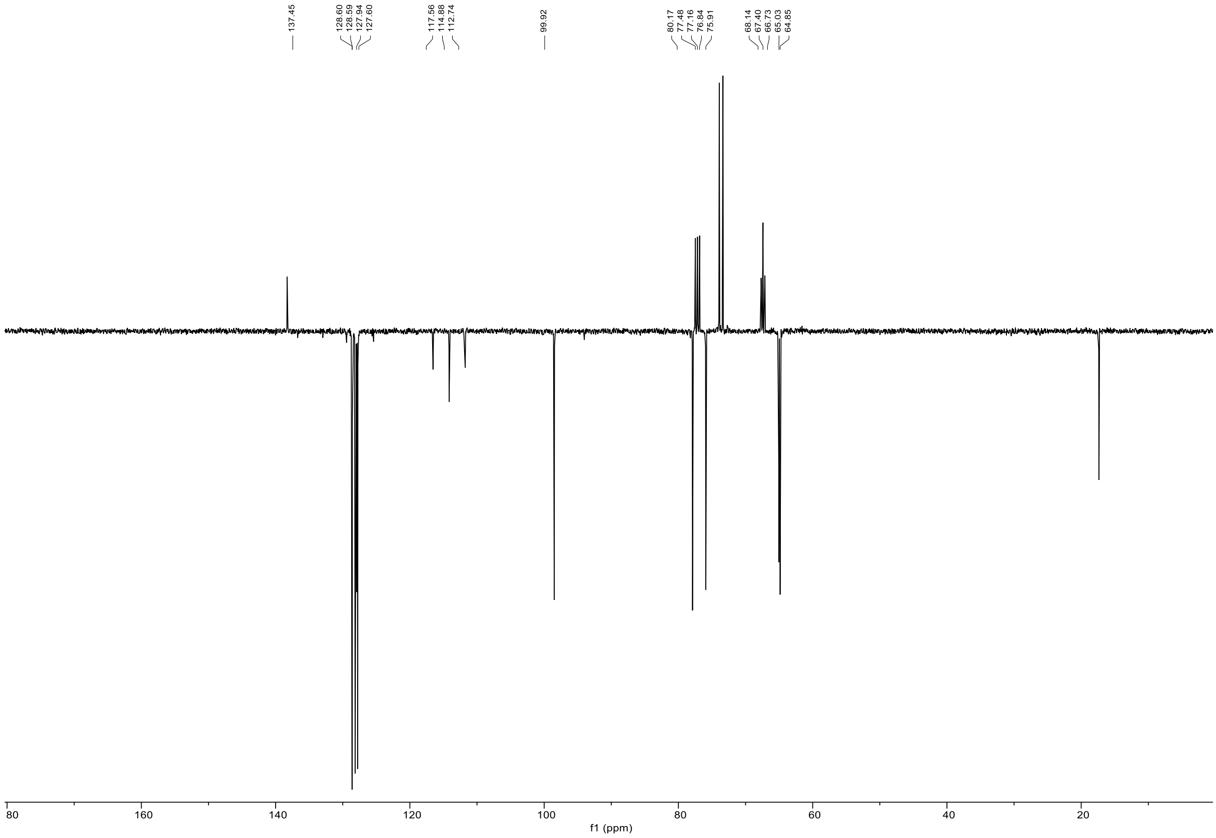

^{1}H NMR, 400 MHz, $CDCl_3$ of **S78 (+DMF)**

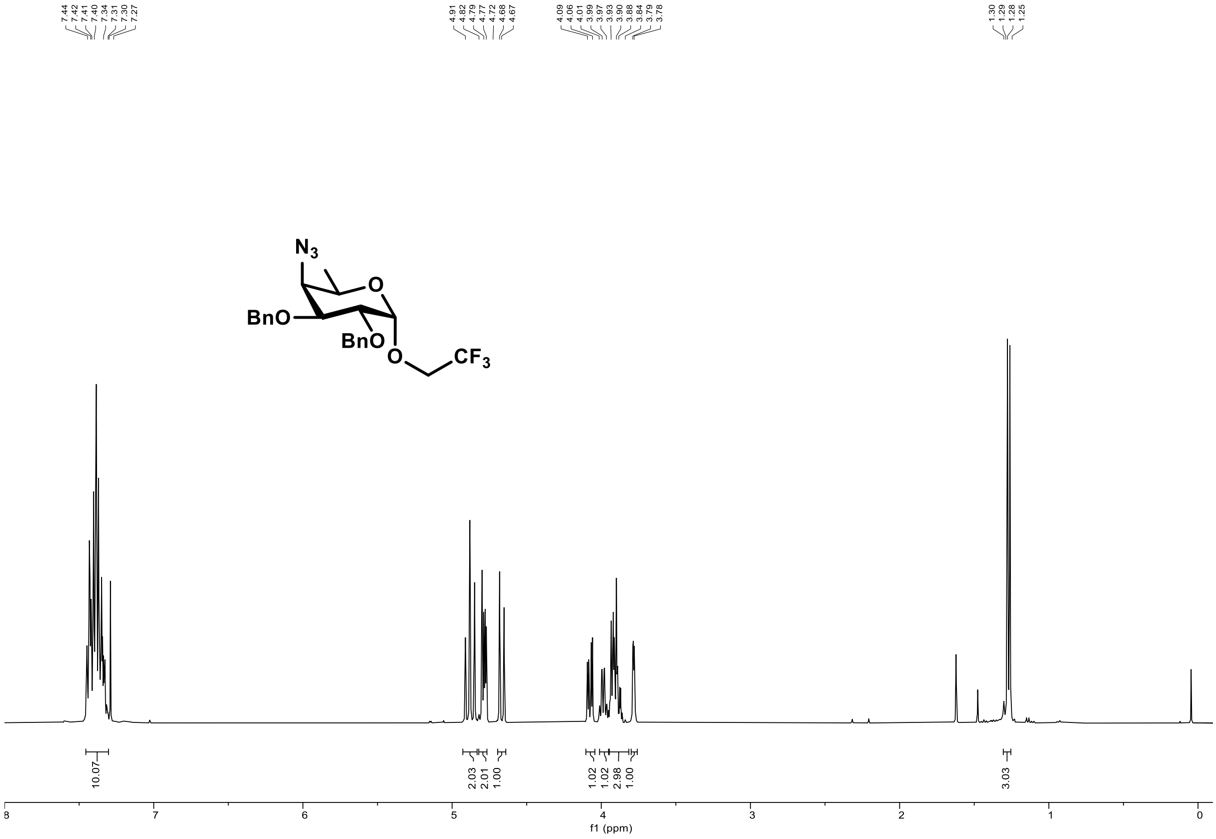

^{13}C NMR, 101 MHz, $CDCl_3$ of **S78 (+DMF)**

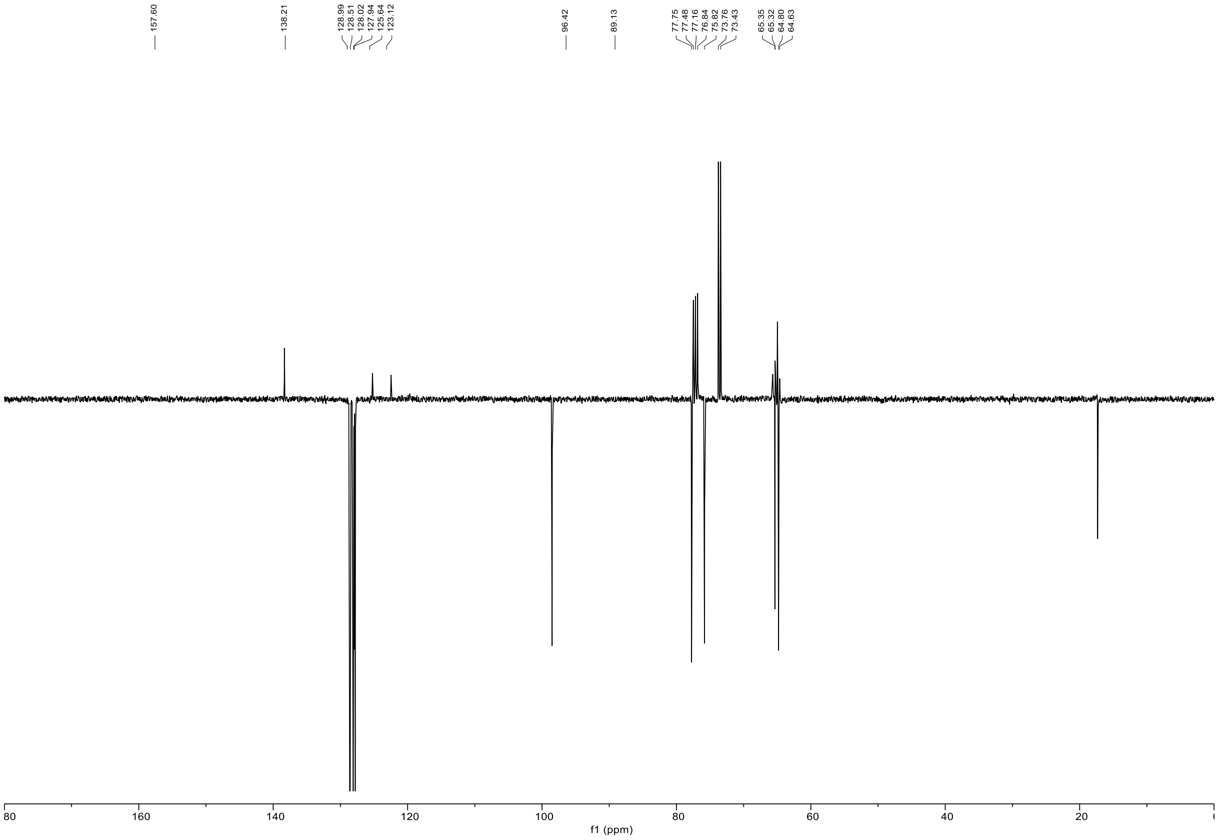

^{1}H NMR, 500 MHz, $CDCl_3$ of **S74 (+DMF)**

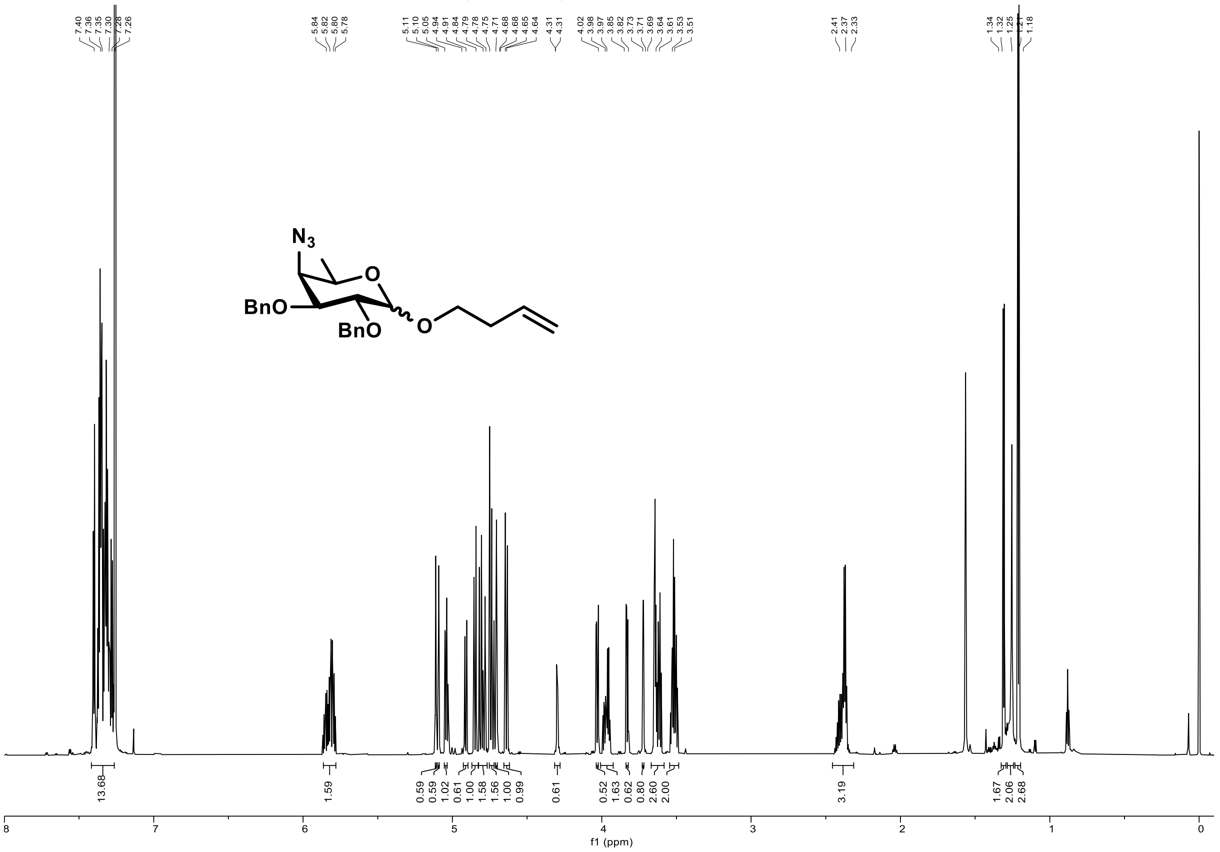

^{13}C NMR, 126 MHz, CDCl$_3$ of **S74 (+DMF)**

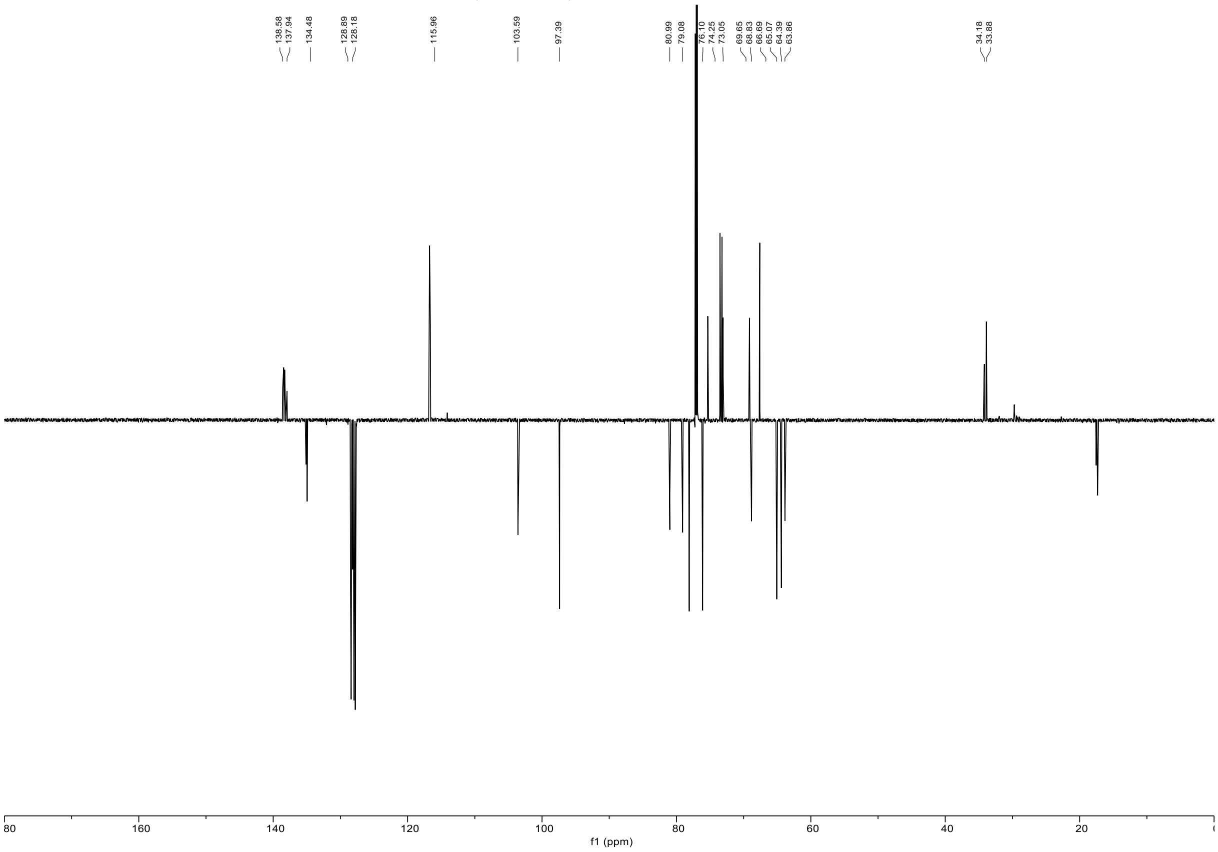

^{1}H NMR, 850 MHz, CDCl$_3$ of **S75 (+TBAI)**

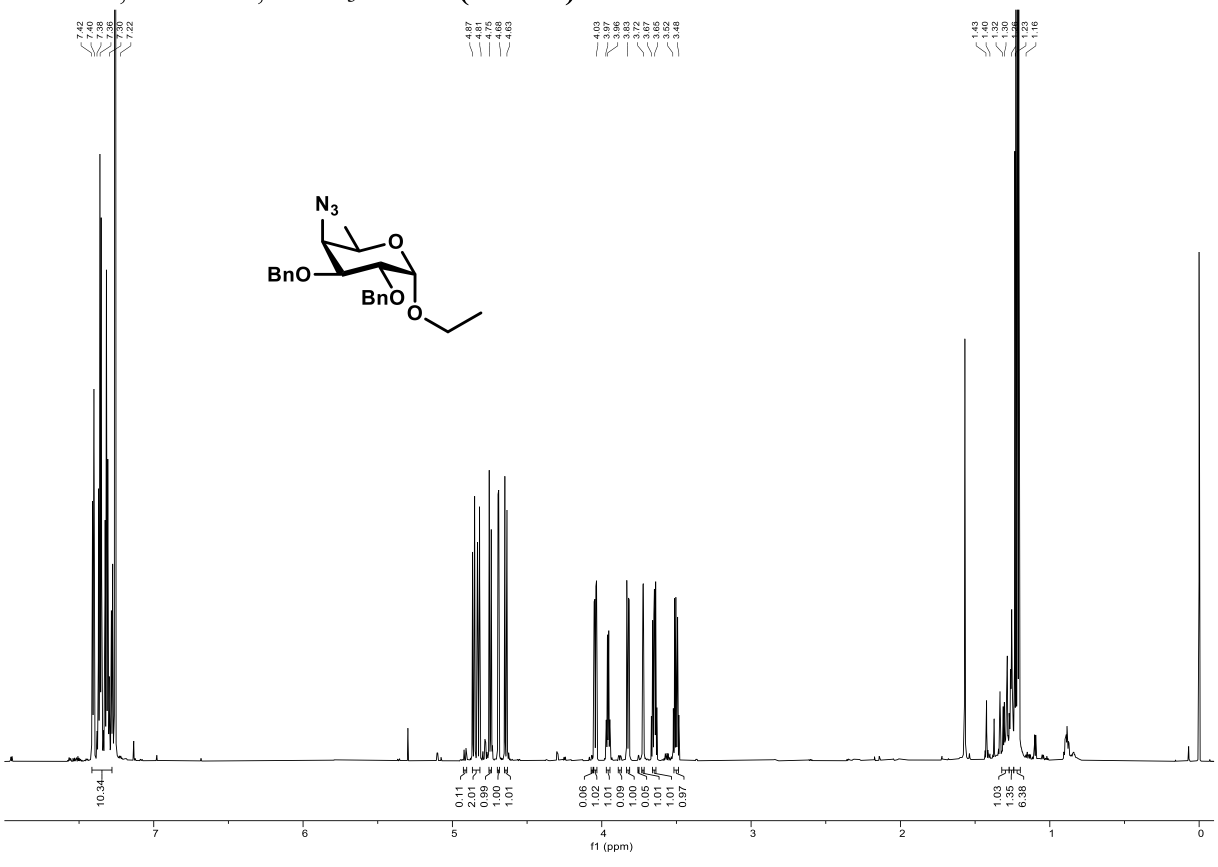

^{13}C NMR, 214 MHz, $CDCl_3$ of **S75 (+TBAI)**

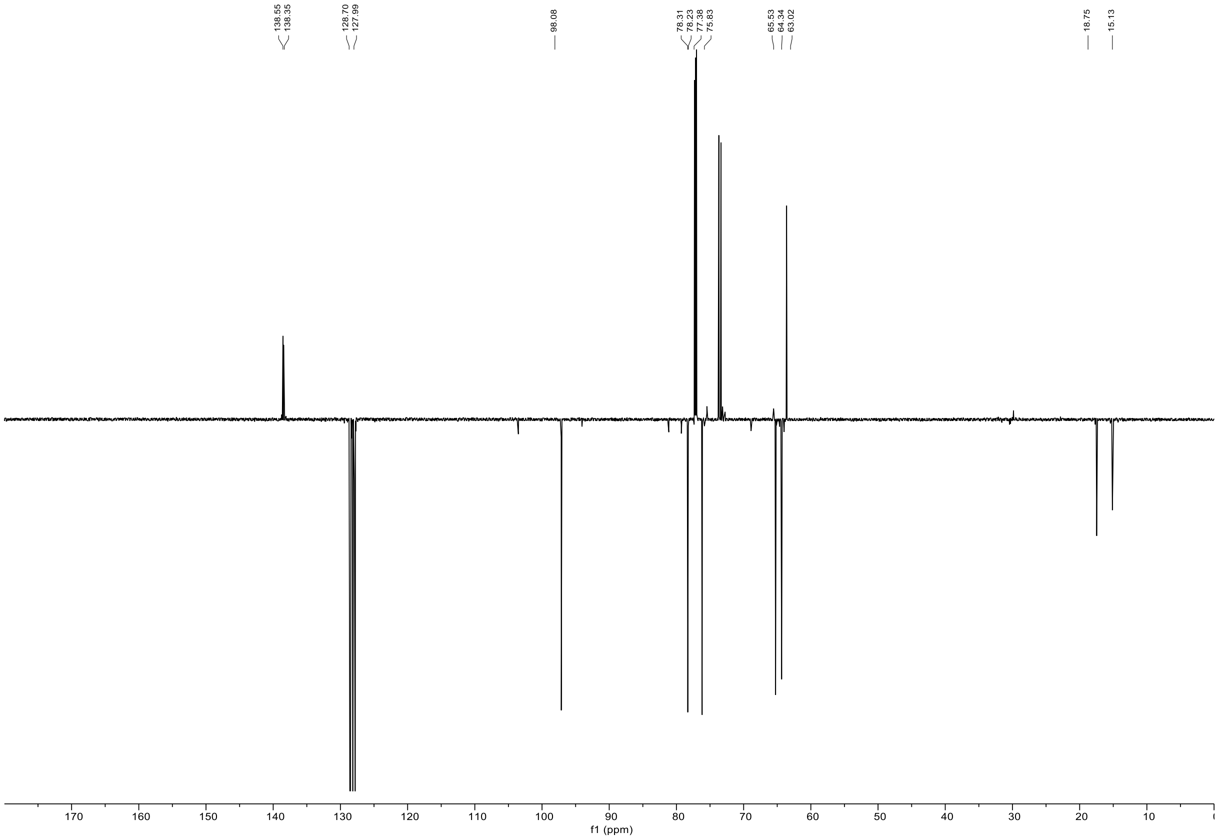

^{1}H NMR, 500 MHz, $CDCl_3$ of **S76 (+TBAI)**

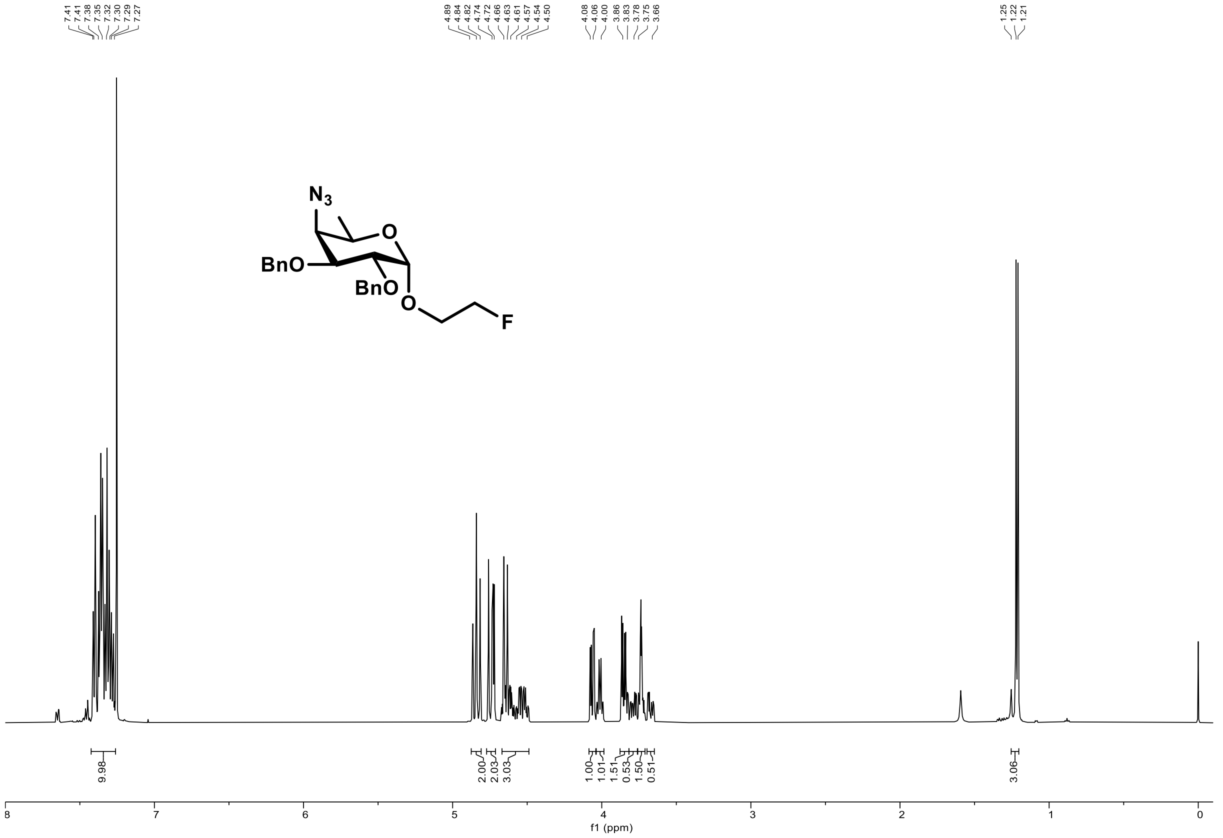

^{13}C NMR, 126 MHz, $CDCl_3$ of **S76 (+TBAI)**

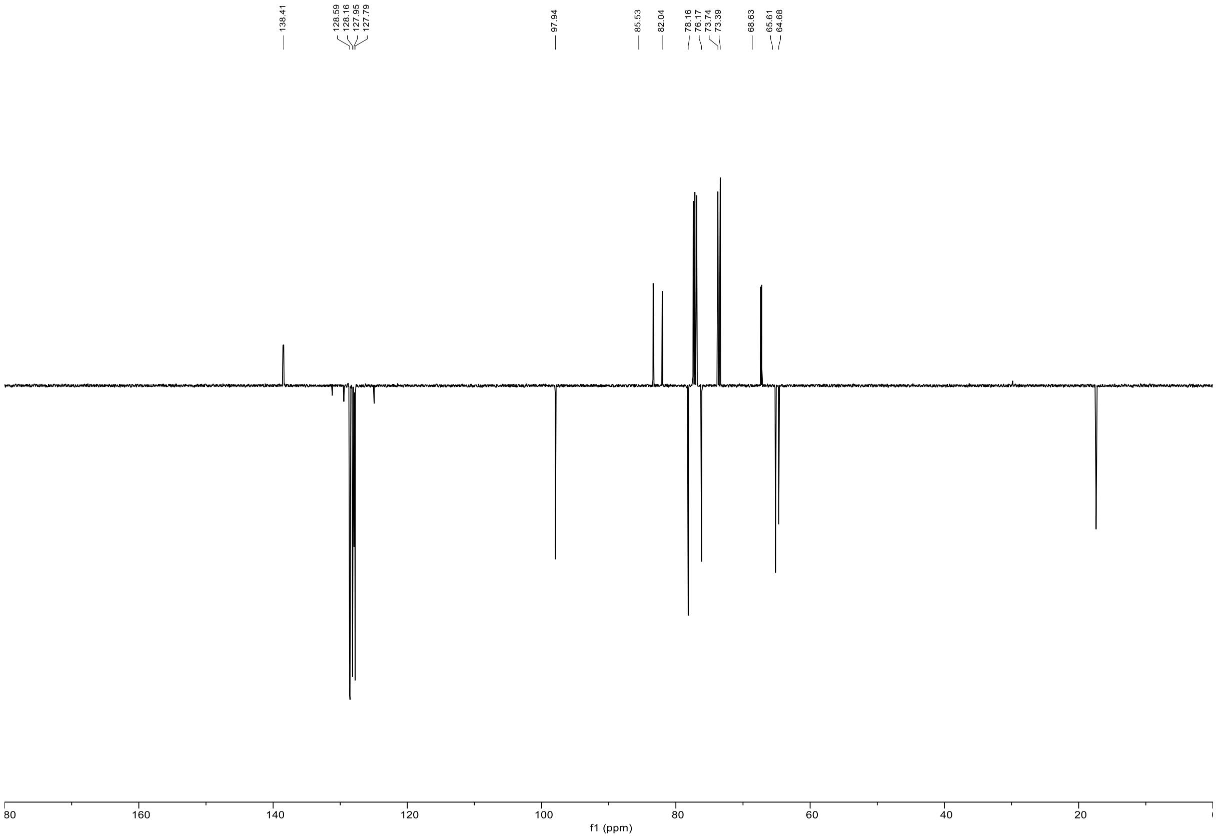

^{1}H NMR, 500 MHz, $CDCl_3$ of **S77 (+TBAI)**

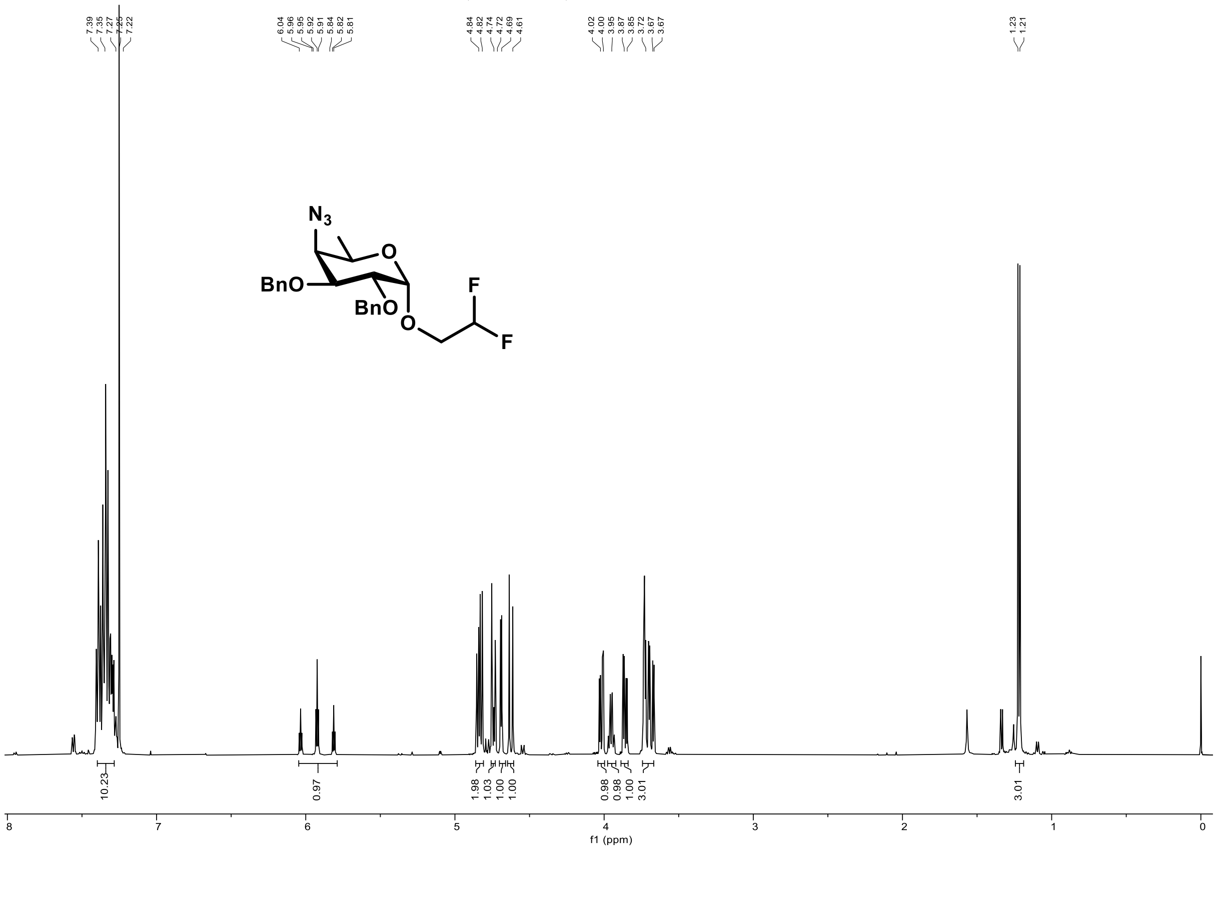

^{13}C NMR, 126 MHz, $CDCl_3$ of **S77 (+TBAI)**

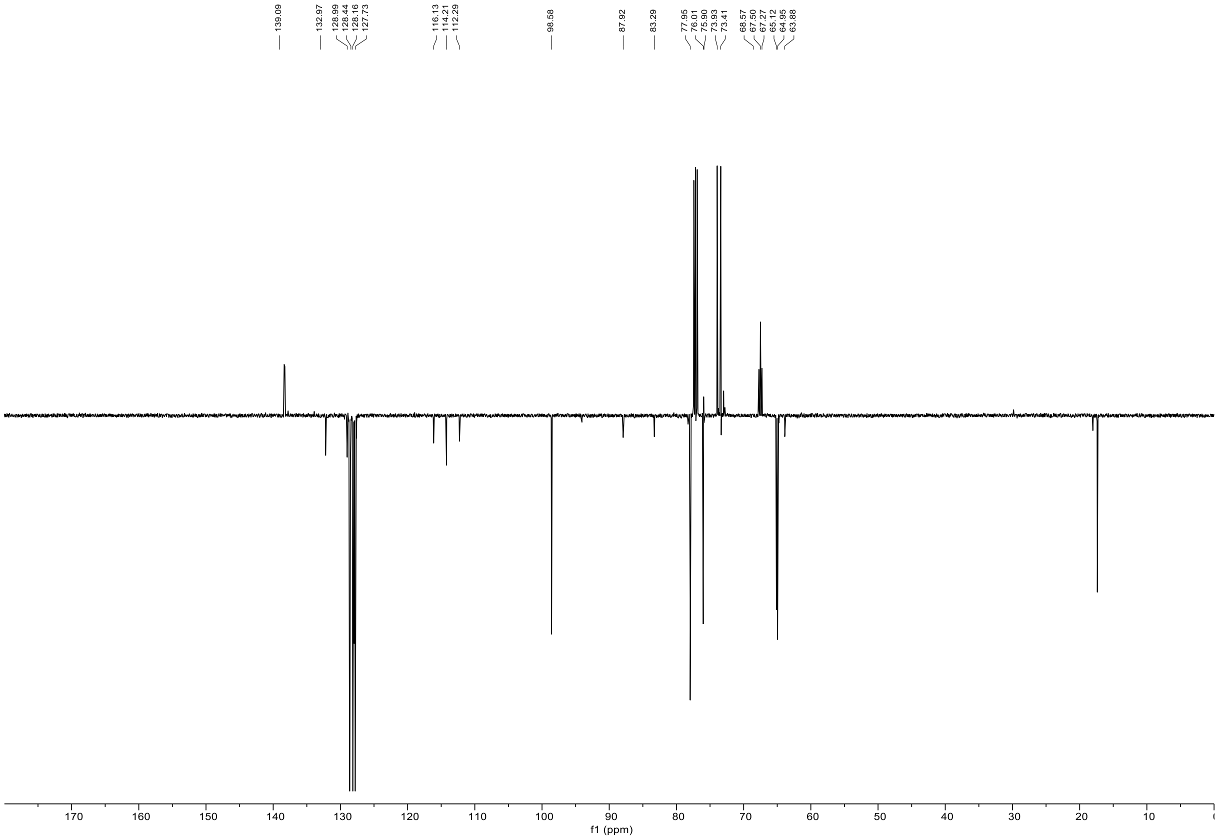

^{1}H NMR, 850 MHz, $CDCl_3$ of **S74 (+TBAI)**

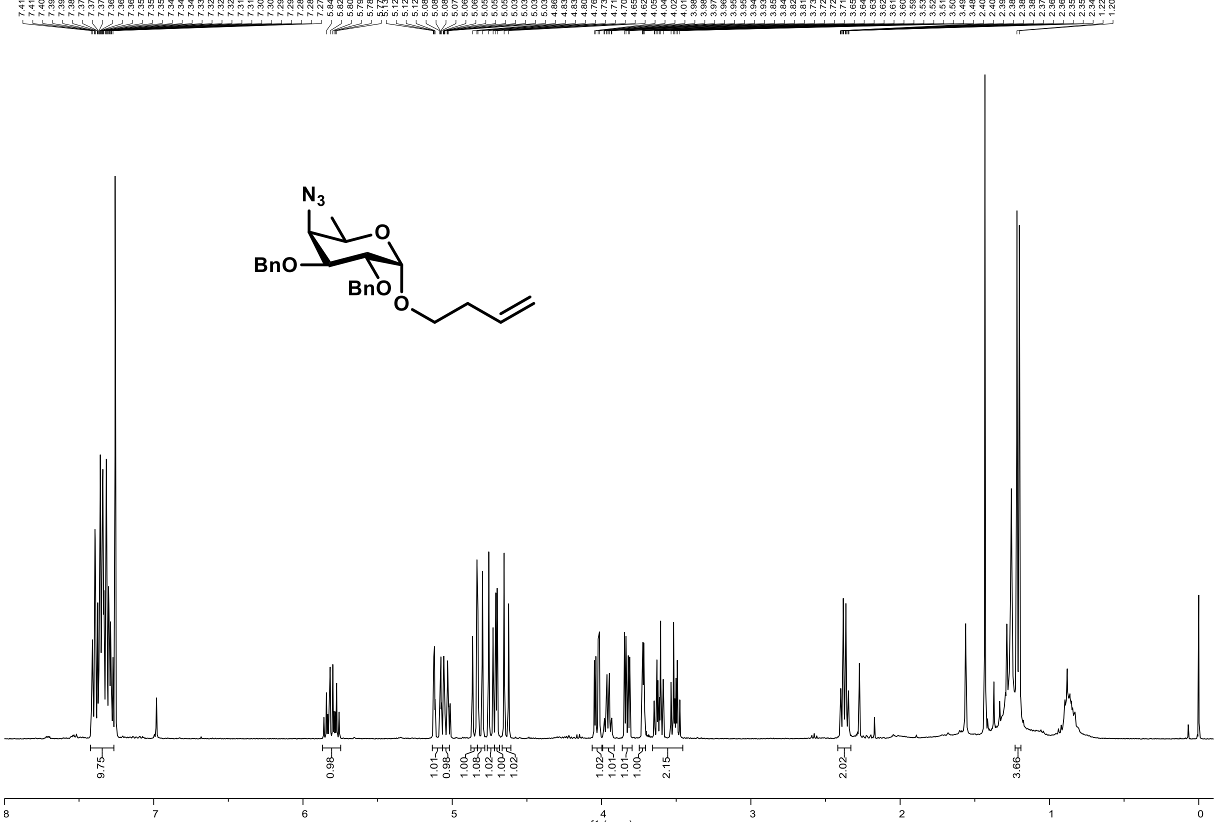

^{13}C NMR, 214 MHz, $CDCl_3$ of **S74 (+TBAI)**

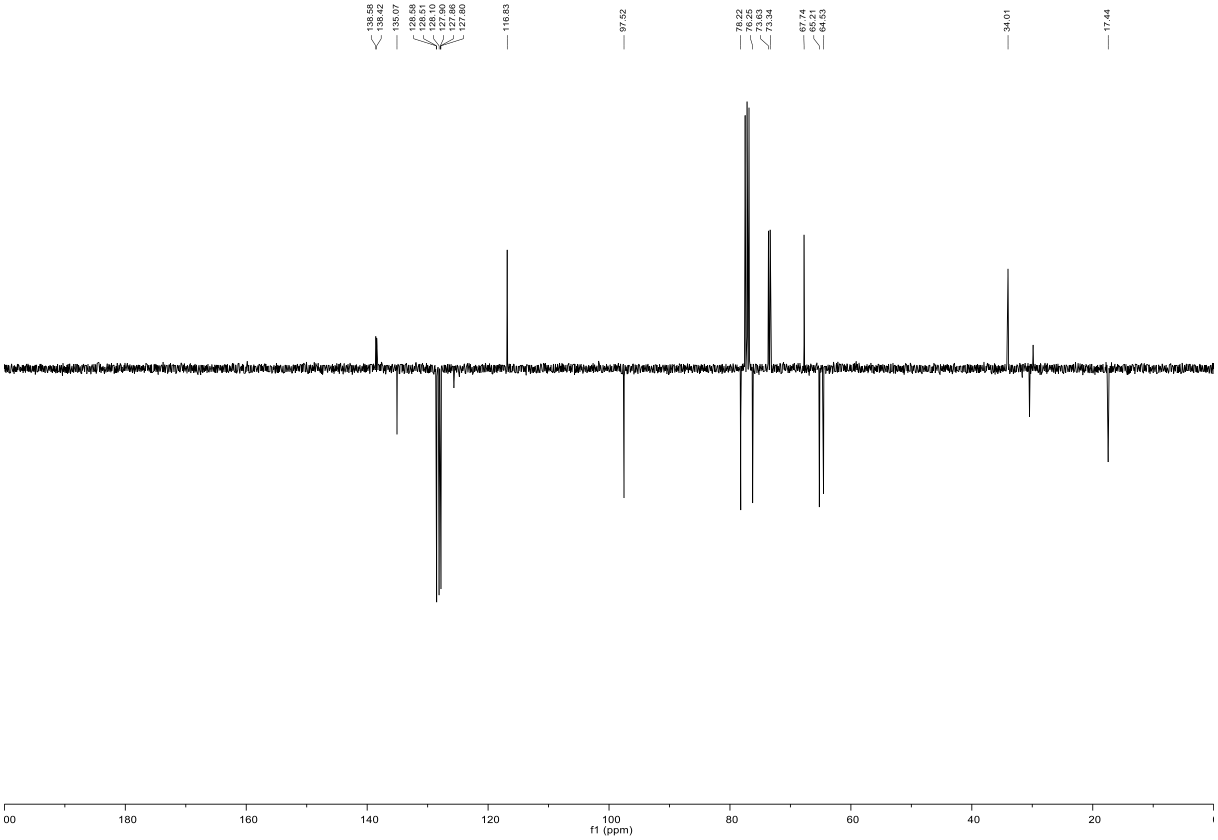

Supplement: Supplementary file 1 — Supplementary [file ANIE-60-937-s001.pdf]
